# Salt-induced phosphoproteomic changes in the subfornical organ in rats with chronic kidney disease

**DOI:** 10.1080/0886022X.2023.2171886

**Published:** 2023-01-30

**Authors:** Xin Wang, Huizhen Wang, Jiawen Li, Lanying Li, Yifan Wang, Aiqing Li

**Affiliations:** a National Clinical Research Center for Kidney Disease, State Key Laboratory of Organ Failure Research, Guangdong Provincial Clinical Research Center for Kidney Disease, Guangdong Provincial Key Laboratory of Renal Failure Research, Nanfang Hospital, Southern Medical University, Guangzhou, China; b Nephrology Division, The Third Affiliated Hospital of Sun Yat-sen University, Guangzhou, China; c The First Affiliated Hospital of Guangdong Pharmaceutical University, Guangzhou, China; d Anshun People’s Hospital of Guizhou Province, Anshun, China

**Keywords:** High-salt diet, chronic kidney disease, subfornical organ, phosphopeptides

## Abstract

**Objectives:**

Subfornical organ (SFO) is vital in chronic kidney disease (CKD) progression caused by high salt levels. The current study investigated the effects of high salt on phosphoproteomic changes in SFO in CKD rats.

**Methods:**

5/6 nephrectomized rats were fed a normal-salt diet (0.4%) (NC group) or a high-salt diet (4%) (HC group) for three weeks, while sham-operated rats were fed a normal-salt diet (0.4%) (NS group). For phosphoproteomic analysis of SFO in different groups, TiO_2_ enrichment, isobaric tags for relative and absolute quantification (iTRAQ) labeling, and liquid chromatography-tandem mass spectrometry (LC-MS/MS) were used.

**Results:**

There were 6808 distinct phosphopeptides found, which corresponded to 2661 phosphoproteins. NC group had 168 upregulated and 250 downregulated phosphopeptides compared to NS group. Comparison to NC group, HC group had 154 upregulated and 124 downregulated phosphopeptides. Growth associated protein 43 (GAP43) and heat shock protein 27 (Hsp27) were significantly upregulated phosphoproteins and may protect against high-salt damage. Differential phosphoproteins with tight functional connection were synapse proteins and microtubule-associated proteins, implying that high-salt diet disrupted brain’s structure and function. Furthermore, differential phosphoproteins in HC/NC comparison group were annotated to participate in GABAergic synapse signaling pathway and aldosterone synthesis and secretion, which attenuated inhibitory neurotransmitter effects and increased sympathetic nerve activity (SNA).

**Discussion:**

This large scale phosphoproteomic profiling of SFO sheds light on how salt aggravates CKD *via* the central nervous system.

## 1. Introduction

People’s health is jeopardized by chronic kidney disease (CKD). According to Global Burden of Disease, Injuries, and Risk Factors Study 2017 (GBD 2017), CKD is not only the 12th leading cause of death with a global prevalence of 9.1% but also a significant determinant of disability-adjusted life-years [1]. A high-salt diet has been demonstrated to hasten CKD progression to end stage kidney disease (ESKD) [2–5]. However, how high salt promotes CKD is not fully understood and thus remains to be explored better to guide treatments (such as salt restriction) to improve renal outcomes.

As a circumventricular organ, subfornical organ (SFO) locates at the caudal of the foramen of Monroe at the confluence of lateral ventricles to the third ventricle. It has a highly vascularized core lacking a blood-brain barrier and possesses a dense population of ion channels and hormone receptors. These properties allow SFO to directly detect the factors in cerebrospinal fluid (CSF) and blood, such as Na^+^, Ang II, and aldosterone [6–8]. For example, Na*_x_* channel expressed explicitly in the glial cells of SFO and organum vasculosum lamina terminalis (OVLT) as the most characterized brain [Na^+^] sensor [9,10]. SFO detects changes in [Na^+^] within physiological ranges and relays this information to other brain loci to control body fluid homeostasis [11,12]. Nomura et al. recently discovered that Na_x_ signals activated by high salt in OVLT are transmitted *via* OVLT-paraventricular nucleus(PVN)-rostral ventral lateral medulla(RVLM) neural pathway to enhance sympathetic nerve activity(SNA) and blood pressure(BP) [13]. Thus, in salt-induced hypertension, Na_x_-positive glial cells in SFO are likely to detect [Na^+^] and relay the signal to other nuclei. Of course, more research is required to test this speculation.

In addition to activating Na*_x_* channel, high salt also affects other functional proteins in SFO. Salt-sensitive rats fed a high-salt diet result in elevated CSF sodium concentration ([Na^+^]). Acute elevations in CSF [Na^+^] cause a rapid, short-term increase in peripheral SNA and BP by activating angiotensinergic pathways from SFO to PVN/supraoptic nucleus (SON) and RVLM [14–16]. In contrast, chronic increases in CSF [Na^+^] mediates a slowly sustained sympathoexcitation that depends on ‘aldosterone-endogenous ouabain (EO)-angiotensin II (Ang II)’ neuromodulatory pathway [14–17]. Furthermore, the salt load can paradoxically activate all major components of intrarenal and cerebral renin-angiotensin systems (RAS) in 5/6-nephrectomized (5/6Nx) rats, which is defined as reno-cerebral RAS axis. This RAS axis is interlinked by afferent and efferent renal sympathetic nerves and is independent of the systemic RAS or BP. Blockade of central RAS or sympathetic nerves significantly attenuates renal fibrosis [18]. Taken together, these data suggest that SFO contributes to the detrimental action of salt in CKD progression.

Phosphorylation has received a great deal of attention as a post-translational modification, which is critical for regulating many cellular events [19]. The disease development is usually mediated by abnormal phosphorylation of functional proteins or signaling pathways [20]. The salt-induced renal injury mechanisms mentioned above involving neurotransmitters and electrochemical signaling pathways. Therefore, research on phosphoproteomic changes in SFO in CKD rats fed a high-salt diet may aid in elucidating the mechanisms above or discovering new molecules relevant to salt-induced injury.

**Scheme 1.**
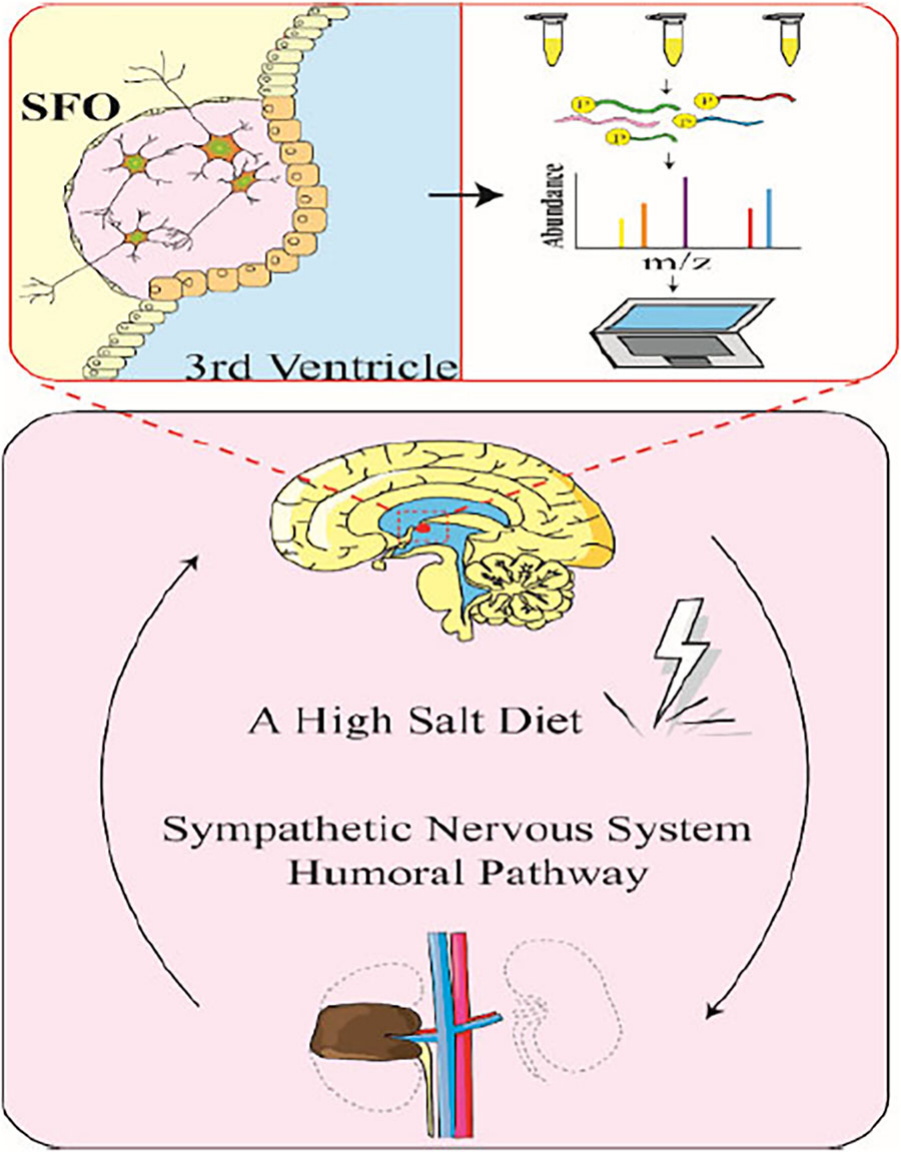
Schematic diagram of phosphoproteomic analysis of SFO in HC group.

As illustrated in Scheme 1, a 5/6Nx rat model of CKD was used to study the salt-induced phosphoproteomic changes in SFO. TiO_2_ enrichment, iTRAQ labeling, and LC-MS/MS were applied for the quantitative determination of differential phosphopeptides. Here, we found that high salt was a risk factor for CKD progression *via* destroying the structure and function of brain, disturbing neurotransmitters’ balance, and enhancing SNA.

## 2. Materials and methods

### 2.1. Animals

Five-week-old male Sprague-Dawley (SD) rats obtained from Southern Medical University Animal Experiment Center were raised in a pathogen-free condition. The Nanfang Hospital Animal Ethics Committee approved the animal study protocol, and the protocol code was NFYY-2017-91.

### 2.2. Treatments

CKD model was induced by five-sixths nephrectomy (5/6Nx), as previously described [21]. The sham operation group also underwent surgery, but no kidney tissue was removed. After the operation of eight weeks, the sham-operated rats received a normal-salt diet (0.4% NaCl, NS, *n* = 8). While 5/6Nx rats were randomly assigned to two groups (*n* = 8 per group) receiving a normal-salt (0.4% NaCl, NC) or high-salt (4% NaCl, HC) diet for three weeks. The rat chow with different salt concentrations was purchased from Trophic Animal Feed Hightech Co., Ltd., Nantong, China.

### 2.3. Measurement of SBP and renal function

A sphygmomanometer (BP-98A, Softron, Japan) equipped with a tail-cuff was used to assess the systolic blood pressure (SBP) in rats. Serum creatinine (SCr) and blood urea nitrogen (BUN) were collected by an automatic biochemical analyzer (AU480,Beckman Coulter, USA) as markers of renal function.

### 2.4. Tissue collection

The rats were sacrificed under deep anesthesia. The brains were carefully peeled out and cut into thin slices in a brain mold. SFO was confirmed and separated according to the rat brain atlas of Paxions and Waston. Then, the tissue samples were stored at −80 °C refrigerator (Thermo Scientific, USA). The entire process was performed on ice.

### 2.5. Protein extraction, digestion, and labeling with iTRAQ reagents

SFO tissue samples were dissolved in the lysis buffer SDT (4% sodium dodecyl sulfate, 1 mM dithiothreitol, 150 Mm Tris-HCl, pH 8) and then subjected to mechanical homogenization. The cellular debris was removed by centrifugation (12,000 g, 4 °C, 10 min). The extracted protein was quantified with a BCA reagent (Beyotime, China). Each SFO sample from three groups was processed with eight independent biological replicates. The tissue homogenate containing 100 μg proteins was digested by trypsin according to the filter-aided sample preparation (FASP) procedure described by Wisniewski [22]. Finally, equal amount of peptide mixtures derived from different groups were labeled with 8-plex iTRAQ. The groups NS, NC, and HC were labeled with 113, 114, and 115 isobaric tags, respectively.

### 2.6. Enrichment of phosphorylated peptides by TiO_2_ beads

After vacuum concentrating and resuspending the labeled peptides in the loading buffer, they were agitated with TiO_2_ beads and centrifuged to obtain the first beads. After the first centrifugation, the process was repeated with new TiO_2_ beads and centrifuged to get the second beads. The phosphorylated peptides were eluted from the beads after washing several times. Finally, phosphorylated peptides were lyophilized and subjected to MS analysis.

### 2.7. Nano-LC-MS/MS analysis

The phosphorylated peptides were separated using a Thermo Scientific Nano-LC system. Briefly, the lyophilized phosphopeptides were resuspended. The solution mixtures were run on a C18 reversed-phase column in buffer A and separated with a linear gradient of buffer B for 240 min. Next, the separated phosphopeptides were subjected to MS analysis using a Q Exactive MS.

### 2.8. Identification and quantitation of phosphorylated peptides and proteins

The Mascot 2.2 engine (Matrix Science, London, UK) and Proteome Discover 1.4 (Thermo Scientific) were used to identify proteins and quantify phosphopeptides. More details were described in our previous study [23]. 

### 2.9. Bioinformatics analysis

All the differential phosphopeptides were processed with a hierarchical cluster analysis using the Cluster program. Protein Analysis Through Evolutionary Relationships (PANTHER) classification system was employed to annotate the differentially phosphorylated proteins with Gene Ontology (GO) format. The phosphoproteins were classified into different signaling pathways by the Kyoto Encyclopedia of Genes and Genomes (KEGG). The interacting proteins network was established by the Search Tool for the Retrieval of Interacting Genes (STRING).

### 2.10. Western blotting analysis

The expression levels of phospho-Hsp27 and phospho-GAP43 in SFO homogenates were determined by western blotting as previously described [23]. The primary antibodies included phospho-Hsp27 (Ser15, Thermo Fisher Scientific Cat#PA1-018), phospho-GAP43 (Ser41, Abcam Cat#ab167162), and GAPDH (Proteintech Cat#60004-1-Ig).

### 2.11. Statistical analysis

SPSS 22.0 was used for statistical analysis. All results were presented as mean ± *SD*. One-way analysis of variance followed by the least significant test was performed to compare differences among groups. *P* values less than 0.05 indicated statistical significance.

## 3. Results

### 3.1. Changes in physiological parameters

Among 5/6 nephrectomy rats, a three-week high-salt diet significantly increased SBP and kidney weight/body weight (kidney wt/body wt) ratio (Table 1). The SBP of HC group (174.9 ± 3.1 mmHg) was 1.4 and 1.2 times higher than NS group (121.1 ± 1.3 mmHg) and NC group (144.9 ± 4.0 mmHg), respectively. Additionally, the kidney wt/body wt ratio in HC group grew up to 5.3, far beyond that in NS (3.5) and NC (4.2) groups. The ratio was relative to the degree of renal fibrosis. However, rats in the high-salt group presented increasing serum creatinine without statistical significance compared with CKD rats on a normal-salt diet, which could not represent the prolonged observation time.

### 3.2. Identification of phosphorylated peptides and sites

**Figure 1.**
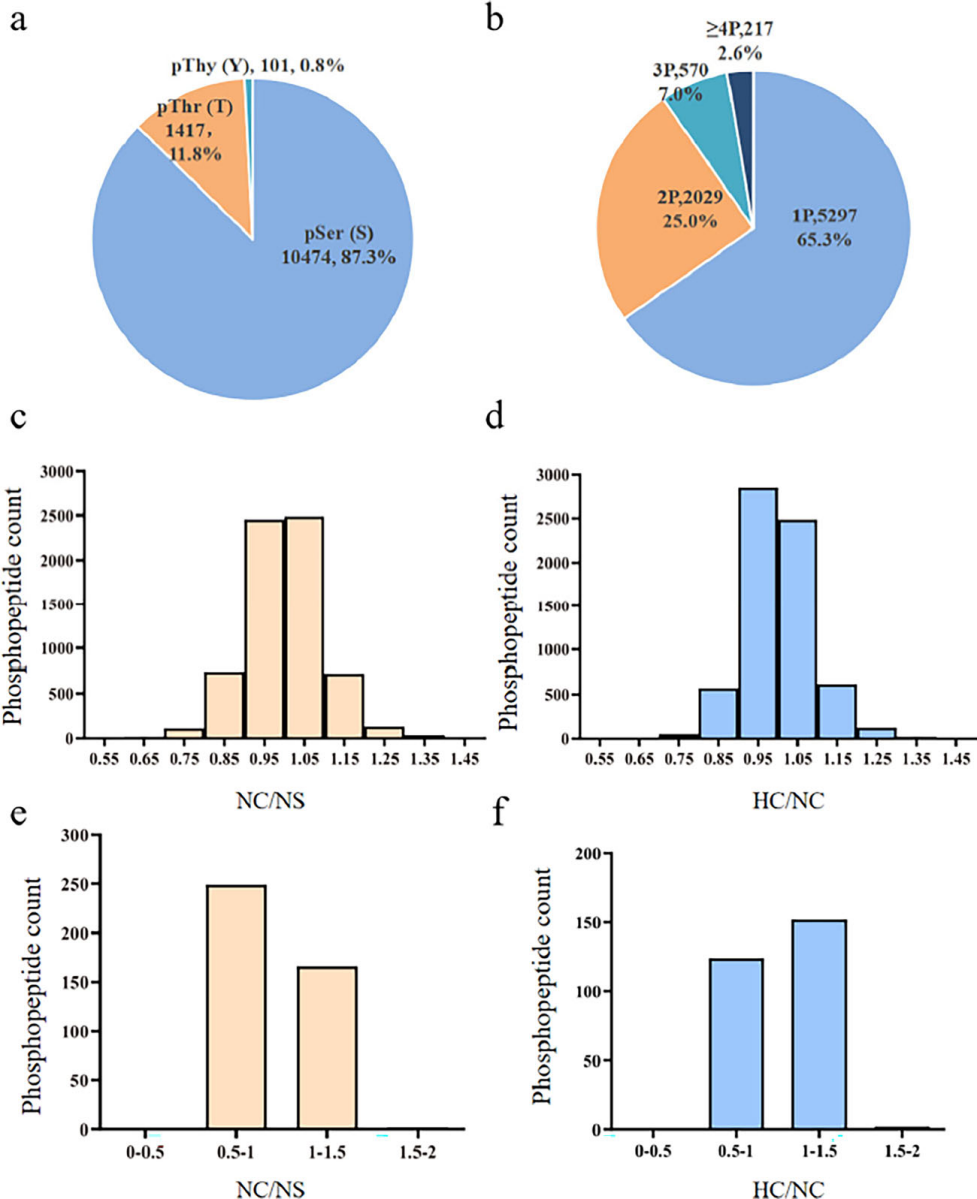
Characterization of phosphopeptides and phosphosites. (a) The Ser, Thr, and Tyr phosphosites with high confidence in SFO phosphoproteome distribution. (b) Percentage distribution of singly, doubly, triply, and quadruply phosphorylated peptides with high confidence. (c,d) Frequency distribution of phosphopeptides quantitation ratio at NC/NS or HC/NC. (e,f) Frequency distribution of differentially phosphorylated peptides ratio at NC/NS or HC/NC. NS: sham operation + normal salt diet; NC: 5/6 Nx + normal salt diet; HC: 5/6 Nx + high salt diet; 5/6 Nx: 5/6 nephrectomy; SFO: subfornical organ.

This study identified 6808 phosphorylated peptides with 11,992 phosphorylation sites (Supplementary Table S1-1). As illustrated in Figures 1(a,b), most phosphorylation sites were located at serine (Ser) residues (87.3%), followed by threonine (Thr) residues (11.8%) and tyrosine (Tyr) residues (0.8%). Phosphorylated peptides had one to four phosphorylation sites. 5297, 2029, 570, and 217 phosphopeptides were identified with singly, doubly, triply, or quadruply phosphorylated sites. Figures 1(c,d) reflect the frequency distribution of phosphopeptide ratios of two comparison groups, i.e., NC/NS and HC/NC. The quantitative ratio distributions of differential phosphopeptides (a fold change >1.2 or <0.83, *p*-value <0.05) are displayed in Figures 1(e,f). The operation of 5/6 nephrectomy induced 418 differential phosphopeptides (168 upregulated and 250 downregulated) corresponding to 327 phosphoproteins (Supplementary Table S1-2). High salt resulted in 278 differential phosphopeptides (154 upregulated and 124 downregulated) (Supplementary Table S1-3) in CKD rats, matching 223 phosphoproteins. Moreover, hierarchical clustering analysis was applied to visualize and simplify the differential phosphopeptide expression patterns of two comparison groups, demonstrating that salt load adversely affected the SFO (Supplementary Figure S1).

**Table 1.** Changes in biochemical and metabolic parameters.

	Sham + normal salt	5/6Nx + normal salt	5/6Nx + high sat
Body wt (g)			
0 week	431 ± 15.7	419 ± 12.0	447 ± 10.9
3 weeks	466 ± 17.8	451 ± 9.5	488 ± 15.5
Kidney wt/body wt (mg/g)	3.5 ± 0.07	4.2 ± 0.15^a^	5.3 ± 0.31^a,b^
Serum creatinine (μmol/ L)			
0 week	32 ± 0.9	67 ± 2.1^a^	72 ± 3.5^a^
3 weeks	33 ± 1.2	74 ± 7.2^a^	91 ± 12.2^a^
SBP (mmHg)			
0 week	120.9 ± 1.8	145.8 ± 2.5^a^	143.7 ± 2.8^a^
3 weeks	121.1 ± 1.3	144.9 ± 4.0^a^	174.9 ± 3.1^a,b^

0 week: 8 weeks after the last surgery; 3 weeks: 11 weeks after the previous operation; kidney wt/body wt: kidney weight/body weight; SBP: systolic blood pressure.

All data are expressed as mean ± SEM (*n* = 15 in each group).

^a^*p* < 0.05 *vs.* sham rats + normal salt.

^b^*p* < 0.05 *vs.* CKD rats + normal salt.

### 3.3. Function analysis of differential phosphoproteins

**Figure 2.**
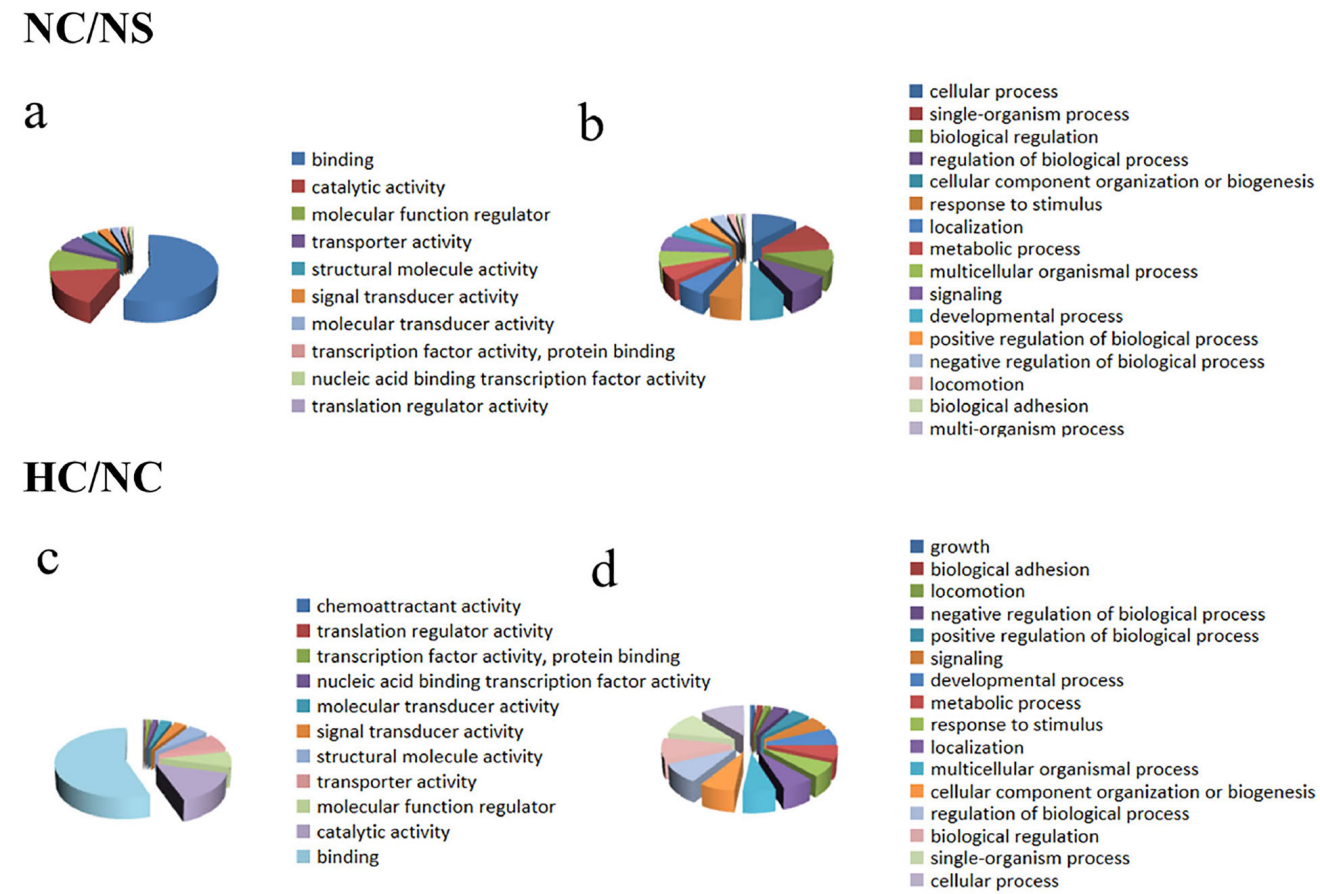
GO analysis of differentially phosphorylated peptides in NC/NS and HC/NC comparison groups. GO analysis of phosphoproteins differently expressed in NC/NS and HC/NC comparison groups based on the molecular function (a,c) and biological process (b,d) using PANTHER classification. NS: sham operation + normal salt diet; NC: 5/6 Nx + normal salt diet; HC: 5/6 Nx + high salt diet; 5/6 Nx: 5/6 nephrectomy.

The complete list of 2661 phosphoproteins corresponding to identified phosphopeptides from SFO in rats with chronic renal failure is depicted in Supplementary Table S2. GO analysis was used to annotate the differential phosphoproteins functionally. As displayed in Figures 2(a,c), the differential phosphoproteins in NC/NS comparison group were classified into 10 categories of molecular functions. Phosphoproteins involved in binding had the highest proportion (56.2%), followed by catalytic activity (17.8%) and molecule function regulation (7.5%). The differential phosphoproteins in HC/NC comparison group were classified into 11 categories of functions, including binding (55.6%), catalytic activity (15.3%), molecule function regulation (7.4%), and so on. In biological process analysis (Figures 2(b,d)), differential phosphoproteins in NC/NS comparison group involved 16 kinds of biological processes. The top three biological processes were cellular process (12.1%), single-organism process (11.1%), and biological regulation (10.1%). In HC/NC comparison group, the differential phosphoproteins were involved in 16 kinds of biological processes, topped by cellular process (11.3%), single-organism process (10.7%), and biological regulation (9.7%). The percentage and number of differential phosphoproteins relevant to molecular functions and biological processes are listed in Supplementary Tables S3 and S4.

**Table 2.** Two representative differential phosphoproteins in HC/NC comparison group.

Phosphoprotein	Protein group accessions	Sequence	Phosphorylation site	NC/NS	HC/NC
GAP43	P07936	iQAsFR	Ser41	1.11	1.34^b^
Hsp27	G3V913	sPsWEPFR	Ser15	1.22^a^	1.71^b^
Hsp27	G3V913	qLsSGVSEIR	Ser86	1.09	1.47^b^

NS: sham operation + normal salt diet; NC: 5/6 Nx + normal salt diet; HC: 5/6 Nx + high salt diet; 5/6 Nx: 5/6 nephrectomy.

^a^*p* < 0.05 *vs.* sham rats + normal salt.

^b^*p* < 0.05 *vs.* CKD rats + normal salt.

Growth associated protein 43 (GAP43) and heat shock protein 27 (Hsp27) are two examples of differential phosphoproteins in Table 2. At phosphorylation site Ser41, the peptide iQAsFR corresponding to protein GAP43 increased 1.34-fold by HC *vs.* NC. GO analysis revealed that GAP43, also known as neuromodulin, was involved in the growth process. For Hsp27, phosphorylation is only slightly induced by 5/6 nephrectomy at Ser15 (1.22-fold) but significantly evoked by high salt at Ser15 (1.71-fold) and Ser86 (1.47-fold). The phosphopeptide of Hsp27 was the most upregulated in HC/NC comparison group. The phosphorylation of GAP43 and Hsp27 may attenuate the salt load-induced SFO lesion.

**Figure 3.**
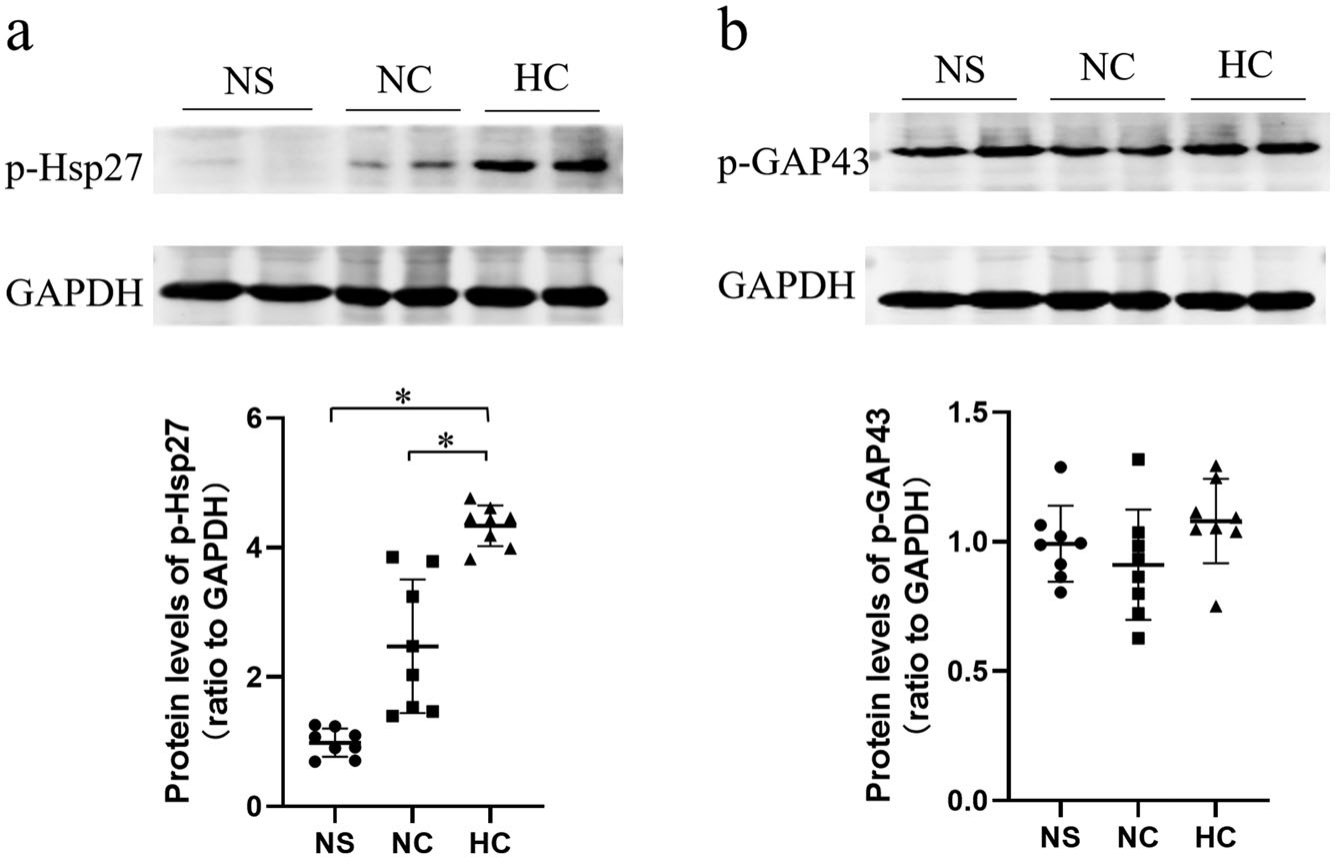
High salt intake induced significant phosphorylation changes of Hsp27 and GAP43. Phosphorylation level of Hsp27(Ser15) (a) and GAP43(Ser41) (b) were validated by western blotting. Data are expressed as the mean±*SD* of three independent experiments (*n* = 8 in each group). *p < 0.001 *vs.* NS group or NC group. NS: sham operation + normal salt diet; NC: 5/6 Nx + normal salt diet; HC: 5/6 Nx + high salt diet; 5/6 Nx: 5/6 nephrectomy.

The phosphorylation levels of Hsp27(Ser15) and GAP43(Ser41) were preliminarily verified by western blotting. As shown in Figure 3, high salt intake leaded to a significant increase in phospho-Hsp27(Ser15) expression in the CKD rats compared to a normal salt diet (Figure 3(a)). However, the expression of phospho-GAP43 was not significantly upregulated (Figure 3(b)). This may have something to do with the fact that western blotting is not as sensitive to detect a 1.34-fold gap for HC/NC comparison group, and enzyme linked immunosorbent assay may be helpful in verifying the 1.34-fold difference. Furthermore, based on the extensive literature, we strongly believe that phospho-GAP43(Ser41) expression is upregulated in HC group.

### 3.4. Protein interaction networks and KEGG pathway analysis

**Figure 4.**
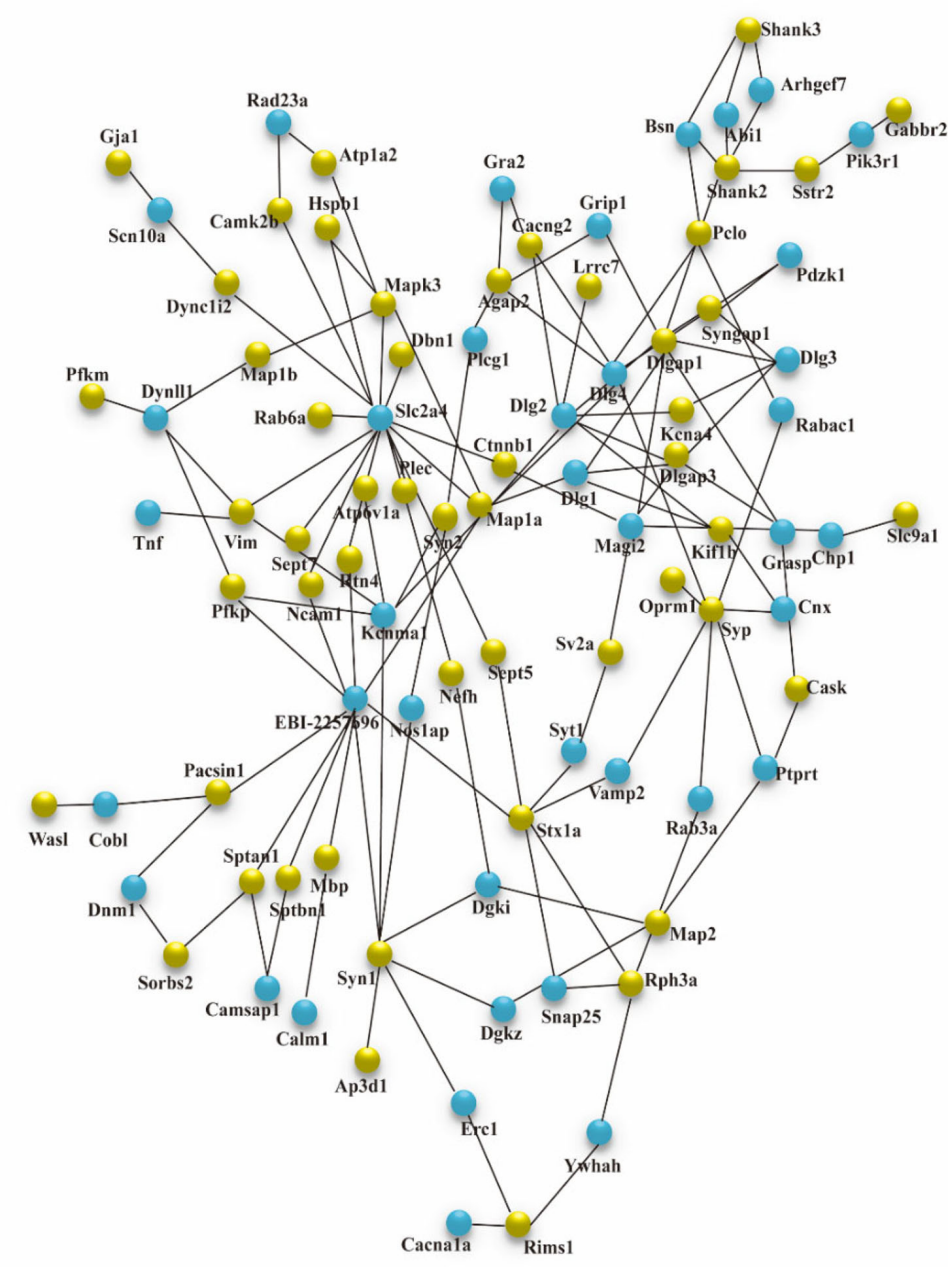
STRING analysis revealed protein interaction networks in SFO phosphoproteome in NC/NS comparison group. Interactions of the identified phosphoproteins were mapped using Search Tool for the Retrieval of Interacting Genes/Proteins database version 9.0 with a confidence cutoff of 0.6. Proteins are presented as nodes connected by lines in the resulting protein association network. The yellow node is the target protein, and the blue node is the other protein that does not directly interact with the target protein. NS: sham operation + normal salt diet; NC: 5/6 Nx + normal salt diet; 5/6 Nx: 5/6 nephrectomy; SFO: subfornical organ.

**Figure 5.**
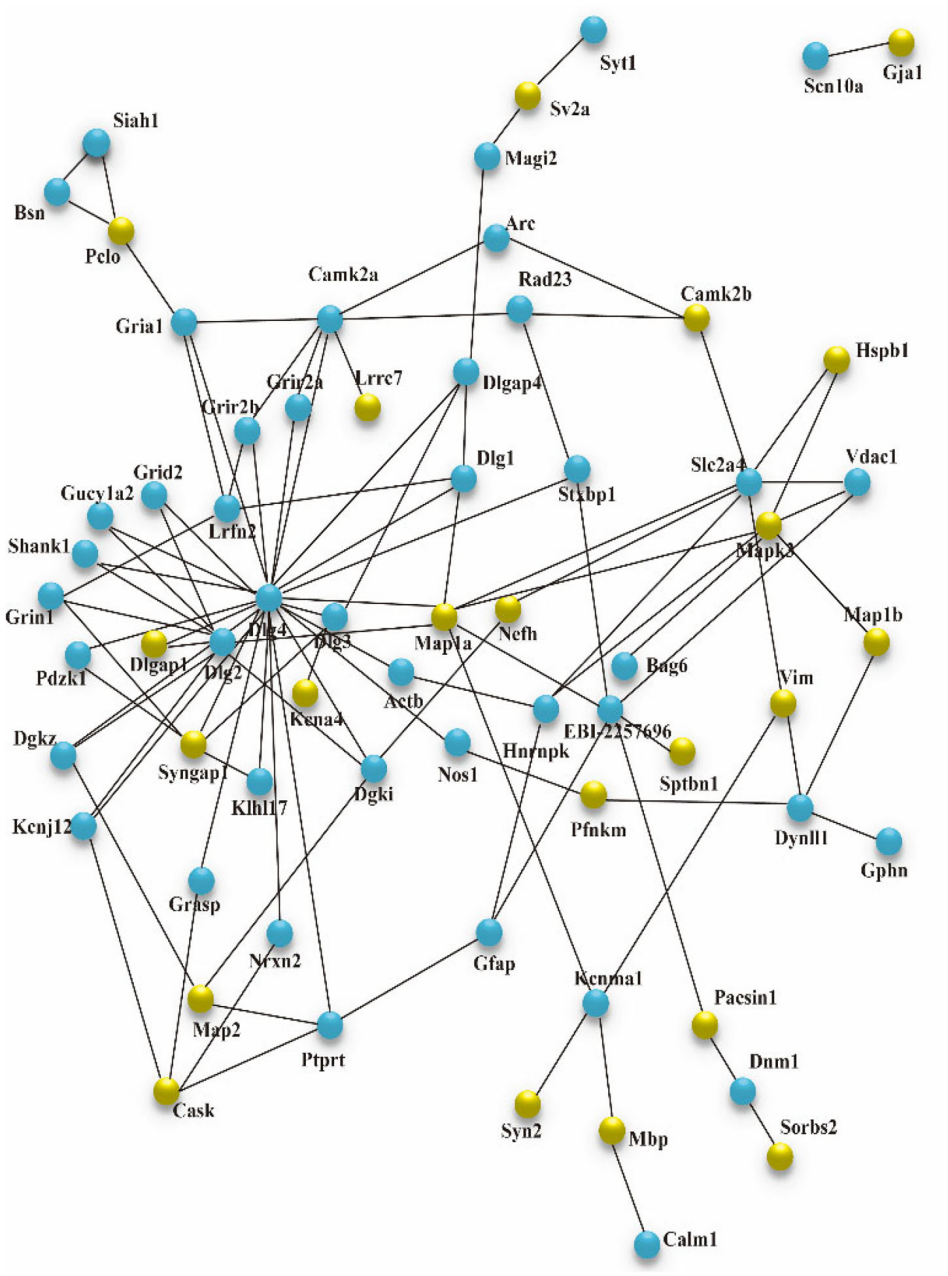
STRING analysis revealed protein interaction networks in SFO phosphoproteome in the HC/NC comparison group. Interactions of the identified phosphoproteins were mapped using the Search Tool for the Retrieval of Interacting Genes/Proteins database version 9.0 with a confidence cutoff of 0.6. Proteins are presented as nodes connected by lines in the resulting protein association network. The yellow node is the target protein, and the blue node is the other protein that does not directly interact with the target protein. NC: 5/6 Nx + normal salt diet + normal salt diet; HC: 5/6 Nx + high salt diet; 5/6 Nx: 5/6 nephrectomy; SFO: subfornical organ.

The STRING database was used to uncover the protein-protein interaction (PPI) network of these differential phosphoproteins. Figure 4 depicted that in NC/NS comparison group, 51 different phosphoproteins were functionally linked either directly or indirectly. Dlgap1, synapsin-1 (Syn1), synaptophysin (Syp), microtubule-associated protein 1 A (Map1a), and syntaxin1A (Stx1a) were the most closely linked proteins. Figure 5 depicted the protein interaction network of HC/NC comparison group, which was made up of 22 differential phosphoproteins. Map1a, Syngap1, and Mapk3 were the network’s central proteins. More interconnected proteins, in general, are more likely to be critical points of pathological changes in SFO.

**Figure 6.**
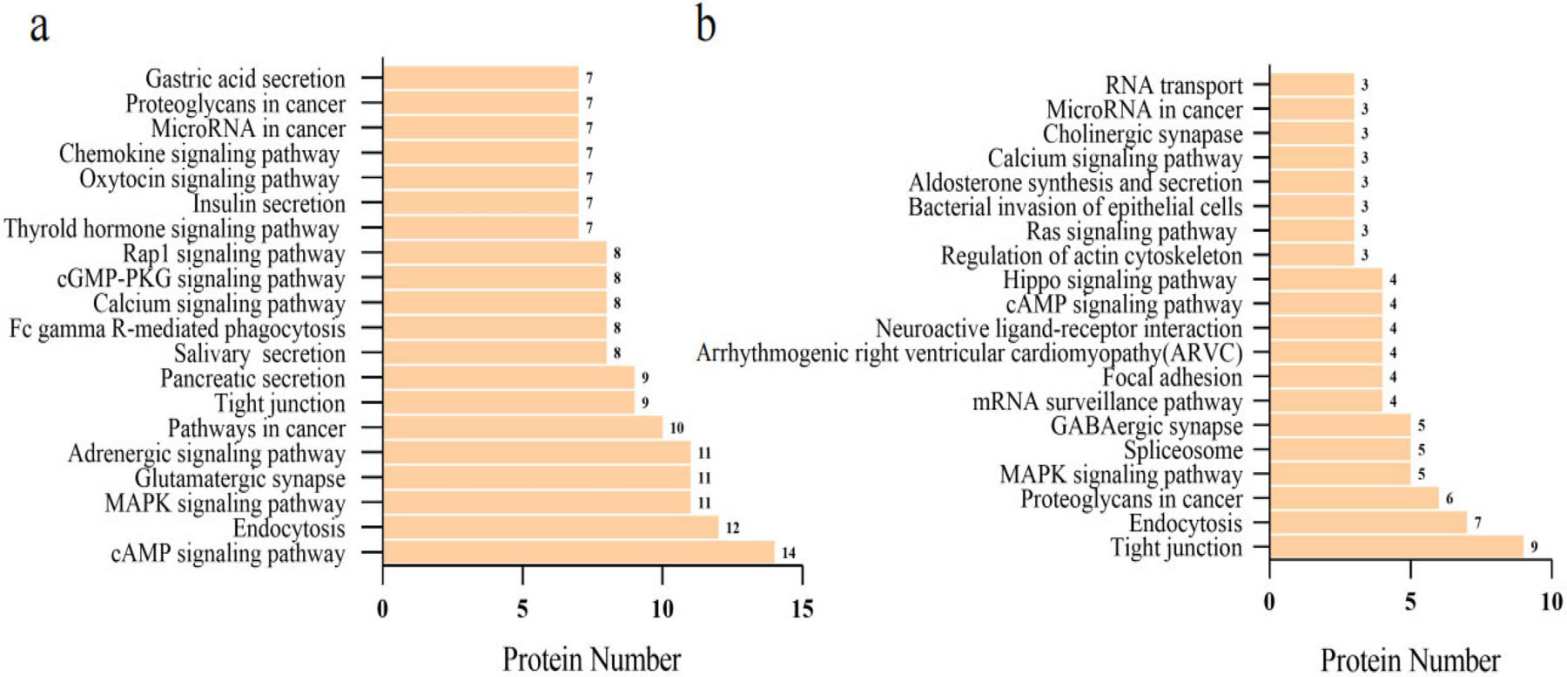
KEGG pathway analysis of differential phosphoproteins in the NC/NS and HC/NC comparison groups. The differential phosphoproteins enriched the top 20 signaling pathways in the NC/NS (a) and HC/NC (b) comparison groups. The horizontal axis is the number of proteins, whereas the vertical ordinates are the terms of KEGG pathways. NS: sham operation + normal salt diet; NC: 5/6 Nx + normal salt diet; HC: 5/6 Nx + high salt diet; 5/6 Nx: 5/6 nephrectomy.

The KEGG pathway annotation of phosphoproteins was used to investigate the signaling pathways in which phosphoproteins participate that could lead to SFO injury. Figure 6 showed that differential phosphoproteins in NC/NS comparison group were primarily associated with cAMP signaling pathway, endocytosis, MAPK signaling pathway, etc. Tight junction, endocytosis, proteoglycans in cancer, and other signaling pathways were found in HC/NC comparison group. Furthermore, the combination of KEGG pathway and STRING analysis will benefit in-depth research.

## 4. Discussion

This study identified 6808 phosphopeptides, corresponding to 2661 phosphoproteins (Supplementary Tables S1-1 and S1-2). Two phosphorylated proteins, GAP43 and Hsp27, among these phosphoproteins, merited additional investigation. Phosphorylated GAP43, with Ser41 as its primary phosphorylation site, is required for neural development and regeneration [24–26]. In this study, phosphorylated GAP43 increased in HC group rather than NC group. The result indicated that salt load was a potent risk factor that deteriorated SFO injury in CKD rats, and SFO made a protective response in return. As a molecular chaperone, Hsp27 repairs misfolded proteins in large oligomers. When phosphorylated at Ser15, Ser78, or Ser82 (corresponding to 86 in rats), it can be converted to the dissociated form [27]. Numerous studies have revealed that phosphorylated Hsp27 protects against cardiovascular and neurological diseases [28–30]. Stetler et al. found that ischemic neuronal injury could be mitigated in mice with sustained activation at Ser15 or Ser82 rather than Ser78 [31], basically consistent with our results. Compared to a normal-salt diet in CKD rats, a high-salt diet significantly enhanced Hsp27 phosphorylation at two sites, Ser15 (1.71-fold) and Ser86 (1.47-fold). Additionally, no phosphorylation of Ser78 was detected. Therefore, these results indicated that SFO strongly reacted to high salt-induced injury.

PPI analysis revealed that differential phosphoproteins with closely related functions in the two comparison groups fell into two categories: synapse proteins and microtubule-associated proteins (MAP). Proteins associated with synapse included Dlgap1, Syn1, Syp, and Stx1a in NC/NS comparison group and Syngap1 in HC/NC group. Dlgap1, a DLAGAP family member, is in charge of stabilizing, recruiting, and transporting important postsynaptic receptors to the postsynaptic membrane [32]. Syngap1 is a GTPase activating protein found in the postsynaptic portion that collaborates with postsynaptic density protein 95 (encoded by Dlg4) [33]. Long term potentiation (LTP) modulated the phosphorylation status of postsynaptic proteins, according to Li et al., Dlgap1 and Syngap1 were the most affected proteins, posing a ‘postsynaptic risk’ for schizophrenia and autism spectrum disorders [34]. Furthermore, uremic encephalopathy is associated with impaired brain synaptic function induced by accumulated uremic toxins [35]. Consequently, phosphoproteomic changes in important postsynaptic proteins regulated by salt load cannot be ignored. MAP is the primary component of neuronal cytoskeleton and facilitates axonal synthesis and transport through controlling microtubule assembly in phosphorylation way [36–38]. Supplementary Table S1-3 displayed that more intense phosphorylation of MAP (Map1a and Map2) was induced by salt load, suggesting that the normal function of axon was seriously disturbed. These findings suggested that high salt levels influenced the phosphorylation of structural and functional proteins in brain. These significant differences in phosphoproteins in SFO induced by high salt or 5/6 nephrectomy provides an insight into the mechanism of nervous system complications in CKD patients.

Gao et al. found that PVN blockade of the p44/42 MAPK pathway attenuated salt-sensitive hypertension by restoring the balance of neurotransmitters, i.e., the increased inhibitory neurotransmitter gamma-aminobutyric acid (GABA) and the decreased excitatory neurotransmitter glutamate [39]. GABAergic neuron can suppress SNA [40,41]. In the present work, differential phosphoproteins mainly converged in glutamatergic synapse and adrenergic signaling in cardiomyocytes in the comparison group NC/NS. The phosphorylation levels of glutaminase and glutamine synthetase were upregulated in NC group (Supplementary Figure S5-1). While, in HC/NC comparison group, differential phosphoproteins involved GABAergic synapse signaling pathway and GABA (B) receptors were further downregulated in HC group (Supplementary Figure S5-2). Thus, salt load primarily diminished the inhibitory effect of GABAergic neurons, leading to significant hypertension. Differential phosphoproteins of the comparison group HC/NC also took part in the signaling pathway of aldosterone synthesis and secretion, which verified the crucial role of ‘aldosterone-MR-ENaC-ouabain pathway’ in salt-induced hypertension. Other signaling pathways, such as the Hippo signaling pathway, have not been reported in the central nervous system. Still, they could be a potential mechanism for high salt-induced central nervous system lesions in CKD rats. Further research would be required to investigate these distinct phosphoproteins and pathways.

## 5. Conclusion

Salt-induced phosphoproteomic changes in the SFO of CKD model rats were investigated using iTRAQ and LC-MS/MS. A total of 6808 unique phosphopeptides were identified, corresponding to 2661 phosphoproteins. 418 phosphorylated peptides (168 upregulated and 250 downregulated) were differentially expressed in NC/NS comparison group. There were 278 salt-induced differential phosphorylated peptides (154 upregulated and 124 downregulated) in HC/NC comparison group. Protective molecular GAP43 and Hsp27 significantly upregulated in response to SFO lesion caused by salt-load. Differential phosphoproteins closely linked in functional were synapse proteins and MAP that were important for brain’s structure and function. Moreover, the KEGG signaling pathways of differential phosphoproteins suggested that high salt aggravated renal injury primarily by decreasing inhibitory neurotransmitters, increasing aldosterone synthesis, and enhancing SNA. In conclusion, this study provides valuable insights into the mechanism of high salt-induced CKD progression from the perspective of the central nervous system.

## Supplementary Material

Supplemental Material
