# Supplementary material for: Salt-induced phosphoproteomic changes in the subfornical organ in rats with chronic kidney disease
Source: Ren Fail. 2023 Jan 30;45(1):2171886. doi: 10.1080/0886022X.2023.2171886 (PMC9888458; doi:10.1080/0886022X.2023.2171886)
Supplement: Supplemental Material [file IRNF_A_2171886_SM9865.zip › 2171886/Copy of Supplementary_Table_S1.pdf]

Supplementary Table S1-2. Complete list of phosphopeptides identi

Sequence	Accessions	Modifications
rFsFk	F1LMW7;Q5U2P5	N-Term(iTRAQ4plex); S3(Phospho); K5(iTRA
gDGGSTTGLsATPPASLP	F1M9C3	N-Term(iTRAQ4plex); S10(Phospho); K25(iT
iLQEYQVQYTPQGDsDD	A0A0G2K1T0	N-Term(iTRAQ4plex); S15(Phospho); K19(iT
lSGLsFkR	A0A0G2K613	N-Term(iTRAQ4plex); S5(Phospho); K7(iTRA
nQWQLsLDDLkk	P13233	N-Term(iTRAQ4plex); S6(Phospho); K11(iTR
aTTPQLTDVSEDLDQQsL	A0A0G2K1U5	N-Term(iTRAQ4plex); S17(Phospho); S20(Ph
rRGsGSVDETLFALPAAS	F1LQN3	N-Term(iTRAQ4plex); S4(Phospho); K28(iTR
sPAEVksPVEAksPAEAk	F1LRZ7	N-Term(iTRAQ4plex); K6(iTRAQ4plex); S7(P
nSQEDsEDsEEkDVk	A0A0G2K7X3	N-Term(iTRAQ4plex); S6(Phospho); S9(Phos
sLGGESsGGTTPVGSFH	F1LQS9	N-Term(iTRAQ4plex); S7(Phospho)
tELsPSFINPNPLEWFAGE	F1LRL9	N-Term(iTRAQ4plex); S4(Phospho); K27(iTR
gGStPPALGDLFAGGFPV	Q9Z0G8	N-Term(iTRAQ4plex); T4(Phospho)
fDWGPAPPTTFkPNsPDL	F1LS01	N-Term(iTRAQ4plex); K12(iTRAQ4plex); S15
gsGSVDETLFALPAASEP	F1LQN3	N-Term(iTRAQ4plex); S2(Phospho); K26(iTR
sPAEAksPAEVksPVEAk	F1LRZ7	N-Term(iTRAQ4plex); K6(iTRAQ4plex); S7(P
aISAFGPsASINGLVk	Q8CGU4	N-Term(iTRAQ4plex); S8(Phospho); K16(iTR
fVQWQSsI	P0C5X8	N-Term(iTRAQ4plex); S7(Phospho)
fsWGAEGQkPGFGYGGR	P02688	N-Term(iTRAQ4plex); S2(Phospho); K9(iTRA
sLVGsWLk	Q4G008	N-Term(iTRAQ4plex); S5(Phospho); K8(iTRA
nQWQLsLDDLk	P13233	N-Term(iTRAQ4plex); S6(Phospho); K11(iTR
sPVEAksPAEVksPVTVk	F1LRZ7	N-Term(iTRAQ4plex); K6(iTRAQ4plex); S7(P
rLsVELTSSLFR	A0A0G2JT93	N-Term(iTRAQ4plex); S3(Phospho)
tFsATVR	Q9WU49	N-Term(iTRAQ4plex); S3(Phospho)
dLWPmVSPEDTQSLsFsE	P34926;Q63330	N-Term(iTRAQ4plex); M5(Oxidation); S15(Ph
lIAHGDADVMVAGGTDsc	G3V6R7	N-Term(iTRAQ4plex); S17(Phospho); C18(Ca
mFPEPTNsGDEGEPELD	Q5XII5	N-Term(iTRAQ4plex); M1(Oxidation); S8(Pho
mDSASQDINLNsPNkGVL	Q6PCT3	N-Term(Acetyl); S12(Phospho); K15(iTRAQ4
vQSLEGEkLsPksDIsPLTF	P15205;F1LRL9	N-Term(iTRAQ4plex); K8(iTRAQ4plex); S10(
lSGLsFk	A0A0G2K613	N-Term(iTRAQ4plex); S5(Phospho); K7(iTRA
kGssEEsVDEDRGLAPEP	A0A0G2K6R9;F1M9N9	K1(iTRAQ4plex); N-Term(iTRAQ4plex); S3(P
tQLAsWSDPTEETGPVA	A0A0G2K1J5	N-Term(iTRAQ4plex); S5(Phospho); K27(iTR
qMxMsPPPGNAGPVIMSL	O08729	N-Term(iTRAQ4plex); X3(D); S5(Phospho); K
sAPPsPPPPGTR	Q6MG88	N-Term(iTRAQ4plex); S1(Phospho); S5(Phos
vkEGMsIVEAMER	P10111	N-Term(iTRAQ4plex); K2(iTRAQ4plex); S6(P
qGFSDkEsPVSDLTSDLY	P15205;F1LRL9	N-Term(iTRAQ4plex); K6(iTRAQ4plex); S8(P
gPQFGsEVELR	Q8CJ04	N-Term(iTRAQ4plex); S6(Phospho)
sLSSsPVkk	B1WC33	N-Term(iTRAQ4plex); S5(Phospho); K8(iTRA
assRPRPDDLEI	P08050	N-Term(iTRAQ4plex); S2(Phospho); S3(Phos
gEEQEHSEPEEGsPR	F1M4M5	N-Term(iTRAQ4plex); S13(Phospho)
dLWPMVSPEDTQSLsFsE	P34926;Q63330	N-Term(iTRAQ4plex); S15(Phospho); S17(Ph
lNWLSVDFNNWkDWED	P83868	N-Term(iTRAQ4plex); K12(iTRAQ4plex); S18
sRsPLAIR	A0A0G2K2M9	N-Term(iTRAQ4plex); S1(Phospho); S3(Phos
eSDFcVEsEEktSAEk	Q66H86	N-Term(iTRAQ4plex); C5(Carbamidomethyl);
aQsLPSVPLScATYSEAL	P0C548	N-Term(iTRAQ4plex); S3(Phospho); C11(Ca
lSGFsFkk	F1LMW7	N-Term(iTRAQ4plex); S5(Phospho); K7(iTRA
lAQATSSSSSTSAAAASS	F1LPG3	N-Term(iTRAQ4plex); M27(Oxidation); S33(P
rLsAEGDk	A0A0G2K9R0	N-Term(iTRAQ4plex); S3(Phospho); K8(iTRA
icsFEEAk	A0A0G2K7T5	N-Term(iTRAQ4plex); C2(Carbamidomethyl);
sFFtDGSLDSWGTSEDAE	F1M2D4	N-Term(iTRAQ4plex); T4(Phospho); K22(iTR
gEGEEDHEsPSSGR	Q5XIK5	N-Term(iTRAQ4plex); S9(Phospho)
dLGsDFQPPDFRDLLEW	A0A0G2KA88	N-Term(iTRAQ4plex); S4(Phospho)

Information Classification: General

lRSsMPGVR	P31000	N-Term(iTRAQ4plex); S4(Phospho)
gPGGsPSGLQk	Q99MC0	N-Term(iTRAQ4plex); S5(Phospho); K11(iTR
sEPQPEEGsPAAGQk	Q1LZ53	N-Term(iTRAQ4plex); S9(Phospho); K15(iTR
tGPVEEAQLsVEEk	Q66LH8	N-Term(iTRAQ4plex); S10(Phospho); K14(iT
iMAPPERDksPSEGDVAP	D3ZBU7	N-Term(iTRAQ4plex); K9(iTRAQ4plex); S10(
lHLESPQPsPkSsPQEAGN	Q5PQP4	N-Term(iTRAQ4plex); S9(Phospho); K11(iTR
iQPQLPDEDGNEsDkEDE	A0A0G2K0E2	N-Term(iTRAQ4plex); S13(Phospho); K15(iT
rQsQQLPEEDcMQLNPSF	Q5FVJ3	N-Term(iTRAQ4plex); S3(Phospho); C11(Car
eAEALLQSMGLTTDSPIVI	D3ZU74	N-Term(iTRAQ4plex); S23(Phospho); K27(iT
dFSGAGGIGVFSEssSVAS	A0A0G2K207	N-Term(iTRAQ4plex); S14(Phospho); S15(Ph
qGFsDkEsPVSDLTSDLY(	P15205;F1LRL9	N-Term(iTRAQ4plex); S4(Phospho); K6(iTRA
lQPEQEsPk	E9PST5	N-Term(iTRAQ4plex); S7(Phospho); K9(iTRA
sNsLLESTDYWLQNQR	P0C6C0	N-Term(iTRAQ4plex); S3(Phospho)
ssPFkVsPLSFGR	Q66H98	N-Term(iTRAQ4plex); S2(Phospho); K5(iTRA
dGDSGsLGSPSASR	D4A8L3	N-Term(iTRAQ4plex); S6(Phospho)
kVVEPANsDsDsELGNIPk	Q14TE9	K1(iTRAQ4plex); N-Term(iTRAQ4plex); S8(P
lENEGsDEDIETDVLYSPQ	Q8VIJ5	N-Term(iTRAQ4plex); S6(Phospho); K23(iTR
qFLIsPPAsPPVGWk	Q6IN33	N-Term(iTRAQ4plex); S5(Phospho); S9(Phos
sHPQSsPVLERPGR	A0JPJ0	N-Term(iTRAQ4plex); S6(Phospho)
eVAkEsPk	P12839	N-Term(iTRAQ4plex); K4(iTRAQ4plex); S6(P
sPVEAksPAEAksPASVk	F1LRZ7	N-Term(iTRAQ4plex); K6(iTRAQ4plex); S7(P
lVLASIDQADFQGFtYVNP	P63319	N-Term(iTRAQ4plex); T15(Phospho); S31(Ph
rPEFFTFGGNTAALtPLsP	F1M9W9	N-Term(iTRAQ4plex); T15(Phospho); S18(Ph
tPGDFNyAYQkPESTTESI	P15205;F1LRL9	N-Term(iTRAQ4plex); Y7(Phospho); K11(iTR
rVPGsSGHLHk	A0A0G2K007	N-Term(iTRAQ4plex); S5(Phospho); K11(iTR
tSSESIVsVPAssTSGSPSI	A0A0G2JTB5	N-Term(iTRAQ4plex); S8(Phospho); S12(Pho
lSGFsFk	F1LMW7	N-Term(iTRAQ4plex); S5(Phospho); K7(iTRA
tSPDSEQPPASEPVWERI	Q80X08	N-Term(iTRAQ4plex); T1(Phospho)
rPsANcDPYAVTEAIVR	P09606	N-Term(iTRAQ4plex); S3(Phospho); C6(Carb
lWAGPDPsSPTLTMSAEA	D3ZFG7	N-Term(iTRAQ4plex); S8(Phospho); K19(iTR
aPDGsLDGEEAk	F1M9X4	N-Term(iTRAQ4plex); S5(Phospho); K12(iTR
qGFsDkEsPVSDLTSDLY(	P15205;F1LRL9	N-Term(iTRAQ4plex); S4(Phospho); K6(iTRA
sTEESLsEDVFtESELSPIF	A0A0G2JZ50	N-Term(iTRAQ4plex); S7(Phospho); T12(Pho
eVNTcNPSTITGTLSRLsL	A0A0G2K6N2	N-Term(iTRAQ4plex); C5(Carbamidomethyl);
vAAALPGMESTQDRsREI	A0A0H2UHP9	N-Term(iTRAQ4plex); S15(Phospho); K23(iT
kAEGAALSNATGAVESts(	Q9Z0W5;A0A0G2JWR2	K1(iTRAQ4plex); N-Term(iTRAQ4plex); T17(
tQsIYDDTk	Q8K5B5	N-Term(iTRAQ4plex); S3(Phospho); K9(iTRA
aDskEsLkAAPk	P34926;Q63330	N-Term(iTRAQ4plex); S3(Phospho); K4(iTRA
sESSAGVcVPLsTSPQVS	P0C0R5	N-Term(iTRAQ4plex); C8(Carbamidomethyl);
sSkGSPtGsSPNNASELSL	Q4KLM7	N-Term(iTRAQ4plex); K3(iTRAQ4plex); T7(P
sPVEAksPAEAk	F1LRZ7	N-Term(iTRAQ4plex); K6(iTRAQ4plex); S7(P
tLDGEAGsPVGPEALPAD	Q6P730	N-Term(iTRAQ4plex); S8(Phospho)
fSEMMDHmGGDEDADLF	Q5PQL9	N-Term(iTRAQ4plex); M8(Oxidation); S26(Ph
aALPAGEGEsPEGAkIDVI	A0A0G2K5Z4	N-Term(iTRAQ4plex); S10(Phospho); K15(iT
gTWPNSVLGEVTEVDSS(	Q6RJR6	N-Term(iTRAQ4plex); T23(Phospho); K35(iT
iGsDPLAYEPk	P26431	N-Term(iTRAQ4plex); S3(Phospho); K11(iTR
eQPAGLPsPGER	A0A0G2K0J7	N-Term(iTRAQ4plex); S8(Phospho)
wmLsLAk	P13233	N-Term(iTRAQ4plex); M2(Oxidation); S4(Pho
eIPFNEDPNPNTHSsGPS	D4A5B3	N-Term(iTRAQ4plex); S15(Phospho); K22(iT
fATLsLHDR	Q5BJW6	N-Term(iTRAQ4plex); S5(Phospho)
tLEEQNQLLSAELGGLRA	M0R3Z1	N-Term(iTRAQ4plex); S20(Phospho); T23(Ph
sIQEIQELDkDDESLR	Q5XI73	N-Term(iTRAQ4plex); S1(Phospho); K10(iTR
tHsDASDDEAFPTSk	Q6TUE6	N-Term(iTRAQ4plex); S3(Phospho); K15(iTR
mQFsFEGPEk	A0A0G2JUG7	N-Term(iTRAQ4plex); S4(Phospho); K10(iTR
aMSMASILTNtMEELEEsF	A0A0G2K207	N-Term(iTRAQ4plex); T11(Phospho); S18(Ph

Information Classification: General

dRsLsPR	F1M7S0;F1M4A0	N-Term(iTRAQ4plex); S3(Phospho); S5(Phos
aEDSsVEQLSTEIEPSREE	Q5QD51	N-Term(iTRAQ4plex); S5(Phospho); K25(iTR
nDFEQGsQGQEGDSNAE	G3V7X5	N-Term(iTRAQ4plex); S7(Phospho); K21(iTR
lHLEsPQPsPk	Q5PQP4	N-Term(iTRAQ4plex); S5(Phospho); S9(Phos
sDsPPAPLRPWVPLR	Q63638	N-Term(iTRAQ4plex); S3(Phospho)
kLEcLPPEPsPDDPDSVk	Q5BK32	N-Term(iTRAQ4plex); K1(iTRAQ4plex); C4(C
aDEDksDTEGkPAR	M0RDJ7	N-Term(iTRAQ4plex); K5(iTRAQ4plex); S6(P
qLsPESLGTLQFGELNLG	P34926;Q63330	N-Term(iTRAQ4plex); S3(Phospho); K19(iTR
lISGPLsPMSR	Q6MG48	N-Term(iTRAQ4plex); S7(Phospho)
vIyDFIEk	F1LSG0	N-Term(iTRAQ4plex); Y3(Phospho); K8(iTRA
sPsWEPFR	G3V913	N-Term(iTRAQ4plex); S3(Phospho)
gLLHGWsQSsTEEVPR	F1LPM3	N-Term(iTRAQ4plex); S7(Phospho); S10(Pho
ePVPAWEGksPEQEVR	P34926	N-Term(iTRAQ4plex); K9(iTRAQ4plex); S10(
rAsLSDIGFGk	O35832	N-Term(iTRAQ4plex); S3(Phospho); K11(iTR
sGLsLEELR	A0A0G2KA12	N-Term(iTRAQ4plex); S4(Phospho)
lATTVsAPDLk	A0A0G2JW88	N-Term(iTRAQ4plex); S6(Phospho); K11(iTR
kSLDsDEsEDEDDDYQQk	Q62785	N-Term(iTRAQ4plex); K1(iTRAQ4plex); S5(P
eHAADGPQLQSLAQAEA	Q5QD51	N-Term(iTRAQ4plex); S18(Phospho); K25(iT
sLmsSPEDLTkDFEELkAE	P15205;F1LRL9	N-Term(iTRAQ4plex); M3(Oxidation); S4(Pho
sPASVksPGEAksPAEAk	F1LRZ7	N-Term(iTRAQ4plex); K6(iTRAQ4plex); S7(P
vLQkPSVFGNDsDDDEAS	Q4FZU3	N-Term(iTRAQ4plex); K4(iTRAQ4plex); S12(
lDPAQsASRENLLEEQGS	P16086	N-Term(iTRAQ4plex); S6(Phospho)
aksPQPPVEEEDEHFDDT	Q63555	N-Term(iTRAQ4plex); K2(iTRAQ4plex); S3(P
kGssGNASEVSVAcLTER	Q2KN99	K1(iTRAQ4plex); N-Term(iTRAQ4plex); S3(P
aSAEGEATAVVSPGVTQ	P15205;F1LRL9	N-Term(iTRAQ4plex); C24(Carbamidomethyl
vEAkEEsEESDEDmGFGL	P19945	N-Term(iTRAQ4plex); K4(iTRAQ4plex); S7(P
ssPPPLPAk	D3ZNS1	N-Term(iTRAQ4plex); S2(Phospho); K9(iTRA
eGsPLkEESLAR	A0A0G2K774	N-Term(iTRAQ4plex); S3(Phospho); K6(iTRA
aSPITNDGEDEFVPsDGID	Q14TE9	N-Term(iTRAQ4plex); S15(Phospho); K20(iT
nSSLSPGLNTSNGDGSET	F1M787	N-Term(iTRAQ4plex); T20(Phospho); K29(iT
vQsLEGEkLsPk	P15205;F1LRL9	N-Term(iTRAQ4plex); S3(Phospho); K8(iTRA
gGRsPDEVTLTSIVPTR	G3V9N7	N-Term(iTRAQ4plex); S4(Phospho)
yLsFTPPEkDGFPSGTPAI	Q64303	N-Term(iTRAQ4plex); S3(Phospho); K9(iTRA
wWQQEEQLEPEEsREEV	O35314	N-Term(iTRAQ4plex); S13(Phospho)
fsGGWR	Q6MG08	N-Term(iTRAQ4plex); S2(Phospho)
lDNTPAsPPRSPAEPSDIP	X4YHC6	N-Term(iTRAQ4plex); S7(Phospho); K22(iTR
sDDTQPsPERPVPR	A0A0G2K4J8	N-Term(iTRAQ4plex); S7(Phospho)
aALIPAGLSDGQFYsPPES	Q5U205	N-Term(iTRAQ4plex); S15(Phospho); S23(Ph
sSSALQkLAEENIFsVDskS	A0A0G2K2J8	N-Term(iTRAQ4plex); K7(iTRAQ4plex); S15(
sPPIPAkPRsPSR	Q63327	N-Term(iTRAQ4plex); K7(iTRAQ4plex); S10(
kEEVksPVEEVk	F1LRZ7	K1(iTRAQ4plex); N-Term(iTRAQ4plex); K5(iT
vTEFNNVTPLSEEEVASIk	A0A0G2JW88	N-Term(iTRAQ4plex); K19(iTRAQ4plex); S22
iTPAIPVVGGILFFFVMGT	Q2TGJ5	N-Term(iTRAQ4plex); T23(Phospho); S26(Ph
tSSLPEsSPSk	F1LR10	N-Term(iTRAQ4plex); S7(Phospho); K11(iTR
sPESLSSPAMEDLAVEWI	P34926;Q63330	N-Term(iTRAQ4plex); S1(Phospho); K20(iTR
vAGEAAETDsEPEPEPR	D4A3V6	N-Term(iTRAQ4plex); S10(Phospho)
tTktPEDGGYScEITEk	P15205;F1LRL9	N-Term(iTRAQ4plex); K3(iTRAQ4plex); T4(P
dLWPMVsPEDtQSLSFSE	P34926;Q63330	N-Term(iTRAQ4plex); S7(Phospho); T11(Pho
fHDsSSPLLTSGTSVAELF	Q5JC29	N-Term(iTRAQ4plex); S4(Phospho); K23(iTR
eSETFSDSSPIEIIDEFPTF	F1LQN3	N-Term(iTRAQ4plex); K24(iTRAQ4plex); S27
aDILAHDPLGEFFADGEA	Q8BFS3	N-Term(iTRAQ4plex); S33(Phospho); K41(iT
eTLFSPVSTEEMLPRPPs	Q63424	N-Term(iTRAQ4plex); S18(Phospho); K21(iT
vSPVLPSSSLEsPQDEDk	Q63358	N-Term(iTRAQ4plex); S12(Phospho); K18(iT
sksESDASSLDAk	A0A0G2K6R9;F1M9N9	N-Term(iTRAQ4plex); K2(iTRAQ4plex); S3(P
vSIANMLAWSSEslk	F1LR18	N-Term(iTRAQ4plex); S13(Phospho); K15(iT

rIsIVEScFGAAGQPLTIPG	B1WBV4	N-Term(iTRAQ4plex); S3(Phospho); C8(Carb
sPASVksPGEAk	F1LRZ7	N-Term(iTRAQ4plex); K6(iTRAQ4plex); S7(P
rEsDESGESAPEEGGEGA	Q5FWU3	N-Term(iTRAQ4plex); S3(Phospho)
sAQEVEAVAsLATR	F1LQJ2	N-Term(iTRAQ4plex); S10(Phospho)
sPAEAkPPAEAk	F1LRZ7	N-Term(iTRAQ4plex); S1(Phospho); K6(iTRA
rRGsGsVDETLFALPAASE	F1LQN3	N-Term(iTRAQ4plex); S4(Phospho); S6(Phos
sLsGGPLGR	B5DF40	N-Term(iTRAQ4plex); S3(Phospho)
wVHFSDAsPEHVTPELTP	F1M1Y0	N-Term(iTRAQ4plex); S8(Phospho)
eHHPEEEFsGSEVEEVPE	F1M842	N-Term(iTRAQ4plex); S9(Phospho); C21(Ca
dMLAsDEEEEPSkVEk	Q9Z2J4	N-Term(iTRAQ4plex); S5(Phospho); K13(iTR
vLIEDtDDEANT	A0A0G2JXZ7	N-Term(iTRAQ4plex); T6(Phospho)
kQSAGPNsPTGGGGGGG	A0A0G2K9R0	N-Term(iTRAQ4plex); K1(iTRAQ4plex); S8(P
aTLLPEGDRsPEEADFPD	A0JPP6	N-Term(iTRAQ4plex); S10(Phospho); K24(iT
vSEHSsPEEESSPHQR	Q6J4I0	N-Term(iTRAQ4plex); S6(Phospho)
gLsLTETELEELR	D4AAR7	N-Term(iTRAQ4plex); S3(Phospho)
gNEQNPEEDAQsDVtEGH	Q63564	N-Term(iTRAQ4plex); S12(Phospho); T15(Ph
tGsSTNNNEEEk	Q6MFX8	N-Term(iTRAQ4plex); S3(Phospho); K12(iTR
dIAFIMDDFQHAMsDSEA	Q9JKS6;D3Z9C7	N-Term(iTRAQ4plex); S14(Phospho)
tESDsDSDkNsDHSGLk	A0A0G2JYC7	N-Term(iTRAQ4plex); S5(Phospho); K9(iTRA
tQSSscERDSPSTSR	D4A8M2	N-Term(iTRAQ4plex); S5(Phospho); C6(Carb
lYPDMLQSSsQLTHSR	A0A0G2K3B8	N-Term(iTRAQ4plex); S10(Phospho)
qADIGsPTNAk	D4A702	N-Term(iTRAQ4plex); S6(Phospho); K11(iTR
eEEPEVEksPVksPEAk	P12839	N-Term(iTRAQ4plex); K8(iTRAQ4plex); S9(P
eFcIPTSSTIEQQNsTR	B8K2Q4	N-Term(iTRAQ4plex); C3(Carbamidomethyl);
hVsDPGLPGk	A0A0G2K3B8	N-Term(iTRAQ4plex); S3(Phospho); K10(iTR
sTEPsHSRDAsPVGVk	Q71RJ2	N-Term(iTRAQ4plex); S5(Phospho); S11(Pho
skDPGsPQPNQEAGADR	F1M4V3	N-Term(iTRAQ4plex); K2(iTRAQ4plex); S6(P
sTEAsPSRDAsPVGLk	Q8VHW9	N-Term(iTRAQ4plex); S5(Phospho); S11(Pho
sPTPVHsPDADRPGPLFV	A0A0U1RS13	N-Term(iTRAQ4plex); S7(Phospho)
eGYPLPYEPPAtDGSPGP	G3V7T8	N-Term(iTRAQ4plex); T12(Phospho)
mSQPGsPSPk	E9PU01	N-Term(iTRAQ4plex); M1(Oxidation); S6(Pho
iPFLESLGLPsPPRsPVk	A0A0H2UHB7	N-Term(iTRAQ4plex); S11(Phospho); S15(Ph
rsPSADQSLDDsPSk	F1M6X3	N-Term(iTRAQ4plex); S2(Phospho); S12(Pho
hPsHSTSPSGPGDEVAR	Q6AY65	N-Term(iTRAQ4plex); S3(Phospho)
ePGEETDkPsPEASSEDT	F1M9G3	N-Term(iTRAQ4plex); K8(iTRAQ4plex); S10(
lFEcMGtGEQEk	D3Z980	N-Term(iTRAQ4plex); C4(Carbamidomethyl);
gFEEEHkDsDDDssDDEQ	D3ZMS1	N-Term(iTRAQ4plex); K7(iTRAQ4plex); S9(P
qRsDDESPSTSSGSSDAD	A0A0G2K964	N-Term(iTRAQ4plex); S3(Phospho)
vFsVTHIk	Q02563	N-Term(iTRAQ4plex); S3(Phospho); K8(iTRA
vDNSSLTGEsEPQtRSPD	P06687	N-Term(iTRAQ4plex); S10(Phospho); T14(Ph
qMGstEGESDVPVLVTSE	A0A0G2K6R9;F1M9N9	N-Term(iTRAQ4plex); S4(Phospho); T5(Phos
lFsVVSR	A0A0G2JSH6	N-Term(iTRAQ4plex); S3(Phospho)
vGIVEPSsAtSGDSDDAAP	D3ZJG4	N-Term(iTRAQ4plex); S8(Phospho); T10(Pho
rAQtPPISSLPAsPSDEVG	A0A0G2JTT6	N-Term(iTRAQ4plex); T4(Phospho); S13(Pho
wVHFSDAsPEHVtPELTPP	F1M1Y0	N-Term(iTRAQ4plex); S8(Phospho); T13(Pho
aEsGGkGER	Q02563	N-Term(iTRAQ4plex); S3(Phospho); K6(iTRA
kDEDsDDESQSSHAGk	A0A0G2K266	N-Term(iTRAQ4plex); K1(iTRAQ4plex); S5(P
eAEASAPGVQEEsSSNR	D3ZB78	N-Term(iTRAQ4plex); S13(Phospho)
kGSLDsDNDDEkDPQHSL	A0A0H2UHB7	K1(iTRAQ4plex); N-Term(iTRAQ4plex); S6(P
eARsPQPNk	B2RYB3;A0A0G2K4F6	N-Term(iTRAQ4plex); S4(Phospho); K9(iTRA
eLsEEEEDEEEEEEEEEE	G3V6L7	N-Term(iTRAQ4plex); S3(Phospho)
nTEGsYQcScPR	F1M5Q4	N-Term(iTRAQ4plex); S5(Phospho); C8(Carb
hSsPHQSEDEEEPR	A2RRU1	N-Term(iTRAQ4plex); S3(Phospho)
asPDGSQTVR	G3V849	N-Term(iTRAQ4plex); S2(Phospho)
gEGAQDEEEGGASsDAT	Q02563	N-Term(iTRAQ4plex); S14(Phospho)

sFEsDAEDHkPLk	A0A0G2K3Q5	N-Term(iTRAQ4plex); S4(Phospho); K10(iTR
vIsQTNLITTVtPEkk	G3V874;A0A0G2K1Q9	N-Term(iTRAQ4plex); S3(Phospho); T12(Pho
dLDLLASVPsPSSVSR	A0A0G2K9N0	N-Term(iTRAQ4plex); S10(Phospho)
aDsRsESPTck	P59649	N-Term(iTRAQ4plex); S3(Phospho); S5(Phos
hSAILAsPNPDEk	P32851	N-Term(iTRAQ4plex); S7(Phospho); K13(iTR
qkEEsPQGSEEk	A0A0G2K6R9;F1M9N9	N-Term(iTRAQ4plex); K2(iTRAQ4plex); S5(P
gDMANSsIEFHPkPQQQR	A0A0G2K9Q6	N-Term(Acetyl); S7(Phospho); K13(iTRAQ4p
lETTPtTSPLPER	P70587	N-Term(iTRAQ4plex); T6(Phospho)
eFVSSDEsSSGENk	Q04931	N-Term(iTRAQ4plex); S8(Phospho); K14(iTR
sGsPSDNSGAEEmEVAL/	P47196	N-Term(iTRAQ4plex); S3(Phospho); M13(Ox
sEVSsPIQR	E9PTG5	N-Term(iTRAQ4plex); S5(Phospho)
sAtLPLPAR	F1M820	N-Term(iTRAQ4plex); T3(Phospho)
lHQVyFDAPScVk	A0A0G2KAD8	N-Term(iTRAQ4plex); Y5(Phospho); C11(Car
gPGDGAEEDEsNTGGR	Q5FVI4	N-Term(iTRAQ4plex); S11(Phospho)
eRsPPLtPk	F1M9F9	N-Term(iTRAQ4plex); S3(Phospho); T7(Phos
vAILtDDEEEQk	A0A0G2JW92	N-Term(iTRAQ4plex); T5(Phospho); K12(iTR
rDsSEGPPGSEGDGGGQ	Q63553	N-Term(iTRAQ4plex); S3(Phospho); K19(iTR
gFsFVNPk	D4A0U0	N-Term(iTRAQ4plex); S3(Phospho); K8(iTRA
tPEELEDDsDFEQEDYDV	D4A6H8	N-Term(iTRAQ4plex); S9(Phospho)
sRsPPPVSk	Q6PDU1	N-Term(iTRAQ4plex); S1(Phospho); S3(Phos
iLLENLGEASSQPsPTQF/	D4A4K4	N-Term(iTRAQ4plex); S14(Phospho)
lLDRsPSR	E2E1S0	N-Term(iTRAQ4plex); S5(Phospho)
iPVPsSPVPIATIDLVQQQ1	A0A0G2JW92	N-Term(iTRAQ4plex); S5(Phospho)
gDGTAVDkLELSEDsPNS	M0R5H1	N-Term(iTRAQ4plex); K8(iTRAQ4plex); S15(
sQGEEVDFAR	O08557	N-Term(iTRAQ4plex); S1(Phospho)
vGsPLTLTDAQTR	F1M2K6	N-Term(iTRAQ4plex); S3(Phospho)
rGDsVDcPPEGR	F1MAS1	N-Term(iTRAQ4plex); S4(Phospho); C7(Carb
qNPEQsADEDAEk	B5DFC8	N-Term(iTRAQ4plex); S6(Phospho); K13(iTR
eDAPPEDkEsESEAk	Q91XU8	N-Term(iTRAQ4plex); K8(iTRAQ4plex); S10(
aAEEARGsEALGGGGGG	P31647	N-Term(iTRAQ4plex); S8(Phospho)
aGsAETESAAk	P07936	N-Term(iTRAQ4plex); S3(Phospho); K11(iTR
rsPSSEDDkDER	A0A0G2JX20	N-Term(iTRAQ4plex); S2(Phospho); K9(iTRA
wHQLQNENHVSsD	F1LPB9	N-Term(iTRAQ4plex); S12(Phospho)
ksDAGISSADAPR	Q562A6	N-Term(iTRAQ4plex); K1(iTRAQ4plex); S2(P
gsPGGDLGAk	F1M110	N-Term(iTRAQ4plex); S2(Phospho); K10(iTR
iPFLESLGLPsPPRSPVk	A0A0H2UHB7	N-Term(iTRAQ4plex); S11(Phospho); K18(iT
eGsRGEEEAGQEEGGSR	A0A0G2JVW5	N-Term(iTRAQ4plex); S3(Phospho)
mDVSESLISSsPVk	D3ZR47	N-Term(iTRAQ4plex); S11(Phospho); K14(iT
vASPSQGQVGSssPk	F1M2K6	N-Term(iTRAQ4plex); S12(Phospho); S13(Ph
hANNTAsMHR	P20272	N-Term(iTRAQ4plex); S7(Phospho)
aQDGAAMEMQPLksEEG	P11505	N-Term(iTRAQ4plex); K13(iTRAQ4plex); S14
iLMSLsAk	D3ZZX1	N-Term(iTRAQ4plex); S6(Phospho); K8(iTRA
lAtPEDkQDIDk	D3ZT07	N-Term(iTRAQ4plex); T3(Phospho); K7(iTRA
rPSAsSPNNTAAk	F1LWX5	N-Term(iTRAQ4plex); S5(Phospho); K13(iTR
vSEEAESQQWDTSkGDQ	G3V6S0	N-Term(iTRAQ4plex); K14(iTRAQ4plex); S19
vAGGsGsESPLLEGR	B2GV22	N-Term(iTRAQ4plex); S5(Phospho); S7(Phos
dMTsEQLDEILR	P06686	N-Term(iTRAQ4plex); S4(Phospho)
aQTAPTktSPR	A0A0A0MXY4	N-Term(iTRAQ4plex); K7(iTRAQ4plex); T8(P
lQEAANYIEsPDRETILDPI	F1M031	N-Term(iTRAQ4plex); S10(Phospho)
gNkHDDGTQsDSENAGVI	A0A0G2K315	N-Term(iTRAQ4plex); K3(iTRAQ4plex); S10(
eNPPVEDssDEDDRR	B2RYD7	N-Term(iTRAQ4plex); S8(Phospho); S9(Phos
tPTPLMANDStETSEAGEI	M0R3N4	N-Term(iTRAQ4plex); T11(Phospho); K30(iT
vIHDNFGIVEGLmTTVHAI	M0R660	N-Term(iTRAQ4plex); M13(Oxidation); T22(P
gEGAQDEEEGGAssDATI	Q02563	N-Term(iTRAQ4plex); S13(Phospho); S14(Ph
cMScSPYREsQEk	F1LMY3	N-Term(iTRAQ4plex); C1(Carbamidomethyl);

sPPGHPHAR	P0C5E3	N-Term(iTRAQ4plex); S1(Phospho)
akDQPDGTSLsPVQsPSQ	A0A140TA95	N-Term(iTRAQ4plex); K2(iTRAQ4plex); S11(
rAsDGGANIQLHAQQLLk	M0RD40	N-Term(iTRAQ4plex); S3(Phospho); K18(iTR
sIVTAEVsSMPAck	D3ZES2	N-Term(iTRAQ4plex); S8(Phospho); C13(Car
iTELDkDLEEVTMQLQDtP	A0A0A0MXV8	N-Term(iTRAQ4plex); K6(iTRAQ4plex); T18(
vPVTYLELLs	D4A7V1	N-Term(iTRAQ4plex); S10(Phospho)
sFDDPIVQTER	B4F772	N-Term(iTRAQ4plex); S1(Phospho)
vSGAEDGERSDskQDk	P30680	N-Term(iTRAQ4plex); S12(Phospho); K13(iT
sLDSVDR	G3V7T8	N-Term(iTRAQ4plex); S1(Phospho)
yTIGDIFVHsPQR	F1M8G8	N-Term(iTRAQ4plex); S10(Phospho)
fLLSSAAAAAASSASsPAT	D3ZL45	N-Term(iTRAQ4plex); S16(Phospho)
kDDSHsAEDsEDEkDDHk	A0A0G2K7X3	N-Term(iTRAQ4plex); K1(iTRAQ4plex); S6(P
sSIETkPDAsPQLPk	D3ZYJ5	N-Term(iTRAQ4plex); K6(iTRAQ4plex); S10(
gAcsTPELPQFESVk	A0A0G2K0F3	N-Term(iTRAQ4plex); C3(Carbamidomethyl);
dDEkEPEEGEDDRDsAN(	G3V9R8	N-Term(iTRAQ4plex); K4(iTRAQ4plex); S15(
nSSAITsPkGsLPPAALEP(	G3V9G3	N-Term(iTRAQ4plex); S7(Phospho); K9(iTRA
iSEsGQFSDGFEDR	A9CMA6	N-Term(iTRAQ4plex); S4(Phospho)
qAAQATGsPHTsPTHGG(	A0A0G2JVM6	N-Term(iTRAQ4plex); S8(Phospho); S12(Pho
sHTGEAIAAR	D3ZT71	N-Term(iTRAQ4plex); S1(Phospho)
gSLPPAALEPQTTVIHNPY	G3V9G3	N-Term(iTRAQ4plex); K23(iTRAQ4plex); S28
mAsPPPSGPPSAAHTPFH	P34926;Q63330	N-Term(iTRAQ4plex); S3(Phospho); S20(Pho
tEEERtPNHDGGk	F1LUV9;A0A0G2K0M8	N-Term(iTRAQ4plex); T6(Phospho); K13(iTR
rDTDAYSDLsDGEk	Q568Z1	N-Term(iTRAQ4plex); S10(Phospho); K14(iT
sFASLFsD	P09951;G3V733;A0A096M	N-Term(iTRAQ4plex); S7(Phospho)
rGsATSSSNTSLT	B1H2A2	N-Term(iTRAQ4plex); S3(Phospho)
qSNVEDsPEk	A0A0G2K7H2	N-Term(iTRAQ4plex); S7(Phospho); K10(iTR
nEkHsPVSPSAk	A0A0G2K6R9;F1M9N9	N-Term(iTRAQ4plex); K3(iTRAQ4plex); S5(P
kADsRsESPTck	P59649	K1(iTRAQ4plex); N-Term(iTRAQ4plex); S4(P
eSRQEsDPEDDDVkkPAL	D3ZAY8	N-Term(iTRAQ4plex); S6(Phospho); K14(iTR
rHsIEk	D3ZEX7	N-Term(iTRAQ4plex); S3(Phospho); K6(iTRA
aAYLkDGskEPIVEMR	F1M8G9	N-Term(iTRAQ4plex); K5(iTRAQ4plex); S8(P
gEAEQsEEEGEEEEDk	P15205;F1LRL9	N-Term(iTRAQ4plex); S6(Phospho); K16(iTR
tQDGVALEIQPLNsQEGLI	Q64542	N-Term(iTRAQ4plex); S14(Phospho); K23(iT
hRPGGsPEHAR	A0A0G2KAJ5	N-Term(iTRAQ4plex); S6(Phospho)
sLsVER	A0A096MJT2	N-Term(iTRAQ4plex); S3(Phospho)
sPAEAksPVEVk	F1LRZ7	N-Term(iTRAQ4plex); S1(Phospho); K6(iTRA
skPAGsDGER	D4ABT8	N-Term(iTRAQ4plex); K2(iTRAQ4plex); S6(P
emcVSGGDHTQLTDLsPE	Q5QD51	N-Term(iTRAQ4plex); M2(Oxidation); C3(Car
tVAWVSNMPHLsADIESA	D3ZCL8	N-Term(iTRAQ4plex); S12(Phospho)
sLPDsGR	G3V7T8	N-Term(iTRAQ4plex); S5(Phospho)
sGDEGSEDEAAsGED	A0A0G2K7X3	N-Term(iTRAQ4plex); S12(Phospho)
sPGGSTMMk	Q920Q0	N-Term(iTRAQ4plex); S1(Phospho); K9(iTRA
sVESEGSSksPQGR	D3ZAB6	N-Term(iTRAQ4plex); K9(iTRAQ4plex); S10(
eGASSsEPNVSDSNAEST	P49620	N-Term(iTRAQ4plex); S6(Phospho); K20(iTR
ySDVEVPASVTGHSFASD	A0A0G2JSM7;D3ZZ99	N-Term(iTRAQ4plex); T23(Phospho); C24(Ca
rtSPsPPAR	D4A8V2	N-Term(iTRAQ4plex); T2(Phospho); S5(Phos
aTTDNsPSSk	P07936	N-Term(iTRAQ4plex); S6(Phospho); K10(iTR
kGEGDAAPSEEkAGSAEt	P07936	K1(iTRAQ4plex); N-Term(iTRAQ4plex); K12(
gGtPSAFDR	Q52KS1;P30835;P47860	N-Term(iTRAQ4plex); T3(Phospho)
sGsPAPAEPVDPNR	F1LVS1	N-Term(iTRAQ4plex); S3(Phospho)
vDIITEEMPENALPsDEDD	B5DFK6	N-Term(iTRAQ4plex); S15(Phospho); K20(iT
aSDSGPSSSSSsSSSPPR	Q07266;A0A0H2UHL9	N-Term(iTRAQ4plex); S12(Phospho)
sISSPkDtAk	Q6AYM6	N-Term(iTRAQ4plex); K6(iTRAQ4plex); T8(P
sRsLsPGR	Q810W7;D3ZAR2;D3ZL3(	N-Term(iTRAQ4plex); S3(Phospho); S5(Phos
lSSPAQsPSQk	F1M2J2	N-Term(iTRAQ4plex); S7(Phospho); K11(iTR

sSPAYcTSssDITEPEQk	D3ZCL8	N-Term(iTRAQ4plex); C6(Carbamidomethyl);
mQVDQEEPHTEEQQPQt	F1LRV4	N-Term(iTRAQ4plex); T17(Phospho); K22(iT
tQDGVALEIQPLNsQEGLI	Q64542	N-Term(iTRAQ4plex); S14(Phospho); K23(iT
qVVPFsSSV	Q9JKB7	N-Term(iTRAQ4plex); S6(Phospho)
sRsGSSSk	A0A0G2K2M9	N-Term(iTRAQ4plex); S1(Phospho); S3(Phos
dkVTDGITksPEk	A0A0U1RRX4;F1MAQ5	N-Term(iTRAQ4plex); K2(iTRAQ4plex); K9(iT
sPLAQmEEER	F1LMC7	N-Term(iTRAQ4plex); S1(Phospho); M6(Oxid
lksLNDLDQANEEQETEFI	A0A0G2JT21	N-Term(iTRAQ4plex); K2(iTRAQ4plex); S3(P
sPPEMSLLHDVGPGPAIk	D3ZBU7	N-Term(iTRAQ4plex); S1(Phospho); K18(iTR
dTEEQHPDALNWEDRPs	P57769	N-Term(iTRAQ4plex); S17(Phospho)
hSHTIGGLPESDDQAELP	F1M031	N-Term(iTRAQ4plex); S19(Phospho); K30(iT
vLGEEGsER	D4A997	N-Term(iTRAQ4plex); S7(Phospho)
dVPLGPEDPkEEDGSFDY	D3ZW56	N-Term(iTRAQ4plex); K10(iTRAQ4plex); S19
dmPHPLAGSSSEEAVGG	D4A060	N-Term(iTRAQ4plex); M2(Oxidation); S22(Ph
vTDGItkSPEk	A0A0U1RRX4;F1MAQ5	N-Term(iTRAQ4plex); T6(Phospho); K7(iTRA
eLVSSSsSGSDSDSEVEk	Q63396	N-Term(iTRAQ4plex); S7(Phospho); K18(iTR
mAsPPPSGPPSAAHTPFI	P34926;Q63330	N-Term(iTRAQ4plex); S3(Phospho); S20(Pho
aYsGSDLPSGTSGGGGA	M0RBD9	N-Term(iTRAQ4plex); S3(Phospho)
dILsDSSYVVRGNAR	A0A0G2JW59	N-Term(iTRAQ4plex); S4(Phospho)
tRsPsPTLGESLAPR	D3ZNS1	N-Term(iTRAQ4plex); S3(Phospho); S5(Phos
tsVQTEDDQLIAGQSAR	A0A0G2JYF7;D4A6H8	N-Term(iTRAQ4plex); S2(Phospho)
yNSPELDEEGYSIRPEEP	F1M1Y0	N-Term(iTRAQ4plex); S20(Phospho); K22(iT
qGDETPSTNNGsDDEk	D3ZX42	N-Term(iTRAQ4plex); S12(Phospho); K16(iT
tPsASHEEQQE	O70593	N-Term(iTRAQ4plex); S3(Phospho)
rPQsPGVsPAHSER	F1M816	N-Term(iTRAQ4plex); S4(Phospho); S8(Phos
gAPGDHGPEGSGGERHS	Q7TP42	N-Term(iTRAQ4plex); T19(Phospho)
vDGPRsPsYGR	D4A9L2	N-Term(iTRAQ4plex); S6(Phospho); S8(Phos
gsPSPAAPGPPAGPLPR	D3ZC15	N-Term(iTRAQ4plex); S2(Phospho)
eEDEEAEsPPEk	A0A0G2K7X3	N-Term(iTRAQ4plex); S8(Phospho); K12(iTR
gNPsPAPSPSPGPGPGP	Q75T81	N-Term(iTRAQ4plex); S4(Phospho)
aAGSGsPPLR	M0RCV5	N-Term(iTRAQ4plex); S6(Phospho)
qGDEEsENsVkR	M0R5U4	N-Term(iTRAQ4plex); S6(Phospho); S9(Phos
tPALsPQRPLTTQQPQSG	G3V733	N-Term(iTRAQ4plex); T1(Phospho); S5(Phos
nTADLVTTcHIHSSsDDEI	D3ZBT9	N-Term(iTRAQ4plex); C9(Carbamidomethyl);
lAsFYER	D4A133	N-Term(iTRAQ4plex); S3(Phospho)
ePPsPQGR	A0A0G2KA27	N-Term(iTRAQ4plex); S4(Phospho)
hAsEPQPGPR	G3V7T8	N-Term(iTRAQ4plex); S3(Phospho)
kFVsDDSIcVLGIQk	P47860	N-Term(iTRAQ4plex); K1(iTRAQ4plex); S4(P
sASAPAsPR	F1M787	N-Term(iTRAQ4plex); S7(Phospho)
gETPVNSTMsIGQAR	G3V8K2	N-Term(iTRAQ4plex); S10(Phospho)
aLELEPLELEGsLAGsPP	P0C6T3	N-Term(iTRAQ4plex); S12(Phospho); S16(Ph
sPsSLLIAGLHLQ	B2RYP3	N-Term(iTRAQ4plex); S3(Phospho)
aTTDNSPsskAEDGPAk	P07936	N-Term(iTRAQ4plex); S8(Phospho); S9(Phos
qLITDLVIsk	G3V733	N-Term(iTRAQ4plex); S9(Phospho); K10(iTR
aTTDNsPSSkAEDGPAkE	P07936	N-Term(iTRAQ4plex); S6(Phospho); K10(iTR
sLAAsPEGSEHWVFIER	A0A0G2K0F3	N-Term(iTRAQ4plex); S1(Phospho); S5(Phos
acEVALEEIkVETEkQsPP	D4A631	N-Term(iTRAQ4plex); C2(Carbamidomethyl);
rQsSEDDVGNAATDYLVG	Q5M9F1	N-Term(iTRAQ4plex); S3(Phospho)
sGsFQGAVR	A0A0G2K9Q6	N-Term(iTRAQ4plex); S3(Phospho)
sTSFMSVsPSk	F1M8Y4	N-Term(iTRAQ4plex); S8(Phospho); K11(iTR
aETEEAEEPEEDGEDNVS	P15205;F1LRL9	N-Term(iTRAQ4plex); K23(iTRAQ4plex); S25
sPLAQMEEER	F1LMC7	N-Term(iTRAQ4plex); S1(Phospho)
tPsLPtPPTREPk	D4A1Q2	N-Term(iTRAQ4plex); S3(Phospho); T6(Phos
gSPGSIQGTTcPGTQLGP	A0A0H2UHC0	N-Term(iTRAQ4plex); C11(Carbamidomethyl
sEAVGPEPGssGEETSDA	D3ZD52	N-Term(iTRAQ4plex); S10(Phospho); S11(Ph

Information Classification: General

qEDSQGsGEEEDFIR	Q9JKS6;D3Z9C7	N-Term(iTRAQ4plex); S7(Phospho)
rEPPsPQGR	A0A0G2KA27	N-Term(iTRAQ4plex); S5(Phospho)
mQPQSsGSLRsNAGAER	F1LVK0	N-Term(iTRAQ4plex); S6(Phospho); S11(Pho
qPWAEPEEMsEEk	Q62634	N-Term(iTRAQ4plex); S10(Phospho); K13(iT
yWLFsPR	F1M8H6	N-Term(iTRAQ4plex); S5(Phospho)
eQVEDSGAESsPk	Q63714	N-Term(iTRAQ4plex); S11(Phospho); K13(iT
gsSPEDSPAGTPHR	D4A205	N-Term(iTRAQ4plex); S2(Phospho)
ePEPLHsPDSER	A0A140TAA3	N-Term(iTRAQ4plex); S7(Phospho)
yGYTHLsTGDLLR	P39069	N-Term(iTRAQ4plex); S7(Phospho)
gVAPADsPDAPR	A0A0G2JTL7	N-Term(iTRAQ4plex); S7(Phospho)
sSLDGPIVLAIEDFHk	A0A0G2JT63	N-Term(iTRAQ4plex); S1(Phospho); K16(iTR
eTPAAsEAPSSAAk	Q05175;A0A0G2K1L8	N-Term(iTRAQ4plex); S6(Phospho); K14(iTR
aLGsPAk	B1WC26	N-Term(iTRAQ4plex); S4(Phospho); K7(iTRA
gLNNDsPERR	F1M287	N-Term(iTRAQ4plex); S6(Phospho)
gNAAGSAEQPAGPtASPF	D3ZAZ1	N-Term(Acetyl); T14(Phospho); K19(iTRAQ4
kRDsDSDADEATPTTTPR	A0A0G2K700	N-Term(iTRAQ4plex); K1(iTRAQ4plex); S4(P
sAAAAPAAEGEDTPtPPAS	Q6AXS3	N-Term(Acetyl); T15(Phospho); K21(iTRAQ4
iAFDAESEPNNSSGsMEk	A0A0G2K4S6	N-Term(iTRAQ4plex); S15(Phospho); K18(iT
eEPEDVGMTPGEASQGs	F1M403	N-Term(iTRAQ4plex); S17(Phospho)
aGLLsSPVAR	B8XCX0	N-Term(iTRAQ4plex); S5(Phospho)
gPDRtPPsEEDSAEAER	O70593	N-Term(iTRAQ4plex); T5(Phospho); S8(Phos
sDQAEEGGsEk	P12839	N-Term(iTRAQ4plex); S9(Phospho); K11(iTR
aTVsPSTDETPAGTLPGG	P34926;Q63330	N-Term(iTRAQ4plex); S4(Phospho)
tSSLPAIPNPFPELAGGAF	P0CE43	N-Term(iTRAQ4plex); S28(Phospho); K40(iT
vSSSGLDsLGR	F1LSE6	N-Term(iTRAQ4plex); S8(Phospho)
qGsPTQsPPADTSFGSR	A0A0G2KAV8	N-Term(iTRAQ4plex); S3(Phospho); S7(Phos
sHsSPSLNPDASPVTAk	D4A404	N-Term(iTRAQ4plex); S3(Phospho); K17(iTR
aSGQAFELILsPR	A0A096MK73	N-Term(iTRAQ4plex); S11(Phospho)
gsPRYsPR	D4A9L2	N-Term(iTRAQ4plex); S2(Phospho); S6(Phos
rsPsPkPTk	F1M6U2	N-Term(iTRAQ4plex); S2(Phospho); S4(Phos
cIELScGsVR	P06686	N-Term(iTRAQ4plex); C1(Carbamidomethyl);
kEGDGSATTDAAPATsPk	P07936	K1(iTRAQ4plex); N-Term(iTRAQ4plex); S16(
tQEISRPNsPSEGEGESSI	Q62901	N-Term(iTRAQ4plex); S9(Phospho)
iEVEDLQNDEEELsPPVM	D4A1V8	N-Term(iTRAQ4plex); S14(Phospho); K25(iT
dTQEIVHDLEsPGIDPSIIk	D3ZHV2	N-Term(iTRAQ4plex); S11(Phospho); K20(iT
rRsPtPPPR	B2RYB3;A0A0G2K4F6	N-Term(iTRAQ4plex); S3(Phospho); T5(Phos
iTNHEDGsPVNEPNETtPL	F1M8G9	N-Term(iTRAQ4plex); S8(Phospho); T17(Pho
tSEEGDFR	Q68FQ2	N-Term(iTRAQ4plex); T1(Phospho)
sNsVEkPVSSLLSR	F1M8I7	N-Term(iTRAQ4plex); S3(Phospho); K6(iTRA
lSAsPQELGkPYLESFQPN	F1LQN3	N-Term(iTRAQ4plex); S4(Phospho); K10(iTR
rNPETtVSTPGPSLPDDEV	M0RB44	N-Term(iTRAQ4plex); T6(Phospho); K22(iTR
lWNYVPGLtPcLPR	F1M5N7	N-Term(iTRAQ4plex); T9(Phospho); C11(Ca
sPASVksPSEAksPAGAk	F1LRZ7	N-Term(iTRAQ4plex); K6(iTRAQ4plex); S7(P
sHGPLLPtIEDSsEEEELRI	Q9JKS6;D3Z9C7	N-Term(iTRAQ4plex); T8(Phospho); S13(Pho
dAQsPVLEVDAASGk	M0RAP6	N-Term(iTRAQ4plex); S4(Phospho); K15(iTR
kEIEVDsSPSVLEILDTAG	P61227	N-Term(iTRAQ4plex); K1(iTRAQ4plex); S7(P
rQDsAGPILDGAR	D4A3V5	N-Term(iTRAQ4plex); S4(Phospho)
aTLWSQPSDSQQYFcPPA	A0A096MJN4	N-Term(iTRAQ4plex); C15(Carbamidomethyl
gNVLPRGsEDEk	A0A0G2K774	N-Term(iTRAQ4plex); S8(Phospho); K12(iTR
aTVPAAAEGEGsPPAAAA	Q99MD2	N-Term(iTRAQ4plex); S12(Phospho)
rGsLPDQk	A0A0G2JTS9	N-Term(iTRAQ4plex); S3(Phospho); K8(iTRA
mAGsAFDFENMk	P50398	N-Term(iTRAQ4plex); S4(Phospho); K12(iTR
qVsVTNDGSQLGALVPSE	A0A0G2JUG7	N-Term(iTRAQ4plex); S3(Phospho); C19(Ca
hVLPtSAPDVTSSLPEGk	A0A140TA95	N-Term(iTRAQ4plex); T5(Phospho); K18(iTR
nSAYTEPGDScVDMETDF	G3V6K8	N-Term(iTRAQ4plex); C11(Carbamidomethyl

gHYEVTGsDDEAGk	A0A0G2JU96	N-Term(iTRAQ4plex); S8(Phospho); K14(iTR
qDSDsDVVDVEDAEQDFI	A0A0G2K5T1	N-Term(iTRAQ4plex); S5(Phospho)
nLFTQTLGLGsQk	A0A0G2JWM2	N-Term(iTRAQ4plex); S11(Phospho); K13(iT
lNWLSVDFNNWkDWEDD	P83868	N-Term(iTRAQ4plex); K12(iTRAQ4plex); S18
sGGGEQPDILSVGILVk	D3ZN60	N-Term(Acetyl); S1(Phospho); K17(iTRAQ4p
aNsDIISLNFR	Q5BJX5	N-Term(iTRAQ4plex); S3(Phospho)
qGsPTkDIELQFQR	D3ZMS5	N-Term(iTRAQ4plex); S3(Phospho); K6(iTRA
hFLEDNsDDAELSk	A0A096MJN4	N-Term(iTRAQ4plex); S7(Phospho); K14(iTR
sPAEVksPVEAk	F1LRZ7	N-Term(iTRAQ4plex); K6(iTRAQ4plex); S7(P
gGDAEEEAGPQAAEPStP	A0A0G2K613	N-Term(iTRAQ4plex); T17(Phospho)
lSSFATSVAEDQsVASLTA	P34926	N-Term(iTRAQ4plex); S13(Phospho); K27(iT
sPAEAksPAEAksPAEAk	F1LRZ7	N-Term(iTRAQ4plex); K6(iTRAQ4plex); S7(P
sDQEAkPSTEDLGDk	Q5I0H3	N-Term(Acetyl); S1(Phospho); K6(iTRAQ4ple
sPVPksPVEEVkPkPEAk	P12839	N-Term(iTRAQ4plex); S1(Phospho); K5(iTRA
qAsPTEVVER	A0A0G2K8F9	N-Term(iTRAQ4plex); S3(Phospho)
gMYDGPVYEVPAtPk	Q62950	N-Term(iTRAQ4plex); T13(Phospho); K15(iT
wMLsLAk	P13233	N-Term(iTRAQ4plex); S4(Phospho); K7(iTRA
eSAsPTIPNLDLLEAHTk	F1LWX5	N-Term(iTRAQ4plex); S4(Phospho); K18(iTR
eLsLPR	D3ZAR2	N-Term(iTRAQ4plex); S3(Phospho)
tQPVTFDEIQEVEEEGVsP	A8IHN8	N-Term(iTRAQ4plex); S18(Phospho); K24(iT
nLsLPFILHEk	B5D5N9	N-Term(iTRAQ4plex); S3(Phospho); K11(iTR
mAGTMETSEMVNGAAE(	G3V6S0	N-Term(iTRAQ4plex); S21(Phospho); K22(iT
dFsLEQLR	Q5XIU9	N-Term(iTRAQ4plex); S3(Phospho)
iSkFsPIEEAk	G3V984	N-Term(iTRAQ4plex); K3(iTRAQ4plex); S5(P
tASIkPEVALLDTQDMENN	D4A781	N-Term(iTRAQ4plex); K5(iTRAQ4plex); S20(
tLDsPQGTNk	Q6RJR6	N-Term(iTRAQ4plex); S4(Phospho); K10(iTR
mAQQPLSLVGcDVLPDPS	F7IXA1	N-Term(iTRAQ4plex); C11(Carbamidomethyl
sPAEVksPVTVk	F1LRZ7	N-Term(iTRAQ4plex); K6(iTRAQ4plex); S7(P
iFSNALPDDLRsPIR	A0A0G2JT23	N-Term(iTRAQ4plex); S12(Phospho)
sHSFDMGDFAAAAAAAP(	G3V7J3	N-Term(iTRAQ4plex); S21(Phospho)
eSSTNNSVsPSESLR	Q4V8F0	N-Term(iTRAQ4plex); S9(Phospho)
eSETFSDSsPIEIIDEFPtFY	F1LQN3	N-Term(iTRAQ4plex); S9(Phospho); T19(Pho
kLGGDGsPTQVDVSQFG	P15205;F1LRL9	K1(iTRAQ4plex); N-Term(iTRAQ4plex); S7(P
sPQAQDMPVSIAGGQTG	P34926	N-Term(iTRAQ4plex); S1(Phospho); C18(Car
eEcPRPMSIsPPDFsPk	P15205;F1LRL9	N-Term(iTRAQ4plex); C3(Carbamidomethyl);
lPAkLsVSk	G3V8L9	N-Term(iTRAQ4plex); K4(iTRAQ4plex); S6(P
aSsLDLNk	A0A0G2K4S1	N-Term(iTRAQ4plex); S3(Phospho); K8(iTRA
gPPTRsPQAQDMPVSIAG	P34926	N-Term(iTRAQ4plex); S6(Phospho); C23(Car
dIIRQPsEEEIIk	Q5U318	N-Term(iTRAQ4plex); S7(Phospho); K13(iTR
sSPVPRSPDREDGEEAPA	G3V9G5	N-Term(iTRAQ4plex); S1(Phospho); K24(iTR
ssSEESATTTSPESISGSV	Q5XFX7	N-Term(iTRAQ4plex); S2(Phospho)
aQPAPPELNSESEDYSPS	O08875	N-Term(iTRAQ4plex); S25(Phospho)
nRDVEMGNsVIEENEMk	A0A0G2K611	N-Term(iTRAQ4plex); S9(Phospho); K17(iTR
akEVsPMSAPNMPSIER	Q923K6	N-Term(iTRAQ4plex); K2(iTRAQ4plex); S5(P
nVTESPsFSAGDNPHVLY	G3V9B3	N-Term(iTRAQ4plex); S7(Phospho); K30(iTR
sPSEAksPAGAksPAEAk	F1LRZ7	N-Term(iTRAQ4plex); K6(iTRAQ4plex); S7(P
lSNGSIDSTDDtSQIVELQI	F1M8A4	N-Term(iTRAQ4plex); T12(Phospho); K24(iT
sPAGAksPAEAksPVVAk	F1LRZ7	N-Term(iTRAQ4plex); S1(Phospho); K6(iTRA
lFDDsDERGTVGNVk	D4A997	N-Term(iTRAQ4plex); S5(Phospho); K15(iTR
rSsSGSTDSEESTDSEEE	D3ZBT9	N-Term(iTRAQ4plex); S3(Phospho); K22(iTR
aATEALGEksPEGTTVSG	A0A140TA95	N-Term(iTRAQ4plex); K9(iTRAQ4plex); S10(
sPsEEEEDGQLVk	P0C6P5	N-Term(iTRAQ4plex); S3(Phospho); K13(iTR
eTEVSkGsAESPDEGITTT	P15205;F1LRL9	N-Term(iTRAQ4plex); K6(iTRAQ4plex); S8(P
lPsDEDESGTEESDNTPLI	A0A0H4SRI7	N-Term(iTRAQ4plex); S3(Phospho); K20(iTR
scGGYLHADRGVITSPk	F1LNU2	N-Term(iTRAQ4plex); S1(Phospho); C2(Carb

aPQSFFDtTPSGR	A0A0G2K4K3	N-Term(iTRAQ4plex); T8(Phospho)
vPsSDEEVVEEPQSR	D3ZF26	N-Term(iTRAQ4plex); S3(Phospho)
aksPmkEEAk	F1LRZ7	N-Term(iTRAQ4plex); K2(iTRAQ4plex); S3(P
aksPVPksPVEEVkPkPEA	P12839	N-Term(iTRAQ4plex); K2(iTRAQ4plex); S3(P
qGEDNsITQDTEDLEkDTI	Q9Z1W6	N-Term(iTRAQ4plex); S6(Phospho); K16(iTR
kIAELEEERsQGSTSNSD\	F1LRL9	N-Term(iTRAQ4plex); K1(iTRAQ4plex); S10(
rEsPPSAER	C5NTX8	N-Term(iTRAQ4plex); S3(Phospho)
eSVAsGDDR	P15205;F1LRL9	N-Term(iTRAQ4plex); S5(Phospho)
iEDVGsDEEDDsGkDkk	P34058	N-Term(iTRAQ4plex); S6(Phospho); S12(Pho
rENGPAsEEGDLGQEHDI	F1M9X4	N-Term(iTRAQ4plex); S7(Phospho); K23(iTR
vkEVHDELEDLPsPPPPLS	M0R423	N-Term(iTRAQ4plex); K2(iTRAQ4plex); S13(
sLMssPEDLTkDFEELkAE	P15205;F1LRL9	N-Term(iTRAQ4plex); S4(Phospho); S5(Phos
dIsPEEIDLkNEPWYk	F1M820	N-Term(iTRAQ4plex); S3(Phospho); K10(iTR
kYQVDLsk	P06686	N-Term(iTRAQ4plex); K1(iTRAQ4plex); S7(P
yNLDAsEEEDSNkk	O35986	N-Term(iTRAQ4plex); S6(Phospho); K13(iTR
aDsFSEGDDLSQGHLAEI	A0A0G2K0E5	N-Term(iTRAQ4plex); S3(Phospho); C19(Car
aAsPEAcEVDSk	A0A0G2K5S2	N-Term(iTRAQ4plex); S3(Phospho); C7(Carb
iNSVSsQLSDGPmPSPSA	Q63475	N-Term(iTRAQ4plex); S6(Phospho); M13(Ox
kAEHIcTHSLSPSEPAVAS	Q6RJR6	N-Term(iTRAQ4plex); K1(iTRAQ4plex); C6(C
tPGDFNYAYQkPEsTtESP	P15205;F1LRL9	N-Term(iTRAQ4plex); K11(iTRAQ4plex); S14
rLSLPGLLsQVSPR	P0C5Y8	N-Term(iTRAQ4plex); S9(Phospho)
iYSSDSDEGsEEDkAQR	Q641X2	N-Term(iTRAQ4plex); S10(Phospho); K14(iT
aESSAATQSPSVsSSSSG	Q6RJR6	N-Term(iTRAQ4plex); S13(Phospho); C34(Ca
dVSDERLsPAksPSLSPSI	P15205;F1LRL9	N-Term(iTRAQ4plex); S8(Phospho); K11(iTR
tGAELDIkDVsDER	P15205;F1LRL9	N-Term(iTRAQ4plex); K8(iTRAQ4plex); S11(
aAsSSSPGSPVASSPSk	A0A0G2K3N1	N-Term(iTRAQ4plex); S3(Phospho); K17(iTR
iLTEEETsPQcQMDR	D3ZWX4	N-Term(iTRAQ4plex); S8(Phospho); C11(Car
eAEDsDSDDNIkR	Q3SWT4	N-Term(iTRAQ4plex); S5(Phospho); K12(iTR
tSsDPNLNNHcQEVR	D3ZW40	N-Term(iTRAQ4plex); S3(Phospho); C11(Car
rGsGSVDETLFALPAASEI	F1LQN3	N-Term(iTRAQ4plex); S3(Phospho); K27(iTR
vPssDEEVVEEPQSR	D3ZF26	N-Term(iTRAQ4plex); S3(Phospho); S4(Phos
aQSPLLPEPLkNQsPVVP/	F1LQZ9	N-Term(iTRAQ4plex); K11(iTRAQ4plex); S14
fYLQDRsPSQSDPVLk	Q5I0D1	N-Term(iTRAQ4plex); S7(Phospho); K16(iTR
sELVsEGRPVETDSESEF	F1LXQ7	N-Term(iTRAQ4plex); S5(Phospho)
lDSsPVLSPGNk	A0A0G2K382	N-Term(iTRAQ4plex); S4(Phospho); K12(iTR
gALDSETGEkQDEAPEVD	F1LWN1	N-Term(iTRAQ4plex); K10(iTRAQ4plex); K19
eGSPLkEEsLAREDsPLSS	A0A0G2K774	N-Term(iTRAQ4plex); K6(iTRAQ4plex); S9(P
nSSSAVsPLYSPVSFcGIP	D3Z9L5	N-Term(iTRAQ4plex); S7(Phospho); C16(Car
eESDDDQSDVDDTEVsPI	P28818	N-Term(iTRAQ4plex); S16(Phospho); K21(iT
eQsDDETEESVk	F1LU97	N-Term(iTRAQ4plex); S3(Phospho); K12(iTR
aDsEEEGAVELAA	A0A173DW30	N-Term(iTRAQ4plex); S3(Phospho)
mETVSNASSSSNPSsPGI	D3ZU56	N-Term(iTRAQ4plex); S15(Phospho)
aAsDPNPAEPAR	D3Z9R2	N-Term(iTRAQ4plex); S3(Phospho)
vsPPESPR	A0A0G2KAV8	N-Term(iTRAQ4plex); S2(Phospho)
qMASQFPPPPTPPPVDS(	D4ADX8	N-Term(iTRAQ4plex); K21(iTRAQ4plex); S31
aFPAYYTSHVQEEQsEVE	P19527	N-Term(iTRAQ4plex); S15(Phospho); K25(iT
iQStPVk	Q6NYB7	N-Term(iTRAQ4plex); T4(Phospho); K7(iTRA
lGADEsEEEGR	G3V700	N-Term(iTRAQ4plex); S6(Phospho)
ssPQEAGNVDIWR	Q5PQP4	N-Term(iTRAQ4plex); S2(Phospho)
rLsLEsEGANEGAAAAPEI	D3ZQL7	N-Term(iTRAQ4plex); S3(Phospho); S6(Phos
kQGGsPDEPDck	A0A0G2K0P0	N-Term(iTRAQ4plex); K1(iTRAQ4plex); S5(P
eGQVEAAQPEQAAEAPA	D4A1U1	N-Term(iTRAQ4plex); S32(Phospho)
aSsLNFLNk	B2GV74	N-Term(iTRAQ4plex); S3(Phospho); K9(iTRA
iDIMVGmGYsQEEIQESLS	F1M836	N-Term(iTRAQ4plex); M7(Oxidation); S10(Ph
vELsEEELk	Q6AZ64	N-Term(iTRAQ4plex); S4(Phospho); K9(iTRA

Information Classification: General

kRsYsPDGk	A0A0G2JSR7	N-Term(iTRAQ4plex); K1(iTRAQ4plex); S3(P
sRTsPVTR	A0A0G2K2M9	N-Term(iTRAQ4plex); S4(Phospho)
tEDGDWEWsDDEMDEkS	A0A0G2K007	N-Term(iTRAQ4plex); S9(Phospho); K16(iTR
qGDEESENsVkR	M0R5U4	N-Term(iTRAQ4plex); S9(Phospho); K11(iTR
aTTDNsPSSkAEDGPAk	P07936	N-Term(iTRAQ4plex); S6(Phospho); K10(iTR
sLAEAksPEkAk	F1LRZ7	N-Term(iTRAQ4plex); K6(iTRAQ4plex); S7(P
qEsDPEDDDVkkPALQSS	D3ZAY8	N-Term(iTRAQ4plex); S3(Phospho); K11(iTR
sLAAsPEGSEHWVFIER	A0A0G2K0F3	N-Term(iTRAQ4plex); S5(Phospho)
mAILPGLAsPR	D4A7C3	N-Term(iTRAQ4plex); S9(Phospho)
mAsPPPSGPPSAAHTPFF	P34926;Q63330	N-Term(iTRAQ4plex); M1(Oxidation); S3(Pho
aksPSSDSWTcADASTGR	Q3S4A5	N-Term(iTRAQ4plex); K2(iTRAQ4plex); S3(P
vLNTGsDVEEAVADALk	A0A0G2JZM8	N-Term(iTRAQ4plex); S6(Phospho); K17(iTR
aTsPSTLVSTGSSSR	A0A0G2JW88	N-Term(iTRAQ4plex); S3(Phospho)
tELsPSRAsPGk	Q4KM62	N-Term(iTRAQ4plex); S4(Phospho); S9(Phos
vLTPTQVMNRPSSIsWDG	P31044	N-Term(iTRAQ4plex); S15(Phospho); K23(iT
eMGTDGYsDSEHYLPME	A0A0G2JXK1	N-Term(iTRAQ4plex); S8(Phospho)
vPLLFsDEEDSEVPSGVkF	Q80X08	N-Term(iTRAQ4plex); S6(Phospho); K18(iTR
aEAAAAPAVAPGPAQPGI	A1L1I3	N-Term(iTRAQ4plex); T26(Phospho); S27(Ph
aGNsPEGHcPR	B5DEJ5	N-Term(iTRAQ4plex); S4(Phospho); C9(Carb
ssDDIDYR	F1M3W5	N-Term(iTRAQ4plex); S2(Phospho)
eENQTGPTTtPsDTQDLD	Q920Q0	N-Term(iTRAQ4plex); T10(Phospho); S12(Ph
sGsLsPcPPGDTLPWNLP	D3ZHV2	N-Term(iTRAQ4plex); S3(Phospho); S5(Phos
sNSAPLIHGLSDSSPVFQ	Q6AYT4	N-Term(iTRAQ4plex); S1(Phospho)
yEPAAVsEHGDkk	P06685	N-Term(iTRAQ4plex); S7(Phospho); K12(iTR
rAsGQSFEVILk	A0A0G2K8P5	N-Term(iTRAQ4plex); S3(Phospho); K12(iTR
eNQSEGkGsDsDSEGDNI	Q793F9	N-Term(iTRAQ4plex); K7(iTRAQ4plex); S9(P
wLSTSIPEAQWQSsLAR	P12785	N-Term(iTRAQ4plex); S14(Phospho)
nERHsPVSSIk	A0A0G2K6R9;F1M9N9	N-Term(iTRAQ4plex); S5(Phospho); K11(iTR
aWSsTDSDSSNR	D3ZQR3	N-Term(iTRAQ4plex); S4(Phospho)
sPAEAksPAEAk	F1LRZ7	N-Term(iTRAQ4plex); S1(Phospho); K6(iTRA
kGEEQSAPDVTGTEsDAE	F1LWN1	N-Term(iTRAQ4plex); K1(iTRAQ4plex); S15(
sPAEVksPATVk	F1LRZ7	N-Term(iTRAQ4plex); S1(Phospho); K6(iTRA
aPELLGPEDQLVLGPEQF	Q9Z1H9	N-Term(iTRAQ4plex); S25(Phospho)
lGsFGSITR	A0A0H2UHR7	N-Term(iTRAQ4plex); S3(Phospho)
sREDsPELHPPPGIDDNR	F1M0L3	N-Term(iTRAQ4plex); S5(Phospho)
lVsSESk	E9PT53	N-Term(iTRAQ4plex); S3(Phospho); K7(iTRA
dIPVSLQPVLDTLLQLSGF	D4A929	N-Term(iTRAQ4plex); K20(iTRAQ4plex); S21
ekDLDLEPLsDLEEGLEET	A0A096MJL8	N-Term(iTRAQ4plex); K2(iTRAQ4plex); S10(
eYGsTSSIDR	F1M8G8	N-Term(iTRAQ4plex); S4(Phospho)
sEDDAAsGEHDVQIEGVR	A0A0G2JZX3	N-Term(iTRAQ4plex); S7(Phospho)
ncSETQYEsk	P61983	N-Term(iTRAQ4plex); C2(Carbamidomethyl);
aSTAAPVASPAAPsPGSS	P09951	N-Term(iTRAQ4plex); S14(Phospho); K32(iT
vIsLSGEHSIIGR	Q6LDS4	N-Term(iTRAQ4plex); S3(Phospho)
iSQSEDEEsIVGDGETk	P61808	N-Term(iTRAQ4plex); S9(Phospho); K17(iTR
lkEGLDISHLQGQEELLSs	Q9R1N0	N-Term(iTRAQ4plex); K2(iTRAQ4plex); S19(
mAESSAATQSPSVSSSS	Q6RJR6	N-Term(Acetyl); M1(Oxidation); S23(Phospho
gPSPLVTMTPAVPAVTPV	M0RD40	N-Term(iTRAQ4plex); S22(Phospho); S23(Ph
dFQEDsWGETk	P34926;Q63330	N-Term(iTRAQ4plex); S6(Phospho); K11(iTR
vPSTEAEALAsSLMGLFEI	P50399	N-Term(iTRAQ4plex); S11(Phospho); K19(iT
aPsQSESESEk	O55164	N-Term(iTRAQ4plex); S3(Phospho); K11(iTR
qVLLGPYNPDtSPEVGFFI	G3V6E4	N-Term(iTRAQ4plex); T11(Phospho)
dQPAALPLAAEETVNLPP	A0A0U1RRX4	N-Term(iTRAQ4plex); S19(Phospho); K41(iT
gLSsVPEVAEVETTTk	A0A0U1RRX4;F1MAQ5	N-Term(iTRAQ4plex); S4(Phospho); K16(iTR
lVsPEPPPk	A0A0G2K0F3	N-Term(iTRAQ4plex); S3(Phospho); K9(iTRA
dGGEsPTVLk	B2GUV4	N-Term(iTRAQ4plex); S5(Phospho); K10(iTR

eDSEEQtVkPGPEEGTSE	P34926;Q63330	N-Term(iTRAQ4plex); T7(Phospho); K9(iTRA
ikQEQDYWsEsDkEEADT	Q9Z1T4	N-Term(iTRAQ4plex); K2(iTRAQ4plex); S9(P
eADMPDEVNIDELLELDs	Q99MC0	N-Term(iTRAQ4plex); S18(Phospho)
vIPSVVIEPAsNHEGEEHC	A0A0G2K5Z4	N-Term(iTRAQ4plex); S11(Phospho)
ekDPHRPLsPTER	Q99PJ8	N-Term(iTRAQ4plex); K2(iTRAQ4plex); S9(P
sATPATDGRAtPATEEST	Q63092	N-Term(iTRAQ4plex); T11(Phospho); K31(iT
eENVHsPEDkR	A0A0G2JXZ3	N-Term(iTRAQ4plex); S6(Phospho); K10(iTR
lYLQsPR	F1M2K6	N-Term(iTRAQ4plex); S5(Phospho)
iPVPsSPVPIATIDLVQQQ	A0A0G2JW92	N-Term(iTRAQ4plex); S5(Phospho); M24(Ox
vTAVSDIAEQLLkAsPPGk	F1MA36	N-Term(iTRAQ4plex); K13(iTRAQ4plex); S15
wLAEsPVGLPPEEEDkLT	P34926	N-Term(iTRAQ4plex); S5(Phospho); K16(iTR
mAsPPPSGPPSAAHTPF	P34926;Q63330	N-Term(iTRAQ4plex); S3(Phospho); S20(Pho
vFmsYYFLHVVADIk	F1M208	N-Term(iTRAQ4plex); M3(Oxidation); S4(Pho
iIEQPGcPsPVMEAESTk	D3ZX21	N-Term(iTRAQ4plex); C7(Carbamidomethyl);
kAsSsDDEGGPR	Q5QD51	K1(iTRAQ4plex); N-Term(iTRAQ4plex); S3(P
gImAPPEIDksPSEGDVAF	D3ZBU7	N-Term(iTRAQ4plex); M3(Oxidation); K10(iT
sVGAQsPk	F1LPS3	N-Term(iTRAQ4plex); S6(Phospho); K8(iTRA
eQQEDSSATSFSDLPLYL	A0A0G2JZX5	N-Term(iTRAQ4plex); S26(Phospho)
eLVLALYDYQEksPR	P16086	N-Term(iTRAQ4plex); K12(iTRAQ4plex); S13
fYGGGGGGGsPGk	A0A0G2K0E5	N-Term(iTRAQ4plex); S10(Phospho); K13(iT
lSEQDsPPPcHPLk	D3ZLQ8	N-Term(iTRAQ4plex); S6(Phospho); C10(Ca
tPPksPGDPAk	D3ZQL7	N-Term(iTRAQ4plex); K4(iTRAQ4plex); S5(P
qLSYsDSDLk	D4ACD9	N-Term(iTRAQ4plex); S5(Phospho); K10(iTR
sLMsSPEDLTkDFEELkAE	P15205;F1LRL9	N-Term(iTRAQ4plex); S4(Phospho); K11(iTR
vSSLsPEQEQGLWk	D3Z8B9	N-Term(iTRAQ4plex); S5(Phospho); K14(iTR
lAsDSDAESDSR	A0A0G2KA27	N-Term(iTRAQ4plex); S3(Phospho)
sPVkEEIkPPAEVksPEk	F1LRZ7	N-Term(iTRAQ4plex); K4(iTRAQ4plex); K8(iT
hTPNTSDNEGsDTEVcGF	D3ZXY2	N-Term(iTRAQ4plex); S11(Phospho); C16(Ca
mNAGsPGSEVVTLQQFL	D3ZYD7	N-Term(iTRAQ4plex); S5(Phospho); K22(iTR
kLPEPLNWRsDEEDEDSI	G3V9J8	N-Term(iTRAQ4plex); K1(iTRAQ4plex); S10(
kLsVGAYVSSVR	G3V8Q8	N-Term(iTRAQ4plex); K1(iTRAQ4plex); S3(P
kDDSHSAEDsEDEkDDHk	A0A0G2K7X3	K1(iTRAQ4plex); N-Term(iTRAQ4plex); S10(
fSGEEGEIEDDESGTENR	Q5M7V8	N-Term(iTRAQ4plex); K21(iTRAQ4plex); S23
aEsGDsLSSEDRDLLYSID	M0RDG0	N-Term(iTRAQ4plex); S3(Phospho); S6(Phos
nTIGSsPVADFSAIk	Q5JC29	N-Term(iTRAQ4plex); S6(Phospho); K15(iTR
tGPESSRPNTALLDPSsP	D3ZZK3	N-Term(iTRAQ4plex); S17(Phospho); K34(iT
gSWDSQQDGSGYEsDG	F1LR18	N-Term(iTRAQ4plex); S14(Phospho)
sPESLSsPAmEDLAVEWE	P34926;Q63330	N-Term(iTRAQ4plex); S7(Phospho); M10(Ox
aGsEVEWAEDLALDLGP	Q5U2P5;A0A0G2K2V4	N-Term(iTRAQ4plex); S3(Phospho)
sIQFVDWcPTGFk	Q5XIF6	N-Term(iTRAQ4plex); S1(Phospho); C8(Carb
kNDGVkEssEsTNTTIEDE	P11275	N-Term(iTRAQ4plex); K1(iTRAQ4plex); K6(iT
gSLDsDNDDEkDPQHSLN	A0A0H2UHB7	N-Term(iTRAQ4plex); S5(Phospho); K11(iTR
tAGAQVVLNEQELQLWP	P13233	N-Term(iTRAQ4plex); K22(iTRAQ4plex); S25
eNSAAPsPIRPHstSPAkE	Q920Q0	N-Term(iTRAQ4plex); S7(Phospho); S13(Pho
eGPLsGSYR	P27139	N-Term(iTRAQ4plex); S5(Phospho)
tISNPEGSPsR	Z4YNN0	N-Term(iTRAQ4plex); S10(Phospho)
rLSPWEDLSQAPTMDsPL	F1LR33	N-Term(iTRAQ4plex); S16(Phospho); K20(iT
ekNQWQLsLDDLk	P13233	N-Term(iTRAQ4plex); K2(iTRAQ4plex); S8(P
mNAGSPGsEVVTLQQFL	D3ZYD7	N-Term(iTRAQ4plex); M1(Oxidation); S8(Pho
iQQFDDGGsDEEDIWEEk	D3ZBT9	N-Term(iTRAQ4plex); S9(Phospho); K18(iTR
eEEEGISQEssEEEQ	Q8K585	N-Term(iTRAQ4plex); S10(Phospho); S11(Ph
tSsPTTLPPLAR	D4A554	N-Term(iTRAQ4plex); S3(Phospho)
tAsELLLDR	A0A0G2JWS3	N-Term(iTRAQ4plex); S3(Phospho)
lLRPSLAELHDELEkEPFE	Q9R1N0	N-Term(iTRAQ4plex); K15(iTRAQ4plex); S28
lPSGSGAAsPTTGSAVDIF	A0A0G2JU96	N-Term(iTRAQ4plex); S9(Phospho)

Information Classification: General

rGTGQsDDsDIWDDTALIk	Q2KP10	N-Term(iTRAQ4plex); S6(Phospho); S9(Phos
iGPLQSTLEVGLEsPPLSV	D3ZHB7	N-Term(iTRAQ4plex); S14(Phospho)
lSTsPATR	D3ZG21	N-Term(iTRAQ4plex); S4(Phospho)
tEVVMNNLsPAWk	H1UBM8	N-Term(iTRAQ4plex); S9(Phospho); K13(iTR
aSAEGEATAVVsPGVTQA	P15205;F1LRL9	N-Term(iTRAQ4plex); S12(Phospho); C24(Ca
ySPNTQVEILPQGREsPIP	Q6AYC4	N-Term(iTRAQ4plex); S16(Phospho); K20(iT
sAFTPATATGSsPsPVLG(	D3ZV52	N-Term(iTRAQ4plex); S12(Phospho); S14(Ph
sPAEAksPAEVk	F1LRZ7	N-Term(iTRAQ4plex); S1(Phospho); K6(iTRA
mPsIESDV	G3V9C5	N-Term(iTRAQ4plex); S3(Phospho)
sVLSPAGVGsmGLLGPG(	G3V7K0	N-Term(iTRAQ4plex); S10(Phospho); M11(O
eLHPQLLsPTk	F1LPG6	N-Term(iTRAQ4plex); S8(Phospho); K11(iTR
tPALsPQRPLTTQQPQSG	G3V733	N-Term(iTRAQ4plex); T1(Phospho); S5(Phos
sGsIIGSR	D4A720	N-Term(iTRAQ4plex); S1(Phospho); S3(Phos
sPSLAFPk	Q3T1K9	N-Term(iTRAQ4plex); S1(Phospho); K8(iTRA
rLPycMGNVIPHtycDHMS\	D3ZIT1	N-Term(iTRAQ4plex); Y4(Phospho); C5(Carb
lPSGTLIGEPELEDVVGET	A0A0G2K1W1	N-Term(iTRAQ4plex); S21(Phospho)
ePTPSIAsDISLPIATQELR	D3ZJ32	N-Term(iTRAQ4plex); S8(Phospho)
lGELYSVGEsDkET	O54800	N-Term(iTRAQ4plex); S10(Phospho); K12(iT
kNDGVkESsEstNttIEDED	P11275	K1(iTRAQ4plex); N-Term(iTRAQ4plex); K6(iT
eFLPTSWsPIGVGPAPSL	Q3T1I9	N-Term(iTRAQ4plex); S8(Phospho); K20(iTR
vEcGsDPEENSAR	G3V764	N-Term(iTRAQ4plex); C3(Carbamidomethyl);
vEFSTPFQVEEVDDVsPs	G3V9G5	N-Term(iTRAQ4plex); S16(Phospho); S18(Ph
vNQQPNANDkNsPPk	A0A0G2JVV5	N-Term(iTRAQ4plex); K10(iTRAQ4plex); S12
aLANDGTsPGER	M0R617	N-Term(iTRAQ4plex); S8(Phospho)
sLsGTLTELDEk	D3ZC56	N-Term(iTRAQ4plex); S3(Phospho); K12(iTR
aNVVTsPk	P15791	N-Term(iTRAQ4plex); S6(Phospho); K8(iTRA
gIITDsFGR	A0A0G2JZF5	N-Term(iTRAQ4plex); S6(Phospho)
ktSASPPLEk	A0A0G2K7X3	N-Term(iTRAQ4plex); K1(iTRAQ4plex); T2(P
ssEVEIFVDcEcGk	Q8CGU9	N-Term(iTRAQ4plex); S2(Phospho); C10(Car
sPVEAksPAEVk	F1LRZ7	N-Term(iTRAQ4plex); S1(Phospho); K6(iTRA
fGIHVYQFPEcDsDEDEDF	D3ZT07	N-Term(iTRAQ4plex); C11(Carbamidomethyl
qPEDESPQAFSQsGSPR	F1M7S0	N-Term(iTRAQ4plex); S13(Phospho)
mESEAGADDsAEEGDLLI	G3V9R8	N-Term(iTRAQ4plex); S10(Phospho); K33(iT
lkIsPEQHWDFTAEDLk	F1LP57	N-Term(iTRAQ4plex); K2(iTRAQ4plex); S4(P
qEPLRQPsPQR	D3ZDC0	N-Term(iTRAQ4plex); S8(Phospho)
nNPsPPPDSDLER	F1LX86	N-Term(iTRAQ4plex); S4(Phospho)
iAESHLQTISNLSENQAsE	Q6J4I0	N-Term(iTRAQ4plex); S18(Phospho)
gVFPVSFVHILsD	A0A0G2K451	N-Term(iTRAQ4plex); S12(Phospho)
nLNHVsYGR	P10860	N-Term(iTRAQ4plex); S6(Phospho)
sVAAGAAtAATSYPMsYG	Q63410	N-Term(iTRAQ4plex); T8(Phospho); S16(Pho
nNMsPHGIPAR	F1LQS1	N-Term(iTRAQ4plex); S4(Phospho)
dGskEPIVEmR	F1M8G9	N-Term(iTRAQ4plex); S3(Phospho); K4(iTRA
ssPAtTVTSPNSTPAk	Q05140;A0A0G2K0B6	N-Term(iTRAQ4plex); S2(Phospho); T5(Phos
rPHSLSIMPEAFDsDEELG	G3V984	N-Term(iTRAQ4plex); S14(Phospho)
tEPGsPVkPNVLTASAAPL	A0A0G2K4R1	N-Term(iTRAQ4plex); S5(Phospho); K8(iTRA
qGGASQSDktPEELFHPL(	A0A0G2K2S2	N-Term(iTRAQ4plex); K9(iTRAQ4plex); T10(
aGTksPAVsPSk	F8WFS9	N-Term(iTRAQ4plex); K4(iTRAQ4plex); S5(P
lNQSDSIEDPNsPAGR	Q7TQ77	N-Term(iTRAQ4plex); S12(Phospho)
dSLEAsPVLEDNSSHk	A0A0G2K6R9;F1M9N9	N-Term(iTRAQ4plex); S6(Phospho); K16(iTR
tEQSVtPEQQk	Q9Z0W5;A0A0G2JWR2	N-Term(iTRAQ4plex); T6(Phospho); K11(iTR
gQLsDDEk	E9PTU4	N-Term(iTRAQ4plex); S4(Phospho); K8(iTRA
lTGSTSsLNk	F1LQN9	N-Term(iTRAQ4plex); S7(Phospho); K10(iTR
dETFGEyRSLESDNEEk	Q05695	N-Term(iTRAQ4plex); Y7(Phospho); K17(iTR
tQPVTFDEIQEVEEEGVsF	A8IHN8	N-Term(iTRAQ4plex); S18(Phospho); M20(O
gLVAAYSGEsDsEEEQER	P70501	N-Term(iTRAQ4plex); S10(Phospho); S12(Ph

rPsVDTPVTDVGFLR	A0A0G2K0E5	N-Term(iTRAQ4plex); S3(Phospho)
dAEADRNFDtLDLPk	B4F7F3	N-Term(iTRAQ4plex); T10(Phospho); K15(iTI
sPDcTHDNPLETR	P06687	N-Term(iTRAQ4plex); S1(Phospho); C4(Carb
qSRsPLPSHAR	P02688	N-Term(iTRAQ4plex); S4(Phospho)
iGsLDNITHVPGGGNk	D4A1Q2	N-Term(iTRAQ4plex); S3(Phospho); K16(iTR
kMPLDLsPLATPIIR	Q00969	N-Term(iTRAQ4plex); K1(iTRAQ4plex); S7(P
lEVTsDSEDASEVPEWLR	Q70AM4	N-Term(iTRAQ4plex); S5(Phospho)
sRsPDIQAk	A0A0G2JW20	N-Term(iTRAQ4plex); S3(Phospho); K9(iTRA
nNPAADTEDEEsDGEDR	A0A0G2K0J4	N-Term(iTRAQ4plex); S12(Phospho)
vGVDPDQDPPPNNDsFQ	D3ZCI2	N-Term(iTRAQ4plex); S15(Phospho)
rGsLGFYk	F1MA36	N-Term(iTRAQ4plex); S3(Phospho); K8(iTRA
gVDLLLEGVQGESsPTRR	A0A0G2KB60	N-Term(iTRAQ4plex); S14(Phospho)
iQQVSsPQk	A0A0G2K3H4	N-Term(iTRAQ4plex); S6(Phospho); K9(iTRA
aVPRsPEEDGkDTLQcIAE	D3ZKG7	N-Term(iTRAQ4plex); S5(Phospho); K11(iTR
ePTkSPEsPsSPVSSETTS	Q5QD51	N-Term(iTRAQ4plex); K4(iTRAQ4plex); S8(P
sLsAIDR	A0A0G2K7K2	N-Term(iTRAQ4plex); S3(Phospho)
aLLsSPEGEEk	D4A2D3	N-Term(iTRAQ4plex); S4(Phospho); K11(iTR
sFLQsLEcLR	A8IHN8	N-Term(iTRAQ4plex); S5(Phospho); C8(Carb
sQVEtEDLILkPGVVHVIDI	A0A0H2UHZ1	N-Term(iTRAQ4plex); T5(Phospho); K11(iTR
sPVEVksPEk	F1LRZ7	N-Term(iTRAQ4plex); K6(iTRAQ4plex); S7(P
sATPATDGSATPATDGsV	Q63092	N-Term(iTRAQ4plex); S17(Phospho)
sSAEDRsPENTSSSVAVD	A0A0G2K8K1	N-Term(iTRAQ4plex); S7(Phospho); K25(iTR
aGQENIsVSk	P06686	N-Term(iTRAQ4plex); S7(Phospho); K10(iTR
gEEsQEQPVSDSHQQQD	G3V7X5	N-Term(iTRAQ4plex); S4(Phospho); K21(iTR
rPPEsPPIVEEWNSR	Q5FVL9	N-Term(iTRAQ4plex); S5(Phospho)
sPVEEVkPkPEAk	P12839	N-Term(iTRAQ4plex); S1(Phospho); K7(iTRA
sADAGSQkDsDDSAATPQ	F1M0Z1	N-Term(iTRAQ4plex); K8(iTRAQ4plex); S10(
hSENEtSDREDGLTk	Q3SWT4	N-Term(iTRAQ4plex); T6(Phospho); K15(iTR
rSDAEEVDFAGWLcSTIG	A0A0H2UHI2	N-Term(iTRAQ4plex); C14(Carbamidomethyl
sHGAEGGSGsPEk	Q5BJQ2	N-Term(iTRAQ4plex); S10(Phospho); K13(iT
eEADVIPEsPLSDVGAEDI	G3V8M8	N-Term(iTRAQ4plex); S9(Phospho); K24(iTR
aAsLSMR	Q4G086	N-Term(iTRAQ4plex); S3(Phospho)
sPAVAksPAEVk	F1LRZ7	N-Term(iTRAQ4plex); K6(iTRAQ4plex); S7(P
dLTTGYDsNQPDk	F7FLB2	N-Term(iTRAQ4plex); S8(Phospho); K13(iTR
mASPPPSGPPSAAHTPF	P34926;Q63330	N-Term(iTRAQ4plex); S20(Phospho); K25(iT
mQEsPTLSQQSYNFDPD	X4YHC6	N-Term(iTRAQ4plex); S4(Phospho); C19(Cai
hNPVFGVMs	Q4V7C7	N-Term(iTRAQ4plex); S9(Phospho)
ePsPVsPVTVLk	K4DIC3	N-Term(iTRAQ4plex); S3(Phospho); S6(Phos
kVTGNPEDScASEAPGSs	D3ZCZ3	N-Term(iTRAQ4plex); K1(iTRAQ4plex); C10(
rPNEDsDEDEEk	F1M4W7	N-Term(iTRAQ4plex); S6(Phospho); K12(iTR
nEEDEGHSNSsPR	Q9JJ54	N-Term(iTRAQ4plex); S11(Phospho)
sVsGsPEPTAk	B2RYB3;A0A0G2K4F6	N-Term(iTRAQ4plex); S3(Phospho); S5(Phos
iADSSVQtDDEEGEGR	G3V984	N-Term(iTRAQ4plex); T8(Phospho)
qsPDHPTVGAGMLHITEN	P15205;F1LRL9	N-Term(iTRAQ4plex); S2(Phospho); Y25(Pho
aEPYIAsEYk	F1LSM8	N-Term(iTRAQ4plex); S7(Phospho); K10(iTR
dQsPQGDEEkEPPk	D4A6X3	N-Term(iTRAQ4plex); S3(Phospho); K10(iTR
asTFcGTPDYIAPEILQGLI	D4A0U0	N-Term(iTRAQ4plex); S2(Phospho); C5(Carb
aPHWTSASLTEAAAHPHS	A0A0U1RRX4;F1MAQ5	N-Term(iTRAQ4plex); S18(Phospho); M21(O
iTIPVQTFsNLQIR	P47819	N-Term(iTRAQ4plex); S9(Phospho)
sILLGsDNEDEk	D3ZN60	N-Term(iTRAQ4plex); S6(Phospho); K12(iTR
qAGDPsNTPAEDR	B5DF41	N-Term(iTRAQ4plex); S6(Phospho)
gSLDsDNDDSDcPYSEk	Q9JI66	N-Term(iTRAQ4plex); S5(Phospho); C12(Cai
aPPPLPPVYSMETEtPTAE	F1MAA3	N-Term(iTRAQ4plex); T15(Phospho); K25(iTI
aAsALLLRsPR	B2RZ79	N-Term(iTRAQ4plex); S3(Phospho); S9(Phos
sPVEAksPAEAk	F1LRZ7	N-Term(iTRAQ4plex); S1(Phospho); K6(iTRA

Information Classification: General

tSLNPFDEEDLsSPIEGAV	D3ZL11	N-Term(iTRAQ4plex); S12(Phospho); K36(iT
sLEsDNEEk	Q05695	N-Term(iTRAQ4plex); S4(Phospho); K9(iTRA
tGGGGGAsGSDEDEVSE	E9PTK9	N-Term(iTRAQ4plex); S8(Phospho); K34(iTR
sssSPELQTLQDILGDLGD	D3ZLW4	N-Term(iTRAQ4plex); S2(Phospho); S3(Phos
sPMkEEAksPEk	F1LRZ7	N-Term(iTRAQ4plex); K4(iTRAQ4plex); K8(iT
sTVAScMHR	P11275	N-Term(iTRAQ4plex); S1(Phospho); C6(Carb
eLYsPSGEkDDTDR	G3V9G5	N-Term(iTRAQ4plex); S4(Phospho); K9(iTRA
sPAEVksPAVAksPAEVk	F1LRZ7	N-Term(iTRAQ4plex); K6(iTRAQ4plex); S7(P
lHGsVPNLSR	D4A0G0	N-Term(iTRAQ4plex); S4(Phospho)
lVSQEHLLLsSPEALR	D3ZPJ0	N-Term(iTRAQ4plex); S10(Phospho)
sRsGSIIGSR	D4A720	N-Term(iTRAQ4plex); S1(Phospho); S3(Phos
sDQDSSTSSTSFPk	P47860	N-Term(Acetyl); S1(Phospho); K14(iTRAQ4p
tGAELDIkDVsDERLsPAk	P15205;F1LRL9	N-Term(iTRAQ4plex); K8(iTRAQ4plex); S11(
vsVDsNLFVYSk	F1M3W5	N-Term(iTRAQ4plex); S2(Phospho); S5(Phos
aEGEsEGPNPEPR	B1H240	N-Term(iTRAQ4plex); S5(Phospho)
gsGEGQGQILQR	M0RAP5	N-Term(iTRAQ4plex); S2(Phospho)
kIPDPDsDDVSEVDAR	G3V790	N-Term(iTRAQ4plex); K1(iTRAQ4plex); S7(P
cDEsPNREVQSSEAEALA	D3ZHX3	N-Term(iTRAQ4plex); C1(Carbamidomethyl);
gGsLkGDIGGLTSVLDNQ	Q923K6	N-Term(iTRAQ4plex); S3(Phospho); K5(iTRA
rEsPSPAPkPR	B2RYB3;A0A0G2K4F6	N-Term(iTRAQ4plex); S3(Phospho); K9(iTRA
hPLLSPGGPQsPLR	A0A0G2K5C0	N-Term(iTRAQ4plex); S11(Phospho)
vkEkAEEEGGsEEEVGDk	P12839	N-Term(iTRAQ4plex); K2(iTRAQ4plex); K4(iT
mLAsPEDFETVREEDR	A0A0G2K0F3	N-Term(iTRAQ4plex); S4(Phospho)
aGGAGEGsDDDTSLT	Q5BJN1	N-Term(iTRAQ4plex); S8(Phospho)
sDsPEsDTEREk	D3ZJ92	N-Term(iTRAQ4plex); S3(Phospho); S6(Phos
tGssPTQGIVNk	B0VXR4	N-Term(iTRAQ4plex); S3(Phospho); S4(Phos
iETQTQEEVRDskENTEk	G3V7X2	N-Term(iTRAQ4plex); S12(Phospho); K13(iT
sEAEEGEVRtPTk	A0A0G2K0F3	N-Term(iTRAQ4plex); T10(Phospho); K13(iT
qkSDAEEDGGTGsQDEEI	P35565	N-Term(iTRAQ4plex); K2(iTRAQ4plex); S13(
eLEkPIQSkPQSPVIQATA	Q7TSU1	N-Term(iTRAQ4plex); K4(iTRAQ4plex); K9(iT
vHGHsDEEEEEEQPR	A0A0G2JYN0	N-Term(iTRAQ4plex); S5(Phospho)
tPPGSGEPPk	D4A1Q2	N-Term(iTRAQ4plex); T1(Phospho); K10(iTR
tAGTSFMMtPyVVTR	A9LRT4	N-Term(iTRAQ4plex); T9(Phospho); Y11(Pho
sPsDSMAEGER	F1LPQ9	N-Term(iTRAQ4plex); S3(Phospho)
tEDGGWEWsDDEFDEES	D3ZUC9	N-Term(iTRAQ4plex); S9(Phospho); K21(iTR
asSPcQEFEQFQIIPTVET	A0A0G2KA12	N-Term(iTRAQ4plex); S2(Phospho); C5(Carb
iHVsDQELQSANASVDDS	Q5U300	N-Term(iTRAQ4plex); S4(Phospho)
nDGVkESSEstNttIEDEDT	P11275	N-Term(iTRAQ4plex); K5(iTRAQ4plex); S10(
ksPSEAR	P15205;F1LRL9	N-Term(iTRAQ4plex); K1(iTRAQ4plex); S2(P
sSPAAkPGSTPSRPSsAk	M0R8A4	N-Term(iTRAQ4plex); K6(iTRAQ4plex); S16(
sLMSsPEDLTkDFEELk	P15205;F1LRL9	N-Term(iTRAQ4plex); S5(Phospho); K11(iTR
dRtPEEkDR	P34926;Q63330	N-Term(iTRAQ4plex); T3(Phospho); K7(iTRA
dSsAENLDASQER	D3ZMR2	N-Term(iTRAQ4plex); S3(Phospho)
sAsPDDDLGSSNWEAAD	G3V8R0	N-Term(iTRAQ4plex); S3(Phospho)
fGIHVYQFPEcDsDEDEDF	D3ZT07	N-Term(iTRAQ4plex); C11(Carbamidomethyl
sQPLALPPPPPPPLPLPPI	Q66HK1	N-Term(iTRAQ4plex); S21(Phospho)
qSDsNAsFLR	F1M9N9	N-Term(iTRAQ4plex); S4(Phospho); S7(Phos
qSsFEIPDDVPLPAGWEM	R9PXS9	N-Term(iTRAQ4plex); S3(Phospho); K20(iTR
vIHDNFGIVEGLMTTVHAI	M0R660	N-Term(iTRAQ4plex); T22(Phospho); K24(iT
dVTNLtVGGFTPMSPR	E9PTB2	N-Term(iTRAQ4plex); T6(Phospho)
yGLHEHRDGsPTDR	Q5FVJ4	N-Term(iTRAQ4plex); S10(Phospho)
sTGVSFWTQDsDENEQE	D4ACS0	N-Term(iTRAQ4plex); S11(Phospho)
vcVGsPPAPGFGsSPPGA	D3Z9J7	N-Term(iTRAQ4plex); C2(Carbamidomethyl);
qHEVEIPsPTQk	A0A140TAB1	N-Term(iTRAQ4plex); S8(Phospho); K12(iTR
eSEIsPTNTVSQTTSSGA	G3V8Q9	N-Term(iTRAQ4plex); S5(Phospho); K25(iTR

tRHSPtPQQSNR	B2RYB3;A0A0G2K4F6	N-Term(iTRAQ4plex); T1(Phospho); T6(Phos
sELDTIDsQHR	P31596;Q8K5B5	N-Term(iTRAQ4plex); S8(Phospho)
gYSSPEPDVQDssGsEAQ	A0A0G2JXN8	N-Term(iTRAQ4plex); S12(Phospho); S13(Ph
eRLEscDR	Q5M9I5	N-Term(iTRAQ4plex); S5(Phospho); C6(Carb
sPVTVksPAEAksPVEVk	F1LRZ7	N-Term(iTRAQ4plex); K6(iTRAQ4plex); S7(P
sRTsVQtEDDQLIAGQSAF	A0A0G2JYF7;D4A6H8	N-Term(iTRAQ4plex); S4(Phospho); T7(Phos
sVsNEGLNNSR	A0A0G2K7K9	N-Term(iTRAQ4plex); S3(Phospho)
gHtSPcGMk	D4AD03	N-Term(iTRAQ4plex); T3(Phospho); C6(Carb
aTWGDGGDSsPSNVVSk	M0R4V3	N-Term(iTRAQ4plex); S10(Phospho); K17(iT
sDAFLSk	F1M3W5	N-Term(iTRAQ4plex); S1(Phospho); K7(iTRA
iLGSAsPEEEQEkPILDRP	Q5XIA2	N-Term(iTRAQ4plex); S6(Phospho); K13(iTR
gYSFTTtAER	P60711;P63259	N-Term(iTRAQ4plex); T7(Phospho)
aEEVILAEDEtDVEQR	A0A0G2JXK1	N-Term(iTRAQ4plex); T11(Phospho)
rVIENTDGsEEEMDAR	E9PTU4	N-Term(iTRAQ4plex); S9(Phospho)
gEVNAADAFDIGsFDEED	A0A0G2JZ82	N-Term(iTRAQ4plex); S13(Phospho); K20(iT
rIDIsPSALR	B1WC16	N-Term(iTRAQ4plex); S5(Phospho)
hSSLPTEsDEDIAPAQR	B5DFK6	N-Term(iTRAQ4plex); S8(Phospho)
lVDAIcDkMsDSMDTDPSV	Q63941	N-Term(iTRAQ4plex); C6(Carbamidomethyl);
lHLESPQPsPk	Q5PQP4	N-Term(iTRAQ4plex); S9(Phospho); K11(iTR
dLESLsPR	Q568Z1	N-Term(iTRAQ4plex); S6(Phospho)
qSRPVAGGPGAPPAARP	P09951	N-Term(iTRAQ4plex); S20(Phospho)
lLSTITEPTSAAPcDPPLVE	D3ZS72	N-Term(iTRAQ4plex); C14(Carbamidomethyl
sTSIPSPQAVPGNkcSGM	D3Z8C4	S1(Phospho); N-Term(iTRAQ4plex); K14(iTR
wRPSIGVQVETISDsDtEN	G3V7T8	N-Term(iTRAQ4plex); S15(Phospho); T17(Ph
gGLsDGEGPPGGR	Q02563	N-Term(iTRAQ4plex); S4(Phospho)
gGQDDDDAETGLtEGEGI	G3V6M3	N-Term(iTRAQ4plex); T13(Phospho); K23(iT
qPGFPQPSPSDDPSLsPF	Q99MC0	N-Term(iTRAQ4plex); S16(Phospho)
gSDEVTAsSAATGTSPR	F1LPH1	N-Term(iTRAQ4plex); S8(Phospho)
kALsFDNR	F1LMV8	N-Term(iTRAQ4plex); K1(iTRAQ4plex); S4(P
lPVHVYEVDEEADkDEDA	F1LSM8	N-Term(iTRAQ4plex); K14(iTRAQ4plex); S20
sRsRsPPPVSk	Q6PDU1	N-Term(iTRAQ4plex); S3(Phospho); S5(Phos
vLELcPSISFQSTGEAGDS	D3ZJH2	N-Term(iTRAQ4plex); C5(Carbamidomethyl);
ekNQWQLsLDDLkk	P13233	N-Term(iTRAQ4plex); K2(iTRAQ4plex); S8(P
tGLGESTASLDsttRDEGV	F1MAS4	N-Term(iTRAQ4plex); S12(Phospho); T13(Ph
dEVHVSDEFSENRSSVSk	F1LQN3	N-Term(iTRAQ4plex); K18(iTRAQ4plex); S22
qEATPQAGSDsDSGGGD	D4A3E1	N-Term(iTRAQ4plex); S11(Phospho)
sLPGsALELR	F1LSE6	N-Term(iTRAQ4plex); S1(Phospho); S5(Phos
kAsGPPVSELITk	P15865;A0A0G2K654;M0I	N-Term(iTRAQ4plex); K1(iTRAQ4plex); S3(P
dENPFASLTATSQPIATAA	F1M8V2	N-Term(iTRAQ4plex); S21(Phospho)
aLALVPGtPTR	P34926	N-Term(iTRAQ4plex); T8(Phospho)
eSEGsPDTDAAPGPDTDV	A0A0G2JW88	N-Term(iTRAQ4plex); S5(Phospho); K22(iTR
gTWPNSVLGEVTEVDSs(	Q6RJR6	N-Term(iTRAQ4plex); S17(Phospho); S20(Ph
gHLLLDSDERPsPEEEEG	G3V676	N-Term(iTRAQ4plex); S12(Phospho); K19(iT
lHGRsFDDPIVQTER	B4F772	N-Term(iTRAQ4plex); S5(Phospho)
aksPVkEEIkPPAEVk	F1LRZ7	N-Term(iTRAQ4plex); K2(iTRAQ4plex); S3(P
gEDLDFWLStTPPPAAAP	B5DFK6	N-Term(iTRAQ4plex); T10(Phospho); K36(iT
iTsFEEAk	P63329	N-Term(iTRAQ4plex); S3(Phospho); K8(iTRA
tcsFGGFDLTNR	F1LU97	N-Term(iTRAQ4plex); C2(Carbamidomethyl);
eLVGDTGSQEGDNEQPs	D3ZR35	N-Term(iTRAQ4plex); S17(Phospho); S19(Ph
sPAEAksPVEVksPASVk	F1LRZ7	N-Term(iTRAQ4plex); K6(iTRAQ4plex); S7(P
yAPtTTPsPEDGFEVQPLH	P28572	N-Term(iTRAQ4plex); T4(Phospho); S8(Phos
vEHLPLLTIEPPSDSsVEL	A0A0G2JUG7	N-Term(iTRAQ4plex); S16(Phospho)
lEAPPPPNLGSGLGTkPLI	D3ZC15	N-Term(iTRAQ4plex); K16(iTRAQ4plex); S26
dEVLtVIRRVDDNWAEGM	M0R6D9	N-Term(iTRAQ4plex); T5(Phospho); K22(iTR
eENQTGPTTtPSDTQDLD	Q920Q0	N-Term(iTRAQ4plex); T10(Phospho); K20(iT

yAsENVNk	Q6NYB7	N-Term(iTRAQ4plex); S3(Phospho); K8(iTRA
aGGGRPsSPSPSVVSEk	F1LLX6	N-Term(iTRAQ4plex); S7(Phospho); K17(iTR
eTELtFSLSPDELGTSSIM	A0A0G2JYD4	N-Term(iTRAQ4plex); T5(Phospho); K20(iTR
eLLSPLsDPDDRYPLIVk	D3ZSX2	N-Term(iTRAQ4plex); S7(Phospho); K18(iTR
iAFDAESEPNNSSGsMEk	A0A0G2K4S6	N-Term(iTRAQ4plex); S15(Phospho); K18(iT
dAsPVGLk	Q8VHW9	N-Term(iTRAQ4plex); S3(Phospho); K8(iTRA
yFDDEFTAQSITItPPDR	P47197	N-Term(iTRAQ4plex); T14(Phospho)
aGDmLEDsPkRPk	Q8VHK7	N-Term(iTRAQ4plex); M4(Oxidation); S8(Pho
kVAELEEEQsQGsSSYSD	P34926;Q63330	N-Term(iTRAQ4plex); K1(iTRAQ4plex); S10(
lVVLDEELEGVsPDELkDE	M0RDJ4	N-Term(iTRAQ4plex); S12(Phospho); K17(iT
rPTEAVsPk	A0A0G2QC38	N-Term(iTRAQ4plex); S7(Phospho); K9(iTRA
dDVsPEEQGk	D4A1J3	N-Term(iTRAQ4plex); S4(Phospho); K10(iTR
nVEDTDGSsPEDLmAALT	Q6RJR6	N-Term(iTRAQ4plex); S9(Phospho); M14(Ox
gsVQELSTIQIR	Q63881	N-Term(iTRAQ4plex); S2(Phospho)
sLPsNGELDPDVLESMAS	B2DD29	N-Term(iTRAQ4plex); S4(Phospho); C21(Ca
qLSYsDSDLkR	D4ACD9	N-Term(iTRAQ4plex); S5(Phospho); K10(iTR
aETEEAEEPEEDGEDNVs	P15205;F1LRL9	N-Term(iTRAQ4plex); S18(Phospho); K23(iT
aIGSGESEtPPSTPTSQEI	D4A224	N-Term(iTRAQ4plex); T9(Phospho)
eTAPTSAYSsPAR	D3ZC55	N-Term(iTRAQ4plex); S10(Phospho)
sLAESGLSWFsESEEk	F1M031	N-Term(iTRAQ4plex); S11(Phospho); K16(iT
sADVsPTTEGVk	D3ZQG6	N-Term(iTRAQ4plex); S1(Phospho); S5(Phos
tSSkEssPVPsPTSDRk	G3V6S0	N-Term(iTRAQ4plex); K4(iTRAQ4plex); S6(P
dGskEPIVEMR	F1M8G9	N-Term(iTRAQ4plex); S3(Phospho); K4(iTRA
eSEAGTGSsEHEDGER	F1M3W5	N-Term(iTRAQ4plex); S9(Phospho)
eENLPEEsEER	Q5BJK8	N-Term(iTRAQ4plex); S8(Phospho)
sPSVDLLGSPEPLATQQs	Q66HG9	N-Term(iTRAQ4plex); S18(Phospho); C25(Ca
rVIHFVSGETMEEYstDED	D3ZIM7	N-Term(iTRAQ4plex); S15(Phospho); T16(Ph
eHHPEEEFsGsEVEEVPE	F1M842	N-Term(iTRAQ4plex); S9(Phospho); S11(Pho
kGsPGLTR	F1LMT5	N-Term(iTRAQ4plex); K1(iTRAQ4plex); S3(P
tsPYGVPGSPATR	F1LRY7	N-Term(iTRAQ4plex); S2(Phospho)
rAsGQAFELILkPPsPISEA	P21818	N-Term(iTRAQ4plex); S3(Phospho); K12(iTR
hNPVFGVms	Q4V7C7	N-Term(iTRAQ4plex); M8(Oxidation); S9(Pho
gTEPSPGGTPQPsRPGsI	D3ZUL4	N-Term(iTRAQ4plex); S13(Phospho); S17(Ph
aAAtPESQEPQAk	A0A0G2K613	N-Term(iTRAQ4plex); T4(Phospho); K13(iTR
lNETELTDLEAQQEsPPkN	D4A517	N-Term(iTRAQ4plex); S15(Phospho); K18(iT
rAEPPkPEVVDSTESIPVs	A0A096MK35	N-Term(iTRAQ4plex); K6(iTRAQ4plex); S19(
eDSPLSSPSsQPGtPDEQ	A0A0G2K774	N-Term(iTRAQ4plex); S10(Phospho); T14(Ph
sPGGSSGGEGALIGSGG	P97612	N-Term(iTRAQ4plex); S29(Phospho)
fIDkDQQPsGsEGEDDDAI	A0A0G2JZB6	N-Term(iTRAQ4plex); K4(iTRAQ4plex); S9(P
iTELDkDLEEVTmQLQDtP	A0A0A0MXV8	N-Term(iTRAQ4plex); K6(iTRAQ4plex); M13(
fRVsmDGEWLcLR	Q5FVJ0;A0A0G2K6A9	N-Term(iTRAQ4plex); S4(Phospho); M5(Oxid
fMDyVQLHSTDk	A0A0U1RS13	N-Term(iTRAQ4plex); Y4(Phospho); K12(iTR
eGDGsATTDAAPATSPk	P07936	N-Term(iTRAQ4plex); S5(Phospho); K17(iTR
kStPVRsPGGSTMMk	Q920Q0	N-Term(iTRAQ4plex); K1(iTRAQ4plex); T3(P
lQDSsDPDTcSEEEVSSR	A0A1B0GWY5	N-Term(iTRAQ4plex); S5(Phospho); C10(Ca
eMTTsADQITEVk	F1M4Q5	N-Term(iTRAQ4plex); S5(Phospho); K13(iTR
lLRQsPPLAGR	Q2THW7	N-Term(iTRAQ4plex); S5(Phospho)
fDLYDVDSksPNLSk	B5DEX3	N-Term(iTRAQ4plex); K9(iTRAQ4plex); S10(
gFsYFGEDLmP	F1LMV8	N-Term(iTRAQ4plex); S3(Phospho); M10(Ox
aIsPTSATSSGR	A0A0G2K9S4	N-Term(iTRAQ4plex); S3(Phospho)
lLLLERPsPVR	M0R3Z8	N-Term(iTRAQ4plex); S8(Phospho)
tSIGSSSSDEYkQEDSQG	Q9JKS6;D3Z9C7	N-Term(iTRAQ4plex); K12(iTRAQ4plex); S19
rsSDPSLNEk	Q5PQT2	N-Term(iTRAQ4plex); S2(Phospho); K10(iTR
nSLETVGtPDSGR	Q62747	N-Term(iTRAQ4plex); T8(Phospho)
kHsPGEITGPGGHFMTSD	P34926;Q63330	N-Term(iTRAQ4plex); K1(iTRAQ4plex); S3(P

gmDASLsPTk	D3ZHV2	N-Term(iTRAQ4plex); M2(Oxidation); S7(Pho
rPsLEk	A0A0U1RRX4;F1MAQ5	N-Term(iTRAQ4plex); S3(Phospho); K6(iTRA
qEsDPEDDDVk	D3ZAY8	N-Term(iTRAQ4plex); S3(Phospho); K11(iTR
mAPTPIPTRsPSDSSTAS	Q07266;A0A0H2UHL9	N-Term(iTRAQ4plex); M1(Oxidation); S10(Ph
vEPPHssHEDLTDDLSTR	F1LUG5	N-Term(iTRAQ4plex); S6(Phospho); S7(Phos
qksVDGDEAk	A0A0G2JW68	N-Term(iTRAQ4plex); K2(iTRAQ4plex); S3(P
iPIVAQNSEEEsPLsPVGQ	D3Z9C7	N-Term(iTRAQ4plex); S12(Phospho); S15(Ph
gMHIHQVGGsPPASSTSS	D3ZDU2	N-Term(iTRAQ4plex); S10(Phospho); C19(Ca
gYSYFTGssPPLSPSTPSI	G3V984	N-Term(iTRAQ4plex); S8(Phospho); S9(Phos
dkEPDVLFVGDsMVQLMC	O35264	N-Term(iTRAQ4plex); K2(iTRAQ4plex); S12(
sTPSQVTSAEEkDGHsPM	P34926	N-Term(iTRAQ4plex); K12(iTRAQ4plex); S16
gFsEEQLR	P37805	N-Term(iTRAQ4plex); S3(Phospho)
qLDHEsDDADREDDER	F1M3W5	N-Term(iTRAQ4plex); S6(Phospho)
nSASEEDHLPLQVLQsP	A0A0G2JSH6	N-Term(iTRAQ4plex); S16(Phospho)
sPATVksPVEAksPAEVk	F1LRZ7	N-Term(iTRAQ4plex); K6(iTRAQ4plex); S7(P
rGGPISFSSsR	G3V9Y1	N-Term(iTRAQ4plex); S10(Phospho)
eMQVsDR	Q8CH83	N-Term(iTRAQ4plex); S5(Phospho)
fsPVLGR	D3ZNS8	N-Term(iTRAQ4plex); S2(Phospho)
dIkEEsDEEDDDDEESGR	D3ZTA8	N-Term(iTRAQ4plex); K3(iTRAQ4plex); S6(P
sTsMQDTVR	A0A0U1RS13	N-Term(iTRAQ4plex); S3(Phospho)
eTQDkAEAESDGQtEETA	D4A554	N-Term(iTRAQ4plex); K5(iTRAQ4plex); T14(
mHDsHLsSEEPk	P31596;Q8K5B5	N-Term(iTRAQ4plex); M1(Oxidation); S4(Pho
tQADPEDIAAHSGREELsS	Q5D022	N-Term(iTRAQ4plex); S18(Phospho); K26(iT
lEDSDsATSEADTDIAAk	Q4KM37	N-Term(iTRAQ4plex); S6(Phospho); K18(iTR
eDDGTFGEYsDAEDHkPL	Q6QRP0	N-Term(iTRAQ4plex); S10(Phospho); K16(iT
tsPSSSPASLSR	P19836	N-Term(iTRAQ4plex); S2(Phospho)
aASADsTTEGAPTDGFTV	A0A140TAB4	N-Term(iTRAQ4plex); S6(Phospho); K22(iTR
rDsDMTGHIQQPGGR	Q75T81	N-Term(iTRAQ4plex); S3(Phospho)
skEEQELHDIHsTR	A0A1B0GWN8	N-Term(iTRAQ4plex); K2(iTRAQ4plex); S12(
gkEELSAsGkGSPR	D3ZF03	N-Term(iTRAQ4plex); K2(iTRAQ4plex); S8(P
rPPVVVNLStSPR	D3ZTF0	N-Term(iTRAQ4plex); T10(Phospho)
nSGATFTEGsWSPELPFD	G3V6N0	N-Term(iTRAQ4plex); S10(Phospho); K23(iT
yGmGTsVER	F7FKI5	N-Term(iTRAQ4plex); M3(Oxidation); S6(Pho
vSSAPIPcPSPGPAPSAVP	Q156J1	N-Term(iTRAQ4plex); C8(Carbamidomethyl);
sRPSsPAVR	O35986	N-Term(iTRAQ4plex); S5(Phospho)
kAsGSENEGDYNPGR	Q14TE9	N-Term(iTRAQ4plex); K1(iTRAQ4plex); S3(P
rsPPPSPTTQR	Q08877	N-Term(iTRAQ4plex); S2(Phospho)
vLHGAQtsDEEkDF	D4A4W6	N-Term(iTRAQ4plex); T7(Phospho); S8(Phos
fRDPGsENEFADDEHSTV	O88420	N-Term(iTRAQ4plex); S6(Phospho)
eHLGQGssQEMEk	G3V7X2	N-Term(iTRAQ4plex); S7(Phospho); S8(Phos
vDENENcsSLGSPSEPPQ	D4AAU4	N-Term(iTRAQ4plex); C7(Carbamidomethyl);
ssSPVTELTARsPVkQDk	A0A0G2K2M9	N-Term(iTRAQ4plex); S2(Phospho); S12(Pho
eGILADcEkETcsPVsEGE	D3ZSU3	N-Term(iTRAQ4plex); C7(Carbamidomethyl);
dADEEDsDEETSHLER	Q3B8P7	N-Term(iTRAQ4plex); S7(Phospho)
vsPLSFGR	Q66H98	N-Term(iTRAQ4plex); S2(Phospho)
dAsPVGVk	Q71RJ2	N-Term(iTRAQ4plex); S3(Phospho); K8(iTRA
pGPTPSATNVGsSGRSPS	B2RZD1	N-Term(iTRAQ4plex); S12(Phospho); K19(iT
eYAENIGDGRsPEFR	Q4V8K2	N-Term(iTRAQ4plex); S11(Phospho)
sLPVALEsDEETGDELk	D3ZWH2	N-Term(iTRAQ4plex); S8(Phospho); K17(iTR
sALTVQFVTGsFIEk	P61227	N-Term(iTRAQ4plex); S11(Phospho); K15(iT
sPGEAksPAEAk	F1LRZ7	N-Term(iTRAQ4plex); K6(iTRAQ4plex); S7(P
sPAEVksPATVk	F1LRZ7	N-Term(iTRAQ4plex); K6(iTRAQ4plex); S7(P
dEskEPIVEVR	F1LUV9;A0A0G2K0M8	N-Term(iTRAQ4plex); S3(Phospho); K4(iTRA
dLAEFSELEYSEmGSSFk	F1LQN3	N-Term(iTRAQ4plex); M13(Oxidation); K18(i
eAHsDDNPSEGDGAVR	D3ZYR0	N-Term(iTRAQ4plex); S4(Phospho)

dYNASAsTISPPSSMEEDI	P15205;F1LRL9	N-Term(iTRAQ4plex); S7(Phospho); K19(iTR
eGPEPPEEVPAPTtPPAPI	Q6MG48	N-Term(iTRAQ4plex); T14(Phospho); K19(iT
gDQVSQNGLPAEQGsPR	G3V6S0	N-Term(iTRAQ4plex); S15(Phospho)
aDSEASsGPLLDDk	F1M5M9	N-Term(iTRAQ4plex); S7(Phospho); K14(iTR
aAEFPDFYDsEEQMGPH(	O35314	N-Term(iTRAQ4plex); S10(Phospho); K24(iT
rDsLtSPEDELGAEVGDEA	D3ZT47	N-Term(iTRAQ4plex); S3(Phospho); T5(Phos
rAPsVANVGSHcDLSLk	C0JPT7	N-Term(iTRAQ4plex); S4(Phospho); C12(Ca
kAsIQk	M0R5U4	N-Term(iTRAQ4plex); K1(iTRAQ4plex); S3(P
tFEELsGIHDPkLHHPsQG	F1LPZ6	N-Term(iTRAQ4plex); S6(Phospho); K12(iTR
tPsTDEQQQVQEEVk	D3ZBW5	N-Term(iTRAQ4plex); S3(Phospho); K15(iTR
gPPtRSPQAQDTPVSIAG(	Q63330	N-Term(iTRAQ4plex); T4(Phospho); C23(Ca
iISsIEQk	P62260	N-Term(iTRAQ4plex); S4(Phospho); K8(iTRA
dETFGEYRsLEsDNEEk	Q05695	N-Term(iTRAQ4plex); S9(Phospho); S12(Ph
aNSWQLVEtPEkR	G3V927	N-Term(iTRAQ4plex); T9(Phospho); K12(iTR
kGsLDsDNDDEkDPQHSL	A0A0H2UHB7	N-Term(iTRAQ4plex); K1(iTRAQ4plex); S3(P
sLsADNFIGIQR	F1M4U0	N-Term(iTRAQ4plex); S3(Phospho)
tSLPQHVVNPPVGAAAAG	D3ZWQ0	N-Term(iTRAQ4plex); S23(Phospho); K29(iT
tLQPSNPTEGSNPDTLFQ	A0A0G2K366	N-Term(iTRAQ4plex); S20(Phospho)
sASSDTSEELNAQDsPk	Q9JJ19	N-Term(iTRAQ4plex); S15(Phospho); K17(iT
rAsVcAEAYNPDEEEDDAI	P12369	N-Term(iTRAQ4plex); S3(Phospho); C5(Carb
amSMASILTNTMEELEEs	A0A0G2K207	N-Term(iTRAQ4plex); M2(Oxidation); S18(Ph
yNtISYR	A0A0G2K774	N-Term(iTRAQ4plex); T3(Phospho)
eHFcEYTEDELNEILNGMI	A0A0G2K6R9;F1M9N9;A0	N-Term(iTRAQ4plex); C4(Carbamidomethyl);
qGsFTIDkPSSNIPIELIPHI	A0A0G2K315	N-Term(iTRAQ4plex); S3(Phospho); K8(iTRA
tGTWsPER	A0A0G2JXK1	N-Term(iTRAQ4plex); S5(Phospho)
aVEEQGDDQDsEk	D4ABT8	N-Term(iTRAQ4plex); S11(Phospho); K13(iT
eEAQPAsPSSVQDR	A0A0G2K0A8	N-Term(iTRAQ4plex); S7(Phospho)
vFDkDGNGyISAAELR	P62161	N-Term(iTRAQ4plex); K4(iTRAQ4plex); Y9(P
iQQQLGEEAsPR	A0A0G2JZE7	N-Term(iTRAQ4plex); S10(Phospho)
sAtPPPTEPASLPQEPPkF	Q6AY21	N-Term(iTRAQ4plex); T3(Phospho); K18(iTR
lSSQLsAGEEk	Q9Z1W6	N-Term(iTRAQ4plex); S6(Phospho); K11(iTR
eRsLESSQR	G3V849	N-Term(iTRAQ4plex); S3(Phospho)
tTYVQDsPAETk	F1LNZ5	N-Term(iTRAQ4plex); S7(Phospho); K12(iTR
vAVVRtPPkSPSASk	D4A1Q2	N-Term(iTRAQ4plex); T6(Phospho); K9(iTRA
lSQSsPDNITDPk	A0A0G2K7K9	N-Term(iTRAQ4plex); S5(Phospho); K13(iTR
eSVIGASEVAPPAAPPSS	A0A0G2K2L0	N-Term(iTRAQ4plex); S22(Phospho)
nLAkPGVTSTSDsEEDDD	Q566D6	N-Term(iTRAQ4plex); K4(iTRAQ4plex); S13(
sLSEDsGEEMDSVDPVTL	D3ZFH5	N-Term(iTRAQ4plex); S6(Phospho); K22(iTR
tLNIFPEVmLGPGLEEASs	D3ZH78	N-Term(iTRAQ4plex); M9(Oxidation); S19(Ph
rQsFTSLALR	A0A0G2K1R9	N-Term(iTRAQ4plex); S3(Phospho)
gRLsPVPVPR	Q5PQR0	N-Term(iTRAQ4plex); S4(Phospho)
tVEEPSNPEASSSTSVTPI	O54857	N-Term(iTRAQ4plex); S21(Phospho)
eDLsDVTDEDTGPAQPPF	D3ZKQ4	N-Term(iTRAQ4plex); S4(Phospho); K21(iTR
wPFDRsAEDLDLLNASFQ	D4ABN3	N-Term(iTRAQ4plex); S6(Phospho); K22(iTR
rGsmNNEMLSPEVGPVRI	Q5U2N3	N-Term(iTRAQ4plex); S3(Phospho); M4(Oxid
lPDsPTDFMHQIIDQVFQA	D4A626	N-Term(iTRAQ4plex); S4(Phospho)
gEPQcsPEGPAR	F1LRY7	N-Term(iTRAQ4plex); C5(Carbamidomethyl);
sPASVksPSEAk	F1LRZ7	N-Term(iTRAQ4plex); S1(Phospho); K6(iTRA
gAPGDHGPEGsGGER	Q7TP42	N-Term(iTRAQ4plex); S11(Phospho)
lTLVcsTAPGPLELDLTGD	Q5XI73	N-Term(iTRAQ4plex); C5(Carbamidomethyl);
nyVmIDDISELTkDSTPTA	G3V984	N-Term(iTRAQ4plex); Y2(Phospho); M4(Oxid
lQPEQEsPkkcEAEEAEPP	E9PST5	N-Term(iTRAQ4plex); S7(Phospho); K9(iTRA
lTGLERAEsNkIR	A7L638	N-Term(iTRAQ4plex); S9(Phospho); K11(iTR
dTASLSTTPsEsPR	M0R423	N-Term(iTRAQ4plex); S10(Phospho); S12(Ph
sDQAEEGGsEkEGsSEkD	P12839	N-Term(iTRAQ4plex); S9(Phospho); K11(iTR

ecSLsPk	D4A559	N-Term(iTRAQ4plex); C2(Carbamidomethyl);
vAAGHELQPLAIVDQRPS	P08050	N-Term(iTRAQ4plex); S19(Phospho); S23(Ph
sQEPISNDQkDsDDDkEk	Q9Z1W6	N-Term(iTRAQ4plex); K10(iTRAQ4plex); S12
kGTGDcsDEEVDGkADG	G3V6P7	N-Term(iTRAQ4plex); K1(iTRAQ4plex); C6(C
eLsAERPLNEQIAEAEADk	F1M7L6	N-Term(iTRAQ4plex); S3(Phospho); K19(iTR
esSVEEEVWHFLR	D4A830	N-Term(iTRAQ4plex); S2(Phospho)
sPVEEVkAkEPPk	F1LRZ7	N-Term(iTRAQ4plex); S1(Phospho); K7(iTRA
sPVksPEAk	P12839	N-Term(iTRAQ4plex); K4(iTRAQ4plex); S5(P
aGDAPsEEkkGEGDAAPS	P07936	N-Term(iTRAQ4plex); S6(Phospho); K9(iTRA
eVAGETGsPELSGVPR	G3V8G4	N-Term(iTRAQ4plex); S8(Phospho)
dSLDDLFPNEDEQsPAPS	B2GV74	N-Term(iTRAQ4plex); S14(Phospho)
rPMEEDGEEksPSk	A0A0G2K2T6	N-Term(iTRAQ4plex); K10(iTRAQ4plex); S11
dFQEDSWGETkHsPGVS	P34926;Q63330	N-Term(iTRAQ4plex); K11(iTRAQ4plex); S13
lGSTAPQVLNTsSPAQQA	A0A0G2K911	N-Term(iTRAQ4plex); S12(Phospho); K23(iT
eVEEDSEDEEMSEDEDD	P13383	N-Term(iTRAQ4plex); S18(Phospho); S19(Ph
yMAENPTAGVVQEEEED	D4A031	N-Term(iTRAQ4plex); S23(Phospho); K32(iT
rGPAETAAADSEADtDPEI	B2RYN2	N-Term(iTRAQ4plex); T15(Phospho)
tSISDPPEsPPLLPPREPV	D4A3T0	N-Term(iTRAQ4plex); S9(Phospho)
sGkNsQEDsEDsEEkDVk	A0A0G2K7X3	N-Term(iTRAQ4plex); K3(iTRAQ4plex); S5(P
aQsPLLPEPLk	F1LQZ9	N-Term(iTRAQ4plex); S3(Phospho); K11(iTR
kStPVRsPGGSTMmk	Q920Q0	K1(iTRAQ4plex); N-Term(iTRAQ4plex); T3(P
sPFEIIsPPAsPPEMTGQR	P34926	N-Term(iTRAQ4plex); S7(Phospho); S11(Pho
qDEDsDSEmGPPTEEPEI	F1M1U0	N-Term(iTRAQ4plex); S5(Phospho); M9(Oxid
sLVVsDEEEAEEEAEk	D4A2C4	N-Term(iTRAQ4plex); S5(Phospho); K16(iTR
lSLEsEGANEGAAAAPELS	D3ZQL7	N-Term(iTRAQ4plex); S5(Phospho)
kIAELEEERsQGsTSNSDV	F1LRL9	K1(iTRAQ4plex); N-Term(iTRAQ4plex); S10(
hAAPAPsAksSPSkHQPPI	Q62950	N-Term(iTRAQ4plex); S7(Phospho); K9(iTRA
eSVPSAsPTDTMk	A0A0G2JYD4	N-Term(iTRAQ4plex); S7(Phospho); K13(iTR
eDsGTFSLGk	G3V7J2	N-Term(iTRAQ4plex); S3(Phospho); K10(iTR
kVAVVRtPPk	D4A1Q2	N-Term(iTRAQ4plex); K1(iTRAQ4plex); T7(P
lAADVGkGsSQR	Q05962	N-Term(iTRAQ4plex); K7(iTRAQ4plex); S9(P
eSDILsDEEEDFHHPLk	D3ZMS5	N-Term(iTRAQ4plex); S6(Phospho); K17(iTR
aGSAETESAAkAttDNSPS	P07936	N-Term(iTRAQ4plex); K11(iTRAQ4plex); T13
eRPPFIDLETFNAGSDAG	Q5U1X7	N-Term(iTRAQ4plex); S19(Phospho)
eQSSsPSEPNPNPELR	A1A5P0	N-Term(iTRAQ4plex); S5(Phospho)
rAsGEEQGcLD	D3ZYS1	N-Term(iTRAQ4plex); S3(Phospho); C9(Carb
vIISt PSADAPmFVmGVNY	D3ZWV2	N-Term(iTRAQ4plex); T5(Phospho); M12(Ox
gAEQGsEEEkEEk	Q6MG08	N-Term(iTRAQ4plex); S6(Phospho); K10(iTR
gPPtRSPQAQDmPVSIAG	P34926	N-Term(iTRAQ4plex); T4(Phospho); M12(Ox
kGtGDcsDEEVDGk	G3V6P7	K1(iTRAQ4plex); N-Term(iTRAQ4plex); T3(P
tVtPASsAktsPAk	P47942	N-Term(iTRAQ4plex); T3(Phospho); S7(Phos
rsPPGPGVPRPPPR	A9CMA6	N-Term(iTRAQ4plex); S2(Phospho)
sADEcEGGSTPRPPEDSI	D4A1G8	N-Term(iTRAQ4plex); C5(Carbamidomethyl);
sSSVssPELk	A0A0G2K2M9	N-Term(iTRAQ4plex); S5(Phospho); S6(Phos
tTRSPDTSAYcYETMEk	P15205;F1LRL9	N-Term(iTRAQ4plex); T1(Phospho); C11(Car
mskSPVDSSVLEGVk	F1LMT5	M1(Oxidation); N-Term(iTRAQ4plex); S2(Pho
eQIEVIAPsPER	Q9Z1Z1	N-Term(iTRAQ4plex); S9(Phospho)
eSsPVPsPTSDRk	G3V6S0	N-Term(iTRAQ4plex); S3(Phospho); S7(Phos
eLTVDPTAAGDVRPIMHH	F1M8L1	N-Term(iTRAQ4plex); S35(Phospho); K42(iT
vTEEEDGLGQksPDANIPI	F1M4V3	N-Term(iTRAQ4plex); K11(iTRAQ4plex); S12
qRPAsPPPFLPATTEAEP	Q5XHX2	N-Term(iTRAQ4plex); S5(Phospho); K29(iTR
kQDDsPPRPIIGPALPPGF	Q4V893	N-Term(iTRAQ4plex); K1(iTRAQ4plex); S5(P
iAcDEEFsDSEDEGEGGR	Q99PA1	N-Term(iTRAQ4plex); C3(Carbamidomethyl);
dRsPIRGsPR	D3ZEI6	N-Term(iTRAQ4plex); S3(Phospho); S8(Phos
kDPEERPTFEYLQGFLED	A0A0G2K2E3	N-Term(iTRAQ4plex); K1(iTRAQ4plex); Y27(

sSSGMGGRtPVSRGR	A0A0A0MXW9	N-Term(iTRAQ4plex); T9(Phospho)
sTLPVDEGsPLEkLEQk	A0A0G2JW88	N-Term(iTRAQ4plex); S9(Phospho); K13(iTR
skDEEADsDNYEVLFNLE	D3ZZ32	N-Term(iTRAQ4plex); K2(iTRAQ4plex); S8(P
vLPALsPA	P25093	N-Term(iTRAQ4plex); S6(Phospho)
dLWPMVSPEDTQSLSFS	P34926;Q63330	N-Term(iTRAQ4plex); S20(Phospho); K23(iT
qAEVADQQTTDLPAENG	Q5U1W8	N-Term(iTRAQ4plex); S26(Phospho); K30(iT
kEDENQTEDPSTSPsPG	F1M8I7	N-Term(iTRAQ4plex); K1(iTRAQ4plex); S15(
sIcTLQITDTtGSHQFPAM	D3ZHX3	N-Term(iTRAQ4plex); C3(Carbamidomethyl);
lScsLEDLR	Q5UDQ9	N-Term(iTRAQ4plex); C3(Carbamidomethyl);
aGDsDEESRPDDk	G3V8P6	N-Term(iTRAQ4plex); S4(Phospho); K13(iTR
aPHWTSASLTEAAAHPH	A0A0U1RRX4;F1MAQ5	N-Term(iTRAQ4plex); S18(Phospho); K22(iT
fLSFsSLESLSSELEQTk	Q63128	N-Term(iTRAQ4plex); S5(Phospho); K18(iTR
fGQAATMEGIGAIGGtPP	Q5FVM4	N-Term(iTRAQ4plex); T16(Phospho); K33(iT
aEAPPLQREDsGTFSLGk	G3V7J2	N-Term(iTRAQ4plex); S11(Phospho); K18(iT
mEQLsDEEIDHGAEEDsD	D4ADX8	N-Term(Acetyl); S5(Phospho); S17(Phospho)
qPsIELPSMAVASTk	Q4QQV6	N-Term(iTRAQ4plex); S3(Phospho); K15(iTR
mLAsPEDFETVR	A0A0G2K0F3	N-Term(iTRAQ4plex); S4(Phospho)
gEAAAERPGEAAVASsPS	F1LMW7	N-Term(iTRAQ4plex); S16(Phospho); K19(iT
aAFSkDEskEPIVEVR	F1LUV9;A0A0G2K0M8	N-Term(iTRAQ4plex); K5(iTRAQ4plex); S8(P
dATDVPPPPcQtPAGPET	B0BN02	N-Term(iTRAQ4plex); C10(Carbamidomethyl
aSQPGPTAESQsSPHRR	A0A0G2K162	N-Term(iTRAQ4plex); S12(Phospho)
tELsPSFINPNPLEWFAGE	P15205	N-Term(iTRAQ4plex); S4(Phospho); K42(iTR
tPTTLSIkPLGAIsPVLNR	D3ZXY2	N-Term(iTRAQ4plex); K8(iTRAQ4plex); S14(
lAtPEDk	D3ZT07	N-Term(iTRAQ4plex); T3(Phospho); K7(iTRA
tQPTDEEMLFIySHFk	P11030	N-Term(iTRAQ4plex); Y12(Phospho); K16(iT
iIGVHQEDELLEcLsPATS	F1LM09	N-Term(iTRAQ4plex); C13(Carbamidomethyl
gGPISFSSsR	G3V9Y1	N-Term(iTRAQ4plex); S9(Phospho)
qAEDTISNASSQLSsPPT	F1M386	N-Term(iTRAQ4plex); S15(Phospho)
vIHDNFGIVEGLmtTVHAI	M0R660	N-Term(iTRAQ4plex); M13(Oxidation); T14(P
eVLPDHVGVLEGIDLsPE	F1M471	N-Term(iTRAQ4plex); S16(Phospho)
yQDEDGPHDHsLPR	Q63622	N-Term(iTRAQ4plex); S11(Phospho)
dIDTEPQSSILEsPkk	A0A0G2JZ27	N-Term(iTRAQ4plex); S13(Phospho); K15(iT
lsSPVLHR	Q07266;A0A0H2UHL9	N-Term(iTRAQ4plex); S2(Phospho)
dLEEVTMQLQDtPEk	A0A0A0MXV8	N-Term(iTRAQ4plex); T12(Phospho); K15(iT
mQFsFEEYEk	A0A0G2JZX5	N-Term(iTRAQ4plex); S4(Phospho); K10(iTR
iPFLESLGLPsPPR	A0A0H2UHB7	N-Term(iTRAQ4plex); S11(Phospho)
gPLVFGEPIAASLGTDGT	Q62950	N-Term(iTRAQ4plex); S22(Phospho); K23(iT
sTGVSFWTQDSDENEQE	D4ACS0	N-Term(iTRAQ4plex); S20(Phospho); K28(iT
rTLsIDk	P30835	N-Term(iTRAQ4plex); S4(Phospho); K7(iTRA
kVPEPsPVTR	D3ZCG2	N-Term(iTRAQ4plex); K1(iTRAQ4plex); S6(P
qLsEENANLQEYVEk	A0JPQ3	N-Term(iTRAQ4plex); S3(Phospho); K15(iTR
sGsISNYR	P54645	N-Term(iTRAQ4plex); S3(Phospho)
isPGDER	Q6AY81	N-Term(iTRAQ4plex); S2(Phospho)
gsPHLLR	D3ZE26	N-Term(iTRAQ4plex); S2(Phospho)
mGQAGStIsNSHAQPFDF	P08050	N-Term(iTRAQ4plex); T7(Phospho); S9(Phos
iEEELGskAk	M0R5J4	N-Term(iTRAQ4plex); S7(Phospho); K8(iTRA
tcTLPLTSLGR	D3ZWQ5	N-Term(iTRAQ4plex); T1(Phospho); C2(Carb
ssSLFGTASVALHAk	F1LZS9	N-Term(iTRAQ4plex); S2(Phospho); K15(iTR
dQAQEDAQVAAEILEIADt	P48037	N-Term(iTRAQ4plex); T19(Phospho); K24(iT
sQPGsPGR	D4A1Q9	N-Term(iTRAQ4plex); S5(Phospho)
aNMHISEsQQEFFR	D3ZU88	N-Term(iTRAQ4plex); S8(Phospho)
aQNPAYFEGkPAsLDEGA	A0A0G2JZX5	N-Term(iTRAQ4plex); K10(iTRAQ4plex); S13
sELDQLRQEAEQLk	P54311	N-Term(Acetyl); S1(Phospho); K14(iTRAQ4p
qDsLNQNPVSQR	G3V9C5	N-Term(iTRAQ4plex); S3(Phospho)
rVsRsLDGAPIGVVDQSLM	A0A0G2K162	N-Term(iTRAQ4plex); S3(Phospho); S5(Phos

Information Classification: General

sPAEAksPAEVksPATVk	F1LRZ7	N-Term(iTRAQ4plex); K6(iTRAQ4plex); S7(P
aSsLEDLVLk	Q6MG76	N-Term(iTRAQ4plex); S3(Phospho); K10(iTR
qEtVDcLk	P11275;P15791	N-Term(iTRAQ4plex); T3(Phospho); C6(Carb
gGPAEEPsPRGsPR	D3ZXD8	N-Term(iTRAQ4plex); S8(Phospho); S12(Pho
rYsPVAk	P31016	N-Term(iTRAQ4plex); S3(Phospho); K7(iTRA
vTGVYELSLcHVADAGsP	F1M4A4	N-Term(iTRAQ4plex); C10(Carbamidomethyl
nSGMPsGAAAIAVLPVTLI	P11348	N-Term(iTRAQ4plex); S6(Phospho)
sPVPksPVEEVkPkPEAk	P12839	N-Term(iTRAQ4plex); K5(iTRAQ4plex); S6(P
aQPsSSEDELDSVFFk	F1LXQ7	N-Term(iTRAQ4plex); S4(Phospho); K16(iTR
sLAEAksPEk	F1LRZ7	N-Term(iTRAQ4plex); K6(iTRAQ4plex); S7(P
lIPITGGNAGsPEDQHGk	M0RAP6	N-Term(iTRAQ4plex); S11(Phospho); K18(iT
vGIVEPSsATSGDSDDAA	D3ZJG4	N-Term(iTRAQ4plex); S8(Phospho); K39(iTR
gsVsEDELIAAIk	Q64548	N-Term(iTRAQ4plex); S2(Phospho); S4(Phos
sEGESQTVVSSGSDPk	Q3SWT7	N-Term(Acetyl); S1(Phospho); K16(iTRAQ4p
dLWPmVSPEDTQSLSFS	P34926;Q63330	N-Term(iTRAQ4plex); M5(Oxidation); S20(Ph
tGMYVAtLAGSQSPkPLM	G3V8F1	N-Term(iTRAQ4plex); T7(Phospho); K15(iTR
sPNTAILIk	Q9QXY2	N-Term(iTRAQ4plex); S1(Phospho); K9(iTRA
tPEELEDVsDLEDDHEVR	F1M4I1	N-Term(iTRAQ4plex); S9(Phospho)
vHSDsETDDIGFIPSk	P31016	N-Term(iTRAQ4plex); S5(Phospho); K16(iTR
iVFEDGNISVsk	Q62950	N-Term(iTRAQ4plex); S11(Phospho); K12(iT
wNsNDFIDAFASPTEVEG	Q5U2N3	N-Term(iTRAQ4plex); S3(Phospho); M25(Ox
aTIEEVPsEDEEEDAADG	D4ADP2	N-Term(iTRAQ4plex); S8(Phospho); K28(iTR
aNskSEGsPVLPHEPSk	D3ZZQ0	N-Term(iTRAQ4plex); S3(Phospho); K4(iTRA
eSSPVPsPTSDR	G3V6S0	N-Term(iTRAQ4plex); S7(Phospho)
eLkPEQETtPR	A0A0G2K0F3	N-Term(iTRAQ4plex); K3(iTRAQ4plex); T9(P
gsPSVAGAR	D3ZT16	N-Term(iTRAQ4plex); S2(Phospho)
gGcPGPGGAGtPPPR	O35052	N-Term(iTRAQ4plex); C3(Carbamidomethyl);
lQPQEIsPPPTANLDR	O35346	N-Term(iTRAQ4plex); S7(Phospho)
hVDPPsPR	B0BMV7	N-Term(iTRAQ4plex); S6(Phospho)
gLMAGGRPEGQYsEDED	B1H241	N-Term(iTRAQ4plex); S13(Phospho); T18(Ph
lNATTsLEQDk	E9PT53	N-Term(iTRAQ4plex); S6(Phospho); K11(iTR
rVsHsPPPk	B2RYB3;A0A0G2K4F6	N-Term(iTRAQ4plex); S3(Phospho); S5(Phos
iTNHEDGsPVNEPNETTP	F1M8G9	N-Term(iTRAQ4plex); S8(Phospho); K24(iTR
aTLPsPDkLPGFk	Q5U300	N-Term(iTRAQ4plex); S5(Phospho); K8(iTRA
gSSAAAPPGsPPPGR	D4A3D9	N-Term(iTRAQ4plex); S10(Phospho)
eDDAEsGGRRsEAEEGE	A0A0G2K0F3	N-Term(iTRAQ4plex); S6(Phospho); S11(Pho
sNsSEASSGDFLDLk	Q6AXU6	N-Term(iTRAQ4plex); S3(Phospho); K15(iTR
rAsFAEk	A0A0G2K1J5	N-Term(iTRAQ4plex); S3(Phospho); K7(iTRA
sDVEIPAtVTAFSFEDDSV	Q62847;A0A0G2JW28	N-Term(iTRAQ4plex); T8(Phospho); S22(Pho
lTESMTNVLEGDSMDQD	Q8R5M1	N-Term(iTRAQ4plex); S20(Phospho); K28(iT
tLsDESIYSSQR	F1LS65	N-Term(iTRAQ4plex); S3(Phospho)
vcSsMDENDGPGEEESD	O08875;A0A0G2KB92	N-Term(iTRAQ4plex); C2(Carbamidomethyl);
qQAsTDSPcSLR	Q9Z2X9	N-Term(iTRAQ4plex); S4(Phospho); C9(Carb
vWEDRPSsAGSTPFPYNI	A0A0G2JU91	N-Term(iTRAQ4plex); S8(Phospho)
aFsGEGQk	P0C627	N-Term(iTRAQ4plex); S3(Phospho); K8(iTRA
aVLLTAVELLQEyDsNSD	F1LU97	N-Term(iTRAQ4plex); Y13(Phospho); S15(Ph
lEGGLPQEPTsR	A0A0G2JSM7	N-Term(iTRAQ4plex); S11(Phospho)
nkPSAtDsEGEDDEDmTk	Q6MG08	N-Term(iTRAQ4plex); K2(iTRAQ4plex); T6(P
mAPTPIPTRsPsDSSTAS	Q07266;A0A0H2UHL9	M1(Oxidation); N-Term(iTRAQ4plex); S10(Ph
sSGDGTEWDkDDLsPTA	P58405	N-Term(iTRAQ4plex); K10(iTRAQ4plex); S14
lSPsPTSQR	G3V8L3	N-Term(iTRAQ4plex); S4(Phospho)
eVLDAsDkEGLsPAk	Q5RKH1	N-Term(iTRAQ4plex); S6(Phospho); K8(iTRA
vVQEYIDAFSDyANFk	Q03348	N-Term(iTRAQ4plex); Y12(Phospho); K16(iT
aMGDEEsGDSDGsPkTsP	G3V7T8	N-Term(iTRAQ4plex); S7(Phospho); S13(Pho
sLsELESLk	D4A9D8	N-Term(iTRAQ4plex); S3(Phospho); K9(iTRA

nPsLGEIQV	M0RD53	N-Term(iTRAQ4plex); S3(Phospho)
aEsPVkEk	P12839	N-Term(iTRAQ4plex); S3(Phospho); K6(iTRA
vSsAGLSLk	P23565;G3V8Q2	N-Term(iTRAQ4plex); S3(Phospho); K9(iTRA
gAGAAssTDSLDTLSNGP	F1LRZ7	N-Term(iTRAQ4plex); S6(Phospho); S7(Phos
vVtPPAASDIPADSPAPGF	F1M1Y0	N-Term(iTRAQ4plex); T3(Phospho)
dLsLEEIQkk	P21818;A0A096MK73	N-Term(iTRAQ4plex); S3(Phospho); K9(iTRA
nLGTPTSSTPRPSItPtkk	Q5U2P5;A0A0G2K2V4	N-Term(iTRAQ4plex); T15(Phospho); T17(Ph
aAQQTkGsYmEVEDNR	A0A0H2UHZ1	N-Term(iTRAQ4plex); K6(iTRAQ4plex); S8(P
skPPGSPVSSMSADQAS	A0A0G2JWF4	N-Term(iTRAQ4plex); S1(Phospho); K2(iTRA
vQSLEGEkLsPk	P15205;F1LRL9	N-Term(iTRAQ4plex); K8(iTRAQ4plex); S10(
dIDTEPQSSILEsPk	A0A0G2JZ27	N-Term(iTRAQ4plex); S13(Phospho); K15(iT
aATATRPPGPPPAPQPPs	D3ZH75	N-Term(iTRAQ4plex); S18(Phospho); S23(Ph
lLStSPALQGTPASR	O35095	N-Term(iTRAQ4plex); T4(Phospho)
ktSLVIVESTDDQPQVFEk	A0A0G2K6R9;F1M9N9;A0	N-Term(iTRAQ4plex); K1(iTRAQ4plex); T2(P
qSELSAEEsPEk	Q5BJT7	N-Term(iTRAQ4plex); S9(Phospho); K12(iTR
sSDWssEEEEPVRk	Q6PST4	N-Term(iTRAQ4plex); S5(Phospho); S6(Phos
aPEsDTGDEDQDQER	A0A0G2K0F3	N-Term(iTRAQ4plex); S4(Phospho)
rDPsLEEIQk	P63043	N-Term(iTRAQ4plex); S4(Phospho); K10(iTR
dkYEPAAVsEHGDkk	P06685	N-Term(iTRAQ4plex); K2(iTRAQ4plex); S9(P
kEEsEEsEDDmGFGLFD	A0A0G2K4Q1	K1(iTRAQ4plex); N-Term(iTRAQ4plex); S4(P
tIAPALVsk	M0R5J4	N-Term(iTRAQ4plex); S8(Phospho); K9(iTRA
eIssPSSPVkVEVTVTDPV	A0A0G2K6R9;F1M9N9	N-Term(iTRAQ4plex); S3(Phospho); S4(Phos
qPsLDVDVGDPNPDVVSF	Q01066	N-Term(iTRAQ4plex); S3(Phospho)
eGDGsATTDAAPATsPk	P07936	N-Term(iTRAQ4plex); S5(Phospho); S15(Pho
aDGYEPPVQEsV	M0R6L4	N-Term(iTRAQ4plex); S11(Phospho)
sDYmEIQEGVNNsNEDFF	P63142	N-Term(iTRAQ4plex); M4(Oxidation); S13(Ph
gPPEsDSPk	D3Z9Z0	N-Term(iTRAQ4plex); S5(Phospho); K9(iTRA
vAPLATTEDSLASIPFIDEF	F1M2D4	N-Term(iTRAQ4plex); S22(Phospho); K30(iT
fVsFTSR	Q9JK73	N-Term(iTRAQ4plex); S3(Phospho)
lFDDsDER	D4A997	N-Term(iTRAQ4plex); S5(Phospho)
gsYmEVEDNRsQVETEDL	A0A0H2UHZ1	N-Term(iTRAQ4plex); S2(Phospho); M4(Oxid
sAPTSPcDQELk	F1LMV8	N-Term(iTRAQ4plex); S1(Phospho); C7(Carb
vEAkEEsEEsDEDmGFGL	P19945	N-Term(iTRAQ4plex); K4(iTRAQ4plex); S7(P
ksSTVsEDVEATVPMLQR	Q4V8I7	K1(iTRAQ4plex); N-Term(iTRAQ4plex); S2(P
lGSLTPEIVStPSSPEEEDk	P0C627	N-Term(iTRAQ4plex); T11(Phospho); K20(iT
sEGGAAWDtPGAsQEELF	Q63638	N-Term(iTRAQ4plex); T9(Phospho); S13(Pho
kVELsESEEDk	B2RYB3;A0A0G2K4F6	N-Term(iTRAQ4plex); K1(iTRAQ4plex); S5(P
dLSsEEk	Q9JJW1	N-Term(iTRAQ4plex); S4(Phospho); K7(iTRA
sPAEAksPVVAksPAEAk	F1LRZ7	N-Term(iTRAQ4plex); K6(iTRAQ4plex); S7(P
dTAGDAsESALLk	P06686	N-Term(iTRAQ4plex); S7(Phospho); K13(iTR
sTsQGSINSPVYSR	A0A0G2JW01;F1LWK7	N-Term(iTRAQ4plex); S3(Phospho)
dFQEYVEPGEDFPAsPQF	G3V7G0	N-Term(iTRAQ4plex); S15(Phospho)
eksPPDQSAVPNTPPstPV	A0A0G2JSM7;D3ZZ99	N-Term(iTRAQ4plex); K2(iTRAQ4plex); S3(P
tSASPPLEksGDEGsEDEA	A0A0G2K7X3	N-Term(iTRAQ4plex); K9(iTRAQ4plex); S10(
rPsLLR	Q9P290	N-Term(iTRAQ4plex); S3(Phospho)
nTEEsSSPVRk	E9PSN4	N-Term(iTRAQ4plex); S5(Phospho); K11(iTR
lVGMDPEQAsIANQGEYIF	A0JPI6	N-Term(iTRAQ4plex); S10(Phospho)
iNHTVILDDPFDDPPDLLIF	D4AEG3	N-Term(iTRAQ4plex); S23(Phospho); K28(iT
kRsPsPSPTPEAk	D4A510	N-Term(iTRAQ4plex); K1(iTRAQ4plex); S3(P
aLVHQLsNESR	D3ZHZ3	N-Term(iTRAQ4plex); S7(Phospho)
eYTmsWMk	A0A0G2JWM2	N-Term(iTRAQ4plex); M4(Oxidation); S5(Pho
fFScMQsDFSk	A0A0G2JWM2	N-Term(iTRAQ4plex); C4(Carbamidomethyl);
kVAELEEEQsQGSSSYSD	P34926;Q63330	N-Term(iTRAQ4plex); K1(iTRAQ4plex); S10(
lVLASIDQADFQGFTYVNF	P63319	N-Term(iTRAQ4plex); S31(Phospho)
aGsDSFGPGEWDDR	A0A0G2K6F5	N-Term(iTRAQ4plex); S3(Phospho)

nTYDVVyLk	P31016	N-Term(iTRAQ4plex); Y7(Phospho); K9(iTRA
ekPNSGGtkSDsASDSQE	F1M110	N-Term(iTRAQ4plex); K2(iTRAQ4plex); T8(P
sPAEAkPPAEAksPAEAks	F1LRZ7	N-Term(iTRAQ4plex); K6(iTRAQ4plex); K12(
qAsEQNWANYSAEQNR	P08050	N-Term(iTRAQ4plex); S3(Phospho)
eGEsDGDYPER	D4A997	N-Term(iTRAQ4plex); S4(Phospho)
eSILFTVIQPYPsPk	D4A3K7	N-Term(iTRAQ4plex); S13(Phospho); K15(iT
sSEAAANPcLISHSVSSDF	A0A0G2K5B0	N-Term(iTRAQ4plex); C9(Carbamidomethyl);
aEEPSsPPVPk	Q68FQ3	N-Term(iTRAQ4plex); S6(Phospho); K11(iTR
aELPPPYTAIAsPGTSGIP	Q4V888	N-Term(iTRAQ4plex); S12(Phospho); C23(Ca
sPSEAksPAGAk	F1LRZ7	N-Term(iTRAQ4plex); K6(iTRAQ4plex); S7(P
lMHDAsDSEVDQDDVVEV	Q9JKL8	N-Term(iTRAQ4plex); S6(Phospho); K19(iTR
tLsQsSESGTLPSGPPGHT	A0A0G2JSU4	N-Term(iTRAQ4plex); S3(Phospho); S5(Phos
eAGHGPGkDEMtDEELAE	A0A0G2K9Q6	N-Term(iTRAQ4plex); K8(iTRAQ4plex); T12(
dkEVsDDEAEEk	P82995	N-Term(iTRAQ4plex); K2(iTRAQ4plex); S5(P
gLsLPDIk	Q5BKB9	N-Term(iTRAQ4plex); S3(Phospho); K8(iTRA
qGFSDkEsPVSDLTSDLY(	P15205;F1LRL9	N-Term(iTRAQ4plex); K6(iTRAQ4plex); S8(P
aENYDIsPADR	Q5U300	N-Term(iTRAQ4plex); S7(Phospho)
tVPLFPHPSVPSLSPTESk	A0A0G2K6Z8	N-Term(iTRAQ4plex); K19(iTRAQ4plex); S25
vIsGSPPDSVGDTGAEVT	G3V9G5	N-Term(iTRAQ4plex); S3(Phospho)
dTSATFQSVDGsPQAEQI	B2RYM6	N-Term(iTRAQ4plex); S12(Phospho); K25(iT
tLcVIDPENRDsLEETDIDI/	F1M1T9	N-Term(iTRAQ4plex); C3(Carbamidomethyl);
rAEVQALLQDYVsTQSAE	Q5XI97	N-Term(iTRAQ4plex); S13(Phospho)
eFITGDVEPTDAESAWHs	Q5U2Z3	N-Term(iTRAQ4plex); S18(Phospho); K25(iT
tASISsSPSEGTPAVGSYC	F1M062	N-Term(iTRAQ4plex); S6(Phospho); C20(Car
kIAELEEERsQGsTSNSDV	F1LRL9	N-Term(iTRAQ4plex); K1(iTRAQ4plex); S10(
nLAsPEGTLATLGLk	F1M3W5	N-Term(iTRAQ4plex); S4(Phospho); K15(iTR
vTmILQsPSFR	F8WFS9	N-Term(iTRAQ4plex); M3(Oxidation); S7(Pho
gPSsQEDEsGGIEDSPDR	A0A0G2K0F3	N-Term(iTRAQ4plex); S4(Phospho); S9(Phos
tLVMAVYDFDRFsk	P21707	N-Term(iTRAQ4plex); S13(Phospho); K14(iT
qPGVQsPSR	P15205;F1LRL9	N-Term(iTRAQ4plex); S6(Phospho)
qQLSPsPGQEAGILPETEI	D3ZKL3	N-Term(iTRAQ4plex); S6(Phospho); K19(iTR
lYGSGGPPPTGEEDtSEkI	P06761	N-Term(iTRAQ4plex); T15(Phospho); K18(iT
vQNsPPVGR	X4YHC6	N-Term(iTRAQ4plex); S4(Phospho)
aSLGsDDGAQTk	M0R5U4	N-Term(iTRAQ4plex); S5(Phospho); K12(iTR
gTGPGGQLQDLDcssSDD	Q3KRC3	N-Term(iTRAQ4plex); C13(Carbamidomethyl
lAALkDEPQTVPDVPsFGI	P52909	N-Term(iTRAQ4plex); K5(iTRAQ4plex); S16(
sQsLQDQPTR	A0A0G2K382	N-Term(iTRAQ4plex); S3(Phospho)
iYQFPDcDsDEDEDFk	A0A096MJN4	N-Term(iTRAQ4plex); C7(Carbamidomethyl);
nRsSNVVPYDFNR	Q6LDZ3	N-Term(iTRAQ4plex); S3(Phospho)
sPAEVksPVEAk	F1LRZ7	N-Term(iTRAQ4plex); S1(Phospho); K6(iTRA
aGTLSITEFADMLSGNAG	A0A0G2K1J5	N-Term(iTRAQ4plex); S22(Phospho)
kDPQDMEGEksPAsPFAC	A0A0U1RRX4;F1MAQ5	N-Term(iTRAQ4plex); K1(iTRAQ4plex); K10(
sAPEQEQPATVsQEEDQ\	G3V874;A0A0G2K1Q9	N-Term(iTRAQ4plex); S12(Phospho); K30(iT
aTtPPNQGRPDsPVYANL	A0A0G2K1Z2	N-Term(iTRAQ4plex); T3(Phospho); S12(Pho
aQsPTPSLPASWk	D4A702	N-Term(iTRAQ4plex); S3(Phospho); K13(iTR
eDsmDmDmSPLRPQNYL	P13084	N-Term(iTRAQ4plex); S3(Phospho); M4(Oxid
eTSVSkEDtDQEEk	Q566D6	N-Term(iTRAQ4plex); K6(iTRAQ4plex); T9(P
rVsRsLDGAPIGVVDQSLn	A0A0G2K162	N-Term(iTRAQ4plex); S3(Phospho); S5(Phos
qIsQDVk	A0A0G2K3U1	N-Term(iTRAQ4plex); S3(Phospho); K7(iTRA
rPsPGEVSk	O35867	N-Term(iTRAQ4plex); S3(Phospho); K9(iTRA
lAEPEEEEPEAEGRQDED	F1M1U0	N-Term(iTRAQ4plex); S21(Phospho); M23(O
qSsmDSEGNFNPQPVDT	F1LRZ1	N-Term(iTRAQ4plex); S3(Phospho); M4(Oxid
tGSsPTQGIVNk	B0VXR4	N-Term(iTRAQ4plex); S4(Phospho); K12(iTR
kGsPELQTVHSWPGDR	D4A148	N-Term(iTRAQ4plex); K1(iTRAQ4plex); S3(P
dHLsPDFYDESETDPGAE	A0A0G2K4J8	N-Term(iTRAQ4plex); S4(Phospho)

eQLmNSSLGSGTAsLR	P31016	N-Term(iTRAQ4plex); M4(Oxidation); S14(Ph
sGGDtHsPPR	F1M5M9	N-Term(iTRAQ4plex); T5(Phospho); S7(Phos
lGIHEDsTNR	P34058	N-Term(iTRAQ4plex); S7(Phospho)
aTTDNsPSskAEDGPAkEI	P07936	N-Term(iTRAQ4plex); S6(Phospho); S9(Phos
rEsLTk	A0JPL9	N-Term(iTRAQ4plex); S3(Phospho); K6(iTRA
mESPIPILPLPTPDsETEk	D3ZT07	N-Term(iTRAQ4plex); S15(Phospho); K19(iT
dksPSEGDVAPPk	D3ZBU7	N-Term(iTRAQ4plex); K2(iTRAQ4plex); S3(P
rPsPPAAGk	D3ZF45	N-Term(iTRAQ4plex); S3(Phospho); K9(iTRA
sSSSsPTSPGSFR	A0A0G2JY17	N-Term(iTRAQ4plex); S5(Phospho)
sPAGAksPAEAksPVVAk	F1LRZ7	N-Term(iTRAQ4plex); K6(iTRAQ4plex); S7(P
mFGsNLDLLDPGQ	G3V8Q6	N-Term(iTRAQ4plex); S4(Phospho)
aAQPPTETAESsQAEEEk	P07936	N-Term(iTRAQ4plex); S12(Phospho); K18(iT
ssGQESESIPEYTAEEER	Q5BJR4	N-Term(iTRAQ4plex); S2(Phospho)
sDYMEIQEGVNNsNEDFR	P63142	N-Term(iTRAQ4plex); S13(Phospho); K23(iT
aMSMASILTNTMEELEEs	A0A0G2K207	N-Term(iTRAQ4plex); S18(Phospho)
sAsQAEEEPcLEPVk	A0A0G2JSZ0	N-Term(iTRAQ4plex); S3(Phospho); C10(Ca
yTVPLPsPVQDSENLSGE	A0A0U1RRX4;F1MAQ5	N-Term(iTRAQ4plex); S7(Phospho); K29(iTR
gDQEEELDRPVSsPGEAE	D3ZG78	N-Term(iTRAQ4plex); S13(Phospho); K20(iT
mHQVMsIEEVER	D3ZDR2	N-Term(iTRAQ4plex); S6(Phospho)
aGAPLGGsGFk	D4A9R4	N-Term(iTRAQ4plex); S8(Phospho); K11(iTR
sLsGSSPcPk	A0A0G2K2M9	S1(Phospho); N-Term(iTRAQ4plex); S3(Phos
sPGEAksPAEAksPAEVk	F1LRZ7	N-Term(iTRAQ4plex); S1(Phospho); K6(iTRA
lDsPTMSR	P26431	N-Term(iTRAQ4plex); S3(Phospho)
fTDQSLsPEDAESLSVLSV	P34926;Q63330	N-Term(iTRAQ4plex); S7(Phospho); K26(iTR
mDSASQDINLNsPNk	Q6PCT3	N-Term(Acetyl); S12(Phospho); K15(iTRAQ4
aAsLTEDR	M0RD03	N-Term(iTRAQ4plex); S3(Phospho)
iTFTPSsGISSEVTVPR	F1LQ63	N-Term(iTRAQ4plex); S7(Phospho)
aERHsPVFSGkPEk	A0A0G2K6R9;F1M9N9	N-Term(iTRAQ4plex); S5(Phospho); K11(iTR
aQGQIRsPTk	P52481	N-Term(iTRAQ4plex); S7(Phospho); K10(iTR
sVVsFDk	A0A0G2JW97	N-Term(iTRAQ4plex); S4(Phospho); K7(iTRA
sLINEISAsPPLPR	F1LSD3	N-Term(iTRAQ4plex); S9(Phospho)
lNLFSLDPDIDtLk	A0A0G2K939	N-Term(iTRAQ4plex); T12(Phospho); K14(iT
kAEsEELEIQkPQVk	Q9QXY2	K1(iTRAQ4plex); N-Term(iTRAQ4plex); S4(P
lPPGkPGVGDVSRPFSPP	F1M1Y0	N-Term(iTRAQ4plex); K5(iTRAQ4plex); S22(
rTLsIDkGF	P30835	N-Term(iTRAQ4plex); S4(Phospho); K7(iTRA
nVEDTDGssPEDLmAALT	Q6RJR6	N-Term(iTRAQ4plex); S8(Phospho); S9(Phos
kFsSPPPLAVSR	Q99JE4	N-Term(iTRAQ4plex); K1(iTRAQ4plex); S3(P
kQkGsEENLDETR	A0A0G2JSM7;D3ZZ99	N-Term(iTRAQ4plex); K1(iTRAQ4plex); K3(i
gDDDEEsDEEAVk	A0A0G2JTI7	N-Term(iTRAQ4plex); S7(Phospho); K13(iTR
kHSAILAsPNPDEk	P32851	N-Term(iTRAQ4plex); K1(iTRAQ4plex); S8(P
sHsLEGPSk	G3V849	N-Term(iTRAQ4plex); S3(Phospho); K9(iTRA
tEATQGLDYVPSAGTIsPT	P34926	N-Term(iTRAQ4plex); S17(Phospho); K26(iT
kPsPQPsPPR	A1L1L5	K1(iTRAQ4plex); N-Term(iTRAQ4plex); S3(P
fLEDDtSDPTYTSALGGk	F1MAJ0	N-Term(iTRAQ4plex); T6(Phospho); K18(iTR
rRtAsPPPPPk	B2RYB3;A0A0G2K4F6	N-Term(iTRAQ4plex); T3(Phospho); S5(Phos
ksAsEDSIR	A0A0G2K207	K1(iTRAQ4plex); N-Term(iTRAQ4plex); S2(P
aPTLAELDSSDsEEETLHI	D3ZEX7	N-Term(iTRAQ4plex); S12(Phospho); K19(iT
fPLmYEENsRENPFk	G3V7X2	N-Term(iTRAQ4plex); M4(Oxidation); S9(Pho
sRSLsESYELSSDLQDk	A0A0G2JUG7	N-Term(iTRAQ4plex); S5(Phospho); K17(iTR
aGDMLEDsPk	Q8VHK7	N-Term(iTRAQ4plex); S8(Phospho); K10(iTR
tEDGTANAGsDYEFTEGT	M0R3V7	N-Term(iTRAQ4plex); S10(Phospho); K22(iT
lPLSTLTAPPSPVNFSGsP	D3ZPT0	N-Term(iTRAQ4plex); S18(Phospho)
sPAEAksPAEVksPAVAk	F1LRZ7	N-Term(iTRAQ4plex); K6(iTRAQ4plex); S7(P
emLLEDVGsEEEPEEDDE	A0A0G2K7X3	N-Term(iTRAQ4plex); M2(Oxidation); S9(Pho
qEtVEcLR	P11730;F1M3F8	N-Term(iTRAQ4plex); T3(Phospho); C6(Carb

dtVDSAGtSPTAVLAAGED	A0A0G2K272	N-Term(iTRAQ4plex); T2(Phospho); T8(Phos
asSRPRPDDLEI	P08050	N-Term(iTRAQ4plex); S2(Phospho)
sEADtLPRsGEQER	D3ZEI4	N-Term(iTRAQ4plex); T5(Phospho); S9(Phos
sPDPEMVPQGsPVR	D3ZN76	N-Term(iTRAQ4plex); S11(Phospho)
sPcSLk	P34926;Q63330	N-Term(iTRAQ4plex); S1(Phospho); C3(Carb
gILAADEsVGSMAk	P09117	N-Term(iTRAQ4plex); S8(Phospho); K14(iTR
sNIsPNFNFMGQLLDFER	Q63340	N-Term(iTRAQ4plex); S4(Phospho)
nkPSVLEDsSFER	Q9Z2X9	N-Term(iTRAQ4plex); K2(iTRAQ4plex); S9(P
gSYMEVEDNRsQVETEDI	A0A0H2UHZ1	N-Term(iTRAQ4plex); S11(Phospho); K21(iT
sVDEVNYWDk	Q5EB89	N-Term(iTRAQ4plex); S1(Phospho); K10(iTR
qNsLEYMDQNDDRLk	D3ZEI4	N-Term(iTRAQ4plex); S3(Phospho); K15(iTR
qAsPSGTPTPEADTTLLk	A0JPL9	N-Term(iTRAQ4plex); S3(Phospho); K18(iTR
sMDELNHDFQALALEGR	D3Z8L5	N-Term(iTRAQ4plex); S1(Phospho)
dSVDSGSTTPNsPVSESA	A0A0H2UHW6	N-Term(iTRAQ4plex); S12(Phospho); C20(Ca
qLQIDDPDPVGNAALPDD	D4AB66	N-Term(iTRAQ4plex); S30(Phospho)
eQGEPVsPSEDESFSWP	F1LXQ7	N-Term(iTRAQ4plex); S7(Phospho); K20(iTR
gVTASSSsPPASVPk	A0A0G2K0M8	N-Term(iTRAQ4plex); S8(Phospho); K15(iTR
lTMGMLksPNTAILk	Q9QXY2	N-Term(iTRAQ4plex); K7(iTRAQ4plex); S8(P
kkDLsLEEIQk	P21818;A0A096MK73	K1(iTRAQ4plex); N-Term(iTRAQ4plex); K2(iT
iVEIPFNsTNk	P06685	N-Term(iTRAQ4plex); S8(Phospho); K11(iTR
vkEVHDELEDLPsPPPPLS	M0R423	N-Term(iTRAQ4plex); K2(iTRAQ4plex); S13(
gPAHLVtSL	D4AAL4	N-Term(iTRAQ4plex); T7(Phospho)
sVsPTVNPMSTAMQNER	F1LQC5	N-Term(iTRAQ4plex); S3(Phospho)
mHQVMsIEEVER	D3ZDR2	N-Term(iTRAQ4plex); M1(Oxidation); S6(Pho
vADVPVSEATTVLGDVHS	A0A0U1RRX4;F1MAQ5	N-Term(iTRAQ4plex); S30(Phospho); K34(iT
qSsTADAPELR	A0A0G2K9C8	N-Term(iTRAQ4plex); S3(Phospho)
eFGYDSPHDLDsD	Q8CIV0	N-Term(iTRAQ4plex); S12(Phospho)
sGPALPPEGtLARSPsIDS	F1M787	N-Term(iTRAQ4plex); T10(Phospho); S16(Ph
tDETGsPLSR	Q2VUH7	N-Term(iTRAQ4plex); S6(Phospho)
aEAESDGQtEETADPQNA	D4A554	N-Term(iTRAQ4plex); T9(Phospho)
sPGEAksPAEAksPAEVk	F1LRZ7	N-Term(iTRAQ4plex); K6(iTRAQ4plex); S7(P
tVTAMDVVyALk	P62804	N-Term(iTRAQ4plex); Y9(Phospho); K12(iTR
eGSIGGsGGsGGGGGGG	D3ZCL8	N-Term(iTRAQ4plex); S7(Phospho); S10(Pho
sAsSDTSEELNAQDsPkR	Q9JJ19	N-Term(iTRAQ4plex); S3(Phospho); S15(Pho
gSAFTYPsQQSPR	F1LPA2	N-Term(iTRAQ4plex); S8(Phospho)
rEsRQEsDPEDDDVk	D3ZAY8	N-Term(iTRAQ4plex); S3(Phospho); S7(Phos
eESSDNEHsVEHDVTILLE	A0A0G2JXZ3	N-Term(iTRAQ4plex); S9(Phospho)
smsDPkPLsPTADESSR	Q9JKS6;D3Z9C7	N-Term(iTRAQ4plex); M2(Oxidation); S3(Pho
qsPTHEMNPRGAQEGHIY	Q56A26	N-Term(iTRAQ4plex); S2(Phospho); K20(iTR
ikPTGLLTVPsPQI	D3ZA89	N-Term(iTRAQ4plex); K2(iTRAQ4plex); S11(
kksPSEGTDEk	D4A404	K1(iTRAQ4plex); N-Term(iTRAQ4plex); K2(iT
hSAPsPPVSVGsEEHPHS	D3ZYS1	N-Term(iTRAQ4plex); S5(Phospho); S12(Pho
aIGGIILtASHNPGGPNGD	P38652	N-Term(iTRAQ4plex); T8(Phospho); K23(iTR
tPALSPQRPLTTQQPQSG	G3V733	N-Term(iTRAQ4plex); T1(Phospho); K21(iTR
aIQDSLEVGsQkR	Q6IE65	N-Term(iTRAQ4plex); S10(Phospho); K12(iT
sQsLTNAFNLPEPAPPRP	P09951	N-Term(iTRAQ4plex); S3(Phospho); S21(Pho
tASPSTSsIDSTTDSR	D4A346	N-Term(iTRAQ4plex); S8(Phospho)
aHGGHPGsPR	D3ZFB6	N-Term(iTRAQ4plex); S8(Phospho)
eVVPDsPGDk	P34926;Q63330	N-Term(iTRAQ4plex); S6(Phospho); K10(iTR
sTIEEITTSSsQkM	P23565	N-Term(iTRAQ4plex); S11(Phospho); K13(iT
vGGWSSIYSVtIP	B0BNF1	N-Term(iTRAQ4plex); T11(Phospho)
vNGDAsPAAAEPGAk	F1LMW7	N-Term(iTRAQ4plex); S6(Phospho); K15(iTR
aTQQsLGEQSNPR	G3V927	N-Term(iTRAQ4plex); S5(Phospho)
vEPPHsSHEDLTDDLSTR	F1LUG5	N-Term(iTRAQ4plex); S6(Phospho)
yQLsIHEREDSPQSHVLVI	P06686	N-Term(iTRAQ4plex); S4(Phospho); K20(iTR

Information Classification: General

nsPLDcGSASPNk	D4A0B4	N-Term(iTRAQ4plex); S2(Phospho); C6(Carb
qLQSEQPQTAAARs	E9PTI1	N-Term(iTRAQ4plex); S14(Phospho)
sLNPALDGLTcGLTSHDk	G3V9M3	N-Term(iTRAQ4plex); S1(Phospho); C11(Car
sAGFIPIkEDFsPEk	F1LRL9	N-Term(iTRAQ4plex); K8(iTRAQ4plex); S12(
vEFSTPFQVEEVDDVSPs	G3V9G5	N-Term(iTRAQ4plex); S18(Phospho); K20(iT
tcFsPNR	D4AC36	N-Term(iTRAQ4plex); C2(Carbamidomethyl);
eSsPVPsPTSDR	G3V6S0	N-Term(iTRAQ4plex); S3(Phospho); S7(Phos
gPsPAScIDQLPIk	Q505J5	N-Term(iTRAQ4plex); S3(Phospho); C7(Carb
nLTSSsLNDISDkPEkDQL	Q9JI66;A0A0H2UHB7	N-Term(iTRAQ4plex); S6(Phospho); K13(iTR
qGAIYHGLATLLNQPsPM	F1LT10	N-Term(iTRAQ4plex); S16(Phospho)
gSLAsLDsLRk	D3ZZZ9	N-Term(iTRAQ4plex); S5(Phospho); S8(Phos
sPQAQDTPVSIAGGQTG	Q63330	N-Term(iTRAQ4plex); S1(Phospho); C18(Car
kNVTESPsFSAGDNPHVL	G3V9B3	N-Term(iTRAQ4plex); K1(iTRAQ4plex); S8(P
sPAEAksPASVksPGEAk	F1LRZ7	N-Term(iTRAQ4plex); S1(Phospho); K6(iTRA
sHsAGEVGEDSR	D4ADD3	N-Term(iTRAQ4plex); S3(Phospho)
lPAYLAtSPIPTEDQFFk	D3ZYB4	N-Term(iTRAQ4plex); T7(Phospho); K18(iTR
sPVVAk	F1LRZ7	N-Term(iTRAQ4plex); S1(Phospho); K6(iTRA
lSYAGGRPPSYAGsPVHH	Q9QXY2	N-Term(iTRAQ4plex); S14(Phospho)
cGSGPVHISGQHLVAVEE	P13084;Q7TP95	N-Term(iTRAQ4plex); C1(Carbamidomethyl);
kFQFPGEIDsSETELAEM	O88397	N-Term(iTRAQ4plex); K1(iTRAQ4plex); S10(
kLsGDQITLPTTVDYSSVF	Q5PQP2	N-Term(iTRAQ4plex); K1(iTRAQ4plex); S3(P
lTPSPDIIVLSDNEAsSPR	Q4V8E1	N-Term(iTRAQ4plex); S16(Phospho)
sPVPsPPGSPR	D3ZVM5	N-Term(iTRAQ4plex); S5(Phospho)
iQAsFR	P07936	N-Term(iTRAQ4plex); S4(Phospho)
qSSsPYEDkDk	A0A0G2K2M9	N-Term(iTRAQ4plex); S4(Phospho); K9(iTRA
sGSsSPDSEITELk	B1WC02	N-Term(iTRAQ4plex); S4(Phospho); K14(iTR
mLPHAPGVQMQAIPEDA	Q4QQW4	N-Term(iTRAQ4plex); M1(Oxidation); S22(Ph
vEsTsGSEcENHTk	M0R9R0	N-Term(iTRAQ4plex); S3(Phospho); S5(Phos
gQEEQsPGLEDkDLDFk	Q64548	N-Term(iTRAQ4plex); S6(Phospho); K12(iTR
eGPGGIVQGPPMLEAsPF	B1WC33	N-Term(iTRAQ4plex); S16(Phospho)
gAEDAPDADTAIINAEGsC	A0A0G2K5U6	N-Term(iTRAQ4plex); S18(Phospho); K25(iT
nAPPAGDEPLAEtEsEsEA	A0A0G2K1U5	N-Term(iTRAQ4plex); T13(Phospho); S15(Ph
gSDsDSEGDNPEkk	Q793F9	N-Term(iTRAQ4plex); S4(Phospho); K13(iTR
rLsMYGVDLHHAk	A0A0G2K162	N-Term(iTRAQ4plex); S3(Phospho); K13(iTR
lkskEsLQEAGk	P53987	N-Term(iTRAQ4plex); K2(iTRAQ4plex); S3(P
nPSVIFsScGDLDLPEHQT	C0IXW5	N-Term(iTRAQ4plex); S7(Phospho); C9(Carb
sALSSsLR	A0A0G2JUG7	N-Term(iTRAQ4plex); S6(Phospho)
eTENEEQTQLTQNAVscP	G3V6L7	N-Term(iTRAQ4plex); S16(Phospho); C17(Ca
dWGSSsGSQGR	P31016	N-Term(iTRAQ4plex); S6(Phospho)
sLPSNGELDPDVLESmAS	B2DD29	N-Term(iTRAQ4plex); S1(Phospho); M16(Ox
aDLPVITIDPAsPQsPESVI	P26431	N-Term(iTRAQ4plex); S12(Phospho); S15(Ph
aSYDSVEDSSESENsPVV	Q9JKS6;D3Z9C7	N-Term(iTRAQ4plex); S15(Phospho)
lEAStSDPLPAGGGSVLPC	G3V8J5	N-Term(iTRAQ4plex); T5(Phospho)
qAsEDSNDLTPTLPETPVI	A0A0G2K451	N-Term(iTRAQ4plex); S3(Phospho)
iLFFStPk	Q3B8P5	N-Term(iTRAQ4plex); T6(Phospho); K8(iTRA
gmkDDkDEEDDGTGsPHI	P29066	N-Term(iTRAQ4plex); M2(Oxidation); K3(iTR
dSAPTtPtSPTEFLTPGLR	D4A1D3	N-Term(iTRAQ4plex); T6(Phospho); T8(Phos
mDSAVQAsLSLPAtPVGk	Q78PB6	N-Term(iTRAQ4plex); S8(Phospho); T14(Pho
sGAQASSTPLsPTR	G3V8L3	N-Term(iTRAQ4plex); S11(Phospho)
eELEQQtDGDcDEEDDDk	Q7TP42	N-Term(iTRAQ4plex); T7(Phospho); C11(Car
gRkDEDsDDESQSSHAGI	A0A0G2K266	N-Term(iTRAQ4plex); K3(iTRAQ4plex); S7(P
rTsPVQTR	F1LSQ0	N-Term(iTRAQ4plex); S3(Phospho)
eRNGNQELPGtRSDLGk	Q6AXP1	N-Term(iTRAQ4plex); T11(Phospho); K17(iT
aTQGLDYVPSAGTIsPTS	Q63330	N-Term(iTRAQ4plex); S15(Phospho); K24(iT
lGGSVLEQIPFLQNcEDEI	F1M6V8	N-Term(iTRAQ4plex); C15(Carbamidomethyl

Information Classification: General

kEsLSESR	Q9JM59	N-Term(iTRAQ4plex); K1(iTRAQ4plex); S3(P
lQDDEAVDIEkPLLPSQPA	F1M842	N-Term(iTRAQ4plex); K11(iTRAQ4plex); S21
lPYLVELsPGR	F1LT10	N-Term(iTRAQ4plex); S8(Phospho)
vQTELkEETQPLSHsPk	D3ZXY2	N-Term(iTRAQ4plex); K6(iTRAQ4plex); S15(
sFGNPFEGSR	O88831	N-Term(iTRAQ4plex); S1(Phospho)
mNQLLSRtPALsPQRPLT	G3V733	N-Term(iTRAQ4plex); T8(Phospho); S12(Pho
aTsPQkPk	Q63327	N-Term(iTRAQ4plex); S3(Phospho); K6(iTRA
kLGGLLPAGGENGSEATI	G3V9D1	N-Term(iTRAQ4plex); K1(iTRAQ4plex); S20(
dTTsPMELAALEk	F1LPG3	N-Term(iTRAQ4plex); S4(Phospho); K13(iTR
fsGWYDADLSPAGHEEAk	P25113	N-Term(iTRAQ4plex); S2(Phospho); K18(iTR
eSETFsDsSPIEIIDEFPTF	F1LQN3	N-Term(iTRAQ4plex); S6(Phospho); S8(Phos
lELVEsLDSDEVDLk	F1M031	N-Term(iTRAQ4plex); S6(Phospho); K15(iTR
aSALFsSDEEDQWSVADS	Q80X08	N-Term(iTRAQ4plex); S6(Phospho); K21(iTR
qGsPTQSPPADTSFGSR	A0A0G2KAV8	N-Term(iTRAQ4plex); S3(Phospho)
rAtSPQkPk	Q63327	N-Term(iTRAQ4plex); T3(Phospho); K7(iTRA
eAEGsPETEk	D4A1J3	N-Term(iTRAQ4plex); S5(Phospho); K10(iTR
tEATQGLDYVPSAGTIsPt	P34926	N-Term(iTRAQ4plex); S17(Phospho); T19(Ph
aIEQADLLQEEDEsPR	B5DFI3	N-Term(iTRAQ4plex); S14(Phospho)
vEEEQEADEEDVsEEETE	Q52KJ9	N-Term(iTRAQ4plex); S13(Phospho)
lkDLGHPVEEEDEsGDQE	P41542	N-Term(iTRAQ4plex); K2(iTRAQ4plex); S14(
aPTPGGGHPDsPGLPAP	O35430	N-Term(iTRAQ4plex); S11(Phospho)
dFQEDsWGETkHsPGVSI	P34926;Q63330	N-Term(iTRAQ4plex); S6(Phospho); K11(iTR
dQGGALGPAPGAsILSSS	F1M3A4	N-Term(iTRAQ4plex); S13(Phospho)
sPSADQSLDDSPSk	F1M6X3	N-Term(iTRAQ4plex); S1(Phospho); K14(iTR
rPVsGEYGPAAVPEcEPG	D3ZKX8	N-Term(iTRAQ4plex); S4(Phospho); C15(Ca
sLsAMEVk	P50475	N-Term(iTRAQ4plex); S3(Phospho); K8(iTRA
nmTVEQLLTGsPTSPTVE	F1M403	N-Term(iTRAQ4plex); M2(Oxidation); S11(Ph
gDSPVTADVSRGsPEcVG	D4A5X1	N-Term(iTRAQ4plex); S13(Phospho); C16(Ca
sPPDQSAVPNtPPStPVkL	D3ZZ99	N-Term(iTRAQ4plex); T11(Phospho); T15(Ph
mkGEtPVNSTMSIGQAR	G3V8K2	N-Term(iTRAQ4plex); K2(iTRAQ4plex); T5(P
cETsPPSSPR	F1M863	N-Term(iTRAQ4plex); C1(Carbamidomethyl);
sPPGGAHGsLk	O88917	N-Term(iTRAQ4plex); S9(Phospho); K11(iTR
gMMEALPDGLsVEDILNV	G3V7X2	N-Term(iTRAQ4plex); S11(Phospho); K28(iT
lEAVSHTSDMHcGYGDsP	Q08163	N-Term(iTRAQ4plex); C12(Carbamidomethyl
gFksPPcEDFSVTGESEk	P34926;Q63330	N-Term(iTRAQ4plex); K3(iTRAQ4plex); S4(P
qLDHESDDADREDDERs	F1M3W5	N-Term(iTRAQ4plex); S17(Phospho)
aELEEMEETHPsDEEGEE	P34926;Q63330	N-Term(iTRAQ4plex); S12(Phospho); K20(iT
rEsPsPAPkPR	B2RYB3;A0A0G2K4F6	N-Term(iTRAQ4plex); S3(Phospho); S5(Phos
kNDGVkESSEstNttIEDED	P11275	K1(iTRAQ4plex); N-Term(iTRAQ4plex); K6(iT
kPASVSPTTPPsPTEGEA	G3V7G0	N-Term(iTRAQ4plex); K1(iTRAQ4plex); S12(
akPSPAPsPTISAPDASGF	A0A0G2JY69	N-Term(iTRAQ4plex); K2(iTRAQ4plex); S8(P
rVEsEEsGDEEGk	Q5HZV9	N-Term(iTRAQ4plex); S4(Phospho); S7(Phos
aESEAETPGtPGEFESk	P63047	N-Term(Acetyl); T10(Phospho); K17(iTRAQ4
aEDEGSTEEGGkADEDks	M0RDJ7	N-Term(iTRAQ4plex); K12(iTRAQ4plex); K17
sDVIHAPLPsPVDk	B5DF63	N-Term(iTRAQ4plex); S10(Phospho); K14(iT
nkLEGDsDVDSELEDR	D3ZFD0	N-Term(iTRAQ4plex); K2(iTRAQ4plex); S7(P
tPQLALIFR	Q9JKB7	N-Term(iTRAQ4plex); T1(Phospho)
kPASVsPttPPSPTEGEAS	G3V7G0	K1(iTRAQ4plex); N-Term(iTRAQ4plex); S6(P
rsPtPPPR	B2RYB3;A0A0G2K4F6	N-Term(iTRAQ4plex); S2(Phospho); T4(Phos
eSVsPEDSEk	A0A0G2JVK8	N-Term(iTRAQ4plex); S4(Phospho); K10(iTR
eTSSPAsPQNLQSDTPAF	P34926	N-Term(iTRAQ4plex); S7(Phospho)
gDDDEEsDEEAVkk	A0A0G2JTI7	N-Term(iTRAQ4plex); S7(Phospho); K13(iTR
eQLMNSsLGsGTASLR	P31016	N-Term(iTRAQ4plex); S7(Phospho); S10(Pho
iIsAHGck	P06687	N-Term(iTRAQ4plex); S3(Phospho); C7(Carb
sNSQcDSHDEEIsPTPPNI	P81377	N-Term(iTRAQ4plex); C5(Carbamidomethyl);

lALLIHNDsLTSLIYGEQSE	D3ZNF5	N-Term(iTRAQ4plex); S9(Phospho); K21(iTR
kGVDLLLEGVQGESsPTR	A0A0G2KB60	N-Term(iTRAQ4plex); K1(iTRAQ4plex); S15(
fAMNPFYEPNsPIR	D3ZVF4	N-Term(iTRAQ4plex); S11(Phospho)
sPssEEVQGENAGR	F1M084	N-Term(iTRAQ4plex); S3(Phospho); S4(Phos
mLAEsDEsGDEESVSQTI	D4A9D8	N-Term(iTRAQ4plex); M1(Oxidation); S5(Pho
tIksPcDSGYSYETIEk	P15205;F1LRL9	N-Term(iTRAQ4plex); K3(iTRAQ4plex); S4(P
aEETASASGDGDPsGEEI	F1M3D2	N-Term(iTRAQ4plex); S14(Phospho); K36(iT
sATSSSPGsPIHSLETSL	P11506;P11505	N-Term(iTRAQ4plex); S9(Phospho)
dATAEQQEAGsDSENSR	D3ZKR9	N-Term(iTRAQ4plex); S11(Phospho)
lAATEETAIsPR	B2RYB2	N-Term(iTRAQ4plex); S10(Phospho)
dksPSEGDVAPPkR	D3ZBU7	N-Term(iTRAQ4plex); K2(iTRAQ4plex); S3(P
hLPsPPLTQEVDLHSIAGF	D4A3T0	N-Term(iTRAQ4plex); S4(Phospho)
sAFTPATATGSSPsPVLG	D3ZV52	N-Term(iTRAQ4plex); S14(Phospho); K22(iT
wTkPSSFsDSER	Q4KM57	N-Term(iTRAQ4plex); K3(iTRAQ4plex); S8(P
lAMQEFMILPVGAsSFR	M0R5J4	N-Term(iTRAQ4plex); S14(Phospho)
aIEQsIEQEEGLNR	P61265	N-Term(iTRAQ4plex); S5(Phospho)
mDTsPSk	Q5M7A3	N-Term(Acetyl); S4(Phospho); K7(iTRAQ4ple
vLTPTQVmNRPSsISWDG	P31044	N-Term(iTRAQ4plex); M8(Oxidation); S13(Ph
aTFAGGsRDEVITcQPGD	C0IXW5	N-Term(iTRAQ4plex); S7(Phospho); C14(Ca
iAVDLsDQEEETAGQNEN	Q5U4E4	N-Term(iTRAQ4plex); S6(Phospho)
sSDRPVALVTGANk	A0A0G2JSV2	N-Term(Acetyl); S1(Phospho); K14(iTRAQ4p
sSQPsPTAVPASDSPSAk	D4A644	N-Term(iTRAQ4plex); S5(Phospho); K18(iTR
lANHEEGsDEEEEETPkk	Q66HA5	N-Term(iTRAQ4plex); S8(Phospho); K17(iTR
rSDsLLSFR	A1A5P0	N-Term(iTRAQ4plex); S4(Phospho)
sRsPVDSPVPASmFAPEF	D2XV59	N-Term(iTRAQ4plex); S3(Phospho); M13(Ox
sPVEEVk	F1LRZ7	N-Term(iTRAQ4plex); S1(Phospho); K7(iTRA
ssSLkSsPSk	F1M8H6	N-Term(iTRAQ4plex); S2(Phospho); K5(iTRA
sLSsPTDNLELSAR	A0A0G2K527	N-Term(iTRAQ4plex); S1(Phospho); S4(Phos
tVLPFsQEFQR	A0A0G2K472	N-Term(iTRAQ4plex); S6(Phospho)
dAAFsSLSPPAVPATAcPI	Q6DG50	N-Term(iTRAQ4plex); S5(Phospho); C17(Ca
ekSPPDQSAVPNtPPSTPY	A0A0G2JSM7;D3ZZ99	N-Term(iTRAQ4plex); K2(iTRAQ4plex); T13(
aGQENIsVSkR	P06686	N-Term(iTRAQ4plex); S7(Phospho); K10(iTR
sNSLDDSTEEVQSmDVsF	D4A4L4	N-Term(iTRAQ4plex); M14(Oxidation); S17(P
gNksPsPPPDGsPAATPEI	Q5HZA7	N-Term(iTRAQ4plex); K3(iTRAQ4plex); S4(P
tTRTPEEGGYSYEISEk	P15205;F1LRL9	N-Term(iTRAQ4plex); T1(Phospho); K17(iTR
tLSsSSMDLSR	B2GV74	N-Term(iTRAQ4plex); S4(Phospho)
gGHGAINAPSAPDAsPPH	F1LSB5	N-Term(iTRAQ4plex); S15(Phospho)
eVIQQDsSPk	Q9JKS6	N-Term(iTRAQ4plex); S7(Phospho); K10(iTR
eHALLAyTLGVk	F1M6C2	N-Term(iTRAQ4plex); Y7(Phospho); K12(iTR
vEMGTSSQNDVDMSWIP	Q4V7D1	N-Term(iTRAQ4plex); K26(iTRAQ4plex); S28
rPDPDsDEDEDYERER	Q6AY02	N-Term(iTRAQ4plex); S6(Phospho)
dPQDMEGEksPAsPFAQT	A0A0U1RRX4;F1MAQ5	N-Term(iTRAQ4plex); K9(iTRAQ4plex); S10(
vAEIPFNsTNk	P06686;P06687	N-Term(iTRAQ4plex); S8(Phospho); K11(iTR
iGNEESDLEEAcVLPHsPI	D4ACN3	N-Term(iTRAQ4plex); C12(Carbamidomethyl
lGPPSsPSAADkEENPAVI	D4ADS9	N-Term(iTRAQ4plex); S6(Phospho); K12(iTR
mEDIDQSSLVSSSTDsPP	F1LQN3	N-Term(Acetyl); S16(Phospho); K25(iTRAQ4
lTVENsPkQETGISEGQGF	B0BNB2	N-Term(iTRAQ4plex); S6(Phospho); K8(iTRA
ssFLNAk	F1M386	N-Term(iTRAQ4plex); S2(Phospho); K7(iTRA
yREGsPLkEESLAR	A0A0G2K774	N-Term(iTRAQ4plex); S5(Phospho); K8(iTRA
kLGGDGsPTQVDVSQFG	P15205;F1LRL9	N-Term(iTRAQ4plex); K1(iTRAQ4plex); S7(P
tGAELDIkDVSDERLsPAk	P15205;F1LRL9	N-Term(iTRAQ4plex); K8(iTRAQ4plex); S16(
dGsPDAPATPEk	A0A0U1RRX4;F1MAQ5	N-Term(iTRAQ4plex); S3(Phospho); K12(iTR
eQEPLPQTLDcPEsPPk	A0A0G2K7H0	N-Term(iTRAQ4plex); C11(Carbamidomethyl
tQcPVFAEVGcSPcGTsDG	F1LZX5	N-Term(iTRAQ4plex); C3(Carbamidomethyl);
mHDsHLsSEEPk	P31596;Q8K5B5	N-Term(iTRAQ4plex); S4(Phospho); S7(Phos

aASEDMTsDEER	D4A853	N-Term(iTRAQ4plex); S8(Phospho)
fLENAAADFsDEDEDEDID	G3V6L8	N-Term(iTRAQ4plex); S10(Phospho); K23(iT
dSLESsPVEPk	A0A0G2K6R9;F1M9N9	N-Term(iTRAQ4plex); S6(Phospho); K11(iTR
rAsQsSLESSSGPPcIR	A0A0G2KAV8	N-Term(iTRAQ4plex); S3(Phospho); S5(Phos
yGTELNQGDMkPPSYDSI	G3V6L8	N-Term(iTRAQ4plex); K11(iTRAQ4plex); T23
sPSDYSNFDPEFLNEkPQ	D4A0U0	N-Term(iTRAQ4plex); K16(iTRAQ4plex); S20
gSEPEtPSEk	Q9QXY2	N-Term(iTRAQ4plex); T6(Phospho); K10(iTR
aSVcNLQIsPk	D3ZTF6	N-Term(iTRAQ4plex); C4(Carbamidomethyl);
mGPSSSPIPSPSPsPTDS	P63319	N-Term(iTRAQ4plex); M1(Oxidation); S14(Ph
tLEEQNQLLsAELGGLRA	M0R3Z1	N-Term(iTRAQ4plex); S10(Phospho); S20(Ph
vMDsDEDDDY	D4ADF5	N-Term(iTRAQ4plex); S4(Phospho)
kDLsLEEIQk	P21818;A0A096MK73	N-Term(iTRAQ4plex); K1(iTRAQ4plex); S4(P
lSLPGLLSQVsPR	P0C5Y8	N-Term(iTRAQ4plex); S11(Phospho)
sPVVAksPAEAk	F1LRZ7	N-Term(iTRAQ4plex); K6(iTRAQ4plex); S7(P
rAPPGPLPPAASPGPPAA	F1LRY7	N-Term(iTRAQ4plex); S19(Phospho); S26(Ph
kSAsEDSIR	A0A0G2K207	N-Term(iTRAQ4plex); K1(iTRAQ4plex); S4(P
iDsPGLk	F1M4A0	N-Term(iTRAQ4plex); S3(Phospho); K7(iTRA
tLNEAIEkSSGEALsVESk	D3Z980	N-Term(iTRAQ4plex); K8(iTRAQ4plex); S15(
iQAAAsPPANATAASDTN	A0A0G2JSH4	N-Term(iTRAQ4plex); S6(Phospho)
yGPVSVADTTGSGAADAI	B5DEN5	N-Term(iTRAQ4plex); K18(iTRAQ4plex); S28
hsRAIELIkNGGR	F1M7H7	N-Term(iTRAQ4plex); S2(Phospho); K9(iTRA
rQssTADAPELR	A0A0G2K9C8	N-Term(iTRAQ4plex); S3(Phospho); S4(Phos
mLAEsDEsGDEESVSQTI	D4A9D8	N-Term(iTRAQ4plex); M1(Oxidation); S5(Pho
sLDGAPIGVVDQSLMk	A0A0G2K162	N-Term(iTRAQ4plex); S1(Phospho); K16(iTR
sGPAGSSGGRGGAGGP	B0BNA9	N-Term(iTRAQ4plex); S20(Phospho)
gQPVLtPPDQLVIANIDQS	F1M2P8	N-Term(iTRAQ4plex); T6(Phospho)
eGPRPEQGPDIPGQQEE	F1LSB5	N-Term(iTRAQ4plex); S18(Phospho)
mEDSMDMDMsPLRPQN	P13084	N-Term(Acetyl); S10(Phospho); C21(Carbam
gPSPLVTMTPAVPAVTPV	M0RD40	N-Term(iTRAQ4plex); S23(Phospho)
sDFDSNPFADPDLNNPFk	A0A0G2K1I6	N-Term(Acetyl); S1(Phospho); K18(iTRAQ4p
lVLASIDQADFQGFTYVN	P63319	N-Term(iTRAQ4plex); S31(Phospho); M38(O
nLSQIENVHGYVLQSHIsF	Q63622	N-Term(iTRAQ4plex); S18(Phospho); K21(iT
msYHPGRGcPRGR	E9PTR3	N-Term(iTRAQ4plex); S2(Phospho); C9(Carb
tAFDEAIAELDtLSEESYkD	P63102	N-Term(iTRAQ4plex); T12(Phospho); K19(iT
aEVSSSIYFHkPREsPPLL	B4F7C1	N-Term(iTRAQ4plex); K11(iTRAQ4plex); S15
tGEPDEEEGTFRssIR	O08589	N-Term(iTRAQ4plex); S13(Phospho); S14(Ph
sPPRPAkPRsPPIPAkPR	Q63327	N-Term(iTRAQ4plex); S1(Phospho); K7(iTRA
vIstPPTPIFPISDDIGAIPIk	F1LMY3	N-Term(iTRAQ4plex); S3(Phospho); T4(Phos
sPEQVksPAk	F1LRZ7	N-Term(iTRAQ4plex); K6(iTRAQ4plex); S7(P
vGLVEDSPSTAGDGDDS	F1LPG3	N-Term(iTRAQ4plex); S28(Phospho)
kTSDDGGDsPEHDADIPE	D3ZJK3	N-Term(iTRAQ4plex); K1(iTRAQ4plex); S9(P
vPVLIsPTLk	F1M0N1	N-Term(iTRAQ4plex); S6(Phospho); K10(iTR
tsSkESSPVPSPTSDR	G3V6S0	N-Term(iTRAQ4plex); S2(Phospho); K4(iTRA
nFYDsDDEEkk	A0A0G2JWD6	N-Term(iTRAQ4plex); S5(Phospho); K10(iTR
kLEsPPPSTNPFLEDETA	B4F763	N-Term(iTRAQ4plex); K1(iTRAQ4plex); S4(P
gEAEQsEEEGEEEEDkAE	P15205;F1LRL9	N-Term(iTRAQ4plex); S6(Phospho); K16(iTR
aVEYFQDNSPDsPELNk	O54922	N-Term(iTRAQ4plex); S12(Phospho); K17(iT
eQkLsTDDLk	Q62861	N-Term(iTRAQ4plex); K3(iTRAQ4plex); S5(P
qksDAEEDGGtGsQDEED	P35565	N-Term(iTRAQ4plex); K2(iTRAQ4plex); S3(P
kVPSAsDSDSR	A0A0G2JYC7	N-Term(iTRAQ4plex); K1(iTRAQ4plex); S6(P
eLETPsEEk	G3V8G4	N-Term(iTRAQ4plex); S6(Phospho); K9(iTRA
vDNSVEAEVNSkssLEDE	M0R9R0	N-Term(iTRAQ4plex); K12(iTRAQ4plex); S13
gPDEAIEDGEEGsEDDAE	Q4V8H8	N-Term(iTRAQ4plex); S13(Phospho); K23(iT
kDSFSQESSPssPSDLAk	Q9JKS6;D3Z9C7	N-Term(iTRAQ4plex); K1(iTRAQ4plex); S11(
eLsAPAR	B2RZ79	N-Term(iTRAQ4plex); S3(Phospho)

Information Classification: General

lTGPSSVGPGPTDPDsDD	F1LPM7	N-Term(iTRAQ4plex); S16(Phospho)
qSNASsDVEAEEk	Q566D6	N-Term(iTRAQ4plex); S6(Phospho); K13(iTR
sPEPEATLTFPFLDk	F1M392	N-Term(iTRAQ4plex); S1(Phospho); K15(iTR
sPGYEPGsPR	D3ZGK0	N-Term(iTRAQ4plex); S8(Phospho)
tESVTSGPLSPEGsPskSF	F8WFS9	N-Term(iTRAQ4plex); S14(Phospho); S16(Ph
aPSQPPsPTEER	D3ZGD0	N-Term(iTRAQ4plex); S7(Phospho)
wQsPVTk	A0A0G2K4F6	N-Term(iTRAQ4plex); S3(Phospho); K7(iTRA
amRTLSSPsLQTDGLmAs	F1LVA9	N-Term(iTRAQ4plex); M2(Oxidation); S9(Pho
aQNPAYFEGkPAsLDEGA	A0A0G2JZX5	N-Term(iTRAQ4plex); K10(iTRAQ4plex); S13
lEGDsDVDSELEDR	D3ZFD0	N-Term(iTRAQ4plex); S5(Phospho)
aESSAATQSPSVSSSSS(	Q6RJR6	N-Term(Acetyl); S30(Phospho); C34(Carbam
rLscDGLGPSDLLGkPLAR	A0A0G2K876	N-Term(iTRAQ4plex); S3(Phospho); C4(Carb
lPDSTDkEVVDTLsNGsET	D3ZE49	N-Term(iTRAQ4plex); K7(iTRAQ4plex); S14(
mDsDSGEQSEGEPGTAA	F1LX20	N-Term(Acetyl); M1(Oxidation); S3(Phospho)
vETEkQsPPHGEAk	D4A631	N-Term(iTRAQ4plex); K5(iTRAQ4plex); S7(P
mNEDGLtPEQR	P62762	N-Term(iTRAQ4plex); T7(Phospho)
gPSQVSsPSQPPQk	D3ZGN7	N-Term(iTRAQ4plex); S7(Phospho); K14(iTR
kEPAISsQNSPEAR	Q4KLL0	N-Term(iTRAQ4plex); K1(iTRAQ4plex); S7(P
dLTNNNIPHLLsPk	E2E1S0	N-Term(iTRAQ4plex); S12(Phospho); K14(iT
lSQGsGSSITAAGMR	D3ZCL8	N-Term(iTRAQ4plex); S5(Phospho)
aSGQSFEVILksPSDLSPE	A0A0G2K8P5	N-Term(iTRAQ4plex); K11(iTRAQ4plex); S12
aEIHTQPsDkER	Q924W2	N-Term(iTRAQ4plex); S8(Phospho); K10(iTR
kEEQAASPVAEDVGDVA\	D3ZYB4	N-Term(iTRAQ4plex); K1(iTRAQ4plex); S19(
nEDAsFPSTPEPVk	F1LQN3	N-Term(iTRAQ4plex); S5(Phospho); K14(iTR
rNsLPQIPTLNLESR	P70600	N-Term(iTRAQ4plex); S3(Phospho)
sRsAPPNLWAAQR	O35147	N-Term(iTRAQ4plex); S3(Phospho)
aGsPQLDDIR	Q64350	N-Term(iTRAQ4plex); S3(Phospho)
lTFDSSFsPNTGkk	Q9Z2L0	N-Term(iTRAQ4plex); S8(Phospho); K13(iTR
aVGSMPTAGSAGsVPEN	A0A0G2K9N0	N-Term(iTRAQ4plex); S13(Phospho); K27(iT
amGDEDsDESGGGSPkP	G3V927	N-Term(iTRAQ4plex); M2(Oxidation); S7(Pho
dSsPTLASk	F1LS65	N-Term(iTRAQ4plex); S3(Phospho); K9(iTRA
qLEQQSAVcEGEGQVTsT	G3V7I8	N-Term(iTRAQ4plex); C9(Carbamidomethyl);
sTGDSQNLGSSsPGk	M0R4R4	N-Term(iTRAQ4plex); S12(Phospho); K15(iT
aAHLPLLtIEPPsDSSVDLS	A0A0G2JZX5	N-Term(iTRAQ4plex); T8(Phospho); S13(Pho
vPSPGsSEDDLQEEEQLE	G3V7X2	N-Term(iTRAQ4plex); S6(Phospho); K22(iTR
gSHSQTPsPGALPLGR	P09951	N-Term(iTRAQ4plex); S8(Phospho)
aGGsGGALDEAR	A0A0G2JX30	N-Term(iTRAQ4plex); S4(Phospho)
gLcSGPGAGEEsPAATLP	Q5XIU9	N-Term(iTRAQ4plex); C3(Carbamidomethyl);
tLVLSSsPtSPTQEPLAAG	A0A0G2K1B4	N-Term(iTRAQ4plex); S7(Phospho); T9(Phos
skSAPtsPcDQELk	F1LMV8	N-Term(iTRAQ4plex); K2(iTRAQ4plex); T6(P
sAVGFsNGSLEQR	D4A3F5	N-Term(iTRAQ4plex); S6(Phospho)
dSYVGDEAQskR	P68035;P60711;P63259	N-Term(iTRAQ4plex); S10(Phospho); K11(iT
sLsDPkPLsPTAEESAk	G3V984	N-Term(iTRAQ4plex); S3(Phospho); K6(iTRA
aAAELLQSQGSQAGGsQ	B5DFG5	N-Term(iTRAQ4plex); S16(Phospho); K20(iT
tEARSsDEENGPPsSPDLI	D3ZH75	N-Term(iTRAQ4plex); S6(Phospho); S14(Pho
rPEAVQNTsADPSEDQkD	A0A0G2K876	N-Term(iTRAQ4plex); S9(Phospho); K17(iTR
tDHGAEIVYksPVVSGDTs	D4A1Q2	N-Term(iTRAQ4plex); K10(iTRAQ4plex); S11
ksSVEGLEPAENk	B2RYW7	N-Term(iTRAQ4plex); K1(iTRAQ4plex); S2(P
gMIIEHEGDRPAsk	M0R3V7	N-Term(iTRAQ4plex); S13(Phospho); K14(iT
aNsQDSLASR	A0A0H2UHQ3	N-Term(iTRAQ4plex); S3(Phospho)
aSQGSLsPQEEVMEADLL	F1M842	N-Term(iTRAQ4plex); S7(Phospho); K30(iTR
cHAEHtPEEEIDHTGAk	B4F772	N-Term(iTRAQ4plex); C1(Carbamidomethyl);
nSTLsEEDYIER	Q9ET45	N-Term(iTRAQ4plex); S5(Phospho)
rDTSSNDINGGVEPTPSN	D3ZUY8	N-Term(iTRAQ4plex); T21(Phospho); S23(Ph
gIMAPPEIDksPSEGDVAF	D3ZBU7	N-Term(iTRAQ4plex); K10(iTRAQ4plex); S11

dDISEIQSLAsDHSVR	F1M4A0	N-Term(iTRAQ4plex); S11(Phospho)
nYkSsPEIQEPIkPLEk	D3ZJ01	N-Term(iTRAQ4plex); K3(iTRAQ4plex); S5(P
tAkDsDDDDDVTVTVDR	P32851	N-Term(iTRAQ4plex); K3(iTRAQ4plex); S5(P
nAETVksPEkEGVPGVEk	B5DFK6	N-Term(iTRAQ4plex); K6(iTRAQ4plex); S7(P
gRAsPAEGGcSIR	Q6P6S0	N-Term(iTRAQ4plex); S4(Phospho); C10(Ca
sPSTTLPk	A0A0G2JW88	N-Term(iTRAQ4plex); S1(Phospho); K8(iTRA
eSVPEFPLsPPk	A0A096MK73	N-Term(iTRAQ4plex); S9(Phospho); K12(iTR
tLNIFPEVMLGPGLEEASs	D3ZH78	N-Term(iTRAQ4plex); S19(Phospho)
sPPDQSAVPNtPPsTPVkL	A0A0G2JSM7	N-Term(iTRAQ4plex); T11(Phospho); S14(Ph
yWFTMER	O54715	N-Term(iTRAQ4plex); Y1(Phospho)
vEGsPNQAk	F1M853	N-Term(iTRAQ4plex); S4(Phospho); K9(iTRA
aSIANSDGPPAGSQtPPFI	A0A0G2K8Q4	N-Term(iTRAQ4plex); T15(Phospho); K19(iT
eTEsPEPTEk	Q5BJN8	N-Term(iTRAQ4plex); S4(Phospho); K10(iTR
kVAAGHELQPLAIVDQRP	P08050	N-Term(iTRAQ4plex); K1(iTRAQ4plex); S20(
sGAGsPPGTR	A0A0G2K2M9	N-Term(iTRAQ4plex); S5(Phospho)
aVAGVmITAsHNR	D3Z955	N-Term(iTRAQ4plex); M6(Oxidation); S10(Ph
iRsGYFDER	Q5BJT9	N-Term(iTRAQ4plex); S3(Phospho)
sEVQAHsPSRk	F1MAQ5;A0A0U1RRX4	N-Term(iTRAQ4plex); S7(Phospho); K11(iTR
sVDISLGGsPR	A0A0G2K6Z8	N-Term(iTRAQ4plex); S9(Phospho)
sDDskssSPEPVTHLk	D4A6C5	N-Term(iTRAQ4plex); S4(Phospho); K5(iTRA
lPSISDLDSIFGPVLsPk	F1M1Y0	N-Term(iTRAQ4plex); S16(Phospho); K18(iT
gVVVAtGDR	P06687	N-Term(iTRAQ4plex); T6(Phospho)
lIAAEGDAsPGEDR	Q9JKA7	N-Term(iTRAQ4plex); S9(Phospho)
gDQALsFLk	Q05962	N-Term(Acetyl); S6(Phospho); K9(iTRAQ4ple
kEETPGtPk	Q7TP42	N-Term(iTRAQ4plex); K1(iTRAQ4plex); T7(P
kYsScSTIFLDDSTVSQPN	F1MA89	N-Term(iTRAQ4plex); K1(iTRAQ4plex); S3(P
sILkPSTPVPMPESEEVGE	Q3ZB99	N-Term(iTRAQ4plex); K4(iTRAQ4plex); T21(
skEPSSPGTDDVFTPGPS	P26431	N-Term(iTRAQ4plex); K2(iTRAQ4plex); S20(
dcVGsEVENAcANPAAGT	P16617	N-Term(iTRAQ4plex); C2(Carbamidomethyl);
tSEsSEEVPEEEPEkR	D3ZKX8	N-Term(iTRAQ4plex); S4(Phospho); K15(iTR
akPAmPQDSVPsPR	A0A0G2K5E7	N-Term(iTRAQ4plex); K2(iTRAQ4plex); M5(
gVVTNGLDVsPAEEkk	P12839	N-Term(iTRAQ4plex); S10(Phospho); K15(iT
fNGEDVDtISPTLGFNIk	G3V8V0	N-Term(iTRAQ4plex); T8(Phospho); K18(iTR
gADDAADADTAIINAEGG	A0A0G2JUT1	N-Term(iTRAQ4plex); S22(Phospho); K25(iT
sPAEAksPAEAksPVEVk	F1LRZ7	N-Term(iTRAQ4plex); K6(iTRAQ4plex); S7(P
sPPPSPTTQR	Q08877	N-Term(iTRAQ4plex); S1(Phospho)
kDSGsSSVFAESPGGk	Q9QXY2	N-Term(iTRAQ4plex); K1(iTRAQ4plex); S5(P
lPsPTAAPQQSASQATPM	P09951	N-Term(iTRAQ4plex); S3(Phospho)
aLsFDNR	F1LMV8	N-Term(iTRAQ4plex); S3(Phospho)
dGkYDLDFksPADPSR	P07323	N-Term(iTRAQ4plex); K3(iTRAQ4plex); K9(i
aLPTAQAGALLLALPPAsF	M0R686	N-Term(iTRAQ4plex); S18(Phospho)
yHGHsMSDPGVSYR	F7FKI5	N-Term(iTRAQ4plex); S5(Phospho)
eVsGHSPQHSk	Q9EQL9	N-Term(iTRAQ4plex); S3(Phospho); K11(iTR
gTGQsDDSDIWDDTALIk	Q2KP10	N-Term(iTRAQ4plex); S5(Phospho); K18(iTR
qSsSPYEDkDkk	A0A0G2K2M9	N-Term(iTRAQ4plex); S3(Phospho); K9(iTRA
dLWPMVSPEDTQSLSFS	P34926;Q63330	N-Term(iTRAQ4plex); S20(Phospho); K23(iT
eEEEQETQEkGsGQEEEI	P12839	N-Term(iTRAQ4plex); K10(iTRAQ4plex); S12
fSEMMDHmGGDEDADLF	Q5PQL9	N-Term(iTRAQ4plex); M8(Oxidation); S26(Ph
sTPLPGPPGsPR	B2DD29	N-Term(iTRAQ4plex); S10(Phospho)
aVsEVVRPASTPPIMASAI	Q9QXY2	N-Term(iTRAQ4plex); S3(Phospho); K20(iTR
sSISPmDEPVPDSEsPIEk	P15205;F1LRL9	N-Term(iTRAQ4plex); M6(Oxidation); S15(Ph
sGYssPGsPGTPGSR	D4A1Q2	N-Term(iTRAQ4plex); S4(Phospho); S5(Phos
rNsLTGEEGELAk	Q99P55	N-Term(iTRAQ4plex); S3(Phospho); K13(iTR
tPTVPDLPGVLPsPPDGPI	A0A0G2K2D5	N-Term(iTRAQ4plex); S13(Phospho)
nVNENFGPNTEMHLVPIL	Q4V8H5	N-Term(iTRAQ4plex); K28(iTRAQ4plex); T30

Information Classification: General

tALALAITDsELSDEEASIL	A0A0G2K1U5	N-Term(iTRAQ4plex); S10(Phospho)
iRsFPDFPIPGVLFR	P36972	N-Term(iTRAQ4plex); S3(Phospho)
aIsLEGEPR	Q63622	N-Term(iTRAQ4plex); S3(Phospho)
eSLLsPPAVPDPGVMTPG	D4A1G8	N-Term(iTRAQ4plex); S5(Phospho)
yAYFNGcssPTAPLSPMSI	P08050	N-Term(iTRAQ4plex); C7(Carbamidomethyl);
qAEDTISNASSQLSsPPtS	F1M386	N-Term(iTRAQ4plex); S15(Phospho); T18(Ph
hYsPVEcDk	Q63622	N-Term(iTRAQ4plex); S3(Phospho); C7(Carb
mPFQAsPGGk	A0A0G2JWK2	N-Term(iTRAQ4plex); S6(Phospho); K10(iTR
tDQTPctPEDDLEEGMAk	A0A0G2K400	N-Term(iTRAQ4plex); C6(Carbamidomethyl);
dIsNLEENDETR	P31662	N-Term(iTRAQ4plex); S3(Phospho)
tSISDPPEsPPLLPPR	D4A3T0	N-Term(iTRAQ4plex); S9(Phospho)
gADYTEPDsPREPPGGAI	A0A0G2JXK1	N-Term(iTRAQ4plex); S9(Phospho)
tTPPPSAPTAR	F1LXQ7	N-Term(iTRAQ4plex); T1(Phospho)
tSEDTSSGsPPk	B1WC49	N-Term(iTRAQ4plex); S9(Phospho); K12(iTR
tREQESsGEEDNDLSPEE	A0A0H2UHA0	N-Term(iTRAQ4plex); S7(Phospho)
vALAAGsPTRPPPAR	D3ZTF0	N-Term(iTRAQ4plex); S7(Phospho)
aQNSTDsLAEEEEAEAAA	A0A0G2K0E5	N-Term(iTRAQ4plex); S7(Phospho)
tQLWASEPGtPPAPTSLP	M0R7E6	N-Term(iTRAQ4plex); T10(Phospho); K25(iT
tTASTQMNVQSsR	F1M5N7;D3ZCG2	N-Term(iTRAQ4plex); S12(Phospho)
aSsLNENVDHSALLk	B1H262	N-Term(iTRAQ4plex); S3(Phospho); K15(iTR
lGGDGsPTQVDVSQFGSI	P15205;F1LRL9	N-Term(iTRAQ4plex); S6(Phospho); K19(iTR
vTIEsSPDLEk	F1MA82	N-Term(iTRAQ4plex); S5(Phospho); K11(iTR
aALIPAGLSDGQFYSPPE	Q5U205	N-Term(iTRAQ4plex); S23(Phospho); K30(iT
aGsDTELAAPk	P12785	N-Term(iTRAQ4plex); S3(Phospho); K11(iTR
qVsTENESTLVHR	D4A2D3	N-Term(iTRAQ4plex); S3(Phospho)
rEsQTALVVNQR	Q569C0	N-Term(iTRAQ4plex); S3(Phospho)
eAQELGsPEDR	A0A1B0GWY5	N-Term(iTRAQ4plex); S7(Phospho)
mGPSSSPIPSPsPsPTDS	P63319	N-Term(iTRAQ4plex); S12(Phospho); S14(Ph
rRDsDGVDGFEAEGk	B1WC00	N-Term(iTRAQ4plex); S4(Phospho); K15(iTR
sGsPmAR	P02688	N-Term(iTRAQ4plex); S3(Phospho); M5(Oxid
aLGSLGGsPSLPDQDk	Q5QD51	N-Term(iTRAQ4plex); S8(Phospho); K16(iTR
kGVDLLLEGVQGEsSPTR	A0A0G2KB60	N-Term(iTRAQ4plex); K1(iTRAQ4plex); S14(
mGPSSSPIPSPsPsPTDS	P63319	N-Term(iTRAQ4plex); S12(Phospho); S14(Ph
gYtSDDDTWEPEVHLEDc	G3V8T1	N-Term(iTRAQ4plex); T3(Phospho); C18(Car
mAsPPPSGPPSAAHTPFI	P34926;Q63330	N-Term(iTRAQ4plex); M1(Oxidation); S3(Pho
rksPENAEGk	M0R5I3	N-Term(iTRAQ4plex); K2(iTRAQ4plex); S3(P
kAGsDGDIMDSSTETPPI	D3ZC15	N-Term(iTRAQ4plex); K1(iTRAQ4plex); S4(P
eLsPDFYQPGPDYIk	D3ZQ55	N-Term(iTRAQ4plex); S3(Phospho); K15(iTR
eTDGEVSVPGWDGQDQ	F1LZD2	N-Term(iTRAQ4plex); T19(Phospho); K27(iT
kGDVEGSQsQDEGEGSG	P97690	N-Term(iTRAQ4plex); K1(iTRAQ4plex); S9(P
vNNSsLIGLGYTQTLkPGI	Q9Z2L0	N-Term(iTRAQ4plex); S5(Phospho); K16(iTR
eTAFEFLSsA	O88989	N-Term(iTRAQ4plex); S9(Phospho)
qQQLQDEFLEHEAHLLSC	D4A1H7	N-Term(iTRAQ4plex); S20(Phospho)
skTPPSNLSPIEDAsPTEE	G3V984	N-Term(iTRAQ4plex); S1(Phospho); K2(iTRA
rPDPDsDEDEDYER	Q6AY02	N-Term(iTRAQ4plex); S6(Phospho)
eAFLNTEDkGDSLDsVEA	P16086	N-Term(iTRAQ4plex); K9(iTRAQ4plex); S15(
dYNASAStISPPSSmEEDI	P15205;F1LRL9	N-Term(iTRAQ4plex); T8(Phospho); M15(Ox
ssGATPVSGPPPPAVSST	Q9QXY2	N-Term(iTRAQ4plex); S2(Phospho)
dELTEsPk	Q6AXS5	N-Term(iTRAQ4plex); S6(Phospho); K8(iTRA
tESVTSGPLsPEGsPskSP	F8WFS9	N-Term(iTRAQ4plex); S10(Phospho); S14(Ph
tEVLLDQPEIWGSGQPINI	F1M1Y0	N-Term(iTRAQ4plex); M21(Oxidation); S23(P
sPtsPVPVPVM	P63319	N-Term(iTRAQ4plex); T3(Phospho); S4(Phos
eVVDsTTSSL	P31421	N-Term(iTRAQ4plex); S5(Phospho)
sVHQIcSGQVVLsLsTAVk	B1H246	N-Term(iTRAQ4plex); C6(Carbamidomethyl);
kEGDGsATTDAAPAtSPk	P07936	N-Term(iTRAQ4plex); K1(iTRAQ4plex); S6(P

Information Classification: General

vGAHGsEEAVVFR	D3ZH41	N-Term(iTRAQ4plex); S6(Phospho)
gPTsPTTPR	Q6AY81	N-Term(iTRAQ4plex); S4(Phospho)
sSSFSEEkGEsDDEkPR	E9PST5	N-Term(iTRAQ4plex); K8(iTRAQ4plex); S11(
mEsPTkEIEEFESNSLk	A0A0G2KAI1	M1(Oxidation); N-Term(Acetyl); S3(Phospho)
lsRSDEQLSSLDR	Q9QYV3	N-Term(iTRAQ4plex); S2(Phospho)
eLAGVLPQVHGsWESLNI	A1A5P0	N-Term(iTRAQ4plex); S12(Phospho)
tRtPDEVLEAEAEPk	D4A554	N-Term(iTRAQ4plex); T3(Phospho); K15(iTR
sAsTESGFHNHTDTAEGD	O35430	N-Term(iTRAQ4plex); S3(Phospho)
eFITGDVEPTDAESAWHs	Q5U2Z3	N-Term(iTRAQ4plex); S18(Phospho); K25(iT
sFSISPsR	E9PTE1	N-Term(iTRAQ4plex); S7(Phospho)
aASGWTPEGPDEEDMsD	D3Z9I3	N-Term(iTRAQ4plex); S16(Phospho)
eREDEsEDESDILEESPcC	A0A0G2JT23	N-Term(iTRAQ4plex); S6(Phospho); C18(Ca
tHPRPsVSLPLPTVLLDR	F1M3W5	N-Term(iTRAQ4plex); S6(Phospho)
sGTsQEELR	F1M4A4	N-Term(iTRAQ4plex); S4(Phospho)
gMDASLsPTk	D3ZHV2	N-Term(iTRAQ4plex); S7(Phospho); K10(iTR
iAsISk	D3ZFJ6	N-Term(iTRAQ4plex); S3(Phospho); K6(iTRA
iSLPLPNFsSLNLR	P31000	N-Term(iTRAQ4plex); S9(Phospho)
aAENNSGVESSSGYGsP(	D4A9D6	N-Term(iTRAQ4plex); S16(Phospho)
kLEkEEEEGIsQEssEEEQ	Q8K585	K1(iTRAQ4plex); N-Term(iTRAQ4plex); K4(iT
cLsPSSAPSLGPDGGGG(	O55170	N-Term(iTRAQ4plex); C1(Carbamidomethyl);
fLLSEsGSGk	M0RAQ6	N-Term(iTRAQ4plex); S6(Phospho); K10(iTR
sVSTPSEAGsQDDLGPLT	A0A173DW30	N-Term(iTRAQ4plex); S10(Phospho)
lRSsAsESR	A0A0G2JZX5	N-Term(iTRAQ4plex); S4(Phospho); S6(Phos
lADMYGGMDsDkDS	P55280	N-Term(iTRAQ4plex); S10(Phospho); K12(iT
eEGTGEsAGGSPARsLTR	B5DF41	N-Term(iTRAQ4plex); S7(Phospho); S15(Pho
iLDPNSGEPAPVLSSPPPT	F1MAA5	N-Term(iTRAQ4plex); S29(Phospho); K32(iT
eGkPEDEVEPEDEEksDE	M0RBL8	N-Term(iTRAQ4plex); K3(iTRAQ4plex); K15(
eGINPGYDDYADsDEDQH	Q04931	N-Term(iTRAQ4plex); S13(Phospho)
aETEEAEEPEEDGEDNVS	P15205;F1LRL9	N-Term(iTRAQ4plex); S20(Phospho); S22(Ph
dGPGDDRHStksDLPVHT	D4A1G8	N-Term(iTRAQ4plex); T10(Phospho); K11(iT
mVEPENAVTITPLRPEDD	D3ZWS0	N-Term(iTRAQ4plex); S20(Phospho)
rMPsPk	P28818	N-Term(iTRAQ4plex); S4(Phospho); K6(iTRA
sELPsSAPGGGGV	P59649	N-Term(iTRAQ4plex); S5(Phospho)
sPAEVksPATVksPGEAk	F1LRZ7	N-Term(iTRAQ4plex); K6(iTRAQ4plex); S7(P
cYEsPPSsPEMNIPSLNN(	B5DFE0	N-Term(iTRAQ4plex); C1(Carbamidomethyl);
qGLANHsPGASR	Q4G016	N-Term(iTRAQ4plex); S7(Phospho)
nFRPAGGAVLHsPGAMFI	Q4V7D2	N-Term(iTRAQ4plex); S12(Phospho)
eLsLDDPEVEQVR	Q5XI97	N-Term(iTRAQ4plex); S3(Phospho)
sVQPIsEER	B2GVB4	N-Term(iTRAQ4plex); S6(Phospho)
sPAEAksPVVAk	F1LRZ7	N-Term(iTRAQ4plex); K6(iTRAQ4plex); S7(P
sPVEVkSPAsVksPSEAk	F1LRZ7	N-Term(iTRAQ4plex); K6(iTRAQ4plex); S10(
nLLsVAYk	P62260;P61983;P68511;P	N-Term(iTRAQ4plex); S4(Phospho); K8(iTRA
lVDVIcEkMsESLDTADPA	P63012	N-Term(iTRAQ4plex); C6(Carbamidomethyl);
tLYEEAEEASDISQQVANI	O35095	N-Term(iTRAQ4plex); S22(Phospho)
vFEDNsNEkLFNEEEGPN	D4A997	N-Term(iTRAQ4plex); S6(Phospho); K9(iTRA
nWPSDEMPDFPksDDSk	D4A6C5	N-Term(iTRAQ4plex); K12(iTRAQ4plex); S13
lGLGTLSsSLSR	M0RAP5	N-Term(iTRAQ4plex); S8(Phospho)
rQsLGGFLk	Q3ZB98	N-Term(iTRAQ4plex); S3(Phospho); K9(iTRA
wVDSsPENSGSDWDSAF	Q6MG51	N-Term(iTRAQ4plex); S5(Phospho); K27(iTR
sNsTETLSPAk	M0R5P8	N-Term(iTRAQ4plex); S3(Phospho); K11(iTR
sPsTESQLASk	F8WFS9	N-Term(iTRAQ4plex); S3(Phospho); K11(iTR
kAsGsENEGDYNPGR	Q14TE9	K1(iTRAQ4plex); N-Term(iTRAQ4plex); S3(P
rLsVGSSMR	D3ZDC2	N-Term(iTRAQ4plex); S3(Phospho)
yGsQEHPIGDk	A0A0G2JW85	N-Term(iTRAQ4plex); S3(Phospho); K11(iTR
sESkEDVFGLVPFEEITGS	Q4G045	N-Term(iTRAQ4plex); S1(Phospho); K4(iTRA

Information Classification: General

sAADsIsESVPVGPk	D3ZVQ0	N-Term(iTRAQ4plex); S5(Phospho); S7(Phos
skGEEEEAHLHIDILQSGs	D4A9W8	N-Term(iTRAQ4plex); K2(iTRAQ4plex); S19(
dGPNALTPPPTtPEWVk	P06686;P06687;P06685	N-Term(iTRAQ4plex); T12(Phospho); K17(iT
sPAVAksPAEVksPAEVk	F1LRZ7	N-Term(iTRAQ4plex); K6(iTRAQ4plex); S7(P
fGsPPQRDPNWIGDR	G3V793	N-Term(iTRAQ4plex); S3(Phospho)
tSsGSDTDIYSFHSATEQE	A0A0G2JZ27	N-Term(iTRAQ4plex); S3(Phospho)
sPsPISTLSHLAGSAGR	A0A0H2UHW6	N-Term(iTRAQ4plex); S1(Phospho); S3(Phos
sFsLPADPILQAAk	M0R4A0	N-Term(iTRAQ4plex); S3(Phospho); K14(iTR
sYEDLTELEDR	B1WC06	N-Term(iTRAQ4plex); S1(Phospho)
mERPSISVISPTsPGALk	A0A0G2KAV8	N-Term(iTRAQ4plex); S13(Phospho); K18(iT
eEVksPVEEVk	F1LRZ7	N-Term(iTRAQ4plex); K4(iTRAQ4plex); S5(P
nHPDRGsDtSPEAEASSG	Q9WVR6	N-Term(iTRAQ4plex); S7(Phospho); T9(Phos
aHLLSLLQQAETsPTHR	D3ZVN5	N-Term(iTRAQ4plex); S13(Phospho)
rSmEVNFLSYVVLstAALP	Q8CIY7	N-Term(iTRAQ4plex); M3(Oxidation); S14(Ph
sEADTLPRsGEQER	D3ZEI4	N-Term(iTRAQ4plex); S9(Phospho)
lPNLAsPSAEGPPRPcPGA	Q5XI21	N-Term(iTRAQ4plex); S6(Phospho); C16(Car
gSsGEGLPFAEEGNLTIk	D4A9T0	N-Term(iTRAQ4plex); S3(Phospho); K18(iTR
lTYSQETcENLQEMLGELI	Q9WU70	N-Term(iTRAQ4plex); C8(Carbamidomethyl);
vVHAVDGLsENGIQPLSS	Q920Q0	N-Term(iTRAQ4plex); S9(Phospho); K27(iTR
sRSPtPQNQDGDTMVEk	D3ZYJ5	N-Term(iTRAQ4plex); T5(Phospho); K17(iTR
fPVPELsDQEDDSSSEED	Q5XIP1	N-Term(iTRAQ4plex); S7(Phospho)
sPFEIIsPPAsPPEmTGQR	P34926	N-Term(iTRAQ4plex); S7(Phospho); S11(Pho
vESEEsGDEEGkk	Q5HZV9	N-Term(iTRAQ4plex); S6(Phospho); K12(iTR
mtPPLPAR	P47819	N-Term(iTRAQ4plex); T2(Phospho)
lLQTcFSsPTDDSMDR	Q6IMX7	N-Term(iTRAQ4plex); C5(Carbamidomethyl);
iATLAsGLEVGk	P06687	N-Term(iTRAQ4plex); S6(Phospho); K12(iTR
vLTTFLPDsSVSGTSL	C0IXW5	N-Term(iTRAQ4plex); S9(Phospho)
sMsAPSDLEMIGNEDLEF	F1LZX5	N-Term(iTRAQ4plex); S3(Phospho)
eAsEGAAEAPR	F1M9X4	N-Term(iTRAQ4plex); S3(Phospho)
yVLSSQHRPDIsPTER	F1LT10	N-Term(iTRAQ4plex); S12(Phospho)
mGQAGstISNSHAQPFDF	P08050	N-Term(iTRAQ4plex); M1(Oxidation); S6(Pho
tEDGDWEWsDDEmDEkS	A0A0G2K007	N-Term(iTRAQ4plex); S9(Phospho); M13(Ox
vEEEDTGDPFGFDsDDES	D4ADT3	N-Term(iTRAQ4plex); S14(Phospho); K24(iT
sLFLEQSSAGPYANSDEE	A0A0G2K4J8	N-Term(iTRAQ4plex); S23(Phospho); K27(iT
eVSSGsQGQPEGAVIEGI	F1M9X4	N-Term(iTRAQ4plex); S6(Phospho)
qSscESITDEFSR	P0C6C0	N-Term(iTRAQ4plex); S3(Phospho); C4(Carb
iVPSLLFNLQHVEEAESRs	F1LTW9	N-Term(iTRAQ4plex); S19(Phospho); K28(iT
mLAESDEsGDEESVSQTI	D4A9D8	N-Term(iTRAQ4plex); M1(Oxidation); S8(Pho
vGLQsPSVETR	A0A0G2K652	N-Term(iTRAQ4plex); S5(Phospho)
aADPPAENSsAPEAEQGG	D3ZEV0	N-Term(iTRAQ4plex); S10(Phospho)
dAATEEPGsPVkstPASPV	F8WFS9	N-Term(iTRAQ4plex); S9(Phospho); K12(iTR
gSNPFEDEAEEELAtPEAE	O54924	N-Term(iTRAQ4plex); T15(Phospho); K22(iT
rPQPSAPQLLPEDGsVED	Q62866	N-Term(iTRAQ4plex); S15(Phospho)
vPATSkPSPTPTPTPAGA	A0A0G2K0M8	N-Term(iTRAQ4plex); K6(iTRAQ4plex); S20(
iSTAsGDGR	P63095	N-Term(iTRAQ4plex); S5(Phospho)
tDEEIsS	P61765	N-Term(iTRAQ4plex); S6(Phospho)
sLSEVcLR	D3ZWQ0	N-Term(iTRAQ4plex); S1(Phospho); C6(Carb
kLIGTAVPQRTsPTGPk	Q5XIT1	N-Term(iTRAQ4plex); K1(iTRAQ4plex); S12(
tAkDsDDDDDVTVTVDRD	P32851	N-Term(iTRAQ4plex); K3(iTRAQ4plex); S5(P
vAEQTFIkDDYLETLSsPk	F1M1Y0	N-Term(iTRAQ4plex); K8(iTRAQ4plex); S17(
dGSEQTDEEAEGPFsDDE	B0BNL5	N-Term(iTRAQ4plex); S15(Phospho); K23(iT
eSTAAYQTSsSPPIDAAA	P15205;F1LRL9	N-Term(iTRAQ4plex); S10(Phospho)
sIGSASPGPGQPPLsSPT	Q2THW7	N-Term(iTRAQ4plex); S15(Phospho)
sLTIRPAIELQELDsPDDA	D3ZXB1	N-Term(iTRAQ4plex); S15(Phospho); K24(iT
rsLsGSSPcPk	A0A0G2K2M9	N-Term(iTRAQ4plex); S2(Phospho); S4(Phos

Information Classification: General

ncEsDTEENIAR	Q5BJN2	N-Term(iTRAQ4plex); C2(Carbamidomethyl);
sFLSEPSsPGR	D3ZKC9	N-Term(iTRAQ4plex); S8(Phospho)
kALsFDNRGEEHR	F1LMV8	N-Term(iTRAQ4plex); K1(iTRAQ4plex); S4(P
kPGAGGsPALVR	D4A644	N-Term(iTRAQ4plex); K1(iTRAQ4plex); S7(P
tsIHNFMAHPEFR	P11506	N-Term(iTRAQ4plex); S2(Phospho)
lLASPDAsTLENSWSPDE	G3V887	N-Term(iTRAQ4plex); S8(Phospho); K20(iTR
kEDSDEDEDEEDEDDsDI	P13383	N-Term(iTRAQ4plex); K1(iTRAQ4plex); S16(
lNkTDEEIsS	P61765	N-Term(iTRAQ4plex); K3(iTRAQ4plex); S9(P
sPAEVksPAEAksPAEAk	F1LRZ7	N-Term(iTRAQ4plex); K6(iTRAQ4plex); S7(P
aLGVISNFQSsPk	D3ZH14	N-Term(iTRAQ4plex); S11(Phospho); K13(iT
eYGsPLk	D4A3G2	N-Term(iTRAQ4plex); S4(Phospho); K7(iTRA
sSLLLDTVTsIPsSR	P34926	N-Term(iTRAQ4plex); S10(Phospho); S13(Ph
vPLPGPGsPEVk	D3ZE17	N-Term(iTRAQ4plex); S8(Phospho); K12(iTR
aMGDEDsGDSDTSPkPSI	G3V849	N-Term(iTRAQ4plex); S7(Phospho); K15(iTR
mAPTPIPTRsPsDSSTAST	Q07266;A0A0H2UHL9	N-Term(iTRAQ4plex); S10(Phospho); S12(Ph
sGsPSDNSGAEEMEVALA	P47196	N-Term(iTRAQ4plex); S3(Phospho); K19(iTR
lQNsADEEEk	Q641X2	N-Term(iTRAQ4plex); S4(Phospho); K10(iTR
sLGGQQGsPk	A0A0G2JUG7	N-Term(iTRAQ4plex); S8(Phospho); K10(iTR
sLSGSTNTVssEGVTk	D3ZU84	N-Term(iTRAQ4plex); S10(Phospho); S11(Ph
lcYVALDFEQEmATAASsS	P60711;P63259	N-Term(iTRAQ4plex); C2(Carbamidomethyl);
sSSVTsIDkESR	Q9WU70	N-Term(iTRAQ4plex); S6(Phospho); K9(iTRA
sQsLTNAFNLPEPAPPRP	P09951	N-Term(iTRAQ4plex); S3(Phospho); K26(iTR
gGADEDSASDLsDSER	A0A0G2K2Y5	N-Term(iTRAQ4plex); S12(Phospho)
gHYEFPVVDDLssGDTkEI	D3Z9D0	N-Term(iTRAQ4plex); S12(Phospho); S13(Ph
rEsPESEGPIYEGLIL	G3V8A5	N-Term(iTRAQ4plex); S3(Phospho)
yDSNVSGQSsFGTSPAAD	Q5XIG4	N-Term(iTRAQ4plex); S10(Phospho); K22(iT
ssDSDVSDVSAISR	A0A0G2KAV8	N-Term(iTRAQ4plex); S2(Phospho)
dcGFLsQALSFR	P31016	N-Term(iTRAQ4plex); C2(Carbamidomethyl);
sPTSPVPVPVM	P63319	N-Term(iTRAQ4plex); S1(Phospho)
tQDDLVEQNSSYVQDsPS	Q5U2U7	N-Term(iTRAQ4plex); S16(Phospho); K19(iT
sTGVQtSPDLR	D4A4J1	N-Term(iTRAQ4plex); T6(Phospho)
eDGGPPsLER	Q5I0K2	N-Term(iTRAQ4plex); S7(Phospho)
sSQALLSQLYQsPNRR	O09032	N-Term(iTRAQ4plex); S12(Phospho)
sQsADAPGPTER	Q9Z2X5	N-Term(iTRAQ4plex); S3(Phospho)
lGYILTcPsNLGTGLR	Q5BJT9	N-Term(iTRAQ4plex); C7(Carbamidomethyl);
aSQSsLESSSGPPcIR	A0A0G2KAV8	N-Term(iTRAQ4plex); S5(Phospho); C14(Ca
dSALQDTDDsDDDPVLIP	D4A8L4	N-Term(iTRAQ4plex); S10(Phospho)
rGsETEQLLTPNPGYGTQ	D3ZRF9	N-Term(iTRAQ4plex); S3(Phospho)
vPATSkPSPTPTPtPAGAA	A0A0G2K0M8	N-Term(iTRAQ4plex); K6(iTRAQ4plex); T14(
vkAsPITNDGEDEFVPSD	Q14TE9	N-Term(iTRAQ4plex); K2(iTRAQ4plex); S4(P
aPsWIDTGLSEMR	P23928	N-Term(iTRAQ4plex); S3(Phospho)
tMDkSELVQk	P35213	N-Term(Acetyl); T1(Phospho); K4(iTRAQ4ple
sTIGVMVtASHNPEEDNG	B2RYN0	N-Term(iTRAQ4plex); T8(Phospho); K20(iTR
rQEPLRQPsPQR	D3ZDC0	N-Term(iTRAQ4plex); S9(Phospho)
lLsFQR	A0A0G2JW08	N-Term(iTRAQ4plex); S3(Phospho)
gVNFAEEPMRSDsENGEI	P12369	N-Term(iTRAQ4plex); S13(Phospho)
lTSIGsDEDEETETYQEk	G3V6N7	N-Term(iTRAQ4plex); S6(Phospho); K18(iTR
tYEtPPPSPGLDPTFSNQF	O88953	N-Term(iTRAQ4plex); T4(Phospho)
kAGGANYDAQsE	B0BNA5	N-Term(iTRAQ4plex); K1(iTRAQ4plex); S11(
tSPcQsPTAPEYSAPSLPII	D4ABN3	N-Term(iTRAQ4plex); C4(Carbamidomethyl);
eNFFQVFsPVFER	Q7TQ19	N-Term(iTRAQ4plex); S8(Phospho)
dGVANVsIEDR	Q6LDS4	N-Term(iTRAQ4plex); S7(Phospho)
nVNENFGPNTEmHLVPIL	Q4V8H5	N-Term(iTRAQ4plex); M12(Oxidation); K28(iT
mGQAGstIsNSHAQPFDF	P08050	N-Term(iTRAQ4plex); S6(Phospho); T7(Phos
sGDSSGSGSGGGGAAPA	F1MAS1	N-Term(iTRAQ4plex); S24(Phospho); K31(iT

vFsVLREESESVLTLk	P63329;A0A0G2K7T5	N-Term(iTRAQ4plex); S3(Phospho); K16(iTR
mQVDQEEPHTEEQQPQ	F1LRV4	N-Term(iTRAQ4plex); T17(Phospho); K22(iT
dYcEADGLsER	A0A0G2K0F3	N-Term(iTRAQ4plex); C3(Carbamidomethyl);
gLSEkENsE	A0A0G2JSR2	N-Term(iTRAQ4plex); K5(iTRAQ4plex); S8(P
nWYQSMIPQsPsPPLDER	F1M1T9	N-Term(iTRAQ4plex); S10(Phospho); S12(Ph
iQGsPTPASk	D3ZE17	N-Term(iTRAQ4plex); S4(Phospho); K10(iTR
kGEDLDFWLsTTPPPAAA	B5DFK6	N-Term(iTRAQ4plex); K1(iTRAQ4plex); S10(
dSFSQESsPsSPSDLAk	Q9JKS6;D3Z9C7	N-Term(iTRAQ4plex); S8(Phospho); S10(Pho
sDIsPLTPR	P15205;F1LRL9	N-Term(iTRAQ4plex); S4(Phospho)
tASPPPPPkR	B2RYB3;A0A0G2K4F6	N-Term(iTRAQ4plex); T1(Phospho); K9(iTRA
iVPSLLFNLQHVEEAEsRs	F1LTW9	N-Term(iTRAQ4plex); S17(Phospho); S19(Ph
fSIEsLLEEETQVR	F1LPM3	N-Term(iTRAQ4plex); S5(Phospho)
eGEsLEDLMk	Q5XI38	N-Term(iTRAQ4plex); S4(Phospho); K10(iTR
dFsGGWR	F1M8H5	N-Term(iTRAQ4plex); S3(Phospho)
tAAAEPPPPPPPPPEDDP	P33568	N-Term(iTRAQ4plex); S22(Phospho)
iLTGsSEPEAAPAPR	G3V6X7	N-Term(iTRAQ4plex); S5(Phospho)
tsFEEGTGEcEVFSk	B4F772	N-Term(iTRAQ4plex); S2(Phospho); C10(Car
aQENYEGSEEVsPPQTk	D3ZC84	N-Term(iTRAQ4plex); S12(Phospho); K17(iT
yLsFTPPEk	Q64303	N-Term(iTRAQ4plex); S3(Phospho); K9(iTRA
gSVSsYDR	Q9Z0W5;A0A0G2JWR2	N-Term(iTRAQ4plex); S5(Phospho)
nAIsLPQLNQATYDSLVVS	A1A5P0	N-Term(iTRAQ4plex); S4(Phospho); K20(iTR
skPADSDsDDDPLEAFMA	D4A031	N-Term(iTRAQ4plex); K2(iTRAQ4plex); S8(P
aAGGGGGsGEDEAQSR	Q569C9	N-Term(iTRAQ4plex); S8(Phospho)
gAASGPAAAEEAGsEEAC	F1LRY7	N-Term(iTRAQ4plex); S14(Phospho)
tEELPRPQsPSDLDSLDG	P54258	N-Term(iTRAQ4plex); S9(Phospho)
qSLGEsPR	A0A0G2JW01;F1LWK7	N-Term(iTRAQ4plex); S6(Phospho)
gTEETSWsGEER	Q63624	N-Term(iTRAQ4plex); S8(Phospho)
tRHsPTPQQSNR	B2RYB3;A0A0G2K4F6	N-Term(iTRAQ4plex); S4(Phospho)
vLENLDRsNEk	Q5U3Z5	N-Term(iTRAQ4plex); S8(Phospho); K11(iTR
tTPsPkTPPGSGEPPksGE	D4A1Q2	N-Term(iTRAQ4plex); S4(Phospho); K6(iTRA
fFTTGSDSESESSLsGEE	B5DFC8	N-Term(iTRAQ4plex); S15(Phospho); K22(iT
yDAIFDSLsPVDGFLSGDk	Q5JC29	N-Term(iTRAQ4plex); S9(Phospho); K19(iTR
lLGGFQsLGGPGEPGR	Q5BJT1	N-Term(iTRAQ4plex); S7(Phospho)
dARPAsPAGIPk	D3ZS76	N-Term(iTRAQ4plex); S6(Phospho); K12(iTR
tLsDESVcSNR	F1M8G8	N-Term(iTRAQ4plex); S3(Phospho); C8(Carb
kNDGVkESsEsTNTTIEDE	P11275	N-Term(iTRAQ4plex); K1(iTRAQ4plex); K6(iT
rPDYAPMEsSDEEDEEFQ	D4ACM9	N-Term(iTRAQ4plex); S9(Phospho); K21(iTR
kVPVPEGLDLDAWINEPR	B5DFK6	N-Term(iTRAQ4plex); K1(iTRAQ4plex); S19(
aETEtSPGHAPDR	D4AE69	N-Term(iTRAQ4plex); T5(Phospho)
rPPsPDVIVLSDSEQPSsP	G3V8R7	N-Term(iTRAQ4plex); S4(Phospho); S18(Pho
qDVDNAsLAR	P31000	N-Term(iTRAQ4plex); S7(Phospho)
gTGRPNsPQR	B4F786	N-Term(iTRAQ4plex); S7(Phospho)
kVVEPANSDsDSELGNIPI	Q14TE9	N-Term(iTRAQ4plex); K1(iTRAQ4plex); S10(
rNNWkLEPAFsSk	D4AEI6	N-Term(iTRAQ4plex); K5(iTRAQ4plex); S11(
iIPTLEEGLQLPsPTATSQ	P61980	N-Term(iTRAQ4plex); S13(Phospho); C29(Ca
aSGQSFEVILkSPsDLsPE	A0A0G2K8P5	N-Term(iTRAQ4plex); K11(iTRAQ4plex); S14
dSAsPGAASGLDPLDSAR	F1LRY7	N-Term(iTRAQ4plex); S4(Phospho)
sVGsPEESTER	F1LWN1	N-Term(iTRAQ4plex); S4(Phospho)
sPQMVSAIVR	A0A0G2JT93	N-Term(iTRAQ4plex); S1(Phospho)
nGGsPALNNNPR	P97544	N-Term(iTRAQ4plex); S4(Phospho)
eSLkEEDEsDDDNm	P18422	N-Term(iTRAQ4plex); K4(iTRAQ4plex); S9(P
eDDAESGGRRsEAEEGE	A0A0G2K0F3	N-Term(iTRAQ4plex); S11(Phospho)
dMPHPLAGSSSEEAVGG	D4A060	N-Term(iTRAQ4plex); S22(Phospho)
yELSNDATQsDDEEkLQS	A0A0G2JX77	N-Term(iTRAQ4plex); S10(Phospho); K15(iT
aAPAtPPPPVk	D3ZWX4	N-Term(iTRAQ4plex); T5(Phospho); K11(iTR

dSTVsFSETP	Q8R2E7	N-Term(iTRAQ4plex); S5(Phospho)
eLALSsPEDLTQDFEELkF	P34926;Q63330	N-Term(iTRAQ4plex); S6(Phospho); K18(iTR
aGIPQHHPPMAQNLQYPI	F1LNP8	N-Term(iTRAQ4plex); S20(Phospho); K24(iT
vkEYEEEIHsLkER	D3ZCL8	N-Term(iTRAQ4plex); K2(iTRAQ4plex); S10(
gFEEEHkDsDDDssDDEQ	D3ZMS1	N-Term(iTRAQ4plex); K7(iTRAQ4plex); S9(P
eSALSRAsDEsLRk	Q7TP93	N-Term(iTRAQ4plex); S8(Phospho); S11(Pho
kSEVQAHsPSR	F1MAQ5;A0A0U1RRX4	N-Term(iTRAQ4plex); K1(iTRAQ4plex); S8(P
sAsAPAsPR	F1M787	N-Term(iTRAQ4plex); S3(Phospho); S7(Phos
sEsLDEEEk	D3ZQR3	N-Term(iTRAQ4plex); S3(Phospho); K9(iTRA
skELsPGSGQk	A0A0H2UHC0	N-Term(iTRAQ4plex); K2(iTRAQ4plex); S5(P
gFsPQHk	P63329	N-Term(iTRAQ4plex); S3(Phospho); K7(iTRA
eIQNGTLHEsDSEHVPQD	D4A4Z9	N-Term(iTRAQ4plex); S10(Phospho); K20(iT
sPTENVNTPVGk	A0A0G2KA27	N-Term(iTRAQ4plex); S1(Phospho); K12(iTR
mADTGsPGMQR	A0A0G2KA12	N-Term(iTRAQ4plex); S6(Phospho)
nSPTFksFEER	Q499Q2	N-Term(iTRAQ4plex); K6(iTRAQ4plex); S7(P
gSLGsLDR	B4F7F3	N-Term(iTRAQ4plex); S5(Phospho)
sPAEVksPVTVk	F1LRZ7	N-Term(iTRAQ4plex); S1(Phospho); K6(iTRA
gEVAPkEtPk	A0A0G2K613	N-Term(iTRAQ4plex); K6(iTRAQ4plex); T8(P
sAPPNLWAAQR	O35147	N-Term(iTRAQ4plex); S1(Phospho)
sAkDsDDEEEVVHVDR	P61265	N-Term(iTRAQ4plex); K3(iTRAQ4plex); S5(P
sATPATDGR	Q63092	N-Term(iTRAQ4plex); S1(Phospho)
sPGEAksPAEAk	F1LRZ7	N-Term(iTRAQ4plex); S1(Phospho); K6(iTRA
aSLQNLLSAsQAQLR	P25093	N-Term(iTRAQ4plex); S10(Phospho)
nASGVVNsSPR	Q4V7D3	N-Term(iTRAQ4plex); S8(Phospho)
sFsIsPVR	E9PTE1	N-Term(iTRAQ4plex); S3(Phospho); S5(Phos
fEQSSDSISsDEEELR	Q3B7K9	N-Term(iTRAQ4plex); S10(Phospho)
nSAPASMsPDGTR	M0R6K4	N-Term(iTRAQ4plex); S8(Phospho)
aTQGLDYVPSAGTISPTs	Q63330	N-Term(iTRAQ4plex); S18(Phospho); S19(Ph
dEPPsEGEAEEEEkEk	P19527	N-Term(iTRAQ4plex); S5(Phospho); K14(iTR
eTEItPEDGED	G3V874;A0A0G2K1Q9	N-Term(iTRAQ4plex); T5(Phospho)
vVNQDsT	M0RBB2	N-Term(iTRAQ4plex); S6(Phospho)
kGDsSAEELk	A0A0G2JYG9	N-Term(iTRAQ4plex); K1(iTRAQ4plex); S4(P
gEPVGGQkEPVPAWEGk	P34926	N-Term(iTRAQ4plex); K8(iTRAQ4plex); K17(
eNVEYIEREEsDGEYDEF	O35986	N-Term(iTRAQ4plex); S11(Phospho)
ePLPVEQDDDIEVIVDETS	D3ZFK6	N-Term(iTRAQ4plex); S26(Phospho)
eGEVGsDGER	A0A1B0GWY6	N-Term(iTRAQ4plex); S6(Phospho)
sPGtPAGEGSGSPPk	A0A0G2JSM7;D3ZZ99	N-Term(iTRAQ4plex); S1(Phospho); T4(Phos
qAEVADQQTTDLPAENGI	Q5U1W8	N-Term(iTRAQ4plex); S26(Phospho); K30(iT
aTEEPSGTGsDELIk	G3V8L9	N-Term(iTRAQ4plex); S10(Phospho); K15(iT
gkPSsEDEkEAGGLR	Q6MGB4	N-Term(iTRAQ4plex); K2(iTRAQ4plex); S5(P
lPsVGDQEPPGHEk	D4ADU2	N-Term(iTRAQ4plex); S3(Phospho); K14(iTR
sFDFEGSLsPVIAPk	F1M801	N-Term(iTRAQ4plex); S9(Phospho); K15(iTR
lIQHAQIPPEDSEIITNMAH	P61765	N-Term(iTRAQ4plex); S29(Phospho)
sGSIVELLAGGGSScsPVL	P51111	N-Term(iTRAQ4plex); S1(Phospho); C15(Ca
lcDFGFARTLAAPGDVYTI	Q9JM01	N-Term(iTRAQ4plex); C2(Carbamidomethyl);
rGIsALLLNQGDGDR	A0A0G2JZK7	N-Term(iTRAQ4plex); S4(Phospho)
qEPLGsDSEGVNcLAYDE	B2RYG6	N-Term(iTRAQ4plex); S6(Phospho); C13(Ca
eQFLEAVTVSAGDEEEDE	A0A0G2JZK7	N-Term(iTRAQ4plex); S21(Phospho)
sVStSPSILPAFLk	D3ZF21	N-Term(iTRAQ4plex); S1(Phospho); T4(Phos
qNSEALIENDELDcsPE	D3ZSU3	N-Term(iTRAQ4plex); C14(Carbamidomethyl
dVAGDAsESALLk	P06687	N-Term(iTRAQ4plex); S7(Phospho); K13(iTR
dVNAAIAtIk	P68370	N-Term(iTRAQ4plex); T8(Phospho); K10(iTR
yAPTTTPsPEDGFEVQPL	P28572	N-Term(iTRAQ4plex); S8(Phospho); K22(iTR
aGDmLEDsPk	Q8VHK7	N-Term(iTRAQ4plex); M4(Oxidation); S8(Pho
pTTQQsPQDEQEk	G3V8A5	N-Term(iTRAQ4plex); S6(Phospho); K13(iTR

lTGPPPsFEGk	Q09QM4	N-Term(iTRAQ4plex); S7(Phospho); K11(iTR
sDNGELEDkPPAPPVR	Q64303	N-Term(Acetyl); S1(Phospho); K9(iTRAQ4ple
rkSSSsVHLMEPQtTVVHN	P11730	N-Term(iTRAQ4plex); K2(iTRAQ4plex); S6(P
rHsTSDLSDATFSDIR	F1M2D4	N-Term(iTRAQ4plex); S3(Phospho)
dLsPPLNGSTVScSPDR	P34926	N-Term(iTRAQ4plex); S3(Phospho); C13(Car
tEAVsPTVSQLSAVFENSE	O35867	N-Term(iTRAQ4plex); S5(Phospho); K28(iTR
sADVsPTTEGVk	D3ZQG6	N-Term(iTRAQ4plex); S5(Phospho); K12(iTR
sSLLLDTVTsIPSSR	P34926	N-Term(iTRAQ4plex); S10(Phospho)
ekEAAGGNDSSGATsPIN	Q05695	N-Term(iTRAQ4plex); K2(iTRAQ4plex); S15(
eVGDVIALSDIsPSGAADF	Q3KR56	N-Term(iTRAQ4plex); S12(Phospho)
sLEAIEEkEsSEENGkL	A0A0G2K4N6	N-Term(iTRAQ4plex); K8(iTRAQ4plex); S10(
sGNYPSSLsNETDR	Q68FQ9	N-Term(iTRAQ4plex); S9(Phospho)
aAGGIILtASHcPGGPGGE	D3ZVR9	N-Term(iTRAQ4plex); T8(Phospho); C12(Car
aAFDDAIAELDTLsEESYk	P62260	N-Term(iTRAQ4plex); S14(Phospho); K19(iT
tSFDENDsEELEDkDSk	A0A0H2UHV4	N-Term(iTRAQ4plex); S8(Phospho); K14(iTR
gWsPPPEVR	G3V9U0	N-Term(iTRAQ4plex); S3(Phospho)
sLDsDEsEDEDDDYQQk	Q62785	N-Term(iTRAQ4plex); S4(Phospho); S7(Phos
qQLQELGAGsPGPVTNk	G3V8Y7	N-Term(iTRAQ4plex); S10(Phospho); K17(iT
tSSESIVSVPASSTSGSPs	A0A0G2JTB5	N-Term(iTRAQ4plex); T1(Phospho); S19(Pho
gATADDSGGGsPVLAAk	A0A0G2JZ27	N-Term(iTRAQ4plex); S11(Phospho); K17(iT
aAAtPEsQEPQAk	A0A0G2K613	N-Term(iTRAQ4plex); T4(Phospho); S7(Phos
dGsFEGLTVk	Q4G027	N-Term(iTRAQ4plex); S3(Phospho); K10(iTR
sMsHQAAIASQR	B1WBS4	N-Term(iTRAQ4plex); S3(Phospho)
fYYSSGSSsPTHAk	Q6IRG7	N-Term(iTRAQ4plex); S9(Phospho); K14(iTR
lGVSVSPsR	Q6TQE1	N-Term(iTRAQ4plex); S8(Phospho)
ssPVcPTAPVETEPk	F1MAF8	N-Term(iTRAQ4plex); S2(Phospho); C5(Carb
rDEsDEEPPRVER	A0A0G2JZ88	N-Term(iTRAQ4plex); S4(Phospho)
sEGGAAWDTPGAsQEEL	Q63638	N-Term(iTRAQ4plex); S13(Phospho)
sLDGASVNENHEIYMk	A0A0G2K1Q9	N-Term(iTRAQ4plex); S1(Phospho); K16(iTR
lLPSSGPSAAEVSTAEEPS	D3ZG78	N-Term(iTRAQ4plex); S20(Phospho)
dGsLNQSSGYR	A0A0G2K2E3	N-Term(iTRAQ4plex); S3(Phospho)
mLPtPSk	F1MAM6	N-Term(iTRAQ4plex); T4(Phospho); K7(iTRA
gNDsDGEAESDDPEk	Q4KLL7	N-Term(iTRAQ4plex); S4(Phospho); K15(iTR
sGsGYEGsTSWk	Q0ZCA7	N-Term(iTRAQ4plex); S3(Phospho); S8(Phos
sDAEEDGGtGsQDEEDSk	P35565	N-Term(iTRAQ4plex); T9(Phospho); S11(Pho
sPSEAksPAGAk	F1LRZ7	N-Term(iTRAQ4plex); S1(Phospho); K6(iTRA
sPAsPFAQTFGTNLEDIk	A0A0U1RRX4;F1MAQ5	N-Term(iTRAQ4plex); S4(Phospho); K18(iTR
sADcsVEEEPWkR	P31596	N-Term(iTRAQ4plex); C4(Carbamidomethyl);
tVGTNtPPsPGFGR	F1LN42	N-Term(iTRAQ4plex); T6(Phospho); S9(Phos
kGAQTsPVPR	F1LR04	N-Term(iTRAQ4plex); K1(iTRAQ4plex); S6(P
hsGGSPEHLQk	A0A0G2KAJ5	N-Term(iTRAQ4plex); S2(Phospho); K11(iTR
rDsPLQGSGQQNSQAGQ	A0A097PIG6	N-Term(iTRAQ4plex); S3(Phospho)
dYNASASTIsPPssMEEDk	P15205;F1LRL9	N-Term(iTRAQ4plex); S10(Phospho); S13(Ph
sQsVVDPTALk	A6JUQ6	N-Term(iTRAQ4plex); S3(Phospho); K11(iTR
lDLVsYFGk	P13233	N-Term(iTRAQ4plex); S5(Phospho); K9(iTRA
sHsLDDLQGDADVGk	F1LU97	N-Term(iTRAQ4plex); S3(Phospho); K15(iTR
hPEGIVSEVEMLSsQER	Q5QD51	N-Term(iTRAQ4plex); S14(Phospho)
sLPGSALELR	F1LSE6	N-Term(iTRAQ4plex); S1(Phospho)
dTSPDkGELVsDEEEDT	D3ZAF7	N-Term(iTRAQ4plex); K6(iTRAQ4plex); S11(
sQSSDTEQPsPTSGGGk	Q5RKG9	N-Term(iTRAQ4plex); S10(Phospho); K17(iT
ikESETFsDsSPIEIIDEFPT	F1LQN3	N-Term(iTRAQ4plex); K2(iTRAQ4plex); S8(P
hPPVsPGk	A0A0G2K6R9;F1M9N9	N-Term(iTRAQ4plex); S5(Phospho); K8(iTRA
yGSGYLLAStPESEL	P31662	N-Term(iTRAQ4plex); T10(Phospho)
rEsVAsGDDRAEEDmDEA	P15205;F1LRL9	N-Term(iTRAQ4plex); S3(Phospho); S6(Phos
sPDRTEEVLSPDGSPSks	Q62847;A0A0G2JW28	N-Term(iTRAQ4plex); K17(iTRAQ4plex); S18

rASLsDLTHLEDIEGLTVR	A0A096MJC6	N-Term(iTRAQ4plex); S5(Phospho)
vQRPEDAsGGSSPSGTSI	F1M8I7	N-Term(iTRAQ4plex); S8(Phospho); K18(iTR
rRsPsPAPPPPPPPPPR	B2RYB3;A0A0G2K4F6	N-Term(iTRAQ4plex); S3(Phospho); S5(Phos
sLGSGEGESESRPGk	Q5BKB9	N-Term(iTRAQ4plex); S1(Phospho); K15(iTR
gNsGLGFSIAGGTDNPHI(	P31016	N-Term(iTRAQ4plex); S3(Phospho); K28(iTR
sRtSPAPWk	A0A0G2K2M9	N-Term(iTRAQ4plex); S1(Phospho); T3(Phos
gDsLkEPTSIADSSR	Q2THW7	N-Term(iTRAQ4plex); S3(Phospho); K5(iTRA
fsPPPVGGQGk	A0A0G2K9J0	N-Term(iTRAQ4plex); S2(Phospho); K11(iTR
fLEQSRsPSVsPSk	F1LSG8	N-Term(iTRAQ4plex); S7(Phospho); S11(Pho
sVsDNDIR	P46462	N-Term(iTRAQ4plex); S3(Phospho)
wGQTLPTTSsTDEGNPk	A0A0G2K677	N-Term(iTRAQ4plex); S10(Phospho); K17(iT
gSIPTSLTALSLASAsPPLS	F1M863	N-Term(iTRAQ4plex); S16(Phospho)
qLsSGVSEIR	G3V913	N-Term(iTRAQ4plex); S3(Phospho)
lLDLSDSTsVASFPSADET	D3ZN37	N-Term(iTRAQ4plex); S9(Phospho)
sPFEIISPPAsPPEMTGQR	P34926	N-Term(iTRAQ4plex); S11(Phospho)
csPTVALVEFSSSPQLR	F1M392	N-Term(iTRAQ4plex); C1(Carbamidomethyl);
iGFGPsPFsLTEGLPk	F1LRZ7	N-Term(iTRAQ4plex); S6(Phospho); S9(Phos
eNQSEGkGSDsDSEGDN	Q793F9	N-Term(iTRAQ4plex); K7(iTRAQ4plex); S11(
mEEtEALEGAIPHSADcEL	F1M575	N-Term(Acetyl); M1(Oxidation); T4(Phospho)
kAsPPSV	F1M9V7	N-Term(iTRAQ4plex); K1(iTRAQ4plex); S3(P
kDDQsPLDIk	A0A0U1RRX4;F1MAQ5	N-Term(iTRAQ4plex); K1(iTRAQ4plex); S5(P
vFDDsDEkEDEEDTDVR	D4A997	N-Term(iTRAQ4plex); S5(Phospho); K8(iTRA
vAsEEEEVPLVVYLk	B1VKB4	N-Term(iTRAQ4plex); S3(Phospho); K15(iTR
yPQsPTNSk	G3V746	N-Term(iTRAQ4plex); S4(Phospho); K9(iTRA
sSsPILSEEEAEGSLR	M0R7T1	N-Term(iTRAQ4plex); S3(Phospho)
skEEQELHDIHsTRsk	A0A1B0GWN8	N-Term(iTRAQ4plex); K2(iTRAQ4plex); S12(
rtHSDAsDDEAFPTSk	Q6TUE6	N-Term(iTRAQ4plex); T2(Phospho); S7(Phos
kLADMYGGMDsDkDS	P55280	N-Term(iTRAQ4plex); K1(iTRAQ4plex); S11(
lEGDAEESLGsPSDPSFSI	A0A0G2JTT6	N-Term(iTRAQ4plex); S11(Phospho); K27(iT
sRtLsQSSESGTLPSGPP(	A0A0G2JSU4	N-Term(iTRAQ4plex); T3(Phospho); S5(Phos
hsPGVSkEDSEEQTVkPG	P34926;Q63330	N-Term(iTRAQ4plex); S2(Phospho); K7(iTRA
aPEGEETEFYVsPEDLEA	A0A0G2JX56	N-Term(iTRAQ4plex); S12(Phospho)
gALSPITDTtEVDTGIHVPS	A0A0G2K7K9	N-Term(iTRAQ4plex); T10(Phospho)
vLTNFksSEIEQAVQSLDR	A1L108	N-Term(iTRAQ4plex); K6(iTRAQ4plex); S7(P
lQAASWDSQscPQERPA(	D4ABB2	N-Term(iTRAQ4plex); S10(Phospho); C11(Ca
sQMEFSISSLSVQEPSTA	D3ZY47	N-Term(iTRAQ4plex); S1(Phospho); K29(iTR
sYsSPDITQALQEEEk	D3ZN39	N-Term(iTRAQ4plex); S3(Phospho); K16(iTR
vDsFTDR	Q80Z30	N-Term(iTRAQ4plex); S3(Phospho)
aLFTLEDGDsPSQSFDAG	A0A0G2KB60	N-Term(iTRAQ4plex); S10(Phospho); K19(iT
dLLNELGsPkEEATEG	D3Z9L5	N-Term(iTRAQ4plex); S8(Phospho); K10(iTR
sFsQPGLR	D4AC16	N-Term(iTRAQ4plex); S3(Phospho)
sEQPEEVLsSEEETAGVE	Q63475	N-Term(iTRAQ4plex); S9(Phospho); K21(iTR
aEEAkDEPPsEGEAEEEE	P19527	N-Term(iTRAQ4plex); K5(iTRAQ4plex); S10(
aVHTsPTGGLGAR	G3V864	N-Term(iTRAQ4plex); S5(Phospho)
lEQEQEGEkDAATEEPGS	F8WFS9	N-Term(iTRAQ4plex); K9(iTRAQ4plex); K21(
tPLESETQSVAPPtGssEE	G3V8G4	N-Term(iTRAQ4plex); T14(Phospho); S16(Ph
eHsNEHVTESVADLLALE	P31662	N-Term(iTRAQ4plex); S3(Phospho); K24(iTR
fPVREDLsDVTDEDTGPA	D3ZKQ4	N-Term(iTRAQ4plex); S8(Phospho); K25(iTR
lVSFHDDsDEDLLHI	Q63002	N-Term(iTRAQ4plex); S8(Phospho)
sDSVLPASHGHLPQAGsL	A0A0G2K382	N-Term(iTRAQ4plex); S17(Phospho)
tsPTGPk	Q5XIT1	N-Term(iTRAQ4plex); S2(Phospho); K7(iTRA
sIPHITSDR	Q62950	N-Term(iTRAQ4plex); S1(Phospho)
eAAEAEPAEPGsPSAETE	F1LMW7	N-Term(iTRAQ4plex); S12(Phospho); K29(iT
aMGDEDsDESGGGSPkP	G3V927	N-Term(iTRAQ4plex); S7(Phospho); K16(iTR
tLTDEVNsPDADRR	D4A510	N-Term(iTRAQ4plex); S8(Phospho)

eYskSMDESR	D3ZCL8	N-Term(iTRAQ4plex); S3(Phospho); K4(iTRA
tVsDLALQNAFGER	F1M0J7	N-Term(iTRAQ4plex); S3(Phospho)
gHWDDGTNDsDLEk	P34926	N-Term(iTRAQ4plex); S10(Phospho); K14(iT
kPVTPDLLMtPsDQGDVD	G3V8F1	K1(iTRAQ4plex); N-Term(iTRAQ4plex); T10(
lSPWEDLSQAPTMDsPLE	F1LR33	N-Term(iTRAQ4plex); S15(Phospho); K19(iT
eAAGGNDsSGATSPINPA	Q05695	N-Term(iTRAQ4plex); S8(Phospho)
qPDPFsPR	Q9Z2X9	N-Term(iTRAQ4plex); S6(Phospho)
tGSNIsGASSDISLDEQYk	D4A9D8	N-Term(iTRAQ4plex); S6(Phospho); K19(iTR
sPsPPPDGsPAATPEIR	Q5HZA7	N-Term(iTRAQ4plex); S3(Phospho); S9(Phos
eIGHAsPPSLsPk	D3ZWM3	N-Term(iTRAQ4plex); S6(Phospho); S11(Pho
qILSADLRsPVYLTVLk	F1M9V7	N-Term(iTRAQ4plex); S9(Phospho); K17(iTR
kPGDGDSFYSDFPPGGs	M0R5P8	N-Term(iTRAQ4plex); K1(iTRAQ4plex); S17(
eTSsPAsPQNLQSDTPAF	P34926	N-Term(iTRAQ4plex); S4(Phospho); S7(Phos
eSEPQAAADATEVkEsAE	Q05175;A0A0G2K1L8	N-Term(iTRAQ4plex); K14(iTRAQ4plex); S16
nSEDDNAADQSEsPPkk	F1M9G3	N-Term(iTRAQ4plex); S13(Phospho); K16(iT
nLIMsGYSPEHDVSGISDI	B2RYN6	N-Term(iTRAQ4plex); S5(Phospho)
sEEEQSSAsVk	G3V9R8	N-Term(iTRAQ4plex); S9(Phospho); K11(iTR
mAsPPPsGPPSAAHTPFH	P34926;Q63330	M1(Oxidation); N-Term(iTRAQ4plex); S3(Pho
sQSsDtEQPSPTSGGGk	Q5RKG9	N-Term(iTRAQ4plex); S4(Phospho); T6(Phos
ksNIsVyEVDGk	Q5XI06	K1(iTRAQ4plex); N-Term(iTRAQ4plex); S2(P
eAEPEGPSVPGEGSTVEs	D4A6A8	N-Term(iTRAQ4plex); S18(Phospho); K27(iT
eEAsDDDMEGDEAVVR	M3ZCQ2	N-Term(iTRAQ4plex); S4(Phospho)
rIsGLIYEETR	P62804	N-Term(iTRAQ4plex); S3(Phospho)
ePAPGDPGcGPGELRPPs	A0A0G2JZ50	N-Term(iTRAQ4plex); C9(Carbamidomethyl);
gkGsGGLNLGNFFASR	P14925	N-Term(iTRAQ4plex); K2(iTRAQ4plex); S4(P
sTLINSLFLTDLYsPEYPG	F1LMC7	N-Term(iTRAQ4plex); S14(Phospho)
aTsVGDQLEAPAR	D4A3X6	N-Term(iTRAQ4plex); S3(Phospho)
rVGtTLAAPk	D4A1S2	N-Term(iTRAQ4plex); T4(Phospho); K10(iTR
lQEETGEEEARPEsGLk	F1M9X4	N-Term(iTRAQ4plex); S14(Phospho); K17(iT
aAQPPTETAEssQAEEEk	P07936	N-Term(iTRAQ4plex); S11(Phospho); S12(Ph
tNQAILSQLYQsPNR	A0A1B0GWQ5	N-Term(iTRAQ4plex); S12(Phospho)
ssASFSTTAVSAR	P61765	N-Term(iTRAQ4plex); S2(Phospho)
yFQISQDEDGsESED	B2RZD5	N-Term(iTRAQ4plex); S11(Phospho)
fQIQDIVVQTQEGREtRsA	A0A140UHX6	N-Term(iTRAQ4plex); T16(Phospho); S18(Ph
sEPVkEEGsELEQPFAQA	A0A0G2JW97	N-Term(iTRAQ4plex); K5(iTRAQ4plex); S9(P
kQEtPDEVGSQR	F1LMV8	N-Term(iTRAQ4plex); K1(iTRAQ4plex); T4(P
sPTQLGk	B5DEI5	N-Term(iTRAQ4plex); S1(Phospho); K7(iTRA
lDLIsEGEEPR	O54698	N-Term(iTRAQ4plex); S5(Phospho)
sPHLPAPTGLFDDDDNDs	Q80X08	N-Term(iTRAQ4plex); S18(Phospho); K31(iT
skGDSDVsDEEAAPQSk	E9PSN4	N-Term(iTRAQ4plex); K2(iTRAQ4plex); S8(P
lAGLGPEDDPDtDDEQEP	D3ZBC7	N-Term(iTRAQ4plex); T12(Phospho)
gNTSSsPPGPR	Q66HR5	N-Term(iTRAQ4plex); S6(Phospho)
mEsPTkEIEEFESNSLk	A0A0G2KAI1	N-Term(Acetyl); S3(Phospho); K6(iTRAQ4ple
sTLNPQWNEsFTFk	F1M2P8	N-Term(iTRAQ4plex); S10(Phospho); K14(iT
lEGNsPQGsSHGVk	Q5M970	N-Term(iTRAQ4plex); S5(Phospho); S9(Phos
lEETSkVsIEETVAkEEESL	A0A0U1RRX4;F1MAQ5	N-Term(iTRAQ4plex); K6(iTRAQ4plex); S8(P
sEsPkEPEQLR	D4ACJ7	N-Term(iTRAQ4plex); S3(Phospho); K5(iTRA
sAAAAPAAEGEDtPtPPAS	Q6AXS3	N-Term(Acetyl); T13(Phospho); T15(Phospho
sGDGEsTPVEHVR	A0A0G2K472	N-Term(iTRAQ4plex); S6(Phospho)
sAsPAPADVAPAQEDLR	D3ZYS7	N-Term(iTRAQ4plex); S3(Phospho)
qTPPASPSPQPAEDRPPS	A0A1B0GWS4	N-Term(iTRAQ4plex); S19(Phospho); K29(iT
vPsPLtDEEGDFILIHHEDL	Q5FVK6	N-Term(iTRAQ4plex); S3(Phospho); T6(Phos
tILYFDEEPELEMESLTDs	Q9JKS6;D3Z9C7	N-Term(iTRAQ4plex); S19(Phospho)
rRPGPSPDGLLEEGsPTM	A0A0G2K5K1	N-Term(iTRAQ4plex); S15(Phospho); K21(iT
tLSVAAAFNEDEDsEPEEI	A0A0H2UHW4	N-Term(iTRAQ4plex); S14(Phospho); K24(iT

aEDsSEGHEEEVLGGHG	Q63259	N-Term(iTRAQ4plex); S4(Phospho); K19(iTR
tEkPskSPPPPPPR	Q9QXY2	N-Term(iTRAQ4plex); K3(iTRAQ4plex); S5(P
dLLHPsPEEEk	M0RA26	N-Term(iTRAQ4plex); S6(Phospho); K11(iTR
dSVPSDsPPITVQPQANT	D3ZVU6	N-Term(iTRAQ4plex); S7(Phospho)
kVELsEsEEDkGSk	B2RYB3;A0A0G2K4F6	N-Term(iTRAQ4plex); K1(iTRAQ4plex); S5(P
sYsPDGk	A0A0G2JSR7	N-Term(iTRAQ4plex); S3(Phospho); K7(iTRA
nSNsDGWESWEGASGE(	Q3S4A5	N-Term(iTRAQ4plex); S4(Phospho)
lkIsPEQHWDFTAEDLkDL	F1LP57	N-Term(iTRAQ4plex); K2(iTRAQ4plex); S4(P
aATATRPPGPPPAPQPPS	D3ZH75	N-Term(iTRAQ4plex); S22(Phospho)
dTDAYSDLsDGEk	Q568Z1	N-Term(iTRAQ4plex); S9(Phospho); K13(iTR
tITLEVEPSDtIENVk	P0CG51	N-Term(iTRAQ4plex); T11(Phospho); K16(iT
eVAkEsPkEEk	P12839	N-Term(iTRAQ4plex); K4(iTRAQ4plex); S6(P
fTGYQTIQQQSSSETEGE	Q923W4	N-Term(iTRAQ4plex); S26(Phospho); S27(Ph
tVTVIsPEDEQkGEk	D4A3C2	N-Term(iTRAQ4plex); S6(Phospho); K12(iTR
rGsPEEATER	Q02294	N-Term(iTRAQ4plex); S3(Phospho)
lSWGTENLDNVALSSsPIH	A0A0G2JZ56	N-Term(iTRAQ4plex); S16(Phospho)
nSsPDEVLVSNMDTSR	Q9JHE0	N-Term(iTRAQ4plex); S3(Phospho)
sGsRPQSPsGDAESR	D4A5J1	N-Term(iTRAQ4plex); S3(Phospho); S9(Phos
sRDsGDENEPIQER	P0C5E3	N-Term(iTRAQ4plex); S4(Phospho)
gEEEEEEEEDEPVPEAPs	D4A507	N-Term(iTRAQ4plex); S18(Phospho)
qTESNPAVSNsDEEGNET	A0A0G2JXZ3	N-Term(iTRAQ4plex); S11(Phospho); K20(iT
tFAPLPEFEQEEDDGEVT	D3ZUM5	N-Term(iTRAQ4plex); S21(Phospho)
sGDEtPGsEAPGDk	D4ABT8	N-Term(iTRAQ4plex); T5(Phospho); S8(Phos
eGGGDSSASSPTEEEQE	A0A0G2K613	N-Term(iTRAQ4plex); C24(Carbamidomethyl
sPSGFSADEAESsPPLPG	Q1EG89	N-Term(iTRAQ4plex); S13(Phospho); K36(iT
sVSTTNIAGHFNDEsPLGI	A0A1B0GWY5	N-Term(iTRAQ4plex); S15(Phospho)
ttSPDLFESQSLTSASSkP	F1LQ45	N-Term(iTRAQ4plex); T2(Phospho); K18(iTR
vQIQLENLEsSVRDR	D3ZDX5	N-Term(iTRAQ4plex); S10(Phospho)
lENEGsDEDIETDVLYSPQ	Q8VIJ5	N-Term(iTRAQ4plex); S6(Phospho); M20(Ox
sLDSsGAGEk	D3ZT71	N-Term(iTRAQ4plex); S5(Phospho); K10(iTR
sDVsDQEEDEESER	G3V7E6	N-Term(iTRAQ4plex); S4(Phospho)
scSsPVFSR	D4ACG7	N-Term(iTRAQ4plex); C2(Carbamidomethyl);
kGATPAEDDEDNDIDLFG	Q68FR9	N-Term(iTRAQ4plex); K1(iTRAQ4plex); S19(
ySTLLsPSEQEk	Q5FVC7	N-Term(iTRAQ4plex); S6(Phospho); K12(iTR
sksEsPkEPEQLR	D4ACJ7	N-Term(Acetyl); K2(iTRAQ4plex); S3(Phosph
tAsPPPPPk	B2RYB3;A0A0G2K4F6	N-Term(iTRAQ4plex); S3(Phospho); K9(iTRA
vFVcDQLEsPTGFSLGSAI	D4A626	N-Term(iTRAQ4plex); C4(Carbamidomethyl);
sPAEVksPAEVksPAEAk	F1LRZ7	N-Term(iTRAQ4plex); K6(iTRAQ4plex); S7(P
gVVTNGLDVsPAEEk	P12839	N-Term(iTRAQ4plex); S10(Phospho); K15(iT
vRDTsQEPcGR	A0A0G2K1R9;A0A0G2K1(	N-Term(iTRAQ4plex); S5(Phospho); C9(Carb
sVYSDRDDEsPDssPEEQ	A0A0G2K6R9;F1M9N9	N-Term(iTRAQ4plex); S10(Phospho); S13(Ph
sFGNPFEPQAR	F1LQD2	N-Term(iTRAQ4plex); S1(Phospho)
hSLDsDEEDDDEGSSk	B4F786	N-Term(iTRAQ4plex); S5(Phospho); K16(iTR
dksPSEGDVtPPkR	D3ZBU7	N-Term(iTRAQ4plex); K2(iTRAQ4plex); S3(P
mEsPEGAGPGEITk	Q5M831	N-Term(Acetyl); S3(Phospho); K14(iTRAQ4p
nLsITSPPPGPAk	F1M9F9	N-Term(iTRAQ4plex); S3(Phospho); K13(iTR
sLSESYELSSDLQDk	A0A0G2JUG7	N-Term(iTRAQ4plex); S1(Phospho); K15(iTR
hSsLPTEsDEDIAPAQR	B5DFK6	N-Term(iTRAQ4plex); S3(Phospho); S8(Phos
hLsNVsSTGSIDMVDSPQ	D4A1Q2	N-Term(iTRAQ4plex); S3(Phospho); S6(Phos
eERPGAAGGGAsAPGSP	D3ZE55	N-Term(iTRAQ4plex); S12(Phospho)
asGDYDNDcTNPITPLcTQ	D3Z8C7	N-Term(iTRAQ4plex); S2(Phospho); C9(Carb
lcDFGSASHVADNDITPyL	Q5RKH1	N-Term(iTRAQ4plex); C2(Carbamidomethyl);
lSPWEDLSQAPTmDsPLE	F1LR33	N-Term(iTRAQ4plex); M13(Oxidation); S15(P
yFDEEFTAQMITItPPDQD	P47196	N-Term(iTRAQ4plex); T14(Phospho); C24(Ca
nVEsGEDELASR	G3V9N1	N-Term(iTRAQ4plex); S4(Phospho)

rsTPTPELTSk	F1M1Y0	N-Term(iTRAQ4plex); S2(Phospho); K11(iTR
gTGDcsDEEVDGkADGAD	G3V6P7	N-Term(iTRAQ4plex); C5(Carbamidomethyl);
kVAsVEEGDESLDSPGSQ	D4AAX6	N-Term(iTRAQ4plex); K1(iTRAQ4plex); S4(P
kAPLVASDSSSSGGsDSE	A0A0G2JV49	N-Term(iTRAQ4plex); K1(iTRAQ4plex); S15(
sPSSsTGSIASSR	M0R5P8	N-Term(iTRAQ4plex); S5(Phospho)
gDLSQHATPLPTPAVLPG	Q5XI21	N-Term(iTRAQ4plex); S20(Phospho); K31(iT
aTVTPsPVk	A0A0G2K7X3	N-Term(iTRAQ4plex); S6(Phospho); K9(iTRA
ssPEIAPSDVTDESEVVTA	Q5JC29	N-Term(iTRAQ4plex); S2(Phospho); K24(iTR
iPEISIQDMTAQVTsPSGk	C0JPT7	N-Term(iTRAQ4plex); S15(Phospho); K19(iT
mkPLSAsQVsVHVGMDDI	Q64535	N-Term(iTRAQ4plex); K2(iTRAQ4plex); S7(P
isGAPDkYESR	G3V9B3	N-Term(iTRAQ4plex); S2(Phospho); K7(iTRA
kEVQsPEQVk	B1WC16	N-Term(iTRAQ4plex); K1(iTRAQ4plex); S5(P
gGSLGcQTETDSDtQSPP	Q9JKS6;D3Z9C7	N-Term(iTRAQ4plex); C6(Carbamidomethyl);
tAsFGGITVLTR	D3ZQR3	N-Term(iTRAQ4plex); S3(Phospho)
rPGPSPDGLLEEGsPTMV	A0A0G2K5K1	N-Term(iTRAQ4plex); S14(Phospho); K20(iT
sDDSkSssPEPVTHLk	D4A6C5	N-Term(iTRAQ4plex); K5(iTRAQ4plex); S7(P
wLDDLLAsPPPNSGGAR	D3ZF26	N-Term(iTRAQ4plex); S8(Phospho)
mSISEGTVsDk	P15205;F1LRL9	N-Term(iTRAQ4plex); S9(Phospho); K11(iTR
eFDsDSLR	A0A0G2K1R9;A0A0G2K1(	N-Term(iTRAQ4plex); S4(Phospho)
rDEQsEsDHMDQETVSR	D3ZKH2	N-Term(iTRAQ4plex); S5(Phospho); S7(Phos
sPVEAksPAEVk	F1LRZ7	N-Term(iTRAQ4plex); K6(iTRAQ4plex); S7(P
sLsPIIGk	A0A0G2K0F3	N-Term(iTRAQ4plex); S3(Phospho); K8(iTRA
tSskEsSPVPSPTSDR	G3V6S0	N-Term(iTRAQ4plex); S3(Phospho); K4(iTRA
sGSALLQSQSsTEDAkDE	D3ZYQ9	N-Term(iTRAQ4plex); S11(Phospho); K16(iT
rRssEVEIFVDcEcGk	Q8CGU9	N-Term(iTRAQ4plex); S3(Phospho); S4(Phos
sASSDtsEELNAQDSPk	Q9JJ19	N-Term(iTRAQ4plex); T6(Phospho); S7(Phos
eTDPGsPR	Q3KRF2	N-Term(iTRAQ4plex); S6(Phospho)
nLAkPGVtSTSDsEEDDD(	Q566D6	N-Term(iTRAQ4plex); K4(iTRAQ4plex); T8(P
kEsPPTPDQGAASR	D4A280	N-Term(iTRAQ4plex); K1(iTRAQ4plex); S3(P
qLTAcPELPHNVQEILGLA	B1H264	N-Term(iTRAQ4plex); C5(Carbamidomethyl);
qGRPsPtSPVkPSSPASkP	Q9QYV3	N-Term(iTRAQ4plex); S5(Phospho); T7(Phos
tPsLPTPPTR	D4A1Q2	N-Term(iTRAQ4plex); S3(Phospho)
eTAEADVAsLNR	A0A0G2JSQ4	N-Term(iTRAQ4plex); S9(Phospho)
sQsESsDEVTELDLSHGk	D4A5B3	N-Term(iTRAQ4plex); S3(Phospho); S6(Phos
gTGLQPGEEELPDIAPPLY	D3ZVQ0	N-Term(iTRAQ4plex); T20(Phospho); K25(iT
rLEDEQPPTLsPk	A0A0G2K104	N-Term(iTRAQ4plex); S11(Phospho); K13(iT
tSsTGEEEESLAILR	A0A0G2JU75	N-Term(iTRAQ4plex); S3(Phospho)
dSsPPTEQR	A0A140TAD1	N-Term(iTRAQ4plex); S3(Phospho)
vEQALSEVAssLQsSAPk	F1LUY5	N-Term(iTRAQ4plex); S10(Phospho); S11(Ph
nSDLFTVLsR	C0IXW5	N-Term(iTRAQ4plex); S9(Phospho)
sFGsPNR	F1LQC8	N-Term(iTRAQ4plex); S4(Phospho)
qDEMDEsDDDLDDkPSPI	A0A0G2K451	N-Term(iTRAQ4plex); S7(Phospho); K14(iTR
yAALsLsETSLTEk	D3ZTF0	N-Term(iTRAQ4plex); S5(Phospho); S7(Phos
tcDSPQNPmDLISDPVPDS	Q3KR59	N-Term(iTRAQ4plex); T1(Phospho); C2(Carb
iEkLPsISDLDSIFGPVLsPI	F1M1Y0	N-Term(iTRAQ4plex); K3(iTRAQ4plex); S6(P
aQSVsPVQATPSGGSAQ	D4A853	N-Term(iTRAQ4plex); S5(Phospho); K22(iTR
kQEAVQEVsFDEEPGPTQ	D3ZL30	N-Term(iTRAQ4plex); K1(iTRAQ4plex); S9(P
qEQSsTEIPLQAESDQAA	Q5QD51	N-Term(iTRAQ4plex); S5(Phospho); K23(iTR
sLmssPEDLTkDFEELk	P15205;F1LRL9	N-Term(iTRAQ4plex); M3(Oxidation); S4(Pho
iAELEEERsQGSTSNSDW	F1LRL9	N-Term(iTRAQ4plex); S9(Phospho); K20(iTR
tPPSNLSPIEDAsPTEELR	Q9JKS6;G3V984;D3Z9C7	N-Term(iTRAQ4plex); S13(Phospho)
aTHPPSStPPNPLLNR	G3V6K8	N-Term(iTRAQ4plex); T8(Phospho)
fSASEASFLEGEAsPPLGA	Q810W7	N-Term(iTRAQ4plex); S14(Phospho)
gQGTAsPGsVSDLAQTVk	M0R5U4	N-Term(iTRAQ4plex); S6(Phospho); S9(Phos
iDVFsPVEFNk	F1M4I4	N-Term(iTRAQ4plex); S5(Phospho); K11(iTR

aksPVkEGAk	F1LRZ7	N-Term(iTRAQ4plex); K2(iTRAQ4plex); S3(P
tVEGTEPkPQGVIRsPQGI	A0A0G2K6R9;F1M9N9	N-Term(iTRAQ4plex); K8(iTRAQ4plex); S15(
eHQASQEAQQQQQHQG	D3ZE26	N-Term(iTRAQ4plex); S22(Phospho); K26(iT
lsTAITLLPLEEGR	D3ZNS1	N-Term(iTRAQ4plex); S2(Phospho)
rFQSVPAQPGQTsPLLQY	F1M779	N-Term(iTRAQ4plex); S13(Phospho); K30(iT
iHVsDQELQSANASVDDS	Q5U300	N-Term(iTRAQ4plex); S4(Phospho); K24(iTR
lLSAsQk	P10888	N-Term(iTRAQ4plex); S5(Phospho); K7(iTRA
lkEEIAEVANEIESLGStEE	B2GUV0	N-Term(iTRAQ4plex); K2(iTRAQ4plex); T18(
sLsDPkPLSPTAEESAk	G3V984	N-Term(iTRAQ4plex); S3(Phospho); K6(iTRA
eLQAAGksPEELEk	Q6P6V0	N-Term(iTRAQ4plex); K7(iTRAQ4plex); S8(P
rssSELSPEIVEk	A0A0G2K2M9	N-Term(iTRAQ4plex); S2(Phospho); S3(Phos
yLTAsQLVPPDNQDTR	A0A140TAJ5	N-Term(iTRAQ4plex); S5(Phospho)
qSVMNsPTATMNNTHSN	G3V746	N-Term(iTRAQ4plex); S6(Phospho)
hRDtGILDSIGR	P02688	N-Term(iTRAQ4plex); T4(Phospho)
gPLtSGSDEDNVALPLGD	A0A096MIY2	N-Term(iTRAQ4plex); T4(Phospho); C33(Car
tTFSENLsPk	F1LVK0	N-Term(iTRAQ4plex); S8(Phospho); K10(iTR
sQGsTSNsDWMk	F1LRL9	N-Term(iTRAQ4plex); S4(Phospho); S8(Phos
aELVVFkGLmsDPmtDLDt	P0C6R4	N-Term(iTRAQ4plex); K7(iTRAQ4plex); M10(
lDLTGtSGAAVPAR	D3ZC55	N-Term(iTRAQ4plex); T6(Phospho)
eALNIIGDISTSTVSTPVPI	A0A0A0MY48	N-Term(iTRAQ4plex); S32(Phospho)
mVSSEGsTESEHLDGME	F1M8A4	N-Term(iTRAQ4plex); S7(Phospho); K21(iTR
sLPELDRDksDSETEGLVI	A0A0G2K0F3	N-Term(iTRAQ4plex); K9(iTRAQ4plex); S10(
tQsSGQDGRPQEELHAEI	F1M8H6	N-Term(iTRAQ4plex); S3(Phospho); K21(iTR
gPPDFssDEEREPTPVLG	Q62733	N-Term(iTRAQ4plex); S6(Phospho); S7(Phos
sDSRsESPEPGyVVTSSG	P0C5Y8	N-Term(iTRAQ4plex); S5(Phospho); Y12(Pho
vGEsFEETLEETVVSTk	P23565;G3V8Q2	N-Term(iTRAQ4plex); S4(Phospho); K17(iTR
hLsNVSSTGSIDMVDSPQ	D4A1Q2	N-Term(iTRAQ4plex); S3(Phospho); K32(iTR
mASPPPSGPPSAAHTPF	P34926;Q63330	N-Term(iTRAQ4plex); M1(Oxidation); S20(Ph
vNHEPEPASGAsPGATIPI	Q5HZA7	N-Term(iTRAQ4plex); S12(Phospho); K19(iT
vDNSsLTGESEPQTR	P06686;P06687;P06685;F	N-Term(iTRAQ4plex); S5(Phospho)
nDGVkEssEstNtTIEDEDT	P11275	N-Term(iTRAQ4plex); K5(iTRAQ4plex); S7(P
sLELNGAGEPVEGAPR	F1M6V8	N-Term(iTRAQ4plex); S1(Phospho)
hIPAALPVSGTLSsSNPDL	F1M386	N-Term(iTRAQ4plex); S14(Phospho)
tDDTsGEDNDEkEAVASk	A0A0G2JU91	N-Term(iTRAQ4plex); S5(Phospho); K12(iTR
eSSDStNttIEDEDAk	G3V9G3	N-Term(iTRAQ4plex); T6(Phospho); T8(Phos
nLLEDDsDEEEDFFLR	A0A096MJ99	N-Term(iTRAQ4plex); S7(Phospho)
tsPEPQREDAsPAPGTTAI	A0A0G2K7C1	N-Term(iTRAQ4plex); S2(Phospho); S11(Pho
tASGSsVTSLEGTR	Q6JE36	N-Term(iTRAQ4plex); S6(Phospho)
sASEDSIR	A0A0G2K207	N-Term(iTRAQ4plex); S1(Phospho)
aATAQQsPPSVGGk	A0A0G2JZB6	N-Term(Acetyl); S7(Phospho); K14(iTRAQ4p
kAEDsDsEPEPEDNVR	D4A914	K1(iTRAQ4plex); N-Term(iTRAQ4plex); S5(P
gLMAGGRPEGQYsEDED	B1H241	N-Term(iTRAQ4plex); S13(Phospho)
lHRGAPVNVssSDLTGR	Q06486	N-Term(iTRAQ4plex); S10(Phospho); S11(Ph
aLPGHLkPFETLLsQNQG	P04906	N-Term(iTRAQ4plex); K7(iTRAQ4plex); S14(
ickPLHELIMQLLEEtPEEk	O35264	N-Term(iTRAQ4plex); C2(Carbamidomethyl);
lEPDQVsPR	F1M3G7	N-Term(iTRAQ4plex); S7(Phospho)
rQVDEGVWPPPNNLLNQ	Q9QXY2	N-Term(iTRAQ4plex); S18(Phospho); K20(iT
sAssGAEGDVSSEREP	B0K014	N-Term(iTRAQ4plex); S3(Phospho); S4(Phos
qGAIVAVTGDGVNDsPAL	P06686;P06687;P06685	N-Term(iTRAQ4plex); S15(Phospho); K19(iT
eSDETVTVALsPk	E9PTE1	N-Term(iTRAQ4plex); S11(Phospho); K13(iT
iNVYYNEAtGGk	G3V7C6	N-Term(iTRAQ4plex); T9(Phospho); K12(iTR
sTSATDTHHVELAR	A0A0H2UHR5	N-Term(iTRAQ4plex); S1(Phospho)
iAcDEEFsDSEDEGEGGR	Q99PA1	N-Term(iTRAQ4plex); C3(Carbamidomethyl);
asLkDLEGk	P55053	N-Term(Acetyl); S2(Phospho); K4(iTRAQ4ple
tQESTNVPsDQEGTSk	G3V7E6	N-Term(iTRAQ4plex); S9(Phospho); K16(iTR

Information Classification: General

gsTEScNTTTEDEDLk	P11730;F1M3F8	N-Term(iTRAQ4plex); S2(Phospho); C6(Carb
gtQksIIIHTSEDGk	P20272	N-Term(iTRAQ4plex); T2(Phospho); K4(iTRA
gsPTRPNPPVR	Q62952	N-Term(iTRAQ4plex); S2(Phospho)
kkGsISDGTNILDEVTFPA	D4ABB2	N-Term(iTRAQ4plex); K1(iTRAQ4plex); K2(iT
kLEAAEDIAyQLSR	P67779	N-Term(iTRAQ4plex); K1(iTRAQ4plex); Y10(
hRPsSPAtPPPk	B2RYB3;A0A0G2K4F6	N-Term(iTRAQ4plex); S4(Phospho); T8(Phos
eISssPTSk	A0A0G2K2M9	N-Term(iTRAQ4plex); S4(Phospho); S5(Phos
vEsEEsGDEEGkk	Q5HZV9	N-Term(iTRAQ4plex); S3(Phospho); S6(Phos
aEsAEsGGPPGAQDSAAI	A0A140TAI1	N-Term(iTRAQ4plex); S3(Phospho); S6(Phos
aEDDGGEsEGDASEk	D4A997	N-Term(iTRAQ4plex); S8(Phospho); K15(iTR
dDPSVINIsDEMSk	D4AD03	N-Term(iTRAQ4plex); S9(Phospho); K14(iTR
skEsLQEAGk	P53987	N-Term(iTRAQ4plex); K2(iTRAQ4plex); S4(P
dYDEEEQGYDsEkEk	A0A0G2QC38	N-Term(iTRAQ4plex); S11(Phospho); K13(iT
sGMsPEQNk	A0A0G2K2M9	N-Term(iTRAQ4plex); S4(Phospho); K9(iTRA
sTSPPPsPEVWAESRTLG	D4A559	N-Term(iTRAQ4plex); S7(Phospho)
sPFEIISPPAsPPEmTGQR	P34926	N-Term(iTRAQ4plex); S11(Phospho); M15(O
aPsPAPAVsPk	Q63624	N-Term(iTRAQ4plex); S3(Phospho); S9(Phos
rDDEcPSTsSPVSFPGSD(	M0R851	N-Term(iTRAQ4plex); C5(Carbamidomethyl);
gPGLGsTQGQTIALPAQG	P86252	N-Term(iTRAQ4plex); S6(Phospho)
lGGsAESLIPGFQATQGD/	P0C1X8	N-Term(iTRAQ4plex); S4(Phospho); K32(iTR
mDSAVQASLsLPATPVGk	Q78PB6	N-Term(iTRAQ4plex); S10(Phospho); K18(iT
qFLSETEkLsPEDR	Q00981	N-Term(iTRAQ4plex); K8(iTRAQ4plex); S10(
eDsmDmDMSPLRPQNYL	P13084	N-Term(iTRAQ4plex); S3(Phospho); M4(Oxid
lELSEDsPNSEQELDk	M0R5H1	N-Term(iTRAQ4plex); S7(Phospho); K16(iTR
yGDILHVINAsDDEWWQ/	Q63622	N-Term(iTRAQ4plex); S11(Phospho)
lSPAksPSLSPSPPsPIEk	P15205;F1LRL9	N-Term(iTRAQ4plex); K5(iTRAQ4plex); S6(P
akPAsPAR	P34926	N-Term(iTRAQ4plex); K2(iTRAQ4plex); S5(P
sSsPVNVkk	Q63555	N-Term(Acetyl); S3(Phospho); K8(iTRAQ4ple
eEVAsEPEEAASPITPk	Q5RJN5	N-Term(iTRAQ4plex); S5(Phospho); K17(iTR
eSVGGsPQSk	D4A559	N-Term(iTRAQ4plex); S6(Phospho); K10(iTR
qIDsSPVGGETDEMTR	D3ZN76	N-Term(iTRAQ4plex); S4(Phospho)
yGTDNsEETk	D3ZVB7	N-Term(iTRAQ4plex); S6(Phospho); K10(iTR
ePkIDEtYVLtPSVk	A0A096MJ77	N-Term(iTRAQ4plex); K3(iTRAQ4plex); T7(P
kPGNNEGsGAPsPLSk	Q05140	N-Term(iTRAQ4plex); K1(iTRAQ4plex); S8(P
vDYLTPDFPSLsYPNYYTI	D3ZRI1	N-Term(iTRAQ4plex); S12(Phospho)
mDsYEQEEDIDQIVAEVk	O35430	N-Term(iTRAQ4plex); S3(Phospho); K18(iTR
sSLsSAQADFNQLAELDR	P16086	N-Term(iTRAQ4plex); S4(Phospho)
qMsFDLTk	A0A0H4SRI7	N-Term(iTRAQ4plex); S3(Phospho); K8(iTRA
qITQEDDDsDEEVAPENF	B2RYA6	N-Term(iTRAQ4plex); S9(Phospho); K24(iTR
tSESTSPPVVPPLHsPk	Q63430	N-Term(Acetyl); S15(Phospho); K17(iTRAQ4
lAAGAESPQPASGNsPSE	F1LMV8	N-Term(iTRAQ4plex); S15(Phospho); K23(iT
sPVFSFSPEPGVGDEAR	B2DD29	N-Term(iTRAQ4plex); S1(Phospho)
etSPISSHPVTWQPSk	A0A0G2KAV8	N-Term(iTRAQ4plex); T2(Phospho); K16(iTR
qAsTDAGTAGALTPQHVF	R9PXS9	N-Term(iTRAQ4plex); S3(Phospho)
qSSLAEPVsPSk	Q66HA0	N-Term(iTRAQ4plex); S9(Phospho); K12(iTR
fFTTGsDsESESSLSGEEL	B5DFC8	N-Term(iTRAQ4plex); S6(Phospho); S8(Phos
kSLsDsESDDSk	Q5RJK5	N-Term(iTRAQ4plex); K1(iTRAQ4plex); S4(P
gMYDGPVFDLTTtPk	Q62952	N-Term(iTRAQ4plex); T13(Phospho); K15(iT
lkEGLDISHLQGQEELLsS	Q9R1N0	N-Term(iTRAQ4plex); K2(iTRAQ4plex); S18(
gLATGDsPLGPMTHHGEI	O88794	N-Term(iTRAQ4plex); S7(Phospho)
vDHGAEIITQSPsR	A0A0U1RRX4;F1MAQ5	N-Term(iTRAQ4plex); S13(Phospho)
gGsQGEEVGELPR	P13233	N-Term(iTRAQ4plex); S3(Phospho)
sSPVPRsPDREDGEEAP/	G3V9G5	N-Term(iTRAQ4plex); S1(Phospho); S7(Phos
aEEAtPVAALR	G3V874;A0A0G2K1Q9	N-Term(iTRAQ4plex); T5(Phospho)
sSTVsEDVEATVPMLQR	Q4V8I7	N-Term(iTRAQ4plex); S5(Phospho)

Information Classification: General

gIDPPQVLSPDMVPPsER	A0A0G2QC53	N-Term(iTRAQ4plex); S16(Phospho); K38(iT
eEcPRPmsIsPPDFSPk	P15205;F1LRL9	N-Term(iTRAQ4plex); C3(Carbamidomethyl);
sLEGGGcPVR	A0A0G2JZ38	N-Term(iTRAQ4plex); S1(Phospho); C7(Carb
sASsGAEGDVSSER	B0K014	N-Term(iTRAQ4plex); S4(Phospho)
eHGPFRsVEDLVR	Q5XI74	N-Term(iTRAQ4plex); S7(Phospho)
nVMSAFGLTDDQVsGPPS	Q4R1A4	N-Term(iTRAQ4plex); S14(Phospho)
wDEQTSNTkGDDDEEsD	A0A0G2JTI7	N-Term(iTRAQ4plex); K9(iTRAQ4plex); S16(
tPDAsPEPk	A0A0G2JTW5	N-Term(iTRAQ4plex); S5(Phospho); K9(iTRA
lkGQEDNLASAVDATTGQ	D4A4Z0	N-Term(iTRAQ4plex); K2(iTRAQ4plex); C21(
aEETGMSSEDDGLPRsPI	D3ZBU7	N-Term(iTRAQ4plex); S16(Phospho); K33(iT
tsFQGEk	B1H238	N-Term(iTRAQ4plex); S2(Phospho); K7(iTRA
kPsDEEFAVR	Q923I5	N-Term(iTRAQ4plex); K1(iTRAQ4plex); S3(P
sRPWSPSASYDLR	G3V8P8	N-Term(iTRAQ4plex); S1(Phospho)
lSPAksPsLsPSPPsPIEk	P15205;F1LRL9	N-Term(iTRAQ4plex); K5(iTRAQ4plex); S6(P
vGLLssPNQASSPGGTVV	Q5BJW6	N-Term(iTRAQ4plex); S5(Phospho); S6(Phos
mAPTPIPtRSPSDSSTAST	Q07266;A0A0H2UHL9	N-Term(iTRAQ4plex); T8(Phospho)
sSSVtSIDk	Q9WU70	N-Term(iTRAQ4plex); T5(Phospho); K9(iTRA
rGsMNNEMLSPEVGPVRI	Q5U2N3	N-Term(iTRAQ4plex); S3(Phospho)
nsLPQIPTLNLESR	P70600	N-Term(iTRAQ4plex); S2(Phospho)
sPsPGRLEEDGDELAR	Q3B7D0	N-Term(iTRAQ4plex); S3(Phospho)
qIFGDyk	P60203	N-Term(iTRAQ4plex); Y6(Phospho); K7(iTRA
eADPGETsSEAPSETR	F1M084	N-Term(iTRAQ4plex); S8(Phospho)
qINQEEFkGFsYFGEDLm	F1LMV8	N-Term(iTRAQ4plex); K8(iTRAQ4plex); S11(
gLsATVTGGQk	P60203	N-Term(iTRAQ4plex); S3(Phospho); K11(iTR
eSDDkPEIEDVGsDEEEE	P82995	N-Term(iTRAQ4plex); K5(iTRAQ4plex); S13(
aDPTVQLAPsPPksPk	Q9JKS6;D3Z9C7	N-Term(iTRAQ4plex); S10(Phospho); K13(iT
qGsGVILR	Q63768	N-Term(iTRAQ4plex); S3(Phospho)
rVAGGSGSEsPLLEGR	B2GV22	N-Term(iTRAQ4plex); S10(Phospho)
tGGVLSLIQcTLIEEPDTsD	Q99JE4	N-Term(iTRAQ4plex); C10(Carbamidomethyl
nkPSAtDsEGEDDEDMTk	Q6MG08	N-Term(iTRAQ4plex); K2(iTRAQ4plex); T6(P
sPsPQFAPQk	Q7TP36	N-Term(iTRAQ4plex); S3(Phospho); K10(iTR
gmYDGPVFDLTTtPk	Q62952	N-Term(iTRAQ4plex); M2(Oxidation); T13(Ph
vGHDsELENQDk	A0A173DW30	N-Term(iTRAQ4plex); S5(Phospho); K12(iTR
sHsPTHTPTPETkPPTEDI	D3ZWG2	N-Term(iTRAQ4plex); S3(Phospho); K13(iTR
lPEEPSsEDEQQVEk	D3ZV52	N-Term(iTRAQ4plex); S7(Phospho); K15(iTR
lADLYGskDTFDDDS	F1MAH6	N-Term(iTRAQ4plex); S7(Phospho); K8(iTRA
tEPAsEEQEPAEDTDQAR	Q5QD51	N-Term(iTRAQ4plex); S5(Phospho)
fSGEEGEIEDDEsGTENR	Q5M7V8	N-Term(iTRAQ4plex); S13(Phospho); K21(iT
mAGPPAPPGGsPR	A0A0G2JXK1	N-Term(iTRAQ4plex); S11(Phospho)
aEEEGGsDGsAAEAEPR	Q4G061	N-Term(iTRAQ4plex); S7(Phospho); S10(Pho
sTIEEITTSSsQk	P23565	N-Term(iTRAQ4plex); S11(Phospho); K13(iT
lQESFQGAPDVIsPR	A0A096MKD9	N-Term(iTRAQ4plex); S13(Phospho)
vLGPPVQLAVDDSsDEGE	A0A0G2JXS2	N-Term(iTRAQ4plex); S14(Phospho); K22(iT
qTPQAPQsPR	F1MA98	N-Term(iTRAQ4plex); S8(Phospho)
aAPPPPPPPPPLESsPR	A0A0G2JU89	N-Term(iTRAQ4plex); S15(Phospho)
eMDMsPEEVVPSPR	Q66H79	N-Term(iTRAQ4plex); S5(Phospho)
mSYLTAMGADYLscDSR	Q3ZB99	N-Term(iTRAQ4plex); S13(Phospho); C14(Ca
eLNFVTDSVDQELPSsPk	A0A0G2K779	N-Term(iTRAQ4plex); S16(Phospho); K18(iT
gSDsIAYDk	A0A0G2JSM7;D3ZZ99	N-Term(iTRAQ4plex); S4(Phospho); K9(iTRA
eAAGTDIENsPQHk	A4L691	N-Term(iTRAQ4plex); S10(Phospho); K14(iT
aFWMAIGGDRDEIEGLSs	Q9R0Z7	N-Term(iTRAQ4plex); S18(Phospho)
eTEVSkGsAEsPDEGITTT	P15205;F1LRL9	N-Term(iTRAQ4plex); K6(iTRAQ4plex); S8(P
aDLPVItIDPASPQSPESVI	P26431	N-Term(iTRAQ4plex); T7(Phospho); K27(iTR
hSHPGLGTSQsPk	O70150	N-Term(iTRAQ4plex); S11(Phospho); K13(iT
lSLGsPESR	D4AAS1	N-Term(iTRAQ4plex); S5(Phospho)

lGIHEDsQNR	P82995	N-Term(iTRAQ4plex); S7(Phospho)
mSQPGsPSPk	E9PU01	N-Term(iTRAQ4plex); S6(Phospho); K10(iTR
nDGVkESSEstNtTIEDEDT	P11275	N-Term(iTRAQ4plex); K5(iTRAQ4plex); S10(
rGsPGGVEMNVELPQQE	Q66H43	N-Term(iTRAQ4plex); S3(Phospho)
mEEPLGsPPAALSALEk	A0A0G2K2U5	N-Term(Acetyl); S7(Phospho); K17(iTRAQ4p
lNINSsPDEHEPLLR	F1LQC5	N-Term(iTRAQ4plex); S6(Phospho)
eEGtGEsAGGSPARsLTR	B5DF41	N-Term(iTRAQ4plex); T4(Phospho); S7(Phos
vPsPLTDEEGDFILIHHED	Q5FVK6	N-Term(iTRAQ4plex); S3(Phospho)
ssSPNNTNPFGSTFcFGL	A0A0G2JWC6	N-Term(iTRAQ4plex); S2(Phospho); C15(Ca
dLsLEEIQk	P21818;A0A096MK73	N-Term(iTRAQ4plex); S3(Phospho); K9(iTRA
tQADPEDIAAHSGREELss	Q5D022	N-Term(iTRAQ4plex); S18(Phospho); S19(Ph
eIQGDGPPSsPTNDPTVk	F1LPP8	N-Term(iTRAQ4plex); S10(Phospho); K18(iT
eRPPFIDLETFNAGsDAG	Q5U1X7	N-Term(iTRAQ4plex); S15(Phospho); S19(Ph
rAsPsPPPkR	B2RYB3;A0A0G2K4F6	N-Term(iTRAQ4plex); S3(Phospho); S5(Phos
aGAYDFPsPEWDTVTPE	P11275;G3V9G3;F1LNI8;	N-Term(iTRAQ4plex); S8(Phospho); K19(iTR
nLTSSsLNDISDkPEk	Q9JI66;A0A0H2UHB7	N-Term(iTRAQ4plex); S6(Phospho); K13(iTR
lITTDsDGTYk	D3Z9D0	N-Term(iTRAQ4plex); S6(Phospho); K11(iTR
rTEsVPSDINNPVDR	D3ZHL1	N-Term(iTRAQ4plex); S4(Phospho)
sATsMSGAEQDSPGTDk	F7FD99	N-Term(iTRAQ4plex); S4(Phospho); K17(iTR
ssPPATDPGPVPSsPR	Q499N6	N-Term(iTRAQ4plex); S2(Phospho); S14(Pho
gHAGGQRPEPsSPDGPA	A0JPL9	N-Term(iTRAQ4plex); S11(Phospho)
iAcEEEFsDsDEEGEGGR	Q4QQW4	N-Term(iTRAQ4plex); C3(Carbamidomethyl);
gSsQPNLSTSYSEQEYGk	F1LQ45	N-Term(iTRAQ4plex); S3(Phospho); K18(iTR
gSAEsPDEGITTTEGEGE	P15205;F1LRL9	N-Term(iTRAQ4plex); S5(Phospho); C19(Ca
kEELsDSVDR	A0A0G2JXZ3	N-Term(iTRAQ4plex); K1(iTRAQ4plex); S5(P
gAEQGsEEEkEEkEGEVk	Q6MG08	N-Term(iTRAQ4plex); S6(Phospho); K10(iTR
sAGFIPIkEDFsPEkk	F1LRL9	N-Term(iTRAQ4plex); K8(iTRAQ4plex); S12(
hIPPAPLASsPTFck	P22462	N-Term(iTRAQ4plex); S10(Phospho); C14(Ca
gMcEQSYWNPLEEPPcQ	F1LUY5	N-Term(iTRAQ4plex); C3(Carbamidomethyl);
gEGAQDEEEGGAssDAtE	Q02563	N-Term(iTRAQ4plex); S13(Phospho); S14(Ph
nLsLEAIQLcDR	Q6AYN4	N-Term(iTRAQ4plex); S3(Phospho); C10(Ca
sALTPPTDmPTPTTDk	Q5FVJ0;A0A0G2K6A9	N-Term(Acetyl); S1(Phospho); M9(Oxidation)
sPGTPAGEGSGSPPk	A0A0G2JSM7;D3ZZ99	N-Term(iTRAQ4plex); S1(Phospho); K15(iTR
aAFskDEskEPIVEVR	F1LUV9;A0A0G2K0M8	N-Term(iTRAQ4plex); S4(Phospho); K5(iTRA
hTVIAAQsLEALSGLQk	Q9QXY2	N-Term(iTRAQ4plex); S8(Phospho); K17(iTR
lSGINEIPRPFSPPITSNtS	D3ZYR1	N-Term(iTRAQ4plex); T19(Phospho)
eAEDsDsDDNIkR	Q3SWT4	N-Term(iTRAQ4plex); S5(Phospho); S7(Phos
eEcPRPmsISPPDFSPk	P15205;F1LRL9	N-Term(iTRAQ4plex); C3(Carbamidomethyl);
aGSGEDPVLAPsGTPPPS	Q56B11	N-Term(iTRAQ4plex); S12(Phospho)
lHSsSLELGPRPLEQENE	D4ABP3	N-Term(iTRAQ4plex); S4(Phospho)
tAITTAsIR	Q4V7D4	N-Term(iTRAQ4plex); S7(Phospho)
aSSSTDGQLAsPGENGE	F1LMV8	N-Term(iTRAQ4plex); S11(Phospho)
dTVIIVsEPSEDEESHDLP	D3Z9Z9	N-Term(iTRAQ4plex); S7(Phospho)
rGFsEEQLR	P37805	N-Term(iTRAQ4plex); S4(Phospho)
ktSPSDSMTk	F1LP59	N-Term(iTRAQ4plex); K1(iTRAQ4plex); T2(P
rNtAPEAQESGLPSGLR	Q5BJT1	N-Term(iTRAQ4plex); T3(Phospho)
mLQALsPk	P19836	N-Term(iTRAQ4plex); S6(Phospho); K8(iTRA
sETsPVAPR	F1M386	N-Term(iTRAQ4plex); S4(Phospho)
aSDPQsPPQVSR	F1M9G6	N-Term(iTRAQ4plex); S6(Phospho)
qVQEAIAGISSAAQATsPT	A0A0G2JY03	N-Term(iTRAQ4plex); S17(Phospho); K23(iT
tHtSPPRAPSsPGR	D3ZEI4	N-Term(iTRAQ4plex); T3(Phospho); S11(Pho
aLHSEHAVDTHsDsEEEL	B2GV94	N-Term(iTRAQ4plex); S12(Phospho); S14(Ph
sRsPVDSPVPASMFAPEF	D2XV59	N-Term(iTRAQ4plex); S3(Phospho)
aEILATDVSVEDEEGTGs	G3V6M0	N-Term(iTRAQ4plex); S18(Phospho); K20(iT
mLAEsDEsGDEESVSQTI	D4A9D8	N-Term(iTRAQ4plex); S5(Phospho); S8(Phos

kDPQDMEGEksPASPFAQ	A0A0U1RRX4;F1MAQ5	K1(iTRAQ4plex); N-Term(iTRAQ4plex); K10(
aASPYSQRPAsPTAVR	F1M2K6	N-Term(iTRAQ4plex); S11(Phospho)
dIQRPESEHGLsPDSENF	G3V8Q9	N-Term(iTRAQ4plex); S12(Phospho); K21(iT
eQSSQEsGEETR	O35314	N-Term(iTRAQ4plex); S7(Phospho)
kksPATPQAkPEGAAGAA	D4A0E2	N-Term(iTRAQ4plex); K1(iTRAQ4plex); K2(iT
aANktPPkSPGDPAk	D3ZQL7	N-Term(iTRAQ4plex); K4(iTRAQ4plex); T5(P
sTLPVDEGsPLEk	A0A0G2JW88	N-Term(iTRAQ4plex); S9(Phospho); K13(iTR
dksPSEGDVTPPkR	D3ZBU7	N-Term(iTRAQ4plex); K2(iTRAQ4plex); S3(P
fGEESkEPFsDEDEDDND	Q6P9X1	N-Term(iTRAQ4plex); K6(iTRAQ4plex); S10(
gSDDVLLSGAVPEYEVGH	M0RC54	N-Term(iTRAQ4plex); S20(Phospho)
tDHGAEIVYksPVVsGDTs	D4A1Q2	N-Term(iTRAQ4plex); K10(iTRAQ4plex); S11
lsQGsGSSITAAGMR	D3ZCL8	N-Term(iTRAQ4plex); S2(Phospho); S5(Phos
aHsPVAVQVPGMQNNIAD	F1LXV3	N-Term(Acetyl); S3(Phospho); K25(iTRAQ4p
eGGSGsPSFSSPMDIFDM	Q4QR73	N-Term(iTRAQ4plex); S6(Phospho)
dSSGEVLSsV	A0A0H2UHZ1	N-Term(iTRAQ4plex); S9(Phospho)
vVSsTsEEEEAFTEk	A0A0G2JZ50	N-Term(iTRAQ4plex); S4(Phospho); S6(Phos
tHLQAGLsPDTIEk	F1LRF3	N-Term(Acetyl); S8(Phospho); K14(iTRAQ4p
nMTVEQLLTGSPTsPTVE	F1M403	N-Term(iTRAQ4plex); S14(Phospho); K21(iT
tPDSLEPsPLkESPcRDSL	A0A0G2K6R9;F1M9N9	N-Term(iTRAQ4plex); S8(Phospho); K11(iTR
sRSLsESYELSSDLQDk	A0A0G2JUG7	N-Term(iTRAQ4plex); S1(Phospho); S5(Phos
aEEVILAEDEtDVEQRHPF	A0A0G2JXK1	N-Term(iTRAQ4plex); T11(Phospho)
aADDkVAILtDDEEEQk	A0A0G2JW92	N-Term(Acetyl); K5(iTRAQ4plex); T10(Phosp
nLIPMDPNGLSDPyVk	P63319;F1M2P8	N-Term(iTRAQ4plex); Y14(Phospho); K16(iT
eSAsYQEALAR	P47819	N-Term(iTRAQ4plex); S4(Phospho)
lLLPGSSPssPEDEVkDR	C0IXW5	N-Term(iTRAQ4plex); S9(Phospho); S10(Pho
aGRPRsEEDNELNLPNLA	P22288	N-Term(iTRAQ4plex); S6(Phospho)
eTPQtPSR	A0A0G2K2M9	N-Term(iTRAQ4plex); T5(Phospho)
qAGSHSNsFR	Q03348	N-Term(iTRAQ4plex); S8(Phospho)
nDGVkESsEstNtTIEDEDT	P11275	N-Term(iTRAQ4plex); K5(iTRAQ4plex); S8(P
eQDVVPFQEsPk	Q9WV48	N-Term(iTRAQ4plex); S10(Phospho); K12(iT
sSIsPmDEPVPDSEsPIEk	P15205;F1LRL9	N-Term(iTRAQ4plex); S4(Phospho); M6(Oxid
gsISDGTNILDEVTFPAR	D4ABB2	N-Term(iTRAQ4plex); S2(Phospho)
lLHEDLDEsDDDVDEk	A0A0G2K808	N-Term(iTRAQ4plex); S9(Phospho); K16(iTR
eLLGLEDEsPAH	D3ZSZ4	N-Term(iTRAQ4plex); S9(Phospho)
lLHEDLDEsDDDVDEkLQR	A0A0G2K808	N-Term(iTRAQ4plex); S9(Phospho); K16(iTR
dGATsEEETELEk	Q76MT4	N-Term(iTRAQ4plex); S5(Phospho); K13(iTR
gkAsPFEEDQNR	A0A0G2K8F9	N-Term(iTRAQ4plex); K2(iTRAQ4plex); S4(P
sPAEVksPAEVk	F1LRZ7	N-Term(iTRAQ4plex); S1(Phospho); K6(iTRA
gWAFGsNSLPIAGSVGMG	D4A769	N-Term(iTRAQ4plex); S6(Phospho)
sQVVALLGAQLsPAR	E9PU32	N-Term(iTRAQ4plex); S12(Phospho)
mSEsLDTADPAVTGAk	P63012	N-Term(iTRAQ4plex); S4(Phospho); K16(iTR
isNAEPEPR	B0BNF1	N-Term(iTRAQ4plex); S2(Phospho)
gILAADEsVGSmAk	P09117	N-Term(iTRAQ4plex); S8(Phospho); M12(Ox
eLsEGEEkGDPEk	O88420	N-Term(iTRAQ4plex); S3(Phospho); K8(iTRA
eTcSPVsEGEEFSSPATN	D3ZSU3	N-Term(iTRAQ4plex); C3(Carbamidomethyl);
asPEAEEAAR	A0A0G2K3F4	N-Term(iTRAQ4plex); S2(Phospho)
sSsFSQGEAEPR	Q63638	N-Term(iTRAQ4plex); S3(Phospho)
fWEVISDEHGIDPTGSYHG	P85108	N-Term(iTRAQ4plex); S21(Phospho)
qLsDIEER	P12839	N-Term(iTRAQ4plex); S3(Phospho)
yEMAcAIADAFNLPSSHLR	A0A0G2JT30	N-Term(iTRAQ4plex); C5(Carbamidomethyl);
dcLcQLcAQPmSSsPk	F1LWK7	N-Term(iTRAQ4plex); C2(Carbamidomethyl);
gDWsALGk	P08050	N-Term(iTRAQ4plex); S4(Phospho); K8(iTRA
sLPTTVPEsPNYR	F1M062	N-Term(iTRAQ4plex); S9(Phospho)
eQNsPIYISR	Q792I0	N-Term(iTRAQ4plex); S4(Phospho)
sDTQLsEDNNE	A0A0G2JZ56;F1M9N9	N-Term(iTRAQ4plex); S6(Phospho)

Information Classification: General

nAPPAGDEPLAEtEsESE/	A0A0G2K1U5	N-Term(iTRAQ4plex); T13(Phospho); S15(Ph
rVsADGQPFQR	A0A140TAC8	N-Term(iTRAQ4plex); S3(Phospho)
aSAPATPLsPTR	G3V7U4	N-Term(iTRAQ4plex); S9(Phospho)
rGtGGVDTAAVGGVFDVS	P07335	N-Term(iTRAQ4plex); T3(Phospho)
lLDFGsLSNLQVTQPTVGI	A0A0H2UHQ8	N-Term(iTRAQ4plex); S6(Phospho); K22(iTR
sksDNQISDk	Q2KN99	N-Term(iTRAQ4plex); K2(iTRAQ4plex); S3(P
dTASLSTTPSEsPR	M0R423	N-Term(iTRAQ4plex); S12(Phospho)
lDsPPPSPITEASEAAEAA	D3ZF26	N-Term(iTRAQ4plex); S3(Phospho)
kLDPAQSAsRENLLEEQG	P16086	N-Term(iTRAQ4plex); K1(iTRAQ4plex); S9(P
kVEEEQEADEEDVsEEET	Q52KJ9	N-Term(iTRAQ4plex); K1(iTRAQ4plex); S14(
mSPNETLFLEsTNk	Q0D2L6	N-Term(iTRAQ4plex); S11(Phospho); K14(iT
dATEDsITEDDkR	P0CD96	N-Term(iTRAQ4plex); S6(Phospho); K12(iTR
kEDkPEVQsPVk	F1M403	K1(iTRAQ4plex); N-Term(iTRAQ4plex); K4(i
gPGDVVSIEILPGAAAtPS	D3ZAZ1	N-Term(iTRAQ4plex); T17(Phospho)
eGEGGAGAPDSSSFsPk\	P34926	N-Term(iTRAQ4plex); S15(Phospho); K17(iT
tLLDHEYDDDSEYNVDSY	A6JUQ6	N-Term(iTRAQ4plex); K27(iTRAQ4plex); S30
gALGsPVAAAGAAMQETI	A1L1K8	N-Term(iTRAQ4plex); S5(Phospho); C20(Ca
dSEVSTkPEGVHAPNQPs	Q64548	N-Term(iTRAQ4plex); K7(iTRAQ4plex); S18(
sDSEGsDYTPGk	E9PU01	N-Term(iTRAQ4plex); S6(Phospho); K12(iTR
tVDEQEDMDLQIsPDR	A0A0G2K6R9;F1M9N9	N-Term(iTRAQ4plex); S13(Phospho)
qTLINIPSLNDsDsEVEDIS	Q3ZB99	N-Term(iTRAQ4plex); S12(Phospho); S14(Ph
rGsDELYAIk	P63319	N-Term(iTRAQ4plex); S3(Phospho); K10(iTR
vkDEFTAEkEAsPPSSADk	A0A0U1RRX4;F1MAQ5	N-Term(iTRAQ4plex); K2(iTRAQ4plex); K9(i
lPSESDLLEGEVtDEDEEA	A0A0G2K4N6	N-Term(iTRAQ4plex); T13(Phospho); M21(O
lGAPENSGIStLER	F1LYQ8	N-Term(iTRAQ4plex); T11(Phospho)
sPcsLkEQQPHk	P34926;Q63330	S1(Phospho); N-Term(iTRAQ4plex); C3(Carb
hFIsGDEPk	P13233	N-Term(iTRAQ4plex); S4(Phospho); K9(iTRA
tSPsSFPSPGPQPSGPSW	D3ZG37	N-Term(iTRAQ4plex); S4(Phospho); K37(iTR
rREsQTALVVNQR	Q569C0	N-Term(iTRAQ4plex); S4(Phospho)
dQTEDPASsPTSk	D4A2A3	N-Term(iTRAQ4plex); S9(Phospho); K13(iTR
eTQTPLATHQsEEDEEDE	A0A173DW30	N-Term(iTRAQ4plex); S11(Phospho); K24(iT
dNsISPESDVDTASTISLV	A0A0G2K315	N-Term(iTRAQ4plex); S3(Phospho)
lALEsPEVQDLQLER	B5DFF4	N-Term(iTRAQ4plex); S5(Phospho)
rPIIPGMEFsR	P48303	N-Term(iTRAQ4plex); S10(Phospho)
tLEEEMsLEk	D4ABN3	N-Term(iTRAQ4plex); S7(Phospho); K10(iTR
sAkDsDDEEEVVHVDRDH	P61265	N-Term(iTRAQ4plex); K3(iTRAQ4plex); S5(P
esPPLLPLGTPc	B4F7C1	N-Term(iTRAQ4plex); S2(Phospho); C12(Ca
ncSsPAFSk	D4A1R8	N-Term(iTRAQ4plex); C2(Carbamidomethyl);
iAELEEERsQGsTSNsDW	F1LRL9	N-Term(iTRAQ4plex); S9(Phospho); S12(Pho
wEEIStPGSPPDtPPGNPV	Q6AXV0	N-Term(iTRAQ4plex); T6(Phospho); T13(Pho
kLNFNsEGEAEELMVDNV	P27139	N-Term(iTRAQ4plex); K1(iTRAQ4plex); S6(P
qEPDPGPNVEPSItPPAVI	P34926	N-Term(iTRAQ4plex); T14(Phospho)
qHLENDPGsNEDTDIPk	Q920J4	N-Term(iTRAQ4plex); S9(Phospho); K17(iTR
yEPsDkDRQSPPAk	Q6TQE1	N-Term(iTRAQ4plex); S4(Phospho); K6(iTRA
kPSPEELDQGIDPNsPLF(	Q5XIR9	N-Term(iTRAQ4plex); K1(iTRAQ4plex); S15(
rMsksPVDSSVLEGVk	F1LMT5	N-Term(iTRAQ4plex); S3(Phospho); K4(iTRA
rsPsPPPAR	B2RYB3;A0A0G2K4F6	N-Term(iTRAQ4plex); S2(Phospho); S4(Phos
hSAEVAGYQDSIGQLEsD	P23565;G3V8Q2	N-Term(iTRAQ4plex); S17(Phospho)
qQsLPLRPIIPLVAR	A0A0G2JWM9	N-Term(iTRAQ4plex); S3(Phospho)
rEsPGEWGADVPR	F1LSB5	N-Term(iTRAQ4plex); S3(Phospho)
sNsWQGNVGGNkk	M0R5P8	N-Term(iTRAQ4plex); S3(Phospho); K12(iTR
iPssPDLkk	P63142	N-Term(iTRAQ4plex); S3(Phospho); S4(Phos
eAGHGsDkEEISk	Q64542	N-Term(iTRAQ4plex); S6(Phospho); K8(iTRA
dGVEDGDSQGDGsSQPD	P97924	N-Term(iTRAQ4plex); S13(Phospho)
vYIsSPHcsPAHNk	D3Z9L5	N-Term(iTRAQ4plex); S4(Phospho); C8(Carb

aQLEEASsAR	P23565;G3V8Q2	N-Term(iTRAQ4plex); S8(Phospho)
eVAMTEHkMsVEEVcR	P06687	N-Term(iTRAQ4plex); K8(iTRAQ4plex); S10(
dAAQSsPAFGDR	P06214	N-Term(iTRAQ4plex); S6(Phospho)
qVVPPFkPNISGEFGLDNI	Q9R1X8	N-Term(iTRAQ4plex); K7(iTRAQ4plex); T31(
lGPGGGsSR	B0BN86	N-Term(iTRAQ4plex); S7(Phospho)
ikEsPSEQESR	D4A626	N-Term(iTRAQ4plex); K2(iTRAQ4plex); S4(P
tLPsPPPEEAHLPLAGQVI	G3V984	N-Term(iTRAQ4plex); S4(Phospho)
aFLEYVAESLEPPsPFELL	A0A0H2UHQ3	N-Term(iTRAQ4plex); S14(Phospho)
dLQILPPPLIPtPPSDEAR	Q4V896	N-Term(iTRAQ4plex); T12(Phospho)
vYQTsPTDEDEEDDESSA	D3ZZQ0	N-Term(iTRAQ4plex); S5(Phospho)
sWsDPTPMk	A0A140TAI4	N-Term(iTRAQ4plex); S3(Phospho); K9(iTRA
vTDALPEPEPAGAMAAsE	Q8CGS4	N-Term(iTRAQ4plex); S17(Phospho)
vQPHGsLEQcWk	Q541U0	N-Term(iTRAQ4plex); S6(Phospho); C10(Ca
vSGsPSSGFR	P12839	N-Term(iTRAQ4plex); S4(Phospho)
aQPAGsPSHQIQQR	G3V7P7	N-Term(iTRAQ4plex); S6(Phospho)
nVVHQLsVTLEDLYNGITk	Q4QR73	N-Term(iTRAQ4plex); S7(Phospho); K19(iTR
qSQIQVFEDGADTtsPETF	A0A0G2K428	N-Term(iTRAQ4plex); T14(Phospho); S15(Ph
eLEDLLsPLEESVk	P17955	N-Term(iTRAQ4plex); S7(Phospho); K14(iTR
sPsIDSIQk	F1M2K6;F1M787	N-Term(iTRAQ4plex); S3(Phospho); K9(iTRA
dsSDEHcVDISSVGTPLAF	Q923K6	N-Term(iTRAQ4plex); S2(Phospho); C7(Carb
tEGDsPAAALsPQmHQES	A0A0G2K6R9;F1M9N9;A0	N-Term(iTRAQ4plex); S5(Phospho); S11(Pho
eSTQPPEDsSPPASSESS	A0A0G2JVW5	N-Term(iTRAQ4plex); S9(Phospho)
eVDPSTGELQSLQMPESI	O08623	N-Term(iTRAQ4plex); S22(Phospho); K34(iT
hLSVPNIIITPPtPTGMGLS	D3ZSX5	N-Term(iTRAQ4plex); T13(Phospho)
sSPTSEDSATALQtTLSLIS	D4A748	N-Term(iTRAQ4plex); T14(Phospho); M21(O
gsDDAPDADTAIINAEGGO	Q1WIM3	N-Term(iTRAQ4plex); S2(Phospho); K25(iTR
sLEALEAWIVEAEGILQAO	Q8VHJ9	N-Term(iTRAQ4plex); S24(Phospho)
gsLAsLDSLR	D3ZZZ9	N-Term(iTRAQ4plex); S2(Phospho); S5(Phos
sDSELEVkPSESLLR	D3ZYB4	N-Term(iTRAQ4plex); S1(Phospho); K8(iTRA
gPSSQEDESGGIEDsPDR	A0A0G2K0F3	N-Term(iTRAQ4plex); S15(Phospho)
akPAAQSEEETAAsPAAsI	Q4G061	N-Term(iTRAQ4plex); K2(iTRAQ4plex); S14(
aGEAsAEsTGAADGAPQE	Q05175	N-Term(iTRAQ4plex); S5(Phospho); S8(Phos
fALGLSGGsLVSmLAR	P85971	N-Term(iTRAQ4plex); S9(Phospho); M13(Ox
kPGNNEGSGAPsPLSk	Q05140	N-Term(iTRAQ4plex); K1(iTRAQ4plex); S12(
ksPPPAk	A0A0G2JWM2	N-Term(iTRAQ4plex); K1(iTRAQ4plex); S2(P
fDLYDVDSksPDLSk	D3ZGN2	N-Term(iTRAQ4plex); K9(iTRAQ4plex); S10(
rSsLVDEAPEDEEFEQk	G3V7E6	N-Term(iTRAQ4plex); S3(Phospho); K17(iTR
eLDDISLTPDPEPTHEDsV	A0A0G2K6A9	N-Term(iTRAQ4plex); S18(Phospho)
sPVkEEAk	F1LRZ7	N-Term(iTRAQ4plex); S1(Phospho); K4(iTRA
sRsPLLNDR	Q5RKH1	N-Term(iTRAQ4plex); S1(Phospho); S3(Phos
iAELEEERsQGsTSNSDW	F1LRL9	N-Term(iTRAQ4plex); S9(Phospho); S12(Pho
vPVPEGLDLDAWINEPPs	B5DFK6	N-Term(iTRAQ4plex); S18(Phospho); S20(Ph
qENGAVVPLQAGDGEEG	D3ZFB6	N-Term(iTRAQ4plex); S24(Phospho); K29(iT
kEEGASRDsLEGVGQGG	D4A1J3	N-Term(iTRAQ4plex); K1(iTRAQ4plex); S9(P
sLmssPEDLTkDFEELkAE	P15205;F1LRL9	N-Term(iTRAQ4plex); M3(Oxidation); S4(Pho
aVAtSPSLEAPk	D3ZH78	N-Term(iTRAQ4plex); T4(Phospho); K12(iTR
iPNQFQGsPPTPSDQSVk	B4F7C7	N-Term(iTRAQ4plex); S8(Phospho); K18(iTR
ssSLDALGPAR	D3ZY47	N-Term(iTRAQ4plex); S2(Phospho)
eEAAGcNTSAEkETSsPAs	P34926	N-Term(iTRAQ4plex); C6(Carbamidomethyl);
nSASSsPVASEAPSR	D3ZWA1	N-Term(iTRAQ4plex); S6(Phospho)
eLFsPLHALNFGIGGDTTF	O35264	N-Term(iTRAQ4plex); S4(Phospho)
yNLDAsEEEDSNk	O35986	N-Term(iTRAQ4plex); S6(Phospho); K13(iTR
aVNVsEESDWTENSFQG	Q80Z30	N-Term(iTRAQ4plex); S5(Phospho); K26(iTR
dLSsSPPGPYGQEmYVFF	A0A0G2JVK3	N-Term(iTRAQ4plex); S4(Phospho); M14(Ox
qLsLEGAGLAIEEFk	Q9JKC9	N-Term(iTRAQ4plex); S3(Phospho); K15(iTR

Information Classification: General

sRTSVQTEDDQLIAGQSA	A0A0G2JYF7;D4A6H8	N-Term(iTRAQ4plex); S1(Phospho)
rAsFAk	Q9JIH7	N-Term(iTRAQ4plex); S3(Phospho); K6(iTRA
qIcVVmLETLSQsPPk	Q4V8F6	N-Term(iTRAQ4plex); C3(Carbamidomethyl);
aLsLPETLTSk	D3ZSV8	N-Term(iTRAQ4plex); S3(Phospho); K11(iTR
sRLsLGAGGR	B5DF40	N-Term(iTRAQ4plex); S4(Phospho)
eSPtVSPR	D3ZH28	N-Term(iTRAQ4plex); T4(Phospho)
gSSDGRGsDSESDLPHR	F1M392	N-Term(iTRAQ4plex); S8(Phospho)
eSSPTYsPGFSDSTSGAk	P15205;F1LRL9	N-Term(iTRAQ4plex); S7(Phospho); K18(iTR
dLDGsYDQLTGHPPGPSI	Q9JM59	N-Term(iTRAQ4plex); S5(Phospho); K18(iTR
sSPAYcTSSsDITEPEQk	D3ZCL8	N-Term(iTRAQ4plex); C6(Carbamidomethyl);
lQEDPNYsPQR	A0A0G2K7C1	N-Term(iTRAQ4plex); S8(Phospho)
vSALEEDMDDVEssEEEE	D3ZGL5	N-Term(iTRAQ4plex); S13(Phospho); S14(Ph
sLVGTPYWmAPEVISR	D4A280	N-Term(iTRAQ4plex); S1(Phospho); M9(Oxid
lDGETAsDSESR	Q91XU8	N-Term(iTRAQ4plex); S7(Phospho)
sTTEPGPPGYSVsPPVPG	D3ZEI4	N-Term(iTRAQ4plex); S13(Phospho)
sSLFPFEDAFLDDSHGDG	D4AD99	N-Term(iTRAQ4plex); S26(Phospho)
gPLEAPQDGEAEEGTTsD	Q5QD51	N-Term(iTRAQ4plex); S17(Phospho); K21(iT
sEDLPsSPQVSVAAVETk	F1M842	N-Term(iTRAQ4plex); S6(Phospho); K18(iTR
eEGVSQLGLGTPSAPEsF	D3ZF26	N-Term(iTRAQ4plex); S17(Phospho); K20(iT
lVDSDGsLAEVPk	Q5RKL5	N-Term(iTRAQ4plex); S7(Phospho); K13(iTR
sPAEAksPAEAkPPAEAks	F1LRZ7	N-Term(iTRAQ4plex); K6(iTRAQ4plex); S7(P
tEDGDWEWsDDEmDEk	A0A0G2K007	N-Term(iTRAQ4plex); S9(Phospho); M13(Ox
gsPPLLDAPAsPPQAPR	M0RDK4	N-Term(iTRAQ4plex); S2(Phospho); S11(Pho
yHHVNsYDEDDTTEEEPV	Q810W7	N-Term(iTRAQ4plex); S6(Phospho)
lQDSsDPDtcSEEEVSSRL	A0A1B0GWY5	N-Term(iTRAQ4plex); S5(Phospho); T9(Phos
tVQkDADEEDsDEETSHLI	Q3B8P7	N-Term(iTRAQ4plex); K4(iTRAQ4plex); S11(
lLEDsEsGEEAVAR	A0A0G2K2E8	N-Term(iTRAQ4plex); S5(Phospho); S7(Phos
aSPEEPGDsPPLRR	A2RRU4	N-Term(iTRAQ4plex); S9(Phospho)
gNEQNPEEDAQSDVTEG	Q63564	N-Term(iTRAQ4plex); Y25(Phospho); K39(iT
lRLsPsPTSQR	G3V8L3	N-Term(iTRAQ4plex); S4(Phospho); S6(Phos
dQNLPQILEESRsE	P97846	N-Term(iTRAQ4plex); S13(Phospho)
kVAEQTFIkDDYLETLsSP	F1M1Y0	K1(iTRAQ4plex); N-Term(iTRAQ4plex); K9(iT
ePITVSsEQMSHFR	P27139	N-Term(iTRAQ4plex); S7(Phospho)
sLsPPQSQSk	Q6GMN2	N-Term(iTRAQ4plex); S3(Phospho); K10(iTR
eQGAESRSstPLPTVSSS/	Q62733	N-Term(iTRAQ4plex); S9(Phospho); T10(Pho
iLEENTTSAEAsSEQDGE	B1WBT4	N-Term(iTRAQ4plex); S12(Phospho); C19(Ca
dGPcSPPsPDELPANVk	D3ZWS0	N-Term(iTRAQ4plex); C4(Carbamidomethyl);
rLsPPGGsGSGVPGGGPL	F1LQ41	N-Term(iTRAQ4plex); S3(Phospho); S8(Phos
tFcGTPDYIAPEIIAYQPYG	F1LS36;P63319;F1M2P8;	N-Term(iTRAQ4plex); T1(Phospho); C3(Carb
lDGETAsDsESRAETAPPI	Q91XU8	N-Term(iTRAQ4plex); S7(Phospho); S9(Phos
tEPASEEQEPAEDTDQAR	Q5QD51	N-Term(iTRAQ4plex); S20(Phospho); K25(iT
eRDHsPTPSVFNSDEER	Q5U317	N-Term(iTRAQ4plex); S5(Phospho)
aGSsPTQGAQNEAPR	D3ZG21	N-Term(iTRAQ4plex); S4(Phospho)
eEDEEDEDEPTETETsGE	D3ZH75	N-Term(iTRAQ4plex); S16(Phospho)
rPDsPGQDAPR	A0A0G2JWK6	N-Term(iTRAQ4plex); S4(Phospho)
lNATtSLEQDkIEPPR	E9PT53	N-Term(iTRAQ4plex); T5(Phospho); K11(iTR
aMGDEDsDEsGGGsPkPS	G3V927	N-Term(iTRAQ4plex); S7(Phospho); S10(Pho
aETAPPPTSIDDtPEVLNR	Q91XU8	N-Term(iTRAQ4plex); T13(Phospho)
qDLPGALscNAIQEALELD	G3V8V0	N-Term(iTRAQ4plex); S8(Phospho); C9(Carb
sGSPGTPAYPLSDLLFQS	D4A4L2	N-Term(iTRAQ4plex); S1(Phospho)
sLMssPEDLTkDFEELk	P15205;F1LRL9	N-Term(iTRAQ4plex); S4(Phospho); S5(Phos
lEEELSSLETELGPsPPAP	F1M4I4	N-Term(iTRAQ4plex); S15(Phospho)
sDVEIPATVTAFSFEDDS\	Q62847;A0A0G2JW28	N-Term(iTRAQ4plex); S22(Phospho); K25(iT
hSAPsPPVsVGsEEHPHS	D3ZYS1	N-Term(iTRAQ4plex); S5(Phospho); S9(Phos
sFDHLISDTk	A0A0G2JWL3	N-Term(iTRAQ4plex); S1(Phospho); K10(iTR

Information Classification: General

tPDSLEPSPLkEsPcRDsLI	A0A0G2K6R9;F1M9N9	N-Term(iTRAQ4plex); K11(iTRAQ4plex); S13
gGPGkLsPR	F1M842	N-Term(iTRAQ4plex); K5(iTRAQ4plex); S7(P
eEPILtLVDEAIVk	F1LMV8	N-Term(iTRAQ4plex); T6(Phospho); K14(iTR
dVINRsPTQLGk	B5DEI5	N-Term(iTRAQ4plex); S6(Phospho); K12(iTR
nEDGRDEPGQLsPGVQY	O35832	N-Term(iTRAQ4plex); S12(Phospho)
iIPQEELNsPTsAPSSEk	A0A0G2JXR9	N-Term(iTRAQ4plex); S9(Phospho); S12(Pho
dVPLGPEDPkEEDGsFDY	D3ZW56	N-Term(iTRAQ4plex); K10(iTRAQ4plex); S15
rLsLPGLLSQVsPR	P0C5Y8	N-Term(iTRAQ4plex); S3(Phospho); S12(Pho
kLAGLGPEDDPDtDDEQE	D3ZBC7	N-Term(iTRAQ4plex); K1(iTRAQ4plex); T13(
sDIsPLtPR	P15205;F1LRL9	N-Term(iTRAQ4plex); S4(Phospho); T7(Phos
gGGGGGsPGPTAGPEPL	F1M6V8	N-Term(iTRAQ4plex); S7(Phospho)
vGSVLDSRVsVDsNLFVY	F1M3W5	N-Term(iTRAQ4plex); S10(Phospho); S13(Ph
mSIsEGTVsDk	P15205;F1LRL9	N-Term(iTRAQ4plex); S4(Phospho); S9(Phos
vQIsPDSGGLPER	M0R961	N-Term(iTRAQ4plex); S4(Phospho)
tGLEQEEQPLNLSDsPR	D4A3P4	N-Term(iTRAQ4plex); S15(Phospho)
akDPPRsPPDSQSEQLLL	A0A0H2UHT1	N-Term(iTRAQ4plex); K2(iTRAQ4plex); S7(P
lySLGk	P13233	N-Term(iTRAQ4plex); Y2(Phospho); K6(iTRA
vPsTETEALASNLMGMFE	P50398	N-Term(iTRAQ4plex); S3(Phospho); K19(iTR
kGSDGAsPPASPSIIPR	D3ZG83	N-Term(iTRAQ4plex); K1(iTRAQ4plex); S7(P
kGAEEEEEEEDDDsEEEI	D4A559	N-Term(iTRAQ4plex); K1(iTRAQ4plex); S14(
aPPAPGAVSGsSGEVDEI	B1WBX7	N-Term(iTRAQ4plex); S11(Phospho); K22(iT
gSYmEVEDNRsQVETEDI	A0A0H2UHZ1	N-Term(iTRAQ4plex); M4(Oxidation); S11(Ph
rGsMNNEMLSPEVGPVR	Q5U2N3	N-Term(iTRAQ4plex); S3(Phospho)
kSHsSPSLNPDAsPVTAk	D4A404	N-Term(iTRAQ4plex); K1(iTRAQ4plex); S4(P
nFsGGk	P11275	N-Term(iTRAQ4plex); S3(Phospho); K6(iTRA
kMsSSGAk	P13233	N-Term(iTRAQ4plex); K1(iTRAQ4plex); S3(P
aEDGAAPsPSSEtPk	F1LMW7	N-Term(iTRAQ4plex); S8(Phospho); T13(Pho
fLSQIEsDRLALLQVR	P05708;M0RAQ6	N-Term(iTRAQ4plex); S7(Phospho)
lkEGLDISHLQGQEELLSS	Q9R1N0	N-Term(iTRAQ4plex); K2(iTRAQ4plex); K22(
iPDSTHAQLESSSSsFES(	A0A096P6M3	N-Term(iTRAQ4plex); S15(Phospho); K20(iT
eVEEELAASGGQsPTGE(	D3ZHV2	N-Term(iTRAQ4plex); S13(Phospho)
tQPDGTSVPGEPAsPISQI	F1LW91	N-Term(iTRAQ4plex); S14(Phospho)
aGkYDLDFksPDDASR	M0R5J4	N-Term(iTRAQ4plex); K3(iTRAQ4plex); K9(iT
yESSLYSQEYSkPVVAsFI	P15205;F1LRL9	N-Term(iTRAQ4plex); K12(iTRAQ4plex); S17
qksEEPSVSLPFLQTALLR	A0A0G2K315	N-Term(iTRAQ4plex); K2(iTRAQ4plex); S3(P
wRPHsPDGPR	A0A096MKC0	N-Term(iTRAQ4plex); S5(Phospho)
tEEEGktDGEGNAEDGAk	Q62861	N-Term(iTRAQ4plex); K6(iTRAQ4plex); T7(P
gRsRtPLTSR	A0A0G2K2M9	N-Term(iTRAQ4plex); S3(Phospho); T5(Phos
nPTEAATAPAsPk	A0A0G2K0M8	N-Term(iTRAQ4plex); S11(Phospho); K13(iT
fHDsEGDDTEETEDYR	B1WC16	N-Term(iTRAQ4plex); S4(Phospho)
rVEsEEsGDEEGkk	Q5HZV9	N-Term(iTRAQ4plex); S4(Phospho); S7(Phos
kPTSDILkEESDDDQSDV	P28818	N-Term(iTRAQ4plex); K1(iTRAQ4plex); K8(iT
sEVTSQSGLSNSsDSLDS	G3V927	N-Term(iTRAQ4plex); S13(Phospho)
qLTAcPELPHNVQEILGLA	B1H264	N-Term(iTRAQ4plex); C5(Carbamidomethyl);
vRHDtPDPsPPR	G3V8F3	N-Term(iTRAQ4plex); T5(Phospho); S9(Phos
sGsSQELDGkPSVSPQEF	A0A0G2K2M9	N-Term(iTRAQ4plex); S3(Phospho); K10(iTR
gNEEVAsHPELSPR	A0A096MJM8	N-Term(iTRAQ4plex); S7(Phospho)
tPEEPELEAEATk	Q62882	N-Term(iTRAQ4plex); T1(Phospho); K13(iTR
tERHsPVsPSSk	A0A0G2K6R9;F1M9N9	N-Term(iTRAQ4plex); S5(Phospho); S8(Phos
aTTSAsASPTLR	D4A0I5	N-Term(iTRAQ4plex); S6(Phospho)
aAVAPPPPPLPGSSNSGS	Q9Z0G8	N-Term(iTRAQ4plex); T20(Phospho); K27(iT
lPSSSSEMGSQDGsPLR	Q99MZ4	N-Term(iTRAQ4plex); S14(Phospho)
rVsTDLPEGQDVYTAAcN	A0A0G2JTT6	N-Term(iTRAQ4plex); S3(Phospho); C17(Ca
tEETPSQLDQDtQVQDMD	D3ZQM0	N-Term(iTRAQ4plex); T12(Phospho); K29(iT
lAFLPPDPTYTLMcDESGs	Q6AY17	N-Term(iTRAQ4plex); C14(Carbamidomethyl

iNSIMSVVTNTLVEELEEs	O88420	N-Term(iTRAQ4plex); S19(Phospho)
tEATQGLDYVPSAGTISPt	P34926	N-Term(iTRAQ4plex); T19(Phospho); S20(Ph
tFQLQLLsPSSSVVPAFNT	B2RYN6	N-Term(iTRAQ4plex); S8(Phospho); K27(iTR
hLPVsPGk	A0A0G2K6R9;F1M9N9	N-Term(iTRAQ4plex); S5(Phospho); K8(iTRA
asSPDDDVQQVVFDLIck	D4A0Y2	N-Term(iTRAQ4plex); S2(Phospho); C17(Car
aPVSVAcGGEsPLDGIcLS	D4A927	N-Term(iTRAQ4plex); C7(Carbamidomethyl);
iAcDEEFsDsEDEGEGGR	Q99PA1	N-Term(iTRAQ4plex); C3(Carbamidomethyl);
sAGPPsPSSR	Q9JHL4	N-Term(iTRAQ4plex); S6(Phospho)
aLEAPPEVEEEDVsANEE	Q5RK33	N-Term(iTRAQ4plex); S14(Phospho)
fsPIEEAk	G3V984	N-Term(iTRAQ4plex); S2(Phospho); K8(iTRA
mLPHAPGVQmQAIPEDA	Q99PA1	N-Term(iTRAQ4plex); M10(Oxidation); S22(F
iHPMAYQLQLQAASNFks	P67874	N-Term(iTRAQ4plex); K17(iTRAQ4plex); S18
rLsDPLLLPHR	F1LWM1	N-Term(iTRAQ4plex); S3(Phospho)
eADIDssDEsDVEEDVDQI	D3Z9E6	N-Term(iTRAQ4plex); S6(Phospho); S7(Phos
dmSQsPTSSFVR	A0A0G2K939	N-Term(iTRAQ4plex); M2(Oxidation); S5(Pho
sDsAPPTPVNR	A0A0G2KA57	N-Term(iTRAQ4plex); S3(Phospho)
gcsPAHAHGHH	D3Z9R8	N-Term(iTRAQ4plex); C2(Carbamidomethyl);
qPsGAGLcDITEGTVVPEI	Q9WU70	N-Term(iTRAQ4plex); S3(Phospho); C8(Carb
hIAEcHPEcsPNEDR	D3ZQM2	N-Term(iTRAQ4plex); C5(Carbamidomethyl);
mEIQVPPsPTDVAk	F1LXQ7	N-Term(iTRAQ4plex); S8(Phospho); K14(iTR
gQEEQsPGLEDk	Q64548	N-Term(iTRAQ4plex); S6(Phospho); K12(iTR
mGPSSSPIPsPsPsPTDSI	P63319	N-Term(iTRAQ4plex); S10(Phospho); S12(Ph
sVSEINsDDELPGR	A0A0G2JYB3	N-Term(iTRAQ4plex); S7(Phospho)
dLAEFSELEYSEMGSSFk	F1LQN3	N-Term(iTRAQ4plex); K18(iTRAQ4plex); S20
lFDsIcNNk	P04897;P10824	N-Term(iTRAQ4plex); S4(Phospho); C6(Carb
sPVTVksPAEAk	F1LRZ7	N-Term(iTRAQ4plex); K6(iTRAQ4plex); S7(P
kETEsPEPTEk	Q5BJN8	N-Term(iTRAQ4plex); K1(iTRAQ4plex); S5(P
lGIyTVLFER	G3V6H5	N-Term(iTRAQ4plex); Y4(Phospho)
aPVHFVEPLsPTGVPGHR	A0A0G2K5T1	N-Term(iTRAQ4plex); S10(Phospho)
nEsEPSEMk	B5DEI5	N-Term(iTRAQ4plex); S3(Phospho); K9(iTRA
sSDPDLSVNSDQESGVEI	A0A0G2K1P1	N-Term(iTRAQ4plex); S1(Phospho); C21(Car
sPAEAkPPAEAksPAEAk	F1LRZ7	N-Term(iTRAQ4plex); S1(Phospho); K6(iTRA
ttSISPALAR	D3ZZQ0	N-Term(iTRAQ4plex); T2(Phospho)
iNSVSSQLsDGPMPSPSA	Q63475	N-Term(iTRAQ4plex); S9(Phospho)
rRPGAsPTGETPTIEEGEI	Q9JJS4	N-Term(iTRAQ4plex); S6(Phospho)
sSPATTVTsPNSTPAk	Q05140;A0A0G2K0B6	N-Term(iTRAQ4plex); S9(Phospho); K16(iTR
sQFSEsESRSPLEEESHS	A0A0G2JZ88	N-Term(iTRAQ4plex); S6(Phospho); K23(iTR
dSGLEDGGEsPSFDTPSC	D4AD03	N-Term(iTRAQ4plex); S10(Phospho)
tIGGGDDsFTTFFcETGAC	Q5XIF6	N-Term(iTRAQ4plex); S8(Phospho); C14(Car
kVPLPGPGsPEVk	D3ZE17	N-Term(iTRAQ4plex); K1(iTRAQ4plex); S9(P
sSIsPMDEPVPDsEsPIEk	P15205;F1LRL9	N-Term(iTRAQ4plex); S4(Phospho); S13(Pho
aSLASLDsNPSTAEk	A0A0G2K939	N-Term(iTRAQ4plex); S8(Phospho); K15(iTR
iEDVGsDEEDDSGkDkk	P34058	N-Term(iTRAQ4plex); S6(Phospho); K14(iTR
tALPTSGsSTGELELLAGE	O88339	N-Term(iTRAQ4plex); S8(Phospho)
aATESFAsDPILYRPVAVA	P11980	N-Term(iTRAQ4plex); S8(Phospho); K23(iTR
eTDDIEsPkR	F1M392	N-Term(iTRAQ4plex); S7(Phospho); K9(iTRA
tSEPEAETNAGNsPVTTN	M0R9T2	N-Term(iTRAQ4plex); S13(Phospho)
lDEDAsPNEEkGDVPk	B2GV74	N-Term(iTRAQ4plex); S6(Phospho); K11(iTR
ekDMSQsPTSSFVR	A0A0G2K939	N-Term(iTRAQ4plex); K2(iTRAQ4plex); S7(P
tsPLLLDR	A0A0G2K2M9	N-Term(iTRAQ4plex); S2(Phospho)
aENsLSDGGPADSVEAAK	Q5U2Z3	N-Term(Acetyl); S4(Phospho); K18(iTRAQ4p
yAALsLSETSLTEk	D3ZTF0	N-Term(iTRAQ4plex); S5(Phospho); K14(iTR
wRsLQQLAEER	P16086	N-Term(iTRAQ4plex); S3(Phospho)
sQDQDsEAHELSR	Q6TQE1	N-Term(iTRAQ4plex); S6(Phospho)
sEDYDAGGsQDDEGSHD	D4A0X3	N-Term(iTRAQ4plex); S9(Phospho)

nHPDSELESTANRHsEET	O35314	N-Term(iTRAQ4plex); S15(Phospho)
atPPPPPPPTYR	F1M1Y0	N-Term(iTRAQ4plex); T2(Phospho)
fQEQEcPPsPEPTRk	A0A0G2K508	N-Term(iTRAQ4plex); C6(Carbamidomethyl);
gEQGEkGssTEAEPAk	Q5BK20	N-Term(iTRAQ4plex); K6(iTRAQ4plex); S8(P
hGEsAWNLENR	P25113	N-Term(iTRAQ4plex); S4(Phospho)
gIVVyTGDR	P06685	N-Term(iTRAQ4plex); Y5(Phospho)
sAPssPTLDcEk	Q78PB6	N-Term(iTRAQ4plex); S4(Phospho); S5(Phos
rSLtNsHLEk	Q9JM15	N-Term(iTRAQ4plex); T4(Phospho); S6(Phos
eLsEGEEk	O88420	N-Term(iTRAQ4plex); S3(Phospho); K8(iTRA
sTESSIGsGk	D4AAL4	N-Term(iTRAQ4plex); S8(Phospho); K10(iTR
vFkEEGsPDR	A0A0G2K5A4	N-Term(iTRAQ4plex); K3(iTRAQ4plex); S7(P
tEHFTTPAVGDSVQPSLA	D3ZJ01	N-Term(iTRAQ4plex); S21(Phospho); K23(iT
cLsPDDSTVk	P34926;Q63330	N-Term(iTRAQ4plex); C1(Carbamidomethyl);
fPLMYEENsRENPFk	G3V7X2	N-Term(iTRAQ4plex); S9(Phospho); K15(iTR
eGTAIGsEEEEAAGVcGP	Q566R3	N-Term(iTRAQ4plex); S7(Phospho); C16(Ca
eDRVVsPEk	F1LQN3	N-Term(iTRAQ4plex); S6(Phospho); K9(iTRA
nFTFNDDFsPSSTSSADL	O35867	N-Term(iTRAQ4plex); S9(Phospho); K26(iTR
ekEAAGGNDssGAtSPINF	Q05695	N-Term(iTRAQ4plex); K2(iTRAQ4plex); S10(
vAAAGSGPsPPcSPGHDF	Q99M64	N-Term(iTRAQ4plex); S9(Phospho); C12(Ca
lsPSLLATALEGPYPVEEI	D3ZWS0	N-Term(iTRAQ4plex); S2(Phospho); C20(Ca
aRsVLVPPGPDSFR	A0A0G2K207	N-Term(iTRAQ4plex); S3(Phospho)
kPVDWGAAsPAVQSFR	P0C5E9	N-Term(iTRAQ4plex); K1(iTRAQ4plex); S9(P
tTPQsPDAQASR	D3ZSZ6	N-Term(iTRAQ4plex); S5(Phospho)
lAEMYGGDDsDRD	D3ZFQ5	N-Term(iTRAQ4plex); S10(Phospho)
qAsGPSSAGATTTVSEkP	A0A0G2JZF2	N-Term(iTRAQ4plex); S3(Phospho); K17(iTR
aMGDEDsGDsDtSPkPSP	G3V849	N-Term(iTRAQ4plex); S7(Phospho); S10(Ph
aLRPGDLPPsPDDVk	G3V8D6	N-Term(iTRAQ4plex); S10(Phospho); K15(iT
ysPENFPYR	Q5PQJ7	N-Term(iTRAQ4plex); S2(Phospho)
eYTmsWmk	A0A0G2JWM2	N-Term(iTRAQ4plex); M4(Oxidation); S5(Ph
rLsQSSLESATSPScS	G3V7H4	N-Term(iTRAQ4plex); S3(Phospho); C15(Ca
aPVVLQPEQIVsEEETPP	E9PST5	N-Term(iTRAQ4plex); S12(Phospho); K23(iT
vARPQILEPRPQsPDLcD	A0A096MJN4	N-Term(iTRAQ4plex); S13(Phospho); C17(C
tLDVksPEAk	F1LRZ7	N-Term(iTRAQ4plex); K5(iTRAQ4plex); S6(P
lTQADISEQPAMTTVVPQ	D4ADX8	N-Term(iTRAQ4plex); S22(Phospho); K24(iT
fTDQsLsPEDAESLSVLSV	P34926;Q63330	N-Term(iTRAQ4plex); S5(Phospho); S7(Phos
dDGsWEVIEGYR	P10860	N-Term(iTRAQ4plex); S4(Phospho)
sRsESDLSQPESDEEGYA	A0A0G2JTT6	N-Term(iTRAQ4plex); S3(Phospho)
sTsTPNVHMVSTTLPVDS	P11345	N-Term(iTRAQ4plex); S3(Phospho)
gPSPLVTmTPAVPAVTPV	M0RD40	N-Term(iTRAQ4plex); M8(Oxidation); S23(P
sPAEVksPAEAk	F1LRZ7	N-Term(iTRAQ4plex); S1(Phospho); K6(iTRA
tRLsPPR	M0R3R6	N-Term(iTRAQ4plex); S4(Phospho)
lsTAscPTPk	M0R423	N-Term(iTRAQ4plex); S2(Phospho); S5(Phos
gGPcVEScGAPAPGsPPR	O88831	N-Term(iTRAQ4plex); C4(Carbamidomethyl);
lSGsPEHFQk	A0A0G2KAJ5	N-Term(iTRAQ4plex); S4(Phospho); K10(iTR
fRVsMDGEWLcLR	Q5FVJ0;A0A0G2K6A9	N-Term(iTRAQ4plex); S4(Phospho); C11(Ca
kSStPEEVk	P45592	N-Term(iTRAQ4plex); K1(iTRAQ4plex); T4(P
gTEVIIITTDTsPSGTk	Q5BJT1	N-Term(iTRAQ4plex); S12(Phospho); K17(iT
rGsGDTSSLIDPDTSLSEL	Q4V7E8	N-Term(iTRAQ4plex); S3(Phospho)
gILRtPDTIR	F1M779	N-Term(iTRAQ4plex); T5(Phospho)
sSSIsSIDkDSk	D3ZU84	N-Term(iTRAQ4plex); S5(Phospho); K9(iTRA
rPPsPEPSAk	G3V6S0	N-Term(iTRAQ4plex); S4(Phospho); K10(iTR
eQGVESPGAQPSSsPk	B2GV14	N-Term(iTRAQ4plex); S14(Phospho); K16(iT
dTAYAAsPLQHLEPR	D3ZL45	N-Term(iTRAQ4plex); S7(Phospho)
aAsTDLGAGEAVVGk	F1LNT3	N-Term(iTRAQ4plex); S3(Phospho); K15(iTR
lEQEIQALEsEESQISAk	D4A4D8	N-Term(iTRAQ4plex); S10(Phospho); K18(iT

Information Classification: General

gPSsQEDEsGGIEDSPDR	A0A0G2K0F3	N-Term(iTRAQ4plex); S4(Phospho); S9(Phos
sPPVAMETASTGVAAVPI	Q64548	N-Term(iTRAQ4plex); S24(Phospho); K29(iT
tQSksPDSTLR	G3C8Z1	N-Term(iTRAQ4plex); K4(iTRAQ4plex); S5(P
sPVDSPVPASMFAPEPSs	D2XV59	N-Term(iTRAQ4plex); S18(Phospho)
vLEkPDPGsLSSR	A0A0G2K0E5	N-Term(iTRAQ4plex); K4(iTRAQ4plex); S9(P
sLQLVDSR	F1LVA9	N-Term(iTRAQ4plex); S1(Phospho)
eNPPVEDSsDEDDRR	B2RYD7	N-Term(iTRAQ4plex); S9(Phospho)
dVmtDETNNEETESPSQE	P15205	N-Term(iTRAQ4plex); M3(Oxidation); T4(Pho
eIVSsPASDAVGELEQQLI	D3ZB48	N-Term(iTRAQ4plex); S5(Phospho); K19(iTR
iLEQQNsSR	F1LMC7	N-Term(iTRAQ4plex); S7(Phospho)
aLsFDNRGEEHR	F1LMV8	N-Term(iTRAQ4plex); S3(Phospho)
vTsEPEAVSEk	A0A0U1RRX4;F1MAQ5	N-Term(iTRAQ4plex); S3(Phospho); K11(iTR
eEDEEAEsPPEkk	A0A0G2K7X3	N-Term(iTRAQ4plex); S8(Phospho); K12(iTR
fSHNYLsDsDTEAk	D3ZFD0	N-Term(iTRAQ4plex); S7(Phospho); S9(Phos
sVYGVTDESQSQsPVHIP	F1LS67	N-Term(iTRAQ4plex); S13(Phospho)
vGVDsDQEHLGVPDNER	D3ZJ86	N-Term(iTRAQ4plex); S5(Phospho)
aPPDsPTTPVR	D3ZAU7	N-Term(iTRAQ4plex); S5(Phospho)
rRsPsPPPAR	B2RYB3;A0A0G2K4F6	N-Term(iTRAQ4plex); S3(Phospho); S5(Phos
gGSPLTTTQGGsPTk	F1M787	N-Term(iTRAQ4plex); S12(Phospho); K15(iT
sTPFIVPSsPTEQGQR	F1M842	N-Term(iTRAQ4plex); S9(Phospho)
aTAPQTQHVsPmR	Q68FR9	N-Term(iTRAQ4plex); S10(Phospho); M12(O
kADAPQASGYHsEGETLk	Q6IE24	N-Term(iTRAQ4plex); K1(iTRAQ4plex); S12(
kGVFPVSFVHILsD	A0A0G2K451	N-Term(iTRAQ4plex); K1(iTRAQ4plex); S13(
aGTksPAVsPSkASEDAk	F8WFS9	N-Term(iTRAQ4plex); K4(iTRAQ4plex); S5(P
sEsLTAENR	A0A0G2JTT6	N-Term(iTRAQ4plex); S3(Phospho)
nSTPsEPDSGQGPPPEEE	Q8VHK7	N-Term(iTRAQ4plex); S5(Phospho); K26(iTR
dDNLPLTEAVRPktPPVVI	Q6AYB6	N-Term(iTRAQ4plex); K13(iTRAQ4plex); T14
vLSPLRsPPLIGSESAYED	P15205;F1LRL9	N-Term(iTRAQ4plex); S7(Phospho); K26(iTR
sLsVPVDLSR	D3ZH55	N-Term(iTRAQ4plex); S3(Phospho)
nDGVkESSEStNttIEDEDT	P11275	N-Term(iTRAQ4plex); K5(iTRAQ4plex); T11(
eVGtSPEDLQELLLPTPLE	A0A0H2UHH8	N-Term(iTRAQ4plex); T4(Phospho)
kVNAEGsVDTVFSQVcTY	P39069	N-Term(iTRAQ4plex); K1(iTRAQ4plex); S7(P
asPPGLELk	F1LVZ8	N-Term(Acetyl); S2(Phospho); K9(iTRAQ4ple
esPVSDLTSDLYQDk	P15205;F1LRL9	N-Term(iTRAQ4plex); S2(Phospho); K15(iTR
dPsLEEIQk	P63043	N-Term(iTRAQ4plex); S3(Phospho); K9(iTRA
yNDWsDDDDDSNESk	Q5XID7	N-Term(iTRAQ4plex); S5(Phospho); K15(iTR
rMsDEFEGSFk	O35147	N-Term(iTRAQ4plex); S3(Phospho); K11(iTR
kEEsEEsEDDMGFGLFD	A0A0G2K4Q1	K1(iTRAQ4plex); N-Term(iTRAQ4plex); S4(P
qSSVTSQSQDVASLSsPS	D4A8B3	N-Term(iTRAQ4plex); S16(Phospho)
tPsELHNDNFSLSTIAEGS	F1M4R7	N-Term(iTRAQ4plex); S3(Phospho)
tVTVIsPEDEQk	D4A3C2	N-Term(iTRAQ4plex); S6(Phospho); K12(iTR
eAEDEEsDHN	M0RD03	N-Term(iTRAQ4plex); S7(Phospho)
nSFGLAPAAPLQVHAPLs	A0A0G2K2V2	N-Term(iTRAQ4plex); S18(Phospho); K37(iT
eEcPRPMsIsPPDFsPk	P15205;F1LRL9	N-Term(iTRAQ4plex); C3(Carbamidomethyl);
sAIDsDIGETEDVQAGGTI	D3ZXD2	N-Term(iTRAQ4plex); S5(Phospho)
gEEHsPLER	P54287	N-Term(iTRAQ4plex); S5(Phospho)
gPQFGSEVELRHs	Q8CJ04	N-Term(iTRAQ4plex); S13(Phospho)
qEkEPTkSPESPssPVSSE	Q5QD51	N-Term(iTRAQ4plex); K3(iTRAQ4plex); K7(iT
sPPLIGSESAYEDFLSADI	P15205;F1LRL9	N-Term(iTRAQ4plex); S1(Phospho); K20(iTR
vSEEAESQQWDTSkGDQ	G3V6S0	N-Term(iTRAQ4plex); K14(iTRAQ4plex); S19
vALLLLDQGAsPHAAAk	A0A0G2K1R9;A0A0G2K1(	N-Term(iTRAQ4plex); S11(Phospho); K17(iT
iAcEEEFsDSDEEGEGGR	Q4QQW4	N-Term(iTRAQ4plex); C3(Carbamidomethyl);
iLDQmPATPSsPMYVD	F1LP57	N-Term(iTRAQ4plex); M5(Oxidation); S11(Ph
sLDAEPSVPSAAkPAsPEI	D3ZV33	N-Term(iTRAQ4plex); K13(iTRAQ4plex); S16
gRPPAEkLsPNPPk	E9PTG1	N-Term(iTRAQ4plex); K7(iTRAQ4plex); S9(P

tFQQIQEEEDDDYPGsYS	B5DEK0	N-Term(iTRAQ4plex); S16(Phospho); K34(iT
dGsPPPAFkPEPPk	Q3ZB99	N-Term(iTRAQ4plex); S3(Phospho); K9(iTRA
sAPSsAPSTPLSTDAPEFl	M0R970	N-Term(iTRAQ4plex); S5(Phospho); K23(iTR
hDTSASTQsTPASSR	G3V6S0	N-Term(iTRAQ4plex); S9(Phospho)
rYsPsPPPk	B2RYB3;A0A0G2K4F6	N-Term(iTRAQ4plex); S3(Phospho); S5(Phos
ekEDAHADPEIQPMkEDD	Q6QRP0	N-Term(iTRAQ4plex); K2(iTRAQ4plex); K15(
gQAYAtEWSDDESGNPF(	A0A0G2JWR2	N-Term(iTRAQ4plex); T6(Phospho); K34(iTR
vMLGEtNPADSkPGTIR	P19804	N-Term(iTRAQ4plex); T6(Phospho); K12(iTR
sQPELSTGk	F1LMT5	N-Term(iTRAQ4plex); S1(Phospho); K9(iTRA
tSSESIVSVPASSTSGsPS	A0A0G2JTB5	N-Term(iTRAQ4plex); S17(Phospho)
tLVLVsPGDMk	D3ZAU7	N-Term(iTRAQ4plex); S6(Phospho); K11(iTR
lSsPPR	F1M7S2	N-Term(iTRAQ4plex); S3(Phospho)
skPsAASPkPGALk	P15205;F1LRL9	N-Term(iTRAQ4plex); K2(iTRAQ4plex); S4(P
eSALSRAsDESLR	Q7TP93	N-Term(iTRAQ4plex); S8(Phospho)
nmTVEQLLTGsPtSPTVEF	F1M403	N-Term(iTRAQ4plex); M2(Oxidation); S11(Ph
dPLEEIQPLNDLDEGQPP	D3ZL30	N-Term(iTRAQ4plex); S22(Phospho)
tEsRDVDGRPEIQR	D4A110	N-Term(iTRAQ4plex); S3(Phospho)
eDSPLSSPSsQPGTPDEQ	A0A0G2K774	N-Term(iTRAQ4plex); S10(Phospho); K23(iT
eSsPtYSPGFSDSTSGAk	P15205;F1LRL9	N-Term(iTRAQ4plex); S3(Phospho); T5(Phos
ePAPGDPGcGPGELRPP	A0A0G2JZ50	N-Term(iTRAQ4plex); C9(Carbamidomethyl);
vEEEQEADEEDVsEEETE	Q52KJ9	N-Term(iTRAQ4plex); S13(Phospho); K25(iT
gsDTGSMLk	A0A0G2KB60	N-Term(iTRAQ4plex); S2(Phospho); K9(iTRA
tESVTSGPLSPEGsPskSF	F8WFS9	N-Term(iTRAQ4plex); S14(Phospho); S16(Ph
smEDPFFVVk	Q63635	N-Term(Acetyl); S1(Phospho); M2(Oxidation)
lLGLNELDDLLGkEsPSk	F1M2K6	N-Term(iTRAQ4plex); K13(iTRAQ4plex); S15
sDANTDLIGGsPk	P53987	N-Term(iTRAQ4plex); S11(Phospho); K13(iT
fSHNYLsDSDTEAk	D3ZFD0	N-Term(iTRAQ4plex); S7(Phospho); K14(iTR
sEsPPAELPSLR	A0A0A0MXY4	N-Term(iTRAQ4plex); S3(Phospho)
eQESSGEEDNDLsPEER	A0A0H2UHA0	N-Term(iTRAQ4plex); S13(Phospho)
sPsAVGPSGGGLR	Q9JKM5	N-Term(iTRAQ4plex); S3(Phospho)
eVPSkEEQsPVk	P15205;F1LRL9	N-Term(iTRAQ4plex); K5(iTRAQ4plex); S9(P
gVSDEDtDEEkETLk	D3ZX42	N-Term(iTRAQ4plex); T7(Phospho); K11(iTR
eTEALLQSIGISPEPPLVP	A0A173DW30	N-Term(iTRAQ4plex); S23(Phospho); K27(iT
sHsPLPAPPSk	G3V6S8	N-Term(iTRAQ4plex); S3(Phospho); K11(iTR
eAEGsPETEkk	D4A1J3	N-Term(iTRAQ4plex); S5(Phospho); K10(iTR
eTESAPGsPR	F1M062	N-Term(iTRAQ4plex); S8(Phospho)
sGPFGQIFRPDNFVFGQS	P85108;B4F7C2;P69897;(	N-Term(iTRAQ4plex); S1(Phospho); K26(iTR
aDsVEQQDGAALEk	P34926	N-Term(iTRAQ4plex); S3(Phospho); K14(iTR
gSDsEDEVLR	Q641X2	N-Term(iTRAQ4plex); S4(Phospho)
sGTSTPTTPGSTAITPGtP	A0A0U1RRX4;F1MAQ5	N-Term(iTRAQ4plex); T18(Phospho)
skDsGLPSQGLSFk	Q63622	N-Term(iTRAQ4plex); K2(iTRAQ4plex); S4(P
vPsPGPWGPEAGAGADS	D3ZIM5	N-Term(iTRAQ4plex); S3(Phospho)
eAAGGNDssGATSPINPA	Q05695	N-Term(iTRAQ4plex); S8(Phospho); S9(Phos
eDLVcSAALHsPQEsPVGl	F1LQN3	N-Term(iTRAQ4plex); C5(Carbamidomethyl);
fcDYGkAtGAEEYAQQDV\	P13233	N-Term(iTRAQ4plex); C2(Carbamidomethyl);
eHQIsPGDFPNLk	Q8R491	N-Term(iTRAQ4plex); S5(Phospho); K13(iTR
sSDWsSEEEEPVR	Q6PST4	N-Term(iTRAQ4plex); S5(Phospho)
lTFScLGGsDNFk	P83868	N-Term(iTRAQ4plex); C5(Carbamidomethyl);
lRLsPSPTSQR	G3V8L3	N-Term(iTRAQ4plex); S4(Phospho)
eDsPQSHVLVMk	P06686	N-Term(iTRAQ4plex); S3(Phospho); K12(iTR
aGGGAGGsGGsGPSAILF	F1M7K7	N-Term(iTRAQ4plex); S8(Phospho); S11(Pho
gRGsTISEAEcHQAR	D3ZB78	N-Term(iTRAQ4plex); S4(Phospho); C11(Car
qDsDVGkEPk	A0A0G2K0D3	N-Term(iTRAQ4plex); S3(Phospho); K7(iTRA
eHSGLsPQDDTNSGMSIF	A0A0G2JZG4	N-Term(iTRAQ4plex); S6(Phospho)
eESEEsDEDmGFGLFD	P19945;D4A4D5	N-Term(iTRAQ4plex); S6(Phospho); M10(Ox

nLEPcDNtPIIDNITPVVDG	A0A0G2K070	N-Term(iTRAQ4plex); C5(Carbamidomethyl);
sASsPkPDTk	Q5FVI4	N-Term(iTRAQ4plex); S4(Phospho); K6(iTRA
tPPATSQAsDPEEk	A0A0G2KBA5	N-Term(iTRAQ4plex); S9(Phospho); K14(iTR
rAsGPGtPPAAEPSVLTTk	G3V6N1	N-Term(iTRAQ4plex); S3(Phospho); T7(Phos
iHLPLNYPPGsPDLGR	Q00A22	N-Term(iTRAQ4plex); S11(Phospho)
sGsPVkR	Q9EQS0	N-Term(Acetyl); S3(Phospho); K6(iTRAQ4ple
eAELGPENtAAREESEAE	P34926;Q63330	N-Term(iTRAQ4plex); T9(Phospho); K20(iTR
gQEEQsPGLEDkDLDFkD	Q64548	N-Term(iTRAQ4plex); S6(Phospho); K12(iTR
aSGQSFEVILksPsDLSPE	A0A0G2K8P5	N-Term(iTRAQ4plex); K11(iTRAQ4plex); S12
fSIsPDSLS	P70673	N-Term(iTRAQ4plex); S4(Phospho)
kDsEEEVSLLGSQDIEEGI	Q4G036	N-Term(iTRAQ4plex); K1(iTRAQ4plex); S3(P
rsSDGsLSHEDLAk	D3ZZ81	N-Term(iTRAQ4plex); S2(Phospho); S6(Phos
yDLDFksPADPSR	P07323	N-Term(iTRAQ4plex); K6(iTRAQ4plex); S7(P
eREkEIsDDEAEEEk	P34058	N-Term(iTRAQ4plex); K4(iTRAQ4plex); S7(P
mDsDsGEQSEGEPGTAA	F1LX20	N-Term(Acetyl); M1(Oxidation); S3(Phospho)
dYcsEEEDIT	D3ZU88	N-Term(iTRAQ4plex); C3(Carbamidomethyl);
iLsFGSWEDLsP	Q923V4	N-Term(iTRAQ4plex); S3(Phospho); S11(Pho
tLHsPPLQLR	P0C644	N-Term(iTRAQ4plex); S4(Phospho)
dLTGGGDLTsPDASASSd	O35867	N-Term(iTRAQ4plex); S10(Phospho); C18(Ca
tTQSLQDFPVADsEEEAE	Q5U2Y6	N-Term(iTRAQ4plex); S13(Phospho); K23(iT
nMTVEQLLTGSPtsPTVEI	F1M403	N-Term(iTRAQ4plex); T13(Phospho); S14(Ph
aSDPSALLtPQEEDcAEGI	F1M3W5	N-Term(iTRAQ4plex); T9(Phospho); C15(Car
tLTDEVNsPDADR	D4A510	N-Term(iTRAQ4plex); S8(Phospho)
tESVTSGPLSPEGsPSk	F8WFS9	N-Term(iTRAQ4plex); S14(Phospho); K17(iT
eAAAAPAVAPGPAQPGH	Q3MUI2	N-Term(iTRAQ4plex); T25(Phospho); S26(Ph
eTQTPLATHQsEEDEEDE	A0A173DW30	N-Term(iTRAQ4plex); S11(Phospho); M20(O
dTSSNDINGGVEPTPSNV	D3ZUY8	N-Term(iTRAQ4plex); T20(Phospho); S22(Ph
vSMILQsPAFcEELESMIQ	A0A0G2JSM7;D3ZZ99	N-Term(iTRAQ4plex); S7(Phospho); C11(Car
gEQVsQNGLPAEQGSPR	Q9WUX0	N-Term(iTRAQ4plex); S5(Phospho)
eGLRPGDTTsTFcGTPNY	Q9R1X8	N-Term(iTRAQ4plex); S10(Phospho); C13(Ca
sLTNPtPIQQQLR	A0A0G2JTA7	N-Term(iTRAQ4plex); T6(Phospho)
qNsLNDEGcQSDLEDNLS	A6Y7S3	N-Term(iTRAQ4plex); S3(Phospho); C9(Carb
kGSPVSEIGWETPPPEsP	D3ZWC6	N-Term(iTRAQ4plex); K1(iTRAQ4plex); S17(
sSsVTsIDkESR	Q9WU70	N-Term(iTRAQ4plex); S3(Phospho); S6(Phos
tSTAPSsPGVDSVPLQR	F1M787	N-Term(iTRAQ4plex); S7(Phospho)
aLSDsYELSTDLQDk	A0A0G2JZX5	N-Term(iTRAQ4plex); S5(Phospho); K15(iTR
rEIssSPTSk	A0A0G2K2M9	N-Term(iTRAQ4plex); S4(Phospho); S5(Phos
kAPQSLEMMAIESPPPSE	D3ZE17	N-Term(iTRAQ4plex); K1(iTRAQ4plex); C24(
rPEAAsPQk	F1LPP6	N-Term(iTRAQ4plex); S6(Phospho); K9(iTRA
sPsPLQAPEk	F1LTW9	N-Term(iTRAQ4plex); S3(Phospho); K10(iTR
aSGDGEsLDEDSEFTLAS	Q5U2Z3	N-Term(iTRAQ4plex); S7(Phospho)
iVPSLLFNLQHVEEAESRs	F1LTW9	N-Term(iTRAQ4plex); S19(Phospho); S21(Ph
sPAEVksPAEAk	F1LRZ7	N-Term(iTRAQ4plex); K6(iTRAQ4plex); S7(P
aGSRLsAEDR	A0A0G2K4U8	N-Term(iTRAQ4plex); S6(Phospho)
lRTDsQSEAVAIQAIR	Q8CGU4	N-Term(iTRAQ4plex); S5(Phospho)
eALsDDEDEALYQk	D4A9F1	N-Term(iTRAQ4plex); S4(Phospho); K14(iTR
nLVPmDPNGLSDPyVk	F1LS36;F1LS42	N-Term(iTRAQ4plex); M5(Oxidation); Y14(Ph
lRPGsPEPPPR	A0A0G2JU85	N-Term(iTRAQ4plex); S5(Phospho)
aQsLPLPSLAGPDASVGk	A0A140UHX6	N-Term(iTRAQ4plex); S3(Phospho); K18(iTR
dPIEDINsPEHIQR	A0A0A0MXV8	N-Term(iTRAQ4plex); S8(Phospho)
eDMAALEkDYEEVGVDsV	P68370	N-Term(iTRAQ4plex); K8(iTRAQ4plex); S17(
vPsDSSLGTPDGRPELR	P97531	N-Term(iTRAQ4plex); S3(Phospho)
hFIEDWsEEDVSk	A0A0H2UHE3	N-Term(iTRAQ4plex); S7(Phospho); K13(iTR
gSsLLDR	F1LUY5	N-Term(iTRAQ4plex); S3(Phospho)
gHkHEDGtQSDSEDPLAk	D4A1G8	N-Term(iTRAQ4plex); K3(iTRAQ4plex); T8(P

aFsEDEALAQQDSk	F1LR18	N-Term(iTRAQ4plex); S3(Phospho); K14(iTR
sGSLsPcPPGDTLPWNLF	D3ZHV2	N-Term(iTRAQ4plex); S5(Phospho); C7(Carb
dQFsPDDFLGR	D3ZV52;M0R7A6	N-Term(iTRAQ4plex); S4(Phospho)
kPPEFEGGESLsSGNLcQ	A0A0G2K7H9	K1(iTRAQ4plex); N-Term(iTRAQ4plex); S12(
iNFDsNSAYR	B2RZ24	N-Term(iTRAQ4plex); S5(Phospho)
tHTsPPR	D3ZEI4	N-Term(iTRAQ4plex); S4(Phospho)
lPQSGSQSSMIPsPPEDD	D3ZX42	N-Term(iTRAQ4plex); S13(Phospho); K36(iT
sGDNSSSSLGDVVTGTR	G3V6K8	N-Term(iTRAQ4plex); S1(Phospho)
gTGPGGQLQDLDcsssDD	Q3KRC3	N-Term(iTRAQ4plex); C13(Carbamidomethyl
gSGTGHPGTGVPQAPDs	D3ZWC6	N-Term(iTRAQ4plex); S17(Phospho)
sWSTAtSPLGGERPFR	F1M392	N-Term(iTRAQ4plex); T6(Phospho)
hGGGIVADLsQQsLkDGV	Q5HZV9	N-Term(iTRAQ4plex); S10(Phospho); S13(Ph
lLDAEDVDVPsPDEk	D3ZHV2	N-Term(iTRAQ4plex); S11(Phospho); K15(iT
asPAEGGcSIR	Q6P6S0	N-Term(iTRAQ4plex); S2(Phospho); C8(Carb
dSFSQESSPSsPSDLAk	Q9JKS6;D3Z9C7	N-Term(iTRAQ4plex); S11(Phospho); K17(iT
aPsDDGsLk	D3ZFD0	N-Term(iTRAQ4plex); S3(Phospho); S7(Phos
qLEYQQFEDDkLsQksSsS	G3V874;A0A0G2K1Q9	N-Term(iTRAQ4plex); K11(iTRAQ4plex); S13
sDsNAsFLR	A0A0G2K6R9	N-Term(iTRAQ4plex); S3(Phospho); S6(Phos
dLsPQQMVVR	F1M9C9	N-Term(iTRAQ4plex); S3(Phospho)
kSLDsDESEDEDDDYQQI	Q62785	N-Term(iTRAQ4plex); K1(iTRAQ4plex); S5(P
sATSGDIWPGLSAYDNsF	B1WC16	N-Term(iTRAQ4plex); S17(Phospho)
hQIVcINNDLSEEsPDESE	D3ZYK9	N-Term(iTRAQ4plex); C5(Carbamidomethyl);
scYNLTNPSDsGR	F1LUY5	N-Term(iTRAQ4plex); C2(Carbamidomethyl);
sETSSNPSsPEIcPNk	D3ZPI4	N-Term(iTRAQ4plex); S9(Phospho); C13(Car
aDLPVITIDPAsPQsPEsVI	P26431	N-Term(iTRAQ4plex); S12(Phospho); S15(Ph
dkTESVTSGPLSPEGSPS	F8WFS9	N-Term(iTRAQ4plex); K2(iTRAQ4plex); K19(
nDsWGSFDLR	D3ZC82	N-Term(iTRAQ4plex); S3(Phospho)
kTSFDQDsDVDIFPSDFTS	Q14TE9	N-Term(iTRAQ4plex); K1(iTRAQ4plex); S8(P
qLAQEAHNIYHEFLSSQA	Q5BKB9	N-Term(iTRAQ4plex); S20(Phospho)
eAELGPENTAAREEsEAE	P34926;Q63330	N-Term(iTRAQ4plex); S15(Phospho); K20(iT
nAPPAGDEPLAETEsESE	A0A0G2K1U5	N-Term(iTRAQ4plex); S15(Phospho); K31(iT
rGPAEGEEAVPAERsPEc	A0A0G2K4R1	N-Term(iTRAQ4plex); S15(Phospho); C18(Ca
iEIIQPLLDMAAGTSNAAP	F1M994	N-Term(iTRAQ4plex); S30(Phospho); K36(iT
aTTPPNQGRPDsPVYANI	A0A0G2K1Z2	N-Term(iTRAQ4plex); S12(Phospho); K22(iT
aNsFVGTAQYVSPELLTE	O55173	N-Term(iTRAQ4plex); S3(Phospho); K19(iTR
sMEDLVNEFDEk	P97577	N-Term(iTRAQ4plex); S1(Phospho); K12(iTR
sVSTPSEAGsQDsGDGAV	D3ZU74	N-Term(iTRAQ4plex); S10(Phospho); S13(Ph
qEtVDcLkk	P11275;P15791	N-Term(iTRAQ4plex); T3(Phospho); C6(Carb
iDIsPSTFR	Q5M7V8	N-Term(iTRAQ4plex); S4(Phospho)
qRPQEAPEAEPGsPR	F1M8S4	N-Term(iTRAQ4plex); S13(Phospho)
tLGsPEAQk	D3ZN16	N-Term(iTRAQ4plex); S4(Phospho); K9(iTRA
tVDEQEDmDLQIsPDRk	A0A0G2K6R9;F1M9N9	N-Term(iTRAQ4plex); M8(Oxidation); S13(Ph
gNSELsEAENMDTPPDDE	A0A0G2K2T6	N-Term(iTRAQ4plex); S6(Phospho); K20(iTR
lADYVVPGcQTVLsPTASE	F1LZX5	N-Term(iTRAQ4plex); C9(Carbamidomethyl);
vLGFcQLNLPsGk	P06686	N-Term(iTRAQ4plex); C5(Carbamidomethyl);
sVTSNQSDGTQEScEsPD	D4ACZ5	N-Term(iTRAQ4plex); C14(Carbamidomethyl
ssPDNSPVHGMLR	A0A0G2K104;F1LNC7	N-Term(iTRAQ4plex); S2(Phospho)
vRsLPEIDGLSk	F1LLX6;F1LWT1	N-Term(iTRAQ4plex); S3(Phospho); K12(iTR
aAQPPTEtAESSQAEEEkI	P07936	N-Term(iTRAQ4plex); T8(Phospho); K18(iTR
sAIYGFGDkGsLR	Q6AY30	N-Term(iTRAQ4plex); K9(iTRAQ4plex); S11(
vESTsGSEcENHTk	M0R9R0	N-Term(iTRAQ4plex); S5(Phospho); C9(Carb
dIPEPPPVcYPWLPATPSA	F1LXQ7	N-Term(iTRAQ4plex); C9(Carbamidomethyl);
fLTEELsLDQDR	P80254	N-Term(iTRAQ4plex); S7(Phospho)
lPsISDLDSIFGPVLsPk	F1M1Y0	N-Term(iTRAQ4plex); S3(Phospho); S16(Pho
eDTkMsIsEGTVSDk	P15205;F1LRL9	N-Term(iTRAQ4plex); K4(iTRAQ4plex); S6(P

Information Classification: General

aELAPETTEtPVEtPETVQ	D3ZFB6	N-Term(iTRAQ4plex); T10(Phospho); T14(Ph
vYcVEGTPINFSTATSLSD	G3V8Q9	N-Term(iTRAQ4plex); C3(Carbamidomethyl);
tLsFDLQQR	F1LQY6	N-Term(iTRAQ4plex); S3(Phospho)
sFGSGTELGHWVTtPPDI	Q5EB62	N-Term(iTRAQ4plex); T14(Phospho)
sNsDITISDIDTEDVLDQH	F1M8G8	N-Term(iTRAQ4plex); S3(Phospho)
eVAEVsPLSAANMSIAVk	A0A0G2JXK1	N-Term(iTRAQ4plex); S6(Phospho); K18(iTR
lGGDGsPTQVDVSQFGS	P15205;F1LRL9	N-Term(iTRAQ4plex); S6(Phospho); K19(iTR
aLsPVIPIIPR	A0A0G2JVD6	N-Term(iTRAQ4plex); S3(Phospho)
sPDREDGEEAPAGGFLF	G3V9G5	N-Term(iTRAQ4plex); S1(Phospho); K18(iTR
scYNLTNPSDsGRGsSLL	F1LUY5	N-Term(iTRAQ4plex); C2(Carbamidomethyl);
sNsTETLsPAk	M0R5P8	N-Term(iTRAQ4plex); S3(Phospho); S8(Phos
qQGVLQSsPk	G3V864	N-Term(iTRAQ4plex); S8(Phospho); K10(iTR
dLLsPESGSLVR	G3V6M0	N-Term(iTRAQ4plex); S4(Phospho)
aRPSQLPEQSSSAQQNG	F1M8L1	N-Term(iTRAQ4plex); S23(Phospho); K29(iT
sQLQEsPPR	D3ZDV9	N-Term(iTRAQ4plex); S6(Phospho)
msPFPLTSMDk	M0R423	N-Term(iTRAQ4plex); S2(Phospho); K11(iTR
eTDPsPEERDk	A0A0G2K0F3	N-Term(iTRAQ4plex); S5(Phospho); K11(iTR
eSPPQPPADDGsEEPGs	D4A1Q2	N-Term(iTRAQ4plex); S12(Phospho); S17(Ph
kSsQDSDTPAQASPPEE	F1LQ24	N-Term(iTRAQ4plex); K1(iTRAQ4plex); S3(P
eEDEELLGtNDSDETELL	Q6P6G5	N-Term(iTRAQ4plex); T9(Phospho); K22(iTR
sPVDSSVLEGVk	F1LMT5	N-Term(iTRAQ4plex); S1(Phospho); K12(iTR
sPTSPVPVPVm	P63319	N-Term(iTRAQ4plex); S1(Phospho); M11(Ox
fLEESVAmsPEER	D4ABI6	N-Term(iTRAQ4plex); M8(Oxidation); S9(Pho
eTPSSSPVsPQESPk	F1M820	N-Term(iTRAQ4plex); S9(Phospho); K15(iTR
ySEPRPWsSTDSDSSLR	F1LNT3	N-Term(iTRAQ4plex); S8(Phospho)
tTHYGSLPQksQR	P02688	N-Term(iTRAQ4plex); K10(iTRAQ4plex); S11
eTISAIDtSPkENtPVR	D4A4M0	N-Term(iTRAQ4plex); T8(Phospho); K11(iTR
aSPELPsPPR	D3ZHG4	N-Term(iTRAQ4plex); S7(Phospho)
kRSPtENVNTPVGk	A0A0G2KA27	N-Term(iTRAQ4plex); K1(iTRAQ4plex); T5(P
sMLQtPPDQNLSGSk	A0A0G2K2M9	N-Term(iTRAQ4plex); T5(Phospho); K15(iTR
rGTGGVDTAATGsVFDIS	Q5BJT9	N-Term(iTRAQ4plex); S13(Phospho)
dVMSDETNNEETEsPSQ	F1LRL9	N-Term(iTRAQ4plex); S14(Phospho); K24(iT
lTFDTTFsPNTGk	P81155	N-Term(iTRAQ4plex); S8(Phospho); K13(iTR
gRPSsPQk	O35867	N-Term(iTRAQ4plex); S5(Phospho); K8(iTRA
ePEPAPPYHsPEDSR	F1M6X3	N-Term(iTRAQ4plex); S10(Phospho)
qSYGESPQLLsPTPTEGD	A0A0G2JTB5	N-Term(iTRAQ4plex); S11(Phospho)
qLQALTcDLEsLR	P47819	N-Term(iTRAQ4plex); C7(Carbamidomethyl);
tTGLLPVNNEDQQsPER	D4A3J9	N-Term(iTRAQ4plex); S14(Phospho)
iTsEGGDGATk	P12839	N-Term(iTRAQ4plex); S3(Phospho); K11(iTR
yFDsGDYNMAk	P60841	N-Term(iTRAQ4plex); S4(Phospho); K11(iTR
aEEVMLAEEDkNAEEksP	Q02294	N-Term(iTRAQ4plex); K11(iTRAQ4plex); K16
dGTAPPPQSPSSPGSGQ	G3V9N7	N-Term(iTRAQ4plex); S22(Phospho)
eEPPAPksPPk	D3ZAZ1	N-Term(iTRAQ4plex); K7(iTRAQ4plex); S8(P
ekEVVPDsPGDk	P34926;Q63330	N-Term(iTRAQ4plex); K2(iTRAQ4plex); S8(P
dPsPVPSFGEGTk	Q6AYE6	N-Term(iTRAQ4plex); S3(Phospho); K13(iTR
aEDDGGEsEGDAsEkDA	D4A997	N-Term(iTRAQ4plex); S8(Phospho); S13(Pho
tPPGSGEPPksGER	D4A1Q2	N-Term(iTRAQ4plex); K10(iTRAQ4plex); S11
eTEENPAGAcENQGsPLk	D3ZMW5	N-Term(iTRAQ4plex); C10(Carbamidomethyl
eLSPGsGPGETR	A0A0G2K162	N-Term(iTRAQ4plex); S6(Phospho)
aPQTSsPPPVR	B2RYB3;A0A0G2K4F6	N-Term(iTRAQ4plex); S6(Phospho)
qSsAPASPAASAAGLAGC	P11730	N-Term(iTRAQ4plex); S3(Phospho); K21(iTR
sSsLDMNR	A0A0G2KB70	N-Term(iTRAQ4plex); S3(Phospho)
lSGPGGSGsFR	G3V8Q2	N-Term(iTRAQ4plex); S9(Phospho)
vAIIRtPPk	A0A0U1RRX4;F1MAQ5	N-Term(iTRAQ4plex); T6(Phospho); K9(iTRA
sPsPTLGESLAPR	D3ZNS1	N-Term(iTRAQ4plex); S1(Phospho); S3(Phos

Information Classification: General

tLSDVEDQkELAsPVsPEL	A0A0G2JU89	N-Term(iTRAQ4plex); K9(iTRAQ4plex); S13(
qLHIEGASLELsDDDTESk	G3V9Y1	N-Term(iTRAQ4plex); S12(Phospho); K19(iT
aVSsPTVSR	D3ZQL7	N-Term(iTRAQ4plex); S4(Phospho)
rHPPtPPDPSGGLPR	Q6MFX8	N-Term(iTRAQ4plex); T5(Phospho)
gPDsGAEVEkEk	P34926;Q63330	N-Term(iTRAQ4plex); S4(Phospho); K10(iTR
sPSSLSANITSsPk	D3ZBB7	N-Term(iTRAQ4plex); S12(Phospho); K14(iT
sRESLNVDVVk	A0A140TAB3	N-Term(iTRAQ4plex); S1(Phospho); K11(iTR
lQTHDESSLPLPQPsPFm	A0A0G2KAV8	N-Term(iTRAQ4plex); S15(Phospho); M18(O
rRDPsLEEIQk	P63043	N-Term(iTRAQ4plex); S5(Phospho); K11(iTR
gGSLGcQTETDSDTQSPF	Q9JKS6;D3Z9C7	N-Term(iTRAQ4plex); C6(Carbamidomethyl);
nLESGRsPsEEEEDGQLV	P0C6P5	N-Term(iTRAQ4plex); S7(Phospho); S9(Phos
vVSStSEEEEAFTEk	A0A0G2JZ50	N-Term(iTRAQ4plex); T5(Phospho); K15(iTR
aAsPPAsASDLIEQQQk	G3V8E2	N-Term(iTRAQ4plex); S3(Phospho); S7(Phos
gNAEGssDEEGkLVIDEPA	Q8VHK7	N-Term(iTRAQ4plex); S6(Phospho); S7(Phos
nVEDTDGSsPEDLMAALT	Q6RJR6	N-Term(iTRAQ4plex); S9(Phospho); K23(iTR
lDNVGEARsPQDR	Q63472	N-Term(iTRAQ4plex); S9(Phospho)
eSAPADsPPEEGk	Q3ZB98	N-Term(iTRAQ4plex); S7(Phospho); K13(iTR
eSTEVQSLsLEEGEMETD	Q5QD51	N-Term(iTRAQ4plex); S9(Phospho); K21(iTR
fStSGLSISGLNPLPNPSYI	P23565;G3V8Q2	N-Term(iTRAQ4plex); T3(Phospho)
dSsSTNLESMDTS	P97536	N-Term(iTRAQ4plex); S3(Phospho)
gVNFAEEPMRsDsENGEE	P12369	N-Term(iTRAQ4plex); S11(Phospho); S13(Ph
eTDYPAGEELsDSGQVDF	E9PSJ4	N-Term(iTRAQ4plex); S11(Phospho)
skDkEDLVcSAALHsPQES	F1LQN3	N-Term(iTRAQ4plex); K2(iTRAQ4plex); K4(iT
vFsQENVcDR	D3ZBU7	N-Term(iTRAQ4plex); S3(Phospho); C8(Carb
gsPSGLAPILR	G3V8P8	N-Term(iTRAQ4plex); S2(Phospho)
dVEDMELsDVEDDGSk	A0A0G2JTD1	N-Term(iTRAQ4plex); S8(Phospho); K16(iTR
dLSPPLNGSTVScSPDRR	P34926	N-Term(iTRAQ4plex); C13(Carbamidomethyl
aSNSLIAGLsQDDEDPPLP	Q6AYG3	N-Term(iTRAQ4plex); S10(Phospho); C30(Ca
hLsVPNIIITPPtPTGMGLS	D3ZSX5	N-Term(iTRAQ4plex); S3(Phospho); T13(Pho
rGTGQsDDSDIWDDTALII	Q2KP10	N-Term(iTRAQ4plex); S6(Phospho); K19(iTR
aAQLVDIELsPVSALR	A0A0G2K219	N-Term(iTRAQ4plex); S10(Phospho)
aEPHSEDDSRDAsPPEPA	F1LN57	N-Term(iTRAQ4plex); S13(Phospho); K26(iT
aAsPPASASDLIEQQQk	G3V8E2	N-Term(iTRAQ4plex); S3(Phospho); K17(iTR
aALSATIFSEGQTLDsPQm	Q5HZE4	N-Term(iTRAQ4plex); S16(Phospho); M19(O
vTVHQVsPQHR	Q498D5	N-Term(iTRAQ4plex); S7(Phospho)
gEPDPERSsPPMLSADDA	Q9QXY2	N-Term(iTRAQ4plex); S9(Phospho)
qAsISGPAPPk	P09951	N-Term(iTRAQ4plex); S3(Phospho); K11(iTR
aELAPETTEtPVETPETVQ	D3ZFB6	N-Term(iTRAQ4plex); T10(Phospho); K32(iT
eQPGDEYsEEEESVLk	A0A0G2K5C7	N-Term(iTRAQ4plex); S8(Phospho); K16(iTR
eDPLLtPVPASENPFR	O35355	N-Term(iTRAQ4plex); T6(Phospho)
gATADDsGGGsPVLAAk	A0A0G2JZ27	N-Term(iTRAQ4plex); S7(Phospho); S11(Pho
sPEFPTPLFSGPLEPVAS	B1WBT4	N-Term(iTRAQ4plex); S35(Phospho)
kEGDGSATTDAAPAtSPk	P07936	N-Term(iTRAQ4plex); K1(iTRAQ4plex); T15(
sAsQDcIETTPGAQEGk	O35431	N-Term(iTRAQ4plex); S3(Phospho); C6(Carb
sPAEAksPVVAksPAEAk	F1LRZ7	N-Term(iTRAQ4plex); S1(Phospho); K6(iTRA
iLDQmPAtPSsPMYVD	F1LP57	N-Term(iTRAQ4plex); M5(Oxidation); T8(Pho
ePAPcVQQPTVEVNALQT	Q3ZB98	N-Term(iTRAQ4plex); C5(Carbamidomethyl);
lSTDDLkTEEEGktDGEGN	Q62861	N-Term(iTRAQ4plex); K7(iTRAQ4plex); K13(
lFQGYsFVAPsILFk	A0A0G2K366	N-Term(iTRAQ4plex); S6(Phospho); S11(Pho
eSkSsDDIDYR	F1M3W5	N-Term(iTRAQ4plex); K3(iTRAQ4plex); S5(P
vAAAGSGPsPPcsPGHDR	Q99M64	N-Term(iTRAQ4plex); S9(Phospho); C12(Car
lPPPPDQLsPEPPLPAAQP	Q66HA5	N-Term(iTRAQ4plex); S9(Phospho); K36(iTR
kLNsDEEGESSGk	Q641X2	N-Term(iTRAQ4plex); K1(iTRAQ4plex); S4(P
kYSScStIFLDDSTVSQPN	F1M4U0	K1(iTRAQ4plex); N-Term(iTRAQ4plex); C5(C
rNEAFVckPDPSPPsPSEE	D3ZWS0	N-Term(iTRAQ4plex); C7(Carbamidomethyl);

rHsTEGEEGDVSDVGSR	A0A0G2K9A9	N-Term(iTRAQ4plex); S3(Phospho)
sPASVksPSEAk	F1LRZ7	N-Term(iTRAQ4plex); K6(iTRAQ4plex); S7(P
lDDGHLNNSLGsPVQADV	B4F7A3	N-Term(iTRAQ4plex); S12(Phospho)
sLPkEEVAsEPEEAASPIT	Q5RJN5	N-Term(iTRAQ4plex); K4(iTRAQ4plex); S9(P
rLsQIGVENTEENR	P09117	N-Term(iTRAQ4plex); S3(Phospho)
kPSPSTsPTVR	D3ZQL6	N-Term(iTRAQ4plex); K1(iTRAQ4plex); S7(P
sTsPPPSPEVWAESR	D4A559	N-Term(iTRAQ4plex); S3(Phospho)
mTScPSDscDFNDPR	D4A3X6	N-Term(iTRAQ4plex); C4(Carbamidomethyl);
nSSNAGVGsPSNTIGR	A0A0G2JTD7	N-Term(iTRAQ4plex); S9(Phospho)
iEPGsSSPR	D3ZLR4	N-Term(iTRAQ4plex); S5(Phospho)
tGMYVATLAGSQsPkPLM	G3V8F1	N-Term(iTRAQ4plex); S13(Phospho); K15(iT
sVEDEMDsPGEEPFYTG(	Q63785	N-Term(iTRAQ4plex); S8(Phospho)
kEPVANQDPVsPSLVQGF	P10980	N-Term(iTRAQ4plex); K1(iTRAQ4plex); S11(
tLNsckVPQIR	Q99JE4	N-Term(iTRAQ4plex); S4(Phospho); C5(Carb
sAELDAsDSSSSSNLSLAI	F1M836	N-Term(iTRAQ4plex); S7(Phospho); K19(iTR
iLSFGsWEDLSP	Q923V4	N-Term(iTRAQ4plex); S6(Phospho)
fAsFIER	P19527	N-Term(iTRAQ4plex); S3(Phospho)
eVItSDELLtHLGNcLLsIkP	Q5BJQ2	N-Term(iTRAQ4plex); T4(Phospho); T10(Pho
iGFGPsPFSLTEGLPk	F1LRZ7	N-Term(iTRAQ4plex); S6(Phospho); K16(iTR
aQTLPTSVVTITSESsPGk	G3V6S0	N-Term(iTRAQ4plex); S16(Phospho); K19(iT
rLsPSAsPPR	B2RYB3;A0A0G2K4F6	N-Term(iTRAQ4plex); S3(Phospho); S7(Phos
gNksPsPPPDGSPAATPE	Q5HZA7	N-Term(iTRAQ4plex); K3(iTRAQ4plex); S4(P
nEksEEEQSSASVk	G3V9R8	N-Term(iTRAQ4plex); K3(iTRAQ4plex); S4(P
kAEsPVk	P12839	N-Term(iTRAQ4plex); K1(iTRAQ4plex); S4(P
sPAEVksPATVksPVEAk	F1LRZ7	N-Term(iTRAQ4plex); K6(iTRAQ4plex); S7(P
vIsPSEDRk	F1M2P8	N-Term(iTRAQ4plex); S3(Phospho); K9(iTRA
nVDHEHLGsR	D3ZCQ9	N-Term(iTRAQ4plex); S9(Phospho)
vksPAEEPLFPAPLLLR	D3ZGN7	N-Term(iTRAQ4plex); K2(iTRAQ4plex); S3(P
iLGAWLAEETSsLR	O35095	N-Term(iTRAQ4plex); S12(Phospho)
gLSDHAsLDGQELGAQSF	B2GUX8	N-Term(iTRAQ4plex); S7(Phospho)
eSVPEFPLsPPkk	A0A096MK73	N-Term(iTRAQ4plex); S9(Phospho); K12(iTR
sPAEVksPAVAk	F1LRZ7	N-Term(iTRAQ4plex); K6(iTRAQ4plex); S7(P
sERENADDsDAETQDHSI	A0A0G2JV78	N-Term(iTRAQ4plex); S9(Phospho)
lRTEsIEEHATPNSAPAQV	F1M3W5	N-Term(iTRAQ4plex); S5(Phospho)
dIsPEEIDLk	F1M820	N-Term(iTRAQ4plex); S3(Phospho); K10(iTR
mLDPSsSEEESDEILEEE	F1LLX6	N-Term(Acetyl); S6(Phospho); K21(iTRAQ4p
vILHPTPNsPk	F1LLX6	N-Term(iTRAQ4plex); S9(Phospho); K11(iTR
gQGTLQHsPVGPAGPASI	A0A0G2JWP1	N-Term(iTRAQ4plex); S8(Phospho)
sLMsSPEDLTk	P15205;F1LRL9	N-Term(iTRAQ4plex); S4(Phospho); K11(iTR
dVsPsPETETAk	A0A0G2JW88	N-Term(iTRAQ4plex); S3(Phospho); S5(Phos
sPSLSPSPPsPIEk	P15205;F1LRL9	N-Term(iTRAQ4plex); S10(Phospho); K14(iT
kVAELEEEQsQGsSsYsDV	P34926;Q63330	K1(iTRAQ4plex); N-Term(iTRAQ4plex); S10(
aHsVDVEk	D3ZZM9	N-Term(iTRAQ4plex); S3(Phospho); K8(iTRA
qEPEsEEEEEEkQEkEEk	F1LT30	N-Term(iTRAQ4plex); S5(Phospho); K12(iTR
vAELEEEQsQGsSSYSDW	P34926;Q63330	N-Term(iTRAQ4plex); S9(Phospho); S12(Pho
aTsPFNNVGEVQVPEMSI	M0R963	N-Term(iTRAQ4plex); S3(Phospho); K20(iTR
ssPSSPSPTLGR	D3ZXD8	N-Term(iTRAQ4plex); S2(Phospho)
sTPVRsPGGSTMMk	Q920Q0	N-Term(iTRAQ4plex); S6(Phospho); K14(iTR
sDkGHsPEDDWTEFSSEE	A0A0G2K1R9	N-Term(iTRAQ4plex); K3(iTRAQ4plex); S6(P
eLDDISLtPDPEPTHEDPN	Q5FVJ0	N-Term(iTRAQ4plex); T8(Phospho)
sNsQENVEASHPSQDAk	O35430	N-Term(iTRAQ4plex); S3(Phospho); K17(iTR
iFVGGLsPDTPEEk	Q9JJ54	N-Term(iTRAQ4plex); S7(Phospho); K14(iTR
sLSYsPVER	A0A0G2K2M9	N-Term(iTRAQ4plex); S5(Phospho)
sPAEAksPVEVksPEk	F1LRZ7	N-Term(iTRAQ4plex); K6(iTRAQ4plex); S7(P
aVAGVMITAsHNR	D3Z955	N-Term(iTRAQ4plex); S10(Phospho)

vFksEsEDGMR	O88420	N-Term(iTRAQ4plex); K3(iTRAQ4plex); S4(P
sAGPQSQLLASVIAEkSRs	F8WFS9	N-Term(iTRAQ4plex); K16(iTRAQ4plex); S19
vDRGEsQILScR	P97689	N-Term(iTRAQ4plex); S6(Phospho); C11(Car
tSEDTSsGSPPkk	B1WC49	N-Term(iTRAQ4plex); S7(Phospho); K12(iTR
qDEDsDSEMGPPTEEPEE	F1M1U0	N-Term(iTRAQ4plex); S5(Phospho); K27(iTR
vQPPSPLPSSIDsNssPEE	A0A0G2K6R9;F1M9N9	N-Term(iTRAQ4plex); S13(Phospho); S15(Ph
sSsESYTQSFQSR	Q99M64	N-Term(iTRAQ4plex); S3(Phospho)
sLEPESQQNsAEGsPSEG	D4A0C3	N-Term(iTRAQ4plex); S10(Phospho); S14(Ph
lDGETAsDSESRAETAPP	Q91XU8	N-Term(iTRAQ4plex); S7(Phospho)
sFSEDAVTDSsGsGTLPR	Q5I0K2	N-Term(iTRAQ4plex); S11(Phospho); S13(Ph
nEsSHGGEFGcSMEELR	P11506;D4A8B3	N-Term(iTRAQ4plex); S3(Phospho); C11(Car
kLsEADNR	Q08013	N-Term(iTRAQ4plex); K1(iTRAQ4plex); S3(P
mGPSSSPIPsPsPsPTDSk	P63319	N-Term(iTRAQ4plex); S10(Phospho); S12(Ph
sPATVksPGEAk	F1LRZ7	N-Term(iTRAQ4plex); S1(Phospho); K6(iTRA
gTGGVDTAATGsVFDISN	Q5BJT9	N-Term(iTRAQ4plex); S12(Phospho)
rPsSPAPGPSQPATDSR	B2RZ23	N-Term(iTRAQ4plex); S3(Phospho)
gPRtPsPPPPILEDIILGk	E9PSN4	N-Term(iTRAQ4plex); T4(Phospho); S6(Phos
kIAELEEERsQGstSNSDW	F1LRL9	K1(iTRAQ4plex); N-Term(iTRAQ4plex); S10(
aPSsDEEcFFDLLSk	G3V9X2	N-Term(iTRAQ4plex); S4(Phospho); C8(Carb
tDIAGETEsVSDcADDVSE	A0A0G2JYI0	N-Term(iTRAQ4plex); S9(Phospho); C13(Car
sDDALPDGLsPk	D4A3V4	N-Term(iTRAQ4plex); S10(Phospho); K12(iT
kVELsESEEDkGSk	B2RYB3;A0A0G2K4F6	K1(iTRAQ4plex); N-Term(iTRAQ4plex); S5(P
sQPPsPGPVASPAPPDLD	D4AD05	N-Term(iTRAQ4plex); S5(Phospho)
aVIEsDQEQGR	Q6AY84	N-Term(iTRAQ4plex); S5(Phospho)
aTEEPsGtGSDELIk	G3V8L9	N-Term(iTRAQ4plex); S6(Phospho); T8(Phos
eSPsPSGPEDk	D4A644	N-Term(iTRAQ4plex); S4(Phospho); K11(iTR
qPsEEEIIk	Q5U318	N-Term(iTRAQ4plex); S3(Phospho); K9(iTRA
rGsDELTVPR	Q9QXY2	N-Term(iTRAQ4plex); S3(Phospho)
kGsSEESVDEDR	A0A0G2K6R9;F1M9N9	N-Term(iTRAQ4plex); K1(iTRAQ4plex); S3(P
qADVPAAVTDAAATtPAA	P07936	N-Term(iTRAQ4plex); T15(Phospho); K23(iT
tAGAVAGktPDAsPEPk	A0A0G2JTW5	N-Term(iTRAQ4plex); K8(iTRAQ4plex); T9(P
gASPVPESSLTADLLSVD	A0A0G2K0B6	N-Term(iTRAQ4plex); S27(Phospho); K33(iT
rsGSGYEGsTSWk	Q0ZCA7	N-Term(iTRAQ4plex); S2(Phospho); S9(Phos
sDAEEDGGTGsQDEEDS	P35565	N-Term(iTRAQ4plex); S11(Phospho); K18(iT
ttSPEEAQTQPYIAMDWE	Q5D006	N-Term(iTRAQ4plex); T2(Phospho); K22(iTR
acVVHGsDLk	P06686;P06685	N-Term(iTRAQ4plex); C2(Carbamidomethyl);
nIVTPRtPPPSQGk	P02688	N-Term(iTRAQ4plex); T7(Phospho); K14(iTR
tLAtSTLPLSPTLASNSQG	A0A0G2JSZ0	N-Term(iTRAQ4plex); T4(Phospho)
qsVMNsPTATMNNTHSNI	G3V746	N-Term(iTRAQ4plex); S2(Phospho); S6(Phos
aEAAAAPAVAPGPAQPGI	A1L1I3	N-Term(iTRAQ4plex); S20(Phospho); T22(Ph
gDVTAEEAAGAsPAk	A0A0G2K613	N-Term(iTRAQ4plex); S12(Phospho); K15(iT
sLsVPAAGTAkPPTLPR	D3ZLW4	N-Term(iTRAQ4plex); S3(Phospho); K11(iTR
eQLMNSsLGSGTASLR	P31016	N-Term(iTRAQ4plex); S7(Phospho)
dDDDIDLFGsDDEEESED	B5DEN5	N-Term(iTRAQ4plex); S10(Phospho); K20(iT
sLsTSGESLYHVLGLDk	A0A0G2JX56	N-Term(iTRAQ4plex); S3(Phospho); K17(iTR
gPTEQLVsPEPEVHDIER	P20650	N-Term(iTRAQ4plex); S8(Phospho)
aAsSEAAPHHQPPPESR	Q63638	N-Term(iTRAQ4plex); S3(Phospho)
dPFVLEENEIYGFGLPsPF	A0A0G2JYD4	N-Term(iTRAQ4plex); S17(Phospho)
kGtGDcsDEEVDGkADGA	G3V6P7	N-Term(iTRAQ4plex); K1(iTRAQ4plex); T3(P
gILAADESTGsIAk	P05065	N-Term(iTRAQ4plex); S11(Phospho); K14(iT
fAFSPLsEEDEEDEQkEPM	D4A017	N-Term(iTRAQ4plex); S7(Phospho); K16(iTR
sEQSSmSIEFGQEsPEHS	P15205;F1LRL9	N-Term(iTRAQ4plex); M6(Oxidation); S14(Ph
vTMILQSPsFR	F8WFS9	N-Term(iTRAQ4plex); S9(Phospho)
kRDsDsDADEATPTTTPR	A0A0G2K700	K1(iTRAQ4plex); N-Term(iTRAQ4plex); S4(P
eFLFsPNDSASDIAk	Q5BJT2	N-Term(iTRAQ4plex); S5(Phospho); K15(iTR

Information Classification: General

rGssPGSLEIPk	A0A140TAA3	N-Term(iTRAQ4plex); S3(Phospho); S4(Phos
eAEEESsGGEEEDEDENI	Q07205	N-Term(iTRAQ4plex); S7(Phospho); K24(iTR
dVEMGNsVIEENEMk	A0A0G2K611	N-Term(iTRAQ4plex); S7(Phospho); K15(iTR
sPVkEEAksPAEAk	F1LRZ7	N-Term(iTRAQ4plex); S1(Phospho); K4(iTRA
yIFSPGGLAESPGQLEQQ	F1M7U7	N-Term(iTRAQ4plex); S21(Phospho)
qDEAPEVDkQsGSPENW	F1LWN1	N-Term(iTRAQ4plex); K9(iTRAQ4plex); S11(
qDRQsLGESPR	A0A0G2JW01;F1LWK7	N-Term(iTRAQ4plex); S5(Phospho)
rEEQTDTsDGESVTHHIR	A0A0G2K6Q5	N-Term(iTRAQ4plex); S8(Phospho)
eGDGSATTDAAPATsPkA	P07936	N-Term(iTRAQ4plex); S15(Phospho); K17(iT
dDSHSAEDsEDEkDDHk	A0A0G2K7X3	N-Term(iTRAQ4plex); S9(Phospho); K13(iTR
rDsFSENEk	D3ZEL0	N-Term(iTRAQ4plex); S3(Phospho); K9(iTRA
rNSEGSEAscTEGSLTPSI	F1LXQ7	N-Term(iTRAQ4plex); S9(Phospho); C10(Ca
kEEsAGEEQAAk	P19527	N-Term(iTRAQ4plex); K1(iTRAQ4plex); S4(P
aTTPQLTDVSEDLDQQSL	A0A0G2K1U5	N-Term(iTRAQ4plex); S20(Phospho)
mQPQSSGsLR	F1LVK0	N-Term(iTRAQ4plex); S8(Phospho)
vIsTPPTPIFPISDDIGAIPII	F1LMY3	N-Term(iTRAQ4plex); S3(Phospho); K22(iTR
gLDFEIPPPsPPLNLHELS	Q9QXY2	N-Term(iTRAQ4plex); S10(Phospho); K30(iT
sENSSTQssPEMPTTk	Q5EB70	N-Term(iTRAQ4plex); S8(Phospho); S9(Phos
aESILDkEDkVPLAAVAGS	B3DMA1	N-Term(iTRAQ4plex); K7(iTRAQ4plex); K10(
eSDDkPEIEDVGsDEEEE	P82995	N-Term(iTRAQ4plex); K5(iTRAQ4plex); S13(
dPNAsPGDAGEQAIR	P85972	N-Term(iTRAQ4plex); S5(Phospho)
sSLAETLDStGSLDPQR	Q9WTW8	N-Term(iTRAQ4plex); T10(Phospho)
ekAEEEGGsEEEVGDk	P12839	N-Term(iTRAQ4plex); K2(iTRAQ4plex); S9(P
sQNNFVAILDLPEGEHQY	P80386	N-Term(iTRAQ4plex); S1(Phospho); K19(iTR
iYQFPDcDsDEDEDFkLQI	A0A096MJN4	N-Term(iTRAQ4plex); C7(Carbamidomethyl);
gTVVYGEPITASLGTDGS	P47942	N-Term(iTRAQ4plex); S22(Phospho); K23(iT
iAFDAESEPNNSSGsmEk	A0A0G2K4S6	N-Term(iTRAQ4plex); S15(Phospho); M16(O
hTEPNETtPLTEPEkGPVE	F1LUV9	N-Term(iTRAQ4plex); T8(Phospho); K15(iTR
sPDTSAYcYETMEk	P15205;F1LRL9	N-Term(iTRAQ4plex); S1(Phospho); C8(Carb
lPSESDLLEGEVtDEDEEA	A0A0G2K4N6	N-Term(iTRAQ4plex); T13(Phospho)
tGLcsPEDNSLTPLLDEVV	D3ZT47	N-Term(iTRAQ4plex); C4(Carbamidomethyl);
ncEAPYIPEVsSPTDTSNF	A0A0G2K5Z1	N-Term(iTRAQ4plex); C2(Carbamidomethyl);
lsQETEALGR	Q63083	N-Term(iTRAQ4plex); S2(Phospho)
tSFSVGsDDELGPIR	A0A0G2KB46	N-Term(iTRAQ4plex); S7(Phospho)
qGVDDIEkFEDEGAGFEE	P15205;F1LRL9	N-Term(iTRAQ4plex); K8(iTRAQ4plex); S20(
aSsPSLFSAEGk	M0RBZ7	N-Term(iTRAQ4plex); S3(Phospho); K12(iTR
tHSTSSsIGSGESPFSR	D4ACZ5	N-Term(iTRAQ4plex); S7(Phospho)
fGEAVVVGRPGsGDGDSI	Q923K6	N-Term(iTRAQ4plex); S12(Phospho)
tLsQsSESGTLPSGPPGH	A0A0G2JSU4	N-Term(iTRAQ4plex); S3(Phospho); S5(Phos
aAAEDGEEPksEPETk	Q99PF3	N-Term(iTRAQ4plex); K10(iTRAQ4plex); S11
sLsLESTDR	A0A0G2K5Z1	N-Term(iTRAQ4plex); S3(Phospho)
aAQLQGsPAPEk	F1LW91	N-Term(iTRAQ4plex); S7(Phospho); K12(iTR
aPsPSESSAScTSk	D4A3V4	N-Term(iTRAQ4plex); S3(Phospho); C11(Ca
akFsIGsDEDDSPGLSIk	G3V8P8	N-Term(iTRAQ4plex); K2(iTRAQ4plex); S4(P
gAItPPR	P49621	N-Term(iTRAQ4plex); T4(Phospho)
nVEPPsPTPAAR	F1LSD1	N-Term(iTRAQ4plex); S6(Phospho)
fGsADNIAHLk	D3ZE26	N-Term(iTRAQ4plex); S3(Phospho); K11(iTR
rVGEQDPVPTPAELtSPIR	F1M820	N-Term(iTRAQ4plex); T15(Phospho)
nksNEDQSMGNWQIR	G3V8L3	N-Term(iTRAQ4plex); K2(iTRAQ4plex); S3(P
tLENsPISScDTSDAEGPL	G3V6Z3	N-Term(iTRAQ4plex); S5(Phospho); C10(Ca
sDSPEsDTEREk	D3ZJ92	N-Term(iTRAQ4plex); S6(Phospho); K12(iTR
yGTDNsEETkFIQDYEDk	D3ZVB7	N-Term(iTRAQ4plex); Y1(Phospho); S6(Phos
aLSSGGSItSPPLSPALPk	A0A096MKC0	N-Term(iTRAQ4plex); T9(Phospho); K19(iTR
gSTSstRLHEPEk	P97710	N-Term(iTRAQ4plex); S5(Phospho); T6(Phos
aGNsDSEEEDANER	Q80X08	N-Term(iTRAQ4plex); S4(Phospho)

eSSEStNttIEDEDTk	P11275	N-Term(iTRAQ4plex); T6(Phospho); T8(Phos
vHAYFAPVtPPPSVGGsR	A0A0H2UI14	N-Term(iTRAQ4plex); T9(Phospho); S17(Pho
hsSPHQsEDEEEPR	A2RRU1	N-Term(iTRAQ4plex); S2(Phospho); S7(Phos
sIFREEsPLR	B1WC16	N-Term(iTRAQ4plex); S7(Phospho)
gsTDTDIEELENATYk	Q9EPS1	N-Term(Acetyl); S2(Phospho); K16(iTRAQ4p
sAYQDYDsDSDVPEELkR	F1LSG8	N-Term(iTRAQ4plex); S8(Phospho); K17(iTR
dSsVPGsPSNIVAk	D4A559	N-Term(iTRAQ4plex); S3(Phospho); S7(Phos
aLRPGDLPPsPDDVkR	G3V8D6	N-Term(iTRAQ4plex); S10(Phospho); K15(iT
aLDISAsDDEmARPk	G3V9E4	N-Term(iTRAQ4plex); S7(Phospho); M11(Ox
sPQAQDmPVSIAGGQTG	P34926	S1(Phospho); N-Term(iTRAQ4plex); M7(Oxid
asGDDSPIFEDDESPLcSL	Q8R5H8	N-Term(Acetyl); S2(Phospho); C17(Carbamid
aVDWIFSHIDDLDAEAAm	D3ZVQ0	N-Term(iTRAQ4plex); M18(Oxidation); S21(P
smsHQAAIASQR	B1WBS4	N-Term(iTRAQ4plex); M2(Oxidation); S3(Pho
lTSNQSQGSALDksDDSD	F1M5N7	N-Term(iTRAQ4plex); K13(iTRAQ4plex); S14
asAGsGMEEVR	D3ZBB3	N-Term(iTRAQ4plex); S2(Phospho); S5(Phos
mLsPAMSFLk	Q6P9X1	N-Term(Acetyl); S3(Phospho); K10(iTRAQ4p
eVPSkEEQsPVkAEVAEk	P15205;F1LRL9	N-Term(iTRAQ4plex); K5(iTRAQ4plex); S9(P
eGEGGAGAPDSSsFSPk	P34926	N-Term(iTRAQ4plex); S13(Phospho); K17(iT
rDsVLAASR	A0A0G2KAH9	N-Term(iTRAQ4plex); S3(Phospho)
sQGsTSNSDWMk	F1LRL9	N-Term(iTRAQ4plex); S4(Phospho); K12(iTR
nHsPVQVEADEQHLELQ(	D4AD05	N-Term(iTRAQ4plex); S3(Phospho)
dSQPPsPSLLEEPk	D4A5F4	N-Term(iTRAQ4plex); S6(Phospho); K14(iTR
lDHELsLDR	F1M3W5	N-Term(iTRAQ4plex); S6(Phospho)
sFSsASDGENEER	A0A0G2K0J7	N-Term(iTRAQ4plex); S4(Phospho)
yVTsDELLHk	F1M0J7	N-Term(iTRAQ4plex); S4(Phospho); K10(iTR
sSsISSIDk	D3ZU84	N-Term(iTRAQ4plex); S3(Phospho); K9(iTRA
tTNPFYNtM	F1M4Q5	N-Term(iTRAQ4plex); T8(Phospho)
tEGtPPPPGQPAk	F1LZX5	N-Term(iTRAQ4plex); T4(Phospho); K13(iTR
lEEQDsGGSNTPEQDDTS	Q810W7	N-Term(iTRAQ4plex); S6(Phospho)
kEESEEsDEDMGFGLFD	D4A4D5	N-Term(iTRAQ4plex); K1(iTRAQ4plex); S7(P
aSIsPSNVSALEPQTEmG	F1LQN3	N-Term(iTRAQ4plex); S4(Phospho); M17(Ox
eGEAAGGDHETESTsDkE	O35052	N-Term(iTRAQ4plex); S15(Phospho); K17(iT
aELQEVQITEEkPLLPGQt	A0A0G2JSU4	N-Term(Acetyl); K12(iTRAQ4plex); T19(Phos
sVYSDRDDESPDsSPEE(	A0A0G2K6R9;F1M9N9	N-Term(iTRAQ4plex); S13(Phospho); K19(iT
lLkEGEEPTVYsDDEEPkD	P70580	N-Term(iTRAQ4plex); K3(iTRAQ4plex); S12(
yVAAAFPsAcGk	F1LQJ7	N-Term(iTRAQ4plex); S8(Phospho); C10(Ca
dLPSFLVPSLPsPQk	D4A5F4	N-Term(iTRAQ4plex); S12(Phospho); K15(iT
nVDNAEGsDTDY	A0A0G2JTT6	N-Term(iTRAQ4plex); S8(Phospho)
lITtDsDGTYk	D3Z9D0	N-Term(iTRAQ4plex); T4(Phospho); S6(Phos
kAPsPPPLGLPLR	Q7TP36	N-Term(iTRAQ4plex); K1(iTRAQ4plex); S4(P
sVsNLkPVPVIGSk	Q6AY30	N-Term(iTRAQ4plex); S3(Phospho); K6(iTRA
eTSPSSNNsSEELSSALQ	Q9ESB5	N-Term(iTRAQ4plex); S9(Phospho); K21(iTR
lsSPAQsPSQk	F1M2J2	N-Term(iTRAQ4plex); S2(Phospho); S7(Phos
fNLMSDAPGDsPR	Q00A22	N-Term(iTRAQ4plex); S11(Phospho)
gNVVPsPLPTR	Q9WU49	N-Term(iTRAQ4plex); S6(Phospho)
mLPHAPGVQMQAIPEDA	Q4QQW4	N-Term(iTRAQ4plex); S22(Phospho); K32(iT
aPSLPAPPHsGAPGSPR	Q5BJT1	N-Term(iTRAQ4plex); S10(Phospho)
hQMScDDIEVLSTGTPLQ	D3ZWH2	N-Term(iTRAQ4plex); C5(Carbamidomethyl);
fSTFsGSITGPLYTHR	P12839	N-Term(iTRAQ4plex); S5(Phospho)
gSPVSEIGWETPPPEsPR	D3ZWC6	N-Term(iTRAQ4plex); S16(Phospho)
sGsPMAR	P02688	N-Term(iTRAQ4plex); S3(Phospho)
ltHGDSPSEALIGPLGQVk	Q9Z1X1	N-Term(iTRAQ4plex); T2(Phospho); K19(iTR
dSYVGDEAQsk	P68035;P60711;P63259	N-Term(iTRAQ4plex); S10(Phospho); K11(iT
dFLGsPEDSEk	G3V6I1	N-Term(iTRAQ4plex); S5(Phospho); K11(iTR
iNENsDkEAEVEESSEk	F1MAK1	N-Term(iTRAQ4plex); S5(Phospho); K7(iTRA

sAsEPSLNR	F1M9C3	N-Term(iTRAQ4plex); S3(Phospho)
asGVAVSDGVIk	P45592	N-Term(Acetyl); S2(Phospho); K12(iTRAQ4p
aFTEVFLDDSHsPANR	F1MAF8	N-Term(iTRAQ4plex); S12(Phospho)
lGtPPGGGAAGk	F1LSH6	N-Term(iTRAQ4plex); T3(Phospho); K12(iTR
sATVsPQQPQAQQR	F1M820	N-Term(iTRAQ4plex); S5(Phospho)
aAYFGVyDTAk	Q05962	N-Term(iTRAQ4plex); Y7(Phospho); K11(iTR
aAASVVSGPPVASDcAsP	D3ZE17	N-Term(iTRAQ4plex); C15(Carbamidomethyl
aVPAtPPR	F1M1Y0	N-Term(iTRAQ4plex); T5(Phospho)
rESDEsGEsAPEEGGEGA	Q5FWU3	N-Term(iTRAQ4plex); S6(Phospho); S9(Phos
rPGPEHSLGLEkEsDEEPI	D3ZIL9	N-Term(iTRAQ4plex); K12(iTRAQ4plex); S14
rPAAAAAAGSAsPR	R9PXV8	N-Term(iTRAQ4plex); S12(Phospho)
sSESVVDEDGGR	D3ZL30	N-Term(iTRAQ4plex); S1(Phospho)
smsSAFcSLLAPER	P97680	N-Term(iTRAQ4plex); M2(Oxidation); S3(Pho
gREDVSNFDDEFTSEAPI	A0A0G2K6J2	N-Term(iTRAQ4plex); T20(Phospho)
aGGPASGTGsATsGGTV(	Q80XF7	N-Term(iTRAQ4plex); S10(Phospho); S13(Ph
hLLNsPTDPFNR	F1M8V2	N-Term(iTRAQ4plex); S5(Phospho)
qDTGsYTPDTkPk	D4ABB2	N-Term(iTRAQ4plex); S5(Phospho); K11(iTR
gLQPGEEELPDIsPPIVIPD	D3ZDI9	N-Term(iTRAQ4plex); S13(Phospho); K23(iT
ysEVSFLELDk	A0A0G2JT23	N-Term(iTRAQ4plex); S2(Phospho); K11(iTR
akPAMPQDSVPsPR	A0A0G2K5E7	N-Term(iTRAQ4plex); K2(iTRAQ4plex); S12(
ysPSQNsPIHHIPSR	B1WC16	N-Term(iTRAQ4plex); S2(Phospho); S7(Phos
kLDEDAsPNEEkGDVPk	B2GV74	K1(iTRAQ4plex); N-Term(iTRAQ4plex); S7(P
dQPAALPLAAEETVNLPP	F1MAQ5	N-Term(iTRAQ4plex); S19(Phospho); S23(Ph
vsPQQcSEPETk	D3ZKQ4	N-Term(iTRAQ4plex); S2(Phospho); C6(Carb
aGVLASLRsPEAEAVR	A0A0G2K677	N-Term(iTRAQ4plex); S9(Phospho)
vDNSSLTGESEPQTRsPD	P06687	N-Term(iTRAQ4plex); S16(Phospho); C19(Ca
lGPGVPQDGsDEEDEEW	M0RDF7	N-Term(iTRAQ4plex); S10(Phospho); K22(iT
tEDGDWEWsDDEMDEk	A0A0G2K007	N-Term(iTRAQ4plex); S9(Phospho); K16(iTR
eISSsPTSk	A0A0G2K2M9	N-Term(iTRAQ4plex); S5(Phospho); K9(iTRA
rLsPPGGSGSGVPGGGPI	F1LQ41	N-Term(iTRAQ4plex); S3(Phospho)
ySDtTDSDPENEPFDEDQ	O54857	N-Term(iTRAQ4plex); T4(Phospho); K24(iTR
tTHYGsLPQk	P02688	N-Term(iTRAQ4plex); S6(Phospho); K10(iTR
sGAYAALTLLGsSPPSVG	F1LQS9	N-Term(iTRAQ4plex); S12(Phospho)
gASAATGVPLESDEDsND	A0A0G2K1U1	N-Term(iTRAQ4plex); S16(Phospho); C28(Ca
qGsPVPTcGPLTDEDVVR	Q3KRC5	N-Term(iTRAQ4plex); S3(Phospho); C8(Carb
eVHDELEDLPsPPPPLsPI	M0R423	N-Term(iTRAQ4plex); S11(Phospho); S17(Ph
sQGsTSNsDWmk	F1LRL9	N-Term(iTRAQ4plex); S4(Phospho); S8(Phos
eGssEkDEGEQEEEGETE	P12839	N-Term(iTRAQ4plex); S3(Phospho); S4(Phos
sMsDPkPLSPTADESSR	Q9JKS6;D3Z9C7	N-Term(iTRAQ4plex); S3(Phospho); K6(iTRA
fTDQsLsPEDAEsLSVLSV	P34926;Q63330	N-Term(iTRAQ4plex); S5(Phospho); S7(Phos
eIAQDFktDLR	D3ZJ08;D3ZK97	N-Term(iTRAQ4plex); K7(iTRAQ4plex); T8(P
qPVELtPTDk	F1LS36	N-Term(iTRAQ4plex); T6(Phospho); K10(iTR
sRsPGtPAGEGSGSPPk	A0A0G2JSM7;D3ZZ99	N-Term(iTRAQ4plex); S3(Phospho); T6(Phos
tPEDGGYScEITEk	P15205;F1LRL9	N-Term(iTRAQ4plex); T1(Phospho); C9(Carb
aTLLNVPDLsDSIHSANAS	F1M4A0	N-Term(iTRAQ4plex); S10(Phospho)
aGDMLEDsPkRPk	Q8VHK7	N-Term(iTRAQ4plex); S8(Phospho); K10(iTR
sPPIPAkPR	Q63327	N-Term(iTRAQ4plex); S1(Phospho); K7(iTRA
rEsDGTPGGLASLENER	F1M820	N-Term(iTRAQ4plex); S3(Phospho)
eLPASEGGAGQsPADSG.	A0A0G2K1W1	N-Term(iTRAQ4plex); S12(Phospho)
qLQALTcDLESLRGtNESL	P47819	N-Term(iTRAQ4plex); C7(Carbamidomethyl);
eGEAAGGDHETEstSDkE	O35052	N-Term(iTRAQ4plex); S13(Phospho); T14(Ph
wNsNDFIDAFASPTEVEG	Q5U2N3	N-Term(iTRAQ4plex); S3(Phospho); K28(iTR
gSNYHLsDNDASDVE	Q921A2	N-Term(iTRAQ4plex); S7(Phospho)
eIsDDEAEEEk	P34058	N-Term(iTRAQ4plex); S3(Phospho); K11(iTR
vLsPLRsPPLIGSESAYED	P15205;F1LRL9	N-Term(iTRAQ4plex); S3(Phospho); S7(Phos

Information Classification: General

eLATHAAPLGsYAR	D3ZTF0	N-Term(iTRAQ4plex); S11(Phospho)
eAsPPSSADk	A0A0U1RRX4;F1MAQ5	N-Term(iTRAQ4plex); S3(Phospho); K10(iTR
rsPTSTGDTELkEER	A0A0G2JTD8	N-Term(iTRAQ4plex); S2(Phospho); K12(iTR
sPAEVk	F1LRZ7	N-Term(iTRAQ4plex); S1(Phospho); K6(iTRA
gSVsMDLD	Q66HA8	N-Term(iTRAQ4plex); S4(Phospho)
vSTSQAsPQSAASPR	D3ZHK4	N-Term(iTRAQ4plex); S7(Phospho)
kLSSNcSGVEGDVtDEDE	F1M324	K1(iTRAQ4plex); N-Term(iTRAQ4plex); C6(C
lSPQGQQPVsPQSGSPQ(	A0A096MIT7	N-Term(iTRAQ4plex); S10(Phospho)
eERLsAsPQELGkPYLESF	F1LQN3	N-Term(iTRAQ4plex); S5(Phospho); S7(Phos
aEsPPADAk	A0A0G2K315	N-Term(iTRAQ4plex); S3(Phospho); K9(iTRA
gsGsVDETLFALPAASEP\	F1LQN3	N-Term(iTRAQ4plex); S2(Phospho); S4(Phos
lIPDPksESk	F1LS36;F1LS42	N-Term(iTRAQ4plex); K6(iTRAQ4plex); S7(P
sLEGEAAALR	F1LRZ7	N-Term(iTRAQ4plex); S1(Phospho)
sLGSLSQGSANATVLDVA	F1M386	N-Term(iTRAQ4plex); S1(Phospho); K24(iTR
dkEDLVcSAALHsPQEsPV	F1LQN3	N-Term(iTRAQ4plex); K2(iTRAQ4plex); C7(C
gSYGsDPEEEEEYR	Q3ZB99	N-Term(iTRAQ4plex); S5(Phospho)
sLGTGAPVIESPYGESIsP	A0A0G2JWP1	N-Term(iTRAQ4plex); S18(Phospho); K28(iT
vGsLk	M0R423	N-Term(iTRAQ4plex); S3(Phospho); K5(iTRA
nWYQSMIPQsPSPPLDEF	F1M1T9	N-Term(iTRAQ4plex); S10(Phospho)
sHSGAsPDNTAPPPPPPF	A0A0G2KB70	N-Term(iTRAQ4plex); S6(Phospho)
dHIIEVVGsSMPLIGDHQD	P09951	N-Term(iTRAQ4plex); S9(Phospho); K22(iTR
gIDPPQVLsPDMVPPSER	A0A0G2QC53	N-Term(iTRAQ4plex); S9(Phospho)
iLDQmPATPsSPmYVD	F1LP57	N-Term(iTRAQ4plex); M5(Oxidation); S10(Ph
tEEVGENTQVEDTEPSsP	F1M842	N-Term(iTRAQ4plex); S17(Phospho); K21(iT
aIsAPTsPTR	A0A0U1RS39	N-Term(iTRAQ4plex); S3(Phospho); S7(Phos
akPVVSDFDsDEEQDENE	E9PTG1	N-Term(iTRAQ4plex); K2(iTRAQ4plex); S10(
vsMDGEWLcLR	Q5FVJ0;A0A0G2K6A9	N-Term(iTRAQ4plex); S2(Phospho); C9(Carb
dGAESSRDsMEINEADFR	D3ZF86	N-Term(iTRAQ4plex); S9(Phospho)
aAsPsPQSVR	B2RYB3;A0A0G2K4F6	N-Term(iTRAQ4plex); S3(Phospho); S5(Phos
rGsSPGSLEIPk	A0A140TAA3	N-Term(iTRAQ4plex); S3(Phospho); K12(iTR
dVLsPLRPSR	F1M4A4	N-Term(iTRAQ4plex); S4(Phospho)
sQEEAPGHR	M0RDR2	N-Term(iTRAQ4plex); S1(Phospho)
kAAsPsPQSVR	B2RYB3;A0A0G2K4F6	K1(iTRAQ4plex); N-Term(iTRAQ4plex); S4(P
lGsPVDGLEDMDRDDLTD	M0RD44	N-Term(iTRAQ4plex); S3(Phospho)
lGTGGGGsPDkSPSAQEL	I6L9H8	N-Term(iTRAQ4plex); S8(Phospho); K11(iTR
tTsPEEAQSQPYIAmDWE	Q6IE71	N-Term(iTRAQ4plex); S3(Phospho); M15(Ox
sLkEQAVkITNLTQQLEQA	F1LMV6	N-Term(iTRAQ4plex); S1(Phospho); K3(iTRA
tDIGRLsPEAk	D3ZLW4	N-Term(iTRAQ4plex); S7(Phospho); K11(iTR
lLLDPSNtPTk	Q6P791	N-Term(iTRAQ4plex); T8(Phospho); K11(iTR
sPVLLPk	Q712J2	N-Term(iTRAQ4plex); S1(Phospho); K7(iTRA
aFEDEQPPTLsPk	F1LNC7	N-Term(iTRAQ4plex); S11(Phospho); K13(iT
nVEDTDGssPEDLMAALT	Q6RJR6	N-Term(iTRAQ4plex); S8(Phospho); S9(Phos
kVSVTVDYIRPAsPATETV	D4A8Y5	N-Term(iTRAQ4plex); K1(iTRAQ4plex); S13(
sLDSLDPAGLLTSPk	G3V849	N-Term(iTRAQ4plex); S1(Phospho); K15(iTR
vMLGEtNPADskPGTIR	P19804	N-Term(iTRAQ4plex); T6(Phospho); S11(Pho
hIPRPPQLGsPNYck	P25122	N-Term(iTRAQ4plex); S10(Phospho); C14(Ca
ePSSPGTDDVFTPGPSDs	P26431	N-Term(iTRAQ4plex); S18(Phospho)
iPSsPDLkk	P63142	N-Term(iTRAQ4plex); S4(Phospho); K8(iTRA
aksPVk	F1LRZ7	N-Term(iTRAQ4plex); K2(iTRAQ4plex); S3(P
aNsLDNER	D3ZJG4	N-Term(iTRAQ4plex); S3(Phospho)
iELLPSRPcVPsPVPPR	D4A3E3	N-Term(iTRAQ4plex); C9(Carbamidomethyl);
dFSPGGsPPGTPPASPLS	D3Z881	N-Term(iTRAQ4plex); S7(Phospho)
sYIETETESR	A0A0G2K6R9;F1M9N9	N-Term(iTRAQ4plex); S1(Phospho)
aSVSDLsPR	Q5M7V8	N-Term(iTRAQ4plex); S7(Phospho)
tSFDQDsDVDIFPSDFTSE	Q14TE9	N-Term(iTRAQ4plex); S7(Phospho)

rLsNGSIDSTDDTSQIVEL(	F1M8A4	N-Term(iTRAQ4plex); S3(Phospho); K25(iTR
hSPVsPSSk	A0A0G2K6R9;F1M9N9	N-Term(iTRAQ4plex); S5(Phospho); K9(iTRA
gAEEEEEEEDDDsEEEIk	D4A559	N-Term(iTRAQ4plex); S13(Phospho); K18(iT
qsPDEPLR	I6L9G6	N-Term(iTRAQ4plex); S2(Phospho)
lsLGAGGR	B5DF40	N-Term(iTRAQ4plex); S2(Phospho)
lGGSLIVAFEGsPV	Q7M0E3	N-Term(iTRAQ4plex); S12(Phospho)
rsEtPPHWR	G3V6Y9	N-Term(iTRAQ4plex); S2(Phospho); T4(Phos
sQGLSLDAVYHcLNR	A0A0G2K9M4	N-Term(iTRAQ4plex); S1(Phospho); C12(Ca
vAELEEEQsQGsSSYsDW	P34926;Q63330	N-Term(iTRAQ4plex); S9(Phospho); S12(Pho
nQSLGQsAPSLTAGLk	D3ZAR2	N-Term(iTRAQ4plex); S7(Phospho); K16(iTR
vSTGDTsPYGTEDSSPAS	Q4G008	N-Term(iTRAQ4plex); S7(Phospho)
aPssDEEcFFDLLSk	G3V9X2	N-Term(iTRAQ4plex); S3(Phospho); S4(Phos
ekEIsDDEAEEEk	P34058	N-Term(iTRAQ4plex); K2(iTRAQ4plex); S5(P
dVEmGNsVIEENEMk	A0A0G2K611	N-Term(iTRAQ4plex); M4(Oxidation); S7(Pho
eksLDLPDR	F1LSL6	N-Term(iTRAQ4plex); K2(iTRAQ4plex); S3(P
aEEEGGSEEEVGDksPQE	P12839	N-Term(iTRAQ4plex); K14(iTRAQ4plex); S15
qGVDDIEkFEDEGAGFEE	P15205;F1LRL9	N-Term(iTRAQ4plex); K8(iTRAQ4plex); S19(
vNASASsLk	P85845	N-Term(iTRAQ4plex); S7(Phospho); K9(iTRA
sEsPTADSDAsSLHEQPQ	Q6AYP2	N-Term(iTRAQ4plex); S3(Phospho); S11(Pho
gGEDGSGsPHsPPHLcSk	Q9JIH7	N-Term(iTRAQ4plex); S8(Phospho); S11(Pho
eLEQHIQTSDPENFQSEE	F1MAA1	N-Term(iTRAQ4plex); S20(Phospho)
aAPsPDVTVGASPVLDEI(	D3ZJH2	N-Term(iTRAQ4plex); S4(Phospho); K24(iTR
sDAAVDTSSEITTk	P06302	N-Term(Acetyl); S1(Phospho); K14(iTRAQ4p
vNASASsLkk	P85845	N-Term(iTRAQ4plex); S7(Phospho); K9(iTRA
sTsLESWLTSYk	G3V6M0	N-Term(iTRAQ4plex); S3(Phospho); K12(iTR
sSsLPSYGR	D4A559	N-Term(iTRAQ4plex); S3(Phospho)
rAVPARVsLGIttVLtMSTIV	P50573	N-Term(iTRAQ4plex); S8(Phospho); T12(Pho
kAQVsPQS	B2RYB3	N-Term(iTRAQ4plex); K1(iTRAQ4plex); S5(P
aEEEGGsEEEVGDk	P12839	N-Term(iTRAQ4plex); S7(Phospho); K14(iTR
qLsPESLGTLQFGELNLG	P34926;Q63330	N-Term(iTRAQ4plex); S3(Phospho); K19(iTR
tEGYAAFQEDssGDEAES	Q5FVT1	N-Term(iTRAQ4plex); S11(Phospho); S12(Ph
tESTYLAEIHRQsPk	D3ZYJ5	N-Term(iTRAQ4plex); S13(Phospho); K15(iT
eQQSWVIsPPQPSPQk	A0A0G2K7K9	N-Term(iTRAQ4plex); S8(Phospho); K16(iTR
sASTISk	P63142	N-Term(iTRAQ4plex); S1(Phospho); K7(iTRA
dELHIVEAEAMNYEGsPIk	P13084;Q7TP95	N-Term(iTRAQ4plex); S16(Phospho); K19(iT
rVPGssGHLHk	A0A0G2K007	N-Term(iTRAQ4plex); S5(Phospho); S6(Phos
tDsTSDGRPAWMR	F1LRT9	N-Term(iTRAQ4plex); S3(Phospho)
tSTYDNVPNPPVSQGNPA	D3ZGL1	N-Term(iTRAQ4plex); S22(Phospho); K29(iT
tERHsPVSPSSk	A0A0G2K6R9;F1M9N9	N-Term(iTRAQ4plex); S5(Phospho); K12(iTR
vGSLtPPSSPk	P0C1X8	N-Term(iTRAQ4plex); T5(Phospho); K11(iTR
tVDEQEDMDLQIsPDRk	A0A0G2K6R9;F1M9N9	N-Term(iTRAQ4plex); S13(Phospho); K17(iT
vAAGHELQPLAIVDQRPs	P08050	N-Term(iTRAQ4plex); S18(Phospho); S19(Ph
iPSsPDLk	P63142	N-Term(iTRAQ4plex); S4(Phospho); K8(iTRA
dGMDTScSTGsPGAATAN	Q9JKM5	N-Term(iTRAQ4plex); C7(Carbamidomethyl);
dIMLATGkLsPDAIPGk	P12785	N-Term(iTRAQ4plex); K8(iTRAQ4plex); S10(
gNAQESLDTVsPk	A0A0U1RRX4;F1MAQ5	N-Term(iTRAQ4plex); S11(Phospho); K13(iT
lTSVSsSVDFDQR	D4A105	N-Term(iTRAQ4plex); S6(Phospho)
aATEALGEksPEGTTVSG	A0A140TA95	N-Term(iTRAQ4plex); K9(iTRAQ4plex); S10(
qSHDENLIsP	Q5I0L4	N-Term(iTRAQ4plex); S9(Phospho)
aADEEPDSPtEALQTAAE	G3V727	N-Term(Acetyl); T10(Phospho); K23(iTRAQ4
kREtDDEGEDD	Q9EST6	N-Term(iTRAQ4plex); K1(iTRAQ4plex); T4(P
eRLEscDRR	Q5M9I5	N-Term(iTRAQ4plex); S5(Phospho); C6(Carb
tVGtPIASVPGSTNTGTVP	A0A0G2JTW5	N-Term(iTRAQ4plex); T4(Phospho); K23(iTR
eGPALDRQsPESTLTPTF	O35141	N-Term(iTRAQ4plex); S9(Phospho); K20(iTR
gQAYAtEWSDDESGNPF(	Q9Z0W5	N-Term(iTRAQ4plex); T6(Phospho); K34(iTR

Information Classification: General

gADsGGEkEEGVNR	G3V6C3	N-Term(iTRAQ4plex); S4(Phospho); K8(iTRA
vDsPTVTTTLk	A0A140TAB3	N-Term(iTRAQ4plex); S3(Phospho); K11(iTR
kAEGEPQEEsPLkSk	B1WC16	K1(iTRAQ4plex); N-Term(iTRAQ4plex); S10(
eGLLPTSDQEAGGPSDIF	Q80X08	N-Term(iTRAQ4plex); S22(Phospho); K26(iT
vAAHAAHSPSsAEWIAcLI	D3KR63	N-Term(Acetyl); S11(Phospho); C17(Carbam
nDGVkEsSESTNTTIEDEI	P11275	N-Term(iTRAQ4plex); K5(iTRAQ4plex); S7(P
mFSNPDNGsPAMTHR	Q9JI66;A0A0H2UHB7	N-Term(iTRAQ4plex); S9(Phospho)
eSPPQPPADDGSEEPGS	D4A1Q2	N-Term(iTRAQ4plex); S20(Phospho); K23(iT
lPsEPGMTLLTIR	B1WBV1	N-Term(iTRAQ4plex); S3(Phospho)
tASLTSAAsIDGSR	A0A0G2JSU4	N-Term(iTRAQ4plex); S9(Phospho)
tESVTSGPLSPEGSPSksI	F8WFS9	N-Term(iTRAQ4plex); K17(iTRAQ4plex); S18
sVLVPPGPDSFR	A0A0G2K207	N-Term(iTRAQ4plex); S1(Phospho)
lDNTPAsPPR	X4YHC6	N-Term(iTRAQ4plex); S7(Phospho)
qAEVADQQTTDLPAENG	Q5U1W8	N-Term(iTRAQ4plex); T19(Phospho); S26(Ph
lGSLTPEIVSTPSsPEEED	P0C627	N-Term(iTRAQ4plex); S14(Phospho); K20(iT
lsLDEDEEPk	A0A0G2K6N2	N-Term(iTRAQ4plex); S2(Phospho); K10(iTR
aETSLGIPsPTEISAEVk	D3ZC89	N-Term(iTRAQ4plex); S9(Phospho); K18(iTR
hsGQDVHVVLk	A0A0G2K911	N-Term(iTRAQ4plex); S2(Phospho); K11(iTR
eEPLsEEEPcTSTAVPSPE	Q9QZ86	N-Term(iTRAQ4plex); S5(Phospho); C10(Ca
rLsGGAVPSASMTR	G3V7P8	N-Term(iTRAQ4plex); S3(Phospho)
eGsPANWk	Q4KM87	N-Term(iTRAQ4plex); S3(Phospho); K8(iTRA
sDsLILDHQWELEk	F1M4A4	N-Term(iTRAQ4plex); S3(Phospho); K14(iTR
lEtSDGDEEGNAEVNk	A0A0G2JSH6	N-Term(iTRAQ4plex); T3(Phospho); K16(iTR
nDGSQsDTAVGTVGAGG	A0A0G2KAV8	N-Term(iTRAQ4plex); S6(Phospho); K18(iTR
aDPVLLNNHSNLkPAPTV	Q5FVI4	N-Term(iTRAQ4plex); K13(iTRAQ4plex); T28
kQtPPASPSPQPAEDRPP	A0A1B0GWS4	N-Term(iTRAQ4plex); K1(iTRAQ4plex); T3(P
gPAGsPcEEGDDAEEDG	Q6TQE1	N-Term(iTRAQ4plex); S5(Phospho); C7(Carb
iNVYYNEAtGGNYVPR	B4F7C2	N-Term(iTRAQ4plex); T9(Phospho)
sMSSIPPYPASSLAGsSPI	D3ZZ81	N-Term(iTRAQ4plex); S16(Phospho)
gLGPPsPPAPPR	D3ZMX6	N-Term(iTRAQ4plex); S6(Phospho)
tLPDSQQVPSSsPASk	A0A0G2K326	N-Term(iTRAQ4plex); S11(Phospho); K15(iT
qNsEEASLPGEDIVEDNk	F1M6X3	N-Term(iTRAQ4plex); S3(Phospho); K18(iTR
sPAVSPSkAsEDAk	F8WFS9	N-Term(iTRAQ4plex); K8(iTRAQ4plex); S10(
iLMDsPEDADIFHTEEMk	P80386	N-Term(iTRAQ4plex); S5(Phospho); K18(iTR
aNsVTmDGQGLQITPIPG	P49621	N-Term(iTRAQ4plex); S3(Phospho); M6(Oxid
lAALALASSENSsTPEEcE	Q5RJN5	N-Term(iTRAQ4plex); S13(Phospho); C18(Ca
lIRPEYAWIVQPASGAVSI	D3ZBL6	N-Term(iTRAQ4plex); S24(Phospho); K26(iT
tITDTNLEtPsDTER	D3ZBU7	N-Term(iTRAQ4plex); T9(Phospho); S11(Pho
mSksPVDSSVLEGVk	F1LMT5	N-Term(iTRAQ4plex); K3(iTRAQ4plex); S4(P
aAGskEHLPHTEWQGQE	G3V7X5	N-Term(iTRAQ4plex); S4(Phospho); K5(iTRA
lDNVGEARsPQDRsPILA	Q63472	N-Term(iTRAQ4plex); S9(Phospho); S14(Pho
qLAFsTVGTPDYIAPEVFM	A0A0U1RRV1	N-Term(iTRAQ4plex); S5(Phospho); K25(iTR
sSVSDLVNSLTSEMLMLS	Q62747	N-Term(iTRAQ4plex); S21(Phospho); C30(Ca
sTPAsPVQsPTR	F8WFS9	N-Term(iTRAQ4plex); S5(Phospho); S9(Phos
aEEAAQENAsDYLAIPSEI	Q9JK00	N-Term(iTRAQ4plex); S10(Phospho); K20(iT
sQsLPTTLLSPVR	D3ZLC3	N-Term(iTRAQ4plex); S3(Phospho)
dLEFsAGSPGkDTNESPW	G3V7U9	N-Term(iTRAQ4plex); S5(Phospho); K11(iTR
yNTDcVQGLTHsk	P06687	N-Term(iTRAQ4plex); C5(Carbamidomethyl);
kVPEQPPVLPQLDsQHL	Q63083	N-Term(iTRAQ4plex); K1(iTRAQ4plex); S14(
nVSTDsAENEEk	E9PSJ4	N-Term(iTRAQ4plex); S6(Phospho); K12(iTR
rSEAEEGEVRtPTk	A0A0G2K0F3	N-Term(iTRAQ4plex); T11(Phospho); K14(iT
qVDEGVWPPPNNLLNQs	Q9QXY2	N-Term(iTRAQ4plex); S17(Phospho); K19(iT
qNsLEYmDQNDDR	D3ZEI4	N-Term(iTRAQ4plex); S3(Phospho); M7(Oxid
aLGLEEsPAEGSk	P34926	N-Term(iTRAQ4plex); S7(Phospho); K13(iTR
gDVVLVVPsDSEADQDA	A0A0G2K5Z4	N-Term(iTRAQ4plex); S9(Phospho); K24(iTR

mGPSSSPIPSPSPSPtDSI	P63319	N-Term(iTRAQ4plex); T16(Phospho); K19(iT
eVDVEkVsAFENPYVDAIk	Q7M0H6	N-Term(iTRAQ4plex); K6(iTRAQ4plex); S8(P
sNQTNHkPsPDQDEEEGI	F1LPE9	N-Term(iTRAQ4plex); K7(iTRAQ4plex); S9(P
rVHSDsETDDIGFIPSk	P31016	N-Term(iTRAQ4plex); S6(Phospho); K17(iTR
lTPAEEESNSSQLLsP	D3ZNK1	N-Term(iTRAQ4plex); S15(Phospho)
aWALFPPsDDYDDTDDD	P0C5X8	N-Term(iTRAQ4plex); S8(Phospho); K26(iTR
aFGEsstEsDEDEEEGcGH	Q6MFY6	N-Term(iTRAQ4plex); S5(Phospho); S6(Phos
kGTGDcsDEEVDGk	G3V6P7	N-Term(iTRAQ4plex); K1(iTRAQ4plex); C6(C
vVSMLQGSGAmPTLVVE	F1LU68	N-Term(iTRAQ4plex); M11(Oxidation); S25(P
ncPsPMQTGAATDDSk	O09032	N-Term(iTRAQ4plex); C2(Carbamidomethyl);
cQVAFsYLPQNDDELELk	M0RBZ7	N-Term(iTRAQ4plex); C1(Carbamidomethyl);
eISSPSsPVk	A0A0G2K6R9;F1M9N9	N-Term(iTRAQ4plex); S7(Phospho); K10(iTR
aPSDDGsLk	D3ZFD0	N-Term(iTRAQ4plex); S7(Phospho); K9(iTRA
kDPQDmEGEksPAsPFAQ	A0A0U1RRX4;F1MAQ5	N-Term(iTRAQ4plex); K1(iTRAQ4plex); M6(C
tASEPPLAPPVsEEEDEEE	Q62829	N-Term(iTRAQ4plex); S12(Phospho); K40(iT
sELQAYIEQcQDsPTSGk	D3ZRF9	N-Term(iTRAQ4plex); C10(Carbamidomethyl
mDSAVQASLsLPATPVGk	Q78PB6	N-Term(iTRAQ4plex); M1(Oxidation); S10(Ph
aDIDQsPVSk	G3V6Y9	N-Term(iTRAQ4plex); S6(Phospho); K10(iTR
dVMsDETNNEETEsPSQE	F1LRL9	N-Term(iTRAQ4plex); S4(Phospho); S14(Pho
ekEPAEGNESSEAPSPVN	Q6QRP0;A0A0G2K3Q5	N-Term(iTRAQ4plex); K2(iTRAQ4plex); S22(
vIHFVSGETmEEYstDEDE	D3ZIM7	N-Term(iTRAQ4plex); M10(Oxidation); S14(P
sLVGTPYWMAPELISR	B5DF62	N-Term(iTRAQ4plex); S1(Phospho)
sQsPSLPPLPEDLEk	E9PST5	N-Term(iTRAQ4plex); S3(Phospho); K15(iTR
sTPsLERPEPR	A0A0G2JUG7	N-Term(iTRAQ4plex); S4(Phospho)
sRsPsTESQLASk	F8WFS9	N-Term(iTRAQ4plex); S3(Phospho); S5(Phos
gSDTsPEAEASSGGGGV	Q9WVR6	N-Term(iTRAQ4plex); S5(Phospho); K20(iTR
eGTQQASEGYFSQsQEE	Q07266	N-Term(iTRAQ4plex); S14(Phospho); C26(Ca
hsPGVSkEDSEEQtVkPGI	P34926;Q63330	N-Term(iTRAQ4plex); S2(Phospho); K7(iTRA
aELVLIDEDDEksLREk	D4A4D8	N-Term(iTRAQ4plex); K12(iTRAQ4plex); S13
akPVVSDDDsDEEQEEDF	G3V790	N-Term(iTRAQ4plex); K2(iTRAQ4plex); S10(
dSPEsPFEVIIDk	Q6RJR6	N-Term(iTRAQ4plex); S5(Phospho); K13(iTR
nLEAVETLGStSTIcSDk	P06686;P06687;P06685	N-Term(iTRAQ4plex); T11(Phospho); C15(Ca
aPSQAAESsPTkk	A0A0G2K4S1	N-Term(iTRAQ4plex); S9(Phospho); K12(iTR
kPPAPPsPVQsQSPSTNW	B2RYB3;A0A0G2K4F6	K1(iTRAQ4plex); N-Term(iTRAQ4plex); S7(P
sFSLGDLSHsPQTAQHVE	D4A8V2	N-Term(iTRAQ4plex); S10(Phospho)
sPPLLEsPDASR	F1LMM2	N-Term(iTRAQ4plex); S7(Phospho)
vGLLSsPNQASSPGGTVV	Q5BJW6	N-Term(iTRAQ4plex); S6(Phospho)
qTsIETDR	B5DEJ8	N-Term(iTRAQ4plex); S3(Phospho)
aAVPSGAsTGIYEALELR	M0R5J4;P07323	N-Term(iTRAQ4plex); S8(Phospho)
rSsEPSIDYLDAk	Q6REY9	N-Term(iTRAQ4plex); S3(Phospho); K13(iTR
dFDQDAsDkEFPENVEk	D4A997	N-Term(iTRAQ4plex); S7(Phospho); K9(iTRA
eIHDVAGDGDSLGsPGPT	D4A1G8	N-Term(iTRAQ4plex); S14(Phospho)
eMcVSGGDHTQLTDLsPE	Q5QD51	N-Term(iTRAQ4plex); C3(Carbamidomethyl);
aDLEEQLsDEEk	Q3T1K5	N-Term(Acetyl); S8(Phospho); K12(iTRAQ4p
kVEcTPTPITPSTMPSFSF	D4A554	K1(iTRAQ4plex); N-Term(iTRAQ4plex); C4(C
sLmSsPEDLTk	P15205;F1LRL9	N-Term(iTRAQ4plex); M3(Oxidation); S5(Pho
kSLsDSESDDSk	Q5RJK5	N-Term(iTRAQ4plex); K1(iTRAQ4plex); S4(P
qHkDsPEILSR	P0C5Y8	N-Term(iTRAQ4plex); K3(iTRAQ4plex); S5(P
rVsGDYNGEQk	A0A0G2K4N6	N-Term(iTRAQ4plex); S3(Phospho); K11(iTR
sLSRtPsPPPFR	A0A0G2JZB4	N-Term(iTRAQ4plex); T5(Phospho); S7(Phos
tTPPTQkPPsPPMSGk	F1LYA6	N-Term(iTRAQ4plex); K7(iTRAQ4plex); S10(
kDsEGYSESPDLEFEYAD	G3V8E2	N-Term(iTRAQ4plex); K1(iTRAQ4plex); S3(P
gVDkPPsPSPIEMk	A0A0G2K4M7	N-Term(iTRAQ4plex); K4(iTRAQ4plex); S7(P
sLAQHIPGPGsIEGMk	D3ZU84	N-Term(iTRAQ4plex); S11(Phospho); K16(iT
qkELsEGEEk	O88420	N-Term(iTRAQ4plex); K2(iTRAQ4plex); S5(P

Information Classification: General

vGMGLGLQVAAALtAMDN	Q5FVR2	N-Term(iTRAQ4plex); T14(Phospho)
aFVESTASSTSEs	F1M0G0	N-Term(iTRAQ4plex); S13(Phospho)
qELQDsLSDPEDILDRER	D4A148	N-Term(iTRAQ4plex); S6(Phospho)
sPAEAksPAEAkPPAEAk	F1LRZ7	N-Term(iTRAQ4plex); K6(iTRAQ4plex); S7(P
sTDIGFtPPSsPPTRPR	F1M5N7	N-Term(iTRAQ4plex); T7(Phospho); S11(Pho
dmDEPsPVPNVEEVTLPk	D4A510	N-Term(iTRAQ4plex); M2(Oxidation); S6(Pho
gSLAsLDSLR	D3ZZZ9	N-Term(iTRAQ4plex); S5(Phospho)
hSTLLSVPDSTLASMFSP	A6Y7S3	N-Term(iTRAQ4plex); S20(Phospho)
qEPGsPPPAPLAPVLPAF	D3ZV88	N-Term(iTRAQ4plex); S5(Phospho); C27(Ca
sQVIHSGHFmVSSPHSDs	Q8VIP2	N-Term(iTRAQ4plex); M10(Oxidation); S18(P
eSSEStNttIEDEDTkVR	P11275	N-Term(iTRAQ4plex); T6(Phospho); T8(Phos
tVkPTYIGsPVk	D4A4L4	N-Term(iTRAQ4plex); K3(iTRAQ4plex); S9(P
tVGHSVVsPQDTVQR	Q5RK25	N-Term(iTRAQ4plex); S8(Phospho)
dATEDsITEDDkRR	P0CD96	N-Term(iTRAQ4plex); S6(Phospho); K12(iTR
hLEGEEDGNsDQSQTSET	Q3B7U1	N-Term(iTRAQ4plex); S10(Phospho)
gLAsPDR	Q5QD51	N-Term(iTRAQ4plex); S4(Phospho)
vPLHLEsPHEER	D3ZSZ6	N-Term(iTRAQ4plex); S7(Phospho)
vHAYFAPVtPPPSVGGSR	A0A0H2UI14	N-Term(iTRAQ4plex); T9(Phospho)
sPAVsPSk	F8WFS9	N-Term(iTRAQ4plex); S5(Phospho); K8(iTRA
tsPTSGEGTRPGGIIHVYG	B0VXR4	N-Term(iTRAQ4plex); S2(Phospho); K25(iTR
sPEQVksPAkEEAk	F1LRZ7	N-Term(iTRAQ4plex); K6(iTRAQ4plex); S7(P
kVEGsPNQAk	F1M853	N-Term(iTRAQ4plex); K1(iTRAQ4plex); S5(P
rDEsDEEPPR	A0A0G2JZ88	N-Term(iTRAQ4plex); S4(Phospho)
fIIGSVSEDNsEDEISNLVk	P11497	N-Term(iTRAQ4plex); S11(Phospho); K20(iT
gQPAQGPVSEEsPPsPkP	Q80X08	N-Term(iTRAQ4plex); S12(Phospho); S15(Ph
yFPtQALNFAFk	Q09073;Q05962	N-Term(iTRAQ4plex); T4(Phospho); K12(iTR
tVEIPEDEVVRtPR	Q9Z1W6	N-Term(iTRAQ4plex); T12(Phospho)
sPDTSAYcYETmEk	P15205;F1LRL9	S1(Phospho); N-Term(iTRAQ4plex); C8(Carb
sDPLLMGIPTSENPFk	G3V6P8	N-Term(iTRAQ4plex); S1(Phospho); K16(iTR
aFPkEELAsDLEEMATSSI	Q5RJN5	N-Term(iTRAQ4plex); K4(iTRAQ4plex); S9(P
dWTSEsPR	F1LSB5	N-Term(iTRAQ4plex); S6(Phospho)
lMTANsPEVHG	P61808	N-Term(iTRAQ4plex); S6(Phospho)
vQIPVSHPDPDPVsDNED	F1M4A0	N-Term(iTRAQ4plex); S14(Phospho)
tWcGsPPYAAPELFEGk	M0RD40	N-Term(iTRAQ4plex); C3(Carbamidomethyl);
sPVEVksPEk	F1LRZ7	N-Term(iTRAQ4plex); S1(Phospho); K6(iTRA
hVTLPSsPR	A0A0G2JU89	N-Term(iTRAQ4plex); S7(Phospho)
nAAGEGGLPATPGSGPW	D3ZWQ0	N-Term(iTRAQ4plex); S22(Phospho)
kMQVPTAEEMSSLTPEsS	B2DD29	N-Term(iTRAQ4plex); K1(iTRAQ4plex); S17(
yLAEFATGNDR	P62260	N-Term(iTRAQ4plex); Y1(Phospho)
hTEPNEtTPLTEPEk	F1LUV9	N-Term(iTRAQ4plex); T7(Phospho); K15(iTR
nLEQILNGGEsPk	P58405	N-Term(iTRAQ4plex); S11(Phospho); K13(iT
aADGERSPLLSEAGDGG	Q5PPM8	N-Term(Acetyl); T38(Phospho); K48(iTRAQ4
fRRsEtPPHWR	G3V6Y9	N-Term(iTRAQ4plex); S4(Phospho); T6(Phos
aIYQGPSsPDk	P22734	N-Term(iTRAQ4plex); S8(Phospho); K11(iTR
vNLEESTGVENsPTGARR	Q62991	N-Term(iTRAQ4plex); S12(Phospho); K19(iT
tGDLGIPPNPEDRsPsPEP	F1LSC3	N-Term(iTRAQ4plex); S14(Phospho); S16(Ph
aVSEVsINR	G3V849	N-Term(iTRAQ4plex); S6(Phospho)
gsEPDDAQLQPPEDDDVI	Q5FVJ4	N-Term(iTRAQ4plex); S2(Phospho)
eSPPQPPADDGSEEPGS	D4A1Q2	N-Term(iTRAQ4plex); K23(iTRAQ4plex); T25
aSLQSGQEGPGDSPGsQ	Q9ES26	N-Term(iTRAQ4plex); S16(Phospho); K20(iT
aVSEVsINRSLDsLDPAGL	G3V849	N-Term(iTRAQ4plex); S6(Phospho); S13(Pho
rSYsSSSGSLMPSLENLDI	P19527	N-Term(iTRAQ4plex); S4(Phospho); K30(iTR
sIEEAcLTLQHLNR	P34926;Q63330	N-Term(iTRAQ4plex); S1(Phospho); C6(Carb
cLsDPGPHPEPGEGEPFI	P26431	N-Term(iTRAQ4plex); C1(Carbamidomethyl);
yEkIsLPk	D4A7D3	N-Term(iTRAQ4plex); K3(iTRAQ4plex); S5(P

Information Classification: General

asPTGTPGTTPPPSPGG(	B2DD29	N-Term(iTRAQ4plex); S2(Phospho)
gLSAsLPDLDSESWIEVk	F1M062	N-Term(iTRAQ4plex); S5(Phospho); K18(iTR
dIkFDDsDTEQIAEEGDDN	D3ZYT8	N-Term(iTRAQ4plex); K3(iTRAQ4plex); S7(P
sLSLGGHVGFDSLPDQLV	B0BNF1	N-Term(iTRAQ4plex); S1(Phospho); K20(iTR
wVNQPPsPDGR	Q6IE24	N-Term(iTRAQ4plex); S7(Phospho)
mEDIDQSSLVSSSTDsPP	F1LQN3	N-Term(Acetyl); M1(Oxidation); S16(Phospho
sksMTAELEELASIR	A0A0U1RS13	N-Term(iTRAQ4plex); K2(iTRAQ4plex); S3(P
aIEPQkEEADENYNsVNTI	G3V8I4	N-Term(iTRAQ4plex); K6(iTRAQ4plex); S15(
nWYQSmIPQsPSPPLDEF	F1M1T9	N-Term(iTRAQ4plex); M6(Oxidation); S10(Ph
aDskESLk	P34926;Q63330	N-Term(iTRAQ4plex); S3(Phospho); K4(iTRA
mkDTDsEEEIR	P62161;Q5U206	N-Term(iTRAQ4plex); K2(iTRAQ4plex); S6(P
eTLFSPVSTEEMLPRPPs	Q63424	N-Term(iTRAQ4plex); S18(Phospho); K21(iT
nsGSSPEAAGVPASVsPP	D3ZT56	N-Term(iTRAQ4plex); S2(Phospho); S16(Pho
dFQDYIEPEEGcQGsPQR	A0A096MIY2	N-Term(iTRAQ4plex); C12(Carbamidomethyl
aGNEsPVQELR	A0A0G2K7S6;A0A0G2JX1	N-Term(iTRAQ4plex); S5(Phospho)
gGYStPQTLANR	A0A0G2K104;F1LNC7	N-Term(iTRAQ4plex); T5(Phospho)
lAIFGPLccsSLEYSIR	F1LM73	N-Term(iTRAQ4plex); C8(Carbamidomethyl);
hGGYENAVHsGALDD	A0A096MJA0	N-Term(iTRAQ4plex); S10(Phospho)
eGSLsPVNSQk	A0A0G2K6U6	N-Term(iTRAQ4plex); S5(Phospho); K11(iTR
nVTESPsFSAGDNPHVLY	G3V9B3	N-Term(iTRAQ4plex); S7(Phospho)
tSIDAYDNFDNIsLAQR	F1M779	N-Term(iTRAQ4plex); S13(Phospho)
qNSsPPSSLNk	P11506	N-Term(iTRAQ4plex); S4(Phospho); K11(iTR
hAAPAPSAkssPSk	Q62950	N-Term(iTRAQ4plex); K9(iTRAQ4plex); S10(
eSPsHGLELSAPEk	Q924N5	N-Term(iTRAQ4plex); S4(Phospho); K14(iTR
aksELALAPAPAPAATHsF	Q6AYB2	N-Term(iTRAQ4plex); K2(iTRAQ4plex); S3(P
aENAAAsPEVGSADVAN\	Q80X08	N-Term(iTRAQ4plex); S7(Phospho); K21(iTR
aDSsDETDTSFAER	A0A0G2K0F3	N-Term(iTRAQ4plex); S4(Phospho)
ssPVESLk	D4ACF1	N-Term(iTRAQ4plex); S2(Phospho); K8(iTRA
vIHFVSGETMEEYstDEDE	D3ZIM7	N-Term(iTRAQ4plex); S14(Phospho); T15(Ph
gATDtSPLTR	F1M5N7	N-Term(iTRAQ4plex); T5(Phospho)
lPcLPVkLsPPLPPk	F1LNB2	N-Term(iTRAQ4plex); C3(Carbamidomethyl);
vQEAQDSDsDTEGGATG	A0A0G2JWM2	N-Term(iTRAQ4plex); S9(Phospho)
gSHSQtPsPGALPLGR	P09951	N-Term(iTRAQ4plex); T6(Phospho); S8(Phos
qIAsDSPHAsPk	A0A0G2K9J0	N-Term(iTRAQ4plex); S4(Phospho); S10(Pho
sIQsDAPVYLR	A0A0G2K315	N-Term(iTRAQ4plex); S4(Phospho)
ssPSEVYLEAER	D3ZNF5	N-Term(iTRAQ4plex); S2(Phospho)
eRsPALk	Q5M7V8	N-Term(iTRAQ4plex); S3(Phospho); K7(iTRA
dFYLATsPPDSFLDDHHL1	P54645	N-Term(iTRAQ4plex); S7(Phospho)
iINLGPVHPGPLsPEPQPm	Q5PPM8	N-Term(iTRAQ4plex); S13(Phospho); M19(O
dksDSETEGLVFAR	A0A0G2K0F3	N-Term(iTRAQ4plex); K2(iTRAQ4plex); S3(P
lSPsPSSR	G3V7U4;D3ZLC1	N-Term(iTRAQ4plex); S4(Phospho)
sLSGSsPcPk	A0A0G2K2M9	N-Term(iTRAQ4plex); S6(Phospho); C8(Carb
vPEkPPsPkEsPHFYR	D3ZQ55	N-Term(iTRAQ4plex); K4(iTRAQ4plex); S7(P
ascEALYSEMDPEER	Q02294	N-Term(iTRAQ4plex); S2(Phospho); C3(Carb
rGsGsVDETLFALPAASEF	F1LQN3	N-Term(iTRAQ4plex); S3(Phospho); S5(Phos
eLGSsVDEVEQLIR	A0A0G2K677	N-Term(iTRAQ4plex); S5(Phospho)
lLPYPTLAsPPFD	A0A0G2K9T8	N-Term(iTRAQ4plex); S9(Phospho)
lcTcGTADsDEGR	Q921A2	N-Term(iTRAQ4plex); C2(Carbamidomethyl);
rEDYQEILDsPIk	Q5M7A3	N-Term(iTRAQ4plex); S10(Phospho); K13(iT
tTPPTQkPPsPPMSGk	F1LYA6	N-Term(iTRAQ4plex); T1(Phospho); K7(iTRA
sLPkEEVAsEPEEAAsPITI	Q5RJN5	N-Term(iTRAQ4plex); K4(iTRAQ4plex); S9(P
sPmkEEAksPEk	F1LRZ7	N-Term(iTRAQ4plex); M3(Oxidation); K4(iTR
sQsVEPESQEk	Q6AXY7	N-Term(iTRAQ4plex); S3(Phospho); K11(iTR
lSSNcSGVEGDVtDEDEG/	F1M324	N-Term(iTRAQ4plex); C5(Carbamidomethyl);
aLLDVLQRPLsPGNSESL1	P97924	N-Term(iTRAQ4plex); S11(Phospho); K26(iT

gSSQPGQAPsAGTSDTAI	Q3ZB98	N-Term(iTRAQ4plex); S10(Phospho)
sVDAYDSYWESR	Q66HP6	N-Term(iTRAQ4plex); S1(Phospho)
eGGAGGGFGsPMDIFDM	P63036	N-Term(iTRAQ4plex); S10(Phospho)
vAQSDGEEsPAEEGQLLC	A0A0G2K0X9	N-Term(iTRAQ4plex); S9(Phospho)
iGDLQAAIEDEMEsDENEI	D3ZFD0	N-Term(iTRAQ4plex); S14(Phospho); K30(iT
rsPGAPSIR	A0A0G2K6R9;F1M9N9	N-Term(iTRAQ4plex); S2(Phospho)
fDDsDTEQIAEEGDDNLAI	D3ZYT8	N-Term(iTRAQ4plex); S4(Phospho); K25(iTR
aLEQSHSLPLPTPASTsPT	A0A0G2JU75	N-Term(iTRAQ4plex); S17(Phospho); K20(iT
lEEGVAsDEEEAEGAEPG	F1LNF0	N-Term(iTRAQ4plex); S7(Phospho)
fVsEGDGGR	Q9JID1	N-Term(iTRAQ4plex); S3(Phospho)
tAFDEAIAELDTLsEESYk	P63102	N-Term(iTRAQ4plex); S14(Phospho); K19(iT
vPEESFPQsPR	B2GUW9	N-Term(iTRAQ4plex); S9(Phospho)
tLIPsPPPRPk	D3ZHG8	N-Term(iTRAQ4plex); S5(Phospho); K11(iTR
sDAEEVDFAGWLcSTIGL	A0A0H2UHI2	N-Term(iTRAQ4plex); C13(Carbamidomethyl
gEGPAIPGDtPPPTPR	F1LSE6	N-Term(iTRAQ4plex); T10(Phospho)
eGVLYVGsk	P37377;A0A0G2JSQ1	N-Term(iTRAQ4plex); S8(Phospho); K9(iTRA
mNVPETMNEVLDmsDDE	A0A0G2K1Q7	N-Term(iTRAQ4plex); M13(Oxidation); S14(P
ccMETNsPcSSSGENETY	F1M3W5	N-Term(iTRAQ4plex); C1(Carbamidomethyl);
dEAcAPTVGALRPcAtYPQ	D4ABZ4	N-Term(iTRAQ4plex); C4(Carbamidomethyl);
gGssGEELEDEEPVk	A0A0G2JYC7	N-Term(iTRAQ4plex); S3(Phospho); S4(Phos
sSDWssEEEEPVR	Q6PST4	N-Term(iTRAQ4plex); S5(Phospho); S6(Phos
sPAGAksPAEAk	F1LRZ7	N-Term(iTRAQ4plex); K6(iTRAQ4plex); S7(P
gSYmEVEDNRSQVEtEDL	A0A0H2UHZ1	N-Term(iTRAQ4plex); M4(Oxidation); T15(Ph
tESVTSGPLsPEGsPSk	F8WFS9	N-Term(iTRAQ4plex); S10(Phospho); S14(Ph
aEYVFIVDsDGEDEATcR	D4A3C4	N-Term(iTRAQ4plex); S9(Phospho); C17(Car
aEsPETSAVESTQSTPQk	D3ZU56	N-Term(iTRAQ4plex); S3(Phospho); K18(iTR
aLEAELDAQHLSETFDNII	G3V8Q9	N-Term(iTRAQ4plex); S22(Phospho); K24(iT
gQPAQGPVSEESPPsPkP	Q80X08	N-Term(iTRAQ4plex); S15(Phospho); K17(iT
lTYSQEMcDNIQDMLGDL	D3ZU84	N-Term(iTRAQ4plex); C8(Carbamidomethyl);
eksSDDTTDAQMDEQDLN	B5DF63	N-Term(iTRAQ4plex); K2(iTRAQ4plex); S3(P
aMGDEESGESDSsPk	F1LSL6	N-Term(iTRAQ4plex); S13(Phospho); K15(iT
sDVcDLANYsPPVNR	B0BMZ5	N-Term(iTRAQ4plex); C4(Carbamidomethyl);
aISAPTsPTR	A0A0U1RS39	N-Term(iTRAQ4plex); S7(Phospho)
sRsDVDMDAAAEAAR	F1LQS9	N-Term(iTRAQ4plex); S3(Phospho)
lSNASASGQWsPTSDWIL	D4A0C3	N-Term(iTRAQ4plex); S11(Phospho); K21(iT
hYsQDcSSIk	F1M2D4	N-Term(iTRAQ4plex); S3(Phospho); C6(Carb
iNPYMsSPcHIEMILTEk	P24049	N-Term(iTRAQ4plex); S6(Phospho); C9(Carb
hDTPDAsPPR	G3V8F3	N-Term(iTRAQ4plex); S7(Phospho)
sRSPtPESR	Q3ZB99	N-Term(iTRAQ4plex); S1(Phospho); T5(Phos
kAEGAALSNATGAVESTS	Q9Z0W5;A0A0G2JWR2	N-Term(iTRAQ4plex); K1(iTRAQ4plex); S25(
tSLPPISPGRYsPIPk	Q63622	N-Term(iTRAQ4plex); S12(Phospho); K16(iT
aPSQAAESsPTk	A0A0G2K4S1	N-Term(iTRAQ4plex); S9(Phospho); K12(iTR
sAYQDYDSDsDVPEELk	F1LSG8	N-Term(iTRAQ4plex); S10(Phospho); K17(iT
gQLTksPLAQMEEER	F1LMC7	N-Term(iTRAQ4plex); K5(iTRAQ4plex); S6(P
ssPPPPLPAEERPGTk	D4A853	N-Term(iTRAQ4plex); S2(Phospho); K16(iTR
tIsLDk	D3Z8C4;P30835	N-Term(iTRAQ4plex); S3(Phospho); K6(iTRA
hTQEALkAsPk	P34926;Q63330	N-Term(iTRAQ4plex); K7(iTRAQ4plex); S9(P
rSsDPALTGLSTSVSDNNI	A0A0G2K9M8	N-Term(iTRAQ4plex); S3(Phospho)
lSDLQtQLsHEIQSDVLtIN	Q5EB90	N-Term(iTRAQ4plex); T6(Phospho); S9(Phos
dENERsSIsPMDEPVPDS	P15205;F1LRL9	N-Term(iTRAQ4plex); S6(Phospho); S9(Phos
eVGVYEALkDDsWLk	O88989	N-Term(iTRAQ4plex); K9(iTRAQ4plex); S12(
aGsLESDGcPk	A0A0G2JWK2	N-Term(iTRAQ4plex); S3(Phospho); C9(Carb
qADLSFSsPVEMk	G3V9R8	N-Term(iTRAQ4plex); S8(Phospho); K13(iTR
gkPVPVHGsR	P13233	N-Term(iTRAQ4plex); K2(iTRAQ4plex); S9(P
gHTEENLsPVSk	D3ZKX8	N-Term(iTRAQ4plex); S8(Phospho); K12(iTR

Information Classification: General

lSWGTENLDNVALSSsPIH	A0A0G2JZ56	N-Term(iTRAQ4plex); S16(Phospho); S20(Ph
sSDWSsEEEEPVRk	Q6PST4	N-Term(iTRAQ4plex); S6(Phospho); K14(iTR
iDsDDDGEkEGDEk	Q3SWT4	N-Term(iTRAQ4plex); S3(Phospho); K9(iTRA
eEQGkGDEEIGPcSASLsI	A0A0G2JYI0	N-Term(iTRAQ4plex); K5(iTRAQ4plex); C13(
qNsDPTSEGPGPSPNPPS	A0A0G2K382	N-Term(iTRAQ4plex); S3(Phospho); K29(iTR
tTEsPSATkPDDkEQSk	F1LQZ9	N-Term(iTRAQ4plex); S4(Phospho); K9(iTRA
tGNESGSNLsDSGSVk	A0A0G2JZF2	N-Term(iTRAQ4plex); S10(Phospho); K16(iT
tISIDENMEPSPtGDFYPSI	O70187	N-Term(iTRAQ4plex); T13(Phospho)
yDLDFksPDDASR	M0R5J4	N-Term(iTRAQ4plex); K6(iTRAQ4plex); S7(P
hVPsPLNLEEVQk	F1M1Y0	N-Term(iTRAQ4plex); S4(Phospho); K13(iTR
lYADSDsGEDSDkR	F1M9C8	N-Term(iTRAQ4plex); S7(Phospho); K13(iTR
aALANEDcPHIDQALtPDE	P25122	N-Term(iTRAQ4plex); C8(Carbamidomethyl);
aAAAPSASSTPTVIAsPk	F1LNZ5	N-Term(iTRAQ4plex); S16(Phospho); K18(iT
aVDQDDDENsEEDEMDS	D4A648	N-Term(iTRAQ4plex); S10(Phospho)
iAATILTsPDLR	P00507	N-Term(iTRAQ4plex); S8(Phospho)
tMIIsPER	Q9ET34	N-Term(iTRAQ4plex); S5(Phospho)
sDsETEGLVFAR	A0A0G2K0F3	N-Term(iTRAQ4plex); S3(Phospho)
rSEsPPAELPSLR	A0A0A0MXY4	N-Term(iTRAQ4plex); S4(Phospho)
sSGSLsPGLETEDPLEAR	D3ZF26	N-Term(iTRAQ4plex); S6(Phospho)
aLSGSATLVSSsPk	D3ZE26	N-Term(iTRAQ4plex); S11(Phospho); K13(iT
rGIsALLLNQGDGDRk	A0A0G2JZK7	N-Term(iTRAQ4plex); S4(Phospho); K16(iTR
nDLQsPTEHISDYGFGGV	F1M5M9	N-Term(iTRAQ4plex); S5(Phospho)
sTsLNERPk	D3ZLW4	N-Term(iTRAQ4plex); S3(Phospho); K9(iTRA
aPVIsGVTk	D3ZQL7	N-Term(iTRAQ4plex); S5(Phospho); K9(iTRA
nMAPSQQsPVR	F1LYA6	N-Term(iTRAQ4plex); S8(Phospho)
gEWQSETQDtMk	Q6MFX8	N-Term(iTRAQ4plex); T10(Phospho); K12(iT
tVLSSSEDAIEPVsPPEGM	A0A0G2JU91	N-Term(iTRAQ4plex); S14(Phospho)
wAHDkFsGEEGEIEDDES	Q5M7V8	N-Term(iTRAQ4plex); K5(iTRAQ4plex); S7(P
aLLLQGTEsLNR	F1LNC4	N-Term(iTRAQ4plex); S9(Phospho)
tEsPDVSHQPR	D3ZC89	N-Term(iTRAQ4plex); S3(Phospho)
vAAGHELQPLAIVDQRPS	P08050	N-Term(iTRAQ4plex); S19(Phospho)
sGsIDDSFTDFQEVPASSI	Q9JKC9	N-Term(iTRAQ4plex); S3(Phospho); K19(iTR
iQTSLTsASLGSADENSM	D3ZLW4	N-Term(iTRAQ4plex); S7(Phospho); K26(iTR
gsLNEQIALVLIR	A0A096MJJ6	N-Term(iTRAQ4plex); S2(Phospho)
tPVDYIDLPYsSSPSR	F1M820	N-Term(iTRAQ4plex); S11(Phospho)
eALVEPASEsPRPALAR	Q9JJ19	N-Term(iTRAQ4plex); S10(Phospho)
yLATAsTMDHAR	P02688	N-Term(iTRAQ4plex); S6(Phospho)
vAsPGGLR	A0A0H2UHF5	N-Term(iTRAQ4plex); S3(Phospho)
iPESSHPPLESSSSsFESC	A0A0G2KAV8	N-Term(iTRAQ4plex); S15(Phospho); K20(iT
sPAEAksPAEAksPAEVk	F1LRZ7	N-Term(iTRAQ4plex); K6(iTRAQ4plex); S7(P
dAATEEPGsPVk	F8WFS9	N-Term(iTRAQ4plex); S9(Phospho); K12(iTR
rGEDPPtPPPRPQk	A0A0G2K4S1	N-Term(iTRAQ4plex); T7(Phospho); K14(iTR
tVTPAssAk	P47942	N-Term(iTRAQ4plex); S6(Phospho); S7(Phos
lLLQVQHAsk	Q09073;Q05962	N-Term(iTRAQ4plex); S9(Phospho); K10(iTR
qkELsEGEEkGDPEk	O88420	N-Term(iTRAQ4plex); K2(iTRAQ4plex); S5(P
gPPLPPPPPLPLtTSPPPS	A0A0G2JV35	N-Term(iTRAQ4plex); T13(Phospho)
lADDEGDsDSEsVGQSR	Q2V6G6	N-Term(iTRAQ4plex); S8(Phospho); S12(Pho
sPVEVksPAsVksPSEAk	F1LRZ7	N-Term(iTRAQ4plex); K6(iTRAQ4plex); S7(P
qEPLGsDSEGVNcLAYDE	B2RYG6	N-Term(iTRAQ4plex); S6(Phospho); C13(Car
lPAsDGLDLSQAAAR	P23565;G3V8Q2	N-Term(iTRAQ4plex); S4(Phospho)
dGsPDAPATPEkEEVPFS	A0A0U1RRX4;F1MAQ5	N-Term(iTRAQ4plex); S3(Phospho); K12(iTR
vFGLHSVsR	Q62950	N-Term(iTRAQ4plex); S8(Phospho)
ekEVDGLLTSDPMGsPVS	B3DMA1	N-Term(iTRAQ4plex); K2(iTRAQ4plex); S15(
eSNsNQcVYAAHNSVVIDI	P31596	N-Term(iTRAQ4plex); S4(Phospho); C7(Carb
sGDETPGSEAPGDk	D4ABT8	N-Term(iTRAQ4plex); S1(Phospho); K14(iTR

Information Classification: General

tLSEENVYEDILDPPMk	D3ZLQ1	N-Term(iTRAQ4plex); T1(Phospho); K17(iTR
eLVGDTGSQEGDNEQPs	D3ZR35	N-Term(iTRAQ4plex); S17(Phospho); K31(iT
qSsMDSEGNFNPQPVDT	F1LRZ1	N-Term(iTRAQ4plex); S3(Phospho); K25(iTR
lAWLAEPDAHsHFDYDPR	P25122	N-Term(iTRAQ4plex); S11(Phospho)
kSsFSNSADDIk	B2RYU7	N-Term(iTRAQ4plex); K1(iTRAQ4plex); S3(P
nSTLSDSGMIDNLPDsPD	G3V8J0	N-Term(iTRAQ4plex); S16(Phospho); K22(iT
sPAEAksPASVk	F1LRZ7	N-Term(iTRAQ4plex); K6(iTRAQ4plex); S7(P
kLEkEEEEGISQEssEEEQ	Q8K585	N-Term(iTRAQ4plex); K1(iTRAQ4plex); K4(iT
cPsPTMSLPSSWk	B1VKB4	N-Term(iTRAQ4plex); C1(Carbamidomethyl);
sILLGsDNEDEkLNk	D3ZN60	N-Term(iTRAQ4plex); S6(Phospho); K12(iTR
gsYmEVEDNRSQVEtEDL	A0A0H2UHZ1	N-Term(iTRAQ4plex); S2(Phospho); M4(Oxid
eTWDTAEEDsGTDSEYD	F1MAA1	N-Term(iTRAQ4plex); S10(Phospho); K21(iT
aSEVQELQNDLDRETsSL	A0A0G2QC53	N-Term(iTRAQ4plex); S16(Phospho); K25(iT
ekAEEEGGsEEEVGDksP	P12839	N-Term(iTRAQ4plex); K2(iTRAQ4plex); S9(P
lTVEEAtASVSEGGGLQG	F1LWT1	N-Term(iTRAQ4plex); T7(Phospho); M21(Ox
vPSTYPSTLSEELsPPSH	F1LW91	N-Term(iTRAQ4plex); S14(Phospho); K21(iT
tVPPAVPGVtFLSGGQSE	P09117	N-Term(iTRAQ4plex); T10(Phospho)
rESVAsGDDRAEEDMDE	P15205;F1LRL9	N-Term(iTRAQ4plex); S6(Phospho); K21(iTR
gGDSIGEtPtPGASk	Q9ET34	N-Term(iTRAQ4plex); T8(Phospho); T10(Pho
eVEEDSEDEEmsEDEDD	Q9QZX1	N-Term(iTRAQ4plex); M11(Oxidation); S12(P
gGsFEMTDDDSAIR	A0A0G2JW51	N-Term(iTRAQ4plex); S3(Phospho)
aSESDTEEFYDAPEDVHL	A0A0G2K7S6;A0A0G2JX	N-Term(Acetyl); S26(Phospho); K29(iTRAQ4
gSAEsPDEGITTTEGEGE	P15205;F1LRL9	N-Term(iTRAQ4plex); S5(Phospho); C19(Ca
aDYAQLLEDMQNAFRsLE	D4A133	N-Term(iTRAQ4plex); S16(Phospho)
rLTGPPPsFEGk	Q09QM4	N-Term(iTRAQ4plex); S8(Phospho); K12(iTR
dLEFcsTEEEkEPGDR	M0RC12	N-Term(iTRAQ4plex); C5(Carbamidomethyl);
dLLsISDGR	D3ZCQ9	N-Term(iTRAQ4plex); S4(Phospho)
sPNPSDGGGDTPLAQsD	Q331S7	N-Term(iTRAQ4plex); S16(Phospho); C31(Ca
eQTGQMADHLEGLssDD	D4A8C8	N-Term(iTRAQ4plex); S14(Phospho); S15(Ph
tAQDPGIsPSQSLcAEGS	A0A0G2JZF5	N-Term(iTRAQ4plex); S8(Phospho); C14(Ca
lRsEDGVEGDLGETQSR	A0A0G2JU96	N-Term(iTRAQ4plex); S3(Phospho)
vAILtDDEEEQkR	A0A0G2JW92	N-Term(iTRAQ4plex); T5(Phospho); K12(iTR
sPEAktPAkEEAk	F1LRZ7	N-Term(iTRAQ4plex); K5(iTRAQ4plex); T6(P
mkGEtPVNSTMSIGQAR	G3V8K2	N-Term(Acetyl); K2(iTRAQ4plex); T5(Phosph
ssSSEEASQELESDDG	A0A0G2K8X3	N-Term(iTRAQ4plex); S2(Phospho)
sVDIsPTR	A0A0G2K2R0	N-Term(iTRAQ4plex); S5(Phospho)
rRsPPPR	A0A0U1RRV7	N-Term(iTRAQ4plex); S3(Phospho)
sEsPGRMDEPk	D3ZGJ3	N-Term(iTRAQ4plex); S3(Phospho); K11(iTR
isLNDEEEEk	D4A831	N-Term(iTRAQ4plex); S2(Phospho); K10(iTR
wGQTLPttSSTDEGNPk	A0A0G2K677	N-Term(iTRAQ4plex); T7(Phospho); T8(Phos
lEASYSDsPPGEEDLLVH	Q5FWU3	N-Term(iTRAQ4plex); S8(Phospho); K24(iTR
asNETLISYGIPDSDEQR	D3Z8L2	N-Term(iTRAQ4plex); S2(Phospho)
rYsPPIQR	B2RYB3;A0A0G2K4F6	N-Term(iTRAQ4plex); S3(Phospho)
hIkEEPLsEEEPcTSTAVP	Q9QZ86	N-Term(iTRAQ4plex); K3(iTRAQ4plex); S8(P
dkDSsEPDENPATEPR	D3ZEI4	N-Term(iTRAQ4plex); K2(iTRAQ4plex); S5(P
aFLAELEQNsPk	D3ZHV2	N-Term(iTRAQ4plex); S10(Phospho); K12(iT
eMLLEDVGsEEEPEEDDE	A0A0G2K7X3	N-Term(iTRAQ4plex); S9(Phospho); K24(iTR
rLPSSPAsPSPk	A0A0G2K0F3	N-Term(iTRAQ4plex); S8(Phospho); K12(iTR
tGQAGsLSGSPkPFSPQA	A0A0G2K9F7	N-Term(iTRAQ4plex); S6(Phospho); K12(iTR
lEsFESLR	A0A1B0GWY5	N-Term(iTRAQ4plex); S3(Phospho)
etkSPVkEEAk	F1LRZ7	N-Term(iTRAQ4plex); T2(Phospho); K3(iTRA
tSSLSsDSSNLR	Q331S7	N-Term(iTRAQ4plex); S6(Phospho)
sLAESGLSWFsESEEktPk	F1M031	N-Term(iTRAQ4plex); S11(Phospho); K16(iT
kDFDQDAsDkEFPENVEk	D4A997	K1(iTRAQ4plex); N-Term(iTRAQ4plex); S8(P
sSAsAPDVDDPEAFPALA	Q6AXS5	N-Term(iTRAQ4plex); S4(Phospho)

Information Classification: General

aEETETASsPPEkDR	B2RZ73	N-Term(iTRAQ4plex); S9(Phospho); K13(iTR
lEVPAERsPR	A0A0G2JTA7	N-Term(iTRAQ4plex); S8(Phospho)
vGSEkGsTGsRDGk	Q80XF7	N-Term(iTRAQ4plex); K5(iTRAQ4plex); S7(P
tVsLPTR	F1M5N7	N-Term(iTRAQ4plex); S3(Phospho)
hMDPPPAPMQDRsPsPR	P15205;F1LRL9	N-Term(iTRAQ4plex); S13(Phospho); S15(Ph
sSPNVANQPPsPGGk	F1LT10	N-Term(iTRAQ4plex); S11(Phospho); K15(iT
dFATAPPTsPAk	F1MAF8	N-Term(iTRAQ4plex); S9(Phospho); K12(iTR
sNsWQGNVGGNk	M0R5P8	N-Term(iTRAQ4plex); S3(Phospho); K12(iTR
gDEDFQsDSDSFNPTLW	P70587	N-Term(iTRAQ4plex); S7(Phospho)
eEQTDTsDGESVTHHIR	A0A0G2K6Q5	N-Term(iTRAQ4plex); S7(Phospho)
fNAsQLITQR	A0A0G2KB63	N-Term(iTRAQ4plex); S4(Phospho)
kNVLGHMQQGGsPTPFD	Q52KS1	N-Term(iTRAQ4plex); K1(iTRAQ4plex); S12(
aASPPsYTLDLGESQLAP	A0A0G2JT26	N-Term(iTRAQ4plex); S6(Phospho); K22(iTR
vVEcEDGSsDkDARPALE	D3ZKX8	N-Term(iTRAQ4plex); C4(Carbamidomethyl);
sPGHEGEGGGEWPGISG	F1LSB5	N-Term(iTRAQ4plex); S1(Phospho)
gRsFMNNWEVYk	Q52KS1	N-Term(iTRAQ4plex); S3(Phospho); K12(iTR
tDTAADGEtSATEsDQEE	G3V874;A0A0G2K1Q9	N-Term(iTRAQ4plex); T9(Phospho); S14(Pho
nSLETVGtPDsGRGR	Q62747	N-Term(iTRAQ4plex); T8(Phospho); S11(Pho
sFDLGSPkPGDETTPQG	P97924	N-Term(iTRAQ4plex); K8(iTRAQ4plex); S19(
sEcDsSPEPk	A0A0G2K2M9	N-Term(iTRAQ4plex); C3(Carbamidomethyl);
aPTAAPsPEPR	A0A0G2JW97	N-Term(iTRAQ4plex); S7(Phospho)
lQLGSsLDSSNASVSSNL	F1LVR4	N-Term(iTRAQ4plex); S6(Phospho); K24(iTR
mYPESTTGsPAR	P62483	N-Term(iTRAQ4plex); S9(Phospho)
sHsLEAPGk	G3V7T8	N-Term(iTRAQ4plex); S3(Phospho); K9(iTRA
sPAEVksPVTVksPAEAk	F1LRZ7	N-Term(iTRAQ4plex); K6(iTRAQ4plex); S7(P
sPsPLQRPASLDLDSR	Q63358	N-Term(iTRAQ4plex); S3(Phospho)
aEEEcENDGVGGGLLcS	O88377	N-Term(iTRAQ4plex); C6(Carbamidomethyl);
ySscSTIFLDDSTVSQPNL	F1MA89	N-Term(iTRAQ4plex); S3(Phospho); C4(Carb
nFTQsPEQQR	Q8R4T5	N-Term(iTRAQ4plex); S5(Phospho)
eEGTGESAGGsPAR	B5DF41	N-Term(iTRAQ4plex); S11(Phospho)
wAAHGtSPEDFSLTLGAR	D3ZNS1	N-Term(iTRAQ4plex); T6(Phospho)
sAPDGSNAsPSHTASQPN	D4A1V8	N-Term(iTRAQ4plex); S9(Phospho); K24(iTR
nsSYFVEWIPNNVk	P85108;B4F7C2;P69897;(	N-Term(iTRAQ4plex); S2(Phospho); K14(iTR
qFLISPPAsPPVGWk	Q6IN33	N-Term(iTRAQ4plex); S9(Phospho); K15(iTR
eETQDTGTGsDDGGFSE	P97887	N-Term(iTRAQ4plex); S10(Phospho)
sSPAPLsAALtAkGtGGGG	F1LXC7	N-Term(iTRAQ4plex); S7(Phospho); T11(Pho
lDQPVsAPPsPR	D4AEK9	N-Term(iTRAQ4plex); S6(Phospho); S10(Pho
lADDEGDsDSESVGQSR	Q2V6G6	N-Term(iTRAQ4plex); S8(Phospho)
tsPEPQREDAsPAPGTTA	A0A0G2K7C1	N-Term(iTRAQ4plex); S2(Phospho); S11(Pho
eEQSGPVDEkGNDSDGE	Q4KLL7	N-Term(iTRAQ4plex); K10(iTRAQ4plex); S20
ikEEVLsESETEAHQDAAA	D3ZUB7	N-Term(iTRAQ4plex); K2(iTRAQ4plex); S7(P
eQDsPPMkPGVLDAIR	Q64548	N-Term(iTRAQ4plex); S4(Phospho); K8(iTRA
vGVEELsEEDQNEHR	D3ZCI2	N-Term(iTRAQ4plex); S7(Phospho)
gAGAASSTDSLDtLSNGP	F1LRZ7	N-Term(iTRAQ4plex); T13(Phospho); C21(Ca
eAGsPAQEFSk	D3ZY45	N-Term(iTRAQ4plex); S4(Phospho); K11(iTR
gcSQDDEcVsLR	G3V849	N-Term(iTRAQ4plex); C2(Carbamidomethyl);
kAEGAGtEEEGTQk	Q05175;A0A0G2K1L8	N-Term(iTRAQ4plex); K1(iTRAQ4plex); T7(P
yEEEVLsREDAEGR	P19527	N-Term(iTRAQ4plex); S7(Phospho)
hTESGPAELDsPk	Q99ND8	N-Term(iTRAQ4plex); S11(Phospho); K13(iT
lPISsGSTSSSR	F1LMW3	N-Term(iTRAQ4plex); S5(Phospho)
sPQGLSDTGYSSDGISGS	Q9JKS6;D3Z9C7	N-Term(iTRAQ4plex); S1(Phospho); K31(iTR
lYQSAGGMPGGMPGGFF	M0RCB1	N-Term(iTRAQ4plex); T32(Phospho)
qQPVSESPPtDEAAGSGG	Q4G061	N-Term(iTRAQ4plex); T10(Phospho)
nsLESISSIDR	D3ZWS0	N-Term(iTRAQ4plex); S2(Phospho)
sLkEENFVASVELWNk	A0A0G2KA27	N-Term(iTRAQ4plex); S1(Phospho); K3(iTRA

Information Classification: General

sVIEGVDEDsDLsDDEPSA	Q6AZ61	N-Term(iTRAQ4plex); S10(Phospho); S13(Ph
fFVSVPGNVLAtLNGSVLD	B0VXR4	N-Term(iTRAQ4plex); T12(Phospho); K38(iT
yAYFNGcSsPTAPLSPMS	P08050	N-Term(iTRAQ4plex); C7(Carbamidomethyl);
fPHSsPNVk	P49621	N-Term(iTRAQ4plex); S5(Phospho); K9(iTRA
aEEEGGsEEEVGDksPQE	P12839	N-Term(iTRAQ4plex); S7(Phospho); K14(iTR
rPADIRsPEQVksPAk	F1LRZ7	N-Term(iTRAQ4plex); S7(Phospho); K12(iTR
gTGVVtSVPSDSPDDFAA	Q5PPJ6	N-Term(iTRAQ4plex); T6(Phospho)
lDHALsSPSSPcEEIk	Q6AYN4	N-Term(iTRAQ4plex); S6(Phospho); C12(Ca
aEGEPQEEsPLk	B1WC16	N-Term(iTRAQ4plex); S9(Phospho); K12(iTR
lTYHGVsPck	B2GV23	N-Term(iTRAQ4plex); S7(Phospho); C9(Carb
mEEPLGsPPAALSALEk	A0A0G2K2U5	N-Term(Acetyl); M1(Oxidation); S7(Phospho)
nEVNTmLGQsTEELR	P02650	N-Term(iTRAQ4plex); M6(Oxidation); S10(Ph
yGPVSVADTTGSGAADAI	B5DEN5	N-Term(iTRAQ4plex); K18(iTRAQ4plex); S28
lGGksPPPPPPPPR	M0R5H1	N-Term(iTRAQ4plex); K4(iTRAQ4plex); S5(P
lAHAcGSDWIPVGsSGSk	O35095	N-Term(iTRAQ4plex); C5(Carbamidomethyl);
mQVPTAEEMSSLTPESsI	B2DD29	N-Term(iTRAQ4plex); S17(Phospho); K22(iT
vsPVNDVDVGVYQLmQS	D4A7H9	N-Term(iTRAQ4plex); S2(Phospho); M15(Ox
rVEsEESGDEEGkk	Q5HZV9	N-Term(iTRAQ4plex); S4(Phospho); K13(iTR
vSmILQsPAFcEELESMIQ	A0A0G2JSM7;D3ZZ99	N-Term(iTRAQ4plex); M3(Oxidation); S7(Pho
sDEEDEDsDFGEEQR	G3V9J8	N-Term(iTRAQ4plex); S8(Phospho)
tPPSNLsPIEDAsPTEELR	Q9JKS6;G3V984;D3Z9C7	N-Term(iTRAQ4plex); S7(Phospho); S13(Pho
kLEESASFESLsPSSRPDS	G3V8Q9	N-Term(iTRAQ4plex); K1(iTRAQ4plex); S12(
vIHFVSGETMEEYstDEDE	D3ZIM7	N-Term(iTRAQ4plex); S14(Phospho); T15(Ph
lsSPPREFPPATAR	F1M7S2	N-Term(iTRAQ4plex); S2(Phospho)
eNIPTPALEPQTTVIHNPD	P15791	N-Term(iTRAQ4plex); K22(iTRAQ4plex); T30
vDNSSLTGESEPQtRSPD	P06685	N-Term(iTRAQ4plex); T14(Phospho)
eNLLLsDSPPcR	F1LQT3	N-Term(iTRAQ4plex); S6(Phospho); C11(Ca
sRtPPVTR	A0A0G2K2M9	N-Term(iTRAQ4plex); S1(Phospho); T3(Phos
iDMIFTPGPPStPk	F1LPA2	N-Term(iTRAQ4plex); T12(Phospho); K14(iT
lGVQLcGPGssPEPEHGS	A0A0G2K0E5	N-Term(iTRAQ4plex); C6(Carbamidomethyl);
kSDSNAsFLR	A0A0G2K6R9	N-Term(iTRAQ4plex); K1(iTRAQ4plex); S7(P
qQYQSsPDAAVQ	O55173	N-Term(iTRAQ4plex); S6(Phospho)
eAINNVDTSSSDFtILQEM	A0A0G2JW97	N-Term(iTRAQ4plex); T14(Phospho); K30(iT
sPVEVk	F1LRZ7	N-Term(iTRAQ4plex); S1(Phospho); K6(iTRA
yFGTDAVPDGsDDDEVTS	D3ZYQ8	N-Term(iTRAQ4plex); S11(Phospho)
tTDEPIEETEAPcEIsELYD	Q56A26	N-Term(iTRAQ4plex); C13(Carbamidomethyl
rLSTsPVR	G3V874;A0A0G2K1Q9	N-Term(iTRAQ4plex); S5(Phospho)
kPGGGDLPLHSAsDDETF	D4A0X3	N-Term(iTRAQ4plex); K1(iTRAQ4plex); S13(
eGPIGGEsDSEEVR	D4A0A1	N-Term(iTRAQ4plex); S8(Phospho)
tSLAAEENDsSSEGPEEEI	D3ZKX8	N-Term(iTRAQ4plex); S10(Phospho)
rEQDsPPMkPGVLDAIR	Q64548	N-Term(iTRAQ4plex); S5(Phospho); K9(iTRA
aGIPQHHPPmAQNLQYPI	F1LNP8	N-Term(iTRAQ4plex); M10(Oxidation); S20(P
sAsSGAEGDVSSEREP	B0K014	N-Term(iTRAQ4plex); S3(Phospho)
dsDDEEEVVHVDR	P61265	N-Term(iTRAQ4plex); S2(Phospho)
dkEDLVcSAALHsPQEsPV	F1LQN3	N-Term(iTRAQ4plex); K2(iTRAQ4plex); C7(C
qScLHPFTEDDAVDPNDs	Q80ZB2	N-Term(iTRAQ4plex); C3(Carbamidomethyl);
sPTNsSEIFTPAHEENVR	B0BN72	N-Term(iTRAQ4plex); S5(Phospho)
sLTNSHLEk	Q9JM15	N-Term(iTRAQ4plex); S1(Phospho); K9(iTRA
lADsPsRSPSkVEVTEk	A0A0G2K2V4	N-Term(iTRAQ4plex); S4(Phospho); S6(Phos
sETAPVPQPASVAPEkPA	D4A3K5	N-Term(Acetyl); S1(Phospho); K16(iTRAQ4p
sVYSDRDDESPDssPEEQ	A0A0G2K6R9;F1M9N9	N-Term(iTRAQ4plex); S13(Phospho); S14(Ph
fSGEEGEIEDDEsGTENR	Q5M7V8	N-Term(iTRAQ4plex); S13(Phospho)
qFVsQELVNL	Q6AY17	N-Term(iTRAQ4plex); S4(Phospho)
eMEAPkSPGtAR	D4ABN3	N-Term(iTRAQ4plex); K6(iTRAQ4plex); T10(
qPPIsPTSk	A0A0G2K6R9;F1M9N9	N-Term(iTRAQ4plex); S5(Phospho); K9(iTRA

Information Classification: General

aPsMEGTTGk	G3V927	N-Term(iTRAQ4plex); S3(Phospho); K10(iTR
iLGIPVIITEQyPk	F2Z3T7	N-Term(iTRAQ4plex); Y12(Phospho); K14(iT
aTsPLPck	Q6AYK3	N-Term(iTRAQ4plex); S3(Phospho); C7(Carb
gSDDAPDADTAIINAEGG	Q1WIM3	N-Term(iTRAQ4plex); S20(Phospho); K25(iT
iAAPELHkGDsDSEEDEP/	B2RYA6	N-Term(iTRAQ4plex); K8(iTRAQ4plex); S11(
eNSAAPsPIRPHSTSPAkE	Q920Q0	N-Term(iTRAQ4plex); S7(Phospho); K18(iTR
sLGLSLsGGEQEDAVR	P81799	N-Term(iTRAQ4plex); S7(Phospho)
vQSLEGEkLsPksDIsPLtPl	P15205;F1LRL9	N-Term(iTRAQ4plex); K8(iTRAQ4plex); S10(
sSDEENGPPSsPDLDR	D3ZH75	N-Term(iTRAQ4plex); S11(Phospho)
sPTMEQAVQTASAHLPAF	A0A0G2JUK2	N-Term(iTRAQ4plex); S1(Phospho)
qEsMPILPSWR	D3ZDP7	N-Term(iTRAQ4plex); S3(Phospho)
gPPEGSETmPYIDEsPTm	A0A0G2JTR4	N-Term(iTRAQ4plex); M9(Oxidation); S15(Ph
gLVAAYSGDsDNEEELVE	Q5EB71	N-Term(iTRAQ4plex); S10(Phospho)
sADEcEGGSTPRPPEDSL	D4A1G8	N-Term(iTRAQ4plex); C5(Carbamidomethyl);
hTPVsPSGk	A0A0G2K6R9;F1M9N9	N-Term(iTRAQ4plex); S5(Phospho); K9(iTRA
tDGDWDDQEVLDYFsDkE	B1WC16	N-Term(iTRAQ4plex); S15(Phospho); K17(iT
vVEPANsDSDSELGNIPk	Q14TE9	N-Term(iTRAQ4plex); S7(Phospho); K18(iTR
kPPPPASPGssDSSAR	A0A0H2UHQ3	K1(iTRAQ4plex); N-Term(iTRAQ4plex); S10(
hMsQPEPEQk	E9PST5	N-Term(iTRAQ4plex); S3(Phospho); K10(iTR
tLsDYNIQk	P0CG51	N-Term(iTRAQ4plex); S3(Phospho); K9(iTRA
tPPPSQGk	P02688	N-Term(iTRAQ4plex); T1(Phospho); K8(iTRA
aLDIDsDEEPEPk	D4A1D2	N-Term(iTRAQ4plex); S6(Phospho); K13(iTR
gTYLTHEAkGsDDAPDAD	Q1WIM3	N-Term(iTRAQ4plex); K9(iTRAQ4plex); S11(
sRSPGTPAGEGSGSPPk	A0A0G2JSM7;D3ZZ99	N-Term(iTRAQ4plex); S1(Phospho); K17(iTR
kVVsPLQsPTk	A0A0G2K6Z8	N-Term(iTRAQ4plex); K1(iTRAQ4plex); S4(P
rEFITGDVEPTDAESAWH	Q5U2Z3	N-Term(iTRAQ4plex); S19(Phospho); K26(iT
rLSNASASGQWsPtSDWI	D4A0C3	N-Term(iTRAQ4plex); S12(Phospho); T14(Ph
sDGSLDDGEGVHR	Q7TNK0	N-Term(iTRAQ4plex); S1(Phospho)
sHsANDSEEFFREDDGG/	Q4G008	N-Term(iTRAQ4plex); S3(Phospho)
eLGsTEDIYLASR	A0A0G2K988	N-Term(iTRAQ4plex); S4(Phospho)
lVLASIDQADFQGFtYVNP	P63319	N-Term(iTRAQ4plex); T15(Phospho)
gMcEQSYWNPLEEPPcQs	F1LUY5	N-Term(iTRAQ4plex); C3(Carbamidomethyl);
eHLGQGSsQEMEk	G3V7X2	N-Term(iTRAQ4plex); S8(Phospho); K13(iTR
eLFDYsPPLHk	B1WC16	N-Term(iTRAQ4plex); S6(Phospho); K11(iTR
mEPPsPTQEGGPSQNAL	A0A0G2JXK1	N-Term(iTRAQ4plex); S5(Phospho); K36(iTR
akEEGsWk	P07340	N-Term(iTRAQ4plex); K2(iTRAQ4plex); S6(P
dDDEMNTWIQAITSAISSI	G3V6S0	N-Term(iTRAQ4plex); K20(iTRAQ4plex); S29
aPTPsSGLDSk	D4A168	N-Term(iTRAQ4plex); S5(Phospho); K11(iTR
aVEEQGDDQDsEkSkPAC	D4ABT8	N-Term(iTRAQ4plex); S11(Phospho); K13(iT
sATTTPSGsPR	P0C1X8	N-Term(iTRAQ4plex); S9(Phospho)
sSsFTLLQSLAIEDSR	F1LZX5	N-Term(iTRAQ4plex); S3(Phospho)
eEQSGPVDEkGNDsDGE,	Q4KLL7	N-Term(iTRAQ4plex); K10(iTRAQ4plex); S14
vksPGsGHVk	D3ZQG6	N-Term(iTRAQ4plex); K2(iTRAQ4plex); S3(P
nSQEDSEDsEEkDVk	A0A0G2K7X3	N-Term(iTRAQ4plex); S9(Phospho); K12(iTR
gFGsEEGSR	Q27W01	N-Term(iTRAQ4plex); S4(Phospho)
gSIPSASsPTSPALPR	Q9WV48	N-Term(iTRAQ4plex); S8(Phospho)
eSPPQPPADDGSEEPGsI	D4A1Q2	N-Term(iTRAQ4plex); S17(Phospho); K23(iT
lQTHDESSLPLPQPsPFM	A0A0G2KAV8	N-Term(iTRAQ4plex); S15(Phospho)
gsLGkDtTSPMELAALEk	F1LPG3	N-Term(iTRAQ4plex); S2(Phospho); K5(iTRA
gLYDGPVcEVsVTPk	P47942	N-Term(iTRAQ4plex); C8(Carbamidomethyl);
gRLtPsPDIIVLSDNEAsSP	Q4V8E1	N-Term(iTRAQ4plex); T4(Phospho); S6(Phos
sDsDSSTLPR	M0R8P6	N-Term(iTRAQ4plex); S3(Phospho)
csPPVPSPLASEk	Q3KR59	N-Term(iTRAQ4plex); C1(Carbamidomethyl);
gSLIstDSGNSLPDRNPEk	F1LSM8	N-Term(iTRAQ4plex); S5(Phospho); T6(Phos
aksPMkEEAk	F1LRZ7	N-Term(iTRAQ4plex); K2(iTRAQ4plex); S3(P

Information Classification: General

dLLSDLQDISDsER	D4A3G2	N-Term(iTRAQ4plex); S12(Phospho)
ekLQEEGGsEEEEVGSPS	D4A1H8	N-Term(iTRAQ4plex); K2(iTRAQ4plex); S9(P
hIVSNDSsDSDDEAQGPk	P50393	N-Term(iTRAQ4plex); S8(Phospho); K18(iTR
rPLsPQSk	Q6TQE1	N-Term(iTRAQ4plex); S4(Phospho); K8(iTRA
rLPSTDAsPASGGk	A0A0G2K3Z3	N-Term(iTRAQ4plex); S8(Phospho); K14(iTR
hDsPEDVk	O35814	N-Term(iTRAQ4plex); S3(Phospho); K8(iTRA
wsPPQNYk	Q499R6	N-Term(iTRAQ4plex); S2(Phospho); K8(iTRA
sSPSTGsLDSGSESk	Q5FVC7	N-Term(iTRAQ4plex); S7(Phospho); K15(iTR
rPSEsDkEEELEk	Q5QD51	N-Term(iTRAQ4plex); S5(Phospho); K7(iTRA
aSAAEGSEAsPPsLR	D4A5J1	N-Term(iTRAQ4plex); S10(Phospho); S13(Ph
tVPPAVPGVTFLsGGQSE	P05065	N-Term(iTRAQ4plex); S13(Phospho); K30(iT
rAGDMLEDsPkRPk	Q8VHK7	N-Term(iTRAQ4plex); S9(Phospho); K11(iTR
fGLEGDEESTMLEESVsP	D3ZA84	N-Term(iTRAQ4plex); S17(Phospho); K19(iT
rsSASPDVLQGQPPR	A0A096MJH0	N-Term(iTRAQ4plex); S2(Phospho)
nSVTEDsPQPPLPSVR	F1M842	N-Term(iTRAQ4plex); S7(Phospho)
qLSVPAsDEEDEVPVPVP	Q6MG08	N-Term(iTRAQ4plex); S7(Phospho)
sSsLESLQTAVAEVR	F1LW22	N-Term(iTRAQ4plex); S3(Phospho)
nGILAIEGTGSDVDDDMs	D3ZC84	N-Term(iTRAQ4plex); S18(Phospho); K22(iT
eYPsPPPSPLR	A0A0G2K9J0	N-Term(iTRAQ4plex); S4(Phospho)
gDMsDEDDENEFFDAPEI	D4A9D8	N-Term(iTRAQ4plex); S4(Phospho); M21(Ox
eSEEEEkkEEsAGEEQAA	P19527	N-Term(iTRAQ4plex); K7(iTRAQ4plex); K8(iT
gRLDsGQLGLYPANYVEA	Q9Z0W5;A0A0G2JWR2	N-Term(iTRAQ4plex); S5(Phospho)
dtPGGRSDGDTDSVSTM	A0A0G2K382	N-Term(iTRAQ4plex); T2(Phospho)
kGssEESVDEDR	A0A0G2K6R9;F1M9N9	K1(iTRAQ4plex); N-Term(iTRAQ4plex); S3(P
eDAPPEDkEsESEAkLDG	Q91XU8	N-Term(iTRAQ4plex); K8(iTRAQ4plex); S10(
vQTtPPPTIQGQk	P0C1X8	N-Term(iTRAQ4plex); T4(Phospho); K13(iTR
kPASVsPtTPPSPTEGEAS	G3V7G0	K1(iTRAQ4plex); N-Term(iTRAQ4plex); S6(P
eELAsDLEEMATSSISVPk	Q5RJN5	N-Term(iTRAQ4plex); S5(Phospho); K19(iTR
skDkEDLVcSAALHsPQEs	F1LQN3	N-Term(iTRAQ4plex); K2(iTRAQ4plex); K4(iT
sFFtDGsLDSWGTSEDAE	F1M2D4	N-Term(iTRAQ4plex); T4(Phospho); S7(Phos
sAFTtSDVVR	Q9WUY1	N-Term(iTRAQ4plex); T5(Phospho)
vASPSQGQVGSSsPk	F1M2K6	N-Term(iTRAQ4plex); S13(Phospho); K15(iT
aDFsEEYk	P13233	N-Term(iTRAQ4plex); S4(Phospho); K8(iTRA
vDIITEEmPENALPsDEDD	B5DFK6	N-Term(iTRAQ4plex); M8(Oxidation); S15(Ph
ktSDDGGDsPEHDADIPE	D3ZJK3	K1(iTRAQ4plex); N-Term(iTRAQ4plex); T2(P
nFTNsPEESAk	Q5GFD9	N-Term(iTRAQ4plex); S5(Phospho); K11(iTR
aAEEEDsDEDAVLAPSGV	M0R920	N-Term(iTRAQ4plex); S7(Phospho)
fkGPGDTSNFDDYEEEEI	A1L1M0	N-Term(iTRAQ4plex); K2(iTRAQ4plex); S21(
gtPQtPkDDPVLTLLSNR	D4A1C0	N-Term(iTRAQ4plex); T2(Phospho); T5(Phos
gmYDGPVYEVPAtPk	Q62950	N-Term(iTRAQ4plex); M2(Oxidation); T13(Ph
eVEDkEsEGEEEDEDEDL	O35986	N-Term(iTRAQ4plex); K5(iTRAQ4plex); S7(P
sLSmDsQVPVYsPSLDLk	D3ZVF2	N-Term(iTRAQ4plex); M4(Oxidation); S6(Pho
aANktPPksPGDPAk	D3ZQL7	N-Term(iTRAQ4plex); K4(iTRAQ4plex); T5(P
eSLkEEDEsDDDNM	P18422	N-Term(iTRAQ4plex); K4(iTRAQ4plex); S9(P
rVPsPTPVPk	A0A0G2K2M9	N-Term(iTRAQ4plex); S4(Phospho); K10(iTR
tRsFDNLTTTcDNMVPLAS	Q5PQT2	N-Term(iTRAQ4plex); S3(Phospho); C11(Ca
hGAGGIsPAGQEVDANLC	D3ZNK1	N-Term(iTRAQ4plex); S7(Phospho); K19(iTR
nDSIVTPsPPQAR	A0A0G2KAL9	N-Term(iTRAQ4plex); S8(Phospho)
aLsPTGSSR	Q62768	N-Term(iTRAQ4plex); S3(Phospho)
kIIPTLEEGLQLPSPtATSQ	P61980	N-Term(iTRAQ4plex); K1(iTRAQ4plex); T16(
fSTYSQsPPDTPSLR	Q4FZU8	N-Term(iTRAQ4plex); S7(Phospho)
lLkPGEEPSEYtDEEDTkD	Q5XIU9	N-Term(iTRAQ4plex); K3(iTRAQ4plex); T12(
dEPPsEGEAEEEEk	P19527	N-Term(iTRAQ4plex); S5(Phospho); K14(iTR
aQPGsPQALGGPHGPAT	D3ZE17	N-Term(iTRAQ4plex); S5(Phospho); K19(iTR
qWNLTsPSEETLNEGELL	A0A0G2K7K9	N-Term(iTRAQ4plex); S6(Phospho); K22(iTR

Information Classification: General

stkPWNGGRsMLDTIk	F1LUR6	N-Term(iTRAQ4plex); T2(Phospho); K3(iTRA
tVsDNsLSSSk	Q8R511	N-Term(iTRAQ4plex); S3(Phospho); S6(Phos
sLPELDRDk	A0A0G2K0F3	N-Term(iTRAQ4plex); S1(Phospho); K9(iTRA
eIkPSERPLsPk	Q5XXR3	N-Term(iTRAQ4plex); K3(iTRAQ4plex); S10(
aPPAkPksPPRPAkPRsPP	Q63327	N-Term(iTRAQ4plex); K5(iTRAQ4plex); K7(iT
kVVDYSQFQEsDDADEDY	A0A0G2K7X3	N-Term(iTRAQ4plex); K1(iTRAQ4plex); S11(
sPSDLsPESPVLSSPPk	A0A0G2K8P5	N-Term(iTRAQ4plex); S6(Phospho); K17(iTR
eIEQEETLSAPsPsPSPSS	G3V857	N-Term(iTRAQ4plex); S12(Phospho); S14(Ph
eLALssPEDLTQDFEELkR	P34926;Q63330	N-Term(iTRAQ4plex); S5(Phospho); S6(Phos
dFyELEPEk	P53534	N-Term(iTRAQ4plex); Y3(Phospho); K9(iTRA
aLVADEPEDLDtEDEGLIS	G3V7X4	N-Term(iTRAQ4plex); T12(Phospho)
qPsIELPSmAVASTk	Q4QQV6	N-Term(iTRAQ4plex); S3(Phospho); M9(Oxid
rIDIsPSTFR	Q5M7V8	N-Term(iTRAQ4plex); S5(Phospho)
kQsQQLELLESELR	D4A7M0	N-Term(iTRAQ4plex); K1(iTRAQ4plex); S3(P
rEsGEGEEEVADSAR	F1LT49	N-Term(iTRAQ4plex); S3(Phospho)
iEDVGsDEEDDsGkDk	P34058	N-Term(iTRAQ4plex); S6(Phospho); S12(Pho
lkELFDYsPPLHk	B1WC16	N-Term(iTRAQ4plex); K2(iTRAQ4plex); S8(P
dLLHAVHPEsPSLk	A0A0G2JXT6	N-Term(iTRAQ4plex); S10(Phospho); K14(iT
wVEENIPSSFTDVALPVLY	D4A7B6	N-Term(iTRAQ4plex); S21(Phospho)
eTDPsPEER	A0A0G2K0F3	N-Term(iTRAQ4plex); S5(Phospho)
tPsPTSSk	P52481	N-Term(iTRAQ4plex); S3(Phospho); K8(iTRA
rGPGAAGPGAsGSGQGE	P22909	N-Term(iTRAQ4plex); S11(Phospho)
aAYFGIyDTAk	Q09073	N-Term(iTRAQ4plex); Y7(Phospho); K11(iTR
nEDAsFPSTPEPVkDSSR	F1LQN3	N-Term(iTRAQ4plex); S5(Phospho); K14(iTR
cLAFHDIsPQAPTHFLVIPk	D4A269	N-Term(iTRAQ4plex); C1(Carbamidomethyl);
fSDEGDSDSALDSVSPcP	G3V7H1	N-Term(iTRAQ4plex); C17(Carbamidomethyl
iLDRcsTILVQGk	P06686	N-Term(iTRAQ4plex); C5(Carbamidomethyl);
vLsSTsEEDEPGVVk	F1LWN1	N-Term(iTRAQ4plex); S3(Phospho); S6(Phos
eksPPDQSAVPNtPPSTPV	A0A0G2JSM7;D3ZZ99	N-Term(iTRAQ4plex); K2(iTRAQ4plex); S3(P
qEVTGSsAVSPIR	Q9Z1T4	N-Term(iTRAQ4plex); S7(Phospho)
aEEDEILNRsPR	P35565	N-Term(iTRAQ4plex); S10(Phospho)
gsLPPAALEPQTTVIHNPV	G3V9G3	N-Term(iTRAQ4plex); S2(Phospho); K23(iTR
lsLEsEGANEGAAAAPELS	D3ZQL7	N-Term(iTRAQ4plex); S2(Phospho); S5(Phos
sSRsSPELTR	A0A0G2K2M9	N-Term(iTRAQ4plex); S1(Phospho); S4(Phos
aRsYIETETESR	A0A0G2K6R9;F1M9N9	N-Term(iTRAQ4plex); S3(Phospho)
mkLDHELsLDR	F1M3W5	N-Term(iTRAQ4plex); K2(iTRAQ4plex); S8(P
isLNDEEEEkGTNPEGLSY	D4A831	N-Term(iTRAQ4plex); S2(Phospho); K10(iTR
sEADtLPR	D3ZEI4	N-Term(iTRAQ4plex); T5(Phospho)
sALFAQINQGEsITHALk	Q08163	N-Term(iTRAQ4plex); S12(Phospho); K18(iT
gYSYFTGSsPPLSPSTPSI	G3V984	N-Term(iTRAQ4plex); S9(Phospho); K27(iTR
sMsSAFcSLLAPER	P97680	N-Term(iTRAQ4plex); S3(Phospho); C7(Carb
tLEVVSPSQSVTGSAGHT	P15205;F1LRL9	N-Term(iTRAQ4plex); S23(Phospho); K28(iT
gSVsEDELIAAIk	Q64548	N-Term(iTRAQ4plex); S4(Phospho); K13(iTR
eDFsPEkk	P15205;F1LRL9	N-Term(iTRAQ4plex); S4(Phospho); K7(iTRA
dVsDERLsPAkSPSLSPSP	P15205;F1LRL9	N-Term(iTRAQ4plex); S3(Phospho); S8(Phos
vkEVHDELEDLPsPPPPLs	M0R423	N-Term(iTRAQ4plex); K2(iTRAQ4plex); S13(
asLSLAPVNIFk	Q5XIM9	N-Term(Acetyl); S2(Phospho); K12(iTRAQ4p
tWTFcGTPEYVAPEVILNk	Q64595	N-Term(iTRAQ4plex); T1(Phospho); C5(Carb
qLAELETEDGLREsP	Q8K3W5	N-Term(iTRAQ4plex); S14(Phospho)
tAsEGsEAETPEAPk	M0RC12	N-Term(iTRAQ4plex); S3(Phospho); S6(Phos
aELEEmEETHPsDEEGEE	P34926;Q63330	N-Term(iTRAQ4plex); M6(Oxidation); S12(Ph
aTLLNVPDLsDsIHSANAS	F1M4A0	N-Term(iTRAQ4plex); S10(Phospho); S12(Ph
sLDSDEsEDEDDDYQQk	Q62785	N-Term(iTRAQ4plex); S7(Phospho); K17(iTR
nkPSATDsEGEDDEDMTk	Q6MG08	N-Term(iTRAQ4plex); K2(iTRAQ4plex); S8(P
vIRPLDQPSsFDATPYIk	P97536	N-Term(iTRAQ4plex); S10(Phospho); K18(iT

vENMDIsDEEDGGALPSG	Q5XIW8	N-Term(iTRAQ4plex); S7(Phospho); K33(iTR
dDDDIDLFGsDDEEESED	B5DEN5	N-Term(iTRAQ4plex); S10(Phospho); K20(iT
eVVPDsPGDkGSTNR	P34926;Q63330	N-Term(iTRAQ4plex); S6(Phospho); K10(iTR
rEsVAsGDDRAEEDMDEA	P15205;F1LRL9	N-Term(iTRAQ4plex); S3(Phospho); S6(Phos
vDLsDEEAkETENLk	Q4V7D0	N-Term(iTRAQ4plex); S4(Phospho); K9(iTRA
tAPSAGMQPQPsLcSSAM	F1M3F8	N-Term(iTRAQ4plex); S12(Phospho); C14(Ca
aQIsSPNLR	D3ZMW5	N-Term(iTRAQ4plex); S4(Phospho)
gGEATWSGSEFEISFPDs	A0JN16	N-Term(iTRAQ4plex); S18(Phospho)
sEQPEEVLssEEETAGVE	Q63475	N-Term(iTRAQ4plex); S9(Phospho); S10(Pho
sPGDEDDkDckEEENk	Q923W4	S1(Phospho); N-Term(iTRAQ4plex); K8(iTRA
qVPQPSSAsLDEYTLMR	F1MAL5	N-Term(iTRAQ4plex); S9(Phospho)
dEILPTtPISEQk	A0A0G2K689	N-Term(iTRAQ4plex); T7(Phospho); K13(iTR
eGTQASEGYFSQsQEEE	A0A0H2UHL9	N-Term(iTRAQ4plex); S13(Phospho); C25(Ca
aPMPLLPPPPPPEPQSSE	A0A0G2JWK2	N-Term(iTRAQ4plex); S22(Phospho); C34(Ca
sTGGtPPDSSLVTYELHSI	Q6AYH5	N-Term(iTRAQ4plex); T5(Phospho); K24(iTR
gsPPLLDAPASPPQAPR	M0RDK4	N-Term(iTRAQ4plex); S2(Phospho)
sDISERPSVDDVESETGs	Q5U2P5;A0A0G2K2V4	N-Term(iTRAQ4plex); S18(Phospho)
fNFESVPEsPGEk	B1H266	N-Term(iTRAQ4plex); S9(Phospho); K13(iTR
gSGDTSNFDDYEEEEIRV	P68182	N-Term(iTRAQ4plex); S19(Phospho); K23(iT
aELVLIDEDDEksLR	D4A4D8	N-Term(iTRAQ4plex); K12(iTRAQ4plex); S13
rDGVEDGDsQGDGSSQP	P97924	N-Term(iTRAQ4plex); S9(Phospho)
sLSGSTNTVSsEGVTk	D3ZU84	N-Term(iTRAQ4plex); S11(Phospho); K16(iT
vkEVHDELEDLPsPPPPLs	M0R423	N-Term(iTRAQ4plex); K2(iTRAQ4plex); S13(
tATATGEQsPGAR	F7EYF1	N-Term(iTRAQ4plex); S9(Phospho)
eIkPSEkPVsPk	Q923I5	N-Term(iTRAQ4plex); K3(iTRAQ4plex); K7(iT
aFYGsEEDEAk	D4AE00	N-Term(iTRAQ4plex); S5(Phospho); K11(iTR
ekPDsDDDLDIESLVTAk	A0A0G2K101	N-Term(iTRAQ4plex); K2(iTRAQ4plex); S5(P
sIDkTEVcPWESHGQsPLI	A0A0A0MY13	N-Term(iTRAQ4plex); K4(iTRAQ4plex); C8(C
dTVDsAGTSPTAVLAAGE	A0A0G2K272	N-Term(iTRAQ4plex); S5(Phospho)
dFSGAGGIGVFSEsSSVA	A0A0G2K207	N-Term(iTRAQ4plex); S14(Phospho); K20(iT
stPTAEDVTAPLVEER	D4A1Q2	N-Term(iTRAQ4plex); T2(Phospho)
fRGsGDTSNFDDYEEEEII	P68182	N-Term(iTRAQ4plex); S4(Phospho)
aAEPHFGtLPk	D3ZHL1	N-Term(iTRAQ4plex); T8(Phospho); K11(iTR
tTNSsLEEIIDk	D4A4K4	N-Term(iTRAQ4plex); S5(Phospho); K12(iTR
rDsFIGTPYWMAPEVVMc	G3V7I8	N-Term(iTRAQ4plex); S3(Phospho); C18(Car
lQGLLQDLGEAAAQQPPF	F1LR76	N-Term(iTRAQ4plex); S20(Phospho)
rAsGQAFELILSPR	A0A096MK73	N-Term(iTRAQ4plex); S3(Phospho)
aIPtRIIYDFLmExsPVAk	Q7M0D5	N-Term(Acetyl); T4(Phospho); M12(Oxidation
vQEAQDsDsDTEGGATG	A0A0G2JWM2	N-Term(iTRAQ4plex); S7(Phospho); S9(Phos
sHQNsPTELNTDR	G3V7P7	N-Term(iTRAQ4plex); S5(Phospho)
rITsPLMEPSSIEk	P34064	N-Term(iTRAQ4plex); S4(Phospho); K14(iTR
aLENGEADEPsFSDPEDF	Q4G061	N-Term(iTRAQ4plex); S11(Phospho); S23(Ph
eLESGTEEsDEERSPNPR	D3ZGU6	N-Term(iTRAQ4plex); S9(Phospho)
iYEFPEtDDEEENkLVk	F1LMC7	N-Term(iTRAQ4plex); T7(Phospho); K14(iTR
qSLPYPQPGLEsPGIESP	D4A758	N-Term(iTRAQ4plex); S12(Phospho); K25(iT
gGsSAsLHNSLMR	O54701	N-Term(iTRAQ4plex); S3(Phospho); S6(Phos
hPWIsHR	P11275	N-Term(iTRAQ4plex); S5(Phospho)
tLEVVsPSQSVTGSAGHT	P15205;F1LRL9	N-Term(iTRAQ4plex); S6(Phospho); Y21(Pho
sHsSPSLNPDAsPVTAk	D4A404	N-Term(iTRAQ4plex); S3(Phospho); S12(Pho
mQGQsPPAPTR	F1M949	N-Term(iTRAQ4plex); S5(Phospho)
dQPDGTSLsPVQsPSQS	A0A140TA95	N-Term(iTRAQ4plex); S9(Phospho); S13(Pho
sPsQGDEDSALILTQDNLI	A0A0G2K1P1	N-Term(iTRAQ4plex); S3(Phospho); K19(iTR
iPESELGsPTLTSAQk	A0A0G2K9J0	N-Term(iTRAQ4plex); S8(Phospho); K16(iTR
lVEQANsPk	A0A0G2K1S0	N-Term(iTRAQ4plex); S7(Phospho); K9(iTRA
sDSNAsFLR	A0A0G2K6R9	N-Term(iTRAQ4plex); S6(Phospho)

Information Classification: General

kLETAVNLAWTAGNsNTF	Q9Z2L0	N-Term(iTRAQ4plex); K1(iTRAQ4plex); S15(
iGEHTPsSLAIMENANVLA	P05065	N-Term(iTRAQ4plex); S7(Phospho)
fGsPPQR	G3V793	N-Term(iTRAQ4plex); S3(Phospho)
lDEGtPPEPk	D3ZA84	N-Term(iTRAQ4plex); T5(Phospho); K10(iTR
gsPkPAVVEAVTTAGSQD	D3Z941	N-Term(iTRAQ4plex); S2(Phospho); K4(iTRA
tSDVNETQPPQsE	G3V9Y1	N-Term(iTRAQ4plex); S12(Phospho)
lSSPAAFLPAcNsPSk	D4A5B3	N-Term(iTRAQ4plex); C11(Carbamidomethyl
eGkPEDEVEPEDEEksDE	M0RBL8	N-Term(iTRAQ4plex); K3(iTRAQ4plex); K15(
kSADAGSQkDsDDSAATF	F1M0Z1	N-Term(iTRAQ4plex); K1(iTRAQ4plex); K9(iT
tGNAYLTAELStPDGSVAD	D3ZQG6	N-Term(iTRAQ4plex); T12(Phospho); K26(iT
sTETSDFENIEsPLNER	F1MAA1	N-Term(iTRAQ4plex); S12(Phospho)
tHPVVsPSSk	A0A0G2K6R9;F1M9N9	N-Term(iTRAQ4plex); S6(Phospho); K10(iTR
sPLPSHAR	P02688	N-Term(iTRAQ4plex); S1(Phospho)
qEAGQIsPSRPTSVPTAP	B5DFM1	N-Term(iTRAQ4plex); S7(Phospho); K27(iTR
eWYQSTIPQsPSPAPDD	A0A140TAB1	N-Term(iTRAQ4plex); S10(Phospho)
sADWTEELGAR	A0A0G2JZ27	N-Term(iTRAQ4plex); S1(Phospho)
aRsMDSSDLSDGAVTLQ	A0A0G2K527	N-Term(iTRAQ4plex); S3(Phospho); K23(iTR
cEEDHAALVNQESEQsD	D4A0I5	N-Term(iTRAQ4plex); C1(Carbamidomethyl);
sDAAPAAsDSkPSSAEPA	A0A0G2K1L8	N-Term(iTRAQ4plex); S8(Phospho); K11(iTR
qQAPLPAPPNDsDNDEE	G3V917	N-Term(iTRAQ4plex); S12(Phospho); K24(iT
ikESEtFsDsSPIEIIDEFPT	F1LQN3	N-Term(iTRAQ4plex); K2(iTRAQ4plex); T6(P
qNsATESADSIEIYVPEAQ	G3V927	N-Term(iTRAQ4plex); S3(Phospho)
aDAPDAGAQsDSELPSY	A0A0G2K3N1	N-Term(iTRAQ4plex); S10(Phospho)
ssPsESPLmEk	Q9JJP0	N-Term(iTRAQ4plex); S2(Phospho); S4(Phos
rPSGDVLETFNFLENADD	P58405	N-Term(iTRAQ4plex); S19(Phospho); K34(iT
sGQGDAPPtPPTPVDLDL	D4AB17	N-Term(iTRAQ4plex); T9(Phospho); K24(iTR
iGFPSTsPAk	A0A0G2JZF2	N-Term(iTRAQ4plex); S7(Phospho); K10(iTR
fDsNEEDTTSVFAPSFGL	Q14TE9	N-Term(iTRAQ4plex); S3(Phospho); K19(iTR
aSEEEEIGGPEDSEAVQ	Q62882	N-Term(iTRAQ4plex); T20(Phospho); K32(iT
sLPsLEDIDPDVLDSmHS	M9MMM8	N-Term(iTRAQ4plex); S4(Phospho); M16(Ox
vLSsIEQk	P61983;P68511;P68255;P	N-Term(iTRAQ4plex); S4(Phospho); K8(iTRA
sMsAPGNLLLk	F1LQD2	N-Term(iTRAQ4plex); S3(Phospho); K11(iTR
dksDsETEGLVFAR	A0A0G2K0F3	N-Term(iTRAQ4plex); K2(iTRAQ4plex); S3(P
qmxmsPPPGNAGPVIMS	O08729	N-Term(iTRAQ4plex); M2(Oxidation); X3(V);
gLEPGVFTLAEEDDEPEA	A0A0G2K5K1	N-Term(iTRAQ4plex); S22(Phospho)
lSsSGLR	G3V7V0	N-Term(iTRAQ4plex); S3(Phospho)
rGGGsGGGDESEGEEVD	A0A0G2JUX5	N-Term(iTRAQ4plex); S5(Phospho)
nPPPQDYEsDDESYEVLD	Q5BJS4	N-Term(iTRAQ4plex); S9(Phospho)
kLEQEQEGEkDAATEEP	F8WFS9	K1(iTRAQ4plex); N-Term(iTRAQ4plex); K10(
qGsFSEDVISHk	D3ZHV2	N-Term(iTRAQ4plex); S3(Phospho); K12(iTR
eEIkPPAEVksPEk	F1LRZ7	N-Term(iTRAQ4plex); K4(iTRAQ4plex); K10(
vSGAGLsPSR	D3ZF26	N-Term(iTRAQ4plex); S7(Phospho)
aVVVsPkEENk	A1L1L5	N-Term(iTRAQ4plex); S5(Phospho); K7(iTRA
iADFGFSNLFTPGQLLkTV	M0RD40	N-Term(iTRAQ4plex); K17(iTRAQ4plex); C2
gFGFGQGAGALVHsE	P47875	N-Term(iTRAQ4plex); S14(Phospho)
sEtAPAAPAAAPPAEk	A0A0G2K654	N-Term(Acetyl); T3(Phospho); K16(iTRAQ4p
eTSLLAVPGALsPLAIPNA	Q66H20	N-Term(iTRAQ4plex); S12(Phospho)
hIkEEPLsEEEPcTSTAVP	Q9QZ86	N-Term(iTRAQ4plex); K3(iTRAQ4plex); S8(P
sSQALLSQLYQsPNR	O09032	N-Term(iTRAQ4plex); S12(Phospho)
tVFAGAVPVLPAsPPPk	A0A0G2QC53	N-Term(iTRAQ4plex); S13(Phospho); K17(iT
qQAAYYAQtSPQGMPQH	Q32PX7	N-Term(iTRAQ4plex); T9(Phospho)
sRtLsQsSESGTLPSGPP	A0A0G2JSU4	N-Term(iTRAQ4plex); T3(Phospho); S5(Phos
rQPsPQPSPR	A0A0G2K2M9	N-Term(iTRAQ4plex); S4(Phospho)
yIEkGsPLNSPLDR	D3ZH36	N-Term(iTRAQ4plex); K4(iTRAQ4plex); S6(P
eVVAGsPELGQDFEHVS	A0A0G2K677	N-Term(iTRAQ4plex); S6(Phospho); K22(iTR

sAGPQSQLLAsVIAEksRs	F8WFS9	N-Term(iTRAQ4plex); S11(Phospho); K16(iT
tDSREDEIsPPPPNPVVk	P09456	N-Term(iTRAQ4plex); S9(Phospho); K18(iTR
dVSDERLsPAk	P15205;F1LRL9	N-Term(iTRAQ4plex); S8(Phospho); K11(iTR
tGTAEMSsILEER	P15999	N-Term(iTRAQ4plex); S8(Phospho)
iDIsPSALR	B1WC16	N-Term(iTRAQ4plex); S4(Phospho)
iSsPPAGPPADLEDEEGLI	D3ZQJ3	N-Term(iTRAQ4plex); S3(Phospho); K19(iTR
rRtLsGPVTGLLATAR	D3ZE17	N-Term(iTRAQ4plex); T3(Phospho); S5(Phos
isSksPGHMVILNQTk	A0A0G2K904	N-Term(iTRAQ4plex); S2(Phospho); K4(iTRA
rSsFLNAk	F1M386	N-Term(iTRAQ4plex); S3(Phospho); K8(iTRA
gSsEESVDEDR	A0A0G2K6R9;F1M9N9	N-Term(iTRAQ4plex); S3(Phospho)
vPLAAVAGSEGPEQLQPI	B3DMA1	N-Term(iTRAQ4plex); C19(Carbamidomethyl
qLHIEGAsLELsDDDTESk	G3V9Y1	N-Term(iTRAQ4plex); S8(Phospho); S12(Pho
gPQYGsLER	Q9Z0W5	N-Term(iTRAQ4plex); S6(Phospho)
vESVFETLVEDsPEEESTI	Q6P136	N-Term(iTRAQ4plex); S12(Phospho); K21(iT
mEALEVDDIsPALEVTEDI	D4A959	N-Term(Acetyl); S10(Phospho); K26(iTRAQ4
aFYGsEEDEAkGPGsEEA	D4AE00	N-Term(iTRAQ4plex); S5(Phospho); K11(iTR
lTGQESGLGDsPPFEk	G3V8M8	N-Term(iTRAQ4plex); S11(Phospho); K16(iT
nVTESPsFsAGDNPHVLY	G3V9B3	N-Term(iTRAQ4plex); S7(Phospho); S9(Phos
tSsVVTLEVAk	F1LT10	N-Term(iTRAQ4plex); S3(Phospho); K11(iTR
tLNEWSSQIsPDLVR	Q2VC85	N-Term(iTRAQ4plex); S10(Phospho)
fPPGLEAAEQSAEGLGsG	P34926	N-Term(iTRAQ4plex); S17(Phospho); K19(iT
lALQYVPEPsPGk	B5DFB1	N-Term(iTRAQ4plex); S10(Phospho); K13(iT
sLDSLDPAGLLtSPk	G3V849	N-Term(iTRAQ4plex); S1(Phospho); T12(Pho
aNLsPDR	Q6TQE1	N-Term(iTRAQ4plex); S4(Phospho)
kIVEPEVVGEsDSEVEGD	D4ACM9	N-Term(iTRAQ4plex); K1(iTRAQ4plex); S11(
eEHGGLIRsPR	F2Z3T9	N-Term(iTRAQ4plex); S9(Phospho)
eVHDELEDLPsPPPPLsPI	M0R423	N-Term(iTRAQ4plex); S11(Phospho); S17(Ph
gQLDLsQEEP	Q5XIC4	N-Term(iTRAQ4plex); S6(Phospho)
vLQSPVsSEDHAILQAVIT	F1MAB7	N-Term(iTRAQ4plex); S7(Phospho); K24(iTR
sPVEVksPASVk	F1LRZ7	N-Term(iTRAQ4plex); S1(Phospho); K6(iTRA
gDsETDLEALFNAVMNPk	R9PXS9	N-Term(iTRAQ4plex); S3(Phospho); K18(iTR
aDALQAGAsQFETSAAk	Q19LA7	N-Term(iTRAQ4plex); S9(Phospho); K17(iTR
mESLGsPR	F1M842	N-Term(iTRAQ4plex); S6(Phospho)
sGTSSPQsPVFR	G3V6Z3	N-Term(iTRAQ4plex); S8(Phospho)
kQDsDEMPFGcIk	D3ZSP7	N-Term(iTRAQ4plex); K1(iTRAQ4plex); S4(P
iYEFPEtDDEEENk	F1LMC7	N-Term(iTRAQ4plex); T7(Phospho); K14(iTR
sSScGDAELLGQATLPVG	Q5U2P5;A0A0G2K2V4	S1(Phospho); N-Term(iTRAQ4plex); C4(Carb
gsEENLDETR	A0A0G2JSM7;D3ZZ99	N-Term(iTRAQ4plex); S2(Phospho)
gAQADHFTQTPLsPGSQV	Q5XI97	N-Term(iTRAQ4plex); S13(Phospho)
aEEPsEEEADAPkPk	M0R965	N-Term(iTRAQ4plex); S5(Phospho); K13(iTR
rSDSYQEDIYPMTPGTEP	F1LMV9	N-Term(iTRAQ4plex); T21(Phospho)
tGGVLSLIQctLIEEPDTSD	Q99JE4	N-Term(iTRAQ4plex); C10(Carbamidomethyl
eALGFMRNQHFEVPFIAR	A0A0G2K0J0	N-Term(iTRAQ4plex); Y19(Phospho)
gEPNVSylcSR	A0A0G2JSH4;A0A0G2K7V	N-Term(iTRAQ4plex); Y7(Phospho); C9(Carb
dYNASASTIsPPSSMEEDI	P15205;F1LRL9	N-Term(iTRAQ4plex); S10(Phospho); K19(iT
skDAsPINR	Q5RKH1	N-Term(iTRAQ4plex); K2(iTRAQ4plex); S5(P
aAQQTkGsYMEVEDNR	A0A0H2UHZ1	N-Term(iTRAQ4plex); K6(iTRAQ4plex); S8(P
hGGGIVADLSQQsLk	Q5HZV9	N-Term(iTRAQ4plex); S13(Phospho); K15(iT
dVmsDETNNEETEsPSQE	F1LRL9	N-Term(iTRAQ4plex); M3(Oxidation); S4(Pho
ysVLQQHAEADGVEALDT	O54861	N-Term(iTRAQ4plex); S2(Phospho); K23(iTR
dGDDEDGsDEDVVHNED	D4A054	N-Term(iTRAQ4plex); S8(Phospho); K32(iTR
mQFsFEEYEk	A0A0G2JZX5	N-Term(iTRAQ4plex); M1(Oxidation); S4(Pho
amSMASILTNTMEELEEs	A0A0G2K207	N-Term(iTRAQ4plex); M2(Oxidation); S18(Ph
aAHLPLLTIEPPsDSSVDL	A0A0G2JZX5	N-Term(iTRAQ4plex); S13(Phospho)
gPDRtPPSEEDSAEAER	O70593	N-Term(iTRAQ4plex); T5(Phospho)

Information Classification: General

sAsASPLTPcSAPR	D4A644	N-Term(iTRAQ4plex); S3(Phospho); C10(Ca
gsPTGSSPNNASELSLAS	Q4KLM7	N-Term(iTRAQ4plex); S2(Phospho); K22(iTR
dkEVsDDEAEEkEEk	P82995	N-Term(iTRAQ4plex); K2(iTRAQ4plex); S5(P
lLGLNELDDLLGkEsPSkD	F1M2K6	N-Term(iTRAQ4plex); K13(iTRAQ4plex); S15
sSESAHVATLPAR	F1MA36	N-Term(iTRAQ4plex); S1(Phospho)
qEQDYWsEsDkEEADTPS	Q9Z1T4	N-Term(iTRAQ4plex); S7(Phospho); S9(Phos
sQGGGESISPtPPEGLAP	A0A0G2JTR4	N-Term(iTRAQ4plex); T11(Phospho); K24(iT
tsPQLSENLGTSDLDLHA	F1LXQ7	N-Term(iTRAQ4plex); S2(Phospho); S19(Pho
tDsREDEIsPPPPNPVVk	P09456	N-Term(iTRAQ4plex); S3(Phospho); S9(Phos
kEEsEEsDDDmGFGLFD	P02401	K1(iTRAQ4plex); N-Term(iTRAQ4plex); S4(P
gLPsNNSLDFLDEEYEVP	A0A0G2K876	N-Term(iTRAQ4plex); S4(Phospho)
dMPGGFLFDGLsDDEDD	Q6AY24	N-Term(iTRAQ4plex); S12(Phospho)
sTLSMsPR	P19103	N-Term(iTRAQ4plex); S6(Phospho)
sSISPMDEPVPDSEsPIEk	P15205;F1LRL9	N-Term(iTRAQ4plex); S15(Phospho); K19(iT
eTSVSkEDTDQEEkAsNE	Q566D6	N-Term(iTRAQ4plex); K6(iTRAQ4plex); K14(
tGSEPsPPQGGGSLGPSA	D3ZE17	N-Term(iTRAQ4plex); S6(Phospho)
gDsPELkPR	Q6P730	N-Term(iTRAQ4plex); S3(Phospho); K7(iTRA
aSVSGPNsPSETR	P82458	N-Term(iTRAQ4plex); S8(Phospho)
dIISLAPGsPNPk	A0A0G2K4P6	N-Term(iTRAQ4plex); S9(Phospho); K13(iTR
tEATQGLDYVPSAGTISP	P34926	N-Term(iTRAQ4plex); S20(Phospho); S21(Ph
gSYMEVEDNRsQVETED	A0A0H2UHZ1	N-Term(iTRAQ4plex); S11(Phospho); K21(iT
aAVLSDsEDEDkASAAk	Q3SWT4	N-Term(iTRAQ4plex); S7(Phospho); K12(iTR
sPPSGSQSLLEGIMPASG	D3ZF26	N-Term(iTRAQ4plex); S1(Phospho); K20(iTR
tESLPSAQQYVPFGGk	D3ZWV8	N-Term(iTRAQ4plex); T1(Phospho); K16(iTR
sLPITIEMLk	M0R7T1	N-Term(iTRAQ4plex); S1(Phospho); K10(iTR
aVNSTREtPPk	A0A096MKG6	N-Term(iTRAQ4plex); T8(Phospho); K11(iTR
wLAEsPVGLPPEEEDk	P34926	N-Term(iTRAQ4plex); S5(Phospho); K16(iTR
lEkPLTIGTFsLNSR	D3ZGN2	N-Term(iTRAQ4plex); K3(iTRAQ4plex); S11(
vVTEksPTDWALFTYEGN	Q9JHL4	N-Term(iTRAQ4plex); K5(iTRAQ4plex); S6(P
hPEPVPEEGsEDELPPQV	Q5FWU3	N-Term(iTRAQ4plex); S10(Phospho); K20(iT
qEIPEDFLDkPTLLsPEEL	Q5XIK5	N-Term(iTRAQ4plex); K10(iTRAQ4plex); S15
vIISAPsTDVPmFVmGVN	F1M269	N-Term(iTRAQ4plex); S7(Phospho); M12(Ox
eHFcEYTEDELNEILNGm	A0A0G2K6R9;F1M9N9;A0	N-Term(iTRAQ4plex); C4(Carbamidomethyl);
wRPSIGVQVETISDsDTE	G3V7T8	N-Term(iTRAQ4plex); S15(Phospho)
sFSLASSGNsPISQR	B5DF74	N-Term(iTRAQ4plex); S10(Phospho)
gILAADEStGsIAk	P05065	N-Term(iTRAQ4plex); T9(Phospho); S11(Pho
aNsVTMDGQGLQITPIPG	P49621	N-Term(iTRAQ4plex); S3(Phospho); K29(iTR
gILAADESTGsIAkR	P05065	N-Term(iTRAQ4plex); S11(Phospho); K14(iT
fDQLFDDEsDPFEVLk	Q6AXS5	N-Term(iTRAQ4plex); S9(Phospho); K16(iTR
gGIDNPAItSDQEVDDk	Q6TUE6	N-Term(iTRAQ4plex); T9(Phospho); K17(iTR
tLGSQSFNTSDSTMLTPP	D3ZGN7	N-Term(iTRAQ4plex); S19(Phospho); S20(Ph
dQLRDGsPPPAFkPEPPk	Q3ZB99	N-Term(iTRAQ4plex); S7(Phospho); K13(iTR
eDTDQEEkAsNEDVTk	Q566D6	N-Term(iTRAQ4plex); K8(iTRAQ4plex); S10(
gAIYSIPITEDGGGGsSTP	G3V8G4	N-Term(iTRAQ4plex); S16(Phospho)
aALLkAsPk	D3ZHE6	N-Term(iTRAQ4plex); K5(iTRAQ4plex); S7(P
vQDkPEsPSGSTQIQR	Q63358	N-Term(iTRAQ4plex); K4(iTRAQ4plex); S7(P
eMSASNFEssPEIEESPA	A0A0G2K2M9	N-Term(iTRAQ4plex); S9(Phospho); S10(Pho
fHAPALGPEPAAQTPEGs	D4A205	N-Term(iTRAQ4plex); S18(Phospho); K27(iT
kEANELsDsAGEDDPAEL	D4A2C4	N-Term(iTRAQ4plex); K1(iTRAQ4plex); S7(P
sGGNSSSsLGDIVPSSR	A0A0G2JSZ0	N-Term(iTRAQ4plex); S8(Phospho)
sDkENDQEHDEsDNEVL	A0A0G2JXN8	N-Term(iTRAQ4plex); K3(iTRAQ4plex); S12(
rVPSPGSsEDDLQEEEQL	G3V7X2	N-Term(iTRAQ4plex); S8(Phospho); K23(iTR
fTQSYDYLtDEEER	B0BMV0	N-Term(iTRAQ4plex); T9(Phospho)
tYAEMEQtITLDHSEGFSV	A0A0G2K6R9;F1M9N9;A0	N-Term(iTRAQ4plex); T8(Phospho); C25(Ca
rPLPVEsPDTQR	D4A4V3	N-Term(iTRAQ4plex); S7(Phospho)

fRYsGR	A0A0G2K0F3;G3V874;A0	N-Term(iTRAQ4plex); S4(Phospho)
kYsNSDNTETk	A0A0A0MY13	N-Term(iTRAQ4plex); K1(iTRAQ4plex); S3(P
sTSAPQMsPGSSDNQSS	A0A0G2JYC6	N-Term(iTRAQ4plex); S8(Phospho); K25(iTR
tAEEkEPAAPAsPAPsPVP	D4A644	N-Term(iTRAQ4plex); K5(iTRAQ4plex); S12(
lAEmYGGGEsDkDA	Q8R5H2	N-Term(iTRAQ4plex); M4(Oxidation); S10(Ph
lLDFGsLSNLQVTQPTVGı	A0A0H2UHQ8	N-Term(iTRAQ4plex); S6(Phospho); M19(Ox
gDQALsFLkDFLAGGIAAA	Q05962	N-Term(Acetyl); S6(Phospho); K9(iTRAQ4ple
sPPDQSAVPNTPPstPVk	A0A0G2JSM7;D3ZZ99	N-Term(iTRAQ4plex); S14(Phospho); T15(Ph
mVGGGLQSAQASVPTEE	P20417	N-Term(iTRAQ4plex); S20(Phospho); K27(iT
rQsTDLPTGWEEAYTFEG	E9PTG5	N-Term(iTRAQ4plex); S3(Phospho)
qDRQsLGEsPR	A0A0G2JW01;F1LWK7	N-Term(iTRAQ4plex); S5(Phospho); S9(Phos
gQSQTWPDtSSPEVR	A0A0G2K2M9	N-Term(iTRAQ4plex); T9(Phospho)
lFVLLPEQsPGSYSk	F1MAA1	N-Term(iTRAQ4plex); S9(Phospho); K15(iTR
sTPAsPVQSPTR	F8WFS9	N-Term(iTRAQ4plex); S5(Phospho)
iTsPLMEPSSIEk	P34064	N-Term(iTRAQ4plex); S3(Phospho); K13(iTR
aEsGDSLSSEDR	M0RDG0	N-Term(iTRAQ4plex); S3(Phospho)
sFsMQDLTTIR	D4A193	N-Term(iTRAQ4plex); S3(Phospho)
vPFAPGPsPPPLLGNmDQ	D3ZQJ3	N-Term(iTRAQ4plex); S8(Phospho); M16(Ox
tPSISPVRVsPNNR	F1M787	N-Term(iTRAQ4plex); S10(Phospho)
tESGYGsESSLR	D4ACN6	N-Term(iTRAQ4plex); S7(Phospho)
qPAsPDGRAsPVNEDVR	Q62901	N-Term(iTRAQ4plex); S4(Phospho); S10(Pho
ysLEGGR	A0A0G2K6R9;F1M9N9;A0	N-Term(iTRAQ4plex); S2(Phospho)
sAsPERMD	D4A720	N-Term(iTRAQ4plex); S3(Phospho)
gGFFsPWk	A0A0G2JXR9	N-Term(iTRAQ4plex); S5(Phospho); K8(iTRA
aSsLGEIDDSSELR	A0A0G2K6J2	N-Term(iTRAQ4plex); S3(Phospho)
sPATVksPGEAk	F1LRZ7	N-Term(iTRAQ4plex); K6(iTRAQ4plex); S7(P
dGQDAIAQsPEkEAk	A0A0G2K904	N-Term(iTRAQ4plex); S9(Phospho); K12(iTR
dNLTQDLGtLR	P47819	N-Term(iTRAQ4plex); T9(Phospho)
mAGPPAPPGGsPR	A0A0G2JXK1	N-Term(iTRAQ4plex); M1(Oxidation); S11(Ph
sLEETLHNVDLssDDELPQ	Q66H98	N-Term(iTRAQ4plex); S12(Phospho); S13(Ph
lGEQGPEPGPTPPQtPTP	D4AAV2	N-Term(iTRAQ4plex); T15(Phospho); S20(Ph
vAVVRtPPksPSASk	D4A1Q2	N-Term(iTRAQ4plex); T6(Phospho); K9(iTRA
dGkEDETstDVDEkPk	P53987	N-Term(iTRAQ4plex); K3(iTRAQ4plex); S8(P
nISLssEEEAEGLAGHPR	D3ZKQ4	N-Term(iTRAQ4plex); S5(Phospho); S6(Phos
hNsLEDAEQGk	D3ZXL9	N-Term(iTRAQ4plex); S3(Phospho); K11(iTR
dSsTcPGDYVLSVSENSR	Q5U2U2	N-Term(iTRAQ4plex); S3(Phospho); C5(Carb
eDENDAsDDEDDDEkRR	D4A8C8	N-Term(iTRAQ4plex); S7(Phospho); K15(iTR
ekSPPDQsAVPNtPPsTPV	A0A0G2JSM7	N-Term(iTRAQ4plex); K2(iTRAQ4plex); S8(P
eWSLESSPAQNWtPPQP	Q3S4A5	N-Term(iTRAQ4plex); T13(Phospho); K18(iT
kELsEEsEDEELQLEEFPN	Q7TQ19	N-Term(iTRAQ4plex); K1(iTRAQ4plex); S4(P
eEAksPAEAksPAEAk	F1LRZ7	N-Term(iTRAQ4plex); K4(iTRAQ4plex); S5(P
wDsDNDEENEDPPISMk	D4AA13	N-Term(iTRAQ4plex); S3(Phospho); K17(iTR
tYEtPPPsPGLDPTFSNQP	O88953	N-Term(iTRAQ4plex); T4(Phospho); S8(Phos
rPADIRsPEQVk	F1LRZ7	N-Term(iTRAQ4plex); S7(Phospho); K12(iTR
dGWNQNHFITPVsTLER	F1M2K6	N-Term(iTRAQ4plex); S13(Phospho)
eGPGGIVQGPPmLEAsPP	B1WC33	N-Term(iTRAQ4plex); M12(Oxidation); S16(P
vIsQTNLITTVTPEk	G3V874;A0A0G2K1Q9	N-Term(iTRAQ4plex); S3(Phospho); K15(iTR
dLWPMVsPEDTQSLSFSE	P34926;Q63330	N-Term(iTRAQ4plex); S7(Phospho); S22(Pho
tPPksPSASk	D4A1Q2	N-Term(iTRAQ4plex); T1(Phospho); K4(iTRA
lGIAVIHGEAQDAESDLVD	Q6AZ40	N-Term(iTRAQ4plex); S23(Phospho)
gAPAPsPPTsPPPPLPPDI	F1LPA3	N-Term(iTRAQ4plex); S6(Phospho); S10(Pho
gEsIEPLDPSEk	G3V6N1	N-Term(iTRAQ4plex); S3(Phospho); K12(iTR
ssNVVPYDFNR	Q6LDZ3	N-Term(iTRAQ4plex); S2(Phospho)
tAFDDAIAELDtLNEDSYkD	P61983	N-Term(iTRAQ4plex); T12(Phospho); K19(iT
lAsPSGSTSSGLEVVAPEY	O08629	N-Term(iTRAQ4plex); S3(Phospho); C37(Ca

Information Classification: General

aSPEEPGDsPPLR	A2RRU4	N-Term(iTRAQ4plex); S9(Phospho)
sAsLATk	F1M362	N-Term(iTRAQ4plex); S3(Phospho); K7(iTRA
lEREDssEEEEEEIDDEEIE	D4ACM9	N-Term(iTRAQ4plex); S6(Phospho); S7(Phos
eAALILGVsPTANk	M0R6L8	N-Term(iTRAQ4plex); S9(Phospho); K14(iTR
tAsPWk	P16086	N-Term(iTRAQ4plex); S3(Phospho); K6(iTRA
msGFIYQGk	Q923I5	N-Term(iTRAQ4plex); M1(Oxidation); S2(Pho
gDLsDVEEEEEEEMDVDE	D3ZBP2	N-Term(iTRAQ4plex); S4(Phospho); K24(iTR
vkEDsEAESTESVPLVck	A0A0A0MY13	N-Term(iTRAQ4plex); K2(iTRAQ4plex); S5(P
gDVTAEEAAGAsPAkANG	A0A0G2K613	N-Term(iTRAQ4plex); S12(Phospho); K15(iT
gAGQQQsQEMMEVDR	Q5HZV9	N-Term(iTRAQ4plex); S7(Phospho)
sDASsDQGPVSPEk	D3ZMR2	N-Term(iTRAQ4plex); S5(Phospho); K14(iTR
gSEsPNSFLDQEYR	Q9Z1T4	N-Term(iTRAQ4plex); S4(Phospho)
eAVsAASEDDIFR	E9PT56	N-Term(iTRAQ4plex); S4(Phospho)
nDQEPPPEALDFsDDEkE	A0A0G2K5K8	N-Term(iTRAQ4plex); S13(Phospho); K17(iT
sAsQSsLDkLDQELk	E9PSJ4	N-Term(iTRAQ4plex); S3(Phospho); S6(Phos
eSTESSNTtIEDEDVk	P15791	N-Term(iTRAQ4plex); T9(Phospho); K16(iTR
tTPPTQkPPsPPmSGk	F1LYA6	N-Term(iTRAQ4plex); K7(iTRAQ4plex); S10(
skPVFSESLsD	F1LP21	N-Term(iTRAQ4plex); K2(iTRAQ4plex); S10(
nSAGGTSVGWDsPPASP	B5DFL0	N-Term(iTRAQ4plex); S12(Phospho)
sSQsLPSDSSPQLQQEQ	D4ABN3	N-Term(iTRAQ4plex); S4(Phospho)
vQSLEGEkLsPksDISPLTF	P15205;F1LRL9	N-Term(iTRAQ4plex); K8(iTRAQ4plex); S10(
eAGVVHsDAEkDQEEEEQ	D3ZAY8	N-Term(iTRAQ4plex); S7(Phospho); K11(iTR
aPAPAAPAAEPQAEAPVA	Q05175;A0A0G2K1L8	N-Term(iTRAQ4plex); S20(Phospho); K27(iT
aILVDLEPGTMDsVR	P85108;P69897;Q4QRB4	N-Term(iTRAQ4plex); S13(Phospho)
eESEEsDEDMGFGLFD	P19945;D4A4D5	N-Term(iTRAQ4plex); S6(Phospho)
vAELEEEQsQGSSSYSDW	P34926;Q63330	N-Term(iTRAQ4plex); S9(Phospho); K20(iTR
gQVLAAAyELQR	F1MA36	N-Term(iTRAQ4plex); Y8(Phospho)
dQTLsPTIISGLHSIAR	A0A0G2K0X9	N-Term(iTRAQ4plex); S5(Phospho)
aGGANYDAQsE	B0BNA5	N-Term(iTRAQ4plex); S10(Phospho)
nADAVNQMAVcPGSAAD	O55164	N-Term(iTRAQ4plex); C11(Carbamidomethyl
vIRsADVsPTTEGVk	D3ZQG6	N-Term(iTRAQ4plex); S4(Phospho); S8(Phos
aMGDEDsGDsDTSPkPSP	G3V849	N-Term(iTRAQ4plex); S7(Phospho); S10(Pho
sIQTsPTEEDR	A0A0G2JZ27	N-Term(iTRAQ4plex); S5(Phospho)
eEAksPEkEETR	F1LRZ7	N-Term(iTRAQ4plex); K4(iTRAQ4plex); S5(P
gktSPAEEk	A0A0G2K6R9;F1M9N9	N-Term(iTRAQ4plex); K2(iTRAQ4plex); T3(P
lALSDsPDGRPGGFWR	P25122	N-Term(iTRAQ4plex); S6(Phospho)
lGsPAGsPVLGISGR	D3ZEY4	N-Term(iTRAQ4plex); S3(Phospho); S7(Phos
eHLGQGSsQEmEk	G3V7X2	N-Term(iTRAQ4plex); S8(Phospho); M11(Ox
aLsPEGALR	D4A198	N-Term(iTRAQ4plex); S3(Phospho)
sMTDLLSAEDIkk	P02625	N-Term(Acetyl); S1(Phospho); K12(iTRAQ4p
aGEASAEsTGAADGAPQE	Q05175	N-Term(iTRAQ4plex); S8(Phospho); K23(iTR
kEEsEESDDDmGFGLFD	P02401	K1(iTRAQ4plex); N-Term(iTRAQ4plex); S4(P
nEVNTMLGQsTEELR	P02650	N-Term(iTRAQ4plex); S10(Phospho)
tsPPVSVR	B5DF41	N-Term(iTRAQ4plex); S2(Phospho)
aQLEPVAsPAk	A0A0G2K1J5	N-Term(iTRAQ4plex); S8(Phospho); K11(iTR
vAGGSGsESPLLEGR	B2GV22	N-Term(iTRAQ4plex); S7(Phospho)
sPVkEEIkPPAEVksPEk	F1LRZ7	N-Term(iTRAQ4plex); S1(Phospho); K4(iTRA
ePGSSHLGsPDTAR	Q5BKB9	N-Term(iTRAQ4plex); S9(Phospho)
sAEALGPGALVsPR	F1M2D4	N-Term(iTRAQ4plex); S1(Phospho); S12(Pho
eYGStSSIDVQGVPEQSF	F1LYG2	N-Term(iTRAQ4plex); T5(Phospho)
kVPVPEGLDLDAWINEPF	B5DFK6	N-Term(iTRAQ4plex); K1(iTRAQ4plex); S19(
mDDsEVESTASILASVk	D3ZZZ9	N-Term(Acetyl); S4(Phospho); K17(iTRAQ4p
fLPIsSTPQPER	D3ZEX7	N-Term(iTRAQ4plex); S5(Phospho)
eQENMDDANsEQQFR	C0IXW5	N-Term(iTRAQ4plex); S10(Phospho)
kPTSDILkEESDDDQSDV	P28818	K1(iTRAQ4plex); N-Term(iTRAQ4plex); K8(iT

iGPDcEETLPDLPsPDADH	Q4V8C2	N-Term(iTRAQ4plex); C5(Carbamidomethyl);
tPPkSPSASk	D4A1Q2	N-Term(iTRAQ4plex); T1(Phospho); K4(iTRA
aGMPGSSGLGsPLGR	F1LVK0	N-Term(iTRAQ4plex); S11(Phospho)
mDLPGsPSR	D3ZAU7	N-Term(iTRAQ4plex); S6(Phospho)
gDMsDEDDENEFFDAPEI	D4A9D8	N-Term(iTRAQ4plex); S4(Phospho); K28(iTR
dGELPVEDDIDLsDVELDI	Q63081	N-Term(iTRAQ4plex); S13(Phospho); K22(iT
iYHLPDAEsDEDEDFk	Q91Y81	N-Term(iTRAQ4plex); S9(Phospho); K16(iTR
fYYSsGSSsPTHAk	Q6IRG7	N-Term(iTRAQ4plex); S5(Phospho); S9(Phos
lDSsPVLsPGNk	A0A0G2K382	N-Term(iTRAQ4plex); S4(Phospho); S8(Phos
tVsDNSLSSSk	Q8R511	N-Term(iTRAQ4plex); S3(Phospho); K11(iTR
fGDVEADsPVEQTIIQDHS	F1M3W5	N-Term(iTRAQ4plex); S8(Phospho); K22(iTR
nIDQSEFEGFsFVNsEFLk	F1LS42	N-Term(iTRAQ4plex); S11(Phospho); S15(Ph
iDYIAGLDsR	P36972	N-Term(iTRAQ4plex); S9(Phospho)
sAEDLTEGSYDAILSAEQI	B1WBW4	N-Term(iTRAQ4plex); S1(Phospho); K21(iTR
vsPVNDVDVGVYQLMQSI	D4A7H9	N-Term(iTRAQ4plex); S2(Phospho); K24(iTR
lSQSSQDsSPVR	A7BJV7	N-Term(iTRAQ4plex); S8(Phospho)
sSSSsQESLNRPFSSk	A0A0G2JZM8	N-Term(iTRAQ4plex); S5(Phospho); K16(iTR
gPPEGSETMPYIDEsPTm	A0A0G2JTR4	N-Term(iTRAQ4plex); S15(Phospho); M18(O
sTPVRsPGGSTMmk	Q920Q0	N-Term(iTRAQ4plex); S6(Phospho); M13(Ox
sMGsQEDDSGNkPSSYS	Q68FW9	N-Term(iTRAQ4plex); S4(Phospho); K12(iTR
gSPTGssPNNASELSLASI	Q4KLM7	N-Term(iTRAQ4plex); S6(Phospho); S7(Phos
sDSLDNEEkPPAPPLR	Q62829	N-Term(Acetyl); S1(Phospho); K9(iTRAQ4ple
sEDDSAkFDsNEEDTTSV	Q14TE9	N-Term(iTRAQ4plex); K7(iTRAQ4plex); S10(
aALPAGEGEsPEGAk	A0A0G2K5Z4	N-Term(iTRAQ4plex); S10(Phospho); K15(iT
nHsLEEEFER	F1LMT5	N-Term(iTRAQ4plex); S3(Phospho)
sLAALDALNtDDENDEEEY	D4ACM9	N-Term(iTRAQ4plex); T10(Phospho); K23(iT
rPsPDEPMTNLELk	D4A4T9	N-Term(iTRAQ4plex); S3(Phospho); K14(iTR
gEHsPDDNIIFFk	D3ZBT4	N-Term(Acetyl); S4(Phospho); K13(iTRAQ4p
vIENTDGsEEEMDAR	E9PTU4	N-Term(iTRAQ4plex); S8(Phospho)
dSGSSSVFAEsPGGk	Q9QXY2	N-Term(iTRAQ4plex); S11(Phospho); K15(iT
tENSTSTPAAkPk	P43278	N-Term(Acetyl); T1(Phospho); K11(iTRAQ4p
qTLPPFQPQITDDYGLDN	P09217	N-Term(iTRAQ4plex); T31(Phospho); K39(iT
sLPELDRDksDsETEGLVF	A0A0G2K0F3	N-Term(iTRAQ4plex); K9(iTRAQ4plex); S10(
dGHsPEGSTATYR	Q642G4	N-Term(iTRAQ4plex); S4(Phospho)
qkGsEENLDETR	A0A0G2JSM7;D3ZZ99	N-Term(iTRAQ4plex); K2(iTRAQ4plex); S4(P
sPGsPQLSR	A0A096MIT7	N-Term(iTRAQ4plex); S4(Phospho)
vADGQIsTEVSEAPVASDI	P23978	N-Term(iTRAQ4plex); S7(Phospho); K19(iTR
lLGSGsPSSSAR	Q496Z5	N-Term(iTRAQ4plex); S6(Phospho)
nLHWGEksPSYGVPSAPI	Q5EB62	N-Term(iTRAQ4plex); K7(iTRAQ4plex); S8(P
rRPPsPEPSAk	G3V6S0	N-Term(iTRAQ4plex); S5(Phospho); K11(iTR
fDGEsDkEQFDDDQk	A0A0G2K7K9	N-Term(iTRAQ4plex); S5(Phospho); K7(iTRA
iVSQNVGDVFPGIQDSGQ	D4A1U1	N-Term(iTRAQ4plex); T20(Phospho)
sQPPsPGPVAsPAPPDLD	D4AD05	N-Term(iTRAQ4plex); S5(Phospho); S11(Pho
kQtPPASPSPQPAEDRPP	A0A1B0GWS4	N-Term(iTRAQ4plex); K1(iTRAQ4plex); T3(P
vEAkEEsEEsDEDMGFGL	P19945	N-Term(iTRAQ4plex); K4(iTRAQ4plex); S7(P
sRtPPAIR	A0A0G2K2M9	N-Term(iTRAQ4plex); S1(Phospho); T3(Phos
sSILPFtPPVVk	O70436	N-Term(Acetyl); T7(Phospho); K12(iTRAQ4p
kPEkPLFSSTsPQDSSPR	O70187	N-Term(iTRAQ4plex); K1(iTRAQ4plex); K4(iT
vPEkGDFsPFGNTQGPSF	F1LMT8	N-Term(iTRAQ4plex); K4(iTRAQ4plex); S8(P
kLHFsVYDFDR	A0A0G2JSR2	N-Term(iTRAQ4plex); K1(iTRAQ4plex); S5(P
vEGIEGsPAEEAR	A0A0G2JYI0	N-Term(iTRAQ4plex); S7(Phospho)
vDVsPTSQR	Q9ER34	N-Term(iTRAQ4plex); S4(Phospho)
rHStEGPEDPPPWAEk	Q5BJT1	N-Term(iTRAQ4plex); T4(Phospho); K16(iTR
sSsPLSk	Q5U300	N-Term(Acetyl); S3(Phospho); K7(iTRAQ4ple
lASDDRPsPPR	P70501	N-Term(iTRAQ4plex); S8(Phospho)

Information Classification: General

sPPYTAFLGNLPYDVTED	Q5RKG9	N-Term(iTRAQ4plex); S1(Phospho); K21(iTR
ktSGLGSPR	D4AA54	N-Term(iTRAQ4plex); K1(iTRAQ4plex); T2(P
ltRESGTDFPIPAVPPGTD	A0A096MJN4	N-Term(iTRAQ4plex); T2(Phospho); K24(iTR
gQGTHLPRPGEDEDEED	Q5I0K2	N-Term(iTRAQ4plex); S27(Phospho)
mNVPETMNEVLDMsDDE	A0A0G2K1R9	N-Term(iTRAQ4plex); S14(Phospho); K20(iT
fHLNDLAcHSVLPLLLTSS	F1M3W5	N-Term(iTRAQ4plex); C8(Carbamidomethyl);
aQQEAEAAQRLsEEEAQ	Q1RP74	N-Term(iTRAQ4plex); S12(Phospho)
iGHHsTSDDSSAYR	Q5EB89	N-Term(iTRAQ4plex); S5(Phospho)
rGSTTSIPsPQSDGGDPN	D4ACS3	N-Term(iTRAQ4plex); S9(Phospho)
tLEtPAAQMEGFLNR	G3V6S0	N-Term(iTRAQ4plex); T4(Phospho)
tRSPDVISSASTALSQDIP	Q3ZAV8	N-Term(iTRAQ4plex); T1(Phospho)
tkSPTPESSTIASYVTLR	E9PTG5	N-Term(iTRAQ4plex); T1(Phospho); K2(iTRA
vAQsDGEEsPAEEGQLLG	A0A0G2K0X9	N-Term(iTRAQ4plex); S4(Phospho); S9(Phos
gASsPDMEPSYGGGLFDI	D4A0I5	N-Term(iTRAQ4plex); S4(Phospho); K20(iTR
tSAWLDDGDELDFsPPR	D4A9G6	N-Term(iTRAQ4plex); S14(Phospho)
kVQIPVSHPDPDPVsDNE	F1M4A0	K1(iTRAQ4plex); N-Term(iTRAQ4plex); S15(
sPAVsPSkAsEDAk	F8WFS9	N-Term(iTRAQ4plex); S5(Phospho); K8(iTRA
rSsEVEIFVDcEcGk	Q8CGU9	N-Term(iTRAQ4plex); S3(Phospho); C11(Ca
rADQLADEsLESTR	P60881	N-Term(iTRAQ4plex); S9(Phospho)
kGsGSEQEGEEEEGGER	Q32PZ5	N-Term(iTRAQ4plex); K1(iTRAQ4plex); S3(P
mDsDsGEQSEGEPGTAA	F1LX20	N-Term(Acetyl); S3(Phospho); S5(Phospho);
sQVEtEDLILkPGVVHVIDI	A0A0H2UHZ1	N-Term(iTRAQ4plex); T5(Phospho); K11(iTR
sFcPGGTDSVsPPPSVIAC	F1M403	N-Term(iTRAQ4plex); C3(Carbamidomethyl);
aSAGPVPGAVVIAEGLHP	D3ZSY8	N-Term(iTRAQ4plex); S22(Phospho); K34(iT
atGAEEYAQQDVVR	P13233	N-Term(iTRAQ4plex); T2(Phospho)
kSSPsTGSLDSGSESk	Q5FVC7	N-Term(iTRAQ4plex); K1(iTRAQ4plex); S5(P
lPYsVLLEIQk	A0A0G2K931	N-Term(iTRAQ4plex); S4(Phospho); K11(iTR
qNsDPTSENPPLPTR	D3ZZQ0	N-Term(iTRAQ4plex); S3(Phospho)
tAFDEAIAELDTLsEESYkD	P63102	N-Term(iTRAQ4plex); S14(Phospho); K19(iT
aGtkSPAVSPSk	F8WFS9	N-Term(iTRAQ4plex); T3(Phospho); K4(iTRA
aGEQQLsEPEDmEMEAG	F1LM09	N-Term(iTRAQ4plex); S7(Phospho); M12(Ox
qEDPASkDPEsPEGQAQ	D4A1J3	N-Term(iTRAQ4plex); K7(iTRAQ4plex); S11(
sAsQAEEEPcLEPVkk	A0A0G2JSZ0	N-Term(iTRAQ4plex); S3(Phospho); C10(Ca
scDsDEDYEAGGTGR	D4AA54	N-Term(iTRAQ4plex); C2(Carbamidomethyl);
gIEFPMADLDALsPIHTPQ	A0A0G2K9L2	N-Term(iTRAQ4plex); S13(Phospho)
iEDVGsDEEDDSGkDk	P34058	N-Term(iTRAQ4plex); S6(Phospho); K14(iTR
kVQVAPLQGsPPLSHDDF	Q3ZB99	N-Term(iTRAQ4plex); K1(iTRAQ4plex); S10(
eSTsSkSPPR	Q5U2Y1	N-Term(iTRAQ4plex); S4(Phospho); K6(iTRA
sLsTSGEsLYHVLGLDk	A0A0G2JX56	N-Term(iTRAQ4plex); S3(Phospho); S8(Phos
vQPPSPLPSSIDSNsSPEE	A0A0G2K6R9;F1M9N9	N-Term(iTRAQ4plex); S15(Phospho); K28(iT
hSPVsPSAk	A0A0G2K6R9;F1M9N9	N-Term(iTRAQ4plex); S5(Phospho); K9(iTRA
sADTPSAPsPPPTPAPVP	F1M3L7	N-Term(iTRAQ4plex); S9(Phospho); K33(iTR
aVDssSEEIEVEVPVVDR	D3ZKX8	N-Term(iTRAQ4plex); S4(Phospho); S5(Phos
mETDAPQPGLASPDsPH	Q8K3P4	N-Term(Acetyl); S15(Phospho); C20(Carbam
mDVsPEPPLAPQPPASW	D5MTG9	N-Term(iTRAQ4plex); S4(Phospho); K34(iTR
lAEGEEEkPEPDGSsEESI	A0A096MKG6	N-Term(iTRAQ4plex); K8(iTRAQ4plex); S15(
gQGTAsPGSVSDLAQTVk	M0R5U4	N-Term(iTRAQ4plex); S6(Phospho); K18(iTR
sPLLSASHSGNVTPTAPP	Q4V888	N-Term(iTRAQ4plex); S24(Phospho)
ekDDVsPQPLMTDLER	A0A0G2JYD4	N-Term(iTRAQ4plex); K2(iTRAQ4plex); S6(P
eRQEsESEQELVNk	D3ZNI3	N-Term(iTRAQ4plex); S5(Phospho); K14(iTR
cStPTYcDLGk	A0A0G2JSR0	N-Term(Acetyl); C1(Carbamidomethyl); T3(P
aPPQQPsPASSPSAVQFF	Q9JJS4	N-Term(iTRAQ4plex); S7(Phospho)
fSEMMDHMGGDEDVDLP	P83868	N-Term(iTRAQ4plex); S29(Phospho); K33(iT
sSSAAASSSTSSsAILPAA	M0RDW5	N-Term(iTRAQ4plex); S13(Phospho)
sPATVksPVEAk	F1LRZ7	N-Term(iTRAQ4plex); K6(iTRAQ4plex); S7(P

kETsPETSLIQDEVALk	A0A0U1RRX4;F1MAQ5	N-Term(iTRAQ4plex); K1(iTRAQ4plex); S4(P
qQLAAGTSAGsPEk	D4A206	N-Term(iTRAQ4plex); S11(Phospho); K14(iT
iEPFsSPPELPDVMkPQDS	A0A140TA99	N-Term(iTRAQ4plex); S5(Phospho); K15(iTR
aPtPGGGHPDsPGLPAPA	O35430	N-Term(iTRAQ4plex); T3(Phospho); S11(Pho
rLPSSPASPsPkGtPEk	A0A0G2K0F3	N-Term(iTRAQ4plex); S10(Phospho); K12(iT
vSALAGPSsDDENEEESk	D3ZLS5	N-Term(iTRAQ4plex); S9(Phospho); K18(iTR
ekPNSGGTksDSASDSQE	F1M110	N-Term(iTRAQ4plex); K2(iTRAQ4plex); K9(iT
sGQAsPSRPESPRPPFDL	P21575	N-Term(iTRAQ4plex); S5(Phospho)
tAGAVAGktPDASPEPk	A0A0G2JTW5	N-Term(iTRAQ4plex); K8(iTRAQ4plex); T9(P
tSASIGsLcADAR	P60203	N-Term(iTRAQ4plex); S7(Phospho); C9(Carb
smSAPSDLEMIGNEDLEF	F1LZX5	N-Term(iTRAQ4plex); S1(Phospho); M2(Oxid
lSYAsAESLETMSEAELPL	Q9QXY2	N-Term(iTRAQ4plex); S5(Phospho)
mDsDSGEQSEGEPGTAA	F1LX20	N-Term(Acetyl); S3(Phospho); K25(iTRAQ4p
eSSESTNTtIEDEDTkVR	P11275	N-Term(iTRAQ4plex); T9(Phospho); K16(iTR
gRsFEGNLNTYk	P47860	N-Term(iTRAQ4plex); S3(Phospho); K12(iTR
vQVAPLQGsPPLSHDDR	Q3ZB99	N-Term(iTRAQ4plex); S9(Phospho)
aVPVsPSAVEEDEDEDGH	F1M4A0	N-Term(iTRAQ4plex); S5(Phospho)
rPEAAsPQkER	F1LPP6	N-Term(iTRAQ4plex); S6(Phospho); K9(iTRA
kSEPsSPDHGSSAIEQDL	F1M084	N-Term(iTRAQ4plex); K1(iTRAQ4plex); S5(P
qGAIVAVTGDGVNDsPAL	P06686;P06687;P06685	N-Term(iTRAQ4plex); S15(Phospho); K19(iT
iVEPEVVGEsDSEVEGDA	D4ACM9	N-Term(iTRAQ4plex); S10(Phospho)
isVGSDADLVIWDPDSVk	P47942	N-Term(iTRAQ4plex); S2(Phospho); K18(iTR
eTISAIDTsPkENTPVR	D4A4M0	N-Term(iTRAQ4plex); S9(Phospho); K11(iTR
eSQSNLIADGDGSEVsDE	F1LMD9	N-Term(iTRAQ4plex); S16(Phospho); C23(Ca
ekPNSGGTksDsAsDSQE	F1M110	N-Term(iTRAQ4plex); K2(iTRAQ4plex); K9(iT
sLDGAPIGVVDQSLmk	A0A0G2K162	N-Term(iTRAQ4plex); S1(Phospho); M15(Ox
kSPATPQAkPEGAAGAAA	D4A0E2	N-Term(iTRAQ4plex); K1(iTRAQ4plex); K9(iT
rVNsPESER	D3ZAU7	N-Term(iTRAQ4plex); S4(Phospho)
kTEEAAEDVFkDTEssPGS	F1M0A6	N-Term(iTRAQ4plex); K1(iTRAQ4plex); K11(
vDLAGsPDQEASGLPDPF	G3V9X2	N-Term(iTRAQ4plex); S6(Phospho)
mDsYEQEEDIDQIVAEVk	O35430	N-Term(iTRAQ4plex); M1(Oxidation); S3(Pho
tAGtPDGPEAPPGPDAsPI	Q5U2N3	N-Term(iTRAQ4plex); T4(Phospho); S17(Pho
rSGLILtIPLcLEQtNmDELS	B5DFL9	N-Term(iTRAQ4plex); T7(Phospho); C11(Car
gSYLTHEAsGLDEQGEAF	F1M7V6	N-Term(iTRAQ4plex); S9(Phospho)
aEGAGtEEEGTQk	Q05175;A0A0G2K1L8	N-Term(iTRAQ4plex); T6(Phospho); K13(iTR
tSTFcGTPEFLAPEVLTDT	Q63433	N-Term(iTRAQ4plex); T1(Phospho); C5(Carb
sPSLsPSPPsPIEk	P15205;F1LRL9	N-Term(iTRAQ4plex); S5(Phospho); S10(Pho
ksSTVSEDVEATVPMLQR	Q4V8I7	N-Term(iTRAQ4plex); K1(iTRAQ4plex); S2(P
fQSVQIQATLsPPLQTk	D3ZHW0	N-Term(iTRAQ4plex); S11(Phospho); K17(iT
lLsPGTSk	D3ZV52	N-Term(iTRAQ4plex); S3(Phospho); K8(iTRA
tYQVPPVQPAsPGSHNAL	Q9ERH3	N-Term(iTRAQ4plex); S11(Phospho)
dYDDMsPR	P61980	N-Term(iTRAQ4plex); S6(Phospho)
sAPtSPcDQELk	F1LMV8	S1(Phospho); N-Term(iTRAQ4plex); T4(Phos
gQESsSDQEQVDVESIDF	B5DEG7	N-Term(iTRAQ4plex); S5(Phospho); K20(iTR
ickPLHELImQLLEEtPEEk	O35264	N-Term(iTRAQ4plex); C2(Carbamidomethyl);
sVTSNQsDGtQEScESPD	D4ACZ5	N-Term(iTRAQ4plex); S7(Phospho); T10(Pho
aGEEDEGDVDsDWDHET	F1M0U5	N-Term(iTRAQ4plex); S11(Phospho); K20(iT
gIVYAIsPDR	A0A0G2KB92	N-Term(iTRAQ4plex); S7(Phospho)
sTEESLSEDVFTESELsPI	A0A0G2JZ50	N-Term(iTRAQ4plex); S17(Phospho)
hSWDsPAFNNDVVQR	A0A0G2JZX5	N-Term(iTRAQ4plex); S5(Phospho)
dEDSMDMDmsPLRPQSY	Q7TP95	N-Term(iTRAQ4plex); M9(Oxidation); S10(Ph
eRsPALksPLQSVVVR	Q5M7V8	N-Term(iTRAQ4plex); S3(Phospho); K7(iTRA
lAIQEMVSLTsPSAPPTSR	F1M863;F1M8A4	N-Term(iTRAQ4plex); S11(Phospho)
rLsLESEGANEGAAAAPE	D3ZQL7	N-Term(iTRAQ4plex); S3(Phospho)
mPFPVNHGASsEDSLLQ	Q00981	N-Term(iTRAQ4plex); S11(Phospho); K21(iT

kGTAASTTGASAsSPR	D4A206	N-Term(iTRAQ4plex); K1(iTRAQ4plex); S13(
eSPPQPPADDGSEEPGS	D4A1Q2	N-Term(iTRAQ4plex); K23(iTRAQ4plex); S24
tLSVAAAFNEDEDsEPEEı	A0A0H2UHW4	N-Term(iTRAQ4plex); S14(Phospho); M19(O
sPVVsGDTsPR	D4A1Q2	N-Term(iTRAQ4plex); S5(Phospho); S9(Phos
aQMSPPSDQEsPPSSET	E9PU32	N-Term(iTRAQ4plex); S11(Phospho)
rHsGQDVHVVLk	A0A0G2K911	N-Term(iTRAQ4plex); S3(Phospho); K12(iTR
aVDWIFSHIDDLDAEAAM	D3ZVQ0	N-Term(iTRAQ4plex); S21(Phospho)
vNLksPSSEEVQGENAGR	F1M084	N-Term(iTRAQ4plex); K4(iTRAQ4plex); S5(P
aFYGsEEDEAkGPGSEEA	D4AE00	N-Term(iTRAQ4plex); S5(Phospho); K11(iTR
sEPSsPDHGSSAIEQDLA	F1M084	N-Term(iTRAQ4plex); S5(Phospho); K27(iTR
tESVTSGPLsPEGsPskSP	F8WFS9	N-Term(iTRAQ4plex); S10(Phospho); S14(Pl
lGLALNFSVFYYEILNsPEI	P63102;P35213	N-Term(iTRAQ4plex); S17(Phospho); K20(iT
dDISEIQSLAsDHsVR	F1M4A0	N-Term(iTRAQ4plex); S11(Phospho); S14(Pl
lLLPGSSPSsPEDEVkDR	C0IXW5	N-Term(iTRAQ4plex); S10(Phospho); K16(iT
dWEDDsDEDmSNFDR	P83868	N-Term(iTRAQ4plex); S6(Phospho); M10(Ox
sMDSSDLSDGAVTLQEYI	A0A0G2K527	N-Term(iTRAQ4plex); S1(Phospho); K21(iTR
dScsSPPSLNYGk	Q7TP36	N-Term(iTRAQ4plex); C3(Carbamidomethyl);
hsPTEDEEIAk	P15205;F1LRL9	N-Term(iTRAQ4plex); S2(Phospho); K11(iTR
gPSSQEDESGGIEDsPDR	A0A0G2K0F3	N-Term(iTRAQ4plex); S15(Phospho); C21(Ca
lsPAksPSLSPSPPsPIEk	P15205;F1LRL9	N-Term(iTRAQ4plex); S2(Phospho); K5(iTRA
gQEDNLASAVDATTGQE	D4A4Z0	N-Term(iTRAQ4plex); C19(Carbamidomethyl
lAEVLQLGsDILPQYk	Q63472	N-Term(iTRAQ4plex); S9(Phospho); K16(iTR
tAEEkEPAAPAsPAPSPVF	D4A644	N-Term(iTRAQ4plex); K5(iTRAQ4plex); S12(
stELLIR	D3ZJ08;D3ZK97	N-Term(iTRAQ4plex); T2(Phospho)
kPsPPQAAETPALELPLP	D4ACV0	N-Term(iTRAQ4plex); K1(iTRAQ4plex); S3(P
aEGAGTEEEGTQkEsEPC	Q05175;A0A0G2K1L8	N-Term(iTRAQ4plex); K13(iTRAQ4plex); S15
ykPAPLAALPNTLDPAEYI	F1LPG5	N-Term(iTRAQ4plex); K2(iTRAQ4plex); S21(
eVkSSPsEsPLmEk	Q9JJP0	N-Term(iTRAQ4plex); K3(iTRAQ4plex); S7(P
sQsEYcNVGTk	A0A0G2K9M4	N-Term(iTRAQ4plex); S3(Phospho); C6(Carb
tAsEGSEAETPEAPk	M0RC12	N-Term(iTRAQ4plex); S3(Phospho); K15(iTR
dYNASASTIsPPssmEEDk	P15205;F1LRL9	N-Term(iTRAQ4plex); S10(Phospho); S13(Pl
iPNANVSGsHR	Q63881	N-Term(iTRAQ4plex); S9(Phospho)
sLSsSPQAQPPRPAELSD	Q9JHZ4	N-Term(iTRAQ4plex); S4(Phospho)
aASAAAATAAASAATAAS	O08629	N-Term(Acetyl); S23(Phospho); K35(iTRAQ4
mLAESDEsGDEESVSQTI	D4A9D8	N-Term(iTRAQ4plex); S8(Phospho); K19(iTR
iPIVAQNsEEESPLSPVGC	D3Z9C7	N-Term(iTRAQ4plex); S8(Phospho)
dLGHPVEEEDEsGDQEDI	P41542	N-Term(iTRAQ4plex); S12(Phospho)
nSGPVLEVNTDFsPEVk	A0A0G2K2M9	N-Term(iTRAQ4plex); S13(Phospho); K17(iT
ekEVVPDsPGDkGSTNR	P34926;Q63330	N-Term(iTRAQ4plex); K2(iTRAQ4plex); S8(P
sNSPLPSIQLQPQsPSASI	Q00A22	N-Term(iTRAQ4plex); S14(Phospho); K19(iT
iVsAQSLAEDDVE	Q62760	N-Term(iTRAQ4plex); S3(Phospho)
gSSDSLDSLsPR	O35867	N-Term(iTRAQ4plex); S10(Phospho)
nVPQEEsLEDSDVDADFk	Q56R18	N-Term(iTRAQ4plex); S7(Phospho); K18(iTR
qGDNIsDDEDEVR	G3V8I4	N-Term(iTRAQ4plex); S6(Phospho)
sSSGMGGRmPVsRGR	A0A0G2K8K9	N-Term(iTRAQ4plex); M9(Oxidation); S12(Pl
eQEssGEEDNDLSPEER	A0A0H2UHA0	N-Term(iTRAQ4plex); S4(Phospho); S5(Phos
fLENAAADFsDEDEDEDII	G3V6L8	N-Term(iTRAQ4plex); S10(Phospho)
nSWIQIIQDTINSLNRDED	F1M3G7	N-Term(iTRAQ4plex); S24(Phospho); K30(iT
mLSSsFEDDPFFADSFLA	D3ZCQ9	N-Term(iTRAQ4plex); S5(Phospho)
kLDEDAsPNEEk	B2GV74	N-Term(iTRAQ4plex); K1(iTRAQ4plex); S7(P
qSLSsADNLEPDAQGHP\	Q7TSU1	N-Term(iTRAQ4plex); S5(Phospho)
kQFsLENVPEGELLQDGk	D3ZT79	N-Term(iTRAQ4plex); K1(iTRAQ4plex); S4(P
sEEQGLQDPEkQEsPVEE	D4A1J3	N-Term(iTRAQ4plex); K11(iTRAQ4plex); S14
dWEDDsDEDMSNFDR	P83868	N-Term(iTRAQ4plex); S6(Phospho)
sDADLVGGGEDGDDyDD	F1LMY3	N-Term(iTRAQ4plex); Y15(Phospho); Y20(Pl

Information Classification: General

eTPSSSPVsPQEsPkHEN	F1M820	N-Term(iTRAQ4plex); S9(Phospho); S13(Pho
aDSEAssGPLLDDk	F1M5M9	N-Term(iTRAQ4plex); S6(Phospho); S7(Phos
eGDGsATTDAAPATsPkA	P07936	N-Term(iTRAQ4plex); S5(Phospho); S15(Pho
gQLsDDEkFLFVDk	E9PTU4	N-Term(iTRAQ4plex); S4(Phospho); K8(iTRA
tSsPAQGVGASVSAMRPD	P47987	N-Term(iTRAQ4plex); S3(Phospho)
gNDsDGEAESDDPEkk	Q4KLL7	N-Term(iTRAQ4plex); S4(Phospho); K15(iTR
nLEAVETLGSTSTIcSDktC	P06686;P06687;P06685	N-Term(iTRAQ4plex); C15(Carbamidomethyl
sETAPAETTAPAPVEksPA	D3ZBN0	N-Term(Acetyl); K16(iTRAQ4plex); S17(Phos
sNSGRELtDEEILASVMIk	A0A0G2K7S6;A0A0G2JXT	N-Term(iTRAQ4plex); T8(Phospho); K19(iTR
nIIsPPWDFR	A0A0G2JT88	N-Term(iTRAQ4plex); S4(Phospho)
vASPGGLRDsTVR	A0A0H2UHF5	N-Term(iTRAQ4plex); S10(Phospho)
lPHTGPADDGAtRSPsIDS	F1M2K6	N-Term(iTRAQ4plex); T12(Phospho); S16(Ph
rSAsADNLILPR	F1MA89	N-Term(iTRAQ4plex); S4(Phospho)
eEyQREILGNAk	P20720	N-Term(iTRAQ4plex); Y3(Phospho); K12(iTR
dDtSPPkDk	F1LXQ7	N-Term(iTRAQ4plex); T3(Phospho); K7(iTRA
lISGDAEPtPEQEEk	D4A2D3	N-Term(iTRAQ4plex); T9(Phospho); K15(iTR
gTPALTSETAQSsPPTGL	D4A1Z8	N-Term(iTRAQ4plex); S13(Phospho)
vLHAQcHSTPDsAEDVRk	F1LM19	N-Term(iTRAQ4plex); C6(Carbamidomethyl);
qPAGsPRGRPR	F1M5X7	N-Term(iTRAQ4plex); S5(Phospho)
vDGGGGEEPAGsFEDAE	F1LSH6	N-Term(iTRAQ4plex); S12(Phospho)
kNDGVkESSEsTNTTIEDE	P11275	K1(iTRAQ4plex); N-Term(iTRAQ4plex); K6(iT
dFSGAGGIGVFSESSSVA	A0A0G2K207	N-Term(iTRAQ4plex); S19(Phospho); K20(iT
rPAsDDEcPSDSk	D3ZK36	N-Term(iTRAQ4plex); S4(Phospho); C8(Carb
skTPPsNLsPIEDAsPTEEL	G3V984	N-Term(iTRAQ4plex); K2(iTRAQ4plex); S6(P
sEFsDLEQSVk	D3ZYR4	N-Term(iTRAQ4plex); S4(Phospho); K11(iTR
dGsPDAPAtPEkEEVPFSE	A0A0U1RRX4;F1MAQ5	N-Term(iTRAQ4plex); S3(Phospho); T9(Phos
vVDLMAYMAskE	M0R660;E9PTN6;D3ZQT(	N-Term(iTRAQ4plex); S10(Phospho); K11(iT
vSEEDQsLENsEADVk	G3V7X4	N-Term(iTRAQ4plex); S7(Phospho); S11(Pho
tPEEGGYSYEISEk	P15205;F1LRL9	N-Term(iTRAQ4plex); T1(Phospho); K14(iTR
sQsLPNSLDYAQTSER	Q66H15	N-Term(iTRAQ4plex); S3(Phospho)
aEEQLPPLLSPPsPSTPHS	A0A140TA95	N-Term(iTRAQ4plex); S13(Phospho)
eALELLksAIAk	M0R5J4	N-Term(iTRAQ4plex); K7(iTRAQ4plex); S8(P
aGEQQLsEPEDMEMEAG	F1LM09	N-Term(iTRAQ4plex); S7(Phospho)
aAEEAFVNDIDEsSPGTEY	Q5PPP1	N-Term(iTRAQ4plex); S13(Phospho)
tLsQSSESGTLPSGPPGH	A0A0G2JSU4	N-Term(iTRAQ4plex); S3(Phospho); C24(Ca
sPPEMLESDcPSPLELk	Q01066	N-Term(iTRAQ4plex); S1(Phospho); C10(Ca
tTTtPEVk	P15205;F1LRL9	N-Term(iTRAQ4plex); T4(Phospho); K8(iTRA
mDsFDEDLARPSGLLAQE	Q5M7V8	N-Term(iTRAQ4plex); S3(Phospho)
fDTtPNELVQLNk	A0A0G2JZ50	N-Term(iTRAQ4plex); T4(Phospho); K13(iTR
lRPFTFSQsTPIGLDR	D3ZWB4	N-Term(iTRAQ4plex); S9(Phospho)
aAQGPAGsPDkGk	Q9QXY2	N-Term(iTRAQ4plex); S8(Phospho); K11(iTR
tsMGGTQQQFVEGVR	A0A0G2JT93	N-Term(iTRAQ4plex); S2(Phospho)
gsPQQIDHAk	M0R961	N-Term(iTRAQ4plex); S2(Phospho); K10(iTR
qPPAALIkPASSPPSQSAs	D3ZE17	N-Term(iTRAQ4plex); K8(iTRAQ4plex); S19(
kLPsDTEkEDR	F1LQN3	N-Term(iTRAQ4plex); K1(iTRAQ4plex); S4(P
mSLQVMDSEPEGQsPPR	G3V864	N-Term(iTRAQ4plex); S14(Phospho)
iLDTSSLTQSAPAsPTNk	D3ZDU2	N-Term(iTRAQ4plex); S14(Phospho); K18(iT
sPAEAksPAEVk	F1LRZ7	N-Term(iTRAQ4plex); K6(iTRAQ4plex); S7(P
aHsPQGEGEIPLHR	A0A0U1RS13	N-Term(iTRAQ4plex); S3(Phospho)
gASsPDmEPSYGGGLFDI	D4A0I5	N-Term(iTRAQ4plex); S4(Phospho); M7(Oxid
tDTAADGEtsAtESDQEED	G3V874;A0A0G2K1Q9	N-Term(iTRAQ4plex); T9(Phospho); S10(Pho
nLPDLTAQETETSLsP	Q5XIQ6	N-Term(iTRAQ4plex); S15(Phospho)
vENALNNLDDsPSPGDR	Q7TP36	N-Term(iTRAQ4plex); S11(Phospho)
dGmDTScSTGsPGAATAN	Q9JKM5	N-Term(iTRAQ4plex); M3(Oxidation); C7(Car
eLEAERsLmssPEDLTkDR	P15205;F1LRL9	N-Term(iTRAQ4plex); S7(Phospho); M9(Oxid

gGAPLPPsGSkDQAVGA(	O35458	N-Term(iTRAQ4plex); S8(Phospho); K11(iTR
gVQAGNsDTEGGQPGR	E9PST5	N-Term(iTRAQ4plex); S7(Phospho)
sQsVVEAGTLk	F1LRF3	N-Term(iTRAQ4plex); S3(Phospho); K11(iTR
sGDEmIFDPTmSk	Q6P685	N-Term(Acetyl); S1(Phospho); M5(Oxidation)
sLEEEESQL	Q8R4T5	N-Term(iTRAQ4plex); S1(Phospho)
lPsLSVQPVSNADWVWL\	D3ZF86	N-Term(iTRAQ4plex); S3(Phospho); K19(iTR
fSFYGNLsPR	F1M8G8	N-Term(iTRAQ4plex); S8(Phospho)
dFQEYVEPGEDFPAsPQF	G3V7G0	N-Term(iTRAQ4plex); S15(Phospho)
sMEDPFFVVk	Q63635	N-Term(Acetyl); S1(Phospho); K10(iTRAQ4p
aAVGLTGNDIAtPPNk	A0A0G2JW88	N-Term(iTRAQ4plex); T12(Phospho); K16(iT
vsPSPSQESLSSSk	A0A0G2K7S6;A0A0G2JX7	N-Term(iTRAQ4plex); S2(Phospho); K14(iTR
vGSLTPPssPk	P0C1X8	N-Term(iTRAQ4plex); S8(Phospho); S9(Phos
iVNDDQSFyADIYMEDGLI	P47942;Q62952	N-Term(iTRAQ4plex); Y9(Phospho); K20(iTR
qNDVEIPsPTQk	F1M1T9	N-Term(iTRAQ4plex); S8(Phospho); K12(iTR
sGVAVPTsPk	Q9JID1	N-Term(iTRAQ4plex); S8(Phospho); K10(iTR
kVEEEQEADEEDVsEEET	Q52KJ9	N-Term(iTRAQ4plex); K1(iTRAQ4plex); S14(
lcAGIMITAsHNPk	F7FLB2	N-Term(iTRAQ4plex); C2(Carbamidomethyl);
sFsLGDLSHsPQTAQHVE	D4A8V2	N-Term(iTRAQ4plex); S3(Phospho); S10(Pho
sENSSTQsSPEMPTTk	Q5EB70	N-Term(iTRAQ4plex); S8(Phospho); K16(iTR
hAsLDGASPYFk	F1MAS1	N-Term(iTRAQ4plex); S3(Phospho); K12(iTR
rGsLPDNSILHR	F1LPM3	N-Term(iTRAQ4plex); S3(Phospho)
tSVPESSVAAGTGsPSk	F1M392	N-Term(iTRAQ4plex); S14(Phospho); K17(iT
aGSsPRSSAQGR	D3ZGH9	N-Term(iTRAQ4plex); S4(Phospho)
eFDELSPsAQR	P11507	N-Term(iTRAQ4plex); S8(Phospho)
fLEQSRsPSVSPSk	F1LSG8	N-Term(iTRAQ4plex); S7(Phospho); K14(iTR
rVsVcAETFNPDEEEDNDI	G3V8Q6	N-Term(iTRAQ4plex); S3(Phospho); C5(Carb
sSSsPELQTLQDILGDLGI	D3ZLW4	N-Term(iTRAQ4plex); S4(Phospho); K20(iTR
aPAPAAPAAEPQAEAPVA	Q05175;A0A0G2K1L8	N-Term(iTRAQ4plex); S19(Phospho); K27(iT
sTDIGFTPPSsPPTRPR	F1M5N7	N-Term(iTRAQ4plex); S11(Phospho)
dTTDDLsVVETkEPF	F1LPT0	N-Term(iTRAQ4plex); S7(Phospho); K12(iTR
tNTNVNcPIEcFMPLDVQA	F1LST1	N-Term(iTRAQ4plex); C7(Carbamidomethyl);
sYsPDGkESPSDk	A0A0G2JSR7	N-Term(iTRAQ4plex); S3(Phospho); K7(iTRA
nLEAPYIPDVSsPSDTSNF	A0A0G2KB58	N-Term(iTRAQ4plex); S12(Phospho)
sPAEAksPAEAkPPAEAk	F1LRZ7	N-Term(iTRAQ4plex); S1(Phospho); K6(iTRA
gIsALLLNQGDGDR	A0A0G2JZK7	N-Term(iTRAQ4plex); S3(Phospho)
iFSEAtPk	A0A0G2JWM2	N-Term(iTRAQ4plex); T6(Phospho); K8(iTRA
mGPSSSPIPSPSPsPTDS	P63319	N-Term(iTRAQ4plex); M1(Oxidation); S14(Ph
tGsPLTVATGNDQAATEA	F1M1Y0	N-Term(iTRAQ4plex); S3(Phospho); K19(iTR
nLDIERPTYtNLNR	Q5XIF6;P68370	N-Term(iTRAQ4plex); T10(Phospho)
qSNASsDVEAEEkETSVS	Q566D6	N-Term(iTRAQ4plex); S6(Phospho); K13(iTR
aDGATsDDLDLHDDR	F1M4A0	N-Term(iTRAQ4plex); S6(Phospho)
vIISTPSADAPmFVmsVNH	F1M2N4	N-Term(iTRAQ4plex); M12(Oxidation); M15((
tNSPDLDSQSLSLSSGAD	A0A0G2JTB5	N-Term(iTRAQ4plex); T1(Phospho)
tNtFcGTPDYIAPEILLGQk	F1LM10	N-Term(iTRAQ4plex); T3(Phospho); C5(Carb
dDIFSSsEEEGkDR	Q63190	N-Term(iTRAQ4plex); S7(Phospho); K12(iTR
sVsLSk	A0A0G2K5B0	N-Term(iTRAQ4plex); S3(Phospho); K6(iTRA
aAAtPEPAVAQPDSTALE(	Q63092	N-Term(iTRAQ4plex); T4(Phospho); K29(iTR
tVSETPAVPPVsEDEDDD	P35465	N-Term(iTRAQ4plex); S12(Phospho); K35(iT
aEDGAAPsPSSETPk	F1LMW7	N-Term(iTRAQ4plex); S8(Phospho); K15(iTR
kGGEFDEFVNDDtDDDLF	Q32PZ5	N-Term(iTRAQ4plex); K1(iTRAQ4plex); T13(
gVQVETISsGDGR	Q62658	N-Term(iTRAQ4plex); S9(Phospho)
aTAPQTQHVsPMR	Q68FR9	N-Term(iTRAQ4plex); S10(Phospho)
qMNMsPPPGNAGPVIMSI	O08729	N-Term(iTRAQ4plex); S5(Phospho); K21(iTR
gAVPHsSPNLAEVk	A0A0G2QC41	N-Term(iTRAQ4plex); S6(Phospho); K14(iTR
lAGsLVFSVDk	Q9Z2X9	N-Term(iTRAQ4plex); S4(Phospho); K11(iTR

aADLGQIPDVDIDsDGIFk	D3ZP47	N-Term(Acetyl); S14(Phospho); K19(iTRAQ4
aEAPAGPALGLPsPEVES	D3ZXL5	N-Term(iTRAQ4plex); S13(Phospho)
gsVSDEEMMELR	Q5XI38	N-Term(iTRAQ4plex); S2(Phospho)
sLAPDLsDDEHDPVDSISI	D3ZFD0	N-Term(iTRAQ4plex); S7(Phospho)
tSPAPWk	A0A0G2K2M9	N-Term(iTRAQ4plex); T1(Phospho); K7(iTRA
aAsPAkPSSLDLVPNLPR	B1VKB4	N-Term(iTRAQ4plex); S3(Phospho); K6(iTRA
vAkPksPEPEATLTFPFLD	F1M392	N-Term(iTRAQ4plex); K3(iTRAQ4plex); K5(iT
lAEDsTWPPTNRTDyAVN	F1MA70	N-Term(iTRAQ4plex); S5(Phospho); Y15(Pho
gGGsEDEPSPGGLTER	Q5U2U7	N-Term(iTRAQ4plex); S4(Phospho)
aVDSsSEEIEVEVPVVDR	D3ZKX8	N-Term(iTRAQ4plex); S5(Phospho)
rTsPPVSVR	B5DF41	N-Term(iTRAQ4plex); S3(Phospho)
nIDQSEFEGFsFVNSEFLk	F1LS42	N-Term(iTRAQ4plex); S11(Phospho); K19(iT
qSDsNASFLR	F1M9N9	N-Term(iTRAQ4plex); S4(Phospho)
eLEAERsLmssPEDLTk	P15205;F1LRL9	N-Term(iTRAQ4plex); S7(Phospho); M9(Oxid
dksPVREPIDNLTPEER	Q5BJP4	N-Term(iTRAQ4plex); K2(iTRAQ4plex); S3(P
tSskEsSPVPSPTSDRk	G3V6S0	N-Term(iTRAQ4plex); S3(Phospho); K4(iTRA
eSPVPPSStPPPPPEIsPS	D4A6X3	N-Term(iTRAQ4plex); T9(Phospho); S17(Pho
ePEPAPPYHsPEDSRDHA	F1M6X3	N-Term(iTRAQ4plex); S10(Phospho); K20(iT
mSPLASPPSsPPHYQR	A0A0G2K4S1	N-Term(iTRAQ4plex); S10(Phospho)
ektPsPkEEDEEAESPPEk	A0A0G2K7X3	N-Term(iTRAQ4plex); K2(iTRAQ4plex); T3(P
tsPsPPTQTPHQEAAPR	Q9JJS4	N-Term(iTRAQ4plex); S2(Phospho); S4(Phos
fYFENLWsR	P11275	N-Term(iTRAQ4plex); S8(Phospho)
kLYESLLVGsLPDMNk	A2RRU1	N-Term(iTRAQ4plex); K1(iTRAQ4plex); S10(
sTsQQGEEPQk	A0A096MIT7	N-Term(iTRAQ4plex); S3(Phospho); K11(iTR
fSEmMDHMGGDEDVDLF	P83868	N-Term(iTRAQ4plex); M4(Oxidation); S26(Ph
kGttPPRSPEASPk	D3ZQ55	N-Term(iTRAQ4plex); K1(iTRAQ4plex); T3(P
sNSSYLGSDETGsGDELF	D3ZS72	N-Term(iTRAQ4plex); S13(Phospho); C19(Ca
aVDTLsPDWDFDRVDDG	D4A7I6	N-Term(Acetyl); S6(Phospho); K20(iTRAQ4p
eSEAGTGSSEHEDGERE	F1M3W5	N-Term(iTRAQ4plex); S19(Phospho)
iDDIPGLSDTsPDLSSR	G3V6N7	N-Term(iTRAQ4plex); S11(Phospho)
fIIGsVsEDNSEDEISNLVk	P11497	N-Term(iTRAQ4plex); S5(Phospho); S7(Phos
eNGPAsEEGDLGQEHDIT	F1M9X4	N-Term(iTRAQ4plex); S6(Phospho); K22(iTR
dISLsDYk	A0A0G2K3Z9	N-Term(iTRAQ4plex); S5(Phospho); K8(iTRA
ePPGsPPSSATk	A0A0G2K712	N-Term(iTRAQ4plex); S5(Phospho); K12(iTR
rPAASSAAAASASASsPA(	O08629	N-Term(iTRAQ4plex); S16(Phospho); C31(Ca
rDsQSsNEFLTISDSk	Q4G045	N-Term(iTRAQ4plex); S3(Phospho); S6(Phos
eAGsPPDLASSPR	M0R5E3	N-Term(iTRAQ4plex); S4(Phospho)
rkPGAGGsPALVR	D4A644	N-Term(iTRAQ4plex); K2(iTRAQ4plex); S8(P
hEGLAETPETSPESLSFsI	A0A0G2K6R9;F1M9N9	N-Term(iTRAQ4plex); S18(Phospho); K20(iT
rEDsPGPEVQPMDk	G3V8D6	N-Term(iTRAQ4plex); S4(Phospho); K14(iTR
gLLHGWSQSsTEEVPR	F1LPM3	N-Term(iTRAQ4plex); S10(Phospho)
rYPSSISSsPQk	A0A0G2JYC6	N-Term(iTRAQ4plex); S9(Phospho); K12(iTR
tEATQGLDYVPSAGTISPT	P34926	N-Term(iTRAQ4plex); S20(Phospho); S21(Ph
lPYLVELsPGRR	F1LT10	N-Term(iTRAQ4plex); S8(Phospho)
mDSASQDINLNsPNk	Q6PCT3	N-Term(Acetyl); M1(Oxidation); S12(Phospho
dkTESVTSGPLSPEGsPsI	F8WFS9	N-Term(iTRAQ4plex); K2(iTRAQ4plex); S16(
sPsLGNVPNTPASTISAR	D4A1G8	N-Term(iTRAQ4plex); S3(Phospho)
lVLPMDDSEPALNSLDDtF	D3ZJ86	N-Term(iTRAQ4plex); T18(Phospho)
sAEsPRDAADLFTDLENA	D3ZMR7	N-Term(iTRAQ4plex); S4(Phospho); K22(iTR
vELPLEDQVGDLEASsEE	Q5QD51	N-Term(iTRAQ4plex); S16(Phospho); K19(iT
kAAsLTEDR	M0RD03	N-Term(iTRAQ4plex); K1(iTRAQ4plex); S4(P
tGESARsEDDAASGEHD\	A0A0G2JZX3	N-Term(iTRAQ4plex); S7(Phospho)
gATPAEDDEDNDIDLFGs	Q68FR9	N-Term(iTRAQ4plex); S18(Phospho); K25(iT
eEGTGEsAGGsPAR	B5DF41	N-Term(iTRAQ4plex); S7(Phospho); S11(Pho
nAETVksPEk	B5DFK6	N-Term(iTRAQ4plex); K6(iTRAQ4plex); S7(P

kLPSESDLLEGEVtDEDEE	A0A0G2K4N6	K1(iTRAQ4plex); N-Term(iTRAQ4plex); T14(
lRPPsDPETVYk	D3ZWA1	N-Term(iTRAQ4plex); S5(Phospho); K12(iTR
eLSSEPRtPPAQk	P34926;Q63330	N-Term(iTRAQ4plex); T8(Phospho); K13(iTR
vsVDsNLFVYSksDAFLSk	F1M3W5	N-Term(iTRAQ4plex); S2(Phospho); S5(Phos
eSRQEsDPEDDDVk	D3ZAY8	N-Term(iTRAQ4plex); S6(Phospho); K14(iTR
gVLADREsPEQPFVVLNk	Q9EPY0	N-Term(iTRAQ4plex); S8(Phospho); K18(iTR
gSGVAQsPQQQPPQQPS	F1LPG3	N-Term(iTRAQ4plex); S7(Phospho); K26(iTR
lHFFMPGFAPLtSR	P85108;B4F7C2;P69897;(	N-Term(iTRAQ4plex); T12(Phospho)
aAAVAAAGAGEPLsPEEL	Q75Q41	N-Term(Acetyl); S14(Phospho); K21(iTRAQ4
ssSPVTELTARsPVk	A0A0G2K2M9	N-Term(iTRAQ4plex); S2(Phospho); S12(Pho
tVTPASsAk	P47942	N-Term(iTRAQ4plex); S7(Phospho); K9(iTRA
sETGSTISMSsLER	D4A831	N-Term(iTRAQ4plex); S11(Phospho)
rQsIPEEFk	F5HTD6	N-Term(iTRAQ4plex); S3(Phospho); K9(iTRA
tAsFSESR	A0A0G2K5E7	N-Term(iTRAQ4plex); S3(Phospho)
tAAPsPDRRDsAsPGAAS(	F1LRY7	N-Term(iTRAQ4plex); S5(Phospho); S11(Pho
sHRNSSAVsPk	G3V8F3	N-Term(iTRAQ4plex); S9(Phospho); K11(iTR
nTFTAWsDEDSDYEIDDR	F1M062	N-Term(iTRAQ4plex); S7(Phospho); K22(iTR
kAEGEPQEEsPLk	B1WC16	N-Term(iTRAQ4plex); K1(iTRAQ4plex); S10(
gGsPLTTTQGGsPTk	F1M787	N-Term(iTRAQ4plex); S3(Phospho); S12(Pho
sPsPTLGESLAPR	D3ZNS1	N-Term(iTRAQ4plex); S3(Phospho)
eTEALLQSIGISPEPPLVP	A0A173DW30	N-Term(iTRAQ4plex); M22(Oxidation); S23(F
asGEmASAQYITAALR	A0A0G2JVV5	N-Term(iTRAQ4plex); S2(Phospho); M5(Oxid
sPSEGPVAtTSPR	G3V8Q9	N-Term(iTRAQ4plex); T9(Phospho)
vSVsPGR	B2RYB3;A0A0G2K4F6	N-Term(iTRAQ4plex); S4(Phospho)
lGLAVIHGEAQcTELDMDI	Q63468	N-Term(iTRAQ4plex); C12(Carbamidomethyl
sLSsPTDNLELSAR	A0A0G2K527	N-Term(iTRAQ4plex); S4(Phospho)
eVDGLLTSDPMGsPVSSk	B3DMA1	N-Term(iTRAQ4plex); S13(Phospho); K18(iT
iQQAGNTsPR	Q9JJ76	N-Term(iTRAQ4plex); S8(Phospho)
sLDSLDk	C0IXW5	N-Term(iTRAQ4plex); S1(Phospho); K7(iTRA
vLSAPPHFHFGQtNRTPE	Q68FR6	N-Term(iTRAQ4plex); T13(Phospho)
dDSHsAEDsEDEkDDHk	A0A0G2K7X3	N-Term(iTRAQ4plex); S5(Phospho); S9(Phos
aHsPVQSGLPGMQTLk	B0LT89	N-Term(Acetyl); S3(Phospho); K16(iTRAQ4p
kEESEEsEDDmGFGLFD	A0A0G2K4Q1	K1(iTRAQ4plex); N-Term(iTRAQ4plex); S7(P
vQPPSPLPSsIDsNSSPEE	A0A0G2K6R9;F1M9N9	N-Term(iTRAQ4plex); S10(Phospho); S13(Pl
tSAsPPLEk	A0A0G2K7X3	N-Term(iTRAQ4plex); S4(Phospho); K9(iTRA
ksksPTLR	Q5RKH1	N-Term(iTRAQ4plex); K1(iTRAQ4plex); S2(P
lASELtDPDQPHEDPAEDI	Q7TP93	N-Term(iTRAQ4plex); T6(Phospho); K20(iTR
nTtPTGATPPAGMASTR	A0A0G2JW88	N-Term(iTRAQ4plex); T3(Phospho)
kGPGSLLVSMDQLAsYGI	O88420	N-Term(iTRAQ4plex); K1(iTRAQ4plex); S15(
gTVTGERQsGDGQESTE	G3V793	N-Term(iTRAQ4plex); S9(Phospho); K22(iTR
eVksSPSESPLMEk	Q9JJP0	N-Term(iTRAQ4plex); K3(iTRAQ4plex); S4(P
eLHDMFVDMAMLVEsQG	P61265	N-Term(iTRAQ4plex); S15(Phospho)
sQDTEVDMk	Q794F9	N-Term(Acetyl); S1(Phospho); K9(iTRAQ4ple
gSLPPAALEsSDSTNTTIE	F1LNI8	N-Term(iTRAQ4plex); S10(Phospho); K24(iT
tVsPEGYSER	M0R991	N-Term(iTRAQ4plex); S3(Phospho)
aEEEGGsEEEVGDksPQE	P12839	N-Term(iTRAQ4plex); S7(Phospho); K14(iTR
eDkPEVQsPVk	F1M403	N-Term(iTRAQ4plex); K3(iTRAQ4plex); S8(P
mFLAYQDQsFDIPDR	Q9Z2X9	N-Term(iTRAQ4plex); S9(Phospho)
sLsAPLHPEFEEVYR	D3ZBX9	N-Term(iTRAQ4plex); S3(Phospho)
tIQIIGNWGcQsR	Q63713	N-Term(iTRAQ4plex); C10(Carbamidomethyl
isPIQkPQGLDPVEILQER	E9PTG1	N-Term(iTRAQ4plex); S2(Phospho); K6(iTRA
sYDVPPPPMEPDHPFYSI	P25113	N-Term(iTRAQ4plex); S1(Phospho); K21(iTR
eNsREALVEPAsESPRPA	Q9JJ19	N-Term(iTRAQ4plex); S3(Phospho); S12(Pho
eALGADMGsVTFEDVAVI	Q99PJ6	N-Term(iTRAQ4plex); S9(Phospho); T20(Pho
eEcPRPMSIsPPDFSPk	P15205;F1LRL9	N-Term(iTRAQ4plex); C3(Carbamidomethyl);

tASsPVLSDk	G3V6M0	N-Term(iTRAQ4plex); S4(Phospho); K10(iTR
kEsPPtPDQGAASR	D4A280	K1(iTRAQ4plex); N-Term(iTRAQ4plex); S3(P
tEDVMFISDNEsFNPALW	M0R9T2	N-Term(iTRAQ4plex); S12(Phospho)
dVPLGPEDPkEEDGSFDy	D3ZW56	N-Term(iTRAQ4plex); K10(iTRAQ4plex); Y18
mQDQQEYYVTtPGAEVE	A0A0G2K6R9;F1M9N9;A0	N-Term(iTRAQ4plex); T11(Phospho); K21(iT
ekVAsDTEDTDR	Q62901	N-Term(iTRAQ4plex); K2(iTRAQ4plex); S5(P
aPSVsPPVPPTk	Q9Z0G8	N-Term(iTRAQ4plex); S5(Phospho); K12(iTR
yLELPPRPsPESSESDWE	D3ZVU6	N-Term(iTRAQ4plex); S9(Phospho); K31(iTR
tGWDTSESELsEGELER	F1LTJ8	N-Term(iTRAQ4plex); S11(Phospho)
sEsARGSDsEDEVLR	Q641X2	N-Term(iTRAQ4plex); S3(Phospho); S9(Phos
tEDEEFLIQHLLQAPsPPR	A0A0G2K5M6	N-Term(iTRAQ4plex); S16(Phospho)
gPPDFsSDEEREPTPVLG	Q62733	N-Term(iTRAQ4plex); S6(Phospho)
sGsPSDNsGAEEMEVALA	P47196	N-Term(iTRAQ4plex); S3(Phospho); S8(Phos
yPADEDFRtSQEDLR	A0A0G2JYP3	N-Term(iTRAQ4plex); T9(Phospho)
aSEsPDSAAR	F1MAD2	N-Term(iTRAQ4plex); S4(Phospho)
aSGsENEGDYNPGR	Q14TE9	N-Term(iTRAQ4plex); S4(Phospho)
sAsDTNLQk	M0R8V0	N-Term(iTRAQ4plex); S3(Phospho); K9(iTRA
lLPSSGPSAAEVSTAEEP	D3ZG78	N-Term(iTRAQ4plex); S20(Phospho); S22(Ph
vPFAPGPsPPPLLGNMDQ	D3ZQJ3	N-Term(iTRAQ4plex); S8(Phospho)
lLQsPck	Q62833	N-Term(iTRAQ4plex); S4(Phospho); C6(Carb
aSIsPSNVSALEPQTEMG	F1LQN3	N-Term(iTRAQ4plex); S4(Phospho); K22(iTR
lGTGGGGsPDksPSAQEL	I6L9H8	N-Term(iTRAQ4plex); S8(Phospho); K11(iTR
eLsGESDELGISQDEQLS	M0RBZ7	N-Term(iTRAQ4plex); S3(Phospho); K19(iTR
rFsMEGISNILQSGIR	P97577	N-Term(iTRAQ4plex); S3(Phospho)
aSVSGPNsPSESR	A0A0G2JWJ3	N-Term(iTRAQ4plex); S8(Phospho)
sQSESsDEVTELDLSHGk	D4A5B3	N-Term(iTRAQ4plex); S6(Phospho); K18(iTR
vLHGAQtSDEEkDF	D4A4W6	N-Term(iTRAQ4plex); T7(Phospho); K12(iTR
sRSPPPVSk	Q6PDU1	N-Term(iTRAQ4plex); S1(Phospho); K9(iTRA
sMsASSGLSAR	A0A0G2K5Z1	N-Term(iTRAQ4plex); S3(Phospho)
dGWNQNHFItPVStLER	F1M2K6	N-Term(iTRAQ4plex); T10(Phospho); T14(Ph
tVVLtPTYSGEADALLPSLI	P0C5Y8	N-Term(iTRAQ4plex); T5(Phospho)
tWVTSSADPVSEHGVsTS	D3ZF26	N-Term(iTRAQ4plex); S16(Phospho); K24(iT
dVYTIFDTEVESTsPk	B5DEY3	N-Term(iTRAQ4plex); S14(Phospho); K16(iT
qmxMsPPPGNAGPVIMSL	O08729	N-Term(iTRAQ4plex); M2(Oxidation); X3(P);
ssTPLPTVSSSAENTR	Q62733	N-Term(iTRAQ4plex); S2(Phospho)
gcVAsPVEGGR	D4A702	N-Term(iTRAQ4plex); C2(Carbamidomethyl);
kSsLPNSk	M0RB22	N-Term(iTRAQ4plex); K1(iTRAQ4plex); S3(P
akPsPAPsPTISAPDASGP	A0A0G2JY69	N-Term(iTRAQ4plex); K2(iTRAQ4plex); S4(P
sDQEAkPSTEDLGDkk	Q5I0H3	N-Term(Acetyl); S1(Phospho); K6(iTRAQ4ple
nLGsINTELQDVQR	Q4KM74	N-Term(iTRAQ4plex); S4(Phospho)
vAsVEEGDESLDSPGSQS	D4AAX6	N-Term(iTRAQ4plex); S3(Phospho)
rAsPsPPPk	B2RYB3;A0A0G2K4F6	N-Term(iTRAQ4plex); S3(Phospho); S5(Phos
tDDVsEktsLADQEEVR	D3ZJK8	N-Term(iTRAQ4plex); S5(Phospho); K7(iTRA
eRsPPLTPk	F1M9F9	N-Term(iTRAQ4plex); S3(Phospho); K9(iTRA
gGGSGGGDEsEGEEVDE	A0A0G2JUX5	N-Term(iTRAQ4plex); S10(Phospho)
sVsASYQADAk	A0A0G2JU83	N-Term(iTRAQ4plex); S3(Phospho); K11(iTR
sATTTGsPGTEk	A0A140TAA3	N-Term(iTRAQ4plex); S7(Phospho); K12(iTR
kQNLLAsR	A0A0G2JXK1	N-Term(iTRAQ4plex); K1(iTRAQ4plex); S7(P
tPtPEPAEVETR	Q3SWT7	N-Term(iTRAQ4plex); T3(Phospho)
dGSGyIDENELDALLk	P47728	N-Term(iTRAQ4plex); Y5(Phospho); K16(iTR
iEsYEGR	Q5U205	N-Term(iTRAQ4plex); S3(Phospho)
yDERPGPsPLPHR	B2RZ74	N-Term(iTRAQ4plex); S8(Phospho)
lPTPQPYPsPQDcVEATVc	D3ZF03	N-Term(iTRAQ4plex); S9(Phospho); C13(Ca
mAPTPIPTRSPsDssTAST	Q07266;A0A0H2UHL9	N-Term(iTRAQ4plex); S12(Phospho); S14(Ph
nQEDEEEISTsPGVSEFVS	Q6AYB6	N-Term(iTRAQ4plex); S11(Phospho); C25(Ca

Information Classification: General

tsPEPQREDASPAPGTTA	A0A0G2K7C1	N-Term(iTRAQ4plex); S2(Phospho); M22(Ox
kEtEsEAEDDNLDDLER	B2RYB3	K1(iTRAQ4plex); N-Term(iTRAQ4plex); T3(P
dLSSsPPGPYGQEMYVFF	A0A0G2JVK3	N-Term(iTRAQ4plex); S5(Phospho)
tQEScGIAPLtPsQSPkPEV	D3ZVM5	N-Term(iTRAQ4plex); C5(Carbamidomethyl);
tEEVLSPDGsPSk	Q62847;A0A0G2JW28	N-Term(iTRAQ4plex); S10(Phospho); K13(iT
sFQEILQIVsPAR	D3ZKH2	N-Term(iTRAQ4plex); S10(Phospho)
vAVVRtPPk	D4A1Q2	N-Term(iTRAQ4plex); T6(Phospho); K9(iTRA
sEEETsPLVTHQNR	A0A0F7R5I4	N-Term(iTRAQ4plex); S6(Phospho)
qNsLEYMDQNDDR	D3ZEI4	N-Term(iTRAQ4plex); S3(Phospho)
sGSRPQsPSGDAESR	D4A5J1	N-Term(iTRAQ4plex); S7(Phospho)
iADLQsPLFR	D4A1D3	N-Term(iTRAQ4plex); S6(Phospho)
kGPRtPsPPPPILEDIILGk	E9PSN4	N-Term(iTRAQ4plex); K1(iTRAQ4plex); T5(P
yDIPAtAATATSPTPNPVP	D3ZN95	N-Term(iTRAQ4plex); T6(Phospho); K27(iTR
vSVTVDYIRPAsPATETVF	D4A8Y5	N-Term(iTRAQ4plex); S12(Phospho)
gMIIEHEGDRPASktEIEMI	M0R3V7	N-Term(iTRAQ4plex); K14(iTRAQ4plex); T15
sSsIsSIDkDSk	D3ZU84	N-Term(iTRAQ4plex); S3(Phospho); S5(Phos
hMsEsPNRk	G3V6Y9	N-Term(iTRAQ4plex); S3(Phospho); S5(Phos
sETMVNAQQtPLGtPk	Q920Q0	N-Term(iTRAQ4plex); T10(Phospho); T14(Ph
sETAPAAPAAPAPVEk	M0R7B4	N-Term(Acetyl); S1(Phospho); K16(iTRAQ4p
eTAVQEGSSsPVADIR	Q5U2M8	N-Term(iTRAQ4plex); S10(Phospho)
sNSLDDSTEEVQSMDVsF	D4A4L4	N-Term(iTRAQ4plex); S17(Phospho); K21(iT
gEDVNRtLEGGR	P62775	N-Term(iTRAQ4plex); T7(Phospho)
skFDsDDEDEDAENLEAV	F1MAQ8	N-Term(iTRAQ4plex); K2(iTRAQ4plex); S5(P
fFDsEDEDFEILSL	Q4KM36	N-Term(iTRAQ4plex); S4(Phospho)
aVGTPGGNSGGAGPGIS	Q6MG48	N-Term(iTRAQ4plex); M19(Oxidation); S25(F
aAASGPASASAPAAEILLt	Q5PQL7	N-Term(iTRAQ4plex); T19(Phospho)
gTGDcsDEEVDGk	G3V6P7	N-Term(iTRAQ4plex); C5(Carbamidomethyl);
eLEkPIQSkPQsPVIQATA(	Q7TSU1	N-Term(iTRAQ4plex); K4(iTRAQ4plex); K9(iT
kLScsLEDLR	Q5UDQ9	K1(iTRAQ4plex); N-Term(iTRAQ4plex); C4(C
nIIEATHMYDNVAEAcSTT	A0A0G2KB46	N-Term(iTRAQ4plex); C16(Carbamidomethyl
gIsALLLNQGDGDRk	A0A0G2JZK7	N-Term(iTRAQ4plex); S3(Phospho); K15(iTR
gPAGEASAsPPVR	D3ZMX6	N-Term(iTRAQ4plex); S9(Phospho)
qPLTsPGSVSSSR	D4A559	N-Term(iTRAQ4plex); S5(Phospho)
sSTYTkLsDPAVcGDR	B5DF41	N-Term(iTRAQ4plex); K6(iTRAQ4plex); S8(P
nRVPsAGDVER	Q3KRD5	N-Term(iTRAQ4plex); S5(Phospho)
sSLQEPSsPGQSR	A0A0G2JYB3	N-Term(iTRAQ4plex); S8(Phospho)
kLDPAQSAsR	P16086	N-Term(iTRAQ4plex); K1(iTRAQ4plex); S9(P
vcMLLSTAVYImGtFGATV	A0A0G2K2E7	N-Term(iTRAQ4plex); C2(Carbamidomethyl);
esSPFINSTDTEk	D3ZGI9	N-Term(iTRAQ4plex); S2(Phospho); K13(iTR
yLLSQSsPAPLTAAEEELF	Q5RJR2	N-Term(iTRAQ4plex); S7(Phospho)
sGEDEQQEQTIAEDLVVT	A0A0G2JVS2	N-Term(Acetyl); S1(Phospho); K19(iTRAQ4p
eWGGtESPR	F1LSB5	N-Term(iTRAQ4plex); T5(Phospho)
kDPQDmEGEkSPAsPFA(	A0A0U1RRX4;F1MAQ5	N-Term(iTRAQ4plex); K1(iTRAQ4plex); M6(C
iYHLPDAEsDEDEDFkEQ	Q91Y81	N-Term(iTRAQ4plex); S9(Phospho); K16(iTR
qISEDVDGPDNHEsPENW	A0A0G2K0B4	N-Term(iTRAQ4plex); S14(Phospho)
aAAASAAEAGIAtPGTEDS	Q9ESH1	N-Term(iTRAQ4plex); T13(Phospho); K25(iT
sPSQSsLPAVVVETFSAT	Q5HZA7	N-Term(iTRAQ4plex); S6(Phospho)
nFLNALtSPIEYQR	F1M0Z1	N-Term(iTRAQ4plex); T7(Phospho)
eSSESTNttIEDEDTkVR	P11275	N-Term(iTRAQ4plex); T8(Phospho); T9(Phos
sPsQESIGAR	D4A9T0	N-Term(iTRAQ4plex); S3(Phospho)
rEESPMDVDQPsPSAQDT	A0A0G2JVW5	N-Term(iTRAQ4plex); S12(Phospho); K32(iT
rPAsPsSPEHLPATPAESF	M0RBT5	N-Term(iTRAQ4plex); S4(Phospho); S6(Phos
lGNPYcsPTLVR	D3ZWT6	N-Term(iTRAQ4plex); C6(Carbamidomethyl);
lsVDYGk	Q5XIF6;P68370	N-Term(iTRAQ4plex); S2(Phospho); K7(iTRA
sATLsSTDSTVSEMQDEV	Q5QD51	N-Term(iTRAQ4plex); S5(Phospho); K19(iTR

gLATGDsPLGPmTHHGEI	O88794	N-Term(iTRAQ4plex); S7(Phospho); M12(Ox
aEEEDLAAVPFLAsDNEE	D4ACN3	N-Term(iTRAQ4plex); S14(Phospho); K23(iT
aLEsPERPFLAILGGAk	P16617	N-Term(iTRAQ4plex); S4(Phospho); K17(iTR
sPEQVksPAkEEAk	F1LRZ7	N-Term(iTRAQ4plex); S1(Phospho); K6(iTRA
rGsETEQLLTPNPGYGTQ	D3ZRF9	N-Term(iTRAQ4plex); S3(Phospho); T27(Pho
aIHEQAEsPR	Q5PQS2	N-Term(iTRAQ4plex); S8(Phospho)
sAADsISESVPVGPk	D3ZVQ0	N-Term(iTRAQ4plex); S5(Phospho); K15(iTR
lAAGAEsPQPASGNsPSE	F1LMV8	N-Term(iTRAQ4plex); S7(Phospho); S15(Pho
lQIsSPPFDQPMSR	G3V6J5	N-Term(iTRAQ4plex); S4(Phospho)
tPTkSsPQPAAAAR	D3ZL45	N-Term(iTRAQ4plex); K4(iTRAQ4plex); S6(P
aDGIPNDSsDSEMEDR	D3ZIV8	N-Term(iTRAQ4plex); S9(Phospho)
sEsPEPGYVVTSSGLLLPY	P0C5Y8	N-Term(iTRAQ4plex); S3(Phospho)
sAGPQSQLLASVIAEksR	F8WFS9	N-Term(iTRAQ4plex); K16(iTRAQ4plex); S17
qQEEAQAAAAAAsAESR	A0A0G2K207	N-Term(iTRAQ4plex); S13(Phospho)
sLSFPILNPALSQSNQPsP	D4A3V4	N-Term(iTRAQ4plex); S18(Phospho)
gIkDDEHIVLEPGDLFPPF	F1LUG5	N-Term(iTRAQ4plex); K3(iTRAQ4plex); S20(
dENERsSIsPmDEPVPDS	P15205;F1LRL9	N-Term(iTRAQ4plex); S6(Phospho); S9(Phos
vIsPSEDR	F1M2P8	N-Term(iTRAQ4plex); S3(Phospho)
lAEMYGGGEsDkDA	Q8R5H2	N-Term(iTRAQ4plex); S10(Phospho); K12(iT
iPAkTtPsPk	D4A1Q2	N-Term(iTRAQ4plex); K4(iTRAQ4plex); T6(P
rEQVEDSGAEsSPk	Q63714	N-Term(iTRAQ4plex); S11(Phospho); K14(iT
eYTMsWMk	A0A0G2JWM2	N-Term(iTRAQ4plex); S5(Phospho); K8(iTRA
iEDVGsDEEDDSGk	P34058	N-Term(iTRAQ4plex); S6(Phospho); K14(iTR
tSAsPPLEkSGDEGsEDEA	A0A0G2K7X3	N-Term(iTRAQ4plex); S4(Phospho); K9(iTRA
kASSsDDEGGPR	Q5QD51	N-Term(iTRAQ4plex); K1(iTRAQ4plex); S5(P
sVPTIDsGNEDDDSSFk	A0A0H2UHV4	N-Term(iTRAQ4plex); S7(Phospho); K17(iTR
asGEMASAQYITAALR	A0A0G2JVV5	N-Term(iTRAQ4plex); S2(Phospho)
tGEPDEEEGtFRSSIR	O08589	N-Term(iTRAQ4plex); T10(Phospho)
rLDsSGER	D3ZLS5	N-Term(iTRAQ4plex); S4(Phospho)
mERPSISVIsPTsPGALk	A0A0G2KAV8	N-Term(iTRAQ4plex); S10(Phospho); S13(Ph
kEVAkEsPk	P12839	K1(iTRAQ4plex); N-Term(iTRAQ4plex); K5(iT
dSEGRNEDAsFPSTPEPV	F1LQN3	N-Term(iTRAQ4plex); S10(Phospho); K19(iT
aALSATIFSEGQTLDsPQN	Q5HZE4	N-Term(iTRAQ4plex); S16(Phospho)
eTMSGQTAFLGsPESR	A0A0G2JV78	N-Term(iTRAQ4plex); S12(Phospho)
dLDVAVLVGsMPR	O88989	N-Term(iTRAQ4plex); S10(Phospho)
aQPAPPELNSESEDYsPS	O08875	N-Term(iTRAQ4plex); S16(Phospho)
tSLPDAPVGIsSESPSDPR	D4A1J3	N-Term(iTRAQ4plex); S11(Phospho)
sPAEAksPVEVk	F1LRZ7	N-Term(iTRAQ4plex); K6(iTRAQ4plex); S7(P
gkPVPVHGsRk	P13233	N-Term(iTRAQ4plex); K2(iTRAQ4plex); S9(P
sPEsPsSPVSSETTSSFk	Q5QD51	N-Term(iTRAQ4plex); S4(Phospho); S6(Phos
sPEPcPGQQAPGPEPSQ	Q5XIB5	N-Term(iTRAQ4plex); C5(Carbamidomethyl);
dSAPTTPTsPTEFLTPGLF	D4A1D3	N-Term(iTRAQ4plex); S9(Phospho)
qsPALSk	F1LYQ8	N-Term(iTRAQ4plex); S2(Phospho); K7(iTRA
eVHDELEDLPsPPPPLsPF	M0R423	N-Term(iTRAQ4plex); S11(Phospho); S17(Ph
iVHSsPSSDGk	D3ZHG4	N-Term(iTRAQ4plex); S5(Phospho); K11(iTR
gRGsPASGALEPTk	F1LXF1	N-Term(iTRAQ4plex); S4(Phospho); K14(iTR
sSDASsEsVQtVSQIEVES	A0A0G2JZ50	N-Term(iTRAQ4plex); S6(Phospho); S8(Phos
aDSEGDLsENDDGAGDLI	Q63014	N-Term(iTRAQ4plex); S8(Phospho)
rsPSADQSLDDSPSk	F1M6X3	N-Term(iTRAQ4plex); S2(Phospho); K15(iTR
sGSGYEGsTSWk	Q0ZCA7	N-Term(iTRAQ4plex); S8(Phospho); K12(iTR
nILVQtPESESPQSR	Q91ZY8	N-Term(iTRAQ4plex); T6(Phospho)
lPHTGPADDGATRsPSIDS	F1M2K6	N-Term(iTRAQ4plex); S14(Phospho); K22(iT
dGAAVAAVSADRDsPPcS	Q80XF7	N-Term(iTRAQ4plex); S14(Phospho); C17(Ca
lSDIsPIGR	A0A0G2KA12	N-Term(iTRAQ4plex); S5(Phospho)
sAVRPsPsPER	A0A0G2K2M9	N-Term(iTRAQ4plex); S6(Phospho); S8(Phos

Information Classification: General

qQTQQVQSPVDSATMsP	A0A0G2JTA7	N-Term(iTRAQ4plex); S16(Phospho)
vEsEESGDEEGk	Q5HZV9	N-Term(iTRAQ4plex); S3(Phospho); K12(iTR
qSNAssDVEAEEk	Q566D6	N-Term(iTRAQ4plex); S5(Phospho); S6(Phos
lEPVsPPsPPHADPELELT	A0A0G2JU91	N-Term(iTRAQ4plex); S5(Phospho); S8(Phos
eWSNsLEk	D3Z9G0	N-Term(iTRAQ4plex); S5(Phospho); K8(iTRA
wSsQGMLR	D3ZC55;D3ZVM5	N-Term(iTRAQ4plex); S3(Phospho)
lHFFmPGFAPLTsR	P85108;B4F7C2;P69897;(	N-Term(iTRAQ4plex); M5(Oxidation); S13(Ph
aGsPLcNSNLQDSEEDVE	A0A0G2KB46	N-Term(iTRAQ4plex); S3(Phospho); C6(Carb
lTSscPDLPSQSDk	Q9Z158	N-Term(iTRAQ4plex); S4(Phospho); C5(Carb
sVEQsPPR	M0R4J7	N-Term(iTRAQ4plex); S5(Phospho)
aEEkEPLTEkPkDsPGEAk	F1LRZ7	N-Term(iTRAQ4plex); K4(iTRAQ4plex); K10(
sRNsPLLDR	O08679	N-Term(iTRAQ4plex); S4(Phospho)
tDPAPMAPPPRGEEEEEE	D4A507	N-Term(iTRAQ4plex); S29(Phospho)
dAsPPEPASPTIGLDk	F1LN57	N-Term(iTRAQ4plex); S3(Phospho); K16(iTR
sPATVksPVEAk	F1LRZ7	N-Term(iTRAQ4plex); S1(Phospho); K6(iTRA
aSQPGPTAESQSsPHR	A0A0G2K162	N-Term(iTRAQ4plex); S13(Phospho)
sGYHDDsDEDLLE	O54861	N-Term(iTRAQ4plex); S7(Phospho)
acLsPSQLk	Q5BJP9	N-Term(Acetyl); C2(Carbamidomethyl); S4(Pl
eTISAIDTsPk	D4A4M0	N-Term(iTRAQ4plex); S9(Phospho); K11(iTR
tDDVsEkTsLADQEEVR	D3ZJK8	N-Term(iTRAQ4plex); S5(Phospho); K7(iTRA
vSLsPHSANPk	Q5BJV5	N-Term(iTRAQ4plex); S4(Phospho); K11(iTR
ssGREEDEEELLR	A0A0G2JW01	N-Term(iTRAQ4plex); S2(Phospho)
hEGLAETPETsPESLSFsF	A0A0G2K6R9;F1M9N9	N-Term(iTRAQ4plex); S11(Phospho); S18(Ph
sSsVGSSSSYPISSAVPR	A0A0G2K1J5	N-Term(iTRAQ4plex); S3(Phospho)
sSsPVNVk	Q63555	N-Term(Acetyl); S3(Phospho); K8(iTRAQ4ple
eFGFLPTtPSEQR	A0A0G2K6Y9	N-Term(iTRAQ4plex); T8(Phospho)
tEEVLSPDGsPSksPSk	Q62847;A0A0G2JW28	N-Term(iTRAQ4plex); S10(Phospho); K13(iT
aVPAtPPRtGSPLTVATGN	F1M1Y0	N-Term(iTRAQ4plex); T5(Phospho); T9(Phos
eVsPMSAPNMPSIER	Q923K6	N-Term(iTRAQ4plex); S3(Phospho)
sAAEELELTVLk	D4A739	N-Term(iTRAQ4plex); S1(Phospho); K12(iTR
eSsEsTNTTIEDEDTk	P11275	N-Term(iTRAQ4plex); S3(Phospho); S5(Phos
dATDVPPPPcQtPAGPETI	B0BN02	N-Term(iTRAQ4plex); C10(Carbamidomethyl
sHSsPsLNPDAsPVTAk	D4A404	N-Term(iTRAQ4plex); S4(Phospho); S6(Phos
sLGQNPTEAELQDMINEV	P62161	N-Term(iTRAQ4plex); T25(Phospho)
mGPSSSPIPSPsPsPTDS	P63319	N-Term(iTRAQ4plex); M1(Oxidation); S12(Ph
tPGLSQSLALsSDESLDMI	O35867	N-Term(iTRAQ4plex); S11(Phospho); K31(iT
yRsPYSGPk	Q5BJP4	N-Term(iTRAQ4plex); S3(Phospho); K9(iTRA
ePsLHEIGEk	M0RBB2	N-Term(iTRAQ4plex); S3(Phospho); K10(iTR
sNVASTAAcSSAsSLGLGI	P23565;G3V8Q2	N-Term(iTRAQ4plex); C9(Carbamidomethyl);
dMPHPLAGSSSEEAVGG	D4A060	N-Term(iTRAQ4plex); S19(Phospho); S22(Ph
isDsESEELPkPR	Q3SWT4	N-Term(iTRAQ4plex); S2(Phospho); S4(Phos
sHkDDsEIDFSALcPk	A0A0G2JUQ3	N-Term(iTRAQ4plex); K3(iTRAQ4plex); S6(P
lkSEADtLPR	D3ZEI4	N-Term(iTRAQ4plex); K2(iTRAQ4plex); T7(P
lDtFcGSPPYAAPELFQGk	F1M836;O08679;A0A0G2	N-Term(iTRAQ4plex); T3(Phospho); C5(Carb
sNtPMGDkDDDDDDDADI	A0A0G2JU89	N-Term(iTRAQ4plex); T3(Phospho); K8(iTRA
eDAHADPEIQPMkEDDGT	Q6QRP0	N-Term(iTRAQ4plex); K13(iTRAQ4plex); S23
kAsPEPEGETAGk	D4AAZ8	N-Term(iTRAQ4plex); K1(iTRAQ4plex); S3(P
lSRsPLk	G3V874;A0A0G2K1Q9	N-Term(iTRAQ4plex); S4(Phospho); K7(iTRA
eVQsPEQVk	B1WC16	N-Term(iTRAQ4plex); S4(Phospho); K9(iTRA
gPkPEPPGSGsPAPPR	F1M4M5	N-Term(iTRAQ4plex); K3(iTRAQ4plex); S11(
tsmGGTQQQFVEGVR	A0A0G2JT93	N-Term(iTRAQ4plex); S2(Phospho); M3(Oxic
eTsPTRGEPVGGQk	P34926	N-Term(iTRAQ4plex); S3(Phospho); K14(iTR
gDAASAsPSSTPLIR	Q6MG76	N-Term(iTRAQ4plex); S7(Phospho)
gGsLETPSk	M0R3U3	N-Term(iTRAQ4plex); S3(Phospho); K9(iTRA
tSPLNAEVVQYAk	B1WBR8	N-Term(iTRAQ4plex); T1(Phospho); K13(iTR

Information Classification: General

tESVTSGPLSPEGSPskSF	F8WFS9	N-Term(iTRAQ4plex); S16(Phospho); K17(iT
dFQDYIEPEEGcQGsPQR	A0A096MIY2	N-Term(iTRAQ4plex); C12(Carbamidomethyl
dSScQTSNSTEktPsPPEP	Q3ZB98	N-Term(iTRAQ4plex); C4(Carbamidomethyl);
sPDEATAADQEsEDDLSA	F1LYQ8	N-Term(iTRAQ4plex); S12(Phospho)
sRtAsGSSVTSLEGTR	Q6JE36	N-Term(iTRAQ4plex); T3(Phospho); S5(Phos
qIcVVMLETLSQsPPk	Q4V8F6	N-Term(iTRAQ4plex); C3(Carbamidomethyl);
eGSIGGSGGsGGGGGGG	D3ZCL8	N-Term(iTRAQ4plex); S10(Phospho); K19(iT
iLLPNsSQDELMEVEk	A0A0G2K2M9	N-Term(iTRAQ4plex); S6(Phospho); K16(iTR
aGFQTEDFSLYAcAsPk	F1M9C3	N-Term(iTRAQ4plex); C13(Carbamidomethyl
nDSIVtPsPPQAR	A0A0G2KAL9	N-Term(iTRAQ4plex); T6(Phospho); S8(Phos
sGsFGk	B2GV74	N-Term(iTRAQ4plex); S3(Phospho); K6(iTRA
vPSAPGQEsPVPDTESTA	P34926	N-Term(iTRAQ4plex); S9(Phospho); M20(Ox
sINHQIEsPGER	F1M392	N-Term(iTRAQ4plex); S8(Phospho)
aAADPFDTssGSVQLIAIkF	F1LXC7	N-Term(iTRAQ4plex); S9(Phospho); S10(Pho
vQPEIEELDPsPSMPGQQ	P47861	N-Term(iTRAQ4plex); S11(Phospho); K22(iT
aTENDIANFFsPLNPIR	D4ABK7	N-Term(iTRAQ4plex); S11(Phospho)
ssFSTNVVYTTPGTR	D4A3N4	N-Term(iTRAQ4plex); S2(Phospho)
eNEMDENLEQVsGIIGNLF	P60881	N-Term(iTRAQ4plex); S12(Phospho)
aVPIAVADEGEsESEDDD	D3ZLQ8	N-Term(iTRAQ4plex); S12(Phospho); K20(iT
vTQILQsPAFR	Q62847;A0A0G2JW28	N-Term(iTRAQ4plex); S7(Phospho)
lANHEEGsDEEEEETPk	Q66HA5	N-Term(iTRAQ4plex); S8(Phospho); K17(iTR
iLQIsPEGPLQGR	A0A0G2K939	N-Term(iTRAQ4plex); S5(Phospho)
aLDISAsDDEMARPk	G3V9E4	N-Term(iTRAQ4plex); S7(Phospho); K15(iTR
eVsPEVVR	E9PSN4	N-Term(iTRAQ4plex); S3(Phospho)
gGLsPANDTGAk	Q8K1Q0	N-Term(iTRAQ4plex); S4(Phospho); K12(iTR
dVATSPIsPTENNTTPPDA	P35465	N-Term(iTRAQ4plex); S8(Phospho)
sPVTVk	F1LRZ7	N-Term(iTRAQ4plex); S1(Phospho); K6(iTRA
iDEPDTPYHNMIGDDEDV	A0A0H2UHA0	N-Term(iTRAQ4plex); C19(Carbamidomethyl
msGSSGTPyLGSkISLISk	A0A0G2JZY0	N-Term(Acetyl); S2(Phospho); Y9(Phospho);
tAAPsPDRR	F1LRY7	N-Term(iTRAQ4plex); S5(Phospho)
sNsDESNFSEk	Q9QYN5	N-Term(iTRAQ4plex); S3(Phospho); K11(iTR
tLVLSsSPTSPTQEPLAAG	A0A0G2K1B4	N-Term(iTRAQ4plex); S6(Phospho); K20(iTR
gRPPAEkLsPNPPNLTk	G3V790	N-Term(iTRAQ4plex); K7(iTRAQ4plex); S9(P
qNsATESAESIEIYIPEAQT	G3V849	N-Term(iTRAQ4plex); S3(Phospho)
eSsESTNTTIEDEDTk	P11275	N-Term(iTRAQ4plex); S3(Phospho); K16(iTR
rLEDEQPPtLsPk	A0A0G2K104	N-Term(iTRAQ4plex); T9(Phospho); S11(Pho
eLALssPEDLTQDFEELk	P34926;Q63330	N-Term(iTRAQ4plex); S5(Phospho); S6(Phos
gGSISVQVNSVkFDsE	F1LPS3	N-Term(iTRAQ4plex); K12(iTRAQ4plex); S15
iSENYsDksDVENADESSS	Q9JI66;A0A0H2UHB7	N-Term(iTRAQ4plex); S6(Phospho); K8(iTRA
sRVsVsPGR	B2RYB3;A0A0G2K4F6	N-Term(iTRAQ4plex); S4(Phospho); S6(Phos
sLsPGkENINSLEVEk	D3ZAY8	N-Term(iTRAQ4plex); S3(Phospho); K6(iTRA
yLGQGLQYGsFTDLR	G3V984	N-Term(iTRAQ4plex); S10(Phospho)
fPEFcSsPSPPVEVk	D3ZUT9	N-Term(iTRAQ4plex); C5(Carbamidomethyl);
aAAAAAAAGDsDSWDAD	A0JPM9	N-Term(Acetyl); S11(Phospho); M21(Oxidatio
sGDEMIFDPTMSk	Q6P685	N-Term(Acetyl); S1(Phospho); K13(iTRAQ4p
eLEAERsLMssPEDLTk	P15205;F1LRL9	N-Term(iTRAQ4plex); S7(Phospho); S10(Pho
rGsAGGVGDR	M0RAT6	N-Term(iTRAQ4plex); S3(Phospho)
sksAPTSPcDQELk	F1LMV8	N-Term(iTRAQ4plex); K2(iTRAQ4plex); S3(P
sSscGDAELLGQATLPVG	Q5U2P5;A0A0G2K2V4	N-Term(iTRAQ4plex); S3(Phospho); C4(Carb
gPGQMTTNADTDGDsYkI	F8WFS9	N-Term(iTRAQ4plex); S15(Phospho); K17(iT
tPsPPEPEPAGTAQk	Q3ZB98	N-Term(iTRAQ4plex); S3(Phospho); K15(iTR
tPsPPEEAsPLPsPTASPN	A0A0G2K5Z4	N-Term(iTRAQ4plex); S3(Phospho); S9(Phos
sQTEEDcTEELFDFLHAR	Q5M9I5	N-Term(iTRAQ4plex); S1(Phospho); C7(Carb
gIPLPTGDTsPEPELLPGD	Q5RK09	N-Term(iTRAQ4plex); S10(Phospho); K25(iT
sPFNSPsPQDSPR	O70188	N-Term(iTRAQ4plex); S7(Phospho)

eLEDEEENsDEDELDSHT	B1WBQ5	N-Term(iTRAQ4plex); S9(Phospho); K21(iTR
lLDPEDIsVDHPDEk	G3V6S0	N-Term(iTRAQ4plex); S8(Phospho); K15(iTR
aAAAAAAAGDsDSWDAD	A0JPM9	N-Term(Acetyl); S11(Phospho); K27(iTRAQ4
aAVGLTGNDIAtPPNkELP	A0A0G2JW88	N-Term(iTRAQ4plex); T12(Phospho); K16(iT
nAsVPNLR	Q5XII9	N-Term(iTRAQ4plex); S3(Phospho)
hDsPEDVkR	O35814	N-Term(iTRAQ4plex); S3(Phospho); K8(iTRA
sFSIsPVR	E9PTE1	N-Term(iTRAQ4plex); S5(Phospho)
mLPHAPGVQMQAIPEDA	Q99PA1	N-Term(iTRAQ4plex); S22(Phospho); K32(iT
aEASsPVPYLSPETNPAS	F1M4A0	N-Term(iTRAQ4plex); S5(Phospho); K24(iTR
eTAEDREsPVSEEIk	D3ZWM3	N-Term(iTRAQ4plex); S8(Phospho); K15(iTR
aAFDDAIAELDTLsEESYk	P62260	N-Term(iTRAQ4plex); S14(Phospho); K19(iT
rLsNVsLTGLGTVR	A0A0G2K928	N-Term(iTRAQ4plex); S3(Phospho); S6(Phos
sSSELsPEIVEk	A0A0G2K2M9	N-Term(iTRAQ4plex); S6(Phospho); K12(iTR
mFPEPTNsGDEGEPELD	Q5XII5	N-Term(iTRAQ4plex); S8(Phospho)
dQPAALPLAAEEtVNLPPS	F1MAQ5	N-Term(iTRAQ4plex); T13(Phospho); K37(iT
eGSVSIVGAVsPPGGDFS	D4A133	N-Term(iTRAQ4plex); S11(Phospho); K37(iT
tTPsPk	D4A1Q2	N-Term(iTRAQ4plex); S4(Phospho); K6(iTRA
qSNVEDsPEkTcSIPIPTIIV	A0A0G2K7H2	N-Term(iTRAQ4plex); S7(Phospho); K10(iTR
rsPGAIk	F1M1Y0	N-Term(iTRAQ4plex); S2(Phospho); K7(iTRA
mNcsPTSQIDNIGEEEMD	F1M6A8	N-Term(iTRAQ4plex); C3(Carbamidomethyl);
eVLDAsDkEGLsPAkR	Q5RKH1	N-Term(iTRAQ4plex); S6(Phospho); K8(iTRA
lQEEGGsEEEEVGSPSED	D4A1H8	N-Term(iTRAQ4plex); S7(Phospho)
aEDDscHLAPVSIPEPHR	P34926;Q63330	N-Term(iTRAQ4plex); S5(Phospho); C6(Carb
hGGGIVADLSQQsLkDGV	Q5HZV9	N-Term(iTRAQ4plex); S13(Phospho); K15(iT
wAHDkFsGEEGEIEDDEs	Q5M7V8	N-Term(iTRAQ4plex); K5(iTRAQ4plex); S7(P
lsWPESEGkPR	Q8CGU4	N-Term(iTRAQ4plex); S2(Phospho); K9(iTRA
gRLtPsPDIIVLSDNEASSP	Q4V8E1	N-Term(iTRAQ4plex); T4(Phospho); S6(Phos
sPATVksPGEAksPAEAk	F1LRZ7	N-Term(iTRAQ4plex); K6(iTRAQ4plex); S7(P
dSLtSPEDELGAEVGDEA	D3ZT47	N-Term(iTRAQ4plex); T4(Phospho); K21(iTR
qAQsPGGVSSEVEVLk	F1LSE6	N-Term(iTRAQ4plex); S4(Phospho); K16(iTR
qTLPSsPTTTk	D4AAF8	N-Term(iTRAQ4plex); S6(Phospho); K11(iTR
eVSADLsEEENLLTDFk	F1LMY3	N-Term(iTRAQ4plex); S7(Phospho); K17(iTR
aRsDDYLSR	F1M2D4	N-Term(iTRAQ4plex); S3(Phospho)
dLkGPSsQEDESGGIEDS	A0A0G2K0F3	N-Term(iTRAQ4plex); K3(iTRAQ4plex); S7(P
dYNASASTISPPssmEEDk	P15205;F1LRL9	N-Term(iTRAQ4plex); S13(Phospho); S14(Ph
sPDRTEEVLSPDGsPSk	Q62847;A0A0G2JW28	N-Term(iTRAQ4plex); S14(Phospho); K17(iT
aGGPASGTGSATsGGTV	Q80XF7	N-Term(iTRAQ4plex); S13(Phospho)
kGGQAVDSGIPLLsVSDP	P0C1X8	N-Term(iTRAQ4plex); K1(iTRAQ4plex); S14(
dSsTSPGDYVLSVSENSR	Q63768	N-Term(iTRAQ4plex); S3(Phospho)
kIVEPEVVGEsDsEVEGD	D4ACM9	K1(iTRAQ4plex); N-Term(iTRAQ4plex); S11(
kLEGNsPQGsSHGVk	Q5M970	N-Term(iTRAQ4plex); K1(iTRAQ4plex); S6(P
dTIELcTAEsIDNLR	D3ZFC5	N-Term(iTRAQ4plex); C6(Carbamidomethyl);
eEADVIPEsPLsDVGAEDI	G3V8M8	N-Term(iTRAQ4plex); S9(Phospho); S12(Pho
sRsPSTESQLASk	F8WFS9	N-Term(iTRAQ4plex); S3(Phospho); K13(iTR
tLNMTTsPEEk	A0A096MJ75	N-Term(iTRAQ4plex); S7(Phospho); K11(iTR
kGsISDGTNILDEVTFPAR	D4ABB2	N-Term(iTRAQ4plex); K1(iTRAQ4plex); S3(P
rEQDsPPmkPGVLDAIR	Q64548	N-Term(iTRAQ4plex); S5(Phospho); M8(Oxid
dSTSSAEDGEGsDGPGG	F1LN57	N-Term(iTRAQ4plex); S12(Phospho); K18(iT
lkFsDDEDEEDVVk	D3ZY45	N-Term(iTRAQ4plex); K2(iTRAQ4plex); S4(P
gIEGAQAsPLIPGAGEIINA	P16970	N-Term(iTRAQ4plex); S8(Phospho); K25(iTR
sPsTGLYANLEk	A0A0G2JWM2	N-Term(iTRAQ4plex); S3(Phospho); K12(iTR
aASAAAATAAASAATAAS	O08629	N-Term(Acetyl); S23(Phospho); S26(Phospho
sFPsRSPSQGDEDSALILT	A0A0G2K1P1	N-Term(iTRAQ4plex); S4(Phospho); K24(iTR
wLEEADSSTGsPTSIDk	P62516	N-Term(iTRAQ4plex); S11(Phospho); K17(iT
tALsDLFLEQLLR	Q5BK33	N-Term(iTRAQ4plex); S4(Phospho)

tQsLDEEQk	F1LW90	N-Term(iTRAQ4plex); S3(Phospho); K9(iTRA
sEQPLSQVLPTLsPEHk	A0A0G2K2M9	N-Term(iTRAQ4plex); S13(Phospho); K17(iT
sSLAETLDStGsLDPQR	Q9WTW8	N-Term(iTRAQ4plex); T10(Phospho); S12(Ph
kPARPNSEVLLsGSEDAD	A0A0H2UHV4	N-Term(iTRAQ4plex); K1(iTRAQ4plex); S12(
vNHALsEGLGVIAcIGEk	P48500	N-Term(iTRAQ4plex); S6(Phospho); C14(Car
mkPAMETAAEENtEQSQE	E9PSV8	N-Term(Acetyl); K2(iTRAQ4plex); T13(Phosp
eQSDISVsPR	A0A0G2JZB4	N-Term(iTRAQ4plex); S8(Phospho)
sTELVLsPDMPR	D3ZHK4	N-Term(iTRAQ4plex); S7(Phospho)
rHstEGEEGDVSDVGSR	A0A0G2K9A9	N-Term(iTRAQ4plex); S3(Phospho); T4(Phos
kQQEEAQAAAAAAsAESF	A0A0G2K207	N-Term(iTRAQ4plex); K1(iTRAQ4plex); S14(
gQHQAHsLER	P60203	N-Term(iTRAQ4plex); S7(Phospho)
dGQDAIAQsPEk	A0A0G2K904	N-Term(iTRAQ4plex); S9(Phospho); K12(iTR
qSVGSGsPVQTVGLENR	G3V8Q9	N-Term(iTRAQ4plex); S7(Phospho)
eGSsEkDEGEQEEEGETE	P12839	N-Term(iTRAQ4plex); S4(Phospho); K6(iTRA
tckDPIEDINsPEHIQR	A0A0A0MXV8	N-Term(iTRAQ4plex); C2(Carbamidomethyl);
ePNSENVDISsGGGVTGV	Q7TQ88	N-Term(iTRAQ4plex); S11(Phospho); K19(iT
gPPsPPAPVMHsPSR	D4A8G7	N-Term(iTRAQ4plex); S4(Phospho); S12(Pho
tLQPPEsPR	D3ZNS1	N-Term(iTRAQ4plex); S7(Phospho)
gPPLPPPPPLPLtTSPPPs	A0A0G2JV35	N-Term(iTRAQ4plex); T13(Phospho); S19(Ph
gSEGYLAATYPAVGQTsP	A0A0G2JWL3	N-Term(iTRAQ4plex); S17(Phospho)
aPsPPWPQGPLSPGTGS	D4A352	N-Term(iTRAQ4plex); S3(Phospho)
nEkHsPVsPSAk	A0A0G2K6R9;F1M9N9	N-Term(iTRAQ4plex); K3(iTRAQ4plex); S5(P
lEQEQEGEkDAATEEPGs	F8WFS9	N-Term(iTRAQ4plex); K9(iTRAQ4plex); S18(
gFcLIPQQsINEAIR	M0R5P7	N-Term(iTRAQ4plex); C3(Carbamidomethyl);
kAEAAAAPAVAPGPAQP(	A1L1I3	N-Term(iTRAQ4plex); K1(iTRAQ4plex); T27(
eEAksPEk	F1LRZ7	N-Term(iTRAQ4plex); K4(iTRAQ4plex); S5(P
lDNEsPEPDTR	D3ZK16	N-Term(iTRAQ4plex); S5(Phospho)
mPsIPEEPEHGDLER	Q63638	N-Term(iTRAQ4plex); S3(Phospho)
ePTPSIAsDIsLPIATQELR	D3ZJ32	N-Term(iTRAQ4plex); S8(Phospho); S11(Pho
dSLGDFIEHYAQLGPSsPI	A0A0G2JUX5	N-Term(iTRAQ4plex); S17(Phospho)
lSDGEYIsDGEEGEDAITG	A0A0G2K1R9	N-Term(iTRAQ4plex); S8(Phospho); K23(iTR
gPPsPPAPVMHSPSR	D4A8G7	N-Term(iTRAQ4plex); S4(Phospho)
lAAGAESPQPASGNsPSE	F1LMV8	N-Term(iTRAQ4plex); S15(Phospho)
tLsPTPSAEGFQDGR	A0A0G2JW01;F1LWK7	N-Term(iTRAQ4plex); S3(Phospho)
sVQPEVELSGGSGsGGD	F1M031	N-Term(iTRAQ4plex); S14(Phospho)
vSRPGSPGSEVPAAsVtR	D3ZMM2	N-Term(iTRAQ4plex); S15(Phospho); T17(Ph
lNIPVsQVNPR	P06686;P06687	N-Term(iTRAQ4plex); S6(Phospho)
sPAEAksPAEVksPAVAk	F1LRZ7	N-Term(iTRAQ4plex); S1(Phospho); K6(iTRA
ekFPEFcSSPsPPVEVk	D3ZUT9	N-Term(iTRAQ4plex); K2(iTRAQ4plex); C7(C
sETAPAAPAAPAPAEk	P15865	N-Term(Acetyl); S1(Phospho); K16(iTRAQ4p
eVAEVsPMSAANISIAAR	Q02294	N-Term(iTRAQ4plex); S6(Phospho)
qkIDsDDDGEkEGDEk	Q3SWT4	N-Term(iTRAQ4plex); K2(iTRAQ4plex); S5(P
sQSVsPTSFLTISHEGSEE	F1M8D5	N-Term(iTRAQ4plex); S5(Phospho); C22(Car
mTDsFTEQADQVTAEVG	P11348	N-Term(iTRAQ4plex); S4(Phospho); K18(iTR
aDsIEIYIPEAQTR	F1LSL6	N-Term(iTRAQ4plex); S3(Phospho)
gNHEcAsINR	A0A0G2JYS8	N-Term(iTRAQ4plex); C5(Carbamidomethyl);
kEEQAASPVAEDVGDVAY	D3ZYB4	N-Term(iTRAQ4plex); K1(iTRAQ4plex); S20(
sSSVsSPELk	A0A0G2K2M9	N-Term(iTRAQ4plex); S5(Phospho); K10(iTR
vkEYEEEIHsLk	D3ZCL8	N-Term(iTRAQ4plex); K2(iTRAQ4plex); S10(
vVITQsPGk	D3ZWX2	N-Term(iTRAQ4plex); S6(Phospho); K9(iTRA
rPWPQHQcsAPADLGYE(	Q80Z30	N-Term(iTRAQ4plex); C8(Carbamidomethyl);
ySDVEVPASVTGHSFASD	A0A0G2JSM7;D3ZZ99	N-Term(iTRAQ4plex); C24(Carbamidomethyl
dQFGsSHsLPEVQQHMR	Q9JKS6;D3Z9C7	N-Term(iTRAQ4plex); S5(Phospho); S8(Phos
eMQNLsQHGR	A0A0G2K1I6	N-Term(iTRAQ4plex); S6(Phospho)
sSsLGDLLR	D3Z9K4	N-Term(iTRAQ4plex); S3(Phospho)

iSVYYNEAtGGk	P69897	N-Term(iTRAQ4plex); T9(Phospho); K12(iTR
lVsQDNFGFDLPAVEAATI	G3V6S0	N-Term(iTRAQ4plex); S3(Phospho); K19(iTR
vQSQEEIHsDEEDQASEP	Q6TQE1	N-Term(iTRAQ4plex); S9(Phospho); K19(iTR
lQQPSAsPQGSYSVESR	A0A0G2KB60	N-Term(iTRAQ4plex); S7(Phospho)
gDVVTGSDAQVsVPVQAI	A0A0G2K712	N-Term(iTRAQ4plex); S12(Phospho); K27(iT
aGTLSITEFADmLSGNAG	A0A0G2K1J5	N-Term(iTRAQ4plex); M12(Oxidation); S22(P
iIYGGsVTGATck	P48500	N-Term(iTRAQ4plex); S6(Phospho); C12(Car
gPDsGAEVEk	P34926;Q63330	N-Term(iTRAQ4plex); S4(Phospho); K10(iTR
vGTITsTPNR	Q5M821	N-Term(iTRAQ4plex); S6(Phospho)
kGEGDAAPsEEk	P07936	N-Term(iTRAQ4plex); K1(iTRAQ4plex); S9(P
gFPAHEQEDEDsLEEDSQ	Q5BJV5	N-Term(iTRAQ4plex); S12(Phospho)
rAPPGPLPPAASPGPPAA	F1LRY7	N-Term(iTRAQ4plex); S19(Phospho)
qTPRsPDSTANVR	B5DEJ1	N-Term(iTRAQ4plex); S5(Phospho)
kPSLcQVPsTSSTDGAGD	F1LR18	K1(iTRAQ4plex); N-Term(iTRAQ4plex); C5(C
kLSLPtDLkPDLDVk	Q9WU70	K1(iTRAQ4plex); N-Term(iTRAQ4plex); T6(P
hyGGLTGLNk	P25113	N-Term(iTRAQ4plex); Y2(Phospho); K10(iTR
eEAAAGSGsAGEGDGk	Q5U2S7	N-Term(iTRAQ4plex); S9(Phospho); K16(iTR
sENAFDQAVDsD	Q80Z26	N-Term(iTRAQ4plex); S11(Phospho)
eISSPSsPVkVEVTVTDPV	A0A0G2K6R9;F1M9N9	N-Term(iTRAQ4plex); S7(Phospho); K10(iTR
eEVAsEPEEAAsPITPk	Q5RJN5	N-Term(iTRAQ4plex); S5(Phospho); S12(Pho
gLLSGQTsPTNAR	B1H257	N-Term(iTRAQ4plex); S8(Phospho)
kEsPPPLVPPAAR	Q4QR85	N-Term(iTRAQ4plex); K1(iTRAQ4plex); S3(P
sSQSLHcLsPR	D4ACG7	N-Term(iTRAQ4plex); C7(Carbamidomethyl);
sPVEDPDsDTMGTSVDES	Q3MID9	N-Term(iTRAQ4plex); S8(Phospho)
kYsIck	Q63327	N-Term(iTRAQ4plex); K1(iTRAQ4plex); S3(P
aEDGAAPSPSSEtPkk	F1LMW7	N-Term(iTRAQ4plex); T13(Phospho); K15(iT
nVTESPsFSAGDNPHVLY	G3V9B3	N-Term(iTRAQ4plex); S7(Phospho); K30(iTR
aLDPAPLAQPTPVGSVQT	D3ZYW8	N-Term(iTRAQ4plex); S19(Phospho)
sGkNSQEDsEDsEEkDVk	A0A0G2K7X3	N-Term(iTRAQ4plex); K3(iTRAQ4plex); S9(P
iNSIMSVVTNTLVEELEEs	O88420	N-Term(iTRAQ4plex); S19(Phospho); K22(iT
aASAALILPQsPSMk	Q68FS9	N-Term(Acetyl); S11(Phospho); K15(iTRAQ4
nIDEDELLGkLsEEELk	P70566	N-Term(iTRAQ4plex); K10(iTRAQ4plex); S12
sLVGTPYWMAPEVISR	D4A280	N-Term(iTRAQ4plex); S1(Phospho)
mGPSSSPIPSPSPsPTDS	P63319	N-Term(iTRAQ4plex); S14(Phospho); K19(iT
gPQLPTVSPSPPVMIPTV	F1LQZ9	N-Term(iTRAQ4plex); S27(Phospho)
sLSsPPSSk	O70187	N-Term(iTRAQ4plex); S4(Phospho); K9(iTRA
aPPGPLPPAASPGPPAAs	F1LRY7	N-Term(iTRAQ4plex); S18(Phospho)
sPPRPAkPRsPPIPAkPR	Q63327	N-Term(iTRAQ4plex); K7(iTRAQ4plex); S10(
lTFDSSFsPNTGk	Q9Z2L0	N-Term(iTRAQ4plex); S8(Phospho); K13(iTR
eAEEEssGGEEEDEDENI	Q07205	N-Term(iTRAQ4plex); S6(Phospho); S7(Phos
gLFGQRtPtPPR	D4AD05	N-Term(iTRAQ4plex); T7(Phospho); T9(Phos
tREQEssGEEDNDLSPEE	A0A0H2UHA0	N-Term(iTRAQ4plex); S6(Phospho); S7(Phos
iALESVGQPEELMEsDNcS	O08623	N-Term(iTRAQ4plex); S15(Phospho); C18(Ca
eGMSNNsTTsISQAR	M0R809	N-Term(iTRAQ4plex); S7(Phospho); S10(Pho
kREDsVSEVTVEDSGESL	Q5M7A4	N-Term(iTRAQ4plex); K1(iTRAQ4plex); S5(P
iGELEVGQMENIsPGQIID	A0A0G2K9S4	N-Term(iTRAQ4plex); S13(Phospho)
dVSPsPETETAk	A0A0G2JW88	N-Term(iTRAQ4plex); S5(Phospho); K12(iTR
sIDAALFR	D3ZWQ0	N-Term(iTRAQ4plex); S1(Phospho)
aSPAsPVsPSDcPSPR	F1LU97	N-Term(iTRAQ4plex); S5(Phospho); S8(Phos
lMDDNEsENNQESMPSLS	A0A0G2JSW5	N-Term(iTRAQ4plex); S7(Phospho)
qcEGITsPESSk	Q4FZT2	N-Term(iTRAQ4plex); C2(Carbamidomethyl);
sAVsPDLHITPIYEGR	F1M2K6	N-Term(iTRAQ4plex); S4(Phospho)
eEsEAEVkEDVIEk	P34926;Q63330	N-Term(iTRAQ4plex); S3(Phospho); K8(iTRA
tSPSkPSSAPALRPGPk	A0A0G2JW88	N-Term(iTRAQ4plex); T1(Phospho); K5(iTRA
rHGASAAPsPPPR	D3ZSY8	N-Term(iTRAQ4plex); S9(Phospho)

Information Classification: General

iLSsTTSk	P23565;G3V8Q2	N-Term(iTRAQ4plex); S4(Phospho); K8(iTRA
tDTAADGETSATEsDQEE	G3V874;A0A0G2K1Q9	N-Term(iTRAQ4plex); S14(Phospho); K23(iT
vksPGSGHVk	D3ZQG6	N-Term(iTRAQ4plex); K2(iTRAQ4plex); S3(P
sPVEVksPASVk	F1LRZ7	N-Term(iTRAQ4plex); K6(iTRAQ4plex); S7(P
tLSDVEDQkELAsPVSPEL	A0A0G2JU89	N-Term(iTRAQ4plex); K9(iTRAQ4plex); S13(
iEsPkLER	Q66HA8	N-Term(iTRAQ4plex); S3(Phospho); K5(iTRA
ikESETFsDSSPIEIIDEFPT	F1LQN3	N-Term(iTRAQ4plex); K2(iTRAQ4plex); S8(P
kLEDAEkEEEQsMLAMED	D4AD03	N-Term(iTRAQ4plex); K1(iTRAQ4plex); K7(iT
asPLLSDk	M0R6K4	N-Term(iTRAQ4plex); S2(Phospho); K8(iTRA
eQASHLGGAVFsGAGNIA	A0A0G2JSQ1	N-Term(iTRAQ4plex); S12(Phospho); K25(iT
tDLLNPEEAEDQLADIAsV	A0A0G2JXK1	N-Term(iTRAQ4plex); S18(Phospho)
qVQsLTcEVDALk	P31000	N-Term(iTRAQ4plex); S4(Phospho); C7(Carb
sPVVAksPAEAksPAEAkP	F1LRZ7	N-Term(iTRAQ4plex); K6(iTRAQ4plex); S7(P
tLsIDkGF	P30835	N-Term(iTRAQ4plex); S3(Phospho); K6(iTRA
tSLEPQksLGEEGLNR	Q99JE6	N-Term(iTRAQ4plex); K7(iTRAQ4plex); S8(P
lLsPGTSkltPTELPk	D3ZV52	N-Term(iTRAQ4plex); S3(Phospho); K8(iTRA
kDYLIENEALPsP	D4A5L7	N-Term(iTRAQ4plex); K1(iTRAQ4plex); S12(
sEDSGIGLSASsPELSEHL	A0A0G2JXD9	N-Term(iTRAQ4plex); S12(Phospho)
aVAGDAsESALLk	P06685	N-Term(iTRAQ4plex); S7(Phospho); K13(iTR
eRDHsPTPSVFNsDEER	Q5U317	N-Term(iTRAQ4plex); S5(Phospho); S13(Pho
sPSEAksPAGAksPAEAk	F1LRZ7	N-Term(iTRAQ4plex); S1(Phospho); K6(iTRA
lcYVALDFENEmATAASsS	P68035	N-Term(iTRAQ4plex); C2(Carbamidomethyl);
lPAAVSLYsPTDEQSVMQ	Q9JKS6;D3Z9C7	N-Term(iTRAQ4plex); S9(Phospho); K19(iTR
sLQDLDMPsPEDEGQTR	D3ZXX7	N-Term(iTRAQ4plex); S9(Phospho)
nNNPQSELAAAHGDsESI	F1M820	N-Term(iTRAQ4plex); S15(Phospho)
sRtAsLTSAASIDGSR	A0A0G2JSU4	N-Term(iTRAQ4plex); T3(Phospho); S5(Phos
eQSEsLSELR	D4A0A1	N-Term(iTRAQ4plex); S5(Phospho)
nPLFDHSTLSAPVPGPHS	Q6AY81	N-Term(iTRAQ4plex); S19(Phospho)
gktPEEIR	Q6PEC4	N-Term(iTRAQ4plex); K2(iTRAQ4plex); T3(P
sVsPQTLEAYIR	A0A0G2K590	N-Term(iTRAQ4plex); S3(Phospho)
qDDsPPRPIIGPALPPGFII	Q4V893	N-Term(iTRAQ4plex); S4(Phospho); K20(iTR
aIEQADLLQEEDEsPRSVI	B5DFI3	N-Term(iTRAQ4plex); S14(Phospho)
tPSPLALEDTAELssPPLSI	Q4QQV6	N-Term(iTRAQ4plex); S14(Phospho); S15(Ph
tISSSPsMESLPGGR	A0A0G2K712	N-Term(iTRAQ4plex); S7(Phospho)
ssPNVANQPPsPGGk	F1LT10	N-Term(iTRAQ4plex); S2(Phospho); S11(Pho
kEskEEAPEATk	P15205;F1LRL9	K1(iTRAQ4plex); N-Term(iTRAQ4plex); S3(P
lDsPSRsPSkVEVTEk	Q5U2P5	N-Term(iTRAQ4plex); S3(Phospho); S7(Phos
tSETSIsPPGSSIGSPNR	A0A0G2K875	N-Term(iTRAQ4plex); S7(Phospho)
fGsAAPR	P31421	N-Term(iTRAQ4plex); S3(Phospho)
hDDGTQsDSENAGVHR	A0A0G2K315	N-Term(iTRAQ4plex); S7(Phospho)
aELEEmEETHPsDEEGEE	P34926;Q63330	N-Term(iTRAQ4plex); M6(Oxidation); S12(Ph
lQcDVEDIItPEPETSPPFP	D3ZU84	N-Term(iTRAQ4plex); C3(Carbamidomethyl);
fGsNINLEADDS	M0R5N4	N-Term(iTRAQ4plex); S3(Phospho)
gNAEGssDEEGkLVIDEPA	Q8VHK7	N-Term(iTRAQ4plex); S6(Phospho); S7(Phos
sETAPAETTAPAPVEk	D3ZBN0	N-Term(Acetyl); S1(Phospho); K16(iTRAQ4p
lNNLSPASHLASSsPPPGI	Q9QXY2	N-Term(iTRAQ4plex); S14(Phospho)
cETsPPPTPR	F1M8A4	N-Term(iTRAQ4plex); C1(Carbamidomethyl);
aELEEMEETHPsDEEGEE	P34926;Q63330	N-Term(iTRAQ4plex); S12(Phospho); K20(iT
ssPEIQEPIkPLEk	D3ZJ01	N-Term(iTRAQ4plex); S2(Phospho); K10(iTR
nIVtPRtPPPSQGk	P02688	N-Term(iTRAQ4plex); T4(Phospho); T7(Phos
tIGGGDDsFNTFFSETGA(	P68370	N-Term(iTRAQ4plex); S8(Phospho); K20(iTR
qPLQFHVFDAEDGATsPS	D4ACG7	N-Term(iTRAQ4plex); S16(Phospho); C28(Ca
dADVSEEsPPPLPER	G3V7Q4	N-Term(iTRAQ4plex); S8(Phospho)
lSLsTNcIEk	A0A096MJZ0	N-Term(iTRAQ4plex); S4(Phospho); C7(Carb
rVPVsPVAGS	B0BND5	N-Term(iTRAQ4plex); S5(Phospho)

nLEQILNGGEsPkQk	P58405	N-Term(iTRAQ4plex); S11(Phospho); K13(iT
gDIGGLTsVLDNQRSPLSI	Q923K6	N-Term(iTRAQ4plex); S8(Phospho); K21(iTR
sFSEDAVTDSSGsGTLPR	Q5I0K2	N-Term(iTRAQ4plex); S13(Phospho)
tQsVYDDTk	P31596	N-Term(iTRAQ4plex); S3(Phospho); K9(iTRA
fGESEEVEMEVEsDEEDQ	D3ZQM0	N-Term(iTRAQ4plex); S13(Phospho); K20(iT
kEEsEESDDDMGFGLFD	P02401	N-Term(iTRAQ4plex); K1(iTRAQ4plex); S4(P
sPDFTNENPLETR	P06685	N-Term(iTRAQ4plex); S1(Phospho)
gAySLSIR	A0A0G2K2E3	N-Term(iTRAQ4plex); Y3(Phospho)
gsPSTVSSSYk	P12839	N-Term(iTRAQ4plex); S2(Phospho); K11(iTR
dQPAALPLAAEETVNLPP	A0A0U1RRX4	N-Term(iTRAQ4plex); S19(Phospho); S23(Ph
iDASkNEEDEGHSNSsPR	Q9JJ54	N-Term(iTRAQ4plex); K5(iTRAQ4plex); S16(
vESISVGSVsPGGVk	G3V874;A0A0G2K1Q9	N-Term(iTRAQ4plex); S10(Phospho); K15(iT
nEDLSELLNGMDEELDsP	A0A0G2K1R9;A0A0G2K1(	N-Term(iTRAQ4plex); S17(Phospho); K24(iT
gAQGFGFTIADsPTGQR	A0A0G2JXR9	N-Term(iTRAQ4plex); S12(Phospho)
nSsPPPSPNk	P11505	N-Term(iTRAQ4plex); S3(Phospho); K10(iTR
sLEPESQQNSAEGsPSEQ	D4A0C3	N-Term(iTRAQ4plex); S14(Phospho)
qRGsWDSQQDGSGYESI	F1LR18	N-Term(iTRAQ4plex); S4(Phospho)
gHGGsFIQEcR	F1M0J7	N-Term(iTRAQ4plex); S5(Phospho); C10(Ca
sLSSGFsPEEAQQQDEEI	D3ZF26	N-Term(iTRAQ4plex); S7(Phospho); K20(iTR
qGLEAMEEILsSPEPPDPA	Q6AY84	N-Term(iTRAQ4plex); S11(Phospho); C28(Ca
mVMPSWFDLMGLsPDAP	Q9QYL8	N-Term(iTRAQ4plex); S13(Phospho); K24(iT
dGLAPEkTsPDREk	A0A0G2JZB4	N-Term(iTRAQ4plex); K7(iTRAQ4plex); S9(P
eYVSNDATQsDDEEkLQS	A0A0G2K7S6	N-Term(iTRAQ4plex); S10(Phospho); K15(iT
ekVVtPPAASDIPADSPAP	F1M1Y0	N-Term(iTRAQ4plex); K2(iTRAQ4plex); T5(P
sEEELDESNPFyEPkPTSI	F1LVX2	N-Term(iTRAQ4plex); Y12(Phospho); K15(iT
qDEMDEsDDDLDDkPSPI	A0A0G2K451	N-Term(iTRAQ4plex); S7(Phospho); K14(iTR
tEEVLSPDGSPSksPSk	Q62847;A0A0G2JW28	N-Term(iTRAQ4plex); K13(iTRAQ4plex); S14
eSQSNLIADGDGsEVsDE	F1LMD9	N-Term(iTRAQ4plex); S13(Phospho); S16(Ph
sGssPEMkEk	A0A0G2K2M9	N-Term(iTRAQ4plex); S3(Phospho); S4(Phos
kQESESWsPEAcLGVk	B5DEJ8	N-Term(iTRAQ4plex); K1(iTRAQ4plex); S8(P
aPTsDEIHITVGEAR	P63319	N-Term(iTRAQ4plex); S4(Phospho)
tcDsPQNPMDLISDPVPDS	Q3KR59	N-Term(iTRAQ4plex); C2(Carbamidomethyl);
aSQVkPGDsLPR	P22909	N-Term(iTRAQ4plex); K5(iTRAQ4plex); S9(P
aSADLMSYcEEHARsDPL	G3V6P8	N-Term(iTRAQ4plex); C9(Carbamidomethyl);
vSDQNsPVLPk	F1LSM0	N-Term(iTRAQ4plex); S6(Phospho); K11(iTR
mAtFGSAGSINYPDkk	F1M5M9	N-Term(iTRAQ4plex); T3(Phospho); K15(iTR
vLDTPLsEGDEPTTLPAQ	A0A140UHX6	N-Term(iTRAQ4plex); S7(Phospho)
eRDSGLEDGGEsPSFDTI	D4AD03	N-Term(iTRAQ4plex); S12(Phospho)
gPYPPSQEIPMEVFDPsP	G3V6K8	N-Term(iTRAQ4plex); S17(Phospho); K21(iT
vDNEEWTGGGGHsR	O35314	N-Term(iTRAQ4plex); S13(Phospho)
tLVMAVyDFDR	P21707	N-Term(iTRAQ4plex); Y7(Phospho)
lAEPVASSPVLIVAAAAPtS	F1MAS1	N-Term(iTRAQ4plex); T19(Phospho)
rsPPPsPTTQR	Q08877	N-Term(iTRAQ4plex); S2(Phospho); S6(Phos
iSRPGDsDDSR	B5DFG2	N-Term(iTRAQ4plex); S7(Phospho)
gHYTEGAELVDsVLDVVR	P85108;P69897;Q4QRB4;	N-Term(iTRAQ4plex); S12(Phospho)
sPVVMVDEILSSsPPk	D3ZCC3	N-Term(iTRAQ4plex); S13(Phospho); K16(iT
gSPVSEIGWEtPPPEsPR	D3ZWC6	N-Term(iTRAQ4plex); T11(Phospho); S16(Ph
kGNAEGssDEEGkLVIDEI	Q8VHK7	N-Term(iTRAQ4plex); K1(iTRAQ4plex); S7(P
gsTAVGIRGtNIVVLGVEk	F1M6I7	N-Term(iTRAQ4plex); S2(Phospho); T10(Pho
lDPYDssEDDkEYVGFATL	A0A096MJN4	N-Term(iTRAQ4plex); S6(Phospho); S7(Phos
hPPVLtPPDQEVIR	F1LS42	N-Term(iTRAQ4plex); T6(Phospho)
fWEVISDEHGIDPTGTYH(	B4F7C2;G3V7C6	N-Term(iTRAQ4plex); S21(Phospho)
sDAAPAASDskPsSAEPAI	A0A0G2K1L8	N-Term(iTRAQ4plex); S10(Phospho); K11(iT
tPPLQQsPSVIR	B5DF49	N-Term(iTRAQ4plex); S7(Phospho)
qDEmDEsDDDLDDkPSPI	A0A0G2K451	N-Term(iTRAQ4plex); M4(Oxidation); S7(Pho

mDGNLRQsPQLLPR	D3ZNK1	N-Term(iTRAQ4plex); S8(Phospho)
qsSFEIPDDVPLPAGWEm	R9PXS9	N-Term(iTRAQ4plex); S2(Phospho); M18(Ox
sLSPAAIVTVELHYEQGsF	Q5U2P5;A0A0G2K2V4	N-Term(iTRAQ4plex); S18(Phospho)
gkVPVTYLELLs	D4A7V1	N-Term(iTRAQ4plex); K2(iTRAQ4plex); S12(
kHsPsPPPPTATESR	D3ZJU5	K1(iTRAQ4plex); N-Term(iTRAQ4plex); S3(P
sPsDNSLNTTGIIcR	F1MAQ4	N-Term(iTRAQ4plex); S3(Phospho); C14(Ca
rQsSTADAPELR	A0A0G2K9C8	N-Term(iTRAQ4plex); S3(Phospho)
sIEPDGIDITLSSSLPQAGI	F1LWN1	N-Term(iTRAQ4plex); S21(Phospho); K27(iT
kPSLVSDLPWEGASPQS	D3ZMX6	N-Term(iTRAQ4plex); K1(iTRAQ4plex); S28(
vkEVHDELEDLPsPPPPLs	M0R423	N-Term(iTRAQ4plex); K2(iTRAQ4plex); S13(
dAkPATEPTPsPQAR	G3V8H8	N-Term(iTRAQ4plex); K3(iTRAQ4plex); S11(
dVNNFDQDFTREEPILtLV	F1LMV8	N-Term(iTRAQ4plex); T17(Phospho); K25(iT
tSSFAEPGGGGGGGGGG	A0A0G2K7W7	N-Term(iTRAQ4plex); S23(Phospho); K32(iT
sHGPLLPTIEDSsEEEELR	Q9JKS6;D3Z9C7	N-Term(iTRAQ4plex); S13(Phospho); K26(iT
eNSAAPSPIRPHstSPAkE	Q920Q0	N-Term(iTRAQ4plex); S13(Phospho); T14(Ph
aEkPSALNsEEEAHEQST	G3V7X5	N-Term(iTRAQ4plex); K3(iTRAQ4plex); S9(P
tLsQSSESGTLPSGPPGH	A0A0G2JSU4	N-Term(iTRAQ4plex); S3(Phospho); M20(Ox
sYsPDGkESPSDkk	A0A0G2JSR7	N-Term(iTRAQ4plex); S3(Phospho); K7(iTRA
sPGtPAGEGSGsPPkWQI	A0A0G2JSM7;D3ZZ99	N-Term(iTRAQ4plex); T4(Phospho); S12(Ph
tSVSSVSAsPSR	F1LRZ7	N-Term(iTRAQ4plex); S9(Phospho)
sPsSEEVQGENAGR	F1M084	N-Term(iTRAQ4plex); S3(Phospho)
vQVDGSIGRsPTDEITNLV	M0R5U4	N-Term(iTRAQ4plex); S10(Phospho); K27(iT
aNRADsEEEGAVELAA	A0A173DW30	N-Term(iTRAQ4plex); S6(Phospho)
fQEQEcPPsPEPTR	A0A0G2K508	N-Term(iTRAQ4plex); C6(Carbamidomethyl);
ePPGQPEEsPEAETSTLD	D3ZJG4	N-Term(iTRAQ4plex); S9(Phospho); K23(iTR
gLYDGPVcEVsVtPk	P47942	N-Term(iTRAQ4plex); C8(Carbamidomethyl);
lLTRtPDIAQR	A0A0G2K007	N-Term(iTRAQ4plex); T5(Phospho)
vLGPPVQLAVDDssDEGE	A0A0G2JXS2	N-Term(iTRAQ4plex); S13(Phospho); S14(Ph
aASDGQYENQsPEATSPI	F1LN42	N-Term(iTRAQ4plex); S11(Phospho)
qQEsDDSLVDYGEGGEG	D3ZW56	N-Term(iTRAQ4plex); S4(Phospho)
lSVEIPcPPPVSEADSsIDE	A0A0U1RRX4;F1MAQ5	N-Term(iTRAQ4plex); C7(Carbamidomethyl);
fPQGPPSQMGsPMGNR	Q4KLH4	N-Term(iTRAQ4plex); S11(Phospho)
kEEsPPPPk	D4ACW0	N-Term(iTRAQ4plex); K1(iTRAQ4plex); S4(P
hTGPNsPDTANDGFVR	Q499R8	N-Term(iTRAQ4plex); S6(Phospho)
lSIsALFVTPk	P13233	N-Term(iTRAQ4plex); S4(Phospho); K11(iTR
nsAEIAPPLPVR	F1LVA9	N-Term(iTRAQ4plex); S2(Phospho)
tsPDTLR	Q6PDU1	N-Term(iTRAQ4plex); S2(Phospho)
sGAWFFGSGPQQPSQD	A0A0G2KAV8	N-Term(iTRAQ4plex); S20(Phospho)
hSLsGSSPGIk	A0A0G2K2M9	N-Term(iTRAQ4plex); S4(Phospho); K11(iTR
rPsEsDkEEELEk	Q5QD51	N-Term(iTRAQ4plex); S3(Phospho); S5(Phos
gADNDGsGSESGYTTPk	D3ZC82	N-Term(iTRAQ4plex); S7(Phospho); K17(iTR
vPcNVEGIsPELEk	B2RYK0	N-Term(iTRAQ4plex); C3(Carbamidomethyl);
eGsPLHk	A0A0G2JZF2	N-Term(iTRAQ4plex); S3(Phospho); K7(iTRA
gDsETDLEALFNAVmNPk	R9PXS9	N-Term(iTRAQ4plex); S3(Phospho); M15(Ox
fLEESVAMsPEER	D4ABI6	N-Term(iTRAQ4plex); S9(Phospho)
lISsVDDFTEFV	D3ZLW4	N-Term(iTRAQ4plex); S4(Phospho)
vVFENEQDSNsLTk	F1LVK0	N-Term(iTRAQ4plex); S11(Phospho); K14(iT
sEsLSNcSIGk	A0A0G2K2G7	N-Term(iTRAQ4plex); S3(Phospho); C7(Carb
kDEWGLAAPIsPGPLTPM	A0A0U1RRX4;F1MAQ5	N-Term(iTRAQ4plex); K1(iTRAQ4plex); S11(
rGLsLDSAVPEEHQILGGL	Q8CGU9	N-Term(iTRAQ4plex); S4(Phospho); K23(iTR
wIPLSSESQAPLAQPEsPT	D3ZH40	N-Term(iTRAQ4plex); S17(Phospho)
sLsPPWEQER	A0A0G2JXM0	N-Term(iTRAQ4plex); S3(Phospho)
sIELESVR	P12839	N-Term(iTRAQ4plex); S1(Phospho)
gsLTGVQTcR	D3ZN06	N-Term(iTRAQ4plex); S2(Phospho); C9(Carb
nNNPsPPPDSDLER	A0A0G2JWT3	N-Term(iTRAQ4plex); S5(Phospho)

vTEFNNVTPLSEEEVASIk	A0A0G2JW88	N-Term(iTRAQ4plex); K19(iTRAQ4plex); S22
mGQAGSTISNsHAQPFDI	P08050	N-Term(iTRAQ4plex); S11(Phospho); K26(iT
vTVsSAk	Q4V8H7	N-Term(iTRAQ4plex); S4(Phospho); K7(iTRA
iVPsPDDSk	D3ZKG7	N-Term(iTRAQ4plex); S4(Phospho); K9(iTRA
gVIDLAEVEAVAPGtPTIG/	M0RAP5	N-Term(iTRAQ4plex); T15(Phospho); K22(iT
vIENTDGsEEEmDAR	E9PTU4	N-Term(iTRAQ4plex); S8(Phospho); M12(Ox
qDsDsDVVDVEDAEQDFI	A0A0G2K5T1	N-Term(iTRAQ4plex); S3(Phospho); S5(Phos
aQEDAsDETPkQDR	D3ZSC1	N-Term(iTRAQ4plex); S6(Phospho); K11(iTR
lPsPTk	D3ZCL8	N-Term(iTRAQ4plex); S3(Phospho); K6(iTRA
vISQTNLITTVtPEkk	G3V874;A0A0G2K1Q9	N-Term(iTRAQ4plex); T12(Phospho); K15(iT
eTSVSkEDtDQEEkAsNED	Q566D6	N-Term(iTRAQ4plex); K6(iTRAQ4plex); T9(P
mEPAAAAPAQRLADPtGE	F1LNC3	N-Term(iTRAQ4plex); M1(Oxidation); T16(Ph
gIPLPTGDtsPEPELLPGD	Q5RK09	N-Term(iTRAQ4plex); T9(Phospho); S10(Pho
lAIQGPEDsPSR	D3ZPN3	N-Term(iTRAQ4plex); S9(Phospho)
lDEDAsPNEEk	B2GV74	N-Term(iTRAQ4plex); S6(Phospho); K11(iTR
aAsVELPQR	Q63638	N-Term(iTRAQ4plex); S3(Phospho)
sLsELESLkLPAESNEk	D4A9D8	N-Term(iTRAQ4plex); S3(Phospho); K9(iTRA
vEQALSEVAssLQSSAPk	F1LUY5	N-Term(iTRAQ4plex); S10(Phospho); S11(Ph
vRGEsSEALk	P34926	N-Term(iTRAQ4plex); S5(Phospho); K10(iTR
dLTGQVStPPVk	P15205;F1LRL9	N-Term(iTRAQ4plex); T8(Phospho); K12(iTR
lsPAkSPSLSPSPPSPIEk	P15205;F1LRL9	N-Term(iTRAQ4plex); S2(Phospho); K5(iTRA
ssPSEsPLMEk	Q9JJP0	N-Term(iTRAQ4plex); S2(Phospho); S6(Phos
tDGsIsGDRQPVTVADYIS	B1H2A6	N-Term(iTRAQ4plex); S4(Phospho); S6(Phos
sQkQEEENPAEETGEEk	P60841	N-Term(Acetyl); S1(Phospho); K3(iTRAQ4ple
vLHAQcHSTPDsAEDVR	F1LM19	N-Term(iTRAQ4plex); C6(Carbamidomethyl);
tPSPPEEASPLPSPTASPI	A0A0G2K5Z4	N-Term(iTRAQ4plex); T1(Phospho)
qQAAMGsQGNLSAEVEQ	Q811A7	N-Term(iTRAQ4plex); S7(Phospho)
qNALLFAEEEDGEAGAED	M0RDR2	N-Term(iTRAQ4plex); K19(iTRAQ4plex); S21
kPPPPASPGsSDSSAR	A0A0H2UHQ3	N-Term(iTRAQ4plex); K1(iTRAQ4plex); S10(
gIFcQIDSGSDLTtDLIVQR	O88637	N-Term(iTRAQ4plex); C4(Carbamidomethyl);
gsPGLTR	F1LMT5	N-Term(iTRAQ4plex); S2(Phospho)
sLSNSNPDIsGTPTSPDDI	F1LRS2	N-Term(iTRAQ4plex); S10(Phospho)
vTEMLVNVLNIcSDDELGs	P63329	N-Term(iTRAQ4plex); C12(Carbamidomethyl
tVsDGTISASk	Q2HWF0	N-Term(iTRAQ4plex); S3(Phospho); K11(iTR
hGAsDDYFIPsQEFLEAEF	G3V7X5	N-Term(iTRAQ4plex); S4(Phospho); S11(Pho
tLAsPk	P21818	N-Term(iTRAQ4plex); S4(Phospho); K6(iTRA
yYsDSDDELTVEQR	M0RC54	N-Term(iTRAQ4plex); S3(Phospho)
mFGsNLDLLDPGQ	G3V8Q6	N-Term(iTRAQ4plex); M1(Oxidation); S4(Pho
asLSDLSSLEEVEGMSVR	Q6AYH3	N-Term(iTRAQ4plex); S2(Phospho)
gSDsDSEGDNPEk	Q793F9	N-Term(iTRAQ4plex); S4(Phospho); K13(iTR
rVEsEESGDEEGk	Q5HZV9	N-Term(iTRAQ4plex); S4(Phospho); K13(iTR
sEVQAHsPSR	F1MAQ5;A0A0U1RRX4	N-Term(iTRAQ4plex); S7(Phospho)
rPSGDVLETFNFLENADD	P58405	N-Term(iTRAQ4plex); S19(Phospho); M26(O
vFVGGLsPDTSEEQIk	Q3SWU3	N-Term(iTRAQ4plex); S7(Phospho); K16(iTR
dLYRPLsSDDLDSVGDSV	Q9WVP7	N-Term(iTRAQ4plex); S7(Phospho)
aSIGNIALsPSPVR	F1M1Y0	N-Term(iTRAQ4plex); S9(Phospho)
cIELSSGsVk	P06687	N-Term(iTRAQ4plex); C1(Carbamidomethyl);
aStEGANNMPk	P31596;Q8K5B5	N-Term(Acetyl); T3(Phospho); K11(iTRAQ4pl
lAIQEMVSLTsPSAPASSR	F1LSE6	N-Term(iTRAQ4plex); S11(Phospho)
vEEVEEDsEVLASEk	Q5QD51	N-Term(iTRAQ4plex); S8(Phospho); K15(iTR
iGDLQAAIEDEmEsDENEI	D3ZFD0	N-Term(iTRAQ4plex); M12(Oxidation); S14(F
gMkDDkDEEDDGtGSPHL	P29066	N-Term(iTRAQ4plex); K3(iTRAQ4plex); K6(iT
kkEPAISSQNsPEAR	Q4KLL0	N-Term(iTRAQ4plex); K1(iTRAQ4plex); K2(iT
yLATAsTmDHAR	P02688	N-Term(iTRAQ4plex); S6(Phospho); M8(Oxid
vIETVVISETGEsPEcVGAI	Q5QD51	N-Term(iTRAQ4plex); S13(Phospho); C16(Ca

lGLAVIHGEAQctELDmDD	Q63468	N-Term(iTRAQ4plex); C12(Carbamidomethyl
sLMssPEDLTk	P15205;F1LRL9	N-Term(iTRAQ4plex); S4(Phospho); S5(Phos
gQGAsPVAMQk	P85972	N-Term(iTRAQ4plex); S5(Phospho); K11(iTR
iQNTGDyYDLYGGEk	P41499	N-Term(iTRAQ4plex); Y7(Phospho); K15(iTR
aEsGDSLSSEDRDLLYSIE	M0RDG0	N-Term(iTRAQ4plex); S3(Phospho)
gIEDAsDEEDLR	G3V9N7	N-Term(iTRAQ4plex); S6(Phospho)
asPALGSGPDGSGDSLEI	R9PXR4	N-Term(iTRAQ4plex); S2(Phospho)
qsPDHPTVGAGMLHITEN	P15205;F1LRL9	N-Term(iTRAQ4plex); S2(Phospho); K38(iTR
qVQQNsLHR	D3ZV52	N-Term(iTRAQ4plex); S6(Phospho)
sSIsPMDEPVPDSEsPIEk	P15205;F1LRL9	N-Term(iTRAQ4plex); S4(Phospho); S15(Pho
eLALsSPEDLTQDFEELk	P34926;Q63330	N-Term(iTRAQ4plex); S5(Phospho); K18(iTR
dPQDMEGEksPASPFAQT	A0A0U1RRX4;F1MAQ5	N-Term(iTRAQ4plex); K9(iTRAQ4plex); S10(
nVGNNSFNDIMEANLPsP	A0A0G2K451	N-Term(iTRAQ4plex); S17(Phospho); K21(iT
dDkDEEDDGTGsPHLNNI	P29066	N-Term(iTRAQ4plex); K3(iTRAQ4plex); S12(
sNATNSSYsPPTGR	D3ZWV8	N-Term(iTRAQ4plex); S9(Phospho)
rLSPWEDLSQAPTmDsPL	F1LR33	N-Term(iTRAQ4plex); M14(Oxidation); S16(F
fIDkDQQPsGSEGEDDDA	A0A0G2JZB6	N-Term(iTRAQ4plex); K4(iTRAQ4plex); S9(P
tFTGEHVVALMPEAGsPQ	B4F774	N-Term(iTRAQ4plex); S16(Phospho)
kIAELEEERsQGSTSNSD	F1LRL9	N-Term(iTRAQ4plex); K1(iTRAQ4plex); S10(
sSsLESQGk	A0A0G2K2R0	N-Term(iTRAQ4plex); S3(Phospho); K9(iTRA
dVEmGNsVIEENEmk	A0A0G2K611	N-Term(iTRAQ4plex); M4(Oxidation); S7(Pho
eSDDkPEIEDVGsDEEEE	P82995	N-Term(iTRAQ4plex); K5(iTRAQ4plex); S13(
gSVGPAPSLSELDLRPPs	D3ZWQ0	N-Term(iTRAQ4plex); S18(Phospho)
sQGPRDTDDDEEEPDPY	D4A1G8	N-Term(iTRAQ4plex); S1(Phospho)
aQTLPTSVVTITSESsPGk	G3V6S0	N-Term(iTRAQ4plex); S16(Phospho); K19(iT
sIQHSIsMPAMR	A0A0G2K865	N-Term(iTRAQ4plex); S7(Phospho)
dYNASAsTISPPsSMEEDk	P15205;F1LRL9	N-Term(iTRAQ4plex); S7(Phospho); S13(Pho
aLAGcDFLtISPk	Q9EQS0	N-Term(iTRAQ4plex); C5(Carbamidomethyl);
rAPSPVVsPTELSk	A0A0G2K7H2	N-Term(iTRAQ4plex); S8(Phospho); K14(iTR
aFPkEELAsDLEEmATSSI	Q5RJN5	N-Term(iTRAQ4plex); K4(iTRAQ4plex); S9(P
iSSESFSSLGSDYLETsPE	Q9WVI4	N-Term(iTRAQ4plex); S17(Phospho); C24(Ca
ssGQESESIPEYTAEEERI	Q5BJR4	N-Term(iTRAQ4plex); S2(Phospho)
rLGtPPGGGAAGk	F1LSH6	N-Term(iTRAQ4plex); T4(Phospho); K13(iTR
sSSPLPTVQLHPQsPTAG	Q68HB8	N-Term(iTRAQ4plex); S14(Phospho); K19(iT
kLPSESDLLEGEVtDEDEE	A0A0G2K4N6	N-Term(iTRAQ4plex); K1(iTRAQ4plex); T14(
eASPEsEDEDEALPGTDV	M0R8J3	N-Term(iTRAQ4plex); S6(Phospho)
sPVGksPPATGSAYGSSQ	Q5M7V8	N-Term(iTRAQ4plex); K5(iTRAQ4plex); S6(P
sLGDASPSVPESVGAR	M0R4J7	N-Term(iTRAQ4plex); S1(Phospho)
ekAEEEGGSEEEVGDksP	P12839	N-Term(iTRAQ4plex); K2(iTRAQ4plex); K16(
dkEDLVcSAALHsPQESPV	F1LQN3	N-Term(iTRAQ4plex); K2(iTRAQ4plex); C7(C
kksPNELVDDLFk	A0A0G2K911	K1(iTRAQ4plex); N-Term(iTRAQ4plex); K2(iT
kLGQsPEGEEASR	O70150	N-Term(iTRAQ4plex); K1(iTRAQ4plex); S5(P
lGLDFPNLPYLIDGsRk	G3V983	N-Term(iTRAQ4plex); S15(Phospho); K17(iT
aHsPVQSGLPGmQTLk	B0LT89	N-Term(Acetyl); S3(Phospho); M12(Oxidation
rPIIPGmEFsR	P48303	N-Term(iTRAQ4plex); M7(Oxidation); S10(Ph
iPIVAQNsEEEsPLSPVGQ	D3Z9C7	N-Term(iTRAQ4plex); S8(Phospho); S12(Pho
cTDPELVsPDSGcETTLEE	A0A0G2K0F3	N-Term(iTRAQ4plex); C1(Carbamidomethyl);
gsYMEVEDNR	A0A0H2UHZ1	N-Term(iTRAQ4plex); S2(Phospho)
gNPSAVPAPIPcVPPLPPF	Q9Z0G8	N-Term(iTRAQ4plex); C12(Carbamidomethyl
nPWsDDESkSESDLEEAE	Q14TE9	N-Term(iTRAQ4plex); S4(Phospho); K9(iTRA
sEsPPPLSEPk	D3ZWU1	N-Term(iTRAQ4plex); S3(Phospho); K11(iTR
sSSDTSTPEELk	D4A0X3	N-Term(iTRAQ4plex); S1(Phospho); K12(iTR
iAQLEEELEEEQGNtELIN	G3V6P7	N-Term(iTRAQ4plex); T15(Phospho)
sPAEVksPAEVk	F1LRZ7	N-Term(iTRAQ4plex); K6(iTRAQ4plex); S7(P
sATLssTDSTVSEMQDEV	Q5QD51	N-Term(iTRAQ4plex); S5(Phospho); S6(Phos

Information Classification: General

qEPEsEEEEEEkQEk	F1LT30	N-Term(iTRAQ4plex); S5(Phospho); K12(iTR
dAcEPEsGPDPSTVR	G3V984	N-Term(iTRAQ4plex); C3(Carbamidomethyl);
tIsAQDTLAYATALLNEk	F1LSM0	N-Term(iTRAQ4plex); S3(Phospho); K18(iTR
eLGDsSGEGPEFVEEDEE	E9PU01	N-Term(iTRAQ4plex); S5(Phospho)
tVMSGENADEASAAPGHI	P10499	N-Term(iTRAQ4plex); S22(Phospho)
kDVEAGGSQSQTPGSTA	B5DEI5	N-Term(iTRAQ4plex); K1(iTRAQ4plex); S23(
tsskEssPVPSPTSDRk	G3V6S0	N-Term(iTRAQ4plex); S2(Phospho); S3(Phos
eVPPPPVEEsEEEDDDGL	Q0PMD2	N-Term(iTRAQ4plex); S10(Phospho); K20(iT
sETSsNPSsPEIcPNk	D3ZPI4	N-Term(iTRAQ4plex); S5(Phospho); S9(Phos
hIkEEPLsEEEPcTSTAVPs	Q9QZ86	N-Term(iTRAQ4plex); K3(iTRAQ4plex); S8(P
sQGsTSNSDWmk	F1LRL9	N-Term(iTRAQ4plex); S4(Phospho); M11(Ox
eRsLDSVDR	G3V7T8	N-Term(iTRAQ4plex); S3(Phospho)
sGDPAPPDsPTWEAk	Q63624	N-Term(iTRAQ4plex); S9(Phospho); K15(iTR
lsDcSDYSGQFLQPEAWR	P62957	N-Term(iTRAQ4plex); S2(Phospho); C4(Carb
dGGEALVsPDGTVTEAPR	D3ZWL6	N-Term(iTRAQ4plex); S8(Phospho)
nFYDsDDEEk	A0A0G2JWD6	N-Term(iTRAQ4plex); S5(Phospho); K10(iTR
kGSPVSEIGWEtPPPEsPI	D3ZWC6	K1(iTRAQ4plex); N-Term(iTRAQ4plex); T12(
iEPIPGEsPk	A0A0H2UHV4	N-Term(iTRAQ4plex); S8(Phospho); K10(iTR
dAATEEPGSPVkstPAsPV	F8WFS9	N-Term(iTRAQ4plex); K12(iTRAQ4plex); S13
rPEsDEDSLGSSGR	P70531	N-Term(iTRAQ4plex); S4(Phospho)
fQFPGEIDSsETELAEMk	O88397	N-Term(iTRAQ4plex); S10(Phospho); K18(iT
sAsEPSLHR	P11345	N-Term(iTRAQ4plex); S3(Phospho)
kLNkTDEEIsS	P61765	N-Term(iTRAQ4plex); K1(iTRAQ4plex); K4(iT
eGLDISHLQGQEELLSsQ	Q9R1N0	N-Term(iTRAQ4plex); S17(Phospho); K20(iT
dSDsDADEATPTTTPR	A0A0G2K700	N-Term(iTRAQ4plex); S4(Phospho)
gILSLPHQAsPVSR	A9UK05	N-Term(iTRAQ4plex); S10(Phospho)
fTAsPLEEER	P46844	N-Term(iTRAQ4plex); S4(Phospho)
kLQsMESQVEEWcR	A0A0G2K677	K1(iTRAQ4plex); N-Term(iTRAQ4plex); S4(P
tWGDAGAAAGGGtPSk	P12369	N-Term(iTRAQ4plex); T13(Phospho); K16(iT
vIcLVGAGISTsAGIPDFR	A0A0G2JWM2	N-Term(iTRAQ4plex); C3(Carbamidomethyl);
vkAsPITNDGEDEFVPsDG	Q14TE9	N-Term(iTRAQ4plex); K2(iTRAQ4plex); S4(P
sEQSSMSIEFGQEsPEHS	P15205;F1LRL9	N-Term(iTRAQ4plex); S14(Phospho)
tLPsEDEEGEEDkEFYLR	Q8VHJ9	N-Term(iTRAQ4plex); S4(Phospho); K13(iTR
nEDLSELLNGmDEELDsP	A0A0G2K1R9;A0A0G2K1	N-Term(iTRAQ4plex); M11(Oxidation); S17(P
aSGNPsPPALGEGPRPGI	D3ZS76	N-Term(iTRAQ4plex); S6(Phospho); C21(Car
vTPLELEVLEEIVQTMDTs	Q9JHW0	N-Term(iTRAQ4plex); S19(Phospho)
tLSNAEDYLDDEDsD	Q6P686	N-Term(iTRAQ4plex); S14(Phospho)
tIVMGAsFR	Q9EQS0	N-Term(iTRAQ4plex); S7(Phospho)
aGAsEGEDEDDAADEDA	M0R3U3	N-Term(iTRAQ4plex); S4(Phospho)
hVPASAVVSSAMNSAPVL	F1M2D4	N-Term(iTRAQ4plex); S23(Phospho)
eSDSAEGDETEsPEQPTR	F1M241	N-Term(iTRAQ4plex); S12(Phospho)
dkDSEVSTkPEGVHAPNQ	Q64548	N-Term(iTRAQ4plex); K2(iTRAQ4plex); K9(iT
sVsDQFYR	Q07205	N-Term(iTRAQ4plex); S3(Phospho)
qTAEPAGLsLRGESDSGs	Q5I0Q2	N-Term(iTRAQ4plex); S9(Phospho); S18(Pho
sPAEVksPAVAk	F1LRZ7	N-Term(iTRAQ4plex); S1(Phospho); K6(iTRA
kLAAGAESPQPASGNsPS	F1LMV8	N-Term(iTRAQ4plex); K1(iTRAQ4plex); S16(
vIHDNFGIVEGLMtTVLAIT	A0A0G2K7M1	N-Term(iTRAQ4plex); T14(Phospho); K24(iT
iIPQEELNsPTSAPSSEk	A0A0G2JXR9	N-Term(iTRAQ4plex); S9(Phospho); K18(iTR
qDAIGkPPPPTPAGNQsP	A0A0G2K9F7	N-Term(iTRAQ4plex); K6(iTRAQ4plex); S17(
gGLLTSEEDSGFSTsPk	Q62806	N-Term(iTRAQ4plex); S15(Phospho); K17(iT
qLsLLDSR	D3ZC56	N-Term(iTRAQ4plex); S3(Phospho)
sETMVNAQQTPLGtPk	Q920Q0	N-Term(iTRAQ4plex); T14(Phospho); K16(iT
lDERPSsPIPLLPPPk	A0A0G2K6T6	N-Term(iTRAQ4plex); S7(Phospho); K16(iTR
iNHTVILDDPFDDPPDLLIF	D4AEG3	N-Term(iTRAQ4plex); S23(Phospho); K28(iT
kSEsEVEEAAAIIAQRPDN	Q07266;A0A0H2UHL9	N-Term(iTRAQ4plex); K1(iTRAQ4plex); S4(P

eNYEDQEPLAGQEsPITL/	D3ZF26	N-Term(iTRAQ4plex); S14(Phospho)
kHsQTDLVSR	B3DMA0	N-Term(iTRAQ4plex); K1(iTRAQ4plex); S3(P
sYsSSSGSLMPSLENLDL	P19527	N-Term(iTRAQ4plex); S3(Phospho); K29(iTR
nLESGRsPSEEEEDGQLV	P0C6P5	N-Term(iTRAQ4plex); S7(Phospho); K19(iTR
gsTTSIPSPQSDGGDPNQ	D4ACS3	N-Term(iTRAQ4plex); S2(Phospho)
sPVPSQQAsPk	Q9JKS6	N-Term(iTRAQ4plex); S9(Phospho); K11(iTR
eGEEPTVYsDDEEPkDEA	P70580	N-Term(iTRAQ4plex); S9(Phospho); K15(iTR
qmxMsPPPGNAGPVIMSL	O08729	N-Term(iTRAQ4plex); M2(Oxidation); X3(T);
sFMQVLcEkYsPENFPYR	Q5PQJ7	N-Term(iTRAQ4plex); C7(Carbamidomethyl);
qTsQDDkPAFR	Q99N97	N-Term(iTRAQ4plex); S3(Phospho); K7(iTRA
dLVDVASGVDEDELEPLtI	D3ZJ01	N-Term(iTRAQ4plex); T18(Phospho); K42(iT
vPQSLTTAPAsPPVLQR	D3ZTF0	N-Term(iTRAQ4plex); S11(Phospho)
qAQsPSGVSSEVEVLk	F1M863;F1M8A4	N-Term(iTRAQ4plex); S4(Phospho); K16(iTR
iLDPDPDPPsPESPTETFA	A0A0G2KB48	N-Term(iTRAQ4plex); S10(Phospho)
dGGsGNSTIIVSR	A0A0G2JVW5	N-Term(iTRAQ4plex); S4(Phospho)
tLFLGIPDENFEDHSAPPs	D3ZW27	N-Term(iTRAQ4plex); S19(Phospho); K23(iT
eTIkVEtPTDIHSEk	A0A0G2K6R9;F1M9N9	N-Term(iTRAQ4plex); K4(iTRAQ4plex); T7(P
kYNTDcVQGLTHsk	P06687	N-Term(iTRAQ4plex); K1(iTRAQ4plex); C6(C
sAPcGSLAFSGDR	P34926	N-Term(iTRAQ4plex); S1(Phospho); C4(Carb
eDTkMsIsEGTVsDk	P15205;F1LRL9	N-Term(iTRAQ4plex); K4(iTRAQ4plex); S6(P
iLDQMPAtPsSPMYVD	F1LP57	N-Term(iTRAQ4plex); T8(Phospho); S10(Pho
hGsPTAPIGAGSPEFTEQ	A0A0G2K6Z8	N-Term(iTRAQ4plex); S3(Phospho)
fSQLPEMVMGsPPPPVPF	F1LXC7	N-Term(iTRAQ4plex); S11(Phospho)
sQQQkPEEEAVSSSQsPA	Q64548	N-Term(iTRAQ4plex); K5(iTRAQ4plex); S16(
sGGDTHsPPR	F1M5M9	N-Term(iTRAQ4plex); S7(Phospho)
fLQksPERSPPAGtVSNPP	D3ZWM3	N-Term(iTRAQ4plex); K4(iTRAQ4plex); S5(P
vAsPANVGTLHTLSR	D3ZJR6	N-Term(iTRAQ4plex); S3(Phospho)
tEGDsPAAALSPQMHQES	A0A0G2K6R9;F1M9N9;A0	N-Term(iTRAQ4plex); S5(Phospho); K26(iTR
aAsPSPQSVR	B2RYB3;A0A0G2K4F6	N-Term(iTRAQ4plex); S3(Phospho)
nDESQLsPATR	A0A0G2K1J5	N-Term(iTRAQ4plex); S7(Phospho)
aELGMNDsPSQsPPVk	G3V9U0	N-Term(iTRAQ4plex); S8(Phospho); S12(Pho
sDVETATDsDTESR	F1LSL6	N-Term(iTRAQ4plex); S9(Phospho)
gLASsPPGGALRPR	D3ZWQ0	N-Term(iTRAQ4plex); S5(Phospho)
rGsQPDAELDGAGTSLLR	D3ZWQ0	N-Term(iTRAQ4plex); S3(Phospho)
ssLYEGLEkPESR	P11506;P11505	N-Term(iTRAQ4plex); S2(Phospho); K9(iTRA
rLREHIQNFQEssNtHPGS	Q3MHS0	N-Term(iTRAQ4plex); S12(Phospho); S13(Ph
aSsFSGISILTR	G3V9G7	N-Term(iTRAQ4plex); S3(Phospho)
eAsGDSPkEYVGSPPPLP	B1WC06	N-Term(iTRAQ4plex); S3(Phospho); K8(iTRA
sVSAIsSDSISTSADNFSP	D3ZA31	N-Term(iTRAQ4plex); S6(Phospho)
rtSmGGTQQQFVEGVR	A0A0G2JT93	N-Term(iTRAQ4plex); T2(Phospho); M4(Oxid
eATLPPVsPPk	M0RD03	N-Term(iTRAQ4plex); S8(Phospho); K11(iTR
rSGsFQGAVR	A0A0G2K9Q6	N-Term(iTRAQ4plex); S4(Phospho)
vQEAQDsDsDTEGGATG(	A0A0G2JWM2	N-Term(iTRAQ4plex); S7(Phospho); S9(Phos
vETVsPSQk	D4ABH1	N-Term(iTRAQ4plex); S5(Phospho); K9(iTRA
tAQVPsPPR	E9PST5	N-Term(iTRAQ4plex); S6(Phospho)
nIILAPESсEGsPR	G3V6I1	N-Term(iTRAQ4plex); C9(Carbamidomethyl);
eEAksPAEAk	F1LRZ7	N-Term(iTRAQ4plex); K4(iTRAQ4plex); S5(P
gASPVTPSsTPPPPTDPQ	D4A1G8	N-Term(iTRAQ4plex); S9(Phospho); K21(iTR
tELcENVDPNsPAAk	F1LZS5	N-Term(iTRAQ4plex); C4(Carbamidomethyl);
sALtPPTDMPTPTTDk	Q5FVJ0;A0A0G2K6A9	N-Term(Acetyl); T4(Phospho); K16(iTRAQ4pl
aSPScFHsEDEDSLk	P81377	N-Term(Acetyl); C5(Carbamidomethyl); S8(Pl
gsPGEEWAPEVEEASQR	A0A0G2JZ27	N-Term(iTRAQ4plex); S2(Phospho)
tIVIRPPFLRPEVsDGQITV	Q2THW7	N-Term(iTRAQ4plex); S14(Phospho); K21(iT
hGASAAPsPPPR	D3ZSY8	N-Term(iTRAQ4plex); S8(Phospho)
sGSIVELLAGGGSScsPVL	P51111	N-Term(iTRAQ4plex); C15(Carbamidomethyl

kFLEESVAMsPEER	D4ABI6	N-Term(iTRAQ4plex); K1(iTRAQ4plex); S10(
eENQTGPTttPsDTQDLDN	Q920Q0	N-Term(iTRAQ4plex); T9(Phospho); T10(Pho
aQAPELQQEEERPGAVG	F1LPA3	N-Term(iTRAQ4plex); S18(Phospho)
tAGTPDGPEAPPGPDAsP	Q5U2N3	N-Term(iTRAQ4plex); S17(Phospho); K24(iT
eVMsPTGSAR	Q8K3P4	N-Term(iTRAQ4plex); S4(Phospho)
eLEQHIQTsDPENFQSEE	F1MAA1	N-Term(iTRAQ4plex); S9(Phospho)
eEGAsPLDFD	A0A0G2K6U1	N-Term(iTRAQ4plex); S5(Phospho)
lFSSSSsPPPAk	B0VXR4	N-Term(iTRAQ4plex); S7(Phospho); K12(iTR
eLESGTEEsDEER	D3ZGU6	N-Term(iTRAQ4plex); S9(Phospho)
kQAEDTISNASSQLSsPPT	F1M386	N-Term(iTRAQ4plex); K1(iTRAQ4plex); S16(
gINLPTPPGLsPSDLDAFV	Q66HA5	N-Term(iTRAQ4plex); S11(Phospho)
tAsGsSVTSLEGTR	Q6JE36	N-Term(iTRAQ4plex); S3(Phospho); S5(Phos
aALGLQDsDDEDAAVDID	Q62780	N-Term(iTRAQ4plex); S8(Phospho); K28(iTR
aIDIYEQVGTSAMDsPLLk	P54921	N-Term(iTRAQ4plex); S15(Phospho); K19(iT
lPEDDEPPARPPPPPPAG	F1LQN3	N-Term(iTRAQ4plex); S20(Phospho); K36(iT
tEVLLDQPEIWGSGQPINI	F1M1Y0	N-Term(iTRAQ4plex); S23(Phospho); K25(iT
fYYSSGsssPTHAk	Q6IRG7	N-Term(iTRAQ4plex); S7(Phospho); S8(Phos
sLQQLAEER	P16086	N-Term(iTRAQ4plex); S1(Phospho)
rAGDMLEDsPk	Q8VHK7	N-Term(iTRAQ4plex); S9(Phospho); K11(iTR
vNTAEDVsPTSTQSLTTE	A0A0G2JYB3	N-Term(iTRAQ4plex); S8(Phospho); K20(iTR
nHPDRGsDTSPEAEASSC	Q9WVR6	N-Term(iTRAQ4plex); S7(Phospho); K25(iTR
eSsDSTNTTIEDEDAk	G3V9G3	N-Term(iTRAQ4plex); S3(Phospho); K16(iTR
vNPAPSDSSPGELFAGGF	F1LNC3	N-Term(iTRAQ4plex); C19(Carbamidomethyl
mVVPkAAIyQTQAFYAk	Q6TXF1	N-Term(iTRAQ4plex); K5(iTRAQ4plex); Y9(P
gGSsGEELEDEEPVk	A0A0G2JYC7	N-Term(iTRAQ4plex); S4(Phospho); K15(iTR
eNMsLPSNLQLNDLTPDIF	D3Z9V8	N-Term(iTRAQ4plex); S4(Phospho)
ecVGsPDPDLEPGEAN	Q9JHZ4	N-Term(iTRAQ4plex); C2(Carbamidomethyl);
sNsLPHPAGGGk	F1M3D2	N-Term(iTRAQ4plex); S3(Phospho); K12(iTR
sAGGREDSEsPGTQR	Q6AXU6	N-Term(iTRAQ4plex); S10(Phospho)
gTEVSSAAQAPAPAAsPP	Q9WVR8	N-Term(iTRAQ4plex); S16(Phospho); K30(iT
ePVANQDPVsPSLVQGR	P10980	N-Term(iTRAQ4plex); S10(Phospho)
lSLsDGEsGEEk	A0A0G2JXZ3	N-Term(iTRAQ4plex); S4(Phospho); S8(Phos
gQPVLtPPDQLVIANIDQS	F1M2P8	N-Term(iTRAQ4plex); T6(Phospho); S25(Pho
lAGGQTSQPTTPLTsPQR	F1M8V2	N-Term(iTRAQ4plex); S15(Phospho)
lTMGMLksPNTAILIkDEAF	Q9QXY2	N-Term(iTRAQ4plex); K7(iTRAQ4plex); S8(P
tPVkEPNSENVDIsSGGG	Q7TQ88	N-Term(iTRAQ4plex); K4(iTRAQ4plex); S14(
skscDDGLNTFRDEGR	F1M2D4	N-Term(iTRAQ4plex); K2(iTRAQ4plex); S3(P
vTNDIsPESSPGVGR	G3V7E6	N-Term(iTRAQ4plex); S6(Phospho)
vASVsLEGLNLAR	F1M8A4	N-Term(iTRAQ4plex); S5(Phospho)
tVtPAssAkTSPAk	P47942	N-Term(iTRAQ4plex); T3(Phospho); S6(Phos
qSLPYPQPGLEsPGIESPT	D4A758	N-Term(iTRAQ4plex); S12(Phospho); K25(iT
qmGsTEGESDVPVLVTSE	A0A0G2K6R9;F1M9N9	N-Term(iTRAQ4plex); M2(Oxidation); S4(Pho
asPGPGELGk	O55170	N-Term(iTRAQ4plex); S2(Phospho); K10(iTR
eEEQEDLTkDMDEPsPVP	D4A510	N-Term(iTRAQ4plex); K9(iTRAQ4plex); S15(
aLNAEtPksSPLPAk	G3V656	N-Term(iTRAQ4plex); T6(Phospho); K8(iTRA
gsYmEVEDNR	A0A0H2UHZ1	N-Term(iTRAQ4plex); S2(Phospho); M4(Oxid
sPNELVDDLFk	A0A0G2K911	N-Term(iTRAQ4plex); S1(Phospho); K11(iTR
sPSPPPDGsPAATPEIR	Q5HZA7	N-Term(iTRAQ4plex); S9(Phospho)
eRPktPPSNLsPIEDAsPTE	Q9JKS6;D3Z9C7	N-Term(iTRAQ4plex); K4(iTRAQ4plex); T5(P
lSNVsSSGSINLLESPQLA	A0A0U1RRX4;F1MAQ5	N-Term(iTRAQ4plex); S5(Phospho); K31(iTR
lVTGDRNNsScR	P08050	N-Term(iTRAQ4plex); S9(Phospho); C11(Car
tNTGsAmLEQIAMSDR	P09951;G3V733	N-Term(iTRAQ4plex); S5(Phospho); M7(Oxid
gkGsPQAQTVR	F1M6X3	N-Term(iTRAQ4plex); K2(iTRAQ4plex); S4(P
qLTEMLPSILNQLGADsLT	Q5U3Y8	N-Term(iTRAQ4plex); S17(Phospho)
sPAEAksPASVk	F1LRZ7	N-Term(iTRAQ4plex); S1(Phospho); K6(iTRA

Information Classification: General

rLPSSPAsPsPk	A0A0G2K0F3	N-Term(iTRAQ4plex); S8(Phospho); S10(Pho
lGSsPPQTLk	Q5XI44	N-Term(iTRAQ4plex); S4(Phospho); K10(iTR
qGLksPPESLNDLGAFESI	F1LSG8	N-Term(iTRAQ4plex); K4(iTRAQ4plex); S5(P
kAEsPVkEk	P12839	K1(iTRAQ4plex); N-Term(iTRAQ4plex); S4(P
dkPmYDEIFYTLsPVDGk	Q8R491	N-Term(iTRAQ4plex); K2(iTRAQ4plex); M4(O
qLEYQQFEDDkLsQk	G3V874;A0A0G2K1Q9	N-Term(iTRAQ4plex); K11(iTRAQ4plex); S13
gSGTAsDDEFENLR	A0A0G2JVW5	N-Term(iTRAQ4plex); S6(Phospho)
eTFAsDTNEALASLDEAQ	Q63068	N-Term(iTRAQ4plex); S5(Phospho); K25(iTR
dtGSPPASEEATGDSR	B0BN77	N-Term(iTRAQ4plex); T2(Phospho)
aQRLsQETEALGR	Q63083	N-Term(iTRAQ4plex); S5(Phospho)
isPEQHWDFTAEDLk	F1LP57	N-Term(iTRAQ4plex); S2(Phospho); K15(iTR
rFsFcFSPEPEAEAAAGP(	O08623	N-Term(iTRAQ4plex); S3(Phospho); C5(Carb
kGSDEVTASSAAtGTSPR	F1LPH1	N-Term(iTRAQ4plex); K1(iTRAQ4plex); T13(
sGksPsPSPTSPGSLR	Q9WVP7;O08875;A0A0G:	N-Term(iTRAQ4plex); K3(iTRAQ4plex); S4(P
lDPPPsPHANR	A0A0G2K847	N-Term(iTRAQ4plex); S6(Phospho)
kPSASLPsPSGSR	M0RCT5	N-Term(iTRAQ4plex); K1(iTRAQ4plex); S8(P
sEQSSmSIEFGQEsPEHS	P15205;F1LRL9	N-Term(iTRAQ4plex); M6(Oxidation); S14(Ph
msGFMYQGk	Q5XXR3	N-Term(iTRAQ4plex); S2(Phospho); K9(iTRA
sATPATDGRAtPAtEESTV	Q63092	N-Term(iTRAQ4plex); T11(Phospho); T14(Ph
gsMPVPYk	A0JPL9	N-Term(iTRAQ4plex); S2(Phospho); K8(iTRA
asPALGSGPDGSGDSLEr	R9PXR4	N-Term(iTRAQ4plex); S2(Phospho); M18(Ox
eAASPVVQEAsPELSPEE	Q5I0E3	N-Term(iTRAQ4plex); S11(Phospho)
sDsDSSTLAk	D4AEJ5	N-Term(iTRAQ4plex); S3(Phospho); K10(iTR
eAAtPEEEGGAPEk	F1LRZ1	N-Term(iTRAQ4plex); T4(Phospho); K14(iTR
ttIcGk	P60203	N-Term(iTRAQ4plex); T2(Phospho); C4(Carb
lRPVsPEEIR	D3ZEX7	N-Term(iTRAQ4plex); S5(Phospho)
eLSHsPPRDNSFESLEFR	A0A0G2K2M9	N-Term(iTRAQ4plex); S5(Phospho)
nQsPVVPAR	F1LQZ9	N-Term(iTRAQ4plex); S3(Phospho)
sLGGAVGSAASGR	M0RBD9	N-Term(iTRAQ4plex); S1(Phospho)
tsPEPQREDASPAPGTTA	A0A0G2K7C1	N-Term(iTRAQ4plex); S2(Phospho)
gANNNNTtNPAPAPSk	Q5PSQ6	N-Term(iTRAQ4plex); T8(Phospho); K16(iTR
aEPEksEGAAEEQPEPAP	Q05175;A0A0G2K1L8	N-Term(iTRAQ4plex); K5(iTRAQ4plex); S6(P
sREEEWDPEYTPk	Q5M7V8	N-Term(iTRAQ4plex); S1(Phospho); K13(iTR
sSDTsQAVITTPPPPSMPI	A0A0G2JW28	N-Term(Acetyl); S5(Phospho); K20(iTRAQ4p
isDSESEELPkPR	Q3SWT4	N-Term(iTRAQ4plex); S2(Phospho); K11(iTR
aITSLLGGGsPk	A0A0G2K0J4	N-Term(iTRAQ4plex); S10(Phospho); K12(iT
rVsGDGAQDLDR	F1MAL5	N-Term(iTRAQ4plex); S3(Phospho)
tSTsVTSVNQASTSR	A0A0A0MXV8	N-Term(iTRAQ4plex); S4(Phospho)
tQSPHsPkEESER	F1LXQ7	N-Term(iTRAQ4plex); S6(Phospho); K8(iTRA
gDGGSTPTPGDSLQNPD	D3Z899	N-Term(iTRAQ4plex); S24(Phospho)
dTGILDsIGR	P02688	N-Term(iTRAQ4plex); S7(Phospho)
aEQEPTAEQLAQIAAENE	Q5XI73	N-Term(Acetyl); S23(Phospho); K27(iTRAQ4
aASPAsPSGPk	B0BN10	N-Term(iTRAQ4plex); S6(Phospho); K11(iTR
lDVTTPEsPk	D3ZEX7	N-Term(iTRAQ4plex); S8(Phospho); K10(iTR
dGkEDETStDVDEkPk	P53987	N-Term(iTRAQ4plex); K3(iTRAQ4plex); T9(P
qPtPPFFGR	M0R3R6	N-Term(iTRAQ4plex); T3(Phospho)
aAQAsLSALNDPSAVEQA	G3V7L1	N-Term(iTRAQ4plex); S5(Phospho); K22(iTR
sSssVHLMEPQTTVVHNA	P11730	N-Term(iTRAQ4plex); S3(Phospho); S4(Phos
sRELHDVDLAEVkPLVEk	Q6JE36	N-Term(Acetyl); S1(Phospho); K13(iTRAQ4p
sPDLGEYDPLtQADSDES	D3ZWJ9	N-Term(iTRAQ4plex); T11(Phospho); K29(iT
lNQQGAVtPk	Q99MS0	N-Term(iTRAQ4plex); T8(Phospho); K10(iTR
iAAPELHkGDsDsEEDEPA	B2RYA6	N-Term(iTRAQ4plex); K8(iTRAQ4plex); S11(
rDsSDDWEIPDGQITVGQ	F1M9C3	N-Term(iTRAQ4plex); S3(Phospho)
ssENAIQEEQFFGR	D4A318	N-Term(iTRAQ4plex); S2(Phospho)
tTPSPktPPGSGEPPk	D4A1Q2	N-Term(iTRAQ4plex); K6(iTRAQ4plex); T7(P

Information Classification: General

vVQTsPSAR	D4A1G8	N-Term(iTRAQ4plex); S5(Phospho)
aVEEQGDDQDsEkSkPAC	D4ABT8	N-Term(iTRAQ4plex); S11(Phospho); K13(iT
ekEPAEGNEsSEAPsPVN	Q6QRP0;A0A0G2K3Q5	N-Term(iTRAQ4plex); K2(iTRAQ4plex); S10(
aAPEDADsPPAVDVIPVDI	D3ZLG1	N-Term(iTRAQ4plex); S8(Phospho); K24(iTR
sPAEAksPAEAk	F1LRZ7	N-Term(iTRAQ4plex); K6(iTRAQ4plex); S7(P
dIEGFLEENQNkLsPQELT	D3ZHV2	N-Term(iTRAQ4plex); K12(iTRAQ4plex); S14
vTLDPELEEALASAsDTEL	P70566	N-Term(iTRAQ4plex); S15(Phospho); K36(iT
gPQLPTVSPSPPVmIPTV	F1LQZ9	N-Term(iTRAQ4plex); M14(Oxidation); S27(P
tVIIEQSWGsPk	P63039	N-Term(iTRAQ4plex); S10(Phospho); K12(iT
nLAkPGVTStsDSEEDDD(	Q566D6	N-Term(iTRAQ4plex); K4(iTRAQ4plex); T10(
vVEPANsDsDSELGNIPk	Q14TE9	N-Term(iTRAQ4plex); S7(Phospho); S9(Phos
sPAEAksPVEVksPEk	F1LRZ7	N-Term(iTRAQ4plex); S1(Phospho); K6(iTRA
aELGMNDsPSQSPPVk	G3V9U0	N-Term(iTRAQ4plex); S8(Phospho); K16(iTR
sSsVESLSPGGDLFGLGII	G3V6M0	N-Term(iTRAQ4plex); S3(Phospho); K20(iTR
tSLAAEENDssSEGPEEEI	D3ZKX8	N-Term(iTRAQ4plex); S10(Phospho); S11(Ph
iGIVGGcQEyTGAPYFAGI	D4AAT7	N-Term(iTRAQ4plex); C7(Carbamidomethyl);
nLsSEEVARPR	F1M1Y0	N-Term(iTRAQ4plex); S3(Phospho)
aTQPsLTELTTLk	G3V849	N-Term(iTRAQ4plex); S5(Phospho); K13(iTR
sRDESASETstPsEHSAAF	D3ZFD0	N-Term(iTRAQ4plex); S10(Phospho); T11(Ph
msPkPELTEEQk	G3V9W0	N-Term(iTRAQ4plex); S2(Phospho); K4(iTRA
kVAELEEEQsQGsSSYsD	P34926;Q63330	K1(iTRAQ4plex); N-Term(iTRAQ4plex); S10(
kLsSAMSAAk	O88989	N-Term(iTRAQ4plex); K1(iTRAQ4plex); S3(P
kEQSDISVsPR	A0A0G2JZB4	N-Term(iTRAQ4plex); K1(iTRAQ4plex); S9(P
dkPMYDEIFYTLsPVDGk	Q8R491	N-Term(iTRAQ4plex); K2(iTRAQ4plex); S13(
aFHAALRNsPINTk	A1L108	N-Term(iTRAQ4plex); S9(Phospho); K14(iTR
fEQSSDSIssDEEELR	Q3B7K9	N-Term(iTRAQ4plex); S9(Phospho); S10(Pho
kETEsEAEDDNLDDLER	B2RYB3	N-Term(iTRAQ4plex); K1(iTRAQ4plex); S5(P
vDsTTcLFPVEEk	P82808	N-Term(iTRAQ4plex); S3(Phospho); C6(Carb
eSPSEGsLQPsSIQIFANT	Q62962	N-Term(iTRAQ4plex); S7(Phospho); S11(Pho
dLYPFTVNtSNQGSR	A0A0G2K939	N-Term(iTRAQ4plex); T9(Phospho)
tRtPsPTSSk	P52481	N-Term(iTRAQ4plex); T3(Phospho); S5(Phos
nSWDsPAFSNDVIR	A0A0G2JUG7	N-Term(iTRAQ4plex); S5(Phospho)
eIEVDSSPSVLEILDTAGT	P61227	N-Term(iTRAQ4plex); S24(Phospho)
sPVVSGDtSPR	D4A1Q2	N-Term(iTRAQ4plex); T8(Phospho)
vPSAPGQEsPVPDTESTA	P34926	N-Term(iTRAQ4plex); S9(Phospho)
sGGIVIsPFRLEELTNR	D4AEK9	N-Term(iTRAQ4plex); S7(Phospho)
eksPELPEPSVR	B2RYB3;A0A0G2K4F6	N-Term(iTRAQ4plex); K2(iTRAQ4plex); S3(P
rGSSAAAPPGsPPPGR	D4A3D9	N-Term(iTRAQ4plex); S11(Phospho)
rFtPEEEMFk	A0A0G2K6R9;F1M9N9	N-Term(iTRAQ4plex); T3(Phospho); K10(iTR
eNQSEGkGsDsDSEGDNI	Q793F9	N-Term(iTRAQ4plex); K7(iTRAQ4plex); S9(P
nVIIWGNHsSTQYPDVNH	O88989	N-Term(iTRAQ4plex); S9(Phospho); K20(iTR
sTSLLSsPDSEPR	D4A8L4	N-Term(iTRAQ4plex); S7(Phospho)
sDENVLDsPk	F1LVK0	N-Term(iTRAQ4plex); S8(Phospho); K10(iTR
lEFIQQsPTR	D3ZZZ0	N-Term(iTRAQ4plex); S7(Phospho)
aQcETLsPDGLPEEQPQT	A0A0G2JVW5	N-Term(iTRAQ4plex); C3(Carbamidomethyl);
fQSVPAQPGQtSPLLQYF(	F1M779	N-Term(iTRAQ4plex); T11(Phospho); K29(iT
wVHFsDAsPEHVtPELTPF	F1M1Y0	N-Term(iTRAQ4plex); S5(Phospho); S8(Phos
sGSSSVAAMk	F2Z3T8	N-Term(Acetyl); S1(Phospho); K10(iTRAQ4p
eSRtPEELTILGETQEEED	D4A3H5	N-Term(iTRAQ4plex); T4(Phospho)
sPPDQSAVPNtPPSTPVk	A0A0G2JSM7;D3ZZ99	N-Term(iTRAQ4plex); T11(Phospho); K18(iT
dALRsLTDLNQDPSR	G3V864	N-Term(iTRAQ4plex); S5(Phospho)
sLsPGGAALGYR	M0R3Z8	N-Term(iTRAQ4plex); S3(Phospho)
qGtSSPDLPTWVk	D4A1J3	N-Term(iTRAQ4plex); T3(Phospho); K13(iTR
sPGTPAGEGSGsPPkWQ	A0A0G2JSM7;D3ZZ99	N-Term(iTRAQ4plex); S12(Phospho); K15(iT
tTTSEELEDMLESGNPAIF	P32851	N-Term(iTRAQ4plex); T1(Phospho); M25(Ox

nVEPsskNLMHFELsAEsQ	A0A0G2KB96	N-Term(iTRAQ4plex); S5(Phospho); S6(Phos
aAPQsPSVPk	F1LS02	N-Term(iTRAQ4plex); S5(Phospho); K10(iTR
gLEQLTksPVEFVEYNk	F1M084	N-Term(iTRAQ4plex); K7(iTRAQ4plex); S8(P
sSSGLATYsPPMGPVSEF	A0A0G2JWL3	N-Term(iTRAQ4plex); S9(Phospho)
asPcLDR	A0A0G2JZ56	N-Term(iTRAQ4plex); S2(Phospho); C4(Carb
ssSEITLSEcDVEEPGDPR	F1LYG2	N-Term(iTRAQ4plex); S2(Phospho); C10(Car
aRsmsPPPk	G3V6S8	N-Term(iTRAQ4plex); S3(Phospho); M4(Oxid
lLQTcFSsPTDDSmDR	Q6IMX7	N-Term(iTRAQ4plex); C5(Carbamidomethyl);
gLNLDGTPALSTLGGFSP	Q9R1Q2	N-Term(iTRAQ4plex); S20(Phospho); K21(iT
gGNVFEALIQDEsEEEkEE	Q6MG08	N-Term(iTRAQ4plex); S13(Phospho); K17(iT
tNPPTQkPPsPPVSGR	Q9QZM5	N-Term(iTRAQ4plex); K7(iTRAQ4plex); S10(
rsPsPAPPPPPPPPPR	B2RYB3;A0A0G2K4F6	N-Term(iTRAQ4plex); S2(Phospho); S4(Phos
qGEDNsITQDTEDLEk	Q9Z1W6	N-Term(iTRAQ4plex); S6(Phospho); K16(iTR
eTSPtRGEPVGGQkEPVP	P34926	N-Term(iTRAQ4plex); T5(Phospho); K14(iTR
nsLTQEV	Q63135	N-Term(iTRAQ4plex); S2(Phospho)
gVsPPPSPFFSAILAAFQP	C0IXW5	N-Term(iTRAQ4plex); S3(Phospho); C22(Car
gFsYFGEDLMP	F1LMV8	N-Term(iTRAQ4plex); S3(Phospho)
ySTDVsVDEVk	P10860	N-Term(iTRAQ4plex); S6(Phospho); K11(iTR
tSSkEssPVPsPTSDR	G3V6S0	N-Term(iTRAQ4plex); K4(iTRAQ4plex); S6(P
rDVYLsPR	A0A0A0MXW9;A0A0G2K8	N-Term(iTRAQ4plex); S6(Phospho)
aSVGAMGGGVGASSSGG	A0A0G2K7W7	N-Term(iTRAQ4plex); S27(Phospho); K42(iT
hQPAAsPVVVR	Q63327	N-Term(iTRAQ4plex); S6(Phospho)
dSGSDEDFLMEDDDDsD	A0A0G2K7X3	N-Term(iTRAQ4plex); S16(Phospho); K22(iT
eVAEVsPLsAANMSIAVk	A0A0G2JXK1	N-Term(iTRAQ4plex); S6(Phospho); S9(Phos
hSTNAEsILGTSSDLDEET	A0A0G2K0M5	N-Term(iTRAQ4plex); S7(Phospho); K26(iTR
ssSAPNVHINTIEPVNIDDI	F1M9C3	N-Term(iTRAQ4plex); S2(Phospho)
eQSESVNTVPDsPSk	M0RBY8	N-Term(iTRAQ4plex); S12(Phospho); K15(iT
mELSPRsPPEMLESDcPS	Q01066	N-Term(Acetyl); S7(Phospho); C16(Carbamid
rtSPSkPSSAPALRPGPk	A0A0G2JW88	N-Term(iTRAQ4plex); T2(Phospho); K6(iTRA
msGFIYQGk	Q923I5	N-Term(iTRAQ4plex); S2(Phospho); K9(iTRA
gGssGEELEDEEPVkk	A0A0G2JYC7	N-Term(iTRAQ4plex); S3(Phospho); S4(Phos
fIDkDQQPsGSEGEDDDA	A0A0G2JZB6	N-Term(iTRAQ4plex); K4(iTRAQ4plex); S9(P
aEEEGGSEEEVGDksPQE	P12839	N-Term(iTRAQ4plex); K14(iTRAQ4plex); S15
tPSPkEEDEEAEsPPEkk	A0A0G2K7X3	N-Term(iTRAQ4plex); K5(iTRAQ4plex); S13(
dksPSEGDVTPPk	D3ZBU7	N-Term(iTRAQ4plex); K2(iTRAQ4plex); S3(P
fIDkDQQPsGsEGEDDDAI	A0A0G2JZB6	N-Term(iTRAQ4plex); K4(iTRAQ4plex); S9(P
vQGEAVSNIQENTQTPTV	B2RYX0	N-Term(iTRAQ4plex); S22(Phospho); K35(iT
nkPSATDsEGEDDEDmTk	Q6MG08	N-Term(iTRAQ4plex); K2(iTRAQ4plex); S8(P
nEEPVRsPER	D3ZJU5	N-Term(iTRAQ4plex); S7(Phospho)
iEkLPsISDLDSIFGPVLSP	F1M1Y0	N-Term(iTRAQ4plex); K3(iTRAQ4plex); S6(P
gLLsILR	P37996	N-Term(iTRAQ4plex); S4(Phospho)
mQFsFEGPEk	A0A0G2JUG7	N-Term(iTRAQ4plex); M1(Oxidation); S4(Pho
rNsDSAGPFAVPDHLR	D3ZSP7	N-Term(iTRAQ4plex); S3(Phospho)
ePAISSQNsPEAR	Q4KLL0	N-Term(iTRAQ4plex); S9(Phospho)
dMDEPsPVPNVEEVTLPk	D4A510	N-Term(iTRAQ4plex); S6(Phospho); K18(iTR
kLEkEEEEGISQESsEEEQ	Q8K585	N-Term(iTRAQ4plex); K1(iTRAQ4plex); K4(iT
eLEGPIYsR	B5DEJ1	N-Term(iTRAQ4plex); S8(Phospho)
nSSAltSPk	G3V9G3;F1LNI8	N-Term(iTRAQ4plex); T6(Phospho); K9(iTRA
skPIPIMPAsPQk	O35303	N-Term(iTRAQ4plex); K2(iTRAQ4plex); S10(
vSNPSDsDGEDEDVR	Q76KC6	N-Term(iTRAQ4plex); S7(Phospho)
nLGGAYsVTYRDk	A0A0G2JU16	N-Term(iTRAQ4plex); S7(Phospho); K13(iTR
ePTSASESENIAAIPSPVtS	Q3ZAU5	N-Term(iTRAQ4plex); T19(Phospho)
tPTPLMANDSTEtSEAGEE	M0R3N4	N-Term(iTRAQ4plex); T13(Phospho); K30(iT
mEDsMDMDMSPLRPQNY	P13084	N-Term(Acetyl); M1(Oxidation); S4(Phospho)
iFSIAsPAk	Q561R9	N-Term(iTRAQ4plex); S6(Phospho); K9(iTRA

tLsGEEEAESVGVASR	Q66H43	N-Term(iTRAQ4plex); S3(Phospho)
sISGASSGLSTsPLSSPR	M9MMM8	N-Term(iTRAQ4plex); S12(Phospho)
sMsDPkPLsPTADESSR	Q9JKS6;D3Z9C7	N-Term(iTRAQ4plex); S3(Phospho); K6(iTRA
qPLLLsEDEEDTkR	B5DFC8	N-Term(iTRAQ4plex); S6(Phospho); K13(iTR
gNVLASHSPMsPENIk	F1M4N6	N-Term(iTRAQ4plex); S11(Phospho); K16(iT
gPRPDLEtSPGAAAR	D3ZC15	N-Term(iTRAQ4plex); T8(Phospho)
skSPPPPEEEAkDEEEDQ	D4ABT8	N-Term(iTRAQ4plex); S1(Phospho); K2(iTRA
sTGDITAAGVtEANkEPRS	Q3ZB99	N-Term(iTRAQ4plex); T11(Phospho); K15(iT
rSsLDGSESAk	D4AAS1	N-Term(iTRAQ4plex); S3(Phospho); K11(iTR
eGVILTNESVAsPEQPEEI	D4A3S8	N-Term(iTRAQ4plex); S12(Phospho); K22(iT
fFVAVPGQVVSPQsSSSG	E9PSJ4	N-Term(iTRAQ4plex); S14(Phospho); K25(iT
rRPsSPAPGPSQPATDSR	B2RZ23	N-Term(iTRAQ4plex); S4(Phospho)
lHSQDPLLsPER	D4A4M0	N-Term(iTRAQ4plex); S9(Phospho)
vEAkEEsEESDEDMGFGL	P19945	N-Term(iTRAQ4plex); K4(iTRAQ4plex); S7(P
sPSDLsPEsPVLSSPPk	A0A0G2K8P5	N-Term(iTRAQ4plex); S6(Phospho); S9(Phos
aSsPQTEPLPR	F1LMT8	N-Term(iTRAQ4plex); S3(Phospho)
dkEDLVcSAALHsPQESPV	F1LQN3	N-Term(iTRAQ4plex); K2(iTRAQ4plex); C7(C
sVSESHTPcPSESTSDTV	D3Z994	N-Term(iTRAQ4plex); S1(Phospho); C9(Carb
gPPEGSETMPYIDEsPTM	A0A0G2JTR4	N-Term(iTRAQ4plex); S15(Phospho)
eGcPsPPGQTNk	Q99PD6	N-Term(iTRAQ4plex); C3(Carbamidomethyl);
rLDEDLAGycR	P13233	N-Term(iTRAQ4plex); Y9(Phospho); C10(Ca
kPGGGDLPLHsAsDDETP	D4A0X3	K1(iTRAQ4plex); N-Term(iTRAQ4plex); S11(
vAAPSSsPSAEAEYWR	D3ZF86	N-Term(iTRAQ4plex); S7(Phospho)
nGPsPTTPQTPGPQk	D4A1G8	N-Term(iTRAQ4plex); S4(Phospho); K15(iTR
qEAVQEVsFDEEPGPTGA	D3ZL30	N-Term(iTRAQ4plex); S8(Phospho); K21(iTR
lIPLDEEcAMDETPYVETL	A0A0G2K3G9	N-Term(iTRAQ4plex); C8(Carbamidomethyl);
sPDLGEYDPLTQADsDEs	D3ZWJ9	N-Term(iTRAQ4plex); S15(Phospho); S18(Ph
lARPFPTGtPPPLPPk	F1M1Y0	N-Term(iTRAQ4plex); T9(Phospho); K16(iTR
nISLSsEEEAEGLAGHPR	D3ZKQ4	N-Term(iTRAQ4plex); S6(Phospho)
aAFscDk	M0R660;D3ZWV2;E9PTN	N-Term(iTRAQ4plex); S4(Phospho); C5(Carb
aMSMASILTNTMEELEEs	A0A0G2K207	N-Term(iTRAQ4plex); S18(Phospho); K21(iT
mNQLLSRtPALsPQRPLT	G3V733	N-Term(iTRAQ4plex); M1(Oxidation); T8(Pho
lQEETGEEEARPEsGLkG	F1M9X4	N-Term(iTRAQ4plex); S14(Phospho); K17(iT
sLsDSESDDSk	Q5RJK5	N-Term(iTRAQ4plex); S3(Phospho); K11(iTR
sSVSDLVNSLTSEmLMLS	Q62747	N-Term(iTRAQ4plex); M14(Oxidation); S21(P
aAAGtGGSLR	F7FLS6	N-Term(iTRAQ4plex); T5(Phospho)
aLLsPEDYQR	Q6P7C9	N-Term(iTRAQ4plex); S4(Phospho)
sQEEsPVPQPR	Q3ZB99	N-Term(iTRAQ4plex); S5(Phospho)
eLGsLEEDANPDEEGVQk	B2RYF8	N-Term(iTRAQ4plex); S4(Phospho); K18(iTR
nSSMGsPVNQQPk	D4AAI7	N-Term(iTRAQ4plex); S6(Phospho); K13(iTR
gsYMEVEDNRSQVEtEDL	A0A0H2UHZ1	N-Term(iTRAQ4plex); S2(Phospho); T15(Pho
rTEGYAAFQEDssGDEAE	Q5FVT1	N-Term(iTRAQ4plex); S12(Phospho); S13(Ph
tEVcPWESHGQsPLEDEN	A0A0A0MY13	N-Term(iTRAQ4plex); C4(Carbamidomethyl);
kFQEQEcPPsPEPTR	A0A0G2K508	K1(iTRAQ4plex); N-Term(iTRAQ4plex); C7(C
gSLPPAALESSDstNTTIEI	F1LNI8	N-Term(iTRAQ4plex); S13(Phospho); T14(Ph
dVmSDETNNEETEsPSQE	F1LRL9	N-Term(iTRAQ4plex); M3(Oxidation); S14(Ph
eIEVDSSPSVLEILDTAGT	P61227	N-Term(iTRAQ4plex); S24(Phospho); M25(O
sRsPVDSPVPASMFAPER	D2XV59	N-Term(iTRAQ4plex); S3(Phospho); S19(Pho
gAsSSsGVVVQVR	D4A4F9	N-Term(iTRAQ4plex); S3(Phospho); S6(Phos
rPTTPGPTPTtPPQPPDPS	Q6MFY6	N-Term(iTRAQ4plex); T11(Phospho)
sEGsPVLPHEPSk	D3ZZQ0	N-Term(iTRAQ4plex); S4(Phospho); K13(iTR
tQEScGIAPLTPSQsPkPE	D3ZVM5	N-Term(iTRAQ4plex); C5(Carbamidomethyl);
nDGVkESsEsTNTTIEDED	P11275	N-Term(iTRAQ4plex); K5(iTRAQ4plex); S8(P
lLQTTNNsPMNSkPQQIk	D2XV59	N-Term(iTRAQ4plex); S8(Phospho); K13(iTR
nAEEESEsEAEEGD	Q6P7P5	N-Term(iTRAQ4plex); S8(Phospho)

Information Classification: General

eSVAsGDDRAEEDmDEA	P15205;F1LRL9	N-Term(iTRAQ4plex); S5(Phospho); M14(Ox
vDGPRsPSYGR	D4A9L2	N-Term(iTRAQ4plex); S6(Phospho)
sFFtDGSLDSWGTSEDAE	F1M2D4	N-Term(iTRAQ4plex); T4(Phospho); K22(iTR
gSLPPAALEPQTTVIHNPV	G3V9G3	N-Term(iTRAQ4plex); K23(iTRAQ4plex); S26
eLPPsPEkk	A0A0G2JW88	N-Term(iTRAQ4plex); S5(Phospho); K8(iTRA
tSFSVGsDDELGPIRk	A0A0G2KB46	N-Term(iTRAQ4plex); S7(Phospho); K16(iTR
kVPVPEGLDLDAWINEPF	B5DFK6	K1(iTRAQ4plex); N-Term(iTRAQ4plex); S19(
gTGPPsPTkDR	D4AE85	N-Term(iTRAQ4plex); S6(Phospho); K9(iTRA
yAPttTPsPEDGFEVQPLH	P28572	N-Term(iTRAQ4plex); T4(Phospho); T5(Phos
fLAAVQLPDGFNLLcStPP	A0A0G2JXT9	N-Term(iTRAQ4plex); C15(Carbamidomethyl
sPQGLELPLHNR	A0A0G2K6R9;F1M9N9	N-Term(iTRAQ4plex); S1(Phospho)
vTmILQSPsFREELEGLIQ	F8WFS9	N-Term(iTRAQ4plex); M3(Oxidation); S9(Pho
aAFDDAIAELDTLsEESYk	P62260	N-Term(iTRAQ4plex); S14(Phospho); K19(iT
lSLGsDDEADLVk	Q2IBD4	N-Term(iTRAQ4plex); S5(Phospho); K13(iTR
tEGDSPAAALsPQmHQES	A0A0G2K6R9;F1M9N9;A0	N-Term(iTRAQ4plex); S11(Phospho); M14(O
aSAAEGSEAsPPSLR	D4A5J1	N-Term(iTRAQ4plex); S10(Phospho)
skPIPImPAsPQk	O35303	N-Term(iTRAQ4plex); K2(iTRAQ4plex); M7(O
aRsMsPPPk	G3V6S8	N-Term(iTRAQ4plex); S3(Phospho); S5(Phos
kADIDQsPVSk	G3V6Y9	N-Term(iTRAQ4plex); K1(iTRAQ4plex); S7(P
ySDTtDsDPENEPFDEDQ	O54857	N-Term(iTRAQ4plex); T5(Phospho); S7(Phos
eLEEEWVIVsDEEIEEAk	A0A0G2K6R9;F1M9N9	N-Term(iTRAQ4plex); S10(Phospho); K18(iT
lLDPEDVDVSsPDEk	D3ZC56	N-Term(iTRAQ4plex); S11(Phospho); K15(iT
gTLDEEDEEADsDTDDID	B1WBZ7	N-Term(iTRAQ4plex); S12(Phospho)
vSQSGLsPER	Q8K4Y6	N-Term(iTRAQ4plex); S7(Phospho)
iLGAWLAEETSsLRk	O35095	N-Term(iTRAQ4plex); S12(Phospho); K15(iT
eGLDISHLQGQEELLSSQ	Q9R1N0	N-Term(iTRAQ4plex); K20(iTRAQ4plex); S21
vVSMLQGSGAMPTLVVE	F1LU68	N-Term(iTRAQ4plex); S32(Phospho)
eSETFSDSsPIEIIDEFPTF	F1LQN3	N-Term(iTRAQ4plex); S9(Phospho); K24(iTR
aGSATPLsPTR	D3ZLC1	N-Term(iTRAQ4plex); S8(Phospho)
aEEAkDEPPsEGEAEEEE	P19527	N-Term(iTRAQ4plex); K5(iTRAQ4plex); S10(
aETSESSGSAPAVPEASA	A0A0G2JWK2	N-Term(iTRAQ4plex); S19(Phospho); K21(iT
lHFsVYDFDR	A0A0G2JSR2	N-Term(iTRAQ4plex); S4(Phospho)
mNQLLSRtPALsPQRPLT	G3V733	N-Term(iTRAQ4plex); T8(Phospho); S12(Pho
aFSFPVsPER	M0R4J7	N-Term(iTRAQ4plex); S7(Phospho)
vTEFNNVTPLsEEEVASIk	A0A0G2JW88	N-Term(iTRAQ4plex); S11(Phospho); K19(iT
gASSSsGVVVQVR	D4A4F9	N-Term(iTRAQ4plex); S6(Phospho)
gAAQNIIPAStGAAk	M0R660	N-Term(iTRAQ4plex); T11(Phospho); K15(iT
lNQLESSLLEEVsDEDTLk	D3ZL75	N-Term(iTRAQ4plex); S13(Phospho); K19(iT
rTsMGGTQQQFVEGVR	A0A0G2JT93	N-Term(iTRAQ4plex); S3(Phospho)
sPESPSsPVSSETTSSFk	Q5QD51	N-Term(iTRAQ4plex); S7(Phospho); K18(iTR
sVVEGISsQPGSSPSGTV	F1M3D2	N-Term(iTRAQ4plex); S8(Phospho)
eMNNYsPGR	A0A0G2K9M8	N-Term(iTRAQ4plex); S6(Phospho)
fESAFLSHVVSQHQsLLGI	P15999	N-Term(iTRAQ4plex); S15(Phospho)
rGsRSsVEPk	A0A0G2K2M9	N-Term(iTRAQ4plex); S3(Phospho); S6(Phos
qGLQcQVcsFVVHR	P63319	N-Term(iTRAQ4plex); C5(Carbamidomethyl);
eTsPETSLIQDEVALk	A0A0U1RRX4;F1MAQ5	N-Term(iTRAQ4plex); S3(Phospho); K16(iTR
sDsPSPLR	F1M9Y7	N-Term(iTRAQ4plex); S3(Phospho)
wHTLTNEIPGAVLsD	A0A0G2K8B5	N-Term(iTRAQ4plex); S14(Phospho)
qEVTGSSAVsPIRk	Q9Z1T4	N-Term(iTRAQ4plex); S10(Phospho); K14(iT
lPPsPTR	D4A732	N-Term(iTRAQ4plex); S4(Phospho)
eTQQEPGEEsPVPSEEHL	X4YHC6	N-Term(iTRAQ4plex); S10(Phospho); K23(iT
eSSDsTNtTIEDEDAk	G3V9G3	N-Term(iTRAQ4plex); S5(Phospho); T8(Phos
iDVESTELAssESPQAAEL	A0A0G2K5Z4	N-Term(iTRAQ4plex); S10(Phospho); S11(Ph
sAsADNLILPR	F1MA89	N-Term(iTRAQ4plex); S3(Phospho)
qRsIEAER	D3ZV52	N-Term(iTRAQ4plex); S3(Phospho)

Information Classification: General

vLkIsEEDELDTk	Q5U318	N-Term(iTRAQ4plex); K3(iTRAQ4plex); S5(P
iPAsARSPVELQNR	A0A0G2K526	N-Term(iTRAQ4plex); S4(Phospho)
sSscESLGAQLPAAR	D3ZA31	N-Term(iTRAQ4plex); S3(Phospho); C4(Carb
eGLDISHLQGQEELLsSQ	Q9R1N0	N-Term(iTRAQ4plex); S16(Phospho); K20(iT
lPsPTPENk	Q6P730	N-Term(iTRAQ4plex); S3(Phospho); K9(iTRA
aPEAAVsDDGkSDDELLS	F1M031	N-Term(iTRAQ4plex); S7(Phospho); K11(iTR
kEEsEESEDDMGFGLFD	A0A0G2K4Q1	N-Term(iTRAQ4plex); K1(iTRAQ4plex); S4(P
tLSkEsPSHGLELSAPEk	Q924N5	N-Term(iTRAQ4plex); K4(iTRAQ4plex); S6(P
sPDRTEEVLSPDGsPskSI	Q62847;A0A0G2JW28	N-Term(iTRAQ4plex); S14(Phospho); S16(Ph
vNNsSLIGVGYTQTLRPG	P81155	N-Term(iTRAQ4plex); S4(Phospho); K20(iTR
rEHANIDAQSGSQASNPS	A0A0G2JWM2	N-Term(iTRAQ4plex); S22(Phospho)
tNQAILsQLYQSPNRR	A0A1B0GWQ5	N-Term(iTRAQ4plex); S7(Phospho)
aETEEAEEPEEDGEDNVs	P15205;F1LRL9	N-Term(iTRAQ4plex); S18(Phospho); K23(iT
tPSPEPIDkDFYSEFGDk	Q4G017	N-Term(iTRAQ4plex); T1(Phospho); K9(iTRA
vIsLTAcLVNQNLR	P01830	N-Term(iTRAQ4plex); S3(Phospho); C7(Carb
tPALsPQRPLTTQQPQSG	G3V733	N-Term(iTRAQ4plex); S5(Phospho); K21(iTR
qLsPEsLGTLQFGELNLGI	P34926;Q63330	N-Term(iTRAQ4plex); S3(Phospho); S6(Phos
sAEALGPGALVSPR	F1M2D4	N-Term(iTRAQ4plex); S1(Phospho)
kIAELEEERsQGstSNSDW	F1LRL9	K1(iTRAQ4plex); N-Term(iTRAQ4plex); S10(
qTLINIPSLNDsDSEVEDIS	Q3ZB99	N-Term(iTRAQ4plex); S12(Phospho)
kSDsNAsFLR	A0A0G2K6R9	K1(iTRAQ4plex); N-Term(iTRAQ4plex); S4(P
yAGESFPGIYDALFDINNk	P70627	N-Term(iTRAQ4plex); Y1(Phospho); K19(iTR
gNAEGsSDEEGkLVIDEP	Q8VHK7	N-Term(iTRAQ4plex); S6(Phospho); K12(iTR
eESGEQLsPPVEAGQGPI	F7IXA3	N-Term(iTRAQ4plex); S8(Phospho)
aGkEsEEEEEDTEQNk	F1LPS3	N-Term(iTRAQ4plex); K3(iTRAQ4plex); S5(P
lGDsLQDLYR	Q9Z1T4	N-Term(iTRAQ4plex); S4(Phospho)
qksDAEEDGGTGsQDEEI	P35565	N-Term(iTRAQ4plex); K2(iTRAQ4plex); S3(P
sSkGsPTGSSPNNASELS	Q4KLM7	N-Term(iTRAQ4plex); K3(iTRAQ4plex); S5(P
gVLsSPSLAFTPPIR	Q68FY1	N-Term(iTRAQ4plex); S4(Phospho)
gANEsLER	P23565;G3V8Q2	N-Term(iTRAQ4plex); S5(Phospho)
mLAESDEsGDEESVSQTI	D4A9D8	N-Term(iTRAQ4plex); S8(Phospho); K19(iTR
nVSksDSDLIAYPSk	F1M2J2	N-Term(iTRAQ4plex); K4(iTRAQ4plex); S5(P
aAETVPDLPsPPTEAPAP	A0A0G2K9L2	N-Term(iTRAQ4plex); S10(Phospho)
sSSPsSLPALGPLWTANE	D3ZQJ3	N-Term(iTRAQ4plex); S5(Phospho); K25(iTR
gADDAADADTAIINAEGG	A0A0G2JUT1	N-Term(iTRAQ4plex); S22(Phospho); K25(iT
tNTGsAMLEQIAMSDR	P09951;G3V733	N-Term(iTRAQ4plex); S5(Phospho)
iYASsSPPDTGQR	Q4QRB9	N-Term(iTRAQ4plex); S5(Phospho)
eHGcSSsDDDTDVDVEGI	A2VD12	N-Term(iTRAQ4plex); C4(Carbamidomethyl);
tLMLPLtEGSLcLR	D3Z899	N-Term(iTRAQ4plex); T7(Phospho); C12(Car
vASLQEELQDIsPEEEVAY	D3ZKP1	N-Term(iTRAQ4plex); S12(Phospho); K20(iT
ySLsPSk	D4A054	N-Term(iTRAQ4plex); S4(Phospho); K7(iTRA
nEAAVPAAAAGsPVPVIAI	Q63014	N-Term(iTRAQ4plex); S12(Phospho); K35(iT
eHANIDAQSGSQASNPS	A0A0G2JWM2	N-Term(iTRAQ4plex); S21(Phospho)
eAGALDSLPGIPLATSSEI	P10683	N-Term(iTRAQ4plex); S23(Phospho)
lLEQEALMEQQDPksPSH	A0A0G2K132	N-Term(iTRAQ4plex); K14(iTRAQ4plex); S15
qMGsTEGESDVPVLVTSE	A0A0G2K6R9;F1M9N9	N-Term(iTRAQ4plex); S4(Phospho)
sSsPVTELTAR	A0A0G2K2M9	N-Term(iTRAQ4plex); S3(Phospho)
tAPSSPLTsPSDTR	G3V9L1	N-Term(iTRAQ4plex); S9(Phospho)
dVMTDETNNEETEsPSQE	P15205	N-Term(iTRAQ4plex); S14(Phospho); K24(iT
vLGATLsPELIQk	F1M9V7	N-Term(iTRAQ4plex); S7(Phospho); K13(iTR
gHYEFPVVDDLsSGDTk	D3Z9D0	N-Term(iTRAQ4plex); S12(Phospho); K17(iT
aEkPSALNsEEEAHEQsTI	G3V7X5	N-Term(iTRAQ4plex); K3(iTRAQ4plex); S9(P
lcYVALDFEQEMATAASsS	P60711;P63259	N-Term(iTRAQ4plex); C2(Carbamidomethyl);
gAYDAQGtLSk	P02688	N-Term(iTRAQ4plex); T8(Phospho); K11(iTR
tGNAYLTAELsTPDGsVAI	D3ZQG6	N-Term(iTRAQ4plex); S11(Phospho); S16(Ph

tsRGSEPDDAQLQPPEDD	Q5FVJ4	N-Term(iTRAQ4plex); S2(Phospho)
tSGAkDDVsPEEQGk	D4A1J3	N-Term(iTRAQ4plex); K5(iTRAQ4plex); S9(P
rPTVLILDEAtSALDAESEF	A0A0H2UHH3	N-Term(iTRAQ4plex); T11(Phospho)
gTGQsDDsDIWDDTALIk	Q2KP10	N-Term(iTRAQ4plex); S5(Phospho); S8(Phos
eQScDLAPSGEVsPVkPL(	D4A2G4	N-Term(iTRAQ4plex); C4(Carbamidomethyl);
yPSGsSSPPPR	A0A0G2K6Z8	N-Term(iTRAQ4plex); S5(Phospho)
sAYQDYDsDsDVPEELkR	F1LSG8	N-Term(iTRAQ4plex); S8(Phospho); S10(Pho
kPPAPPsPVQSQSPSTNV	B2RYB3;A0A0G2K4F6	N-Term(iTRAQ4plex); K1(iTRAQ4plex); S7(P
gTNEsLER	P47819;P31000	N-Term(iTRAQ4plex); S5(Phospho)
aAPALtPPDR	P63319	N-Term(iTRAQ4plex); T6(Phospho)
sSsPVGLAk	D4ACF1	N-Term(iTRAQ4plex); S3(Phospho); K9(iTRA
iVEPEVVGEsDsEVEGDA\	D4ACM9	N-Term(iTRAQ4plex); S10(Phospho); S12(Ph
aLELEPLELEGSLAGsPP(	P0C6T3	N-Term(iTRAQ4plex); S16(Phospho)
tVSASsTGDLPk	B1WBT4	N-Term(iTRAQ4plex); S6(Phospho); K12(iTR
fVEWLQNAEEESESEGEE	Q9WTT7	N-Term(iTRAQ4plex); S19(Phospho)
aksVLk	A0A0G2K2S2	N-Term(iTRAQ4plex); K2(iTRAQ4plex); S3(P
sADcsVEEEPWk	P31596	N-Term(iTRAQ4plex); C4(Carbamidomethyl);
sEtAPAETTAPAPVEksPA	D3ZBN0	N-Term(Acetyl); T3(Phospho); K16(iTRAQ4pl
eGcGEGGksPELPGVQEI	F1LQD2	N-Term(iTRAQ4plex); C3(Carbamidomethyl);
tFTGEHVVALmPEAGsPQ	B4F774	N-Term(iTRAQ4plex); M11(Oxidation); S16(P
lDPYDsSEDDkEYVGFATI	A0A096MJN4	N-Term(iTRAQ4plex); S6(Phospho); K11(iTR
gGIDNPAITsDQEVDDkk	Q6TUE6	N-Term(iTRAQ4plex); S10(Phospho); K17(iT
sDsDYDLSPk	A0A140TAB1;F1M1T9	N-Term(iTRAQ4plex); S3(Phospho); K10(iTR
aSGQAFELILkPPsPISEAI	P21818	N-Term(iTRAQ4plex); K11(iTRAQ4plex); S14
tTGTIPsPQNLR	O88954	N-Term(iTRAQ4plex); S7(Phospho)
lTGsQEVk	O88954	N-Term(iTRAQ4plex); S4(Phospho); K8(iTRA
lVEDERsDREEtEssEGEE	G3V8V4	N-Term(iTRAQ4plex); S7(Phospho); T12(Pho
mPPLIADsPk	G3V715	N-Term(iTRAQ4plex); S8(Phospho); K10(iTR
tEQTFsPAk	D3ZZ99	N-Term(iTRAQ4plex); S6(Phospho); K9(iTRA
aLQsPEHHIDPIYEDR	F1M787	N-Term(iTRAQ4plex); S4(Phospho)
sGSIIGSR	D4A720	N-Term(iTRAQ4plex); S1(Phospho)
lGLDFPNLPYLIDGsHk	A0A0G2K4U2	N-Term(iTRAQ4plex); S15(Phospho); K17(iT
gRsEEELEASk	D3ZGN7	N-Term(iTRAQ4plex); S3(Phospho); K11(iTR
sNEQIVSEk	F1LPH1	N-Term(iTRAQ4plex); S1(Phospho); K9(iTRA
lGIAVIHGEAQDAESDLVD	Q6AZ40	N-Term(iTRAQ4plex); S23(Phospho); M26(O
gGASGPATPAPPQLsPV(	D4A919	N-Term(iTRAQ4plex); S15(Phospho)
ssPQLDPLR	A0A0G2JUK2	N-Term(iTRAQ4plex); S2(Phospho)
lQSGELsPEAVFFTYLGk	P97612	N-Term(iTRAQ4plex); S7(Phospho); K18(iTR
ssPADLVTVTDQk	P97618	N-Term(iTRAQ4plex); S2(Phospho); K13(iTR
gWLRDPNAsPGDAGEQA	P85972	N-Term(iTRAQ4plex); S9(Phospho)
gTPTPQVQAAAAPSPsPF	D3ZC31	N-Term(iTRAQ4plex); S16(Phospho); K23(iT
sPQGLSDTGYSSDGISGS	Q9JKS6;D3Z9C7	N-Term(iTRAQ4plex); S1(Phospho); K31(iTR
vETPPLSAsPPQQRPPGF	Q3B8N7	N-Term(iTRAQ4plex); S9(Phospho)
lLkPGEEPSEYtDEEDTkD	Q5XIU9	N-Term(iTRAQ4plex); K3(iTRAQ4plex); T12(
eELAsDLEEmATSSISVPk	Q5RJN5	N-Term(iTRAQ4plex); S5(Phospho); M10(Ox
aEDDGGESEGDAsEkDAI	D4A997	N-Term(iTRAQ4plex); S13(Phospho); K15(iT
yELLTAANEVIAEEIkDLMt	Q561R7	N-Term(iTRAQ4plex); Y1(Phospho); K16(iTR
tQDDLVEQNSSYVQDsPS	Q5U2U7	N-Term(iTRAQ4plex); S16(Phospho); K19(iT
rStPtPELTSk	F1M1Y0	N-Term(iTRAQ4plex); T3(Phospho); T5(Phos
qkEDVEGVGTsDGEGAA(	Q6YDN7	N-Term(iTRAQ4plex); K2(iTRAQ4plex); S11(
ePsLHDLTEFSR	A0A0G2JZ77	N-Term(iTRAQ4plex); S3(Phospho)
tDTAADGETsATEsDQEE	G3V874;A0A0G2K1Q9	N-Term(iTRAQ4plex); S10(Phospho); S14(Ph
sSSELsPEIVEk	A0A0G2K2M9	N-Term(iTRAQ4plex); S1(Phospho); S6(Phos
aAVVTsPPPTTAPHk	A0A0G2JSM7;D3ZZ99	N-Term(iTRAQ4plex); S6(Phospho); K15(iTR
sHLVNEVPVLAsPDLLSEV	A0A0G2K6R9;F1M9N9	N-Term(iTRAQ4plex); S12(Phospho); K23(iT

Information Classification: General

lFGHSQLLQsDDDIGDHQ	D3ZW49	N-Term(iTRAQ4plex); S10(Phospho)
sPAGAksPAEAk	F1LRZ7	N-Term(iTRAQ4plex); S1(Phospho); K6(iTRA
fALGLSGGsLVSMLAR	P85971	N-Term(iTRAQ4plex); S9(Phospho)
kVVEPANsDsDSELGNIPk	Q14TE9	N-Term(iTRAQ4plex); K1(iTRAQ4plex); S8(P
lFGTSPAAEATIsPPEPAP	D3ZKQ4	N-Term(iTRAQ4plex); S13(Phospho)
rPYsPEk	D3ZXL5	N-Term(iTRAQ4plex); S4(Phospho); K7(iTRA
wEPTMGEPsSSIPTTVGS	Q9Z136	N-Term(iTRAQ4plex); S9(Phospho); K23(iTR
sDFFGPSQEHSAYQTIDS	Q642A2	N-Term(iTRAQ4plex); S21(Phospho); K32(iT
aAQLQGsPAPEkGEVLGD	F1LW91	N-Term(iTRAQ4plex); S7(Phospho); K12(iTR
dAsLEELQk	A0A0G2K8P5	N-Term(iTRAQ4plex); S3(Phospho); K9(iTRA
lPEEGGSsRAEDsSEGHE	Q63259	N-Term(iTRAQ4plex); S8(Phospho); S13(Pho
lTLEGLEEDDDDDDkAsPT	A0A0G2K9B2	N-Term(iTRAQ4plex); K15(iTRAQ4plex); S17
aTAAIQVPDDIcHsPTWEA	D3ZZH5	N-Term(iTRAQ4plex); C12(Carbamidomethyl
smFAGVPTmRESsPk	D4A1D8	N-Term(iTRAQ4plex); M2(Oxidation); M9(Oxi
mLGEDsDEEDEADTTAG	D3ZTF1	N-Term(iTRAQ4plex); S6(Phospho); K18(iTR
eEPLsEEEPcTSTAVPsPE	Q9QZ86	N-Term(iTRAQ4plex); S5(Phospho); C10(Ca
aVsPTEPTPR	D3ZGZ9	N-Term(iTRAQ4plex); S3(Phospho)
aIYQGPSsPDkS	P22734	N-Term(iTRAQ4plex); S8(Phospho); K11(iTR
dYNASASTIsPPsSMEEDk	P15205;F1LRL9	N-Term(iTRAQ4plex); S10(Phospho); S13(Ph
lFDVGGQRsER	P59215	N-Term(iTRAQ4plex); S9(Phospho)
vENMSSNQDGNDsDEFM	D4AAU6	N-Term(iTRAQ4plex); S13(Phospho)
wLDEsDAEmELR	D4ABI7	N-Term(iTRAQ4plex); S5(Phospho); M9(Oxid
sLPsLEDIDPDVLDSMHSL	M9MMM8	N-Term(iTRAQ4plex); S4(Phospho); C21(Ca
eEAsDDDmEGDEAVVR	M3ZCQ2	N-Term(iTRAQ4plex); S4(Phospho); M8(Oxid
kAEHIcTHSLSPSEPAVAS	Q6RJR6	N-Term(iTRAQ4plex); K1(iTRAQ4plex); C6(C
gVGIIsEGNETVEDIAAR	P06686;P06687;P06685	N-Term(iTRAQ4plex); S6(Phospho)
vLGPsSSENQEGTLTDSM	P19814	N-Term(iTRAQ4plex); S5(Phospho); K19(iTR
kDsGSSSVFAEsPGGk	Q9QXY2	N-Term(iTRAQ4plex); K1(iTRAQ4plex); S3(P
qSQIQVFEDGADTTsPET	A0A0G2K428	N-Term(iTRAQ4plex); S15(Phospho); K25(iT
akPAAQSEEETAAsPAAS	Q4G061	N-Term(iTRAQ4plex); K2(iTRAQ4plex); S14(
tVTPASSAktsPAk	P47942	N-Term(iTRAQ4plex); K9(iTRAQ4plex); T10(
sLTEMSSDFGHsSPPPQF	F1LPJ6	N-Term(iTRAQ4plex); S12(Phospho)
tPsPPEEAsPLPSPTASPN	A0A0G2K5Z4	N-Term(iTRAQ4plex); S3(Phospho); S9(Phos
iEIIQPLLDmAAGTSNAAP	F1M994	N-Term(iTRAQ4plex); M10(Oxidation); T25(P
kVTGtLDANR	P85845	N-Term(iTRAQ4plex); K1(iTRAQ4plex); T5(P
rEsVASGDDR	P15205;F1LRL9	N-Term(iTRAQ4plex); S3(Phospho)
kFQEQEcPPsPEPTRk	A0A0G2K508	N-Term(iTRAQ4plex); K1(iTRAQ4plex); C7(C
kLsDASDER	B2RYP3	N-Term(iTRAQ4plex); K1(iTRAQ4plex); S3(P
rQEsGSSLEPPSGLALED(	F1MAQ4	N-Term(iTRAQ4plex); S4(Phospho)
eEEEGISQESsEEEQ	Q8K585	N-Term(iTRAQ4plex); S11(Phospho)
vPsAPGQEsPVPDTESTA	P34926	N-Term(iTRAQ4plex); S3(Phospho); S9(Phos
dDYLEtLSSPk	F1M1Y0	N-Term(iTRAQ4plex); T6(Phospho); K11(iTR
aVTcDIsEDEED	P19836	N-Term(iTRAQ4plex); C4(Carbamidomethyl);
aTTEEPETDEVDQVsESN	G3V7I8	N-Term(iTRAQ4plex); S15(Phospho); S19(Ph
sTPAcsPILR	D3ZYJ5	N-Term(iTRAQ4plex); C5(Carbamidomethyl);
lSSNcSGVEGDVtDEDEG	F1M324	N-Term(iTRAQ4plex); C5(Carbamidomethyl);
vTDGITksPEkR	A0A0U1RRX4;F1MAQ5	N-Term(iTRAQ4plex); K7(iTRAQ4plex); S8(P
iINLGPVHPGPLsPEPQPN	Q5PPM8	N-Term(iTRAQ4plex); S13(Phospho)
gSDTsPEAEASsGGGGVA	Q9WVR6	N-Term(iTRAQ4plex); S5(Phospho); S12(Pho
mGQAGStISNSHAQPFDF	P08050	N-Term(iTRAQ4plex); M1(Oxidation); T7(Pho
yTsDEMLER	A0A0G2K875	N-Term(iTRAQ4plex); S3(Phospho)
tVAsDsEEEVGkEESSVk	Q3SWT4	N-Term(iTRAQ4plex); S4(Phospho); S6(Phos
qPSsPGPQPR	B5DFL0	N-Term(iTRAQ4plex); S4(Phospho)
vTAsPELAEAAAGR	D3ZWA1	N-Term(iTRAQ4plex); S4(Phospho)
sPAEAksPVVAk	F1LRZ7	N-Term(iTRAQ4plex); S1(Phospho); K6(iTRA

hTPPTIGGsLPYR	F1LYA6	N-Term(iTRAQ4plex); S9(Phospho)
vTMILQSPsFREELEGLIQ	F8WFS9	N-Term(iTRAQ4plex); S9(Phospho); K23(iTR
sSPAEMDENEL	Q5XIB9	N-Term(iTRAQ4plex); S1(Phospho)
lDQPVSAPPsPR	D4AEK9	N-Term(iTRAQ4plex); S10(Phospho)
sSsSERPEPQQk	B1WBT4	N-Term(iTRAQ4plex); S3(Phospho); K12(iTR
gFSQYGVSGsPTk	D3Z9C0	N-Term(iTRAQ4plex); S10(Phospho); K13(iT
sVAssQPAkPTk	F1M4A0	N-Term(iTRAQ4plex); S4(Phospho); S5(Phos
qRsPIALPVk	F1MAQ8	N-Term(iTRAQ4plex); S3(Phospho); K10(iTR
eRDkEVsDDEAEEk	P82995	N-Term(iTRAQ4plex); K4(iTRAQ4plex); S7(P
lTYSQETcENLQEmLGEL	Q9WU70	N-Term(iTRAQ4plex); C8(Carbamidomethyl);
sSPGLYNAPVQDSLQPTA	B1VKB4	N-Term(iTRAQ4plex); S20(Phospho)
sLsAPQDk	Q3B7U0	N-Term(iTRAQ4plex); S3(Phospho); K8(iTRA
aFSIQGAPSDsGPLR	A0A0G2K5Z4	N-Term(iTRAQ4plex); S11(Phospho)
wGQPPsPTPVPRPPDAD	E9PU01	N-Term(iTRAQ4plex); S6(Phospho); S22(Pho
dLsPISk	Q8VHX0	N-Term(iTRAQ4plex); S3(Phospho); K7(iTRA
mNVPETMNEVLDMSDDE	A0A0G2K1Q7	N-Term(iTRAQ4plex); T23(Phospho); K28(iT
eLAsPVSPELR	A0A0G2JU89	N-Term(iTRAQ4plex); S4(Phospho)
iAELEEERsQGSTSNSDW	F1LRL9	N-Term(iTRAQ4plex); S9(Phospho); M19(Ox
gPPGcSPcPEsPSR	B2RYB4	N-Term(iTRAQ4plex); C5(Carbamidomethyl);
eGIARsPPEVSEVETDLG	F1LMT8	N-Term(iTRAQ4plex); S6(Phospho)
aGPLGGSsYEEEEEEEG	F1LNP8	N-Term(iTRAQ4plex); S8(Phospho)
gAIYSIPItEDGGGGSsTPE	G3V8G4	N-Term(iTRAQ4plex); T9(Phospho); S17(Pho
fQLTDsQIYEVLSVIR	P47942	N-Term(iTRAQ4plex); S6(Phospho)
aEEEGGsDGSAAEAEPR	Q4G061	N-Term(iTRAQ4plex); S7(Phospho)
tALsDVcGGSVFPAAGAG	Q5RJK6	N-Term(iTRAQ4plex); S4(Phospho); C7(Carb
aGkEsEEEEEDTEQNkk	F1LPS3	N-Term(iTRAQ4plex); K3(iTRAQ4plex); S5(P
lQSTENLLPMsPEEFDEM	F1M9D6	N-Term(iTRAQ4plex); S11(Phospho); K20(iT
gLLSQGsPLSWEETQR	P19468	N-Term(iTRAQ4plex); S7(Phospho)
qLAGGPcVGGLPSYAtFP	D3ZH40	N-Term(iTRAQ4plex); C7(Carbamidomethyl);
rGsWDSLPDTSSTDR	D4A8G3	N-Term(iTRAQ4plex); S3(Phospho)
gHWFLsPR	Q6AYN4	N-Term(iTRAQ4plex); S6(Phospho)
hLEMQFEQLQQAPASED	D3ZH78	N-Term(iTRAQ4plex); S18(Phospho); S20(Ph
lSLsDGESGEEk	A0A0G2JXZ3	N-Term(iTRAQ4plex); S4(Phospho); K12(iTR
dMSQsPTSSFVR	A0A0G2K939	N-Term(iTRAQ4plex); S5(Phospho)
rPPsPEPSAkVsEEAESQ	G3V6S0	N-Term(iTRAQ4plex); S4(Phospho); K10(iTR
sLDGEVGTGQYATTk	G3V874	N-Term(iTRAQ4plex); S1(Phospho); K15(iTR
lDSDsDEEDTQFFSMASG	D3ZT64	N-Term(iTRAQ4plex); S5(Phospho)
vmLGEtNPADSkPGTIR	P19804	N-Term(iTRAQ4plex); M2(Oxidation); T6(Pho
tLTSPADAAGFLQGSRDs	D3ZMG0	N-Term(iTRAQ4plex); S18(Phospho); K23(iT
iLLVDsPGMGDGEDEQQE	D3ZD80	N-Term(iTRAQ4plex); S6(Phospho); K23(iTR
rPEsPPSILAPPVVPTADk	A0A0G2K809	N-Term(iTRAQ4plex); S4(Phospho); K19(iTR
lSPFFTLDLsPTEDk	B5DEI5	N-Term(iTRAQ4plex); S10(Phospho); K15(iT
yVEsDDEkPTDENVNEk	A0A0G2JXZ3	N-Term(iTRAQ4plex); S4(Phospho); K8(iTRA
mLAEsDEsGDEESVSQTD	D4A9D8	N-Term(iTRAQ4plex); S5(Phospho); S8(Phos
dYNASAsTISPPSSmEED	P15205;F1LRL9	N-Term(iTRAQ4plex); S7(Phospho); M15(Ox
eTQSPAPLQNSSGDPAE	P53987	N-Term(iTRAQ4plex); S20(Phospho)
aTLsDsEIETNSATSTIFGk	A0A0G2KA27	N-Term(iTRAQ4plex); S4(Phospho); S6(Phos
lVIGsLPAHLsPHLFGGFk	M0RBD9	N-Term(iTRAQ4plex); S5(Phospho); S11(Pho
qRsPPVTk	B2RYB3;A0A0G2K4F6	N-Term(iTRAQ4plex); S3(Phospho); K8(iTRA
nsLTGEEGELAk	Q99P55	N-Term(iTRAQ4plex); S2(Phospho); K12(iTR
tAsLTSAAsIDGSR	A0A0G2JSU4	N-Term(iTRAQ4plex); S3(Phospho); S9(Phos
iYQyIQSR	D4ACC4	N-Term(iTRAQ4plex); Y4(Phospho)
nMANLSGVNGsPQSALD	G3V746	N-Term(iTRAQ4plex); S11(Phospho)
tGTGDVAtcDSPR	D3ZYR0	N-Term(iTRAQ4plex); T8(Phospho); C9(Carb
tVFAGAVPVLPAsPPPkDS	A0A0G2QC53	N-Term(iTRAQ4plex); S13(Phospho); K17(iT

Information Classification: General

aDEPSsPAEEkDEGGGk	D3ZD80	N-Term(iTRAQ4plex); S6(Phospho); K11(iTR
aDPVLLNNHSNLkPAPTV	Q5FVI4	N-Term(iTRAQ4plex); K13(iTRAQ4plex); S24
lPNSkQsPDEPLR	I6L9G6	N-Term(iTRAQ4plex); K5(iTRAQ4plex); S7(P
eDEIsPPPPNPVVk	P09456	N-Term(iTRAQ4plex); S5(Phospho); K14(iTR
sSDSSQAVITtPPPPSMPH	Q62847	N-Term(Acetyl); T11(Phospho); K20(iTRAQ4
aAEIPAVAScWVGPQVsP	A0A0G2K2M9	N-Term(iTRAQ4plex); C10(Carbamidomethyl
rSSRssPELTR	A0A0G2K2M9	N-Term(iTRAQ4plex); S5(Phospho); S6(Phos
fGGsGSQVDSAR	Q4FZT9	N-Term(iTRAQ4plex); S4(Phospho)
sIGsPESTPk	F1M031	N-Term(iTRAQ4plex); S4(Phospho); K10(iTR
tMIIsPERLDPFADGGktPD	Q9ET34	N-Term(iTRAQ4plex); S5(Phospho); K17(iTR
gAGDEVSELPAPARsPPR	F1MAS1	N-Term(iTRAQ4plex); S15(Phospho)
sASssEELINTLNFLDEAQ	F1LVX2	N-Term(iTRAQ4plex); S4(Phospho); S5(Phos
skscDDGLNTFR	F1M2D4	N-Term(iTRAQ4plex); K2(iTRAQ4plex); S3(P
sTsPPPsPEVWAESR	D4A559	N-Term(iTRAQ4plex); S3(Phospho); S7(Phos
yRPLHNAASHEGLAATsG	F1LN57	N-Term(iTRAQ4plex); S17(Phospho)
gAYVPSsPTR	Q6AY57	N-Term(iTRAQ4plex); S7(Phospho)
wAsSREETGAPVEDSPG(	O35314	N-Term(iTRAQ4plex); S3(Phospho); K20(iTR
qPsAGGEEEEk	F1LM55	N-Term(iTRAQ4plex); S3(Phospho); K11(iTR
eVsDkDRDFAAAAAk	G3V874;A0A0G2K1Q9	N-Term(iTRAQ4plex); S3(Phospho); K5(iTRA
wGQTLPTTssTDEGNPkR	A0A0G2K677	N-Term(iTRAQ4plex); S9(Phospho); S10(Pho
eTEGEEQAQYLQVTscPk	P63142	N-Term(iTRAQ4plex); S15(Phospho); C16(Ca
sSGLQPQGAPEAR	Q70AM4	N-Term(iTRAQ4plex); S1(Phospho)
eGsQGELTPANSQSR	P97834	N-Term(iTRAQ4plex); S3(Phospho)
fSIGsDEDDSPGLSIk	G3V8P8	N-Term(iTRAQ4plex); S5(Phospho); K16(iTR
tEGDsPAAALsPQMHQES	A0A0G2K6R9;F1M9N9;A0	N-Term(iTRAQ4plex); S5(Phospho); S11(Pho
iLHTLLASGEDALDFTQES	Q66HC5	N-Term(iTRAQ4plex); S28(Phospho)
dTSSNDINGGVEPTPSNV	D3ZUY8	N-Term(iTRAQ4plex); S19(Phospho); T20(Ph
lEEQADSIAGAIQSDPVEG	Q63475	N-Term(iTRAQ4plex); S20(Phospho)
rNsELFQSLISk	A6Y7S3	N-Term(iTRAQ4plex); S3(Phospho); K12(iTR
rPSPEDDLtDDENGDIQLC	A0A0G2K0B4	N-Term(iTRAQ4plex); T9(Phospho)
kEVLDAsDkEGLsPAk	Q5RKH1	N-Term(iTRAQ4plex); K1(iTRAQ4plex); S7(P
sHLVNEVPVLAsPDLLSEV	A0A0G2K6R9;F1M9N9	N-Term(iTRAQ4plex); S12(Phospho); M22(O
aHNLQDGQISDTGDPGEI	D3ZCG2	N-Term(iTRAQ4plex); S21(Phospho)
eEQsPVkAEVAEk	P15205;F1LRL9	N-Term(iTRAQ4plex); S4(Phospho); K7(iTRA
sPESLsSPAMEDLAVEWE	P34926;Q63330	N-Term(iTRAQ4plex); S1(Phospho); S6(Phos
ssTVsEDVEATVPMLQR	Q4V8I7	N-Term(iTRAQ4plex); S2(Phospho); S5(Phos
kGSLDsDNDDsDcPYSEk	Q9JI66	K1(iTRAQ4plex); N-Term(iTRAQ4plex); S6(P
sLPEIDGLSk	F1LLX6;F1LWT1	N-Term(iTRAQ4plex); S1(Phospho); K10(iTR
eFITGDVEPTDAESAWHs	Q5U2Z3	N-Term(iTRAQ4plex); S18(Phospho); K25(iT
aDEVTLsEAGSTTGPAEP	Q920Q0	N-Term(iTRAQ4plex); S7(Phospho)
tGNESGSNLsDsGSVk	A0A0G2JZF2	N-Term(iTRAQ4plex); S10(Phospho); S12(Ph
kFsSPPPLAIGTSSPVR	P28818	N-Term(iTRAQ4plex); K1(iTRAQ4plex); S3(P
sAkDsDDEEEVVHVDRDH	P61265	N-Term(iTRAQ4plex); K3(iTRAQ4plex); S5(P
kVMDsDEDDDY	D4ADF5	N-Term(iTRAQ4plex); K1(iTRAQ4plex); S5(P
kIGELEVGQMENIsPGQIII	A0A0G2K9S4	N-Term(iTRAQ4plex); K1(iTRAQ4plex); S14(
aSHsAVDITk	M0R423	N-Term(iTRAQ4plex); S4(Phospho); K10(iTR
eVksSPSEsPLMEk	Q9JJP0	N-Term(iTRAQ4plex); K3(iTRAQ4plex); S4(P
tDDVsEkTSLADQEEVR	D3ZJK8	N-Term(iTRAQ4plex); S5(Phospho); K7(iTRA
iLDQMPATPSsPMYVD	F1LP57	N-Term(iTRAQ4plex); S11(Phospho)
nRsPTLScR	F1LXQ7	N-Term(iTRAQ4plex); S3(Phospho); C8(Carb
lQDSSDPDTcSEEEVSSR	A0A1B0GWY5	N-Term(iTRAQ4plex); C10(Carbamidomethyl
gPEsPQktPPPPAPPSFEE	D3ZUJ7	N-Term(iTRAQ4plex); S4(Phospho); K7(iTRA
sLEVIPEkTsDENGD	F1M324	N-Term(iTRAQ4plex); K8(iTRAQ4plex); S10(
vLLLEANRHsPGPER	A0A0G2JZF2	N-Term(iTRAQ4plex); S10(Phospho)
rEDsPGPEVQPmDk	G3V8D6	N-Term(iTRAQ4plex); S4(Phospho); M12(Ox

Information Classification: General

eTcsPVsEGEEFSSPATN	D3ZSU3	N-Term(iTRAQ4plex); C3(Carbamidomethyl);
aTLsDSEIETNSATSTIFG	A0A0G2KA27	N-Term(iTRAQ4plex); S4(Phospho); K20(iTR
vFLAEcYNLsPTkDk	Q03351	N-Term(iTRAQ4plex); C6(Carbamidomethyl);
sGDEmIFDPTMSk	Q6P685	N-Term(Acetyl); S1(Phospho); M5(Oxidation)
rNLLEDDsDEEEDFFLR	A0A096MJ99	N-Term(iTRAQ4plex); S8(Phospho)
aEEDSksIQsDAPVYLR	A0A0G2K315	N-Term(iTRAQ4plex); K6(iTRAQ4plex); S7(P
lQPLPScEVcTPPsPEEVQ	O70521	N-Term(iTRAQ4plex); C7(Carbamidomethyl);
dIcNDVLsLLEk	P63102	N-Term(iTRAQ4plex); C3(Carbamidomethyl);
sASQSsLDkLDQELk	E9PSJ4	N-Term(iTRAQ4plex); S6(Phospho); K9(iTRA
ssPPATDPGPVPSSPR	Q499N6	N-Term(iTRAQ4plex); S2(Phospho)
fNTsDVSAIEk	P05708	N-Term(iTRAQ4plex); S4(Phospho); K11(iTR
fsEVLk	P07335	N-Term(iTRAQ4plex); S2(Phospho); K6(iTRA
gQTPLtEGSEDLDGHsDP	B2GV94	N-Term(iTRAQ4plex); T6(Phospho); S16(Pho
kLLLDPSNtPTk	Q6P791	N-Term(iTRAQ4plex); K1(iTRAQ4plex); T9(P
aGGsVESLR	F1LU97	N-Term(iTRAQ4plex); S4(Phospho)
sVPLAEEEDFDskEWVIID	D3ZAU7	N-Term(iTRAQ4plex); S12(Phospho); K13(iT
gTVTDFsGFDGR	P14668	N-Term(iTRAQ4plex); S7(Phospho)
eSEAGtGSSEHEDGERE	F1M3W5	N-Term(iTRAQ4plex); T6(Phospho); S19(Pho
tPEELDDsDFETEDFDVR	A0A0G2JYF7	N-Term(iTRAQ4plex); S8(Phospho)
tEATQGLDYVPSAGTIsPT	P34926	N-Term(iTRAQ4plex); S17(Phospho); K26(iT
vSEEDQsLENSEADVk	G3V7X4	N-Term(iTRAQ4plex); S7(Phospho); K16(iTR
yTAGSAsPTPTFk	F1LVK0	N-Term(iTRAQ4plex); S7(Phospho); K13(iTR
qSNAsSDVEAEEkETSVS	Q566D6	N-Term(iTRAQ4plex); S5(Phospho); K13(iTR
eVSMDDHkLsLDELHR	P06685	N-Term(iTRAQ4plex); K8(iTRAQ4plex); S10(
sPPEVSEVETDLGAVPDL	F1LMT8	N-Term(iTRAQ4plex); S1(Phospho)
nLLPTPEsPVTR	Q63569	N-Term(iTRAQ4plex); S8(Phospho)
sGYSSPGsPGTPGSR	D4A1Q2	N-Term(iTRAQ4plex); S8(Phospho)
gVDLLLEGVQGESsPTR	A0A0G2KB60	N-Term(iTRAQ4plex); S14(Phospho)
gLsLDSAVPEEHQILGGLT	Q8CGU9	N-Term(iTRAQ4plex); S3(Phospho); K22(iTR
eGsPIPHDPDLGSk	D3ZU55	N-Term(iTRAQ4plex); S3(Phospho); K14(iTR
sGYSsPGsPGTPGSR	D4A1Q2	N-Term(iTRAQ4plex); S5(Phospho); S8(Phos
mQVPTAEEMSSLTPEsS	B2DD29	N-Term(iTRAQ4plex); M1(Oxidation); S16(Ph
wLDEsDAEMELR	D4ABI7	N-Term(iTRAQ4plex); S5(Phospho)
sTEDLsPQR	M0R9T2	N-Term(iTRAQ4plex); S6(Phospho)
dATEDsITEDDk	P0CD96	N-Term(iTRAQ4plex); S6(Phospho); K12(iTR
sASSDTSEELNAQDsPkR	Q9JJ19	N-Term(iTRAQ4plex); S15(Phospho); K17(iT
dENERsSIsPMDEPVPDs	P15205;F1LRL9	N-Term(iTRAQ4plex); S6(Phospho); S9(Phos
lkEEIAEVANEIESLGStEE	B2GUV0	N-Term(iTRAQ4plex); K2(iTRAQ4plex); T18(
eLEAERsLMssPEDLTkD	P15205;F1LRL9	N-Term(iTRAQ4plex); S7(Phospho); S10(Pho
sYsPDGkEsPSDkk	A0A0G2JSR7	N-Term(iTRAQ4plex); S3(Phospho); K7(iTRA
aTTEEPETDEVDQVSES	G3V7I8	N-Term(iTRAQ4plex); S19(Phospho)
dVQAAPEPSHPPkEPAP	Q6DGF9	N-Term(iTRAQ4plex); K13(iTRAQ4plex); S19
skESVPEFPLsPPk	A0A096MK73	N-Term(iTRAQ4plex); K2(iTRAQ4plex); S11(
aPLAQPEsPTASAGEDV	D4ABZ4	N-Term(iTRAQ4plex); S8(Phospho)
tNsLEEk	Q63211	N-Term(iTRAQ4plex); S3(Phospho); K7(iTRA
dVATsPIsPTENNTTPPDA	P35465	N-Term(iTRAQ4plex); S5(Phospho); S8(Phos
ekPELVDDLsPR	Q9JKS6;D3Z9C7	N-Term(iTRAQ4plex); K2(iTRAQ4plex); S10(
sPAtPQAkPEGAAGAAAE	D4A0E2	N-Term(iTRAQ4plex); T4(Phospho); K8(iTRA
lPELDPRGsDEENLDSET	Q63358	N-Term(iTRAQ4plex); S9(Phospho)
dELHIVEAEAmNYEGsPIk	P13084;Q7TP95	N-Term(iTRAQ4plex); M11(Oxidation); S16(P
sSsPEPVTHLk	D4A6C5	N-Term(iTRAQ4plex); S3(Phospho); K11(iTR
ekLDLVsYFGk	P13233	N-Term(iTRAQ4plex); K2(iTRAQ4plex); S7(P
fADVLGLEEETLGSVPsP	Q00981	N-Term(iTRAQ4plex); S17(Phospho); C20(Ca
dNLTLWTSDsAGEEcDAA	P68255	N-Term(iTRAQ4plex); S10(Phospho); C15(Ca
mSISEGTVsDk	P15205;F1LRL9	N-Term(iTRAQ4plex); M1(Oxidation); S9(Pho

gSsQLDVNEEVEALIVk	Q810G8	N-Term(iTRAQ4plex); S3(Phospho); K17(iTR
eSPPQPPADDGSEEPGS	D4A1Q2	N-Term(iTRAQ4plex); T19(Phospho); S20(Ph
tGSVQTsPSSTPVTGR	A0A096P6M3	N-Term(iTRAQ4plex); S7(Phospho)
tVSPGSVsPIHGQGQAVE	M0R7A6	N-Term(iTRAQ4plex); S8(Phospho); K21(iTR
eGsSEkDEGEQEEEGETE	P12839	N-Term(iTRAQ4plex); S3(Phospho); K6(iTRA
hEDGTQsDSEDPLAk	D4A1G8	N-Term(iTRAQ4plex); S7(Phospho); K15(iTR
kVAAGHELQPLAIVDQRP	P08050	K1(iTRAQ4plex); N-Term(iTRAQ4plex); S19(
tWtLcGTPEYLAPEIILSk	P68182;A1L1M0	N-Term(iTRAQ4plex); T3(Phospho); C5(Carb
qPcPsESDMVIDEDk	Q566D6	N-Term(iTRAQ4plex); C3(Carbamidomethyl);
dVYLsPR	A0A0A0MXW9;A0A0G2K8	N-Term(iTRAQ4plex); S5(Phospho)
tEELAAEDTcAGPILQEkP	D3Z8X7	N-Term(iTRAQ4plex); C10(Carbamidomethyl
sQRtQDENPVVHFFk	P02688	N-Term(iTRAQ4plex); T4(Phospho); K15(iTR
vYYsPPVAR	A0A0G2KA88	N-Term(iTRAQ4plex); S4(Phospho)
qkGSYLTHEAsGLDEQGE	F1M7V6	N-Term(iTRAQ4plex); K2(iTRAQ4plex); S11(
aLETIPItPIER	F1LVK0	N-Term(iTRAQ4plex); T8(Phospho)
eTPSSSPVSPQEsPkHEN	F1M820	N-Term(iTRAQ4plex); S13(Phospho); K15(iT
nVLAAGNVSGEELDENY\	D3ZAL7	N-Term(iTRAQ4plex); S24(Phospho)
tEEVLSPDGsPSksPSkk	Q62847;A0A0G2JW28	N-Term(iTRAQ4plex); S10(Phospho); K13(iT
nFsVGR	G3V9G3;P11730	N-Term(iTRAQ4plex); S3(Phospho)
tLEVVsPsQSVTGSAGHTI	P15205;F1LRL9	N-Term(iTRAQ4plex); S6(Phospho); S8(Phos
kEEsEEsDDDMGFGLFD	P02401	K1(iTRAQ4plex); N-Term(iTRAQ4plex); S4(P
gSEEDPLLsPVETWk	Q6TUE6	N-Term(iTRAQ4plex); S9(Phospho); K15(iTR
aMGDEESGDSDGsPk	G3V7T8	N-Term(iTRAQ4plex); S13(Phospho); K15(iT
gLTAEDsGGDSk	D3ZUQ2	N-Term(iTRAQ4plex); S7(Phospho); K12(iTR
iAELEEERsQGsTSNSDW	F1LRL9	N-Term(iTRAQ4plex); S9(Phospho); S12(Pho
gIsPIVFDR	Q9QY02	N-Term(iTRAQ4plex); S3(Phospho)
vLSSTINNQVAEADEAAE	A0A0G2K1Z2	N-Term(iTRAQ4plex); S24(Phospho); K29(iT
dLPDGHAVsPR	A0A0G2K5C0	N-Term(iTRAQ4plex); S9(Phospho)
gLFGGGAQsLDREELFGE	Q9WU70	N-Term(iTRAQ4plex); S9(Phospho); K23(iTR
eSVAsGDDRAEEDMDEA	P15205;F1LRL9	N-Term(iTRAQ4plex); S5(Phospho); K20(iTR
eLPPsPEk	A0A0G2JW88	N-Term(iTRAQ4plex); S5(Phospho); K8(iTRA
iDPNPVDSESAPsE	A2VCX1	N-Term(iTRAQ4plex); S13(Phospho)
sNcYMVcAGESMsPGQGI	F1LRZ1	N-Term(iTRAQ4plex); C3(Carbamidomethyl);
rPPEMLLSsSWPSATLk	D3ZDS0	N-Term(iTRAQ4plex); S9(Phospho); K17(iTR
eITALAPsTMk	P68035;P60711;P63259	N-Term(iTRAQ4plex); S8(Phospho); K11(iTR
nTSYAsFER	O88764	N-Term(iTRAQ4plex); S6(Phospho)
aQEQQDsPTEPSGSPQE	A0A0G2JZ88	N-Term(iTRAQ4plex); S7(Phospho)
ttPSPktPPGSGEPPk	D4A1Q2	N-Term(iTRAQ4plex); T2(Phospho); K6(iTRA
sAEkADsVEQQDGAALEk	P34926	N-Term(iTRAQ4plex); K4(iTRAQ4plex); S7(P
sLTRsPPAIR	A0A0G2K2M9	N-Term(iTRAQ4plex); S1(Phospho); S5(Phos
kIGELEVGQmENIsPGQII	A0A0G2K9S4	K1(iTRAQ4plex); N-Term(iTRAQ4plex); M10(
sRtPLLPR	A0A0G2K2M9	N-Term(iTRAQ4plex); S1(Phospho); T3(Phos
gPAGAAAFLHEAPsPPPS	D3ZT47	N-Term(iTRAQ4plex); S14(Phospho); K26(iT
sQFLEEGMGGTVPHLsPE	D4A6H3	N-Term(iTRAQ4plex); S16(Phospho)
rRtPsPPPR	B2RYB3;A0A0G2K4F6	N-Term(iTRAQ4plex); T3(Phospho); S5(Phos
vVETQWDVSSAASPESP	D4A5J1	N-Term(iTRAQ4plex); C20(Carbamidomethyl
aALPAGEGEsPEGAkIDVI	A0A0G2K5Z4	N-Term(iTRAQ4plex); S10(Phospho); K15(iT
lSPEEPAPEAPEtPEAPGC	Q9EQZ1	N-Term(iTRAQ4plex); T13(Phospho)
sLmssPEDLTk	P15205;F1LRL9	N-Term(iTRAQ4plex); M3(Oxidation); S4(Pho
scsPELQQk	A0A0G2JSV2	N-Term(iTRAQ4plex); C2(Carbamidomethyl);
sAPSsPTLDcEk	Q78PB6	N-Term(iTRAQ4plex); S5(Phospho); C10(Car
iDVESTELASSEsPQAAEI	A0A0G2K5Z4	N-Term(iTRAQ4plex); S13(Phospho); K27(iT
yEIsPEAYER	Q1RP74	N-Term(iTRAQ4plex); S4(Phospho)
gsPPPTYSPSMR	D4A4P8	N-Term(iTRAQ4plex); S2(Phospho)
qSGtPPGVGAPGIPGASV	D3ZJ01	N-Term(iTRAQ4plex); T4(Phospho)

Information Classification: General

aEALTSLHGDDQDsEDE\	Q63002	N-Term(iTRAQ4plex); S14(Phospho); K25(iT
lAVEALSsLDGDLSGR	P07335	N-Term(iTRAQ4plex); S8(Phospho)
eFGDGsDENEVEEQEAk	Q8R497	N-Term(iTRAQ4plex); S6(Phospho); K17(iTR
vSkDEVQNEGTAAEADsF	F1LM55	N-Term(iTRAQ4plex); K3(iTRAQ4plex); S17(
fVQDHFDHNIsPTIGASFM	P97632	N-Term(iTRAQ4plex); S11(Phospho); K21(iT
mAsPPPSGPPsAAHTPFH	P34926;Q63330	N-Term(iTRAQ4plex); S3(Phospho); S11(Pho
tDHGAEIVYksPVVSGDTS	D4A1Q2	N-Term(iTRAQ4plex); K10(iTRAQ4plex); S11
sPVTVksPAEAk	F1LRZ7	N-Term(iTRAQ4plex); S1(Phospho); K6(iTRA
sDGEsDSPEkEAEFAPYP	O35430	N-Term(iTRAQ4plex); S5(Phospho); K10(iTR
nLQLLTQASTDcsP	M0R3J7	N-Term(iTRAQ4plex); C12(Carbamidomethyl
gAycLSVSDFDNAk	Q9WUD9	N-Term(iTRAQ4plex); Y3(Phospho); C4(Carb
iGAGPQTVETGsETEDEA	M0R9R0	N-Term(iTRAQ4plex); S12(Phospho); K28(iT
gDSEIPSSGDEGEIVsAEC	O70474	N-Term(iTRAQ4plex); S16(Phospho); K26(iT
hMQMsSQEALNk	Q923K6	N-Term(iTRAQ4plex); S5(Phospho); K12(iTR
vVSsIEQk	P63102	N-Term(iTRAQ4plex); S4(Phospho); K8(iTRA
eVHDELEDLPsPPPPLSP	M0R423	N-Term(iTRAQ4plex); S11(Phospho); K26(iT
tDVSNFDEEFTGEAPTLsF	Q63433	N-Term(iTRAQ4plex); S18(Phospho)
vEQALSEVASSLQsSAPk	F1LUY5	N-Term(iTRAQ4plex); S14(Phospho); K18(iT
eLsHSPPR	A0A0G2K2M9	N-Term(iTRAQ4plex); S3(Phospho)
gQTPLTEGSEDLDGHsDF	B2GV94	N-Term(iTRAQ4plex); S16(Phospho)
qSLNsPsPGETEMDLLVT	F1LXC7	N-Term(iTRAQ4plex); S5(Phospho); S7(Phos
sPSPSPTsPGSLR	Q9WVP7;O08875;A0A0G	N-Term(iTRAQ4plex); S8(Phospho)
kEEENVDsDDEGELQDLL	Q3MHS2	N-Term(iTRAQ4plex); K1(iTRAQ4plex); S8(P
aEEAQRtDNEGE	M0R5I3	N-Term(iTRAQ4plex); T7(Phospho)
iAcDEEFsDsEDEGEGGR	Q99PA1	N-Term(iTRAQ4plex); C3(Carbamidomethyl);
vDNSSLTGESEPQTRsPE	F1LRK1	N-Term(iTRAQ4plex); S16(Phospho); C19(Ca
vTQILQsPAFREDLEcLIQ	A0A0G2JW28	N-Term(iTRAQ4plex); S7(Phospho); C16(Car
sPAsVksPGEAk	F1LRZ7	N-Term(iTRAQ4plex); S4(Phospho); K6(iTRA
sGSVTNMQAEEcSTtPQR	A0A0G2K2M9	N-Term(iTRAQ4plex); C12(Carbamidomethyl
eETSScsPPGVLLEk	F1LR18	N-Term(iTRAQ4plex); C6(Carbamidomethyl);
iLAtPPQEDAPSVDIANIR	P50137	N-Term(iTRAQ4plex); T4(Phospho)
gPPQsPVFEGVYNNSR	B3DMA1	N-Term(iTRAQ4plex); S5(Phospho)
vGEQIVIGtPGTVLDWcSk	Q68FX3	N-Term(iTRAQ4plex); T9(Phospho); C17(Car
vGsLDNVGHLPAGGTVk	A0A0G2JW88	N-Term(iTRAQ4plex); S3(Phospho); K17(iTR
dIsPSkDNMsPPkR	D3ZBU7	N-Term(iTRAQ4plex); S3(Phospho); K6(iTRA
sLSPGR	Q810W7;D3ZAR2;D3ZL3(	N-Term(iTRAQ4plex); S1(Phospho)
nASTPQQLPLEPVSPtGQ	D4A644	N-Term(iTRAQ4plex); T16(Phospho); C30(Ca
qksPEIHR	B1WC16	N-Term(iTRAQ4plex); K2(iTRAQ4plex); S3(P
aLYLEPSDGVsPTQETGE	M0RAP5	N-Term(iTRAQ4plex); S11(Phospho)
aAGDTVVIENNDIsPEMG	D4A4Z9	N-Term(iTRAQ4plex); S14(Phospho); K22(iT
wGsNELPAEEGk	P11507	N-Term(iTRAQ4plex); S3(Phospho); K12(iTR
rLPAsDGLDLSQAAAR	P23565;G3V8Q2	N-Term(iTRAQ4plex); S5(Phospho)
eQQPsPGPAGSR	D4AAX6	N-Term(iTRAQ4plex); S5(Phospho)
rPPsPDVIVLsDSEQPsSP	G3V8R7	N-Term(iTRAQ4plex); S4(Phospho); S11(Pho
nVPGLATPSsPTQk	A0A0G2JV49	N-Term(iTRAQ4plex); S10(Phospho); K14(iT
kMPsIESDV	G3V9C5	N-Term(iTRAQ4plex); K1(iTRAQ4plex); S4(P
eGSVsPkDEEAR	Q52KK2	N-Term(iTRAQ4plex); S5(Phospho); K7(iTRA
sGskENVQETQI	P0CC10	N-Term(iTRAQ4plex); S3(Phospho); K4(iTRA
gDFsPFGNTQGPSR	F1LMT8	N-Term(iTRAQ4plex); S4(Phospho)
rFVQWQsSI	P0C5X8	N-Term(iTRAQ4plex); S7(Phospho)
sLAEsIDDALNcR	A0A0G2JUG7	N-Term(iTRAQ4plex); S5(Phospho); C12(Car
sASQLPsPPLLPTPPPk	A0A0G2K0S0	N-Term(iTRAQ4plex); S7(Phospho); K17(iTR
aAQPSVTAsPQTEEFFDL	G3V9X2	N-Term(iTRAQ4plex); S9(Phospho)
gSVGPAPsLSELDLRPPsI	D3ZWQ0	N-Term(iTRAQ4plex); S8(Phospho); S18(Pho
gTDDsPkNSQEDLQDR	M0RBL8;M0RDJ7	N-Term(iTRAQ4plex); S5(Phospho); K7(iTRA

dVSDERLsPAksPsLsPSP	P15205;F1LRL9	N-Term(iTRAQ4plex); S8(Phospho); K11(iTR
asPGPGGLSGGESLLVk	F1M6V8	N-Term(iTRAQ4plex); S2(Phospho); K17(iTR
vGIFSSQSVSsPLLETVQQ	A0A0G2K2M9	N-Term(iTRAQ4plex); S11(Phospho)
gELEHsQQEEDGEEAMA	F8QYX1	N-Term(iTRAQ4plex); S6(Phospho); K28(iTR
tDGEAsPLkEAETkEEEEE	Q923K6	N-Term(iTRAQ4plex); S6(Phospho); K9(iTRA
lLRDPDGsAEDDSLEQTS	A0A0G2K6R9;F1M9N9	N-Term(iTRAQ4plex); S8(Phospho); K25(iTR
sVSTPSEAGSQDsGDGA	D3ZU74	N-Term(iTRAQ4plex); S13(Phospho)
lGGsAVISLEGkPL	P45592	N-Term(iTRAQ4plex); S4(Phospho); K12(iTR
eEAksPEkAk	F1LRZ7	N-Term(iTRAQ4plex); K4(iTRAQ4plex); S5(P
sAsTDLGTADVVLGR	G3V9G7	N-Term(iTRAQ4plex); S3(Phospho)
lVLVsPTSEQYDSLLR	D2XV59	N-Term(iTRAQ4plex); S5(Phospho)
dGAsPSESGPGSR	Q9JKL8	N-Term(iTRAQ4plex); S4(Phospho)
sEsLDEEEkLELQR	D3ZQR3	N-Term(iTRAQ4plex); S3(Phospho); K9(iTRA

ions,describe the modified amino acids, positions, and modifications; phosphoRS Site Probabilities,g

Information Classification: General

fied from SFO in rats with chronic renal failure. （the significance of NC v

phosphoRS Site Probabilities	phosphoRS Binomial Peptide Score	IonScore	Charge
S(3): 100.0	135.6324499	18.3	3
S(5): 5.8; T(6): 5.8; T(7): 0.4; S(10): 8	130.0815846	6.35	3
Y(5): 0.0; Y(9): 0.0; T(10): 0.0; S(15):	143.8453596	13.6	4
S(2): 0.0; S(5): 100.0	160.6711428	17.6	3
S(6): 100.0	371.5569038	30.97	3
T(2): 0.0; T(3): 0.0; T(7): 0.0; S(10): 6	148.5535569	18.85	3
S(4): 99.9; S(6): 0.1; T(10): 0.0; S(18	460.2593209	42.11	3
S(1): 0.0; S(7): 100.0; S(13): 100.0	464.674667	49.18	2
S(2): 0.0; S(6): 100.0; S(9): 100.0	290.5967555	29.52	2
S(1): 0.0; S(6): 33.0; S(7): 33.0; T(10	239.0927402	61.04	3
T(1): 3.8; S(4): 92.5; S(6): 3.8; T(22):	341.2071435	51.64	4
S(3): 95.0; T(4): 5.0	165.7502548	36.37	3
T(9): 3.9; T(10): 3.9; S(15): 92.2	130.6225193	19.84	4
S(2): 100.0; S(4): 0.0; T(8): 0.0; S(16	432.4925707	99.17	2
S(1): 0.0; S(7): 100.0; S(13): 100.0	416.6329255	38.42	3
S(3): 2.4; S(8): 97.5; S(10): 0.1	158.5654179	42.31	3
S(6): 0.0; S(7): 100.0	145.796863	17.23	2
S(2): 100.0; Y(14): 0.0	443.2774495	67.04	2
S(1): 0.0; S(5): 100.0	215.9313798	36.33	2
S(6): 100.0	346.0047306	45.82	2
S(1): 0.0; S(7): 100.0; S(13): 100.0; T	309.0190757	14.28	3
S(3): 100.0; T(7): 0.0; S(8): 0.0; S(9):	256.8186397	40.18	3
T(1): 0.1; S(3): 99.9; T(5): 0.0	113.1607711	16.19	2
S(7): 99.6; T(11): 0.4; S(13): 0.0; S(1	154.5169701	28.11	4
T(15): 0.1; S(17): 48.7; S(20): 48.7; S	114.5836057	15.19	2
T(6): 4.7; S(8): 95.3; S(22): 0.0	114.7826991	17.22	3
S(3): 48.9; S(5): 0.1; S(12): 48.9; S(1	201.963872	71.83	3
S(3): 0.0; S(10): 100.0; S(13): 100.0;	214.2479444	24.71	3
S(2): 0.0; S(5): 100.0	176.2255845	31.78	2
S(3): 100.0; S(4): 100.0; S(7): 100.0;	123.6248453	11.72	3
T(1): 0.0; S(5): 99.8; S(7): 0.2; T(10):	180.5763232	63.61	3
S(5): 100.0; S(17): 0.0	136.6925265	43.61	3
S(1): 100.0; S(5): 100.0; T(11): 0.0	146.3116018	24.03	2
S(6): 100.0	151.7637865	18.62	3
S(4): 0.0; S(8): 100.0; S(11): 0.0; T(1	403.1451301	58.73	3
S(6): 100.0	270.4011674	25.29	2
S(1): 0.0; S(3): 33.3; S(4): 33.3; S(5):	114.7682359	6.71	3
S(2): 100.0; S(3): 100.0	123.4995182	17.74	3
S(7): 50.0; S(13): 50.0	103.0925707	23.54	2
S(7): 0.0; T(11): 88.3; S(13): 6.0; S(1	217.2831178	57.68	3
S(5): 0.0; S(18): 100.0; S(23): 0.0	391.3083093	51.45	3
S(1): 100.0; S(3): 100.0	80.61212013	8.89	3
S(2): 0.0; S(8): 6.4; T(12): 96.8; S(13	147.7391292	22.86	3
S(3): 99.8; S(6): 0.2; S(10): 0.0; T(13	151.377646	21.78	3
S(2): 0.0; S(5): 100.0	147.5421808	12.12	2
T(5): 0.0; S(6): 0.3; S(7): 0.3; S(8): 0.	51.41225582	2	3
S(3): 100.0	144.5340186	15.14	2
S(3): 100.0	191.4783993	23.9	2
S(1): 0.0; T(4): 4.0; S(7): 91.9; S(10):	142.8072473	13.32	3
S(9): 100.0; S(11): 0.0; S(12): 0.0	100.9817793	12.72	2
S(4): 100.0	73.11170448	0.02	5

Information Classification: General

S(3): 98.7; S(4): 1.3		129.3223156	20.2	3
S(5): 100.0; S(7): 0.0		270.5290383	48.29	2
S(1): 0.0; S(9): 100.0		266.5581962	45.62	2
T(1): 0.0; S(10): 100.0		93.99316438	17.31	2
S(10): 96.0; S(12): 4.0		178.0772963	12.73	4
S(5): 0.0; S(9): 0.1; S(12): 99.9; S(13		265.3489567	43.56	3
S(13): 100.0		198.3685458	15.94	3
S(3): 100.0; S(17): 0.0		156.1554026	14.04	3
S(8): 0.0; T(12): 0.0; T(13): 0.0; S(15		253.4681651	50.39	4
S(3): 0.0; S(12): 7.0; S(14): 93.0; S(1		210.6461147	62.49	3
S(4): 100.0; S(8): 100.0; S(11): 0.0; T		273.1382148	46.48	3
S(7): 100.0		193.4115296	33.54	2
S(1): 3.2; S(3): 96.8; S(7): 0.0; T(8): (		163.4438813	28.74	3
S(1): 0.2; S(2): 99.8; S(7): 100.0; S(1		142.3561404	16.79	3
S(4): 0.1; S(6): 99.9; S(9): 0.0; S(11):		211.2445862	29.13	2
S(8): 100.0; S(10): 100.0; S(12): 100.		70.82657228	11.79	3
S(6): 100.0; T(12): 0.0; Y(16): 0.0; S(		334.3703724	49.47	3
S(5): 100.0; S(9): 100.0		399.9185488	87.81	2
S(1): 0.0; S(5): 1.9; S(6): 98.1		148.2194336	14.11	2
S(6): 100.0		168.2434248	12.86	2
S(1): 0.0; S(7): 100.0; S(13): 1.5; S(1		490.0564468	50.51	2
S(5): 0.9; T(15): 49.3; Y(16): 49.3; S(		81.32592957	7.06	4
T(6): 0.0; T(11): 0.7; T(15): 99.3; S(1		114.0141398	6.45	3
T(1): 0.0; Y(7): 0.0; Y(9): 0.0; S(14): 2		138.7184767	38.73	4
S(5): 50.0; S(6): 50.0		67.68505447	3.26	4
T(1): 0.5; S(2): 0.0; S(3): 0.0; S(5): 0.		63.96823577	25.72	2
S(2): 0.0; S(5): 100.0		137.1940676	21.61	2
T(1): 50.0; S(2): 50.0; S(5): 0.0; S(11		133.7013767	11.36	4
S(3): 100.0; Y(9): 0.0; T(12): 0.0		261.0635498	31.74	3
S(8): 32.9; S(9): 32.9; T(11): 32.9; T(		135.0207669	11.13	3
S(5): 100.0		322.5516771	54.05	2
S(4): 100.0; S(8): 100.0; S(11): 0.0; T		335.8782818	33.56	3
S(1): 0.0; T(2): 0.0; S(5): 100.0; S(7):		120.9556676	13.79	3
T(4): 0.0; S(8): 16.7; T(9): 16.7; T(11)		113.479448	14.71	3
S(10): 0.0; T(11): 0.0; S(15): 100.0		198.8051835	15.31	3
S(8): 0.0; T(11): 0.0; S(16): 0.7; T(17)		169.5050557	28.24	4
T(1): 0.0; S(3): 100.0; Y(5): 0.0; T(8):		244.2382085	48.24	2
S(3): 100.0; S(6): 100.0		172.5852725	11.03	2
S(1): 0.0; S(3): 0.0; S(4): 0.0; S(12): 3		124.6474098	16.35	3
S(1): 0.1; S(2): 3.6; S(5): 92.7; T(7): 3		238.4553847	85.12	3
S(1): 0.0; S(7): 100.0		298.2132688	32.66	2
T(1): 99.5; S(8): 0.5; T(24): 0.0		104.1936528	5.94	3
S(2): 0.0; S(26): 100.0; S(29): 100.0		78.39333757	2.08	3
S(10): 100.0; S(20): 0.0; T(21): 0.0; S		226.7848865	32.73	5
T(2): 0.0; S(6): 0.0; T(12): 0.0; S(16):		245.7370236	106.3	3
S(3): 100.0; Y(8): 0.0		220.3064753	43.5	2
S(8): 100.0		205.6754502	26.38	2
S(4): 100.0		126.3715261	27.46	2
T(12): 3.7; S(14): 3.7; S(15): 3.7; S(1		134.181863	21.54	3
T(3): 0.0; S(5): 100.0		183.5342236	24.88	2
T(1): 0.0; S(10): 0.0; S(20): 100.0; T(		158.3534409	4.12	3
S(1): 100.0; S(14): 0.0		280.3068079	33.02	2
T(1): 0.0; S(3): 100.0; S(6): 0.0; T(13)		290.3401576	62.43	2
S(4): 100.0		286.9178544	49.88	2
S(3): 0.0; S(6): 0.0; T(9): 0.0; T(11): 1		266.524674	64.53	3

Information Classification: General

S(3): 100.0; S(5): 100.0	51.50154078	6.76	3
S(4): 0.2; S(5): 99.8; S(10): 0.0; T(11	184.6564666	21.27	3
S(7): 50.0; S(14): 50.0	87.72429574	19.59	3
S(5): 100.0; S(9): 100.0	124.6972433	3.01	3
S(1): 0.0; S(3): 100.0	154.5564317	15.45	3
S(10): 5.1; S(16): 94.9	79.68516826	7.55	4
S(6): 98.7; T(8): 1.3	166.089063	20	2
S(3): 100.0; S(6): 0.0; T(9): 0.0	412.447359	110.2	2
S(3): 0.0; S(7): 99.9; S(10): 0.1	158.0390461	26.98	2
Y(3): 100.0	245.3957488	51.12	2
S(1): 0.0; S(3): 100.0	208.7741579	23.55	2
S(7): 96.1; S(9): 7.5; S(10): 92.5; T(1	168.888693	28.27	3
S(10): 100.0	652.9905043	76.59	2
S(3): 97.6; S(5): 2.4	135.7230546	29.2	3
S(1): 0.0; S(4): 100.0	200.2252928	25.13	2
T(3): 33.3; T(4): 33.3; S(6): 33.3	208.8137327	56.77	2
S(2): 0.0; S(5): 100.0; S(8): 100.0; Y(	357.1680165	66.1	3
S(11): 0.1; S(18): 97.6; S(20): 2.2; T(	295.960553	29.48	3
S(1): 0.0; S(4): 96.2; S(5): 3.8; T(10):	182.2707338	15.27	3
S(1): 0.0; S(4): 0.0; S(7): 100.0; S(13	382.7530786	44.3	2
S(6): 0.0; S(12): 100.0; S(18): 0.0; S(	243.5730248	49.04	3
S(6): 50.0; S(8): 50.0; S(18): 0.0	216.4146131	11.91	3
S(3): 100.0; T(18): 0.0; T(24): 0.0; Y(	196.4534969	22.57	4
S(3): 100.0; S(4): 100.0; S(8): 0.0; S(	211.0263167	23.71	3
S(2): 0.0; T(8): 0.0; S(12): 0.0; T(16):	454.9926151	110.68	3
S(7): 100.0; S(10): 0.0	284.2115138	63.1	2
S(1): 0.0; S(2): 100.0	148.4667849	48.54	2
S(3): 100.0; S(9): 0.0	341.1727037	46.48	2
S(2): 0.0; T(5): 0.0; S(15): 100.0; Y(2	315.6746977	45.57	3
S(2): 0.0; S(3): 0.0; S(5): 99.8; T(10):	115.0861112	33.19	3
S(3): 100.0; S(10): 100.0	295.3984587	52.1	2
S(4): 100.0; T(9): 0.0; T(11): 0.0; S(1	261.9759442	49.27	3
Y(1): 0.2; S(3): 99.5; T(5): 0.2; S(14):	143.1572303	4.64	3
S(13): 99.9; S(18): 0.1	348.4962107	30.29	3
S(2): 100.0	143.9765004	16.5	2
T(4): 0.0; S(7): 100.0; S(11): 0.0; S(1	211.4750497	20.04	3
S(1): 0.0; T(4): 0.0; S(7): 100.0	150.1301421	9.7	2
S(9): 47.5; Y(14): 47.5; S(15): 5.4; S(	85.11515827	3.62	3
S(1): 0.1; S(2): 0.1; S(3): 0.1; S(15): 9	49.15347464	1.62	5
S(1): 0.0; S(10): 50.0; S(12): 50.0	143.467461	6.65	4
S(6): 100.0	448.2571016	59.59	2
T(2): 0.0; T(8): 0.0; S(11): 0.0; S(17):	477.4978809	59.91	4
T(2): 0.0; T(19): 0.4; T(23): 90.2; S(2	73.85558234	1.53	6
T(1): 2.4; S(2): 2.4; S(3): 95.2; S(7): 0	65.81579559	17.84	2
S(1): 100.0; S(4): 0.0; S(6): 0.0; S(7):	360.3718841	133.95	2
T(8): 50.0; S(10): 50.0	214.5898755	30.01	2
T(1): 0.0; T(2): 0.0; T(4): 100.0; Y(10)	377.3387222	58.77	2
S(7): 100.0; T(11): 0.0; S(13): 0.0; S(	209.1021433	28	3
S(4): 33.3; S(5): 33.3; S(6): 33.3; T(1	173.5221217	12.92	3
S(2): 0.0; T(4): 0.0; S(6): 0.0; S(8): 0.	172.5889916	33.55	3
T(20): 0.0; S(25): 0.0; S(33): 94.9; S(	292.2670727	40.21	5
T(2): 0.0; S(5): 0.0; S(8): 0.0; T(9): 0.	354.3358275	62.52	3
S(2): 0.0; S(7): 0.4; S(8): 5.9; S(9): 0.	132.9060758	13.7	4
S(1): 0.0; S(3): 100.0; S(5): 0.0; S(8):	303.7863135	38.67	2
S(2): 0.0; S(10): 0.0; S(11): 0.1; S(13	153.9387568	41.66	3

Information Classification: General

S(3): 100.0; S(7): 0.0; T(17): 0.0	260.6856581	49.8	3
S(1): 0.0; S(4): 0.0; S(7): 100.0	280.5858314	44.92	2
S(3): 100.0; S(6): 0.0; S(9): 0.0	243.3036878	26.13	3
S(1): 0.0; S(10): 99.8; T(13): 0.2	105.9028548	12.12	2
S(1): 100.0	160.0177008	17.62	2
S(4): 100.0; S(6): 100.0; T(10): 0.0; S	302.0755244	23.22	3
S(1): 0.0; S(3): 100.0	292.9882752	36.98	2
S(5): 0.0; S(8): 100.0; T(13): 0.0; T(1	527.5767052	113.36	3
S(9): 99.8; S(11): 0.2; T(19): 0.0; S(2	207.43089	22.74	3
S(5): 100.0; S(12): 0.0	322.716395	35.23	3
T(6): 100.0; T(12): 0.0	210.8413192	43.52	2
S(3): 0.2; S(8): 99.8; T(10): 0.0; S(18	92.98834333	20.21	3
T(2): 0.0; S(10): 100.0	236.2550538	19.13	3
S(2): 0.0; S(5): 0.1; S(6): 99.9; S(11):	219.6573032	53.68	2
S(3): 100.0; T(5): 0.0; T(7): 0.0	301.4418807	57.38	2
S(12): 99.2; T(15): 90.6; Y(25): 5.1; Y	105.3680221	33.45	3
T(1): 3.0; S(3): 93.9; S(4): 3.0; T(5): (	82.02737636	10.58	2
S(14): 99.9; S(16): 0.1; Y(19): 0.0	279.8062684	56.3	4
T(1): 0.0; S(3): 2.4; S(5): 48.9; S(7): 4	116.142261	14.42	3
T(1): 0.0; S(3): 1.4; S(4): 1.4; S(5): 48	95.4590866	20.47	2
Y(2): 0.0; S(8): 2.8; S(9): 2.8; S(10): 4	46.65329186	14.11	3
S(6): 100.0; T(8): 0.0	160.252041	16.53	2
S(9): 100.0; S(13): 100.0	196.462102	4.16	3
T(6): 0.0; S(7): 0.0; S(8): 0.0; T(9): 0.	244.5982722	43.26	2
S(3): 100.0	160.8515502	20.89	3
S(1): 0.0; T(2): 0.0; S(5): 50.7; S(7): 5	121.5965529	24.44	2
S(1): 0.0; S(6): 100.0	268.4734136	31.33	3
S(1): 0.1; T(2): 3.3; S(5): 96.6; S(7): (	132.5112438	19.9	2
S(1): 0.0; T(3): 0.0; S(7): 100.0; T(22	161.6945236	11.02	4
Y(3): 0.0; Y(7): 0.0; T(12): 50.0; S(15	188.8800553	28.57	3
S(2): 0.0; S(6): 50.0; S(8): 50.0	79.48342228	18.17	2
S(6): 0.0; S(11): 100.0; S(15): 100.0	448.5036877	35.69	3
S(2): 100.0; S(4): 0.0; S(8): 0.0; S(12	207.8160439	14.05	2
S(3): 49.3; S(5): 49.3; T(6): 1.3; S(7):	157.0126394	28.2	2
T(6): 0.0; S(10): 100.0; S(14): 0.0; S(	218.2459814	22.42	3
T(7): 100.0	52.8133971	9.18	2
S(9): 100.0; S(13): 100.0; S(14): 100.	165.86213	34.13	3
S(3): 100.0; S(7): 0.0; S(9): 0.0; T(10	133.0210934	25.19	3
S(3): 100.0; T(5): 0.0	144.7413158	14.91	2
S(4): 25.5; S(5): 25.5; T(7): 25.5; S(1	110.8148541	15.89	3
S(4): 100.0; T(5): 100.0; S(9): 0.0; T(	272.3267633	73.99	2
S(3): 100.0; S(6): 0.0	158.9952773	19.85	2
S(7): 44.3; S(8): 44.3; T(10): 17.2; S(	132.6306045	43.27	3
T(4): 100.0; S(8): 0.0; S(9): 0.0; S(13	197.9820131	25.65	3
S(5): 0.0; S(8): 100.0; T(13): 100.0; T	440.1029819	91.93	2
S(3): 100.0	106.6859896	17.71	2
S(5): 100.0; S(9): 0.0; S(11): 0.0; S(1	203.1938948	22.26	3
S(5): 91.1; S(13): 3.0; S(14): 3.0; S(1	129.8527583	25.31	2
S(3): 4.0; S(6): 96.0; S(17): 0.0; T(21	123.4927669	7.78	3
S(4): 100.0	129.9453662	15.97	2
S(3): 100.0	304.2152011	101.62	2
T(2): 0.0; S(5): 0.1; Y(6): 99.8; S(9): (	102.9523616	7.95	2
S(2): 50.0; S(3): 50.0; S(7): 0.0	243.4397359	42.78	3
S(2): 2.6; S(6): 94.8; T(8): 2.6	70.47036598	11.53	2
S(13): 3.0; S(14): 97.0; T(17): 0.0; Y(	407.5036953	106.88	3

Information Classification: General

S(1): 0.0; S(4): 100.0	193.4710178	5.79	4
S(3): 96.0; T(5): 4.0; T(9): 0.0; T(10):	168.5669176	25.34	4
S(7): 0.0; S(10): 99.5; S(12): 0.2; S(1	126.0220486	17.25	3
S(3): 100.0; S(5): 95.3; S(7): 2.4; T(9	181.0377531	20.66	2
S(2): 0.0; S(7): 100.0	326.0044352	77.07	2
S(5): 100.0; S(9): 0.0	313.5982093	38.48	2
S(6): 3.3; S(7): 96.7	191.2903554	35.16	3
T(3): 0.0; T(4): 0.0; T(6): 33.3; T(7): 3	182.077748	26.77	2
S(4): 0.1; S(5): 0.1; S(8): 49.1; S(9): 4	100.6672267	11.98	2
S(1): 0.1; S(3): 99.8; S(5): 0.0; S(8): (	213.881318	31.39	3
S(1): 0.0; S(4): 2.2; S(5): 97.8	138.7248149	13.8	2
S(1): 0.0; T(3): 100.0	128.2059429	25.54	2
Y(5): 100.0; S(10): 0.0	316.6921913	59.18	2
S(11): 97.4; T(13): 2.6	87.10355424	9.05	2
S(3): 100.0; T(7): 100.0	97.07077902	8.11	3
T(5): 100.0	215.6250167	26.7	3
S(3): 93.8; S(4): 6.2; S(10): 0.0; S(18	100.0013701	4.69	4
S(3): 100.0	250.9344677	32.14	2
T(1): 0.0; S(9): 100.0; Y(16): 0.0	503.8957928	117.79	2
S(1): 100.0; S(3): 100.0; S(8): 0.0	209.7868913	27.88	3
S(10): 0.0; S(11): 0.0; S(14): 50.0; T(	325.8797755	72.46	3
S(5): 99.8; S(7): 0.2	77.4652926	4.59	3
S(5): 50.0; S(6): 50.0; T(12): 0.0; T(2(	304.7077407	73.74	3
T(4): 0.0; S(12): 0.0; S(15): 100.0; S(	372.5685521	66.89	3
S(1): 100.0	149.0706853	10.4	2
S(3): 100.0; T(6): 0.0; T(8): 0.0; T(12)	284.7979801	49.97	2
S(4): 100.0	132.6591412	16.04	2
S(6): 100.0	337.2077436	73.11	2
S(10): 100.0; S(12): 0.0	710.2409145	84.13	2
S(8): 100.0; T(21): 0.0	280.7516579	36.25	3
S(3): 100.0; T(6): 0.0; S(8): 0.0	274.722117	73.18	2
S(2): 99.9; S(4): 0.1; S(5): 0.1	82.80114288	11.84	2
S(11): 1.9; S(12): 98.1	219.7906508	34.78	3
S(2): 100.0; S(7): 0.0; S(8): 0.0	163.6245291	18.37	2
S(2): 100.0	258.992291	53.53	2
S(6): 0.0; S(11): 97.7; S(15): 2.3	276.5110982	32.88	3
S(3): 100.0; S(16): 0.0	67.6804936	7.5	2
S(4): 4.6; S(6): 90.3; S(9): 0.3; S(10):	72.79330638	9.03	3
S(3): 0.1; S(5): 1.9; S(11): 49.9; S(12	135.8206124	43.43	2
T(5): 0.2; S(7): 99.8	97.15265616	9.77	3
S(14): 100.0	197.6300104	14.32	3
S(4): 1.6; S(6): 98.4	140.805855	46.69	2
T(3): 100.0	396.1869674	31.84	2
S(3): 49.4; S(5): 49.4; S(6): 1.1; T(10	103.4509422	12.36	2
S(2): 0.0; S(7): 6.3; T(12): 0.5; S(13):	136.7465275	5.91	3
S(5): 100.0; S(7): 50.0; S(9): 50.0	254.3117036	49.29	2
T(3): 50.0; S(4): 50.0	212.4646847	43.02	2
T(3): 0.0; T(6): 0.0; T(8): 50.0; S(9): 5	121.6701472	14.82	2
Y(7): 33.3; S(10): 33.3; T(15): 33.3; T	82.81642943	3.07	3
T(8): 5.5; S(10): 94.1; S(12): 0.4	72.81421421	11.72	3
S(8): 100.0; S(9): 100.0	344.2714916	46.02	2
T(1): 0.0; T(3): 0.0; S(10): 49.9; T(11)	290.5017182	35.69	3
T(14): 0.0; T(15): 0.0; T(20): 0.5; T(2:	116.1368759	8.08	4
S(13): 100.0; S(14): 100.0; T(17): 0.0	360.8024737	110.36	3
S(3): 0.0; S(5): 0.0; Y(7): 0.0; S(10): 1	277.1785491	36.36	2

Information Classification: General

S(1): 100.0		110.2621898	11.07	3
T(8): 0.2; S(9): 4.4; S(11): 95.4; S(15		217.8642524	38.19	3
S(3): 100.0		258.7628534	18.91	3
S(1): 0.0; T(4): 0.0; S(8): 50.0; S(9): 5		152.6437439	31.56	3
T(2): 0.0; T(12): 0.0; T(18): 100.0		315.9324667	42.8	4
T(4): 0.0; Y(5): 0.0; S(10): 100.0		246.1106434	54.2	2
S(1): 100.0; T(9): 0.0		240.7759046	32.74	2
S(2): 0.0; S(10): 0.0; S(12): 100.0		196.9542881	14.91	2
S(1): 100.0; S(4): 0.0		150.7036786	17.07	2
Y(1): 0.0; T(2): 0.0; S(10): 100.0		242.4246309	42.63	3
S(4): 0.0; S(5): 0.0; S(12): 0.0; S(13):		287.3158243	68.42	3
S(4): 0.4; S(6): 99.6; S(10): 100.0		128.437064	16.61	4
S(1): 0.0; S(2): 0.0; T(5): 0.0; S(10): 1		181.3670552	19.39	3
S(4): 97.7; T(5): 2.3; S(13): 0.0		216.8481421	32.04	3
S(15): 2.3; S(22): 97.7		261.216661	23.13	3
S(2): 6.8; S(3): 6.8; T(6): 87.3; S(7): 8		135.422764	10.46	4
S(2): 0.0; S(4): 100.0; S(8): 0.0		273.4491058	36.26	2
T(6): 94.1; S(8): 5.9; T(11): 3.0; S(12)		144.6172909	46.2	3
S(1): 100.0; T(3): 0.0		186.9212493	37.44	3
S(2): 0.0; T(12): 1.8; T(13): 1.8; S(25)		156.585285	38.38	5
S(3): 100.0; S(7): 0.0; S(11): 0.0; T(1		411.2178592	60.4	3
T(1): 0.0; T(6): 100.0		254.4665268	38.32	2
T(3): 0.0; Y(6): 0.0; S(7): 0.0; S(10): 1		169.9428657	11.51	2
S(1): 0.0; S(4): 0.1; S(7): 99.9		94.07808488	17.13	2
S(3): 96.9; T(5): 2.9; S(6): 0.1; S(7): 0		147.0833344	23.17	2
S(2): 0.0; S(7): 100.0		232.601941	45.58	2
S(5): 100.0; S(8): 0.0; S(10): 0.0		177.7710823	29.36	2
S(4): 100.0; S(6): 1.9; S(8): 98.1; T(1		240.969529	19.39	2
S(2): 94.7; S(6): 5.3; S(20): 0.0; S(21		95.9357079	15.62	4
S(3): 100.0		100.7602801	19.23	3
Y(3): 0.0; S(8): 100.0		250.4331876	20.32	3
S(6): 100.0		233.0723297	48.93	2
T(1): 0.0; S(14): 100.0; S(20): 0.0		336.0949022	77.69	3
S(6): 100.0		79.67998552	22.15	2
S(1): 0.0; S(3): 100.0		112.2555292	18.12	2
S(1): 100.0; S(7): 100.0		429.8955664	35.51	2
S(1): 0.0; S(6): 100.0		126.7966501	14.48	3
S(5): 0.0; T(10): 0.0; T(13): 0.0; S(16)		186.1817915	38.61	3
T(1): 0.0; S(6): 0.0; S(12): 100.0; S(1		375.9295046	88.71	4
S(1): 0.0; S(5): 100.0		100.4901599	16.1	2
S(1): 0.0; S(6): 0.0; S(12): 100.0		172.8134299	43.88	2
S(1): 100.0; S(5): 0.0; T(6): 0.0		188.1987888	39.84	2
S(1): 0.0; S(4): 0.0; S(7): 1.6; S(8): 1.		192.5365573	21.42	2
S(4): 2.2; S(5): 2.2; S(6): 93.3; S(11):		186.0793357	53.19	2
Y(1): 0.0; S(2): 0.0; S(9): 0.0; T(11): 0		152.3504343	14.42	3
T(2): 99.9; S(3): 0.1; S(5): 100.0		147.3407909	13.85	2
T(2): 0.0; T(3): 0.0; S(6): 100.0; S(8):		231.268634	46.34	2
S(9): 99.6; S(15): 0.3; T(18): 0.0; S(2		111.6203587	15.35	4
T(3): 99.9; S(5): 0.1		121.8940033	17.32	2
S(1): 0.0; S(3): 100.0		382.3123156	82.99	2
T(5): 0.0; S(15): 100.0; Y(26): 0.0		363.5693484	52.76	3
S(2): 0.0; S(4): 0.0; S(7): 0.0; S(8): 0.		348.9085144	60.17	2
S(1): 0.0; S(3): 0.0; S(4): 0.0; T(8): 10		180.3786217	13.87	2
S(1): 0.0; S(3): 100.0; S(5): 100.0		172.9626354	10.48	2
S(2): 0.0; S(3): 0.0; S(7): 99.9; S(9): 0		144.079039	30.27	2

S(1): 0.0; S(2): 0.2; Y(5): 0.5; T(7): 1.	120.3395789	11.7	3
T(10): 0.0; T(17): 100.0	277.2297299	36.47	3
T(1): 0.0; S(14): 100.0; S(20): 0.0	420.1306833	82.27	3
S(6): 2.4; S(7): 2.4; S(8): 95.1	83.18122899	30.23	2
S(1): 96.1; S(3): 49.0; S(5): 50.8; S(6	46.44190668	15.93	2
T(4): 0.0; T(8): 1.4; S(10): 98.6	281.9429992	20.12	2
S(1): 100.0	153.7211257	27.09	2
S(3): 100.0; T(16): 0.0	280.3623006	33.99	3
S(1): 100.0; S(6): 0.0	254.4345881	40.45	3
T(2): 0.0; S(17): 48.7; T(18): 48.7; T(	227.1828705	22.64	4
S(2): 0.0; T(4): 0.0; S(11): 0.0; S(19):	202.1146638	29.61	3
S(7): 100.0	137.2843697	26.05	2
S(15): 31.8; Y(18): 31.8; S(19): 31.8;	133.5729905	16.01	4
S(9): 0.0; S(10): 0.0; S(11): 0.0; S(19	184.4119441	27.21	3
T(2): 0.0; T(6): 1.4; S(8): 98.6	221.6791988	24.1	2
S(4): 0.0; S(5): 99.9; S(6): 0.0; S(7): (	297.2416078	59.75	2
S(3): 100.0; S(7): 0.0; S(11): 0.0; T(1	418.3661452	37.91	3
Y(2): 0.1; S(3): 50.0; S(5): 50.0; S(9):	152.9602854	10.07	2
S(4): 47.7; S(6): 2.3; S(7): 2.3; Y(8): 4	97.07999476	10.48	3
T(1): 50.0; S(3): 50.0; S(5): 100.0; T(	208.6543948	54.23	3
T(1): 0.0; S(2): 100.0; T(5): 0.0; S(15	299.9952897	81.27	2
Y(1): 0.0; S(3): 0.0; Y(11): 0.0; S(12):	223.7887111	39.36	4
T(5): 0.0; S(7): 0.0; T(8): 0.0; S(12): 9	161.240397	34.05	2
T(1): 0.0; S(3): 100.0; S(5): 0.0	159.8962783	12.21	2
S(4): 100.0; S(8): 99.9; S(12): 0.1	166.0999103	16.42	2
S(11): 3.2; S(17): 3.2; T(19): 90.5; S(	121.1957171	6.73	4
S(6): 100.0; S(8): 50.0; Y(9): 50.0	83.78099788	4.32	3
S(2): 100.0; S(4): 0.0	199.5010181	19.64	2
S(8): 100.0	136.9506364	30.23	2
S(4): 100.0; S(8): 0.0; S(10): 0.0; S(2	190.2649578	40.6	3
S(4): 50.0; S(6): 50.0	134.8844339	11.51	2
S(6): 100.0; S(9): 100.0	174.9856426	25.73	2
T(1): 100.0; S(5): 100.0; T(11): 0.0; T	394.376599	40.97	3
T(2): 0.0; T(7): 0.0; T(8): 0.0; S(13): (	191.8237293	21.73	3
S(3): 100.0; Y(5): 0.0	145.6225648	22.75	2
S(4): 100.0	152.4519699	20.04	2
S(3): 100.0	145.0825633	14.2	2
S(4): 100.0; S(7): 0.0	156.0102924	24.83	3
S(1): 0.0; S(3): 0.1; S(7): 99.9	128.6340596	28.25	2
T(3): 0.0; S(7): 0.0; T(8): 1.5; S(10): 9	315.3180637	72.42	2
S(12): 100.0; S(16): 100.0	180.7610284	7.44	3
S(1): 0.0; S(3): 97.5; S(4): 2.5	177.8281905	13.78	2
T(2): 0.0; T(3): 0.0; S(6): 0.0; S(8): 10	171.7836141	13.07	3
T(4): 0.0; S(9): 100.0	213.6751556	52	2
T(2): 0.0; T(3): 0.0; S(6): 99.8; S(8): (	336.621657	35.36	3
S(1): 100.0; S(5): 100.0; S(9): 0.0	112.7156258	6.51	3
T(13): 0.4; S(17): 99.6	131.3486747	6.59	5
S(3): 96.3; S(4): 3.7; T(13): 0.0; Y(15	184.9472156	18.62	3
S(1): 0.0; S(3): 100.0	254.20864	48.82	2
S(1): 0.0; T(2): 0.0; S(3): 0.0; S(6): 1.	149.2054874	41.38	3
T(3): 0.0; S(18): 0.0; S(20): 0.2; S(22	223.6938382	47.04	3
S(1): 100.0	328.3682018	38.22	2
T(1): 11.0; S(3): 94.4; T(6): 94.1; T(9	70.82132119	2.14	3
S(2): 0.0; S(5): 0.0; T(9): 0.0; T(10): (	76.69173112	17.38	3
S(1): 0.0; S(10): 50.0; S(11): 97.5; T(	186.1687638	41.35	3

Information Classification: General

S(4): 0.0; S(7): 100.0	264.4595234	35.97	2
S(5): 100.0	212.955591	26.44	3
S(5): 89.4; S(6): 5.6; S(8): 5.6; S(11):	108.6328793	3.91	3
S(10): 100.0	364.8061416	55.22	2
Y(1): 0.0; S(5): 100.0	138.4831158	26.76	2
S(6): 0.0; S(10): 1.9; S(11): 98.1	184.4148244	47.03	2
S(2): 97.3; S(3): 2.7; S(7): 0.0; T(11):	119.8708424	24.02	2
S(7): 99.9; S(10): 0.1	165.9973719	14.64	3
Y(1): 0.1; Y(3): 0.0; T(4): 0.1; S(7): 97	107.500268	18.2	3
S(7): 100.0	302.7567645	62.49	2
S(1): 50.0; S(2): 50.0	479.0698691	66.63	3
T(2): 0.0; S(6): 100.0; S(10): 0.0; S(1	261.2376771	42.53	2
S(4): 100.0	161.4264384	29.1	2
S(6): 100.0	122.9226859	16.34	2
S(6): 3.0; T(14): 48.5; S(16): 48.5	63.52924579	5.76	3
S(4): 50.0; S(6): 50.0; T(12): 0.0; T(1	235.8938725	32.53	3
S(1): 0.0; T(13): 0.0; T(15): 96.3; S(1	297.2161651	36.22	3
S(7): 0.0; S(12): 0.0; S(13): 50.0; S(1	308.3227893	45.34	3
T(9): 0.0; S(14): 0.1; S(17): 99.9; S(1	369.9449612	104.12	3
S(5): 50.0; S(6): 50.0	191.0509558	36.31	2
T(5): 100.0; S(8): 94.4; S(12): 5.6	113.1421486	2.3	3
S(1): 0.0; S(9): 100.0	138.9444483	18.36	2
T(2): 50.0; S(4): 50.0; S(6): 0.0; T(7):	303.0322504	60.87	3
T(1): 0.0; S(2): 0.0; S(3): 0.0; S(21): (	169.8921616	32.73	5
S(2): 0.0; S(3): 0.0; S(4): 0.0; S(8): 10	156.5267704	34.06	2
S(3): 100.0; T(5): 0.1; S(7): 99.9; T(1	226.8313054	40.49	2
S(1): 0.0; S(3): 49.3; S(4): 49.3; S(6):	240.6682631	49.04	3
S(2): 0.0; S(11): 100.0	359.8116815	78.72	2
S(2): 100.0; Y(5): 50.0; S(6): 50.0	64.62113001	9.73	3
S(2): 100.0; S(4): 100.0; T(8): 0.0	146.6285631	16.65	3
S(5): 98.5; S(8): 1.5	215.9711526	39.77	2
S(6): 0.0; T(8): 0.0; T(9): 0.0; T(15): 2	368.8786796	46.09	3
T(1): 0.0; S(5): 0.0; S(9): 100.0; S(11	220.9970947	16.29	3
S(14): 100.0; Y(20): 0.0	256.8981784	37.3	3
T(2): 0.0; S(11): 100.0; S(17): 0.0	234.4928822	37.55	4
S(3): 100.0; T(5): 100.0	148.6956897	15.62	3
T(2): 0.2; S(8): 99.8; T(16): 4.3; T(17)	183.091302	49.61	3
T(1): 0.0; S(2): 100.0	149.3935178	24.96	2
S(1): 0.0; S(3): 100.0; S(9): 0.0; S(10	179.1774879	23.63	3
S(2): 0.1; S(4): 99.9; Y(12): 0.0; S(15	304.3087258	28.33	3
T(5): 47.3; T(6): 47.3; S(8): 2.7; T(9):	115.2374007	8.95	3
Y(4): 0.0; T(9): 100.0	230.7431037	32.75	3
S(1): 0.0; S(4): 0.0; S(7): 100.0; S(9):	331.8312081	38.39	2
S(1): 0.0; T(8): 52.1; S(12): 52.1; S(1	113.9414911	1.04	4
S(4): 100.0; S(13): 0.0	240.2781452	49.96	2
S(7): 94.9; S(8): 5.0; S(10): 0.0; T(17	161.1245427	30.97	3
S(4): 100.0	195.689473	37.27	3
T(2): 0.0; S(5): 0.0; S(8): 0.0; S(10): (	408.3870156	51.23	3
S(8): 100.0	166.2665978	10.47	3
T(2): 0.0; S(12): 100.0	319.21485	56.36	3
S(3): 100.0	103.2639908	15.76	3
S(4): 100.0	143.7544302	20.11	3
S(3): 99.9; T(5): 0.1; S(9): 0.0; S(17):	432.6059144	87.24	3
T(5): 50.0; S(6): 50.0; T(11): 0.0; S(1	155.9184363	26.28	3
S(2): 0.0; Y(4): 0.0; T(5): 0.0; S(10): (	238.5716145	33.76	3

Y(3): 0.0; T(6): 1.8; S(8): 98.2		212.4012681	68.49	2
S(3): 0.1; S(5): 99.9		342.1177574	68.53	3
T(4): 0.0; T(6): 0.0; S(11): 100.0		205.8509013	46.65	2
S(5): 0.0; S(18): 100.0; S(23): 0.0		246.1835295	45.11	4
S(1): 100.0; S(11): 0.0		247.3963636	70.57	2
S(3): 100.0; S(7): 0.0		192.7163204	33.67	2
S(3): 100.0; T(5): 0.0		315.6723756	30.01	3
S(7): 100.0; S(13): 0.0		240.8765809	24.48	2
S(1): 0.0; S(7): 100.0		218.3150582	25.7	3
S(16): 33.3; T(17): 33.3; S(19): 33.3;		223.2867379	70.63	3
S(2): 0.0; S(3): 0.0; T(6): 0.0; S(7): 0.		194.44226	60.61	3
S(1): 0.0; S(7): 100.0; S(13): 100.0		333.9751302	14.93	3
S(1): 100.0; S(8): 0.0; T(9): 0.0		343.1755898	52.69	3
S(1): 100.0; S(6): 100.0		174.1663945	19.08	4
S(3): 99.9; T(5): 0.1		147.8387818	12.85	2
Y(3): 0.0; Y(8): 0.0; T(13): 100.0		386.9507707	63.57	2
S(4): 100.0		192.4724963	22.61	2
S(2): 0.0; S(4): 50.0; T(6): 50.0; T(17)		265.6819431	59.98	3
S(3): 100.0		97.57848753	10.96	2
T(1): 0.0; T(5): 0.0; S(18): 100.0		291.105834	76.07	3
S(3): 100.0		175.2177014	31.99	3
T(4): 0.0; T(7): 0.0; S(8): 0.0; T(19): 0		64.91516528	16.88	4
S(3): 100.0		124.6992262	8.72	2
S(2): 0.0; S(5): 100.0		248.7193537	31.35	3
T(1): 0.0; S(3): 0.0; T(13): 7.6; S(20):		150.5127005	31.4	4
T(1): 0.0; S(4): 100.0; T(8): 0.0		212.6329305	42.65	2
S(7): 0.0; S(18): 0.0; Y(23): 50.0; S(2		296.0600497	40.22	3
S(1): 0.0; S(7): 100.0; T(10): 0.0		106.9056241	20.46	4
S(3): 0.0; S(12): 100.0		221.9031057	21.67	3
S(1): 0.0; S(3): 0.0; Y(20): 96.3; S(21		180.8183759	12.67	3
S(2): 0.1; S(3): 0.0; T(4): 0.0; S(7): 3.		101.3276095	14.01	2
S(2): 0.0; T(4): 0.0; S(6): 0.1; S(8): 96		223.5661531	14.69	4
S(7): 100.0; T(9): 0.0; S(14): 0.0; S(1		264.2077065	21.17	3
S(1): 100.0; S(10): 0.0; T(16): 0.0; T(		442.854373	84.04	2
S(8): 0.0; S(10): 100.0; S(15): 100.0		273.5274853	19.27	3
S(6): 98.0; S(8): 2.0		141.7488053	10.63	3
S(2): 0.0; S(3): 100.0		237.9328108	31.55	2
T(4): 50.0; S(6): 50.0; S(15): 0.0; T(2		342.8511304	59.4	4
S(7): 100.0		422.7717872	34.95	2
S(1): 33.3; S(2): 33.3; S(7): 33.3		164.4992903	13.96	4
S(1): 6.0; S(2): 86.1; S(3): 6.0; S(6): 0		90.3114983	12.87	3
S(10): 0.0; S(12): 0.0; Y(15): 0.0; S(1		173.8413068	13.48	3
S(9): 100.0		291.4140687	29.27	3
S(5): 100.0; S(8): 0.0; S(14): 0.0		230.8139337	32.7	3
T(3): 0.1; S(5): 3.4; S(7): 96.4; S(9): 0		317.6628633	43.51	4
S(1): 0.0; S(3): 0.0; S(7): 100.0; S(13		385.7826746	52.89	2
S(2): 0.1; S(5): 0.1; S(8): 0.1; T(9): 1.		173.9962436	22.27	3
S(1): 100.0; S(7): 100.0; S(13): 100.0		248.8541713	24.38	3
S(5): 99.8; T(10): 0.2		143.8320599	14.4	3
S(2): 47.7; S(3): 47.7; S(4): 1.5; S(6):		166.3132522	33.09	3
T(3): 0.0; S(10): 100.0; T(14): 0.0; T(		214.2980026	35.17	3
S(1): 0.0; S(3): 100.0		269.6432043	40.5	2
T(2): 0.3; S(5): 5.3; S(8): 94.4; S(11):		231.9475659	37.89	4
S(3): 100.0; S(8): 0.0; T(10): 0.0; S(1		244.1161977	68.82	2
S(1): 0.2; Y(5): 99.8; T(14): 0.0; S(15		98.58914493	37.36	3

Information Classification: General

S(4): 95.8; T(8): 4.2; T(9): 0.0; S(11):	68.74497568	7.09	3
S(3): 100.0; S(4): 0.0; S(14): 0.0	288.813936	40.17	2
S(3): 100.0	135.6223004	4.62	2
S(3): 100.0; S(8): 100.0	534.5749177	39.13	2
S(6): 99.9; T(8): 0.1; T(11): 0.0; T(18)	214.5108993	18.73	3
S(10): 100.0; S(13): 0.0; T(14): 0.0; S	223.2005099	13.78	3
S(3): 100.0; S(6): 0.0	106.862311	13.38	3
S(2): 0.0; S(5): 100.0	185.7618754	41.19	2
S(6): 100.0; S(12): 100.0	404.3491595	30.36	3
S(7): 100.0; T(19): 0.0	280.1328686	39.84	4
S(13): 100.0; S(19): 0.0; T(23): 0.0; T	266.9120352	27.28	4
S(1): 0.0; S(4): 100.0; S(5): 100.0; T(	337.6093133	41.85	3
S(3): 100.0; Y(15): 0.0	188.2307164	17.34	3
Y(2): 0.0; S(7): 100.0	288.8262237	29.85	3
Y(1): 0.0; S(6): 100.0; S(11): 0.0	281.1899459	45.72	2
S(3): 99.9; S(5): 0.1; S(11): 0.0	268.3879229	46.91	3
S(3): 100.0; S(11): 0.0	229.3616002	30.99	2
S(3): 0.0; S(5): 0.1; S(6): 97.5; S(9): 2	181.7838967	45.92	2
T(7): 0.0; S(9): 0.0; S(11): 0.0; S(13):	128.545779	23.65	5
T(1): 0.0; Y(7): 0.0; Y(9): 0.0; S(14): 9	213.4841466	32.8	4
S(3): 0.0; S(9): 99.0; S(12): 1.0	284.5074747	35.58	3
Y(2): 0.0; S(3): 0.0; S(4): 0.0; S(6): 0.	159.9184555	25.38	3
S(3): 0.0; S(4): 0.0; T(7): 0.0; S(9): 0.	68.55273689	20.05	3
S(3): 6.2; S(8): 93.8; S(12): 93.4; S(1	146.594634	18.62	4
T(1): 0.0; S(11): 100.0	493.2728672	63.09	2
S(3): 91.5; S(4): 2.8; S(5): 2.8; S(6): 0	148.073915	42.71	2
T(3): 0.0; T(7): 1.7; S(8): 98.3	214.7492691	38.26	2
S(5): 100.0; S(7): 0.0	179.646557	24.72	2
T(1): 0.0; S(2): 2.1; S(3): 97.9	203.4783938	23.16	2
S(3): 98.3; S(5): 1.7; T(9): 0.0; S(17):	472.9882	46.92	3
S(3): 100.0; S(4): 100.0; S(14): 0.0	182.7539988	32.65	2
S(3): 0.0; S(14): 100.0	259.8657523	23.95	3
Y(2): 99.9; S(7): 0.1; S(9): 0.0; S(11):	131.3317972	8.83	3
S(1): 0.0; S(5): 99.9; T(12): 0.0; S(14	151.5546004	14.28	3
S(3): 0.1; S(4): 99.9; S(8): 0.0	198.3004663	37.21	2
S(5): 0.0; T(7): 0.0; S(21): 3.1; S(23):	270.1485376	29.11	3
S(3): 97.6; S(9): 2.5; S(15): 99.7; S(1	216.1809726	33.93	4
S(2): 48.9; S(3): 48.9; S(4): 2.1; S(7):	175.0388055	37.79	3
S(3): 0.0; S(8): 48.8; T(13): 2.4; S(16	113.6126067	5.23	3
S(3): 100.0; T(7): 0.0; S(10): 0.0	255.7964412	26.83	2
S(3): 100.0	401.3713636	81.49	2
T(3): 0.0; S(5): 0.0; S(8): 97.7; S(9): 0	226.7487329	79.77	2
S(3): 100.0	190.2387151	41.74	2
S(2): 100.0; S(6): 0.0	230.1191855	30.39	2
S(4): 0.0; T(11): 0.0; S(17): 0.0; S(26	233.2704418	27.42	4
Y(5): 0.0; Y(6): 0.0; T(7): 0.0; S(8): 0.	440.6561665	106.74	3
S(3): 50.0; T(4): 50.0	144.8829211	26	2
S(6): 100.0	282.2594177	52.4	2
S(1): 0.0; S(2): 100.0	334.5962002	76.45	2
S(3): 100.0; S(6): 100.0; S(20): 0.0	517.2534927	82.48	3
S(5): 100.0	104.9104382	17.03	2
S(19): 0.0; S(20): 0.0; T(28): 0.0; S(3	373.2371293	57.52	3
S(2): 0.0; S(3): 100.0	204.918326	42.12	2
Y(9): 4.6; S(10): 95.4; S(17): 0.0; S(1	123.4401989	15.8	3
S(4): 100.0	147.2770032	18.51	2

Information Classification: General

S(3): 50.0; Y(4): 50.0; S(5): 99.9	79.00108074	6.58	4
S(1): 0.0; T(3): 2.8; S(4): 97.2; T(7): (	94.24390247	13.33	3
T(1): 0.0; S(9): 100.0; S(17): 0.0	414.4622291	55.48	3
S(6): 50.0; S(9): 50.0	124.6962595	16.27	2
T(2): 0.0; T(3): 0.0; S(6): 49.3; S(8): 4	196.5937105	13.77	3
S(1): 0.0; S(7): 100.0	224.2370229	10.95	3
S(3): 100.0; S(17): 0.0; S(18): 0.0; T(	205.6468554	12.46	3
S(1): 0.0; S(5): 100.0; S(9): 0.0	242.9120909	36.01	3
S(9): 100.0	159.5949065	22.52	2
S(3): 100.0; S(7): 0.0; S(11): 0.0; T(1	316.1466873	35.88	3
S(3): 100.0; S(5): 0.0; S(6): 0.0; S(8):	152.3958511	16.41	3
T(4): 1.8; S(6): 98.2	429.2002777	67.61	3
T(2): 2.8; S(3): 97.2; S(5): 0.0; T(6): (	160.6339619	38.68	2
T(1): 0.0; S(4): 100.0; S(6): 0.0; S(9):	322.768502	51.1	2
T(3): 0.0; T(5): 0.0; S(12): 0.1; S(13):	366.0367876	32.67	3
T(4): 1.9; Y(7): 49.0; S(8): 49.0; S(10	181.2970593	16.42	3
S(6): 100.0; S(11): 0.0; S(15): 0.0	139.9127814	9.64	4
S(20): 0.0; T(22): 0.0; T(25): 66.7; T(	189.0298179	37.4	3
S(4): 100.0	186.2572252	32.34	3
S(1): 1.6; S(2): 98.4; Y(7): 0.0	187.2470654	22.47	2
T(5): 0.0; T(8): 0.1; T(9): 3.1; T(10): 9	251.3404601	72.37	3
S(1): 4.4; S(3): 95.6; S(5): 100.0; T(1	225.7486756	20	3
S(1): 50.0; S(3): 50.0; S(11): 0.0; S(1	218.0495957	36.09	3
Y(1): 0.0; S(7): 100.0	164.957173	37.27	4
S(3): 100.0; S(6): 0.0	209.5040236	28.68	3
S(4): 0.2; S(9): 99.8; S(11): 95.7; S(1	199.4709539	32.74	3
S(3): 0.0; T(4): 0.0; S(5): 0.0; S(13): 1	380.5706208	74.35	2
S(5): 100.0; S(8): 0.0; S(9): 0.0	169.8679736	11.29	2
S(3): 0.1; S(4): 99.9; T(5): 0.1; S(7): (	197.4582156	16.87	2
S(1): 100.0; S(7): 100.0	524.1647527	41.19	2
S(6): 0.0; T(11): 0.0; T(13): 0.1; S(15)	198.3636095	31.56	3
S(1): 100.0; S(7): 100.0; T(10): 0.0	385.3845128	39.77	2
S(25): 50.0; S(26): 50.0; S(33): 0.0	146.3731747	16.28	4
S(3): 0.1; S(6): 0.1; T(8): 99.9	84.39103504	19.6	2
S(1): 0.0; S(5): 100.0	165.8529335	14.41	3
S(3): 100.0; S(4): 0.0; S(6): 0.0	130.5447482	20.14	2
S(5): 0.0; T(12): 0.0; S(17): 0.0; S(21	371.8225191	50.91	4
S(10): 100.0; T(19): 0.0	283.7791255	31.07	3
Y(2): 0.0; S(4): 33.1; T(5): 33.1; S(6):	163.5507673	21.38	2
S(1): 0.0; S(7): 100.0	443.1914292	80.18	2
S(3): 0.0; T(5): 0.0; Y(7): 0.0; S(9): 10	143.2953207	19.84	2
S(2): 0.0; T(3): 0.0; S(9): 0.0; S(14): 9	373.1220248	101.65	3
S(3): 98.4; S(5): 1.6; S(9): 0.0	207.1396845	43.19	3
S(2): 0.0; S(4): 0.0; S(9): 100.0; T(16	275.3710762	40.64	2
S(8): 0.0; S(18): 1.6; S(19): 88.1; S(2	87.1242451	9.74	5
S(4): 0.0; S(5): 0.0; T(8): 0.0; S(10): (	92.09958741	35.28	3
S(3): 0.0; T(7): 0.0; T(9): 0.0; T(16): (	238.2644353	42.14	4
S(6): 100.0; T(10): 0.0	311.0181924	45.43	2
S(3): 0.0; T(4): 0.0; S(11): 50.0; S(12	176.5467306	30.2	3
S(3): 99.9; S(5): 0.0; S(7): 0.0; S(9): (	123.1092823	16.08	2
Y(7): 0.0; T(11): 50.0; S(12): 50.0	226.7926454	62.61	3
T(13): 0.0; S(19): 100.0; S(23): 0.0; S	457.0016792	123.45	3
S(3): 2.0; S(4): 98.0; T(13): 0.0; T(14)	217.9630531	47.85	3
S(3): 100.0	261.1205304	47.36	2
S(5): 100.0; T(7): 0.0	227.279984	38.45	2

Information Classification: General

S(3): 3.5; T(7): 89.6; T(16): 3.5; S(17)	183.2032591	2.96	3
Y(7): 0.0; S(9): 100.0; S(11): 100.0; T	306.1140189	38.72	3
S(18): 100.0	300.584291	57.11	3
S(4): 0.0; S(11): 100.0; T(21): 0.0; T(	288.9278916	61.56	3
S(9): 97.6; T(11): 2.4	135.4865997	9.23	4
S(1): 0.0; T(3): 0.0; T(6): 3.3; T(11): 9	275.0772482	53.35	3
S(6): 100.0	100.0037841	20.75	2
Y(2): 0.0; S(5): 100.0	137.1940676	24.53	2
S(5): 50.0; S(6): 50.0; T(12): 0.0; T(2	237.5616569	32.31	3
T(2): 0.0; S(5): 0.0; S(15): 100.0	194.2982625	33.62	4
S(5): 100.0; T(18): 0.0	418.1319401	51.06	3
S(3): 100.0; S(7): 0.0; S(11): 0.0; T(1	355.0820974	31.12	3
S(4): 0.2; Y(5): 0.2; Y(6): 99.6	91.20434256	11.06	3
S(9): 100.0; S(16): 0.0; T(17): 0.0	280.9021181	43.86	3
S(3): 98.9; S(4): 50.6; S(5): 50.6	163.9038787	25.56	2
S(11): 99.9; S(13): 0.1	271.5187543	31.01	3
S(1): 0.0; S(6): 100.0	143.8797642	12.77	2
S(6): 0.0; S(7): 0.0; T(9): 0.0; S(10): (	193.7143601	16.97	3
Y(7): 0.0; Y(9): 0.0; S(13): 100.0	466.5953355	45.92	3
Y(2): 0.0; S(10): 100.0	254.3084055	45	2
S(2): 0.0; S(6): 100.0	227.5004997	29	3
T(1): 0.0; S(5): 100.0	237.87951	33.29	2
S(3): 33.3; Y(4): 33.3; S(5): 33.3; S(7	202.6479027	52.05	2
S(1): 0.0; S(4): 50.0; S(5): 50.0; T(10	227.471037	34.92	3
S(2): 1.8; S(3): 49.1; S(5): 49.1	156.0355575	9.88	3
S(3): 100.0; S(5): 0.0; S(9): 0.0; S(11	195.2962303	31.42	2
S(1): 0.0; S(15): 100.0	288.0974942	17.82	3
T(2): 0.1; T(5): 0.1; S(6): 0.1; S(11): 9	152.6924685	30.7	3
S(5): 100.0; S(8): 0.0; T(12): 0.0; S(2	335.3890091	62.26	3
S(10): 100.0; S(17): 0.0	222.7068703	37.93	4
S(3): 100.0; Y(7): 0.0; S(9): 0.0; S(10	191.3058814	29.11	3
S(4): 0.0; S(6): 0.1; S(10): 99.9	187.1452722	17.06	3
S(2): 0.0; S(13): 49.9; T(15): 49.9; S(	128.8816735	19.69	4
S(3): 100.0; S(6): 96.3; S(8): 3.6; S(9	246.2466123	23.98	3
T(2): 0.0; S(5): 50.0; S(6): 50.0; S(12	204.7498237	41.18	2
T(1): 0.0; S(5): 0.0; S(6): 0.0; T(10): (	267.0196256	44.8	4
S(2): 0.0; S(5): 0.0; S(10): 0.0; Y(12):	209.0711948	34.55	3
S(1): 0.2; S(4): 0.2; S(6): 3.8; S(7): 95	153.9205871	32.82	3
S(3): 100.0; S(19): 0.0	369.6585448	64.3	2
S(1): 100.0; T(10): 0.0	353.8625264	58.53	2
S(8): 100.0; S(9): 100.0; S(11): 92.4;	211.816905	17.92	3
S(2): 0.0; S(5): 100.0; S(16): 0.0; T(2	320.6965568	59.05	3
T(1): 0.0; S(18): 0.0; S(24): 0.1; S(25	586.1455849	106.37	3
S(3): 0.0; S(7): 100.0; S(13): 66.7; T(	129.8287181	5	3
S(5): 100.0; S(7): 0.0; Y(8): 0.0	144.3567633	19.45	2
T(1): 0.0; S(3): 0.0; S(8): 50.0; S(10):	190.3796681	29.74	2
S(3): 0.0; S(9): 0.0; T(13): 0.0; S(16):	332.3937619	36.17	3
S(8): 100.0	231.2549331	22.58	3
S(5): 96.0; S(8): 4.0; T(12): 0.0; S(20	172.5232726	38.91	3
S(9): 100.0	501.5915743	102.74	2
S(7): 0.0; S(10): 100.0; S(11): 100.0	111.2176719	32.29	2
T(1): 0.0; S(2): 1.1; S(3): 49.4; T(5): 4	240.1783377	44.47	2
T(1): 0.0; S(3): 100.0	151.1706871	19.55	2
S(5): 0.0; S(28): 4.9; T(29): 4.9; T(31	139.1935247	10.77	4
S(3): 0.0; S(5): 0.0; S(9): 100.0; T(11	279.0121158	36.87	2

T(3): 50.1; S(6): 50.1; S(9): 99.9; T(1	161.9095696	36.1	3
S(6): 0.0; T(7): 0.0; S(14): 100.0; S(1	326.8636739	74.56	3
S(2): 1.4; T(3): 49.3; S(4): 49.3; T(7):	96.43132937	18.81	2
T(1): 0.0; S(9): 100.0	236.6537987	40.54	2
S(2): 0.0; T(8): 0.0; S(12): 100.0; T(1	428.2064255	118.23	3
Y(1): 0.0; S(2): 0.0; T(5): 0.0; S(16): 1	330.3616465	24.79	3
S(1): 0.0; T(4): 0.0; T(7): 0.3; T(9): 0.	218.949597	25.53	3
S(1): 100.0; S(7): 100.0	482.6053857	36.45	2
S(3): 100.0; S(6): 0.0	115.6957857	9.04	2
S(1): 0.0; S(4): 0.0; S(10): 91.2; Y(23	72.29534158	0.59	7
S(8): 99.9; T(10): 0.1	98.55860922	8.66	3
T(1): 100.0; S(5): 100.0; T(11): 0.0; T	322.7469361	41.71	3
S(1): 100.0; S(3): 100.0; S(7): 0.0	154.7817248	15.48	2
S(1): 100.0; S(3): 0.0	206.5486351	55.25	2
Y(4): 100.0; T(13): 100.0; Y(14): 99.7	78.33876522	20.86	4
S(3): 0.0; T(5): 0.0; T(19): 1.9; S(20):	323.4013096	43.4	2
T(3): 0.2; S(5): 4.2; S(8): 91.4; S(11):	126.4414553	30.42	3
Y(5): 0.0; S(6): 0.0; S(10): 100.0; T(1	269.095886	31.9	2
S(8): 99.5; S(9): 99.5; S(11): 53.1; T(	85.70589136	20.75	3
T(5): 0.0; S(6): 0.1; S(8): 99.9; S(17):	177.8245113	31.05	3
S(5): 100.0; S(11): 0.0	111.8242267	15.92	2
S(4): 0.0; T(5): 0.0; S(16): 100.0; S(1	125.6109205	10.63	3
S(12): 100.0	156.2533004	9.84	3
T(7): 2.7; S(8): 97.3	173.2520805	30.31	2
S(1): 0.0; S(3): 100.0; T(5): 0.0; T(7):	334.3548412	78	2
T(5): 50.0; S(6): 50.0	116.8943286	26.83	2
T(4): 0.0; S(6): 100.0	220.2402358	33.23	2
T(2): 49.0; S(3): 49.0; S(5): 1.9	75.36868397	14.57	3
S(1): 0.0; S(2): 100.0	335.0055802	76.55	2
S(1): 100.0; S(7): 100.0	402.6289237	27.86	2
Y(6): 0.0; S(13): 100.0	433.357769	81.03	3
S(6): 0.0; S(11): 0.9; S(13): 49.6; S(1	202.260073	39.8	2
S(3): 0.0; S(10): 100.0	232.9886366	36.83	3
S(4): 100.0; T(12): 0.0	341.9837736	45.59	3
S(8): 100.0	272.1795395	11.92	2
S(4): 100.0; S(9): 0.0	136.7160237	14.74	2
S(4): 0.0; T(8): 0.0; S(10): 0.0; S(13):	299.4907486	95.26	3
S(6): 0.0; S(12): 100.0	237.0184743	54.68	2
S(6): 98.5; Y(7): 1.5	180.3134396	25.2	2
S(1): 4.0; T(8): 31.0; T(11): 31.0; S(1	43.63599804	7.76	4
S(4): 100.0	167.6650734	32.24	2
S(3): 100.0	120.4821195	3.8	3
S(1): 0.1; S(2): 99.9; T(5): 99.9; T(6):	222.1642554	47.84	2
S(4): 0.0; S(6): 0.0; S(14): 100.0; S(2	337.6779808	60.32	3
T(1): 0.0; S(5): 100.0; T(13): 0.0; S(1	299.4015393	37.29	3
S(5): 0.0; S(7): 2.3; T(10): 97.7; S(22	199.0756304	7.72	4
T(3): 0.0; S(5): 100.0; S(9): 100.0; S(	310.3240944	46.2	2
S(4): 0.0; S(6): 0.0; S(12): 100.0	278.7775527	44.96	2
S(2): 0.0; S(6): 100.0; S(13): 0.0; S(1	246.2955632	43.12	3
T(1): 0.0; S(4): 1.7; T(6): 98.3	187.361017	48.38	2
S(4): 100.0	108.3820298	11.44	2
T(2): 0.0; S(4): 1.4; T(5): 1.4; S(6): 48	116.9474226	14.02	2
T(3): 4.3; Y(7): 91.4; S(9): 4.3; S(12):	115.4228305	13.34	3
T(1): 0.0; T(5): 0.0; S(18): 100.0	167.1606577	26.84	3
Y(6): 0.0; S(7): 0.1; S(10): 99.9; S(12	249.3565982	54.72	2

Information Classification: General

S(3): 100.0; T(6): 0.0; T(9): 0.0		169.6825283	29.47	3
T(10): 100.0		164.9144504	18.01	3
S(1): 100.0; T(5): 0.0; T(12): 0.0		236.7135988	42.23	2
S(2): 0.0; S(4): 100.0; S(8): 0.0		228.962837	35.58	2
S(3): 100.0; T(8): 0.0		155.1643172	25.52	3
S(7): 100.0; T(11): 0.0		228.4497316	37.02	3
T(4): 50.0; S(5): 50.0; S(7): 0.0; S(11		230.1539799	42.75	2
S(1): 0.0; S(3): 100.0		184.2678394	16.55	3
T(7): 0.0; S(12): 100.0		250.6190264	55.89	2
S(15): 100.0; S(26): 0.0; S(30): 0.0		98.04512926	16.47	3
S(3): 100.0; Y(7): 0.0		183.1175116	16.62	3
S(13): 49.6; S(14): 49.6; T(16): 0.9		289.4945953	53.08	3
S(5): 2.0; S(6): 98.0		154.6037503	10.06	2
S(5): 6.4; T(13): 93.6; T(24): 0.0		101.1682391	4.67	4
T(3): 6.1; S(5): 93.7; S(8): 3.4; S(10):		125.3050001	47.29	3
S(1): 0.0; S(3): 100.0		146.7045052	16.67	2
S(4): 50.0; S(5): 50.0		109.6311394	23.26	3
S(1): 0.0; S(5): 100.0		219.6191591	30.54	2
S(1): 0.0; T(5): 100.0		209.4181531	24.82	4
S(1): 0.0; S(7): 100.0		359.1115661	44.16	2
S(1): 0.0; T(3): 0.0; T(6): 0.0; S(9): 0.		183.4082509	57.67	3
S(1): 0.2; S(2): 3.6; S(7): 85.2; T(11):		177.420654	39.54	3
S(7): 100.0; S(9): 0.0		302.5688007	45.37	2
S(4): 100.0; S(10): 0.0; S(12): 0.0; S(		179.8461339	54.4	2
S(5): 100.0; S(14): 0.0		326.0648421	42.77	3
S(1): 100.0		420.2906032	35.67	2
S(1): 0.0; S(6): 0.0; S(10): 100.0; S(1		444.7102663	68.51	3
S(2): 94.4; T(6): 5.3; S(7): 0.3; T(14):		61.60589946	4.21	3
S(2): 0.0; S(15): 0.0; T(16): 0.0; S(23		336.0662277	42.55	3
S(1): 0.0; S(8): 2.0; S(10): 98.0		156.8512363	19.5	2
S(9): 50.0; S(12): 50.0; T(21): 0.0; S(		203.6424232	63.48	3
S(3): 99.9; S(5): 0.1		109.8305133	21.05	2
S(1): 0.0; S(7): 100.0		394.0416754	51.92	2
T(3): 32.9; T(4): 32.9; Y(6): 1.4; S(8):		64.5409966	14.94	2
S(3): 0.0; S(7): 0.0; S(11): 0.0; T(15):		268.5208954	35.07	4
S(4): 100.0; T(6): 0.0; S(8): 0.0; S(11		219.7857098	52.58	3
S(9): 100.0		275.8502471	43.41	2
S(3): 100.0; S(6): 100.0; T(9): 0.0		250.3496003	52.3	2
T(3): 0.0; S(9): 0.0; S(12): 0.0; S(17):		169.496152	19.03	3
S(6): 100.0		118.971353	21.9	2
S(8): 0.0; S(10): 1.2; S(11): 98.8		408.9603202	69.39	2
S(1): 0.0; S(3): 100.0; S(5): 100.0; T(		240.9734653	47.18	2
S(4): 0.0; S(5): 0.0; T(8): 100.0		281.4168499	76.77	2
S(2): 100.0; T(7): 0.0; T(16): 0.0; T(2		162.5057461	15.82	4
Y(4): 0.0; S(7): 100.0; Y(9): 0.0		286.9178544	55.74	2
S(3): 100.0		362.2753413	25.03	2
S(2): 97.3; T(3): 2.7; T(7): 0.0; Y(10):		283.0573966	52.52	3
T(5): 48.1; S(6): 48.1; S(8): 0.0; T(10		73.61801915	12.24	5
T(2): 0.0; T(7): 0.0; S(9): 100.0		313.0038159	50.48	2
S(1): 0.0; S(6): 100.0		205.6809657	37.43	2
S(6): 50.0; T(8): 50.0		149.7121835	23.97	2
S(2): 0.0; S(5): 100.0; S(10): 0.0; Y(1		355.9962086	55.33	2
Y(9): 0.0; S(10): 0.0; T(13): 0.0; T(15		272.0025427	37.32	3
S(3): 100.0; S(9): 100.0		126.8482615	21.65	3
S(1): 100.0; S(7): 100.0		323.789846	29.77	2

T(1): 0.0; S(2): 0.0; S(12): 48.9; S(13	195.7576853	70.31	3
S(1): 0.0; S(4): 100.0	236.6793622	50.12	2
T(1): 0.1; S(8): 49.9; S(10): 49.9; S(1	179.2673163	30.68	3
S(1): 4.9; S(2): 65.0; S(3): 65.0; S(4):	205.2877023	44.81	3
S(1): 0.0; S(9): 100.0	317.3012286	16.78	2
S(1): 100.0; T(2): 0.0; S(5): 0.0	206.9246391	21.37	3
Y(3): 0.0; S(4): 50.0; S(6): 50.0; T(12	231.215401	32.07	2
S(1): 0.0; S(7): 100.0; S(13): 100.0	346.3486457	43.34	2
S(4): 100.0; S(9): 0.0	110.9569345	13.48	3
S(3): 0.0; S(10): 97.6; S(11): 2.4	225.3219425	29.33	3
S(1): 100.0; S(3): 99.8; S(5): 0.2; S(9	98.45032529	25.55	3
S(1): 100.0; S(5): 0.0; S(6): 0.0; T(7):	256.4796097	83.66	2
T(1): 0.0; S(11): 100.0; S(16): 100.0	276.5020441	13.03	3
S(2): 100.0; S(5): 100.0; Y(10): 0.0; S	430.6598743	79.63	2
S(5): 100.0	219.2948292	37.87	2
S(2): 100.0	304.2862764	40.44	2
S(7): 100.0; S(11): 0.0	298.241857	49.02	3
S(4): 100.0; S(11): 0.0; S(12): 0.0	266.3694392	38.37	2
S(3): 100.0; T(12): 0.0; S(13): 0.0	237.1352844	47.08	3
S(3): 100.0; S(5): 0.0	146.9137755	30.27	3
S(5): 0.0; S(11): 100.0	154.3484755	30.51	3
S(11): 100.0	260.4930225	53.92	3
S(4): 100.0; T(10): 0.0	205.795752	17.46	3
S(8): 100.0; T(12): 0.0; S(13): 0.0; T(	263.6704249	64.8	2
S(1): 0.1; S(3): 99.9; S(6): 50.0; T(8):	71.29368844	12.56	2
T(1): 50.1; S(3): 50.1; S(4): 95.6; T(6)	90.57115901	17.23	2
T(3): 0.0; T(5): 0.0; S(12): 98.2; T(16)	367.4566857	34.01	3
S(1): 0.0; T(10): 100.0; T(12): 0.0	160.1544116	23.35	2
S(3): 0.0; T(11): 0.0; S(13): 100.0; S(	551.7747765	55.89	3
S(8): 0.0; S(12): 0.0; T(18): 0.0; S(21	344.6363042	45.85	3
S(5): 100.0	399.8837689	74.13	2
T(1): 100.0; S(5): 0.0	223.1882623	28.49	2
T(1): 0.0; T(4): 0.0; S(5): 0.0; T(9): 10	190.1420187	28.14	2
S(1): 0.0; S(3): 100.0; S(5): 0.0	141.9717286	19.34	2
T(1): 0.0; S(9): 100.0; S(17): 0.0	388.5408949	105.62	2
S(2): 50.0; S(3): 50.0; T(16): 0.0; T(1	179.6293968	16.62	3
S(4): 100.0; S(10): 0.0; S(14): 0.0; S(	253.4149422	33.35	3
S(7): 50.7; S(8): 50.7; S(10): 49.3; T(	199.4230244	31.93	3
S(2): 100.0; S(4): 0.0	120.8629544	16.74	2
S(1): 0.0; S(2): 0.0; S(9): 2.5; T(10): 2	230.9320869	12	3
S(1): 0.0; S(4): 2.2; S(5): 97.8; T(10):	257.7898972	31.41	3
T(3): 100.0	121.5295847	12.13	4
S(2): 0.0; S(3): 100.0; S(10): 0.0	281.5764152	58.26	2
S(1): 0.0; S(3): 100.0; S(10): 0.0; S(1	473.0685003	112.4	2
Y(6): 0.0; S(13): 100.0	709.1887212	56.71	3
S(1): 0.0; S(21): 100.0	402.1773752	55.72	3
S(2): 0.0; S(4): 100.0; S(7): 100.0	221.5655249	27.27	2
S(2): 1.5; S(3): 98.5	386.1732489	78.75	2
T(14): 0.0; T(15): 0.0; T(20): 1.8; T(2	459.172242	92.91	3
T(3): 0.0; T(6): 4.8; T(11): 90.3; S(14)	96.32842407	2.64	2
Y(1): 0.0; S(10): 96.3; T(12): 3.7	105.688396	14.11	3
S(1): 0.0; T(2): 0.0; S(5): 0.0; T(8): 0.	168.2616699	38.36	3
S(5): 96.0; S(13): 52.0; S(14): 52.0; S	102.7346725	29.26	3
S(8): 100.0; T(10): 0.0	254.8175228	38.9	3
S(2): 0.0; S(5): 95.2; T(7): 4.5; T(9): 0	194.9265925	27.48	3

Information Classification: General

T(1): 50.0; S(4): 50.0; T(6): 99.9; S(1	61.83969385	14.65	3
S(1): 0.0; T(5): 0.0; S(8): 100.0	242.8888035	45.75	2
Y(2): 0.0; S(3): 0.0; S(4): 0.0; S(12): 1	194.26662	31.34	3
S(5): 100.0	214.9066426	14.76	2
S(1): 0.0; T(4): 0.0; S(7): 100.0; S(13	610.0760506	48.08	2
S(1): 0.0; T(3): 50.0; S(4): 50.0; T(7):	269.9889951	70.84	2
S(1): 0.0; S(3): 100.0; S(10): 0.0	187.1961213	22.75	2
T(3): 96.5; S(4): 3.5	87.02754606	20.42	3
T(2): 0.0; S(9): 2.4; S(10): 97.6; S(12	259.0586398	42.33	2
S(1): 100.0; S(6): 0.0	264.1284128	32.31	2
S(4): 0.0; S(6): 100.0; T(20): 0.0	145.1056292	9.33	4
Y(2): 0.0; S(3): 0.0; T(5): 49.4; T(6): 1	146.1246931	18.38	2
T(11): 100.0	279.8271009	40.08	2
T(6): 0.0; S(9): 100.0	291.7551119	31.53	2
S(13): 50.0; T(19): 50.0	164.8308708	33.16	2
S(5): 100.0; S(7): 0.0	160.4872366	26.04	3
S(2): 0.0; S(3): 0.0; T(6): 0.0; S(8): 10	403.4722923	114.05	2
S(10): 100.0; S(12): 0.0; T(15): 0.0; S	335.2026363	55.09	3
S(5): 0.0; S(9): 100.0	281.7344304	37.6	2
S(4): 50.0; S(6): 50.0	181.7198149	35.07	2
S(2): 0.0; S(20): 94.9; S(22): 5.1	143.2931903	8.95	4
S(3): 0.0; T(4): 0.0; T(6): 0.0; T(9): 0.	185.6638738	48.27	3
S(1): 4.1; T(2): 4.1; S(3): 91.6; S(6): 0	187.2859393	3.02	3
S(4): 0.0; T(11): 0.1; S(13): 6.2; S(15	172.0770451	5.69	3
S(4): 100.0	292.0696436	72.82	2
T(10): 0.0; T(13): 100.0	424.1970932	62.72	3
S(8): 0.0; S(10): 0.0; S(14): 0.0; S(16	637.8846055	75.68	2
S(2): 0.0; T(6): 32.8; S(8): 32.8; S(9):	197.6786308	39.95	2
S(4): 100.0	165.0398285	11.8	3
Y(6): 0.0; S(20): 100.0; S(23): 100.0	143.5421488	10.84	4
S(1): 51.0; S(3): 51.0; S(5): 98.1; S(1	142.3226629	5.18	3
S(7): 0.0; S(9): 0.0; S(12): 0.0; T(13):	141.0888219	41.42	3
S(8): 100.0	215.9152243	8.86	3
T(1): 0.3; S(6): 33.1; T(7): 33.1; S(9):	63.2809768	1.34	3
S(6): 3.2; S(10): 3.2; S(14): 3.2; S(15	102.2987981	8.39	6
T(4): 0.0; S(9): 1.9; S(11): 96.2; S(13	184.0945382	28.98	2
S(1): 100.0; S(5): 100.0	224.565845	22.95	2
S(3): 100.0; S(8): 0.0; T(12): 0.0	268.1675726	56.27	3
S(7): 0.0; T(9): 0.0; T(11): 0.0; S(12):	299.0284296	32.92	3
T(8): 98.7; T(10): 1.3	231.1645412	49.63	2
S(2): 0.0; S(5): 100.0; T(8): 0.0; T(16	391.1786879	78.21	2
T(2): 0.0; S(6): 0.0; T(12): 0.0; S(16):	260.6318879	95.88	3
S(7): 50.0; S(12): 50.0	108.265442	11.67	4
S(5): 100.0; T(13): 0.0	249.1756822	30.05	3
S(3): 100.0	232.9635402	8.73	3
S(9): 33.3; T(10): 33.3; T(11): 33.3; S	129.8287181	41.31	4
T(2): 1.7; S(3): 98.3	172.0519352	25.48	2
T(1): 0.0; S(3): 100.0; T(10): 0.0	237.4560941	33.16	2
T(6): 0.0; S(8): 0.0; S(17): 100.0; S(1	287.5530776	102.46	3
S(1): 0.0; S(7): 100.0; S(13): 50.0; S(	365.5901446	43.19	2
Y(1): 0.1; T(4): 49.0; T(5): 49.0; T(6):	244.5504487	43.37	3
T(8): 0.0; S(13): 49.3; S(15): 49.3; S(	264.1665227	54.1	3
S(11): 0.0; T(15): 0.0; Y(21): 0.0; T(2	206.4789366	29.89	4
T(5): 100.0	133.7938035	5.71	4
T(5): 0.0; T(8): 0.0; T(9): 0.1; T(10): 9	252.0788262	72.18	3

Information Classification: General

Y(1): 0.0; S(3): 100.0		191.4783993	24.08	2
S(7): 50.0; S(8): 50.0; S(10): 0.0; S(1		88.93390434	11.14	3
T(2): 49.4; T(5): 49.4; S(7): 1.1; S(9):		234.1230007	62.09	3
S(4): 50.0; S(7): 50.0; Y(13): 0.0		219.3007974	19.09	3
S(7): 0.0; S(12): 0.0; S(13): 0.1; S(15		208.8175214	49.59	3
S(3): 100.0		139.4092605	20.5	2
Y(1): 0.0; T(7): 0.0; S(10): 0.0; T(12):		207.4261197	21.02	3
S(8): 100.0		116.3076051	23.45	2
S(10): 100.0; S(13): 99.9; S(14): 0.1;		362.1545259	97.73	3
S(12): 100.0		396.8742628	62.54	3
T(3): 0.0; S(7): 100.0		155.3996227	7.69	3
S(4): 100.0		285.0481388	43.05	2
T(5): 0.1; S(8): 50.0; S(9): 50.0; T(18		185.2963939	62.38	3
S(2): 100.0; S(7): 0.0; T(8): 0.0		299.7001883	69.98	2
S(1): 50.0; S(4): 50.0; S(15): 0.0; S(1		390.8580816	90.61	2
S(3): 0.0; Y(4): 1.1; S(5): 98.9; S(7): (		272.3205718	21.78	3
T(3): 0.0; S(18): 48.4; S(20): 48.4; S(		226.9218817	22.6	4
S(4): 0.0; S(7): 0.0; T(9): 99.4; S(12):		216.4700695	37.68	3
T(2): 0.0; T(5): 0.0; S(6): 0.0; Y(8): 1.		231.0267023	40.21	2
S(1): 0.0; S(5): 0.0; S(8): 0.0; S(11): 5		233.9434333	59.75	3
S(1): 100.0; S(5): 100.0; T(7): 0.0; T(		326.5316044	64.28	2
T(1): 1.8; S(2): 49.1; S(3): 49.1; S(6):		292.2989157	22.42	3
S(3): 100.0		207.6272394	13.62	3
S(2): 0.0; T(6): 0.0; S(8): 1.7; S(9): 98		231.6066751	71.84	2
S(8): 100.0		201.5690542	16.43	2
S(1): 0.0; S(3): 0.0; S(9): 0.0; T(15): 5		282.7362965	69.94	3
S(7): 0.0; T(10): 0.0; Y(14): 0.0; S(15		191.428438	16.53	3
S(9): 100.0; S(11): 100.0; T(19): 0.0;		184.7684459	12.67	3
S(3): 100.0; T(7): 0.0		193.1466862	14.27	3
T(1): 50.0; S(2): 50.0; Y(4): 0.0; S(9):		127.8596259	13.82	2
S(3): 100.0; S(15): 99.9; S(18): 0.1		344.3374847	40.68	4
S(9): 100.0		223.067813	30.04	2
T(2): 0.0; S(5): 0.1; T(9): 0.0; S(13): 9		122.8446394	1.21	4
T(4): 50.0; S(7): 50.0		219.3775199	41.48	2
T(4): 0.0; T(7): 0.0; S(15): 99.9; Y(20		246.1835295	47.71	3
S(12): 0.0; T(13): 0.0; S(15): 0.0; S(1		192.5808151	34.24	5
S(3): 0.0; S(6): 5.3; S(7): 84.2; S(9): 5		145.1522509	7.09	3
S(1): 0.0; S(5): 0.0; S(6): 0.0; S(15): (		249.2074402	43.01	3
S(9): 100.0; S(11): 100.0		285.8253045	28.46	4
T(2): 0.0; T(12): 0.0; T(18): 100.0		269.622997	54.95	3
S(4): 100.0		242.4246309	29.45	3
Y(4): 100.0; S(9): 0.0; T(10): 0.0		261.149245	37.87	3
S(5): 100.0; T(7): 0.0; T(8): 0.0; T(14		321.2335468	45.36	3
S(2): 50.0; T(3): 50.0; S(7): 100.0; S(		120.6809912	0.11	4
S(4): 0.0; S(5): 100.0; T(9): 0.0; S(11		406.4411791	85.2	2
T(3): 0.0; T(4): 50.0; S(5): 50.0; T(10		276.245956	69.32	2
S(5): 100.0		92.87993885	8.65	3
Y(4): 0.0; S(8): 2.8; S(10): 97.2; S(14		128.7970807	7.56	3
S(3): 98.5; Y(4): 1.5		249.3582079	66.16	2
S(3): 100.0; T(5): 0.0; S(6): 0.0; T(8):		212.8028774	47.01	2
S(8): 100.0		144.4670313	7.33	3
T(1): 0.0; S(2): 0.0; S(5): 0.0; S(6): 0.		176.2401452	29.29	3
S(2): 50.0; S(3): 50.0; S(6): 0.0		147.3991618	18.6	3
S(2): 0.0; T(5): 0.0; T(8): 100.0; S(11		312.2434078	59.34	2
S(3): 100.0; T(8): 0.0; T(16): 0.0; S(1		222.4838097	31.71	4

Information Classification: General

S(5): 0.1; S(7): 99.9; T(9): 0.0	147.8387818	14.47	2
S(3): 100.0	127.1096584	19.63	3
S(3): 100.0	328.2475213	69.94	2
T(4): 0.0; T(8): 3.1; S(10): 96.8; S(12)	262.0052407	24.48	3
S(6): 100.0; S(7): 100.0; T(12): 0.0; S	127.7454102	8.07	3
S(3): 100.0	197.6467073	19.15	2
S(8): 0.0; S(12): 100.0; S(15): 100.0	218.2639332	44.2	3
S(10): 100.0; S(14): 0.0; S(15): 0.0; T	371.3566956	63.64	3
Y(2): 0.0; S(3): 0.0; Y(4): 0.0; T(6): 3.	175.8881625	58.81	3
S(12): 100.0; Y(20): 0.0	128.4901591	17.18	4
S(1): 0.0; T(2): 0.0; S(4): 0.0; T(7): 0.	192.4325507	23.62	4
S(3): 100.0	200.9740951	14.69	2
S(6): 100.0	406.3887552	44.23	2
S(2): 0.0; S(4): 0.0; S(16): 100.0	275.6721043	37.48	2
S(1): 0.0; T(4): 0.0; S(7): 100.0; S(13	487.7809392	52.71	2
S(6): 1.0; S(8): 33.0; S(9): 33.0; S(10	136.6985843	24.47	3
S(5): 100.0	154.4399585	17.26	2
S(2): 100.0	195.7606292	26.85	2
S(6): 100.0; S(16): 0.0	218.1877521	43.71	3
S(1): 0.0; T(2): 0.1; S(3): 99.9; T(7): 0	117.1753494	18.63	2
T(2): 0.2; S(10): 99.8; T(14): 0.0; T(1	144.6976268	36	3
S(4): 100.0; S(7): 99.9; S(8): 0.1	163.238377	17.21	3
T(1): 0.0; S(12): 5.3; S(18): 89.4; S(1	129.2202198	5.45	3
S(4): 0.0; S(6): 100.0; T(8): 0.0; S(9):	289.7990101	60.47	2
T(5): 0.0; Y(9): 3.0; S(10): 97.0	137.4355868	2.87	4
T(1): 3.7; S(2): 96.3; S(4): 0.0; S(5): 0	124.3882642	25.86	2
S(3): 0.1; S(6): 49.0; T(7): 49.0; T(8):	230.6571717	52.44	3
S(3): 100.0; T(6): 0.0	296.9549578	39.71	2
S(1): 0.0; S(12): 100.0; T(13): 0.0	425.5995729	51.24	3
S(6): 0.1; S(8): 96.6; S(12): 3.2	121.7253992	5.29	3
S(9): 1.5; T(10): 97.0; S(11): 1.5	298.3243435	32.97	3
S(2): 0.2; T(5): 90.6; T(7): 0.2; S(10):	153.2062669	46	3
Y(1): 0.0; T(5): 50.0; S(6): 50.0	118.3864472	17.8	2
S(2): 0.0; S(3): 0.0; S(10): 0.0; S(16):	236.1056524	27.15	3
S(1): 0.0; S(4): 0.1; S(5): 99.9	123.195262	9.33	3
S(3): 99.9; S(5): 0.1; Y(11): 0.0	188.2825497	36.07	3
S(2): 100.0; S(6): 0.0; T(8): 0.0; T(9):	299.0377148	49.33	3
T(7): 100.0; S(8): 100.0	223.4598108	29.67	3
S(6): 100.0; S(16): 0.0; T(17): 0.0; S(	287.019111	26.33	3
S(7): 100.0; S(8): 100.0	193.9669338	37.72	2
S(8): 48.7; S(9): 48.7; S(12): 0.1; S(1	165.2730511	33.74	3
S(1): 5.6; S(2): 88.9; S(3): 5.6; T(6): 0	141.0834589	18	4
T(11): 0.1; S(13): 99.9; S(16): 100.0;	375.4290107	97.94	3
S(7): 100.0; T(11): 0.0; S(12): 0.0	457.0991878	94.73	2
S(2): 100.0; S(5): 0.0	138.0106858	24.52	2
S(3): 100.0	129.715777	25.19	2
T(4): 0.0; S(6): 49.3; T(8): 49.3; S(12)	138.7868704	10.08	2
Y(2): 0.0; S(11): 100.0	217.7194945	15.46	3
S(1): 0.0; S(8): 99.8; T(12): 0.2	144.1588435	29.23	3
S(1): 0.0; T(4): 0.0; T(9): 0.0; S(11): 1	207.0328971	35.71	3
S(1): 0.0; S(7): 100.0	440.701263	51.14	2
S(1): 0.0; S(7): 100.0; T(10): 0.0	326.7094058	43.58	2
S(3): 100.0	305.9159118	26.15	2
S(6): 0.0; Y(10): 0.0; S(11): 0.0; S(15	137.8887206	20.79	4
S(4): 100.0; S(9): 0.0	377.8052168	53.31	2

Y(2): 0.0; S(5): 0.0; S(7): 0.1; T(8): 2.	365.3775304	68.72	3
T(13): 0.0; T(14): 100.0	317.993369	46.65	3
S(5): 0.0; S(15): 100.0	324.3788243	61.4	2
S(3): 0.0; S(6): 0.0; S(7): 100.0	202.2139677	47.71	2
Y(8): 0.0; S(10): 100.0	293.2198185	37.04	4
S(3): 100.0; T(5): 96.3; S(6): 3.7	262.4648085	24.81	4
S(4): 99.8; S(10): 0.2; S(15): 0.0	133.667757	12.73	3
S(3): 100.0	95.56872055	14.45	3
T(1): 50.1; S(6): 99.9; S(17): 50.1	51.07125461	1.9	3
T(1): 0.0; S(3): 50.0; T(4): 50.0	423.6667325	79.79	2
T(4): 99.9; S(6): 0.1; T(12): 0.0; S(15)	474.7120844	71.71	3
S(3): 0.0; S(4): 100.0	165.2236026	37.35	2
T(3): 0.0; Y(7): 0.2; S(9): 99.8; S(12):	201.3667863	20.48	3
S(3): 0.0; T(9): 100.0	295.4079563	31.71	2
S(3): 100.0; S(6): 99.2; S(17): 0.4; T(	118.6612533	12.19	3
S(1): 0.0; S(3): 100.0	279.5359811	51.46	2
T(1): 0.0; S(2): 0.0; T(19): 0.0; S(20):	254.8844283	38.66	3
T(1): 0.0; S(5): 0.0; T(8): 0.0; S(11): 0	174.1549673	27.98	2
S(1): 0.0; S(3): 0.0; S(4): 0.0; T(6): 0.	441.9810883	111.05	2
S(3): 100.0; Y(9): 0.0; S(20): 0.0	493.1055306	72.53	3
S(3): 0.0; S(6): 0.0; T(9): 0.0; T(11): 0	202.6944273	57.4	3
Y(1): 0.0; T(3): 100.0; S(5): 0.0; Y(6):	157.2145479	21.91	2
Y(6): 0.0; T(7): 0.0; S(24): 100.0	476.6907046	96.48	3
S(3): 96.4; T(5): 3.6; S(10): 0.0; S(11)	206.7658183	17.22	4
T(1): 0.1; T(3): 2.4; S(5): 97.5	121.4529223	19.59	2
S(11): 100.0	447.9517845	73.07	2
S(7): 98.2; S(9): 1.8; S(10): 0.0	211.7886567	28.84	2
Y(9): 99.9; S(11): 0.1	148.9864453	7.98	3
S(10): 100.0	225.226265	25.76	2
S(1): 3.5; T(3): 96.5; T(7): 0.0; S(11):	168.3071251	6.96	3
S(2): 0.0; S(3): 0.0; S(6): 100.0	206.5848922	49.81	2
S(3): 100.0; S(6): 0.0; S(7): 0.0	183.5342236	15.95	2
T(1): 0.0; T(2): 0.0; Y(3): 0.0; S(7): 10	244.4947114	40.88	2
T(6): 100.0; S(10): 0.0; S(12): 0.0; S(	96.04085068	8.83	4
S(2): 49.3; S(4): 1.3; S(5): 49.3; T(10	175.0668178	28.52	3
S(2): 0.0; S(7): 0.0; S(17): 3.8; S(18):	206.2246816	47.59	3
T(8): 0.0; S(9): 0.0; T(10): 0.1; S(11):	274.6144577	46.62	3
S(1): 0.0; S(3): 0.0; S(6): 100.0; S(12	193.6022585	33.47	3
T(1): 0.0; S(18): 50.0; S(19): 50.0	274.8460085	52.25	3
S(3): 100.0; T(5): 0.0; S(6): 0.0	129.3673795	31.86	3
S(4): 100.0	197.4009071	35.41	3
T(1): 0.0; S(6): 0.0; S(11): 0.0; S(12):	193.3691188	28.27	3
S(4): 100.0; T(7): 0.0; T(11): 0.0; S(2	164.6358688	16.05	3
S(6): 100.0; S(16): 0.0; S(21): 0.0	229.7966089	20.49	3
S(3): 100.0; S(10): 0.0	195.1303407	21.1	4
S(4): 50.0; T(6): 50.0; S(20): 0.0; T(2	127.4965696	2	4
S(6): 100.0	297.1580003	27.28	2
S(1): 100.0; S(4): 0.0; S(7): 100.0; S(	380.8584699	33.91	2
S(11): 100.0	342.0942768	52.89	2
T(2): 0.1; S(6): 49.9; T(7): 49.9; T(17)	179.6293968	31.48	3
Y(2): 100.0; S(9): 0.0; T(12): 0.0; S(1	112.5623654	22.4	6
S(7): 100.0; T(22): 0.0; T(26): 0.0; S(	110.4394836	8.19	5
T(2): 4.5; S(9): 95.5	79.47462418	5.5	3
T(2): 0.0; S(4): 0.0; S(6): 0.1; T(7): 0.	286.2962946	48.81	2
S(1): 0.0; S(9): 100.0; S(14): 50.0; S(	223.7427955	70.78	4

S(3): 0.0; S(5): 100.0		161.4264384	24.68	2
S(18): 50.0; S(19): 50.0; S(22): 4.4; S		226.6623679	9.79	3
S(1): 0.0; S(6): 0.0; S(12): 100.0		235.4208648	44.38	2
T(3): 0.0; S(7): 100.0		350.5982061	47.58	2
S(3): 100.0		227.2121449	24.15	3
S(2): 98.3; S(3): 1.7		252.7567525	38.21	3
S(1): 100.0		175.8786384	9.06	3
S(1): 0.0; S(5): 100.0		156.0455215	13.35	3
S(6): 100.0; S(18): 0.0		158.588196	2.24	4
T(6): 0.0; S(8): 100.0; S(12): 0.0		400.9152007	81.2	2
S(2): 0.0; S(14): 100.0; S(18): 0.0; Y(		312.1833328	66.43	3
S(11): 99.9; S(13): 0.1		222.0976863	8.8	4
S(6): 0.0; T(10): 4.1; S(13): 95.9; S(1		175.7274243	22.14	4
S(3): 0.0; T(4): 0.0; T(11): 33.3; S(12		177.354971	44.33	3
S(6): 0.0; S(12): 14.7; S(18): 92.7; S(		107.7757493	18.66	3
Y(1): 0.0; T(7): 0.0; Y(21): 4.1; S(23):		178.776814	53.15	3
T(6): 0.0; S(11): 0.0; T(15): 100.0		192.8239571	27.34	3
T(1): 0.0; S(2): 0.0; S(4): 0.0; S(9): 10		138.544304	0.39	3
S(1): 2.1; S(5): 97.9; S(9): 100.0; S(1		228.4105836	15.66	3
S(3): 100.0		286.9674737	50.85	2
S(2): 8.8; T(3): 82.5; S(7): 90.6; S(11		74.21739247	12.86	4
S(1): 0.0; S(7): 100.0; S(11): 100.0; T		486.8656284	87.17	2
S(5): 96.5; S(7): 3.5; T(13): 0.0; S(25		248.165396	29.6	3
S(1): 0.1; S(5): 99.9		163.4438813	37.25	3
S(2): 0.0; S(5): 100.0; S(19): 0.0		490.0116976	106.45	3
S(10): 99.2; S(13): 92.2; T(14): 7.3; S		95.58997617	7.33	4
S(7): 93.7; S(10): 93.7; S(11): 11.5; S		155.7761689	23.64	5
S(2): 0.0; S(5): 0.1; S(7): 99.8; T(9): 0		150.2566989	12.42	2
S(3): 100.0; T(5): 0.0; S(7): 0.0		263.1367362	58.08	2
T(7): 100.0		106.9242196	8.23	4
S(9): 50.0; S(10): 50.0		179.7094524	7.38	2
S(2): 0.0; S(6): 100.0		312.9112658	43.17	3
S(3): 0.0; T(6): 0.0; S(8): 0.0; T(13): 9		170.7451861	10.53	3
T(10): 0.0; S(15): 0.1; S(19): 99.9; S(		407.5155911	90.95	3
S(3): 1.0; S(4): 49.5; S(5): 49.5; S(7):		231.6071289	84.4	2
S(3): 100.0		362.690053	26.96	2
S(4): 87.2; T(5): 6.1; S(7): 6.1; Y(19):		85.45057318	7.58	4
S(6): 100.0		363.0389634	50.06	2
T(4): 96.2; S(6): 3.8; S(15): 0.0; T(21		270.4698788	51.95	3
T(3): 100.0; S(7): 100.0		124.2264802	29.01	2
T(1): 0.2; T(3): 99.8; S(6): 51.1; S(7):		121.2338122	26.15	2
S(2): 100.0		211.3593526	18.46	4
S(1): 0.0; S(9): 0.3; T(10): 0.3; S(17):		64.94876353	18.24	4
S(1): 0.0; S(2): 0.0; S(3): 6.2; S(5): 96		122.3645544	18.69	2
T(1): 98.2; T(2): 1.8; S(4): 0.0; T(7): 0		382.9317829	78	3
S(2): 50.0; S(4): 50.0; S(8): 0.0; S(9):		154.2748435	29.97	3
S(9): 100.0		148.0961579	19.05	2
S(2): 3.2; S(3): 96.8; S(7): 33.3; T(9):		194.8159206	12.36	2
T(3): 0.0; T(7): 0.0; S(21): 0.0; T(28):		132.8805577	11.61	6
T(2): 0.0; S(12): 100.0		165.5601411	18.31	3
S(5): 100.0; T(13): 0.0; T(14): 0.0; T(		377.1042381	51.82	3
S(5): 100.0		226.2057501	14.1	4
S(8): 98.4; S(10): 1.6		458.4262648	82.06	2
S(3): 100.0; S(8): 100.0		227.0082238	10.14	2
T(8): 0.0; Y(11): 0.0; Y(19): 0.2; T(21		159.9807422	21.35	4

Information Classification: General

S(1): 4.0; S(2): 88.0; S(3): 4.0; T(9): 4	103.5145582	4.28	3
S(1): 0.0; T(2): 0.0; S(9): 100.0	305.8039596	43.74	3
S(1): 0.0; S(8): 100.0; Y(11): 0.0	453.5079449	66.07	3
S(6): 100.0	155.9504944	20.26	2
S(7): 0.0; T(11): 0.0; S(13): 0.0; S(15	374.9458625	59.37	3
T(9): 0.0; T(10): 0.0; T(19): 0.0; S(23)	176.5760016	17.96	5
T(7): 0.0; S(11): 0.0; T(12): 1.7; S(13)	315.9118325	34.45	3
S(1): 0.0; T(4): 96.7; T(8): 0.1; T(10):	172.3367944	54.73	3
S(2): 0.1; S(4): 99.9	108.2007527	4.16	2
S(4): 100.0; S(8): 0.0	299.9593499	43.01	2
T(5): 0.0; S(6): 0.0; S(8): 0.0; T(10): 0	266.6200461	39.37	5
S(3): 93.0; S(5): 3.5; S(6): 3.5; S(9): 0	151.5865396	29.75	3
T(6): 1.3; T(16): 98.7	86.42814407	9.29	4
S(11): 99.8; T(13): 0.2; S(15): 0.0	189.5754672	18.52	3
S(5): 100.0; S(17): 100.0	106.8598076	15.48	4
S(3): 100.0; S(8): 0.0; S(13): 0.0; T(1	207.3270592	45.45	3
S(4): 100.0; T(10): 0.0	248.4116411	37.47	2
S(15): 50.0; S(16): 50.0; S(18): 0.0	365.9441074	69.25	2
S(4): 0.0; S(8): 100.0	482.4239844	55.6	2
T(3): 0.0; T(12): 100.0; T(18): 0.0; Y(2	266.9043658	28.49	3
S(2): 0.0; T(7): 1.2; S(10): 32.9; S(12	168.134353	21.06	3
T(1): 2.9; S(4): 94.2; S(6): 2.9; T(22):	371.8008922	32.84	4
T(1): 0.0; T(3): 0.0; T(4): 0.0; S(6): 0.0	276.5662982	34.05	3
T(3): 100.0	109.0633303	21.28	2
T(1): 0.0; T(4): 0.0; Y(12): 98.1; S(13)	291.1473106	55.92	3
S(15): 97.0; T(18): 2.9; S(19): 0.1	202.3544352	22.34	3
S(5): 2.7; S(7): 0.1; S(8): 2.7; S(9): 94	127.8292604	24.75	2
T(5): 0.0; S(7): 0.0; S(10): 0.0; S(11):	279.058372	68.46	3
T(14): 87.5; T(15): 87.5; T(20): 44.0;	48.14044685	5.02	4
S(16): 98.0; T(20): 2.0	340.2168372	56.84	3
Y(1): 0.0; S(11): 100.0	285.1221564	45.76	2
T(4): 0.0; S(8): 0.0; S(9): 0.0; S(13): 1	187.9454287	17.98	3
S(2): 100.0; S(3): 0.0	154.0182053	29.49	3
T(6): 0.0; T(12): 100.0	202.089798	42.91	3
S(4): 100.0; Y(8): 0.0	270.0675747	47.17	2
S(6): 0.0; S(11): 100.0	387.8785223	72.62	2
S(12): 0.0; T(15): 0.0; T(18): 0.0; Y(2	221.3232572	44.7	3
S(1): 0.0; T(2): 0.0; S(5): 0.0; T(8): 0.	284.2088797	52.15	3
T(2): 0.0; S(4): 100.0	183.1121343	19.71	3
S(6): 100.0; T(9): 0.0	200.6792756	16.65	3
S(3): 100.0; Y(12): 0.0	187.0278979	22.66	3
S(1): 0.0; S(3): 100.0; S(5): 0.0; Y(7):	156.6114412	18.14	2
S(2): 100.0	185.1929271	19.3	2
S(2): 100.0	67.91428953	18.2	3
S(6): 2.9; T(7): 97.1; S(9): 100.0; S(1	415.3542418	92.61	3
S(7): 100.0	255.2550649	22.81	3
T(1): 99.9; T(3): 0.1; T(7): 0.1; S(8): 0	126.7947759	12.34	2
S(1): 95.7; S(2): 4.1; S(3): 0.2; T(7): 0	97.07999476	6.36	3
T(19): 97.7; S(21): 2.3	409.1104685	69.78	3
S(1): 0.0; S(5): 100.0	108.3536957	5.39	2
S(6): 0.1; S(8): 99.9	175.184049	14.67	3
Y(6): 0.0; S(13): 100.0	378.0527874	69.2	3
S(1): 100.0	223.76183	11.63	2
S(3): 100.0; S(10): 0.0	259.0227583	44.91	2
S(3): 100.0; S(5): 100.0; S(17): 0.0	279.0159787	20.6	4

S(1): 0.0; S(7): 100.0; S(13): 100.0; T	409.1390821	38.56	3
S(2): 0.0; S(3): 100.0	293.2637522	49.85	2
T(3): 100.0	208.1321523	28.28	2
S(8): 100.0; S(12): 100.0	394.4692889	37.76	2
Y(2): 0.0; S(3): 100.0	150.9211606	21.44	3
T(2): 0.0; Y(5): 0.0; S(8): 0.0; S(17): 1	135.0364562	25	3
S(2): 3.0; S(6): 97.0; T(17): 0.0; T(20)	283.1348396	28.83	3
S(1): 0.0; S(6): 100.0	413.9825309	44.83	2
S(4): 49.2; S(5): 49.2; S(6): 1.5; S(12	115.2793497	27.79	3
S(1): 0.0; S(7): 100.0	383.005915	53.72	2
T(5): 0.1; S(11): 99.9	176.2329087	16.9	3
S(7): 3.5; S(8): 85.5; T(10): 3.5; S(11	247.8567036	73.4	3
S(2): 100.0; S(4): 100.0	263.9018434	56.4	2
S(1): 100.0; S(5): 0.0; T(7): 0.0; S(10	205.5800607	63.89	2
S(7): 0.0; T(11): 0.0; S(13): 0.0; S(15	381.6052178	52.81	3
T(1): 0.0; Y(4): 0.0; T(7): 90.2; S(11):	109.5643858	13.61	4
S(1): 100.0; T(4): 0.0	234.8657366	36.08	2
T(1): 0.0; S(9): 100.0	427.9506153	82.23	3
S(3): 2.4; S(5): 97.6; T(7): 0.1; S(15):	205.5800607	28.3	3
S(9): 0.0; S(11): 100.0	266.1924926	65.71	2
S(3): 100.0; S(12): 0.0; T(14): 0.0; T(	315.8566361	40.58	3
T(2): 0.0; S(8): 100.0; Y(25): 0.0	293.208322	81.99	3
S(3): 99.9; S(5): 99.9; S(8): 0.1; S(16	168.6937019	9.63	4
S(2): 0.0; S(3): 0.0; S(7): 100.0; T(9):	290.1825279	44.78	2
T(8): 1.4; T(9): 98.6	237.9183964	18.7	2
S(2): 3.8; S(4): 96.2	61.35282907	20.51	2
T(11): 100.0	133.287338	23.54	2
S(7): 100.0; T(11): 0.0	330.6661461	76.13	2
S(6): 100.0	109.4608124	21.49	3
Y(12): 50.0; S(13): 50.0; T(18): 99.8;	245.112558	20.28	3
T(4): 2.2; T(5): 2.2; S(6): 95.6	172.5978828	35.72	2
S(3): 100.0; S(5): 100.0	197.6704252	21.95	2
T(2): 0.0; S(8): 100.0; T(16): 0.0; T(1	295.4246547	36.01	3
T(2): 0.0; S(5): 100.0	219.7906508	16.86	3
S(2): 0.0; S(3): 0.0; S(10): 100.0	332.1607915	81.06	2
S(6): 100.0; S(11): 100.0	159.8845546	21.09	2
S(1): 0.0; S(3): 98.6; S(4): 1.4; S(7): 0	337.280591	59.81	2
S(3): 100.0	122.4999749	14.58	3
S(1): 0.0; T(8): 100.0; T(10): 0.0; S(1	212.6327847	21.38	3
T(2): 0.0; S(4): 0.0; T(6): 0.0; S(13): 0	218.8446009	38.6	3
T(1): 0.0; S(3): 100.0; S(6): 0.0; Y(8):	248.472602	55.43	2
S(3): 3.5; S(4): 96.5; S(16): 0.0; T(25	237.9109013	45.31	3
S(4): 33.1; T(5): 33.1; S(7): 33.1; S(1	114.0827197	7.73	2
S(7): 49.9; S(8): 49.9; S(11): 0.2; T(1	135.0292785	7.37	3
S(3): 100.0	72.57101779	29.2	2
T(5): 0.0; Y(13): 66.5; S(15): 66.5; S(	137.4050005	25.65	3
T(10): 50.0; S(11): 50.0	223.6775327	24.46	2
S(4): 2.9; T(6): 97.1; S(8): 100.0; T(1	255.8302009	46.97	3
T(4): 0.0; T(8): 3.6; S(10): 96.4; S(12	342.3280699	46.4	3
S(1): 0.0; S(2): 0.0; T(6): 0.0; S(14): 1	424.4782756	90.09	3
S(2): 0.0; S(4): 100.0; T(6): 0.0; S(7):	180.673712	26.63	2
S(6): 100.0; S(12): 100.0	432.8129193	67.69	2
Y(5): 0.0; S(10): 0.0; Y(12): 100.0	247.0914471	60.37	2
S(7): 50.0; S(10): 50.0; S(13): 99.8; T	108.2447596	9.43	3
S(1): 0.0; S(3): 100.0; S(7): 0.0	234.8657366	40.43	2

Information Classification: General

S(3): 100.0		195.0543401	39.09	2
S(3): 100.0		245.7092271	20	2
S(2): 0.0; S(3): 100.0; S(7): 0.0		185.8203201	45.97	2
S(6): 73.4; S(7): 73.4; T(8): 13.3; S(1		138.9519532	27.62	3
T(3): 100.0; S(8): 0.0; S(14): 0.0; S(2		319.0311226	65.11	3
S(3): 100.0		251.9043047	24.52	3
T(4): 99.9; T(6): 24.2; S(7): 24.2; S(8)		60.48310113	5.14	3
T(5): 2.6; S(8): 48.7; Y(9): 48.7		101.7696955	1.55	3
S(1): 95.8; S(6): 4.2; S(9): 0.0; S(10):		247.0706874	55.98	3
S(3): 0.0; S(10): 100.0		417.0271612	53.67	2
T(4): 0.0; S(8): 0.0; S(9): 0.0; S(13): 1		119.4357747	17.37	3
T(3): 0.0; T(5): 0.0; S(18): 100.0; S(2		341.0192176	22.69	3
S(3): 0.0; T(4): 50.0; S(5): 50.0; T(11)		216.8481421	50.64	2
T(2): 50.0; S(3): 50.0; S(9): 0.0; T(10)		477.2552199	67.69	3
S(2): 0.0; S(5): 0.0; S(9): 100.0		348.3247111	56.85	2
S(1): 0.0; S(2): 0.0; S(5): 100.0; S(6):		292.5526404	40.21	2
S(4): 100.0; T(6): 0.0		367.69479	77.69	2
S(4): 100.0		216.65911	20.39	3
Y(3): 0.0; S(9): 100.0		95.01144209	4.79	5
S(4): 100.0; S(7): 100.0		232.0264152	18.57	2
T(1): 0.0; S(8): 100.0		197.0879032	49.14	2
S(3): 91.2; S(4): 91.2; S(6): 8.8; S(7):		342.9101923	73.55	4
S(3): 100.0; S(17): 0.0		257.8300475	45.81	2
S(5): 99.7; T(7): 0.2; T(8): 0.2; T(14):		143.7504771	47.12	2
Y(4): 0.0; S(11): 100.0		214.683901	32.4	2
S(1): 0.0; Y(3): 0.0; S(13): 100.0		145.5830933	18.99	3
S(5): 50.0; S(7): 50.0		86.97382459	12.5	2
T(6): 0.0; T(7): 0.0; S(10): 0.0; S(13):		298.4850342	69.87	3
S(3): 100.0; T(5): 0.0; S(6): 0.0		161.4264384	32.16	2
S(5): 100.0		134.2126018	9.24	2
S(2): 93.2; Y(3): 6.8; S(11): 50.0; T(1		154.4825472	17.44	4
S(1): 100.0; T(4): 0.0; S(5): 0.0		296.2207196	46.28	2
S(7): 100.0; S(10): 100.0		294.3556864	42.2	2
S(2): 94.4; S(3): 2.8; T(4): 2.8; S(6): 1		308.214308	48.73	3
S(3): 0.0; T(5): 1.2; S(10): 19.7; T(11)		88.71776403	12.19	3
S(1): 0.0; T(9): 100.0; S(13): 100.0		250.1959979	51.74	2
S(5): 100.0; S(7): 0.0		199.5935615	13.42	3
S(3): 4.1; S(4): 95.9		53.78945827	16.24	2
S(1): 0.0; S(7): 100.0; S(13): 100.0		388.5702845	71.17	2
T(2): 0.0; S(7): 100.0; S(9): 0.0		343.0199734	77.92	2
S(1): 0.0; T(2): 0.1; S(3): 99.9; S(6): (		178.41687	47.58	2
Y(5): 0.0; S(15): 100.0		466.5016953	102.33	2
S(3): 100.0; S(8): 0.8; T(13): 8.1; S(1		117.3716634	4.5	3
T(1): 0.0; S(2): 0.0; S(4): 0.1; S(10): 9		140.3368818	9.04	3
S(3): 100.0		108.7346798	19.32	3
T(2): 0.0; S(5): 33.3; S(6): 33.3; S(7):		69.5587269	6.44	2
S(10): 100.0; Y(17): 0.0		299.6637137	63.87	2
T(4): 0.0; S(23): 100.0; T(27): 0.0; S(		236.5932385	32.06	4
S(3): 100.0; S(5): 100.0; S(7): 0.0; T(		224.1866606	21.82	3
S(7): 97.2; S(10): 2.8		106.3928316	12.95	3
Y(2): 0.0; T(3): 0.0; S(5): 100.0		174.600537	21.98	2
S(3): 0.0; S(7): 100.0; S(10): 0.0		215.1107673	46.17	2
S(10): 100.0; S(13): 0.0; S(14): 0.0; S		390.9935687	73.36	3
S(5): 0.0; T(15): 0.0; Y(16): 0.0; S(28)		180.1860821	25.25	3
S(3): 100.0; S(5): 0.0		331.1461076	58.31	2

Information Classification: General

T(2): 0.0; Y(3): 0.0; Y(7): 100.0		262.2322648	52.94	2
S(5): 3.2; T(8): 48.4; S(10): 48.4; S(1		123.3446083	16.45	4
S(1): 0.0; S(13): 100.0; S(19): 100.0		276.1697907	13.79	3
S(3): 100.0; Y(10): 0.0; S(11): 0.0		450.6098646	82.28	2
S(4): 2.2; Y(8): 97.8		180.0889454	23.4	2
S(2): 0.0; T(6): 0.1; Y(11): 0.0; S(13):		127.8259026	30.35	3
S(1): 0.0; S(2): 0.0; S(12): 0.0; S(14):		428.5545269	63.15	3
S(5): 50.0; S(6): 50.0		116.8231968	14.33	2
Y(7): 48.8; T(8): 48.8; S(12): 2.2; T(1		158.2784468	15.12	3
S(1): 0.0; S(3): 0.0; S(7): 100.0		187.0450152	6.54	3
S(6): 100.0; S(8): 0.0		348.5273318	46.05	3
T(1): 0.2; S(3): 99.8; S(5): 100.0; S(6		224.5018306	46.73	2
T(12): 100.0		196.8866143	30.2	5
S(5): 100.0		394.2996098	37.09	2
S(3): 100.0		181.2015238	26.68	2
S(4): 0.0; S(8): 100.0; S(11): 0.0; T(1		384.9791654	84.85	2
Y(4): 0.0; S(7): 100.0		288.4312584	39.71	2
T(1): 0.0; S(9): 0.0; S(12): 0.0; S(14):		210.4464924	13.25	4
S(3): 50.0; S(5): 50.0; S(9): 0.0; T(13		283.5468844	53.37	3
T(2): 0.0; S(3): 0.0; T(5): 0.2; S(8): 4.		203.0901605	43.31	3
T(1): 0.0; S(12): 99.8; T(16): 0.2; T(2		172.0496483	19.91	3
Y(11): 2.0; S(13): 93.9; T(14): 2.0; S(		191.1272654	24.54	3
T(4): 0.0; T(10): 0.0; S(14): 0.0; S(18		206.5922992	25.46	3
T(1): 0.2; S(3): 91.8; S(5): 4.0; S(6): 4		207.3156692	54.87	3
S(10): 100.0; S(13): 99.6; T(14): 0.2;		259.6383427	55.54	4
S(4): 100.0; T(8): 0.0; T(11): 0.0		326.6875957	85.43	2
T(2): 0.0; S(7): 100.0; S(9): 0.0		207.018451	25.27	2
S(3): 94.9; S(4): 5.1; S(9): 100.0; S(1		249.8434967	59.18	3
T(1): 0.0; Y(7): 0.0; S(13): 100.0		260.8361767	15.9	3
S(6): 100.0; S(8): 0.0		131.7602581	21.31	2
S(4): 50.0; S(6): 50.0; T(17): 0.0		157.7634409	28.83	3
Y(2): 0.0; S(4): 0.0; T(10): 0.0; T(15):		150.8611656	9.84	3
S(4): 100.0		137.2843697	14.85	2
S(2): 0.0; S(5): 100.0; T(11): 0.0		310.3054226	66.15	2
T(2): 0.0; S(14): 66.7; S(15): 66.7; S(		207.9444147	27.7	3
T(10): 2.8; S(16): 48.5; S(20): 48.5; S		188.4379012	25.86	4
S(1): 0.0; S(3): 100.0; T(9): 0.0		210.4485204	35.33	2
Y(2): 0.0; S(9): 100.0		460.1317264	78	2
S(3): 50.0; S(4): 50.0; Y(9): 0.0		156.3223339	17.59	3
S(1): 100.0; S(7): 100.0		406.4711235	35.78	2
T(3): 0.0; S(5): 0.0; T(7): 0.0; S(14): 0		313.8429479	77.2	3
S(11): 100.0; S(14): 100.0; T(19): 0.0		215.8025811	38.92	4
S(1): 0.0; T(10): 50.0; S(12): 50.0; S(		249.7846828	26.15	3
T(2): 0.1; T(3): 99.9; S(12): 100.0; Y(		198.3959729	18.16	3
S(3): 100.0; T(5): 0.0; S(7): 0.0; S(11		211.5822697	32.04	2
S(3): 100.0; S(9): 0.0; Y(16): 0.0		164.2015749	35.07	3
T(2): 0.0; S(3): 0.0; S(5): 0.0; T(9): 10		284.9117422	16.1	3
S(3): 100.0; S(5): 100.0; S(17): 0.0		166.86183	16.54	3
S(3): 100.0		206.3250965	10.89	2
S(3): 100.0; S(8): 0.0		171.9634582	26.48	3
S(19): 49.9; S(21): 49.9; T(27): 0.2; S		191.3552916	43.2	4
S(2): 50.0; S(3): 50.0; S(6): 0.0; T(17		154.8795257	18.41	3
T(1): 0.0; S(3): 1.9; S(4): 96.2; T(6): 1		240.7645844	53.64	2
S(3): 100.0; T(8): 0.0; S(11): 0.0		190.8091369	31.14	4
S(4): 100.0; Y(8): 0.0; S(11): 0.0; T(1		408.9335135	83.75	2

S(6): 3.4; S(7): 3.4; S(10): 0.1; T(12):	117.7908619	33.59	2
S(1): 97.9; T(5): 51.0; S(7): 51.0	50.47224687	3.2	2
S(7): 98.2; T(8): 1.8	254.8029522	26.01	2
T(2): 0.0; T(3): 0.1; S(6): 11.6; S(8): 9	88.78820943	9.2	4
S(3): 100.0; T(5): 0.0	126.8185909	12.01	3
S(3): 0.0; T(12): 0.0; S(15): 50.0; T(1	213.7699318	29.68	3
S(3): 100.0; S(5): 0.0	311.9896018	28.97	3
S(3): 100.0	104.620719	12.67	3
S(1): 0.0; S(2): 0.0; S(3): 0.0; S(4): 0.	170.8417051	18.22	2
S(1): 0.0; S(7): 100.0; S(13): 100.0	518.2637472	53.45	2
S(4): 100.0	279.5126079	71.43	2
T(6): 0.0; T(8): 1.0; S(11): 49.5; S(12	258.586534	42.69	2
S(1): 0.0; S(2): 100.0; S(6): 0.0; S(8):	340.1688685	97.28	2
S(1): 0.0; Y(3): 0.0; S(13): 100.0	147.5013618	14.24	3
S(3): 0.0; S(6): 0.0; T(9): 0.0; T(11): 0	281.5450366	92.6	3
S(1): 0.0; S(3): 100.0	204.6010468	18.64	2
Y(1): 0.0; T(2): 0.0; S(7): 99.9; S(12):	217.073905	50.93	3
S(12): 50.0; S(13): 50.0	237.1844907	37.34	3
S(6): 100.0	286.9482309	48.79	2
S(8): 100.0	188.0974937	46.61	2
S(1): 100.0; S(3): 100.0; S(5): 0.0; S(	174.1524626	21.37	2
S(1): 100.0; S(7): 100.0; S(13): 100.0	437.7048426	39.44	2
S(3): 100.0; T(5): 0.0; S(7): 0.0	137.3803505	27.86	2
T(2): 0.0; S(5): 50.0; S(7): 50.0; S(13	443.5027216	132.54	2
S(3): 0.0; S(5): 0.0; S(12): 100.0	318.921666	67.07	2
S(3): 100.0; T(5): 0.0	154.3746013	19.46	2
T(2): 0.0; T(4): 0.0; S(6): 1.9; S(7): 98	307.1591505	101.64	2
S(5): 100.0; S(9): 0.0	179.9520389	5.74	4
S(7): 100.0; T(9): 0.0	241.8920289	39.27	2
S(1): 0.0; S(4): 100.0	176.2255845	19.75	2
S(1): 0.0; S(7): 3.3; S(9): 96.7	110.4487842	32.03	3
S(5): 0.0; T(12): 100.0	317.1349502	64.7	2
S(4): 100.0	334.0446411	23.62	4
S(12): 16.3; S(16): 16.3; S(21): 21.0;	97.83034115	30.55	5
T(2): 0.0; S(4): 100.0	192.606935	19.92	3
T(5): 0.5; S(8): 99.7; S(9): 99.7; T(18	174.6236279	45.82	3
S(3): 98.2; S(4): 1.8; S(11): 0.0	212.1151159	38.4	3
S(5): 100.0; T(12): 0.0	165.634229	19.76	3
S(7): 100.0	303.6765436	42.2	2
S(3): 0.0; S(8): 100.0	371.5239445	48.9	2
S(1): 1.6; S(3): 98.4; S(8): 0.0	226.1966573	22.89	3
T(1): 0.0; T(4): 0.0; Y(9): 0.0; S(12): 0	456.90258	82.18	3
S(3): 100.0; S(7): 100.0	251.543775	28.69	3
T(6): 49.9; S(7): 49.9; T(10): 0.1; Y(1	174.8290675	17.91	2
T(3): 100.0; S(5): 100.0	281.4421568	25.77	3
S(2): 100.0; S(4): 100.0; S(7): 0.0	120.832636	21.6	3
T(3): 0.0; S(9): 0.1; S(10): 0.1; S(12):	149.3291756	30.11	3
Y(5): 0.0; S(9): 100.0	237.1432391	22.69	3
S(1): 0.0; S(3): 2.4; S(5): 97.6; S(7): 0	291.8949798	44.98	3
S(8): 100.0	302.5688007	68.73	2
T(1): 0.0; T(5): 0.0; S(10): 99.9; Y(12	316.3816395	59.4	3
S(4): 47.4; T(5): 47.4; T(7): 0.1; S(11	133.3467438	20.97	3
S(1): 0.0; S(7): 100.0; S(13): 100.0	390.575367	39.54	3
S(9): 100.0	198.0771424	29.87	3
T(3): 100.0	163.5931775	15.74	2

Information Classification: General

T(2): 0.1; S(5): 0.8; T(8): 99.1; S(9): 9	121.5283708	19.55	3
S(2): 50.0; S(3): 50.0	139.3355903	10.83	3
S(1): 0.0; T(5): 100.0; S(9): 100.0	190.343372	21.74	2
S(1): 0.0; S(11): 100.0	202.7372281	26.75	3
S(1): 0.0; S(4): 100.0	59.23360438	13.65	2
S(8): 100.0; S(11): 0.0	373.5715715	75.76	2
S(1): 0.0; S(4): 100.0	314.1869498	35.19	3
S(4): 0.0; S(9): 50.0; S(10): 50.0	137.9212928	12.93	3
S(2): 0.0; Y(3): 0.0; S(11): 99.9; T(15	333.2047928	38.13	4
S(1): 100.0; Y(7): 0.0	368.7903306	51.03	2
S(3): 100.0; Y(6): 0.0	427.8183153	52.81	3
S(3): 100.0; S(5): 0.0; T(7): 0.0; T(9):	244.1458731	58.95	2
S(1): 100.0	175.5212166	20.21	3
S(2): 0.0; S(5): 4.5; S(7): 4.5; T(8): 86	111.7970781	35.74	3
T(21): 0.0; S(23): 0.0; S(30): 99.9; T(	411.3912684	68.01	3
S(7): 100.0; S(9): 0.0; S(13): 0.0; S(1	301.6258413	51.27	3
T(3): 0.1; S(5): 2.1; S(6): 2.1; S(7): 2.	221.7841705	55.48	2
T(2): 0.0; S(8): 99.9; T(11): 0.1	183.9967947	31.37	3
S(5): 100.0	137.8241568	10.05	3
S(8): 98.7; T(9): 1.3	246.1791754	50	2
S(13): 99.9; S(19): 0.1; T(23): 33.2; T	131.5610598	7.83	4
T(7): 98.2; S(8): 1.8	135.6442286	14.26	2
S(1): 0.0; S(3): 100.0; T(5): 0.0; S(10	211.9278085	47.56	2
S(6): 100.0	203.0837868	31.35	3
S(7): 0.0; T(10): 0.0; T(11): 0.0; S(18	207.0316211	44	4
S(2): 0.0; S(3): 100.0; T(4): 0.0	280.1003702	45.85	2
Y(4): 0.0; S(6): 0.0; S(12): 100.0	190.3591711	24.4	2
S(1): 0.0; T(10): 93.9; S(14): 11.8; S(	134.9884894	8.76	3
T(1): 0.0; T(4): 50.0; S(6): 50.0; S(9):	126.4165968	8.42	2
S(5): 100.0; T(9): 0.0; T(12): 0.0	126.084354	31.63	3
S(1): 0.0; S(7): 100.0; S(13): 100.0	460.9554523	39.65	2
T(1): 0.0; T(3): 0.0; Y(9): 100.0	343.8132958	75.39	2
S(3): 0.0; S(7): 99.5; S(10): 99.5; S(2	163.1339104	8.84	3
S(1): 3.3; S(3): 93.4; S(4): 3.3; T(6): (	141.6181685	14.14	2
S(2): 0.0; T(5): 0.0; Y(6): 0.0; S(8): 98	245.3757327	47.67	2
S(3): 100.0; S(7): 100.0	93.74058941	4.51	3
S(3): 0.2; S(4): 4.2; S(9): 95.6; T(15):	148.2183481	21.86	4
S(1): 4.3; S(3): 95.7; S(9): 99.8; T(11	163.3416046	2.6	3
S(2): 50.0; T(4): 50.0; Y(18): 0.0	181.1223181	24.33	4
T(4): 0.0; T(8): 0.0; S(11): 100.0	163.7141713	36.46	2
S(3): 97.7; S(5): 2.3; T(8): 0.1	114.9149999	8.04	2
S(2): 0.0; S(5): 100.0; S(9): 0.1; S(12	293.967645	44.97	3
T(8): 97.8; S(10): 2.2	416.6952183	76.66	3
T(1): 100.0; S(5): 0.0; T(11): 0.0; T(1	335.1425048	19.51	3
S(5): 0.0; S(10): 100.0	118.0691914	7.89	3
S(1): 0.0; S(3): 100.0; T(5): 0.0; S(19	412.8023534	52.2	3
T(1): 0.0; S(3): 3.1; S(5): 3.1; T(6): 3.	85.52142345	17.9	2
S(8): 100.0	146.0762659	25.93	2
S(6): 100.0	326.0881004	56.55	2
S(1): 0.0; T(2): 0.0; T(7): 0.0; T(8): 0.	112.9039471	18.09	2
S(5): 50.0; S(6): 50.0; Y(8): 0.0; S(9):	116.0304583	51.75	2
S(6): 100.0	86.06629437	1.88	3
T(2): 0.0; S(5): 100.0; S(10): 0.0	287.4172357	53.28	2
S(6): 50.0; S(7): 50.0; T(12): 0.0; S(1	163.0573432	25.28	3
Y(1): 33.3; S(4): 33.3; S(11): 33.3; S(	99.93765253	12.86	4

Information Classification: General

S(2): 100.0; S(8): 0.0; S(10): 0.0		179.3679794	35	2
S(4): 0.0; T(9): 0.0; S(14): 100.0		284.1979887	27.65	2
S(1): 100.0; T(10): 0.0; T(14): 0.0; S(		299.7363967	47.17	3
S(1): 0.0; S(12): 100.0		447.037622	57.45	2
S(4): 0.0; T(5): 0.0; S(16): 0.0; S(18):		364.3146561	85.5	3
T(1): 0.0; S(4): 100.0		155.4993986	13.66	2
S(2): 0.1; S(3): 99.9; S(7): 100.0; T(9		269.1467036	48.96	2
S(3): 100.0; S(6): 0.0		234.8204683	28.82	3
T(3): 99.9; S(4): 0.0; S(5): 0.1; S(6): (		248.9710609	22.65	3
Y(5): 0.0; T(10): 4.8; S(16): 95.2		138.5062378	25.74	4
S(2): 0.0; S(5): 100.0; S(8): 100.0		221.2981215	22.83	3
S(1): 100.0; T(7): 0.0; S(10): 0.0; T(1(		369.2073022	84.46	3
T(4): 1.8; S(6): 49.1; S(8): 49.1; S(10		244.9310914	44.16	4
S(1): 100.0; S(7): 100.0; S(10): 0.0; S		214.0730299	17.03	3
S(1): 0.0; S(3): 100.0; S(11): 0.0		212.0335918	27.65	2
Y(4): 0.0; T(7): 97.1; S(8): 2.9; T(12):		221.4246541	54.56	3
S(1): 100.0		116.8340089	28.96	2
S(2): 0.0; Y(3): 0.0; S(10): 0.2; Y(11):		190.6802646	21.88	4
S(3): 0.0; S(9): 0.0; S(22): 100.0		142.7352962	14.68	3
S(10): 99.9; S(11): 0.1; T(13): 0.0		303.5628086	45.37	3
S(3): 100.0; T(8): 0.0; T(11): 0.0; T(1		334.9242226	29.2	4
T(2): 0.0; S(4): 0.0; S(11): 0.0; S(16):		386.2862229	66.67	2
S(1): 0.0; S(5): 100.0; S(9): 0.0		168.1338669	14.29	2
S(4): 100.0		155.6086872	17.68	2
S(2): 0.1; S(3): 2.1; S(4): 97.8; Y(6): (		141.1424796	18.06	2
S(1): 0.0; S(3): 0.2; S(4): 3.8; S(5): 95		100.1044757	14.73	3
S(22): 100.0		235.5858384	10.07	5
S(3): 96.3; T(4): 5.5; S(5): 49.1; S(7):		86.66451585	14.9	2
S(6): 100.0		271.0847942	58.93	2
S(16): 100.0		390.8323351	70.67	2
T(10): 0.0; S(18): 100.0		349.5202433	81.6	3
T(13): 100.0; S(15): 100.0; S(17): 10(		192.119298	67.6	3
S(2): 1.9; S(4): 98.1; S(6): 0.0		184.6898314	31.76	2
S(3): 100.0; Y(5): 0.0		261.7139164	23.39	4
S(3): 100.0; S(6): 100.0		156.2330414	17.33	4
S(3): 0.0; S(7): 50.0; S(8): 50.0; T(19		194.9205135	18.41	3
S(1): 0.0; S(4): 33.3; S(5): 33.3; S(6):		95.39249704	13.12	2
T(2): 0.0; T(8): 0.0; T(11): 0.0; S(16):		343.2541921	79.42	3
S(4): 1.5; S(5): 32.8; S(6): 32.8; S(8):		71.76896298	5.82	2
S(1): 100.0; S(4): 0.0; S(15): 0.0; S(1		220.3830753	54.57	3
T(7): 0.0; S(12): 100.0; S(15): 97.5; S		375.5220732	116.99	2
S(2): 0.0; Y(3): 0.0; S(5): 0.0; S(9): 0.		387.9362584	80.21	2
S(4): 3.5; T(5): 93.1; S(6): 3.5; S(15):		233.5073886	39.7	3
S(3): 100.0; S(6): 0.0; T(10): 0.0; T(1		243.7905677	35.29	3
S(5): 1.9; T(6): 98.1		96.67888256	16.41	2
T(13): 0.0; S(15): 100.0		160.9380317	22.75	4
S(2): 0.0; T(5): 97.0; T(6): 3.0; T(8): 3		191.1531339	21.53	2
S(3): 0.0; S(8): 96.8; S(10): 3.2; T(14		245.621042	54.64	3
S(1): 0.0; S(6): 0.0; S(7): 0.0; T(8): 5(		243.2949427	38.5	2
T(7): 100.0		319.9582829	34.23	3
S(7): 100.0; S(11): 0.0; S(13): 0.0; S(		160.2637131	27.52	3
T(2): 1.9; S(3): 98.1; T(7): 0.0		121.2463108	18.63	2
T(11): 50.0; S(13): 50.0		32.7268541	13.49	3
T(2): 0.0; Y(7): 0.0; S(10): 33.3; T(13		224.8175638	56.7	3
S(4): 0.0; S(20): 100.0; S(28): 0.0		172.0475908	32.41	3

Information Classification: General

S(3): 100.0; S(5): 0.0; S(7): 0.0	146.529016	24.58	3
S(16): 0.0; S(21): 100.0; S(25): 0.0; T	427.6948179	50.43	3
Y(3): 0.0; S(8): 100.0	305.0920498	52.26	2
T(3): 0.0; T(9): 0.0; S(13): 0.3; S(15):	119.22183	10.57	4
S(1): 100.0; S(9): 0.0	260.9593456	38.8	2
S(6): 4.7; T(8): 95.3; S(12): 100.0; T(	245.8795476	30.94	4
T(2): 0.0; S(3): 100.0	129.2829205	10.97	3
S(14): 4.7; T(17): 4.7; S(20): 90.7	173.0257255	16.75	4
T(2): 0.0; T(3): 1.8; S(4): 98.2	212.2613411	48.79	2
S(2): 94.8; Y(5): 4.9; S(10): 0.3	119.2293947	7.17	3
S(2): 0.0; T(4): 2.6; S(6): 97.4; S(8): 5	388.0259514	100.7	2
S(6): 100.0; S(9): 0.0	314.3055996	76.24	2
S(2): 0.0; S(6): 97.5; S(7): 2.5; S(14):	270.880991	78.82	2
S(3): 99.9; T(5): 0.1; S(7): 0.0; T(12):	216.2181013	57.33	2
T(3): 98.2; S(4): 1.8	135.210482	5.53	3
S(5): 100.0; T(8): 0.0	216.5776956	36.66	2
T(1): 0.0; T(4): 0.0; Y(9): 0.0; S(12): 0	185.1132586	58.75	4
S(14): 100.0	481.2649213	87.65	2
S(13): 50.0; T(17): 50.0	464.5585357	113.87	2
S(14): 100.0	411.4442978	50.65	3
T(3): 0.0; S(11): 100.0	396.1222759	44.63	3
S(6): 97.5; T(10): 2.5; S(13): 100.0; S	247.848284	43.93	2
S(13): 100.0; S(16): 0.0; S(17): 0.0; S	198.5125581	29.23	3
S(1): 100.0; S(3): 0.0; S(7): 0.0; S(11	272.2494791	89.41	2
S(4): 100.0; Y(7): 0.0	213.67582	12.74	3
S(1): 0.0; S(3): 100.0	246.9839648	34.83	2
T(3): 0.0; T(9): 3.4; S(11): 93.2; T(13)	227.2284497	28.67	3
S(3): 0.0; T(6): 0.2; S(10): 3.9; S(13):	226.1475891	29.99	3
S(1): 0.0; S(6): 0.0; T(11): 7.6; S(14):	233.2175364	30.77	3
T(5): 100.0; S(9): 0.0; T(10): 0.0; S(1	272.1647774	33.56	3
T(3): 50.0; S(4): 50.0; S(7): 0.0; S(8):	186.9212493	28.7	2
S(1): 0.0; S(9): 100.0	144.4670313	14.12	3
S(11): 100.0	206.8524561	59.33	3
S(5): 0.0; T(7): 0.0; S(8): 0.0; Y(14): 0	250.8334611	33.62	3
S(4): 100.0; S(11): 0.0; T(13): 0.0; S(	418.4437779	82.69	3
S(6): 0.0; S(17): 100.0	158.7719514	20.16	3
T(9): 0.0; S(12): 100.0; T(19): 0.0; S(	461.8035267	56.12	3
S(3): 100.0; S(5): 100.0	134.3501448	18.71	4
S(8): 93.8; S(9): 12.1; S(11): 88.4; T(	133.7759507	14.69	3
S(4): 0.0; S(6): 0.0; T(8): 0.0; T(9): 0.	176.0205118	36.61	2
S(4): 0.0; S(8): 100.0; T(10): 0.0; S(1	234.2615839	45.21	3
S(4): 100.0; S(7): 100.0	199.7670094	32.06	3
S(3): 0.0; T(7): 0.0; T(10): 100.0; S(1	244.9740919	50.72	2
S(6): 0.0; T(7): 0.0; S(18): 99.8; T(20)	202.001644	11.57	3
S(1): 0.0; S(10): 100.0	291.2314438	34.31	3
S(7): 100.0; S(11): 0.0	344.2303356	50.97	2
T(1): 100.0	298.4470065	41.9	2
S(4): 0.7; S(6): 99.3; T(8): 100.0; T(9)	123.1932768	6.43	3
S(2): 100.0; T(4): 100.0	98.28047984	13.39	3
S(2): 0.0; S(4): 100.0; S(8): 0.0	156.6534386	28.82	2
T(2): 0.1; S(3): 2.4; S(4): 2.4; S(7): 95	501.6907048	107.92	3
S(7): 100.0	287.9254072	46.4	2
S(6): 3.0; S(7): 97.0; S(10): 100.0; T(	222.1642554	67.48	2
S(3): 100.0	174.2694138	33.29	2
S(1): 0.0; S(3): 0.0; S(7): 0.0; S(13): 1	172.08311	22.96	3

Information Classification: General

S(9): 100.0; T(11): 0.0; S(12): 0.0; Y(	338.5728625	75.12	3
S(14): 2.1; S(15): 97.8; T(17): 0.0	328.9534338	22.63	3
Y(7): 0.0; S(11): 100.0	341.8573054	41.6	2
S(1): 0.0; S(3): 100.0; S(4): 100.0	114.1390212	10.71	2
S(5): 100.0; S(8): 100.0; S(13): 0.0; S	97.58456591	12.54	3
T(1): 0.0; S(4): 100.0; S(8): 0.0; Y(10	332.3004634	44.97	2
T(4): 0.1; S(6): 0.0; S(8): 0.1; S(14): 9	242.2047967	48.69	3
S(1): 0.2; T(3): 0.2; S(4): 0.2; S(5): 0.	182.100754	38.48	2
T(3): 0.0; S(11): 97.1; S(13): 2.8; S(1	161.7340547	40.34	2
T(4): 0.0; T(7): 98.1; S(10): 1.9	213.7371618	65.76	2
S(3): 100.0; S(5): 0.0	221.1808599	28.88	3
S(4): 100.0; T(8): 0.0; S(15): 0.0	202.1821343	36.89	4
S(1): 0.0; T(4): 0.0; T(7): 0.0; T(9): 0.	274.3132621	66.18	2
T(2): 0.0; S(5): 2.0; S(6): 2.0; S(8): 96	191.5841501	14.09	2
S(14): 98.9; S(15): 1.1	405.3873039	55.86	3
S(5): 100.0	264.644107	37.65	2
T(3): 50.0; S(4): 50.0; S(6): 0.0	116.815431	18.09	2
T(3): 0.0; T(5): 0.0; S(12): 3.3; S(13):	204.0397016	20.61	3
T(2): 0.0; S(7): 100.0; T(13): 0.0; S(1	306.1633499	35.89	3
S(6): 100.0; T(12): 0.0	357.661356	74.06	3
S(1): 50.0; S(2): 50.0; T(10): 0.0	182.9531674	33.72	3
S(1): 0.0; S(2): 0.1; S(5): 97.8; T(7): 2	224.6323472	37.44	3
S(8): 100.0; T(15): 0.0	265.6756071	46.16	4
S(2): 0.0; S(4): 100.0; S(7): 0.0	139.5686949	28.07	3
S(1): 7.0; S(3): 86.1; S(7): 7.0; S(12):	118.2364842	5.51	3
S(1): 100.0	138.7505825	15.81	2
S(1): 0.1; S(2): 50.0; S(3): 50.0; S(6):	132.4520387	12.75	2
S(1): 100.0; S(3): 2.7; S(4): 97.3; T(6	227.6941081	57.58	2
T(1): 0.0; S(6): 100.0	222.2361981	29.49	2
S(5): 2.7; S(6): 97.3; S(8): 0.0; T(15):	323.5302568	42.72	3
S(3): 0.0; S(8): 0.0; T(13): 100.0; S(1	205.5122186	37.42	4
S(7): 100.0; S(9): 0.0	286.153528	30.64	2
S(1): 0.0; S(3): 0.0; S(7): 0.0; T(8): 0.	92.3581611	10.02	3
S(4): 100.0; S(6): 100.0; S(12): 100.0	313.0422311	40.95	3
T(1): 98.5; T(2): 1.5; T(4): 0.0; Y(10):	431.9245743	68.26	3
T(1): 0.0; S(3): 0.1; S(4): 90.6; S(5): 3	110.1541337	7.04	2
S(10): 0.0; S(15): 100.0	237.5858339	42.66	4
S(7): 50.0; S(8): 50.0	90.05840054	20.2	2
Y(7): 100.0; T(8): 0.0	185.6546838	30.6	3
T(5): 0.0; S(6): 0.0; S(7): 0.0; S(14): 0	299.1929451	39.62	3
S(6): 100.0; Y(12): 0.0	206.7273689	20.89	3
S(10): 100.0; S(13): 100.0; T(18): 0.0	353.1688539	58.88	3
S(8): 98.8; T(9): 1.2	280.3999301	53.06	2
S(6): 0.0; S(17): 100.0	378.1730187	72.3	3
S(5): 50.0; S(6): 50.0; S(8): 0.0; S(22	265.1086793	66.48	3
S(7): 0.0; S(8): 0.0; S(11): 0.0; S(12):	524.4892325	99	2
T(2): 0.0; S(6): 100.0; T(11): 0.0; S(1	352.9179574	70.17	3
S(1): 0.0; S(2): 100.0	127.0360352	21.29	2
Y(1): 0.0; S(5): 100.0; S(11): 0.0	269.423185	31.13	2
S(7): 100.0; T(9): 0.0; S(14): 0.0; S(1	469.466768	79.76	2
T(1): 0.0; S(11): 0.0; S(16): 100.0	231.8819234	20.02	3
S(3): 100.0; T(9): 0.0	256.8698736	51.24	2
T(8): 0.0; S(14): 100.0	277.499167	62.21	2
T(1): 0.0; S(12): 96.3; T(16): 0.1; S(1	133.1134484	6.8	2
S(4): 100.0; S(7): 99.8; S(8): 0.2	142.4488856	15.62	3

Information Classification: General

S(3): 0.0; T(7): 0.0; S(8): 100.0		239.16058	43.06	2
S(10): 100.0		524.9853566	57.76	3
S(2): 0.0; S(5): 2.5; S(6): 97.5		176.3463317	15.83	2
S(3): 100.0; S(5): 96.4; S(6): 3.6; S(9		206.1439426	31.36	3
Y(1): 0.0; T(3): 0.0; S(14): 48.0; Y(15)		147.5332424	52.1	4
S(1): 0.0; S(3): 0.0; Y(5): 0.0; S(6): 0.		438.3856831	39.99	3
S(2): 0.0; T(6): 100.0; S(8): 0.0		181.4024136	25.28	2
S(2): 0.0; S(9): 100.0		128.5329087	12.86	3
S(4): 0.0; S(5): 0.0; S(6): 0.0; S(10): (		263.0580851	16.4	3
T(1): 0.2; S(10): 99.6; S(20): 90.6; T(		90.36263863	6.85	3
S(4): 100.0; Y(10): 0.0		193.4005536	60.74	2
S(4): 100.0		358.728242	40.06	3
S(2): 0.0; S(8): 0.0; S(11): 100.0		261.1060008	38.96	2
S(1): 0.0; S(7): 100.0		360.7342862	52.15	2
S(12): 0.0; S(19): 100.0; S(25): 50.0;		413.9658124	40.05	3
S(2): 0.0; S(4): 97.1; S(7): 2.9		125.4794319	16.77	3
S(3): 100.0		150.7036786	25.49	2
T(1): 0.0; S(9): 0.0; S(10): 0.0; S(15):		110.8283969	7.73	5
S(6): 100.0; T(12): 0.0; S(15): 0.0; T(		417.7416771	114.55	2
Y(1): 0.0; S(5): 0.0; T(9): 0.0; T(10): (		393.3592322	53.49	4
S(2): 100.0		189.5970531	12.61	3
S(3): 100.0; S(4): 0.1; T(5): 100.0		238.0394696	29.91	2
S(5): 100.0; S(8): 100.0; S(13): 0.0; S		244.6953209	32.47	3
S(1): 100.0; S(13): 0.0		427.9635377	95.1	2
S(1): 0.0; S(6): 4.0; S(7): 45.9; S(18):		69.19401413	0.73	3
T(6): 100.0; S(19): 0.0; S(25): 0.0; Y(		457.6269674	55.87	3
S(18): 100.0; S(24): 0.0; S(25): 0.0		217.6265835	20.38	3
S(4): 0.0; S(10): 100.0; Y(17): 0.0		298.3424815	43.88	3
S(3): 0.0; T(7): 0.0; T(9): 0.0; T(16): (		259.3726964	65.19	3
S(1): 100.0; S(5): 0.0; S(21): 0.0; T(2		189.7797021	25.66	3
S(5): 0.0; T(15): 3.4; Y(16): 3.4; S(28		128.4129396	27.21	4
S(3): 0.0; Y(11): 0.0; S(15): 0.1; S(18		350.12519	65.5	3
S(2): 97.2; Y(3): 2.8		128.3765692	6.49	2
T(1): 0.0; T(12): 99.8; S(14): 0.2; S(1		290.2437364	54.56	4
S(4): 0.0; S(5): 0.0; S(6): 0.1; Y(8): 0.		181.051951	23.49	3
T(1): 0.0; T(10): 98.2; S(13): 50.9; S(		163.1311744	29.23	3
S(1): 100.0; S(10): 100.0		176.0795074	10.28	5
S(3): 99.7; T(4): 94.9; T(7): 5.3; S(13		185.8084337	24.6	3
S(1): 0.0; S(7): 100.0		228.6916008	37.87	2
S(7): 0.0; S(9): 0.0; T(10): 0.0; S(17):		372.1554963	88.96	3
T(2): 0.0; S(3): 0.0; S(9): 100.0; T(28		178.8124242	17.26	3
S(6): 98.9; T(8): 1.1		240.4433706	47.7	2
T(1): 2.6; S(2): 2.6; S(3): 94.8; S(6): (		180.3410782	18.01	2
Y(3): 0.0; S(5): 100.0		321.1078677	35.47	2
S(4): 100.0; S(8): 0.0; T(9): 0.0; T(17		366.0092169	87.85	3
S(6): 100.0		550.5616189	55.87	3
Y(4): 0.0; S(9): 0.5; S(12): 99.5		92.68347646	3.69	3
S(5): 97.3; T(6): 2.7		140.2323583	10	3
S(3): 100.0; T(11): 18.2; S(13): 90.9;		65.98372583	14.49	4
S(4): 0.0; S(6): 100.0; S(8): 0.0; S(10		145.4468432	14.73	3
T(4): 0.0; S(6): 100.0		229.0382538	33.81	2
S(4): 0.0; S(11): 5.3; S(13): 95.0; S(1		161.6175753	3.05	3
S(13): 100.0; T(22): 0.0		188.1598864	67.94	3
S(3): 0.0; S(5): 0.0; S(8): 48.9; S(9): 4		140.8506366	23.88	3
S(3): 100.0		152.9257534	30.41	2

T(2): 0.0; S(5): 0.0; S(6): 0.0; T(12): (	203.8397161	39.09	3
S(2): 0.0; S(5): 0.0; S(6): 100.0	293.0695552	69.44	2
S(1): 100.0; T(7): 0.0; T(9): 0.0	167.7462169	20.53	3
S(1): 0.0; Y(4): 0.0; S(8): 100.0	119.2614247	21.78	2
T(1): 0.0; S(3): 0.0; T(5): 0.0; S(6): 0.	391.3775342	48.14	3
S(3): 0.0; S(7): 98.4; T(9): 1.6	281.4272082	42.26	2
S(3): 100.0; T(6): 0.0	181.1399869	26.09	2
T(4): 8.6; S(6): 1.1; S(7): 8.6; S(9): 73	79.16996458	4.15	3
Y(6): 0.0; S(13): 100.0	151.0481758	28.89	3
S(5): 100.0; S(9): 0.0	284.4525232	52.44	2
S(3): 0.0; S(4): 0.0; T(7): 0.0; S(9): 0.	399.1391212	103.12	3
S(3): 100.0; S(10): 0.0	346.3458701	38.2	3
S(4): 0.0; T(5): 0.0; T(12): 0.1; S(14):	257.9034584	14.13	3
S(3): 48.4; S(5): 48.4; S(9): 3.0; T(15	126.3661786	17.68	3
T(3): 50.0; S(7): 50.0	124.8703145	6.9	4
T(7): 100.0	175.3687026	17.93	2
S(3): 0.0; S(6): 0.0; S(7): 99.9; S(9): (	235.9798229	39.01	2
S(6): 33.3; S(7): 33.3; S(10): 33.3	182.1466334	25.62	2
T(3): 0.0; S(12): 100.0	225.3546766	42.16	3
S(2): 0.0; S(5): 100.0; S(7): 0.0; S(8):	210.0066661	65.67	2
S(2): 0.0; S(5): 0.0; S(12): 95.8; S(14	210.7087558	25.53	3
T(5): 0.0; S(8): 100.0	195.823221	22.59	2
S(7): 0.0; S(19): 100.0; S(20): 100.0	378.6820201	62.41	3
S(5): 100.0; S(8): 0.0; T(9): 0.0	306.5114928	72.87	2
S(3): 100.0; T(9): 0.0; S(14): 0.0	280.9778249	44.17	3
S(1): 1.6; S(3): 98.4	268.5554437	33.4	3
S(3): 100.0	269.7889557	44.34	2
T(2): 0.0; S(5): 0.0; S(6): 50.0; S(8): 5	289.3599774	62.48	2
S(4): 0.0; T(7): 0.0; S(10): 0.4; S(13):	154.9253529	29.19	3
S(7): 50.0; S(10): 50.0; S(14): 0.1; S(	133.824131	26.35	3
S(2): 2.4; S(3): 97.6; T(5): 0.0; S(8): (	133.9296402	22.72	2
S(6): 0.0; T(16): 4.0; S(17): 92.0; T(1	175.7835971	54.36	3
S(1): 0.0; T(2): 0.0; S(5): 0.0; S(10): 3	113.9930048	22.76	2
T(8): 51.1; S(13): 51.1; S(15): 85.5; S	80.26075674	3.98	3
S(3): 0.0; S(6): 98.7; S(7): 1.3	529.3068271	110.15	2
S(2): 0.0; S(4): 0.0; T(6): 0.0; S(8): 10	258.9293619	48	3
S(4): 100.0	50.25165783	7.09	2
S(4): 0.0; S(12): 100.0; T(16): 0.0	388.7712726	92.62	2
T(1): 0.0; S(5): 4.1; S(6): 4.1; S(7): 92	214.4361869	44.61	3
S(1): 0.0; S(3): 4.5; T(6): 97.7; S(7): 9	274.8989827	46.65	2
S(1): 0.0; S(6): 97.0; S(9): 3.0	134.9204685	5.66	2
S(2): 0.0; Y(3): 0.0; S(10): 100.0	212.3382781	43.82	2
S(1): 2.3; S(3): 97.7; S(9): 100.0; T(1	300.8968198	54.46	2
S(8): 0.0; S(11): 0.0; S(16): 0.0; T(18	168.910559	24.56	3
T(1): 0.2; S(5): 4.3; S(6): 95.5; S(14):	195.061127	39.04	3
T(8): 3.3; S(9): 96.7; S(13): 0.0	192.1859069	23.23	4
T(1): 0.0; Y(9): 100.0; S(11): 0.0; S(1	406.388271	68.27	2
S(2): 99.9; S(3): 0.1	185.9787138	7.79	3
S(13): 100.0	250.8108914	17.37	3
S(3): 100.0; S(6): 0.0; S(9): 0.0	255.9737376	61.79	2
S(2): 0.0; S(5): 0.1; S(7): 99.9; S(29):	411.7931313	62.01	3
T(6): 100.0; T(14): 0.0	335.3805555	47.26	3
S(2): 0.0; T(3): 0.0; S(5): 100.0; Y(9):	267.0428569	66.15	2
T(3): 0.1; S(4): 0.0; S(5): 0.0; T(15): (	108.3412063	9.65	3
S(11): 99.9; S(13): 0.1	215.4195904	22.13	3

Information Classification: General

S(4): 0.0; S(8): 50.0; S(11): 50.0; S(1	276.5374143	62.5	2
Y(2): 3.4; S(4): 93.2; S(5): 3.4	133.1675825	11.59	4
T(1): 0.0; S(5): 100.0; T(12): 0.0; T(1	551.5196449	97.45	2
T(4): 1.9; S(7): 98.1	236.4516391	6.47	3
S(4): 100.0; S(11): 0.0	178.645691	15.55	2
S(1): 100.0; S(3): 0.0; T(4): 0.0; T(5):	150.4252065	24.33	2
S(2): 0.0; S(9): 100.0	361.0339092	91.12	2
T(1): 0.0; S(18): 2.3; S(19): 97.7	365.1032535	54.61	3
S(1): 0.0; S(6): 0.1; T(11): 97.4; S(14	309.9346932	48.36	4
Y(1): 100.0; T(4): 0.0	127.1886942	14.76	2
S(4): 100.0	169.3592518	24.78	2
S(2): 0.0; S(6): 0.0; S(13): 3.3; T(15):	181.6909732	42.31	3
T(2): 0.0; S(4): 100.0; T(8): 0.0	162.4089102	15.41	2
S(19): 1.8; S(20): 98.2	452.5398262	38.17	3
S(1): 0.0; S(5): 50.0; T(9): 50.0	88.64230666	8.18	2
T(8): 50.0; S(10): 50.0	179.3251908	23.09	2
S(3): 100.0; Y(5): 0.0	133.9354812	16.62	3
S(1): 0.0; S(7): 98.3; S(9): 1.7	140.2128427	16.85	2
S(1): 0.0; S(5): 0.0; S(9): 100.0	235.8553225	50.16	2
S(1): 0.0; S(4): 98.2; S(6): 67.2; S(7):	127.332423	10.11	3
S(3): 0.0; S(5): 0.0; S(9): 0.0; S(16): 1	484.4248817	116.67	2
T(6): 100.0	115.61033	18.05	2
S(9): 100.0	206.2585796	22.44	2
S(6): 100.0	143.338785	36.66	2
T(4): 0.0; T(7): 100.0	266.3002606	27.87	2
Y(2): 49.2; S(3): 49.2; S(4): 1.5; S(6):	294.6818757	51.19	3
S(1): 0.0; S(6): 0.0; T(7): 0.0; S(14): (	178.2688903	23.51	3
S(1): 0.0; S(5): 0.0; S(6): 0.0; T(9): 0.	354.5108805	49.57	3
S(5): 100.0; T(18): 0.0	351.713981	65.39	3
T(1): 0.1; S(2): 3.2; S(4): 3.2; S(5): 93	137.1677308	6.84	3
S(9): 0.1; S(12): 99.9	209.0862442	35.68	3
T(4): 0.0; S(10): 100.0	171.8399978	24.2	3
T(8): 92.4; S(10): 7.0; T(12): 0.6	78.40524043	5.83	3
T(10): 0.0; S(22): 100.0	212.9037675	25.97	3
S(1): 0.0; S(7): 100.0; S(13): 100.0	355.1113345	24.14	3
S(1): 100.0; S(5): 0.0; T(7): 0.0; T(8):	225.2981182	26.08	2
S(3): 1.1; S(5): 33.0; S(6): 33.0; S(7):	161.8456885	19.3	3
S(3): 99.9; T(5): 0.1; S(11): 0.0; S(13	244.8333386	46.31	3
S(3): 100.0	198.4896227	33.51	2
Y(4): 0.0; S(10): 99.9; S(15): 0.1	175.0464353	2.59	3
T(4): 0.0; S(18): 100.0	437.3910617	88.28	2
Y(1): 0.0; S(5): 100.0; S(7): 0.0; S(12	266.4819805	45.51	2
S(3): 1.4; S(6): 49.3; S(10): 49.3	124.6147137	5.85	2
T(2): 0.0; S(5): 100.0; S(8): 0.0; T(14	392.6698442	57.54	2
S(2): 1.9; S(3): 49.0; S(4): 49.0; Y(6):	114.7381846	8.02	3
S(7): 0.0; T(11): 0.0; S(13): 0.0; S(15	253.1035917	45.08	3
T(7): 0.0; S(12): 100.0	229.2552543	29.64	3
S(2): 0.0; S(26): 50.0; S(29): 50.0	122.9495616	1.13	3
S(1): 0.0; T(2): 0.0; S(10): 100.0	245.780231	26.02	2
S(3): 100.0; S(10): 0.0; T(11): 0.0; S(	430.6812486	56.5	3
S(1): 0.0; S(2): 0.0; S(4): 0.0; S(13): (	457.2241058	81.92	2
S(1): 0.0; Y(3): 0.2; S(4): 99.9; S(5): 1	169.0208878	15.22	2
S(3): 100.0; T(5): 0.0	369.9060941	57.56	3
T(1): 0.0; T(3): 0.0; S(13): 100.0	237.2895735	39.68	3
T(10): 0.0; T(20): 0.0; T(30): 99.5; S(3	263.2617702	38.22	4

Information Classification: General

T(1): 0.0; T(8): 2.1; S(10): 48.9; S(13		184.8008561	33.26	3
S(3): 100.0		224.1400848	25.29	3
S(3): 100.0		245.2889969	34.6	2
S(2): 0.1; S(5): 99.9; T(16): 0.0; S(19		279.8705273	46.83	3
Y(1): 0.1; Y(3): 0.0; S(8): 11.0; S(9): 8		102.1492815	17.56	3
T(5): 0.0; S(7): 0.0; S(10): 5.0; S(11):		145.9969822	21.39	3
Y(2): 0.0; S(3): 100.0		177.9559746	45.91	2
S(6): 100.0		273.8478321	59.7	2
T(1): 0.0; T(4): 3.5; T(7): 96.5		108.9289162	9.7	3
S(3): 100.0; T(11): 0.0		304.7005586	34.94	2
T(1): 0.0; S(2): 0.0; S(4): 0.0; S(9): 10		214.8011179	21.73	2
Y(4): 0.0; T(5): 0.0; S(9): 100.0		236.7941413	28.32	3
T(1): 49.2; T(2): 49.2; S(6): 0.0; T(9):		112.3157447	20.26	2
T(1): 0.0; S(2): 0.0; T(5): 0.0; S(6): 0.		187.1360311	40.99	2
T(1): 0.0; S(6): 2.7; S(7): 97.3; S(15):		302.200355	61.08	3
S(7): 100.0; T(9): 0.0		341.886149	50.95	2
S(4): 33.3; T(5): 33.3; S(7): 33.3; Y(2		214.8920681	45.74	3
T(1): 0.0; S(6): 50.0; T(10): 50.0; T(1		245.778421	48.83	3
T(1): 0.0; T(2): 0.0; S(4): 0.0; T(5): 0.		282.7787823	58.02	2
S(2): 0.0; S(3): 100.0; S(11): 0.0		312.4645847	66.18	2
S(6): 99.9; T(8): 0.1; S(13): 0.0; S(17		205.1694874	25.47	3
T(2): 33.3; S(5): 33.3; S(6): 33.3		74.65166529	10.45	3
S(9): 0.0; Y(14): 0.0; S(15): 0.0; S(19		171.2864245	14.98	3
S(3): 100.0; T(5): 0.0		282.2594177	48.26	2
S(3): 100.0; T(4): 0.0; S(8): 0.0; T(9):		184.4148244	40.1	2
S(3): 100.0; T(5): 0.0		255.6403902	21.01	3
S(7): 100.0		251.3106559	45.55	2
S(4): 0.0; S(5): 0.0; S(6): 0.0; S(10): 0		385.6204261	32.95	3
S(4): 100.0		236.6731877	47.41	2
S(1): 0.0; S(3): 100.0		86.10430999	23.2	2
S(4): 0.2; S(8): 96.1; S(10): 3.7		115.9313226	8.1	3
S(14): 50.0; S(15): 50.0; T(17): 0.0		218.7924969	32.26	3
S(4): 0.0; S(5): 0.0; S(6): 0.0; S(10): 0		301.3360147	22.03	3
Y(2): 0.1; T(3): 93.7; S(4): 3.1; T(8): 3		223.9375817	44.77	3
S(3): 100.0; S(7): 0.0; S(11): 0.0; T(1		290.5046472	26.85	3
S(3): 100.0		101.711365	11.92	4
S(4): 100.0; S(11): 0.0; S(12): 0.0; T(		308.97324	33.6	3
S(3): 100.0; Y(7): 0.0; Y(13): 0.0		137.3883001	12.52	3
T(2): 0.0; S(7): 33.3; T(19): 33.3; T(2		80.44049315	1.96	3
S(7): 0.0; S(9): 100.0; S(16): 0.0; S(1		310.1259321	67.8	3
S(4): 50.0; S(5): 50.0; Y(11): 0.0; T(1		253.1804965	31.46	3
T(2): 0.0; S(8): 0.0; S(9): 100.0		163.7075634	33.09	2
S(17): 50.0; S(20): 50.0		284.3294015	40.89	3
S(1): 95.8; T(3): 4.2; S(6): 0.0; S(9): 0		220.1879397	24.55	3
S(6): 100.0; Y(12): 0.0		322.3988089	48.93	2
T(6): 0.0; S(12): 0.2; S(15): 99.8		183.6029149	16.26	3
Y(2): 0.1; S(5): 90.9; S(7): 2.9; T(8): 2		140.7144568	60.49	2
S(1): 49.2; S(2): 49.2; T(5): 1.7; S(8):		293.2709634	23.4	3
T(4): 98.6; S(6): 1.4		140.805855	29.8	2
T(1): 0.0; S(3): 0.0; T(5): 0.0; S(6): 0.		170.3124146	14.25	4
T(1): 0.0; S(13): 0.0; S(20): 50.0; S(2		283.6221328	36.3	3
S(1): 100.0; T(3): 99.9; S(4): 0.1		155.6416435	3.71	2
S(5): 97.7; T(6): 2.2; T(7): 0.1; S(8): 0		138.5465654	29.1	2
S(1): 91.9; S(7): 91.9; S(13): 3.9; S(1		37.94911082	12.97	3
S(6): 100.0; T(8): 0.0; T(9): 0.0; T(15		132.0388308	9.39	3

S(6): 100.0		267.7611257	42.36	3
T(3): 3.1; S(4): 96.8; T(6): 0.1; T(7): 0		111.5815701	18.53	2
S(1): 0.0; S(2): 0.0; S(3): 0.0; S(5): 0.		224.0111384	50.99	2
S(3): 50.0; T(5): 50.0; S(13): 0.0; S(1		261.0909122	25.66	3
S(2): 99.9; S(4): 0.1; S(9): 0.0; S(10):		184.6694108	2.96	3
S(12): 100.0; S(15): 0.0; S(21): 0.0; S		424.9068023	58.75	3
T(1): 1.9; T(3): 98.1		314.3283368	37.87	3
S(1): 0.3; S(3): 46.4; T(4): 3.4; S(6): 4		109.4401155	13.53	3
T(4): 0.0; T(10): 0.0; S(14): 0.0; S(18		400.1558838	63.27	3
S(1): 0.0; S(3): 49.5; S(5): 49.5; S(7):		100.161113	17.41	2
S(3): 0.0; T(6): 0.0; S(16): 100.0		172.0132938	18.06	2
S(6): 100.0; S(10): 0.0; S(16): 0.0		269.3706752	26.09	3
T(1): 96.7; S(6): 3.3; S(8): 0.0; T(13):		158.1004157	10.95	3
S(1): 0.0; T(3): 0.0; S(4): 100.0		184.1352155	25.87	2
S(5): 0.0; S(7): 100.0; T(9): 0.0		215.3517711	42.84	2
S(3): 100.0; S(5): 0.0		132.2641677	23.48	2
S(2): 0.0; S(9): 98.5; S(10): 1.5		337.955172	29.02	2
S(6): 0.0; S(10): 0.0; S(11): 0.0; S(12		413.966205	94.56	2
S(11): 100.0; S(14): 100.0; S(15): 10(		124.8278417	38.51	3
S(3): 99.9; S(5): 0.1; S(6): 0.0; S(9): (		133.4185137	26.44	2
S(4): 0.0; S(6): 100.0; S(8): 0.0		215.9711526	35.68	2
S(1): 0.0; S(3): 0.0; T(4): 0.0; S(6): 0.		341.5342429	79.89	2
S(3): 66.6; S(4): 66.6; S(6): 66.6; S(8		42.90041125	12.04	2
Y(5): 0.0; S(10): 100.0; S(14): 0.0		261.0847355	33.4	2
T(4): 0.0; S(7): 99.9; S(11): 0.1; S(15		275.8564603	42.81	2
S(6): 0.0; S(14): 0.0; S(15): 0.0; T(19		315.6418264	32.06	3
S(16): 100.0		325.4580159	7.61	4
Y(7): 0.0; Y(10): 0.0; S(13): 100.0; Y(		317.1277634	66.37	3
T(3): 0.0; S(18): 93.7; S(20): 93.7; S(		194.8164928	34.52	4
S(9): 4.6; T(10): 97.7; S(12): 97.7; T(		150.1403521	8.98	5
T(9): 0.0; T(11): 0.0; Y(19): 50.0; S(2(		207.9510112	16.82	3
S(4): 100.0		106.9809983	11.42	3
S(1): 0.1; S(5): 97.7; S(6): 2.2		218.4520348	57.83	2
S(1): 0.0; S(7): 100.0; T(10): 0.0; S(1		312.62433	48.81	2
Y(2): 0.4; S(4): 99.3; S(7): 6.0; S(8): 9		168.4153703	33.13	3
S(7): 100.0; S(11): 0.0		111.0227824	30.73	2
S(12): 100.0; S(21): 0.0		134.7332713	4.66	4
S(3): 100.0		263.8124155	61.5	2
S(1): 0.0; S(6): 100.0		178.7208689	26.69	2
S(1): 0.0; S(7): 100.0		427.1095754	49.28	2
S(1): 0.0; S(7): 98.5; S(10): 1.5; S(13		430.8364755	66.46	2
S(4): 100.0; Y(7): 0.0		206.3604072	34.42	2
S(10): 97.3; S(12): 2.7; T(15): 0.0; T(		271.9626662	40.64	3
T(1): 0.0; Y(3): 0.0; S(10): 0.0; S(13):		435.8213245	94.28	3
S(6): 100.0		207.2871136	13.74	3
S(4): 0.0; S(13): 97.0; S(16): 3.0		147.9760063	6.38	3
T(5): 0.0; S(7): 0.0; S(8): 97.9; S(9): 2		246.3702944	34.84	2
S(3): 100.0		230.2535363	32.22	3
S(4): 4.7; S(5): 95.3; S(9): 0.0; S(11):		200.5548625	56.14	3
S(1): 0.0; S(3): 100.0; T(4): 0.0; T(6):		286.9674737	54.2	2
S(1): 0.0; S(3): 100.0; T(4): 0.0; S(6):		267.9993409	49.06	2
S(3): 100.0; S(5): 100.0; Y(11): 0.0		193.5971117	29.89	2
S(3): 100.0; S(6): 0.0; S(7): 0.0		133.5886242	24.56	3
Y(1): 0.0; S(3): 100.0		259.189626	25.02	2
S(1): 50.0; S(3): 50.0; T(17): 0.0; S(1		103.618242	2.59	4

S(1): 0.0; S(5): 100.0; S(7): 100.0; S(	316.707857	71.41	2
S(1): 0.0; S(17): 4.1; S(19): 95.8; T(2	131.7016214	4.95	5
T(7): 0.0; T(11): 3.0; T(12): 97.0	158.0810983	27.5	3
S(1): 0.0; S(7): 100.0; S(13): 100.0	440.1866302	54.43	2
S(3): 100.0	181.9815279	5.3	3
T(1): 0.1; S(2): 3.4; S(3): 93.1; S(5): 3	276.9208516	39.99	3
S(1): 100.0; S(3): 100.0; S(6): 0.0; T(	198.1754022	37.71	3
S(1): 0.0; S(3): 100.0	309.0562589	78.35	2
S(1): 100.0; Y(2): 0.0; T(6): 0.0	317.5733443	44.71	2
S(5): 0.0; S(7): 0.0; S(10): 0.0; T(12):	207.5932903	25.27	3
S(5): 100.0	261.6189901	29.37	2
S(7): 100.0; T(9): 95.6; S(10): 4.4; S(	225.4034866	43.01	3
S(5): 0.0; T(12): 3.0; S(13): 94.0; T(1	155.4732302	35.15	3
S(2): 3.8; S(9): 47.1; Y(10): 47.1; S(1	121.2104351	7.27	6
S(1): 0.0; T(5): 0.0; S(9): 100.0	333.8508135	46.36	2
S(6): 99.6; S(8): 0.4	122.3131424	8.38	3
S(2): 4.5; S(3): 95.5; T(16): 0.0	106.4744384	7.35	3
T(2): 0.0; Y(3): 0.0; S(4): 0.0; T(7): 0.	398.5397058	82.98	3
S(9): 100.0; S(17): 0.0; S(18): 0.0; S(	337.1220498	67.33	3
S(1): 49.4; S(3): 49.4; T(5): 1.1; T(13)	147.2777629	14.44	3
S(7): 3.1; S(13): 3.1; S(14): 46.9; S(1	72.41555194	13.24	2
S(1): 0.0; S(7): 100.0; S(11): 99.9; T(	329.7425386	40.25	2
S(3): 0.0; S(6): 100.0	364.8061416	59.14	2
T(2): 100.0	202.3243392	30.72	2
T(4): 1.0; S(7): 49.5; S(8): 49.5; T(10)	303.4469853	82.48	2
T(3): 0.1; S(6): 99.9	176.5737229	45.03	2
T(3): 0.1; T(4): 0.1; S(9): 46.7; S(10):	98.69614604	30.93	2
S(1): 2.1; S(3): 97.9; S(6): 0.0; T(19):	341.8503134	60.48	3
S(3): 100.0	244.6034692	33.02	2
Y(1): 0.0; S(4): 0.0; S(5): 0.0; S(12): 9	192.9530052	31.79	4
S(6): 99.8; T(7): 99.8; S(9): 0.4; S(11	241.2812328	48.28	3
T(1): 0.0; S(9): 99.6; S(17): 0.4	118.7543643	6.57	3
T(6): 0.0; S(14): 99.9; S(18): 0.1; S(2	207.7427687	51.94	3
S(1): 0.0; S(7): 0.0; S(8): 0.0; Y(12): 0	225.7175299	80.57	3
S(3): 0.1; S(4): 3.3; S(6): 96.6	324.6843179	68.41	3
S(2): 2.9; S(3): 91.4; S(6): 2.9; T(8): 2	82.53945	12.61	2
S(4): 4.8; S(17): 45.2; S(19): 45.2; S(	77.26978139	2.55	4
S(5): 0.1; S(8): 99.8; S(13): 0.1; S(15	216.8057056	26.53	3
S(5): 100.0; S(7): 0.0; T(10): 0.0	260.2394048	43.16	2
S(9): 0.2; S(10): 99.8	147.178738	13.28	2
T(4): 0.0; S(9): 100.0; S(13): 99.8; T(	240.2338044	30.35	3
S(2): 0.0; T(15): 100.0	395.616643	85.36	3
S(5): 0.0; S(15): 100.0	152.5105359	8.69	3
T(4): 0.0; S(5): 0.0; S(8): 0.0; T(10): 0	253.7430223	58.39	3
S(2): 0.0; T(3): 0.0; S(5): 100.0	211.149737	30.19	2
T(1): 0.0; S(6): 100.0; S(7): 0.0	138.7505825	38.5	2
S(1): 100.0; S(3): 0.0	188.7635723	23.01	2
T(5): 0.0; T(11): 50.0; S(12): 50.0; T(	145.4766912	9.14	4
T(1): 0.0; S(5): 100.0; T(12): 0.0; T(1	409.2880358	51.69	3
T(5): 0.0; Y(11): 0.0; T(14): 0.0; S(16)	246.0227387	24.42	4
S(3): 0.0; T(6): 0.0; S(15): 100.0; T(2	247.1195427	44.47	3
S(2): 0.0; T(3): 0.0; Y(6): 0.0; T(8): 0.	400.1281012	117.8	2
S(1): 0.0; S(4): 0.0; S(6): 0.0; S(15): 3	163.6197827	23.97	3
S(1): 0.0; T(3): 0.0; S(15): 100.0; T(2	335.44929	55.53	3
S(2): 99.9; S(4): 3.0; S(6): 0.2; S(7): 9	111.0380525	28.79	2

Information Classification: General

S(4): 100.0; T(6): 0.0		248.4116411	35.62	2
S(1): 0.0; S(4): 0.0; S(7): 1.9; S(8): 98		218.9184057	43.07	2
S(4): 100.0		217.1408512	11.15	4
S(7): 100.0		211.6977305	15.5	3
T(1): 3.6; S(2): 96.4		125.6111991	7.4	3
S(4): 1.7; S(8): 32.7; T(9): 32.7; S(13		119.5797939	8.85	3
S(4): 4.6; S(16): 95.4		152.0179956	42.81	3
T(4): 0.0; S(9): 98.7; S(10): 1.3		217.5754401	19.85	2
S(1): 0.0; S(7): 100.0; S(13): 100.0		348.6145984	36.93	2
S(6): 0.0; S(10): 2.8; S(11): 97.2		169.8586211	41.22	3
Y(2): 0.0; S(4): 100.0		165.8245409	23.15	2
S(1): 0.0; S(2): 0.0; T(7): 0.0; T(9): 2.		342.1081199	83.62	2
S(8): 100.0		368.3175589	61.09	2
S(7): 49.9; S(10): 49.9; T(12): 0.1; S(		165.0682676	16.14	3
T(4): 0.0; T(8): 99.8; S(10): 0.2; S(12		327.8001365	27.72	3
S(1): 0.0; S(3): 100.0; S(5): 0.0; S(8):		642.1883014	60.87	3
S(4): 100.0		241.7885077	24.25	2
S(1): 0.0; S(8): 100.0		241.8920289	52.79	2
S(1): 0.0; S(3): 0.0; S(5): 0.0; T(6): 0.		354.3358275	88.39	2
Y(3): 0.0; T(14): 0.0; S(17): 0.0; S(18		232.7334272	26.73	3
S(1): 0.0; S(2): 0.0; S(3): 0.0; T(5): 1.		257.4003181	39.74	2
S(1): 0.2; S(3): 95.8; T(5): 4.0; S(19):		224.3939037	24.19	3
S(7): 0.1; S(9): 0.1; S(12): 97.4; S(14		146.2443556	13.23	2
Y(3): 0.0; S(12): 100.0; S(13): 99.9; T		262.0169984	17.69	3
S(3): 100.0; S(6): 0.0; Y(11): 0.0		238.8101937	14.59	3
Y(1): 0.0; S(3): 0.0; S(6): 0.3; S(9): 3.		99.34596411	13.14	3
S(1): 0.0; S(2): 100.0; S(4): 0.0; S(7):		321.8942311	78.83	2
S(6): 100.0; S(10): 0.0		327.6954192	39.96	2
S(1): 99.9; T(3): 0.1; S(4): 0.0		177.1764567	10.13	2
T(1): 0.0; S(10): 0.1; S(11): 2.6; Y(12		141.7067164	12.99	3
S(1): 0.0; T(2): 0.0; T(6): 97.8; S(7): 2		195.1994493	15.44	2
S(7): 100.0		235.013979	39.52	2
S(1): 0.0; S(2): 0.0; S(7): 33.3; Y(10):		158.76954	17.37	3
S(1): 0.0; S(3): 100.0; T(10): 0.0		254.429223	64.76	2
Y(3): 0.0; T(6): 0.0; S(9): 100.0; T(13		328.3757556	62.4	2
S(2): 0.0; S(4): 0.0; S(5): 100.0; S(8):		260.4173138	33.88	2
S(2): 0.0; T(7): 0.0; S(10): 100.0		309.135201	81.36	2
S(3): 100.0; T(5): 0.0; T(10): 0.0; Y(1		503.6606467	97.04	3
T(4): 0.3; S(5): 0.3; S(8): 4.9; T(10): 8		246.6223775	63.99	3
S(4): 97.5; T(7): 2.5; S(17): 0.0; Y(25		260.1364995	39.75	3
S(3): 100.0; T(7): 0.0; S(10): 0.0		185.581812	18.35	2
T(1): 100.0; S(5): 0.0		160.8515502	23.53	3
S(1): 0.0; T(2): 0.0; T(8): 2.2; S(10): 9		254.0710422	52.7	3
S(9): 100.0		107.5567842	3.26	3
S(3): 100.0		135.5469151	18.48	2
S(11): 6.3; S(13): 93.7		188.0965024	28.98	4
T(2): 0.0; S(3): 0.0; S(6): 100.0; T(12		424.371679	93.72	2
T(1): 0.0; Y(2): 0.1; T(4): 99.9; S(8): 0		286.5174096	70.12	3
Y(7): 0.0; S(11): 100.0		242.7644016	46.54	2
T(1): 0.0; S(2): 0.0; S(6): 99.9; T(8): 0		236.1680247	34.43	3
S(8): 100.0		236.6537987	56.36	2
S(7): 100.0		356.8762088	41.8	2
T(10): 0.0; T(20): 0.0; T(30): 100.0; S		263.8068622	30.2	5
S(6): 100.0; T(7): 100.0; S(9): 94.6; S		230.4559652	50.84	3
S(1): 0.0; S(4): 0.0; S(5): 0.0; S(7): 0.		336.5465587	38.76	3

Information Classification: General

S(3): 100.0; S(9): 0.0; S(11): 0.0; T(1		209.1919174	26.54	3
T(10): 0.0; T(17): 99.8; S(25): 0.2; T(		231.6898497	30.92	5
Y(2): 0.0; S(9): 100.0		151.8401349	10.63	2
S(3): 0.0; S(8): 100.0		288.0753904	20.78	2
Y(3): 0.0; S(5): 0.0; S(10): 100.0; S(1		317.6106236	49.63	2
S(4): 97.6; T(6): 2.4; S(9): 0.0		134.2273995	22.87	2
S(10): 90.7; T(11): 4.7; T(12): 4.7; S(		215.8025811	53.78	3
S(2): 0.0; S(4): 0.0; S(7): 50.0; S(8): 5		196.8394905	21.35	3
S(1): 0.0; S(4): 100.0; T(7): 0.0		214.6097819	38.53	2
T(1): 98.2; S(3): 1.8		147.918234	22.02	3
S(4): 0.1; S(17): 99.9; S(19): 99.9; S(		175.0736354	32.3	3
S(2): 3.4; S(5): 96.6; T(11): 0.0		128.9489099	7.82	2
S(4): 100.0		287.8464422	40.34	2
S(3): 100.0		119.2938558	13.99	2
T(1): 0.0; S(22): 100.0		209.3898486	16.01	3
T(3): 1.9; S(5): 98.1; S(6): 0.0		316.1200659	66.11	2
T(1): 1.3; S(2): 98.7; T(7): 0.0; S(14):		273.7880852	66.54	2
Y(5): 0.0; S(8): 0.0; S(12): 100.0; T(1		299.0399785	60.09	3
Y(1): 0.0; S(3): 100.0; T(5): 0.0		240.8481906	51.47	2
S(2): 0.0; S(4): 33.3; S(5): 33.3; Y(6):		129.715777	29.57	2
S(4): 100.0; T(12): 0.0; Y(13): 0.0; S(		255.1466345	55.45	3
S(1): 0.0; S(6): 0.1; S(8): 99.9		421.2410608	63.42	3
S(8): 100.0; S(15): 0.0		102.8856638	19.48	2
S(4): 0.0; S(14): 100.0		548.8845331	132.8	2
T(1): 0.0; S(9): 100.0; S(11): 0.0; S(1		202.5433647	9.84	3
S(2): 2.1; S(6): 97.9		92.46401243	20.73	2
T(2): 0.0; T(5): 0.0; S(6): 1.4; S(8): 98		226.0058416	44.12	2
T(1): 0.0; S(4): 100.0; T(6): 0.0; S(10		157.0186626	11.62	3
S(8): 100.0		82.71155808	0.25	3
T(1): 0.0; T(2): 0.1; S(4): 3.7; T(7): 96		191.022056	11.67	4
T(3): 0.0; T(4): 0.0; S(6): 0.0; S(8): 0.		267.7024926	48.7	3
Y(1): 0.0; S(7): 4.8; S(9): 95.0; S(16):		104.2824078	8.49	2
S(7): 100.0		230.4688911	39.9	2
S(6): 100.0		249.4725646	32.5	3
T(1): 0.0; S(3): 100.0; S(6): 0.0; S(9):		141.9500004	14.66	2
S(8): 96.2; S(9): 7.7; S(11): 96.0; T(1		248.2493017	21.91	3
Y(4): 0.0; S(9): 99.9; S(10): 0.1		238.1578832	19.69	3
S(19): 100.0; S(21): 100.0; S(23): 0.0		394.0298565	58.61	3
T(3): 1.3; T(5): 49.3; S(6): 49.3		194.9902344	27.43	3
S(4): 100.0; S(11): 0.0; S(13): 0.0; S(		327.4808636	48.57	3
S(7): 100.0		188.7625902	31.62	2
T(2): 0.0; S(7): 100.0		100.8617045	8.93	2
S(8): 2.4; S(10): 95.2; S(12): 2.4		236.342007	25.71	3
S(11): 50.0; S(12): 50.0		75.85495347	7.94	3
T(4): 0.0; S(13): 49.0; T(15): 49.0; T(		347.3369695	72.93	4
S(2): 0.0; S(5): 0.0; S(12): 8.0; S(14):		146.9514335	32.39	4
S(2): 0.0; S(4): 100.0; S(9): 0.0; S(16		292.4754009	71.04	2
S(1): 0.0; S(4): 100.0; S(8): 0.0; T(9):		234.9458532	47.34	2
S(1): 100.0; S(6): 0.0		147.8387818	13.51	2
S(4): 100.0		148.6128475	29.03	2
S(2): 0.0; S(9): 100.0		369.513654	66.85	2
S(6): 98.4; S(11): 1.6		279.5111027	57.27	2
S(9): 0.0; S(10): 0.0; S(11): 0.0; S(19		293.8846055	46.47	3
Y(1): 0.0; S(4): 0.0; T(8): 50.0; S(10):		408.2417768	82.74	3
T(5): 100.0		173.8117096	37.18	2

S(2): 32.7; T(3): 32.7; S(5): 32.7; S(7	94.18900721	29.82	2
S(5): 1.8; S(6): 98.2; T(11): 0.0	404.8112083	51.8	3
Y(16): 0.0; S(20): 100.0	184.4203634	7.5	3
Y(4): 0.0; S(10): 100.0	131.266508	4.39	4
S(9): 100.0; S(13): 100.0; S(14): 100.	107.1257009	10.7	3
S(2): 0.4; S(5): 6.1; S(8): 93.5; S(11):	92.70734847	5.82	4
S(2): 0.0; S(8): 100.0; S(10): 0.0	179.7881015	15.18	3
S(1): 50.0; S(3): 50.0; S(7): 100.0	88.7037555	19.52	2
S(1): 0.0; S(3): 100.0	250.1994136	37.3	2
S(1): 0.0; S(5): 100.0; S(8): 0.0	128.5329087	2.1	3
S(3): 100.0	111.4586596	10.58	3
T(6): 0.1; S(10): 96.9; S(12): 3.0	218.9845723	19.64	3
S(1): 98.3; T(3): 1.7; T(8): 0.0	261.1450181	49.39	2
T(4): 1.7; S(6): 98.3	223.4414569	33.27	2
S(2): 0.0; T(4): 0.0; S(7): 100.0	385.6084826	29.26	2
S(2): 0.0; S(5): 100.0	143.8797642	17.38	2
S(1): 100.0; S(7): 100.0; T(10): 0.0	381.1041783	42.48	2
T(8): 100.0	286.8975499	18.52	2
S(1): 100.0	293.5370472	59.34	2
S(1): 0.0; S(5): 100.0	516.3572794	69.04	3
S(1): 97.3; T(3): 2.6; T(6): 0.1	86.67522226	10.72	2
S(1): 100.0; S(7): 100.0	446.4447424	36.65	2
S(2): 0.0; S(8): 0.0; S(10): 100.0	326.6735097	71.06	2
S(3): 0.0; S(8): 50.0; S(9): 50.0	101.8373686	21.25	2
S(1): 0.0; S(3): 100.0; S(5): 100.0	135.514255	17.93	2
S(4): 0.0; S(5): 0.0; S(7): 0.0; S(9): 1.	288.605314	64.96	2
S(2): 0.0; S(6): 1.7; S(8): 98.3; T(12):	214.2260625	29.41	2
T(2): 0.0; Y(7): 0.0; S(10): 0.0; T(13):	247.037273	41.68	3
S(5): 100.0	326.4725712	30.19	3
T(2): 0.0; T(5): 100.0	272.6435372	49.15	2
S(6): 2.0; T(7): 98.0	88.42759982	28.96	2
S(4): 98.3; S(5): 1.7	176.4260886	11.58	2
S(18): 100.0	319.368135	16.48	3
Y(5): 0.0; S(11): 100.0; Y(15): 0.0	316.3762989	67.02	2
T(18): 0.0; S(19): 0.0; T(22): 0.2; T(2	242.3660668	35.47	3
S(6): 100.0	113.9043767	21.75	2
S(1): 100.0; T(4): 100.0; S(10): 0.0; S	347.2701397	57.79	2
T(9): 0.0; T(10): 0.0; T(19): 0.0; S(23)	146.4579756	24.11	3
T(2): 0.0; S(6): 0.1; T(8): 3.0; S(10): 9	187.416743	30.44	3
S(4): 2.4; S(5): 97.6	215.2726585	13.75	3
S(3): 100.0	326.8895527	49.54	3
S(1): 0.0; S(7): 2.0; S(9): 98.0	218.4780729	45.1	3
S(12): 0.0; T(16): 0.0; T(27): 0.1; S(2	242.5614836	11.47	4
S(1): 97.1; S(3): 2.9; S(13): 33.3; S(1	268.7630356	36.52	2
T(9): 0.0; Y(17): 0.0; T(18): 0.0; Y(20)	64.68925627	9.28	5
S(4): 100.0	374.9062215	67.73	3
S(6): 99.9; S(8): 0.1; Y(16): 0.0	278.7371987	61.13	3
T(8): 0.0; S(10): 0.0; S(21): 100.0	442.4665282	95.21	3
S(1): 100.0; S(3): 97.2; T(4): 2.7; S(5	159.03918	39.48	2
S(3): 0.0; S(15): 100.0	237.3473503	94.77	2
S(7): 100.0; S(9): 0.0	357.3999209	92.49	2
T(8): 100.0	228.0880296	52.81	2
Y(1): 0.0; T(4): 0.0; T(5): 0.0; T(6): 0.	377.2074082	88.66	3
S(8): 100.0	232.4173498	45.93	2
T(2): 0.0; T(3): 0.0; S(6): 100.0	204.3471015	43.33	2

Information Classification: General

T(2): 0.0; S(7): 100.0		71.06410486	12.91	3
S(1): 100.0		338.5633986	35.3	2
S(3): 24.8; S(4): 24.8; S(5): 24.8; S(6		105.8120346	3.15	4
S(3): 94.4; T(4): 2.8; S(5): 2.8; S(8): (		201.2688801	33.16	3
S(3): 100.0; S(9): 0.0; T(10): 0.0; S(1		204.4064293	47.57	3
T(1): 0.0; S(5): 97.8; T(7): 2.1; S(9): (		335.2678154	64.45	3
S(1): 0.0; S(5): 100.0; T(7): 0.0; T(8):		302.9567823	64.4	2
S(1): 0.0; S(2): 0.0; T(7): 1.9; T(9): 1.		300.437796	74.12	2
S(10): 0.0; S(11): 0.0; T(14): 95.0; S(		115.3601137	5.75	3
S(9): 2.9; S(12): 94.3; S(14): 2.9		180.331027	27.9	2
S(1): 0.0; S(10): 50.0; S(11): 50.0		123.728901	1.86	3
S(1): 0.0; Y(4): 0.0; S(6): 0.0; S(7): 0.		245.7371639	42.08	2
T(8): 99.9; S(10): 0.1		329.9108103	56.89	3
T(12): 45.0; S(14): 45.0; S(17): 2.5; Y		174.4762198	40.1	3
T(1): 0.0; S(2): 0.0; S(8): 100.0; S(16		313.0394174	40.02	3
S(3): 100.0		235.7420151	28.59	2
S(1): 0.1; S(4): 99.9; S(7): 100.0; Y(1		256.3656439	47.68	3
S(10): 100.0; T(15): 0.0		270.8856346	61.43	3
T(1): 2.4; S(2): 97.6; S(3): 0.0; S(5): (		346.1236206	40.15	2
T(3): 0.0; S(7): 0.0; S(11): 100.0		265.2929955	66.61	2
T(4): 100.0; S(7): 100.0		260.5922585	35.71	2
S(3): 100.0; T(8): 0.0		229.1564028	45.16	2
S(1): 0.0; S(3): 100.0; S(10): 0.0		341.5893587	67.84	2
Y(2): 0.0; Y(3): 0.0; S(4): 0.0; S(5): 0.		330.1573501	43.57	2
S(4): 100.0; S(6): 0.0; S(8): 0.0		102.2742471	28.73	2
S(1): 3.8; S(2): 96.2; T(7): 0.0; T(12):		123.7992396	17.26	3
S(4): 100.0		262.2494762	24.46	2
S(1): 0.0; T(9): 0.0; S(13): 100.0		393.4129077	95.52	2
S(1): 100.0; S(6): 0.0; Y(14): 0.0		261.8668683	36.76	3
S(4): 0.0; S(5): 0.0; S(8): 0.0; S(13): (		284.8455911	57.36	3
S(3): 100.0; S(7): 0.0; S(8): 0.0; Y(10		192.4231891	20.62	2
T(4): 100.0; S(6): 0.0		47.30711458	10.97	3
S(4): 100.0; S(10): 0.0		399.6928406	74.87	2
S(1): 0.0; S(3): 100.0; Y(5): 0.0; S(8):		261.8698468	50.1	2
S(1): 0.0; T(9): 100.0; S(11): 100.0; S		185.107397	35.39	3
S(1): 99.9; S(3): 0.2; S(7): 99.9		144.0621384	30.83	2
S(1): 0.0; S(4): 100.0; T(9): 0.0; T(12)		396.0509652	92.87	2
S(1): 0.0; S(5): 100.0		280.5752557	23.33	2
T(1): 0.0; T(4): 3.7; T(6): 96.3; S(9): 1		135.3503877	21.83	2
T(5): 2.3; S(6): 97.7		102.3737798	12.49	2
S(2): 50.0; S(5): 50.0		95.48142156	7.03	3
S(3): 100.0; S(8): 0.0; S(13): 0.0		140.7727905	29.74	3
Y(2): 0.0; S(5): 2.0; S(7): 48.9; T(8): 4		325.6691815	38.06	3
S(1): 0.0; S(3): 100.0; T(8): 0.0		284.8482919	33.46	2
S(5): 98.9; Y(6): 1.1		254.099089	37.78	2
S(1): 0.0; S(3): 100.0		408.9754301	73.15	2
S(7): 0.0; S(13): 3.0; S(14): 97.0		209.8767483	41.65	3
S(1): 100.0; S(5): 0.0		215.3517711	41.08	2
T(2): 0.0; S(3): 0.0; S(11): 100.0; T(1		208.8272284	44.34	2
S(1): 0.0; S(3): 0.0; S(4): 0.0; T(6): 0.		403.4722923	111.51	2
S(4): 92.2; T(6): 3.9; S(8): 4.1; S(10):		301.848688	70.3	3
S(5): 100.0		212.7648348	37.52	3
Y(1): 0.0; S(3): 0.0; Y(5): 0.0; S(9): 3.		104.4490496	21.82	2
S(3): 100.0; S(6): 100.0		105.9811938	18.88	3
S(1): 0.0; T(5): 0.0; S(10): 0.0; S(14):		306.9016344	19.74	4

Information Classification: General

S(3): 96.3; S(5): 3.7; T(8): 0.0; T(17):	197.7618505	12.98	3
S(8): 94.2; S(11): 2.9; S(12): 2.9; S(1	94.13573121	12.92	2
S(3): 100.0; S(5): 100.0	257.7101407	21.39	3
S(1): 100.0; S(4): 0.0; S(9): 0.0; S(11	312.4645847	60.24	2
S(3): 100.0; S(8): 0.0; T(13): 0.0; S(2	250.5528552	58.17	3
S(1): 100.0; T(3): 99.9; S(4): 0.1	184.1124871	24.65	3
S(3): 100.0; T(8): 0.0; S(9): 0.0; S(13	161.4465846	10.4	3
S(2): 100.0	197.9582222	40.41	2
S(5): 1.9; S(7): 96.1; S(9): 1.9; S(11):	279.7226287	27.78	2
S(1): 0.0; S(3): 100.0	204.5101885	25.62	2
T(4): 0.0; T(7): 0.0; T(8): 1.1; S(9): 48	233.1577536	37.09	2
S(2): 0.0; T(5): 0.0; S(6): 0.0; T(8): 0.	240.4343812	61.69	3
S(3): 100.0; S(4): 0.0; S(7): 0.0	264.6911374	28.78	2
S(5): 1.6; S(7): 32.3; T(8): 32.3; S(9):	190.4302641	40.54	3
S(1): 0.0; S(7): 0.0; S(11): 100.0; T(1	429.8117255	80.64	2
S(2): 99.9; T(4): 0.1; S(11): 0.0; S(12	208.4142717	28.51	3
S(6): 100.0; S(9): 100.0; T(11): 0.0	213.2897825	27.89	2
S(4): 4.3; S(9): 4.3; S(11): 91.3; S(13	107.6934358	12.94	3
T(4): 99.3; S(14): 0.7	63.17142905	12.71	4
S(3): 100.0; S(6): 0.0	142.0471455	10.12	2
S(5): 100.0	316.8600261	35.01	3
S(5): 100.0; T(14): 0.0	358.1953774	49.36	2
S(3): 100.0; Y(13): 0.0	328.5938644	42.53	2
Y(1): 0.0; S(4): 100.0; T(6): 0.0; S(8):	200.7738003	26.45	2
S(1): 2.7; S(2): 2.7; S(3): 94.6; S(7): 0	182.6832236	31.87	2
S(1): 0.0; S(12): 99.9; T(13): 50.1; S(	173.9321298	4.18	4
T(2): 2.5; S(4): 97.5; S(7): 100.0; T(1	242.2601998	17.95	3
Y(6): 0.0; S(11): 3.2; S(15): 96.8	188.15532	13.78	3
S(8): 92.5; S(11): 3.7; S(13): 3.7; S(1	186.3332952	49.18	3
S(1): 0.5; T(3): 99.6; S(5): 100.0; S(7	236.216421	29.77	3
S(2): 100.0; S(6): 0.0; S(10): 0.0; T(1	174.9662206	12.81	3
T(7): 0.0; Y(10): 0.0; S(12): 100.0; S(	365.0659913	89.76	2
S(4): 3.7; T(7): 3.7; T(9): 3.7; T(10): 8	290.2966507	60.18	3
T(3): 0.1; S(7): 96.5; S(8): 3.4; S(16):	190.6013427	15.88	3
S(5): 4.2; S(8): 4.2; S(10): 91.6	170.6537	15.1	3
S(1): 100.0; S(6): 0.0; S(8): 0.0; S(9):	200.3075134	26.26	3
S(1): 0.0; Y(2): 0.0; S(3): 99.9; S(4): 0	348.88908	71.03	2
S(3): 100.0; T(5): 0.0	203.7201837	26.55	2
T(4): 49.1; S(10): 49.1; S(12): 1.7; S(	161.1687062	55.89	3
S(8): 100.0; T(14): 0.0	325.1880391	52.26	2
S(1): 0.0; S(3): 100.0	166.9082898	9.59	2
S(1): 0.0; S(9): 98.3; S(10): 1.7; T(14	379.9222656	93.65	3
S(10): 100.0	425.8438065	46.45	3
T(4): 0.1; S(5): 99.7; T(7): 0.1	130.8728333	18.74	2
T(13): 0.0; S(18): 100.0; S(22): 66.7;	187.871807	28.68	3
T(1): 0.0; S(5): 0.9; T(7): 8.6; S(9): 90	51.15548363	6.98	3
S(3): 100.0; T(8): 0.0; S(10): 0.0; Y(2	259.5632706	20.88	4
S(8): 100.0; T(11): 0.0; T(15): 0.0; S(	385.6865968	42.02	3
S(3): 0.0; S(8): 100.0	232.1256197	47.78	3
S(1): 0.0; S(3): 0.0; S(8): 0.0; S(17): 1	274.6834657	49.17	3
T(1): 0.0; S(2): 100.0; T(4): 0.0	120.8629544	26.24	2
S(1): 100.0; T(6): 0.0; S(7): 0.0	149.3781587	26	3
S(12): 100.0; S(14): 0.0; T(17): 0.0; S	433.1864584	118.01	2
S(7): 100.0; S(10): 0.0; S(14): 0.0; S(	136.003085	20.83	4
T(1): 0.0; T(3): 0.0; S(8): 100.0	250.580174	53.75	2

Information Classification: General

Y(2): 0.0; S(3): 98.5; S(5): 1.5; S(9): (	196.1542284	12.53	2
T(1): 0.0; S(3): 100.0	252.8974458	49.68	2
T(7): 0.0; S(10): 100.0	412.5110984	68.38	2
T(4): 0.0; T(10): 100.0; S(12): 100.0	219.3734355	35.17	3
S(2): 0.0; S(8): 0.0; T(12): 0.0; S(15):	313.6600329	85.66	2
S(8): 96.2; S(9): 3.8; T(12): 0.0; S(13	190.5153215	37.07	2
S(6): 100.0	200.311843	18.53	2
T(1): 0.0; S(3): 0.0; S(6): 99.9; S(9): (	205.4418441	56.31	2
S(1): 0.0; S(3): 100.0; S(9): 100.0; T(	512.7934481	73.59	2
S(6): 8.6; S(9): 95.7; S(11): 95.7	102.1745667	11.74	2
S(4): 0.0; S(9): 100.0; Y(12): 0.0; T(1	254.0604073	20.6	2
S(7): 0.0; Y(9): 0.0; S(10): 0.0; S(17):	141.9018066	15.12	3
T(2): 0.6; S(3): 8.9; S(4): 95.2; S(7): (	300.7191779	74.6	3
S(2): 0.0; T(11): 0.0; S(16): 100.0	269.1094219	53.81	3
S(2): 0.0; S(11): 0.0; S(13): 100.0	162.4949024	13.6	3
S(5): 95.5; Y(7): 2.2; S(8): 2.2; S(14):	379.0979994	68.43	3
S(1): 0.0; S(6): 0.0; S(7): 0.0; S(9): 1(	175.6516091	38.55	2
S(3): 100.0; S(7): 0.3; S(11): 0.3; T(1	223.0870026	27.56	3
S(1): 0.0; S(3): 2.2; S(4): 97.8; T(6): (	243.6798916	69.09	2
S(2): 100.0; S(5): 100.0; Y(7): 100.0	90.72530519	16.61	2
S(8): 0.0; S(14): 2.3; T(15): 2.3; S(18	299.2837078	22.26	3
S(4): 100.0	433.0269044	70.72	2
S(3): 100.0; Y(7): 0.0; T(10): 0.0	181.3386306	23.07	2
S(18): 100.0; T(20): 0.0; S(21): 0.0; T	337.4578524	27.88	3
S(4): 100.0; S(15): 0.0	215.4481698	35.7	3
S(1): 0.0; T(2): 0.0; S(6): 0.0; T(10): (	387.0899119	91.94	3
T(2): 0.0; S(3): 100.0	243.6325377	23.82	2
T(4): 97.9; T(5): 2.1	141.1345384	11.74	2
T(5): 0.0; S(14): 100.0	255.4254713	24.26	2
T(6): 0.0; T(8): 0.0; S(11): 100.0; S(1	138.7632456	5.31	4
T(1): 0.0; S(7): 0.0; Y(10): 0.0; S(12):	233.6731485	33.09	2
S(1): 0.1; S(2): 97.5; S(4): 2.4; S(6): (	201.2451297	51.76	2
Y(1): 0.0; S(5): 0.0; S(11): 50.0; S(13	157.7488323	32.12	2
T(10): 51.7; T(16): 51.7; S(18): 96.5	88.46030969	12.55	5
S(1): 0.0; S(9): 100.0; T(19): 0.0; S(2	457.1410854	82.09	3
T(4): 100.0; S(10): 0.0	185.3443659	29.97	2
S(1): 100.0; T(3): 0.0	80.17898415	14.31	2
S(5): 100.0	211.1312004	30.55	2
S(1): 0.0; T(8): 0.0; S(18): 100.0; S(2	309.8017041	40.08	4
S(1): 0.0; S(5): 2.6; S(8): 97.4; S(16):	154.7638066	24.53	3
T(12): 100.0	368.6717507	97.87	2
T(3): 0.0; S(4): 0.0; S(5): 1.7; S(6): 98	203.2290674	60.3	2
S(3): 50.0; T(5): 50.0; S(13): 0.0; S(1	303.820878	47.77	3
S(1): 0.0; T(2): 0.0; S(10): 100.0; T(1	339.5016381	73.22	2
S(5): 100.0; S(9): 96.8; S(10): 3.2	183.8591872	49.44	2
T(4): 3.8; S(5): 0.2; S(8): 96.0; T(12):	163.1311744	6.9	4
S(1): 0.0; S(3): 100.0	278.4025247	21.11	2
S(1): 7.6; T(13): 92.5; T(15): 99.2; S(	90.57510167	12.38	3
S(1): 1.6; S(6): 49.2; T(7): 49.2	144.1764324	31.85	3
S(1): 0.0; S(3): 100.0	366.6645825	92.43	2
T(2): 0.0; S(6): 0.0; S(8): 0.1; S(18): 4	221.4572183	25.95	3
S(3): 100.0; T(6): 100.0	298.6507513	39.95	3
T(1): 0.0; Y(4): 0.0; S(15): 0.0; T(17):	467.1216251	85.98	2
S(6): 0.0; S(15): 99.7; T(17): 0.3	155.9137461	5.1	3
T(1): 0.0; S(3): 0.0; S(14): 100.0	245.4781145	58.16	3

Information Classification: General

S(4): 50.0; S(5): 50.0		341.2174608	31.64	3
T(1): 0.1; S(5): 50.0; S(7): 50.0		129.9977205	14.18	4
S(6): 100.0		187.2626699	31.64	3
S(2): 0.0; S(5): 0.0; S(7): 100.0; T(11		236.326106	33.29	2
S(5): 100.0; S(7): 95.7; S(13): 4.4		126.6735643	14.72	4
S(1): 0.0; Y(2): 0.0; S(3): 100.0		201.6531891	28.7	2
S(2): 0.1; S(4): 99.9; S(9): 0.0; S(14):		193.2210353	46.26	2
S(4): 100.0; T(12): 0.0		167.9420363	12.98	4
T(3): 0.0; T(5): 0.0; S(18): 2.3; S(22):		231.7819296	17.09	4
T(2): 0.0; Y(5): 0.0; S(6): 0.0; S(9): 10		340.4781669	60.76	2
T(1): 0.0; T(3): 0.0; S(9): 2.9; T(11): 9		201.2688801	38.01	3
S(6): 100.0		378.6209927	33.31	2
T(2): 0.0; Y(4): 0.0; T(6): 0.0; S(11): 0		280.0885087	63.06	4
T(1): 0.0; T(3): 0.0; S(6): 100.0		380.5646017	59.54	2
S(3): 100.0; T(8): 0.0		158.8921483	7.75	2
S(2): 0.0; T(5): 0.0; S(14): 0.0; S(15):		407.2991871	84.14	3
S(2): 3.1; S(3): 96.9; S(10): 0.0; T(14		176.4825042	15.58	2
S(1): 2.7; S(3): 97.3; S(7): 50.0; S(9):		228.5123717	22.48	2
S(1): 0.0; S(4): 100.0		202.2049295	21.97	3
S(18): 97.8; T(20): 2.2		428.6194466	55.54	3
T(2): 0.0; S(4): 0.0; S(9): 2.5; S(11): 9		223.8281735	64.55	3
T(1): 0.0; T(18): 0.0; S(21): 100.0		293.2713116	75.26	3
S(1): 0.0; T(5): 100.0; S(8): 100.0		119.514679	29.58	2
S(6): 0.0; S(7): 0.0; S(9): 0.0; S(10): 0		195.9589973	65.08	3
S(1): 0.0; S(3): 0.0; S(6): 0.0; S(12): 0		130.8904359	30.31	4
S(1): 0.0; S(3): 0.0; T(4): 0.0; T(5): 0.		271.3407062	56.39	3
T(1): 3.8; T(2): 92.5; S(3): 3.8; S(9): 0		217.0183509	27.24	3
S(10): 96.4; S(11): 3.6		135.7603428	5.8	3
S(6): 100.0; T(12): 0.0; Y(16): 0.0; S(		372.974113	55.84	3
S(1): 0.0; S(4): 1.9; S(5): 98.1		169.3884981	16.68	2
S(1): 0.0; S(4): 100.0; S(12): 0.0		266.487788	71.52	2
S(1): 0.0; S(3): 0.0; S(4): 100.0; S(8):		189.83949	40.15	2
T(4): 0.0; S(19): 100.0		403.45661	43.3	3
Y(1): 0.0; S(2): 0.0; T(3): 0.0; S(6): 10		231.6812687	56.99	2
S(1): 0.0; S(3): 100.0; S(5): 100.0		257.5397129	34.67	2
T(1): 0.0; S(3): 100.0		290.2511403	30.59	2
S(9): 100.0; T(11): 0.0; S(14): 0.0; S(		324.3592661	88.46	3
S(1): 0.0; S(7): 100.0; S(13): 100.0		469.8736389	49.18	2
T(4): 0.0; S(10): 100.0		407.6405796	88.46	2
T(4): 1.9; S(5): 98.1		159.7252398	14.25	2
S(1): 0.0; Y(3): 0.0; S(4): 0.0; S(10): 1		201.1578179	26.34	3
S(1): 100.0		267.9993409	34.61	2
S(2): 0.0; S(5): 100.0; S(14): 0.0; S(1		335.6039258	90.28	2
S(3): 100.0; S(5): 0.0; T(10): 100.0		188.099343	11.5	3
S(3): 100.0; T(13): 0.0		249.6973996	57.36	2
S(3): 33.3; T(5): 33.3; S(6): 33.3		155.0568852	25.59	3
S(1): 100.0; S(3): 0.0; S(5): 0.0; Y(6):		269.1122936	82.43	2
S(2): 0.1; S(3): 99.9; T(6): 2.5; S(8): 9		237.5073818	69.71	2
S(3): 100.0; S(6): 90.1; S(7): 0.3; T(8		294.6588488	91.42	3
S(12): 100.0; S(16): 0.0; T(20): 0.0		181.1104385	11.26	3
S(2): 100.0; Y(5): 0.0; T(10): 0.0; T(1		330.415213	56.66	3
S(6): 0.0; S(8): 0.0; T(16): 97.2; Y(18		265.1354435	54.31	3
S(2): 0.0; S(8): 0.0; T(12): 0.0; S(15):		315.0101393	56.38	3
Y(1): 0.0; T(7): 0.1; T(12): 0.1; T(14):		294.5921276	73.37	3
S(4): 100.0; S(11): 0.0		178.3405662	14.23	2

S(2): 97.1; T(3): 2.9; T(5): 0.0; T(9): 0	105.5559508	20.34	3
T(2): 0.0; S(6): 100.0	362.657338	68.47	2
S(4): 100.0; S(11): 0.0; S(14): 0.0; S(	368.4070337	74.64	3
S(7): 0.0; S(9): 0.0; S(10): 0.0; S(11):	287.8986144	49.51	3
S(1): 0.0; S(3): 0.0; S(4): 50.0; S(5): 5	261.7139164	60.91	2
S(4): 0.0; T(8): 0.0; T(12): 0.0; S(20):	262.7662373	53.58	3
T(2): 0.0; T(4): 0.0; S(6): 100.0	166.4011184	31.75	2
S(1): 50.0; S(2): 50.0; S(8): 0.0; T(11	286.4347086	87.29	3
S(5): 0.0; T(10): 0.1; T(14): 2.6; S(15	165.7841892	45.49	3
S(5): 3.1; S(7): 96.9; S(10): 100.0	238.6723438	28.98	3
S(2): 100.0; Y(8): 0.0; S(10): 0.0	257.0294796	33.41	2
S(5): 100.0	197.1422402	19.56	2
S(3): 0.0; T(8): 0.0; T(10): 0.3; S(12):	146.93831	21.65	3
T(1): 0.0; S(3): 100.0; T(8): 0.0; T(11)	204.0811473	47.86	2
S(5): 0.0; S(14): 100.0; T(16): 0.0	270.4413349	30.25	3
S(1): 0.0; S(4): 0.2; S(6): 4.5; S(7): 97	279.4366118	43.91	2
S(8): 99.9; S(13): 0.1	216.7357527	54.62	2
S(2): 0.0; S(4): 0.0; T(7): 0.0; S(9): 10	249.3582079	58.08	2
S(4): 97.9; S(6): 2.1	116.8943286	12.1	2
S(5): 100.0; S(7): 100.0; T(14): 0.0; S	168.7560961	23	3
S(1): 0.0; S(7): 100.0	403.3007931	52.03	2
S(1): 0.0; S(3): 100.0	181.8783958	37.11	2
T(1): 0.0; S(2): 2.0; S(3): 98.0; S(6): 5	339.0961987	62.51	2
S(1): 0.0; S(3): 0.0; S(8): 25.0; S(10):	97.16793344	15.06	3
S(3): 100.0; S(4): 100.0	266.3351456	50.39	3
S(1): 9.6; S(3): 81.4; S(4): 81.4; T(6):	57.45783112	10.94	3
T(2): 0.0; S(6): 100.0	102.3573435	14.59	2
T(8): 49.9; S(9): 49.9; T(10): 4.5; S(1	254.9827529	22.7	3
S(3): 100.0; T(6): 0.0; S(13): 0.0	151.7438976	11.82	3
T(3): 0.0; S(25): 3.6; S(26): 96.4; S(2	345.3766127	65.84	3
S(5): 99.8; T(7): 88.4; S(8): 8.9; S(13	87.28759736	5.69	5
T(1): 2.2; S(3): 95.5; T(6): 2.2; T(9): 0	180.886511	30.62	2
T(2): 0.0; S(9): 100.0	233.487265	46.96	2
S(1): 0.1; S(3): 99.8; S(5): 50.0; S(6):	267.3004572	38.16	3
T(2): 0.0; T(20): 100.0	383.271736	97.45	2
T(9): 0.0; S(11): 100.0	290.9219004	45.51	3
T(1): 0.0; S(2): 0.0; S(3): 99.9; T(4): 0	190.5420795	20.99	2
S(2): 0.1; S(3): 97.6; T(6): 2.3	112.6633411	23.65	2
S(6): 96.9; S(10): 51.4; S(11): 51.4; S	124.5977898	45.74	3
S(2): 0.0; T(6): 0.0; S(9): 100.0	246.1106434	46.81	2
S(1): 0.0; S(4): 100.0	113.5986419	18.12	2
S(7): 100.0; S(16): 0.0	392.2817849	59.24	3
Y(1): 0.0; S(5): 100.0; S(7): 93.7; T(9	152.9218908	10.89	2
T(1): 50.0; S(4): 50.0; S(13): 0.0; S(1	240.180786	27.08	3
S(6): 100.0; S(8): 0.0; S(12): 0.0; S(1	460.7627161	63.23	3
S(3): 3.8; S(5): 96.2; T(10): 0.0; S(12	144.7214086	12.82	3
S(9): 100.0; T(17): 0.0	322.9389128	40.53	3
S(4): 4.3; S(5): 91.3; T(6): 4.3; S(14):	178.4807399	16.36	3
S(1): 0.0; S(4): 100.0; S(5): 100.0; T(	294.7744494	33.42	2
S(9): 100.0; S(12): 0.0; T(13): 0.0; S(	483.7890425	75.84	2
T(1): 0.0; S(4): 0.0; S(7): 0.0; S(13): 0	461.47279	99.82	2
T(2): 0.1; S(6): 3.4; S(7): 3.4; T(8): 93	165.9170153	39.73	3
S(2): 0.0; S(4): 0.1; S(7): 50.0; S(14):	183.4071595	60.14	3
T(4): 3.0; S(6): 97.0; S(9): 50.0; S(11	242.3160272	51.72	2
S(5): 100.0	192.4219977	36.04	2

Information Classification: General

S(3): 100.0	265.509882	20.34	2
T(1): 0.0; T(5): 0.0; S(15): 100.0	247.1069666	25.63	5
S(5): 0.0; S(18): 0.0; S(22): 100.0	281.6040149	23.36	4
S(2): 98.0; T(3): 2.0; T(6): 0.0	206.1759032	41.28	2
S(4): 0.0; T(12): 50.0; S(13): 50.0; Y(	243.0508989	42.24	4
S(4): 100.0; S(10): 0.0; S(14): 0.0; S(	345.2933202	42.04	4
S(3): 50.0; S(5): 50.0	134.2435797	21.02	2
S(14): 0.0; S(17): 50.0; T(18): 50.0	380.1334168	46.92	3
S(1): 0.0; S(3): 100.0; S(9): 0.0; T(11	265.5488474	58.23	2
S(8): 100.0	167.8153878	9.33	3
S(2): 99.9; S(3): 96.3; S(4): 3.8; S(7):	183.198821	38.27	3
Y(1): 0.0; T(3): 0.0; S(5): 100.0; T(15)	340.48243	72.77	2
S(2): 0.0; S(6): 96.1; T(8): 3.7; T(10):	201.0779502	31.5	3
T(4): 100.0; S(9): 0.0	138.5374824	14.62	3
T(4): 91.8; S(5): 4.1; S(7): 4.1; T(22):	210.8765395	65.72	3
T(1): 0.0; T(2): 0.0; S(4): 0.0; S(8): 10	243.7863363	43.65	2
S(1): 0.0; S(4): 100.0; T(5): 2.0; S(6):	277.649888	45.22	2
S(11): 100.0; T(15): 100.0; T(19): 100	44.1383042	1.04	5
T(4): 2.2; T(6): 95.7; S(7): 2.2	209.4140432	64.42	2
S(10): 0.0; T(11): 0.0; S(12): 0.0; T(1	219.2404763	11.27	5
S(3): 1.3; S(4): 32.5; S(7): 1.3; T(8): 3	167.189855	18.15	3
S(1): 0.0; S(10): 100.0; S(12): 0.0; T(	289.3630199	31.31	3
T(1): 0.0; S(3): 100.0; S(4): 0.0	347.1062955	35.21	3
S(6): 99.9; S(7): 99.9; T(14): 0.1; S(1	122.7443085	6.99	3
S(1): 94.0; S(3): 94.0; S(5): 6.0; S(7):	93.48629513	31.46	3
S(4): 100.0; T(8): 0.0; T(12): 0.0; S(1	428.7325357	98.14	2
S(3): 97.4; S(6): 0.8; S(7): 0.8; T(8): 0	105.5383699	10.16	4
S(3): 0.0; S(7): 0.0; S(11): 0.0; T(15):	125.6156181	5.51	4
S(9): 0.0; S(12): 100.0; T(16): 0.0	262.4702468	30.3	2
S(4): 0.6; S(5): 33.1; T(7): 33.1; S(10	286.8031712	67.91	2
S(7): 100.0; S(8): 100.0; S(10): 93.8;	142.7459613	19.74	3
S(1): 100.0	182.6019892	5.89	2
S(9): 0.0; T(11): 0.0; S(13): 1.6; S(14	286.8399936	56.1	4
T(1): 0.0; T(4): 0.3; S(5): 99.6; S(17):	92.0761702	1.45	3
S(2): 0.0; S(3): 0.1; S(5): 96.8; T(6): 3	194.3789017	39.92	2
S(7): 100.0	483.7561021	81.34	2
T(1): 5.4; S(2): 94.6; S(11): 100.0; T(	132.5859263	6.2	3
T(1): 0.0; S(3): 0.0; S(5): 0.0; S(6): 10	276.8341998	61.7	2
S(1): 98.2; S(3): 1.8; S(6): 0.0	147.120613	31.23	2
T(3): 0.0; S(7): 100.0; S(10): 0.0	260.9686498	68.21	2
S(5): 100.0; S(7): 100.0	313.6105654	38.2	3
Y(12): 0.1; S(13): 99.9; T(18): 0.0; T(	262.5311228	27.86	3
S(10): 98.1; S(11): 51.0; S(12): 51.0;	125.8616971	7.44	2
T(11): 50.0; S(14): 50.0	139.8916945	16.66	4
T(16): 100.0	582.3027781	70.48	2
S(7): 100.0	219.5514376	32.65	2
S(18): 100.0	320.7052248	17.28	3
S(1): 0.0; S(3): 100.0; S(4): 100.0; S(	378.7761119	75.95	2
T(8): 0.0; S(15): 100.0	287.1518779	46.25	3
S(2): 0.0; T(5): 0.0; T(7): 0.0; S(11): 1	167.5543059	38.24	3
Y(4): 0.0; Y(5): 0.0; T(9): 100.0	338.9003184	59.12	2
S(1): 99.9; T(2): 0.1; S(3): 0.0; T(5): 0	211.5349223	21.18	3
S(8): 100.0; S(10): 0.0	299.5549406	36.74	3
S(2): 100.0	189.83949	11.92	2
T(1): 0.0; S(4): 0.0; T(5): 0.0; S(9): 10	211.5643337	69.33	2

Information Classification: General

S(2): 2.9; T(3): 2.9; S(5): 94.2; T(8): 0	153.3495765	38.12	3
T(2): 100.0; S(5): 100.0; T(10): 0.0; S	173.4782645	7.74	3
S(2): 97.2; T(4): 2.8	157.9299194	10.31	3
S(4): 99.9; S(6): 0.1; T(9): 0.0; T(16):	194.6150103	43.72	3
Y(10): 100.0; S(13): 0.0	299.8730733	30.17	3
S(4): 97.4; S(5): 2.6; T(8): 100.0	251.9601161	22.53	3
S(3): 3.7; S(4): 92.8; S(5): 96.1; T(7):	86.91756899	14.4	2
S(3): 100.0; S(6): 100.0	187.9958081	29.41	2
S(3): 52.6; S(6): 52.6; S(15): 93.5; S(	48.69700229	1.75	3
S(8): 99.9; S(13): 0.1	110.518021	5.68	2
S(4): 0.0; S(9): 100.0; S(13): 0.0	293.8995341	60.86	2
S(1): 0.0; S(4): 100.0	316.9001947	37.52	2
Y(2): 0.0; Y(9): 0.0; S(11): 100.0	121.8789052	4.6	2
S(1): 0.0; S(4): 100.0	228.4875622	34.2	2
S(1): 3.9; T(2): 3.9; S(3): 3.9; S(7): 88	192.3362958	46.23	3
S(1): 0.0; S(7): 0.0; S(11): 97.8; T(16	338.0978497	60.54	2
S(3): 100.0; S(9): 100.0	314.1074173	43.87	2
S(7): 0.0; T(8): 2.9; S(9): 94.3; S(10):	273.9402794	52.32	3
S(6): 100.0; T(7): 0.0; T(11): 0.0	542.0368998	153.74	2
S(4): 100.0; S(7): 0.0; T(15): 0.0; T(2	249.0984019	46.49	3
S(3): 0.0; S(8): 0.1; S(10): 99.9; T(14	326.7067995	69.93	2
S(4): 0.0; T(6): 0.0; S(10): 100.0	374.0421682	40.99	2
S(3): 100.0; S(9): 0.0; Y(16): 0.0	169.7265902	21.1	3
S(4): 3.4; S(7): 96.6; S(10): 0.0	160.2882291	26.91	3
Y(1): 0.0; S(11): 100.0	203.6129194	33.65	3
S(2): 0.0; S(6): 100.0; S(8): 0.0; S(10	339.6204259	22.18	3
S(5): 100.0	252.9936889	26.08	2
S(1): 0.1; S(2): 2.8; S(3): 97.2	127.3412034	4.47	2
S(5): 100.0; S(12): 0.0; T(15): 0.0	333.4569378	56.25	3
S(2): 0.0; S(6): 100.0; S(9): 0.0	208.8748225	42.22	2
S(4): 97.0; S(5): 3.0; T(11): 0.0; T(15	186.9440969	18.4	2
Y(1): 0.0; T(3): 0.0; S(6): 100.0; T(9):	177.0069247	21.17	2
T(7): 51.5; Y(8): 51.5; T(11): 96.9; S(	54.6763814	12.59	3
S(8): 100.0; S(12): 100.0; S(15): 0.0	462.6872445	51.19	3
Y(3): 0.0; T(5): 0.0; S(10): 93.1; S(12	191.5661017	57.64	3
S(3): 98.7; Y(4): 1.3	414.9737716	85.52	2
S(1): 0.0; S(2): 2.4; S(4): 95.3; S(5): 2	271.0169595	37.15	2
S(3): 100.0; T(7): 0.0	204.5101885	31.91	2
T(3): 0.0; S(9): 100.0; S(20): 0.0	354.4261178	74.29	3
T(1): 0.0; S(2): 0.0; S(4): 0.0; T(5): 0.	177.9218953	19.5	2
S(7): 0.0; S(12): 0.0; S(15): 99.9; S(1	344.2877311	45.53	3
S(1): 100.0; S(5): 0.0; S(7): 0.0	221.7781688	32.71	2
T(2): 50.0; S(3): 50.0; S(6): 0.0; S(7):	136.0117801	13.97	3
S(3): 100.0; T(4): 0.0; T(8): 0.0; T(13	360.6305852	87.73	2
S(2): 0.0; S(3): 0.0; S(9): 100.0; S(11	166.2522147	19.15	3
T(3): 95.4; T(4): 4.6; S(6): 0.0; S(8): 0	146.3127121	28.11	3
S(2): 0.2; S(4): 98.3; S(6): 50.8; S(8):	103.2129574	21.74	2
Y(3): 0.0; T(11): 0.0; T(12): 0.0; T(13	309.918321	42.52	3
S(8): 0.0; S(18): 50.0; S(19): 50.0	278.7478895	42.55	3
T(4): 0.1; S(7): 99.9; T(13): 0.0; Y(22	396.963482	83.9	3
T(9): 1.7; S(11): 0.0; S(13): 98.2	345.1697232	46.63	2
S(3): 100.0	380.9363774	53.36	2
S(1): 96.5; S(2): 51.7; S(7): 51.7	153.3089731	22.84	4
T(5): 100.0	230.9348596	30.73	2
S(1): 0.0; S(2): 0.0; T(3): 0.0; S(5): 10	310.8827193	45.76	2

S(9): 0.0; S(16): 0.4; T(20): 0.4; S(25	130.8627972	7.03	4
S(8): 100.0; S(10): 100.0; S(15): 0.0	137.0464447	19.94	3
S(1): 100.0	145.8935471	19.03	2
S(1): 0.0; S(3): 0.0; S(4): 100.0; S(11	249.6973996	66.57	2
S(7): 100.0	138.800047	5.57	3
S(4): 0.0; T(9): 0.0; S(14): 0.0; S(18):	182.239339	45.22	3
T(5): 0.0; S(6): 0.0; T(8): 0.0; S(16): 1	129.8711202	2.35	3
T(1): 0.0; S(5): 100.0	254.4496162	31.08	2
S(10): 0.0; T(15): 0.0; T(16): 0.0; S(2	267.1676652	58.59	3
T(4): 0.0; S(7): 0.0; S(8): 0.0; S(16): 1	356.8505061	58.67	4
T(1): 2.2; S(2): 97.8	91.23787838	16.57	2
S(3): 100.0	293.2637522	34.87	3
S(1): 100.0; S(5): 0.0; S(7): 0.0; S(9):	167.6477106	22.67	3
S(2): 0.3; S(6): 99.7; S(8): 100.0; S(1	251.158112	26.1	3
S(5): 95.0; S(6): 5.2; S(11): 4.7; S(12	86.30531076	14.05	2
T(4): 0.0; T(8): 97.3; S(10): 2.7; S(12	397.9719671	41.52	3
S(1): 0.0; S(2): 0.0; S(3): 0.0; T(5): 50	182.4970479	29.3	2
S(3): 100.0; S(10): 0.0	409.2156784	39.37	4
S(2): 100.0; T(8): 0.0; S(13): 0.0	282.5207998	53.45	2
S(1): 0.0; S(3): 100.0	189.0161883	17.73	2
Y(6): 100.0	145.7924814	31.93	2
T(7): 49.3; S(8): 49.3; S(9): 1.3; S(13	187.0748537	36.77	2
S(11): 50.0; Y(12): 50.0	278.0807872	42.03	3
S(3): 100.0; T(5): 0.0; T(7): 0.0	257.4876922	57.02	2
S(2): 0.0; S(13): 100.0	431.8811577	51.83	3
T(4): 0.0; S(10): 100.0; S(14): 100.0	226.9015666	29.3	3
S(3): 100.0	162.9362016	15.59	2
S(6): 0.0; S(8): 0.0; S(10): 100.0	217.6501011	43.79	3
T(1): 0.0; S(6): 0.0; T(11): 0.0; T(18):	195.4766462	17.31	4
S(4): 2.7; T(6): 97.3; S(8): 100.0; T(1	306.9276424	35.09	3
S(1): 0.0; S(3): 100.0	237.9510925	41.76	2
Y(3): 0.0; T(11): 0.0; T(12): 0.1; T(13	257.5099563	37.2	3
S(5): 100.0	439.5064575	59.92	2
S(1): 96.7; S(3): 3.3; T(5): 0.0; T(7): 0	198.6992547	22.27	3
S(6): 0.0; S(7): 100.0	375.8330635	76.71	2
Y(5): 0.0; S(7): 100.0; T(10): 0.0; S(1	260.6449682	23.32	2
T(1): 0.0; S(5): 100.0; T(14): 0.0	488.4244948	108.41	2
S(2): 0.0; S(13): 50.0; T(15): 50.0	431.725867	51.92	3
S(11): 100.0	411.3779621	67.37	2
S(7): 100.0; S(10): 100.0	467.944351	94.62	2
S(1): 0.0; T(2): 0.0; T(7): 0.0; T(8): 0.	187.089971	35	3
S(4): 0.0; S(13): 100.0	406.3636312	65.82	2
S(13): 2.3; S(14): 97.7	255.2152582	39.96	3
T(2): 0.0; S(8): 100.0	171.0956199	24.73	2
S(14): 50.0; S(15): 50.0	582.8887462	50.4	2
S(5): 100.0; S(12): 0.0	278.4408234	61.97	2
S(2): 0.0; Y(3): 0.0; T(5): 0.0; Y(11): 9	283.1965841	56.71	2
T(6): 0.0; S(8): 0.0; S(15): 2.2; S(16):	199.7300683	52.58	3
S(2): 0.0; S(4): 100.0; Y(7): 0.0	191.5271275	28.56	2
T(5): 0.0; S(10): 100.0	212.4012681	32.65	3
S(17): 4.5; S(18): 95.5	159.5392947	6.04	3
T(2): 0.0; S(5): 2.1; S(8): 97.9; S(11):	363.4675886	82.46	3
T(7): 50.0; S(12): 50.0; S(15): 0.0; S(	279.5529471	71.08	3
S(2): 0.0; T(8): 94.3; S(9): 2.8; S(11):	132.1520347	13.17	3
S(2): 2.8; S(5): 97.2; S(8): 0.0	95.24334334	16.69	2

Information Classification: General

S(7): 100.0	130.1244381	5.2	3
S(2): 0.0; S(6): 100.0; S(8): 0.0	250.0662056	36	2
S(7): 49.8; S(8): 49.8; S(10): 48.1; T(	96.14623415	3.86	3
S(3): 100.0	601.7766324	81.79	3
S(7): 100.0; S(13): 0.0	357.126238	90.82	2
S(5): 50.0; S(6): 50.0	201.3934482	32.08	3
T(4): 100.0; S(7): 100.0; S(11): 0.0; S	136.8517656	24.54	2
S(3): 100.0; T(6): 0.0	220.3105861	25.46	3
S(1): 1.2; S(2): 49.4; S(3): 49.4; T(7):	219.8917818	63.77	2
S(3): 100.0	269.915046	39.11	2
T(1): 0.0; S(12): 0.1; S(18): 99.9; S(1	123.2587218	5.44	3
S(9): 2.6; S(10): 94.8; T(12): 2.6; T(1	187.1452722	44.11	3
T(10): 0.0; S(15): 100.0; S(19): 95.3;	268.3006558	59.79	3
S(3): 100.0; S(5): 100.0	150.6992419	23.87	4
Y(4): 0.0; S(8): 100.0; T(13): 0.0; T(1	343.8768971	60.97	3
T(3): 100.0; S(4): 0.0; S(5): 0.0; S(6):	300.8646716	46.03	3
T(3): 0.0; T(4): 0.0; S(6): 50.0; T(9): 5	153.3211222	32.45	2
T(2): 0.0; S(4): 100.0; S(7): 0.0	254.8392875	31.9	3
S(1): 0.4; T(3): 0.4; S(4): 5.9; S(6): 93	58.41899527	2.88	3
S(1): 4.3; S(2): 95.7; T(6): 0.0; S(13):	163.6792902	21.17	2
S(11): 50.0; S(12): 50.0; T(20): 0.0	222.1558593	19.62	3
S(8): 100.0; S(10): 100.0	427.5579778	85.61	2
S(2): 0.0; S(3): 100.0; S(8): 0.0; T(9):	321.0238441	84.51	2
S(2): 0.0; S(5): 100.0; T(11): 0.0; T(1	306.5400519	79.6	3
S(5): 100.0; S(7): 0.0	218.1107684	14.01	2
S(6): 100.0	226.10809	21.44	3
S(1): 0.0; S(12): 100.0	477.2546952	48.94	2
S(9): 0.2; S(10): 99.8; T(12): 0.0	164.3069998	30.48	3
S(6): 0.0; Y(7): 0.0; S(18): 100.0	124.3134076	8.8	4
S(13): 100.0; S(14): 100.0; T(17): 100	320.8706114	116.35	3
S(3): 100.0	261.1450181	36.66	2
S(1): 96.9; T(4): 3.1; T(7): 0.0; T(11):	205.2838753	36.17	2
S(1): 100.0; T(4): 0.0; S(10): 0.0; S(1	386.6554409	79.43	2
S(4): 100.0; S(8): 100.0	286.0344117	26.35	2
T(2): 0.0; S(8): 100.0; S(13): 0.0	277.8547498	47.55	3
S(2): 0.0; S(12): 0.0; T(16): 4.1; S(17	225.9271697	25.36	4
S(5): 100.0; S(7): 100.0	141.1445246	13.05	2
S(8): 50.0; S(10): 50.0; S(15): 0.0	125.3321372	9.01	3
S(3): 0.0; S(12): 95.9; T(14): 4.1; S(1	231.9872099	37.98	3
S(3): 5.3; S(4): 94.4; S(5): 0.3	107.478095	12.51	3
T(1): 0.0; T(4): 0.0; T(5): 99.9; S(7): 0	169.1615549	30.66	2
S(2): 0.0; S(3): 0.0; S(4): 0.0; T(5): 0.	139.4574577	29.83	3
T(2): 0.0; S(7): 99.9; S(10): 0.1; S(15	189.3741747	17.94	3
S(4): 100.0	223.067813	18.08	2
T(2): 50.0; S(3): 50.0; S(5): 0.0; S(7):	117.187292	42.97	3
T(3): 100.0; S(10): 0.0; S(14): 0.0	162.7662234	21.34	3
S(6): 100.0	202.5393401	35.64	2
S(1): 0.0; T(3): 50.0; S(4): 50.0	115.61033	38.45	2
S(2): 0.0; S(6): 100.0; S(11): 0.0	305.9172464	58.22	2
S(10): 0.0; S(11): 0.0; T(16): 0.0; S(1	383.9321572	89.38	3
T(1): 2.2; T(3): 97.7; S(4): 0.1; S(10):	280.4287882	29.92	2
S(4): 0.0; T(10): 0.2; S(12): 99.8; S(1	329.0032073	89.68	3
S(1): 50.0; S(3): 50.0; S(7): 0.0; S(12	330.2466781	39.69	3
T(6): 0.0; S(9): 0.0; T(16): 3.6; S(18):	147.2972759	35.96	3
S(5): 100.0; S(8): 100.0; S(13): 0.0; S	383.5576019	104.35	2

S(11): 100.0; S(14): 0.0; T(19): 0.0; T	218.3282164	32.43	3
S(3): 0.0; Y(5): 0.0; S(6): 0.0; S(11): 1	180.8141666	13.97	3
S(7): 0.2; S(12): 99.8; S(15): 0.0	204.2072345	10.93	3
S(3): 0.0; S(4): 2.1; S(7): 97.9; T(11):	130.8448751	28.68	2
S(3): 100.0; T(6): 0.0; Y(26): 0.0; S(2	259.9086666	52.82	3
T(5): 100.0; S(9): 0.0	202.7536518	12.4	3
S(1): 0.0; T(2): 0.0; S(9): 100.0	362.446124	56	2
S(3): 99.9; S(5): 0.1; T(10): 0.0	185.0931149	11.76	3
S(5): 0.0; S(10): 100.0; S(24): 0.0	386.6156505	58.79	3
S(2): 0.0; S(8): 0.0; Y(14): 0.0; S(20):	281.947268	46.02	3
T(1): 0.0; Y(9): 0.0; S(11): 100.0; S(1	370.3145435	55.02	2
S(2): 100.0; S(5): 99.9; S(7): 0.1; S(8	159.9863051	20.74	2
S(3): 100.0; T(24): 0.0	213.0949154	40.61	3
S(4): 0.0; S(6): 100.0; S(8): 0.0; S(10	690.7505114	169.6	2
S(2): 0.0; S(3): 0.0; S(8): 2.3; S(9): 97	125.5148041	27.48	2
S(3): 3.3; S(4): 93.7; T(5): 6.3; S(6): 9	162.0945022	27.95	2
T(1): 0.0; S(8): 100.0; T(11): 0.0	226.4574368	54.27	2
T(3): 0.0; T(9): 0.0; S(11): 0.0; T(13):	283.1129773	74.77	3
T(1): 0.0; S(4): 0.0; S(8): 99.7; S(13):	177.6522691	15.71	4
S(1): 98.4; S(3): 50.8; S(5): 50.8; S(7	255.4404755	36.97	3
T(11): 100.0	284.2500846	43.32	4
T(10): 100.0	222.3815087	45.22	3
S(11): 0.0; Y(14): 100.0	148.2278566	26.44	3
S(2): 0.0; S(4): 100.0; Y(5): 0.0	218.7394729	41.27	2
S(6): 0.0; S(7): 0.0; S(9): 100.0; S(10	236.7841228	14.87	3
S(6): 100.0; Y(21): 0.0; S(22): 0.0; S(	110.3743823	3	3
T(2): 2.7; T(5): 97.3; S(7): 0.0	106.5785725	9	2
S(4): 0.0; S(6): 0.0; S(8): 100.0	314.5251568	35.5	2
S(7): 5.3; S(8): 94.7; S(10): 100.0; T(	225.0660097	37.31	3
S(10): 100.0	296.2337567	43.68	2
S(1): 0.0; S(2): 0.1; S(4): 99.9; S(13):	467.585543	65.15	3
S(2): 100.0; S(4): 0.0; T(7): 0.0; T(14	288.1108224	52.49	2
S(9): 100.0	441.0988739	90.05	2
S(9): 100.0	411.1751015	75.9	2
S(9): 100.0; S(20): 0.0	302.3024547	58.83	3
T(4): 50.0; S(5): 50.0; T(9): 0.0	192.6002596	30.16	2
S(4): 100.0	309.2628664	35.52	2
S(1): 100.0; S(7): 100.0	430.8172513	33.07	2
S(6): 50.0; S(8): 50.0; S(14): 0.0	135.6203495	30.77	3
S(1): 0.0; S(12): 100.0	199.8412439	41.52	3
S(2): 0.0; S(4): 100.0; T(7): 0.0; T(13	275.2474117	58.14	2
S(2): 100.0	244.0488843	33	2
S(8): 100.0; S(11): 0.0	276.9667145	40.84	2
S(3): 100.0	318.9254528	31.65	2
T(2): 96.1; S(4): 0.0; S(7): 3.9; S(13):	194.3753933	36.92	3
S(2): 100.0	142.9823941	25.96	2
S(1): 0.0; S(2): 0.0; S(3): 100.0; S(5):	237.5883102	33.48	2
S(6): 0.0; T(14): 4.3; S(16): 4.3; Y(17	183.6252417	3.06	3
S(3): 100.0	235.7633543	37.03	2
Y(1): 0.0; S(15): 33.1; S(16): 33.1; T(	323.1974133	17.49	5
S(12): 2.9; S(13): 2.9; S(14): 94.2	219.0615605	30.89	3
S(4): 100.0	222.4499197	17.66	2
S(1): 0.0; T(4): 0.0; T(5): 0.0; S(9): 10	261.5843972	33.58	2
S(4): 100.0; Y(7): 0.0; S(9): 0.0	243.7863363	32.61	2
S(1): 0.0; T(3): 1.7; S(6): 98.3	201.1264572	49.6	2

Information Classification: General

T(13): 100.0; S(15): 95.7; S(17): 4.3;	274.0374391	109.49	3
S(3): 100.0	150.1289971	15.05	3
S(2): 0.0; T(6): 1.6; S(9): 98.4; T(11):	242.7644016	32.64	2
T(3): 100.0; T(8): 0.0; S(18): 0.0	398.5640594	59.19	3
S(6): 100.0; S(8): 0.0; T(13): 0.0; T(1	354.1812551	72.33	3
S(1): 50.0; S(3): 50.0; S(8): 0.0	98.91371356	7.72	2
T(2): 0.0; S(4): 0.0; S(6): 0.0; T(7): 0.	326.6977265	68.53	2
S(3): 100.0; S(7): 0.0; T(10): 0.0; S(1	412.4308499	121.26	2
S(7): 3.5; S(9): 96.5; S(19): 0.0	256.1622738	29.96	4
S(14): 100.0; T(18): 0.0; S(25): 0.0	520.0145558	71.35	3
S(2): 0.0; T(6): 0.0; S(11): 98.8; T(12)	363.2326567	80.13	2
T(3): 0.0; S(6): 100.0; T(8): 0.0	326.3481037	60.64	2
S(9): 100.0	386.595354	34.52	2
S(7): 0.0; T(17): 100.0; S(19): 0.0	416.2875296	71.55	3
S(11): 0.0; S(12): 0.0; S(13): 0.1; S(1	348.4508774	42.35	4
T(1): 0.0; Y(7): 0.0; S(11): 0.0; Y(13):	291.4616801	29.66	4
S(5): 100.0; T(17): 0.0	388.7382196	61.14	3
S(2): 0.0; S(5): 0.0; T(6): 0.0; S(18): 1	355.3546955	44.68	3
S(1): 0.0; S(3): 0.0; S(6): 100.0; Y(8):	199.8487525	41.71	2
T(1): 0.0; S(13): 100.0	364.17784	73.55	2
T(2): 0.0; S(8): 0.0; S(12): 100.0; S(1	343.5385163	89.32	3
S(3): 97.6; Y(7): 2.4	122.8352034	13.04	2
T(6): 99.9; S(12): 0.1; S(15): 0.0; S(1	110.6980957	19.8	4
S(3): 0.0; S(5): 0.0; T(13): 100.0; S(2	202.6972554	37.47	3
S(7): 0.0; S(10): 2.6; T(11): 97.4	198.6594538	10.79	2
S(1): 100.0; S(4): 100.0	256.0546986	17.13	3
S(4): 100.0	197.3182287	51.86	3
T(1): 49.9; S(2): 49.9; S(4): 0.1; S(5):	212.1341349	68.95	3
S(4): 100.0; T(6): 0.0	234.6736843	41.97	3
T(3): 0.0; S(8): 3.5; S(9): 92.8; T(11):	96.58460149	24.05	2
T(2): 0.0; T(4): 0.0; T(8): 0.0; S(11): 1	503.5126947	63.28	3
S(3): 100.0; S(5): 0.0; S(8): 0.0; T(12)	433.5378103	114.51	2
S(5): 100.0	240.4231287	38.13	2
S(10): 100.0	233.5602682	33.3	3
T(1): 0.0; S(7): 100.0	193.4005536	23.17	2
S(1): 0.0; S(5): 100.0	524.096153	65.3	4
S(2): 100.0; T(10): 0.0	203.9912269	27.23	2
S(3): 0.0; S(4): 100.0; S(8): 0.0	131.2991077	27.35	2
S(9): 100.0; S(12): 96.1; T(13): 3.9; S	194.3167428	40.91	2
S(5): 93.1; T(6): 6.4; S(9): 0.5; T(13):	139.5173196	11.62	4
S(6): 100.0	275.2200622	25.24	3
S(12): 0.0; T(14): 100.0	499.402485	55.3	2
S(9): 100.0; T(13): 0.0	172.5438379	18.29	3
Y(1): 0.0; S(4): 0.2; S(10): 99.8	71.53660387	1.52	3
S(3): 100.0; S(15): 0.0; T(32): 0.0	102.2303536	42.61	5
S(3): 100.0; S(5): 100.0; S(9): 0.0; S(	180.7716542	33.79	3
S(2): 100.0; S(4): 100.0	94.81659226	13.35	3
S(2): 0.0; Y(8): 0.0; S(11): 0.0; S(17):	348.5970255	76.62	2
S(3): 100.0	140.9637178	10.42	3
S(3): 100.0	247.981395	38.62	3
S(1): 0.0; S(3): 100.0	321.4586346	49.95	2
S(3): 100.0; S(4): 100.0	252.2869532	24.72	3
S(6): 100.0; S(12): 0.0	104.6506269	20.83	3
S(8): 3.0; S(13): 48.5; S(14): 48.5; T(	119.3738287	10.07	3
Y(2): 2.8; S(4): 48.6; S(5): 48.6; S(9):	114.6644661	15.07	3

S(7): 1.6; S(8): 98.4		216.65911	44.87	2
T(5): 0.0; S(10): 100.0		199.8092807	16.22	3
S(5): 50.0; S(6): 50.0		210.3699668	49.64	2
S(11): 0.0; S(21): 0.0; T(24): 0.0; T(3		164.4890703	6.58	5
S(7): 2.8; S(8): 97.2		90.2046662	16.53	2
S(4): 100.0; S(6): 0.0; S(10): 0.0		275.2787036	32.8	2
T(1): 0.0; S(4): 100.0; S(20): 0.0; Y(2		442.3429245	73.61	3
Y(5): 0.0; S(9): 0.0; S(14): 100.0; T(2		262.7947786	58.46	4
T(12): 100.0; S(15): 0.0		178.9763384	20.33	3
Y(2): 0.0; T(4): 3.7; S(5): 96.2; T(7): (		280.4272348	71.18	3
S(1): 0.0; S(3): 50.0; T(6): 50.0		146.5263903	17.74	2
T(2): 0.0; S(17): 100.0; S(33): 0.0		162.4317381	39.35	3
S(6): 100.0		294.560348	48.74	3
S(2): 0.0; S(4): 100.0; S(6): 0.0; S(7):		205.2384963	32.08	2
S(6): 98.3; S(8): 1.7		191.852514	13.03	3
S(7): 100.0; T(9): 0.0; Y(14): 0.0; T(1		218.4240116	36.93	4
S(2): 0.0; T(13): 0.4; T(14): 95.9; S(1		224.0098974	43	3
S(7): 100.0; S(12): 0.0		341.8573054	80.08	2
S(1): 0.0; S(3): 100.0; S(6): 0.0		210.2210723	44.53	2
S(2): 50.0; S(3): 50.0; S(11): 0.0; S(1		236.2989368	27.19	3
T(1): 0.9; S(5): 99.2; S(11): 92.0; S(1		80.9861763	22.64	3
S(2): 0.0; T(3): 0.0; S(9): 97.6; S(10):		206.5562326	31	2
S(5): 0.0; T(6): 0.0; S(11): 0.7; S(17):		326.5793265	56.98	3
S(3): 0.0; T(10): 0.0; T(13): 97.4; T(1		269.1557182	29.07	3
S(1): 0.0; S(2): 0.0; T(4): 0.0; S(5): 0.		107.5376203	8.21	3
S(2): 100.0; T(10): 0.0; S(20): 0.0		300.1230153	39.18	3
S(1): 0.0; T(22): 3.7; S(24): 92.5; S(2		284.5647704	44.11	4
S(2): 100.0; S(5): 50.0; S(8): 50.0		156.916223	28.79	2
S(1): 100.0; S(3): 0.0; S(10): 0.0; S(1		217.9586579	15.11	3
S(3): 0.0; S(4): 0.0; S(9): 0.0; S(15): 1		567.5829535	100.86	2
S(7): 0.0; T(11): 0.0; S(14): 100.0; S(		487.808306	111.62	3
S(5): 100.0; S(8): 99.9; T(9): 0.1		434.567324	101.36	2
S(6): 0.1; S(9): 99.9; S(12): 0.0		229.0463219	51.58	2
S(8): 0.0; S(12): 100.0; S(15): 0.0		409.9604069	53.83	3
S(2): 100.0		171.8715172	14.47	3
Y(4): 0.0; S(8): 0.0; S(10): 100.0; S(1		302.18407	23.96	3
S(2): 2.5; S(3): 97.5		255.0618329	18.99	3
S(6): 0.0; T(8): 0.0; T(14): 0.0; S(18):		331.4724989	80.59	3
S(1): 100.0		133.7070925	4.88	2
S(1): 100.0; S(3): 100.0		135.6970131	14.96	3
S(9): 100.0; S(12): 99.7; T(13): 0.3; S		171.6750925	22.9	4
S(18): 100.0; S(20): 100.0; S(22): 10(		159.7635983	18.95	4
S(24): 100.0; S(27): 0.0; T(28): 0.0		382.3343155	44.43	3
S(6): 4.7; S(9): 95.3; S(24): 0.0		175.569357	6.56	4
S(1): 0.0; S(4): 100.0; S(5): 100.0; T(		372.172305	45.67	3
T(4): 95.8; S(5): 2.1; S(7): 2.1		199.1790241	23.75	2
S(8): 100.0; T(11): 0.0; S(13): 0.0; S(		197.4440519	33.41	3
S(1): 0.0; S(2): 50.0; S(3): 50.0		226.5681786	39.82	2
T(8): 0.0; S(9): 0.0; T(14): 2.0; S(15):		330.8188607	136.08	3
S(2): 0.0; S(4): 2.4; S(5): 95.2; S(6): 2		228.2263431	67.73	2
S(4): 100.0; T(17): 0.0; T(18): 0.0		443.2992601	86.76	2
Y(1): 0.0; S(6): 100.0; S(11): 0.0		330.4456264	68.08	2
S(5): 100.0; S(8): 0.0; T(11): 0.0; S(1		340.2278243	42.56	4
S(3): 32.3; S(4): 32.3; S(5): 32.3; Y(1		116.3856465	40.57	3
S(3): 100.0		346.5154129	50.18	3

S(1): 33.3; T(3): 33.3; S(4): 33.3; T(7)	281.7843559	43.9	3
S(3): 100.0	136.8175465	22.6	2
T(9): 0.0; S(11): 0.0; S(13): 100.0	237.0284174	39.58	3
S(3): 100.0; T(7): 0.0; T(9): 0.0; S(10)	113.470585	15.15	2
S(1): 0.3; S(4): 99.7	87.08465233	12.48	3
S(2): 2.1; T(4): 97.9; S(6): 0.0	116.8943286	13.53	2
S(2): 0.0; S(3): 0.0; S(8): 100.0; S(10	158.605597	14.23	3
S(2): 0.0; S(3): 0.0; T(5): 0.0; Y(6): 1.	412.2398349	102.03	2
S(5): 50.0; Y(6): 50.0; T(10): 0.1; S(1	151.8198219	19.76	3
S(1): 0.0; S(2): 0.0; Y(5): 0.0; T(7): 0.	171.0709854	51.51	2
Y(7): 1.3; S(8): 98.7	255.3725429	31.64	2
S(2): 0.0; S(13): 100.0; S(14): 100.0	331.7255712	72.73	3
S(1): 100.0; T(5): 0.0; Y(7): 0.0; S(15)	173.5426773	19.39	2
T(5): 1.8; S(7): 98.2; S(9): 0.0; S(11):	259.8905764	63.59	2
S(1): 0.0; T(2): 0.0; T(3): 0.0; Y(10): (	359.3066041	59.23	2
S(1): 0.0; S(2): 0.0; S(14): 0.0; S(21):	190.6998136	43.55	3
T(15): 0.0; T(16): 50.0; S(17): 50.0	376.8900828	80.95	2
S(1): 0.0; S(6): 50.0; S(7): 50.0; S(11	158.6492049	45.4	3
S(5): 0.0; T(11): 0.0; S(13): 0.0; S(17	316.7044852	54.13	4
S(4): 2.7; S(7): 97.3	151.7637865	27.73	2
S(1): 0.0; S(7): 100.0; S(19): 100.0	224.8336111	17.17	4
T(1): 0.0; S(9): 100.0	213.8255272	74.16	2
S(2): 100.0; S(11): 100.0	436.2682878	56.26	2
Y(1): 0.0; S(6): 96.8; Y(7): 3.2; T(12):	234.6250614	41.85	3
S(4): 6.1; S(5): 93.9; T(9): 0.0; S(11):	172.7439847	11.96	3
T(1): 0.0; S(11): 100.0; T(15): 0.0; S(	396.8491959	70.01	3
S(5): 100.0; S(7): 100.0	250.9965701	54.37	2
S(2): 0.0; S(9): 100.0	219.2088994	19.97	3
S(12): 3.2; T(15): 3.2; Y(25): 93.5; Y(	205.5086115	59.76	3
S(4): 100.0; S(6): 100.0; T(8): 0.0; S(	241.1507693	27.67	2
S(11): 0.0; S(13): 100.0	413.4503049	46.11	2
T(6): 0.0; Y(12): 0.0; T(15): 0.2; S(17)	137.2166243	12.76	4
T(4): 0.0; S(6): 2.1; S(7): 97.9; S(11):	190.0365543	30.49	2
S(1): 0.0; S(3): 100.0; S(7): 0.0; S(9):	229.1564028	58.57	2
S(6): 99.1; S(8): 89.5; S(9): 5.7; T(10	155.0008725	31.21	3
T(6): 2.0; T(7): 47.0; S(8): 47.0; S(12)	182.7895371	35.51	3
S(5): 0.0; S(8): 100.0	274.1911023	49.87	2
S(3): 100.0; S(8): 97.7; S(10): 2.3; T(	248.0189196	24.91	3
T(1): 100.0; T(5): 0.0; Y(8): 0.0; Y(16)	409.9064598	89.26	2
T(5): 5.7; S(7): 91.5; S(9): 49.8; S(11	208.3647365	25.18	3
T(1): 0.0; S(5): 0.0; T(14): 0.0; S(20):	383.6439024	41.04	3
S(5): 99.9; T(7): 0.1; S(9): 0.0; S(13):	173.1178425	19.38	3
S(3): 2.4; S(4): 97.6; T(6): 0.0	215.2726585	42.29	2
T(11): 0.0; T(13): 1.6; T(15): 49.2; S(	195.4870689	47.2	2
S(4): 100.0	268.1272814	27.95	2
T(4): 1.8; T(5): 49.1; S(6): 49.1	168.3101097	7.88	3
S(7): 100.0; S(10): 100.0; S(14): 100.	93.21737717	7.2	3
T(3): 0.0; T(8): 0.0; S(9): 0.0; T(13): 1	390.0911123	49.1	2
S(8): 100.0; S(20): 0.0	197.7270906	43.88	3
S(1): 97.6; S(3): 2.4; T(6): 0.1; Y(9): (	242.036336	39.26	3
S(1): 0.0; S(4): 100.0; S(5): 100.0; T(	458.4698352	55.21	2
S(6): 2.5; S(7): 47.5; T(10): 2.5; S(15	136.8892145	18.28	3
S(1): 0.0; T(8): 0.0; T(10): 0.0; S(13):	299.0572474	53.32	3
S(2): 5.4; S(5): 94.6; S(9): 99.7; S(12	132.3195705	16.36	4
S(1): 100.0; S(7): 0.0; T(9): 0.0	159.3014277	27.83	2

T(1): 0.0; S(4): 0.0; S(8): 0.0; S(13): 2	72.33537327	10.44	4
S(7): 100.0	135.8878168	10.59	2
T(6): 100.0	521.2528023	90	2
S(6): 98.3; T(8): 1.7	262.2475586	21.96	2
S(12): 100.0; Y(17): 0.0	305.1275362	26.78	3
S(9): 99.8; T(11): 0.4; S(12): 99.8; S(	164.1587162	19.83	3
S(15): 0.0; Y(18): 0.0; S(19): 0.0; S(2	217.1333256	17.76	4
S(3): 100.0; S(9): 0.0; S(12): 100.0	317.7849577	58.8	3
T(13): 100.0	450.4263546	54.94	3
S(1): 0.0; S(4): 100.0; T(7): 100.0	195.4901455	32.07	2
S(7): 100.0; T(11): 0.0; S(18): 0.0	423.5776919	71.02	3
S(3): 0.0; S(7): 0.2; S(10): 99.8; S(13	247.540807	35.2	3
S(2): 0.0; S(4): 100.0; T(7): 0.0; S(9):	188.0804444	43.15	2
S(4): 100.0; S(7): 0.0	262.9431081	38.42	2
T(1): 0.0; S(13): 0.0; S(15): 100.0	377.2355553	71.14	2
S(7): 100.0; S(11): 0.0; S(13): 0.0; T(	132.8343248	11.29	4
Y(2): 100.0; S(3): 0.0	162.5015552	24.31	2
S(3): 48.8; T(4): 48.8; T(6): 2.3; S(11)	132.9052819	2.55	3
S(3): 0.1; S(7): 99.9; S(11): 0.0; S(13	181.0060091	19.45	3
S(14): 100.0	540.4157461	68.13	3
S(9): 0.2; S(11): 95.6; S(12): 4.2	192.0155959	21.56	3
S(2): 0.0; Y(3): 0.0; S(11): 97.0; T(15)	368.3522536	47.74	3
S(3): 100.0; S(10): 0.0	247.7546769	26.24	3
S(2): 5.9; S(4): 93.7; S(5): 0.4; S(7): 0	110.3315877	14.64	4
S(3): 100.0	119.0452197	14.74	2
S(3): 100.0; S(4): 0.0; S(5): 0.0	119.3716204	35.49	3
S(8): 100.0; S(10): 2.8; S(11): 2.8; T(	183.1030587	11.23	2
S(3): 0.0; S(7): 100.0	181.9746136	4.59	3
S(8): 0.0; S(18): 0.0; S(19): 0.0; S(23	335.3406759	43.38	3
S(4): 0.0; T(5): 0.0; S(11): 0.0; S(12):	225.7934638	31.91	3
S(9): 33.3; S(13): 33.3; T(15): 33.3	245.3150225	92.45	3
T(1): 0.0; T(6): 0.0; S(7): 0.0; S(14): 5	293.7397408	41.26	2
Y(4): 0.0; S(10): 100.0; S(15): 0.0	142.3856081	2.11	3
Y(1): 0.0; S(3): 0.0; S(4): 0.0; Y(6): 0.	97.76432702	7.41	3
S(3): 100.0; S(7): 0.0; S(9): 0.0; T(15	254.0053151	37	3
S(5): 100.0	147.3991618	24.67	3
T(1): 0.0; T(7): 100.0	243.2131863	21.56	3
S(3): 99.8; T(5): 98.9; T(8): 0.7; S(9):	37.95683666	6.55	3
T(3): 0.0; T(7): 0.0; S(11): 100.0	358.467257	77.34	2
S(4): 100.0; T(9): 0.0; T(12): 0.0; Y(1	371.6004038	59.07	2
S(4): 100.0; S(7): 100.0	286.3270101	14	2
T(3): 0.0; S(4): 0.0; S(11): 0.0; S(16):	181.0367937	10.89	4
S(1): 0.0; T(4): 95.6; S(5): 4.1; S(7): 0	136.2047888	6.65	3
T(3): 0.0; S(25): 0.1; S(26): 0.1; S(28	300.3871042	75.05	3
T(5): 100.0; S(9): 100.0	126.5394411	2.94	2
S(1): 0.2; S(3): 95.4; S(4): 4.3; S(12):	117.4828569	2	3
S(7): 99.9; S(12): 0.1	160.5750346	32.9	3
T(1): 100.0; T(12): 0.0	247.2227457	31.72	2
T(1): 97.0; S(5): 3.1; S(8): 99.8; S(10	90.16923812	16.46	2
T(2): 0.0; T(3): 0.0; S(4): 1.2; S(6): 48	189.2525738	45.95	2
S(13): 0.0; S(14): 0.0; S(16): 0.0; S(1	312.1029288	29.41	3
S(3): 0.0; S(4): 0.0; S(5): 0.0; S(6): 0.	429.2698477	71.29	2
S(3): 99.9; T(4): 0.1; Y(13): 0.0; T(14)	438.1163708	59.51	3
T(1): 0.0; T(4): 0.0; S(6): 0.0; T(12): 4	122.753801	44.2	3
T(9): 0.0; Y(10): 0.0; T(11): 0.0; S(17)	124.9190346	29.28	3

S(3): 0.0; S(6): 0.0; T(9): 0.0; T(11): 0	429.0611163	60.31	3
T(1): 0.0; T(4): 0.0; Y(9): 0.0; S(12): 0	205.6686785	52.16	3
T(1): 0.0; S(8): 99.9; S(10): 0.1; S(11	275.5322358	78.68	3
S(5): 100.0	189.4256255	32.17	3
S(2): 50.0; S(3): 50.0	252.6151703	42.82	3
S(4): 0.0; S(11): 100.0; S(19): 0.0	281.4088442	101.79	2
S(8): 100.0; S(10): 100.0	157.4107275	25.94	3
S(1): 0.0; S(6): 100.0; S(8): 0.0; S(9):	188.9499304	25.62	2
S(14): 100.0	258.7558688	63.61	3
S(2): 100.0	211.6053979	40.25	2
S(22): 100.0	299.8124478	22.88	5
Y(6): 0.0; S(14): 0.0; S(18): 100.0	304.4795172	31.2	4
S(3): 100.0	231.3328741	38.2	3
S(6): 100.0; S(7): 100.0; S(10): 100.0	208.015382	36.16	3
S(3): 0.0; S(5): 97.2; T(7): 2.8; S(8): 0	139.7149668	34.06	2
S(1): 0.0; S(3): 100.0; T(7): 0.0	186.8246249	35.33	2
S(3): 100.0	95.32759535	10.7	2
S(3): 100.0; T(11): 0.0; T(14): 0.0	249.4105689	44.63	2
S(10): 100.0	193.2608598	15.68	3
S(8): 100.0; T(10): 0.0	86.95320221	4.3	3
S(6): 100.0	329.2804855	52.66	2
S(4): 0.0; S(5): 0.0; S(6): 0.0; S(10): 1	330.0003582	18.08	3
S(1): 0.0; S(3): 0.0; S(7): 100.0	347.7439921	55.98	2
S(6): 0.0; Y(10): 2.2; S(11): 2.2; S(15	96.62498426	8.87	3
S(4): 100.0	231.0123673	23.13	2
S(1): 0.0; T(4): 0.0; S(7): 100.0	328.4323204	43.68	2
T(3): 1.5; S(5): 98.5; T(9): 0.0	130.4970362	23.8	2
Y(4): 98.4; T(5): 1.6	229.8685163	36.26	2
S(10): 100.0; T(12): 0.0	348.2391673	44.27	3
S(3): 100.0; S(6): 0.0	246.3553383	35.52	2
S(1): 96.9; S(2): 3.1; S(7): 0.0; S(10):	228.4511099	45.29	3
S(1): 100.0; S(13): 100.0	428.3618616	35	3
T(1): 0.0; T(2): 0.1; S(3): 2.2; S(5): 97	106.3919902	20.83	2
S(3): 0.0; S(5): 0.0; S(6): 0.0; S(9): 10	314.1935856	99.35	2
S(6): 97.2; T(8): 2.8; T(11): 0.0; T(13)	355.9539523	61.2	3
S(1): 0.0; S(2): 0.0; T(5): 0.0; T(6): 0.	227.7286102	47.73	2
S(1): 0.0; S(4): 0.0; S(6): 0.2; S(8): 4.	184.7720962	34.08	4
S(2): 0.0; S(10): 100.0; S(12): 0.0; T(	516.4543538	122.73	2
T(1): 0.2; S(8): 99.8; T(10): 0.0; T(11)	171.8777025	25.16	3
S(9): 100.0	401.2885057	69.93	3
S(1): 0.1; S(2): 3.5; S(4): 96.4; S(13):	315.9543835	22.48	3
S(2): 0.0; S(5): 0.0; S(8): 0.0; S(11): 0	241.7246905	43.42	2
S(6): 100.0; S(12): 0.0	608.2080953	75.95	2
T(1): 0.0; T(5): 0.0; S(6): 0.0; S(8): 98	444.5389605	117.67	2
T(3): 0.0; S(5): 3.1; S(8): 96.8; Y(13):	277.042407	38.48	4
T(2): 0.0; S(7): 100.0	119.2614247	18.18	2
T(1): 0.0; S(2): 0.0; T(8): 0.0; S(13): 1	327.1441261	62.11	3
S(6): 100.0	212.5608515	17.73	3
S(5): 0.0; S(7): 49.3; T(9): 49.3; S(10	313.8044859	50.19	2
T(1): 0.0; S(2): 100.0	166.9082898	21.9	2
S(4): 89.3; S(6): 5.4; S(13): 5.4	88.13977441	40.74	3
Y(1): 0.0; S(5): 48.9; S(7): 1.1; T(9): 0	153.9589389	36.38	3
S(3): 100.0	275.9179874	40.69	3
S(1): 0.0; S(6): 100.0; S(12): 0.0	156.916223	30.94	3
S(1): 0.0; Y(4): 0.0; S(9): 100.0; S(15	270.732264	65.83	2

S(5): 0.0; S(9): 0.1; T(10): 3.5; S(15):		186.6716178	19.93	4
T(2): 100.0; T(10): 0.0; Y(11): 0.0		193.6039748	23.03	2
S(9): 100.0; T(13): 0.0		259.2148944	20.31	3
S(8): 96.5; S(9): 96.5; T(10): 7.0		127.7964765	23.27	3
S(4): 100.0		162.6715378	18.86	3
Y(5): 98.3; T(6): 1.7		186.6025204	27.38	2
S(1): 100.0; S(4): 0.0; S(5): 99.9; T(7		201.5470239	62.76	2
S(2): 97.2; T(4): 2.8; S(6): 100.0		134.3611094	7.37	3
S(3): 100.0		135.766667	33.15	2
S(1): 0.0; T(2): 0.0; S(4): 0.0; S(5): 1.		145.0214169	30.45	2
S(7): 100.0		307.3243208	25.79	2
T(1): 0.0; T(5): 0.0; T(6): 0.0; S(12): 0		93.17582449	3.57	3
S(3): 100.0; S(7): 0.0; T(8): 0.0		293.7737969	54.36	2
Y(5): 0.0; S(9): 100.0		436.4850772	49.63	2
T(3): 0.6; S(7): 99.4; S(22): 0.0; T(23)		109.4636262	3.99	3
S(6): 100.0		161.9898159	9.57	2
T(3): 0.0; S(9): 100.0; S(11): 0.0; S(1		292.7340325	58.56	3
S(10): 100.0; S(11): 99.8; T(14): 50.1		72.01254491	4.1	3
S(6): 0.1; S(9): 99.9; S(13): 0.0		199.8232534	35.05	3
S(2): 100.0; S(4): 0.0; T(8): 0.0; Y(14)		381.7357161	75.25	3
S(3): 100.0; S(12): 0.0		117.3726157	0.25	3
S(9): 96.0; S(14): 4.0		112.5191629	3.25	3
T(1): 0.0; T(2): 0.0; S(5): 100.0; S(11)		351.1838354	53.56	2
Y(5): 0.0; S(10): 100.0		277.0655714	57.89	2
S(3): 99.2; S(6): 0.4; S(7): 0.4; T(11):		120.0737423	13.13	3
S(7): 100.0; S(10): 96.4; T(12): 51.8;		114.8789724	16.19	3
S(10): 100.0		379.6674342	42.17	3
Y(1): 0.1; S(2): 99.9; Y(8): 0.0		121.5295847	11.84	2
Y(2): 0.0; T(3): 50.0; S(5): 50.0		121.2463108	16.06	2
S(3): 97.1; S(5): 2.8; S(6): 0.1; S(9): 0		182.5100446	12.42	2
S(12): 1.6; T(16): 98.4; T(22): 0.0		258.8700491	50.45	3
S(13): 100.0		411.1877529	62.77	3
T(1): 0.0; S(6): 100.0		373.1165416	49.85	2
T(2): 0.0; S(7): 0.0; T(13): 0.0; T(14):		284.3294015	60.39	3
T(2): 0.0; S(5): 100.0; S(7): 100.0; S(		447.2006311	55.37	3
S(4): 100.0; Y(11): 0.0		295.0675491	48.7	2
S(1): 0.1; S(3): 97.4; S(5): 2.5; S(8): 0		333.6771392	51.05	3
S(1): 0.1; T(2): 2.4; S(3): 95.2; T(4): 2		259.417339	44.78	3
S(3): 0.0; T(7): 96.8; T(9): 3.0; T(16):		234.8751259	51.7	4
S(1): 100.0; S(7): 100.0		430.8172513	35.39	2
T(1): 0.1; S(4): 99.9		72.53833963	21.91	3
S(2): 99.9; T(3): 0.1; S(5): 50.0; T(8):		142.180186	38.32	2
S(7): 0.0; S(15): 100.0		381.5351443	65.29	2
S(2): 1.9; S(4): 98.1		182.1360136	28	3
S(4): 100.0		381.0332189	33.55	3
S(2): 0.1; S(3): 2.6; T(4): 97.3		86.56118807	15.04	2
T(2): 0.0; T(8): 0.0; T(9): 0.0; T(11): 2		233.9123214	54.85	2
S(3): 100.0; T(6): 0.0; S(7): 0.0; S(8):		326.3761716	74.36	2
T(5): 100.0; T(8): 0.0		137.387061	22.45	3
S(1): 0.0; S(2): 0.0; S(3): 0.9; S(5): 49		223.3203066	31.4	2
S(4): 100.0; S(8): 0.0		265.0288614	50.55	2
S(6): 0.0; S(12): 0.1; S(13): 2.6; S(14		197.9863351	57.44	2
T(2): 0.0; Y(4): 0.1; S(7): 99.9		142.5969198	22.53	3
S(3): 50.0; T(4): 50.0		127.3438915	19.52	3
S(10): 99.9; S(13): 0.1; S(16): 0.0		175.6414444	32.38	3

Information Classification: General

S(3): 96.9; S(4): 3.1; S(9): 0.0; S(15):		113.8175709	24.96	2
S(1): 0.0; T(8): 0.0; S(10): 0.0; T(11):		185.3173286	19.98	4
T(1): 0.0; S(3): 50.0; S(5): 50.0; S(8):		173.0831167	15.44	2
S(1): 0.0; S(5): 0.0; S(10): 0.0; S(17):		278.5661187	53.71	3
S(9): 99.9; S(11): 0.1; S(12): 0.0		130.8728333	12.73	3
S(1): 100.0; S(7): 0.0		268.1611464	26.82	2
S(8): 1.2; S(9): 98.8		375.2117967	77.49	2
T(4): 97.2; T(7): 2.8; T(12): 0.0; S(14)		174.1488017	25.57	3
S(4): 2.7; S(5): 97.3; S(8): 0.0		258.7993127	45.38	3
S(7): 98.3; S(8): 1.7		211.4867174	26.33	2
S(3): 100.0		116.7756137	8.34	3
T(2): 0.0; S(3): 100.0; S(9): 0.0		178.2775633	60.78	2
S(8): 100.0		341.6979192	47.15	2
S(2): 0.0; Y(5): 0.0; S(7): 100.0; S(9):		347.5696795	69.15	2
S(1): 0.0; Y(3): 0.0; T(6): 0.0; S(9): 0.		242.4406645	45.5	3
S(5): 100.0		279.6366976	38.05	3
S(5): 100.0; T(7): 0.0; T(8): 0.0		309.2862122	36.14	2
S(3): 100.0; S(5): 100.0		188.8307603	25.74	3
S(3): 0.0; T(6): 0.0; T(7): 0.0; T(8): 0.		280.7447547	66.91	2
S(1): 0.0; T(2): 0.0; S(8): 1.9; S(9): 9		257.8284295	38.63	2
T(2): 0.0; T(6): 0.0; S(10): 100.0		169.1496276	46.41	2
S(8): 0.0; Y(10): 0.0; S(12): 99.9; T(1		261.2918243	23.98	3
S(7): 0.0; S(13): 100.0		241.9565966	36.84	3
T(3): 2.6; S(5): 97.4; S(9): 100.0; S(1		300.8587754	27.47	3
S(1): 0.0; S(3): 100.0; T(5): 0.0		201.3773233	32.03	2
S(2): 0.0; T(3): 0.2; S(5): 99.8; S(9): (		229.2522999	28.55	3
T(7): 0.1; T(14): 99.9		212.9604691	8.17	4
S(3): 1.5; S(7): 98.5; S(13): 0.0; S(15		612.1419644	113.66	3
S(1): 0.0; S(3): 100.0; S(9): 0.0		134.8012672	17.89	2
S(7): 49.9; S(8): 49.9; S(10): 0.1; T(1		200.6375019	27.14	3
T(4): 50.0; S(5): 50.0; T(16): 0.0		232.1267134	17.86	3
S(7): 100.0; T(10): 0.0; S(13): 0.0; T(		392.5185292	74.52	3
S(2): 100.0		172.38704	42.93	2
S(2): 99.8; S(5): 0.2; T(8): 0.0; S(9): (		110.9745033	7.08	3
S(3): 100.0		252.2009787	40.44	2
Y(1): 0.0; S(5): 100.0; S(11): 0.0; S(1		257.1628597	29.45	2
S(3): 100.0; S(9): 0.0		220.5489046	39.72	3
S(4): 100.0; S(7): 100.0		437.6833095	34.92	2
S(2): 0.0; S(3): 0.0; T(5): 0.0; S(6): 0.		287.0507399	83.29	2
T(1): 3.4; S(3): 96.6; S(11): 0.0; S(13		231.2402508	57.71	3
T(1): 0.0; T(3): 0.0; S(6): 100.0		304.4305492	60.58	2
S(7): 100.0		215.9711526	46.21	2
S(2): 0.0; S(18): 96.2; T(22): 1.9; S(2		324.9674394	33.5	4
S(8): 100.0; S(10): 100.0; S(15): 100.		267.7290404	27.8	3
S(1): 0.0; S(5): 100.0; T(10): 0.0; T(1		363.8338593	102.58	2
S(5): 100.0		152.652373	10.07	2
S(6): 0.0; S(13): 100.0		228.1324423	40.35	3
T(6): 0.0; S(8): 0.2; S(11): 95.6; S(13		177.2854833	27.94	3
S(1): 100.0; S(7): 0.0; S(9): 0.0; Y(11		482.2426767	98.59	2
S(2): 0.0; S(7): 0.0; T(12): 0.0; S(13):		271.9078068	52.4	4
S(11): 100.0		408.4607934	85.36	3
S(8): 100.0; S(10): 0.0		384.0921377	37.24	3
T(8): 0.1; S(10): 3.2; S(11): 96.5; Y(1		142.9244929	29.45	2
S(1): 0.0; S(7): 0.0; S(10): 0.0; S(16):		238.1621805	42.53	3
S(9): 100.0		101.0941026	12.42	4

T(1): 0.0; Y(13): 0.0; S(16): 32.9; Y(1	276.5799843	55.79	4
S(3): 100.0	266.2636633	42.99	2
S(1): 0.3; S(4): 5.2; S(5): 93.5; S(8): (	100.1305385	15.29	3
T(3): 0.0; S(4): 0.0; S(6): 96.6; T(7): 1	248.1139703	76.04	2
Y(2): 50.0; S(3): 50.0; S(5): 100.0	198.0170406	10.91	3
T(20): 0.1; Y(24): 7.4; S(25): 92.6	111.9364543	0.84	6
Y(4): 0.2; T(6): 99.8; S(9): 0.0; S(13):	265.2139182	68.46	3
T(6): 100.0; S(11): 0.0; T(15): 0.0	213.2903031	22.86	3
S(1): 100.0; S(6): 0.0; T(7): 0.0	187.8265625	28.48	2
T(1): 0.0; S(2): 0.0; S(3): 0.0; S(5): 0.	267.5646092	61.34	2
T(1): 0.0; S(6): 100.0	242.8888035	68.01	2
S(2): 50.0; S(3): 50.0	67.24848393	16.36	2
S(1): 0.0; S(4): 50.0; S(7): 50.0	203.8802076	25.11	2
S(2): 0.1; S(5): 2.7; S(8): 97.2; S(11):	162.3219212	25.37	2
T(3): 0.0; T(9): 0.6; S(11): 0.6; T(13):	209.7979336	45.87	3
S(22): 50.0; S(25): 50.0	367.7393711	91.87	2
T(1): 0.1; S(3): 99.9	146.8968089	9.55	3
S(3): 0.0; S(6): 1.6; S(7): 48.4; S(9): 1	254.9594264	73.66	3
S(2): 0.1; S(3): 99.9; T(5): 2.4; Y(6): (	337.5010414	66.38	2
S(18): 100.0; T(20): 93.2; S(21): 6.9;	98.70048438	15.46	3
S(13): 100.0; T(17): 0.0; S(24): 0.0	280.1574294	60.02	4
S(2): 100.0; T(4): 0.0; S(6): 0.0	142.2386168	34.7	2
T(1): 0.0; S(3): 0.0; T(5): 0.0; S(6): 0.	279.2130446	29.53	3
S(1): 100.0	222.347093	42.44	2
S(15): 99.9; S(17): 0.1	298.5583	27.73	3
S(1): 0.0; T(5): 0.0; S(11): 100.0	247.7003519	37.53	2
S(2): 0.0; Y(5): 0.0; S(7): 100.0; S(9):	312.0992835	60.13	3
S(1): 0.0; S(3): 100.0; S(10): 0.0	224.9160821	32.97	2
S(4): 0.0; S(5): 0.0; S(13): 100.0	338.2961739	68.82	2
S(1): 0.0; S(3): 100.0; S(8): 0.0	239.3581904	33.99	2
S(4): 0.0; S(9): 100.0	422.8452135	25.78	2
S(3): 0.0; T(7): 100.0; T(13): 0.0	282.1678689	38.67	2
T(2): 0.0; S(8): 0.0; S(12): 0.0; T(20):	450.6221407	45.58	4
S(1): 0.0; S(3): 100.0; S(10): 0.0	240.4400656	32.39	3
S(5): 100.0; T(8): 0.0	187.2478413	24.39	2
T(2): 0.0; S(4): 0.0; S(8): 100.0	186.9212493	36.06	2
S(1): 100.0; S(18): 0.0	149.1616338	18.42	3
S(3): 100.0	368.8004268	69.6	2
S(2): 0.0; S(4): 100.0	263.3377917	42.01	2
S(1): 0.0; T(3): 0.0; S(4): 0.0; T(5): 0.	383.7119161	51.8	3
S(1): 2.9; S(4): 97.1; S(8): 0.0; S(12):	178.0564299	20.84	3
S(3): 100.0; S(17): 0.0; T(19): 0.0; T(	167.4279844	28.64	3
S(8): 100.0; S(9): 100.0; T(12): 0.0; S	229.920062	40.26	2
S(6): 0.0; S(11): 100.0; S(15): 100.0	153.2577415	2.16	3
Y(4): 0.0; T(8): 100.0; Y(13): 0.0	252.0788262	33.29	3
S(5): 100.0	299.3250986	36.54	3
S(1): 0.0; S(2): 0.0; S(5): 50.0; S(6): 5	249.2271445	30.54	2
T(2): 0.0; S(4): 0.0; S(9): 100.0	227.4890312	73.45	2
S(4): 100.0; S(6): 0.0; T(8): 0.0; S(9):	183.7163147	29.58	3
S(3): 100.0; S(6): 0.0	306.6032973	39.73	2
S(8): 100.0; S(11): 100.0; S(14): 0.0	214.888309	50.04	2
S(4): 96.3; T(5): 3.6; S(7): 0.1	165.2518088	24.55	3
S(3): 100.0	183.205254	44.29	2
S(3): 2.1; S(6): 97.9; T(11): 0.0; S(13	329.7309311	31.08	3
S(3): 0.1; S(6): 99.9	234.2875943	63.62	2

Information Classification: General

T(8): 100.0; T(15): 0.0; S(22): 0.0		213.9489331	29.23	3
S(1): 0.0; S(3): 1.2; S(4): 98.8; T(9): (		272.4126583	37.11	2
T(1): 0.0; T(5): 3.3; S(6): 3.3; S(9): 93		99.53638091	14.42	2
S(3): 100.0; T(7): 100.0; S(14): 0.0; T		198.3458321	17.36	3
Y(7): 0.0; S(11): 100.0		340.7441896	43.9	3
S(1): 0.0; S(3): 100.0		142.852868	16.35	2
T(9): 50.0; S(15): 50.0		174.585091	6.44	3
S(6): 100.0		163.6084917	11.02	4
S(2): 0.0; S(5): 0.0; S(12): 99.8; S(14		263.7133772	45.64	3
S(2): 0.1; S(4): 99.9; S(7): 0.0; S(9): (		130.9663311	17.8	2
S(3): 100.0; S(8): 0.0; S(12): 0.0		246.4957601	34.18	3
S(2): 50.0; S(3): 50.0; S(6): 97.2; S(8		161.1894219	23.28	2
Y(1): 0.0; S(7): 100.0; S(12): 0.0		257.6817021	24.04	2
S(7): 100.0		283.5782306	29.82	3
S(3): 100.0; S(5): 100.0; S(9): 0.0; T(		196.9158812	48.18	3
Y(2): 0.1; S(4): 99.9; T(10): 0.0		122.8352034	22.94	2
S(3): 2.2; S(6): 97.8; S(11): 100.0		215.8945496	69.34	2
T(1): 0.0; S(4): 100.0		161.6139008	19.54	2
T(3): 0.0; T(9): 50.0; S(10): 50.0; S(1		313.8987637	82.49	2
T(1): 0.0; T(2): 0.0; S(4): 0.0; S(13): 1		259.3668364	75.36	3
T(3): 0.0; T(9): 0.1; S(11): 0.1; T(13):		338.4015405	73.47	3
S(2): 0.0; S(5): 0.0; T(9): 100.0; T(20		262.974614	58.74	4
T(1): 0.0; T(3): 0.0; S(8): 100.0		277.0655714	51.41	2
T(1): 0.0; S(3): 0.0; T(5): 0.0; S(6): 0.		310.8827193	64.08	2
S(19): 0.4; T(21): 0.4; T(24): 0.8; T(2		151.7301456	28.32	4
T(2): 0.0; T(4): 0.0; T(8): 0.2; S(11): 9		156.8435679	14.27	3
T(2): 0.0; S(3): 0.0; S(4): 0.0; T(14): (		320.0363155	50.68	3
S(2): 0.0; S(7): 100.0; S(16): 0.0		427.188603	103.6	2
S(5): 100.0; S(15): 0.0		133.007878	14.42	3
T(8): 32.9; T(9): 32.9; S(10): 32.9; T(		186.2813107	35.71	3
S(1): 0.0; T(3): 0.0; T(6): 100.0		154.921481	32.73	2
S(3): 100.0; S(11): 0.0; S(18): 0.0; S(		240.929883	38.68	3
S(3): 0.0; S(6): 0.0; T(12): 0.0; S(17):		311.498337	41.26	3
S(1): 0.0; S(2): 0.0; S(3): 100.0; T(5):		146.2922207	20.87	2
T(1): 0.0; S(2): 0.0; T(3): 0.0; S(6): 0.		289.9845962	51.79	2
S(3): 1.8; S(5): 96.3; Y(6): 1.8; S(9): (		222.1961057	43.06	2
S(4): 100.0; S(5): 100.0; S(6): 0.0; T(		212.1231779	27.58	2
S(5): 0.0; S(13): 0.0; S(17): 0.0; S(26		330.4139284	42.09	3
S(6): 100.0		165.1009889	19.22	2
S(1): 0.0; S(3): 100.0		199.7254706	32.41	2
S(2): 0.0; S(7): 100.0; S(12): 0.0; T(1		296.7592778	35.38	3
S(4): 0.0; S(17): 5.9; S(19): 94.1; S(2		180.6144029	18.46	4
S(1): 0.0; S(7): 100.0		458.8915705	62.43	2
S(3): 0.0; S(6): 100.0		141.1345384	6.92	3
T(3): 50.0; S(5): 50.0; S(7): 0.0		248.0878087	37.7	3
S(4): 100.0; Y(12): 0.0		397.2979906	81.64	2
S(11): 0.0; Y(14): 100.0		122.2992666	20.04	3
S(5): 100.0		128.5329087	15.84	3
S(3): 100.0; S(8): 0.0; S(15): 0.0		253.4940967	71.91	3
S(8): 100.0		298.6923517	39.58	2
Y(10): 0.0; S(17): 100.0; Y(29): 100.0		135.5453132	13.52	3
S(3): 94.7; S(5): 2.6; S(6): 2.6; T(9): (		142.7569499	14.67	3
S(7): 50.0; S(12): 50.0		147.293248	39.36	2
S(2): 0.0; S(3): 100.0		157.8861946	23.69	2
T(8): 50.0; S(10): 50.0; S(12): 0.0		227.9322821	35.61	3

Information Classification: General

S(3): 100.0; S(13): 0.0	202.7654336	29.88	3
S(1): 3.8; S(3): 92.4; S(5): 3.8; T(12):	183.3863254	21.06	3
S(4): 100.0	198.7843805	13.35	2
S(10): 0.0; S(12): 98.2; S(13): 1.8	383.553033	80.27	3
S(5): 100.0; S(7): 0.0; Y(9): 0.0	313.4637068	31.3	2
T(1): 0.0; T(3): 99.9; S(4): 0.0	95.25329496	17.68	3
S(4): 0.0; S(6): 0.1; S(8): 2.0; S(9): 49	258.5955982	93.38	3
S(1): 100.0; S(5): 0.0; S(6): 0.0; S(7):	299.4650782	81.16	2
T(2): 0.0; S(14): 100.0; S(15): 100.0;	234.7090435	78.85	3
S(2): 0.0; T(4): 0.0; T(9): 0.0; S(17): 1	219.2561702	24.31	3
S(1): 0.0; S(3): 94.4; T(4): 5.0; T(6): 0	75.95626548	7.12	3
S(10): 100.0; S(13): 100.0	236.2845879	26.22	4
S(11): 100.0	431.377211	85.43	2
S(2): 100.0; S(9): 0.0	118.6291025	10.09	2
S(2): 0.0; S(4): 0.0; S(7): 0.0; S(8): 0.	296.523394	77.61	2
S(3): 100.0; S(7): 100.0	244.6558795	41.76	2
Y(4): 0.0; S(13): 94.4; S(16): 94.4; S(	135.9497549	1.13	3
S(1): 0.0; S(3): 100.0; S(6): 100.0	236.7006785	30.91	2
S(3): 100.0	240.1765619	29.87	2
S(2): 0.0; S(5): 100.0; S(8): 0.0; Y(15	327.0648456	46.68	3
S(1): 0.0; T(3): 0.0; S(4): 0.0; S(12): 0	250.1712269	42.43	3
S(11): 5.5; S(14): 94.5; S(18): 0.0	138.5179899	11.97	3
S(1): 0.0; Y(3): 0.0; T(6): 0.0; S(9): 1.	314.0330761	66.24	2
S(1): 0.0; T(3): 0.0; S(4): 0.0; S(5): 0.	279.0422527	61.91	2
T(7): 0.1; S(12): 99.9; S(15): 100.0; S	273.2125402	62.96	3
T(3): 0.0; S(5): 0.0; T(7): 0.0; S(8): 0.	382.1117816	43.21	3
S(3): 100.0; S(6): 0.0	256.9322479	26.52	2
T(2): 0.0; S(3): 0.0; S(8): 100.0; S(15	443.2019072	60.27	3
Y(10): 0.0; S(15): 0.0; S(16): 0.0; S(2	383.3672187	59.04	3
T(9): 0.1; S(15): 99.9	359.4593752	34.55	3
T(13): 2.9; S(15): 48.6; S(17): 48.6; S	185.023849	42.4	4
S(15): 100.0; S(19): 0.0; T(20): 0.0; S	247.0029752	15.75	3
T(14): 0.0; S(15): 0.0; T(25): 0.0; S(3	352.9857475	73.53	4
T(2): 0.0; T(3): 0.0; S(12): 100.0; Y(1	195.5385041	12.18	3
S(3): 100.0; T(7): 0.0; Y(10): 0.0; S(1	425.5074876	97.56	2
S(1): 100.0	373.7697479	61.07	2
S(1): 0.0; S(3): 0.0; T(4): 0.0; S(6): 0.	143.4003908	18.99	3
T(3): 100.0	232.7514028	32.38	2
S(4): 100.0; S(6): 0.0; T(7): 0.0	213.0243474	33.15	2
S(13): 100.0	240.5307959	25.5	3
T(1): 0.0; S(4): 100.0	202.9629862	33.78	2
T(1): 0.0; S(13): 100.0	276.6977547	70.67	2
S(3): 50.0; S(6): 50.0; T(13): 0.0; S(1	194.2045294	49.59	3
Y(4): 0.0; T(11): 0.0; S(14): 100.0; T(	256.3895051	72.16	3
S(11): 100.0	313.6127041	51.84	2
S(1): 0.0; T(3): 0.0; S(4): 0.0; S(7): 0.	433.2990955	112.25	2
S(1): 3.0; S(2): 97.0; S(6): 0.0	125.9465471	8.95	3
S(3): 100.0; S(11): 0.0	290.5066065	37.89	3
T(6): 25.0; T(8): 25.0; S(11): 25.0; S(	167.1054233	20.63	3
S(1): 0.0; Y(4): 0.0; S(11): 100.0	291.1473504	31.27	3
S(3): 33.3; T(4): 33.3; S(5): 33.3; S(7	162.3656962	41.32	2
Y(10): 0.0; T(16): 0.0; S(18): 0.0; T(2	319.6822052	57.68	3
T(3): 0.0; S(7): 100.0	209.0482581	30.51	2
S(3): 100.0; S(5): 0.0; S(9): 0.0; S(16	493.3595115	77.36	2
T(3): 0.0; S(6): 100.0; S(8): 100.0; T(	325.9499	44.83	2

Information Classification: General

T(7): 33.5; T(8): 33.5; T(10): 33.5; T(1	146.8036496	41.21	3
Y(2): 0.0; T(7): 0.0; S(12): 0.2; T(13):	258.6431719	91.32	3
T(1): 0.0; S(3): 100.0	201.3382519	22.5	2
S(1): 0.0; S(4): 0.0; T(6): 0.0; T(13): 2	309.0293713	52.77	3
S(1): 3.9; S(3): 96.1; T(6): 0.0; S(8): 0	266.6850333	36.32	3
S(6): 100.0; S(9): 0.0; S(14): 0.0	397.9322706	98.32	2
S(6): 100.0; T(8): 0.0; S(13): 0.0; S(1	497.7817098	135.86	2
S(3): 100.0	241.4733331	43.67	2
S(1): 100.0	364.9651022	37.84	3
S(1): 0.0; Y(3): 0.0; T(6): 0.0; S(9): 0.	133.526316	13.63	3
S(1): 0.1; S(3): 99.9; T(4): 0.1; T(6): 0	157.3707997	30.52	2
S(7): 1.9; S(8): 98.1	182.1360136	30.75	2
S(4): 100.0; S(7): 0.0; S(9): 0.0	212.9458156	35.01	2
S(4): 0.0; S(10): 0.0; S(11): 0.0; S(12	487.9721726	81.67	3
S(1): 0.0; S(6): 100.0	170.1587989	31.63	2
S(2): 100.0; T(7): 0.0; S(8): 0.0	137.4231501	18.5	2
T(2): 0.1; S(5): 99.9	126.1768323	5.19	3
S(2): 0.0; S(12): 100.0; S(17): 33.3; T	199.5726298	17.46	3
S(2): 48.3; S(3): 48.3; S(6): 3.0; T(8):	94.22955859	9.78	3
T(9): 33.3; S(12): 33.3; T(15): 33.3	128.6517509	32.77	3
S(1): 100.0; S(5): 0.0; S(6): 0.0	219.5379122	34.04	2
S(1): 94.6; T(3): 2.7; S(4): 2.7	138.4790473	15.28	2
S(5): 0.0; S(9): 100.0	221.0446213	36.45	2
T(2): 0.0; S(4): 0.0; S(5): 0.0; S(6): 0.	128.3017505	8.15	3
Y(1): 0.0; S(2): 0.0; S(8): 3.0; S(9): 3.	166.6904999	9.6	3
T(1): 0.0; T(2): 0.0; Y(4): 0.0; S(6): 0.	164.7501416	6.94	3
T(2): 0.0; S(4): 0.0; T(8): 0.1; S(9): 99	227.1763726	50.02	2
S(2): 0.0; S(7): 100.0	273.2794069	47.73	2
S(3): 50.0; T(5): 50.0; T(10): 0.0	192.9446764	26.31	3
S(1): 0.0; T(5): 100.0; S(12): 0.0; S(1	268.8434267	52.72	2
T(3): 0.0; T(8): 0.0; T(11): 4.3; S(13):	162.8241838	16.78	3
S(4): 0.0; T(7): 0.0; T(12): 0.0; S(14):	417.7028584	117.09	2
T(2): 0.0; T(5): 0.0; T(6): 1.5; S(8): 98	247.2227457	68.22	2
S(4): 50.0; S(5): 50.0	70.81922103	9.36	3
Y(8): 0.0; S(10): 100.0; S(14): 0.0	370.4634656	49.99	2
S(2): 0.0; Y(3): 0.0; S(6): 0.0; S(11): 1	328.7114413	69.53	3
T(6): 0.0; S(11): 100.0	177.7779723	27.27	2
T(1): 0.0; T(2): 0.0; S(14): 100.0	309.6820433	32.28	2
T(2): 0.1; S(3): 99.9; T(10): 0.0	151.7721027	32.29	2
Y(1): 0.0; S(4): 100.0; Y(7): 0.0	160.2154709	53.74	2
S(17): 100.0	175.6374122	5.96	4
T(3): 0.0; S(9): 0.0; S(11): 0.0; S(12):	252.1323071	24.12	3
S(8): 100.0	148.2433313	10.87	3
S(8): 100.0	363.6252838	45.85	2
S(3): 100.0; S(7): 0.0; T(12): 0.0	260.8896905	83.56	2
S(8): 100.0; S(13): 100.0	170.4247322	12.13	3
T(1): 0.0; S(5): 0.0; S(11): 100.0	254.3641422	24.76	2
T(2): 0.0; S(15): 100.0	204.5362726	35.38	3
S(3): 0.0; S(6): 100.0; T(11): 0.0	204.0807269	35.98	2
T(4): 95.7; S(5): 2.2; S(6): 2.2	167.8369215	39.83	2
S(2): 0.0; S(3): 100.0; S(7): 0.0; S(11	322.161327	82.26	2
S(1): 0.0; S(2): 0.0; S(3): 100.0	163.5931775	23.43	2
S(2): 0.0; S(7): 50.0; S(9): 50.0	180.3606018	35.5	2
T(6): 100.0	196.8499403	12.71	3
S(1): 100.0; S(3): 100.0; T(5): 0.0; S(	170.9390759	34.08	2

T(1): 0.0; S(3): 0.0; S(13): 100.0; S(1	274.3402879	29.51	3
S(8): 0.0; S(12): 100.0; T(16): 0.0; S(	406.0318162	109.43	2
S(3): 49.4; S(4): 49.4; T(6): 1.2; S(8):	147.2710871	32.84	2
T(5): 100.0; S(10): 0.0	224.1305517	25.73	3
S(4): 100.0	367.1302029	42.28	2
S(1): 0.0; S(3): 0.0; S(4): 0.0; S(6): 0.	179.8465015	53.46	2
S(1): 97.7; S(4): 2.3	180.3606018	12.03	3
T(3): 0.0; S(7): 0.0; S(8): 0.0; S(15): 1	258.3124672	38.35	3
S(5): 100.0	296.4806482	16.77	2
S(3): 0.0; T(8): 0.0; T(10): 0.0; S(12):	292.5791322	61.02	3
S(4): 0.1; S(7): 99.9; S(9): 100.0	314.9286742	23.13	3
S(3): 0.0; S(4): 0.1; T(5): 97.8; S(6): 2	234.5308477	39.39	3
S(3): 100.0; S(7): 100.0; S(9): 0.0	172.34291	8.58	3
S(6): 100.0; S(7): 100.0	268.38356	47.92	3
T(5): 0.0; S(8): 2.5; S(9): 97.5; T(18):	280.4213301	98.85	2
S(9): 100.0	84.53768137	0.94	2
S(2): 0.0; S(7): 100.0	202.436656	43.03	2
S(2): 0.0; T(3): 0.0; S(7): 50.0; S(9): 5	145.3089892	16.46	2
S(2): 49.2; T(3): 49.2; S(4): 1.7; S(7):	291.6878045	67.09	3
S(2): 97.5; S(3): 2.4; S(4): 0.1; T(5): 0	164.226472	22.75	2
S(11): 100.0; S(13): 100.0	425.8509767	87.82	3
T(2): 0.0; Y(4): 0.0; S(11): 100.0; S(1	328.8564576	82.21	2
S(1): 0.0; S(10): 0.0; S(15): 100.0; S(	262.4601634	29.55	3
S(3): 100.0	265.0288614	34.01	2
S(2): 99.9; S(4): 0.1	208.7934181	24.86	2
S(8): 100.0; S(15): 0.0	286.9528417	68.05	2
S(3): 0.0; S(9): 0.0; T(10): 0.0; S(12):	167.0633679	9.53	4
S(2): 0.0; S(4): 0.0; S(10): 100.0; T(2	214.79498	25.97	4
S(3): 100.0; T(10): 0.0; T(13): 99.9; T	361.3796282	31.94	3
T(3): 0.1; S(6): 99.9; S(9): 0.0; T(15):	258.6814908	36.87	3
S(10): 100.0; S(13): 0.0	301.4888745	50.69	2
S(5): 0.0; S(9): 0.0; S(13): 100.0; S(1	302.2776063	31.43	4
S(3): 100.0; S(7): 0.0; S(9): 0.0	450.9865593	122.13	2
S(4): 0.0; T(6): 0.0; S(9): 0.0; T(13): 0	205.2797955	41.78	3
T(2): 0.0; S(7): 100.0	121.4310383	16.13	3
S(8): 2.0; S(9): 98.0; S(14): 0.0; Y(20	359.7854309	39.58	3
S(3): 100.0; S(5): 0.0	212.9541749	40.9	2
T(7): 0.1; T(8): 0.1; T(10): 33.2; T(14)	210.400277	38.57	4
Y(7): 3.5; S(8): 96.5; S(13): 0.0	138.5491969	15.82	3
T(6): 100.0; S(11): 0.0	328.4392394	38.66	2
T(3): 0.0; S(7): 100.0; S(11): 100.0	236.007367	55.53	2
S(1): 0.0; T(6): 0.0; S(10): 0.0; S(18):	346.5073328	25.7	3
S(6): 0.0; T(8): 0.0; T(9): 0.0; T(15): 2	280.5005602	68.9	2
S(1): 50.0; S(3): 50.0; T(9): 0.0; T(10)	101.4799922	5.46	3
S(1): 100.0; S(7): 100.0; S(13): 100.0	241.1315157	11.57	3
T(8): 99.9; S(10): 50.0; S(11): 50.0; Y	149.0974771	18.71	2
T(10): 0.0; T(18): 2.9; T(22): 97.1	257.5804513	26.42	3
S(2): 0.0; T(3): 0.0; T(8): 0.0; T(14): 1	303.3971473	41.47	3
Y(5): 50.0; S(6): 50.0; S(11): 100.0	134.5661063	14.63	2
S(2): 0.0; S(4): 1.7; S(5): 98.3; Y(10):	225.2349553	27.25	2
S(6): 0.1; S(9): 99.9; S(13): 100.0	240.4541169	30.1	2
S(9): 100.0; T(21): 0.0; S(24): 0.0; T(	193.8489192	5.89	4
S(4): 100.0; S(10): 0.0; S(11): 0.0	276.3066057	53.82	3
Y(2): 0.1; S(3): 0.1; S(4): 0.1; S(6): 2.	190.4626919	9.59	3
S(12): 0.0; S(15): 100.0; S(17): 0.0	215.0164721	25.2	4

Information Classification: General

S(3): 100.0; T(4): 0.0; S(12): 0.0; S(1	307.0951704	68.33	3
S(1): 0.0; S(4): 0.0; S(7): 100.0; S(9):	409.4867081	48.62	2
S(9): 50.0; S(12): 50.0; Y(19): 0.0	237.9339517	33.14	3
S(1): 0.0; S(9): 100.0; S(16): 0.0; T(1	307.1611244	28.15	3
S(3): 100.0; T(10): 0.0	258.139376	33.11	2
S(3): 0.0; S(5): 32.9; T(6): 32.9; S(7):	86.01104804	24.65	3
S(1): 0.0; T(2): 0.0; S(3): 100.0; S(7):	296.9549578	56.24	2
T(2): 0.0; S(3): 0.0; S(6): 0.0; S(8): 10	168.4864397	29.36	2
S(2): 0.0; S(3): 0.0; S(9): 100.0; S(11	261.2339685	70	2
S(5): 99.8; S(6): 0.1; S(7): 0.1	138.9949394	12.6	2
T(1): 0.0; Y(4): 0.0; T(7): 0.0; S(11): 4	175.7129127	20.03	4
S(1): 0.0; S(8): 100.0; Y(15): 0.0; T(1	387.4936896	89.9	2
S(11): 98.1; S(13): 1.9	376.2629244	51.97	3
T(1): 0.0; S(4): 100.0	281.8329688	18.69	2
S(1): 0.0; S(7): 100.0; S(9): 0.0; S(10	171.832171	26.08	3
S(3): 50.0; S(6): 50.0; S(11): 0.0	246.8186613	65.8	2
S(3): 100.0	153.8284361	25.25	2
T(4): 94.7; S(5): 5.3; T(10): 100.0; S(	66.57103634	1.56	6
S(6): 100.0; S(9): 0.0; T(11): 0.0	375.2157415	68.14	2
T(3): 0.0; T(6): 0.0; S(7): 0.0; T(10): 0	176.3707096	36.69	2
S(3): 100.0; S(5): 0.0; S(7): 100.0	223.2686181	27.54	2
S(4): 100.0; S(6): 100.0; S(12): 0.0; T	339.3990421	63.15	3
S(4): 100.0; S(9): 0.0; S(10): 0.0; S(1	293.1774314	34.95	3
S(4): 100.0	285.2121136	22	3
S(1): 0.0; S(7): 100.0; T(10): 0.0; S(1	388.2533344	36.54	3
S(3): 100.0; S(5): 0.0	174.2764122	15.64	3
S(9): 100.0	230.663667	24.03	2
S(3): 100.0	537.5602114	52.6	3
T(10): 33.3; S(11): 33.3; S(12): 33.3	302.9736969	53.67	2
S(3): 0.0; S(7): 100.0; S(17): 0.0	178.6881973	28.18	3
S(2): 0.0; S(9): 100.0	330.9220886	40.19	3
S(1): 0.0; S(7): 100.0	306.1855307	27.98	2
S(1): 5.3; S(9): 94.7; T(13): 0.0; S(17	82.90986875	11.13	3
T(3): 6.2; S(5): 87.5; T(11): 6.2; S(14)	191.2584693	38.96	4
S(3): 100.0	347.2692213	56.47	2
S(5): 32.5; S(6): 32.5; S(7): 32.5; S(1	63.90228989	20.97	3
T(6): 1.8; S(9): 98.2	161.4623376	23.37	2
T(4): 0.0; S(8): 100.0; S(17): 0.0	277.2396771	20.93	3
S(1): 0.0; S(4): 100.0; S(5): 0.0; T(10	276.5886452	60.5	2
S(3): 100.0; S(5): 100.0; T(8): 0.0; T(	270.4366326	53.3	2
S(1): 0.0; S(3): 0.0; S(5): 0.0; S(7): 0.	428.8931693	88.73	2
S(10): 94.3; S(13): 61.1; S(14): 61.1;	77.52582339	1.47	3
S(3): 100.0	159.341864	26.29	2
S(5): 100.0	152.9072333	6.6	3
S(9): 100.0; S(12): 99.9; S(13): 0.1; S	305.2154311	80.68	2
T(2): 2.8; S(3): 97.2; S(17): 0.0	216.030874	31.77	3
S(1): 2.2; S(2): 97.8; S(4): 0.1; S(5): 0	196.8970458	45.47	2
S(1): 0.0; T(2): 0.0; S(6): 100.0; S(10	239.7582695	28.32	3
S(1): 0.0; S(6): 100.0; T(12): 0.0; S(1	326.7717778	59.24	3
S(6): 2.5; T(8): 97.5; T(14): 0.0; Y(20)	369.4889051	70.01	3
S(1): 0.0; S(3): 100.0; S(10): 0.0; S(1	373.5098235	75.14	2
S(7): 100.0; T(10): 0.0	290.5142643	77.2	2
S(1): 0.0; S(3): 2.1; Y(4): 2.1; S(5): 95	127.3626541	19.71	2
S(1): 0.0; S(7): 100.0; S(13): 100.0	401.1045764	44.51	2
T(8): 0.0; S(10): 100.0	350.2120833	79.17	2

Information Classification: General

S(4): 100.0; S(6): 100.0		240.5650148	13.78	2
S(1): 0.0; S(6): 0.0; S(11): 9.7; S(17):		165.3619368	35.92	3
S(6): 100.0; S(10): 0.0		281.2234473	28.37	3
T(1): 0.0; S(2): 0.0; T(5): 0.0; S(6): 2.		153.7520442	17.93	2
S(5): 100.0; S(7): 0.0; T(13): 0.0; S(2		291.1044644	57.78	3
S(5): 1.7; S(9): 87.3; S(10): 87.3; S(1		96.47265787	8.71	3
S(1): 0.0; S(2): 0.0; S(3): 100.0; S(5):		224.5005802	64.2	2
S(1): 0.0; S(6): 0.0; S(10): 100.0; S(1		259.1616536	54.93	3
T(5): 0.0; S(7): 99.9; S(9): 0.1; S(11):		346.2635844	50.17	3
S(1): 0.0; S(3): 0.0; T(8): 0.0; S(10): 5		361.2489888	56.2	2
S(3): 98.2; S(4): 1.8; S(12): 0.0		324.8035758	66.21	2
S(3): 100.0		153.781808	16.6	3
S(4): 0.3; S(5): 0.3; S(6): 0.3; S(10): 9		196.0018125	24.35	2
S(1): 100.0; T(4): 0.0; S(7): 100.0		173.6734451	11.51	3
T(2): 0.0; T(7): 0.0; T(10): 0.0; S(12):		436.4864277	95.96	2
S(3): 50.0; S(4): 50.0; S(10): 0.0; T(1		148.9249728	13.24	3
T(4): 100.0; S(6): 100.0		373.1908512	38.2	3
S(10): 100.0; S(13): 92.2; T(14): 7.8;		223.1460031	21.04	3
S(3): 0.0; S(4): 100.0; S(14): 0.0		385.9147543	88.48	2
T(1): 0.0; T(7): 0.1; S(9): 49.9; S(11):		224.6887319	70.94	3
S(1): 0.0; S(10): 100.0		176.5737229	20.32	3
S(5): 2.3; S(7): 0.0; S(13): 97.7		167.6767935	25.78	4
S(1): 0.1; S(5): 99.9; S(11): 0.0		274.9184292	46.99	3
S(5): 100.0		176.5631861	26.46	2
T(2): 0.0; S(6): 98.6; T(8): 50.7; S(10)		225.0621883	25.22	2
S(2): 0.0; S(4): 100.0; S(6): 0.0		211.8495414	26.32	2
S(3): 100.0		267.8509933	37.52	2
S(3): 100.0; T(7): 0.0		290.0022719	59.74	3
S(3): 100.0; S(4): 0.0; S(7): 0.0		214.683901	26.74	3
T(9): 0.0; T(14): 1.9; T(15): 98.1		462.4100343	128.91	2
T(1): 0.0; T(9): 100.0; S(13): 100.0		415.2219589	33.09	2
S(3): 0.0; S(8): 0.0; S(9): 0.0; T(11): 0		291.116269	86.52	3
S(2): 96.8; S(4): 3.2; Y(6): 3.2; S(9): 9		133.3358501	14.61	2
S(1): 0.0; T(9): 0.0; S(11): 100.0; S(1		452.9546431	52.77	3
T(1): 33.3; T(2): 33.3; S(3): 33.3; T(9)		196.4293334	11.57	3
S(7): 100.0		108.8239701	23.55	3
T(4): 0.0; T(7): 100.0; S(11): 0.0		242.147207	40.81	2
T(1): 0.2; T(4): 91.3; S(5): 4.3; T(6): 4		161.7663495	30.15	3
S(2): 100.0; S(6): 100.0; T(8): 0.0; T(		304.9881321	70.21	3
S(20): 92.4; T(22): 23.8; T(25): 27.9;		160.2944718	39	4
T(4): 0.0; S(12): 100.0		366.055846	95.52	2
S(1): 50.0; S(3): 50.0; T(9): 0.0; T(14)		91.77737371	4.07	3
S(6): 50.0; S(7): 50.0; S(10): 0.0; T(1		243.1390071	54.6	2
S(10): 100.0; S(16): 0.0		401.5289724	59.25	3
S(1): 0.0; S(3): 98.8; T(4): 1.2; S(5): 0		553.4083965	90.49	2
T(3): 0.0; S(8): 100.0		253.4940967	46.65	3
S(3): 50.0; S(4): 50.0; S(16): 0.0		136.3264964	27.36	3
Y(11): 0.0; S(17): 100.0		368.3809962	61.12	2
T(3): 100.0; S(7): 100.0		232.4520689	24.88	3
S(8): 0.0; T(9): 1.2; S(11): 98.8		373.5715715	87.54	2
S(4): 0.1; S(7): 99.9		216.2664592	32.56	4
S(1): 0.0; S(4): 0.0; S(5): 0.0; S(7): 0.		245.778421	103.55	3
T(2): 0.0; S(7): 1.4; S(9): 98.6		228.3797385	40.3	2
S(4): 100.0; S(6): 100.0; T(12): 0.0; T		262.9193718	46.16	3
S(5): 100.0; S(9): 0.0; S(11): 0.0		213.0388341	44.46	3

Information Classification: General

S(3): 100.0; S(4): 100.0; S(7): 0.0		231.9530269	22.81	3
S(6): 32.6; S(7): 32.6; Y(22): 32.6; S(		83.35502156	4.56	3
S(7): 100.0		465.4790359	101.3	2
S(1): 100.0; S(9): 100.0		450.0872445	34.68	2
Y(1): 0.0; S(4): 0.0; S(11): 0.0; S(21):		148.5822409	12.04	3
S(11): 50.0; S(13): 50.0		292.5635142	20.75	3
S(5): 100.0; S(9): 0.0		239.230986	34.48	2
T(5): 0.1; T(7): 2.8; S(8): 97.1; S(12):		214.760521	25.61	3
S(5): 0.0; T(7): 0.0; T(8): 0.0; T(14): 2		330.2492392	70.69	2
S(3): 0.0; S(5): 0.0; S(9): 100.0		259.9977253	24.58	3
S(3): 100.0; S(5): 0.0		199.0363602	11.6	2
S(3): 32.7; S(6): 1.9; S(9): 32.7; T(11		129.1444327	19.79	3
S(4): 100.0		284.3827608	51.38	2
T(2): 0.0; T(3): 0.0; T(7): 0.0; S(10): 0		302.9845509	82.85	3
S(5): 2.5; S(6): 0.1; S(8): 97.5		111.4169781	14.86	2
S(3): 49.9; T(4): 49.9; T(7): 0.1; S(13		125.0828827	36.16	3
S(10): 100.0; S(19): 0.0; T(25): 0.0; T		331.4712936	34.44	3
S(1): 0.0; S(4): 0.0; S(5): 0.0; T(6): 0.		346.1662657	72.7	2
S(3): 0.0; S(19): 0.0; S(31): 33.3; T(3		198.9855811	18.87	5
S(2): 0.0; S(13): 100.0		304.3265075	25.55	3
S(5): 100.0		252.2746747	46.63	2
S(1): 0.0; S(2): 0.0; T(6): 0.0; S(9): 1.		350.3894049	78.21	2
S(9): 100.0		452.1441339	63.11	3
S(1): 100.0; Y(18): 0.0		426.3043013	64.31	3
Y(2): 0.0; S(9): 100.0		417.191701	77.17	2
T(2): 0.0; Y(5): 0.0; T(10): 0.0; S(12):		302.012686	63.41	3
S(7): 0.0; S(12): 0.3; S(13): 0.3; S(15		120.0631698	17.82	3
T(2): 0.0; T(7): 3.5; T(8): 96.5; T(11):		168.4962395	29.34	4
S(1): 100.0; T(4): 0.0; S(5): 0.0; Y(7):		378.7946337	73.91	2
S(3): 0.0; S(5): 0.0; T(13): 100.0; S(2		338.0948997	87.48	3
T(1): 0.0; S(5): 100.0; S(10): 0.0; T(1		331.7740175	110.31	2
Y(6): 0.0; S(11): 48.4; S(12): 48.4; T(		247.7069679	68.64	3
S(2): 100.0; T(5): 0.0		178.4916282	35.02	2
T(1): 0.0; S(2): 0.0; S(4): 0.0; S(7): 10		340.7407046	91.04	2
S(19): 50.0; S(20): 50.0; Y(25): 0.0		369.3471306	58.42	3
S(2): 0.0; S(3): 100.0; S(5): 0.0; S(8):		203.7659899	66.29	2
T(1): 0.3; S(3): 4.6; T(4): 4.6; S(5): 4.		71.94534734	4.12	2
S(12): 100.0; S(17): 0.0; S(20): 0.0		233.1525602	25.3	2
T(1): 8.4; S(3): 91.5; S(5): 97.0; S(6):		78.87221032	3.09	3
S(11): 97.4; T(15): 2.6		199.8092807	25.59	3
S(1): 0.0; S(3): 100.0; S(6): 0.0; T(7):		160.8769747	10.07	2
S(7): 100.0		286.2866329	54.99	2
S(3): 99.9; S(5): 0.1; S(7): 0.0; S(8): 0		176.8923296	26.32	3
S(4): 100.0; S(7): 100.0; S(12): 0.0; S		96.68706557	15.64	3
T(4): 100.0		146.5656493	27.81	2
S(6): 100.0; T(8): 0.0		293.3447557	38.88	2
S(3): 100.0		164.2849648	15.34	2
T(10): 0.0; T(15): 50.0; S(16): 50.0		225.8114502	19.03	3
S(3): 100.0; S(8): 0.0		232.5838835	34.16	3
T(1): 0.0; S(5): 100.0; S(8): 0.0; S(9):		270.5286338	63.23	3
S(1): 0.0; S(3): 0.0; S(6): 98.2; T(8): 1		197.7725713	24.19	2
Y(1): 0.1; T(3): 0.1; S(6): 99.9; T(9): 9		87.27136349	20.33	3
S(3): 0.0; S(4): 0.0; S(7): 0.1; T(9): 97		209.7575035	68.06	2
S(2): 0.1; T(3): 35.1; S(4): 35.1; S(5):		105.1522765	10.69	3
S(4): 100.0; S(6): 0.0		188.6753858	25.83	2

Information Classification: General

S(2): 0.0; S(3): 0.0; S(5): 99.9; T(6): (	313.1942266	66.74	2
Y(4): 0.0; T(9): 100.0; S(13): 50.0; S(	257.0219582	37.63	3
S(2): 50.0; S(3): 50.0; S(7): 100.0	157.7460657	48.34	2
S(1): 0.0; S(7): 100.0	221.0190451	33.57	2
S(2): 50.0; T(3): 50.0; T(5): 0.0; T(14)	288.5431467	90.46	2
S(1): 0.0; Y(3): 0.0; Y(6): 0.0; S(8): 9	207.4738379	31.01	2
S(2): 3.4; S(3): 96.6; S(7): 96.6; S(9):	169.4621518	28.65	2
S(10): 100.0	223.1146271	13.36	3
S(5): 50.0; S(7): 50.0	77.65516948	15.46	3
S(1): 100.0; S(10): 0.0; T(16): 0.0; T(	205.2617845	45.86	3
S(2): 96.0; S(6): 4.0; S(14): 0.0; S(18	131.8692475	47.02	3
S(7): 0.0; S(21): 100.0	300.8218756	52.37	3
S(1): 0.1; S(3): 99.9; S(10): 0.0	134.6230383	35.89	2
T(2): 0.0; S(3): 0.0; S(6): 0.0; S(9): 0.	140.464287	10.69	3
S(2): 100.0; S(5): 100.0	190.735315	27.9	2
S(3): 100.0; S(7): 0.0	184.8619236	54.77	2
S(4): 0.0; S(9): 100.0	540.1026099	57.9	2
S(11): 0.0; S(12): 0.0; S(13): 0.0; S(1	405.5606336	97.42	2
S(3): 100.0; S(8): 0.0	162.2779743	13.29	2
S(1): 0.0; S(4): 100.0; T(5): 0.0; S(6):	331.9852047	50.8	2
S(3): 100.0	314.1552971	29.98	4
S(2): 0.0; S(6): 100.0; S(8): 0.0	312.0992835	44.62	2
S(6): 100.0	154.4011192	26.82	3
S(1): 0.0; S(3): 95.5; S(4): 2.2; S(6): 2	133.7024091	19.8	2
Y(1): 0.0; T(3): 50.0; S(4): 50.0	204.8599955	17.24	3
S(1): 0.0; S(2): 0.0; S(3): 100.0; S(5):	219.5514376	29.71	2
T(1): 0.0; T(2): 0.0; Y(6): 0.0; T(8): 10	176.8352025	28.76	2
T(1): 0.0; T(4): 100.0	263.0588696	51.7	2
S(6): 100.0; S(9): 0.0; T(11): 0.0; T(1	426.0606947	135.61	2
S(4): 0.0; S(7): 100.0	200.0579527	18.21	3
S(2): 3.0; S(4): 96.9; S(6): 0.1; S(9): (	273.3145754	38.31	3
T(11): 4.3; S(13): 4.3; T(14): 4.3; S(1	131.4954848	1.94	3
T(9): 0.0; T(19): 100.0	203.2617689	47.72	3
S(1): 0.0; Y(3): 0.0; S(4): 0.0; S(10): (	225.3412187	28.79	3
T(9): 0.2; Y(11): 3.7; S(12): 96.1	231.1646424	24.51	4
Y(1): 0.0; S(8): 100.0	221.851819	58.26	2
S(4): 0.0; S(9): 0.0; S(12): 100.0	309.7380685	37.48	3
S(8): 100.0; T(10): 0.0; Y(12): 0.0	205.5334687	102.89	2
T(3): 50.8; T(4): 50.8; S(6): 98.4; T(9)	145.4794779	40.19	2
S(4): 100.0	179.9342362	24.24	3
S(1): 0.0; S(3): 100.0; S(13): 0.0	278.4408234	27.46	3
T(2): 0.0; S(3): 0.0; S(5): 0.0; S(6): 0.	253.2524752	81.43	2
S(2): 66.6; S(3): 66.6; S(7): 66.6; S(9	87.97969769	14.83	2
S(5): 0.0; S(11): 100.0	212.751974	32.41	2
S(6): 100.0; T(10): 0.0	205.4041048	37	2
S(22): 100.0	419.8917704	51.39	4
S(3): 0.0; S(10): 98.0; S(15): 2.0	244.037854	59.17	3
S(4): 0.0; S(12): 0.0; T(13): 0.0; T(15	362.5821615	64.52	3
S(2): 0.0; T(3): 1.6; S(5): 98.4; S(7): (	312.3059555	79.51	2
S(2): 0.0; S(5): 0.0; T(11): 0.0; S(16):	268.0201523	37.55	3
S(1): 0.0; S(3): 100.0	196.3816824	28.14	2
T(2): 95.6; S(6): 4.4; S(8): 0.0	134.5912763	29.64	3
S(2): 0.0; Y(3): 0.0; S(10): 100.0	175.5749267	41.67	2
S(5): 100.0; S(9): 0.0	221.6791988	47.13	2
S(5): 100.0; S(14): 0.0; S(15): 0.0	196.3417856	14.3	3

Information Classification: General

S(1): 0.0; S(3): 100.0; S(6): 0.0		207.5703537	44.79	2
S(2): 100.0; S(7): 0.0		214.683901	46.3	2
T(3): 0.0; S(10): 0.0; S(12): 100.0		218.8777761	39.31	3
T(3): 100.0		244.6380259	53.84	2
S(1): 0.0; T(3): 1.5; S(5): 98.5		288.9937579	59.86	2
Y(3): 0.0; Y(7): 98.9; T(9): 1.1		170.7392594	37.41	3
S(4): 0.0; S(7): 0.0; S(13): 0.1; S(17):		296.8311597	66.04	2
T(5): 100.0		172.6430532	38.7	2
S(3): 11.1; S(6): 94.4; S(9): 94.4		54.78399597	9.97	2
S(7): 0.0; S(14): 100.0		263.3904901	30.02	2
S(10): 50.0; S(12): 50.0		145.546433	15.54	3
S(1): 100.0; S(2): 0.0; S(4): 0.0		282.227307	35.62	2
S(1): 0.0; S(3): 98.1; S(4): 1.9; S(8): (		207.0793179	57.93	2
S(6): 0.0; T(13): 0.0; S(14): 0.2; T(20)		179.3524226	30.96	4
S(6): 0.0; T(8): 0.2; S(10): 95.5; T(12)		147.3428145	48.11	2
S(5): 100.0; T(7): 0.0		213.9793057	36.02	2
T(3): 0.0; S(5): 100.0; Y(6): 0.0; T(7):		463.2738089	65.5	2
S(13): 100.0; S(22): 0.0		228.9741243	33.77	4
Y(1): 0.0; S(2): 100.0; S(5): 0.0		230.835372	18.56	2
S(9): 0.0; S(12): 100.0		366.8080521	33.91	2
Y(1): 2.6; S(2): 97.4; S(4): 0.0; S(7): 1		308.5784667	38.37	3
S(7): 100.0		341.9177151	46.04	2
T(13): 0.0; S(19): 100.0; S(23): 99.8;		410.6712776	90.14	3
S(2): 100.0; S(7): 0.0; T(11): 0.0		360.7472912	54.73	2
S(6): 1.5; S(9): 98.5		340.9980996	55.04	2
S(4): 0.0; S(5): 0.0; T(7): 0.0; S(10): (		284.5940973	33.84	4
S(10): 99.7; T(19): 0.3		120.5496176	1.93	3
T(1): 0.0; S(9): 100.0		325.1973199	50.37	2
S(3): 2.2; S(4): 95.7; S(5): 2.2; T(7): (		121.2337866	22.9	2
S(3): 100.0; S(8): 0.0; S(10): 0.0; T(2		272.5602387	46.72	3
Y(1): 0.0; S(2): 0.0; T(4): 0.1; T(5): 3.		260.0412527	52.72	3
T(1): 0.0; T(2): 0.0; Y(4): 0.0; S(6): 10		255.2348301	31.76	2
S(1): 4.9; Y(4): 0.0; T(8): 94.8; S(12):		171.622102	51.06	4
S(3): 2.8; T(6): 47.2; S(12): 2.8; S(16		122.1942401	44.26	3
S(3): 100.0; T(7): 0.0; T(12): 0.0		232.3020729	39.54	2
S(11): 100.0; S(17): 100.0; T(21): 0.0		463.7415343	68.45	3
S(1): 0.0; S(4): 96.4; T(5): 5.3; S(6): 4		117.0283577	14.91	2
S(3): 100.0; S(4): 100.0; T(17): 0.0		455.8740474	102.13	3
S(1): 2.0; S(3): 98.0; S(9): 0.0; T(11):		232.0607706	32.88	2
T(2): 0.3; S(5): 99.7; S(7): 100.0; S(1		144.712441	9.23	3
T(8): 100.0		201.1249823	12.77	3
T(6): 100.0; T(8): 0.0		249.7876053	62.23	2
S(1): 2.6; S(3): 97.4; T(6): 100.0; S(1		328.1991669	41.48	2
T(1): 100.0; Y(7): 0.0; S(8): 0.0; T(12)		200.652727	45.34	2
T(2): 0.0; S(10): 95.4; S(12): 4.6; S(1		140.6046317	5.78	3
S(8): 100.0		393.4556365	33.92	2
S(1): 100.0		204.8185974	22.01	3
S(3): 100.0; T(6): 0.0; S(12): 0.0		119.4210951	19.25	3
S(5): 0.0; S(12): 99.7; S(16): 0.1; S(1		295.2743592	89.57	3
T(6): 0.0; S(11): 0.2; T(15): 4.0; S(18)		117.3991703	7.59	3
T(11): 4.5; S(13): 65.2; T(14): 65.2; S		267.1838945	53.88	3
S(3): 100.0; S(12): 0.0; T(14): 0.0; T(		334.8351813	48.23	3
S(2): 0.0; Y(4): 0.0; S(7): 100.0; S(12		271.2238408	35.77	2
S(3): 100.0		326.0219782	49.71	2
S(3): 100.0; S(7): 100.0; S(13): 0.0; S		427.8694426	86.68	3

T(4): 0.0; S(11): 97.8; Y(12): 2.2		208.6154223	36	3
S(3): 100.0; S(6): 0.0; S(7): 0.0		241.8920289	39.75	2
S(2): 99.7; T(4): 0.1; S(5): 0.1; T(6): 0		180.3097069	12.89	3
S(1): 100.0		158.5387059	29.02	2
S(2): 0.0; S(4): 100.0		157.1969734	12.89	2
S(2): 0.0; T(3): 0.0; S(4): 0.0; S(7): 10		235.0582325	53.24	2
S(3): 0.0; S(4): 0.0; S(7): 0.0; T(14): 1		379.1566321	81.73	3
S(2): 0.0; S(10): 100.0; S(13): 0.0; S(		322.3277637	59.46	2
S(5): 100.0; S(7): 100.0; Y(15): 0.0; S		173.3067164	22.4	4
S(3): 100.0		157.3794371	18.29	2
S(2): 100.0; S(4): 100.0; T(8): 0.0; S(		272.4344784	27.26	3
S(7): 50.0; S(9): 50.0		160.8312285	11.66	3
S(1): 100.0		193.1743605	23.6	2
S(1): 96.7; S(4): 3.2; S(6): 0.1; S(9): 0		284.3294015	55.41	3
S(8): 0.0; S(13): 100.0; S(17): 100.0		345.5785493	43.1	4
S(2): 0.0; Y(3): 0.0; S(5): 100.0; Y(13		319.0981152	37.54	2
S(1): 0.0; T(4): 0.0; S(11): 92.8; Y(13		237.4887846	45.82	3
S(3): 100.0		111.0406724	22.27	2
Y(3): 0.0; S(5): 0.0; S(10): 99.9; S(12		266.5321313	44.05	3
S(1): 0.0; S(3): 0.0; S(6): 100.0; T(10		219.0996709	37.79	3
S(9): 50.0; S(10): 50.0		288.6856908	37.98	4
S(9): 100.0; S(16): 0.0		376.5633724	65.54	2
T(8): 1.1; S(10): 33.0; S(11): 33.0; Y(		101.8871815	13.56	2
T(1): 0.0; T(8): 0.0; T(13): 0.0; S(16):		325.8378331	103.05	2
S(3): 99.9; T(6): 50.0; S(7): 50.0; T(9)		100.4583315	15.88	2
S(6): 0.0; S(10): 100.0; S(20): 0.0; S(		207.0090124	45.94	3
S(2): 100.0		251.3106559	46.79	2
S(5): 0.0; S(6): 0.0; S(9): 100.0		221.3886921	11	3
S(3): 100.0; S(5): 99.9; S(8): 0.1		148.5286197	11.52	2
S(3): 49.4; S(4): 1.3; S(7): 49.4		144.1802443	15.25	3
S(4): 100.0; S(9): 0.0		144.416688	10.86	3
S(1): 100.0		252.2009787	31.99	2
S(4): 100.0; S(6): 100.0; S(9): 0.0		233.6010918	28.76	2
S(3): 100.0; T(17): 0.0; S(20): 0.0; T(		242.8916052	24.62	3
T(3): 1.9; S(8): 98.1; S(12): 0.0; S(14		319.3913909	44.55	2
T(1): 33.3; T(2): 33.3; S(3): 33.3; S(9)		132.3797699	12.38	3
S(1): 100.0; T(10): 0.0; T(13): 0.0; S(		153.108613	5.21	5
T(1): 0.0; S(7): 100.0		183.2009807	18.06	3
S(6): 0.0; T(8): 100.0; T(10): 0.0		328.2475213	48.61	2
S(1): 100.0		239.4632177	29.71	2
T(9): 2.2; S(11): 97.8		175.2562657	21.08	2
T(5): 0.0; S(8): 100.0; S(9): 100.0; T(		440.5394713	130.55	2
S(3): 0.0; T(5): 0.1; Y(8): 50.0; S(13):		152.0385655	9.67	4
S(1): 100.0; S(4): 0.0; T(12): 0.0; S(1		240.676523	54.7	2
T(6): 99.9; S(11): 50.1; T(15): 50.1		126.8614547	19.31	2
S(10): 100.0; Y(13): 0.0		235.7640876	12.72	3
S(3): 0.0; S(4): 0.0; T(7): 0.0; T(12): 0		174.9772786	23.77	3
S(3): 1.6; S(4): 98.4		191.1857178	26.18	3
S(3): 100.0		110.5847437	7.12	3
S(3): 100.0		200.6317972	23.25	2
S(6): 0.0; S(12): 100.0		224.1628601	19.59	3
S(3): 0.0; S(7): 100.0; T(11): 0.0; S(1		194.928546	26.43	3
S(1): 100.0; Y(2): 0.0; T(5): 0.0; T(7):		197.8437993	24.62	2
S(2): 0.0; S(4): 0.0; S(7): 100.0		204.8185974	43.89	2
T(1): 0.0; S(2): 0.0; S(7): 100.0; S(14		332.4987161	60.3	3

Information Classification: General

S(3): 100.0; S(6): 0.0; S(9): 0.0; T(10	180.4255765	17.86	3
S(2): 0.0; S(5): 99.9; S(7): 0.1; S(8): 0	123.6613719	20.56	2
S(13): 100.0	472.9634382	102.21	2
S(2): 100.0	194.3069055	20.84	2
S(2): 100.0	106.0865626	16.63	2
S(4): 0.0; S(12): 100.0	218.181571	63.13	2
S(2): 100.0; T(4): 100.0	166.6371829	16.6	3
S(1): 100.0; S(5): 0.0; Y(10): 0.0	191.4418793	31.31	3
S(9): 97.2; S(12): 49.3; S(13): 50.6; S	197.6640012	41.91	3
S(3): 0.0; S(7): 100.0; S(10): 0.0; T(1	191.135786	32.5	3
S(2): 0.0; T(3): 0.3; T(6): 94.5; S(7): 4	134.7332713	14.68	3
S(3): 100.0; S(4): 100.0; S(14): 0.0	301.7897827	34.96	3
S(5): 100.0	373.5240619	49.27	3
S(7): 100.0	421.7016001	93.42	2
S(3): 100.0	188.1987888	15.27	3
S(7): 0.0; S(15): 100.0; S(19): 0.0	518.6470703	88.03	2
S(19): 100.0; S(20): 100.0; Y(25): 0.0	396.1730716	74.75	3
S(4): 0.0; S(6): 0.0; S(7): 99.9	171.5392503	34.74	2
S(1): 0.2; S(3): 48.3; T(5): 48.3; S(8):	124.217542	22.73	3
S(6): 51.0; S(8): 51.0; S(11): 97.9; S(	176.4193843	12.89	3
T(8): 0.0; S(9): 0.0; S(16): 49.1; S(20	161.8533029	31.16	3
S(4): 99.8; T(8): 0.2; S(12): 0.0	204.9462789	26.1	3
S(1): 100.0; T(7): 0.0; S(8): 0.0; S(9):	339.1377622	75.72	2
S(4): 3.2; S(6): 0.1; S(7): 96.7	90.9681782	13.55	3
S(1): 0.0; T(2): 2.1; S(3): 97.9; S(6): 0	166.3641875	43.72	2
S(1): 0.0; S(2): 50.0; S(3): 50.0; S(6):	171.5392503	25.9	2
Too many isoforms		6.55	4
S(5): 100.0; S(8): 0.0	142.6288476	10.66	2
S(7): 100.0	385.8920222	79.08	2
S(3): 100.0; S(6): 0.0; T(9): 0.0	551.0756333	130.28	2
T(1): 0.0; Y(4): 0.0; S(11): 99.7; S(12	155.8746387	34.22	3
T(1): 0.0; S(3): 0.0; T(4): 0.0; Y(5): 0.	294.9134462	40.01	3
S(4): 0.0; S(8): 100.0; S(13): 0.0	170.2889617	12.36	3
S(1): 100.0; S(3): 0.0; T(4): 0.0; S(6):	134.4096187	36.13	2
Y(13): 0.0; S(16): 100.0	289.6983067	46.76	3
S(5): 100.0; S(6): 100.0	114.1750419	14.2	3
T(1): 0.0; S(3): 100.0; T(4): 0.0; S(5):	221.0446213	22.75	2
T(1): 0.0; S(2): 0.0; T(3): 0.0; Y(4): 0.	253.2888318	27.43	3
T(1): 2.1; S(5): 97.8; S(8): 0.1; S(10):	118.0003579	24.85	2
S(3): 1.6; T(5): 98.4; S(8): 0.0; S(9): 0	230.9022088	52.21	2
T(1): 0.0; S(13): 100.0	485.0316806	69.23	3
S(18): 89.4; S(19): 89.4; S(22): 87.5;	107.9313673	11.31	4
S(3): 0.0; S(4): 100.0	181.8783958	31.85	2
T(5): 0.0; S(6): 0.0; S(8): 0.0; T(9): 2.	372.5610355	99.59	2
T(6): 97.7; S(10): 2.3	236.1176187	18.41	3
S(6): 0.0; T(9): 0.0; S(11): 100.0	295.1872532	47.48	2
T(2): 0.7; S(3): 24.8; S(5): 24.8; S(6):	119.1653712	32.81	2
T(3): 0.0; S(10): 100.0; T(14): 0.0; T(	170.2362667	37.92	4
S(2): 0.0; S(9): 100.0	232.601941	21.18	2
S(8): 2.7; T(10): 48.7; T(15): 48.7; T(	158.3347373	25.4	3
T(4): 100.0	281.1408261	28.26	2
S(5): 100.0	168.4161477	12.36	3
T(1): 0.0; T(4): 100.0; S(8): 0.0; S(12	315.9722293	38.44	2
S(9): 100.0; S(12): 0.0; T(13): 0.0; T(	310.1168508	36.86	3
Y(4): 0.2; T(6): 95.9; S(9): 3.9; S(13):	274.5491764	77.32	3

Information Classification: General

S(4): 100.0		577.4964991	65.63	2
S(3): 100.0; T(5): 0.0; T(7): 0.0; T(8):		193.5161027	54.18	2
S(10): 100.0; S(14): 0.0		388.1777368	34.32	3
T(6): 0.0; S(7): 0.0; S(15): 0.0; S(19):		212.6256451	52.49	3
S(8): 48.3; S(10): 3.5; S(11): 48.3		51.22085509	11.17	3
S(7): 96.7; S(8): 3.1; S(10): 0.1; T(11		178.1250198	27.7	3
S(3): 0.0; S(9): 97.4; T(13): 2.6		175.9626026	21.83	2
S(2): 0.0; S(12): 0.1; S(17): 33.4; T(1		196.5774274	58.77	3
S(3): 100.0; T(8): 0.0; T(11): 0.0		158.7493978	10.01	2
T(1): 0.0; S(3): 0.0; T(5): 0.0; S(6): 0.		325.6364534	68.59	2
T(1): 0.0; S(3): 0.0; T(5): 0.0; S(6): 0.		467.2720107	82.95	2
S(1): 100.0; S(10): 0.0		272.107968	28.91	2
T(4): 0.0; S(7): 100.0		249.8582091	33.91	2
T(9): 0.0; T(10): 0.0; T(19): 50.0; S(2		206.8554439	29.78	4
S(3): 0.0; T(5): 0.0; S(10): 0.0; T(11):		252.4690935	53.2	3
S(2): 100.0		167.9846494	17.8	2
T(3): 0.0; S(4): 0.0; S(9): 99.9; T(11):		221.1932951	33.42	2
S(2): 100.0		172.3279059	14.66	3
S(5): 100.0; T(11): 0.0; S(12): 0.0; T(		223.2054474	30.85	3
S(3): 100.0; S(9): 0.0; S(11): 0.0; T(1		215.6562674	20.89	3
S(3): 100.0		168.0977257	22.91	2
S(1): 0.0; S(3): 100.0		344.9783463	50.29	2
T(3): 50.0; S(4): 50.0		257.9261627	58.42	2
S(4): 0.0; S(6): 100.0; T(8): 0.0; T(12		429.1878567	125.24	2
S(10): 0.0; T(17): 0.0; S(23): 0.0; S(2		116.7513811	3.09	4
T(3): 100.0; S(7): 0.0; S(9): 0.0; S(19		257.0167904	20.83	4
S(5): 100.0; T(18): 0.0; S(19): 0.0; S(		311.5375413	36.05	3
Y(4): 0.0; Y(5): 0.0; T(9): 98.2; Y(13):		295.4204597	72.26	2
S(1): 0.0; S(3): 0.0; S(4): 0.0; Y(8): 0.		96.19288042	9.29	3
S(6): 100.0		299.1550904	51.31	2
T(1): 0.0; S(5): 0.0; S(10): 2.1; S(11):		203.2283757	41.96	2
S(3): 100.0; S(7): 0.0		361.3437145	61.54	2
S(1): 0.0; S(5): 0.0; S(7): 50.0; S(10):		186.4900561	10.28	3
S(5): 100.0; T(14): 0.0		289.3587711	45.72	3
S(3): 99.7; T(5): 0.3; T(14): 0.0; T(19		172.8584461	17.85	3
S(8): 24.8; S(9): 0.9; S(12): 24.8; S(1		221.3195705	53.36	3
Y(6): 0.0; S(14): 0.0; S(18): 0.1; S(24		262.1543385	25.07	3
T(1): 0.0; T(3): 0.0; T(5): 3.3; T(9): 96		153.0018142	15.12	2
S(2): 1.9; S(4): 98.1; S(8): 0.0; S(9): (		290.2051834	53.63	3
S(4): 100.0; T(11): 0.0		162.9454329	34.04	4
S(9): 100.0; S(14): 100.0		246.0277664	3.71	3
S(5): 99.8; T(6): 0.2; T(9): 0.0; Y(12):		242.9338488	68.92	3
S(1): 0.0; S(2): 0.0; S(4): 0.0; S(9): 0.		388.2974512	99.59	3
S(1): 0.0; T(2): 0.0; S(5): 100.0; S(9):		454.329537	48.98	2
S(10): 100.0; Y(12): 0.0; S(17): 0.0		293.5504777	62.93	3
S(1): 0.0; S(3): 100.0; T(6): 0.0; T(7):		139.9615	25.63	2
S(5): 50.0; S(8): 50.0; T(13): 0.0; S(1		224.0612383	33.08	3
Y(1): 0.0; T(3): 0.0; T(10): 0.0; S(12):		291.7477627	34.19	3
S(14): 100.0		308.077196	25.31	3
S(3): 0.0; T(4): 0.0; S(6): 100.0		181.5042464	49.45	2
S(2): 0.0; T(11): 100.0; T(13): 0.0		236.3412568	19.2	2
S(17): 100.0		441.5083047	58.57	3
S(3): 100.0; Y(6): 0.0		285.3100587	63.07	2
S(7): 100.0; S(12): 0.0		333.9664672	84.86	2
S(9): 50.0; S(11): 50.0		163.3511174	30.78	3

Information Classification: General

S(4): 0.0; S(5): 0.0; S(6): 0.0; S(10): (	425.0000954	79.08	3
S(8): 100.0; Y(14): 0.0	261.9170537	29.03	3
S(1): 0.0; T(4): 0.0; S(9): 100.0	130.7739949	10.62	3
S(4): 2.5; S(6): 95.1; T(8): 2.5; S(16):	233.3680379	22.64	3
T(2): 0.0; S(8): 0.0; S(10): 0.0; S(11):	406.8184317	77.91	2
S(8): 33.3; Y(11): 33.3; T(14): 33.3; S	124.0951617	23.43	3
S(5): 100.0; S(6): 100.0; T(7): 100.0;	226.9632306	53.81	3
T(3): 0.0; S(7): 100.0	377.9502539	50.05	2
S(3): 0.0; S(8): 0.0; T(13): 0.0; S(25):	134.2231387	14.53	3
S(4): 100.0; T(8): 0.0; T(12): 0.0; S(1	272.5390508	87.76	2
S(6): 97.4; Y(7): 2.6	209.4574638	39.21	3
S(3): 0.0; S(4): 0.1; S(6): 2.2; S(7): 97	149.5198022	40.77	2
S(3): 0.0; S(7): 100.0	251.749939	45.81	2
S(11): 100.0; S(14): 100.0; T(19): 0.0	193.5164392	41.95	4
T(1): 0.0; S(3): 0.0; S(12): 100.0; T(3	446.5745623	42.34	4
S(1): 0.0; Y(6): 0.0; S(13): 100.0; T(1	339.3141374	86.03	2
S(3): 0.0; S(8): 2.7; S(10): 97.3; T(14	218.9897775	49.6	3
S(6): 100.0; S(9): 0.0	255.2003766	44.61	2
S(4): 100.0; T(7): 0.0; T(12): 50.0; S(	298.4777851	116.16	3
S(10): 0.0; S(11): 0.0; S(15): 4.3; S(2	173.798729	13.78	3
S(6): 0.0; T(9): 0.0; Y(13): 4.4; S(14):	275.7764672	49.63	4
S(1): 100.0; T(5): 0.0; Y(7): 0.0; S(15	399.4950527	66.62	2
S(1): 0.0; S(3): 100.0; S(5): 0.0	254.089266	58.17	2
S(1): 0.0; T(2): 2.9; S(4): 97.1	146.9866339	2.79	3
S(1): 0.0; S(3): 100.0; S(5): 97.2; T(6	228.3408398	24.8	2
S(2): 0.0; T(4): 50.0; S(5): 50.0; S(11	284.0445441	58.21	3
T(3): 0.0; S(7): 0.0; Y(10): 0.0; S(12):	203.7622205	111.48	3
S(2): 99.9; S(6): 8.2; S(10): 83.9; T(1	90.75694791	0.54	4
S(13): 100.0	374.9356102	37.42	3
S(6): 3.0; S(10): 97.0	157.8994645	25.19	3
S(2): 0.0; S(5): 100.0	245.3668562	36.48	2
T(7): 1.8; S(10): 48.2; T(11): 48.2; S(	176.1057375	28.28	3
S(3): 0.0; S(8): 50.0; S(9): 50.0; T(11	234.7131834	37.41	2
S(7): 99.5; S(11): 6.5; S(13): 82.0; S(	156.1089511	12.51	4
S(1): 0.0; S(3): 0.0; S(8): 0.1; S(10): 9	237.6871316	47.61	3
S(1): 0.0; S(7): 100.0; S(11): 0.0	273.0738056	41.29	2
S(5): 4.3; S(6): 95.7; S(11): 0.0; S(12	118.6870392	2.89	2
T(2): 0.0; S(3): 100.0; T(6): 0.0	160.6711428	26.56	2
S(5): 0.0; S(8): 97.9; T(9): 2.1; Y(12):	353.3215366	62.03	2
S(2): 1.7; S(3): 98.3; S(6): 0.0; Y(9): (	217.0012327	33.34	3
S(7): 100.0	167.5116189	5.59	3
S(11): 0.0; S(14): 99.9; T(18): 0.1	322.4801586	56.31	3
S(5): 0.0; T(10): 0.0; T(13): 0.0; S(16	337.220109	55.71	3
S(8): 100.0	240.6556217	57.06	2
T(5): 0.0; T(7): 0.0; T(10): 0.0; S(12):	267.6159918	45	4
S(1): 0.0; S(4): 2.1; S(5): 97.9; T(10):	142.489574	36.81	2
S(2): 0.0; S(4): 100.0; S(6): 0.0; S(8):	214.0153719	29.59	3
S(5): 100.0; S(10): 0.0	103.8295086	8.41	3
S(3): 100.0; Y(6): 0.0	158.8978753	17.34	3
S(1): 0.0; S(3): 0.1; T(5): 99.9; S(7): 1	208.2763573	17.6	3
T(1): 0.0; T(2): 0.0; T(5): 0.0; S(10): 1	385.8421517	43.39	3
S(3): 100.0; Y(6): 0.0; S(7): 0.0; S(9):	178.0274412	26.1	3
S(7): 100.0; S(9): 0.0	258.2297218	38.18	3
S(1): 0.0; S(11): 100.0	267.5874468	39.46	3
S(5): 100.0	256.1812392	24.34	2

T(14): 100.0		139.3077875	6.44	3
S(5): 3.7; T(6): 96.3; S(8): 0.0; S(9): (		74.44967222	18.57	2
S(6): 96.1; S(8): 3.9		169.4510518	6.74	3
S(1): 0.0; S(7): 100.0		277.4065954	24.22	2
S(1): 0.0; T(2): 0.0; T(7): 100.0; S(10)		213.4486537	30.65	3
S(6): 100.0; T(15): 0.0		156.8320327	17.39	3
S(2): 0.0; S(5): 100.0; S(8): 0.0		236.8679223	32.45	2
S(2): 0.0; T(3): 0.0; S(6): 0.0; S(10): (		237.0976162	47.13	3
S(5): 100.0; T(24): 0.0; S(26): 0.0		117.8830597	13.31	3
S(1): 0.0; S(6): 0.0; S(12): 0.0; S(13):		136.66002	1.76	4
S(2): 2.7; S(3): 2.7; S(5): 92.0; T(6): 2		276.5094491	25.89	3
T(1): 0.0; T(5): 0.2; Y(6): 0.0; S(9): 99		105.4634649	4.27	3
T(1): 0.0; S(5): 0.0; S(8): 100.0; T(12)		208.2127232	56.53	3
T(3): 49.1; S(6): 49.1; T(8): 1.7		119.5296248	12.79	3
S(10): 99.9; S(13): 0.1; T(15): 0.0; S(		216.4380221	66.07	2
S(4): 100.0		167.2496338	32.08	2
S(7): 100.0		269.6240307	41.42	3
Y(4): 0.0; T(9): 100.0; S(13): 0.0; S(1		304.6794043	52.77	3
S(1): 0.0; S(5): 100.0; S(7): 0.0		243.9206989	39.89	2
T(1): 5.4; S(2): 94.6; T(4): 0.0; S(5): (		140.0779051	19.73	3
S(1): 0.0; S(7): 100.0		406.6413458	38.79	2
S(5): 100.0		212.6329305	16.3	3
S(4): 100.0		300.613281	32.91	2
S(5): 0.0; S(7): 0.0; S(11): 100.0; S(1		335.3213915	103.66	2
S(9): 50.0; S(12): 50.0; S(15): 100.0		264.7227049	17.19	3
Y(1): 0.0; T(4): 100.0		340.8857517	68.84	2
T(1): 0.0; T(12): 100.0		122.6509541	5.08	3
S(1): 100.0; T(4): 0.0; S(5): 0.0; Y(7):		275.7785215	77.93	2
S(1): 100.0; T(10): 0.0; S(11): 0.0		297.206234	34.26	3
S(9): 100.0; T(16): 0.0; S(17): 0.0; S(		415.9268872	62.73	3
T(3): 33.3; S(4): 33.3; S(6): 33.3		91.48133028	16.57	2
T(3): 50.0; S(6): 50.0		197.1205459	54.64	2
S(6): 0.0; S(14): 33.3; S(20): 33.3; Y(		249.5250174	39.87	3
T(1): 0.0; S(5): 100.0; Y(8): 0.0		345.862265	65.84	2
S(1): 100.0; S(7): 100.0		396.3021177	37.03	2
T(3): 0.1; S(6): 97.7; S(7): 2.2		132.359579	34.57	3
T(11): 0.0; S(14): 0.0; S(19): 95.1; S(		142.2861417	13.69	3
T(6): 0.0; S(11): 0.0; S(12): 0.0; T(14)		218.8029266	26.15	3
Y(1): 100.0; T(7): 0.0		201.8006884	25.52	2
T(2): 0.1; T(7): 97.2; T(8): 2.7; T(11):		194.9829644	26.06	3
S(11): 100.0		268.674502	71.41	2
S(7): 0.1; S(11): 0.1; S(29): 0.1; T(31)		102.0196912	8.79	3
S(4): 100.0; T(6): 100.0		188.4840258	18.13	3
Y(3): 0.0; S(7): 1.9; S(8): 98.1		159.7252398	41.22	2
S(6): 0.0; T(7): 0.0; S(12): 100.0; T(1		207.626019	36.94	3
T(1): 0.0; S(14): 100.0; S(16): 100.0;		355.5436724	34.1	3
S(3): 0.0; S(6): 100.0		191.1857178	28.76	2
S(2): 100.0; T(21): 0.0		415.3679441	49.62	3
S(2): 0.0; S(12): 0.0; S(17): 0.1; T(19		265.7742427	43.51	3
S(2): 0.0; S(5): 0.0; S(13): 95.5; S(16		137.992384	16.17	3
S(3): 0.0; S(6): 96.4; S(10): 51.8; S(1		151.5152508	13.43	3
S(2): 2.8; Y(3): 48.4; S(4): 48.4; S(5):		195.1618589	30.73	3
S(1): 100.0; T(8): 0.0		343.8659531	60.84	3
S(3): 100.0		185.3482297	8.4	3
Y(1): 3.8; S(5): 96.2		55.39737826	7.42	3

Information Classification: General

S(2): 99.7; T(4): 0.3; T(6): 0.0; T(9): 0	156.8151442	8.88	3
S(3): 0.0; S(5): 100.0; S(11): 0.0; S(1	474.2792439	100.64	2
S(7): 100.0; T(9): 0.0; S(23): 0.0; S(2	310.1407144	53.84	3
S(1): 96.7; S(3): 3.3; S(12): 0.0; S(19	212.1807425	33.04	3
S(7): 100.0	245.0656004	42.91	2
S(7): 0.0; S(8): 0.0; S(11): 0.0; S(12):	459.9944031	70.07	3
S(1): 2.4; S(3): 95.3; T(5): 2.4; S(13):	203.1393548	43.58	3
Y(13): 2.5; S(15): 97.4; T(18): 0.0	287.243982	34.64	3
Y(3): 0.0; S(5): 0.0; S(10): 50.0; S(12	199.8159259	26.89	3
S(3): 100.0; S(6): 0.0	233.3160832	16.39	2
T(4): 0.0; S(6): 100.0	326.168713	31.05	2
T(2): 0.0; S(5): 0.0; S(8): 0.0; T(9): 0.	230.4713099	34.64	4
S(2): 33.4; S(4): 33.4; S(5): 33.4; S(1	138.1684573	19.78	3
Y(5): 0.0; S(15): 100.0	307.7256996	51.92	3
S(5): 100.0	96.02478778	21.72	3
Y(3): 0.1; S(4): 0.1; T(5): 99.8; T(8): 0	199.1790241	35.9	2
S(10): 94.6; S(11): 2.6; Y(14): 2.6; S(	216.8600134	31.38	3
Y(4): 0.0; S(10): 100.0	158.0010446	14.52	2
S(3): 2.2; S(5): 97.8; S(9): 0.0	172.5978828	23.04	2
T(3): 0.0; S(5): 0.0; S(7): 100.0; S(9):	553.6766554	128.05	2
T(1): 0.0; S(2): 0.0; Y(6): 0.0; S(13): 1	307.3517277	58.47	2
S(3): 50.0; S(4): 50.0; S(7): 0.0; S(8):	233.7343579	47.8	2
S(7): 0.0; S(10): 100.0; S(11): 100.0;	392.12592	30.49	2
S(2): 50.0; S(4): 50.0; S(10): 0.0	250.8108914	21.76	3
S(3): 100.0; T(16): 0.1; S(18): 99.9	391.5318148	32.7	3
S(7): 100.0; S(12): 0.0	201.2866557	27.7	3
S(3): 2.1; S(4): 97.9; T(7): 0.0; T(9): 0	259.1579643	47.5	2
S(1): 0.1; S(2): 99.9; S(6): 0.0	126.3905113	25.64	2
S(6): 0.0; T(9): 0.0; Y(13): 0.0; S(14):	391.7263749	77.58	3
T(3): 0.0; T(5): 98.0; S(6): 2.0; T(9): 0	179.4415263	29.62	2
S(9): 100.0	378.242332	25.14	3
S(7): 2.1; S(9): 97.9; T(11): 0.0; T(16	301.7197002	97.88	2
S(2): 0.0; S(4): 0.0; T(6): 100.0; S(8):	236.5207449	36.34	3
S(4): 98.1; S(6): 1.9; S(10): 100.0	278.6858505	59.13	2
S(1): 0.0; S(4): 100.0; Y(9): 0.0	282.6266183	50.53	2
S(1): 0.0; S(2): 100.0; S(4): 0.0; Y(7):	172.037606	25.19	2
S(3): 100.0	107.0041321	17.97	3
Y(3): 0.0; T(6): 50.0; S(7): 50.0; S(11	192.7909477	20	5
S(13): 100.0	284.9700864	39.59	3
S(3): 100.0; S(5): 0.0; T(7): 0.0	346.2400909	44.34	2
S(2): 0.0; S(4): 100.0; S(6): 0.0; S(7):	146.9112616	23.56	2
S(1): 0.0; S(3): 0.0; S(5): 2.2; S(6): 97	139.6931329	30.95	2
S(7): 100.0; S(11): 100.0; Y(15): 0.0	87.35570582	7.36	5
S(2): 100.0; Y(7): 0.0; S(8): 0.0	393.8387617	54.95	2
S(3): 100.0; S(5): 100.0; T(9): 0.0; S(	268.5406245	33.55	3
S(4): 0.0; S(5): 100.0	246.7097353	38.36	2
Y(4): 0.0; T(6): 0.0; S(9): 100.0	171.1148092	19.5	2
T(3): 0.0; T(6): 0.0; S(9): 100.0	209.3706622	51.82	2
Y(4): 0.0; S(10): 100.0	279.5741883	22.78	3
T(1): 96.9; T(2): 3.1; T(5): 0.0; S(10):	287.478744	29.26	3
S(1): 0.0; S(9): 100.0; S(16): 100.0; T	282.1316774	33.59	4
S(1): 0.0; S(9): 100.0	187.0289736	16.46	2
S(1): 0.0; S(3): 100.0; S(8): 0.0	231.183598	50.7	2
S(2): 0.0; S(3): 0.0; S(6): 0.0; T(13): 1	414.0050134	135.05	2
S(11): 100.0; S(15): 0.0; S(17): 0.0; T	452.2696199	52.21	3

S(2): 0.0; S(3): 0.0; S(10): 32.9; T(13	119.286832	15.69	2
S(1): 100.0; Y(5): 0.0; S(7): 0.0; Y(8):	226.5795138	37.81	2
S(10): 100.0	522.9321155	104.03	2
S(4): 0.0; S(9): 100.0	434.404038	96.86	2
S(14): 100.0; S(23): 0.0; T(29): 0.0	289.6148884	107.25	3
S(2): 100.0; S(7): 0.0	154.4011192	18.71	3
S(4): 99.8; T(6): 0.2; S(20): 0.0; S(22	242.9338488	54.94	3
S(5): 0.0; S(7): 0.0; T(12): 0.1; S(15):	172.3434172	17.14	3
S(7): 100.0; S(19): 0.0; T(22): 0.0	272.2366646	50.89	3
S(3): 100.0	218.2983036	30.11	2
T(1): 0.0; T(12): 0.0; S(14): 99.9; S(1	381.1524978	83.41	2
S(5): 50.0; S(9): 50.0	173.9668449	21.69	2
T(1): 0.0; S(5): 100.0	246.6642917	31.03	3
S(1): 0.0; S(14): 0.0; T(15): 0.0; S(22	275.6312994	47.79	3
T(10): 100.0; T(14): 0.0	260.2348353	49	2
Y(5): 0.0; S(8): 100.0	236.3611112	37.12	2
T(6): 0.0; S(14): 100.0; T(23): 0.0; T(	269.4970589	95.14	3
T(5): 0.0; S(7): 100.0; S(10): 0.0; S(1	266.5979099	33.95	3
T(7): 0.0; T(16): 97.2; Y(17): 2.8	259.9645745	15.14	3
S(3): 100.0; S(4): 100.0	408.3285483	61.37	2
S(1): 0.0; S(2): 0.0; S(5): 100.0; S(6):	276.3951836	48.93	2
S(1): 0.0; S(7): 100.0	358.6611659	51.33	2
S(2): 0.0; Y(3): 0.0; S(11): 99.9; T(15	264.3686251	34.52	4
T(1): 0.0; S(3): 0.0; T(5): 0.0; S(6): 0.	304.4304134	63.88	2
Y(3): 0.0; S(9): 100.0; T(16): 0.0	253.4940967	57.94	2
S(3): 100.0; T(6): 0.0; S(7): 0.0; S(11	372.8027624	82.78	2
S(12): 0.0; T(14): 0.0; S(22): 100.0	387.3383496	77.04	3
S(9): 0.0; S(12): 0.0; S(15): 100.0	273.3508245	41.36	3
T(2): 0.0; Y(3): 0.0; S(4): 0.0; T(20): (	449.584324	86.38	3
S(3): 50.0; S(4): 50.0; T(7): 0.0; T(8):	148.2366705	22.8	3
S(7): 0.0; S(10): 0.0; S(12): 2.6; S(13	165.654134	46.02	2
S(1): 0.0; Y(9): 0.0; S(10): 100.0	359.4178997	64.8	2
S(3): 2.1; T(6): 95.7; S(7): 2.1; T(9): (	145.0825633	35.91	2
S(1): 0.0; S(3): 100.0	168.5494681	19.4	2
S(2): 0.0; S(5): 0.0; S(7): 0.0; S(11): 9	183.2032591	26.55	3
Y(2): 0.0; S(3): 100.0; S(7): 0.0; S(8):	166.2116685	19.88	2
Y(4): 0.0; S(6): 50.0; S(7): 50.0; T(16	288.2284117	76.5	3
T(3): 0.1; S(7): 99.9	108.2519972	20.97	2
S(1): 50.0; S(3): 50.0; T(5): 94.8; S(8	49.73480528	8.25	2
S(8): 0.0; T(11): 0.0; S(16): 30.3; T(1	132.5110111	11.49	4
T(1): 0.0; S(2): 0.0; S(7): 0.0; Y(11): 9	187.3003078	22.86	3
S(3): 0.0; S(8): 50.0; S(9): 50.0; T(11	112.5214558	15.57	2
S(1): 0.0; Y(3): 0.0; Y(6): 0.0; S(8): 2.	173.2533382	33.57	3
T(4): 0.0; S(6): 100.0	326.6735097	30.73	2
S(1): 3.5; S(2): 96.5; T(15): 0.0	119.1162253	16.41	3
T(1): 0.0; S(3): 100.0	130.5473071	26.78	2
T(2): 0.0; S(9): 100.0	287.254075	34.2	2
S(2): 3.8; S(3): 96.2; T(8): 0.0; S(11):	256.2802742	30.96	3
S(2): 93.6; T(6): 93.6; S(9): 12.8; S(1	91.08204984	18.01	3
S(6): 50.0; S(7): 50.0; S(9): 100.0; S(	300.3521095	39.81	3
Y(5): 0.0; S(12): 100.0	306.3671864	27.79	3
S(3): 100.0; S(6): 0.0	268.0109432	51.43	2
S(5): 96.6; S(7): 0.1; S(8): 3.3	125.6111991	16.32	3
S(9): 100.0	227.279984	24.13	3
T(3): 0.0; S(8): 100.0; S(11): 0.0	156.8173138	14.84	3

Information Classification: General

S(2): 0.0; T(5): 0.0; S(14): 2.6; S(15):	219.6957737	45.98	3
S(1): 0.0; S(2): 0.0; S(5): 0.0; S(6): 10	304.0756078	41.92	2
S(3): 100.0	307.7055835	42.42	2
S(14): 0.0; S(16): 0.0; S(18): 97.7; T(	370.8282146	66.02	3
S(3): 100.0; T(6): 0.0; S(7): 0.0; S(13	301.1418883	32.76	3
T(1): 0.0; T(2): 0.2; S(4): 99.5; S(6): 0	146.329222	13.67	3
T(1): 0.0; S(5): 0.0; S(7): 0.0; S(10): 1	358.2461388	66.15	2
T(1): 0.0; S(3): 0.0; S(11): 4.3; T(13):	217.0576775	55.92	3
Y(1): 0.0; S(7): 100.0; S(12): 0.0	275.8908721	40.02	2
S(4): 100.0	301.978702	53.58	3
Y(2): 0.0; S(5): 0.1; S(7): 99.9; S(11):	141.7588657	9.57	3
T(16): 100.0; T(24): 0.0	210.8712639	47.7	3
S(6): 95.7; S(8): 0.0; S(9): 0.2; T(10):	99.39992951	37.8	3
S(10): 100.0; S(17): 0.0; T(19): 0.0	367.1810814	97.11	2
T(4): 2.3; T(7): 2.3; S(8): 95.5	204.0807269	39.93	2
T(1): 0.0; S(5): 100.0	113.2331873	14.42	2
S(1): 0.0; S(3): 100.0; T(5): 0.0	216.7789301	43.39	2
S(2): 2.0; S(4): 98.0; S(11): 0.0	240.2567066	40.46	3
S(1): 0.0; S(2): 0.0; S(4): 0.0; S(6): 10	332.5752799	69.49	2
S(3): 0.0; S(5): 0.0; T(7): 0.1; S(10): 3	143.9051061	41.75	2
S(4): 100.0	273.4106496	13.74	3
S(5): 100.0; T(7): 0.0; S(11): 0.0; Y(1	306.518812	56.05	3
S(1): 0.0; T(2): 0.0; S(3): 100.0	156.289889	11.9	2
S(5): 98.5; T(8): 1.5	182.6986184	45.66	2
S(5): 1.6; S(8): 98.4	204.0567826	16.76	2
S(5): 0.0; T(7): 0.0; T(10): 100.0	223.4190043	21.83	2
T(1): 0.0; S(4): 0.0; S(5): 0.0; S(6): 0.	279.7525609	38.6	3
S(7): 100.0; S(18): 0.0; T(20): 0.0	326.396009	40.65	3
T(7): 0.0; S(9): 100.0	235.2538739	45.53	2
T(1): 50.0; S(3): 50.0; S(7): 0.0	173.5069443	28.8	3
S(18): 0.0; S(19): 100.0	378.0889906	34	3
S(1): 4.3; S(3): 95.4; S(7): 0.2; T(9): 0	126.6347573	12.47	3
T(3): 94.4; S(4): 5.0; T(6): 0.3; S(7): 0	159.9267501	51.31	3
S(2): 100.0	161.4099394	11.23	2
T(1): 0.0; Y(5): 0.0; Y(10): 0.5; S(11):	294.9970911	37.26	2
S(8): 1.3; S(10): 98.7	448.0748984	55.52	2
Y(1): 0.0; T(4): 1.6; S(6): 98.4; T(7): 0	245.1338323	60.76	3
S(3): 100.0	180.1645919	29.35	2
S(4): 0.0; S(5): 0.0; S(11): 0.0; S(12):	96.72642768	0.46	3
S(1): 0.0; S(7): 100.0; S(13): 100.0	452.2177616	46.96	2
T(4): 0.0; S(9): 100.0	319.7547838	89.09	2
T(7): 100.0	176.5189615	16.46	4
T(1): 0.0; T(3): 50.6; S(6): 50.6; S(7):	183.4407543	38.95	2
S(9): 100.0	144.416688	19.59	3
S(5): 100.0	175.0724743	10.07	3
T(13): 33.3; T(14): 33.3; S(15): 33.3;	262.0483521	53.31	3
S(8): 100.0; S(10): 2.3; S(12): 97.7; S	220.0780614	42.35	2
S(1): 0.0; S(7): 100.0; S(10): 51.4; S(	163.8253639	13.45	3
S(6): 100.0; S(8): 0.0; Y(16): 0.0	426.9969917	77.31	3
S(4): 100.0; S(10): 0.0	193.7965031	32.97	2
S(3): 100.0; T(9): 0.0; S(18): 0.0; Y(2	430.8263826	64.37	2
S(6): 0.0; S(8): 100.0	185.8203201	24.72	2
T(9): 0.0; S(10): 0.0; S(15): 97.6; S(1	286.6607241	44.64	3
S(2): 0.0; S(4): 100.0; Y(9): 0.0; S(14	345.1329538	88.29	3
S(1): 100.0; T(5): 0.0; S(8): 0.0	376.1672801	52.53	2

Information Classification: General

T(1): 50.0; S(3): 50.0; Y(8): 0.0		146.5341109	9.95	3
T(6): 0.0; S(8): 0.0; S(17): 50.0; S(19		375.6680496	129.62	3
S(2): 4.2; S(3): 95.8; S(6): 0.0; T(17):		193.5726951	35.89	3
S(11): 96.7; Y(15): 3.3		131.5605526	13.83	3
S(2): 0.0; S(3): 100.0; S(5): 0.0; S(7):		243.8552674	27.61	2
S(2): 0.0; T(3): 0.0; S(5): 0.0; S(7): 0.		267.9827094	50.52	3
S(1): 0.0; S(7): 100.0; S(10): 0.0		376.557166	50.44	2
S(11): 0.0; S(14): 100.0; S(15): 100.0		266.6919181	64.95	2
S(3): 100.0; T(5): 0.0; S(7): 0.0; S(10		194.1100099	47.89	2
S(1): 0.0; S(6): 100.0		294.2388541	46.56	2
S(2): 50.9; Y(3): 50.9; S(11): 49.1; T(		104.5931119	9.02	4
T(2): 0.0; T(5): 0.0; S(10): 50.0; T(12)		187.2180084	41.44	3
S(2): 0.0; T(15): 3.9; S(16): 92.2; S(1		187.118363	29.97	4
S(9): 100.0; S(17): 100.0; S(21): 0.0		317.270392	64.89	2
T(2): 0.0; T(7): 99.8; S(9): 0.1; S(11):		77.38892853	29.53	3
S(3): 0.0; T(4): 0.0; Y(5): 0.0; S(7): 0.		339.4198878	20.92	3
T(1): 0.0; T(10): 50.0; S(13): 50.0; S(		259.7306708	53.24	3
S(3): 0.0; S(6): 100.0		293.6077562	38.42	3
S(4): 0.5; T(8): 99.8; T(10): 99.8; S(1		115.7914218	16.56	2
S(6): 0.0; S(12): 51.6; S(19): 51.6; S(		140.7066876	41.95	3
S(3): 100.0; T(7): 0.0; S(11): 0.0		241.1283109	47.63	2
S(2): 0.0; S(4): 0.0; T(6): 0.0; Y(10): (		119.0199126	14.91	3
S(2): 0.0; S(5): 100.0; T(11): 6.7; T(1		160.7640188	34.51	3
Y(3): 0.0; S(16): 100.0		288.6427918	41.78	3
T(3): 0.0; S(8): 100.0		219.7300352	34.52	3
S(6): 2.4; T(7): 97.6		187.0748537	18.88	3
S(4): 99.9; S(6): 0.1		119.6152248	11.39	2
S(1): 0.0; S(5): 0.0; T(11): 0.0; S(16):		115.2447855	19.57	3
T(3): 0.0; S(14): 100.0; S(15): 100.0;		284.2408921	43.56	3
T(1): 0.0; S(8): 100.0; S(10): 0.0; S(1		275.8938803	29.59	2
S(3): 100.0; T(14): 0.0; S(16): 0.0		271.3323324	25.91	3
T(5): 100.0		317.3783208	34.24	3
S(1): 0.0; T(6): 100.0		222.9569193	27.22	2
T(5): 100.0; S(9): 0.0; T(10): 0.0; S(1		314.5179141	52.85	2
S(1): 33.0; S(2): 33.0; S(3): 33.0; S(4		189.8911198	15.41	2
S(1): 0.0; S(5): 100.0; T(7): 0.0		156.3802473	19.78	2
S(3): 100.0		115.7858611	20.22	3
S(1): 0.0; S(3): 100.0		108.3527481	15.41	2
S(2): 100.0		267.1478245	67.54	2
T(4): 0.0; T(7): 8.8; T(8): 8.8; S(9): 88		144.8203221	37.82	2
S(4): 0.0; Y(5): 0.0; S(6): 0.0; S(8): 9		506.7540465	114.61	3
S(2): 100.0; T(5): 0.0; S(8): 0.0; Y(9):		322.5145331	46.98	2
Y(2): 1.8; S(3): 98.2		158.995539	13.89	3
S(8): 100.0; T(14): 0.0; S(15): 0.0; T(		419.5298368	63.68	3
S(4): 98.2; S(5): 1.8; T(13): 0.0		314.5132635	62.72	2
S(10): 100.0		209.9799614	48.32	3
S(9): 100.0		342.2576177	91.63	3
S(4): 0.0; S(5): 0.0; S(8): 100.0; S(10		189.6250572	24.14	3
T(1): 4.6; S(6): 95.3; S(8): 0.0; S(10):		193.8408033	17.71	4
S(3): 100.0; S(6): 0.0		211.6053979	21.35	2
T(2): 50.0; S(4): 50.0		169.8354081	12.44	3
T(1): 0.0; S(2): 0.0; S(3): 0.1; S(5): 2.		167.1731562	33.29	2
S(1): 0.0; S(5): 0.0; S(8): 0.0; S(11): 9		168.7049183	19.53	4
S(8): 100.0		203.8676643	20.18	4
S(1): 3.4; S(2): 3.4; S(4): 93.2		158.3859089	38.8	2

Information Classification: General

T(4): 0.2; T(6): 99.6; S(8): 0.0; S(9): 0	123.0194063	24.89	3
S(8): 100.0	174.2179893	8.09	2
S(3): 0.0; S(7): 97.7; T(8): 4.6; S(10):	195.1239954	16.07	2
T(1): 0.0; S(3): 100.0; T(6): 0.0	135.3259285	15.68	2
S(13): 100.0; S(15): 100.0	256.438805	9.57	3
S(1): 0.0; S(2): 0.0; S(11): 100.0	314.8795064	70.62	2
T(4): 0.0; T(8): 0.2; S(9): 99.8	126.4431883	28.69	3
S(1): 0.0; S(3): 100.0	351.9231447	61.8	2
S(7): 100.0; S(9): 0.0; S(11): 0.0; T(1	371.2342943	81.39	2
T(4): 0.0; T(6): 1.7; S(7): 98.3; S(11):	279.5004798	62.52	3
S(4): 100.0; T(8): 0.0	179.1496421	15.64	2
S(12): 97.3; T(14): 2.7	227.6854709	29.23	3
S(3): 99.7; S(6): 0.1; Y(7): 0.1; T(8): 0	239.6895838	33.05	3
S(8): 3.8; S(9): 96.2; T(19): 0.0	191.9466886	11.21	3
S(1): 100.0; S(16): 0.0	274.5300288	35.43	3
S(3): 100.0; Y(11): 0.0	230.912529	28.65	3
T(1): 0.0; T(3): 0.0; T(9): 50.0; S(10):	92.10579055	7.51	3
S(2): 0.0; T(5): 0.0; T(8): 100.0; S(11	239.3875893	49.65	3
S(1): 0.0; S(6): 0.0; T(13): 0.0; T(14):	329.7212797	42.07	3
S(1): 0.0; S(5): 50.0; S(6): 50.0	125.6825475	20.09	2
T(3): 0.0; S(7): 100.0	328.2475213	56.27	2
S(5): 50.0; S(6): 50.0; S(9): 0.0; S(10	206.5245851	51.4	3
Y(2): 0.0; S(5): 0.0; T(6): 0.0; T(7): 1.	205.6754502	25.33	2
S(1): 0.0; S(3): 100.0	167.4902755	28.39	2
S(1): 0.0; S(7): 98.5; T(10): 1.5; S(13	515.3199362	49.18	2
S(1): 0.1; S(3): 99.8; S(10): 0.0; S(15	118.4959842	5.81	3
S(18): 0.0; Y(19): 0.0; T(21): 100.0; S	334.512329	82.76	3
Y(1): 0.1; S(2): 2.4; S(3): 97.4; S(5): 0	293.2732807	72.56	2
T(3): 1.9; S(5): 98.1	170.9513002	41.91	2
T(4): 0.0; S(7): 0.0; S(11): 100.0	317.7040495	96.76	2
T(6): 50.0; S(7): 50.0; S(12): 0.0; T(1	149.1316938	29.53	3
S(1): 0.0; S(6): 4.6; S(9): 86.3; S(11):	160.8842708	26.27	3
S(2): 98.6; S(3): 1.4; Y(4): 0.0	319.3980791	64.38	2
S(5): 0.0; S(9): 100.0	382.5786851	79.71	2
T(3): 0.0; T(6): 0.0; T(8): 4.1; S(10): 9	173.6330159	28.95	3
Too many isoforms		11.27	3
S(6): 100.0; S(10): 100.0	238.970509	32.08	2
S(8): 50.0; S(10): 50.0; S(12): 0.0; S(	330.6000993	71.38	2
T(1): 3.4; S(2): 96.6; S(11): 100.0; T(	308.9363445	51.16	3
S(4): 0.0; S(14): 99.8; S(20): 0.2	174.3595604	23.21	3
S(7): 96.4; S(9): 3.5; T(11): 0.1; S(22	179.7312474	10.8	4
S(4): 100.0	417.4061383	67.86	2
S(7): 100.0	372.285812	69.62	2
S(6): 32.6; S(7): 32.6; T(8): 32.6; S(1	331.4196521	94.94	3
S(4): 100.0; S(10): 0.0	214.8279296	48.14	2
S(3): 0.0; S(10): 100.0	319.5773759	72.01	2
T(7): 100.0; T(12): 0.0	435.813757	74.69	2
Y(1): 0.0; S(7): 100.0	224.3751464	30.69	2
T(2): 0.0; S(4): 0.0; S(11): 100.0	295.1872532	66.37	2
S(4): 49.5; S(5): 49.5; S(7): 1.0; T(8):	193.8927329	31.27	2
S(1): 97.2; S(6): 0.7; T(8): 0.7; Y(10):	98.48694622	13.23	3
Y(2): 0.0; S(4): 0.0; S(24): 4.9; S(28):	139.8877683	27.31	3
S(5): 0.0; S(7): 0.3; T(10): 99.6; S(16	117.7708024	13.42	3
S(2): 100.0; S(5): 0.0; S(7): 0.0; S(8):	155.6280326	17.45	2
S(1): 100.0; S(10): 0.0	358.1169904	49.94	3

S(1): 0.0; S(10): 100.0; S(13): 100.0;	169.5115114	43.84	2
S(4): 0.0; T(12): 91.7; S(16): 4.0; S(2	311.3689732	79.05	4
Y(1): 0.0; Y(3): 0.0; S(8): 1.7; S(9): 49	260.121486	43.73	3
S(4): 1.8; S(5): 98.2	159.0592973	22.17	3
S(7): 100.0; S(15): 100.0; S(19): 0.0	242.0080446	38.29	3
S(7): 100.0; S(13): 100.0	97.75245182	8.61	5
T(2): 0.0; T(6): 33.3; S(7): 0.0; S(10):	137.0675021	13.58	3
S(6): 97.5; S(7): 0.1; S(9): 0.0; S(10):	213.8359845	41.81	3
S(9): 100.0	212.1151159	23.3	2
T(2): 0.0; Y(3): 0.0; S(7): 100.0	189.4682083	31.87	3
S(7): 100.0; S(13): 0.0	179.2515569	27.41	2
T(5): 0.0; S(10): 100.0; T(11): 0.0	302.9092392	56.4	2
Y(1): 0.0; S(5): 0.0; T(9): 0.0; T(10): 0	467.4339989	88.72	3
S(5): 100.0	250.5140539	29.18	3
S(7): 0.0; S(14): 96.5; S(15): 3.4; S(1	189.6979992	15.18	3
T(5): 0.0; S(10): 0.0; S(11): 0.0; T(13	267.5539209	47.57	3
S(2): 100.0; Y(12): 0.0; S(17): 0.0; S(	345.5109079	69.64	3
S(4): 100.0; S(7): 0.0	246.3085899	24.92	2
S(2): 0.0; S(7): 100.0; S(16): 0.0	280.83695	67.86	3
S(1): 0.0; S(8): 100.0	323.7531519	71	2
T(1): 0.0; S(4): 0.0; S(7): 100.0; S(13	493.0696136	83.92	2
S(5): 0.0; S(7): 0.0; S(10): 0.0; S(12):	80.46628979	11.85	3
S(6): 0.0; T(9): 0.0; Y(13): 8.0; S(14):	231.8431141	78.98	3
S(2): 50.0; S(3): 50.0; T(12): 0.0	70.58355157	5	3
T(5): 0.0; T(12): 0.0; T(13): 0.0; S(24)	206.866506	22.66	4
S(4): 0.0; S(5): 0.0; T(7): 0.0; S(10): 0	305.3998663	42.66	3
S(6): 50.0; S(8): 50.0	197.6677514	29.89	2
S(1): 100.0; T(3): 100.0; T(7): 0.0	147.4543572	15.91	3
T(6): 0.0; S(11): 3.0; T(12): 97.0	156.2842282	29.57	3
S(10): 100.0; S(11): 100.0; S(18): 0.0	257.4980636	43.64	3
S(2): 0.0; S(4): 0.0; S(7): 100.0	285.9591479	24.78	2
Y(3): 0.0; S(5): 1.8; S(6): 98.2	221.2859567	47.64	2
T(8): 0.2; S(9): 2.6; S(10): 2.6; S(11):	158.4961667	59	3
S(1): 100.0	132.9537525	27.97	2
Y(1): 0.0; T(4): 0.0; S(11): 2.0; T(17):	110.2386697	19.1	2
T(1): 0.0; T(2): 0.0; T(9): 0.0; S(16): 9	260.8213156	53.78	3
S(3): 33.3; T(4): 33.3; S(5): 33.3	86.59024962	15.23	3
S(11): 0.0; S(13): 100.0; T(17): 0.0	387.3714297	59.37	3
S(8): 100.0; S(10): 0.0	391.550348	62.86	2
T(1): 0.0; S(2): 0.0; S(10): 49.1; S(11	268.5114902	49.92	3
S(5): 100.0	383.3788267	49.39	4
Y(16): 0.0; S(20): 100.0	184.7044896	5.31	4
S(1): 0.0; S(3): 100.0; S(4): 0.0; S(11	412.2762872	93.14	2
S(2): 100.0	352.5545401	64.37	2
S(8): 0.0; S(13): 100.0; S(17): 100.0	372.0514869	36.63	3
S(2): 0.0; T(8): 0.0; S(18): 100.0; S(2	270.7804995	37.83	3
S(1): 88.4; T(3): 3.8; S(5): 3.8; S(6): 3	143.161399	6.62	3
S(1): 100.0; T(3): 0.0; S(5): 0.0	218.9827538	40.09	2
S(4): 99.7; S(6): 5.1; S(8): 95.2; S(10	182.9250764	1.52	3
S(1): 97.0; T(3): 3.0; S(11): 0.0; T(20	220.4252189	32.5	3
S(1): 0.0; Y(3): 0.0; S(4): 0.0; S(10): 0	297.7352375	28.54	3
S(2): 0.0; S(13): 50.0; T(15): 50.0	159.8530861	31.9	3
S(4): 100.0	194.659051	33.69	2
S(7): 1.8; T(10): 98.2	223.2076761	26.4	2
S(5): 98.9; T(7): 1.1; S(8): 0.0	267.8509933	35.78	2

Information Classification: General

S(3): 97.9; T(7): 0.1; T(8): 2.1	129.5717156	35.4	2
T(9): 2.8; Y(12): 97.2	117.805693	43.47	3
T(2): 0.0; S(3): 100.0	183.6473655	49.02	2
S(2): 0.0; T(10): 0.0; S(20): 100.0	131.0858234	9	3
S(11): 96.5; S(13): 3.5	206.9711325	17.2	3
S(3): 0.0; S(7): 100.0; S(13): 0.0; T(1	157.2930911	3.8	4
S(1): 0.0; S(5): 1.9; S(7): 98.1	247.0914471	47.34	2
S(3): 0.0; S(10): 100.0; S(13): 100.0;	161.1912312	13.6	3
S(1): 0.0; S(2): 0.0; S(10): 1.7; S(11):	277.2181286	58.2	2
S(1): 99.8; T(3): 0.2; T(10): 0.0; S(12	125.4599558	9.27	3
S(3): 100.0; S(9): 0.0	207.8332947	22.32	2
S(6): 4.1; T(8): 95.7; Y(11): 0.0; S(15	167.4281874	29.41	3
Y(6): 0.0; S(7): 0.1; S(10): 99.9	280.5550288	45.58	2
S(1): 0.0; S(9): 0.0; T(10): 0.0; S(17):	191.3710538	30.68	3
T(2): 0.0; S(5): 98.0; S(7): 2.0	130.4325854	26.87	2
T(1): 0.0; Y(13): 0.0; S(15): 100.0; S(	295.644443	44.16	4
S(7): 100.0; S(9): 0.0; S(11): 0.0	269.7848992	42.2	3
S(7): 0.1; S(10): 99.9; S(11): 99.9; S(	296.5598452	33.69	2
S(3): 100.0	147.918234	19.86	2
T(1): 0.0; S(3): 100.0; Y(5): 0.0	258.0515602	25.35	2
T(1): 100.0; S(5): 0.0	198.7836632	28.93	2
S(6): 100.0	296.8093311	53.8	2
T(2): 7.3; Y(3): 7.3; T(5): 7.3; S(11): 7	69.26477859	1.05	4
S(1): 49.5; S(3): 49.5; T(6): 1.0; S(12	222.127574	44.93	3
S(4): 100.0; S(8): 100.0; T(10): 0.0	302.4232264	42.53	2
T(5): 0.0; T(11): 0.0; S(15): 0.2; S(19	212.8339428	1.8	4
S(3): 99.6; S(6): 0.4; S(8): 0.3; S(12):	83.95761451	36.41	3
S(1): 98.8; S(4): 1.2	323.0332335	57.23	3
S(1): 0.0; S(3): 100.0; S(7): 0.0; T(24	245.6186823	19.02	3
S(4): 100.0; T(5): 0.0; Y(9): 0.0; S(12	247.7003519	47.45	2
S(5): 0.0; T(15): 98.6; Y(16): 1.4	468.3655907	114.1	3
S(6): 0.0; Y(7): 0.0; S(18): 100.0	239.4104569	33.64	3
S(7): 50.0; S(8): 50.0	305.5807038	50.76	2
Y(5): 0.0; S(6): 100.0	301.3196428	42.25	3
S(5): 99.8; T(7): 0.2; S(13): 0.0; S(19	279.5097663	64.27	3
S(6): 100.0	138.2873204	14.7	3
T(7): 0.0; T(13): 0.0; S(14): 0.0; S(17	225.7461303	31.36	4
T(3): 0.0; S(5): 49.4; S(6): 49.4; S(10	111.8529785	17.76	2
S(11): 49.1; S(14): 49.1; S(19): 1.8	188.0644481	18.09	3
S(1): 0.0; T(3): 0.0; T(4): 0.0; T(5): 0.	222.6408829	52.71	2
S(1): 0.0; S(2): 0.0; S(3): 99.9; T(5): 0	281.5816344	76.05	2
S(4): 0.0; S(14): 100.0; S(20): 0.0	224.4892669	17.86	3
S(3): 100.0; S(6): 100.0	284.22366	16.96	3
S(2): 0.0; S(6): 0.0; S(9): 100.0	539.179757	86.51	2
S(4): 100.0; S(8): 0.0	153.5575825	31.92	2
S(2): 0.0; S(5): 2.8; S(7): 2.8; S(8): 94	191.134709	42.06	2
S(2): 0.0; S(12): 0.0; S(17): 49.5; T(1	396.5292397	77.64	2
T(3): 0.0; S(7): 0.0; S(8): 0.0; S(15): 1	209.4170358	34.77	3
S(2): 99.9; T(7): 33.4; T(8): 33.4; S(9	96.55107877	8.92	3
Y(3): 0.0; S(11): 50.0; T(13): 50.0	392.6158653	84.86	2
T(4): 100.0; S(6): 100.0; S(13): 94.4;	178.3996177	12.35	3
S(1): 0.0; S(3): 100.0; S(5): 0.0; S(6):	162.4549001	13.17	2
S(2): 100.0; S(7): 0.0; S(11): 0.0	110.9107928	12.99	2
S(2): 0.0; S(5): 94.5; T(6): 94.5; S(8):	108.6162475	7.5	3
S(3): 100.0	165.5644648	13.01	3

S(4): 0.0; S(10): 50.0; S(12): 50.0	292.9876857	58.82	2
S(9): 100.0; S(16): 0.0; S(18): 0.0	301.8705047	67.62	3
S(4): 0.0; S(7): 33.3; S(8): 33.3; S(10	215.6935216	28.79	2
S(4): 100.0; S(7): 0.0	40.12460467	12.97	3
S(4): 0.0; T(5): 0.0; S(8): 100.0; S(11	180.4484515	35.23	3
S(3): 100.0	192.0466286	19.3	2
S(2): 100.0; Y(7): 0.0	151.5197192	29.42	2
S(1): 0.0; S(2): 0.0; S(4): 1.9; T(5): 1.	232.0181709	29.36	2
S(3): 50.0; S(5): 50.0	141.3399621	7.09	3
S(2): 0.0; S(7): 0.0; S(10): 100.0; S(1	323.0044127	53.05	2
T(1): 0.0; T(10): 33.3; S(13): 33.3; S(	254.1142254	47.8	3
S(9): 100.0	249.6973996	9.09	4
S(9): 0.0; T(10): 0.0; S(15): 0.1; S(17	238.9873198	43.6	3
S(2): 97.6; S(3): 2.4; S(5): 0.0	259.5175977	32.62	3
S(2): 0.0; T(4): 0.0; S(7): 100.0; S(14	225.5026201	49.6	2
S(3): 0.0; S(7): 100.0	420.2704879	79.82	2
S(1): 0.0; S(2): 0.0; S(3): 99.9; S(6): (	237.7091832	57.05	2
T(9): 0.0; S(11): 0.0; S(18): 100.0	190.2649578	59.39	3
Y(2): 0.0; S(4): 100.0; S(8): 0.0	197.1205459	33.17	2
S(4): 100.0; T(20): 0.0	393.146863	81.29	3
S(2): 0.0; S(11): 100.0	219.7899783	14.29	3
S(5): 100.0; Y(11): 0.0; Y(15): 0.0	322.4874444	32.43	2
T(2): 92.3; S(7): 7.6; T(11): 0.1; S(13	123.4280275	15.94	4
S(3): 100.0; S(4): 100.0; S(7): 0.0	304.9002862	49.76	3
S(10): 93.8; S(12): 5.8; T(20): 0.0; S(	111.307845	5.81	3
T(3): 50.0; T(4): 50.0; T(8): 0.0	196.9626509	39.18	3
S(4): 0.0; S(6): 100.0; T(8): 99.7; T(9	144.2430266	10.93	3
S(5): 100.0; T(12): 0.0; S(13): 0.0; S(	368.548893	85.79	2
S(1): 0.0; S(10): 0.0; S(15): 100.0; S(	348.5018945	42.48	3
S(1): 51.7; T(4): 51.7; S(7): 95.4; S(1	109.9121918	16.43	3
S(1): 0.0; T(4): 0.9; T(5): 49.6; S(6): 4	186.9212493	25.85	2
S(3): 0.0; S(5): 0.0; S(11): 0.1; S(12):	202.1563186	41.57	2
S(4): 100.0; Y(7): 0.0	208.9671715	41.35	2
T(5): 0.0; S(15): 100.0; Y(26): 0.0	344.6131607	38.89	3
T(2): 50.0; S(3): 50.0; S(9): 100.0; T(	156.4596013	23.93	3
T(3): 0.0; S(5): 100.0; S(9): 0.0	264.4421742	59.62	2
S(7): 100.0; S(16): 0.0; T(19): 0.0; T(	270.5075561	34.56	3
T(7): 0.0; S(8): 0.0; Y(13): 0.0; S(21):	279.9720145	14.5	4
T(2): 100.0; T(5): 100.0; T(13): 0.0; S	173.4900618	12.66	3
Y(3): 0.0; Y(8): 0.0; T(13): 100.0	295.2449264	47.57	2
S(7): 100.0; S(19): 0.0	362.6367881	33.61	3
S(1): 0.1; S(3): 0.1; S(6): 99.8; Y(11):	224.7471144	11.86	3
T(5): 100.0; S(9): 100.0	403.6158372	19.04	2
S(2): 0.0; S(9): 100.0	436.6809918	38.66	2
S(4): 1.9; T(6): 98.1	152.2811686	21.71	3
T(1): 50.0; S(3): 50.0; T(8): 0.0; T(9):	248.2347454	32.85	3
S(7): 100.0	334.3048797	41.87	3
S(3): 0.0; T(6): 1.3; S(8): 98.7	378.945675	81.91	2
S(3): 97.3; T(5): 2.7; S(7): 0.0; S(8): (	117.7192481	10.36	2
T(5): 0.0; S(14): 25.0; T(16): 25.0; T(	215.2792277	33.32	5
S(2): 0.0; T(3): 0.1; Y(4): 2.2; S(5): 2.	243.2475855	44.88	2
S(9): 0.0; Y(11): 0.1; T(12): 99.9; T(1	264.1374028	26.9	3
S(5): 100.0	592.3195874	82.97	2
S(5): 100.0; T(17): 0.0	266.1849026	34.72	3
T(5): 3.2; S(6): 96.8; S(8): 0.0; T(11):	204.8669639	39.97	3

S(1): 93.6; T(2): 93.6; S(10): 12.8; T(	46.84271746	11.38	3
T(1): 0.0; S(3): 100.0; S(6): 99.9; S(8	163.7724719	32.08	2
S(1): 100.0	131.9292156	10.5	3
S(5): 0.1; S(10): 99.9	164.9328075	10.86	3
S(8): 100.0; S(17): 100.0	68.93787891	15.83	7
Y(5): 0.0; S(6): 0.0; S(11): 100.0; Y(1	246.9074047	58.85	3
S(1): 0.0; S(3): 0.0; S(6): 100.0; S(9):	347.2587768	76.06	2
T(7): 3.8; S(9): 14.0; S(12): 12.0; S(1	36.58222529	17	4
S(5): 100.0; S(6): 100.0; T(11): 0.0	473.523566	73.9	3
Y(3): 100.0	129.5374899	6.13	2
T(12): 100.0; S(19): 0.0	203.6424232	48.38	3
S(3): 100.0; S(8): 0.0; S(13): 0.0; T(1	107.1195728	13.15	3
S(5): 100.0; S(7): 0.0; T(8): 0.0	228.9518535	38.33	2
S(3): 100.0; S(11): 0.0	271.1796771	38.65	3
S(3): 100.0; S(13): 0.0	469.3598113	86.36	2
S(6): 100.0; S(12): 100.0	308.8460609	54.33	2
Y(7): 50.0; S(8): 50.0	332.619091	35.39	4
S(10): 100.0; S(12): 0.0	291.2314438	52.22	3
S(8): 0.0; S(9): 0.0; T(11): 0.0; S(21):	254.55507	51.58	3
T(2): 0.0; S(5): 100.0	211.733388	28.56	2
T(1): 0.0; S(3): 100.0; T(5): 0.0; S(6):	212.2520748	29.81	2
S(11): 100.0; S(13): 0.0	316.9512256	71.8	3
Y(3): 1.4; Y(7): 98.6; T(9): 0.0	201.959696	34	2
S(5): 100.0; S(8): 0.0; T(9): 0.0; S(16	157.0515972	8.45	3
S(8): 100.0; T(13): 0.0	400.8313526	44.5	3
S(2): 0.0; S(7): 0.0; S(9): 0.0; S(13): (	159.5392947	23	3
S(6): 100.0; T(7): 0.0	169.0191072	18.12	3
S(3): 96.6; S(4): 3.4; T(5): 0.1; S(6): 9	171.1239462	47.78	2
S(3): 100.0; S(8): 0.0; T(13): 100.0; S	315.209232	40.67	4
T(4): 2.4; S(6): 97.5; S(7): 0.1; S(10):	137.519363	23.5	2
S(10): 100.0	432.0893706	54.53	2
S(2): 100.0; T(12): 0.0; T(13): 0.0	278.6983155	46.25	3
S(2): 100.0; S(5): 100.0; S(19): 0.0	115.1970756	20.6	3
S(1): 0.1; S(2): 99.9; S(4): 50.0; S(5):	101.5718816	15.83	2
S(3): 50.0; Y(4): 50.0; T(7): 0.0; T(9):	169.5987122	27.05	3
S(8): 100.0	195.4995586	24.46	3
S(2): 100.0; T(12): 0.0; S(18): 0.0; Y(	349.5993275	66.28	3
S(1): 0.0; T(5): 100.0	187.5797624	25.03	2
S(1): 0.0; S(12): 50.0; T(14): 50.0	135.2687664	20.53	3
Y(2): 0.0; S(3): 0.0; Y(4): 0.0; T(6): 0.	353.3604032	67.29	3
S(1): 0.0; S(3): 100.0; S(4): 0.0; S(8):	347.9704832	90.04	2
T(1): 0.0; S(6): 0.0; S(8): 0.0; S(10): (	256.1158254	61.41	4
S(2): 0.0; S(4): 100.0	419.7977995	83.52	2
S(4): 100.0	268.1264929	36.86	2
S(3): 50.0; S(8): 50.0; S(12): 100.0; S	253.8016113	29	3
S(13): 100.0; S(19): 100.0; T(23): 33.	331.9893013	19.24	5
S(2): 100.0; S(4): 0.0	237.5883102	48.93	2
T(1): 99.9; T(3): 0.1; T(7): 0.0; Y(10):	167.5645933	27.4	3
T(7): 0.0; S(14): 100.0	381.6600843	55.52	2
T(1): 0.0; S(3): 100.0; S(6): 100.0; T(	250.5949588	58.76	2
T(9): 0.0; S(12): 100.0; T(19): 0.0	338.3279942	101.09	2
T(2): 0.0; S(10): 100.0; S(12): 100.0;	203.5357252	24.83	3
S(1): 0.0; S(4): 0.0; S(7): 100.0; Y(14	374.500416	84.88	2
S(4): 0.0; T(6): 0.0; S(8): 100.0; T(17)	329.4946553	61.41	3
S(9): 0.0; S(10): 100.0; T(14): 0.0; Y(	331.848157	51	3

S(7): 100.0; S(17): 0.0		355.1997569	61.39	3
S(10): 100.0; S(16): 0.0		274.4472465	73.78	2
S(6): 100.0; S(12): 0.0; T(13): 0.0		402.890549	59.38	2
S(3): 100.0; S(6): 100.0		318.5129821	36.9	3
S(4): 100.0; T(11): 0.0		155.0843505	10.26	3
T(1): 0.0; S(4): 0.0; S(12): 100.0; S(1		349.3899999	62.85	2
S(4): 97.5; S(5): 2.5		141.8721208	15.92	2
T(5): 0.0; S(7): 0.0; S(9): 0.0; S(14): (		150.9468237	15.61	3
S(1): 0.0; S(9): 100.0; S(10): 100.0; T		284.2564628	50.8	3
S(1): 100.0		97.04901698	2.79	3
S(6): 0.0; S(7): 50.0; S(9): 50.0; Y(13		290.7393717	40.84	2
T(6): 0.0; T(7): 99.9; S(10): 0.0		260.4099852	45.2	2
T(3): 0.0; S(6): 0.0; Y(9): 0.0; S(11): (		288.6887501	117.73	3
S(16): 4.4; S(17): 4.4; S(22): 91.2; S(		187.2538862	19.01	4
S(1): 0.0; T(2): 0.0; T(5): 100.0; S(9):		330.0829443	33.92	3
S(2): 100.0; S(11): 0.0		197.7950799	28.95	3
S(1): 0.0; S(4): 0.0; S(8): 0.0; S(14): (		427.1303443	64.44	3
S(5): 0.0; S(9): 100.0		344.3548642	77.33	2
S(2): 0.0; T(5): 0.0; S(6): 0.0; Y(11): (		320.5127119	51.95	3
S(13): 100.0		544.1480456	85.7	2
S(9): 100.0; S(14): 0.0; S(15): 0.0; T(		196.6186726	37.51	3
S(1): 0.0; S(3): 0.0; S(5): 0.0; T(6): 0.		252.6864461	43.72	2
S(13): 100.0; S(19): 100.0; T(23): 0.0		380.2068118	39.5	4
T(1): 0.0; T(3): 0.0; T(5): 1.7; S(9): 98		191.8991404	45.45	2
S(5): 0.0; S(10): 100.0		328.4323204	33.06	2
Y(3): 0.0; S(5): 100.0		255.0751139	52.12	2
S(5): 100.0; S(13): 0.0; T(16): 0.0		320.6583256	47.04	2
S(1): 0.0; T(5): 0.0; S(12): 0.0; S(16):		443.7004718	45.49	3
T(2): 0.0; S(5): 100.0; T(8): 0.0; S(9):		506.6339044	132.13	2
S(3): 0.0; S(12): 3.8; S(14): 88.5; S(1		147.4984716	35.92	3
S(1): 0.1; T(2): 99.9; T(4): 0.0; T(9): (		252.9873292	55.76	2
S(4): 100.0; T(7): 0.0; S(8): 0.0; Y(13		323.7063446	50.29	3
T(8): 100.0		178.7481625	31.17	3
T(1): 0.0; T(2): 0.0; S(4): 0.0; S(5): 99		262.2475586	48.53	2
S(3): 100.0; T(7): 0.0; Y(9): 0.0; T(20		244.4972192	28.67	3
T(19): 50.0; S(20): 50.0; T(25): 0.1		272.0231093	77.25	3
S(3): 100.0; S(12): 0.0		380.6481227	41.42	3
T(4): 9.2; Y(8): 91.7; S(15): 99.1		41.77727215	3.93	3
S(7): 100.0; S(9): 100.0; T(11): 0.0; T		289.0426121	112.52	3
S(1): 0.0; S(5): 100.0; T(7): 0.0; T(11		174.1782447	25.35	2
T(3): 50.0; S(4): 50.0; S(10): 0.0; S(1		192.1596461	23.75	3
S(11): 50.0; S(13): 50.0; S(23): 100.0		112.8840323	18.98	3
S(4): 0.1; T(6): 2.9; S(9): 97.0; S(14):		253.2906143	31.07	3
Y(2): 0.0; T(7): 100.0		322.7141992	34.21	3
S(2): 0.0; Y(5): 0.0; S(12): 99.9; S(17		181.2689179	6.22	4
S(3): 99.9; S(4): 3.6; S(6): 96.6; S(10		149.2672771	27.31	2
S(5): 100.0		88.42759982	12.97	3
T(1): 0.0; S(6): 4.4; S(8): 95.2; S(10):		239.6207609	41.01	3
S(1): 0.1; S(3): 99.8; S(4): 0.1; S(6): (		375.4151959	59.39	3
S(5): 100.0; T(10): 0.0		232.8955341	33.39	2
T(6): 6.0; S(7): 88.3; S(9): 11.3; S(13		154.7314359	36.05	3
S(1): 0.0; S(3): 100.0; S(9): 0.0; T(14		331.7279407	80.83	2
S(4): 0.0; S(8): 100.0; T(10): 0.0; T(1		398.6462211	83.21	2
S(7): 100.0		237.0479657	35.07	2
S(1): 0.0; S(3): 0.0; S(6): 100.0		172.38704	17.26	2

Information Classification: General

T(4): 0.0; T(11): 0.0; S(15): 100.0; T(	306.4949035	29.17	3
T(5): 0.1; S(7): 96.7; S(8): 3.2	204.4805273	35.63	3
S(3): 100.0	89.26647203	11.76	2
T(5): 100.0	241.8920289	49.48	2
S(2): 100.0; T(12): 0.0; T(13): 0.0; S(	294.3915605	64.98	5
T(1): 0.0; S(2): 0.0; T(7): 0.0; S(12): 1	264.38911	34.62	2
S(2): 0.0; S(3): 0.0; S(13): 100.0; S(1	400.4932069	77.2	2
S(16): 100.0	191.7272002	19.43	3
S(2): 0.3; S(7): 5.0; S(11): 94.8; S(14	128.6318559	9.48	3
T(1): 0.0; Y(5): 0.1; T(7): 0.1; S(11): (	304.4266119	72.05	3
S(1): 0.0; T(2): 0.0; T(4): 0.0; S(5): 0.	455.615335	111.16	2
T(1): 0.0; S(6): 100.0; S(8): 0.0; S(9):	269.1095105	54.77	3
S(1): 100.0; S(5): 0.0	194.0775143	41.63	3
S(7): 95.7; S(9): 4.0; T(12): 0.2; S(13	179.4097068	21.13	4
Y(3): 0.0; S(5): 0.0; T(6): 0.0; S(10): 9	289.47398	49.83	3
S(1): 100.0; T(5): 0.0	218.7394729	31.01	2
S(3): 100.0; S(6): 0.0; S(7): 0.0; S(10	272.0451732	47.72	3
S(13): 3.4; S(16): 96.5; T(22): 0.1; S(	351.8361429	53.75	4
S(1): 0.0; S(8): 50.0; S(10): 50.0; S(1	229.1577186	19.98	3
S(12): 100.0; S(21): 0.0; S(22): 0.0	283.7836899	61.22	3
S(4): 6.3; T(6): 93.7; S(8): 96.7; S(10	167.5506221	41.5	3
S(3): 100.0; T(5): 0.0; S(7): 0.0; S(10	490.943667	117.79	2
S(10): 99.9; S(12): 0.1; S(16): 0.0; Y(	348.3399367	82.93	3
S(1): 2.8; S(2): 97.2; S(4): 99.9; S(6):	140.4761401	15.84	2
S(3): 0.0; T(9): 0.0; S(19): 100.0	304.87585	66.9	3
S(1): 0.0; T(9): 100.0; T(12): 0.0	368.7337108	71.32	3
S(5): 0.0; T(6): 2.1; S(7): 97.9	175.4322142	28.74	2
S(3): 100.0; T(8): 0.0; T(9): 0.0; S(10	204.6986775	50.88	3
S(2): 0.0; S(13): 0.0; T(20): 100.0; T(	323.7102013	55.72	3
S(1): 50.0; S(4): 50.0; S(15): 0.0; S(1	406.3743808	68.7	3
S(3): 0.0; S(4): 100.0	156.5669427	40.5	2
S(1): 0.0; S(3): 100.0	261.902526	41.68	2
S(3): 100.0; S(5): 100.0; T(7): 0.0	329.8014473	51.28	3
S(5): 100.0; S(17): 0.0	86.96649746	5.22	3
T(8): 0.0; S(21): 4.8; S(22): 95.2; T(2	242.2910717	29.27	3
S(2): 0.0; S(3): 50.0; S(4): 50.0	149.9504211	22.77	2
S(5): 100.0; S(11): 0.0	414.5758531	87.49	2
Y(7): 0.1; S(9): 99.9; S(13): 0.1; Y(14	252.7465816	55.2	3
T(14): 0.0; S(19): 100.0	601.4278022	102.31	2
S(3): 100.0; S(5): 0.0; S(10): 0.0	295.5247215	42	3
S(11): 100.0	488.9080144	34.18	3
S(2): 0.0; S(7): 50.0; S(9): 50.0	119.4394375	19.8	2
S(5): 100.0	270.7252215	39.59	2
S(7): 0.0; T(11): 0.0; T(18): 5.1; S(22	235.1480055	38.61	4
S(14): 100.0	471.852492	82.13	2
S(1): 2.8; T(3): 97.2	255.8084549	57.06	2
T(2): 0.0; S(3): 0.0; S(12): 100.0	291.7954369	60.54	3
S(8): 100.0; T(14): 0.0; S(15): 0.0; T(	392.3016076	25.41	3
S(1): 0.0; S(2): 0.0; S(7): 0.0; Y(10): (	273.0111359	39.19	2
T(1): 0.0; S(13): 100.0	398.275658	70.04	3
Y(5): 0.0; Y(6): 0.0; T(9): 50.0; S(10):	190.7192649	20.73	3
S(1): 93.7; T(3): 6.3; S(5): 100.0; S(7	178.5023009	9.18	3
S(4): 100.0; S(8): 0.0	138.5194447	17.05	3
Y(1): 0.0; S(6): 100.0; S(10): 0.0	259.343267	39.82	2
S(6): 100.0; S(17): 0.0	372.8985668	87.34	3

S(1): 0.0; S(6): 0.0; S(11): 0.0; S(17):	257.4464831	34.73	4
T(1): 0.0; S(3): 0.0; S(9): 100.0	510.3209701	47.22	3
S(3): 0.0; S(8): 100.0	191.9982834	14.01	3
T(1): 0.0; T(3): 0.0; S(7): 2.6; S(8): 97	144.0295401	29.71	2
S(4): 100.0; S(6): 0.0	234.9530136	41.84	2
S(2): 50.0; S(3): 50.0	269.5738659	57.51	3
T(3): 100.0; S(5): 100.0; T(9): 0.0; T(	125.6966939	11.56	3
S(2): 50.7; S(3): 50.7; S(5): 98.7; T(1	199.2231581	54.75	3
S(2): 0.0; S(3): 100.0	155.4604188	14.62	3
S(2): 96.3; S(3): 3.6; S(6): 0.2	62.92127787	9.71	2
S(9): 0.0; S(21): 0.0; T(23): 2.0; S(25	365.4732654	54.15	4
S(8): 100.0; S(12): 100.0; T(16): 0.0;	399.9241702	77.62	3
Y(4): 1.8; S(6): 98.2	146.6934056	17.53	2
S(3): 0.0; T(7): 3.0; S(12): 96.9; S(17	231.8647373	81.33	3
S(10): 94.4; T(16): 5.6; S(21): 0.0; T(	121.8387283	9.32	3
Y(3): 0.0; S(5): 100.0; S(15): 95.5; T(	259.5731742	30.63	3
T(2): 0.0; S(6): 0.0; S(11): 100.0	232.3412035	49.87	3
T(3): 26.8; S(5): 73.2; S(7): 50.0; S(9	209.1836021	43.47	3
T(1): 0.0; S(2): 0.0; S(3): 100.0; T(6):	229.257805	48.45	2
T(1): 0.0; S(6): 0.0; S(7): 0.0; S(10): 1	296.3397922	56.52	2
S(11): 0.0; S(17): 100.0	342.351215	70.66	3
Y(5): 0.0; S(10): 100.0	240.1046458	35.22	3
S(1): 100.0; S(4): 95.9; T(12): 3.9; S(	128.7762816	44.23	2
S(4): 100.0	142.2264035	18.4	2
S(11): 99.9; S(13): 0.1	280.6504397	44.85	3
S(9): 100.0	240.7759046	22.46	2
S(11): 100.0; S(17): 100.0; T(21): 33.	289.2841762	24.79	4
S(6): 100.0	176.5628218	38.8	2
S(4): 99.4; S(7): 0.6; S(8): 0.0; T(19):	80.13351622	3.39	3
S(1): 100.0; S(7): 100.0; S(10): 0.0	372.2954744	39.79	2
S(3): 100.0; T(5): 0.0	418.3029803	32.38	3
S(9): 100.0; T(13): 0.0; S(14): 0.0	382.655323	101.23	2
S(3): 0.0; S(6): 100.0	172.6430532	34.91	2
S(1): 0.0; T(3): 0.0; S(4): 0.0; S(5): 0.	226.8075664	51.08	2
S(4): 100.0	218.4520348	19.42	3
Y(2): 0.0; T(7): 100.0	377.5587091	78.8	2
S(1): 33.3; S(2): 33.3; S(3): 33.3; T(1	273.3115718	30.28	3
S(2): 100.0; T(9): 0.0	282.6803925	41.33	2
T(8): 0.0; T(10): 0.0; S(13): 100.0; S(	231.6697449	39.46	3
S(5): 100.0	300.1584955	15.17	3
S(2): 0.0; S(4): 0.0; Y(5): 0.0; Y(10): 0	333.6570513	43.98	3
T(1): 0.0; S(6): 0.0; T(11): 0.0; T(18):	115.3601137	19.08	3
Y(19): 100.0	130.2189791	5.86	3
S(6): 1.2; Y(7): 98.8; S(10): 0.0	300.3345064	34.58	2
Y(2): 0.0; S(5): 0.0; S(7): 0.0; T(8): 0.	390.3377209	99.96	2
S(1): 0.0; S(5): 100.0	166.7118732	16.15	3
T(5): 0.0; S(8): 98.5; Y(9): 1.5	406.3408311	55.85	2
S(10): 1.9; S(13): 98.1	293.3861225	51.3	2
S(4): 100.0; T(7): 0.0; T(12): 0.0; S(1	151.3389097	33.56	3
Y(1): 2.8; S(2): 97.2; T(18): 0.0; S(20	302.4494488	46.78	4
S(8): 100.0; S(25): 0.0	343.2023812	63.67	3
S(4): 100.0; Y(8): 0.0	205.5185906	18.9	2
S(3): 0.0; S(6): 0.0; T(9): 0.0; T(11): 0	277.7492049	49.33	4
T(8): 0.0; S(13): 99.9; S(15): 0.1; S(1	292.9941455	33.98	3
T(5): 100.0; S(8): 0.0; S(12): 0.0	386.9383852	47.37	2

Information Classification: General

S(1): 0.0; S(3): 100.0; S(5): 0.0; T(8):		137.6314928	18.39	2
S(2): 99.7; T(4): 0.1; S(6): 0.1; S(7): 0		245.8272142	64.23	3
S(5): 100.0		283.4438415	42.96	2
S(15): 99.9; S(17): 0.1; S(20): 0.0; S(		214.779008	22.63	3
S(1): 100.0; S(2): 0.0; S(4): 0.0; T(9):		262.3735533	57.75	2
Y(5): 0.3; S(7): 99.8; S(9): 99.8; T(16		202.02877	28.95	3
S(1): 0.0; S(7): 5.4; S(9): 5.4; T(11): 8		135.3564977	25.82	3
T(1): 50.0; S(2): 50.0; S(6): 0.0; T(11		298.4268819	58.74	3
T(1): 0.0; S(3): 100.0; S(9): 100.0		399.78322	27.88	3
S(4): 100.0; S(7): 100.0		334.6853255	43.77	2
S(4): 100.0; S(7): 0.0; Y(15): 0.0; T(2		359.1156197	67.8	3
S(12): 100.0; S(21): 0.0; T(22): 0.0		346.291704	83.92	2
S(1): 0.0; T(2): 0.0; S(4): 97.9; S(6): 2		110.9190663	17.46	2
S(1): 0.0; S(2): 0.0; S(4): 0.0; S(13): 0		447.2238915	85.37	2
T(2): 0.0; S(3): 0.0; S(5): 0.0; T(9): 0.		233.9949234	19.38	3
T(1): 0.2; S(3): 0.2; S(6): 99.4; S(13):		254.2883553	31.07	3
S(3): 100.0		195.0543401	29.53	3
S(2): 0.0; S(4): 0.0; S(8): 97.7; S(10):		185.7015873	13.03	2
S(4): 0.0; S(9): 100.0		204.0698424	51.5	3
T(1): 0.0; T(4): 0.0; Y(9): 0.0; S(12): 0		406.7976717	67.82	3
S(2): 0.0; Y(3): 0.0; S(11): 100.0; T(1		417.7151255	34.73	3
S(5): 0.1; S(7): 99.9; S(14): 0.0		261.9759442	33.01	3
S(1): 99.4; S(4): 0.0; S(6): 0.3; S(8): 0		127.4416342	9.71	2
T(1): 97.8; S(3): 2.2; S(6): 0.0; Y(10):		301.4158909	45.83	3
S(1): 100.0; T(5): 0.0		249.7876053	57.05	2
S(4): 0.0; T(5): 0.0; T(8): 100.0		187.2626699	8.64	2
S(5): 100.0		386.0912432	73.52	2
T(6): 0.0; T(9): 0.0; S(11): 50.0; S(14		211.0753797	24.86	3
T(3): 2.6; S(6): 97.4; T(8): 0.1; T(14):		331.8394689	42.13	3
S(10): 100.0		177.0677549	22.61	3
T(12): 0.0; S(15): 100.0		206.2776189	27.9	4
S(4): 33.2; S(7): 33.2; T(8): 33.2; Y(2		74.33264851	6.53	4
Y(6): 0.0; T(7): 0.0; S(24): 100.0		128.1746404	20.94	3
S(4): 0.0; T(11): 0.0; S(13): 2.7; S(15		269.9726624	41.65	3
S(1): 0.0; S(3): 0.0; S(6): 0.0; S(7): 0.		306.3671864	77.91	2
S(8): 3.6; T(9): 96.5; S(11): 99.9		149.3542133	23.36	2
S(3): 99.9; T(5): 0.1; T(14): 0.0; T(19		377.5799313	80.9	3
S(8): 0.0; T(9): 0.0; S(11): 100.0		305.8342956	46.29	3
S(9): 100.0		226.909425	48.92	2
T(9): 96.6; S(10): 3.4		150.9245119	23.09	3
T(1): 49.3; S(4): 49.3; S(6): 30.1; T(9		211.7813729	29.4	3
S(7): 100.0		112.8734173	3.02	4
T(3): 0.0; S(10): 100.0; T(15): 0.0		347.9911296	52.38	2
Y(4): 0.0; S(5): 0.0; T(9): 1.1; S(16): 4		223.5522685	54.87	2
S(7): 100.0		281.1609838	41.9	2
S(7): 100.0; S(9): 0.0; S(11): 0.0; T(1		267.3237528	29.71	2
S(3): 0.0; S(5): 0.1; S(9): 91.5; S(10):		131.140491	41.83	3
T(14): 0.0; S(18): 100.0; S(26): 0.0		294.5136989	40.27	4
S(7): 100.0; S(9): 100.0		142.6128741	8.48	4
S(1): 0.0; S(5): 0.0; S(6): 0.0; S(7): 0.		293.5171792	57.78	2
S(1): 0.0; S(12): 100.0		289.9085649	30.1	3
S(4): 2.2; S(7): 97.8; S(8): 0.0		397.9915144	73.43	3
T(2): 0.0; S(4): 0.0; Y(5): 0.0; Y(7): 2.		199.8994606	27.77	2
T(1): 0.0; Y(2): 0.0; T(8): 99.7; T(10):		233.3493941	63.28	3
S(7): 100.0; T(10): 0.0		164.2772586	6.36	3

Y(3): 50.0; S(4): 50.0		61.55930578	7.5	3
Y(2): 1.0; S(3): 49.5; S(5): 49.5; T(8):		146.3581936	28.2	2
S(1): 0.0; T(2): 0.0; S(3): 0.0; S(8): 99		194.7830625	51.65	3
T(1): 0.0; S(12): 100.0; S(16): 100.0;		243.9546333	37.57	3
Y(5): 50.0; S(10): 50.0		106.4440249	24.62	2
S(6): 100.0; S(8): 0.0; T(13): 0.0; T(1		353.8172935	64.85	3
S(6): 100.0; S(21): 0.0		422.7445645	85.6	3
S(1): 0.0; S(6): 0.0; T(11): 50.0; S(14		221.4937676	37.41	2
S(8): 0.0; S(12): 0.0; T(15): 0.0; S(20		357.9978345	70.72	3
S(3): 97.6; T(4): 2.4; T(8): 0.0; Y(14):		263.0580851	33.24	3
S(5): 100.0; S(9): 100.0		110.9137109	16.57	2
S(3): 0.0; T(5): 0.0; T(9): 33.3; S(10):		205.2932068	21.29	2
S(9): 100.0; S(12): 0.0; Y(13): 0.0; S(		250.0680494	54.75	2
S(1): 0.0; T(2): 0.0; S(5): 100.0; S(9):		339.383958	44.06	2
T(2): 1.7; S(3): 98.3; S(9): 0.0; S(10):		215.9570476	62.41	2
S(3): 100.0; S(6): 0.0; S(8): 0.0; S(9):		261.6520864	59.45	2
S(1): 0.0; S(3): 100.0; T(8): 0.0; T(9):		232.1818073	34.82	2
S(8): 100.0		155.7613588	6.57	3
T(1): 0.0; S(3): 0.0; S(5): 0.0; S(10): 1		279.8312653	31.12	2
T(1): 0.0; S(3): 0.0; Y(5): 2.0; S(7): 98		239.16058	29.55	2
S(4): 100.0; S(10): 100.0		197.2228772	12.08	2
Y(1): 0.0; S(2): 100.0		188.1756743	19.4	2
S(1): 0.0; S(3): 100.0		143.8797642	16.21	2
S(5): 100.0		126.4849269	26.47	2
S(2): 0.0; S(3): 100.0; S(10): 0.0; S(1		214.5340547	40.32	2
S(1): 0.0; T(4): 0.0; S(7): 100.0		349.685818	32.54	2
S(9): 100.0		340.1454452	59.25	2
T(4): 0.0; T(9): 100.0		299.0377148	55.04	2
S(11): 100.0		318.0220989	55.82	2
S(1): 0.0; T(5): 0.0; S(12): 100.0; S(1		228.2606492	39.11	3
T(11): 0.0; T(15): 34.0; T(17): 34.0; S		191.3540591	31.77	3
T(6): 100.0; S(10): 98.2; S(12): 1.8; S		312.8245436	29.66	2
T(7): 50.5; S(8): 50.5; T(9): 99.1		252.0991938	35.32	3
S(3): 50.6; S(5): 50.6; S(6): 98.8		390.2437851	76.98	3
S(3): 100.0		339.263767	31.87	2
S(2): 2.6; S(3): 94.7; T(4): 2.6; Y(9): (		207.159428	31.32	2
S(7): 100.0		135.0228712	22.2	3
S(3): 100.0; S(8): 0.5; T(13): 99.2; S(		136.989449	10.64	4
S(3): 0.0; S(6): 0.0; S(7): 0.0; T(13): 1		264.8131589	53.27	3
S(4): 100.0; S(7): 100.0		270.5377089	38.04	3
S(5): 100.0; S(11): 100.0		218.6967651	15.33	3
S(3): 100.0; S(15): 0.0		219.7115449	27.53	3
T(1): 0.0; Y(2): 0.2; T(4): 99.8; S(8): 1		276.624894	76.03	3
S(7): 100.0		261.7634166	25.46	2
T(10): 0.1; S(13): 50.0; T(14): 50.0		179.1736927	23.93	3
S(16): 100.0		134.2197301	23.89	3
S(3): 100.0; T(5): 0.0; T(9): 0.0; T(10)		398.6719815	102.99	2
S(7): 99.8; T(11): 0.2; S(13): 0.0; S(1		216.4084106	45.8	3
T(1): 100.0; S(5): 100.0; S(7): 0.0; S(		291.1473504	31.4	2
S(15): 0.0; S(23): 100.0		411.3869265	42.94	3
S(6): 99.7; T(9): 5.2; S(10): 95.1; S(2		213.926539	36.47	3
S(3): 100.0; S(10): 0.0		221.851819	36.92	2
S(1): 0.0; S(2): 100.0; Y(7): 0.0		231.0535887	31.41	2
T(1): 0.5; T(12): 76.2; S(17): 5.8; Y(1		93.05377307	5.24	4
S(3): 100.0; S(5): 0.0; S(7): 0.0; T(8):		274.2662164	59.27	4

S(2): 0.0; S(9): 100.0		302.8567063	28.11	2
S(1): 0.0; S(3): 100.0; T(6): 0.0		170.159809	22.33	2
S(6): 100.0; S(7): 100.0		322.5594406	32.61	3
S(9): 99.9; T(11): 0.1		136.5439113	6.92	2
T(1): 0.0; S(3): 100.0		135.0983885	26.2	2
S(2): 100.0; Y(6): 0.0		173.8299312	40.85	2
S(4): 100.0; T(20): 0.0		347.0064475	93.8	3
S(5): 100.0; S(9): 0.0; T(10): 0.0; S(1		223.8552194	33.01	3
T(4): 0.0; S(12): 100.0		132.8535142	27.63	3
S(7): 100.0		358.4818544	49.38	2
S(1): 0.1; S(4): 2.7; S(5): 97.2; S(11):		141.7522452	17.98	2
S(2): 0.0; S(4): 100.0; S(7): 0.0; Y(13		350.0927051	71.67	2
S(4): 97.6; S(7): 2.4		136.7160237	12.93	2
S(13): 100.0		580.0282318	73.06	3
S(1): 0.0; S(3): 97.4; S(5): 51.3; S(6):		119.4247966	12.34	3
S(2): 0.0; T(3): 0.0; S(5): 0.0; S(6): 0.		239.8657168	64.34	2
T(1): 0.0; T(2): 0.0; T(5): 0.0; S(10): 1		187.9764477	9.89	3
S(1): 0.0; S(6): 0.0; S(8): 0.0; S(10): 1		185.3591601	29.73	2
S(2): 0.0; T(6): 0.0; S(7): 0.0; S(12): 9		411.9307715	86.67	2
S(1): 0.0; S(2): 4.1; S(4): 95.9; S(7): 0		184.8629276	45.57	2
S(3): 0.0; S(10): 100.0; S(13): 95.5; S		226.3100439	31.14	3
S(7): 100.0; T(22): 0.0		337.2471945	29.5	3
S(19): 3.9; S(20): 95.9; S(23): 0.2		255.1677921	49.51	3
T(10): 0.0; S(13): 100.0		308.5285818	69.98	2
S(3): 0.0; S(6): 100.0		304.9087949	62.72	2
S(9): 100.0; S(12): 0.0; S(13): 0.0; S(		340.8040972	93.05	2
Y(8): 100.0		202.6970186	28.05	2
T(3): 2.4; S(5): 97.6; T(7): 0.0; S(10):		249.3169542	51.85	3
Y(6): 0.0; S(10): 100.0		175.9390077	53.61	2
S(14): 0.0; S(21): 0.1; T(22): 3.3; S(2		312.5737361	61.63	3
S(4): 100.0; S(8): 50.0; T(10): 50.0; T		301.8113146	47.91	2
S(7): 100.0; S(10): 96.1; T(12): 3.9; S		198.9551792	30.96	3
S(1): 0.0; T(4): 49.5; S(5): 49.5; T(7):		178.2775633	48.59	2
S(5): 100.0; T(11): 0.0		325.0186866	50.98	2
T(3): 50.0; S(4): 50.0		66.72252032	17.37	3
S(4): 0.1; S(6): 99.9		212.3766679	22.69	3
S(3): 100.0; S(7): 100.0; S(13): 0.0		304.9708959	58.4	2
S(7): 2.5; S(8): 97.5		156.5568906	21.61	2
S(3): 100.0		141.7488053	17.67	2
S(1): 98.1; T(3): 1.9; S(7): 0.0		195.689473	43.42	2
S(5): 0.0; S(8): 98.1; T(9): 1.9		404.5690327	134.23	2
S(4): 100.0; S(7): 0.0		334.874843	61.36	2
T(5): 0.0; S(10): 98.5; T(11): 1.5		355.292723	68.72	2
T(1): 0.1; S(2): 99.9; S(6): 0.0		111.1855611	18.84	2
S(8): 100.0		282.6266183	36.14	2
S(5): 0.0; S(7): 100.0; S(9): 0.0		403.5415576	68.18	2
S(1): 100.0; S(15): 100.0		269.7266234	8.8	3
S(4): 0.0; S(5): 0.0; S(9): 100.0; T(12		361.6988178	61.61	2
S(1): 100.0; S(12): 100.0		375.8877993	75.04	2
Y(2): 82.3; S(4): 4.4; T(5): 4.4; S(6): 4		147.3710546	10.81	3
S(19): 100.0; S(21): 100.0; S(23): 100		362.5816471	43.67	3
S(4): 100.0; S(8): 0.0; T(9): 0.0; S(11		198.5125727	34.88	2
S(5): 96.0; S(6): 2.0; T(7): 2.0		167.0976926	18.58	2
S(10): 100.0		355.4790715	53.05	2
T(3): 0.0; S(4): 0.0; S(11): 0.0; S(16):		311.3608226	56.09	3

Information Classification: General

T(8): 0.0; S(14): 100.0		184.8819385	29.99	2
T(1): 100.0; S(5): 0.0; S(7): 0.0; S(9):		165.720378	24.65	2
S(6): 33.3; S(7): 33.3; S(11): 33.3		157.7488323	15.98	2
S(6): 100.0; S(8): 0.0		166.7118732	15.01	2
S(4): 100.0; T(20): 0.0		461.4400007	52.34	5
S(13): 100.0		213.7775891	64.02	3
Y(2): 0.0; S(9): 100.0		280.875648	36.58	3
Y(2): 0.0; Y(3): 0.0; S(4): 0.1; S(5): 9		307.5771971	45.32	2
S(3): 3.1; S(4): 96.9; S(8): 100.0		175.9876907	51.61	2
T(1): 0.0; S(3): 100.0; S(6): 0.0; S(8):		245.0656004	66.61	2
S(8): 100.0; T(13): 0.0; S(19): 0.0		522.6597516	131.87	2
S(5): 0.0; S(11): 100.0; S(15): 100.0		363.9573979	50.92	3
Y(3): 0.0; S(9): 100.0		234.4633602	52.45	2
S(1): 100.0; T(6): 0.0; S(9): 0.0; Y(10		556.7317893	130.94	2
S(2): 100.0; Y(12): 0.0; S(17): 0.0; S(		390.6678469	52.82	3
S(2): 0.0; S(4): 0.0; S(5): 0.1; S(8): 50		144.4619017	10.18	2
S(1): 90.3; S(2): 8.2; S(3): 0.9; S(4): (		10.76830108	4.12	3
S(6): 0.0; T(8): 0.0; Y(11): 0.0; S(15):		339.4065766	42.06	3
S(1): 96.1; T(2): 3.7; S(6): 0.2; S(10):		58.47417267	8.34	3
S(1): 0.0; S(4): 100.0; S(9): 0.0; S(14		228.5746349	49.54	2
S(2): 98.6; T(4): 90.9; S(6): 1.3; S(7):		103.5965353	9.82	3
S(1): 50.0; S(3): 50.0		315.0219304	40.67	2
S(1): 0.0; S(5): 0.0; S(10): 100.0; T(1		245.5017738	41.82	4
S(10): 100.0		440.8339882	84.67	2
S(3): 100.0		294.3149752	49.44	3
S(1): 0.0; T(10): 100.0; Y(19): 0.0		160.5436108	68.96	4
S(3): 100.0; T(9): 0.0		349.7447537	57.88	3
S(4): 100.0		226.2934306	36.13	2
T(5): 0.0; S(8): 100.0		338.4386617	74.43	2
S(2): 0.0; S(4): 0.0; S(5): 0.0; S(6): 0.		375.1069448	106.11	2
T(1): 98.1; S(4): 1.8; T(5): 0.0; S(6): (		174.1782447	49.27	2
T(2): 0.0; T(11): 0.0; Y(14): 0.0; T(21)		225.2049914	38.88	4
S(1): 0.0; S(10): 100.0; S(12): 99.9; T		264.5891844	27.14	3
S(4): 100.0; S(8): 0.0; T(9): 0.0; T(11)		116.5202245	12.7	2
S(4): 100.0; T(11): 0.0		404.9771053	39.07	2
S(1): 0.0; S(4): 100.0; S(8): 0.0		184.1352155	23.91	2
S(7): 97.3; T(8): 2.7; S(11): 0.0; S(17		240.0863614	33.48	4
S(4): 0.0; S(6): 98.0; S(8): 1.9; S(9): (		251.335635	36.19	2
S(8): 86.4; S(10): 6.8; Y(11): 6.8; S(1		113.2355843	2.73	4
S(5): 100.0; S(9): 0.0		108.1012436	13.24	3
S(5): 100.0		433.4961004	53.95	2
S(3): 0.0; S(16): 50.0; T(20): 50.0		169.256277	11.93	3
S(1): 0.0; S(5): 100.0; S(11): 100.0		149.0342759	18.37	3
T(3): 100.0; S(7): 0.0; S(9): 0.0; S(19		325.5854808	32.02	4
S(7): 100.0; S(10): 100.0		397.4814052	75.76	2
S(1): 100.0; T(3): 100.0		139.3357013	19.18	3
S(1): 0.0; S(2): 0.0; T(7): 100.0		217.1825707	46.71	2
S(8): 2.2; S(9): 47.8; T(10): 2.2; S(11		134.8047563	27.98	4
S(8): 99.8; T(13): 0.0; S(17): 0.2		127.4031744	8.21	3
S(5): 100.0; Y(7): 0.0		257.5125149	16.58	3
S(7): 100.0		221.5655249	34.14	2
S(4): 98.0; T(6): 2.0; S(7): 0.0		143.338785	25.97	2
S(3): 50.0; T(4): 50.0		192.472692	16.6	3
S(1): 0.0; S(2): 0.0; S(3): 100.0; S(6):		126.7973744	44.95	2
S(3): 0.0; S(8): 100.0		113.0479028	17.96	3

Information Classification: General

S(1): 97.1; Y(4): 2.9; T(5): 0.0; Y(13):	263.6017949	37.25	3
T(2): 0.0; S(3): 1.9; S(7): 98.1	99.71136148	17.21	2
T(2): 49.4; S(5): 49.4; T(7): 1.2; T(18)	404.5426014	38.47	3
T(4): 0.0; T(18): 0.0; S(22): 0.0; T(26)	234.112022	26.86	3
T(6): 0.0; S(14): 100.0	236.1536527	50.2	3
S(10): 0.0; T(17): 5.1; S(18): 5.1; S(1	207.1445884	31.69	4
S(12): 100.0; S(19): 0.0; S(22): 0.0; S	177.927484	16.1	3
S(5): 99.9; T(6): 0.1; S(7): 0.0; S(10):	134.9957803	27.1	3
S(3): 83.0; T(4): 4.3; T(5): 4.3; S(6): 4	99.43825003	17.46	3
T(1): 0.0; T(4): 100.0	360.3268733	65.32	3
T(1): 50.0; S(3): 50.0; S(8): 0.0; S(9):	204.8103116	28.52	3
T(1): 50.0; S(3): 50.0; T(5): 0.0; S(8):	118.1255136	19.73	3
S(4): 100.0; S(9): 100.0	515.4781781	98.9	2
S(3): 50.0; S(4): 50.0; S(10): 0.0; Y(1	334.2232264	78.86	2
T(1): 0.0; S(2): 0.0; S(14): 100.0	239.5154081	43.66	2
S(7): 0.0; S(15): 99.9; S(21): 50.0; Y(	251.5875954	45.28	4
S(1): 0.0; S(5): 100.0; S(7): 1.9; S(10	282.7667157	33.94	2
S(2): 1.6; S(3): 98.4	346.5417971	36.1	3
S(9): 100.0; S(12): 0.0; T(13): 0.0	222.0976863	20.22	3
S(3): 99.9; S(5): 0.1	247.9234608	39.47	3
S(3): 100.0; S(5): 100.0; S(9): 0.0; T(	300.452841	57.42	3
S(1): 0.0; T(5): 100.0	369.8811671	52.35	4
S(1): 0.0; T(7): 0.0; S(9): 0.3; S(11): 9	135.300258	7.31	3
S(2): 0.0; S(19): 0.0; S(22): 96.8; T(2	428.71477	55.02	3
T(2): 100.0; Y(7): 0.0	421.725242	76.23	2
S(2): 3.1; S(3): 3.1; S(5): 90.6; T(6): 3	141.4255158	15.94	3
Y(3): 0.0; S(4): 100.0	244.124619	73.41	2
S(3): 100.0; T(6): 0.0; S(7): 0.0; T(14)	263.0524842	36.87	2
T(1): 0.0; T(12): 0.0; S(14): 100.0; S(	477.4629164	66.8	3
T(3): 2.4; S(5): 97.6; S(9): 0.0; S(11):	174.2248491	25.64	2
S(7): 100.0; T(19): 0.0	257.0202758	51.09	3
S(6): 0.0; S(11): 100.0	264.4225229	34.07	3
S(1): 0.1; S(3): 99.9	194.8444397	24.84	3
S(1): 0.1; S(4): 99.9; Y(8): 0.0; T(13):	153.3998455	35.1	2
S(13): 100.0; T(17): 0.0	306.8367672	57.08	3
S(6): 100.0; S(12): 0.0	487.9276899	99.45	2
S(10): 100.0; S(14): 0.0	459.3963316	60.47	3
S(2): 20.0; T(3): 20.0; S(4): 20.0; S(5	80.79759801	10.08	2
S(1): 0.0; S(3): 98.2; T(4): 1.8; S(5): (	411.7276094	89.18	2
S(5): 0.0; S(9): 0.0; S(10): 0.0; S(13):	190.1949797	50.81	3
S(2): 0.0; S(5): 97.9; S(7): 2.1	117.1753494	32.92	2
S(1): 0.1; T(4): 2.3; S(6): 48.8; S(9): 4	224.5698883	39.72	3
S(4): 99.0; S(5): 50.5; S(6): 50.5	348.5083466	98.54	2
T(3): 0.0; S(12): 0.0; S(15): 100.0	192.8239571	32.39	3
S(4): 100.0; S(16): 0.0; Y(25): 0.0; T(	408.0191492	53.53	4
S(14): 50.0; S(15): 50.0; S(18): 0.1; S	349.5222121	73.83	3
T(4): 0.0; S(6): 100.0; S(9): 0.0; S(11	417.9981351	99.46	2
S(1): 0.0; S(5): 0.0; S(7): 0.0; S(9): 0.	300.859021	33.31	3
S(6): 100.0; T(12): 0.0	242.2301235	29.1	3
S(5): 99.9; S(7): 0.1	160.7488631	29.04	3
S(2): 0.0; T(3): 100.0; T(5): 0.0; Y(6):	195.4809781	69.83	2
S(7): 100.0; S(10): 0.0; S(11): 0.0; S(	242.5265709	45.43	3
S(2): 0.0; S(26): 0.0; S(29): 100.0	312.6194537	43.04	4
S(1): 0.4; S(2): 68.9; S(3): 5.0; S(7): 5	117.255066	2.81	3
S(1): 0.0; T(4): 0.0; S(7): 100.0	337.5501573	52.45	2

T(3): 50.0; S(4): 50.0; T(7): 0.0; S(8):	143.7630677	33.95	3
T(7): 0.0; S(8): 0.0; S(11): 100.0	201.2684412	34.6	3
S(5): 3.7; S(6): 96.1; S(19): 0.0; S(22	122.2524241	10.34	3
T(3): 100.0; S(11): 100.0	326.8137285	57.85	3
S(4): 0.0; S(5): 0.0; S(8): 0.2; S(10): 9	163.6873931	26.85	4
S(2): 0.0; S(8): 4.1; S(9): 91.8; S(17):	192.2044637	26.79	3
S(5): 4.2; T(8): 4.2; S(10): 91.6; S(12	117.521241	5.14	3
S(1): 0.0; S(5): 99.9; S(7): 0.1; S(11):	184.6801797	16.53	3
T(1): 0.0; T(9): 100.0; S(13): 0.0	161.2171853	12.78	3
T(1): 0.0; S(2): 0.0; S(4): 0.0; S(7): 10	261.016285	49.74	2
S(1): 49.0; S(3): 49.0; S(6): 2.0; T(19	141.8840926	9.46	3
S(2): 0.1; Y(3): 2.2; S(5): 97.8; S(8): 0	238.3509501	46.67	2
S(3): 49.9; S(5): 49.9; S(9): 0.2; T(15	138.7137103	17.84	3
S(2): 0.0; S(3): 0.0; S(5): 0.0; T(6): 0.	246.4014044	27.55	3
S(3): 100.0; T(10): 0.0; Y(11): 0.0	228.7500564	24.7	3
S(9): 100.0; S(13): 0.0	404.1441033	81.75	3
S(5): 100.0; S(7): 0.0; T(19): 0.0; T(2	277.2256207	66.75	3
S(6): 100.0	189.1083519	11.25	4
S(2): 0.3; S(5): 49.8; S(6): 49.8; S(11	132.0969975	23.51	4
T(8): 0.0; S(15): 100.0	485.2582222	87.41	3
S(10): 100.0; S(12): 0.0	446.1241486	116.25	2
S(2): 100.0; S(5): 0.0; S(16): 0.0	402.3655579	74.26	2
T(2): 0.0; S(4): 0.0; T(8): 1.7; S(9): 98	314.5421248	62.08	2
S(2): 0.0; S(4): 0.0; S(13): 0.1; S(16):	277.4265661	83.48	3
S(5): 0.0; T(8): 0.2; S(10): 99.8; S(12	174.3825658	16.98	3
S(1): 100.0; S(13): 0.0	298.7447891	48.98	3
S(2): 0.0; T(5): 0.0; Y(25): 3.7; S(26):	242.058294	58.24	3
S(4): 50.0; S(7): 50.0	167.5583663	22.01	2
T(2): 0.0; T(13): 7.1; S(15): 62.0; S(1	132.4614564	23.29	4
S(6): 100.0; S(12): 0.0	461.7035434	80.72	2
S(3): 97.9; Y(4): 2.1	250.3091192	35.81	3
T(1): 0.0; T(4): 100.0; S(17): 98.4; S(	676.701874	68.54	3
S(2): 0.1; T(7): 1.1; T(15): 2.4; S(21):	108.6204847	2.52	5
S(2): 0.0; Y(3): 0.0; T(5): 0.0; S(9): 10	335.0809127	82.35	2
T(6): 100.0; T(11): 0.0	328.5655323	65.7	2
T(1): 93.8; S(2): 3.1; T(3): 3.1; T(7): 0	284.3755462	53.81	3
S(1): 0.0; S(3): 0.0; S(5): 100.0; S(7):	428.6195038	88.93	2
S(2): 94.7; S(3): 2.6; T(4): 2.6; S(6): 0	238.8020206	19.8	3
S(3): 0.0; T(9): 0.0; S(11): 100.0; T(1	247.9234608	53.92	3
S(3): 100.0; T(6): 0.0; S(7): 0.0	143.8797642	41.49	2
T(1): 0.0; Y(2): 0.0; S(11): 100.0; S(1	377.2102688	67.23	3
Y(2): 0.0; S(6): 100.0	208.6594248	27.83	2
S(1): 96.9; T(4): 6.2; S(5): 96.9	148.5731765	24.93	2
S(4): 2.3; S(5): 48.8; S(6): 48.8; S(15	98.55508522	25.65	3
T(16): 100.0	386.8919642	50.56	3
S(1): 0.0; T(3): 5.5; S(4): 5.5; S(7): 88	133.6220996	32.2	3
S(11): 100.0; T(17): 0.0; S(18): 0.0	295.4343955	73.77	3
Y(4): 0.0; S(7): 100.0	188.7625902	41.22	2
S(1): 0.0; T(2): 0.0; S(5): 0.0; S(7): 0.	368.8166081	67.25	3
S(2): 0.1; S(5): 99.9	267.8135122	55.94	3
S(4): 0.3; S(10): 89.9; S(16): 4.9; Y(1	92.63883521	22.18	3
S(3): 100.0; S(8): 100.0; S(12): 0.0	203.3513706	4.33	4
S(8): 99.9; T(10): 0.0; S(11): 0.1; S(1	222.97425	63.56	3
S(3): 100.0; S(6): 0.0; S(20): 0.0	584.651382	98.26	3
S(10): 2.8; S(11): 97.1; S(14): 0.1	246.8358841	43.95	3

Information Classification: General

T(3): 0.0; S(6): 0.0; T(7): 0.0; T(8): 0.	151.4177574	32.09	3
S(2): 0.0; S(12): 0.0; S(17): 0.4; T(19	222.478202	41.61	3
T(1): 0.0; S(3): 0.0; S(14): 100.0	139.8961483	34.19	3
S(1): 0.0; S(5): 100.0; T(8): 2.2; S(9):	274.3227637	30.88	2
S(4): 0.0; S(7): 0.0; S(11): 99.5; S(14	88.66585438	2.72	3
S(3): 100.0	175.4569682	21.3	4
S(7): 0.0; S(21): 100.0	289.7947435	55.2	3
S(5): 95.4; S(7): 2.3; S(8): 2.3	229.6808718	32.18	2
Y(3): 0.0; S(5): 100.0; S(15): 0.0; T(2	501.0794699	106.4	2
S(1): 0.0; S(4): 2.8; S(5): 97.2; S(10):	314.7143492	83.16	3
T(1): 0.0; S(3): 0.0; T(5): 0.0; S(6): 0.	271.1358804	29.23	3
S(8): 0.0; Y(11): 0.0; Y(12): 0.0; S(17	333.6280153	65.23	3
S(4): 0.0; S(8): 0.0; S(11): 100.0; S(1	256.1076188	55.4	3
S(6): 0.0; S(7): 0.0; S(9): 0.1; S(10): 9	241.5698716	38.49	3
S(6): 100.0; S(11): 0.0	318.0418486	58.57	2
S(1): 100.0; S(4): 0.0; S(5): 0.0; S(8):	264.6248511	67.22	3
S(2): 0.0; S(4): 50.0; S(5): 50.0; S(8):	201.9353509	40.27	2
S(2): 100.0; T(4): 0.0	266.4274262	65.37	2
S(3): 0.0; S(4): 0.0; S(9): 0.0; S(15): 1	362.5101852	58.22	4
S(2): 0.2; S(6): 99.8; S(8): 99.8; S(10	154.7628984	18.14	3
S(8): 0.0; T(13): 0.0; T(14): 0.0; S(21	209.4989852	61.57	2
S(9): 100.0; Y(15): 0.0	265.9068094	48.99	3
T(1): 0.0; S(12): 100.0; S(16): 0.0; S(	326.4672483	44.03	3
S(1): 0.0; T(2): 100.0	201.925029	21.1	2
S(3): 100.0; T(10): 0.0; S(19): 0.0	317.7515789	23.05	3
T(6): 0.0; T(11): 0.0; S(15): 100.0; T(	388.3504779	74.31	3
Y(1): 0.0; T(12): 0.0; Y(18): 0.1; S(21	299.6725114	30.8	3
S(4): 0.3; S(5): 99.5; S(7): 50.1; S(9):	68.47122253	12.49	2
S(1): 0.0; S(3): 100.0; Y(5): 0.0; T(10	210.4156311	24.63	2
T(1): 0.0; S(3): 100.0; S(6): 0.0; T(10	317.5112061	55.62	2
Y(2): 0.3; S(5): 94.9; S(7): 4.8; T(8): 0	196.0067158	26.07	3
S(7): 2.2; S(9): 97.8	162.8477155	25.94	3
S(1): 0.0; S(3): 49.2; S(4): 49.2; S(5):	244.5853965	39.15	3
S(3): 0.0; T(8): 0.0; S(12): 0.0; T(15):	388.4618907	164.05	2
S(5): 0.0; S(8): 100.0; S(13): 0.0; S(1	330.1292692	97.87	2
S(8): 100.0; S(12): 0.0; S(15): 0.0	280.078175	43.95	3
S(12): 100.0	158.2300521	16.67	4
S(2): 0.0; T(10): 0.0; S(13): 100.0	299.3255392	54.9	2
S(8): 100.0; S(14): 0.0; T(15): 0.0	350.2518121	34.5	3
S(1): 0.0; S(3): 0.0; S(7): 0.0; S(14): 9	141.895494	31.4	3
S(3): 50.0; S(6): 50.0	233.1508757	56.24	2
S(2): 0.0; S(3): 0.0; S(5): 0.0; S(8): 2.	246.3702944	51.71	2
S(7): 100.0; S(11): 0.0	456.5178594	94.36	2
S(6): 100.0	485.5663132	74.35	2
S(1): 2.0; S(2): 32.7; S(3): 32.7; S(12	55.122435	3.91	2
S(4): 100.0; S(5): 100.0; S(13): 0.0	322.5588604	59.8	2
S(10): 100.0	371.3872394	115.51	2
S(2): 0.0; T(10): 0.0; S(13): 0.0; S(24	262.0475791	25.65	4
S(3): 2.9; S(4): 94.3; S(5): 2.9; S(15):	216.7012507	42.84	3
S(7): 100.0	406.1185715	52.47	2
S(2): 0.0; S(4): 0.0; S(5): 100.0	342.9238318	89.7	2
S(4): 100.0	197.4440519	17.98	3
S(1): 0.0; S(14): 100.0; T(20): 0.0	350.3574877	76.05	2
S(6): 100.0; S(11): 0.0	460.3703337	90.71	2
S(1): 50.0; Y(15): 100.0; Y(20): 50.0;	197.6690128	29.13	3

Information Classification: General

T(2): 0.0; S(4): 0.0; S(5): 0.1; S(6): 0.	290.9729982	32.34	3
S(3): 0.0; S(6): 100.0; S(7): 100.0	160.752893	16.64	2
S(5): 0.2; T(7): 0.0; T(8): 0.0; T(14): 9	189.7444101	35.01	3
S(4): 100.0	267.1059548	25.91	3
T(1): 0.0; S(2): 0.2; S(3): 99.8; S(11):	209.5979586	11.94	3
S(4): 100.0; S(10): 0.0	382.3943268	55.67	2
T(7): 0.0; S(10): 0.0; T(11): 0.0; S(12	430.8713156	74.58	3
S(1): 0.0; T(3): 0.0; T(8): 0.0; T(9): 0.	424.1062081	52.99	2
S(1): 0.1; S(3): 0.1; T(8): 99.8; S(15):	208.8703907	14.7	3
S(4): 100.0	195.8772665	23.12	2
S(3): 0.0; S(10): 4.4; T(11): 95.6	76.30709932	8.06	3
T(4): 0.0; T(12): 100.0; S(14): 0.2; S(	248.0021129	40.21	4
S(2): 1.5; S(4): 98.5	284.3420483	42.06	3
Y(3): 100.0	39.94393915	7.31	2
T(3): 98.1; S(4): 1.9	157.5298567	9.71	3
S(3): 0.0; T(9): 100.0	335.6704765	61.94	2
T(2): 0.0; T(6): 0.0; S(7): 0.0; T(9): 33	190.9729789	39.4	2
S(8): 0.0; T(9): 0.0; S(12): 100.0	229.9967833	26.63	3
S(5): 100.0	92.87993885	9.37	3
S(12): 100.0	389.6460927	109.31	2
S(8): 32.3; S(9): 32.3; S(11): 32.3; T(	140.4481597	30.7	4
S(3): 0.0; S(12): 0.0; S(14): 0.0; S(15	222.2796547	38.66	4
S(4): 100.0; S(10): 0.0; S(12): 0.0	163.2181324	25.61	2
S(1): 3.4; T(3): 96.6; S(6): 0.0; S(9): 1	310.3370326	35.63	3
S(1): 0.0; S(4): 100.0; S(9): 0.0	281.3343125	52.26	2
S(3): 100.0; T(9): 100.0; S(18): 0.0; Y	437.8490254	44.26	3
Y(7): 0.0; S(10): 100.0	111.1329107	24.42	3
S(2): 0.0; S(7): 100.0; S(11): 100.0	217.606532	52.83	3
T(1): 100.0; Y(7): 0.0; S(8): 0.0; Y(9):	334.2230244	57.5	2
S(1): 0.0; S(3): 100.0; S(7): 0.0; Y(10	356.7426047	78.49	2
S(10): 0.0; S(13): 97.3; S(15): 2.6; T(	274.2925365	36.68	3
S(8): 100.0	197.986032	24.25	3
S(7): 100.0; T(19): 0.0	381.5459669	76.54	3
S(13): 3.7; S(14): 96.3; T(17): 0.0	206.5080949	32.26	3
T(1): 0.0; S(3): 99.9; S(5): 0.0; S(6): 0	363.7388487	66.75	2
S(1): 100.0; S(8): 0.0; S(12): 0.0	186.0704181	13.8	3
T(1): 0.1; T(2): 2.6; T(3): 2.6; T(4): 94	74.78467281	14.68	2
S(3): 100.0; S(12): 0.0	142.163475	3.1	3
T(3): 50.0; T(4): 50.0	141.693845	25.19	2
T(5): 0.0; S(7): 0.0; S(9): 100.0; T(10	201.8073673	30.65	3
S(8): 100.0	246.1037732	38.41	2
T(1): 0.0; S(2): 100.0; T(6): 0.0	307.799894	66.29	2
S(2): 100.0	135.6223004	12.02	3
S(11): 24.7; S(12): 24.7; S(15): 24.7;	69.73286801	6.08	7
S(4): 100.0; T(6): 0.0	201.8693738	1.07	3
S(2): 0.0; S(8): 0.1; S(14): 99.9	180.5037795	21.35	2
T(4): 0.0; S(5): 0.0; S(6): 0.0; T(8): 0.	348.8414899	75.76	2
S(1): 0.0; S(7): 100.0	414.4334224	50.94	2
S(3): 100.0	115.2524925	9.56	3
S(3): 3.3; S(4): 96.7; S(10): 0.0; Y(11	231.7882065	84.25	3
T(1): 0.0; T(3): 0.0; T(9): 100.0; S(10	220.0699659	43.03	3
T(6): 0.0; T(10): 0.0; T(12): 0.0; S(13	242.5900797	49.81	2
S(11): 98.0; S(13): 2.0	266.4042632	35.54	2
T(5): 3.2; S(6): 3.2; S(8): 3.2; T(9): 3.	147.7623008	35.83	2
S(7): 100.0; S(10): 100.0; S(11): 100.	214.7754747	37.62	3

Information Classification: General

S(8): 50.0; S(10): 50.0		335.5300859	23.24	3
S(7): 50.0; T(9): 50.0		74.28460183	3.81	2
S(1): 0.0; S(3): 100.0; T(9): 0.0		284.5195934	49.81	2
S(1): 100.0; T(10): 0.0; S(12): 0.0		156.5568906	31.26	2
S(1): 100.0; S(7): 0.0		154.7969126	33.12	2
S(3): 99.9; S(5): 0.1; S(10): 0.0		198.6439958	37.54	3
S(2): 0.0; Y(4): 0.0; S(8): 100.0		195.8772665	25.44	2
Y(5): 0.0; S(15): 100.0		461.1248437	42	3
S(1): 100.0		215.5953725	44.72	2
T(6): 0.0; T(12): 100.0		396.851371	73.66	2
S(2): 100.0; S(4): 0.0; S(6): 0.0; S(9):		131.486014	43.43	2
S(3): 0.0; T(5): 98.0; S(8): 2.0; S(9): 1		212.7320643	31.1	2
S(7): 50.0; Y(9): 50.0; Y(13): 0.0		211.159318	47.63	3
S(8): 50.0; T(10): 50.0		283.3202793	60.45	2
S(1): 0.0; T(7): 0.1; S(8): 99.9		164.347307	23.15	3
S(14): 100.0; T(18): 0.0		539.6078149	91.22	2
T(8): 0.1; S(10): 99.9		240.2933414	35.27	3
S(1): 0.0; S(3): 100.0; S(8): 0.0; S(10		253.9466912	47.02	3
S(1): 0.0; S(4): 1.8; S(5): 1.8; T(6): 0.		260.8700284	77.65	2
S(3): 100.0; S(8): 0.0; Y(10): 0.0		179.4026409	28.36	3
S(3): 100.0; S(8): 0.0		199.3940828	32.78	3
T(1): 0.0; S(2): 0.0; S(6): 0.0; S(7): 0.		167.3617287	31.44	3
S(3): 3.7; S(4): 96.0; S(7): 0.2; S(8): (		105.8430515	5.29	2
S(6): 0.0; S(8): 100.0		278.8870458	67.62	2
S(5): 1.7; S(7): 98.3; S(9): 0.0; S(11):		254.9023815	37.11	2
S(3): 100.0; T(8): 0.0		441.6423955	54.19	3
S(1): 0.0; S(2): 0.0; S(3): 0.0; S(4): 10		383.0036078	59.38	3
S(19): 95.6; S(20): 4.2; S(23): 0.2		211.8851335	42.73	3
S(1): 0.0; T(2): 0.0; T(7): 0.0; S(10): 5		215.2171768	34.77	3
T(2): 0.0; T(3): 0.0; S(7): 100.0; T(11)		189.1140309	20.06	3
T(1): 0.0; T(3): 0.0; S(24): 100.0		303.063419	32.3	3
S(1): 0.0; Y(2): 0.2; S(3): 96.0; S(9): 3		115.2749432	9.9	3
Y(6): 0.0; S(11): 2.7; S(12): 97.3; S(1		349.101832	83.96	3
S(1): 100.0; S(7): 100.0		432.3823186	33.16	3
S(3): 100.0		432.0122324	94.95	2
S(3): 0.0; T(6): 100.0		189.3233421	19.08	2
S(4): 0.0; S(5): 0.0; S(6): 0.0; S(10): (		307.1902053	36.01	3
T(1): 0.0; S(3): 100.0; T(6): 0.0; T(9):		374.4774569	113.58	2
T(8): 1.3; Y(9): 1.3; T(10): 97.4		352.3871508	49.81	2
S(2): 0.0; S(5): 0.0; S(6): 100.0; T(15		299.5555883	45.04	3
T(5): 97.6; S(6): 2.4		164.7203397	33.09	3
S(4): 32.2; T(5): 32.2; S(7): 32.2; S(1		65.25726279	3.52	4
T(1): 96.4; S(3): 3.6; S(8): 0.0; S(10):		235.5149659	40.44	3
T(1): 50.0; T(3): 50.0; T(7): 0.0; Y(10)		233.1479488	43	3
S(5): 2.7; S(6): 2.7; S(7): 94.6		171.7250152	33.29	2
S(1): 0.0; S(3): 100.0; S(5): 0.0		112.0421023	24.89	2
T(4): 100.0; S(14): 0.0; T(15): 0.0; T(		267.7270871	34.56	3
T(1): 0.0; S(3): 4.2; T(5): 4.2; S(12): 9		235.4943527	46.76	3
S(8): 100.0; S(10): 0.0; S(11): 0.0; T(		383.3325929	97.76	2
T(13): 100.0; S(20): 0.0		302.0404491	29.69	3
T(6): 1.9; S(8): 1.9; S(9): 96.3		276.9145649	48.2	2
T(2): 0.0; T(6): 0.0; S(10): 100.0		307.5246689	66.92	2
S(5): 100.0; S(17): 0.0		328.0886723	62.6	3
S(6): 97.7; S(7): 2.3		217.3395506	48.84	3
S(4): 100.0; S(8): 0.0		183.8490556	19.87	2

Information Classification: General

S(14): 100.0		249.818299	53.19	2
S(13): 100.0; S(18): 0.0		175.0594873	32.01	3
S(2): 100.0; S(4): 0.0		310.0778825	54.3	2
S(1): 0.0; S(7): 100.0; S(16): 0.0; S(1		298.9757102	33.04	3
T(1): 97.9; S(2): 2.1		114.9437311	19.68	2
S(3): 100.0; S(8): 0.0; S(9): 0.0		187.1489404	13.69	3
S(6): 100.0; T(12): 0.0; T(14): 0.0		196.2802825	33.03	4
S(5): 48.0; T(6): 48.0; T(10): 2.2; T(1		113.1348449	1.29	3
S(4): 100.0; S(9): 0.0; T(14): 0.0		266.0058679	60.58	2
S(4): 1.5; S(5): 98.4; S(6): 0.0		328.0640661	73.92	2
T(2): 50.0; S(3): 50.0; S(7): 0.0		141.7488053	46.97	3
S(5): 0.0; S(11): 100.0; S(15): 0.0		574.2540584	80.4	3
S(2): 0.0; S(4): 100.0; S(7): 0.0		211.7505123	23.23	2
S(7): 100.0; S(10): 100.0; S(11): 100.		221.765338	29.03	3
S(3): 100.0; T(13): 0.0		233.7359134	16.82	4
T(1): 0.2; S(2): 0.2; S(3): 99.4; S(6): 5		171.1064779	19.23	3
S(2): 0.0; S(7): 0.5; S(8): 0.5; T(9): 99		138.1454373	7.37	3
Y(8): 0.0; S(10): 100.0; S(14): 0.0		207.115418	15.65	4
S(2): 0.0; S(6): 0.3; S(9): 0.3; S(10): 9		86.92976768	11.55	3
T(3): 100.0; S(5): 100.0; S(15): 0.0		153.6195428	15.78	3
T(1): 2.8; S(2): 97.2; S(4): 100.0; T(7)		270.7904187	57.69	2
Y(2): 0.0; S(8): 100.0		212.8547565	37.58	2
Y(3): 0.0; S(5): 0.0; S(10): 100.0		205.5800607	29.54	3
S(1): 0.0; T(2): 0.0; S(3): 100.0		229.257805	43.63	2
S(2): 0.0; S(26): 100.0; S(29): 100.0		66.48344981	5.63	4
T(3): 94.9; T(4): 5.1; S(8): 0.3; S(12):		69.64017231	13.33	4
S(1): 0.0; S(3): 0.0; S(4): 0.0; Y(5): 0.		185.0441444	21.95	3
T(4): 0.0; S(6): 100.0; S(18): 0.0		175.187724	27.21	3
S(2): 0.0; T(6): 0.0; S(8): 0.0; S(9): 0.		240.3068307	25.86	3
S(8): 2.3; T(10): 2.3; S(11): 95.3; S(1		216.1575181	26.67	2
S(5): 90.2; S(7): 9.8; S(11): 9.8; S(16		85.62532098	9.4	3
S(6): 100.0; T(18): 0.0		456.0858749	67.13	3
S(3): 1.6; S(5): 98.4; Y(7): 0.0		139.8436989	37.27	2
S(5): 100.0; S(8): 0.0; S(9): 0.0; T(11		332.8217638	41.71	2
S(5): 0.0; S(6): 0.0; S(11): 3.2; S(13):		324.3597746	66.82	3
S(3): 97.9; S(5): 51.1; S(6): 51.1; T(1		173.9321298	32.42	3
S(4): 100.0; S(10): 0.0; S(11): 0.0		244.8352885	44.65	2
S(8): 100.0		80.07495775	10.73	4
T(7): 0.0; T(10): 0.0; S(11): 0.0; S(14)		330.3921217	59.09	2
S(4): 100.0		450.1976089	37.91	3
S(7): 1.8; S(9): 1.8; S(10): 96.4; T(11		284.9931383	58.77	2
Y(2): 0.0; S(4): 0.0; S(5): 0.0; S(7): 0.		137.7244629	14.83	3
T(1): 0.0; T(4): 0.0; Y(9): 0.0; S(12): 0		282.4029216	70.58	3
Y(3): 0.0; S(8): 100.0		309.6393537	45.38	2
S(3): 0.0; S(5): 0.0; S(12): 100.0		178.4487297	33.24	2
T(3): 0.0; S(5): 0.0; T(7): 0.0; S(8): 0.		305.0047458	24.24	3
S(1): 0.0; S(3): 100.0; T(10): 0.0; S(1		260.1873092	35.89	2
S(8): 0.0; S(14): 0.0; T(18): 50.0; S(2		213.2628157	34.44	3
S(1): 2.8; S(4): 97.2; T(13): 0.0		269.8923713	38.98	3
S(15): 1.6; S(16): 98.4		363.8338593	92.47	2
S(4): 100.0; T(6): 0.0		213.698223	18.12	2
T(1): 0.9; S(4): 49.5; S(7): 49.5; S(13		227.7403204	29.93	3
T(3): 0.0; S(18): 100.0		528.4580621	89.64	3
T(4): 66.7; S(7): 66.7; S(11): 66.7		86.65114447	16.83	2
T(4): 0.0; S(7): 100.0		275.0631537	43.81	2

Information Classification: General

S(4): 0.0; S(6): 0.0; T(14): 100.0; S(2	405.4555561	62.42	3
S(5): 100.0; T(9): 0.0; Y(11): 0.0	107.3867004	18.64	3
S(3): 0.0; S(4): 0.0; T(8): 100.0	220.8340872	29.9	2
S(2): 100.0; S(5): 100.0; Y(10): 0.0; S	303.8223731	31.5	3
S(2): 0.0; S(6): 100.0	308.3063692	35.05	2
S(8): 100.0	216.7781383	23.48	3
S(2): 0.0; S(7): 100.0; S(17): 0.0; T(2	213.6345821	40.93	3
T(12): 98.8; S(13): 1.2	336.050503	49.91	3
S(14): 100.0	388.7459072	71.11	2
S(1): 33.3; S(2): 33.3; S(3): 33.3; T(6	204.5503841	19.29	4
T(1): 0.0; T(3): 0.0; S(6): 1.9; S(7): 98	180.9033139	33.07	2
S(1): 0.0; T(3): 0.0; S(5): 0.0; T(6): 0.	275.0648519	45.18	2
S(3): 100.0	202.7803844	34.08	3
T(1): 0.0; S(3): 100.0; S(5): 0.0; S(7):	152.0768185	39.55	2
T(1): 0.1; S(5): 100.0; S(11): 99.9; S(	118.5366763	15.07	3
S(1): 0.0; S(5): 4.7; S(6): 4.7; S(9): 90	50.10440304	2.12	3
T(2): 0.0; T(4): 0.1; S(7): 96.5; S(11):	230.2967356	40.54	3
S(10): 100.0	428.7799489	49.28	2
S(3): 100.0; T(6): 0.0; T(7): 0.0; T(8):	238.5611848	45.93	2
S(1): 0.0; S(3): 100.0; T(5): 0.0; S(9):	282.3192842	48.22	2
T(2): 0.0; S(8): 0.0; S(12): 0.0; T(20):	234.3229984	41.62	3
S(2): 100.0; S(7): 0.0; Y(10): 0.0; T(1	239.3739048	60.33	2
S(1): 0.0; S(3): 0.0; T(9): 96.2; T(10):	183.3435415	20.18	2
S(2): 0.0; S(4): 100.0	106.2145471	10.53	2
T(13): 0.0; S(23): 100.0	325.7815663	40.59	5
S(1): 0.0; S(3): 1.9; S(4): 98.1; T(6): 0	257.3362739	86.68	2
T(7): 0.0; S(8): 0.0; S(13): 0.0; S(16):	275.2977931	92.49	2
T(7): 50.0; S(8): 50.0	152.2403165	36.06	2
S(1): 100.0; S(4): 0.0	180.1934766	29.62	2
S(3): 0.0; T(13): 96.2; T(16): 3.8	153.4204193	14.03	4
S(3): 3.8; S(5): 96.4; S(9): 99.9	195.5330625	18.95	3
S(3): 100.0; S(7): 0.0; T(14): 0.0	440.3131797	87.04	2
S(4): 0.0; S(7): 100.0	261.9759442	37.76	2
S(5): 5.0; S(9): 5.0; S(10): 85.0; S(13	191.6872812	31.33	3
T(1): 0.0; S(2): 0.0; S(4): 100.0	264.2153396	38.53	2
S(2): 100.0; S(4): 100.0; T(6): 0.0	115.4736806	6.88	4
S(3): 0.0; T(6): 100.0	240.2474807	28.36	3
T(2): 0.1; T(3): 99.9; T(5): 0.0; T(8): 0	186.6900396	14.73	2
S(5): 0.0; S(9): 0.0; S(15): 97.8; Y(16	247.215131	53.9	3
T(2): 0.0; T(4): 0.0; S(9): 100.0; S(15	418.6281004	59.6	3
S(4): 50.0; S(5): 50.0; S(7): 0.0; S(9):	159.6197129	33.91	3
S(15): 100.0	354.3665989	54.7	3
S(1): 100.0; T(4): 0.0	193.0883759	65.03	2
S(2): 0.0; S(10): 49.1; S(11): 1.7; S(1	308.5133077	91.7	3
T(1): 0.0; S(3): 100.0; Y(7): 0.0; S(8):	116.1915856	15.38	2
S(7): 100.0; S(15): 100.0; S(19): 0.0	435.7128955	70.29	2
S(8): 100.0	256.3409149	26.72	3
Y(5): 0.0; S(9): 100.0	247.9541348	53.56	2
S(1): 0.0; S(3): 100.0; Y(14): 0.0	306.9793491	42.32	3
T(1): 0.0; S(12): 100.0	176.0781729	40.94	2
S(2): 100.0	228.4448625	9.31	3
S(1): 50.0; Y(2): 50.0; Y(16): 0.0; S(1	188.1611573	6.16	3
S(3): 100.0; S(12): 92.6; S(14): 7.4	91.20153964	2.84	3
S(9): 50.0; T(11): 50.0; T(20): 50.0; S	99.37126559	17.17	3
S(8): 0.0; S(10): 100.0; S(15): 0.0	241.045141	24.96	3

Information Classification: General

T(1): 0.0; S(3): 1.3; S(4): 49.4; S(8): 4	133.2860638	24.23	2
S(3): 100.0; T(6): 100.0; S(13): 0.0	142.3268357	20.37	3
T(1): 0.0; S(8): 99.9; S(12): 0.1	166.9039695	51.6	3
S(15): 33.3; Y(18): 33.3; S(19): 33.3;	148.8981172	26.33	4
Y(7): 0.0; Y(8): 0.0; T(10): 0.2; T(11):	201.8448926	31.32	3
S(5): 100.0; T(7): 0.0; T(10): 0.0	283.7802726	43.47	2
S(3): 1.4; S(5): 98.6; T(11): 0.0	305.9172464	54.23	2
Y(1): 0.0; S(9): 99.9; S(12): 0.0; S(13	245.1660177	42.68	4
T(1): 0.0; T(5): 0.0; S(6): 0.0; S(8): 0.	381.8654446	74.59	2
S(1): 0.2; S(3): 99.8; S(7): 3.6; S(9): 9	118.1828046	3.74	2
T(1): 0.0; S(16): 100.0	267.9793741	42.68	3
S(6): 95.4; S(7): 4.6; T(14): 0.0; S(19	210.0244947	56.85	3
S(1): 3.8; S(3): 96.2; S(5): 3.8; S(8): 9	277.7549641	37.08	3
Y(1): 0.0; T(9): 98.5; S(10): 1.5	236.8486549	21.06	2
S(2): 0.0; S(4): 96.7; S(7): 3.3	100.1463746	22.57	2
S(2): 0.1; S(4): 99.9; Y(10): 0.0	144.0610795	18.59	2
S(1): 0.0; S(3): 100.0; T(5): 0.0	202.8098917	35.11	2
S(4): 0.0; S(5): 0.0; S(8): 0.0; S(13): 0	140.8811198	9.84	3
S(8): 100.0	296.4788347	23.27	2
S(4): 100.0	148.6246441	16.67	2
S(2): 50.0; S(4): 50.0; S(6): 0.0; S(9):	340.5129189	59.08	3
T(3): 3.2; S(8): 96.8; S(12): 100.0; S(	225.1811633	41.17	3
S(3): 50.0; S(6): 50.0; S(12): 0.0; S(1	249.3503794	23.96	3
S(3): 100.0; S(8): 0.0; S(13): 0.0	345.4917078	41.93	3
S(2): 0.0; S(4): 0.0; S(8): 98.0; S(10):	204.3145703	32.71	2
S(1): 1.5; S(3): 32.3; S(5): 32.3; S(6):	132.6890679	5.96	3
T(7): 100.0; S(8): 0.0	273.5469659	37.48	2
S(1): 98.4; S(3): 1.6; S(8): 0.0	179.2025997	22.33	3
S(1): 0.0; S(3): 100.0; S(5): 0.0; S(6):	164.5504248	29.28	2
T(10): 99.9; S(13): 50.1; T(14): 50.1	152.4876261	5.15	3
T(1): 0.1; T(5): 96.5; T(7): 3.4; Y(8): 0	209.9693077	68.23	3
T(1): 0.0; T(4): 0.1; S(5): 0.1; S(6): 0.	144.8787001	29.9	3
Y(3): 0.0; T(4): 0.0; T(8): 0.0; S(12): 0	173.981707	39.96	3
S(5): 100.0; S(17): 0.0	108.9924885	24.96	3
S(1): 0.1; S(2): 99.9; T(3): 0.1; T(7): 0	245.5640382	76.49	2
S(5): 100.0	139.5997831	22.54	2
S(2): 1.7; S(3): 98.3; S(7): 0.0	150.8326265	28.42	3
S(4): 100.0; S(8): 100.0; T(10): 0.0; S	212.1617369	52.71	3
S(1): 100.0; S(8): 0.0; T(9): 0.0	412.5520147	41.32	2
S(4): 100.0; T(7): 0.0	309.0562589	69.02	2
S(3): 100.0; S(10): 0.0; S(13): 0.0; S(	438.6521727	122.83	2
S(3): 100.0; S(5): 100.0	160.9138875	9.02	2
T(1): 0.0; S(5): 100.0; T(8): 100.0; S(	340.5066813	66.68	2
S(3): 100.0; T(7): 0.0	208.5514322	21.3	3
S(4): 0.0; S(10): 100.0	308.8298003	89.74	2
S(1): 0.0; S(3): 100.0; S(5): 0.0; Y(6):	169.9528285	19.02	2
S(1): 0.0; T(3): 0.0; T(4): 0.8; T(5): 49	228.7406406	74.67	2
S(7): 100.0	317.7725689	18.36	2
T(1): 0.0; T(3): 100.0; T(11): 0.0	203.9912269	29.28	2
S(3): 50.0; Y(5): 50.0	190.3034141	40.07	2
S(3): 50.0; Y(4): 50.0	94.46872383	7.94	2
Y(1): 0.0; S(8): 100.0	129.0435085	4.32	3
T(3): 0.0; Y(7): 3.1; S(9): 96.9; T(17):	178.4780478	39.61	3
T(4): 0.7; T(8): 9.6; S(10): 58.2; S(12)	192.8652733	6.38	3
S(9): 1.4; T(10): 1.4; S(11): 47.9; S(1	302.1271115	98.41	3

T(1): 50.0; S(2): 50.0; S(11): 0.0; T(1	159.3211544	10.95	3
T(3): 100.0; S(5): 100.0	317.0274886	60.61	2
S(3): 1.1; S(4): 49.5; S(5): 49.5; Y(10	307.7619974	79.84	2
T(1): 0.0; S(4): 0.0; T(11): 100.0; S(1	219.2564279	14.37	3
T(1): 0.0; S(6): 0.0; S(10): 100.0; S(1	279.5126079	51.77	2
S(1): 0.0; S(10): 100.0	311.6171181	61.25	2
T(6): 100.0	144.3567633	14.04	2
S(1): 0.0; T(5): 50.0; S(6): 50.0; T(10	229.7573261	58.21	2
S(3): 100.0; Y(6): 0.0	432.4626105	62.43	2
S(1): 0.0; S(3): 0.0; S(7): 100.0; S(9):	334.905655	34.56	2
S(6): 100.0	241.8920289	42.71	2
T(5): 100.0; S(7): 100.0	153.4424178	13.85	4
Y(1): 0.0; T(6): 99.0; T(9): 0.3; T(11):	150.3497699	22.17	3
S(2): 0.0; T(4): 0.0; Y(7): 0.0; S(12): 1	223.3698641	14.48	3
S(13): 4.7; T(15): 95.3	164.8653699	9.45	4
S(1): 0.0; S(2): 0.1; S(3): 99.9; S(5): 9	252.9198672	35.03	2
S(3): 100.0; S(5): 100.0	112.2754654	10.72	2
S(1): 0.0; T(3): 0.0; T(10): 100.0; T(1	215.4195904	62.5	2
S(1): 100.0; T(3): 0.0	266.0058679	59.99	2
T(2): 0.0; S(8): 0.1; S(9): 0.1; S(10): 9	162.3330066	21.27	2
S(1): 0.0; S(3): 0.0; S(7): 0.0; T(8): 0.	308.3287077	69.8	3
T(7): 100.0	279.5359811	19.28	2
S(1): 0.0; S(5): 100.0; S(19): 0.0; S(2	390.0238431	51.66	3
S(4): 100.0; S(13): 0.0	314.859159	58.28	2
T(4): 47.9; S(9): 47.9; S(17): 0.3; T(1	48.27410065	6.47	2
S(4): 0.0; S(8): 0.0; S(10): 0.0; T(19):	357.0091354	99.27	2
T(2): 0.0; S(6): 100.0	309.6439959	64.95	2
S(8): 0.0; S(12): 100.0; T(18): 0.0; S(	408.8619794	51.47	3
S(3): 3.5; S(5): 96.5	107.2944016	13.06	3
T(6): 0.0; Y(9): 0.0; S(17): 0.0; T(18):	308.3870831	64.23	3
S(3): 100.0	363.7039956	69.12	2
S(7): 0.0; S(9): 100.0	336.4132963	59.74	2
T(4): 50.0; S(5): 50.0; S(8): 0.0; S(10	206.5943585	34.88	2
S(1): 0.0; S(2): 0.0; T(3): 0.0; Y(4): 0.	280.7293862	28.3	2
S(5): 100.0	207.6272394	27.61	3
S(1): 0.0; S(2): 0.0; S(7): 2.0; S(8): 98	179.4920973	24.94	2
S(7): 0.0; S(9): 100.0	231.268634	28.82	2
S(6): 31.9; T(7): 2.2; Y(10): 31.9; T(1	90.1472841	4.96	4
S(2): 50.0; S(3): 50.0; S(8): 0.0; T(9):	143.467461	32.45	2
Y(1): 0.0; S(4): 0.0; S(6): 0.0; S(7): 10	271.9071796	42.96	2
S(1): 100.0; T(10): 0.0; T(18): 0.0	297.2601536	111.45	2
T(5): 97.2; S(7): 2.8	106.1755126	7.56	2
S(11): 99.5; S(14): 0.5; T(19): 0.0; T(	83.72718143	10.76	4
Y(2): 0.0; S(9): 100.0; T(19): 0.0	459.3634559	63.28	2
S(3): 0.0; S(14): 100.0	279.8062684	55.03	3
S(5): 0.0; T(13): 100.0; T(16): 0.0; S(	349.1627396	105.12	3
S(1): 33.1; S(3): 33.1; S(5): 0.7; S(6):	416.6570281	44.59	4
T(7): 50.0; S(8): 50.0; Y(12): 0.0	222.6624783	38.84	2
S(2): 0.0; S(3): 0.0; S(5): 0.0; T(6): 0.	346.0267676	59.67	3
S(1): 0.1; S(3): 97.0; S(6): 2.9	105.0556697	21.34	2
S(4): 0.0; S(12): 100.0; S(14): 0.0; T(	246.6168879	67.04	3
S(4): 96.5; S(6): 51.7; S(7): 51.7; T(1	117.7658894	10.38	3
Y(5): 0.0; S(7): 50.0; T(9): 50.0	269.2047931	44.53	2
S(2): 100.0; Y(5): 0.0	188.0437846	29.7	2
S(1): 0.0; T(3): 49.6; S(5): 49.6; S(6):	317.9824083	58.56	2

Information Classification: General

T(4): 50.0; S(7): 50.0; T(13): 0.0; Y(2:	335.9956775	42.23	4
S(14): 100.0; S(25): 0.0; T(26): 0.0; S	319.4560089	47.44	3
S(4): 100.0	463.6719366	34.96	2
S(1): 100.0; S(7): 100.0	181.5264742	11.43	2
S(3): 94.0; T(5): 6.0; T(10): 0.0; Y(15)	226.7433111	20.87	3
S(8): 100.0	129.3905229	18.92	3
S(1): 0.0; S(5): 100.0; S(7): 0.0; S(9):	291.6530817	64.57	2
S(7): 100.0; S(12): 1.5; S(15): 49.3; S	309.7402167	76.29	2
S(4): 50.0; S(5): 50.0; S(13): 0.0	126.7155811	14.27	2
T(1): 0.1; T(3): 0.1; S(5): 3.6; S(6): 96	127.0262215	18.91	3
S(8): 1.9; S(9): 96.2; S(11): 1.9	303.5382099	56.2	2
S(1): 0.0; S(3): 100.0; Y(8): 0.0; T(11	425.1814717	100.26	2
S(1): 0.0; S(6): 0.0; S(11): 0.0; S(17):	359.7633997	55.33	3
S(13): 100.0; S(16): 0.0	371.3830125	92.06	2
S(1): 0.0; S(3): 0.0; S(12): 0.0; S(14):	196.0444213	29.73	3
S(20): 100.0; S(24): 100.0	443.6340532	38.9	3
S(6): 95.6; S(7): 4.4; S(9): 100.0; S(1	242.1574765	32.88	3
S(3): 100.0; S(5): 0.0	202.3243392	35.66	2
Y(5): 0.0; S(10): 100.0	307.2697895	46.98	2
T(5): 50.0; T(6): 50.0; S(8): 100.0	131.7998356	14.05	3
S(7): 0.0; S(11): 50.0; S(12): 50.0	123.075251	10.75	3
Y(2): 0.0; T(3): 0.0; S(5): 100.0	201.260859	35.41	2
S(6): 100.0; S(12): 0.0	356.3841467	108.53	2
T(1): 0.0; S(2): 0.5; S(4): 99.5; S(10):	176.1715137	12.06	3
S(3): 1.8; S(4): 1.8; S(5): 96.4	232.4960265	27.37	2
S(1): 0.0; T(4): 0.0; S(7): 100.0; S(14	444.1442704	104.48	2
S(2): 100.0; S(7): 0.0; Y(10): 0.0; T(1	477.0499667	85.87	2
T(1): 0.0; T(10): 95.2; S(13): 2.4; S(1	180.0756002	9.77	3
S(4): 100.0; S(5): 0.0	161.4979567	21.63	2
S(5): 0.0; S(7): 0.0; S(10): 96.4; T(12	177.7400801	30.44	3
S(7): 100.0	275.5570715	25.74	2
S(2): 0.0; S(10): 100.0; S(13): 0.0; T(	238.53908	35.29	2
S(4): 0.0; T(6): 0.0; S(9): 0.0; T(13): 0	268.337265	61.06	2
T(2): 0.0; S(4): 0.0; T(7): 0.0; S(12): 1	277.877771	66.99	2
S(10): 100.0	288.9799662	48.09	2
S(10): 0.0; S(12): 0.0; Y(15): 1.4; S(1	463.0973403	98.71	2
T(1): 0.0; S(2): 0.0; S(11): 50.0; S(12	284.1129724	47.18	2
S(1): 0.0; S(7): 100.0	444.0477126	50.94	2
S(9): 100.0	192.5472343	17.83	4
S(1): 0.0; S(4): 100.0; S(6): 0.0; S(7):	276.7873398	62.62	2
S(1): 0.0; S(16): 0.0; S(28): 4.0; S(29	235.3389081	32.76	3
S(2): 0.0; T(5): 0.0; T(6): 0.0; T(8): 0.	308.1106588	37.17	2
S(2): 100.0; S(6): 0.0	78.7322303	14.45	2
S(11): 100.0; S(17): 100.0; T(21): 33.	459.170283	55.57	3
S(4): 2.4; S(5): 97.6; S(7): 0.0; S(8): 0	167.8369215	43.7	3
S(4): 100.0; S(7): 0.0; T(13): 0.0	105.1752898	21.72	3
Too many isoforms		3.77	3
S(3): 0.0; S(8): 100.0	295.863347	66.56	2
S(2): 100.0; S(4): 0.0; S(8): 0.0; S(12	273.8363515	38.28	2
S(1): 0.0; S(3): 0.0; Y(5): 0.0; S(8): 10	302.7567645	52.3	2
T(6): 100.0; S(9): 0.0; S(11): 0.0; S(1	254.3103608	49.48	2
T(4): 0.0; T(12): 50.0; S(14): 50.0; S(	214.3631443	38.16	4
S(9): 0.0; S(14): 100.0; S(18): 0.0; T(	295.4355269	69.16	4
S(2): 0.0; S(5): 100.0	191.6379393	36.17	2
S(1): 0.0; S(6): 100.0; S(8): 100.0	187.3313973	35.01	2

Information Classification: General

T(3): 0.0; S(8): 0.0; S(12): 0.0; T(14):	315.61281	62.74	3
S(3): 100.0; S(6): 0.0	267.9880516	51.75	2
S(2): 0.0; S(5): 100.0; S(6): 100.0	242.3799363	55.77	2
S(5): 100.0; S(8): 100.0; T(19): 0.0; S	385.7958882	50.39	3
S(3): 0.0; S(5): 100.0	248.2262191	41.18	2
S(2): 2.6; S(3): 97.4	117.9800277	13.85	2
T(12): 50.0; S(13): 50.0	231.5490187	22.34	3
S(3): 100.0; S(8): 0.0; S(13): 0.0; S(2	314.7701722	55.54	3
T(2): 33.3; S(3): 33.3; S(4): 33.3; S(1	201.413949	55.37	2
S(1): 0.0; S(5): 100.0	215.9313798	21.68	2
T(8): 0.0; S(14): 100.0	209.7458587	14.34	3
S(1): 0.0; S(4): 100.0	128.3015508	17.59	3
T(1): 0.0; S(29): 97.1; T(31): 2.9	341.747294	33.77	3
S(3): 100.0; S(9): 0.0; T(11): 0.0	348.3026981	74.41	2
S(1): 100.0; T(4): 0.0; S(7): 100.0	173.3442602	19	3
S(2): 0.0; T(7): 0.0; S(10): 0.0; S(12):	367.051334	52.76	2
S(1): 0.0; Y(3): 0.0; S(7): 100.0	379.5382411	51.15	2
S(4): 100.0; S(6): 0.0	189.3424693	43.14	2
T(2): 0.0; S(4): 0.0; T(8): 0.0; S(9): 10	177.6185277	42.34	3
T(1): 0.0; S(5): 100.0; T(8): 1.6; S(9):	378.3059794	68.85	2
S(2): 0.0; S(4): 100.0; S(7): 0.0	155.1948016	12.27	2
S(1): 2.3; S(2): 97.7	184.0452941	10.5	2
T(7): 90.8; T(10): 4.6; S(11): 4.6; S(1	184.6375849	34.9	3
S(1): 0.0; S(2): 0.0; S(3): 100.0; S(6):	340.7723119	109.85	2
S(1): 0.0; S(2): 0.1; S(3): 99.9	112.6861908	43.99	2
T(7): 50.0; T(8): 50.0; S(10): 0.0	265.201999	31.23	2
T(1): 0.0; S(6): 0.0; S(10): 100.0; S(1	302.8038732	54.13	2
T(5): 100.0; T(9): 96.7; S(11): 3.3; T(	404.0035996	87.39	3
S(3): 100.0; S(6): 0.0; S(12): 0.0	238.574584	32.91	2
S(1): 100.0; T(9): 0.0	325.5729313	50.58	2
S(2): 0.0; S(3): 100.0; S(5): 100.0; T(	345.5496414	76.44	2
T(3): 0.0; T(12): 95.1; T(18): 4.9; Y(2	187.493175	12.8	3
S(1): 0.0; S(3): 100.0; S(4): 100.0; S(	303.2858635	47.23	2
S(1): 0.1; T(7): 5.3; T(25): 47.3; T(33	69.63899161	15.51	4
S(4): 0.0; S(5): 0.0; S(6): 0.0; S(10): 0	395.3260381	19.36	3
T(1): 0.0; S(5): 0.0; S(7): 0.0; S(11): 1	196.9145138	48.14	3
Y(1): 2.1; S(3): 97.9; Y(5): 0.0; S(6): 0	139.8356198	24.95	3
S(3): 100.0	317.9605014	40.05	2
S(1): 0.0; S(5): 0.0; T(6): 0.0; S(10): 0	332.4149696	80.02	2
S(9): 0.1; S(10): 0.1; S(11): 0.1; S(19	98.04011897	11.19	3
S(2): 100.0; S(4): 98.0; S(6): 2.1	228.3620263	23.6	2
S(1): 0.0; S(6): 100.0; S(11): 0.0	155.4030679	19.28	3
S(3): 0.0; T(7): 100.0	76.9194997	1.01	3
T(3): 100.0; S(7): 0.0; Y(10): 0.0	310.6750431	48.31	3
S(1): 0.0; T(3): 100.0	289.555614	39.98	3
T(18): 0.0; Y(22): 6.4; S(23): 93.6	120.7143113	9.14	6
S(3): 100.0; T(10): 0.0	205.9728736	47.64	3
S(2): 0.0; S(4): 100.0	176.2255845	9.41	3
S(4): 100.0	270.4678896	36.48	2
S(9): 1.7; S(11): 98.3	327.891386	48.16	3
T(1): 2.1; S(2): 97.9; T(6): 0.0	189.8288463	65.84	2
T(2): 49.6; S(3): 49.6; T(5): 0.8	267.72993	28.41	2
S(5): 1.6; S(7): 98.4; S(9): 0.0; S(10):	319.5901258	60.25	2
S(3): 100.0; T(6): 0.0; S(8): 0.0	123.5365641	13.73	2
T(1): 50.0; S(2): 50.0; Y(11): 0.0	86.85231714	8.39	3

Information Classification: General

T(1): 0.0; S(3): 0.0; T(5): 0.0; S(6): 0.	203.8444602	33.12	4
Y(5): 0.0; S(15): 100.0	419.9026971	46.2	3
S(2): 0.0; S(3): 0.0; T(6): 0.0; S(7): 0.	241.0030154	16.47	3
S(1): 0.0; T(6): 0.0; S(12): 100.0; S(1	406.9014618	123.18	2
S(1): 2.9; T(3): 97.1; S(5): 100.0; S(7	286.5181283	69.96	3
T(9): 0.0; S(11): 0.0; S(13): 100.0	296.6842418	62.42	2
S(3): 0.0; S(7): 4.5; S(10): 95.5; S(21	202.9650635	45.43	4
S(6): 50.0; S(7): 50.0	155.8413005	35.4	3
T(5): 0.0; S(9): 0.0; Y(11): 0.0; S(15):	194.7260003	41.77	3
S(3): 0.0; T(6): 100.0; S(8): 100.0	221.6249948	27.49	2
S(1): 2.2; S(3): 97.8	90.25188818	16.92	2
S(3): 0.0; S(9): 100.0; T(14): 0.0; S(1	342.870946	62.09	2
S(1): 0.0; S(8): 100.0	134.9484418	21.83	3
T(8): 6.0; S(9): 50.0; S(10): 50.0; S(1	105.9790826	12.82	4
S(11): 95.3; S(13): 4.7	157.2872434	19.61	3
T(2): 0.0; S(11): 100.0	198.5125727	35.83	2
S(1): 0.0; S(2): 100.0; S(4): 0.0; T(5):	250.4874753	54.28	2
S(12): 100.0	268.7793256	42.24	3
S(12): 100.0; S(14): 0.0	483.3757395	84.75	3
T(2): 0.0; S(7): 100.0	222.4940612	27.09	2
S(8): 100.0; T(15): 0.0	432.7354871	79.49	2
S(5): 100.0	244.6597852	41.36	2
S(5): 0.0; S(7): 100.0	302.5643926	57.04	3
S(3): 100.0	209.1388319	31.38	2
S(4): 100.0; T(9): 0.0	318.4841165	52.63	2
T(4): 0.0; S(5): 0.0; S(8): 100.0; T(10)	422.3481692	113.42	2
S(1): 100.0; T(4): 0.0	116.8340089	21.23	2
T(6): 0.0; Y(8): 0.0; S(20): 48.8; S(22)	230.8249732	35.21	4
S(2): 33.3; S(4): 33.3; S(5): 33.3; T(7	55.25864494	18.39	3
T(1): 0.0; S(5): 100.0	166.7118732	16.35	2
S(1): 0.0; S(3): 100.0; S(6): 0.0; S(9):	196.0545885	52.59	2
T(1): 0.0; S(5): 3.0; S(6): 90.8; S(7): 3	204.1639598	57.49	3
S(9): 100.0; T(16): 0.0	121.9833291	11.67	4
S(3): 99.9; T(5): 0.0; S(7): 0.0; S(10):	125.1443106	8.55	3
S(2): 0.0; S(3): 100.0; S(5): 0.0; T(6):	383.3964046	83.89	2
T(9): 100.0; S(11): 100.0	319.9436356	47.48	2
S(5): 100.0; S(6): 100.0; T(11): 0.0	448.5036877	94.9	2
S(3): 0.0; S(5): 0.0; S(10): 0.0; S(15):	208.3047673	22.18	2
S(2): 0.1; Y(5): 50.0; S(6): 50.0; S(9):	181.0029733	6.6	4
S(1): 2.3; S(4): 97.7; S(6): 100.0	182.6074171	14.22	2
S(1): 3.3; S(3): 96.7; S(11): 0.0	151.4177574	18.49	3
Y(1): 0.0; Y(8): 0.0; S(10): 100.0; T(1	335.0685549	56.39	2
S(6): 48.8; S(7): 48.8; S(9): 2.3	114.6741147	4.05	3
S(11): 96.7; S(13): 3.3; T(18): 0.0; S(	303.7498071	57.21	3
S(1): 100.0; T(10): 0.0; S(12): 0.0	219.8675646	47.74	2
S(7): 100.0; S(10): 100.0; S(11): 100.	348.4766371	40.31	3
S(3): 100.0	87.4183177	9.1	2
S(1): 2.4; S(3): 97.5; T(6): 0.1; S(7): 0	191.268026	17.86	3
S(1): 1.3; S(2): 49.3; S(3): 49.3; T(14	406.5280426	56.67	3
T(6): 0.0; T(7): 0.0; T(11): 0.0; S(15):	256.7787389	34.3	3
T(1): 0.0; S(3): 100.0; T(12): 0.0	349.3256465	79.72	2
T(1): 0.0; S(3): 100.0; S(9): 0.0; S(13	226.9365167	32.66	3
S(1): 99.8; T(3): 0.2; T(8): 0.0	170.8307587	28.86	3
T(6): 0.0; T(9): 2.3; S(10): 97.7	314.0703626	63.57	2
S(1): 0.0; S(5): 0.0; S(7): 100.0; S(11	219.4953129	27.2	2

Information Classification: General

S(9): 100.0; S(16): 0.0; T(18): 0.0		236.6267548	37.96	3
S(8): 100.0		348.8351905	52.4	3
S(11): 99.9; S(13): 0.1; T(18): 0.0; S(		358.9538789	65.62	3
T(6): 0.0; T(12): 100.0; S(21): 100.0		236.2494189	36.49	3
S(3): 100.0		193.561749	22.36	2
S(3): 100.0		107.3858654	4.2	3
S(1): 0.0; S(3): 2.1; S(5): 97.9		115.6957857	22.98	2
S(22): 100.0		484.0997262	51.38	3
S(4): 3.5; S(5): 96.4; Y(9): 0.1; S(11):		227.5470881	65.84	3
T(2): 0.0; S(8): 100.0; S(11): 0.0		218.4780729	16.48	2
T(12): 0.0; S(14): 100.0; S(17): 0.0; Y		544.0881053	79.25	3
S(3): 100.0; S(6): 96.7; T(8): 0.0; T(1		181.9690645	28.13	2
S(1): 0.0; S(2): 0.0; S(3): 0.0; S(6): 10		275.7861818	56.78	2
T(6): 0.0; S(8): 100.0; S(22): 0.0		184.2401642	28.74	3
T(13): 100.0; S(19): 0.0; S(23): 0.0; S		457.2866358	94.58	4
S(3): 0.6; S(5): 7.0; S(11): 92.4; S(18		136.1127249	30.93	4
T(1): 2.1; T(2): 95.8; S(4): 2.1		50.87002669	13.02	2
S(2): 0.0; S(7): 100.0; T(11): 0.0; S(1		200.575734	30.12	3
S(2): 100.0		132.1590353	25.31	3
S(4): 99.4; T(6): 0.3; S(7): 0.3; S(20):		112.480637	17.02	3
S(6): 100.0; S(12): 100.0		342.9340563	46.26	2
S(7): 100.0; S(14): 0.0; S(16): 0.0		339.0730763	66.97	2
S(5): 100.0; S(12): 0.0		175.1449174	16.65	3
S(10): 50.0; S(13): 50.0		346.2445999	56.26	2
S(7): 100.0; S(18): 94.2; T(20): 5.8		166.0925795	20.19	4
S(2): 100.0; S(6): 0.0		151.6944172	11.4	3
T(4): 100.0; S(6): 100.0; S(13): 0.0; S		336.6252802	36.46	3
S(1): 0.0; T(4): 0.0; S(7): 100.0; S(13		398.2536775	45.83	2
S(2): 1.6; T(4): 49.2; S(5): 49.2		257.6392384	42.51	3
S(4): 100.0; S(9): 0.0; S(10): 0.0		336.4454548	78.91	2
T(2): 0.0; S(5): 0.1; S(6): 2.3; T(8): 2.		119.1756459	9.52	2
S(3): 0.0; S(7): 100.0; T(14): 0.0		517.8383253	93.23	2
S(3): 100.0; Y(6): 0.0; S(8): 0.0		174.2233984	14.98	3
S(6): 2.2; S(7): 97.8; S(12): 0.0; S(18		305.8916064	59.82	3
Y(2): 0.0; S(5): 2.9; S(7): 94.1; T(8): 2		152.0654972	12.02	3
S(1): 0.0; T(5): 0.0; S(10): 0.0; S(14):		331.2595219	39.04	3
S(6): 0.0; T(8): 0.0; S(10): 0.0; T(12):		318.4058199	78.45	2
S(8): 0.0; S(14): 95.1; S(16): 4.9		155.8092462	20.77	3
S(2): 0.0; S(3): 99.9; T(4): 0.0; S(5): (		397.8049548	102.36	2
S(11): 100.0; S(13): 100.0		278.6075504	49.18	3
S(6): 100.0; S(10): 97.7; S(11): 2.3		220.587002	41.93	2
T(2): 0.0; T(7): 0.0; S(10): 100.0		231.3760287	51.39	2
S(9): 100.0; S(12): 100.0; T(21): 0.0;		181.5273152	36.05	3
S(1): 0.0; S(3): 99.9; S(5): 0.0; T(6): (		276.157676	49.43	2
T(1): 0.0; T(5): 0.0; T(6): 0.0; S(7): 99		177.6185277	34.69	3
S(3): 99.9; S(5): 0.1; T(8): 0.0; T(15):		277.4759108	53.88	3
S(5): 100.0		168.0017576	14.71	4
S(2): 0.0; T(3): 0.0; S(4): 0.0; S(5): 0.		205.4489367	33.76	2
S(4): 100.0		362.4086201	42.7	2
S(8): 100.0		258.8599313	29.19	3
S(1): 0.0; S(3): 97.9; T(4): 2.1; Y(7): (		188.7356398	45.84	2
S(3): 0.0; T(8): 0.0; S(12): 0.0; T(15):		191.8751947	50.89	3
S(1): 0.0; S(4): 4.9; S(6): 4.9; S(8): 4.		110.7169575	22.73	4
S(7): 1.1; S(8): 48.4; T(9): 48.4; S(11		160.5725222	38.17	2
T(1): 0.0; S(4): 100.0		161.4695612	9.54	2

Information Classification: General

T(1): 0.0; S(3): 100.0	129.1910199	8.79	2
S(1): 0.0; S(6): 0.0; T(11): 0.0; S(13):	406.2257011	65.42	3
S(1): 0.0; S(2): 0.0; T(6): 0.0; S(9): 6.	231.5318801	59.63	2
S(7): 0.0; S(12): 99.9; S(14): 0.1	311.0526812	21.64	3
S(6): 100.0	396.687644	49.87	3
T(7): 0.0; T(13): 3.7; S(16): 96.3	159.4931068	37.06	3
S(3): 0.0; S(6): 2.1; S(8): 97.9	185.0036218	32.74	2
S(1): 0.0; T(2): 0.0; S(7): 100.0	151.0768071	23.27	2
S(3): 100.0; T(4): 100.0; S(12): 0.0; S	302.9361446	36.39	3
S(14): 100.0; S(17): 0.0	420.183295	121.61	2
S(7): 100.0	280.9756709	29.77	2
S(9): 100.0	154.5523471	19.59	3
S(2): 0.0; S(5): 1.9; S(7): 98.1; T(11):	328.27895	92.36	2
S(3): 1.8; S(4): 98.2; T(17): 0.0	406.5301328	49.06	4
T(1): 0.0; S(11): 100.0	418.4475011	51.53	3
S(4): 0.0; S(10): 2.5; S(11): 97.5; T(1	225.1112179	47.41	2
S(4): 100.0; S(12): 99.9; S(14): 0.1	281.7633404	36.95	3
T(1): 0.0; S(7): 100.0	203.7912825	34.31	2
T(13): 48.3; T(14): 48.3; S(15): 3.4; S	134.6861513	7.04	3
S(2): 0.0; Y(5): 0.0; T(9): 0.1; Y(10): (	259.3172369	43.63	2
S(3): 100.0; S(12): 0.0; T(15): 0.0; S(	178.9083441	38.5	3
S(5): 100.0; S(8): 100.0; S(10): 0.0	297.6443168	33.59	2
T(13): 0.0; S(18): 100.0	548.0663259	79.96	2
S(9): 100.0	280.3368291	38.58	2
S(21): 0.4; T(23): 0.4; T(26): 5.7; T(2	245.1541458	44.31	3
S(5): 100.0	249.9134679	30.38	2
S(5): 100.0; T(10): 0.0	213.096472	19.54	2
S(3): 100.0	252.1105689	48.59	3
T(3): 0.0; S(5): 0.0; S(8): 100.0; S(11	331.0076094	54.71	2
S(2): 0.0; Y(10): 0.0; S(16): 2.7; S(17	424.4717786	126.14	3
S(2): 0.0; Y(6): 0.1; S(8): 99.9; T(18):	216.2640856	64.83	2
S(4): 100.0; S(12): 0.0; S(14): 0.0	174.0581792	19.21	3
S(7): 0.0; S(12): 0.0; S(15): 100.0; S(	534.2340795	139.17	2
T(1): 0.0; S(3): 97.9; T(5): 2.1; S(7): (	281.7532688	34.61	2
S(1): 0.0; S(9): 0.0; S(12): 0.1; S(14):	264.9716119	58.74	3
S(2): 0.1; S(6): 99.0; S(9): 1.0; S(15):	96.98650714	2.6	3
S(6): 100.0	235.3194773	35.22	2
S(1): 100.0; S(7): 100.0; S(13): 100.0	300.7526232	9.93	3
S(8): 33.3; S(9): 33.3; S(11): 33.3	182.5480455	18.08	3
S(1): 100.0; T(3): 0.0	298.3063888	64.1	2
S(6): 100.0; S(9): 0.0; S(14): 0.0	318.0546285	99.83	2
S(5): 100.0	227.9486419	11.87	3
S(1): 0.1; S(3): 96.7; S(5): 3.2; T(7): (	223.0476551	37.73	3
T(2): 2.1; S(4): 95.8; T(6): 2.1; T(13):	259.6514379	75.43	2
S(3): 100.0; Y(7): 0.0; T(13): 0.0	301.6397676	48.62	2
S(7): 100.0	154.6488668	23.26	2
S(7): 0.0; S(19): 3.5; S(20): 96.5	238.9435185	42.12	4
S(1): 0.0; S(2): 1.1; S(3): 33.0; S(5): 3	90.5688707	10.64	2
Y(4): 0.0; S(10): 100.0	273.0738056	23.29	3
T(4): 0.0; S(6): 100.0	191.5271275	53.32	2
S(9): 100.0; Y(16): 0.0	393.8627033	50.77	2
Y(1): 0.0; S(2): 0.0; S(9): 0.0; T(11): (	299.8259052	50.42	3
S(5): 50.0; S(6): 50.0; S(8): 100.0	225.5658011	29.26	3
S(6): 100.0	241.8920289	43.59	2
S(1): 0.0; S(2): 2.1; S(3): 97.9	140.0443057	24.79	2

Information Classification: General

S(2): 0.0; Y(4): 0.0; Y(5): 0.0; T(9): 10	193.1178666	27.02	3
S(3): 100.0; T(18): 0.0	215.7853637	25.26	3
S(3): 0.0; S(9): 100.0; S(16): 0.0	266.1849026	50.92	3
S(5): 0.0; S(7): 100.0; S(11): 0.0; Y(1	325.119142	73.19	2
T(5): 0.0; S(7): 0.0; S(12): 0.0; T(19):	142.9877187	38.73	3
T(3): 0.0; S(5): 0.0; T(7): 0.0; S(14): 0	181.3114306	13.17	3
Y(3): 0.0; S(6): 100.0; T(8): 0.0; T(11)	326.3481037	77.49	2
S(4): 100.0	317.7724588	51.66	2
T(3): 1.0; T(5): 49.5; S(6): 49.5; T(7):	189.4109455	24.81	2
S(9): 100.0	222.6624388	28.01	2
S(12): 100.0; S(17): 0.0	494.3583522	68.4	3
S(12): 0.0; S(19): 100.0; S(25): 0.0; S	428.2431629	52.8	3
T(2): 0.2; S(5): 99.8; S(8): 0.0; T(9): 0	111.7977594	5.05	2
S(3): 0.0; S(9): 24.7; T(10): 1.0; S(11)	226.1190234	44.81	3
S(3): 3.2; T(6): 96.8	162.8105195	9.19	3
Y(2): 100.0; T(6): 0.0	208.2403416	26.76	2
S(7): 3.0; S(9): 97.0	115.4465567	29.55	2
S(1): 0.0; S(11): 100.0	138.6386646	14.1	2
S(3): 0.2; S(4): 4.0; S(6): 4.0; S(7): 91	305.0988755	61.31	4
S(5): 100.0; S(12): 100.0; T(15): 0.0	270.728507	71.35	2
S(4): 0.0; T(7): 0.0; S(8): 50.0; T(10):	255.5205395	44.85	2
S(3): 100.0	342.6490744	53.77	3
S(1): 0.0; S(2): 0.0; S(4): 0.0; S(9): 10	255.3725429	43.27	2
S(1): 0.0; S(8): 96.2; T(10): 3.7; T(13)	173.9773112	36.78	3
Y(2): 0.0; S(3): 100.0	114.6201163	12.63	3
S(8): 0.0; S(10): 1.5; S(11): 1.5; T(13	265.2103857	53.53	2
T(3): 32.9; S(5): 32.9; S(7): 32.9; S(9	281.7944316	37.48	4
T(11): 0.0; S(15): 0.0; T(18): 0.1; S(1	209.0037314	33.1	3
S(1): 0.0; S(5): 96.0; S(9): 4.0; S(12):	165.095787	18.92	3
S(3): 0.0; S(6): 0.0; T(9): 0.0; T(11): 0	309.9690124	55.32	4
S(3): 0.0; S(11): 97.4; S(13): 2.6	187.2566827	41.46	2
S(12): 100.0	230.8139337	9.18	3
S(1): 100.0; T(5): 0.0; Y(7): 0.0; S(15	377.1639968	63.55	2
S(4): 0.0; S(5): 0.0; S(6): 0.0; S(10): 0	416.072018	43	3
T(6): 0.0; S(8): 0.0; S(10): 0.0; T(17):	473.3315387	65.79	2
S(1): 0.0; S(3): 2.0; S(4): 98.0; S(7): 0	118.8082897	23.95	2
S(11): 0.0; S(18): 100.0; S(24): 0.0; S	257.1775965	28.19	3
S(1): 6.1; S(10): 93.9	77.20781825	7.35	5
T(2): 0.0; S(5): 0.0; S(6): 0.0; S(8): 10	342.1822017	76.72	2
S(6): 100.0; S(7): 100.0; Y(22): 0.0; S	216.3545154	51.39	3
T(7): 100.0; T(9): 100.0	189.3712401	12.36	3
T(1): 0.0; S(6): 100.0; S(7): 100.0; S(	269.3370018	36.65	3
S(5): 0.1; S(15): 49.9; S(19): 49.9; T(	189.0520082	40.3	3
S(4): 0.0; S(7): 99.9; T(8): 3.5; T(9): 9	153.6928694	25.59	2
S(5): 100.0; S(7): 0.0; T(10): 0.0; S(1	314.9057712	73.22	3
S(13): 100.0	359.4520677	52.33	3
S(3): 0.0; S(5): 100.0; T(8): 0.0; T(10)	312.9089899	65.93	2
S(1): 100.0	179.605005	10.32	2
S(2): 0.0; S(5): 100.0; S(8): 95.6; S(1	117.3991703	8.76	2
S(7): 100.0; S(13): 0.0; S(16): 0.0; S(	247.2762324	51.16	3
T(6): 1.7; S(7): 98.3; S(10): 0.0; S(11	228.2187237	33.09	2
S(1): 0.0; S(4): 100.0; T(10): 0.0; Y(1	187.8038729	28.3	3
S(3): 100.0	190.5080963	11.35	3
T(1): 97.0; S(2): 2.9; S(4): 0.1; S(7): 0	170.9390759	21.63	4
S(5): 0.0; S(9): 100.0	140.752874	16.36	3

Information Classification: General

S(3): 2.1; S(4): 2.1; T(5): 2.1; T(6): 91	96.70381186	23.78	2
T(1): 0.0; T(3): 0.0; T(9): 49.2; S(10):	430.5591289	131.14	2
S(3): 100.0; S(6): 0.0	182.9874124	21.63	3
S(1): 0.0; S(7): 100.0; S(10): 0.0	212.4646847	34.06	3
T(1): 0.0; S(3): 0.0; S(13): 100.0; S(1	247.4752323	49.35	2
S(3): 100.0	127.9576989	3.27	3
S(4): 0.0; T(6): 3.6; S(8): 96.3; S(10):	273.9452831	52.33	3
S(12): 100.0; T(21): 0.0; Y(30): 0.0	257.4284476	23.93	4
S(2): 100.0; S(6): 0.0	166.9082898	36.08	2
S(4): 0.0; S(12): 100.0; T(21): 0.0	206.5113179	35.52	3
T(1): 0.0; S(18): 100.0; S(21): 0.0	179.5361439	39.67	3
S(4): 100.0; T(6): 0.0	356.7835863	68.55	2
S(1): 0.0; S(7): 100.0; S(13): 100.0	129.805616	6.8	5
T(1): 0.0; S(3): 100.0	210.8478203	21.26	2
T(1): 0.0; S(2): 0.0; S(8): 100.0	273.3614156	22.52	3
S(3): 100.0; T(6): 0.1; S(7): 0.0; T(10)	226.1374171	12.54	3
Y(3): 0.0; S(12): 100.0	257.9998152	35.16	2
S(1): 0.3; S(4): 0.0; S(9): 4.7; S(11): 4	127.7001957	21.14	3
S(7): 100.0; S(9): 0.0	348.2668602	70.78	2
S(5): 99.9; T(7): 0.1; S(9): 2.9; S(13):	160.970235	40.03	2
S(1): 100.0; S(3): 4.2; S(7): 95.8; S(1	192.9257108	9.41	3
Y(3): 0.0; T(14): 0.0; S(17): 0.1; S(18)	320.5127119	71.94	3
S(6): 0.0; Y(8): 0.7; S(9): 49.6; T(11):	389.284674	106.89	2
S(1): 0.0; S(9): 97.9; T(16): 2.1	201.7212915	49.41	2
S(6): 0.0; S(15): 50.0; S(17): 50.0	185.5578239	21.42	3
S(1): 0.0; T(3): 100.0; S(5): 100.0; T(	327.4158806	71.66	2
S(3): 0.0; S(5): 100.0; S(7): 0.0	242.0456967	29.89	2
S(7): 47.9; T(8): 47.9; S(10): 0.3; S(1	75.26372456	6.94	3
T(3): 100.0	123.8998798	17.59	3
S(1): 0.0; S(3): 100.0; T(6): 0.0; Y(10)	237.0627765	47.85	2
S(4): 100.0	211.445594	16.39	3
S(14): 96.9; S(17): 3.1	218.0495957	50.21	3
T(1): 50.0; S(3): 50.0; T(10): 0.0; S(14	134.61143	48.07	3
T(1): 0.0; S(3): 0.0; S(4): 0.0; S(5): 0.	261.5397516	64.27	2
S(1): 50.0; S(2): 50.0; S(11): 100.0	249.3822297	59.69	2
S(3): 100.0; T(11): 0.0	351.0515465	38.21	2
S(3): 97.2; S(5): 5.6; S(7): 97.0; S(9):	171.5611214	17.68	2
T(1): 0.0; S(2): 0.0; T(4): 0.0; S(5): 2.	252.7073679	59.27	2
S(3): 100.0	105.2530045	4.29	2
T(5): 0.0; S(7): 100.0; S(9): 0.0	260.5630521	68	2
T(9): 0.0; S(12): 100.0; T(19): 0.0; S(	326.649946	39.54	3
T(10): 100.0; T(15): 0.0; S(16): 0.0; S	421.5651667	71.11	3
S(3): 100.0; S(12): 0.0	244.9335281	43.12	2
S(6): 100.0; S(7): 100.0	292.5040196	70.6	2
S(1): 97.8; T(3): 2.2; T(8): 0.0; T(9): 0	223.5820577	70.58	2
S(5): 0.0; S(8): 0.0; S(12): 33.3; S(13	161.9726986	8.27	3
T(3): 2.5; S(4): 97.4; T(8): 0.1	132.8738456	29.55	2
T(9): 0.0; S(12): 100.0; T(19): 0.0	454.9776946	112.74	2
S(1): 1.4; S(2): 98.6	318.7676496	49.89	2
T(4): 100.0; T(7): 100.0; S(11): 0.0	249.73685	25.45	2
T(1): 0.0; S(8): 100.0; T(11): 0.0; S(14	377.3514856	106.4	2
T(15): 47.5; S(16): 47.5; S(18): 1.7; S	341.5575791	114.73	3
S(5): 0.0; S(8): 100.0	335.6170603	72.57	2
S(2): 0.0; S(4): 100.0; T(5): 0.0	246.5566941	48.73	2
S(5): 100.0; S(10): 0.0	160.1381554	23.24	2

Information Classification: General

S(11): 100.0		252.5975928	25.76	3
T(7): 0.0; S(8): 3.9; S(15): 96.1; S(18		157.3177395	16.41	3
S(1): 0.0; S(3): 0.0; T(8): 0.0; S(10): (		318.0546285	63.15	2
T(1): 0.0; S(3): 100.0; Y(5): 0.0; T(8):		232.2366242	49	2
S(4): 0.0; S(13): 100.0		186.6941056	44.55	3
S(4): 100.0; S(7): 0.0		529.4071114	86.6	2
S(1): 100.0; T(5): 0.0; T(12): 0.0		277.7349245	52.22	2
Y(3): 100.0; S(4): 0.0; S(6): 0.0		182.9184436	9.68	2
S(2): 100.0; S(4): 0.0; T(5): 0.0; S(7):		276.5886452	48.96	2
T(13): 0.0; S(19): 100.0; S(23): 99.8;		424.1227283	102.79	3
S(4): 0.0; S(13): 0.1; S(15): 2.6; S(16		240.6230388	37.33	3
S(3): 0.0; S(5): 0.0; S(8): 0.0; S(10): 1		300.1584955	73.69	2
S(5): 0.0; S(17): 100.0; T(23): 0.0		301.5738865	72.75	3
T(8): 0.0; S(12): 50.0; T(14): 50.0		299.4650782	65.35	2
S(2): 0.0; S(3): 100.0; S(7): 0.0		192.0003159	39.76	2
S(1): 0.0; S(6): 2.5; S(10): 2.5; S(14):		303.070002	92.29	3
S(4): 100.0; S(7): 0.0; S(12): 0.0; Y(1		252.0821377	30.27	3
S(5): 100.0		235.8553225	32.25	2
S(1): 0.0; S(3): 1.2; S(4): 49.4; S(7): 4		295.7814316	52.58	3
S(11): 50.0; S(12): 50.0; Y(26): 0.0; T		309.3919312	83.29	3
S(5): 0.0; S(13): 100.0		223.013289	47.95	3
T(8): 50.0; S(9): 50.0		158.1309745	4.83	3
Y(2): 0.0; S(4): 0.0; T(8): 0.0; S(10): (		209.9536853	52.98	4
T(5): 100.0; S(10): 0.0; S(16): 0.0; S(		362.9469974	57.02	3
S(1): 0.0; S(8): 0.0; Y(12): 90.0; T(17		158.7689393	23.77	4
S(7): 100.0; S(16): 0.0		280.6048191	36.34	3
T(1): 0.0; S(6): 0.0; S(10): 0.0; S(12):		451.1861183	66.06	2
S(2): 0.0; S(4): 0.0; S(13): 100.0; S(1		209.6469628	73.63	3
S(1): 0.1; S(3): 99.9; S(4): 100.0		148.4607807	12.95	2
S(4): 0.0; S(6): 0.0; S(8): 100.0		212.1103542	28.21	3
T(3): 2.3; S(4): 97.7; T(10): 0.0		219.5000804	38.63	3
T(1): 50.0; S(4): 50.0; S(13): 0.0; S(1		290.4574581	33.54	3
S(2): 0.0; S(9): 100.0		312.9089899	43.04	2
S(2): 0.0; S(7): 0.0; Y(8): 0.0; S(15): 1		345.0290854	67.08	3
S(2): 0.0; S(6): 100.0		276.5886452	61.72	2
T(3): 33.3; S(6): 33.3; S(9): 33.3; Y(1		122.9306141	8.03	3
T(4): 0.0; S(7): 100.0; T(13): 0.0; T(14		406.2417067	101.82	2
S(4): 0.0; S(12): 100.0; S(14): 0.0; T(		332.2415724	60.41	3
Y(3): 0.0; S(6): 0.0; S(17): 100.0		199.9291915	22.1	3
T(7): 0.0; S(13): 100.0		297.2863452	55.18	3
T(1): 0.0; Y(7): 100.0		202.2189969	28.41	2
S(7): 0.0; S(8): 0.0; T(19): 4.3; S(20):		165.8669802	10.52	3
S(2): 100.0; S(6): 99.9; T(8): 0.1; T(9)		187.2869215	22.16	2
S(2): 0.0; S(7): 100.0; S(10): 0.0		198.9250378	28.83	2
Y(3): 0.0; T(4): 0.0; S(12): 100.0		341.5984011	73.47	2
S(1): 0.0; S(11): 3.9; S(12): 3.9; S(13		139.1928294	29.34	3
S(2): 0.0; S(5): 0.0; T(11): 100.0; S(1		354.1726739	55.92	2
S(7): 100.0; S(8): 100.0		226.8891126	31.71	4
S(2): 4.8; T(3): 95.4; T(10): 99.8		120.0416541	17.79	3
Y(4): 0.1; S(6): 99.9; S(7): 100.0; Y(1		324.7826084	51.85	3
T(6): 100.0		445.1938848	59.88	2
S(6): 0.0; T(14): 0.0; T(16): 0.0; Y(17)		289.759359	37.42	4
S(1): 0.0; S(8): 4.0; S(10): 92.3; S(13		136.2902656	29.52	2
T(1): 0.0; S(7): 50.0; S(9): 50.0		199.1790241	21.73	2
S(7): 100.0; S(16): 0.0		165.4345135	16.59	3

Information Classification: General

S(8): 100.0		109.6907048	5.37	3
S(2): 50.0; S(3): 50.0		183.2926	25.11	3
S(1): 0.0; S(3): 0.0; T(9): 0.0; Y(14): (		421.5365378	67.87	3
T(6): 0.0; Y(7): 0.0; S(12): 100.0		207.4031202	26.42	2
S(3): 100.0; S(5): 100.0; T(10): 0.0; T		125.5449597	14.03	2
S(1): 0.0; S(3): 100.0; S(6): 0.0; T(9):		276.1694585	54.6	2
S(3): 50.0; S(4): 50.0; T(5): 0.0		146.5068757	9.61	2
S(1): 0.0; T(10): 0.0; S(12): 0.0; S(13		244.8344158	29.51	4
S(3): 0.0; S(6): 0.0; S(14): 0.0; S(17):		387.9014399	40.59	4
S(13): 100.0; S(19): 100.0; T(23): 33.		472.0818961	39.34	4
T(6): 0.0; T(9): 0.0; S(11): 100.0		178.6817927	22.77	3
T(10): 0.0; T(17): 100.0		403.9823867	52.89	3
T(1): 0.0; S(2): 0.0; S(3): 0.0; S(21): 3		325.1267125	62.89	3
S(1): 0.0; T(8): 0.0; S(12): 3.8; S(13):		238.0461098	12.11	3
S(3): 0.0; S(7): 0.2; S(13): 97.6; T(14		209.9762667	7.74	3
S(5): 0.0; S(9): 100.0; S(17): 0.0; T(1		311.2671416	39.41	3
T(1): 49.9; S(3): 49.9; S(5): 0.1; S(6):		200.5748143	24.83	3
S(1): 0.3; Y(2): 4.7; S(3): 95.0; S(9): (		112.6181457	3.75	3
S(1): 0.0; T(4): 100.0; S(10): 50.0; S(		226.1635178	21.78	3
T(1): 0.0; S(2): 0.0; S(4): 0.0; S(5): 0.		219.0939546	45.71	2
S(1): 0.0; S(3): 100.0; S(4): 0.0		296.0193945	36.39	2
S(6): 2.6; S(10): 97.3; T(12): 0.1; T(1(		353.9178262	44.33	3
S(6): 100.0		424.8711532	55.46	2
S(9): 100.0; T(13): 0.0		302.4596395	61.1	2
S(9): 100.0; T(14): 0.0; S(15): 0.0; T(		372.6474532	58.93	2
Y(3): 0.0; S(11): 100.0; T(13): 100.0		391.75004	83.66	2
T(3): 0.1; T(5): 99.9		108.1936819	6.77	3
S(13): 100.0; S(14): 100.0		241.4407222	61.27	3
S(3): 0.0; Y(7): 0.0; S(11): 99.9; T(15		204.6070389	44.81	2
S(4): 99.7; S(7): 0.3; Y(11): 0.0; S(24		156.0665637	24.43	3
S(2): 0.0; S(12): 0.0; S(16): 3.6; S(17		185.6623363	26.91	3
S(7): 0.0; S(11): 100.0		272.6949054	46.93	3
S(4): 100.0		152.5341676	19.39	2
T(2): 0.0; S(6): 100.0; T(9): 0.0		298.9259626	72.63	2
S(2): 0.0; S(4): 100.0; T(9): 0.0		284.8482919	55.54	2
S(2): 100.0		195.8027261	35.98	2
T(1): 0.0; S(2): 100.0; T(5): 0.0		124.2109572	27.08	2
S(1): 0.0; S(8): 0.0; S(14): 2.0; T(18):		249.3386274	49.33	3
S(2): 25.0; S(4): 25.0; S(6): 25.0; S(7		80.4272709	6.43	2
S(3): 100.0; S(5): 100.0		365.9180274	31.68	2
S(7): 100.0; S(9): 0.0; S(11): 0.0; Y(1		297.2191243	67.67	2
S(9): 100.0		239.9792354	30.3	3
S(3): 100.0		185.0281753	14.05	3
S(3): 99.9; T(5): 0.1		302.1785286	47.52	3
S(5): 0.0; S(9): 100.0		273.8762873	61.01	3
S(3): 50.0; S(4): 50.0; T(9): 0.0		177.4444414	27.89	2
S(9): 0.0; S(11): 100.0; T(13): 0.0		354.395498	82.42	2
S(1): 0.0; S(3): 100.0; S(5): 0.0; S(8):		222.2361981	35.18	2
S(11): 100.0; T(16): 0.0		181.9808756	14.39	3
S(4): 100.0; S(7): 0.0; T(20): 0.0		312.7753669	45.93	4
S(5): 0.0; S(6): 0.0; S(8): 0.0; S(17): 9		319.9759617	61.15	3
S(1): 0.0; S(3): 100.0		207.91694	26.57	2
S(1): 100.0; S(6): 0.0		231.4764955	20.17	2
S(2): 100.0; T(4): 0.0; T(8): 0.0		124.9242799	10.03	2
S(5): 100.0; S(10): 0.0		223.4929872	32.9	2

Information Classification: General

T(2): 0.0; T(8): 0.0; S(11): 0.0; S(17):	262.7067521	33.39	4
S(6): 0.9; T(7): 33.0; S(9): 33.0; S(11	368.1712986	66.69	4
T(2): 0.0; S(4): 0.0; S(5): 100.0	106.557707	26.64	2
S(4): 100.0; S(8): 0.0	215.6782695	38.25	2
T(15): 100.0; T(17): 0.0	362.5513138	74.71	3
T(5): 0.0; S(8): 100.0	184.110457	38.26	2
S(3): 100.0; S(5): 100.0	309.3600946	65.79	3
S(6): 100.0; T(9): 0.0	202.4576292	18.77	2
S(3): 100.0; T(5): 0.0	163.6983741	18.59	2
S(3): 0.0; T(5): 0.0; T(9): 0.0; T(10): 0	293.4545795	46.76	3
T(2): 0.1; S(3): 0.1; S(5): 0.0; T(9): 99	245.8331764	22.34	3
T(16): 100.0	89.22824378	2.97	4
T(6): 0.4; T(9): 99.8; S(10): 99.8	199.3760381	38.73	3
S(9): 98.5; S(11): 1.5	308.3038024	41.16	2
S(6): 100.0	154.1874795	13.32	2
S(3): 100.0	211.5891639	34.14	2
S(1): 0.0; S(3): 96.1; S(7): 3.9; S(14):	142.1895811	21.2	3
S(6): 0.0; S(10): 100.0; S(11): 100.0;	241.8672279	35.84	3
S(5): 100.0; S(6): 0.0	192.5312548	9.71	3
T(3): 0.0; S(7): 0.1; T(8): 99.9	195.5225756	28.29	2
S(2): 97.0; S(6): 2.9; S(8): 0.1; S(10):	202.5517096	16.56	3
S(1): 0.0; S(2): 100.0; S(4): 100.0; S(	238.9771133	56.04	2
T(1): 0.0; S(4): 100.0; S(6): 100.0; T(	175.5480276	20.45	3
S(1): 100.0; T(13): 0.0	304.8879111	52.55	2
S(8): 0.0; T(9): 0.0; S(12): 100.0	391.8865942	94.12	2
T(1): 97.7; S(3): 2.3; S(9): 0.0; S(13):	349.1195471	33.54	4
S(7): 100.0; S(12): 0.0; T(19): 0.0	246.7093241	31.94	3
S(21): 100.0	330.1544468	23.97	4
S(7): 0.0; S(10): 48.6; S(11): 48.6; S(	191.5811323	33.4	3
S(8): 1.0; S(10): 1.0; T(13): 49.0; T(1	316.1104659	70.58	2
S(2): 100.0; T(6): 0.0	131.296382	17.83	2
S(1): 0.0; S(3): 0.0; S(5): 0.0; S(10): 4	309.3707799	58.86	2
T(2): 0.0; S(13): 3.1; S(19): 96.9; T(2	241.8042296	57.3	3
T(1): 0.0; S(3): 100.0; T(6): 0.0; S(8):	273.1391117	52.82	2
S(4): 99.9; Y(7): 0.1; S(11): 100.0	161.0670182	33.37	2
T(1): 0.0; S(4): 100.0	144.4454755	26.71	2
Y(1): 0.0; Y(2): 3.1; S(3): 96.7; S(5): 0	103.1833109	11.73	2
S(4): 100.0	158.2544111	36.64	2
S(2): 100.0; S(4): 0.0; S(7): 0.0; S(8):	261.6895761	56.95	2
S(2): 0.1; S(4): 99.8; S(6): 0.1	121.4107513	31.53	2
S(4): 100.0; S(7): 0.0	252.7567525	43.21	3
S(1): 0.0; S(7): 100.0; S(9): 0.0	219.1563742	37.15	2
S(3): 0.0; T(9): 0.0; S(19): 100.0	239.4898571	76.48	3
S(7): 100.0; T(10): 0.0; S(11): 0.0	349.0517341	89.03	2
Y(3): 0.0; S(7): 99.9; S(8): 0.1; S(13):	225.6856897	26.57	2
S(2): 0.0; S(9): 98.2; S(11): 1.8	237.1846611	58.13	2
S(5): 0.7; S(6): 49.6; S(8): 49.6	249.0578673	41	2
S(2): 50.0; T(3): 50.0	212.585605	51.55	2
S(8): 4.1; T(10): 0.2; S(11): 95.7; S(1	138.7355649	25.92	3
S(8): 100.0; S(13): 0.0	234.6770352	49.53	3
S(14): 50.0; S(23): 50.0; T(29): 0.0	244.4032785	46.81	3
T(13): 50.0; S(15): 50.0	498.6647381	37.73	3
S(7): 0.0; S(8): 0.0; S(11): 100.0	315.3180637	32.73	2
Y(1): 0.0; T(4): 95.7; S(6): 2.1; T(7): 2	146.5068757	17.02	2
T(4): 0.0; S(8): 0.0; T(10): 0.0; S(13):	334.9330725	58.24	3

Information Classification: General

T(13): 95.7; S(23): 4.3		172.9244456	15	4
S(1): 0.0; S(4): 100.0; S(5): 100.0; T(		251.1376835	49.72	2
S(5): 100.0		299.2606257	74.27	2
T(4): 0.0; Y(7): 98.2; Y(8): 1.8; Y(11):		263.4878207	45.33	2
S(3): 100.0; S(6): 0.0; S(8): 0.0; S(9):		152.0774969	26.42	3
S(6): 100.0		265.2898619	34.95	2
S(2): 100.0; S(7): 0.0; S(12): 0.0; S(1		497.377891	138.2	2
S(2): 100.0; T(7): 0.0; T(16): 0.0; T(2		341.726183	69.87	4
S(6): 100.0		215.9378045	19.5	2
S(1): 0.0; S(2): 1.9; S(4): 98.1; S(13):		421.5078264	91.95	2
S(5): 98.7; S(6): 1.3; T(11): 0.0		383.5955548	108.78	2
S(10): 100.0; S(13): 0.0; T(18): 0.0; T		345.9810808	54.72	3
S(6): 0.0; S(17): 95.9; S(19): 4.1; T(2		242.9365999	29.82	3
T(10): 0.0; S(12): 100.0		519.2019955	76.41	3
S(1): 0.0; T(4): 0.1; S(6): 94.7; S(7): 2		138.2501594	27.66	2
S(3): 0.0; S(9): 4.8; T(13): 4.8; S(16):		118.8604536	5.76	3
S(9): 100.0; S(11): 0.0		469.1468578	45.31	3
T(1): 0.0; T(3): 0.0; S(16): 100.0		369.1574934	49.26	4
S(10): 95.9; S(13): 3.9; T(14): 0.2; S(		224.6758047	27.28	4
S(1): 0.0; S(2): 1.9; S(3): 96.2; S(6): 1		132.0465864	30.4	2
S(7): 100.0		268.5653169	33.4	2
S(2): 0.0; S(13): 100.0		397.7878017	69.15	2
S(2): 0.0; S(8): 0.0; S(10): 0.0; S(18):		218.1756877	28.99	4
S(1): 50.0; T(7): 50.0; Y(17): 0.0; T(2		341.3465253	49.66	3
T(3): 0.0; T(6): 0.0; S(7): 0.0; T(10): 0		342.4399042	71	3
S(1): 0.0; S(5): 0.0; S(7): 100.0		170.9214331	19.15	3
Y(2): 0.2; S(5): 91.1; S(7): 4.2; T(8): 0		165.890685	24.36	2
T(9): 100.0; S(11): 0.0		133.1588721	15.47	2
S(4): 0.0; S(8): 100.0; T(10): 0.0; S(1		234.8204683	38.7	2
S(9): 100.0; T(16): 0.0; S(17): 0.0; S(		200.2955726	17.12	4
S(2): 0.0; S(3): 0.0; S(5): 0.0; S(7): 0.		133.2507123	10.64	3
S(1): 0.1; S(2): 99.9; S(6): 0.0; S(8): 0		191.8237293	39.18	3
T(4): 100.0		193.5593888	35.81	3
S(1): 0.0; S(2): 0.0; S(3): 0.0; T(7): 0.		298.9782976	55.05	3
S(4): 0.0; S(6): 0.0; T(14): 100.0; S(2		463.0438443	69.33	3
S(3): 0.2; S(6): 99.8; T(16): 0.0		118.3078339	29.69	2
S(1): 0.0; S(6): 100.0; T(10): 0.0; S(1		134.8868658	16.68	3
S(1): 100.0; S(6): 0.0; S(8): 0.0; S(12		271.5072541	53.68	2
S(9): 0.0; S(17): 100.0; S(21): 0.0		474.7403714	93.91	2
S(8): 0.0; S(13): 0.0; S(17): 100.0		278.7954952	29.95	4
S(3): 100.0		233.9514086	20.99	4
S(5): 100.0; S(12): 0.0		207.5342695	24.5	3
Y(10): 0.0; S(15): 100.0		264.683721	30.24	3
S(3): 100.0; S(7): 0.0; T(14): 0.0		235.1776696	60.15	2
S(10): 100.0		150.1289971	22.23	3
S(8): 99.1; S(12): 99.1; S(15): 1.8		72.52885637	5.49	3
T(2): 0.0; S(8): 49.4; S(11): 49.4; T(1		206.5937634	62.79	3
S(2): 0.8; Y(3): 99.2		330.8987363	49.31	2
S(4): 0.0; T(22): 100.0; S(30): 0.0; S(		229.0378567	30.39	4
S(4): 100.0; S(8): 0.0; S(10): 0.0; S(1		395.0375688	57.59	3
S(1): 0.0; S(3): 100.0; S(8): 0.0		265.9879698	31.38	2
S(1): 94.2; S(2): 2.9; S(3): 2.9; T(5): 0		114.4908355	14.54	2
T(15): 100.0		193.8961046	27.35	3
S(1): 0.0; S(7): 100.0		428.8330789	49.24	2
S(1): 0.0; T(3): 0.0; S(5): 99.9; S(6): 9		343.9238488	96.64	2

S(5): 100.0		248.3986837	27.79	3
S(7): 99.9; S(12): 0.1; T(13): 0.0		220.2064021	29.16	2
T(1): 0.0; S(3): 100.0; T(7): 0.0; Y(10)		395.1890389	104.94	2
S(5): 100.0; S(6): 0.0		256.6506107	38.56	2
T(1): 0.0; S(4): 0.0; S(12): 0.0; S(22):		515.0824602	96.82	3
S(8): 0.4; S(10): 0.0; T(12): 0.0; S(15		113.6737276	7.63	3
T(1): 4.6; S(2): 90.9; S(3): 5.1; S(6): 9		111.4086994	10.7	3
S(10): 100.0		342.5975446	48.73	2
S(1): 0.0; T(3): 0.1; S(4): 0.1; S(5): 3.		175.9022072	46.48	2
S(8): 100.0; T(14): 0.0; S(15): 0.0; T(		224.2650763	27.3	3
S(1): 0.1; S(4): 97.6; T(5): 2.3; S(6): 0		204.0807269	23.65	2
S(3): 100.0; S(6): 0.0		161.2410713	27.53	3
S(1): 0.0; S(9): 100.0; T(11): 0.0		358.8588427	44.88	2
S(2): 100.0; S(5): 0.0; Y(7): 0.0; S(8):		309.4912981	70.67	2
S(8): 100.0; T(12): 0.0; T(14): 0.0		378.7120243	86.26	2
Y(3): 0.0; S(5): 100.0		189.8412171	23.92	2
S(3): 0.0; S(6): 0.0; T(12): 100.0; S(1		476.3562828	49.8	3
S(8): 100.0		258.8423005	29.78	2
T(4): 92.2; S(9): 7.8; S(13): 92.2; T(1		92.23253377	16.36	3
S(4): 100.0; S(8): 0.0; S(11): 0.0; S(1		171.0089986	20.72	3
S(9): 2.3; S(10): 95.4; T(12): 2.3		207.3450554	41.17	3
S(1): 0.0; S(3): 100.0; S(6): 0.0		185.9826103	30.91	2
T(5): 0.0; S(10): 99.9; S(11): 0.1		147.7553475	14.61	3
S(6): 0.0; S(16): 3.2; S(17): 96.8		204.6287029	34.43	3
S(2): 0.0; S(4): 100.0; T(10): 0.0; T(1		329.6902069	68.23	2
S(4): 0.0; S(10): 96.7; S(13): 3.3		131.8340357	33.21	3
T(2): 0.0; S(4): 100.0		257.095696	47.91	2
S(4): 100.0; S(7): 0.0		234.8204683	39.49	3
T(1): 0.0; T(13): 100.0; S(15): 0.0		316.2114271	86.88	2
S(10): 1.4; T(11): 49.3; S(12): 49.3		259.7297598	59.84	2
S(4): 100.0; T(7): 0.0; S(17): 100.0; Y		367.4159817	39.47	3
S(1): 0.0; S(4): 0.0; S(5): 0.0; S(7): 0.		458.9161516	117.89	3
T(1): 0.0; S(4): 100.0; Y(16): 0.0		267.8462363	51.88	3
S(5): 0.0; S(17): 100.0; T(23): 0.0		196.7485593	14.63	3
S(2): 0.0; S(6): 100.0; S(24): 0.0		139.900209	9.11	3
T(2): 0.0; T(15): 0.0; T(18): 50.0; S(1		243.71879	47.67	3
T(1): 0.0; S(3): 0.0; Y(8): 0.0; S(14): 1		233.8678687	70.84	2
T(1): 0.0; S(7): 100.0		197.0879032	37.25	2
S(4): 100.0; Y(21): 0.0; S(22): 0.0; S(		177.6114777	37.63	3
S(5): 0.0; S(9): 0.2; S(10): 0.2; S(14):		141.4835981	13.57	3
S(2): 0.0; S(4): 0.0; T(10): 0.0; S(12):		332.9648704	81.02	2
S(4): 0.0; S(7): 0.0; T(8): 0.0; S(20): 1		238.2477654	24.56	3
S(1): 0.0; S(3): 100.0; Y(7): 0.0		166.9082898	13.83	2
T(2): 3.3; S(9): 96.6; S(14): 0.1; S(16		153.7730197	11.78	2
S(1): 100.0; S(7): 100.0		178.5343345	14.33	3
S(8): 0.0; S(13): 0.0; S(16): 97.3; S(1		364.5629361	70.72	3
T(14): 48.5; T(15): 48.5; T(20): 2.9; T		151.3585344	18.7	5
S(9): 100.0; T(11): 0.0; S(12): 0.0; S(		324.8206102	64.36	2
T(11): 0.0; S(17): 100.0; T(21): 0.0; T		531.703018	72.9	3
T(5): 0.0; S(6): 0.0; S(10): 0.0; S(13):		162.0248965	35.71	3
S(3): 100.0; S(7): 0.0		163.2378877	20.58	2
S(1): 0.0; T(3): 0.0; T(10): 0.0; T(14):		327.3964102	69.04	2
S(6): 50.0; S(7): 50.0		215.9282442	22.14	3
T(4): 0.0; S(23): 100.0; T(27): 0.0		402.1318288	24.11	5
S(2): 3.6; S(4): 96.4		205.9840278	28.56	4

Information Classification: General

Y(3): 0.0; S(14): 99.9; T(17): 0.1; T(2(	283.1675549	48.48	3
S(3): 1.6; T(5): 98.4; S(9): 0.0	149.5113791	21.95	2
S(1): 2.1; Y(2): 2.1; S(3): 93.7; S(4): 2	298.1249399	58.93	3
S(4): 0.0; S(7): 100.0; S(9): 0.0	384.4107393	31.71	3
S(2): 1.8; T(3): 48.2; T(4): 48.2; S(5):	133.4347695	19.57	3
S(1): 0.0; S(5): 0.0; S(9): 100.0	267.9993409	31.83	2
T(6): 0.0; Y(8): 0.0; S(9): 100.0	531.1771937	104.07	2
S(5): 100.0; S(17): 0.0	130.9651609	52.98	3
S(1): 0.0; Y(10): 4.1; S(11): 95.9; Y(1	165.1504491	3.66	3
T(2): 0.0; S(3): 100.0	189.000168	20.71	2
S(7): 0.0; T(18): 100.0; T(27): 0.0; T(3	439.4502358	83.34	4
S(4): 0.0; T(6): 0.0; T(7): 0.0; S(11): 1	414.9863972	76.79	2
S(4): 100.0; S(6): 0.0; S(9): 0.0; S(10	332.7213829	77.6	2
S(10): 2.8; S(13): 2.8; T(15): 2.8; T(1	164.957212	18.84	3
S(4): 100.0; S(7): 0.0; T(8): 0.0; S(12	289.954465	53.61	2
T(1): 0.0; S(15): 0.0; S(19): 100.0	219.2921786	24.45	3
T(2): 0.0; T(7): 100.0; T(9): 0.0; S(13)	385.6513424	43.87	3
Y(2): 0.0; T(4): 0.0; T(11): 0.0; S(13):	236.4493016	26.41	3
S(1): 100.0; S(6): 0.0; S(10): 0.0	282.1477894	46.87	2
T(3): 6.9; S(6): 93.1; S(8): 100.0; T(1	115.4370246	7.08	3
T(8): 100.0; S(10): 95.8; S(11): 4.2; Y	201.1993609	25.74	2
S(3): 96.8; T(5): 3.2; S(12): 0.0; T(16)	253.7710968	36.53	3
S(2): 0.0; S(11): 100.0	249.7159307	49.56	3
S(1): 0.0; S(12): 2.2; S(13): 32.6; S(1	173.8681997	14.23	5
S(1): 0.0; T(5): 2.2; S(7): 97.8	138.8495255	19.31	2
S(5): 100.0; S(9): 100.0; T(14): 0.0; S	129.1627615	20.83	3
S(3): 100.0; T(9): 0.0; T(12): 0.0; S(14	329.2149376	52.03	3
T(1): 2.0; S(5): 98.0; S(11): 0.0; S(18	471.8474494	100.8	3
S(3): 100.0; S(5): 0.0; S(8): 0.0	129.7804693	10.95	2
S(4): 0.0; S(7): 100.0; T(10): 0.0	286.4538683	51.97	2
S(8): 51.3; S(10): 51.3; S(12): 97.4	110.5173007	6.46	3
S(1): 0.0; T(5): 0.0; T(7): 0.0; S(9): 10	339.6879521	79.75	2
S(4): 50.0; S(5): 50.0	106.2323103	9.38	3
S(3): 100.0; T(14): 0.0; S(15): 0.0	405.7186161	48.22	3
S(1): 0.0; S(2): 100.0; Y(4): 0.0; S(12	292.5785288	45.83	2
S(12): 99.5; S(13): 99.5; T(15): 99.5;	92.72841081	1.84	4
S(2): 0.0; S(3): 100.0; S(5): 0.0; S(8):	185.2382117	43.41	2
S(3): 97.5; S(6): 2.5; Y(10): 0.0; S(13	227.1541818	51.77	3
S(1): 0.0; S(3): 0.0; S(6): 96.9; S(7): 3	219.8971884	64.6	3
T(2): 50.0; S(3): 50.0; T(7): 0.0	149.8844481	29.51	3
T(3): 0.0; S(8): 100.0	206.7791023	33.57	3
S(2): 0.2; S(4): 99.8	105.8202711	15.49	3
S(7): 97.2; S(9): 50.0; T(11): 50.0; T(	163.1405037	59.8	3
T(3): 2.5; S(5): 97.5; S(7): 0.0	132.747061	9.9	2
T(1): 0.0; S(6): 100.0	218.3626809	41.55	2
S(8): 0.0; S(12): 100.0	309.7900643	41.16	2
S(5): 100.0	204.8599955	12.27	2
S(3): 0.1; T(6): 2.1; S(8): 2.1; S(9): 47	157.7825749	31.37	3
T(1): 0.0; S(11): 100.0	333.2917477	58	2
S(1): 0.0; T(4): 100.0; T(7): 0.0; T(11)	311.8491848	78.43	2
S(2): 0.0; S(4): 0.0; S(8): 100.0; S(13	216.8238347	31.8	2
S(2): 100.0; S(15): 0.0	415.4206806	82.77	2
T(1): 0.0; S(14): 100.0; T(19): 0.0	231.0173648	14.59	3
S(4): 0.0; S(8): 100.0	130.8892949	9.9	3
S(1): 0.0; S(3): 0.0; S(13): 2.3; S(14):	351.6971533	65.87	2

Information Classification: General

S(6): 0.0; S(10): 100.0		383.0063038	41.75	3
T(5): 0.0; T(8): 66.7; T(9): 66.7; T(10)		153.8125396	35.43	3
S(18): 100.0		353.1709858	36.55	2
T(1): 0.0; T(4): 0.0; S(17): 100.0; S(2		438.9616693	91.36	2
S(4): 100.0; T(6): 0.0; S(8): 0.0		152.3523714	26.07	2
T(8): 2.3; S(9): 97.7; S(16): 0.0		254.461472	39.92	3
S(5): 100.0		196.8340271	39.87	2
S(3): 0.1; S(4): 0.1; S(5): 0.0; S(6): 0.		216.3797782	26.24	2
S(4): 0.0; T(6): 0.0; S(9): 100.0		319.9835182	50.04	2
T(6): 0.0; S(8): 0.0; S(11): 0.0; S(12):		276.5232658	70.3	3
T(6): 0.0; S(11): 50.0; S(13): 50.0		314.2362499	53.51	2
T(1): 0.0; S(3): 100.0; S(5): 50.0; S(6		244.8459671	62.29	2
S(8): 100.0; S(23): 0.0; S(27): 0.0		477.1639126	82.92	3
Y(5): 0.0; T(10): 0.0; S(11): 0.0; S(15		300.1057262	66.5	3
S(20): 100.0; S(30): 0.0; T(31): 0.0		281.385086	47.28	3
T(1): 0.0; S(13): 0.0; S(20): 0.0; S(23		490.004641	117.99	2
Y(2): 0.0; Y(3): 0.2; S(4): 99.5; S(5): 4		106.0536052	12.41	2
S(1): 100.0		315.624425	31.76	2
S(9): 100.0		191.5229342	20.49	3
T(3): 0.0; S(8): 100.0; T(10): 0.0; S(1		183.5931241	35.67	3
S(7): 100.0; T(9): 0.0; S(10): 0.0; S(1		223.9555481	31.88	3
S(2): 0.0; S(3): 100.0; S(5): 0.0; T(6):		438.4947555	103.51	2
S(6): 0.0; S(8): 0.0; S(9): 0.0; S(20): 9		144.8460194	27.95	3
Y(9): 50.0; T(11): 50.0; Y(15): 0.0		66.44419162	15.18	2
S(3): 0.0; S(4): 100.0		271.2710517	46.46	2
S(4): 100.0; S(7): 0.0; T(15): 0.0		383.4351051	81.42	2
S(5): 100.0		289.0337876	27.56	2
S(1): 0.1; S(3): 99.9		132.3694053	27.53	2
S(1): 0.0; S(8): 0.1; S(10): 50.0; T(13		111.2730358	10.12	2
T(2): 0.0; S(5): 0.0; S(6): 0.0; S(16): 1		317.5392904	72.88	3
S(10): 98.7; S(12): 1.3		374.3037054	64	2
S(2): 0.0; S(4): 100.0; S(8): 100.0		210.7389629	54.36	2
T(6): 100.0; S(19): 0.0; S(25): 4.9; Y(		306.6919981	72.38	3
T(6): 0.0; S(7): 0.0; T(10): 0.0; T(11):		333.6654973	56.86	2
T(2): 0.3; S(8): 94.5; T(11): 5.2		108.7385913	5.35	4
T(1): 0.0; S(8): 0.0; S(14): 48.3; S(15		97.53622857	2.85	3
S(1): 0.0; S(3): 100.0; T(10): 0.0		203.9290567	9.67	3
T(2): 0.0; S(6): 100.0; S(9): 0.0; S(10		284.5760213	88.64	2
S(3): 1.9; S(5): 98.1		244.8352885	45.64	2
T(1): 0.0; T(3): 100.0; S(6): 100.0; S(		189.3240061	19.82	3
S(2): 0.0; Y(5): 0.1; S(12): 99.9; S(17		245.6085343	67.8	3
S(4): 98.3; T(5): 1.7; S(9): 0.0; T(16):		294.0373707	46.44	2
S(2): 100.0		219.1563742	39.33	2
T(8): 0.0; S(15): 100.0; T(24): 0.0		151.2663477	24.55	4
T(6): 100.0; S(9): 50.0; S(10): 50.0		174.1490326	31.19	2
S(2): 0.0; Y(3): 100.0		260.3956313	43.11	2
S(1): 100.0		277.6851781	48.48	2
S(1): 0.0; S(3): 0.0; S(9): 100.0; T(13		506.1288103	78.86	2
T(5): 100.0; S(8): 5.3; S(11): 94.7; S(		246.8593205	30.35	3
S(2): 0.2; S(5): 88.4; S(6): 3.8; S(7): 3		249.6690367	70.53	4
T(3): 0.0; S(9): 100.0; S(10): 0.0		172.0651431	6.33	2
T(1): 1.3; T(3): 49.4; S(5): 49.4; S(14		177.8701071	26.02	2
S(4): 100.0; T(9): 0.0		172.8877223	17.22	2
T(3): 0.0; S(8): 0.0; S(17): 99.9; T(19		255.736415	71.16	3
S(1): 100.0; S(7): 100.0; S(10): 0.0		250.2114474	31.44	2

Information Classification: General

S(4): 0.0; S(5): 0.0; S(8): 100.0; S(10	212.9716485	15.91	3
S(3): 0.0; S(4): 100.0; T(8): 0.0	142.6226224	35.16	2
S(5): 100.0; S(9): 0.0; S(18): 0.0	369.3165685	41.95	3
S(4): 100.0	366.2890989	22.56	3
Y(5): 0.0; Y(10): 0.0; T(11): 0.0; S(13	215.9074255	32.84	3
Y(4): 0.0; S(13): 100.0	301.1863111	39.72	2
S(2): 0.0; T(4): 0.0; S(6): 100.0	361.8710832	81.54	2
T(2): 0.1; S(5): 96.5; T(7): 3.4; S(13):	230.2425461	17.47	3
T(2): 50.0; S(4): 50.0; S(8): 0.0; T(12	153.4571986	16.62	2
S(5): 100.0; T(8): 0.0	274.6124348	37.99	2
S(2): 100.0; T(10): 0.0	194.5471513	33.97	3
S(3): 100.0; S(7): 0.0	288.0139726	42.56	3
S(3): 0.0; T(7): 0.0; S(9): 0.0; S(10): (	382.9084177	36.77	3
S(1): 0.1; S(4): 99.9; S(6): 3.1; S(8): 9	209.1904856	34.52	3
S(6): 100.0	336.350683	49.64	3
S(3): 0.0; S(5): 0.0; S(8): 0.0; S(10): 1	265.6890596	37.8	3
S(1): 0.0; S(4): 0.0; S(5): 0.0; S(7): 0.	384.3162509	98.5	3
S(2): 100.0; Y(6): 0.0	250.3514274	34.47	2
S(1): 0.3; T(3): 99.7; T(6): 95.0; T(11	90.78458468	32.49	3
S(2): 100.0; Y(7): 0.0	162.7972576	9.23	2
S(2): 100.0; S(7): 0.0; S(12): 0.0; S(1	299.7296133	107.23	2
S(4): 0.0; S(11): 100.0; S(15): 0.0	115.5287247	11.43	2
S(1): 0.0; S(3): 100.0; S(5): 0.0; S(6):	120.6338312	9.28	2
T(4): 100.0	215.0539452	36.95	2
T(1): 0.0; T(2): 100.0	118.5948205	19.68	2
S(5): 100.0	237.6033045	29.79	3
S(3): 2.3; S(5): 97.7; S(11): 0.0; S(14	230.6830062	17.18	3
S(3): 100.0	138.9916433	14.36	2
S(1): 100.0; S(8): 0.0; S(11): 0.0	146.9815814	23.75	2
T(1): 2.4; S(2): 97.6; S(11): 0.0; T(16	319.7006345	57.16	3
T(7): 6.7; T(8): 93.3; S(15): 0.1	58.68613337	8.15	3
S(6): 100.0	492.0277731	93.09	3
S(1): 100.0; Y(10): 0.0; T(11): 0.0	168.1231562	16.71	3
S(1): 47.7; S(2): 47.7; T(4): 2.2; S(5):	138.2633051	18.61	3
S(2): 100.0; S(4): 0.0; S(6): 0.0	247.2311222	31.68	2
T(3): 0.0; S(4): 0.0; S(10): 100.0	314.6603067	74.22	2
S(3): 100.0	139.5225355	16	3
T(1): 0.0; S(2): 1.1; T(3): 49.5; S(4): 4	202.4181121	54.61	2
T(1): 0.0; S(3): 0.0; S(6): 100.0; S(11	293.8155648	31.04	2
S(5): 0.0; T(6): 0.0; T(8): 0.0; S(12): (	194.3400474	33.01	3
T(2): 0.0; S(7): 100.0	292.2620456	46.75	2
T(6): 0.0; S(23): 100.0; Y(26): 0.0	295.7803133	40.36	3
S(3): 0.0; S(6): 50.0; S(8): 50.0	132.8763302	11.71	2
T(4): 0.0; T(5): 0.0; S(8): 100.0	274.3319571	51.27	2
T(7): 0.8; S(8): 49.6; T(9): 49.6	248.8001004	29.44	2
T(3): 100.0	180.6181347	17.69	2
S(5): 100.0; S(7): 0.0; S(13): 0.0	309.4111884	51.28	4
S(1): 50.0; S(2): 50.0; S(3): 7.2; S(4):	174.3570733	18.54	3
S(1): 100.0	387.6826771	25.26	3
S(1): 0.0; Y(7): 0.0; T(11): 87.0; S(15	129.4774719	12.76	3
T(8): 100.0	227.6055934	23.3	3
S(11): 100.0; S(13): 100.0	252.3907714	23.92	3
S(3): 98.0; S(4): 2.0; T(15): 0.0	394.170242	40.55	3
S(1): 0.0; S(2): 100.0	282.5207998	50.39	2
T(1): 0.0; T(2): 0.0; S(4): 0.0; T(7): 10	396.0711157	38.55	2

Information Classification: General

T(4): 0.1; S(5): 0.1; S(7): 99.9		118.3864472	22.66	2
S(11): 99.4; S(14): 7.8; S(19): 92.9		85.53430768	3.7	3
S(10): 96.8; S(11): 3.2; S(15): 100.0;		288.3116826	31.5	3
S(8): 100.0; S(21): 0.0		168.4474655	20.18	3
S(1): 0.0; S(7): 100.0		427.4743013	52.35	2
S(14): 100.0; T(19): 0.0		234.6585909	56.26	4
T(2): 0.0; S(13): 2.9; S(15): 91.2; T(1		462.805113	89.92	4
T(6): 0.0; S(8): 0.0; S(10): 0.0; T(17):		408.3424107	68.6	2
T(1): 0.0; S(7): 0.0; S(10): 100.0		224.9160821	48.12	3
T(8): 0.8; S(9): 9.4; T(10): 90.3; S(11		159.4024446	31.17	3
S(7): 100.0; S(9): 100.0; S(11): 0.0		278.3723344	63.39	2
S(1): 100.0; S(7): 100.0; S(13): 100.0		390.1540326	29.41	2
S(8): 100.0; S(10): 0.0; S(12): 0.0		328.4392394	63.08	2
S(1): 2.7; S(2): 2.7; S(3): 94.6; S(6): (		238.4362659	46.57	3
T(1): 0.0; S(2): 0.0; S(10): 99.8; S(11		285.8325528	68.82	3
Y(10): 97.3; T(11): 2.7; Y(15): 0.0; S(		306.2275249	78.7	3
S(3): 98.4; S(4): 1.6		266.14713	46.73	2
T(2): 0.0; S(5): 100.0; T(7): 0.0; T(10		240.2849006	44.25	2
S(1): 0.1; S(5): 92.5; S(7): 92.5; T(9):		165.2925503	31.3	3
S(2): 100.0; T(8): 0.0		168.2631362	8.43	3
S(10): 100.0; S(13): 95.9; S(14): 4.1;		348.0123459	60.81	3
S(3): 100.0; S(4): 0.0; S(7): 0.0		216.65911	40.26	3
S(4): 0.0; S(7): 0.1; S(9): 99.9		113.3373474	6.17	3
Y(5): 0.0; Y(10): 0.0; T(11): 0.0; S(13		323.6629593	47.87	4
S(9): 100.0; T(13): 0.0		131.8340357	4.93	3
S(4): 0.0; S(5): 0.0; S(7): 0.0; S(9): 10		278.3065935	80.25	2
T(3): 0.0; S(5): 100.0		381.3536774	54.49	2
S(3): 100.0; T(4): 0.0; T(5): 0.0		290.7610022	31.9	2
S(2): 99.2; S(4): 91.7; S(7): 9.1; S(11		58.75340783	6.83	3
Y(3): 0.0; T(6): 95.0; T(9): 2.5; S(10):		173.2606074	19.09	2
T(1): 0.0; T(3): 100.0; S(5): 97.7; T(7)		174.1524626	19.07	2
S(2): 0.0; S(5): 100.0; S(9): 0.0		254.2704175	62.94	2
S(6): 0.0; S(7): 0.0; S(9): 0.0; T(16): (		312.2010193	97.89	3
S(1): 0.0; S(5): 1.4; T(8): 97.3; S(9): 1		291.2007059	42.96	2
S(3): 0.0; S(9): 100.0; T(14): 0.0; S(1		466.9732522	96.32	2
S(1): 0.0; S(7): 100.0; T(15): 0.0		176.9331191	16.27	3
S(3): 100.0; S(10): 0.0		305.4895937	33.06	2
S(3): 0.0; S(4): 0.0; S(11): 100.0		246.6275454	29.66	3
T(3): 100.0		290.2825866	59.3	3
S(4): 0.0; S(9): 66.7; S(11): 66.7; S(1		287.1384194	46.3	3
S(9): 49.3; S(10): 49.3; T(11): 1.3; Y(		220.9022027	46.58	3
S(1): 0.0; T(2): 0.0; S(3): 0.0; S(6): 0.		221.7212327	57.43	2
S(1): 0.0; S(8): 100.0		290.0022719	45.37	2
S(7): 100.0; T(9): 0.0		198.5976271	20.54	2
T(5): 0.0; S(7): 100.0; T(18): 0.0; T(1		301.6443392	79.99	2
S(3): 0.0; T(11): 50.0; S(12): 50.0; Y(		264.7576027	51.36	3
S(5): 100.0; S(8): 100.0; T(13): 100.0		261.7971259	55.32	2
S(1): 100.0; S(3): 0.0; S(4): 0.0; S(5):		169.3884981	58.9	2
S(2): 3.6; T(4): 96.4; T(9): 0.0; T(14):		253.4776079	56.87	3
S(1): 0.0; S(6): 0.0; T(11): 100.0; S(1		303.667784	47.91	2
S(5): 100.0; T(7): 0.0; S(14): 0.0		224.8473225	25.93	2
S(1): 0.0; S(3): 100.0; Y(11): 0.0		112.6794072	12.61	2
T(3): 33.3; S(4): 33.3; S(5): 33.3; T(1		161.8185691	17.5	2
S(1): 0.0; T(4): 0.0; S(10): 50.0; S(12		393.146863	34.27	3
T(1): 89.1; T(2): 3.6; T(3): 3.6; S(4): 3		246.1346201	52.71	3

Information Classification: General

S(5): 100.0; S(6): 100.0; S(15): 100.0	82.9601031	16.98	3
S(5): 100.0; S(7): 0.0	288.1654886	52.64	2
T(6): 0.2; S(8): 99.8; Y(15): 0.0	193.3917201	21.25	3
S(1): 0.0; S(2): 0.0; S(3): 0.0; T(7): 0.	223.2401908	33.11	3
S(2): 100.0	156.8289134	26.36	2
S(1): 1.9; S(2): 96.2; S(3): 1.9; T(6): (	306.1534616	37.09	2
S(3): 100.0; S(5): 100.0	112.2754654	13.7	2
T(4): 3.8; S(7): 3.8; S(8): 92.3; T(10):	122.6261093	19.58	3
T(7): 0.0; S(11): 0.4; T(12): 0.0; S(17)	155.5539225	14.94	3
S(13): 100.0	184.9314931	8.38	6
T(1): 0.0; T(5): 0.0; S(10): 98.4; S(14)	346.7125587	58.22	2
S(2): 100.0; S(4): 100.0	300.3085071	41.41	3
S(6): 100.0; T(8): 0.0; T(11): 0.0	289.2213909	91.09	2
T(2): 33.3; S(3): 33.3; T(5): 33.3	144.4258971	6.52	4
S(2): 97.7; T(4): 2.3	89.56586981	28.95	2
S(3): 99.9; S(7): 0.1; S(11): 0.0; S(21	373.6525538	75.05	3
S(3): 98.7; Y(4): 1.3	272.1795395	64.7	2
Y(1): 0.0; S(2): 0.0; T(3): 0.0; S(6): 10	147.557027	25.45	2
T(1): 1.6; S(2): 49.2; S(3): 49.2; S(6):	291.9903744	31.45	3
Y(4): 0.0; S(6): 100.0	167.5207227	24.01	3
S(2): 0.0; S(13): 0.0; S(14): 0.0; S(15	195.2563359	31.79	3
S(6): 100.0	313.8581757	50.32	2
S(2): 0.0; S(4): 0.0; S(16): 100.0; Y(1	211.7299239	29.94	3
S(6): 100.0; S(9): 100.0; S(14): 0.0	182.1154485	26.77	3
S(2): 7.0; T(3): 0.6; S(7): 92.3; T(11):	121.373683	5.71	3
S(1): 2.0; S(2): 96.1; S(3): 2.0; T(11):	351.2382046	60.17	2
S(3): 0.0; S(5): 0.0; T(8): 0.0; S(12): 1	241.0666122	44.99	3
S(4): 0.2; S(7): 99.8; S(14): 0.0; S(18	163.3189823	25.28	3
T(2): 49.4; S(3): 49.4; S(5): 1.2; S(8):	225.4314126	21.92	4
S(2): 100.0; Y(6): 0.0	248.2518011	57.89	2
S(3): 100.0; S(4): 100.0	343.8893044	48.09	2
S(9): 50.0; S(11): 50.0	319.9264392	41.69	3
S(7): 0.0; S(15): 98.1; S(19): 1.9	356.2351752	70.34	2
T(1): 0.0; S(3): 0.0; S(13): 100.0	375.6742388	35.34	3
S(3): 100.0; S(5): 0.0; T(10): 0.0	201.1922261	26.7	2
S(9): 100.0; S(11): 100.0	442.1766518	65.78	3
S(7): 0.0; T(13): 0.0; T(15): 0.0; T(17)	427.9227884	114.73	3
S(4): 0.0; T(6): 0.1; S(8): 99.9; T(17):	248.6755457	65.75	3
S(7): 100.0	142.4122698	7.61	2
S(6): 100.0; S(8): 0.0; S(12): 0.0; S(1	370.5291868	54.95	3
S(4): 100.0	198.4896227	29.4	2
S(4): 100.0	253.862817	41.85	2
S(3): 97.5; S(5): 2.5	232.6982998	27.42	3
S(5): 0.0; S(6): 0.0; S(9): 100.0	343.9797734	70.38	2
S(6): 100.0; T(15): 0.0	255.3720344	38.33	2
S(11): 0.0; S(14): 1.6; S(15): 98.4	360.0189502	63.62	2
Y(7): 50.0; S(8): 50.0	141.7488053	20.61	2
S(2): 0.0; S(3): 0.0; T(6): 50.0; S(7): 5	156.8791013	39.08	2
S(1): 0.0; S(10): 100.0	271.7059119	35.26	3
S(2): 0.0; S(5): 0.0; S(7): 100.0	296.7080363	78.07	2
Y(6): 33.3; S(7): 33.3; T(9): 33.3; Y(1(	89.93518409	3.43	3
T(3): 0.0; S(4): 0.0; S(6): 0.0; S(8): 0.	275.7881212	68.1	3
T(1): 0.0; T(3): 0.0; S(10): 0.1; T(11):	275.9108982	50.26	4
S(4): 99.9; S(10): 0.1; Y(17): 0.0	211.3590975	77.16	3
S(3): 1.8; S(6): 98.2	135.6442286	27.8	2

Information Classification: General

T(1): 0.0; S(3): 100.0; S(10): 0.0; S(1	304.9087949	67.25	2
S(1): 0.0; S(3): 0.0; S(6): 0.0; S(7): 0.	201.1032173	30.03	2
S(1): 0.0; S(3): 100.0; S(9): 99.9; T(1	330.7936762	54.81	2
S(6): 100.0; T(12): 0.0	382.6558968	67.45	2
S(6): 0.0; S(8): 0.0; S(11): 100.0	144.0217318	9.59	3
T(8): 96.8; S(9): 3.2	162.9021045	12.31	2
S(1): 91.7; S(3): 8.3; T(19): 0.0; T(25	123.0750093	19	5
S(1): 0.0; T(2): 0.0; T(6): 0.0; T(11): 9	248.8037793	30.2	3
S(2): 3.2; S(3): 96.8; S(7): 0.0; S(9): (	141.4035874	24.25	3
T(6): 0.0; S(9): 0.0; S(12): 100.0	310.5113065	83.59	2
S(11): 32.7; S(14): 32.7; S(15): 32.7;	123.2349656	4.87	3
S(4): 96.4; S(5): 3.6; S(11): 0.0; T(15	185.1191817	25.5	3
S(3): 0.0; S(9): 100.0	298.2132688	47.44	2
S(7): 100.0; S(10): 0.0	403.3947972	64.1	2
S(1): 0.0; S(3): 0.0; S(6): 100.0; S(9):	319.4800665	55.5	3
S(2): 0.1; S(3): 99.9; T(6): 0.1	168.6740634	35.41	2
S(8): 0.0; S(13): 100.0; S(17): 0.0	361.8075406	49.08	4
S(1): 97.2; S(3): 2.7; S(5): 0.1; T(7): (	240.1210724	42.76	3
S(6): 0.0; T(8): 0.0; Y(11): 0.0; S(15):	360.2672711	91.09	3
S(5): 98.2; T(10): 1.8	235.766301	54.59	2
Y(9): 100.0	218.3164581	21.74	2
S(11): 100.0; S(13): 99.9; T(17): 0.1	344.9387557	45.72	3
S(5): 0.0; S(6): 0.1; S(7): 97.7; S(9): 2	299.6797161	82.45	2
S(4): 91.7; T(6): 4.0; T(7): 4.0; T(10):	91.4248211	16.83	3
S(8): 100.0; T(16): 0.0	134.1047106	14.22	3
T(13): 0.0; Y(15): 0.0; T(18): 0.0; S(2	341.2155711	87.07	3
S(1): 0.0; Y(7): 0.0; T(11): 99.7; S(15	191.9326407	48.16	3
T(7): 0.1; T(9): 99.9	163.4438813	11.84	3
S(3): 0.0; S(5): 0.0; S(6): 100.0	375.4734785	89.22	2
S(4): 100.0	203.7653114	31.36	2
S(3): 0.0; S(6): 0.0; T(9): 0.0; T(11): (	462.1231991	86.6	3
S(6): 50.0; T(8): 50.0; S(12): 99.9; T(	143.3224174	7.55	4
T(5): 0.0; S(14): 100.0	375.1212969	36.91	3
S(1): 0.0; S(3): 100.0; S(5): 0.0; S(7):	283.6900437	61.01	2
S(1): 0.0; S(2): 0.0; S(4): 0.0; S(9): 0.	182.4698262	37.31	3
T(5): 50.0; S(8): 50.0	144.1604465	22.76	2
S(4): 100.0; Y(8): 0.0	208.7935823	26.96	2
S(1): 0.0; S(5): 100.0	229.6419149	31.15	2
S(4): 100.0	266.5321313	50.02	3
S(2): 0.0; S(3): 0.0; S(6): 100.0	236.822701	45.9	2
S(2): 93.0; Y(3): 7.2; S(11): 49.9; T(1	172.9563427	16.01	4
T(2): 0.0; Y(5): 0.0; S(12): 100.0; S(1	199.6543129	56.36	3
T(1): 0.0; S(8): 0.0; S(12): 100.0	488.5377419	97.78	2
S(10): 100.0; T(14): 0.0	424.4613682	53.94	2
S(2): 0.0; S(10): 7.0; S(11): 7.0; S(13	221.5814805	50.75	3
S(4): 0.0; T(7): 0.0; T(12): 0.0; S(14):	345.0133075	109.32	3
S(6): 0.0; S(7): 0.0; S(9): 0.0; T(16): (	268.0482409	65.04	3
S(1): 4.1; S(3): 95.9; S(7): 0.0; S(12):	286.1359152	27.06	3
S(3): 99.8; S(4): 0.2; S(5): 2.9; S(6): 9	183.1138996	51.85	2
T(3): 0.2; T(4): 0.2; T(8): 0.2; T(10): 4	230.9237357	34.67	3
S(1): 0.0; S(4): 100.0; S(12): 0.0	314.0330761	59.45	2
T(1): 0.0; S(4): 0.0; T(11): 0.0; S(13):	223.6600325	24.19	3
S(7): 95.9; S(8): 4.3; S(10): 95.7; T(1	271.0252539	48.04	3
T(4): 0.0; T(5): 0.0; S(8): 100.0; S(12	220.9516543	25.27	4
S(6): 0.1; S(8): 99.9	174.1403901	26.98	2

Information Classification: General

S(2): 0.0; S(5): 100.0		208.3924881	25.96	3
S(6): 100.0; S(8): 0.0; Y(9): 0.0		99.96647489	11.52	3
S(1): 0.1; T(4): 99.7; S(7): 0.1; S(10):		236.0606858	69.65	3
S(2): 0.0; T(12): 0.0; T(13): 0.0; S(25)		192.8406948	26.01	4
S(5): 100.0		390.3678054	34.86	2
T(1): 0.0; S(2): 0.0; S(4): 0.1; S(7): 99		219.0365705	32.03	3
S(19): 97.8; S(21): 2.1; S(23): 0.0		253.7990321	26.77	6
T(2): 0.0; S(6): 100.0; T(8): 0.0		227.5864531	7.67	2
Y(1): 0.0; T(4): 66.6; T(5): 66.7; T(6):		183.6828673	21.21	3
S(16): 3.3; T(17): 96.7; T(24): 0.0		314.8345322	52.73	4
S(1): 100.0		419.159425	63.64	3
T(2): 0.0; S(7): 0.1; S(9): 99.9		261.9306711	34.63	3
T(12): 0.0; S(14): 100.0; S(17): 0.0; Y		394.6231013	112.48	2
S(2): 0.0; S(5): 100.0		334.5962002	47.38	2
T(1): 0.0; S(5): 0.0; S(11): 100.0; S(1		243.4246261	67.01	3
S(2): 0.0; S(7): 0.0; S(10): 100.0; S(1		361.0818518	104.97	2
S(1): 0.0; S(10): 100.0		282.614596	23.57	3
S(3): 100.0; S(5): 100.0		183.2879589	23.74	3
S(7): 100.0; S(10): 0.0		292.9424693	36.93	2
Y(1): 0.0; S(2): 0.0; T(4): 8.0; T(5): 96		222.5263237	33.19	3
S(10): 100.0		399.0314155	72.08	2
S(10): 0.0; S(11): 100.0		344.7906675	59.5	2
T(2): 0.0; S(12): 50.0; T(14): 50.0		325.7527526	83.37	2
S(2): 0.0; S(4): 0.1; S(7): 99.9		175.4322142	28.77	2
T(10): 1.6; S(11): 1.6; S(12): 96.7		292.3959508	46.47	2
S(6): 0.0; S(16): 0.0; S(17): 0.1; S(21		307.1047771	40.76	4
S(3): 0.0; S(8): 0.0; T(13): 0.0; S(25):		368.1999843	62.11	3
S(2): 0.0; T(4): 0.0; S(6): 0.0; S(8): 2.		381.1350651	96.15	2
S(3): 0.0; T(5): 0.0; S(8): 100.0; T(10)		203.8737524	45.53	2
S(10): 100.0		510.7110879	45.51	3
T(3): 0.0; S(4): 0.0; S(6): 0.0; S(7): 0.		413.5664488	117.95	2
S(4): 100.0; Y(6): 0.0		131.0004419	24.62	3
S(6): 0.6; T(8): 99.4; S(12): 86.6; T(1		170.2885291	8.17	5
S(3): 0.0; S(7): 100.0		167.8692797	31.24	2
T(2): 0.0; T(8): 0.0; S(11): 100.0; S(1		276.0098353	48.48	3
S(3): 0.0; S(4): 1.0; S(5): 49.5; S(6): 4		242.2084857	64.4	2
S(10): 0.0; T(11): 100.0		345.487762	63.28	2
S(6): 1.9; S(7): 48.1; S(13): 48.1; T(1		154.5389015	55.87	3
T(2): 0.0; S(3): 100.0; T(7): 0.0		352.6198252	48.65	2
S(1): 0.0; S(4): 0.0; S(6): 1.9; S(7): 98		341.8514405	91.32	2
S(1): 0.0; S(7): 1.0; S(8): 1.0; S(12): 2		129.1456694	35.03	2
Y(5): 1.8; S(6): 98.2		172.0000487	20.38	2
S(3): 0.0; S(7): 0.0; S(11): 0.0; S(15):		308.827464	50.24	3
S(3): 95.5; S(5): 4.7; S(6): 99.8		79.86731836	9.78	3
S(9): 100.0		235.2142262	47.91	3
T(2): 1.8; S(3): 98.2; T(6): 0.0; S(7): 0		309.8469903	83.7	2
S(1): 0.0; S(3): 98.2; S(5): 1.8		134.5530189	14.63	2
T(3): 0.0; T(5): 0.0; S(14): 100.0		268.8434267	41.11	2
T(4): 0.0; S(6): 0.0; S(7): 0.0; S(10): 1		262.0223147	34.79	3
S(4): 0.0; T(6): 100.0		137.0034818	30.44	2
T(2): 0.0; S(10): 100.0; S(14): 0.0; T(		239.2725513	36.45	3
S(2): 0.0; S(3): 0.0; S(5): 99.9; T(6): 0		384.7183266	97.57	2
S(5): 0.0; T(6): 0.0; S(10): 66.6; S(11		207.3617076	69.87	3
S(1): 0.0; S(3): 100.0		328.6918958	62.08	2
S(3): 100.0		163.5931775	18.06	2

S(5): 100.0; T(12): 0.0		263.0588696	40.4	3
S(4): 2.8; S(7): 97.2		113.9624121	6.82	3
S(1): 0.0; S(2): 0.0; S(3): 100.0; S(6):		321.6868435	70.81	2
S(6): 0.0; S(16): 50.1; S(17): 50.1; S(		209.9287821	21.57	3
S(3): 100.0; T(5): 0.0		163.9473706	29.85	2
S(7): 100.0; S(12): 0.0; S(18): 0.0; S(		291.0926397	31.29	3
S(4): 100.0; S(7): 0.0		484.0164047	70.38	2
T(1): 0.0; S(3): 3.0; S(6): 96.9; S(8): (		218.8536499	24.54	3
S(1): 0.0; T(5): 0.0; S(10): 0.0; S(14):		371.1939264	34.95	3
S(4): 99.9; S(5): 0.1; Y(11): 0.0; T(12		220.0948497	21.1	3
S(10): 0.0; S(12): 0.0; S(15): 0.0; S(1		478.9326636	84.5	3
T(1): 0.0; S(7): 1.6; Y(10): 49.2; S(12		128.1516127	7.02	3
T(3): 0.0; S(18): 97.9; S(20): 2.0; S(2		303.3256536	79.08	2
T(1): 97.8; S(3): 2.2; Y(12): 0.0; S(13		270.031262	37.11	3
S(3): 100.0; T(5): 0.0		164.387218	20.95	2
T(1): 0.0; S(5): 100.0; T(11): 0.0; T(1		346.2053097	29.3	3
S(3): 100.0; S(6): 100.0; T(9): 0.0		427.8365794	77.61	2
S(1): 100.0; S(12): 0.0		308.0750705	58.69	2
S(10): 100.0; S(13): 66.7; T(14): 66.7		235.147736	24.92	3
T(2): 0.0; S(8): 49.5; S(12): 49.5; S(1		270.7264891	62.39	2
S(2): 0.0; S(4): 100.0; S(7): 100.0		153.6300417	21.62	3
Y(1): 100.0; S(5): 0.0; Y(10): 0.0		168.8483145	39.67	3
S(6): 96.7; S(7): 3.3		202.4456163	29.54	3
S(3): 0.0; S(8): 100.0		243.1473356	40.89	3
S(5): 100.0; T(12): 0.0		221.4984738	28.7	3
S(4): 100.0; Y(9): 0.0		367.7273501	45.71	2
S(3): 100.0; T(11): 1.3; S(13): 49.3; S		298.6329012	54.92	2
S(1): 33.3; S(2): 33.3; S(5): 33.3; T(7		225.4872497	36.96	3
S(4): 96.4; S(5): 3.6; S(7): 0.0; T(11):		155.0843505	19.46	2
S(5): 100.0		192.4613222	22.22	2
S(5): 0.0; S(8): 100.0; S(13): 0.0; S(1		469.5688701	74	3
S(3): 1.4; S(5): 49.3; S(7): 49.3; Y(12		213.7191383	24.65	3
T(4): 0.0; S(10): 100.0; T(13): 0.0; S(		410.1916246	96.04	2
S(1): 32.6; S(2): 32.6; S(3): 32.6; S(5		234.0124992	58.39	3
T(10): 0.0; S(22): 100.0		173.932714	31.98	3
T(1): 0.0; T(3): 0.0; S(5): 100.0; S(14		355.5786725	52.42	2
Y(2): 0.0; S(4): 0.9; S(5): 49.6; S(6): 4		285.2732521	77.36	2
S(5): 97.0; S(6): 1.5; S(7): 1.5; T(11):		388.0917419	104.2	2
T(1): 0.0; T(7): 3.9; S(10): 96.1		107.8672668	11.2	2
S(3): 0.0; S(12): 100.0; Y(19): 0.0		258.5061392	68.83	3
Y(1): 0.0; S(2): 0.0; S(4): 100.0; S(6):		184.4959939	30.1	2
S(12): 100.0; T(30): 0.0		365.5549252	86.91	4
S(9): 0.0; S(11): 0.0; S(14): 0.0; S(17		379.772855	88.53	3
S(7): 0.0; T(15): 0.0; S(16): 0.0; S(17		210.2388996	47.19	2
S(15): 100.0; S(17): 0.0		335.1653576	36.45	3
S(4): 100.0; T(5): 0.0; S(9): 0.0; T(16		463.1839804	92.64	2
S(1): 0.0; S(2): 0.0; S(3): 99.9; T(6): (		190.3796681	27.15	2
T(1): 0.0; S(4): 3.0; S(5): 3.0; T(8): 0.		144.4307641	11.81	2
T(4): 0.0; T(7): 0.0; T(12): 4.7; S(14):		158.2784468	27.3	3
T(5): 1.3; S(7): 98.7		309.6439959	70.56	2
Y(3): 0.0; S(12): 98.1; S(13): 1.9; T(1		270.0579077	59.89	3
S(5): 99.6; S(9): 0.4; S(17): 50.0; T(1		128.3467812	6.67	3
Y(3): 0.0; T(14): 0.0; S(17): 0.0; S(18		424.3269751	89.33	3
Y(3): 0.0; T(8): 100.0; S(10): 0.0		267.8297916	54.93	2
T(1): 0.1; Y(5): 3.5; T(7): 3.5; S(11): 8		281.1134553	95.62	3

T(1): 49.4; S(2): 49.4; S(5): 1.1; T(24		473.468419	62.35	3
T(1): 0.0; S(2): 0.0; S(9): 100.0		416.4949398	58.36	2
T(3): 0.0; T(11): 99.9; S(12): 0.1; S(1		255.7792294	46.34	3
T(2): 0.0; S(5): 100.0; S(8): 100.0; T(		251.098421	34.06	2
S(3): 0.0; S(9): 0.1; S(13): 99.9		200.7646415	14.94	3
Y(1): 0.0; S(3): 33.0; S(5): 33.0; S(6):		121.2859686	11.64	2
S(1): 0.0; Y(3): 0.0; Y(6): 0.0; S(8): 1(		282.6875938	28.45	3
S(7): 100.0; S(11): 0.0; S(13): 0.0; S(		258.3169829	50.78	3
T(2): 0.0; S(5): 100.0		202.3243392	28.3	2
T(6): 100.0		280.9756709	49.86	2
S(1): 2.7; S(2): 2.7; S(3): 94.5		95.02409436	11.83	2
S(10): 100.0; S(12): 100.0		337.163913	53.19	2
S(12): 0.0; S(16): 100.0		464.4475451	79.53	3
T(1): 0.0; S(3): 0.0; S(5): 0.0; S(6): 98		219.5379122	23.44	2
S(12): 0.0; S(14): 0.1; S(19): 99.9		176.0205118	52.47	2
S(3): 100.0		91.74827702	3.51	3
S(1): 0.0; S(5): 100.0		228.007778	37.31	2
S(1): 50.0; T(3): 50.0; T(8): 0.0; T(9):		234.748124	30.24	3
S(9): 100.0; S(22): 0.0		320.3109636	48.22	2
T(1): 0.0; T(3): 0.0; S(16): 100.0		244.2012182	29.94	3
Y(4): 0.0; S(6): 98.0; S(7): 2.0; Y(13):		456.510763	49.45	4
T(9): 3.3; S(10): 96.7		172.8188403	19.14	4
S(1): 0.0; S(3): 100.0; Y(5): 0.0; S(8):		231.8844124	37.74	2
S(2): 0.0; S(14): 99.9; S(17): 0.1		212.5289145	27.47	4
T(1): 0.0; T(2): 0.0; T(4): 0.0; S(7): 1(		251.1016798	47.67	2
T(2): 0.0; S(4): 100.0		166.9082898	16.22	2
S(7): 100.0; T(12): 100.0; S(14): 100.		196.0823282	44.74	3
S(8): 100.0		286.8975499	46.83	2
T(1): 0.0; T(4): 1.7; S(6): 98.3		197.9551149	23.34	2
S(4): 100.0; Y(13): 0.0		296.4587928	56.69	2
S(1): 100.0; S(3): 0.0; S(7): 0.0		124.6992262	25.54	2
Y(10): 0.0; S(15): 100.0		271.3323324	62.83	3
S(3): 100.0; S(10): 0.0		103.2640577	15.65	2
S(1): 100.0; S(7): 0.0		254.2595365	41.15	2
S(15): 0.0; S(23): 100.0		234.4015629	18.6	3
S(4): 0.0; T(8): 0.0; S(15): 100.0		109.2460187	11.93	2
S(1): 0.0; S(2): 100.0		195.2276793	30.3	2
S(3): 0.1; S(7): 99.9; T(14): 0.0; Y(15		233.6493838	40.85	3
S(1): 0.0; S(2): 100.0; T(8): 0.0; T(10		283.1789534	85.51	2
S(9): 100.0		175.6770654	26.73	3
T(2): 0.0; T(4): 0.0; S(14): 0.0; S(16):		324.9686689	51.85	3
S(1): 100.0; S(6): 0.0; T(8): 0.0; Y(10		197.3440231	15.17	4
T(3): 0.0; S(7): 0.2; S(9): 4.5; T(20): (		182.7288121	15.85	3
S(9): 0.0; Y(11): 50.0; T(12): 50.0; T(		311.2671416	17.82	4
S(5): 100.0; T(12): 0.0; S(13): 0.0; S(		386.5965003	69.43	3
S(8): 0.0; S(13): 100.0		448.8562973	72.51	2
Y(1): 90.6; T(5): 9.5; T(20): 99.9		60.82464443	5.16	4
T(1): 0.0; S(10): 0.0; S(11): 0.0; Y(12		313.2021018	105.74	2
S(2): 50.0; T(3): 50.0; T(5): 100.0; T(		211.240639	23.42	3
T(10): 50.0; S(11): 50.0; S(20): 0.0; S		232.7340003	30.86	3
S(3): 100.0; T(8): 0.0; S(11): 0.0		165.5378519	10.93	2
T(1): 0.0; T(3): 0.0; T(9): 0.1; S(10): 9		388.7634084	118.74	2
S(1): 100.0; S(2): 0.0; S(3): 0.0; S(6):		204.1323515	65.98	2
T(5): 50.0; S(6): 50.0; T(10): 0.0; T(1		255.6534939	53.74	2
S(1): 0.0; S(12): 100.0; S(17): 0.0; S(		403.6735142	89.38	3

Information Classification: General

S(5): 0.0; S(10): 100.0		327.3844564	65.83	3
S(1): 100.0; S(7): 100.0		214.5070041	24.27	2
S(6): 0.0; S(9): 100.0; S(12): 0.0		300.7276732	73.51	2
S(8): 99.9; S(10): 99.9; S(12): 0.1		227.9588398	35.95	2
T(4): 0.0; S(5): 0.0; T(11): 0.1; S(13):		393.39451	98.26	3
Y(3): 0.0; S(4): 100.0		89.95537687	9.57	3
T(4): 0.0; S(9): 0.0; S(10): 0.0; S(11):		68.66863714	14.61	3
S(1): 0.0; S(7): 0.0; S(11): 0.0; Y(13):		244.5896736	66.72	3
S(7): 100.0; T(24): 0.0		333.3562295	50.33	3
S(3): 100.0		286.1648246	41.34	2
S(7): 50.0; S(8): 50.0; S(13): 93.9; S(		178.5538314	29.89	4
T(2): 0.0; S(17): 100.0; T(19): 0.0		266.9038533	52.32	4
T(2): 0.0; S(14): 99.9; T(16): 0.1		175.7763971	24.08	3
S(1): 0.0; T(8): 0.2; S(12): 4.1; S(13):		102.1377199	3.84	3
S(6): 100.0; T(14): 0.0; T(15): 0.0		91.08157473	13.03	3
S(5): 100.0; T(11): 0.0; S(12): 0.0; T(		255.2486849	21.28	3
S(3): 100.0; T(5): 0.0; T(8): 0.0		157.156085	25.55	2
Y(3): 0.0; S(7): 0.0; S(8): 100.0; S(12		267.9880516	38	2
Y(2): 49.1; S(5): 49.1; S(7): 1.8; T(8):		280.4408193	56.71	3
S(9): 100.0		227.5059581	21.01	2
S(5): 0.0; S(6): 0.0; S(13): 100.0		186.1408996	51.62	2
S(5): 100.0		254.8175228	49.06	2
S(1): 1.9; S(4): 98.1; S(15): 0.0; S(18		522.1349853	73.49	3
S(4): 100.0		192.4534824	63.93	2
T(7): 0.0; S(9): 0.0; S(11): 0.0; S(13):		186.8373029	20.92	4
S(6): 100.0; T(11): 0.0		358.8416234	92.33	2
S(5): 32.9; S(6): 32.9; S(7): 32.9; T(1		157.6452797	28.48	3
S(3): 100.0; S(5): 0.0; S(6): 0.0; S(7):		242.6613784	35.3	2
S(2): 0.0; T(13): 2.6; T(14): 2.6; S(15)		295.9684602	104.46	3
S(7): 0.0; T(11): 0.1; S(14): 99.9; S(1		326.8704882	64.94	5
T(1): 0.0; T(3): 0.0; S(6): 49.3; S(7): 4		223.4667537	37.37	2
S(1): 0.1; T(3): 99.6; S(6): 0.1; S(7): 0		241.7139743	48.45	3
T(1): 5.1; S(3): 94.9; S(9): 0.0; S(13):		247.1099753	19.7	4
T(14): 0.2; S(15): 0.2; T(25): 49.8; S(		158.0314693	8.64	5
T(3): 0.0; T(5): 100.0		280.3425078	46.92	3
S(3): 98.3; S(6): 1.7		199.2787577	19.78	2
S(10): 100.0; T(14): 0.0		251.3893119	19.33	3
S(3): 100.0; S(6): 0.0		204.0306951	17	2
S(4): 93.7; S(6): 3.1; S(7): 3.1; S(12):		293.5409052	54.4	3
S(7): 0.0; S(10): 2.2; S(11): 97.8		176.4904707	59.19	2
S(3): 100.0; S(9): 100.0; T(14): 0.0; S		137.5893931	11.99	3
Y(3): 0.0; T(6): 49.5; S(8): 1.0; S(9): 4		131.8111016	41.34	2
T(3): 0.0; S(7): 100.0		188.488598	36.03	2
T(2): 0.0; T(3): 0.0; T(8): 0.0; S(15): 9		263.121431	44.09	3
S(1): 0.0; T(2): 0.0; S(6): 100.0		155.2036465	25.09	2
S(2): 0.0; S(3): 0.0; S(6): 0.2; T(13): 9		180.8183759	54.29	3
T(2): 0.0; T(6): 0.0; S(8): 100.0		350.0893635	32.84	2
S(13): 100.0		407.9635238	62.38	3
S(2): 0.0; T(4): 5.8; S(5): 94.2; S(11):		157.9743675	26.79	3
S(6): 49.2; T(7): 49.2; S(9): 1.6; S(11		271.6337482	52.24	3
Y(1): 0.0; T(2): 2.4; S(3): 97.6		123.6613719	4.45	2
T(1): 0.0; S(4): 100.0; S(6): 100.0; S(		221.6092754	21.63	3
S(3): 50.0; S(4): 50.0		178.4916282	22.76	2
T(2): 0.0; S(4): 100.0		253.2883853	49.32	2
S(1): 100.0; S(7): 100.0		468.1698929	32.97	2

Information Classification: General

T(2): 0.0; T(5): 0.0; S(9): 100.0; Y(12)		151.7637865	44.89	3
T(2): 0.0; S(7): 0.0; S(9): 100.0		378.9476144	42.82	3
S(1): 50.0; S(2): 50.0		134.0674649	18.25	2
S(6): 0.0; S(10): 100.0		296.256831	60.9	2
S(1): 0.1; S(2): 49.9; S(3): 49.9; S(4):		100.6728242	0.95	3
S(3): 0.0; Y(5): 0.0; S(8): 0.0; S(10): 1		247.5192109	34.76	2
S(1): 0.0; S(4): 99.9; S(5): 100.0; T(1		149.6222637	26.43	2
S(3): 100.0		255.4393651	31.75	3
S(7): 100.0		215.8095069	38.77	2
T(2): 0.0; Y(3): 0.0; S(4): 0.0; T(7): 0.		298.1385866	53.92	3
S(1): 0.0; S(2): 0.0; Y(6): 0.0; S(13): 0		275.7235573	51.87	3
S(1): 0.0; S(3): 100.0		187.7977891	29.84	2
S(3): 0.0; S(9): 2.5; S(11): 97.5		223.8536544	48.03	2
S(6): 98.7; T(8): 1.3; T(20): 1.3; S(22)		87.56274134	4.79	5
S(3): 100.0; S(6): 0.0		160.846732	23.66	2
T(6): 0.0; S(14): 0.0; T(23): 96.2; T(2		247.8067057	49.19	4
S(4): 100.0; S(7): 0.0		259.4725871	38.71	2
S(9): 99.8; S(12): 0.2; T(13): 0.0; S(1		134.1679889	14.25	3
S(6): 0.0; S(11): 100.0; S(13): 0.0		294.7986939	42.54	2
S(6): 100.0; S(11): 0.0; T(15): 0.0		475.5983007	68.62	3
S(7): 0.0; S(8): 99.9; Y(9): 0.0		331.5751741	104.47	2
Y(4): 0.0; S(5): 0.4; T(9): 99.1; S(16):		165.3879478	26.09	3
T(4): 0.0; S(6): 100.0; Y(9): 0.0; S(13		321.8202179	47.66	2
S(7): 100.0; S(10): 0.0		446.8970418	103.07	2
T(1): 99.6; S(4): 0.4; S(10): 0.0; Y(19)		58.11844222	8.81	3
S(5): 100.0; T(12): 0.0		305.1788251	54.95	2
S(3): 0.0; T(4): 0.0; S(11): 100.0; S(1		240.2282231	50.67	3
S(4): 0.0; S(7): 99.9; S(10): 0.1; T(14		247.4755935	35.94	2
S(13): 0.0; Y(14): 0.2; T(16): 99.8		191.5261795	22.12	3
S(3): 50.0; S(6): 50.0; T(10): 0.0; S(1		109.0819459	14.62	3
S(6): 100.0		178.9134488	13.28	3
S(15): 5.2; S(18): 64.9; S(20): 64.9; T		233.0591856	31.07	3
S(2): 0.0; S(4): 100.0; S(8): 0.0		228.4605688	34.74	2
S(3): 0.0; S(5): 96.1; T(7): 1.9; S(8): 1		193.3575205	36.34	2
S(4): 100.0; S(8): 0.2; S(12): 99.8; S(		227.3162803	27.53	3
S(1): 100.0; T(8): 0.0; Y(11): 0.0; T(1		409.3811071	88.28	2
S(3): 4.2; S(5): 95.8; T(10): 0.0; S(14		238.4431091	26.19	3
T(6): 100.0; S(11): 0.0; T(15): 0.0		220.1229798	21.87	3
T(1): 0.0; T(3): 0.0; S(4): 0.0; S(15): 0		166.7535662	15.28	3
S(6): 100.0; T(21): 0.0; S(22): 0.0		219.9368557	37.68	3
S(4): 95.2; S(7): 4.8; T(16): 0.0		77.91288189	12.18	3
S(2): 0.0; T(6): 0.0; S(10): 99.9; T(12)		249.8212895	64.38	2
Y(1): 0.0; S(4): 100.0; T(10): 0.0		274.217095	30.35	3
S(5): 100.0; S(8): 100.0; S(13): 0.0; S		406.9240966	72.44	3
Y(2): 0.0; S(5): 2.3; S(7): 47.7; T(8): 4		159.1397059	26.9	3
T(2): 0.0; S(4): 0.0; S(11): 0.0; S(12):		269.745094	63.32	2
T(2): 8.2; S(4): 88.2; S(6): 94.8; T(10)		107.0898057	10.15	3
S(5): 100.0; S(11): 100.0		262.2314662	42.56	3
S(3): 100.0; T(7): 0.0		169.4164901	18.25	3
S(2): 100.0; T(4): 0.0		182.6292067	22.61	2
T(1): 0.0; S(3): 100.0; T(5): 0.0; S(6):		329.8014473	55.75	2
Y(2): 0.0; Y(4): 100.0; S(7): 0.0		207.9474767	26.51	2
S(6): 0.0; S(11): 50.0; S(14): 50.0		363.42575	88.41	2
T(1): 0.0; T(3): 0.0; T(8): 98.1; S(11):		217.748567	40.18	2
T(1): 0.0; S(13): 100.0; S(19): 0.0		282.3784091	38.15	3

Information Classification: General

S(5): 2.1; S(6): 97.9		296.0946376	36.05	3
S(10): 0.0; T(17): 0.0; S(23): 0.1; S(2		263.1108445	45.41	3
S(4): 0.0; S(7): 100.0		153.0901721	5.8	3
S(5): 100.0		492.0746634	69.21	2
S(1): 0.0; S(2): 0.0; S(4): 0.0; S(5): 0.		236.2232843	50.81	2
S(9): 0.0; S(17): 100.0		477.8846176	68.1	3
S(2): 93.7; S(3): 5.9; S(5): 6.3; S(6): 9		70.04546883	7	3
S(4): 100.0; S(6): 0.0; S(10): 0.0		192.205696	24.2	2
S(1): 0.0; S(4): 100.0; S(7): 0.0; T(8):		147.8387818	22.49	2
T(1): 0.0; S(5): 100.0; T(18): 100.0		108.3533605	5.37	4
S(7): 0.0; S(15): 100.0		310.7443951	38.76	3
S(1): 0.2; S(3): 4.4; S(4): 95.4; S(5): 1		188.8981179	34.88	3
S(1): 0.0; S(3): 100.0; T(10): 0.0		242.7644016	33.34	3
S(1): 0.0; T(2): 0.0; S(3): 100.0; S(7):		297.7462757	35.82	2
Y(1): 0.0; S(9): 0.0; T(16): 33.3; S(17		175.7129127	26.15	3
Y(3): 0.0; S(6): 1.6; S(7): 98.4; T(9): 0		240.7177331	32.55	2
S(3): 95.3; S(4): 2.3; T(8): 2.3; S(15):		284.5166047	47.43	3
S(3): 100.0		142.489574	29.07	2
S(3): 100.0		184.6061301	8.9	3
T(4): 0.0; T(7): 0.4; T(8): 0.4; S(9): 99		175.590584	20.35	3
T(2): 0.0; Y(10): 0.0; T(14): 2.4; S(15)		282.0439703	51.15	3
S(1): 99.9; S(2): 0.1		131.2628869	14.81	2
S(3): 100.0; T(8): 0.0; S(12): 0.0; S(1		310.5996617	82.57	2
S(2): 0.0; S(5): 100.0; S(10): 0.0; S(1		396.8287542	116.39	2
T(1): 0.0; S(5): 100.0; S(11): 100.0; S		351.6915379	91.4	3
T(4): 0.0; S(8): 0.0; T(16): 0.0; S(19):		357.5468681	60.72	3
T(2): 0.2; S(3): 0.2; S(4): 0.2; T(14): 0		112.1702112	3.81	3
S(7): 0.0; S(14): 0.0; S(20): 99.9; S(2		454.7597038	116.38	3
S(3): 100.0; S(8): 0.0; S(11): 0.0		261.4742643	27.17	3
S(3): 0.0; T(9): 100.0		318.2823054	61.31	3
S(7): 100.0; S(13): 100.0		168.888693	15.47	4
S(1): 0.0; S(12): 100.0; S(17): 0.0; S(		377.1078321	80.81	3
S(10): 0.0; T(12): 0.0; S(19): 4.0; T(2		183.7356839	22.42	3
S(4): 100.0		264.8212774	29.29	3
S(1): 59.7; S(4): 59.7; S(6): 40.3; S(7		250.928626	27.53	3
S(1): 0.0; S(2): 99.9; T(3): 0.2; S(5): 9		258.7215237	75.93	2
S(3): 4.4; S(6): 95.6; S(11): 100.0; Y(		135.7061382	25.67	3
S(1): 100.0; S(9): 0.0		149.1079162	35.6	2
T(4): 0.0; T(10): 0.0; S(14): 0.1; S(18)		258.5845077	34.14	3
T(5): 2.5; S(7): 97.4; S(11): 0.1; T(12)		225.0125827	45.76	2
T(1): 0.0; S(5): 0.0; S(7): 0.0; S(10): 1		286.9619204	58.81	2
S(3): 50.0; S(4): 50.0; T(12): 0.0; S(1		346.5937244	52.22	3
S(1): 0.0; S(5): 100.0		170.6177353	23.07	5
S(5): 100.0; Y(11): 0.0		293.04543	33.47	2
S(14): 100.0		423.4611953	42.95	4
S(2): 0.0; S(4): 100.0; T(9): 0.0		249.0578673	33.36	2
S(4): 50.0; S(5): 50.0; S(7): 0.0; S(9):		254.3226976	15.51	2
T(1): 0.0; S(5): 97.3; T(8): 1.3; S(9): 1		219.4107164	34.96	4
T(8): 0.0; S(10): 3.3; S(11): 96.7; Y(1		160.8825597	34.84	2
S(3): 100.0; T(5): 0.0; S(7): 0.0		114.5310664	12.52	3
S(4): 0.0; S(5): 0.0; T(9): 0.0; S(11): 2		264.5143241	13.49	3
S(4): 100.0; T(8): 100.0; S(16): 0.0		432.2543911	27.37	3
S(1): 0.0; T(9): 50.0; S(10): 50.0		125.1046935	7.34	3
S(10): 100.0		266.3373081	32.63	2
S(4): 100.0		336.988721	34.12	3

Information Classification: General

T(2): 4.9; S(4): 95.1; S(7): 100.0; S(1	222.5440724	33.73	3
T(2): 1.9; S(4): 98.1; S(6): 0.0; T(10):	354.3246886	86.4	2
Y(7): 0.0; S(10): 100.0; T(12): 0.0	242.0190726	20.45	3
S(1): 100.0; T(10): 0.0; S(12): 0.0	200.1063247	47.59	2
S(8): 100.0	372.3506036	52.53	2
S(5): 0.0; S(7): 100.0; S(10): 100.0; Y	327.1254034	54.85	3
S(6): 0.3; T(11): 4.6; S(14): 90.5; S(2	172.5145642	35.5	3
S(8): 100.0	349.5567317	63.12	2
S(1): 0.0; S(3): 0.0; S(5): 0.0; S(6): 10	284.7678111	45	2
S(1): 2.1; S(2): 97.9; T(6): 0.0; S(13):	213.940454	42.4	2
T(3): 50.0; S(4): 50.0; S(7): 0.0	146.3194093	11.68	2
S(2): 100.0	160.7920699	21.59	2
T(3): 0.0; T(6): 100.0; S(9): 0.0; S(16)	341.3392682	40.89	3
S(7): 0.0; T(9): 100.0; T(11): 0.0	200.3248698	36.28	2
S(4): 100.0; S(7): 0.0	214.9526005	41.92	2
S(1): 0.0; S(12): 100.0	283.8120474	27.95	3
T(2): 0.0; T(4): 2.7; S(7): 97.3	107.968575	22.09	2
S(2): 0.0; T(6): 33.5; S(8): 33.5; S(9):	99.21507937	7.4	3
T(1): 0.0; S(8): 100.0; T(12): 0.0	478.0084046	115.96	2
T(1): 0.0; T(4): 0.0; Y(9): 0.0; S(12): 0	381.4466327	103.03	3
S(2): 0.0; S(7): 100.0; S(11): 0.0	394.3536622	75.6	2
Y(1): 0.0; T(2): 0.0; S(5): 0.0; S(7): 10	297.3566707	70.06	2
S(2): 0.0; S(5): 50.0; S(6): 50.0; T(15	294.263466	32.77	3
S(3): 0.0; S(10): 100.0	169.8493638	22.06	4
S(1): 100.0; S(6): 0.0; T(10): 0.0	360.9245193	82.61	2
T(5): 0.0; S(8): 2.1; T(11): 97.9	171.9312618	32.78	2
S(1): 0.0; Y(3): 0.0; S(4): 0.0; S(5): 0.	310.8578522	67.53	2
S(13): 1.8; S(14): 96.3; T(16): 1.8	283.3434399	47.34	2
S(3): 100.0; S(6): 0.0; T(19): 0.0	307.1646802	55.88	3
S(3): 100.0; S(13): 0.0	174.6774365	46.36	3
S(1): 0.0; Y(3): 0.0; S(4): 2.6; S(5): 97	305.3588166	42.6	2
T(5): 0.0; S(10): 0.0; S(11): 0.0; T(13)	257.6035286	28.06	3
S(5): 100.0	349.685818	56.34	2
S(1): 0.0; T(2): 0.0; S(6): 100.0	232.7514028	31.28	2
T(3): 0.0; S(6): 100.0; T(8): 0.0	319.4238945	71.72	2
S(1): 0.0; S(3): 0.0; S(4): 0.0; T(6): 0.	246.8576126	36.7	3
S(6): 0.0; S(7): 100.0; S(9): 100.0; S(	268.1500335	33.54	3
S(14): 0.0; S(17): 0.1; T(18): 99.9	369.3192482	45.82	4
S(7): 100.0; S(10): 100.0; S(11): 100.	237.7388177	32.96	3
S(1): 0.0; Y(2): 0.1; S(3): 99.9; S(9): 1	280.3288304	12.96	3
T(2): 0.0; T(3): 0.0; T(8): 0.0; S(15): 0	275.6514185	49.22	3
S(9): 0.0; S(19): 99.9; S(22): 0.1	245.53472	21.12	4
S(1): 0.0; S(4): 0.0; S(11): 100.0	375.4780996	47.66	3
S(8): 99.8; T(10): 0.2; S(12): 0.0; S(1	226.2314561	59.42	3
T(1): 0.0; S(3): 100.0	126.3715261	18.69	2
T(4): 2.6; S(5): 97.4; S(8): 100.0; T(1	342.9554645	109.69	2
S(10): 100.0	368.5015486	43.82	3
S(1): 0.1; T(4): 99.9; Y(24): 0.0; S(25	334.0295578	38.17	3
S(9): 100.0; S(16): 0.0; T(18): 0.0; S(	255.5295203	49.99	3
Y(13): 0.1; S(16): 99.9	257.9449564	25.38	3
S(1): 0.0; S(2): 0.0; S(3): 100.0; T(8):	213.3156736	29.66	2
S(7): 100.0; Y(8): 0.0	177.2812565	11.64	2
T(11): 2.3; S(14): 48.9; S(17): 48.9; T	301.5832517	46	4
T(4): 1.0; T(7): 33.0; S(8): 33.0; S(10)	182.2027291	23.3	2
S(2): 0.0; S(4): 0.0; T(7): 0.1; S(9): 99	139.0400432	19.31	2

Information Classification: General

S(2): 50.0; S(3): 50.0		360.5898951	65.14	2
S(2): 0.0; S(12): 48.6; S(17): 48.6; T(		118.5982337	22.08	4
T(1): 0.0; S(3): 0.0; T(6): 2.1; S(7): 97		280.5027995	60.52	2
T(1): 0.0; S(3): 0.0; S(6): 50.0; S(8): 5		147.6376187	20.57	3
S(3): 96.8; S(4): 3.2; T(17): 0.0		336.6929164	64.72	4
T(5): 1.4; S(7): 98.6; S(9): 0.0		370.3842069	57.85	2
S(19): 94.1; S(20): 94.1; S(23): 10.8;		231.4109011	10.53	4
T(1): 0.0; T(3): 100.0; T(7): 0.0; Y(10)		342.1289723	68.7	2
S(5): 100.0; S(7): 0.0		304.8429141	79.85	2
Y(3): 50.0; S(5): 50.0		142.4779005	19.87	2
T(1): 0.0; T(9): 0.0; S(22): 50.0; S(23)		155.1891294	61.66	3
S(1): 2.2; T(4): 97.8		278.5720467	34.52	3
Y(2): 0.1; Y(3): 2.4; S(4): 97.6		112.2788482	21.38	2
S(4): 0.0; Y(5): 0.0; T(7): 0.0; S(11): 1		179.8244256	16.41	3
T(4): 0.0; T(8): 100.0		260.4667816	33.82	2
T(2): 0.0; S(4): 0.0; S(5): 0.0; S(6): 0.		337.3145398	35.7	3
S(9): 0.0; Y(17): 0.0; S(24): 100.0		358.9887468	86.15	3
T(1): 0.0; S(6): 0.0; S(10): 100.0; S(1		236.624443	30.22	3
S(3): 100.0		161.1702508	22.89	2
T(1): 0.0; S(6): 100.0; S(8): 0.0; S(10		308.1085293	32.28	3
S(4): 100.0; S(7): 100.0		467.1320399	73.41	2
S(2): 0.0; S(9): 100.0; T(13): 0.0		262.4421679	46.32	2
S(7): 0.0; S(10): 0.0; S(13): 100.0		241.7563648	44.68	2
T(3): 0.0; S(7): 100.0; S(11): 0.0		191.1258785	32.77	2
S(9): 100.0; S(12): 100.0; T(13): 0.0;		321.6606874	59.09	2
S(3): 100.0		176.3664884	22.27	2
S(3): 0.0; S(4): 0.0; T(5): 0.0; S(24): 1		414.4520066	93.83	3
S(9): 100.0		229.169328	16.52	2
S(9): 100.0; S(19): 0.0; S(20): 0.0; S(		205.3062492	35.43	4
S(2): 0.0; S(5): 100.0		484.6142154	66.79	2
S(5): 100.0		308.999608	43.01	2
S(8): 0.0; S(10): 0.0; S(13): 100.0		178.41687	35.84	2
S(1): 0.0; Y(4): 0.0; S(11): 0.0; S(13):		312.8931185	63.99	2
S(8): 0.0; S(9): 50.0; S(10): 50.0; S(1		221.011963	18.79	3
T(3): 0.0; S(8): 100.0; T(9): 0.0		288.7967023	55.59	2
T(2): 0.8; S(3): 33.1; Y(4): 33.1; S(6):		111.0250089	12.09	2
S(7): 99.8; T(9): 0.2; S(12): 0.0; S(14		101.5112213	8.75	2
T(1): 0.0; T(2): 0.0; S(4): 100.0; T(7):		296.62703	34.04	2
S(1): 0.0; S(7): 100.0		429.1878567	88.87	2
S(1): 100.0; T(3): 0.0; S(5): 100.0		179.4937725	14.95	3
S(14): 100.0		378.540658	52.93	4
S(1): 100.0; T(3): 100.0		119.8279119	12.54	3
S(14): 49.0; S(18): 49.0; Y(22): 2.0		179.7367172	15.63	4
S(1): 0.0; T(11): 0.0; S(16): 100.0		218.2100908	32.33	3
T(3): 100.0; S(5): 100.0		87.60059857	1.27	2
T(4): 0.0; S(9): 0.0; S(10): 0.0; S(13):		361.1683051	44.6	3
S(10): 100.0; S(20): 0.0; T(21): 0.0; S		240.7757224	45.33	4
S(2): 0.0; T(13): 99.9; S(20): 0.1		169.244925	34.29	2
S(1): 0.0; S(4): 100.0; S(5): 100.0; T(		231.6454507	43.29	2
S(1): 0.1; S(3): 99.9		97.97447545	10.91	2
S(1): 0.0; S(4): 0.0; S(5): 98.4; T(7): 1		282.5263383	58.75	2
S(5): 0.0; T(6): 0.0; S(10): 0.0; S(11):		397.7429036	134.97	2
Y(1): 0.0; S(4): 100.0; Y(8): 0.0		210.0006864	31.34	2
S(2): 100.0; T(6): 0.0; Y(7): 0.0; S(8):		127.8562786	9.89	2
S(2): 0.0; T(4): 100.0; S(17): 0.0		434.3204964	96.55	2

Information Classification: General

T(5): 0.0; S(6): 0.0; S(14): 100.0; T(2(	232.9798821	31.38	4
S(7): 1.8; S(8): 98.2; S(14): 0.0	287.2341273	52.5	2
S(6): 100.0	296.523394	87.83	2
S(2): 0.0; T(11): 0.4; S(17): 99.6	153.1549848	42.69	5
S(11): 100.0; T(13): 0.0; S(17): 0.0; T	306.4715918	84.07	3
S(3): 100.0; S(7): 100.0; S(11): 0.0; T	252.1148641	35.68	3
T(1): 0.0; Y(9): 0.0; S(11): 100.0; S(1	379.0918804	56.18	3
S(1): 100.0; T(4): 0.0; S(7): 100.0	239.9429841	30.54	2
S(1): 0.0; S(5): 0.0; S(7): 100.0; Y(17	288.2300896	45.83	2
T(6): 0.2; S(9): 0.2; T(10): 0.2; S(13):	122.1464973	23.26	2
Y(3): 95.2; S(6): 4.5; S(8): 0.2	96.91740793	5.27	2
T(7): 0.0; T(10): 0.1; S(12): 99.9; T(1	297.2965895	81.22	3
S(3): 0.0; S(7): 0.0; S(8): 0.0; S(16): 1	200.8867296	27.77	3
S(5): 50.0; S(6): 50.0	135.6549748	13.62	3
S(3): 0.0; S(4): 100.0	176.2892675	37.75	2
S(11): 100.0; S(17): 0.0; T(21): 0.0; T	173.9336013	15.7	4
T(1): 0.0; S(4): 0.0; T(11): 0.0; T(16):	541.8260121	144.88	2
S(6): 0.0; S(10): 0.1; S(11): 2.6; S(14	270.3609266	71.25	3
S(3): 50.0; S(5): 50.0	145.4717673	18.97	2
T(3): 0.0; T(6): 0.0; S(9): 0.0; S(16): 1	376.5908362	62.08	2
S(2): 4.9; S(5): 99.8; S(7): 95.3; T(11	183.3002584	27.62	3
S(1): 0.0; S(3): 0.0; S(5): 0.0; T(7): 0.	315.8612773	50.69	2
S(8): 100.0; S(19): 0.0	279.2323983	45.09	3
T(7): 100.0	179.4023044	17.07	2
S(8): 100.0; S(10): 100.0	331.0555845	81.96	2
S(4): 0.0; S(5): 0.0; T(7): 0.0; S(10): (	285.5225014	30.24	3
T(2): 0.0; S(7): 100.0	225.3372665	26.19	4
S(1): 0.0; S(4): 100.0; S(7): 100.0	275.7563837	29.92	2
S(1): 0.0; S(3): 0.0; T(5): 0.0; S(13): (	251.0514956	34.62	3
T(3): 0.0; S(4): 0.0; S(5): 0.0; S(7): 10	223.0511199	43.64	2
T(4): 100.0; S(12): 0.0	261.5271106	56.34	3
S(5): 100.0; Y(12): 0.0; S(15): 0.0	277.877771	70.22	2
T(9): 100.0; T(12): 0.0; S(18): 0.0	264.0851066	54.96	3
S(3): 100.0; T(15): 0.0	258.9709773	31.86	3
S(3): 100.0; S(5): 0.0; S(10): 100.0	236.8910395	28.71	3
S(1): 1.7; S(3): 98.3	92.96736449	10.35	2
S(3): 0.0; T(4): 0.0; S(14): 48.8; T(16)	188.7282544	44.08	3
S(3): 100.0	84.1246918	3.91	4
Y(3): 0.0; S(7): 0.0; S(11): 100.0; T(1	311.2118929	97.48	3
T(5): 0.0; S(14): 100.0; S(19): 0.0	125.4095634	23.77	3
S(3): 100.0	304.8916999	73.53	2
S(5): 100.0; S(11): 0.0	277.0974945	64.95	3
S(5): 100.0; S(11): 0.0	124.0344577	18.15	2
S(4): 100.0; S(11): 97.7; S(13): 2.3; S	269.2427179	16.87	3
T(7): 0.0; S(9): 1.4; S(10): 98.5; T(12)	317.0238996	64.04	2
S(4): 100.0; S(7): 0.0	229.4197793	24.31	2
S(3): 50.0; S(5): 50.0	218.5749342	39.6	2
S(1): 0.0; S(3): 100.0; T(10): 0.0	290.0236582	30.37	2
S(4): 100.0; T(9): 0.0; S(13): 0.0	254.9023815	63.28	2
S(7): 97.4; S(8): 2.6	119.6152248	13.64	2
S(1): 0.0; S(5): 100.0	292.8979072	38.48	2
S(1): 0.0; S(3): 0.0; S(7): 100.0; T(13	242.9120909	34.34	3
S(5): 49.5; T(7): 1.0; S(9): 49.5; T(12)	353.4708539	99.05	2
S(2): 0.0; S(8): 96.8; S(10): 3.2; S(18	249.57621	21.79	3
T(2): 0.0; S(5): 100.0; S(9): 0.0	584.7917493	83.65	2

Information Classification: General

S(3): 0.4; S(8): 99.6; S(12): 100.0; S(		109.2737872	17.58	3
S(2): 100.0; S(9): 0.0; S(13): 0.0		126.1204186	37.14	3
S(5): 0.0; S(6): 0.0; S(8): 0.0; S(10): (		394.0645129	105.29	2
S(6): 100.0		229.2097971	23.24	3
T(1): 0.0; S(6): 100.0; T(13): 0.0		302.2337822	14.85	3
S(8): 100.0; S(13): 0.0; T(17): 0.0; S(		455.5826017	82.29	3
S(1): 0.0; S(3): 0.0; T(4): 0.0; S(6): 0.		253.3763071	66.93	2
S(4): 100.0; S(8): 0.0		214.1876805	22.09	2
S(5): 100.0		207.6031135	10.18	2
S(1): 0.0; S(3): 100.0; T(4): 0.0; T(8):		342.0942768	74.77	2
S(5): 99.9; T(7): 0.1; S(8): 0.0; Y(11):		160.9809899	33.51	3
S(4): 99.9; S(6): 0.1; S(8): 0.0; S(12):		150.8335034	35.21	2
S(1): 0.0; S(3): 100.0		312.2566127	41.98	2

greater than 75% indicates that the phosphorylation is more reliable; phosphoRS Binomial Peptide Score,

/s NS)

MH+ [Da]	ΔM [ppm]	NC/NS	ce A
1052.55164	-1.5380937	1.64807549	2.79E-09
2653.33921	2.5206787	1.52605062	4.99E-07
3532.68691	-2.51959651	1.45248742	9.09E-06
1275.70703	0.43629513	1.44122451	1.39E-05
2000.05863	0.38828979	1.43995139	1.46E-05
3360.5112	6.58152424	1.43223127	1.95E-05
3239.66196	2.05951175	1.42095753	2.97E-05
2561.34795	14.3528681	1.4119737	4.12E-05
2330.97759	5.8706533	1.40342912	5.62E-05
2329.06107	1.36278462	1.39541154	7.50E-05
4921.38491	0.52568561	1.39345023	8.05E-05
2662.3696	2.6881029	1.36962659	0.000186
2698.36049	-1.4145483	1.36506077	0.000218
2927.4456	-2.5428519	1.36443562	0.000222
2561.31058	-0.23592323	1.36348636	0.00023
1899.99991	-9.73485201	1.36057992	0.000254
1218.56767	-0.00882029	1.35627486	0.000294
2169.01567	-0.05334274	1.35403858	0.000317
1257.68437	-0.24213498	1.3492183	0.000373
1727.86223	0.81714613	1.34448068	0.000437
2589.37406	-1.85670747	1.3408773	0.000493
1631.86649	1.58954822	1.3403187	0.000502
1005.48912	0.41472928	1.3298996	0.000708
4244.85708	1.0572959	1.32272855	0.000894
3368.60137	-7.03061577	1.321676	0.000925
2896.35367	3.15622631	1.32003745	0.000975
3920.82309	-2.22582489	1.31591603	0.001112
2953.43991	-1.7534728	1.31499592	0.001145
1119.6032	-1.93040462	1.31329846	0.001209
3084.4105	5.28213678	1.30989802	0.001347
3256.57907	1.144262	1.30855352	0.001405
2596.23026	7.73061698	1.30803601	0.001428
1464.6364	-2.89229613	1.3075179	0.001452
1846.90354	-0.6393023	1.30616599	0.001515
3529.69626	-3.04618854	1.30565441	0.001539
1442.67668	-2.15014154	1.30407761	0.001618
1444.81336	-0.22367806	1.30254107	0.001697
1659.72397	-1.32200258	1.302299	0.00171
1920.76872	-2.2183532	1.30131513	0.001763
4228.85447	-0.75859676	1.30048759	0.001809
3760.5831	1.38135493	1.29698064	0.002017
1203.57547	-1.18205677	1.29593884	0.002083
2467.02848	-2.63059989	1.29543605	0.002116
2770.34104	0.63467649	1.2923744	0.002325
1425.78545	-0.90708643	1.28930583	0.002554
4245.84885	-2.96082836	1.28695969	0.002743
1243.63005	1.14518104	1.28670467	0.002764
1351.61699	-2.7902445	1.28178481	0.003207
2858.28	1.35540047	1.27985018	0.003399
1696.65813	0.73282497	1.27941472	0.003443
2656.22144	0.45459553	1.2760039	0.003812

1226.61948	-0.23262868	1.27519925	0.003905
1352.68389	1.83870703	1.27241451	0.00424
1879.86675	-0.5486998	1.2722775	0.004258
1883.92473	0.26642038	1.27168362	0.004333
2633.329	-3.08776529	1.27162789	0.00434
3120.48557	1.90980544	1.27128664	0.004384
3461.69858	-1.86318486	1.26708933	0.004959
2703.25248	0.50800234	1.26617583	0.005093
3151.54726	-2.38180884	1.26354377	0.005498
2337.02982	0.26529768	1.26260131	0.005651
2951.33164	-0.30296282	1.26129837	0.005868
1423.70842	0.81008314	1.26075681	0.00596
2177.99723	-0.60549427	1.25956005	0.006169
1856.8967	0.81982633	1.25825049	0.006406
1516.64507	3.48916837	1.25776053	0.006497
2671.19638	-2.52285831	1.25751301	0.006543
2964.3693	0.55612088	1.25573772	0.006884
2072.0374	5.40135732	1.25415053	0.007203
1770.86968	-4.09335317	1.25292273	0.007458
1399.75542	-0.30191524	1.25133661	0.007801
2519.30034	-0.10905111	1.25106757	0.007861
4431.09878	1.25912083	1.25058887	0.007968
3598.64682	7.28618406	1.24979813	0.008148
4183.79213	2.45797674	1.24907968	0.008314
1542.81533	0.5574375	1.24893415	0.008348
2306.94072	4.08945353	1.24872834	0.008397
1153.58916	-0.47622945	1.24853306	0.008443
3132.43252	-3.45082514	1.24811228	0.008544
2143.01456	0.98516341	1.24718815	0.008768
2327.11814	-2.24789633	1.24692964	0.008832
1556.70378	-2.9776697	1.24554293	0.009181
3609.68479	3.16988075	1.24376152	0.009649
3599.61399	2.53651894	1.24223501	0.010067
3273.53171	-3.66909583	1.24195722	0.010145
2901.41947	3.57047514	1.24120975	0.010357
3490.6	7.78576517	1.24104013	0.010406
1438.67204	1.05400607	1.24091172	0.010443
1981.02434	-0.57329385	1.24082839	0.010467
2993.45707	1.34396527	1.23949372	0.01086
3041.44645	0.73089735	1.23905336	0.010992
1725.9113	-2.0735819	1.23859637	0.011131
3451.71311	3.30055901	1.23560556	0.012082
4682.74985	10.5219686	1.23293897	0.012993
4663.26063	1.98274929	1.23277549	0.01305
4061.83481	-3.24393193	1.23256549	0.013125
1557.77922	-0.78053202	1.2320092	0.013324
1461.68413	-1.00956833	1.23190226	0.013363
1232.64165	5.15629279	1.23186002	0.013378
2746.30039	1.43460499	1.2313858	0.013551
1283.62566	-0.73802645	1.23123936	0.013605
3390.59763	0.41086184	1.23081942	0.01376
2286.11797	3.24971009	1.2299356	0.014092
1975.85039	-1.07240122	1.22792723	0.014875
1567.70928	-0.8816594	1.22745945	0.015063
2847.26401	0.69607283	1.22718234	0.015175

Information Classification: General

1134.48291	0.22696773	1.22694533	0.015271
3203.53269	2.82490045	1.22670641	0.015369
2609.04819	-1.3059971	1.2266883	0.015377
1680.80271	1.49225854	1.22605399	0.01564
1911.99314	-1.67266307	1.22588786	0.015709
2535.23427	-2.84063073	1.22484175	0.016154
2030.97661	0.4671638	1.22448486	0.016308
2813.43291	0.12107162	1.22417901	0.016441
1381.70476	1.15380135	1.22413291	0.016461
1394.72197	0.61168239	1.22348005	0.01675
1229.54729	-0.00527862	1.22330941	0.016826
2086.91068	-0.50678566	1.22225997	0.0173
2206.07964	0.5344968	1.22125467	0.017766
1518.79044	-1.03956028	1.22072853	0.018015
1227.61077	0.38291631	1.21997593	0.018375
1483.79827	-1.96315623	1.21897897	0.018863
2738.09726	0.17926911	1.21894528	0.01888
3931.85886	-11.3893875	1.2183876	0.019158
3496.75302	0.72226291	1.21758968	0.019563
2477.25932	2.28463869	1.2174258	0.019647
3090.45151	0.22744351	1.21739417	0.019663
2621.29593	-3.59467622	1.21695812	0.019889
4346.95034	-2.7907407	1.21673602	0.020004
2300.01688	-2.83191162	1.21672418	0.020011
3407.59092	-0.24850401	1.21648174	0.020138
2647.09722	3.26103154	1.21623741	0.020267
1261.67656	-2.40713783	1.2159822	0.020402
1683.85686	0.6596892	1.21571487	0.020544
3612.66465	0.39158596	1.21536378	0.020733
3205.53733	0.64523748	1.21472017	0.021083
1906.9583	-3.65647646	1.21449486	0.021207
2009.01908	0.24480658	1.21437468	0.021273
2879.46384	1.56939102	1.21367397	0.021663
3057.32022	-5.98259804	1.21323341	0.021912
933.411058	1.06388384	1.21320448	0.021928
2641.34964	0.7061645	1.21253536	0.022311
1804.83684	1.13267354	1.2118818	0.02269
3556.57358	5.58464915	1.21160552	0.022853
3242.62952	3.12664799	1.21132996	0.023016
1757.96767	0.58805013	1.21121623	0.023083
2057.13896	0.56841779	1.21110154	0.023151
3859.91128	0.24204928	1.21098731	0.02322
3822.03051	8.16976504	1.21077109	0.023349
1487.71477	-5.73968999	1.21019699	0.023696
2530.16728	0.36188393	1.20919222	0.024315
2007.85808	-4.23111313	1.20870761	0.024618
2428.12798	-1.47356885	1.20849738	0.024751
4148.89597	1.11521498	1.20846332	0.024773
2797.413	2.75959236	1.20833591	0.024853
3729.77304	1.52371369	1.20809772	0.025005
4723.24415	2.29494479	1.20749857	0.025391
2707.37198	2.30633048	1.20621741	0.026232
3042.47768	-3.43452355	1.20586848	0.026466
1836.89775	1.35629589	1.20583688	0.026487
2004.01371	1.04676297	1.20574834	0.026547

Information Classification: General

2453.24375	-2.28748927	1.20490157	0.027123
1669.89141	1.64305332	1.20314805	0.028352
2172.87925	-0.34963833	1.20257757	0.028763
1655.8123	0.02304496	1.20185722	0.029289
1707.90117	-1.84475216	1.20117296	0.029796
3319.61386	-2.33598893	1.2007616	0.030105
1067.53765	0.8523868	1.20060565	0.030223
2429.14084	0.00698099	0.49555375	4.89E-14
3115.29087	1.62019281	0.50743991	3.32E-13
2348.10294	3.74690804	0.53105813	1.10E-11
1558.66301	-0.79801346	0.5415949	4.62E-11
2212.03891	1.90107274	0.59066531	1.57E-08
3166.45273	2.20865645	0.59606361	2.76E-08
2045.87993	5.70175751	0.61630764	2.02E-07
1713.84661	2.1646721	0.63311647	9.13E-07
4861.98081	2.8895174	0.65104536	4.02E-06
1677.7238	1.82043168	0.65402519	5.08E-06
2836.2311	2.87512097	0.65781407	6.81E-06
2414.0156	1.39013898	0.67097056	1.81E-05
1908.7863	-0.66367746	0.67929135	3.25E-05
2086.97275	-1.06559249	0.68083524	3.61E-05
1469.72588	1.28553011	0.68519785	4.86E-05
2648.28312	-4.7125222	0.69103309	7.14E-05
2221.99419	1.09312786	0.69168188	7.45E-05
1374.70703	3.56918575	0.69214207	7.68E-05
2101.95439	-0.74026255	0.6929043	8.06E-05
2121.98087	0.16061091	0.69516634	9.32E-05
2049.9561	3.06981513	0.69545146	9.49E-05
2712.32876	0.85931969	0.695713	9.65E-05
3137.46933	-1.45717161	0.6963718	0.000101
1399.65434	0.86533776	0.69793701	0.000111
2382.25156	1.55765718	0.70336103	0.000156
2037.87859	0.76468629	0.71057869	0.00024
1941.85405	-1.68076226	0.72084989	0.000433
2798.31058	0.40729122	0.73049732	0.000735
1796.78276	-0.63995355	0.73096302	0.000753
3013.10025	3.02697492	0.73101977	0.000755
2335.94364	1.86321617	0.73191001	0.000792
1298.7116	0.29040344	0.73329124	0.000852
3446.41953	-3.80438848	0.73515563	0.000939
2412.01299	-1.26419225	0.73999323	0.001204
1031.54106	0.30820271	0.74141311	0.001294
4022.83481	2.48150492	0.74243167	0.001362
2369.10111	-0.41278479	0.74263438	0.001375
2509.10796	0.3194183	0.74278051	0.001386
1258.60295	-0.1552736	0.74386297	0.001462
2246.98386	2.82980935	0.74577258	0.001607
1971.86846	13.7182081	0.74691273	0.0017
3530.66196	-0.34947501	0.74756765	0.001755
1394.7011	-1.49917181	0.74810174	0.001801
2706.99443	1.31402722	0.74828105	0.001817
1682.64311	0.6184942	0.75044128	0.002017
1887.76389	0.6112167	0.75120023	0.002092
1241.56645	1.64997109	0.75183039	0.002156
4065.60019	-0.57124561	0.75242154	0.002217

Information Classification: General

2014.9861	0.67166236	0.75497811	0.002502
2364.2582	1.22115126	0.75593598	0.002617
1866.9404	3.90344795	0.75687804	0.002734
1685.68279	-1.0657067	0.75711848	0.002765
1746.86687	0.13200079	0.75920297	0.003044
1887.90972	1.88264697	0.7612303	0.003338
2336.05863	-1.10116999	0.76158398	0.003392
1665.82707	3.19280796	0.76287208	0.003595
1869.79753	-1.00296939	0.76334396	0.003672
2632.25333	-0.19612444	0.76400801	0.003783
1226.59294	2.49292692	0.76407113	0.003794
1149.61528	0.25806351	0.76441961	0.003853
1931.93828	2.76805977	0.76504896	0.003963
1771.69109	1.21484938	0.76561727	0.004064
1472.71506	-0.23649666	0.76570382	0.00408
1757.85093	3.42291007	0.76615197	0.004161
3070.41176	1.34593348	0.76654184	0.004234
1263.63884	0.88098278	0.76770199	0.004455
2555.01177	0.53661152	0.76823441	0.00456
1402.67301	-0.37892997	0.76851901	0.004617
2781.35923	0.51800101	0.77079782	0.005096
1167.59964	-0.64386167	0.7711451	0.005173
3504.79135	-0.51082838	0.77216924	0.005405
3088.46665	3.04572558	0.77359465	0.005744
1361.59087	3.90911204	0.77363676	0.005754
1582.79802	1.35578358	0.77501544	0.006099
1568.66643	1.19218124	0.7757366	0.006287
1828.78349	-0.32996559	0.77610928	0.006386
2172.99614	2.34701234	0.77686183	0.00659
2125.97403	-0.23618549	0.77706352	0.006645
1389.65019	0.02574974	0.77716724	0.006674
1788.79949	-0.52114544	0.77893373	0.00718
1817.77384	0.73857553	0.77929758	0.007289
1642.79997	-2.47987795	0.77937111	0.007311
1226.60332	1.00340763	0.77996106	0.00749
2302.28171	0.08387264	0.78038874	0.007622
1987.81108	-0.05158689	0.78108408	0.007841
1846.91507	2.08721825	0.7812836	0.007905
1863.84685	-1.34128158	0.78145835	0.007962
1362.58728	1.31999485	0.78187224	0.008097
2905.30259	2.32819876	0.78188841	0.008102
1230.67754	0.30839364	0.78437793	0.008956
1884.97087	1.50214208	0.78450067	0.009
1668.83257	0.98801404	0.7847686	0.009097
3792.66721	1.0837084	0.78496764	0.009169
1719.75212	2.80069087	0.78548519	0.00936
1673.76177	2.57566449	0.78576961	0.009466
1525.80083	1.97309419	0.78588103	0.009508
3038.4805	-1.18559183	0.78597145	0.009543
2392.04551	-2.62660398	0.7860158	0.009559
2063.76982	-0.74296591	0.78635191	0.009687
3902.63486	1.88398678	0.78641224	0.00971
2979.527	0.52858893	0.7879524	0.010317
4145.57846	2.32006901	0.78847153	0.010529
2029.83745	-2.31858837	0.78893183	0.01072

Information Classification: General

1179.55423	0.10857584	0.78994851	0.011152
3355.52121	-1.73741963	0.78998957	0.01117
2288.21555	1.75725974	0.7903567	0.01133
1847.89249	2.04444092	0.790759	0.011507
2987.48916	-0.18907244	0.79082685	0.011538
1357.7144	0.52865868	0.79095344	0.011594
1530.69609	0.17457458	0.79116868	0.01169
2220.0479	-1.22616217	0.79142378	0.011806
1015.45378	-3.96113516	0.79205486	0.012095
1756.85941	3.05309139	0.79227817	0.012199
2061.01041	-1.50177363	0.79301481	0.012547
2823.18691	0.96169841	0.79367128	0.012864
2110.11637	0.28238261	0.79367955	0.012868
2017.95902	2.24792838	0.79376211	0.012909
2821.07059	0.7559162	0.79418126	0.013115
3831.95669	-2.43910768	0.79485528	0.013453
1797.74394	-0.57361722	0.7949525	0.013503
2121.89847	-0.03517965	0.7956163	0.013844
1236.58655	0.87028791	0.79572664	0.013902
4589.24482	-1.33399481	0.79644713	0.014282
3017.35416	1.07737731	0.79661318	0.014371
1837.83391	1.01293345	0.79665331	0.014392
1939.85588	1.73943834	0.79678928	0.014465
1097.46965	2.13836522	0.79702574	0.014593
1492.67644	0.20359423	0.797158	0.014666
1500.68413	1.30029362	0.79740819	0.014803
1792.93205	0.07983275	0.79809339	0.015184
1957.8831	0.75997355	0.79917388	0.015801
3601.80849	0.09386552	0.79950748	0.015996
1137.6012	-0.70180672	0.79953537	0.016013
2319.21366	-0.34671268	0.80100355	0.016896
2191.86601	0.78750052	0.80154649	0.017233
2868.3823	2.36685185	0.80176114	0.017367
1424.66985	2.33184091	0.80217285	0.017628
914.446886	0.4162928	0.80220953	0.017652
1833.91765	2.80156408	0.80234525	0.017739
1371.65036	-0.3386853	0.80270998	0.017974
2617.14035	0.05222363	0.80272401	0.017983
2687.277	-0.01006781	0.8039992	0.018826
955.437426	0.78521986	0.80544428	0.019821
1678.57219	-0.02034262	0.80552632	0.019879
1263.5728	0.85725571	0.80608237	0.020274
1802.85234	-0.05867334	0.80624482	0.020391
2335.01689	-0.3704462	0.80633255	0.020454
3202.31797	-0.47903386	0.80643484	0.020528
1272.56133	-0.51031283	0.80658943	0.020641
1375.63823	2.72084071	0.8069191	0.020882
2876.40371	0.95707336	0.80692411	0.020885
1131.49541	0.12693545	0.80699952	0.020941
1617.7404	0.80281364	0.80700637	0.020946
3485.55722	0.72177663	0.80723054	0.021112
1890.78337	-0.08594635	0.80762352	0.021405
1545.82659	1.03788142	0.80819704	0.021839
1163.50981	0.52163183	0.80820277	0.021843
1497.75517	-0.06926908	0.80828155	0.021903

Information Classification: General

2434.99472	-0.74710891	0.80854803	0.022108
2961.32059	0.98688841	0.80860066	0.022149
3269.61911	1.21495564	0.80922834	0.022638
1173.56719	-0.14403042	0.80941825	0.022788
1243.5313	-0.83769605	0.80982992	0.023115
2074.13701	4.36624453	0.81006266	0.023302
1429.61882	2.57531081	0.81009771	0.02333
2876.42539	-1.30827094	0.81019003	0.023405
2213.12711	-0.42878348	0.81031671	0.023507
3781.66933	-3.10790542	0.81078587	0.02389
3440.64219	-6.5898363	0.81089741	0.023982
1199.54399	1.14844579	0.81099065	0.024059
4779.25698	0.75836344	0.8111247	0.02417
3164.40268	-0.27089532	0.81114162	0.024184
1686.90825	2.5368444	0.81128723	0.024306
2196.96953	2.70450752	0.81173511	0.024682
3566.5488	0.85262879	0.81196823	0.024879
2063.87554	-1.61318836	0.81253711	0.025367
1875.90763	-0.35096303	0.81255302	0.025381
1872.87394	0.02094083	0.8127764	0.025575
2042.95049	-0.39073298	0.81297842	0.025751
2865.29824	-3.08164103	0.81407592	0.026726
2061.85137	1.2016667	0.8141861	0.026826
1466.59209	0.24090841	0.81470667	0.027301
1808.79338	-1.6164037	0.81492511	0.027502
2517.01333	-0.48427634	0.81543065	0.027972
1494.62857	1.69800809	0.81547904	0.028018
1749.87908	-0.89685108	0.81623716	0.028737
1756.74419	2.09474081	0.8163857	0.028879
2193.99771	-3.2089749	0.81699558	0.029471
1136.55828	0.06144675	0.81837305	0.030844
1825.76311	1.19392389	0.818957	0.031442
3484.7468	0.48410664	0.81903753	0.031525
2873.31571	8.81757629	0.81922887	0.031724
1109.51409	-0.74772851	0.81939511	0.031897
1091.49919	-1.05560631	0.82018418	0.03273
1299.59599	-0.29273376	0.82019826	0.032745
2221.16123	-2.34440433	0.82022029	0.032768
1067.50334	2.80860684	0.82028842	0.032841
1771.81743	0.40924716	0.82029165	0.032844
3145.52121	-8.96288612	0.82055886	0.033131
1559.83086	-0.42770943	0.82062871	0.033206
2268.01389	-0.87881613	0.8207695	0.033359
1497.85527	1.37790891	0.8208972	0.033497
2815.3815	-1.0834841	0.8213693	0.034013
2218.96506	-1.89515926	0.82151825	0.034178
3335.7179	4.12638831	0.82176762	0.034454
2890.33078	-1.02813005	0.82224824	0.034992
1132.52751	0.54578724	0.82246386	0.035235
1525.72162	0.27133856	0.82272671	0.035534
4157.80771	0.7674898	0.82272945	0.035537
1413.61907	-0.81998509	0.82309729	0.035959
1868.91556	-0.40012718	0.82325925	0.036146
3920.88462	3.21289023	0.82342011	0.036332
2754.16898	-0.71521943	0.82379376	0.036768

Information Classification: General

1949.79057	1.16404425	0.82381778	0.036796
1247.60227	0.64342546	0.8239217	0.036918
2079.87613	-1.92488138	0.82415594	0.037195
1957.85173	1.21248664	0.82522391	0.038477
1192.56755	0.22403893	0.82549487	0.038807
1730.77495	1.43383315	0.82573269	0.0391
1618.6967	-0.77372605	0.82589493	0.0393
1616.70664	-0.50543725	0.82624571	0.039736
1719.82322	0.47679592	0.82635541	0.039873
1376.63249	-0.25526943	0.82703874	0.040737
2109.09171	2.12919003	0.82778138	0.041692
1684.80852	3.05137253	0.82784536	0.041775
1011.5504	2.52414774	0.82784557	0.041775
1381.63237	-1.34151641	0.8280997	0.042107
1973.89927	-4.78504187	0.82813656	0.042155
2331.0712	0.31438102	0.8281987	0.042237
3127.40256	-0.75759105	0.82861857	0.04279
2437.09421	-0.15465203	0.82866444	0.042851
3214.32456	0.24352803	0.82868003	0.042872
1194.63591	-0.45490298	0.82914035	0.043487
2146.84183	-1.41383152	0.82935809	0.04378
1504.64104	0.21301405	0.82936513	0.04379
3068.4318	-0.43945977	0.82936799	0.043793
4180.16096	-0.05294854	0.8294033	0.043841
1301.61943	-1.91525692	0.8305868	0.045465
2023.83721	4.51592739	0.83072235	0.045654
2063.01505	4.90568442	0.83086323	0.045851
1612.82121	-0.30425509	0.83096501	0.045994
1223.5076	-1.30263864	0.83099747	0.04604
1589.81888	0.67901357	0.83134562	0.046531
1404.61333	-0.01740782	0.83139744	0.046605
3001.4813	-1.23870191	0.8314235	0.046642
2473.06125	0.60941235	0.83177877	0.047149
3315.55259	1.6579806	0.8317982	0.047177
2574.29726	1.13957969	0.83191549	0.047345
1367.64695	0.16778704	0.83201746	0.047492
3096.37278	1.1841441	0.83242727	0.048086
1164.46843	-0.57872946	1.19891357	0.031528
1870.98886	0.55928647	1.19871496	0.031684
3183.6456	0.119894	1.19785506	0.03237
3239.66306	2.11063761	1.1977484	0.032456
1909.95023	-0.75386004	1.19758041	0.032591
2435.26396	8.5692207	1.19750898	0.032649
4114.94741	2.77430065	1.19739993	0.032738
1854.9102	0.69305174	1.19738771	0.032748
3320.6312	2.99051672	1.19717116	0.032924
1579.76896	-1.34212615	1.19688503	0.033159
3284.53754	1.76654101	1.19676168	0.03326
1668.82029	0.35193555	1.19548118	0.034331
3036.50278	3.87795936	1.19543415	0.034371
1268.66187	1.26950228	1.19502514	0.034719
1715.74326	1.12543062	1.19497096	0.034766
3225.5654	1.98740496	1.19430237	0.035343
2203.13431	4.20001452	1.19427406	0.035368
3769.54355	1.2210812	1.19412676	0.035496

Information Classification: General

1832.7918	-1.34366292	1.19354063	0.036011
3349.48459	-0.63682699	1.19335511	0.036176
1774.93559	0.71899722	1.19323898	0.036279
3776.54629	-7.02704101	1.19306979	0.03643
1934.98808	-3.9512346	1.1927388	0.036727
1473.726	2.73079744	1.19265659	0.036801
2015.02182	0.81160566	1.19212785	0.037281
1987.90911	10.1649094	1.19147353	0.037882
1753.94767	0.8464838	1.19145896	0.037895
3307.35422	0.74254643	1.19112449	0.038206
3122.50595	1.21257175	1.19104483	0.03828
2505.24717	-0.56673051	1.19097801	0.038343
2029.91581	-2.36347895	1.18951993	0.039728
2826.54897	3.60248535	1.1891928	0.040044
1339.63713	-0.33049271	1.18894915	0.040282
1991.94939	3.28327572	1.18836427	0.040857
1216.63994	-0.36010911	1.18826571	0.040954
2304.17685	1.78575702	1.18812329	0.041096
938.48308	0.19662082	1.18757569	0.041643
3147.42405	1.02213199	1.1872189	0.042003
1678.92185	2.76356949	1.18680326	0.042427
4083.66469	-9.35844281	1.18669431	0.042538
1231.58928	4.22681268	1.18666198	0.042571
1760.94852	-4.25385492	1.1866565	0.042577
4856.27407	1.12169483	1.18658416	0.042651
1428.69866	0.8593115	1.18629438	0.04295
3166.45896	-8.55586893	1.185917	0.043341
1753.98195	-0.34606987	1.18591624	0.043342
1937.99576	-0.49721914	1.1858351	0.043427
2575.11765	-0.70924976	1.18561993	0.043652
1817.80339	-0.07865245	1.18550944	0.043768
3809.74111	1.94692314	1.18537226	0.043912
3183.61484	2.84173234	1.185268	0.044022
3337.61553	3.18305518	1.18453101	0.044807
2422.04935	1.1962013	1.18397599	0.045406
1454.8718	0.67909012	1.18356597	0.045853
1215.6209	-1.29241927	1.18344711	0.045983
3845.88857	2.04327569	1.18285349	0.046638
1938.01921	0.21379448	1.18269801	0.046812
2913.40224	-0.0397866	1.1826784	0.046833
2782.18668	-5.54802449	1.18264145	0.046875
3430.51682	2.52542715	1.18255612	0.04697
2362.07462	3.67508004	1.18219126	0.047379
2212.07114	-1.58593029	1.18212507	0.047454
3586.68081	-7.99665841	1.18192397	0.047681
2449.2293	2.82960262	1.18183405	0.047783
3002.42337	-15.4622777	1.18076793	0.049007
2511.26328	4.92679575	1.18059417	0.049209
2019.96201	-0.36587322	1.1803499	0.049494
2658.07779	-0.02835079	1.18000713	0.049897
2883.39896	-2.43683462	0.8336836	0.049944
1814.82805	-1.06774389	1.17996444	0.049947
4520.03579	1.61990034	1.17987042	0.050058
2544.1519	1.48763425	1.17971451	0.050243
2186.09067	10.9094557	0.83417889	0.050692

1634.72898	-2.62935118	0.83424532	0.050793
1910.86138	5.86145279	1.17917544	0.050886
1790.95549	-0.83607064	0.83433513	0.050929
3169.77129	-0.52163602	1.17909394	0.050984
2562.15085	2.37836247	1.17896345	0.051141
2937.39603	-2.24724683	1.17885095	0.051276
1252.58173	1.06532688	0.83458142	0.051306
1159.47429	-0.54726283	0.83465885	0.051425
2602.16654	0.3200251	1.17870265	0.051456
2909.38613	-2.07576284	1.17868359	0.051479
3552.82705	-2.30921088	1.17868214	0.051481
3560.72751	1.571836	1.17862731	0.051547
2488.24125	1.56487634	1.17855612	0.051633
1492.81583	1.44609317	0.83486232	0.051738
2153.99883	1.11216199	1.17822373	0.052039
2675.1749	6.82593688	0.8350585	0.052042
1631.71465	-4.92432417	1.1781966	0.052072
2170.00054	1.68920696	0.83531912	0.052447
4570.25098	4.30709869	1.17780804	0.052549
4263.74135	-1.60154796	1.17777077	0.052595
1746.973	-1.06453957	1.17764498	0.052751
2283.98838	1.16381394	0.83574734	0.053119
3887.73667	-12.3374732	0.83575011	0.053123
3212.58608	0.20169926	1.17730178	0.053177
1915.92278	-1.31592416	1.17728925	0.053192
1871.89787	-0.63761642	0.83582511	0.053242
2060.87871	-0.00465426	0.83596683	0.053466
1861.80229	-1.7588374	0.83603231	0.05357
1994.84795	-1.44938898	0.83615418	0.053764
3083.55356	-0.19662886	1.17681914	0.053781
1990.81462	-0.9460981	1.17673528	0.053887
2509.36649	-4.69802621	1.17673468	0.053888
2248.12552	0.06411279	1.17663097	0.054019
3608.6605	-8.61253695	1.17660329	0.054054
1581.81389	0.68407048	1.17652958	0.054147
3571.64719	-2.22345196	1.17650261	0.054181
4257.07729	4.56625812	1.17646636	0.054227
3033.50187	0.74926838	0.83655724	0.054408
2673.11185	-2.28113964	1.17625891	0.05449
1763.75383	4.26560572	0.83675488	0.054727
1514.63835	0.19672208	0.83683588	0.054857
2018.86052	-0.11594087	0.83686318	0.054902
1419.64067	1.4209925	0.83706454	0.055228
1092.5219	1.05525087	0.83711651	0.055313
4241.26919	5.76347408	1.17561529	0.055315
3254.50559	1.06008469	1.17556553	0.055379
1140.62834	1.33715526	0.83719631	0.055443
1415.57732	-2.50582811	0.83727046	0.055564
1682.76457	-0.63665119	1.17521359	0.055835
3092.41037	0.22545773	1.17500854	0.056103
1829.84526	-0.70107431	0.83761028	0.056121
3788.70041	-0.06406674	1.1749692	0.056154
1361.70549	-1.01076578	1.17479558	0.056381
2641.23703	-0.42011979	0.83788601	0.056577
1443.72026	-1.3824406	1.17457397	0.056673

Information Classification: General

1629.77724	0.56454686	0.83795987	0.056699
1127.56794	-1.01948299	1.17452779	0.056733
3029.22342	-0.34612032	1.17432198	0.057005
1745.79643	1.05167903	0.83836528	0.057376
2188.05246	1.33088745	0.83840753	0.057447
1915.07572	0.48219822	1.17374086	0.057779
3229.63327	0.26925609	0.83869665	0.057933
2139.00949	3.06651353	0.83870181	0.057942
1349.70879	-3.37662977	0.83870533	0.057948
3033.34531	-0.16953848	0.83872592	0.057983
2251.99417	2.1325443	0.83892698	0.058323
2099.05606	2.2991886	1.17331734	0.058349
1661.78569	-0.44119407	1.17329473	0.05838
1677.78313	-1.28779848	1.17323506	0.05846
2866.44193	0.19549798	1.173207	0.058498
2627.05002	-1.83984113	1.17308654	0.058662
3011.55703	-0.69856161	1.17294183	0.058858
3283.56113	-0.90723338	0.83930477	0.058968
1405.58173	1.17418216	0.83933916	0.059026
1194.48064	0.82305148	0.83946507	0.059243
2656.10062	1.08082872	0.83955612	0.059399
2978.38218	1.94466837	1.17243358	0.059554
2762.35123	8.83853622	1.17230715	0.059728
1942.96303	-0.29743168	1.17229992	0.059738
1702.91343	0.13328078	0.83984459	0.059898
2701.09293	1.96489358	0.83997058	0.060117
2184.07085	4.6120665	1.17189682	0.060296
1621.8311	0.56971792	0.84016705	0.060459
1536.60564	-1.83318404	0.84018021	0.060482
1777.85137	0.81764885	0.8401897	0.060499
2603.24009	-1.90373456	1.17119271	0.061281
1805.91789	0.16721604	1.17111033	0.061398
3899.76333	-2.16652378	1.17100958	0.06154
1161.57781	-0.67917292	1.17098266	0.061578
2254.99527	-11.4598421	0.84093486	0.061812
1117.57561	1.03869678	1.170774	0.061874
3268.88833	-0.46649804	0.84134001	0.062535
2698.29343	-0.63304885	1.17031003	0.062537
1338.56963	0.06901163	0.84148976	0.062804
2137.91313	-1.17486708	0.84155012	0.062913
1613.679	2.05144695	0.84162564	0.063049
3202.56552	-0.19154175	0.84163643	0.063069
1591.83561	1.89098761	1.1698979	0.06313
2190.98833	-0.49724192	0.84170585	0.063194
3687.85749	-4.22335471	1.16980392	0.063267
3932.80295	18.9183983	1.16978887	0.063288
3809.71181	3.41138615	1.16974091	0.063358
1709.72869	-0.69192612	1.1692391	0.06409
2348.18735	7.3941063	1.16919724	0.064151
1546.68828	0.40196893	1.16908263	0.064319
2973.40519	-3.40343136	0.842377	0.064417
4534.26963	-0.82744583	1.16882947	0.064692
2015.01834	-0.2548864	1.16876536	0.064787
1331.72051	-0.7221612	0.84272185	0.065053
1370.68096	0.16597726	0.84275327	0.065111

2774.26664	1.73538289	1.16826244	0.065535
3447.57859	1.65075235	1.16819722	0.065632
2814.21018	2.40667714	1.16796476	0.065981
3271.50766	-4.91570089	0.84328206	0.066096
1929.97976	0.56929823	0.84362111	0.066733
3280.59372	-0.02596567	0.84362513	0.066741
1707.79607	1.09486525	0.84372473	0.066929
1100.56218	-0.01264098	0.84372854	0.066936
3520.78855	0.13833149	0.84374058	0.066959
2707.47646	0.29541739	0.84390576	0.067272
2534.27488	-1.35061871	1.16677694	0.067786
3486.58445	1.43879304	0.8441874	0.067809
2216.11026	-0.13972927	0.8442766	0.067979
2341.11032	1.84629474	1.16651688	0.068187
1653.679	1.73151916	0.84443339	0.06828
2663.33567	-0.30030891	1.16631275	0.068503
1141.5866	0.79463995	0.84461001	0.06862
3440.62168	19.6539827	1.16621772	0.068651
2192.1207	-1.64573226	0.84470559	0.068805
1465.67119	-0.25798633	0.84475582	0.068902
1972.93979	-2.25543977	0.84478044	0.06895
1606.85869	1.2813055	1.16591151	0.069128
1523.71343	-6.47877405	1.16570575	0.069451
3480.76133	1.64945416	1.16561203	0.069598
1955.97361	0.90370835	1.16550515	0.069767
1476.60002	1.88804324	0.845345	0.070049
2792.58268	1.69679986	1.16526096	0.070152
2798.20823	9.53208917	0.84541085	0.070179
2733.29605	-3.12210594	1.16521078	0.070232
3288.45815	0.23788922	1.16509188	0.070421
1633.89713	3.31145628	1.16504473	0.070496
2743.21634	-0.55466056	0.8456529	0.070655
3581.51772	0.25480853	0.84567267	0.070694
2766.16111	-1.98121877	0.84568297	0.070714
1874.95342	1.62054126	1.1647233	0.071008
3923.96474	-0.62587575	1.16447467	0.071407
3112.38572	0.51728958	1.16438713	0.071548
2546.16648	2.03982084	1.16435178	0.071605
2367.09648	-0.94299256	1.16434583	0.071615
1952.91777	-2.19163957	1.16411227	0.071992
3243.41086	0.92047822	1.16399757	0.072178
3258.46543	-0.22528435	0.84646651	0.072275
4003.99179	0.60092687	1.16392626	0.072294
3033.3931	2.79794221	0.84653856	0.07242
1189.53752	0.3428935	1.16356889	0.072877
1368.62432	-2.49894956	0.84682318	0.072994
2668.28427	-3.38173301	1.16347238	0.073035
2129.10422	1.77063388	1.1634683	0.073042
2749.29508	-1.60957708	1.16346817	0.073042
2508.06182	-3.05539643	1.16344539	0.073079
2042.71343	0.62182242	0.84689956	0.073148
1464.75798	-0.02944578	1.16314944	0.073566
1241.62554	-0.32974515	1.16298533	0.073837
3994.89931	-8.06919007	1.16261588	0.074451
1967.96086	2.6462561	1.16261056	0.07446

2541.11423	-0.32598704	1.16248972	0.074661
2661.34879	-0.6711878	1.16246085	0.07471
1056.52287	2.02498899	1.16240655	0.0748
1856.91777	-2.30817537	1.16230448	0.074971
3487.55417	-1.12427331	1.16227818	0.075015
2671.36814	-2.03308568	1.16223426	0.075089
2538.17649	-0.17434207	0.84788017	0.075157
1805.88188	0.37169444	1.16205843	0.075385
1101.46745	1.67914658	0.84810503	0.075624
4786.44371	-0.02388983	1.16189836	0.075655
1630.88523	2.7032702	1.16178322	0.07585
2697.36197	0.10133193	0.84822541	0.075875
1080.46221	1.24900424	0.84827388	0.075976
1214.64775	4.34230983	0.84834979	0.076135
3177.31386	-2.72394261	1.16139772	0.076505
2750.31499	-3.82003336	0.84860086	0.076661
2362.18595	-6.98000161	1.16122897	0.076793
1894.89336	0.41155119	0.84873098	0.076935
3403.34214	0.46950999	1.16072602	0.077658
2514.30338	4.33940431	0.84916338	0.07785
1673.6602	0.40303805	0.84919213	0.077912
2684.19101	-4.27173167	1.16036012	0.078293
2163.08896	-1.40952363	0.8493819	0.078316
1411.63188	-1.19136141	0.84948003	0.078526
1660.83281	2.58483009	1.16013556	0.078684
1183.63384	1.01244254	0.84956941	0.078718
1189.57244	-0.90789422	0.84957717	0.078735
1569.8591	-1.44428467	0.8497797	0.07917
2026.87932	2.53283934	0.84982207	0.079261
1833.91338	0.47187445	0.84991959	0.079472
2845.19529	0.32803273	0.84993703	0.07951
2070.89018	0.72385176	0.84996283	0.079566
3979.63254	1.23960605	0.85004899	0.079752
2569.30753	0.42344854	1.15949643	0.079808
1559.78154	0.20287416	0.8500993	0.079861
1661.72759	-0.80557052	0.85024256	0.080172
3365.54697	-1.00878476	1.15928185	0.080188
1640.82329	1.61777704	1.15920279	0.080329
1283.60161	0.13617835	0.85034556	0.080397
2320.99306	5.98295546	0.85045795	0.080642
1417.65776	3.46144368	0.85047357	0.080676
1644.79209	0.83540954	0.85047463	0.080679
1993.91252	0.09222305	0.85052803	0.080795
3853.73508	-0.92241964	1.158878	0.080908
2646.37076	-1.34773579	1.15878291	0.081078
2866.33706	-4.50102727	0.85078571	0.081361
1721.86296	1.67861083	0.85081303	0.081421
1923.8532	-1.79758372	0.85081362	0.081422
2095.98062	0.88105426	0.8509107	0.081636
1642.79473	1.17910844	0.85093811	0.081697
1259.57854	2.08046549	0.85116093	0.08219
1375.71367	4.66110482	1.15808042	0.082346
2416.04679	1.46883829	0.85129288	0.082483
3163.36191	-17.0172455	1.15798664	0.082516
2259.87554	-2.54899879	0.85133512	0.082577

Information Classification: General

1882.95499	0.24690289	0.85134331	0.082595
2087.98862	-0.16653437	1.15790339	0.082668
1765.74236	5.10146286	0.85145563	0.082846
1459.72905	0.18236604	1.15774602	0.082955
1946.99283	-0.58875209	1.15773391	0.082977
2033.14469	-1.29888805	0.85158347	0.083131
2286.01592	-5.9923295	1.15763827	0.083152
1369.71219	3.10580244	1.15755561	0.083304
2087.77446	-2.46369464	0.85166313	0.08331
3405.52951	-1.73281928	0.85168898	0.083368
1295.67716	1.53578055	1.15747516	0.083451
2137.07993	1.28861436	0.85194375	0.08394
1382.72856	0.16989867	0.8520292	0.084133
3341.69057	1.73479968	1.1571038	0.084136
2888.3126	-3.12885541	1.15710015	0.084143
985.483202	-0.42964139	1.15685305	0.084601
1527.75959	3.24517964	0.85228023	0.084701
1476.69341	-7.09174037	1.15673482	0.084821
3416.80459	0.18287134	1.15645433	0.085345
1611.87273	0.48822888	1.15644926	0.085354
3941.80661	4.33264334	0.85257923	0.085382
3035.37503	0.92767856	0.85260387	0.085438
1400.70439	1.33861355	0.85262104	0.085478
2697.15508	1.27860487	0.85273592	0.085741
2016.96457	-0.78463125	1.15605206	0.086101
2094.17388	2.12084765	1.15604889	0.086107
2991.29276	-0.53807447	1.15601662	0.086168
2085.93491	0.96903341	0.85298702	0.086317
3554.64621	2.28547402	1.15581844	0.086542
1567.70036	0.71868425	0.85310803	0.086596
2783.32761	5.77780922	1.15561103	0.086936
959.450853	0.65450232	1.15547837	0.087189
1695.93901	-0.99582443	1.155471	0.087203
1821.81621	0.85706128	1.15545969	0.087224
2937.38613	0.52795387	0.85341445	0.087306
3531.51816	-0.88084107	1.15522427	0.087674
1211.54155	1.19368997	0.85359634	0.087729
1700.85539	2.24297636	0.85379419	0.088191
2530.19144	2.25085828	1.1549117	0.088275
1830.76348	0.05846366	0.85383061	0.088277
1681.65666	-0.32199814	0.85384582	0.088312
1507.67009	1.04283031	0.85399934	0.088673
1931.79912	0.12659928	0.85403105	0.088747
4495.0226	4.09662317	0.85412021	0.088957
1538.73979	1.02381995	1.15440863	0.089248
2095.99272	0.83591893	0.85431686	0.089421
2549.25913	-0.41341791	1.15431259	0.089435
2740.27937	0.0006823	0.85439919	0.089616
1854.00713	3.45326443	1.15421389	0.089628
1687.80364	0.35440493	1.15411188	0.089827
1581.66741	0.6832645	0.85450164	0.089859
2271.88066	-1.44790478	0.85453355	0.089935
3105.58408	4.19607461	0.85454059	0.089952
1458.73633	0.77153063	1.1538847	0.090272
1805.87712	-2.26455382	1.15368388	0.090667

3994.97934	-0.63205974	0.85490772	0.090827
1418.62993	0.60065788	0.85492829	0.090877
3833.72275	-3.71619531	1.15356555	0.090901
2551.18393	0.7305542	1.1535163	0.090998
2017.05058	-0.87731656	1.15351087	0.091009
1272.53528	0.45786949	0.85533907	0.091864
1979.88494	0.569222	1.15307258	0.091879
2531.33037	-2.62132344	1.15307219	0.091879
1303.6623	-1.34941754	1.15307171	0.09188
2016.06644	0.8159961	1.15279406	0.092435
1323.59369	-0.23700656	1.15268567	0.092652
1739.71282	-0.09435268	0.85577607	0.092924
2636.28806	-2.50095785	1.15252239	0.092981
1805.8366	-0.0242822	0.85581134	0.09301
1592.67058	-0.31737554	0.85583548	0.093069
1453.6917	-0.03254516	0.85588207	0.093183
2125.98795	-0.66750547	0.85627306	0.094141
2372.02251	-3.45307168	0.85632439	0.094267
2255.15805	-2.45398681	1.15188423	0.094273
1589.84122	0.53613894	0.85634009	0.094306
1679.8751	-0.02387826	1.1517728	0.0945
2605.26999	-1.10992395	1.15176328	0.094519
2147.93753	-2.91030235	0.85650701	0.094717
1576.61345	0.09870464	0.85655012	0.094824
1827.72807	-0.48385617	1.15159009	0.094873
1636.75701	-1.05311505	1.15155807	0.094939
2676.3129	1.44940574	1.15147568	0.095108
1800.86077	-0.6624209	0.85669306	0.095178
2993.3721	0.44771074	0.85670735	0.095213
3089.70664	-0.91469647	1.1513496	0.095367
2030.81914	-0.91386985	0.85692899	0.095764
1334.66167	1.55124894	0.85705767	0.096086
1965.83782	0.22374859	0.85707714	0.096134
1389.54843	0.70320013	0.85708529	0.096155
2844.0916	-0.30869852	1.15086359	0.096371
3035.4415	2.36938762	0.85727194	0.096622
2295.04007	1.27575761	0.85731294	0.096725
3306.3497	0.90439995	1.1506122	0.096893
1142.58171	0.67770891	0.85738829	0.096914
2225.16703	0.8246751	1.15059535	0.096929
2481.22373	1.63495938	1.15057095	0.096979
1513.72463	-0.28289099	1.15021064	0.097733
1645.68437	1.22044704	0.85782499	0.098017
2659.0977	2.3461149	1.1500549	0.098061
3372.44272	0.9310286	0.85788736	0.098175
3559.88596	3.80746616	1.14983337	0.098528
1428.56719	-0.46202193	0.85804281	0.09857
2585.22099	-1.09601293	1.14980026	0.098598
2963.52921	-0.43844446	0.85809811	0.098711
1915.91716	3.41693091	0.85826676	0.099142
1863.82523	-0.20164721	1.14953584	0.099158
2338.95829	0.02599682	1.1493869	0.099475
2669.21189	6.18792377	0.85841834	0.09953
1760.88132	-0.54377689	0.85849277	0.099721
2794.29233	1.88355941	1.14913818	0.100006

Information Classification: General

1712.7396	0.48509454	1.14913711	0.100009
1524.67912	-1.37770156	0.85865411	0.100136
3123.27719	-2.0925146	0.85866056	0.100153
1288.54729	-0.07022661	1.14901717	0.100266
2589.38506	2.39132622	1.14899816	0.100306
2366.04863	-0.90222866	0.85872504	0.100319
1400.62993	0.8104291	1.14899185	0.10032
1342.59226	2.61134638	0.8589825	0.100985
2031.92449	-0.92140466	1.148673	0.101006
1135.56841	3.97921756	0.85905441	0.101172
2733.40688	0.1912476	1.14856139	0.101247
1356.59514	-0.1584318	0.85915072	0.101422
2069.92974	-4.83117909	0.85915978	0.101446
2074.88555	-0.69866715	0.85918275	0.101506
2498.07231	-5.13398919	1.14829807	0.101818
1351.72281	0.90785821	0.85933568	0.101904
2076.93486	-0.37230254	0.85952942	0.102411
3067.43698	-0.99446674	0.85953722	0.102432
1600.82878	-3.17669806	1.1479923	0.102484
1140.54289	0.89341527	0.85961583	0.102638
2630.34902	2.12307543	0.85972291	0.102919
4022.8414	-0.53084836	0.85973856	0.102961
2639.23239	-0.47032911	1.14772967	0.103059
2593.16947	9.27877888	1.14768076	0.103166
1379.60784	0.34095536	0.85984109	0.103231
3646.57798	0.35231576	0.85984835	0.10325
2132.97539	-0.80915344	1.14743765	0.103702
1817.80596	1.32054902	0.86004858	0.103779
1318.67575	-0.11673288	1.14735177	0.103891
3336.5436	4.46495214	0.86009512	0.103902
1645.80704	0.21368743	0.86014501	0.104034
3133.49778	-0.83818423	1.14722103	0.10418
2401.29453	-1.23080227	1.14692641	0.104835
3534.60093	0.06647586	1.1468435	0.105019
4805.39294	15.3768589	1.14668188	0.10538
1958.78276	-0.95102813	0.8607307	0.105596
1346.62407	0.11490679	0.86075864	0.105671
1839.03458	-0.40850195	1.14655159	0.105672
2740.30606	-0.00761748	0.86087208	0.105976
1319.72112	0.44830281	1.14633393	0.10616
2571.16289	1.50169234	1.14615973	0.106553
4141.81565	0.32054669	1.14597946	0.10696
2505.17641	0.58439298	1.14596178	0.107
1993.96067	-0.36551545	1.14590651	0.107126
2550.48355	-1.60857334	1.14572037	0.107548
3969.02309	1.31863556	1.14560883	0.107802
1292.63262	-4.00140133	1.14560559	0.10781
1598.67815	-0.61370409	1.14558391	0.107859
3631.48386	0.01666975	0.86158775	0.107913
2547.33281	0.35172892	1.14552765	0.107987
2874.28452	-1.18648114	1.14545039	0.108164
2657.32138	0.79740073	1.14543236	0.108205
4472.22036	-1.41332034	1.14540787	0.108261
2899.42714	0.23329759	1.14538183	0.10832
2576.13535	1.52584908	0.86179271	0.108473

Information Classification: General

1292.61223	-0.30023239	0.86179854	0.108489
1966.97898	-2.46127519	1.14522313	0.108684
2553.22318	-2.13048998	1.14521285	0.108708
2438.2725	-4.02055561	1.14520112	0.108734
2280.9935	0.01538392	1.14512844	0.108901
1154.60991	3.3020503	0.86238121	0.110092
2340.05729	0.79905262	1.14459663	0.110129
1971.9948	0.62488771	1.14454335	0.110253
2978.3483	1.63461537	1.1445044	0.110343
2991.50901	1.17600191	0.86252013	0.110477
1352.71662	-0.85819579	1.14444211	0.110488
1471.65984	2.85347042	0.86252858	0.1105
2793.236	3.40922372	1.14429106	0.11084
1554.79961	-0.86644888	0.86272516	0.111047
2718.22563	-0.64224387	1.14414771	0.111175
1679.82383	-0.35280113	1.14411062	0.111261
4237.76674	-0.96936936	0.86286909	0.111448
3197.41623	2.88907735	1.14398996	0.111544
1561.69853	-1.97949416	0.86292096	0.111593
2153.98789	-0.1857688	1.14377564	0.112047
1638.72807	0.7967414	1.14371796	0.112183
2462.09891	0.40932777	0.8632421	0.112493
1628.79801	1.35651268	0.86342938	0.11302
1900.73369	1.14423019	0.86345509	0.113093
1584.65129	-2.18507635	0.8634563	0.113096
3880.82444	4.24232224	1.14325538	0.113277
3720.69614	5.55751091	1.14313083	0.113573
3195.25559	1.0759864	1.14308432	0.113684
1183.64389	0.01327733	0.86367015	0.113701
1513.71428	-1.72047185	0.8637652	0.113971
2812.44863	2.95465108	0.8639112	0.114386
1227.53386	-0.94435251	0.86397817	0.114576
4182.97329	14.1624159	0.86405353	0.114791
1695.81975	0.23083066	1.1425708	0.114912
3615.77854	2.91120438	0.86413004	0.11501
4967.32313	8.46111188	1.14246118	0.115176
2816.29538	2.50114866	1.14237833	0.115375
2894.39573	-1.99013205	1.14224234	0.115704
3329.53017	-0.26827415	1.14219948	0.115807
3003.48758	0.98108628	0.86442138	0.115845
1908.8646	1.41594151	1.14214939	0.115928
1851.86917	5.44124687	0.86447522	0.115999
1943.89304	4.73854438	0.86459281	0.116338
2156.04653	4.37244506	0.8646451	0.116489
2264.90239	1.7934763	1.14188709	0.116565
1820.85796	-0.67031313	1.1418393	0.116681
1431.79566	0.16247545	1.14181994	0.116728
2240.11759	-1.63091292	1.14181756	0.116734
1502.60381	0.42397362	0.8648158	0.116982
1358.64372	0.24955517	0.86485128	0.117084
1516.87204	-0.85825981	0.86495246	0.117378
3335.42392	-2.29505684	0.86495597	0.117388
1500.73166	1.24224094	0.86499246	0.117494
1556.70696	-0.32024111	0.86501297	0.117553
2883.40664	-0.23388416	1.14136725	0.117834

1390.65666	2.77603365	0.8651298	0.117893
1097.59586	0.0009545	1.14128586	0.118034
1644.68938	0.79671788	0.86536002	0.118564
3110.48215	-0.42419418	1.14097849	0.118791
2338.97733	2.66046795	0.86552176	0.119038
1588.79582	0.88284845	0.86555099	0.119123
2941.34635	3.52604817	0.86556622	0.119168
3093.44309	1.83486536	0.86557239	0.119186
3240.42368	-2.14173479	1.14065651	0.119589
3294.57973	-0.68162867	0.86577382	0.119778
2615.24965	4.26467698	0.86578625	0.119815
1189.53716	0.02662695	1.14054186	0.119874
2168.84892	0.11031284	0.86584803	0.119997
2087.97124	-2.64672946	0.86605979	0.120623
2561.34819	0.24472659	1.14022821	0.120656
1374.66446	-0.24183117	0.86607733	0.120674
1088.45976	3.05853807	0.86608702	0.120703
999.515612	1.07678518	1.14017421	0.120791
2479.97202	0.29464672	0.86628114	0.121279
1248.54143	0.16349853	0.86631955	0.121393
3253.41355	0.41465246	0.86632202	0.121401
1860.74649	-0.76323493	0.86632956	0.121423
3123.43503	-0.27744744	1.13991903	0.121432
2206.97661	-3.50171416	0.86638823	0.121598
2665.20473	0.58956822	0.86642412	0.121704
1400.65349	-0.32507572	0.8665879	0.122193
2495.17893	-0.17237429	0.86663701	0.12234
1878.84136	0.77222725	0.8666523	0.122386
2076.99845	1.45832477	0.86666275	0.122417
1915.00101	-0.03685907	0.86672275	0.122597
1645.8942	2.07418643	0.86672815	0.122613
2821.33591	1.20237976	0.86684675	0.122968
1239.51958	-0.14638837	1.13929317	0.123014
2555.28861	2.58525439	1.13920134	0.123248
1180.59665	0.85338603	0.86695624	0.123297
1948.86563	0.79863807	1.13911902	0.123457
1447.71878	0.84881978	0.86709353	0.123711
2023.86893	1.74484457	1.13889319	0.124034
2963.20951	0.2974844	0.86722201	0.124099
1907.77947	-2.92106925	0.8672447	0.124167
2867.31314	8.4975734	1.13882813	0.124201
2522.26528	0.93059006	1.13874466	0.124415
4052.73008	2.11002364	0.8673387	0.124452
2100.80132	0.62920277	0.86746955	0.124849
1086.54729	0.66587961	1.13844593	0.125183
1140.58953	-0.80937284	0.86764946	0.125396
2152.06279	-0.49559921	1.13825453	0.125677
1963.86509	-0.88594585	0.86784241	0.125984
2200.04184	-4.29767536	0.86785856	0.126034
1995.05527	5.509957	1.13805791	0.126187
1683.87004	1.25390671	1.13805043	0.126206
1725.95391	1.53174463	1.13802289	0.126278
1668.84624	0.83333321	0.86797622	0.126394
2966.3788	1.50303987	1.13788496	0.126637
1879.75542	-1.20047932	1.13787479	0.126663

2903.33768	-0.20688716	1.13773425	0.127029
2308.12955	-2.49879652	1.13773064	0.127039
1963.89372	-2.78576349	0.86820802	0.127105
1772.81706	-1.33352715	1.13770054	0.127117
3438.44106	1.78640404	1.13767809	0.127176
3010.37075	1.68615278	0.86834832	0.127537
2179.09146	-0.87994636	1.13746473	0.127734
1186.68985	-1.89418635	0.86841325	0.127737
2880.34568	-0.61557119	1.13732623	0.128098
2113.98393	-2.55630254	1.13732357	0.128105
3815.89194	1.06098147	0.86861799	0.128371
1285.69914	-1.22425341	0.86863522	0.128424
2496.00931	-0.10328644	0.86874456	0.128763
1925.97612	1.87620057	0.86875033	0.128781
3610.64499	4.28381867	0.86882549	0.129015
1544.75981	0.46474782	0.86882619	0.129017
3150.58298	3.73982522	0.8688405	0.129061
2519.11455	1.94681575	0.86887638	0.129173
2133.94878	2.80689376	0.86889837	0.129241
2637.05344	0.1822542	0.86894746	0.129394
2383.02665	-3.50596983	0.86894906	0.129399
1140.52129	0.51076041	1.13678745	0.12952
3980.7407	4.6331763	0.8692087	0.13021
3060.65639	2.22222797	0.8692509	0.130342
1157.51091	0.00566436	0.86944977	0.130966
1817.76933	0.66917628	0.86960734	0.131462
1724.76164	0.39946299	1.13603628	0.131525
2123.02109	-9.53217979	0.8696339	0.131546
1579.76164	1.15142588	1.13599199	0.131644
2465.26645	-0.65132539	0.86970279	0.131763
1516.74846	-0.92804796	1.13592275	0.13183
1315.61272	0.23046082	0.86976772	0.131969
1707.80327	-2.82704986	0.8699581	0.132571
2036.16311	0.05173625	1.13564194	0.132587
1769.86026	2.33653731	1.13557288	0.132774
2374.14182	0.38056712	0.87010739	0.133045
2963.33438	-0.8087382	0.87012287	0.133095
2819.24485	-0.44768582	1.13506606	0.134151
2596.2872	0.79123084	0.87047153	0.134207
1402.73371	0.87824798	1.13502439	0.134265
1301.72376	1.96909487	1.13499646	0.134341
3370.47251	11.016852	0.87053669	0.134416
2563.16581	-1.32362225	0.87058062	0.134556
2951.3754	1.70498923	0.87062989	0.134714
3334.61587	3.45920879	1.13473677	0.135053
4632.12075	-1.59258694	1.13467477	0.135223
1508.6331	0.60646911	0.87079583	0.135248
1779.86504	-0.28904825	0.87085806	0.135448
1603.66191	-0.007774	0.87090961	0.135614
2944.46317	-8.08751228	1.13451614	0.135659
4600.2954	7.60552667	1.13434977	0.136118
4870.3401	-1.1313686	1.13434678	0.136126
1855.0065	1.26984941	1.13417864	0.136591
1752.72075	-0.21428185	0.87123359	0.136661
4952.0226	1.83933202	0.87124534	0.1367

1188.55705	-0.30950581	1.13410935	0.136783
2849.39243	-0.62368669	1.13407129	0.136889
2734.2957	1.97403508	0.87133136	0.136979
2781.27349	0.06356755	1.13397326	0.137161
2481.20981	-0.61514976	1.13383474	0.137546
1870.84897	-0.21549541	1.13381516	0.137601
2094.17136	0.91947391	1.13378605	0.137682
1454.79709	-0.65235365	1.133766	0.137738
2758.32705	0.11701771	0.87162569	0.137937
1808.85539	0.28690985	1.1335674	0.138293
3932.74179	1.2075815	0.87178912	0.138471
2131.01235	0.94251331	0.87185054	0.138672
2546.19887	2.49075006	1.13338972	0.13879
2709.33213	-0.53218374	0.87191925	0.138898
3761.46238	4.95981591	0.87196674	0.139053
3879.702	-0.40178746	1.13322016	0.139267
2441.01542	-2.83090727	0.87214378	0.139635
2408.2183	-6.6935082	0.87215267	0.139665
2827.18631	1.96207416	1.13306156	0.139714
1560.86504	0.58911764	1.13300135	0.139884
2172.03237	0.16207804	0.87231097	0.140187
2345.04082	0.03530136	1.13285774	0.14029
3313.35019	0.19905646	0.87234829	0.14031
2160.96732	-0.14298754	0.87235545	0.140334
2856.34421	0.69532882	1.13276447	0.140554
3033.36269	-0.39088488	0.87242833	0.140575
2753.41291	4.16493709	0.87244956	0.140645
1717.79741	0.90964161	1.13269098	0.140762
1408.66252	1.7994975	1.13268545	0.140778
1606.97439	-1.63995127	1.13260501	0.141006
1556.80486	0.77650648	0.87261352	0.141188
2408.08976	-0.01763554	1.1324998	0.141306
2602.16086	-1.49126482	1.13248382	0.141351
3726.6727	0.21126335	0.87267067	0.141378
1991.87871	-2.08626753	1.13245611	0.14143
1445.5855	0.41333944	1.13238119	0.141644
3533.73623	1.46741587	0.87275579	0.141661
1961.90532	-0.63727018	1.13223353	0.142066
3861.87925	0.93692161	1.13214769	0.142311
2088.84892	-3.3280282	0.87299046	0.142444
2097.90117	-1.22677579	0.87300016	0.142476
1690.90139	-0.58395278	0.87308469	0.142759
4005.78994	-2.51251364	0.87310695	0.142833
1468.66045	1.93284253	0.87317123	0.143048
2408.04032	0.54611024	1.13183801	0.1432
2091.07614	-0.38798578	0.87333779	0.143607
1591.78618	0.75079886	0.87341032	0.143851
1834.83745	7.98470867	0.87343388	0.14393
4976.41711	0.81982094	1.13154503	0.144045
2994.41343	-1.68080811	0.87349442	0.144134
3296.72202	1.47137538	1.13129379	0.144773
2755.52627	-0.37941096	1.13126709	0.144851
2225.83452	2.05808426	0.87378328	0.14511
1444.65813	0.07777164	0.87380861	0.145195
4319.99965	1.26995265	0.87384708	0.145326

Information Classification: General

1715.81321	0.14001469	1.13104331	0.145502
2382.24515	-3.29939182	1.13103744	0.145519
2999.41257	-0.33024019	0.87398682	0.1458
991.535082	0.48054622	0.87404104	0.145984
4068.93052	1.35264275	1.13084577	0.146079
4085.87019	0.68631211	0.8742905	0.146834
2429.07456	1.5231225	0.8744031	0.147219
2616.20908	0.84101172	0.87442613	0.147298
1316.6043	0.35420333	0.87452754	0.147645
1788.75591	1.73230665	0.87456766	0.147782
2724.29182	2.70457134	0.87459333	0.147871
2400.1807	-0.8041416	1.13015634	0.148107
3610.77895	0.87323817	1.13011567	0.148227
2271.12339	-1.21177779	1.13008281	0.148324
3510.41201	6.03738552	0.87486995	0.148822
1926.98655	0.77171416	1.12983847	0.149049
1618.73784	4.52529577	0.87494921	0.149095
2153.04668	-0.56656546	0.87507003	0.149513
2317.22637	4.22688878	0.87507416	0.149527
2877.25266	4.13219327	1.12967125	0.149547
2016.93802	0.52412795	1.12960147	0.149755
4805.29018	0.78528842	0.87515996	0.149824
2346.33799	-0.89607188	1.12956345	0.149869
1141.57793	3.03979084	0.87520874	0.149993
2354.11448	5.355207	1.12949711	0.150067
2491.20847	2.54892797	1.12935541	0.150491
1218.56279	-0.7250842	1.12931266	0.150619
2796.28745	2.43658896	1.12930189	0.150651
4024.98891	15.6532127	0.87544733	0.150822
2512.27439	-2.93682901	1.12919979	0.150958
1889.79802	2.32084673	0.8756739	0.151611
2299.17423	-2.29335022	0.87574348	0.151855
1132.59949	-0.14511798	1.12883225	0.152065
2144.0015	-2.71422567	0.87581172	0.152093
1705.73894	-1.99274321	1.12878702	0.152202
1746.93279	0.87098747	0.87585763	0.152254
2771.35233	-5.83441723	1.12876333	0.152273
3517.48349	2.25028064	1.12866769	0.152563
1200.66016	0.8092088	1.12864276	0.152638
1477.80109	-0.51308627	1.12863941	0.152649
2162.01542	-4.76559427	0.87597057	0.15265
1107.49443	-0.72500195	1.12859843	0.152773
997.447435	0.19048028	0.87606102	0.152968
1003.52277	2.08709569	1.12850077	0.153069
3225.36106	0.66591043	1.12841924	0.153317
1615.86325	-2.23220177	1.12837068	0.153465
1442.71965	0.08511074	1.12818959	0.154017
1843.96048	2.54618856	1.12812953	0.1542
2882.36277	2.76779385	1.12812799	0.154205
1009.45885	0.37006787	0.87647762	0.154437
1947.85251	-0.83842093	1.12802374	0.154524
2719.27878	-0.10171413	1.12798916	0.15463
1952.94414	-0.30769153	1.12795553	0.154733
1609.74187	-2.08506236	0.87668613	0.155176
2576.28872	-0.96740128	1.12771258	0.155478

2533.3165	0.09321279	1.12766043	0.155639
1442.77568	0.73058091	1.12765043	0.15567
1360.64006	-1.58752698	1.12752956	0.156042
1697.7155	-0.69187074	0.87696052	0.156153
1188.637	-0.88442227	0.87701398	0.156344
2587.19785	6.76592776	0.87703678	0.156425
2607.27811	-0.4813356	1.12739455	0.156459
2746.5728	0.12532164	1.12736861	0.156539
2153.98441	-1.80091724	1.12730494	0.156736
1571.84148	0.53247279	1.12729954	0.156753
2159.06906	-2.14601099	1.12726914	0.156847
3942.8591	0.15361354	1.12725813	0.156881
1779.84416	1.13184611	0.87719534	0.156992
1859.80339	0.27531153	0.87723263	0.157126
4084.93027	2.53239788	1.12713264	0.15727
2759.35366	-5.46319922	0.8773022	0.157375
1324.74907	0.80221702	0.87732328	0.157451
2351.00315	-0.58628364	1.12704312	0.157548
2114.99814	4.50296495	0.87735621	0.157569
1675.85649	1.08279632	0.87740429	0.157742
3406.57981	0.11560028	1.12694379	0.157857
3529.55307	0.57100802	1.1269264	0.157912
2356.13051	-0.28369821	1.12686156	0.158114
1482.65776	-1.12828297	0.87751999	0.158158
1695.85393	-1.06663981	1.12682401	0.158231
1025.48943	-0.34892455	0.87754158	0.158235
1558.69475	-0.46805368	0.8776625	0.158671
1999.99956	1.22200902	0.87783909	0.159309
1128.53189	-0.07159398	1.12642811	0.15947
3009.16404	0.80730633	0.87796461	0.159764
1587.78887	1.18885966	1.12626378	0.159986
1452.70085	0.29872827	0.87809341	0.160231
3016.39853	-1.40751046	0.87810185	0.160262
1883.03684	-1.90275005	0.87812294	0.160338
1529.72417	0.72130161	0.8781603	0.160474
2381.94145	2.05611634	0.87816783	0.160502
1924.87822	0.1159174	1.12604095	0.160689
1176.60202	0.29372586	1.12599962	0.160819
3112.47324	2.32947184	0.87827093	0.160877
3438.60935	1.27371758	1.12596113	0.160941
1609.72063	-1.31491547	1.12594305	0.160998
3423.39255	-2.16687327	1.12592993	0.16104
1573.67595	-2.47724504	0.8783594	0.161199
2744.31351	4.00661761	1.12586385	0.161249
1191.56401	-0.79379495	0.87839607	0.161333
3172.46079	0.90442312	1.12579685	0.161461
1507.73039	1.94264071	1.12570162	0.161763
2577.03733	2.31635481	0.87852757	0.161814
3190.45639	2.06638638	1.12558047	0.162148
3680.66233	-1.45481077	1.12557094	0.162178
1196.58001	0.57754195	0.87865791	0.162291
2151.0352	0.1727302	0.87866516	0.162317
2277.07793	2.63063378	1.12544826	0.162569
2566.99143	-4.26768261	0.87875628	0.162652
1373.72087	2.98786613	1.12536588	0.162832

Information Classification: General

1180.57378	0.51057165	0.87882743	0.162913
1399.7553	-0.38912359	1.12519726	0.163371
1229.67461	0.08119679	1.12519403	0.163381
3008.2919	-9.05365282	1.12511542	0.163633
3358.65744	-0.5036178	1.12511265	0.163642
1714.9354	0.07383478	1.12508335	0.163736
2575.32932	1.34216633	0.87912075	0.163994
2210.99942	0.09972901	1.12493741	0.164204
3485.68723	0.25839282	0.87919025	0.16425
1826.99858	-0.1962481	1.12482052	0.16458
2026.97458	-3.89043251	0.87930491	0.164675
3311.64438	-3.91467248	1.12476392	0.164762
1722.88555	-3.07603392	0.87933488	0.164786
2675.39536	1.78947734	1.12475119	0.164803
1701.77959	-1.59226591	0.87936436	0.164895
2112.87432	-1.12837906	1.12464371	0.16515
1915.72954	-0.85894888	0.87947611	0.165309
1582.80704	-0.6427263	1.12457059	0.165386
2330.18125	-2.72216763	1.12439586	0.165952
2427.90874	0.80921699	1.12439289	0.165961
1267.73003	2.74488055	1.12436543	0.16605
4809.17495	2.0116057	1.12435331	0.16609
2280.05034	-7.16419818	1.12428302	0.166318
2023.84905	-0.54978301	0.87977551	0.166424
1514.65398	0.45750569	0.87981459	0.16657
3144.36545	-1.71152726	1.12415147	0.166745
1281.59587	-0.61291235	0.87997105	0.167154
3494.78061	0.21016072	1.12396498	0.167353
1067.50505	0.65309439	1.12386913	0.167666
1220.49541	0.0685188	1.12380801	0.167865
4693.24282	-0.92233508	0.8801635	0.167876
1700.77812	-1.41025413	1.12378596	0.167938
2727.05644	0.56096497	1.12367395	0.168304
2425.14127	3.5668866	0.88029393	0.168366
4365.23691	-4.81295899	1.12364165	0.16841
2164.87432	1.67625291	1.12362435	0.168467
1804.89621	1.11477411	0.88048487	0.169085
1175.54424	0.58590043	0.88048919	0.169101
2503.31133	2.2456638	1.12342318	0.169128
1645.79851	3.66461191	0.88055993	0.169368
1706.78411	-1.54681782	1.1233336	0.169423
2335.00542	0.72521994	0.8806905	0.169862
2748.25925	-2.47532654	1.12320044	0.169862
2893.07645	-2.260615	0.88070248	0.169907
965.543766	2.43442284	1.12316761	0.16997
1601.77898	0.93643557	1.12316421	0.169981
2315.0833	-1.17738663	1.12311959	0.170129
4323.14858	1.37908197	1.12311948	0.170129
1973.98368	0.68614066	0.88076282	0.170136
1477.72583	-1.5439854	0.88082765	0.170382
1459.62187	-1.54802378	1.12304093	0.170389
1751.73479	-3.71830848	1.12302576	0.170439
2898.38083	1.28733676	1.1229864	0.17057
4351.10739	-4.47479367	1.12290231	0.170848
1719.67693	0.01289868	0.88101586	0.171096

Information Classification: General

1482.75347	3.43095978	0.88110559	0.171438
2801.31508	2.17001027	0.88111997	0.171493
3242.66819	0.05788684	1.12270472	0.171505
2119.89433	-0.38237372	1.12268639	0.171566
1477.56206	1.31569262	0.88115783	0.171637
2087.10495	-0.91740191	0.88125035	0.17199
3350.5997	1.15658773	1.12249764	0.172195
1505.75115	1.32686704	0.88132571	0.172278
2705.33438	-5.45018087	1.12234171	0.172716
1641.85709	-0.16535154	1.12229464	0.172873
2586.12027	-4.16256349	1.12229085	0.172886
2750.1126	-3.02310281	0.88148809	0.172899
3448.47242	-0.10698392	0.88152743	0.17305
1905.87175	1.38015599	0.88154922	0.173134
1210.66838	-0.26079835	1.12220413	0.173176
2871.38262	5.7166391	1.12218768	0.173231
1474.63457	0.91804651	0.88158047	0.173253
3795.94814	3.0036035	0.88159978	0.173328
2511.17301	-0.13042305	1.12211272	0.173483
2975.37137	-1.63215156	1.12210471	0.17351
3259.51535	-0.45967675	1.12209277	0.17355
2361.10837	-0.37005727	1.12207427	0.173612
3218.3851	8.84501146	1.12201475	0.173812
3034.43277	0.26730207	0.88172877	0.173823
3017.37368	1.56282682	1.12191612	0.174144
1853.01042	4.32014586	0.88185109	0.174294
1518.75151	0.44256252	0.8818537	0.174304
3940.67978	2.15175756	0.88190448	0.1745
2060.02695	4.98166116	0.88190711	0.17451
1179.56597	1.66185492	0.88195344	0.174689
2377.19126	0.89559343	1.12174524	0.17472
2546.14304	0.15758087	0.88203678	0.175011
1177.58476	0.01621592	0.8820648	0.175119
1517.71013	0.91737318	1.12158505	0.175262
3714.60495	-4.176141	1.121553	0.17537
3933.90371	-0.91793569	0.88214365	0.175424
1383.64092	1.65609397	1.12149018	0.175583
2390.96367	0.91525674	0.88220819	0.175674
1791.82907	-0.30729511	0.88224377	0.175812
1833.91582	1.803127	1.12130391	0.176215
2582.215	-1.62470102	1.12126723	0.17634
3816.79995	1.87386393	1.12117838	0.176642
3606.67197	0.87667732	1.12115852	0.17671
2844.36142	1.43231182	1.12114669	0.17675
1737.88542	2.22412551	0.88248634	0.176754
3192.3281	-11.2047326	1.12112294	0.176831
2136.99149	0.21896693	0.88257908	0.177115
2592.28946	1.28788213	1.12102704	0.177158
1185.61382	1.6184275	0.88262247	0.177284
1324.68061	-4.43249328	0.88266251	0.17744
4879.06118	5.53480096	0.88269069	0.17755
3119.41697	1.60030055	1.12086958	0.177696
1556.79265	0.16176709	1.12083039	0.17783
2162.06313	-0.15135659	1.12078886	0.177973
2814.18657	-1.255874	1.12078466	0.177987

1890.87517	0.11601457	0.88280601	0.178
1314.49919	-0.43605461	0.88288341	0.178303
1365.62578	-1.67343017	0.88299139	0.178726
2895.34707	-1.31890773	0.88308485	0.179093
1101.59104	0.24443764	0.88331117	0.179983
2462.23697	-0.44499011	1.12017584	0.180082
1838.92655	0.19931422	1.12016478	0.180121
1248.66959	-0.6795101	0.88340692	0.180361
1507.65117	-2.32457908	0.88341385	0.180388
2431.29009	2.26684643	1.12007917	0.180417
1630.7293	-0.74791958	0.88343278	0.180463
2271.02471	-0.95221251	0.88351323	0.18078
2251.94731	4.9858732	0.88356008	0.180966
3128.34519	-9.82319316	1.11982973	0.181282
2367.03897	-0.46924363	0.88364386	0.181297
2041.93559	-1.78155778	0.88374943	0.181715
3486.5687	-0.8603292	0.88379621	0.181901
2568.17337	1.08080557	1.11958607	0.182131
1711.76396	-1.53156959	0.88387272	0.182205
1329.67449	-4.65694781	0.88389508	0.182294
1467.6198	0.2352621	0.88394956	0.18251
2599.23686	2.42290539	1.1194041	0.182766
1130.50249	-0.79282886	1.119403	0.18277
3120.51323	0.5008447	1.11932609	0.18304
1899.82146	-3.26429013	1.11921718	0.183421
1086.49504	0.10806854	1.11916361	0.183609
2001.99907	-1.22590654	0.88424264	0.183679
2081.09218	0.77963479	0.88424769	0.183699
1453.77715	0.31566607	0.88434215	0.184077
1149.57976	0.19242929	0.88436362	0.184163
1717.90207	0.80762844	0.88442365	0.184403
1972.02324	-2.56454476	1.11890669	0.184513
2412.32119	-0.90643066	0.88448972	0.184668
3675.90711	0.37336409	1.11882422	0.184804
1404.75025	0.83251481	1.118761	0.185027
2873.20639	4.72980836	1.11869366	0.185265
1653.9003	2.10678178	0.88470513	0.185533
2046.02121	-0.83965329	1.11856993	0.185703
1805.72087	0.20185515	0.88481617	0.18598
2019.06426	0.2898784	0.88494744	0.18651
1309.64133	1.64338053	0.88498946	0.186679
3540.72287	-3.7020102	1.11826915	0.186771
1538.73996	1.7898096	0.88501974	0.186802
2285.05327	2.95045857	1.11824494	0.186857
1651.83432	1.09236326	0.88508258	0.187056
1440.63883	0.88766013	0.88509934	0.187124
2440.13834	0.48829871	0.8851456	0.187311
2285.05661	0.5909885	0.88521525	0.187593
2312.0936	1.65337029	1.11802582	0.187638
1430.64702	-0.49491243	1.11796216	0.187866
3504.63755	-1.29398278	1.11785188	0.188261
3167.63559	3.03631339	1.11782343	0.188362
2503.30881	1.24064111	1.11781297	0.1884
3178.34397	0.41340957	1.11769667	0.188817
1258.56023	-1.37541069	0.88555797	0.188986

2493.06791	0.55712325	1.11763875	0.189025
1579.76017	0.20932816	1.11763852	0.189026
1878.78032	2.66914455	1.11761323	0.189117
1719.79721	4.52322405	1.11759576	0.189179
1059.51543	0.57210882	1.11756606	0.189286
1716.84819	-0.0268923	0.88564129	0.189326
2353.08469	1.76135355	1.1175449	0.189362
1875.91092	0.89797666	0.88565753	0.189392
4597.29555	2.09941527	1.11752301	0.189441
1622.731	-1.97424588	1.11747636	0.189609
2237.0156	0.33518639	1.11742697	0.189787
2182.09014	0.82360914	1.11742082	0.189809
2169.97483	-0.42335385	1.11735115	0.19006
2987.3862	2.20971963	1.11732405	0.190158
4220.96994	1.62013007	1.11730031	0.190244
2557.13871	0.47229638	1.11726493	0.190371
1739.88323	0.72879011	0.8859154	0.190446
2243.26682	1.55466139	0.88592487	0.190485
1987.13175	-0.27994141	1.11721757	0.190543
1629.85173	1.56079784	1.11716236	0.190742
4378.17837	0.89994792	0.88603204	0.190924
1118.57317	0.3508183	1.11710192	0.190961
2072.92974	1.63526949	0.88619115	0.191578
1727.76194	0.25218047	0.88621125	0.191661
3806.84902	-3.34097059	1.11684828	0.191881
1398.6375	-0.57665362	0.88627158	0.191909
1720.65044	0.45501616	1.11675025	0.192238
2669.31626	-1.0984098	0.88636044	0.192275
1286.575	0.28251025	0.88641602	0.192504
2508.03238	-2.78007369	1.11663722	0.192649
2519.26055	-1.46457832	1.11638414	0.193574
1678.87517	1.31121664	1.11635491	0.193681
2566.22293	-1.83840498	0.88685479	0.194321
2370.01006	-0.07676576	0.88686067	0.194345
1649.74443	0.16794382	0.88695977	0.194757
2252.92911	-0.95786379	1.11605809	0.19477
2992.32094	1.04208658	1.11603029	0.194872
2268.96988	-0.03592593	0.88700685	0.194953
2647.26919	0.0254772	0.88704834	0.195126
1832.05278	-0.51108039	1.11593807	0.195212
1861.9749	-0.30246682	0.88712906	0.195462
2626.2374	2.55901275	0.88715298	0.195562
2574.33176	-0.11967123	0.88718609	0.1957
2617.39927	1.48880968	0.88720986	0.195799
1798.9299	-0.22432346	0.88722817	0.195875
3270.56394	-1.49556442	1.11574606	0.19592
1836.79668	-0.78225418	0.88726644	0.196035
1196.54595	1.39629929	1.11554189	0.196676
1410.6762	0.38758695	1.11552317	0.196745
1909.90544	-0.73573979	1.11551064	0.196791
1589.77556	1.39956489	1.11539753	0.197211
1722.82743	-1.65516841	0.88758561	0.197372
1639.75469	-0.67633859	0.88760145	0.197438
2259.0073	1.1164355	0.88772746	0.197968
2764.37026	-1.08101881	1.11513839	0.198175

Information Classification: General

1714.7719	0.51078787	0.88782514	0.198379
1738.82146	-1.5853085	0.88782522	0.198379
2267.10459	2.82081164	1.11502406	0.198602
2177.1292	1.64284663	1.11494166	0.198909
2604.23691	0.29309689	1.11491005	0.199028
1105.46147	-0.33101108	1.1148597	0.199216
1562.62615	0.24313657	0.88802492	0.199221
1850.93186	-0.41299045	0.88806862	0.199406
2888.48124	-9.98413125	0.88810244	0.199549
2550.25185	1.52395169	0.88814356	0.199723
1594.78489	0.20437024	1.11471877	0.199744
3307.61679	1.42462279	0.88815463	0.19977
3046.45107	-1.31556007	1.11469939	0.199817
2557.22348	1.36380703	1.11469292	0.199841
1454.60002	-2.03637788	0.88817217	0.199844
2406.22739	1.53011697	0.88817795	0.199868
968.545763	3.85591567	0.88820232	0.199971
2504.20131	2.39571444	0.88824192	0.200139
3734.63523	2.56376402	0.88826607	0.200241
2698.30936	1.68587942	1.11456683	0.200314
2661.40932	-0.73977206	1.11448171	0.200635
2235.09575	-3.116008	1.11447299	0.200667
1301.63762	0.33460998	0.8885667	0.201518
945.46715	-0.45020122	1.11416576	0.201826
1795.8477	0.07937686	0.88868427	0.202019
1804.84421	-0.78505477	0.88869385	0.20206
4029.79963	0.52001135	1.11402811	0.202347
2012.7968	2.76591678	0.88878379	0.202444
2447.16362	-2.77442932	1.11395509	0.202624
2015.96916	-2.55038143	1.11393318	0.202707
2883.30484	-3.05710905	0.88895786	0.203188
3683.58689	-1.81429691	0.88896046	0.203199
1976.87981	-0.64392335	0.8889742	0.203258
1894.96352	1.7749062	1.11375867	0.20337
2054.08217	1.91751153	1.11360418	0.203958
3371.54477	6.74279104	1.11354234	0.204194
1044.52178	0.99645062	1.11353199	0.204233
3374.60923	0.76860659	1.11326178	0.205267
1347.54546	0.53612663	0.88945575	0.205327
2734.21396	-3.04728347	1.11322207	0.205419
3324.61138	-0.92749976	1.11313854	0.205739
2280.96367	0.45390711	0.88958227	0.205872
2192.07309	0.60578928	0.88962213	0.206045
2601.26255	2.26084806	1.11304785	0.206087
1320.71965	-0.81486564	0.88973709	0.206542
2858.26455	0.71049166	0.88975083	0.206601
2292.03179	-0.06126442	1.11290289	0.206645
2220.06919	2.89976745	0.889803	0.206827
1583.75591	0.73980703	0.88980448	0.206833
3339.33908	-0.37158691	1.1127665	0.207171
2460.09446	-2.29087177	0.88988462	0.207181
1168.59636	0.62106587	0.88992705	0.207365
2239.10648	-0.28977414	0.88994944	0.207462
2834.35599	-0.98333322	1.1126675	0.207553
4028.7938	2.52781847	1.11257468	0.207912

Information Classification: General

1303.65213	1.82982995	1.11257334	0.207917
3555.81443	-1.20986567	0.89012151	0.20821
1467.7741	0.78360172	0.89013871	0.208285
2463.29922	5.49009304	0.89026783	0.208847
1321.57012	0.46963692	1.11232159	0.208893
3539.79751	-2.11685626	1.11229374	0.209001
1368.76068	-0.41388747	0.89033709	0.209149
3829.69692	-6.11228003	1.11225515	0.209151
1773.8582	-0.14454615	0.8903733	0.209307
2348.04361	-1.67162402	0.89038624	0.209364
3123.40776	3.13860477	1.1121841	0.209427
2072.01763	-5.53691055	1.11218042	0.209441
2669.18608	0.06910534	1.11212599	0.209653
1943.86357	0.94537981	0.89055047	0.210082
1524.86075	-1.0619817	0.89055481	0.210101
1444.64665	1.32609871	0.89056732	0.210156
3700.66421	-1.19925059	1.11181519	0.210866
2066.94267	1.26237264	0.8908369	0.211338
2619.02641	1.51945251	0.89084958	0.211394
4125.70237	2.18081541	0.8908499	0.211395
2417.14725	-0.33690574	0.89090377	0.211632
2626.16069	0.69677001	1.11158183	0.21178
2817.38633	-2.78614391	1.11154408	0.211928
1801.79729	-6.82068421	0.89103155	0.212195
2551.17899	0.78392401	1.11146415	0.212242
1232.61992	-0.09545065	0.8910568	0.212306
2996.48087	-2.74795237	1.11144114	0.212333
2730.2739	-5.77722315	1.11139463	0.212516
3369.58115	0.32751103	0.89111153	0.212547
2175.05362	-0.2828806	0.89120117	0.212943
1341.56157	-0.8335538	0.8912078	0.212972
1375.69544	-1.41026485	0.89121817	0.213018
3355.6102	-3.78342235	1.11122223	0.213195
2546.0806	-4.94161159	0.89129389	0.213353
2513.16373	0.20497726	1.11115822	0.213448
2798.12345	-0.77482704	0.89133796	0.213548
3684.61448	-0.12924817	1.11104019	0.213914
1669.80678	0.05036586	0.89143344	0.213971
3323.36863	-1.67957265	1.11095611	0.214247
2208.0689	0.58284187	0.89151104	0.214315
2516.31296	0.27424984	1.11089928	0.214472
1898.76395	-1.12659187	1.11088424	0.214532
2031.85515	-0.08866881	0.89157239	0.214587
3364.53843	-3.8219312	0.89159031	0.214667
1842.95578	-2.58532298	1.11079026	0.214904
2188.98491	-0.04450465	1.11074173	0.215097
1282.70452	0.29630533	0.89171517	0.215222
2368.00046	0.08070034	1.11067295	0.21537
1211.54514	-0.37734227	0.89175338	0.215392
1474.65752	1.50543557	0.8917743	0.215485
3367.59812	0.47284396	1.11061117	0.215616
2077.91435	-1.53377863	0.89180887	0.215639
1954.84807	0.87155606	1.11059984	0.215661
1253.63274	0.91392671	0.89189383	0.216017
2805.26731	1.12103455	0.89189514	0.216023

Information Classification: General

2712.40848	-0.56292481	1.11048716	0.21611
2409.27774	1.46758226	1.11048361	0.216124
1906.86846	0.2362522	0.89196099	0.216317
1750.68413	2.16677008	0.89197736	0.21639
2520.98233	0.24183312	0.89205139	0.216721
2490.20024	6.69328151	1.11032438	0.216759
3869.69236	1.8083168	0.89208364	0.216865
1981.92168	-0.99743693	0.89210074	0.216941
2018.81182	3.00665809	1.11027169	0.21697
1482.73711	3.299874	0.89217542	0.217276
1995.03092	1.81282474	1.11019174	0.21729
2778.45571	3.44215762	0.89229371	0.217806
2458.19536	-6.19893825	0.8923344	0.217988
1794.83122	0.54381523	1.10999319	0.218086
2121.04465	2.40709292	0.89246295	0.218565
1839.85979	-0.48182493	1.10978272	0.218932
1031.42436	0.61439794	1.10974444	0.219086
2882.44077	1.55622127	1.10972914	0.219147
2722.22678	-0.1074643	0.89265127	0.219413
3020.36332	0.97011586	0.89271117	0.219683
1680.84226	-0.976907	1.10955058	0.219868
2081.99283	-3.21148853	0.89276438	0.219923
2583.1852	1.07234665	0.89291947	0.220623
1304.64457	-2.69030949	1.10931974	0.220802
2817.2446	0.29132133	1.10927593	0.220979
1155.58953	-0.50464475	1.1092367	0.221138
1599.77239	-2.0414367	1.10918751	0.221338
1793.78301	-0.59077206	1.10912839	0.221578
1575.7719	1.88662121	0.89314873	0.221662
3192.55588	0.01002968	1.10902135	0.222013
2588.33779	1.69914477	1.10898286	0.22217
1556.80327	-0.23641073	0.89336158	0.222629
2638.1561	-4.58739084	0.89339269	0.22277
2503.08689	-1.71113529	0.89348025	0.223169
2315.06284	-2.36234273	1.10872258	0.223232
1407.63078	0.00682241	1.10869772	0.223333
2267.06484	2.64868555	0.89352699	0.223382
1498.73992	0.41198797	1.10865963	0.223489
1682.91318	0.63153882	1.10858813	0.223781
3743.76926	1.13840525	1.10850732	0.224113
2246.89157	-1.80721685	1.10844319	0.224375
3544.62192	7.37992936	1.10841455	0.224493
1587.80242	0.11176631	0.89377724	0.224525
2933.36515	-6.53033019	1.10840608	0.224528
2997.42514	-0.56991728	0.89395747	0.225351
2954.36699	1.94987899	1.1081827	0.225446
3283.59372	-0.2095822	1.10816308	0.225527
1134.5772	-2.35529478	0.89405574	0.225802
2003.02593	2.86476821	1.10806831	0.225917
2566.29399	0.95920859	1.10804328	0.226021
2556.32901	0.27083545	1.10796802	0.226331
1552.71636	1.84057603	0.89429303	0.226893
2333.09551	-0.86825287	0.89429936	0.226922
2596.09795	-5.49760098	0.89438192	0.227303
1844.75333	0.17929269	0.89439875	0.227381

Information Classification: General

1564.59331	-1.42272275	0.89444853	0.22761
2998.27494	1.02665571	1.10763864	0.227695
1555.75249	1.89664167	0.89450836	0.227887
2122.91104	-0.0213883	1.10751948	0.228189
4690.06557	-2.0700854	1.10751249	0.228218
3303.55222	1.89127052	0.89458497	0.228241
1428.65544	3.93713305	0.89460169	0.228318
1584.80729	0.84731369	0.89461083	0.22836
2396.1315	-3.88101449	0.89462146	0.22841
3470.56528	0.78184219	1.10736478	0.228833
1427.47002	1.51526957	1.10735287	0.228882
1714.93631	0.60768975	1.10732896	0.228982
1590.87383	0.05757567	1.10732597	0.228994
1695.93877	-1.13978061	1.10730811	0.229069
2872.3989	-1.43343655	1.10720087	0.229516
1360.67127	0.03673422	1.10716604	0.229661
1097.58513	0.45764702	1.10716306	0.229674
2504.30723	8.56780601	1.10715307	0.229716
2294.05303	-0.05631327	0.89491089	0.229751
4418.90005	-1.04238158	0.8949133	0.229763
1818.98929	-2.72477423	1.10714056	0.229768
1634.70452	-0.75911445	0.89498831	0.230111
3476.50083	2.9838363	0.89502081	0.230262
1998.02849	3.1539498	1.10699145	0.230391
2235.99277	-2.07260565	0.89505345	0.230414
4604.2822	-1.09802573	1.10698436	0.230421
3212.43674	3.61887136	1.10691904	0.230695
3173.31955	-2.7009901	1.10690951	0.230735
3729.73496	0.66309504	0.89512983	0.23077
3289.46885	-3.64347956	0.89515815	0.230902
4367.11538	-1.46621928	1.10684548	0.231003
2744.44242	1.77400874	0.89519623	0.231079
1754.78276	-1.25192562	0.89525877	0.231371
3686.80825	2.14140375	0.89527173	0.231432
3150.60276	2.83248032	1.1067213	0.231524
2113.85849	-0.60679322	1.10672109	0.231525
2541.39631	0.70285341	0.89536585	0.231871
2739.4354	3.20453692	1.10661853	0.231956
1582.85674	0.07312846	1.10659907	0.232038
3780.78293	0.17901096	0.89542988	0.232171
3679.73655	-0.08232035	1.106527	0.232342
1434.85686	-0.49424987	0.89548115	0.23241
2029.96465	-1.754263	0.8954911	0.232457
1901.85466	0.84683472	1.10646761	0.232592
3597.79135	2.42750406	0.89553516	0.232663
2734.10447	-1.73109527	0.89553687	0.232671
2321.06156	1.63687595	0.89555616	0.232762
1688.88626	1.79604818	1.10633963	0.233132
3153.30337	-0.01930277	0.89570354	0.233453
1516.72666	1.27866646	0.89574394	0.233642
1429.67021	-0.00327742	1.10615818	0.233899
3023.33475	-0.90573853	1.10610036	0.234144
2845.21836	0.11549421	1.10609912	0.23415
2489.11979	-0.51711898	0.8960424	0.235047
967.473314	0.2572684	1.10585343	0.235193

Information Classification: General

3077.32431	-3.22402239	1.10583579	0.235268
1761.77898	0.39730455	0.89614415	0.235527
2060.02256	-0.32144066	0.89617943	0.235693
1270.55803	-0.43626721	0.89620658	0.235822
2651.2562	-0.64633394	1.10566957	0.235975
1519.69328	1.45397987	0.89632703	0.236391
1213.6259	3.15895609	1.10555977	0.236444
3155.52317	2.17940931	0.89634406	0.236472
2735.27689	1.06618862	1.10542275	0.237029
1802.74382	-0.67856962	1.10535692	0.237311
3785.69712	1.05195206	0.89656857	0.237536
2393.25693	-1.9267363	0.89657037	0.237545
3211.38425	1.06843675	1.10522944	0.237857
2789.14157	1.74583454	1.10518397	0.238052
2049.03774	-0.0541071	0.89678474	0.238564
1513.65068	2.09894159	0.89682827	0.238771
1791.88567	-1.35643926	0.89685445	0.238896
1881.92974	-0.69248135	0.89686122	0.238928
1944.02085	0.71956051	0.89686262	0.238935
1646.77922	6.19214968	0.89689216	0.239075
3395.77256	0.38424982	1.1049021	0.239264
1778.86699	-0.39763618	0.89696138	0.239406
3223.48899	3.71165812	0.89704346	0.239797
1885.8831	0.3955385	1.10463255	0.240427
1962.03	0.47252797	1.10459335	0.240597
1677.83237	-1.17098818	1.10456333	0.240727
1295.61162	0.19648201	1.1044853	0.241064
2041.04033	1.73906504	1.10445507	0.241195
2982.44089	-3.84155612	1.1044195	0.24135
2476.08817	-1.2226471	0.89737064	0.241364
1273.62761	-0.26477495	1.10428454	0.241935
2735.19083	-1.47311448	0.8975403	0.242178
1789.81597	-2.70571533	1.10418063	0.242387
2637.22556	-2.88456377	0.89762689	0.242595
2796.31377	2.50687242	1.10407219	0.242859
1785.87705	0.25366388	0.89779171	0.243389
1284.57073	0.3744517	1.10379556	0.244066
2050.93828	-0.17517805	0.89793603	0.244085
2432.19815	0.64343834	1.10376828	0.244186
2139.97344	-1.15890662	0.89798369	0.244316
1575.72466	-2.45495064	0.89799221	0.244357
1722.79436	0.29070341	0.89799299	0.244361
2349.14018	2.09098526	1.10369042	0.244526
2313.16367	-2.32966384	1.10367569	0.244591
2462.97837	-1.94464888	1.10367303	0.244602
2756.3124	0.83348158	0.89804436	0.244609
2663.24126	2.03858221	1.1036328	0.244779
1899.98325	2.26748545	1.10361998	0.244835
1907.93222	1.71693402	1.10360239	0.244912
1272.57207	1.45672153	1.10358258	0.244999
3524.67405	-2.25351822	1.10353811	0.245194
2329.01932	1.46368975	0.89816758	0.245206
1679.72832	0.04987897	1.10353182	0.245222
3557.59934	-8.05541763	0.89817361	0.245235
2647.34818	2.50340459	0.89827003	0.245703

Information Classification: General

1995.91911	2.47990088	1.10332221	0.246143
2656.44643	0.71055028	0.89840379	0.246352
2234.99077	0.12615488	0.8984237	0.246449
2554.36173	0.1934684	1.10324025	0.246503
1541.70024	-0.67223752	0.89843595	0.246509
1198.63408	1.48711096	1.10322369	0.246576
1694.86565	0.66665148	0.8984691	0.24667
2580.29349	1.26182684	0.89847263	0.246687
3531.70131	-0.00131629	1.1031746	0.246793
1256.53215	2.35313592	0.89854544	0.247041
1297.63982	0.48534775	0.89855409	0.247083
2210.04581	-12.4693254	1.10303061	0.247428
1514.68682	0.14318265	1.10293041	0.247871
2640.42075	-0.70398463	1.10282452	0.24834
1110.50591	-0.23262033	0.89881536	0.248358
1566.7581	0.02804477	0.89888655	0.248706
1366.62659	-0.54495586	1.1027385	0.248721
1593.80181	1.83655913	0.89894839	0.249008
1311.64031	-1.79844442	0.89903227	0.249419
2386.03122	-1.26158568	0.89904579	0.249485
2253.20464	1.05589278	0.89906113	0.249561
1097.54753	0.20741727	0.89907536	0.24963
1624.73528	0.97356841	0.89908908	0.249698
1244.60515	0.53819303	0.89914158	0.249955
1498.7874	-0.31371069	0.89923677	0.250422
2902.43497	2.74093162	1.10234442	0.250474
3666.77011	-0.26360563	0.89926125	0.250543
2900.30875	-3.55421723	1.10230689	0.250641
2996.40488	2.41524228	0.89928937	0.250681
2230.00302	1.21370912	1.10226268	0.250838
1864.92405	0.48907236	1.10223568	0.250959
2155.12333	-6.46187245	1.10221381	0.251056
2335.1475	0.5553353	0.89943542	0.251399
3116.42685	-3.65748998	0.89944045	0.251424
2561.31131	0.05003266	1.10208827	0.251618
1291.61955	2.41490203	0.89955543	0.251991
2052.00125	0.05533889	1.10197908	0.252106
2634.24277	-1.39863011	0.89960451	0.252233
1046.47893	0.05173032	1.10194789	0.252246
2323.14347	4.41017513	0.89963473	0.252382
2152.20293	0.77277012	1.10182063	0.252817
1816.76042	0.51645517	1.1017384	0.253187
1560.74346	1.56175691	0.89980723	0.253235
2305.03911	-3.68679265	0.89982557	0.253325
2068.05063	2.91965621	0.89985142	0.253453
2964.35392	2.49554827	1.10167501	0.253472
2635.17002	3.00768544	0.89993353	0.25386
4602.78696	11.4522185	0.89997531	0.254067
1386.68913	-0.59190425	1.10152376	0.254153
3280.60691	1.73395079	0.90002462	0.254311
2428.10576	-0.9937008	1.10141519	0.254642
1777.63127	-2.2975042	0.90013228	0.254846
1771.88303	0.00082275	1.10136086	0.254888
2394.16971	-0.60288679	0.90014451	0.254907
4912.40005	0.89504518	0.90018799	0.255123

3192.54746	4.88110794	1.10124363	0.255418
1985.05057	-1.28663638	1.10124221	0.255424
1195.58367	-0.35090575	1.10124033	0.255433
2302.1323	2.22056567	0.90027056	0.255533
2940.23215	-10.4795971	0.90037717	0.256064
2876.24961	0.9180727	0.90038949	0.256126
1502.66081	1.25903351	1.10105874	0.256255
1387.6707	1.66004487	1.10101909	0.256435
2404.99527	-7.32595612	1.10101135	0.25647
1658.70403	0.76070891	0.90047382	0.256546
1926.97917	5.01732216	0.9004767	0.25656
2627.16245	-0.7828206	0.90051113	0.256732
1319.64812	0.28309654	0.90054625	0.256908
1560.70574	1.5555625	0.90054946	0.256924
2431.00229	0.29911659	0.90057315	0.257042
1684.89495	-4.01226221	0.90062263	0.257289
2823.20066	-3.8674506	1.10082804	0.257303
2998.55545	0.81231588	1.10082532	0.257315
1634.73186	-0.46256994	0.90064094	0.257381
1965.99187	1.68901401	1.10073698	0.257717
2911.39634	-4.26801325	1.1007324	0.257738
1585.80155	3.19051537	1.10071493	0.257818
3476.59458	2.0679171	1.10071097	0.257836
1427.70452	1.62293539	1.10070778	0.25785
1723.81401	0.41021972	0.90076454	0.257999
1624.82865	-0.15870375	1.10066111	0.258063
1454.62688	-0.87077077	1.10061942	0.258253
2460.11167	-0.22431782	0.90085943	0.258474
2005.91973	-1.11318855	0.90086152	0.258485
945.398485	0.31182486	0.90090082	0.258682
1909.94345	-4.0125468	0.90092544	0.258805
2253.1666	-2.88003837	1.10035301	0.259471
2304.01078	-0.14017302	0.90109624	0.259663
2664.11423	3.39273353	0.90111598	0.259762
3502.582	2.18697726	0.90114147	0.25989
1627.85232	-0.38435439	0.90114334	0.2599
2661.30741	0.70112127	1.10020058	0.260169
2137.01053	-1.32245524	0.90134896	0.260935
3400.53843	-4.40313636	0.90135236	0.260953
2535.0734	0.4181773	1.10002946	0.260955
2615.45908	2.15956829	0.90135724	0.260977
1325.57805	-0.20728433	0.90137149	0.261049
3072.42441	2.03952739	0.90137412	0.261062
2716.26542	-2.53078666	1.10000316	0.261076
1961.75017	-0.88983346	1.09998659	0.261152
2806.39725	-5.41632186	1.09994829	0.261328
2413.04204	2.62178866	0.9014616	0.261504
2784.36911	0.15028259	1.09988552	0.261617
1286.61333	1.05746514	1.09974407	0.262269
3003.42006	-0.40342301	1.09971863	0.262387
3136.52011	1.52443427	1.0996412	0.262744
1414.62151	0.25537551	0.90172157	0.262819
1261.56999	1.46530737	1.09960789	0.262898
2461.21616	-0.18026022	0.90186017	0.263521
2296.04825	0.46149979	0.90186012	0.263521

Information Classification: General

1581.75547	0.70237232	1.09944861	0.263635
1137.54143	-0.68205455	0.90192853	0.263868
2426.11455	2.48061324	1.09937926	0.263956
2424.10398	-3.82462	0.90199301	0.264196
1729.82352	-0.21089986	0.9020119	0.264292
3257.53489	-1.14812997	1.09930135	0.264318
2053.00595	-1.66279961	0.90204353	0.264453
2668.17551	-0.10309421	1.09922816	0.264658
3977.77805	3.58692438	1.09922619	0.264667
1104.52043	-0.27338477	0.90221565	0.265329
2017.75054	0.67926137	0.90225806	0.265545
2605.04258	2.27495562	0.90226877	0.2656
2222.21939	0.64764258	0.90228692	0.265692
1230.54924	0.67176075	0.9023293	0.265909
1374.65776	-0.09155552	0.90237346	0.266134
986.54973	-2.89705017	1.09889066	0.266229
1794.96819	2.54709093	1.09882492	0.266536
2549.06157	-3.27346497	1.09874527	0.266908
2909.19889	-0.11570491	1.09874355	0.266916
2210.00102	-0.91182707	1.09872052	0.267024
1392.70317	1.23091466	0.90264975	0.267547
2140.98564	-1.13592454	1.09860702	0.267555
1296.54717	0.34741457	0.90269902	0.267799
1872.76286	-0.38089096	0.90271613	0.267887
2065.87309	1.00595422	0.90273601	0.267989
3704.86228	0.83120625	1.09850477	0.268034
3888.89272	1.87472419	0.90290148	0.268839
3011.19632	-0.34274921	1.09832289	0.268888
4317.73696	-0.05190624	0.90293017	0.268986
2538.1413	1.36097476	0.9029522	0.269099
2753.2974	-0.03554516	1.09827223	0.269126
1083.56064	-1.67617522	0.90301105	0.269402
1338.60613	0.12804387	1.0982044	0.269445
2519.30254	0.76312118	1.09813623	0.269766
3345.50754	0.5981683	0.90311766	0.269951
1418.66741	1.09306606	0.90316802	0.27021
2696.26504	-0.90864421	0.90318036	0.270274
1752.82378	3.6300098	0.90326356	0.270703
1268.59905	-1.11042005	0.90328851	0.270832
1695.93596	-2.79527967	1.09790824	0.270842
2563.33183	1.95425659	1.09789357	0.270911
1275.69512	-0.08806326	0.90332296	0.27101
3061.52195	0.63709275	0.90336233	0.271213
4021.94895	7.43978279	1.09781489	0.271283
2880.33176	0.55164707	1.09779659	0.27137
2507.12198	2.27794841	1.09776622	0.271513
1414.7437	0.9363127	1.09775308	0.271576
1373.75232	-1.58349454	1.09775178	0.271582
3231.41782	3.37359558	1.09775158	0.271583
1502.73381	-0.26355487	1.09771863	0.271739
1502.73723	2.01094102	1.09770398	0.271808
2028.8438	6.59897956	0.90364155	0.272658
1216.59977	0.60849729	0.90364769	0.27269
1598.74199	-2.15628406	1.09751336	0.272712
3350.65395	0.62676422	1.09744058	0.273058

Information Classification: General

1891.86467	-2.51480874	0.90374507	0.273195
3579.72059	5.76665516	1.09739957	0.273253
2188.09439	0.61981051	1.09737605	0.273365
2531.34941	4.90154898	1.09731837	0.27364
1965.91239	1.73576808	1.09728776	0.273785
3538.60215	1.16449055	1.09727615	0.273841
1941.89536	0.00131798	0.90388765	0.273936
1825.97014	-0.13577868	1.09721289	0.274142
1593.67839	-1.15015344	1.09720342	0.274187
2238.18381	1.3371144	0.90398556	0.274445
1784.93938	-0.77461822	1.09710962	0.274635
2844.23893	-1.85411501	1.09710426	0.27466
2126.08463	-1.41876812	0.90404367	0.274748
4538.2926	1.0805941	1.09707591	0.274796
1798.80937	0.2215191	1.0969295	0.275496
2436.19077	-2.83240724	0.90420107	0.275569
2174.05899	-1.44622879	0.9042158	0.275646
3720.69394	0.19446473	0.9042419	0.275782
3240.61484	-7.54085874	0.90430467	0.27611
2258.04361	3.22622374	1.09672815	0.27646
2248.87627	-0.58562498	1.09665301	0.276821
2361.04277	3.01600132	1.09648675	0.27762
1934.89556	-0.05989406	1.0964309	0.277889
1106.55571	0.61123109	1.09642927	0.277897
2096.87993	0.57757778	1.09641858	0.277948
1526.8499	4.26552366	1.09639315	0.278071
1847.91399	-1.11870551	1.09632557	0.278397
2466.06974	0.40185432	1.09630797	0.278482
1311.56792	-1.51468138	0.90477995	0.2786
2109.02651	0.85568566	0.90479913	0.278701
3241.36045	2.04310817	1.09621023	0.278953
3045.22025	0.28329514	1.09620501	0.278978
2969.27854	2.72432182	1.09616707	0.279162
3258.46946	2.69060035	1.09615141	0.279237
3004.36905	1.18407791	0.90491965	0.279335
1769.71441	-1.48137752	1.09604401	0.279757
3483.82192	2.6068554	0.90500325	0.279775
3396.52475	0.18551039	0.90503621	0.279948
1396.69658	0.82731059	1.09593265	0.280296
2121.87529	1.02100222	1.09591473	0.280383
2937.29239	-0.02306519	1.09587954	0.280553
2818.21048	1.19404534	1.09585799	0.280658
2372.1315	-2.1041924	1.09584814	0.280706
3834.04319	0.4133255	1.09583439	0.280772
1087.49126	0.99608732	0.90521426	0.280888
1004.39519	0.98955105	0.90525538	0.281105
1187.56316	1.6240402	0.90525871	0.281123
2263.27016	-8.76563162	1.09576125	0.281127
2506.11966	0.44082436	0.90534437	0.281575
2696.37465	-1.85686547	1.09566851	0.281578
2921.19028	-0.29699045	1.09562046	0.281812
2811.26738	1.48910885	1.09561482	0.281839
2016.98581	-0.72996996	1.09556697	0.282072
2963.54697	-3.78199253	1.09554053	0.282201
1623.71208	-5.22849372	0.90547562	0.28227

Information Classification: General

1661.6613	1.07313179	1.09542975	0.282741
1387.63896	1.00048673	1.09541549	0.28281
1926.94155	-0.54971763	0.90564538	0.28317
1477.81235	-0.50564818	1.09530606	0.283344
1810.81876	-1.63216871	1.09530035	0.283372
2557.19748	0.96769328	1.09527536	0.283494
4314.66867	1.35885036	0.90573385	0.28364
1503.71831	0.05387811	1.09521903	0.28377
2533.2852	2.09946702	0.90580524	0.28402
1715.89688	-0.18856711	1.09515733	0.284072
1161.58159	1.77539616	1.0951131	0.284288
1879.89507	0.77176116	1.09510367	0.284334
1544.83025	-1.67041391	0.90589198	0.284481
2433.08567	0.12131778	1.09502444	0.284722
3174.44162	-4.17799215	1.09497544	0.284963
2616.2631	1.59172668	0.90598614	0.284982
1530.6906	-1.40390451	0.90598648	0.284984
1326.6668	0.79699411	0.90602133	0.28517
2001.89653	-2.81599281	0.90602158	0.285171
2934.39621	19.3893556	1.09484801	0.285588
1663.81365	-0.36787079	0.90610239	0.285602
3190.59885	-1.14343102	1.0947821	0.285912
1834.71282	1.68465245	0.90618605	0.286049
2599.12552	-3.9462759	0.90620473	0.286149
2012.96921	-0.33974765	0.9062062	0.286157
2642.1652	-7.95673748	0.9062228	0.286245
1648.71758	-0.5085191	0.90624091	0.286342
1624.73857	4.55684964	0.90629941	0.286655
1334.65495	0.10454036	0.90630926	0.286707
2780.33548	0.0942588	0.90635253	0.286939
1384.65849	-0.39030247	0.9063628	0.286994
1280.56438	0.22908137	0.9063765	0.287067
2072.04288	1.05959778	0.90640632	0.287227
1439.62908	0.41720404	1.0944387	0.287604
1958.98857	-0.30950187	0.9065128	0.287797
1886.84709	1.83067149	0.90656316	0.288067
2439.06182	-2.61991028	0.90659553	0.288241
3920.84397	3.32631391	1.09429752	0.288302
3914.02951	5.51396783	0.90666323	0.288605
3983.92636	-0.58223497	1.09407723	0.289393
1686.76689	-0.63553425	0.90681399	0.289415
1588.78983	0.12322223	1.09406608	0.289448
2453.17307	4.4026736	0.90688503	0.289797
1715.88242	0.04318842	0.90695577	0.290178
987.514147	-0.38336682	0.90695618	0.29018
3860.68056	-0.61196513	1.09388931	0.290326
2442.06206	-2.75135616	0.90702729	0.290564
3003.42844	0.85722504	0.90702982	0.290577
1578.70464	0.4175211	1.09383141	0.290614
2723.29886	0.26547852	0.9070753	0.290823
1869.86626	-1.67218577	0.90717766	0.291375
1398.63713	-0.84563772	1.09367755	0.29138
4928.38697	-0.72907218	0.90721331	0.291568
3305.33127	1.82482324	1.09363971	0.291569
3157.52597	2.03823937	1.09361477	0.291694

Information Classification: General

2204.18113	-0.32793996	1.093613	0.291702
4570.02607	0.52690536	1.09358712	0.291831
1538.59343	-1.09704887	0.90726219	0.291832
1360.62358	-0.01142491	0.90728409	0.291951
2449.04643	1.9019719	1.09347126	0.29241
1353.69963	-1.59248053	1.09343491	0.292592
4241.2269	2.83185229	1.09341974	0.292668
2216.92032	-1.67667808	0.90743287	0.292756
1209.60051	0.6499385	1.09338531	0.29284
1415.75956	-3.89378497	0.90746699	0.292941
3643.74094	-1.25156701	0.90748848	0.293057
1903.92583	4.57136965	0.90748958	0.293063
1518.69914	-0.66769088	1.09332184	0.293158
1048.43913	2.02130266	1.09330821	0.293226
3479.63523	-0.73234014	0.90757776	0.293542
1719.84477	0.69707526	0.90760389	0.293683
2074.8897	-1.0825943	0.90761682	0.293753
2261.01798	-1.49924138	1.09318903	0.293823
1449.72978	1.96976492	1.09318526	0.293842
1094.46514	1.39811614	0.90764357	0.293899
2529.32297	0.99349276	1.0931717	0.29391
3177.37021	-3.4292274	1.09312401	0.294149
1629.66655	2.59329431	0.90776039	0.294533
2363.03252	2.32032434	0.90789851	0.295285
2336.11795	12.4517575	0.90795154	0.295574
1097.51201	1.00699524	0.90804322	0.296074
1591.63799	-0.90991051	0.90811444	0.296463
1632.77396	0.93376893	1.09265692	0.2965
1684.85483	2.28103665	1.09265468	0.296512
2599.26113	3.08426279	0.90815503	0.296684
3651.73752	1.61584596	0.9081634	0.29673
2414.15141	4.40589028	0.90824613	0.297182
1765.87615	0.3346393	1.09250926	0.297247
1547.82126	1.73397262	1.09247765	0.297406
1491.62798	0.82325236	1.0924398	0.297598
3163.43991	-0.51550736	1.09241662	0.297715
2930.28019	4.06692151	0.90834777	0.297738
3956.88755	-1.70561851	1.09239962	0.297801
1591.68583	-0.77284852	0.90836859	0.297852
2467.14219	1.34517227	1.09238202	0.297891
1312.5988	-2.07437218	1.09238119	0.297895
1293.61931	0.87737873	0.90837875	0.297908
2511.26694	-1.40184594	0.90838625	0.297949
2089.09513	-0.24621867	0.90840245	0.298037
4423.20522	0.6394913	0.90840915	0.298074
3555.70815	1.19168632	1.09233792	0.298114
1910.86162	0.10182023	0.90854118	0.298798
1401.60295	0.97572011	0.90862858	0.299277
1311.65569	-4.27555255	0.90864563	0.299371
1434.64543	-10.693903	0.90865851	0.299442
2039.78899	0.51217476	0.90870487	0.299697
2301.97466	1.92516183	0.90872173	0.299789
3148.41867	3.19175755	1.09200374	0.299811
3398.47885	1.18189734	1.09198421	0.299911
1413.77666	1.47168003	0.90878498	0.300137

Information Classification: General

1293.54131	3.44949508	1.09192214	0.300227
2588.27616	0.98865	1.09191504	0.300263
3343.59592	5.36819737	0.90884194	0.30045
2301.18642	0.51311494	0.90889244	0.300728
3951.67014	-0.08102073	0.90893505	0.300963
1996.93679	1.1512553	0.90895487	0.301072
1593.79923	0.21971919	0.9089838	0.301232
1147.46807	1.2103839	0.90899829	0.301312
1433.627	-1.21898949	0.90900491	0.301348
1629.85636	-0.6098117	1.091694	0.30139
1168.57779	2.00640592	0.9090786	0.301755
2678.21592	-6.69206687	0.90910247	0.301886
1610.80669	2.30935455	0.90910994	0.301928
1374.56767	0.90883257	1.09157568	0.301995
1709.81621	1.3693917	0.90915005	0.302149
1028.48943	-0.01732486	1.09146127	0.302581
1833.95024	0.73644923	1.09143834	0.302698
1567.84575	0.01238881	1.09141612	0.302812
1434.70085	-0.23143305	1.09138256	0.302984
2197.98392	-0.64487683	0.90930419	0.303001
1099.48845	-1.56834839	0.90932819	0.303134
1763.83489	0.35386869	0.90938898	0.30347
1823.95085	0.60196952	1.09128504	0.303484
1311.61711	-0.29907475	0.90948821	0.30402
1196.52226	-0.99614057	0.90950866	0.304133
2081.8687	0.84282092	0.90952289	0.304212
1514.64226	-0.32732956	1.09113089	0.304277
3390.60569	-1.87172817	1.09112535	0.304305
2344.04605	0.786401	1.09112382	0.304313
1458.56316	-0.71806948	0.90956383	0.304439
986.430712	-0.54097946	0.90957195	0.304484
1575.80107	1.20680802	0.90957695	0.304512
3102.57163	3.01591446	1.09108385	0.304519
2689.10698	0.35546838	1.09107325	0.304573
3517.5875	0.28492006	0.90960641	0.304676
1258.50701	0.03202728	0.90964297	0.304879
1773.76067	-5.2890924	0.90964318	0.30488
3613.59519	1.26180734	0.90966807	0.305018
1901.89579	-1.37872672	1.0909259	0.305332
2072.03885	0.1212389	0.90973607	0.305396
1857.9014	1.46565358	1.0908639	0.305652
1961.98276	-1.8741739	0.90979587	0.305728
3732.92055	1.38434034	0.90980705	0.305791
2337.07475	2.88579986	1.09078951	0.306036
3408.59327	-1.80729152	1.09078177	0.306076
1808.91373	0.00284226	0.90985866	0.306078
3136.33261	-1.02335487	1.09075644	0.306207
3050.28073	-2.03930871	0.91001026	0.306922
1894.96562	4.49320585	1.09059914	0.30702
2186.89141	-0.17571946	0.91005738	0.307185
1643.8189	3.45351609	0.91008055	0.307314
1383.73918	-6.91240024	1.0904522	0.307782
2794.32419	0.92815544	1.09043834	0.307854
1446.64336	0.49414088	1.09043101	0.307892
1883.86357	0.48023083	1.09041352	0.307982

Information Classification: General

1497.75739	-1.28820017	1.09040829	0.308009
1986.92754	-0.79140625	1.09027578	0.308697
3314.62539	-2.3125941	0.91034385	0.308785
2031.92575	0.17671275	1.09022837	0.308944
2025.89499	-5.91671908	1.09022189	0.308978
3171.57053	0.10450905	0.91045403	0.309402
1558.76116	0.46042794	1.09004975	0.309874
1799.92705	-0.13097649	1.09000587	0.310102
2653.25956	-0.07638276	1.08997382	0.31027
2095.98076	-10.6178506	1.08996975	0.310291
2404.1691	-6.91289345	1.08993623	0.310466
1750.73918	-0.57801817	0.91065094	0.310507
2521.2493	-0.82521373	1.08992321	0.310534
3642.76023	-3.82025599	1.08991377	0.310583
2500.09732	-0.14683772	1.08986958	0.310813
1248.59001	0.40695429	1.08983905	0.310973
2465.89176	-3.2367661	1.08981839	0.311081
2092.06382	-1.94181905	1.08976248	0.311373
2226.96245	-1.12546015	0.91083723	0.311553
1841.89006	0.83281148	1.08962409	0.312097
1775.78777	1.17019914	0.91098644	0.312394
1420.69585	-0.37300032	1.08952227	0.312631
1510.69548	0.02440536	0.91106393	0.31283
1886.8571	0.35771803	0.91107368	0.312885
1125.57768	-0.80934662	1.08939001	0.313325
1966.9451	0.77287959	1.08934887	0.313541
1837.81963	-0.14017868	1.08917473	0.314457
2084.89482	-4.57208226	1.08911929	0.314749
2175.00455	0.6126212	1.08907995	0.314957
2779.32236	2.41624095	1.08907879	0.314963
1407.60271	0.33633891	0.91144434	0.31498
1141.59217	-0.80727542	1.08907257	0.314996
1932.75957	0.45571272	0.91159228	0.315818
1693.67339	-0.98121486	1.08880604	0.316403
2673.07853	-1.10619149	0.9116998	0.316428
1721.83281	5.29608366	0.91170023	0.316431
2291.11431	-2.47603916	0.91171491	0.316514
1960.8687	-1.41894126	1.08877438	0.31657
1691.73198	0.43440244	1.0887663	0.316613
1408.75847	2.30142383	0.91173802	0.316645
1544.75168	3.60821848	1.0887567	0.316664
2137.98337	-1.09624113	0.91174536	0.316687
3063.26584	-1.66523	1.08872912	0.31681
1512.79778	4.25621391	1.08871284	0.316896
1409.72698	-3.5765722	1.08871182	0.316902
1924.88774	-0.77687063	1.08869184	0.317007
2150.98239	-4.02621046	1.08863462	0.317311
1266.65788	0.23622228	0.91185502	0.317311
2233.93706	-4.72662489	0.91187202	0.317407
2017.89751	1.02593726	0.91189409	0.317533
3508.68418	1.4577883	1.08855147	0.317751
1186.61863	-3.18791479	0.9119375	0.31778
1810.83232	3.00286741	1.08853284	0.31785
2816.14725	-3.61605764	0.91197418	0.317989
2712.3434	-0.74356551	1.08849581	0.318047

Information Classification: General

2349.18912	-1.76411237	1.0884826	0.318117
2114.99419	-0.78018004	0.91200147	0.318144
2104.03415	-1.62711014	0.91202656	0.318287
1844.8615	-0.82960479	1.08833157	0.318919
3155.51474	-4.52055406	1.08822918	0.319464
1477.68665	1.49547442	1.08817297	0.319764
1930.93631	0.06481271	1.08814555	0.31991
1438.7332	-0.16431426	0.91231584	0.319938
1996.94573	3.6190316	0.91238439	0.32033
1129.50151	0.68804025	0.91241478	0.320503
2187.02495	1.81600237	1.08803425	0.320504
2307.2089	0.45126258	1.08801448	0.320609
1299.64348	0.63472479	1.08795833	0.320909
3047.39023	1.65276052	0.91258885	0.3215
2265.07598	0.69314811	1.08784215	0.32153
2102.00992	-0.53636871	0.91260384	0.321586
2095.02129	2.66275725	1.08777468	0.321891
2621.14176	7.80823284	1.08772617	0.322151
3256.50332	-0.0447399	1.08765478	0.322534
1053.55901	0.56879556	0.91280093	0.322717
1670.87491	1.34108305	1.0876113	0.322767
2411.00469	1.33884876	0.91291511	0.323373
2072.07866	-0.9977517	1.08745533	0.323604
1389.66033	-3.63569488	1.08743533	0.323712
1914.87566	-3.03797777	1.08743146	0.323732
2516.19204	0.48684976	0.91298939	0.323801
2211.92026	0.13648915	0.91299637	0.323841
2144.95694	-1.7100427	0.91300548	0.323893
3133.45786	2.69368849	1.08736429	0.324094
2993.25565	0.53088907	1.08733009	0.324278
3610.71567	-2.44077391	0.91308582	0.324356
3092.34868	3.48444253	0.91313131	0.324618
3851.74179	-3.16763023	1.08722943	0.32482
2532.30429	3.67495576	1.0872267	0.324834
2695.23301	-3.22938916	0.9131782	0.324889
3524.68686	-0.70801591	1.08719486	0.325006
2193.02812	3.5683403	1.08713637	0.325321
1063.45867	0.80911731	1.08710061	0.325514
2353.08194	-0.87330914	1.08707224	0.325668
2069.98662	-0.81084371	0.91331906	0.325702
1115.53545	-1.1450202	0.91334019	0.325824
2681.23972	-1.37961893	0.91334406	0.325846
2615.16135	0.11424546	0.9133797	0.326052
1447.7144	-2.18429304	0.9133915	0.32612
4231.85483	1.39589279	0.9134206	0.326288
3920.59641	-14.5007621	0.91344975	0.326457
3092.47133	0.2832306	1.08690498	0.326571
3062.46476	1.57635513	0.9135062	0.326784
1978.89054	-0.55496651	0.91351033	0.326807
2282.10935	2.00376778	1.08682228	0.327019
1055.53777	0.09381503	0.91362741	0.327485
1249.60718	1.05318628	0.91365327	0.327635
3100.37651	1.35923572	0.91367576	0.327766
2460.11147	6.17435487	0.91369514	0.327878
1812.82439	-0.13066954	1.08662521	0.328087

Information Classification: General

1599.69731	0.54067963	1.08661607	0.328137
1744.84245	2.08587618	1.08659634	0.328244
1956.82305	0.76519215	1.08652005	0.328658
3378.54306	-0.79482855	0.91385643	0.328814
2512.18779	-1.74611175	1.08648332	0.328858
2252.02788	3.44326554	0.91393754	0.329285
1167.53252	0.74773239	1.08638363	0.3294
2340.09087	2.63648362	0.91399845	0.32964
1979.86308	-0.16243137	1.08633909	0.329642
1767.8322	-0.02171074	1.08632391	0.329725
2284.29033	-0.75625156	0.91402867	0.329815
2975.32688	-1.3524181	0.91404345	0.329901
3447.57126	2.43820332	1.08628327	0.329946
3000.48832	-0.48288729	0.9141055	0.330263
2344.06546	-0.51084757	0.91412985	0.330404
2885.36936	1.08221251	0.91422188	0.330941
1548.70586	1.64643635	0.91423122	0.330995
3582.53757	-0.86657195	0.91426603	0.331198
2097.86804	2.98674864	0.91431669	0.331494
2010.89421	-0.49264759	0.91433985	0.331629
3115.54575	0.56207949	1.08596524	0.331682
1990.77434	1.86072869	0.91435642	0.331726
1560.79399	2.31139488	0.91435985	0.331746
3352.53733	-0.70265204	0.91437907	0.331858
1949.98771	2.02398752	1.08591235	0.331971
2831.37906	0.58175632	0.91441648	0.332076
1538.72954	-2.4341599	0.91441659	0.332077
1381.78227	1.12598148	0.91445317	0.332291
2270.07891	-0.15121914	1.08584889	0.332319
3606.70083	1.65832163	0.91447655	0.332427
1956.9749	4.48948751	1.08582463	0.332452
1495.69036	-0.47475339	0.91449925	0.33256
1972.74431	-0.56013642	0.91458481	0.33306
2781.38258	8.94746344	1.08571184	0.33307
3142.49118	2.88489423	1.08559619	0.333704
1741.83318	-1.65964139	0.91471266	0.333809
1098.58269	2.5677633	0.91471748	0.333837
1481.70122	0.4280038	0.91494701	0.335184
4236.86538	1.39391997	0.91496368	0.335282
2262.05436	1.94696533	0.9149984	0.335486
2292.96294	0.1146655	0.91500378	0.335517
1280.57695	1.28582326	1.08524763	0.335622
2408.10825	-4.18744283	1.08520993	0.33583
2066.98418	0.70158163	1.08519891	0.335891
1844.8228	2.3970893	0.91508077	0.33597
3034.60219	2.97872898	1.0851183	0.336336
1667.82475	0.18966621	0.91514493	0.336348
3207.37094	-0.09867993	0.91515659	0.336416
1593.69907	-2.2811729	0.91517213	0.336508
1918.90471	0.94605255	1.08507994	0.336548
3411.66758	-1.09701302	1.08506494	0.336631
2736.24643	0.86497258	0.91527679	0.337124
2982.29546	-0.6127981	0.91529247	0.337217
2620.30442	-0.05030844	0.91529499	0.337231
2974.31742	0.59800774	1.08490857	0.337495

Information Classification: General

2364.03019	2.95986895	0.91534072	0.337501
2027.10329	-0.91712627	0.91535074	0.33756
1661.79984	-1.68459527	1.08489193	0.337588
2233.0689	3.13020123	0.9153771	0.337716
2301.12197	4.73840512	1.08481322	0.338024
1121.51189	0.04468884	0.91543149	0.338037
2148.84307	2.48500087	0.9154426	0.338102
3309.67666	-3.48518997	1.08475187	0.338364
3231.68862	-0.74139607	1.08469747	0.338665
1783.7553	2.1910641	0.91554945	0.338733
2156.10367	2.70559701	1.08467068	0.338814
1930.04363	2.87682632	1.08450813	0.339717
4471.81606	-6.51157261	0.91574318	0.339879
2172.10747	-4.00616745	1.0844632	0.339967
1355.60723	-0.08849678	0.91575798	0.339967
2564.23313	2.64099001	1.08445775	0.339997
1974.84661	-6.62840548	0.91576362	0.34
1821.72966	0.53551116	0.91577919	0.340092
1855.79074	-1.78398262	0.91578423	0.340122
2865.15818	-0.38708432	1.08436435	0.340517
2517.12119	-0.83403314	1.08435155	0.340588
3608.5261	0.69858552	1.08434605	0.340619
1794.70989	1.3245405	0.91589265	0.340765
3811.54941	0.12837135	1.08423489	0.341239
3909.90322	-0.18142441	1.08421833	0.341331
2339.11521	0.0864396	1.08419742	0.341448
2752.32218	-2.16565961	1.08419156	0.341481
2010.01316	-0.3326292	1.08413255	0.34181
2980.37217	3.2218124	1.08405266	0.342256
1318.61345	0.31291889	0.91616615	0.342389
1877.70476	0.24722354	0.91618549	0.342504
1250.53594	0.15750897	0.91624163	0.342838
3822.68442	3.33798398	1.08391112	0.343048
1749.85503	-0.01156239	0.91628605	0.343102
2004.93608	3.86468929	0.91636255	0.343558
1259.66435	0.32225995	0.91637623	0.34364
2731.30313	-0.38353916	0.91638095	0.343668
2561.34795	14.3528681	1.0837544	0.343926
1882.93999	-0.12177499	1.08375217	0.343939
1528.66887	-0.49615654	1.08374222	0.343994
2698.00632	-0.86713161	0.91643947	0.344016
1473.66326	-0.82087508	1.08368812	0.344298
2132.83623	-1.01229555	1.08366814	0.34441
2105.00223	-0.93153442	1.08363325	0.344606
1668.73015	-0.4615259	1.08353748	0.345144
1646.87546	-0.17420963	0.91673084	0.345756
2068.9644	3.75780632	1.08331739	0.346383
2156.90581	1.78172708	1.08330619	0.346446
3691.73178	-2.73168799	1.08329282	0.346522
2264.00736	0.50047147	1.0832401	0.346819
3190.44358	3.84283016	1.08322987	0.346877
2661.2089	-1.81979061	1.08322933	0.34688
2528.18552	-0.62230711	1.0832286	0.346884
3693.52036	3.40043053	1.08319657	0.347065
1529.65605	-2.69462005	1.08318371	0.347137

Information Classification: General

1584.82248	-0.76691062	1.08317348	0.347195
2509.08257	2.50762174	1.08316705	0.347231
2445.13584	-1.11279094	1.08314323	0.347366
2680.22257	0.9764234	0.91705189	0.347679
1447.65557	0.61470207	0.91708539	0.34788
3502.81089	0.98717951	1.08302597	0.348028
1267.68962	-0.42242669	1.08300007	0.348175
2857.34507	-1.13126322	1.08299131	0.348224
2370.18253	-1.75473003	0.9172272	0.348732
2405.09909	-0.33058474	1.08286025	0.348966
1590.77092	-3.66280131	1.08283403	0.349115
1683.90569	0.82305511	0.91729829	0.349159
3119.4144	0.78204682	0.91733744	0.349395
1446.7487	0.85676615	1.08276436	0.34951
2464.19364	-3.97315855	1.0827061	0.34984
2306.06597	-0.83984263	0.91745945	0.350129
1989.95586	0.29791898	1.08264664	0.350178
1521.71099	-0.04153302	1.08257277	0.350598
1192.50139	0.82605538	0.91755086	0.35068
2352.89719	2.11382549	1.08254247	0.35077
1753.9467	0.2973706	1.08252618	0.350863
1182.67595	1.83703665	1.08251424	0.35093
2109.93389	-0.30987256	0.91760529	0.351008
2933.43528	-0.02912914	1.08250063	0.351008
2419.04038	-0.98341036	0.91761014	0.351037
2213.9108	0.75949487	0.91762055	0.3511
1082.46367	0.04189113	0.91770798	0.351628
3315.50132	0.35306595	0.91772668	0.351741
1808.88004	0.97338415	1.08236304	0.351791
4605.19077	-0.48451508	0.91774563	0.351855
3732.8819	2.52671593	0.91775622	0.351919
1290.65898	1.09079598	1.08231268	0.352078
1499.68315	-1.895222	0.91779628	0.352161
2396.0167	0.85452243	1.08228276	0.352249
2967.49834	4.7111448	1.08227595	0.352287
1877.96244	0.61673017	1.08223505	0.352521
1845.85881	-0.64634023	0.91786672	0.352587
1240.53301	0.19449956	0.91790276	0.352805
2359.04679	-0.32352812	1.0821538	0.352985
1375.67522	0.91823795	0.91793763	0.353016
988.437914	0.91913683	0.91793768	0.353016
2704.18155	-3.4829736	1.08211142	0.353227
1960.91484	-0.60011753	0.91797769	0.353258
2825.23935	3.98150337	1.08209402	0.353326
2847.49601	1.3041395	1.08208561	0.353374
2448.27811	1.73265619	1.08203722	0.353651
2854.41794	-2.01422249	1.08200544	0.353832
3717.6771	-2.54302865	0.918074	0.353842
2577.179	-0.7429143	1.08199562	0.353889
2665.20806	0.92192099	1.0819756	0.354003
2277.07964	1.20423654	0.91820408	0.35463
1922.96024	-0.21636928	1.08182543	0.354863
2247.04593	0.31921459	0.91828476	0.35512
2150.99394	-1.64817613	0.91834767	0.355502
1662.83379	-2.71251818	0.91837442	0.355665

Information Classification: General

1670.96428	-2.74290891	1.08168304	0.35568
3220.70844	0.04161138	1.08165502	0.355841
3629.59926	-2.77012124	1.08162231	0.356028
1736.93425	1.49481784	1.08159999	0.356157
3711.95302	0.72612331	0.9184647	0.356214
3051.49209	1.49691872	1.08148765	0.356803
1114.61308	1.71934831	0.91858706	0.356958
2714.3472	3.51238084	1.08144458	0.35705
2269.17339	1.95936658	1.08143379	0.357112
2041.05722	-0.34649016	0.91861576	0.357133
1908.89646	0.25574577	1.08141703	0.357209
2041.97466	1.65608018	0.91872446	0.357796
2440.12717	1.64953813	0.91872623	0.357806
1563.77524	-0.57785038	1.08127899	0.358004
3983.05808	3.5164654	1.08124498	0.3582
1491.73357	0.06020282	0.91883577	0.358475
1775.6978	1.49361282	1.08109246	0.359081
3556.68958	1.07857454	1.08103943	0.359387
1552.78459	-0.47128418	0.91899293	0.359435
4382.13361	0.56545893	1.08101923	0.359504
2603.11594	-3.31847914	0.91901004	0.359539
2732.34006	0.63048807	0.91901014	0.35954
2721.27317	0.41825149	1.08096408	0.359823
2847.25669	7.36522438	1.0809519	0.359894
3159.5372	-3.97581566	1.08093282	0.360004
2252.07451	-3.58683098	1.08093111	0.360014
3611.78481	2.56727504	1.08088895	0.360258
2953.39077	3.81746851	0.91920873	0.360755
2227.10039	0.21152869	1.08080091	0.360768
1843.81828	-0.49375612	0.91921634	0.360802
3131.14341	-0.03179849	1.0807715	0.360939
1819.86931	-0.85446022	0.91926674	0.361111
2858.44521	0.71571623	1.08071099	0.36129
2423.09269	4.25250738	0.91934094	0.361566
2269.82353	-1.89282253	0.91936156	0.361692
2209.93096	0.62920603	0.91944468	0.362203
2835.16349	-0.99641493	0.91948027	0.362421
1576.73052	-1.99713979	1.08048771	0.362587
1088.4738	-0.35170202	0.91954634	0.362827
1564.74797	-0.61217307	0.91955689	0.362892
2264.91977	-0.1176104	0.91960388	0.363181
2929.20737	4.12919831	0.91963242	0.363356
2069.96465	-0.25560431	1.08028968	0.36374
2647.42837	-1.89461878	0.91969962	0.36377
2962.54253	0.99332498	0.9197204	0.363898
1264.5977	-6.20074766	1.08023684	0.364048
2656.34543	-1.25593547	1.08017353	0.364417
1868.70696	0.00025603	0.9198726	0.364835
2180.12162	0.59073934	1.0800998	0.364848
1743.86661	0.55795597	0.91990649	0.365044
1696.82231	2.18934164	1.08006593	0.365046
1748.81266	2.3449754	0.9199478	0.365299
2381.93094	-0.04909652	1.08001172	0.365363
1370.71697	-0.10706509	1.08000321	0.365412
2075.9312	-2.93764914	0.91998223	0.365511

Information Classification: General

2154.90012	0.57882106	0.91999232	0.365574
2206.08597	-1.02926526	1.07984152	0.366359
1401.71198	-0.07602673	0.92013457	0.366452
2660.40342	0.47519109	1.07981997	0.366485
1975.01419	0.07899113	0.92016001	0.36661
1719.82303	0.40288984	1.07979554	0.366628
1383.60381	-0.74583173	0.92017995	0.366733
2014.85906	-1.46209343	1.07976936	0.366782
2731.14713	3.45993037	1.07973027	0.367011
1863.73479	-1.30892567	1.07972644	0.367033
1917.87493	-0.3351478	0.92023809	0.367092
1588.83208	0.80408041	1.07969034	0.367245
2375.98808	-10.4321196	0.9202673	0.367273
1345.60588	-0.21978227	0.92029393	0.367438
3075.50718	-6.44122522	0.92032422	0.367625
2281.06938	0.02772219	1.07960441	0.367749
1469.70598	0.99401918	0.92034886	0.367778
2621.14634	1.27162754	1.07959708	0.367793
2535.30278	-2.44677925	1.07957952	0.367896
3471.64743	-2.48995483	0.92037556	0.367943
2140.0894	-3.27800908	0.92038557	0.368005
2047.00078	0.97299456	0.92039408	0.368058
3176.32578	-13.592283	1.0795338	0.368164
2201.00956	-0.26926884	0.92049829	0.368704
2512.14072	-0.33243062	0.92050602	0.368752
2511.28672	-0.25724611	1.07943309	0.368757
1165.63323	-0.07891875	0.92052332	0.368859
1355.71721	-0.16420483	0.92053465	0.36893
2152.02976	-0.19053015	0.92053871	0.368955
1343.64787	2.38637462	0.92059759	0.36932
1945.83086	-1.18055193	1.07933522	0.369333
1511.65007	-0.3018471	1.07925287	0.369818
2311.15262	-3.19764993	1.0792476	0.369849
2132.02018	2.38433668	0.92071372	0.370041
2937.38065	9.10814427	1.07916185	0.370355
2509.1248	-1.92291825	0.92076496	0.37036
2176.00493	0.40216419	0.92077385	0.370415
1337.64287	1.02535916	1.07915033	0.370423
3136.36264	-4.35672973	0.92077619	0.37043
2026.0053	1.86392513	1.07914363	0.370462
2639.21152	-2.81422356	0.92082434	0.370729
2001.89873	-4.45380203	1.07909828	0.37073
2149.06327	2.93535994	0.9209061	0.371238
2004.96379	0.83164391	1.0790047	0.371283
1597.82004	7.71249342	1.07893207	0.371713
3731.69748	-0.12579187	0.92099897	0.371816
1889.81584	0.81552771	1.0789007	0.371898
2009.95548	1.024992	0.92107526	0.372292
2993.55301	-0.91484334	1.07882208	0.372364
2878.26432	0.62922606	1.07879409	0.372529
1733.83391	-0.08385841	1.07874776	0.372804
1538.69524	-1.08356192	1.07870278	0.373071
2993.3727	1.34031756	1.07865057	0.37338
1351.67302	-0.70584263	1.07861311	0.373603
2072.98247	6.37447443	0.92129623	0.373671

Information Classification: General

4190.07094	2.40216875	1.0785816	0.37379
2438.03995	-0.58126835	1.07857158	0.373849
1255.56389	1.25925267	1.07856692	0.373877
1562.64531	0.03581787	0.9213749	0.374163
1764.85819	1.72996858	1.07849105	0.374328
2715.20963	0.20019777	1.07848351	0.374373
3039.29496	-0.11689312	1.07843182	0.37468
1309.62981	1.41264419	0.92145939	0.374691
2863.24637	-2.80809123	0.92146073	0.374699
3787.77651	-0.92622578	1.07839169	0.374919
1164.55547	1.21868014	0.92155102	0.375265
1545.75473	-0.36515536	1.07830406	0.37544
1745.81522	1.31995597	1.07820268	0.376044
2671.21323	-2.54354344	1.07819728	0.376077
1973.90251	-3.96822076	0.92172427	0.37635
3094.48752	-0.33393403	1.07810464	0.376629
1291.63701	-1.15962195	0.92177417	0.376663
3318.60146	-2.3968423	1.07808696	0.376735
1805.92424	-2.05621323	1.07808423	0.376751
1951.89018	1.13173247	0.92180608	0.376864
1238.60869	2.10871835	1.07804428	0.37699
1886.77703	-0.20148761	0.92183374	0.377037
2663.19742	-0.2108189	1.07798003	0.377374
1386.72246	-0.57588443	1.07792771	0.377686
2820.21933	-0.12688065	0.92195059	0.377771
2225.13535	0.38954653	0.92198546	0.377991
1053.5584	0.88453391	1.0778751	0.378001
1795.88486	1.53991637	1.07784195	0.378199
4443.08266	-9.56385369	1.07775799	0.378702
2561.03244	-1.56127647	0.9221003	0.378713
1454.73088	1.74895558	1.07767916	0.379175
2025.95359	2.59290167	1.07764988	0.37935
1738.78874	-0.0197909	0.92221482	0.379434
2862.35575	-3.66874629	0.9222255	0.379502
2111.96294	0.22250568	1.0775642	0.379864
2029.89458	3.12851161	0.92230027	0.379973
2226.92119	2.46298652	0.92232543	0.380132
2754.15177	0.40275587	0.92232816	0.380149
1415.65935	-2.14454377	0.9223298	0.380159
1965.72429	0.47537837	0.92233677	0.380203
1778.8674	0.59389351	1.07750576	0.380215
1867.903	-2.26649868	0.92234698	0.380268
2696.34519	-8.28170635	0.92238118	0.380483
1333.63689	-1.00993078	1.07744679	0.38057
1927.98589	3.0405526	0.92241021	0.380667
1826.78252	0.11112537	0.92243214	0.380805
2164.88774	0.2649612	1.07740731	0.380807
2373.15158	2.10527863	1.07738688	0.38093
1323.60845	0.93512266	0.92246891	0.381037
1864.86344	-2.49928614	0.92247972	0.381105
2674.12571	0.25437812	1.0773578	0.381105
4599.99863	0.83348802	1.07732033	0.38133
3244.6406	-2.32080645	1.07728698	0.381531
1700.83811	1.24619904	0.92255085	0.381555
1169.57073	1.96739679	0.92255683	0.381593

Information Classification: General

1392.64148	1.80795056	1.07726186	0.381682
1383.65886	0.46454753	1.07725416	0.381729
3226.37583	-1.41201486	1.07722715	0.381891
3641.46128	0.97255685	0.92262114	0.381999
2005.96562	-0.92550408	0.92263719	0.382101
1957.94803	-1.08434991	1.07714243	0.382402
2145.84599	4.02990913	0.92269787	0.382485
2656.27329	-1.67405709	1.07711172	0.382587
2442.08232	6.40997612	1.07709499	0.382688
1442.73882	0.40120221	0.92273218	0.382702
3203.40415	0.59893219	0.92273377	0.382712
2207.04599	-0.55036234	0.92274524	0.382784
3806.64438	1.61191755	0.92281441	0.383222
1540.76455	0.29061749	0.92284739	0.383431
2449.12259	0.90417936	0.92286101	0.383518
2260.14292	-0.231776	0.92290202	0.383778
1581.76262	-1.61051919	0.92293364	0.383978
1922.90915	0.06630822	1.07686321	0.384088
2036.87967	3.63031961	0.92296131	0.384154
1852.79961	-0.24727681	1.07685059	0.384165
2292.06607	0.90699454	0.92296592	0.384183
2319.81743	2.37523487	1.07684138	0.38422
2330.05498	5.22345588	1.07683875	0.384236
3702.60093	0.76242773	1.07683644	0.38425
1545.73613	-2.52741512	0.92297867	0.384264
2648.27634	-0.88887828	1.07681726	0.384366
2449.32207	-0.23893014	1.0768144	0.384384
1991.00681	0.17918306	0.92303107	0.384596
3514.56289	-2.65449315	1.07673491	0.384865
4225.54099	1.37531317	0.92307834	0.384896
1655.79558	0.65257169	0.9230803	0.384909
1954.88005	-1.9094872	0.92310318	0.385054
1693.80351	-0.12038254	0.92314386	0.385313
2397.18486	0.81752221	0.92316195	0.385428
2134.15354	1.09142584	0.92323074	0.385865
3251.69521	0.14633463	0.92324681	0.385967
1941.77349	0.81667546	0.92326087	0.386056
2358.06736	-3.25748554	1.07645795	0.386545
2929.36777	-2.08042545	1.07645324	0.386573
2415.18143	1.14084047	0.92334974	0.386622
1157.60442	-0.33481564	1.07644377	0.386631
2085.90763	-6.27946034	0.92336274	0.386705
2750.25162	-0.39423069	0.92336909	0.386745
1345.63652	-1.2805032	1.07640915	0.386841
1593.78788	-2.5695708	0.92338927	0.386874
2006.97049	-3.63341828	0.92339333	0.3869
1255.67388	1.16863445	1.07636105	0.387133
1167.55522	2.09675735	0.92347107	0.387395
1492.69231	0.6049746	0.92350644	0.38762
2641.29782	0.64225233	0.9235175	0.387691
1751.77397	-0.4925978	1.07622851	0.38794
3841.62375	1.7133677	1.07615401	0.388394
2721.27738	-1.89521204	1.07615055	0.388415
2415.14188	-0.01591813	1.07613966	0.388481
2504.98955	1.09391749	0.9236703	0.388666

Information Classification: General

3736.8259	-0.15078263	1.07608874	0.388791
1882.92954	0.09732009	0.92378241	0.389382
2854.28702	-1.89779196	1.07598348	0.389434
1590.64092	0.47869973	0.92380095	0.3895
3804.83713	6.53195379	0.9238236	0.389645
2135.16501	-2.72677769	0.92383122	0.389694
1739.86321	-4.32305341	1.07587734	0.390082
2025.04172	1.90606644	1.07585541	0.390216
3544.46699	-0.30308795	1.07583061	0.390368
2539.16819	0.96122337	1.07582462	0.390404
2743.19951	-0.96595622	1.0757698	0.390739
1726.73271	-1.53045437	1.07574439	0.390895
2958.42258	1.20342479	1.07574143	0.390913
2750.13359	-1.81546794	0.92402487	0.390933
1203.52995	3.04896883	1.07573678	0.390941
2119.88921	-3.40656718	0.92404256	0.391046
1775.877	4.07146652	0.92405271	0.391111
2980.48624	-2.66671017	0.92408242	0.391301
3493.70278	-3.06431476	1.07566876	0.391358
2392.05997	1.61424706	0.92414327	0.391691
2963.38564	-1.66177389	1.07556122	0.392017
2300.05698	-9.39252682	0.92430286	0.392715
2141.06504	2.68478241	0.924349	0.393011
1448.65361	-0.2300399	0.92438561	0.393247
2374.10501	-5.61671823	1.07535887	0.393259
3080.5206	-2.52629377	0.92440606	0.393378
1139.51897	-2.19110246	0.92441712	0.393449
1314.57085	-0.02047817	0.92442935	0.393528
3051.17777	0.19475343	1.07525073	0.393924
1770.85356	-1.04191351	0.92451339	0.394068
2508.07322	-0.51393847	1.07521455	0.394146
2116.00029	-3.68315669	1.07521089	0.394169
2254.98613	0.7952524	0.92453884	0.394232
1533.69414	-0.88407743	0.92457518	0.394466
3047.41751	2.92257987	1.07515057	0.39454
1805.78752	-3.18150128	1.07514311	0.394586
1745.80779	-1.18652434	1.07508347	0.394953
1833.91667	2.26906455	0.92465871	0.395003
2258.09177	0.30764568	1.07501241	0.395391
1733.94303	-0.11418114	0.92472068	0.395402
1960.92034	1.30417572	0.92476465	0.395686
1236.57439	0.11573834	1.07489897	0.396091
1732.84538	1.28760063	0.92484309	0.396192
1958.93254	0.24575422	1.07488063	0.396204
2813.18479	-1.36454943	0.92486978	0.396364
1254.54961	0.93487385	0.92488068	0.396434
1505.63762	-0.9394419	0.92488786	0.396481
3326.47336	-0.81272586	1.07480305	0.396684
1213.55974	1.22341036	1.07479307	0.396745
3993.99854	0.6334794	0.92493867	0.396808
2265.96585	1.85755881	0.92494133	0.396826
1201.5866	0.74663808	1.07477076	0.396883
1684.8062	-0.13375871	1.07464719	0.397648
1430.68376	2.80154089	0.92510876	0.397907
1475.56243	-2.14140148	0.92516503	0.398271

Information Classification: General

3603.61667	-2.93232189	1.07451408	0.398472
1484.71195	-0.58400901	0.92519764	0.398482
1392.69634	-3.00039473	1.07450938	0.398501
2345.10294	1.05928219	1.07442691	0.399013
2777.4274	3.08703529	1.07441923	0.39906
1633.81706	0.73013089	1.07434885	0.399497
1672.75249	-1.37874253	1.07431925	0.399681
3076.39433	-2.45111356	1.07427579	0.399951
2893.50869	2.17707939	1.0742728	0.399969
3565.54465	-2.95900051	1.07425199	0.400099
1978.94475	0.59195548	1.07425152	0.400102
1862.8261	0.06930515	0.92545619	0.400157
2040.12444	0.96217032	0.92553474	0.400667
2754.34696	0.01186505	1.07414989	0.400734
3058.43178	2.34273698	1.07412755	0.400873
4258.90053	-6.0832364	0.92557194	0.400908
2629.24491	2.4105397	0.92559582	0.401063
2901.43833	0.92585438	0.92561301	0.401175
1610.68462	1.27250936	1.07398147	0.401782
2114.90776	0.33436092	0.92572192	0.401882
3221.41513	-3.29421995	1.0739626	0.4019
1519.77654	0.3412058	1.07394579	0.402005
2692.34292	-5.06179438	0.92574502	0.402032
2820.16385	-4.8677272	1.07393605	0.402065
1667.81133	-0.56265619	1.07392263	0.402149
2030.9498	0.08661896	1.07384094	0.402659
1397.67066	0.12136739	1.07383102	0.402721
4076.91172	-2.84088044	1.0738274	0.402743
1780.93155	0.85764499	0.92589901	0.403034
1730.77422	1.01643565	1.07376044	0.403161
3169.36631	0.4883705	0.92593022	0.403237
2833.27715	0.91127976	1.07373226	0.403337
1963.98967	1.92787276	0.92596412	0.403458
1526.7677	0.34617583	0.92596531	0.403466
1576.75115	5.78907558	1.07367839	0.403674
4149.82924	0.66172331	0.92599966	0.40369
1504.72453	0.38253077	0.92601544	0.403793
1365.61272	1.07365519	0.92611532	0.404444
2825.14311	1.71803863	0.92614195	0.404617
3922.74721	-5.16454672	1.0735173	0.404682
3427.71091	0.76415931	1.07351359	0.404705
2418.18535	1.22613973	1.07351246	0.404712
2277.02048	-3.09499645	0.9261682	0.404789
2130.06205	1.2837988	0.92617034	0.404803
4296.26539	2.87152161	1.07349341	0.404832
2311.14951	0.00733401	1.07349034	0.404851
1268.56794	0.68538992	0.92618308	0.404886
2399.1043	1.89668521	1.07345163	0.405093
1925.12326	0.72661987	0.92621676	0.405106
1679.76889	1.72566335	0.92622771	0.405177
1887.94121	-1.41508213	1.07342286	0.405274
1576.81693	3.55550698	0.92624308	0.405277
1898.91721	-2.60245927	0.92626323	0.405409
2617.08615	-6.06420318	0.92626943	0.405449
2044.8975	0.94468786	0.92633913	0.405905

1285.58586	-3.704359	1.07326594	0.406258
2303.04453	-1.2650727	0.92639693	0.406283
1445.61833	0.28944816	0.92640032	0.406305
4758.28382	-0.38828367	1.07324102	0.406414
1011.47356	-0.57041438	0.92644149	0.406574
1657.80364	-0.03729879	1.07317782	0.406811
3404.76963	-1.19798487	0.92649225	0.406906
3400.69423	1.95232875	0.92649717	0.406938
2293.19931	1.3425076	0.92651154	0.407032
3342.46543	3.30053339	0.9265178	0.407073
1416.6458	-1.07207329	1.07312967	0.407114
3828.56723	-1.96455754	0.92657697	0.407461
1836.87247	0.97904226	0.92659002	0.407546
1204.54509	-2.4355717	1.07303506	0.407709
1728.83347	2.02674867	0.92662581	0.40778
2511.30874	-0.45863487	0.92663022	0.407809
3073.30515	-3.77131492	0.92664428	0.407901
1969.00395	0.91698584	1.072939	0.408314
1342.69206	4.66666966	1.07289285	0.408604
2268.99204	-0.20747236	1.07287086	0.408743
3209.37558	0.52632318	1.07285938	0.408815
2387.00591	2.27132817	1.07283958	0.40894
3894.82065	3.15124346	0.92683611	0.409159
2425.27622	-1.38832253	1.07279594	0.409215
2821.32895	-0.21893605	0.92685603	0.40929
3016.37528	0.22775448	0.92687014	0.409382
3860.87075	-0.15205249	1.07266741	0.410027
1322.59136	2.89574893	1.07266079	0.410069
2057.03977	-0.43278742	0.92704092	0.410504
2085.83647	-0.12077649	0.92707507	0.410729
3683.72531	1.32002624	0.92708184	0.410773
2581.05766	-0.26908107	0.92710732	0.410941
1818.93047	-0.08949181	1.07251739	0.410975
2052.05069	0.94151282	0.92713539	0.411125
1236.70859	0.67112112	0.92715718	0.411269
2241.10105	-1.88030971	1.07239239	0.411766
2376.09226	3.16905769	0.92727082	0.412017
3717.69797	4.4398077	1.07233842	0.412108
1399.75383	-1.43562498	1.07233412	0.412135
1361.61197	1.70116667	1.07231343	0.412266
2761.16445	-0.86734534	1.07230755	0.412304
3764.66494	0.35710964	0.92731859	0.412332
3262.54392	-5.57536412	0.92734483	0.412505
3617.75771	2.50150407	1.07226968	0.412544
3576.71592	-0.25576535	1.07217203	0.413163
1538.81072	2.41631022	0.92747154	0.41334
2295.13034	1.84125095	1.07213808	0.413379
1297.62639	-0.48159302	1.0721228	0.413476
4839.17942	1.15787742	1.07208932	0.413688
1670.75102	0.38237968	0.9275651	0.413958
2269.11089	-1.17572813	1.07202755	0.414081
1881.80071	0.69533316	0.92762933	0.414382
4166.73842	-3.26664944	1.07197859	0.414392
2269.99216	-1.59898473	0.92763435	0.414415
1973.02567	1.0697367	1.07194804	0.414587

Information Classification: General

2286.08127	-1.38374551	0.92767584	0.414689
1047.5595	0.39858446	1.07193192	0.414689
2214.11661	0.53475087	0.92768621	0.414758
1527.83525	5.14739849	1.07192058	0.414761
1197.62253	0.2725491	0.92768936	0.414778
1096.51738	1.57433266	0.92771509	0.414948
1982.83298	0.37604261	1.07188176	0.415008
2172.95342	-1.97038767	1.07185839	0.415157
2252.06009	5.57909007	0.927776	0.415351
2355.0313	0.4608426	0.92786028	0.415909
1570.70146	-0.3130357	0.92788811	0.416093
3518.41147	1.60948788	1.07170041	0.416164
2045.99565	3.32003027	0.92792133	0.416313
1490.61333	0.29756124	0.92796105	0.416576
2105.99883	-1.7893668	0.92798534	0.416737
3307.46885	-6.81793648	1.07156017	0.417059
2485.08672	0.34172676	0.92804508	0.417133
2212.09769	-0.55056233	0.92804876	0.417157
4014.01381	1.91737039	1.07152683	0.417272
1697.86028	0.06407532	0.92806943	0.417294
3242.66909	0.33607769	1.07151746	0.417332
2370.89141	3.26953846	1.07151511	0.417347
1974.91411	-3.42101017	0.92809062	0.417435
2801.17252	1.01893185	0.92809483	0.417463
3216.34238	3.49777555	1.07149232	0.417492
2701.17588	1.5430826	0.9281193	0.417625
1808.74407	-1.82455142	1.07146696	0.417654
1731.81699	-0.82894512	1.07146009	0.417698
4782.0018	0.28771881	1.07145199	0.41775
1545.7293	-0.99671681	1.0714423	0.417812
1881.87236	0.5987398	1.07144117	0.417819
2968.57876	0.6945268	0.92818641	0.41807
1871.84868	0.36600782	0.92819383	0.41812
1426.7188	0.44809023	0.9281996	0.418158
2695.18119	3.00914689	0.92829233	0.418774
3019.33152	-0.50879278	0.92833132	0.419033
2147.98564	-3.11873506	1.07120599	0.419325
2448.19315	0.49550286	0.92839617	0.419464
2772.32695	1.24790318	1.0711281	0.419824
3574.5549	-1.23415147	0.92845427	0.41985
3177.4039	1.59736735	0.92846164	0.419899
2225.96445	2.82365527	0.92847658	0.419998
1694.76604	2.60419998	0.92847796	0.420008
2449.91118	4.18999323	1.07109006	0.420068
1419.65068	0.56145106	0.92850159	0.420165
2180.12363	1.52378686	1.07105292	0.420307
2620.02738	-0.59747378	1.07103001	0.420454
2247.06865	1.03059351	0.92855504	0.420521
2637.2869	5.9227863	1.07094479	0.421001
2637.23343	-0.7859411	1.0709218	0.421149
2561.19219	2.4144891	1.07090911	0.42123
3194.48142	-10.5641154	0.92868456	0.421383
3032.49314	-2.15041552	0.92869585	0.421458
2706.18178	-5.6267095	1.07087041	0.421479
1530.74272	-1.08112224	0.92871217	0.421567

3573.68398	1.16528683	1.07081512	0.421835
1236.67046	0.01573176	0.92877962	0.422017
1937.04997	0.65360036	0.92881079	0.422225
1695.90495	0.94140023	0.9288289	0.422346
2497.12351	0.35760083	1.07073207	0.422369
2375.1044	0.86521229	0.92886342	0.422576
4859.21303	-1.36892511	0.92888842	0.422743
1826.94156	0.20590295	1.07060984	0.423157
2565.16074	0.40208738	0.92896628	0.423263
1289.56548	-0.44899911	1.07058052	0.423346
3373.6688	2.57614481	0.92898995	0.423421
2619.27719	2.30807831	0.92899113	0.423429
1601.67412	-2.03501798	1.07051866	0.423745
1578.76433	-0.16181939	0.92904981	0.423821
2136.99028	-1.34456683	1.07048203	0.423981
2861.43178	1.13058828	0.92913697	0.424404
1048.56719	-0.993765	1.0703848	0.424609
2423.15641	3.5193447	1.07038012	0.424639
2005.0325	-1.6725138	1.07035488	0.424802
2751.16312	0.46481789	0.92921437	0.424921
2497.20792	-0.94842141	1.07032842	0.424973
4039.98886	-0.96499802	1.07027953	0.42529
2096.97068	-1.57801816	0.9292911	0.425435
2415.16884	0.22840976	1.07023906	0.425551
977.469591	0.0028193	0.92931436	0.425591
1307.67807	1.9824475	1.07023034	0.425608
1891.79265	-2.11923975	0.9293835	0.426054
2112.13163	-0.91346143	1.07014283	0.426174
3607.91526	2.3620345	1.07013039	0.426255
2533.1702	0.14679944	0.92943059	0.42637
2939.3494	-1.62141775	1.07010311	0.426432
2148.00347	-2.6441915	1.07007011	0.426645
2297.11979	0.96374117	1.07004322	0.42682
3354.61301	-0.40275091	1.06997503	0.427262
2511.34842	0.84596952	1.06996191	0.427347
1428.66437	-0.7657428	1.06996142	0.42735
2349.04575	0.05882575	0.92958798	0.427426
1434.6738	0.10102797	0.92962628	0.427683
1622.80498	1.23301284	0.92972543	0.428349
2168.80498	-0.03968113	0.92979005	0.428783
2170.9644	0.58995325	1.06974073	0.428784
3923.8146	2.21228629	0.92981111	0.428925
3005.42319	13.7669766	0.92985551	0.429224
4525.21433	-2.72710046	1.06961789	0.429583
1677.7249	-1.1474978	1.06960255	0.429683
2256.07285	-0.87284693	0.92996448	0.429957
1745.79508	-1.48769027	1.06954478	0.430059
1811.86479	5.14132872	0.92998209	0.430076
1759.80425	1.28282308	0.9299984	0.430185
1386.67473	0.03239111	1.06951806	0.430233
2927.50632	-3.28181241	1.06946469	0.430581
1958.86589	0.56513584	0.93007475	0.4307
2812.31632	-1.54711187	1.06940176	0.430991
3651.48703	-0.6119797	0.93013632	0.431115
2494.11252	-1.10554542	1.0693766	0.431155

2615.27579	0.04041191	1.069355	0.431296
3224.40781	8.09132492	0.93016438	0.431304
3244.75595	2.6492786	0.93024832	0.43187
1202.65427	0.46704377	0.93025559	0.43192
2404.11399	-8.59589481	1.06925584	0.431944
2700.25078	0.24209626	1.06922562	0.432141
2461.88926	-3.30435507	0.93030042	0.432222
1166.53203	-0.29598791	0.93031809	0.432341
2650.19455	2.07075186	1.06918266	0.432422
1288.64214	-0.5749133	0.93035335	0.43258
3969.74958	-0.4189903	0.93037742	0.432742
2883.52651	-1.8038108	0.93039429	0.432856
1540.84751	-0.44001961	1.06910865	0.432906
3059.1771	6.50071571	0.93040217	0.432909
1581.67485	0.72201075	0.9304057	0.432933
1364.63408	0.92002823	0.93043073	0.433102
1391.55437	-0.10090313	1.06902573	0.433449
2325.06816	5.60565636	0.93048525	0.433471
2074.82352	0.2878223	1.06900506	0.433584
1879.93851	-5.0201563	0.93054297	0.433861
1684.76921	1.32026262	0.93055694	0.433956
2540.07621	-0.9229143	0.93058768	0.434164
1741.77361	-1.53184715	0.93059823	0.434235
2950.38535	2.00576977	1.06887107	0.434462
1478.70195	4.4833887	1.06879967	0.43493
1725.95391	1.53174463	1.06877866	0.435068
1786.87944	-2.3444055	0.93074429	0.435224
1434.74993	-1.08016842	0.93079693	0.435581
2121.07871	1.09999159	1.06868431	0.435688
1418.61296	1.15206745	0.93082035	0.43574
2620.08359	-0.125194	0.93085668	0.435986
2515.27768	3.32280534	1.0686383	0.43599
1240.64311	0.95813614	1.06857478	0.436408
2154.00713	2.40128123	0.93096573	0.436726
3570.56137	-0.39232364	1.06851939	0.436772
1913.94243	-1.86938159	0.93104293	0.43725
3083.3666	-1.00878229	1.06840162	0.437547
2204.91045	0.10099702	0.93109095	0.437576
2436.06864	0.68362933	1.06838785	0.437638
1817.03336	1.92514223	0.93113228	0.437857
2572.04825	0.9065249	1.06834331	0.437931
1858.90605	1.2024051	0.93115002	0.437978
2522.20171	0.92516589	0.93117067	0.438118
2480.24348	1.28537909	0.93120851	0.438375
2803.45205	-0.06821122	0.93123911	0.438583
1557.73943	-0.39211849	1.06821655	0.438767
2198.96592	-1.12771189	0.93126661	0.43877
2255.07969	-0.37527222	0.93127003	0.438794
1966.91985	0.72148514	1.06820957	0.438813
1138.60015	1.00997264	1.06819138	0.438933
1983.87119	2.32809344	1.06818925	0.438947
1880.94449	-2.76251631	1.06817509	0.439041
1639.80814	0.42341606	1.06814193	0.43926
1725.72214	1.35351218	0.93135336	0.439361
2163.78716	0.65703031	0.93137358	0.439499

Information Classification: General

2820.18203	-0.24387012	0.93137691	0.439521
1514.75261	0.04342314	0.93140293	0.439698
2198.01505	-1.8942087	0.931404	0.439706
2196.99222	6.12171141	0.93142496	0.439848
1536.67197	0.41315747	0.93142764	0.439867
1203.5916	2.03144748	1.06804796	0.439881
1739.72234	1.16934626	0.93143794	0.439937
1632.77616	0.68466165	1.06800508	0.440164
1288.59001	-0.95055908	1.06799498	0.440231
1320.62322	-4.13574127	0.93153677	0.44061
1531.73979	0.09491984	1.06791912	0.440733
2820.38559	0.25348817	1.06790627	0.440818
1489.68535	0.3544595	1.06790266	0.440842
2269.0645	1.83001574	0.93160847	0.441099
3405.44675	2.12341037	0.93161367	0.441135
1426.72087	1.88859231	0.93164002	0.441315
3059.37265	-1.17165156	0.93167374	0.441545
2813.19028	-0.76054778	0.93174628	0.44204
1943.85001	-2.68516691	0.93175295	0.442085
2749.41245	4.34616608	0.93175399	0.442093
1721.88663	0.53170285	1.06770744	0.442135
2084.05771	0.37622519	1.06768907	0.442257
1482.67107	0.27167628	0.9317879	0.442324
1667.63347	-2.55742629	0.93179025	0.44234
2470.25809	1.19400195	0.93180177	0.442419
2593.01529	-1.05682315	0.93180781	0.44246
1945.00504	0.7921497	1.06764817	0.442528
1396.60759	1.48663381	0.93182553	0.442581
1475.61931	0.17754855	1.06763804	0.442595
1920.84648	-1.36718598	0.9318381	0.442667
2882.54166	0.32326641	1.06761993	0.442715
3076.47141	1.07245075	1.06760213	0.442833
1599.8709	-0.65261008	1.06759638	0.442872
2906.48819	1.93877685	0.93188719	0.443003
3200.48545	2.32697512	1.06756332	0.443091
1649.69988	2.0133361	1.06753328	0.443291
2623.12699	-0.31947468	0.93196305	0.443521
2253.07029	-0.03092794	1.06746663	0.443733
3745.73549	2.16086768	1.067388	0.444256
1805.88176	0.3040985	0.93208064	0.444326
1050.5568	-0.95040301	0.93208291	0.444342
1509.66814	1.14933807	1.06736905	0.444382
1976.84685	-0.43703104	1.06736769	0.444391
1497.73725	2.06911267	1.0673657	0.444404
1892.86869	0.90190942	1.06736044	0.444439
1516.79802	-0.27035138	1.06732598	0.444669
2088.07012	-0.71948823	1.06729127	0.4449
2330.10307	1.56630088	0.93218803	0.445062
1365.73633	-0.66157736	1.06723124	0.445299
1765.89104	-1.97208675	0.93223602	0.445391
1433.73979	0.38039852	0.93224344	0.445442
1952.91985	-0.3537762	1.06718973	0.445576
1892.90201	-0.2396366	1.06718945	0.445578
1713.86789	0.91298697	0.93235172	0.446184
2400.1904	4.90982077	1.06704804	0.446521

2165.80107	-0.91573998	0.93242302	0.446673
3204.57461	0.21449551	1.06701679	0.446729
1587.79912	0.57410561	0.93244754	0.446842
2478.14975	0.14853446	0.93245132	0.446868
1752.91568	1.18959881	1.06698594	0.446935
1141.57536	1.62904844	1.06697985	0.446976
1983.80596	0.46904884	0.9324788	0.447056
3141.39438	7.8146816	0.93251124	0.447279
2368.19107	0.96102524	1.06691043	0.44744
1298.62297	0.55990342	1.06690984	0.447443
1654.74497	-0.36686356	1.06690015	0.447508
1543.75066	0.72527925	0.93255252	0.447563
2028.94145	1.92836947	0.93257917	0.447746
2061.85063	2.90721168	1.06685389	0.447817
2735.306	-3.61272536	0.93259633	0.447864
2089.93192	0.48978532	1.06679992	0.448178
1361.65727	-0.76461146	1.06678597	0.448271
1424.66883	0.44963218	0.93266134	0.448311
1756.87627	2.36214806	0.93266265	0.44832
1954.94109	0.93728544	1.06676357	0.448421
1663.7741	-0.20125911	1.06675186	0.4485
2401.17752	0.57600353	0.93269074	0.448513
1913.01615	-0.79500971	1.0666962	0.448872
2467.23465	0.70037642	1.06669139	0.448904
1230.54936	0.77096054	0.93277755	0.44911
4034.73301	-4.31824268	1.06665836	0.449125
2714.51894	-1.0829527	1.06664497	0.449215
3174.58847	1.05707993	1.06662927	0.44932
1296.66789	-0.19358971	1.06661248	0.449433
2971.21579	1.66602833	1.06657454	0.449687
2728.34635	2.82674116	1.06652177	0.450041
3072.53098	-0.51629209	1.0664842	0.450293
1177.59661	-1.75364302	0.93295439	0.450328
2064.96421	1.20496792	1.0664403	0.450587
1426.70732	0.2696344	0.93300453	0.450674
2172.80718	-2.29279204	0.93300616	0.450685
1700.75974	0.19429093	1.06638723	0.450944
2411.91582	1.64175843	1.06637464	0.451028
2175.0103	2.54536955	0.93308713	0.451244
2846.3461	8.19583685	0.9330902	0.451265
1713.85747	1.40103468	1.0663263	0.451353
1398.4821	0.41327866	0.93311744	0.451453
4166.19155	-1.18796116	0.93313614	0.451582
2502.01664	1.53932423	1.06629095	0.45159
2144.94414	-1.15429783	0.93315273	0.451697
1277.56584	1.12573234	0.93322459	0.452193
1666.77463	2.34346038	1.06617268	0.452385
3417.6207	0.25027829	1.06616224	0.452456
2509.16655	1.55956367	1.06616111	0.452463
3712.69961	0.76423282	0.93326639	0.452481
2043.12485	0.28032887	1.06610976	0.452809
2512.04605	1.36636036	0.93333732	0.452972
2004.88786	3.08480173	0.93336948	0.453194
2393.23569	1.28261108	1.0660364	0.453303
2000.11416	4.33232678	0.9333975	0.453388

Information Classification: General

4207.94741	2.61887809	1.0659768	0.453704
1976.02764	1.07497959	1.06595374	0.45386
2668.33097	-1.96060029	1.06594131	0.453944
1756.75762	-2.49285493	0.93349591	0.454069
1476.68753	-1.11407973	0.9334994	0.454093
4672.20056	-0.04536694	0.93350023	0.454098
3915.5958	-4.52293557	0.93350175	0.454109
2154.08457	-1.09116248	1.06589629	0.454247
1314.65447	-0.02415535	1.06587963	0.45436
2147.00102	1.11730792	1.06587145	0.454415
1527.80205	-4.97364703	1.06586241	0.454476
880.43944	-1.80093182	1.06583218	0.45468
1995.14678	-0.93135284	1.06582259	0.454744
1644.76738	-2.2761376	1.06578841	0.454975
3076.452	-1.11648601	1.06578833	0.454976
3024.40288	2.36884115	1.06573916	0.455308
1881.89133	-1.26721501	1.06567832	0.455719
2736.33066	3.16237137	1.06564072	0.455973
2252.92339	-0.28489983	1.06558935	0.45632
3432.49875	-2.11533535	0.93384518	0.456489
3293.36245	1.29932973	0.93387341	0.456685
1263.58928	-0.14823077	0.93390968	0.456937
2923.45255	-0.81974966	0.93392714	0.457058
1480.65373	-1.00035395	1.06544937	0.457268
2453.32688	0.14515557	0.9339574	0.457268
1642.79289	0.05233675	0.93395798	0.457272
1981.8801	0.90407604	1.06537596	0.457765
1506.7293	-1.93774315	1.06535182	0.457929
2173.85088	-0.86833147	1.06534347	0.457985
1365.6646	0.09012462	1.06533991	0.458009
1868.98027	3.82610145	1.06531262	0.458194
2207.0352	1.04327686	0.93411536	0.458366
3173.6332	1.12209466	1.06525577	0.45858
1485.77149	0.63920312	1.06524574	0.458648
1716.83672	-2.94365756	1.06524147	0.458677
1254.54863	0.16442816	0.93418293	0.458836
3166.52744	4.25407256	1.06510457	0.459607
1828.86064	2.05865979	1.06504865	0.459987
1330.56462	0.11085073	0.93434928	0.459993
2632.22696	0.34099008	1.06499983	0.460319
1963.02683	0.30936202	1.06499053	0.460382
2643.25394	-1.29571268	1.06497981	0.460455
2331.99346	3.00151008	1.0649606	0.460585
2872.28025	-0.91799723	1.06495827	0.460601
2675.2053	0.0422707	0.93443769	0.460609
1849.91416	2.87547802	0.9344509	0.460701
1760.71855	2.9636526	1.06491677	0.460884
1813.83794	-3.04551809	0.93450174	0.461056
1465.76667	1.47426768	1.06488653	0.461089
1737.85112	1.48086189	0.93452208	0.461198
1732.75469	0.5078449	1.06486467	0.461238
1882.83707	1.20083952	0.93452791	0.461238
1614.80998	-0.05398642	1.06486268	0.461252
2251.96854	-3.84343215	1.06484205	0.461392
2075.74614	1.31569497	0.9345662	0.461506

3092.51218	0.62662597	1.06481105	0.461604
1529.79509	0.92185161	0.93458456	0.461634
1825.84465	-0.72087681	0.93461741	0.461863
2285.11246	-2.55059212	0.93462186	0.461894
1970.00333	0.77905999	1.06475341	0.461996
996.500231	0.62258312	0.93463718	0.462001
2527.18943	3.55649723	1.06475046	0.462016
2834.39663	0.78776951	0.9346465	0.462066
3475.73874	0.33384225	1.06474059	0.462084
1176.53203	1.18545062	0.93470753	0.462492
2716.25699	0.59046701	1.06465925	0.462638
1949.86467	1.89362997	1.06465247	0.462685
1878.88884	0.57121211	0.93478815	0.463055
2348.09854	5.02687358	1.06455255	0.463367
2869.1063	1.13626302	1.06455002	0.463384
1484.52532	-0.25135749	1.06451073	0.463652
1654.69853	3.70921153	1.06449761	0.463742
1385.74187	-0.31493036	0.93489645	0.463812
2263.99785	-0.6324948	0.9349003	0.463839
3025.34525	0.32416006	1.06444137	0.464126
3060.46708	2.14211354	1.06443579	0.464164
3598.65688	-0.84232607	0.93496022	0.464258
1656.71868	-2.91526355	1.06436356	0.464658
2027.98931	5.71359584	0.93502489	0.464711
3212.52578	-0.37719795	1.06435463	0.464719
3185.36277	0.9709368	1.0642944	0.465132
3401.51035	-4.85878464	1.06427954	0.465233
3112.45976	-2.83309827	0.9351238	0.465403
1977.91111	-1.89056541	1.06414607	0.466148
2989.43277	2.66074227	1.0641007	0.466459
1719.89348	1.34596471	1.06401981	0.467014
3302.46823	2.4422751	1.06401793	0.467026
2418.20597	4.71774104	1.06395577	0.467453
1743.77483	-3.30399758	0.93546356	0.467786
1922.93218	-1.03945164	0.93551564	0.468152
2052.96269	0.47530485	0.93556138	0.468473
1539.70671	0.48927358	0.93556376	0.46849
3493.68711	2.60802012	1.06375507	0.468832
1351.69841	0.76463548	0.93561876	0.468877
1421.7293	0.90547872	0.93562496	0.46892
3202.38059	4.92853711	1.06369767	0.469227
3563.77944	0.07586083	0.93568311	0.469329
1725.91521	0.18971474	0.93570605	0.469491
1285.60334	1.14409652	1.06365799	0.4695
1982.02036	0.72832722	0.93575423	0.46983
1993.88823	0.00145068	1.06358139	0.470028
2143.04532	3.15856049	1.06354457	0.470282
1426.7294	-2.16651487	0.93585031	0.470506
2076.09812	-0.00513057	1.06349887	0.470597
1886.87749	0.44184374	0.93587415	0.470674
3684.51926	19.4301947	0.935943	0.471159
2006.96976	1.59714157	1.06341707	0.471161
1988.8864	-0.88902121	1.06339664	0.471302
971.470201	2.29148289	1.06339288	0.471328
2463.15488	1.41649385	0.93604837	0.471902

1906.87406	3.47127794	1.06330586	0.471929
2898.38193	-9.70237038	0.93607411	0.472083
1520.65349	-0.31257511	0.93623391	0.473211
2460.15708	4.11571662	0.9362348	0.473217
1410.62102	2.73920379	0.93628269	0.473555
1019.47885	-0.33384398	1.063058	0.473642
4200.8657	2.02279103	1.06301678	0.473928
1862.82781	1.49830693	0.93634172	0.473972
3794.59165	1.27829878	1.06299548	0.474075
2226.04227	0.03377315	0.93636742	0.474154
1972.93283	-3.59913656	1.06298233	0.474166
2514.19765	-0.69475768	1.0629648	0.474288
2009.95659	0.3245182	0.93639288	0.474334
1328.58025	1.17305226	0.9363944	0.474345
2136.96245	2.21887029	1.06295409	0.474362
1337.56352	0.39987727	0.93641467	0.474488
3191.44333	-0.0356603	0.93643224	0.474612
1300.50945	0.1389554	0.93645318	0.47476
1396.67461	-2.19604077	0.93646098	0.474815
2658.1395	3.40771595	0.93646115	0.474817
2217.9946	0.50348339	1.06287468	0.474912
3068.36661	0.6286735	0.93647775	0.474934
1694.69829	1.22174328	0.93653172	0.475316
2101.93755	1.12053698	0.93653865	0.475365
3404.57773	-0.89831809	1.06274512	0.475811
2958.53116	5.27213714	1.06273492	0.475882
1420.60051	-0.71366584	1.0627299	0.475917
3279.53281	-0.3074428	1.06269628	0.47615
3204.58042	6.47889908	0.93665627	0.476198
3384.65036	2.43819636	1.06268407	0.476235
3523.67275	3.3606691	0.936672	0.476309
3124.41684	1.40312084	0.93667352	0.47632
3981.00112	2.27607039	1.06265383	0.476445
2764.38645	-1.65337717	1.06263474	0.476578
2422.21587	0.52415281	1.06263169	0.476599
1823.79851	-1.55104151	1.06260885	0.476758
2254.90592	3.3534177	0.93673866	0.476781
1632.83757	-1.0308969	1.06258942	0.476893
1259.6154	0.02281971	0.93675928	0.476927
1872.87302	0.408299	1.06245159	0.477851
1298.65862	-0.74797587	1.06244447	0.477901
2403.10381	1.99910814	0.93692332	0.478091
2533.06528	4.90805921	0.9369328	0.478158
3131.5477	0.456742	0.93695507	0.478316
1800.93157	-0.36433731	0.93696601	0.478394
2635.09551	0.26461399	0.93698071	0.478498
1620.73441	1.34127611	0.93702077	0.478783
1681.91819	3.16169232	0.9370342	0.478878
3526.74277	4.04244644	0.93703521	0.478885
1738.88211	3.0513457	1.06228894	0.478984
1932.83061	2.95509858	1.06226702	0.479137
4151.07639	1.72649986	1.06225214	0.479241
1689.78691	0.90989764	1.06223994	0.479326
2333.16973	0.48690548	0.93712281	0.479507
2218.98931	-1.05313205	1.06220549	0.479566

Information Classification: General

3803.7385	2.74445991	1.0621859	0.479702
4004.9	1.03828591	1.06216244	0.479866
1331.64873	0.74639757	1.06213688	0.480044
2522.1879	1.7703135	1.06211703	0.480183
3542.64878	-0.85768363	0.93724432	0.480371
2184.11992	-1.24427892	0.93725247	0.480429
2294.09551	0.43951227	1.06205555	0.480612
1399.81694	-1.83821893	0.93729614	0.48074
2290.06711	1.70016374	1.0620265	0.480815
2503.04935	2.02163757	0.93733988	0.481051
1582.69792	-1.65269989	1.06194986	0.481351
1439.7509	0.7666541	1.0619393	0.481425
1496.75041	1.70269516	1.06193874	0.481429
3335.62754	-5.90045082	0.93740231	0.481495
1265.59661	-3.30285657	1.06191062	0.481625
1621.76409	1.9098861	1.06189712	0.48172
1670.75278	0.86334399	1.06187939	0.481844
2775.11295	-1.19278338	0.93745487	0.48187
2414.14194	-1.23042035	1.06185707	0.482
2803.29471	10.6137919	0.93747452	0.48201
1584.81377	0.81397695	0.93748305	0.48207
1350.65068	0.70489312	1.06183771	0.482135
1763.76726	-0.43701185	0.93749305	0.482142
1924.91819	1.9781141	1.06181926	0.482264
2193.95908	0.9089789	1.06176402	0.482651
1870.9451	1.92106744	0.93761163	0.482987
2306.09062	-0.64851284	1.06170132	0.48309
1274.62554	-0.58795307	1.06169316	0.483147
2039.10129	0.08345665	0.93764304	0.483211
1970.94939	-0.17244529	1.06168168	0.483228
2433.15177	-0.45527338	1.06167988	0.48324
3111.36772	2.71748014	1.06163054	0.483586
1796.86553	-3.07323255	1.06160176	0.483788
1224.63407	0.02372584	0.93772623	0.483804
1931.82353	-3.01858478	1.0615955	0.483832
2644.15262	-0.25840302	0.93773122	0.48384
1770.85356	-2.40905083	1.0615506	0.484147
2121.99785	2.05703937	1.06147578	0.484672
1403.66741	1.1382306	1.0614671	0.484733
1678.70635	0.04058296	1.06144847	0.484864
3314.68886	-1.12554173	1.06141594	0.485092
3136.27585	-0.50801544	0.93805984	0.486187
1688.90403	3.27108674	0.93806504	0.486225
1811.91643	0.63365764	0.93807562	0.4863
1685.80449	1.09304379	1.06121516	0.486503
2401.96543	0.31845535	1.06120917	0.486546
1763.85564	-0.66892149	0.938111	0.486553
2299.00797	-3.17248894	1.06118716	0.4867
1410.63823	-0.05962372	0.93814795	0.486818
1360.67546	0.85791053	0.93815749	0.486886
2180.10015	2.27831506	0.93815965	0.486901
1133.47881	0.8046594	1.06112336	0.48715
1245.57524	0.52795787	1.06112171	0.487161
1362.81333	1.49639671	0.93826244	0.487637
1615.72551	0.25695326	1.06104355	0.487712

2661.26822	0.65507448	0.93829061	0.487839
2455.16142	5.44821686	0.93831939	0.488045
1127.55925	1.23842744	1.06099404	0.488061
1804.90061	1.62478909	1.0609616	0.488289
1757.86955	0.69580515	1.06095852	0.488311
1743.88347	3.79162394	0.93836072	0.488342
1613.85019	-0.04055906	0.9383772	0.48846
2517.19809	0.57720303	1.06091982	0.488584
1738.91106	1.08115992	0.93847297	0.489146
3506.64402	2.30156979	1.06081534	0.489321
2551.12076	0.087174	0.93849868	0.489331
2039.93167	0.7728089	1.06077367	0.489616
2189.01065	-1.13522996	1.06073469	0.489891
3038.44828	0.90211832	1.06073445	0.489893
2777.24126	3.49275967	1.06071606	0.490023
1680.78069	-0.99837702	0.93862198	0.490216
1681.75444	-0.98478705	0.93866685	0.490538
2737.20903	-5.97920219	1.06061187	0.490759
2751.42643	0.88334323	1.06058404	0.490956
1597.60832	1.61407893	1.06054831	0.491209
3940.64719	-0.5243773	0.93876757	0.491261
2191.91655	0.72366122	1.0605097	0.491482
3138.60495	1.91973378	1.06047891	0.4917
1477.62175	-3.11825383	1.06045298	0.491884
1291.68852	-0.54280791	1.06042877	0.492055
2150.88579	-3.23983439	1.06041864	0.492127
2916.34487	-4.06123479	1.06039624	0.492286
4399.10512	1.92106003	1.06037746	0.492419
2505.2479	0.79098834	1.060362	0.492529
2461.13999	-3.55141699	1.06034785	0.492629
1906.01933	1.33230974	0.93904072	0.493226
3086.4164	-1.00950673	0.93904217	0.493237
2109.04936	1.21178055	0.93906551	0.493405
2207.00461	-0.64726233	0.9390986	0.493643
1511.76047	0.26185634	0.93911565	0.493766
2640.14023	-0.16415558	0.93911771	0.493781
1420.74309	-0.63960125	0.93913351	0.493895
3723.76528	0.95365705	0.93917187	0.494171
2235.98215	1.62906111	1.0601165	0.494271
2005.9727	-1.30682299	0.93923023	0.494592
1921.86321	4.34343007	0.9392525	0.494752
4451.10093	1.60408981	0.93925422	0.494765
2216.08061	-0.11159116	0.9392668	0.494855
2146.94895	-3.59793066	1.06002295	0.494935
2583.26273	-3.60274433	1.06000782	0.495043
2084.85039	1.14065529	0.93929745	0.495077
3119.57187	-2.90513222	1.05995377	0.495427
3265.54538	0.30285092	1.05995137	0.495445
2165.075	1.0112678	1.05995002	0.495454
1682.7509	-0.24733849	0.93935718	0.495507
2023.82427	1.3370967	0.93944207	0.49612
4128.20424	0.38904053	0.93947262	0.496341
1891.89182	-4.89588231	1.05980689	0.496473
2786.30509	-9.33653563	1.05978715	0.496614
2796.28774	-0.93047202	1.05978058	0.49666

Information Classification: General

2040.87687	1.35231433	0.93951976	0.496681
1699.89934	0.06958702	1.05975182	0.496865
2638.26523	8.80374193	1.05973625	0.496976
2721.39676	0.25404894	1.05970378	0.497208
1868.89873	0.14657797	0.93966481	0.497729
1524.80097	-1.04952043	1.05962164	0.497793
1850.84477	0.25872578	1.05956273	0.498214
2012.73345	1.37759734	0.93975011	0.498346
1741.7968	-1.11079063	0.93975235	0.498363
1153.53813	0.87403808	0.93976391	0.498446
2743.37734	1.28372493	0.93982287	0.498873
2453.99516	1.05113746	1.05945559	0.498979
2289.18271	-0.70152766	0.93984168	0.499009
1683.88701	0.84221172	1.05943864	0.4991
2237.04984	3.2768782	1.0594203	0.499231
1574.72722	0.73355582	1.05941348	0.49928
1093.52141	1.27533254	0.93988818	0.499346
3409.65576	1.87650377	0.9398927	0.499378
2015.04997	0.29580237	1.05937449	0.499558
2271.18193	4.19784265	1.05932342	0.499924
1371.63127	0.63930059	1.05932095	0.499941
2423.12455	-0.12109526	0.93999309	0.500106
2063.98752	0.79973609	1.05927648	0.500259
1270.71228	-0.75342925	0.94009237	0.500825
2561.3461	-0.57072011	0.94010227	0.500897
1398.72168	-1.12601224	1.05918586	0.500908
1387.62151	-1.54057883	1.05917592	0.500979
2245.26047	0.18388248	1.059168	0.501036
1769.89556	-0.0281874	0.94016139	0.501326
2064.95047	1.7425626	0.94016934	0.501383
1967.06412	1.30639687	0.94019783	0.50159
1695.94219	0.87560215	1.05908347	0.501641
2285.89725	-2.29859447	0.94020863	0.501669
4468.12026	-7.55470629	1.05906887	0.501746
1526.76189	1.64408025	1.05906404	0.501781
2606.0698	-0.45008927	1.05904059	0.501949
1570.86479	3.25005215	1.05903855	0.501963
2183.07749	2.16779553	0.94025412	0.501999
1575.75505	-1.86946469	1.05897426	0.502424
1708.73271	0.27173025	0.94032235	0.502494
1790.91838	0.20897641	1.05894254	0.502652
3138.28745	3.61802522	0.9403777	0.502896
1252.61724	-0.38575331	0.94042138	0.503213
2919.34989	0.68281229	0.94044614	0.503393
2706.14433	-0.76292748	1.05882508	0.503495
2472.21073	0.90170781	1.05882332	0.503508
1396.66191	2.06029326	1.05877329	0.503867
1803.8743	0.31234464	0.94056089	0.504228
2719.17881	0.88549712	1.05870619	0.50435
3235.4282	0.67393659	0.94061529	0.504623
2195.97588	-2.28628337	0.94062166	0.50467
1856.93144	1.53790445	0.94064546	0.504843
1261.59575	0.89206368	1.05863135	0.504888
2419.23784	0.3700618	1.05858268	0.505238
1550.76421	0.68511705	0.94070527	0.505278

Information Classification: General

1732.72258	-1.82228915	1.05857095	0.505322
3549.79465	0.52074887	0.94072422	0.505416
1643.76981	0.27999235	1.05854543	0.505506
1832.90129	0.51907647	0.94074552	0.505571
3297.35849	1.17528167	0.94075435	0.505636
3503.59861	-1.22855692	1.05846701	0.506071
1717.72026	0.90069526	0.94081464	0.506075
3048.23764	-0.07620284	0.94085898	0.506398
3494.58323	-2.79295958	0.94088664	0.5066
2116.85942	-0.00647244	1.05834662	0.506939
2149.84331	-0.20000644	0.94093655	0.506963
1300.64948	-0.46866399	1.05832692	0.507081
2383.97783	0.16799732	0.94096339	0.507159
1763.87162	0.55212839	0.94098774	0.507337
2277.05864	3.02409812	1.05823903	0.507715
1931.90458	-2.47948687	0.94105444	0.507823
2445.28342	1.44403826	1.05820042	0.507994
3097.33298	-0.7465682	1.05817581	0.508171
2112.95098	3.30960501	0.9411429	0.508469
3180.36472	-0.24308189	1.0581295	0.508506
1582.76163	0.74713211	1.05812593	0.508532
2221.15297	3.71001349	0.94116439	0.508626
2505.22794	5.39436974	1.05810615	0.508675
1455.66191	1.46842691	0.94118148	0.508751
1981.86418	-0.28052329	1.05809519	0.508754
1497.67717	3.92829797	0.9411955	0.508853
1440.72283	0.16493045	1.05786381	0.510428
1353.66492	0.33636607	0.94141325	0.510444
1705.75229	0.13141013	0.94149268	0.511026
2523.2249	1.18214368	0.94149723	0.511059
2189.06133	-0.18062736	1.05776201	0.511165
3480.74521	1.7594149	0.94153236	0.511316
1849.78081	2.50841477	0.94158922	0.511732
2593.11655	0.539492	0.94162452	0.511991
2950.31229	-0.2773565	1.05760606	0.512296
1453.71238	0.73134201	0.94176938	0.513052
1860.00383	2.95885328	1.05750074	0.51306
2754.34677	1.42668129	1.0574906	0.513134
2520.08146	-3.18293252	0.94179293	0.513225
4875.39174	-0.87889578	0.94184271	0.51359
1741.82817	1.90277021	0.94184891	0.513635
2031.12778	1.72437914	0.94188289	0.513884
1874.87322	-3.63691575	1.05738348	0.513912
2783.14194	4.43476865	1.05737989	0.513939
2174.10259	1.88508181	0.94190032	0.514012
2256.06607	-0.26200855	0.94190552	0.51405
1992.90195	-1.33204935	0.94190916	0.514077
2403.14824	3.80153749	0.94191734	0.514137
2861.24485	1.81978976	1.05728825	0.514605
1700.87212	0.6171018	0.94199568	0.514712
3010.39419	-2.12449088	1.0572206	0.515097
3072.27243	0.09073241	0.94208468	0.515366
1502.75652	0.39395957	0.94210295	0.5155
2411.03208	-1.95836906	1.05712388	0.515802
2008.95078	0.00023955	0.94220179	0.516226

Information Classification: General

1675.80521	-0.48893893	1.05702431	0.516527
3070.29453	9.13608808	1.05697476	0.516889
2091.92632	2.11710612	0.94234599	0.517287
2207.12114	0.13413344	1.05688104	0.517573
3309.56174	2.14088628	0.94238555	0.517578
2440.1063	1.71634301	1.05687851	0.517591
1496.69756	0.00137929	0.94240474	0.517719
2321.0178	-4.23948373	0.94241373	0.517785
2729.28081	-2.62779208	0.94241453	0.517791
2471.02091	0.03111094	1.05683957	0.517875
1479.67082	-0.75985418	0.94243927	0.517973
2564.12729	8.56944707	1.05678505	0.518274
1788.87529	0.64310764	1.05677528	0.518345
3280.52268	-0.02350378	0.94251375	0.518521
1314.59868	-0.55902137	0.94252452	0.518601
2659.45713	-1.18891276	0.9425738	0.518964
3488.80661	3.0971743	0.94258569	0.519051
2158.88579	0.11602571	0.94259276	0.519103
4966.59742	0.03243043	1.05665155	0.51925
3651.69394	1.31871505	1.05664606	0.51929
1721.76604	2.74921771	0.94263495	0.519414
2000.92595	-1.76637268	0.94264038	0.519454
2234.00681	-0.27853331	1.05661551	0.519513
2570.24924	-1.50475862	0.94264843	0.519514
3331.51396	1.82449014	1.05661089	0.519547
2793.33213	-1.94816164	1.05649891	0.520367
2296.98594	-1.06586145	1.05639833	0.521104
2846.41762	-0.33658272	0.94287302	0.52117
2049.84184	-0.33067246	1.05637658	0.521263
2804.18424	0.56093101	0.94288904	0.521289
2793.31499	0.5154109	0.94289737	0.52135
3456.48008	1.76172179	1.05635221	0.521442
1327.6425	3.71210881	0.94292516	0.521555
1803.82732	-0.5528209	1.05633418	0.521574
3621.5687	1.21146483	1.0563265	0.52163
1548.75823	2.11008383	1.05630097	0.521817
1947.86345	3.49902302	0.94297024	0.521888
2316.05718	8.48908447	1.05627981	0.521973
2766.11496	-0.31160977	1.05627715	0.521992
2200.03781	-0.23471685	1.05620412	0.522528
1231.56816	-0.52978544	1.05617113	0.52277
1564.79656	-0.60193907	1.05616701	0.522801
1763.77311	-1.73313596	0.94309435	0.522805
2458.1323	-7.87791775	0.94311172	0.522934
935.483873	0.68198112	1.05613638	0.523025
1459.71208	3.93805849	1.05611769	0.523163
1540.78142	3.28493646	0.94314922	0.523211
2286.16001	-0.54152077	1.05607903	0.523447
2174.98807	-0.56875635	1.05607131	0.523503
3240.56504	1.97011746	1.05606173	0.523574
1747.75957	-1.75036483	0.94321335	0.523685
2774.18424	-0.1961103	1.05603478	0.523772
2148.15801	1.10247157	1.05602346	0.523855
1876.84494	0.18889164	0.9432586	0.52402
1746.65874	0.83227763	0.94327751	0.52416

Information Classification: General

2313.85259	-0.62823309	0.94328278	0.524199
2142.98984	0.1143175	1.05594254	0.52445
1967.72807	-0.50380818	0.94332328	0.524499
1457.72637	-0.47974839	1.05591787	0.524632
2051.87871	-1.36976511	1.05589774	0.52478
2485.10405	1.19641067	0.94338409	0.524949
1805.84575	7.26054468	1.05583798	0.52522
2101.10483	0.10147786	0.94342447	0.525248
2002.93686	-1.35827266	0.94343207	0.525304
3353.60739	2.25956449	1.05582481	0.525317
2577.06882	-0.80848697	1.05574801	0.525883
2916.29789	3.36228164	1.05573718	0.525962
1526.68974	-0.40311768	0.94352243	0.525974
3107.45432	-2.50027157	1.05572253	0.52607
1397.53349	3.18315403	1.05572147	0.526078
1390.66069	-1.86110819	1.05570342	0.526211
2640.40825	3.87683372	1.05554928	0.527348
1947.86174	2.11700584	1.05554603	0.527372
1198.60588	-0.27760611	0.94373363	0.52754
1695.73161	1.64924776	1.05551418	0.527607
2838.31411	-1.26401796	1.05548773	0.527802
1891.93743	4.29043177	0.94378971	0.527956
1321.62842	1.07163518	0.94384622	0.528376
1638.64055	0.23509026	0.94388438	0.528659
1572.79264	0.83824871	1.05536648	0.528698
1291.63713	-1.06511363	1.0553507	0.528815
1312.54241	1.7223803	1.05531914	0.529048
1644.82195	-1.07688689	0.94394792	0.529131
2474.99297	0.63524246	1.0552572	0.529506
2331.94864	1.33547143	1.05525234	0.529542
2629.3024	-0.42634259	1.05524468	0.529599
2932.21982	-0.3569139	1.05517658	0.530103
3029.56125	-2.47259762	0.94407908	0.530106
2538.07614	0.05779661	1.05511802	0.530536
3132.49795	-0.24446604	1.05508281	0.530797
1609.77031	0.91874637	0.94418123	0.530866
1993.06675	0.86664339	0.94419814	0.530992
1523.56645	0.57007605	0.94420311	0.531029
1661.73393	1.4653474	1.05504138	0.531104
1710.99186	0.46004977	1.05502652	0.531214
1937.13022	5.41566101	1.05495934	0.531712
2563.20537	1.49873452	0.94429756	0.531732
1577.72319	1.00323682	1.05490292	0.53213
1630.7094	2.47308606	1.05489275	0.532206
1360.70818	-1.84302445	1.05488907	0.532233
4013.81557	3.22739386	1.05486892	0.532383
1819.89969	1.289662	1.05486176	0.532436
3257.45688	-1.36824624	0.9443964	0.532468
1994.95061	0.60343458	1.05484315	0.532574
2145.99955	0.94155243	1.05482942	0.532676
929.40349	0.23098855	1.05482214	0.53273
2287.19529	0.64153559	1.05480621	0.532848
1566.69194	-0.50818542	0.94446483	0.532978
1591.70842	-2.97584517	1.05478113	0.533034
2449.13285	-0.63885781	0.94447687	0.533068

1184.54375	0.68673751	0.94448892	0.533158
1368.69011	0.59967944	1.05475018	0.533264
2029.91379	0.00796909	1.05468548	0.533745
1350.7022	0.0353418	0.94463804	0.53427
1749.84148	0.73318802	0.94464416	0.534316
1573.75669	1.57501118	1.05460858	0.534316
2353.06206	0.32724063	0.94465544	0.5344
1032.5374	1.35182936	0.94466061	0.534438
2252.8499	1.58235769	1.05457889	0.534537
2618.19487	3.29368818	1.05457517	0.534565
1477.73975	0.24661211	1.0545742	0.534572
1460.59966	-1.82687038	0.94468174	0.534596
1795.77141	-9.0793274	1.0545541	0.534722
3198.48745	0.36960087	1.05453608	0.534856
2165.88652	-5.44077067	1.05451645	0.535002
1634.77727	-2.21193531	1.05451311	0.535027
1949.96001	0.93706345	1.0544098	0.535796
3084.53798	-0.06604778	1.05440514	0.535831
1697.83232	2.67675984	1.05437475	0.536057
1848.92595	-1.22632328	0.94488107	0.536085
2023.88834	-1.26809545	0.94488685	0.536128
2527.29448	6.69367683	0.94490279	0.536247
4172.01596	4.56015228	0.94498076	0.53683
1757.79961	-1.35063995	0.9450306	0.537203
1849.96709	0.89369934	1.05421419	0.537254
3366.46792	0.47732929	0.94504914	0.537341
2795.25968	2.02822195	1.05420089	0.537353
2354.88506	-1.56310628	1.05418212	0.537493
1303.64153	2.31566031	1.05417452	0.53755
2368.22696	0.57324036	1.05415298	0.53771
3166.32492	-0.11812146	0.94510341	0.537747
1499.7498	0.01677488	1.05414505	0.53777
3361.72329	-1.25173673	0.94511053	0.537801
3659.4166	0.68037997	0.9451142	0.537828
2150.9915	0.20612674	1.05412739	0.537901
3261.54087	1.45249502	0.94514161	0.538033
1791.69023	0.09423818	1.05407929	0.538261
3690.46653	2.03593543	0.94520307	0.538493
2173.0064	-1.06904655	0.9452094	0.538541
3280.44028	-1.23445796	1.05400929	0.538783
1703.86264	1.06564671	0.94525849	0.538908
1495.76665	1.24408527	0.9452831	0.539092
2016.90459	0.68883495	0.94541997	0.540118
1953.83806	-0.56402112	1.05377553	0.540531
2434.19565	9.2007921	0.94553559	0.540985
1956.00286	2.14923814	1.05369961	0.5411
1330.74845	-0.18114747	1.05369762	0.541115
2011.92972	4.66534802	0.9456465	0.541817
3441.62326	7.64430933	0.94569167	0.542156
2657.25931	-4.89895881	0.94571462	0.542329
3012.19059	1.12430142	0.94577057	0.542749
3390.58957	1.49662071	1.05347736	0.542766
1846.72502	0.04217449	0.94578893	0.542887
1661.70122	-1.30518424	0.94579504	0.542933
3254.55027	-0.36355686	0.9458093	0.54304

Information Classification: General

1680.82285	0.05580228	0.94583357	0.543222
1356.62761	-0.79404597	0.94583779	0.543254
2074.00632	0.31459568	0.94585054	0.54335
998.517137	0.93052234	0.94587313	0.54352
1047.41936	0.67195835	1.05337308	0.543549
1697.7957	-1.15644325	0.94589789	0.543706
3068.29868	-2.56269971	0.94593587	0.543991
2358.13066	-0.58625542	0.94598296	0.544345
3677.81191	3.81695821	0.94598667	0.544373
1253.60185	0.08450799	1.05325512	0.544435
3007.4119	-2.48715957	1.05323935	0.544553
1625.8848	-1.71692524	0.94602921	0.544693
1240.60674	0.95255961	1.05316062	0.545145
2666.34024	0.47098763	1.05315708	0.545172
2859.3395	1.15479292	1.05313536	0.545335
1870.71465	0.49815621	1.05313264	0.545356
3185.52134	-5.30301836	1.05313139	0.545365
871.486742	-2.88743958	0.94613996	0.545527
2369.07895	1.48045272	1.05309706	0.545623
2795.3104	-1.01147197	1.05304828	0.545991
2802.32729	0.43996065	0.9462399	0.546279
2158.03594	-0.62975289	1.05300634	0.546306
2020.87224	-2.15516763	1.05294578	0.546763
2644.21587	1.54662533	0.94631068	0.546813
1304.57683	-0.09694691	0.94637345	0.547286
3440.40049	2.99020784	0.94640508	0.547524
1589.69853	0.68857513	1.05283129	0.547625
2252.93003	2.29990637	1.05282126	0.547701
1303.55571	-0.64794987	0.94643523	0.547751
1595.83591	-2.37347887	0.94647904	0.548082
1363.72483	2.3768232	1.05273279	0.548368
1234.53362	0.14712818	1.05267782	0.548783
1575.75591	1.47083298	0.94660625	0.549042
2892.27921	2.2071372	1.0526097	0.549298
2298.17168	0.57748433	1.05254214	0.549808
2952.33048	12.9068167	1.05252527	0.549936
3305.81953	6.82463223	0.94674657	0.550101
1554.81186	-0.82681024	0.94674691	0.550104
1566.83928	0.6335667	0.94678895	0.550421
1121.65718	-0.19676929	1.05245253	0.550486
1826.89238	5.8861065	0.94680238	0.550523
2857.19609	-0.62886106	1.05244646	0.550532
3088.59316	-0.90265874	0.94680597	0.55055
1881.97344	-4.2041132	0.94685328	0.550908
2234.05474	0.66506819	0.94686594	0.551003
2132.07474	1.51912291	0.94691309	0.55136
2541.08695	-1.19912668	0.94693917	0.551557
1496.84159	-2.27439785	1.05230414	0.551609
1141.66779	-2.49811787	0.94695604	0.551685
1142.49761	1.41872726	1.05225381	0.55199
2138.12802	-1.60627659	1.05219914	0.552404
3908.7114	-2.18138427	1.05219787	0.552414
1438.62114	-0.57273679	0.94708768	0.552681
1155.55425	1.27343005	0.94709431	0.552731
3007.33152	-1.75212278	0.94709667	0.552749

Information Classification: General

3158.5217	-15.5821843	1.05212281	0.552982
1293.64849	3.2673472	0.94712996	0.553001
2478.97173	2.79107106	0.94715099	0.55316
1165.53801	0.76030634	0.94715425	0.553185
954.489611	0.58176331	1.05204072	0.553605
1569.8029	-1.12195992	1.05200481	0.553878
1469.62195	0.72506875	1.05197771	0.554083
1956.91111	-0.44630045	1.0519578	0.554234
2786.11594	1.15200305	1.05190393	0.554643
1940.00846	-0.42850127	1.05187798	0.55484
2632.09732	-0.69948348	0.94742887	0.555266
2192.91483	2.05925655	0.94743237	0.555292
2062.94254	-0.23377522	0.94743274	0.555295
2107.91191	-2.32005277	0.9474338	0.555303
1440.73569	1.29280522	1.0517929	0.555487
2633.18828	2.06545072	1.05173788	0.555905
3701.5261	-1.22817882	1.05170704	0.55614
1244.64971	0.55236576	1.05169396	0.556239
2887.30204	0.60499575	0.9475592	0.556254
2253.92118	-1.2540676	0.94757335	0.556362
3542.50815	0.53254024	0.94757507	0.556375
2764.44522	1.3985743	1.05164839	0.556586
1690.75652	1.41180903	1.05162324	0.556777
1516.84513	-0.6094058	1.05161712	0.556824
1769.85942	-0.39873294	1.0516044	0.556921
1177.53765	0.45854585	1.05158099	0.557099
4307.99038	-8.58060151	1.05156259	0.557239
1212.62334	0.44557478	0.94771394	0.557429
1832.76921	0.77169444	1.051526	0.557518
2399.24272	-1.46918024	1.051488	0.557807
2653.0488	0.64527354	0.9477939	0.558037
2128.06913	0.60037957	0.94781375	0.558187
2204.10221	1.37865835	1.05143115	0.55824
1061.55376	5.20956777	1.051424	0.558295
2513.19339	2.37754379	0.94783472	0.558347
1622.78153	0.44922434	0.94785526	0.558503
1703.72966	-1.9448176	0.94786475	0.558575
3294.53684	-1.16555031	0.94789159	0.558779
1679.83635	0.41254595	0.94790785	0.558903
1437.7603	0.69080013	0.94793716	0.559126
2387.10294	-0.4812839	0.94795733	0.559279
4346.02065	4.64385478	1.05128853	0.559328
1224.65068	2.22436031	0.94796466	0.559335
2079.82231	-0.33468063	1.05127827	0.559406
2239.21579	1.06382319	0.94798293	0.559474
1713.83086	0.54838879	1.05125353	0.559595
1664.7625	-1.48161037	0.94802281	0.559777
2867.41762	2.28496364	1.05121934	0.559856
1363.5999	-0.93442778	1.05114717	0.560407
2727.14719	-2.03156836	1.05114281	0.560441
1676.69072	0.94419731	0.94812012	0.560518
1444.64728	-0.84299692	1.05112715	0.56056
2535.30132	2.62250091	0.94812901	0.560585
2556.2565	-0.82324332	0.94814729	0.560725
3885.57785	-6.4583247	1.05108019	0.560919

Information Classification: General

1772.80644	0.52221331	0.94818135	0.560984
1529.80815	0.98363151	0.94819447	0.561084
2313.21695	-0.70084319	1.05103691	0.56125
3002.41111	-0.39713759	1.05101432	0.561422
2387.10276	2.30489463	1.05099381	0.561579
2811.27335	-1.71869376	0.94827961	0.561732
1885.7564	-14.8322684	0.94829722	0.561867
4550.93515	4.59981972	0.94832317	0.562064
1651.86211	0.58893876	0.94834198	0.562208
1560.73442	-2.77606567	0.94836793	0.562405
2571.28813	-1.34087025	1.0508842	0.562418
1494.74468	-1.87242346	1.05086988	0.562527
1291.61699	0.42246125	1.05086947	0.562531
4165.82778	-1.42415993	0.94841219	0.562743
2483.21793	5.40482573	0.94841758	0.562784
1542.71843	0.33906888	0.94842124	0.562812
2197.127	1.18446669	1.05076779	0.563309
1586.82914	-0.19698728	0.94849614	0.563383
2584.168	2.45363395	0.94852031	0.563568
1613.79673	0.95473417	0.94852869	0.563632
1256.58977	-1.38392147	1.0507153	0.563711
2081.01201	-3.607769	0.94854419	0.56375
2074.90532	-0.17700265	0.9485661	0.563917
1988.91741	0.44129393	0.94856863	0.563936
3857.90005	-2.29844731	1.05068525	0.563942
3683.87563	1.97611381	1.05067234	0.564041
3333.35154	1.77188548	1.05066946	0.564063
2053.94878	-0.65592312	1.050667	0.564082
2414.11539	-1.10380074	0.94862613	0.564375
1366.7011	0.70396039	1.05060636	0.564547
1909.95183	0.38300721	1.05059907	0.564602
2342.05791	-2.59573988	1.05059701	0.564618
1885.96616	1.52088658	0.94866892	0.564702
2489.12143	-0.25118175	1.05051704	0.565232
3428.77256	-4.8755567	0.94873917	0.565238
3315.60605	0.9611874	1.05050768	0.565304
3133.62302	-6.66905613	1.05049284	0.565418
1996.82414	-1.4583265	1.05048528	0.565476
2075.08902	3.36472801	0.94877277	0.565495
2486.18105	-0.25634522	1.05047759	0.565535
2742.33506	-4.2858275	1.05045928	0.565675
3145.51902	-0.20416792	0.94881306	0.565803
3677.58835	2.91841348	0.94889593	0.566436
1531.66789	0.21413156	0.94891768	0.566603
2518.16489	2.35957915	0.94896256	0.566946
1622.86675	1.96659246	0.94897702	0.567056
2461.1193	-3.37303939	0.94899181	0.56717
1890.86148	-2.3682075	0.94902119	0.567394
2293.22129	0.72312854	0.94905919	0.567685
1690.7426	0.85347686	1.050177	0.567845
1956.9583	-2.44192639	0.94909832	0.567984
2500.25058	1.17394983	0.94910473	0.568034
1867.7293	0.34017008	1.05015175	0.568039
1655.81365	0.25844186	1.0501273	0.568227
2823.39804	-2.71442199	0.94915632	0.568428

Information Classification: General

2224.04642	0.74304703	0.94916479	0.568493
2664.36197	3.19450923	0.9491918	0.5687
2650.16489	-0.47853272	0.94929723	0.569508
2271.09775	3.52831026	0.94930116	0.569538
1925.88713	0.47627905	0.94930486	0.569566
3368.41001	1.90564595	1.04993878	0.569679
3188.99814	-2.0866232	0.94933406	0.56979
2008.89336	1.89798309	0.94933688	0.569811
3815.80954	5.67196278	0.94936895	0.570057
2047.86943	-0.52246281	0.94937133	0.570075
2537.19431	2.72220171	0.94938817	0.570205
1398.71196	-0.04049351	0.94939021	0.57022
1257.59661	-0.04221072	1.04981468	0.570636
3832.78921	0.39004917	1.04975292	0.571113
4832.18862	3.52863683	1.04972409	0.571335
2409.10015	4.87441546	0.94953804	0.571354
2156.09085	-0.22208487	0.94957291	0.571621
1427.70061	-1.10609872	1.04967813	0.57169
3191.32395	-0.51751983	1.04967212	0.571737
2888.27854	-3.99571216	0.94964988	0.572212
3580.55752	-3.27923147	1.04955445	0.572646
2044.01457	2.43572918	0.94970713	0.572652
2005.01286	-0.25601753	1.04955328	0.572655
1492.72684	-0.63943851	0.94971742	0.572731
1825.8333	-0.21346315	1.04953515	0.572795
2144.99375	-0.67168902	0.94975906	0.57305
3549.50022	3.42723118	0.94980688	0.573418
3690.6852	-1.52110779	1.04943711	0.573553
2514.30063	-2.34220326	1.04943151	0.573597
2431.0358	-1.27828068	0.94984716	0.573727
1843.88286	-7.64050773	1.04939521	0.573878
2293.10813	9.05980569	1.04938832	0.573931
1813.94744	2.91298081	0.94988683	0.574032
3199.49575	-1.09862781	0.94989736	0.574113
2320.0737	-4.44887335	1.04926397	0.574894
1492.71697	0.26880117	0.95001125	0.574989
1893.94621	1.16122143	1.04921403	0.575281
1173.52751	0.48411226	1.04919713	0.575412
2029.01909	3.28004183	0.95009056	0.575599
1848.90543	3.84084553	0.9501043	0.575704
2525.15146	2.7210546	1.04915766	0.575718
2103.94535	-0.57446723	1.04913464	0.575896
2601.14627	0.37371475	0.95013198	0.575917
1671.70879	-1.88577514	1.04913076	0.575926
4706.29458	-0.44003408	1.04909788	0.576181
1591.74968	-2.03041485	1.04904048	0.576626
1809.84281	-2.85312632	0.95023581	0.576717
1677.85764	0.73953402	0.95028636	0.577106
1620.75986	-1.41753726	1.04896086	0.577244
1645.7631	0.57686054	1.04895544	0.577286
2163.12632	0.80651352	0.95031535	0.577329
2937.29276	-0.00086416	1.04892178	0.577548
1994.03739	-1.03490137	1.04882185	0.578324
1990.00705	-0.07291667	1.04880392	0.578463
1688.84599	-0.50711292	1.0487519	0.578868

Information Classification: General

2379.19297	-4.95084632	1.0487311	0.579029
1526.63713	-0.58477571	1.04864822	0.579674
2382.09848	1.73735864	1.04864542	0.579696
2435.30864	2.31882261	1.04861532	0.57993
2116.9625	1.79560632	1.0485607	0.580355
2380.12387	0.18536194	0.95071334	0.580398
1242.62346	1.82306609	0.95073021	0.580528
2541.21933	0.61709303	0.9507335	0.580554
3580.78012	-0.66516668	0.95073638	0.580576
2549.16811	-1.73341795	1.0485302	0.580593
2569.02573	0.83675811	1.04852887	0.580603
1802.02402	2.82441782	1.04852586	0.580627
1833.89847	0.41242869	0.95074564	0.580648
2018.92508	-0.99326099	1.04852003	0.580672
2558.03862	-0.4352876	0.95076067	0.580764
939.442675	0.96786307	0.95077357	0.580863
1666.81217	3.03731968	1.04848209	0.580968
2063.02365	0.1887801	0.95080956	0.581141
1140.59075	0.26963303	0.95082766	0.581281
2901.36582	5.21807476	0.95084063	0.581381
2184.17827	1.05117564	1.0483934	0.581659
1569.84098	3.02911902	0.95090474	0.581876
1453.60771	-0.02179792	0.95092293	0.582017
2563.23345	-3.3209085	1.04832273	0.58221
2566.24125	3.73807017	0.95095485	0.582264
1814.91557	1.90457482	0.95097396	0.582411
1863.93491	0.7802529	0.9509982	0.582599
2065.84331	2.84243121	1.04823901	0.582863
2114.05277	2.06722718	0.951034	0.582875
2991.4971	-0.93492825	0.95106923	0.583148
1201.50054	-0.16597579	0.95107635	0.583203
1379.61614	1.01562014	0.95116937	0.583923
3512.50595	4.97060011	1.0480767	0.58413
2278.05083	0.60426604	0.95122511	0.584354
1691.84075	1.46204124	0.9512551	0.584586
1217.61719	0.94925957	0.95126511	0.584663
2462.16373	2.19934253	0.95127576	0.584746
3002.49918	5.43159521	1.04799516	0.584768
1480.6624	2.2731815	1.04797917	0.584893
2090.9899	0.61885349	0.95129779	0.584916
1766.88713	-3.24058068	0.95132636	0.585138
4400.01377	-9.39388822	1.04794236	0.58518
1772.79013	-0.16474031	1.04790918	0.58544
1530.73979	-2.99502732	0.9513889	0.585622
2353.16745	1.4768545	0.95139452	0.585666
3214.46152	0.90580296	1.04787412	0.585714
1198.59502	0.0358769	0.95140595	0.585754
2894.19559	-0.50947042	1.04786106	0.585816
4390.9885	1.58055705	1.04785989	0.585826
2332.0759	2.64251758	0.95142894	0.585932
2917.46061	1.4348579	0.95144466	0.586054
3537.73569	-0.86140109	1.04782425	0.586105
1907.91245	-2.24884369	1.04779029	0.58637
2528.17893	0.68266344	1.0477774	0.586471
1489.83835	-0.54319756	0.95150617	0.586531

Information Classification: General

2457.16312	-2.0451268	0.95152831	0.586702
2314.15239	2.8187637	1.04774552	0.586721
3523.59336	-0.82710842	0.95155311	0.586895
2424.23801	-1.43883291	1.04771891	0.586929
1476.6729	-1.67006434	1.04770462	0.587041
2970.35282	-1.12038695	1.04768859	0.587167
2033.03043	3.69958071	0.95158967	0.587178
2575.1832	-3.27652174	1.04767857	0.587245
2385.08951	8.02963214	0.95159922	0.587252
1389.73479	-0.22485608	1.04766528	0.58735
1720.7697	-0.38783861	0.95165417	0.587678
2979.56362	0.28461844	1.04761794	0.587721
2875.33902	-4.49890943	1.04761337	0.587756
2535.07303	0.05636888	1.04760614	0.587813
1423.66825	-1.19287414	1.0475783	0.588031
1488.70244	4.00676234	0.95169983	0.588032
2210.05661	2.62909201	1.04757162	0.588084
1765.73076	0.44468239	0.95171943	0.588184
1513.75115	0.62621361	0.95173873	0.588334
2774.26128	1.16527146	1.04752211	0.588472
2166.98564	1.41579243	1.04751152	0.588555
1526.74419	-0.81878566	0.9517693	0.588571
1927.93779	-1.38000336	1.04750168	0.588632
1848.9014	1.65668101	0.95180351	0.588837
2654.35453	3.2087047	0.95180465	0.588846
2367.1395	-1.58791471	0.95181981	0.588963
1754.68633	-0.67727454	0.95183249	0.589062
1214.62566	-1.28463422	1.0474465	0.589065
3564.57712	0.77968707	1.04744444	0.589081
1242.58489	0.06532024	1.04741618	0.589303
2168.26114	-1.45865697	0.95186465	0.589312
2939.20098	0.06465469	0.95189888	0.589577
1865.8425	-0.22718206	0.95190039	0.589589
1685.76372	5.77789974	0.95190844	0.589652
1472.72734	0.42589053	1.04736849	0.589678
1590.71916	1.3946583	0.95192557	0.589785
1168.63144	-1.31389264	1.04734572	0.589857
3187.47724	0.51634761	1.04734464	0.589865
2545.31321	0.48529944	0.95194437	0.589931
1921.91545	0.37700524	1.04733607	0.589932
1054.50444	-0.60992631	0.95198677	0.59026
1387.65691	2.72808811	1.0472816	0.59036
2487.21693	-1.208618	1.04727561	0.590407
2010.79497	0.32334164	0.95200576	0.590408
3163.52085	0.10995364	1.04726816	0.590466
1797.87346	-1.0037499	0.95201465	0.590477
1654.83147	3.88148117	0.95202027	0.59052
1665.63567	-0.60972761	1.04726017	0.590529
1973.98386	0.74039913	1.04725387	0.590578
2243.08408	-3.04167711	1.0472426	0.590667
2801.35586	-2.33695163	1.04722217	0.590827
2033.04595	-0.65087744	1.04722081	0.590838
1615.74639	0.54290041	1.04720861	0.590934
2796.11406	1.62796314	0.95207689	0.59096
3113.60813	-1.34599017	0.95209722	0.591118

Information Classification: General

1898.83733	0.56485733	0.95210493	0.591178
1701.69402	1.51144994	0.95211874	0.591286
2547.05034	-3.48824378	0.95213523	0.591414
2325.03716	0.28028531	0.9521466	0.591502
3733.69919	1.03478266	1.04707632	0.591974
1164.60047	-0.22994843	1.04706188	0.592088
3023.27652	-3.86828322	0.95226838	0.59245
2430.25211	0.02511989	0.95227111	0.592471
3610.57358	0.54457154	1.04700345	0.592548
1147.4904	0.19062823	1.04697302	0.592788
2500.14702	-6.11085863	0.95233649	0.59298
1496.68645	-2.60330684	0.95243375	0.593737
1570.90006	2.52738319	0.95248649	0.594147
3398.54477	2.29616568	1.04678125	0.594299
1782.85552	0.57831313	0.95251643	0.594381
1319.67876	-4.78128765	1.04670892	0.594869
3455.43601	1.58734626	1.04668016	0.595096
2816.02988	-1.01808446	0.95262595	0.595234
2699.21408	-1.76675674	0.95266994	0.595577
2009.82817	2.43468512	1.04661197	0.595634
1840.67766	-1.09003146	1.04661171	0.595636
1625.86357	0.68990375	1.04659837	0.595742
4613.29067	2.13596339	1.04658973	0.59581
2107.94756	1.66420856	1.0465753	0.595924
2299.95268	-0.88914112	1.04655524	0.596082
2245.04668	-0.40526635	0.95273829	0.59611
3038.47629	5.39190535	0.95274752	0.596182
2486.26993	1.85430923	1.04653803	0.596218
3753.66318	0.7011351	0.95275667	0.596253
3092.39682	-0.38403767	1.04653048	0.596278
1893.76641	-0.00701215	0.95276697	0.596334
1930.85332	2.38225404	1.04650708	0.596463
1224.61113	0.41342359	1.04649217	0.596581
1788.77178	0.71043964	0.95281824	0.596733
2689.28702	-1.19616945	0.95283242	0.596844
1592.70488	1.90825253	0.95283625	0.596874
2531.20182	1.17496712	1.04645097	0.596906
1316.57659	0.9725106	0.95289114	0.597302
1320.54619	-0.39841327	0.95289505	0.597333
3410.60439	-0.61498686	1.04639581	0.597342
2078.13242	1.64142873	0.9529049	0.59741
1541.74565	0.34685845	0.95292233	0.597546
2328.9968	-1.35638269	1.0463614	0.597614
2085.03862	4.58673398	0.95296058	0.597844
2028.01719	-11.5498374	0.95296822	0.597904
1044.55803	-0.03571157	1.04632346	0.597914
1721.93474	2.06667621	1.04631954	0.597945
2964.34	1.97503412	1.04630734	0.598042
2638.15775	0.18258806	0.95299888	0.598143
3135.33982	1.28457119	0.95304981	0.598541
2264.15458	-1.35420614	1.04623836	0.598588
1488.66252	-1.28243661	0.95308693	0.598831
1806.85984	0.58006627	1.04618932	0.598976
1401.76023	-0.30053397	0.9531258	0.599135
1665.80967	0.53259386	0.95319063	0.599641

2644.19529	0.98302769	1.04610424	0.59965
2032.90874	-0.80145998	1.04599513	0.600515
2063.90703	2.48980238	0.95331592	0.600621
3058.39707	1.79033122	1.04597304	0.60069
3422.57956	-2.87074088	1.04594441	0.600917
2505.262	2.0727345	1.0459435	0.600924
1906.85942	-2.09019144	1.04593352	0.601003
3161.40122	3.79038709	0.9533717	0.601057
1896.85881	-1.65328742	0.95340386	0.601309
1857.96933	-1.13286652	0.95341577	0.601402
1925.83683	-0.01669688	0.95342446	0.60147
2975.37705	1.42805851	1.04586669	0.601534
1995.03769	-1.29093112	0.95345665	0.601722
2711.00371	-2.00132219	1.04582361	0.601875
1494.80351	-0.98401185	0.95350033	0.602064
1170.57085	-0.19385414	0.95352234	0.602236
1534.69805	4.75686122	1.0457716	0.602288
1662.83329	-0.02958476	1.04575809	0.602396
2068.95757	0.89505461	1.04574908	0.602467
1585.84807	2.49819624	0.95358081	0.602694
2081.11466	1.87393605	0.95360855	0.602911
2504.10184	-0.6487384	0.95362013	0.603002
1399.7177	-0.57106805	0.95362912	0.603072
1239.69536	0.09018244	1.04566345	0.603148
1438.66313	0.03352852	1.04566236	0.603156
1807.78093	-0.18083617	1.0456529	0.603231
2917.34067	-0.05619523	1.04560528	0.60361
3005.25748	-3.41995941	1.04559721	0.603674
1538.80437	-1.08942515	1.04553952	0.604133
1476.66108	0.66373532	0.95378146	0.604266
2368.22586	0.12200292	1.0455165	0.604316
2385.07029	-1.48116508	0.95379016	0.604334
3035.42008	2.66842725	0.95379113	0.604341
1649.90959	-0.76477088	0.95381401	0.604521
2020.9384	-0.08075905	0.95382613	0.604616
2017.0258	1.05062349	1.04547728	0.604628
1560.69907	-0.5033021	1.04545381	0.604815
980.505052	0.35408442	0.95385249	0.604822
2528.18259	1.15577281	1.04545105	0.604837
2533.28862	3.4486862	1.04544323	0.604899
1568.74626	0.93394266	1.04543373	0.604975
1940.00551	3.14719209	1.04542487	0.605045
1309.60417	-0.21054048	0.95389614	0.605165
1504.8476	-1.00970383	1.04540728	0.605185
2359.18302	-1.97388214	1.04536919	0.605488
3427.8021	-0.44873051	0.95394111	0.605517
2070.76225	-1.77792984	0.9539457	0.605553
2643.28617	-2.64418406	1.04535662	0.605588
3120.34604	1.64505893	1.04534948	0.605645
1708.83745	-0.80484981	0.95396489	0.605704
2805.31328	-3.56868498	0.95399593	0.605947
1225.62126	0.1126931	1.0452852	0.606157
2588.28421	-1.91148257	1.04528295	0.606175
2792.21866	-2.62308181	0.95402894	0.606206
1714.74687	3.32146436	0.95407343	0.606556

2361.11643	-0.49557263	1.04523082	0.606591
3551.51291	-1.28275746	0.95408512	0.606647
3103.42563	2.76275248	0.95408767	0.606667
2364.06278	2.54772971	0.95410324	0.60679
1810.89519	0.18107084	1.04517063	0.607071
2673.21549	-1.90783202	1.04513587	0.607348
1683.90203	-1.35765748	1.04511757	0.607494
2829.23955	2.3530181	0.95422184	0.607721
1845.85845	3.53425598	1.04506979	0.607875
2187.12236	-2.15317085	1.04504647	0.608061
4119.96303	0.95610112	0.95426869	0.608089
2719.05924	-2.44939353	1.04500313	0.608407
3228.54775	-1.08349083	1.04499857	0.608443
3114.39116	0.75635329	1.04495795	0.608768
3452.56089	6.30051253	0.95439822	0.609107
2623.27475	-5.63986268	0.95440127	0.609131
3291.65219	4.30771419	0.95441576	0.609245
2720.19577	-0.15234255	1.0448904	0.609307
1821.80632	8.31192242	0.95442398	0.609309
3763.44907	6.93127405	1.04484551	0.609666
1724.69914	2.05773769	0.95448666	0.609802
3306.41526	0.94544093	1.04482358	0.609841
3782.57187	1.96671247	0.95450411	0.609939
2453.08298	0.67084143	1.04479295	0.610086
1653.86551	3.06818658	1.0447926	0.610089
2308.99436	2.58795904	0.95455653	0.610352
1199.57817	-0.68582546	1.04475653	0.610377
3253.25241	0.53645497	0.95456648	0.61043
3786.55515	1.75954084	0.95457088	0.610465
2184.97881	3.39960477	1.04466526	0.611107
2071.94583	2.10895165	1.04465674	0.611175
1913.94193	-2.14197534	0.95466227	0.611184
2042.10149	-0.12549404	0.95469366	0.611431
2072.96025	-1.20003416	0.95470902	0.611552
1880.70976	3.08877836	0.95474597	0.611842
1098.53252	1.10420206	1.04455705	0.611973
1089.57978	-0.14566718	1.04454749	0.61205
1600.72014	-4.97771447	0.95479039	0.612192
1573.72734	2.30295333	1.04450943	0.612355
2266.98906	0.7728183	1.04450458	0.612393
2897.37357	1.33949196	0.95482123	0.612435
2219.0081	4.27170111	1.04447548	0.612627
1240.63263	0.49791476	0.95486931	0.612814
3106.50693	1.69187615	1.04437105	0.613464
2154.94341	0.12467787	0.95496585	0.613575
1714.86234	-1.88904968	1.04434783	0.61365
3162.35593	2.59042582	1.04433264	0.613772
1591.84586	0.67911579	1.04432416	0.61384
3481.83339	2.42987772	1.04425838	0.614367
1204.57109	-1.72906306	1.04424463	0.614478
1902.04355	0.20920591	1.04422017	0.614674
1477.66265	-1.75279859	1.044208	0.614771
2704.24233	-3.61359026	1.04420515	0.614794
2797.35634	5.26198536	1.04416485	0.615118
1983.86431	-3.17052368	1.0441647	0.615119

2014.92679	2.90515014	0.95518297	0.615287
1377.70403	2.30222956	0.95520739	0.615479
1956.92217	3.47200287	0.95521867	0.615568
997.521288	1.28572005	1.04406327	0.615934
2219.92636	0.61642529	0.9553268	0.616422
1804.88164	-0.95236404	0.95535312	0.61663
1570.77774	1.30242059	1.04397535	0.61664
1615.74724	0.24241512	1.0439618	0.616749
2726.10698	2.089387	0.95539089	0.616928
2164.92972	1.48104223	0.95539384	0.616951
1401.7072	4.54133292	0.95540234	0.617018
2337.14536	1.69033533	0.95540813	0.617064
2594.30179	0.91880785	1.04391458	0.617129
2827.31815	0.30314477	0.95545012	0.617396
2118.89554	-2.07812753	1.04386812	0.617502
1898.8859	-0.22639056	0.95547325	0.617578
3689.62204	10.2750713	1.04382139	0.617878
1849.79935	1.60788174	0.95552982	0.618025
2890.32511	-3.65145964	0.95553394	0.618058
1503.62847	0.50761709	0.9555941	0.618533
1317.63286	0.57284451	0.95560995	0.618659
2761.40036	5.20585221	0.95561225	0.618677
1520.65593	-0.91665193	1.04367489	0.619058
1293.64702	2.1272843	0.95567572	0.619179
2561.34795	0.14940937	1.04365755	0.619197
1962.97226	-2.26806249	0.95570431	0.619405
3847.65708	-0.40951951	0.95572138	0.61954
2630.23516	1.96663808	1.04359503	0.619701
1458.64678	-1.88475076	1.04359376	0.619711
1528.6386	-0.77709444	0.95574395	0.619718
2140.00064	1.14916036	0.95575059	0.619771
2757.2695	-0.915374	1.04355932	0.619989
2065.00737	1.89515909	1.04352659	0.620253
1992.06023	0.17567883	1.04350433	0.620432
2755.09549	7.000787	1.04349573	0.620502
3767.83188	-5.42089025	1.04348243	0.620609
1567.70378	-0.11695429	1.04348132	0.620618
1990.80351	1.96357682	1.04347937	0.620634
2819.16996	-0.51088536	1.04345693	0.620815
3175.34836	1.45511249	0.95588738	0.620853
3438.72085	-1.3334606	1.04341972	0.621115
2121.06499	-0.22125442	1.04339837	0.621287
1993.86492	1.39450912	0.95594339	0.621296
2928.3563	1.19079068	1.04337376	0.621486
1518.71538	4.85366126	0.95597632	0.621557
1649.63945	-1.40115731	0.95597807	0.621571
1946.94573	1.25068185	0.95599922	0.621738
1905.83281	-1.07251243	1.04333175	0.621825
1735.81902	2.73589888	1.04331672	0.621946
1402.67119	1.13702833	1.04328211	0.622226
3461.63742	-2.25153879	1.04326228	0.622386
3570.5676	-0.02387007	1.04325748	0.622424
2466.07724	-5.30870435	0.95613132	0.622785
1444.679	-0.80476225	1.04316662	0.623158
2405.24778	-0.03552584	1.04316266	0.623191

2603.99028	-0.67754717	1.04316222	0.623194
4034.97915	-4.98431758	1.04315985	0.623213
2860.28006	-5.79273722	0.95629396	0.624074
1380.68937	-1.58116157	0.95629567	0.624088
2985.353	2.27616665	1.04304795	0.624118
2371.228	0.80202566	1.04303997	0.624182
2215.0391	-0.46439255	1.04302967	0.624266
2138.01432	2.96727616	0.95632241	0.6243
1681.78411	-4.9786914	0.95632886	0.624351
1529.74134	-0.8208299	0.9563541	0.624551
2021.9832	10.2901921	1.04297524	0.624706
1960.88786	3.79148736	1.04296669	0.624775
4575.01096	1.13401812	0.95641072	0.625
1761.96714	0.87850851	0.95643379	0.625183
2197.03489	-0.1443188	0.95643741	0.625212
2730.28452	-0.48320399	1.04290538	0.625272
3060.51963	-0.25056611	0.95645086	0.625319
2090.99346	-1.59517387	0.95647019	0.625472
3128.44846	-4.80680915	1.04281043	0.62604
2010.72185	0.57892471	0.95654247	0.626046
2357.04448	0.53240086	0.9565867	0.626397
2775.34732	1.1479253	1.04273043	0.626689
3292.38974	3.77668683	0.95663789	0.626803
1749.87888	-0.99774495	0.95665312	0.626924
4662.24233	2.44710065	0.95668912	0.62721
3344.49717	-1.96735105	1.04265623	0.62729
1624.75456	1.39399535	1.04265271	0.627319
1217.55722	0.87699677	1.042642	0.627406
1868.94889	0.89268969	0.9567207	0.627461
2267.96756	1.62839596	0.95672672	0.627509
1492.74455	3.07228365	1.04262925	0.627509
1545.66826	-1.32939951	1.04261425	0.627631
3737.70407	4.60881315	1.04261354	0.627636
1026.54863	1.09423274	0.95680093	0.628098
2269.91411	-0.08599499	1.04255569	0.628106
3048.35868	2.06485888	0.95680227	0.628109
1139.6041	-1.20761785	0.95680655	0.628143
2317.11344	3.00769593	1.04253813	0.628248
1684.72148	1.80910949	1.04253197	0.628298
3690.67453	2.16176311	1.04250056	0.628553
2277.16811	0.67330394	1.04248473	0.628682
3359.57949	1.96518028	1.04246092	0.628875
1788.74297	1.30942269	0.95690369	0.628915
1767.72087	0.97158668	0.95690365	0.628915
3259.5062	-0.20481534	1.04242366	0.629177
3083.26133	-2.65421672	0.95696585	0.629409
2239.00998	-2.21634438	1.04238172	0.629518
1396.70781	0.15964014	0.95699512	0.629642
2422.20169	0.99992028	1.04234535	0.629814
2486.28696	3.48597005	1.04232924	0.629944
2618.0372	-1.95747314	0.95703684	0.629974
2223.86844	0.00766928	0.95705288	0.630102
1400.69328	-0.76419054	1.04229758	0.630202
1641.79387	1.71918107	0.95710705	0.630533
1322.69548	-0.3675305	1.04225646	0.630536

1346.63066	3.0257243	0.95710814	0.630541
1952.11173	0.25622933	0.95712718	0.630693
1241.62029	-0.06076208	1.0421906	0.631071
2744.20584	10.3076813	0.95722691	0.631487
2650.26725	2.34495839	1.04207771	0.63199
2873.45376	0.62807954	0.95729794	0.632052
1841.8792	1.55422211	1.04199019	0.632703
3033.41709	1.86689625	1.04197239	0.632847
1925.79473	3.33370584	0.95739789	0.632849
2540.22983	-5.97339035	1.0419714	0.632856
1567.75151	3.45535785	1.04196687	0.632892
2933.27121	0.28911461	0.95740511	0.632906
2277.00395	-0.07529686	1.04194305	0.633086
4085.74729	-4.66150458	1.04193565	0.633147
1277.64629	-2.39539449	0.95745047	0.633268
2975.31972	0.02068832	0.95746926	0.633417
2239.07334	-0.04060832	0.95749051	0.633587
1985.8991	0.86416793	0.95749225	0.633601
1578.71807	-2.87772923	0.95750741	0.633721
1449.71721	-3.92325846	1.04185028	0.633842
1179.60063	-0.59745962	1.04183283	0.633985
1825.84014	2.95052632	0.9575529	0.634084
3888.77016	-1.87179807	0.9575619	0.634156
1936.93576	-0.57005875	1.04180964	0.634174
1775.93437	-5.08381926	1.04177778	0.634433
4133.86391	-0.23924167	1.04175456	0.634623
2925.36472	2.40308871	0.95763464	0.634736
1567.6514	0.46313858	0.95764144	0.63479
3228.38303	2.87898237	1.04171848	0.634917
1677.79558	6.08806307	1.041705	0.635027
3259.56626	2.44572322	1.04170242	0.635048
3242.37302	-0.66719893	0.95769417	0.635211
1827.81975	0.56977565	1.04167928	0.635237
1713.84903	-0.08467736	0.95770156	0.63527
4022.82676	-3.96312275	0.95771318	0.635362
1446.7389	2.38739439	1.04164452	0.635521
4121.84194	-1.63001125	1.04161984	0.635722
1427.70391	1.20243464	0.95777017	0.635817
2946.33121	-0.50924005	0.95783057	0.636299
1285.58965	-0.75303335	0.95783821	0.63636
1977.9699	2.40697878	1.04153494	0.636415
3447.55698	4.70061937	0.95786272	0.636556
1587.80143	-0.45757995	1.04150863	0.63663
2250.99834	0.34087736	0.95787289	0.636637
1149.47038	0.81708309	0.95787562	0.636659
1748.86943	-0.39050121	1.04149242	0.636763
2695.15654	2.45382384	0.95790346	0.636881
2501.21317	4.57522698	0.95791647	0.636985
2440.19767	6.88619964	1.04141508	0.637395
1988.9666	1.15036277	1.04138884	0.637609
2608.16251	1.74162395	0.95799867	0.637641
1288.55766	2.92535317	1.04133833	0.638022
1736.84477	-4.91013295	0.95804658	0.638024
2435.11008	0.20994495	0.95805113	0.638061
1774.96213	0.03212481	1.04133306	0.638065

Information Classification: General

1829.82146	-3.96025984	1.04131235	0.638235
3004.29929	0.75266251	1.04127044	0.638577
2268.99516	4.01344454	1.0412605	0.638659
1280.69547	-0.91323611	0.958151	0.638859
1711.86197	0.04341992	0.95817177	0.639025
1294.59136	-0.41874797	0.95817465	0.639048
1387.66399	-0.72083125	0.95817499	0.63905
1793.80681	1.29445047	1.04118515	0.639275
2088.01865	-1.73438345	0.95821179	0.639345
1733.72026	-3.6360292	1.04116028	0.639479
3407.74765	4.02772818	0.95824048	0.639574
2112.10537	2.64952957	0.95824432	0.639605
2452.10428	-1.58888054	0.95825366	0.639679
1818.89756	-0.27993605	0.95825696	0.639706
1946.93157	-1.34013265	1.04112482	0.639769
2287.10039	1.19981424	1.04112174	0.639794
1800.88506	-0.60966446	0.95831413	0.640163
2606.1467	1.79703346	0.95832314	0.640235
1463.71111	3.99796725	0.95833267	0.640311
3579.49509	-3.06551484	1.04104289	0.64044
2793.32242	2.22586108	1.04101593	0.640661
2260.10869	-1.99774601	0.95839293	0.640793
4433.94985	-2.77077841	1.0409953	0.64083
1785.71976	0.76238998	0.95845966	0.641327
3420.53794	5.91885977	1.0409118	0.641515
1762.94345	5.18239112	1.04090941	0.641534
2288.03611	0.94693859	1.04088631	0.641724
2404.15825	5.69530884	0.95853304	0.641915
3218.56491	-0.10712121	1.04086273	0.641917
2782.14457	1.15957394	1.04086165	0.641926
1306.61516	-0.7235463	1.04086105	0.641931
1783.88078	-1.25198757	0.95857891	0.642282
1356.59599	-0.23104567	1.04076452	0.642723
3501.55698	2.10096628	0.95865171	0.642865
3759.71628	3.48494672	1.04074468	0.642886
1591.72405	-0.20837645	0.95867161	0.643024
3446.39426	-1.62345716	1.04065582	0.643615
3430.60439	-0.26539839	0.95875041	0.643656
2400.18344	0.76473191	0.95876859	0.643801
2007.94695	4.573683	0.95878114	0.643902
2852.21427	1.66405828	1.04061811	0.643925
2430.11808	1.03774568	0.95878983	0.643972
2215.1397	1.14247253	0.9588023	0.644072
2023.79155	-0.72971695	0.95881073	0.644139
1445.81275	0.50454348	1.04058971	0.644158
2523.15012	0.42815588	1.04058241	0.644218
2217.08988	-0.24374188	0.95883437	0.644329
1605.77825	1.72535713	0.95883814	0.644359
1099.52825	1.53751479	0.95884412	0.644407
4695.40876	1.98798473	0.95887443	0.64465
1906.87212	0.85617239	0.958875	0.644654
3446.63742	1.13443585	1.04049595	0.644929
1942.80681	1.14885024	0.95893626	0.645146
2110.07004	0.70290395	1.04045813	0.64524
2949.39438	-2.23300185	1.04043032	0.645469

2383.16562	2.05618577	1.04041361	0.645606
1572.68242	1.66679909	0.95899556	0.645621
1440.73734	2.43662724	1.04038686	0.645826
1893.05637	-0.40236437	1.04037877	0.645893
3519.03163	2.98059135	1.04036807	0.645981
2734.17679	0.27198857	1.04029992	0.646542
2092.04595	0.25703845	0.95911155	0.646552
3051.41494	-0.78802848	0.95912928	0.646694
2668.24198	0.76762593	1.04027402	0.646755
1537.70525	-0.8782606	1.04024976	0.646955
2945.29483	0.05726321	1.04024905	0.64696
1942.98392	2.03170549	0.95918011	0.647102
1415.71697	0.33286601	0.95918522	0.647143
2069.08811	-0.07753038	0.95918655	0.647154
1844.77812	0.0208538	1.04020911	0.647289
2329.96782	-0.36535429	0.95922941	0.647498
2099.13173	0.69232107	1.04017354	0.647582
1910.99887	0.46372836	1.04015458	0.647738
3415.62082	2.04279893	0.95928794	0.647968
1283.52751	0.12319102	0.9592997	0.648062
1172.58098	0.61566404	0.95933937	0.648381
1921.85959	-2.03607079	0.95934621	0.648436
1587.76824	-1.02170118	0.95937418	0.648661
2331.07102	-1.48869808	0.95938798	0.648771
2659.37528	1.58157181	0.95940725	0.648926
2652.12723	0.3352471	1.03999761	0.649032
1871.01505	4.59456528	1.03998504	0.649136
2023.91191	0.11044607	0.95943701	0.649165
2668.30312	1.27384024	0.95944664	0.649243
1554.7636	-0.62896801	0.9594602	0.649352
1652.77703	0.48998613	0.95949277	0.649613
2722.45292	4.00620766	1.03992646	0.649619
2936.29319	-5.23459042	1.0398869	0.649946
1423.61101	0.67659832	0.95955204	0.65009
1665.75968	-0.31672814	0.95957425	0.650268
1724.86667	1.23721791	1.03984318	0.650306
3632.79966	1.40234388	1.03980189	0.650647
1112.50981	-0.68500632	1.03977413	0.650876
2296.19547	0.57940305	1.03975817	0.651008
3160.46665	0.74660553	0.9596686	0.651027
1779.78862	-2.34717901	1.0397539	0.651044
3333.57949	0.836992	0.95967541	0.651082
1699.87432	-0.88425545	0.9597133	0.651387
1491.74553	-0.09405278	1.03968972	0.651574
3292.55783	1.84389937	1.03966885	0.651746
4458.14649	1.28648409	0.95977173	0.651857
1525.81438	-0.28944973	1.03962682	0.652094
2593.2786	5.14264278	0.95983566	0.652371
1910.88664	0.04133078	0.95990925	0.652964
1951.81609	-0.87183531	1.03949422	0.65319
2703.11284	0.76847236	1.03949042	0.653221
2514.14536	2.29603247	1.0394745	0.653353
2385.93657	1.32557432	1.03946423	0.653438
2481.0723	0.88206915	1.0394288	0.653731
2415.25412	-0.94598418	1.03941278	0.653864

Information Classification: General

3952.7761	2.48631944	0.96002597	0.653904
2627.02568	-1.06429703	1.03938816	0.654068
1925.92143	0.28870207	1.03933079	0.654543
2800.16635	1.36979473	0.96010545	0.654544
2232.10398	1.36898795	1.03931758	0.654652
2200.96831	0.51341834	0.96015712	0.65496
1209.61321	1.86132098	0.96016226	0.655002
3412.53806	4.08896676	1.03925545	0.655166
2761.21231	0.92898535	0.96021042	0.65539
2551.13523	-0.06321757	1.03920344	0.655597
2146.00029	-0.27192942	1.03917987	0.655793
1838.93852	-0.33396372	1.0391778	0.65581
3421.43808	2.51682729	0.96026949	0.655866
4099.99819	2.25545252	0.9602729	0.655893
2969.41715	1.30669083	1.03915215	0.656022
1894.95938	2.55912518	0.96030046	0.656116
2861.28641	1.98715006	1.03913819	0.656138
1834.85295	1.4303992	0.96030366	0.656141
2988.32242	-0.48068854	0.96030368	0.656142
2113.07109	1.98962573	0.96034097	0.656442
2773.19803	-1.8437561	1.03906593	0.656737
1921.93389	-1.01606545	1.03904214	0.656935
3632.80312	0.42363757	1.03902372	0.657088
1470.66887	-1.22696489	1.03901913	0.657126
1995.13811	0.35828206	0.96043084	0.657167
1613.69805	0.33918714	1.03900821	0.657216
2502.22637	1.54049137	1.03900578	0.657236
3081.39511	2.82093611	1.03898874	0.657378
2413.10381	1.50099816	0.96047459	0.65752
2257.06107	-0.79705039	1.03894752	0.65772
1938.92705	4.7367702	1.03894318	0.657756
2490.01792	-1.4649882	0.96050595	0.657773
1535.78464	-1.0139534	1.03892102	0.65794
1717.8488	-0.68447144	1.03891433	0.657995
2972.36515	0.48249692	0.96058392	0.658403
3210.52658	1.2269468	1.03883072	0.65869
1768.91947	-1.89808183	1.03881251	0.658841
2582.25949	-0.67448622	0.96067112	0.659107
3019.17057	1.14272408	1.03871135	0.659681
1722.75774	0.70975066	0.96078923	0.660061
1956.01572	2.10217508	0.96081569	0.660274
4057.76877	4.71999426	1.03860594	0.660558
2284.96048	-3.642045	1.03859322	0.660664
2610.26163	1.05335496	0.96088204	0.66081
3266.70278	3.22288121	1.03850891	0.661365
1620.67436	1.24497866	1.038418	0.662122
1156.55106	-1.99364915	0.96105021	0.66217
3413.54844	1.58361053	0.961079	0.662403
2142.97263	0.63933023	0.96108694	0.662467
1393.63603	-4.0120756	0.96110378	0.662603
3012.29862	1.9014837	0.96112584	0.662782
2399.15996	0.72584078	1.03833268	0.662832
2026.03362	-0.60283566	0.96118245	0.66324
1353.69914	-1.95318256	1.03827857	0.663283
1220.54314	0.16641695	0.96119105	0.663309

2314.17862	-0.40415224	0.96121283	0.663485
2347.16159	0.73659647	1.03823774	0.663623
1012.47423	0.82414706	0.96123068	0.66363
1450.7094	1.68874944	0.96129057	0.664115
3350.77924	-0.87090208	1.03815828	0.664286
1655.69463	1.6744494	1.0381245	0.664568
2014.99199	0.39139517	0.96134766	0.664577
3616.6904	0.55451379	1.03811525	0.664645
3263.49021	-0.36580708	0.96136831	0.664744
3019.4357	-1.03834049	1.03809864	0.664783
2191.95049	-0.3915454	1.03807247	0.665002
1406.7298	1.04689475	0.96141961	0.665159
1088.53727	0.19319289	1.03803847	0.665285
3071.65542	5.61844068	0.96146737	0.665546
2727.16806	-0.45151691	0.96150193	0.665826
1458.63982	1.0302794	1.03797172	0.665842
2896.3881	-0.32890075	1.03795562	0.665977
4713.131	-1.35433298	0.96153898	0.666126
2570.21976	-5.85575809	1.03793251	0.66617
2859.33011	0.42456705	1.03790076	0.666435
3588.68882	12.0998484	0.96159049	0.666544
2533.16265	1.9698518	1.03788065	0.666603
2982.29386	2.23388266	1.03787504	0.66665
1655.68315	-2.89381002	0.96161356	0.666731
4179.83457	-2.96912618	1.03785406	0.666825
2843.40909	-0.59665367	1.03783834	0.666956
1372.71233	0.50962767	1.03780271	0.667254
2459.12271	-1.27829472	0.96169742	0.66741
3821.74326	1.63824453	0.96171619	0.667562
2843.27451	-0.19169799	0.96174503	0.667796
1271.68413	-0.73813141	0.96175997	0.667917
1498.79509	0.50923843	1.03770786	0.668047
2001.87851	-1.27187726	0.96177861	0.668069
2612.22599	-5.93440204	1.03769675	0.66814
3697.77427	2.65830434	0.96186107	0.668737
943.473497	0.46848776	1.03762168	0.668768
2061.76494	0.46798588	0.96188096	0.668899
3069.29862	-0.69630347	0.96190068	0.669059
3055.54106	2.97784371	1.03756118	0.669274
1701.80967	0.5213274	1.03756111	0.669274
2237.23111	1.53267755	1.03752128	0.669608
1154.5689	0.11255716	0.96200059	0.669869
1711.93999	2.55716031	1.03748905	0.669878
4271.13979	2.52061845	0.96203277	0.67013
1657.74883	-0.27048994	1.0374513	0.670194
1744.82927	0.9576476	1.03742738	0.670394
2910.55881	0.55168369	1.03742501	0.670414
3458.65537	-2.79607939	1.03740544	0.670578
1915.94084	0.66713813	1.03736729	0.670897
2016.11808	0.33125269	1.03736424	0.670923
2805.25943	0.75154062	0.96216444	0.671199
3073.21909	-0.42365085	1.03732283	0.67127
1373.68293	1.56564372	0.96218258	0.671347
1957.00273	-0.37565322	0.96219142	0.671419
2765.36167	-0.7777814	1.03730328	0.671434

Information Classification: General

3629.76601	1.89505793	1.03729447	0.671507
2345.16556	1.12708294	0.9622034	0.671516
1584.79081	2.19419289	1.03727565	0.671665
1647.74516	2.03883848	0.96222677	0.671706
1195.62151	0.87173046	1.03726525	0.671752
2290.10849	-0.43870192	1.03723348	0.672019
1973.02713	2.81671834	1.03722233	0.672112
2332.18174	-1.91086631	1.03721907	0.67214
1290.68048	-0.39026561	1.03721359	0.672186
1433.55547	0.30150255	0.96228701	0.672195
3549.8312	1.23813317	0.9623009	0.672308
2535.11368	-0.27520924	1.03719352	0.672354
1230.56243	-1.00748368	0.96231318	0.672408
2736.29605	-1.27312242	0.96232472	0.672501
3201.42771	1.395375	0.96236437	0.672824
3072.34018	-2.54731618	0.96239247	0.673052
2029.97824	2.95387479	1.03705115	0.673548
2854.22147	-1.01835152	1.03704546	0.673596
1501.81047	-0.84291366	0.96247803	0.673748
1968.9561	0.60792937	0.96247818	0.673749
2213.06742	-2.54458365	1.03700283	0.673954
1766.93589	1.48726569	0.96250955	0.674004
1961.95244	2.42660157	0.96251504	0.674048
996.463121	-0.10433982	0.96252217	0.674106
2698.28336	-0.70005707	1.03698428	0.67411
1474.72869	0.14917596	0.96252421	0.674123
4086.85976	-3.80276888	0.96253555	0.674215
1339.59038	0.31676101	1.03696793	0.674247
2919.52658	4.24251842	1.03696592	0.674264
1819.93596	1.49367052	0.96255011	0.674334
2319.08603	1.7827659	1.0369529	0.674373
2033.9793	0.44117736	0.96256787	0.674478
1100.49346	0.57292651	1.03693527	0.674521
1473.68694	0.91739104	1.03688531	0.674941
2066.97763	5.96956145	1.03687848	0.674998
2125.90996	0.28289445	0.96264087	0.675072
3062.46183	3.52750341	1.03686553	0.675107
1373.56975	-0.38989852	0.96264864	0.675135
2448.1807	0.69149914	1.036861	0.675145
2139.02799	2.82228841	1.03682292	0.675465
3718.68601	0.15407954	0.96270175	0.675567
2975.38456	-6.79127787	0.96275024	0.675962
2696.29813	0.55429904	0.96278258	0.676225
1505.65788	0.15621929	0.96280541	0.676411
2397.0418	0.41607352	1.03670673	0.676442
1355.69327	0.7252061	0.96281136	0.676459
2195.00322	-0.48604627	1.03667337	0.676723
1877.97295	0.23412977	1.03666263	0.676813
3207.327	2.02199039	1.03665848	0.676848
2808.34853	1.45037131	1.03665754	0.676856
3946.77646	3.27459007	0.96293288	0.677449
1721.73882	0.90828527	0.96293709	0.677483
2783.29018	-0.15501842	0.9629799	0.677832
2557.26706	0.08902509	0.96298941	0.677909
2066.87944	0.43874354	0.9630378	0.678303

Information Classification: General

1625.74895	0.86093609	0.96308589	0.678695
2515.22385	2.80363528	1.03641834	0.67887
2436.14556	-2.38035397	0.96311021	0.678893
3643.83872	-0.73001739	1.03640422	0.678988
1549.74956	0.19617202	1.03639445	0.679071
3062.29612	1.41181491	1.03637038	0.679274
2590.25168	-4.80115447	0.96319449	0.67958
2542.13376	-0.28878115	1.03624079	0.680366
2425.13541	2.54040615	0.96330017	0.680442
2413.89311	0.82427221	0.96330818	0.680507
2961.35813	1.92550641	0.96331042	0.680525
2794.14482	-0.47682232	0.96331971	0.680601
1102.50774	-0.65965212	1.03619653	0.680739
2412.10918	-1.69140017	1.03618361	0.680848
3125.49826	3.99290985	0.96339667	0.681229
2926.39707	-4.61572915	1.03610948	0.681474
1366.69571	-0.97712805	0.96342801	0.681484
1512.67375	-4.945975	1.03609836	0.681568
1676.88498	-0.79636779	0.96344906	0.681656
3620.7081	1.59932804	1.0360834	0.681694
4023.9979	0.01233149	1.03605939	0.681896
2219.0784	-0.95895982	0.96347996	0.681908
2298.11479	-6.25930765	0.96351128	0.682164
2077.02793	1.60273182	1.03598912	0.68249
1512.83159	-2.33848402	1.03598544	0.682521
1567.80986	0.94000676	1.03598136	0.682555
2164.04521	-0.0992057	1.03597163	0.682638
2044.11087	1.26984115	0.963577	0.6827
3010.43735	-2.15824226	0.96357781	0.682707
2618.24979	4.549494	0.96358413	0.682758
2853.4833	-2.33897527	0.96363139	0.683144
3535.75112	-1.92659523	1.03590991	0.683159
3996.73056	3.35189908	1.03588866	0.683338
2513.17765	-0.56854038	0.96366895	0.683451
1761.82646	-1.41993873	1.03584379	0.683718
1780.83647	-0.51993544	1.03583525	0.68379
3412.79258	-0.52234166	1.03580527	0.684043
1856.9683	-2.09612028	1.03580023	0.684086
2312.05083	-4.60173761	0.9637672	0.684253
2141.98661	1.0868966	0.96379347	0.684467
3951.7822	-1.5376911	0.96379901	0.684513
2488.29677	0.21795456	0.96380229	0.68454
2350.06157	-1.88091402	0.9638071	0.684579
2868.30693	0.3859644	0.96381414	0.684636
1410.8416	-2.12707569	0.96382181	0.684699
2125.05962	3.13855253	0.96385682	0.684985
3887.69162	-3.76722066	1.03567194	0.685171
3015.49721	0.09775792	1.03565079	0.68535
2882.32778	-9.66781266	1.03565041	0.685353
1830.83428	-0.49659635	0.9639037	0.685368
2700.20249	0.96898867	0.96393434	0.685618
2952.40189	-2.02840912	0.96394054	0.685669
2019.82854	-2.78067678	0.96395855	0.685816
3565.68528	3.6384455	1.0355895	0.685868
1618.81125	2.55564943	1.03557689	0.685975

Information Classification: General

1009.47196	-1.74083046	0.9639827	0.686014
1798.86003	0.87925146	0.96398363	0.686021
2900.2919	-1.84603159	1.03556419	0.686082
3372.65732	2.43411922	1.03554869	0.686214
1826.77593	0.04007333	1.03553635	0.686318
2793.42771	4.99894526	0.96408639	0.686861
2589.36161	1.82018531	0.96409992	0.686972
2267.05669	-1.50021668	1.03545461	0.68701
3199.504	2.58575626	0.96412667	0.687191
2538.14151	-0.17400742	0.96412994	0.687217
1576.66435	0.29679074	0.96413594	0.687267
1898.83342	-3.6041486	0.96416448	0.6875
2033.06145	0.7528872	1.03539077	0.687551
1451.70146	0.15727364	0.96417792	0.68761
1799.91606	3.09732429	1.03538253	0.687621
1476.59697	-0.17870355	0.96420035	0.687793
1522.72417	9.72165071	0.96421471	0.687911
2359.12571	-0.76925148	1.0353478	0.687915
1747.90068	1.9206265	1.03532585	0.688101
1496.6375	-0.79279677	0.96425066	0.688205
2098.91411	3.03671252	0.96428213	0.688462
1005.45213	-0.17932122	1.0352774	0.688512
1116.45195	0.53436749	0.96434082	0.688943
1293.63042	2.51261292	0.96438549	0.689308
1702.76067	-2.76467747	0.96441504	0.68955
1683.90239	-1.14017966	1.03511126	0.689921
2099.04302	2.26534126	1.03506897	0.69028
1469.71208	0.18944633	1.03505581	0.690392
1431.66081	2.45442236	0.96452901	0.690483
3863.64658	1.69276935	0.96457814	0.690886
3023.44626	1.81345366	1.0349941	0.690916
2116.13408	2.24395133	1.03498753	0.690972
2529.15067	0.53520654	1.03495099	0.691282
2199.93601	-3.71284734	1.03494505	0.691333
1595.7238	-4.21490137	1.03493499	0.691418
2196.93071	3.17368282	0.96464511	0.691434
2420.96573	3.75639127	1.03491407	0.691596
4012.9125	1.29905446	1.03486899	0.691979
2450.16062	-1.06958358	0.96475112	0.692303
3144.45591	6.65089128	0.96477622	0.692508
2379.17337	1.7269427	1.03479422	0.692614
2388.97904	0.34314181	0.96480021	0.692705
3083.39023	-0.63708892	1.03477871	0.692746
1763.94219	0.77551897	1.0347443	0.693039
2238.0463	0.05071422	1.03470974	0.693333
2031.9697	0.23591872	0.96490391	0.693555
2012.09538	1.70777829	1.03466256	0.693734
3044.31791	1.66078125	0.96493119	0.693779
1591.78777	1.21563022	0.96495063	0.693938
3193.54465	-4.28503432	0.96496898	0.694089
2771.3375	1.67833447	1.03461218	0.694163
1668.79436	-1.71931179	0.96499505	0.694303
1521.6928	4.74558479	1.03457284	0.694498
3685.80556	6.94811424	0.96502213	0.694525
3908.90005	1.07084345	0.96504572	0.694718

1575.71623	-0.67980375	0.96504964	0.69475
1045.55461	1.23831737	1.0345126	0.69501
2913.12064	0.03890024	0.96510396	0.695196
1751.955	0.15146133	0.96511406	0.695279
1057.53191	-0.26509809	1.0344392	0.695636
1414.66777	-0.19162936	1.03443555	0.695667
2991.23581	-2.382072	1.03442543	0.695753
2519.22782	-1.38055975	1.03440521	0.695925
2922.39383	-6.97658177	1.03439828	0.695984
1917.79558	0.12750332	0.96520368	0.696014
1771.79302	-3.38750136	0.96524	0.696312
1852.78716	0.00090337	1.03435821	0.696326
1633.72856	3.53664855	1.03434952	0.6964
2715.23929	-0.24651056	1.03425635	0.697194
2241.07798	0.10430109	0.96535293	0.69724
2151.96196	9.22105576	1.03424746	0.69727
2179.12113	0.75333231	0.96538093	0.69747
1563.75139	-2.06422059	1.03422002	0.697504
2208.02519	2.17786606	0.9653965	0.697597
2325.03642	-0.0175258	0.96540446	0.697663
2873.47519	-1.24220883	1.03418865	0.697771
3383.56748	2.16537724	0.9654298	0.697871
3041.51108	1.26687542	0.96543459	0.69791
1839.90056	-2.12783561	0.96543518	0.697915
2059.74956	0.51657615	0.96544442	0.697991
2626.17558	-1.70727041	1.03415103	0.698092
1542.78166	1.19168495	0.96546139	0.69813
2033.05533	0.18528955	0.96546381	0.69815
1306.50701	0.04615862	1.03414389	0.698153
3324.50718	-1.37073982	1.0341418	0.698171
2006.98247	1.04145093	1.03410177	0.698512
2513.04709	-1.83444321	0.96551589	0.698578
1486.65727	1.97009849	1.03408046	0.698694
1944.96575	0.88666905	1.03408011	0.698697
1458.75757	0.68599071	1.03402456	0.699171
1954.92459	-2.4505297	1.03400823	0.699311
1671.79827	-0.40730262	0.96560525	0.699312
1843.81279	-0.45100057	0.96569575	0.700056
1137.57915	0.47808651	1.0339138	0.700117
1860.92351	-1.80759025	1.03390935	0.700155
2501.09209	0.02411522	0.96571849	0.700243
2333.91948	-2.27604574	0.96573167	0.700352
1944.88286	-1.36522425	0.96575291	0.700526
1066.54216	0.64372191	0.96576879	0.700657
1478.78545	-0.28422691	1.03377699	0.701286
1639.77739	-2.1856328	0.96584986	0.701323
2872.5419	-0.8229751	1.03377056	0.701341
1634.72893	-0.19933905	1.033762	0.701415
1628.75945	1.658141	1.03374894	0.701526
3188.46823	3.12047516	0.96590589	0.701784
4036.86582	1.28603327	1.03367183	0.702186
2047.92534	-0.51258338	0.96595622	0.702198
1595.79411	-0.64564622	1.03365669	0.702315
2064.81084	1.02349055	0.96603471	0.702844
3843.84409	1.17201115	0.96603706	0.702863

Information Classification: General

2458.07598	1.81688165	1.03356881	0.703067
1511.81572	-2.50022081	0.96607505	0.703176
1567.74748	3.4373024	0.96608177	0.703231
1183.52983	-0.09509266	1.0335399	0.703315
3563.51118	0.00796774	1.03350807	0.703587
3198.45884	-0.17598417	1.03350138	0.703644
2291.00663	3.07622385	0.96618556	0.704086
1966.82378	0.51797599	1.03342293	0.704316
1661.77861	-0.3172794	0.96624684	0.70459
1492.71575	1.52577002	1.03338566	0.704635
2801.33232	1.74727194	0.96627104	0.70479
3282.54416	2.05641416	0.96629231	0.704965
1346.64946	1.52702044	0.96629483	0.704986
2637.25078	3.197163	1.0333436	0.704996
3044.54111	5.13460385	1.03330366	0.705338
1514.69621	-0.43619906	1.033276	0.705575
2095.98996	-0.01667395	1.03326985	0.705628
2917.26743	-2.74953473	0.96637331	0.705632
1819.87131	1.46038228	1.03323653	0.705913
2143.85967	-6.21382119	0.96641605	0.705984
2595.18692	1.46213005	0.96643061	0.706104
2030.95806	1.34670987	0.96643407	0.706133
3335.52309	0.75571205	1.03314894	0.706665
1751.84624	0.3657313	0.96650627	0.706728
1513.6428	-0.87201366	1.03312763	0.706847
3009.31338	0.13521295	0.96652806	0.706907
1994.98642	0.14787695	1.03310399	0.70705
1784.80351	1.20690911	0.96655065	0.707094
1918.78813	1.17690755	0.96657979	0.707334
1779.8062	1.17520801	0.9665803	0.707338
1741.86016	-0.60755703	0.9666477	0.707894
4835.26284	-2.40529039	1.03299681	0.70797
2812.30698	0.82162194	1.0329697	0.708203
1601.66911	-1.41747032	0.96674896	0.708729
1773.83562	5.47998231	1.03287688	0.709
1152.55486	1.51998758	0.96681848	0.709302
2640.34902	-0.16345211	1.0328209	0.709481
1342.64983	1.00739013	1.03281497	0.709532
4365.09878	0.40257709	0.96686589	0.709693
1589.84195	0.99682717	1.03279132	0.709735
2315.01396	2.68348894	1.03278262	0.70981
2553.20835	-0.79827382	0.96689187	0.709908
2585.18772	2.69263147	0.9668954	0.709937
3763.83755	0.760612	1.03272192	0.710331
2711.05962	-0.14067885	1.03271512	0.71039
1201.56067	-0.47747894	1.03270915	0.710441
1550.83745	1.43770226	0.96698437	0.710671
2500.27553	-2.16692068	1.03265972	0.710866
2288.10349	3.63639242	1.03264623	0.710982
1794.88675	2.71582724	1.03264595	0.710985
1567.71477	1.62273041	1.03263091	0.711114
1212.57683	2.13967911	0.96704148	0.711143
2201.02695	0.0919372	0.96708178	0.711475
971.457262	0.54727494	0.96708366	0.711491
1434.68698	0.7315435	0.96712201	0.711808

Information Classification: General

2695.32273	2.97505132	1.03254697	0.711836
1270.67558	-0.25466128	0.96717398	0.712237
2922.44785	3.46312211	0.96718969	0.712367
3477.55149	2.93352639	0.96721925	0.712611
2621.13956	-1.14369357	0.96722067	0.712622
4724.2438	-1.70210945	0.96722213	0.712634
2940.39695	5.15592749	0.96722974	0.712697
1756.73801	-1.61952062	0.9672316	0.712713
2608.14402	1.76784177	1.03242007	0.712928
1901.89377	-0.62745602	0.96727122	0.71304
3125.53342	-4.82954912	0.96728029	0.713115
2306.18991	0.67046665	0.96732012	0.713444
2405.00688	1.6828377	0.96732948	0.713521
2413.07402	1.82087581	1.03234923	0.713539
2144.94365	6.59495851	0.96734389	0.71364
3864.6542	0.60450703	0.96736113	0.713783
1965.93035	0.36784127	0.96737003	0.713856
2182.97257	-1.25423838	0.96738518	0.713982
1814.84159	0.72270039	0.96739989	0.714103
2161.90958	-1.38685227	0.96747697	0.71474
2853.10874	0.21606804	1.03218725	0.714934
2843.51528	-0.22113649	0.96752335	0.715124
2696.24302	-2.91672174	0.96753544	0.715224
3534.86094	4.53956508	1.0321464	0.715287
1760.79411	-1.82890118	1.03214323	0.715314
2066.00278	0.60959955	0.9675653	0.715471
1670.94377	3.8929405	0.96757175	0.715524
1876.84441	-6.13122198	1.03210898	0.715609
3670.80869	0.88553716	0.96758238	0.715612
1641.8936	-0.08466672	0.96758997	0.715675
2887.1301	-3.60227261	1.03208357	0.715828
2408.10001	1.2380874	0.96761226	0.715859
2314.14054	1.84594754	0.96767797	0.716402
1842.6646	2.14313555	0.96768901	0.716494
2432.18314	1.17767492	1.03199988	0.716551
2250.00566	1.47623323	0.96769753	0.716564
2312.19797	-1.0064605	1.0319651	0.716851
1443.70806	-0.18901934	0.96776297	0.717106
2254.06548	0.29191089	0.9677665	0.717135
3343.67173	0.44195334	0.96777127	0.717174
1277.64775	-1.24887562	1.03190993	0.717327
3500.86924	0.1821012	1.03184701	0.71787
2262.99028	0.06437224	1.0318453	0.717885
2516.07291	5.43538657	0.96786037	0.717912
4245.01967	0.27225463	1.03179736	0.718299
4120.85007	0.32334917	1.03178095	0.718441
2071.03227	0.53879472	0.96792756	0.718468
2917.42051	-0.31533207	0.96793044	0.718491
2241.07303	0.76030508	0.96797401	0.718852
2072.97416	0.06123863	1.03171816	0.718983
1567.63066	1.02991933	1.03170228	0.719121
3110.43198	3.02036926	1.03169672	0.719169
4598.76577	-2.16373789	0.96803328	0.719343
3233.40342	-4.34211207	1.03165956	0.71949
1725.95378	1.46101845	1.03165388	0.719539

2400.27188	3.46354025	1.03163482	0.719704
1712.84562	-0.17111464	1.03161621	0.719865
3422.60263	-1.18449333	1.03160486	0.719963
2497.11234	-0.82393576	1.03159933	0.72001
2328.17934	2.86906377	1.03156414	0.720315
3915.80344	1.08422553	1.03153784	0.720542
2721.34769	1.84324199	1.03152631	0.720642
2149.01859	-0.49625451	0.96821773	0.720871
2109.0983	1.37947762	0.96823344	0.721001
1532.69475	3.29514222	0.96823659	0.721027
2482.06162	-0.82257225	0.96824008	0.721056
2712.23052	-10.7619729	1.03143439	0.721437
2773.14548	1.33084604	0.96829241	0.721489
2409.08603	-2.0279942	1.03141509	0.721604
1753.85135	0.02520836	0.96830968	0.721633
2040.00217	-0.53672354	1.03139759	0.721756
2804.27231	-0.78926558	1.03139698	0.721761
1636.84109	-0.00802823	1.03139657	0.721765
3454.62343	-5.09968987	1.03134955	0.722172
2452.3165	-0.35063276	0.96838118	0.722225
2426.08769	-0.21483843	1.03133858	0.722267
2284.14165	2.77603679	1.03132906	0.722349
2226.12554	-0.11338983	1.03132167	0.722413
3250.29599	-0.389403	0.96842999	0.72263
2881.29068	5.32652484	1.03128648	0.722718
2014.01603	-0.53521755	1.03128512	0.72273
3532.6146	-0.18347866	0.96846268	0.722901
1297.6021	0.18733015	0.96852966	0.723456
3570.70693	2.2613409	1.031161	0.723805
2047.94682	0.64925636	1.03115357	0.723869
2525.13022	2.24892981	1.03114282	0.723963
2687.15384	0.73440012	1.03107506	0.72455
4406.15156	6.93309163	0.96866189	0.724553
2143.94072	-0.33763673	1.03097486	0.725419
1674.74675	0.29461189	0.96877226	0.725469
2817.29441	4.70572437	1.03095846	0.725561
1870.88518	0.44905732	1.03094796	0.725652
2345.16007	-2.65225732	1.03093045	0.725804
2254.21341	2.08265933	1.03092536	0.725848
1170.63725	-0.11418567	0.96882898	0.725939
2243.11008	0.43031284	0.9688294	0.725943
1222.45732	0.40712209	1.03090224	0.726049
1780.74516	-0.94820628	0.96884717	0.72609
2582.13773	-0.34035177	0.96887147	0.726292
2978.53873	1.42020695	0.9689062	0.72658
2715.06607	1.81722701	0.96891668	0.726667
2560.02903	1.96777127	0.96892232	0.726714
1314.65862	0.79232333	1.03080542	0.726889
3519.63938	0.24210266	1.03080339	0.726907
1995.88401	0.43640828	0.96894954	0.72694
3248.36533	-7.62159886	0.96897327	0.727137
2214.16811	1.32881791	0.9690188	0.727515
2209.09879	-3.12628158	0.96904773	0.727755
3012.44529	0.64410039	0.9690502	0.727776
2582.22708	2.69404408	0.96908061	0.728028

Information Classification: General

1817.89762	-1.1668093	0.96914014	0.728523
4470.95395	1.35615563	1.03060712	0.728612
2990.31241	0.62160128	0.96915705	0.728663
1405.58879	0.38163077	0.96916758	0.728751
2355.02817	-0.47725146	0.96919713	0.728996
1742.93105	0.27693544	0.96920695	0.729078
2900.31479	7.45682958	0.96923832	0.729338
2269.10161	-2.13661708	0.96924092	0.72936
2992.39067	3.00572343	1.030488	0.729647
3182.43894	-1.59636655	0.96929415	0.729802
2731.2363	4.41482935	0.96929506	0.72981
2685.37973	-0.84324783	1.03046284	0.729866
2075.88022	-0.13206898	1.03042955	0.730156
2294.15274	0.31637531	1.03042944	0.730157
2115.72661	1.04446328	0.96934683	0.73024
2669.25418	1.26486379	1.03039848	0.730426
1779.78777	0.81190878	1.03039649	0.730443
1623.74968	-0.56468814	0.96937361	0.730463
3860.70131	-0.94740526	0.96937517	0.730476
2591.25327	-0.16373713	1.03038928	0.730506
2477.97002	-1.32810248	1.0303765	0.730617
2155.16819	1.26565891	0.96941962	0.730845
3292.68747	1.42492029	0.96942248	0.730869
1055.5623	0.40562089	1.030343	0.730909
2930.58689	-0.50540058	1.03028996	0.73137
3245.51145	1.67814123	0.96949062	0.731435
3096.55259	-0.26574008	0.96949533	0.731475
2155.99761	0.74624039	0.96949888	0.731504
1640.72112	-1.13691796	0.96952245	0.7317
1871.85515	1.97228819	1.03024791	0.731736
3079.26743	0.51014467	0.96953168	0.731777
1375.66385	2.77481429	0.96960578	0.732393
3192.53427	0.33502381	1.03016237	0.732481
3058.36406	-2.61219044	1.03015022	0.732587
2425.023	1.03855407	1.03009066	0.733106
2861.36515	-1.57227153	1.03008803	0.733129
3740.42275	2.80313185	1.03008418	0.733162
2200.07329	-2.04016556	1.03007012	0.733285
2327.16501	1.93406126	1.03006433	0.733335
2334.19535	0.35059291	1.03005973	0.733375
1599.73247	3.29690986	0.96977751	0.733822
1444.64372	-0.04218579	0.96977903	0.733834
2405.06255	-0.46181803	1.02998432	0.734033
1715.68669	-0.67739258	1.02997571	0.734108
1761.80229	0.9044105	1.02996352	0.734214
2253.82256	1.53564191	0.96984763	0.734405
2597.03691	1.80495437	0.96988498	0.734716
4039.92202	2.33407383	1.02989126	0.734844
2543.08976	-6.84579375	0.96993789	0.735157
1886.9102	-0.38022543	0.96995471	0.735297
2387.11284	0.77301226	0.9699693	0.735418
2543.30912	-1.10687151	0.97001777	0.735822
2754.27129	0.0905986	0.97001963	0.735837
2099.73052	0.49103861	1.02977549	0.735854
3957.52073	-1.45147179	0.97005318	0.736117

Information Classification: General

2657.22525	1.15593471	0.970111	0.736598
1852.79375	4.31212089	0.97013659	0.736811
2809.26487	3.75663948	1.02964662	0.736979
2153.08554	-1.71237681	1.02961402	0.737264
2514.23581	2.5644615	1.02960125	0.737375
2204.95708	0.61568035	0.97021543	0.737468
3164.53598	-0.81479264	0.97024473	0.737712
2392.18901	0.08742725	1.02953203	0.73798
2460.24021	4.30006475	1.02952545	0.738037
1468.71318	1.70017155	1.0295107	0.738166
1538.78318	1.47580388	1.02949699	0.738286
2711.26357	-1.71093265	0.97031608	0.738307
1536.80051	-0.73208815	0.97033449	0.73846
1817.89861	-2.82806253	0.97034978	0.738588
1514.74628	-0.07975578	0.97036371	0.738704
2010.95195	0.38469125	1.02943879	0.738794
2095.02202	1.45342386	0.97040499	0.739048
2418.13895	-3.57801277	1.0293917	0.739206
1402.7219	2.3679999	1.02935955	0.739487
2156.88848	-0.30134468	1.02931687	0.73986
3083.48012	1.59201448	0.97050271	0.739862
2816.42373	5.57283355	1.02928565	0.740133
1831.77336	-2.1177683	0.97059879	0.740664
2796.2208	-6.37359808	1.02921799	0.740725
1636.7697	-0.79264721	1.02921221	0.740776
2885.28879	-0.28795157	1.02920729	0.740819
1724.82749	1.83755515	1.02916845	0.741159
2226.92521	-1.94180263	0.97066284	0.741198
1956.86882	-1.53627425	1.02916359	0.741201
2019.91374	-0.19872739	0.97068459	0.741379
2364.17246	0.28050777	1.02914126	0.741397
1798.05118	3.34268198	0.9707089	0.741582
2871.14664	0.36755269	1.02910719	0.741695
2576.10074	2.32400079	1.02908274	0.741909
2670.15703	0.91781891	1.02908084	0.741926
2297.06687	-0.73339248	0.97075182	0.74194
1244.63774	-0.02843297	0.97076629	0.742061
2374.08725	0.20137871	1.02906401	0.742073
1886.95122	0.45108413	1.02906279	0.742084
2059.04972	0.11241571	1.02903057	0.742366
1695.87969	0.30365988	0.97080988	0.742425
1848.8416	-0.89513719	1.02901548	0.742498
1448.71671	2.04364112	0.97082353	0.742538
4557.4492	0.18953952	0.97082468	0.742548
1829.9368	-0.14731084	0.97082827	0.742578
2111.92729	0.60058531	1.02899725	0.742658
2199.11675	0.84966726	1.02899357	0.74269
1725.91545	0.33117044	1.02898595	0.742757
1751.83738	1.51278936	0.97085688	0.742817
2429.0579	-2.73678925	0.97086289	0.742867
2941.10154	-0.87541446	1.02890685	0.74345
1939.89507	-3.50440089	1.02890019	0.743508
2036.90264	-0.83941696	1.02887862	0.743697
2095.81841	0.23030323	0.97096927	0.743755
3384.50864	3.33336699	1.02887093	0.743765

Information Classification: General

3274.67136	0.26431231	0.970986	0.743894
1753.75701	-3.0561209	0.97099451	0.743965
1486.77727	1.0633593	1.0288382	0.744051
1755.69829	0.40980062	0.97103026	0.744264
1287.5457	-1.33075228	1.02875435	0.744787
2506.33811	1.2366978	1.02875241	0.744804
1411.65508	1.60208798	0.97110778	0.744911
2491.10465	-0.07903716	1.02873058	0.744995
1464.65947	-0.56591525	1.02872797	0.745018
1906.98247	-2.81535165	0.97113234	0.745116
1787.86882	1.19343924	1.02870958	0.745179
1517.72649	0.5592104	1.02868941	0.745356
2717.27524	3.3796252	1.02863811	0.745806
1723.85307	1.40768522	1.02861569	0.746003
1310.69648	0.37957128	0.97126035	0.746186
2891.2188	-0.23197872	0.9712645	0.74622
1880.9379	0.65821593	1.02856411	0.746456
2400.03989	-4.36084028	1.02851627	0.746876
2078.92339	2.01024363	0.97134901	0.746926
1660.79697	-0.30594498	1.02850957	0.746934
1588.82608	11.5233865	1.02847911	0.747202
1929.93271	-4.26228591	1.02846618	0.747315
1384.64653	1.01590819	0.97139782	0.747334
1502.6646	0.04863361	0.97142189	0.747535
1916.97637	2.18972868	1.02838987	0.747986
2603.08335	-0.20059265	1.02838386	0.748038
2471.21701	0.51627712	1.02837722	0.748097
2912.47593	3.87881854	1.02836242	0.748227
2036.99228	-0.04055612	1.02834645	0.748367
2063.9617	-2.31798186	1.02833711	0.748449
3320.44114	8.14106484	1.02828757	0.748884
1908.89737	1.11148958	0.9715946	0.748979
3204.44529	2.28754637	1.02827424	0.749002
2515.27365	1.72126711	1.02826145	0.749114
1652.8134	0.48208161	0.97161881	0.749181
1260.64775	-0.1631091	0.97161957	0.749188
2240.04599	2.81705153	1.02821184	0.74955
2200.07231	-0.65683125	1.02811053	0.750441
1942.95647	3.08457615	1.02810115	0.750524
2537.19284	-2.06018596	1.02809653	0.750564
1839.75675	2.83880982	1.0280826	0.750687
3537.7521	4.21301461	1.02806397	0.750851
2769.26859	-0.41760491	1.02803821	0.751077
2606.27616	-2.1045739	1.02799473	0.75146
1981.86284	-0.12499982	1.02796831	0.751692
988.530809	-1.05103704	0.97191932	0.751695
3088.51023	0.73780667	1.02794233	0.751921
4091.9216	0.03804486	0.97194904	0.751944
1811.8322	1.03354994	1.02793227	0.75201
2868.33323	4.97632918	0.97196049	0.75204
1528.71099	-1.02059923	0.97197023	0.752121
1647.78191	1.45204308	0.97202664	0.752593
2595.23709	4.2101738	0.97203742	0.752683
1773.91806	2.33135768	1.02784667	0.752763
1503.80657	0.19806632	1.0278257	0.752948

Information Classification: General

2255.07646	7.21186826	1.02782475	0.752956
2371.16874	0.97843067	1.02782077	0.752991
1606.65849	-1.63720892	0.97209326	0.753151
2545.16892	0.00122966	1.02777135	0.753427
1154.5855	0.4664163	0.97212843	0.753445
2201.19596	1.14878637	0.97215421	0.753661
2871.57631	0.25085569	1.02773967	0.753706
2683.18924	-5.69701273	1.02772255	0.753857
1768.74956	-0.70444258	0.97217769	0.753858
2183.01487	-4.09487471	1.02771244	0.753946
1222.64334	0.61293363	0.97219885	0.754035
3202.57688	1.80982824	0.9722426	0.754401
1348.59868	-2.1020996	1.02764423	0.754547
2479.0336	-0.83976745	0.9722673	0.754608
2363.1852	0.20441479	0.97232181	0.755065
2382.13218	0.25312014	1.02754522	0.75542
2776.33573	0.68371116	1.02751439	0.755692
2642.17959	1.23301067	0.97240218	0.755738
1975.91819	-1.84182332	1.02747584	0.756032
2892.3259	-0.98690415	0.97249258	0.756495
2105.90874	-4.18218265	0.97252157	0.756738
1485.66911	0.43729896	1.02738265	0.756854
2319.2424	1.20878172	1.02737602	0.756913
1586.73149	0.82220039	1.02737437	0.756927
4694.73598	-0.20910228	1.02737437	0.756927
2045.01821	-0.60298607	1.02736781	0.756985
2583.07614	3.33543872	0.97257359	0.757174
2532.09555	-3.69896314	1.02728083	0.757753
2426.98331	0.74381209	1.02727657	0.757791
2011.93205	-1.08893603	1.02727052	0.757844
2643.22629	6.81068307	1.02726783	0.757868
2753.29538	1.57446726	0.97271926	0.758395
1308.63237	-0.24184218	1.02719089	0.758547
1522.73894	-0.24239197	0.97275037	0.758656
3564.61338	1.53540845	0.97276414	0.758771
2261.98532	-3.44591993	1.0271413	0.758985
1507.69109	0.00155059	0.97281199	0.759173
1633.91005	-2.54812948	1.02710141	0.759338
2511.19927	3.88183004	1.02704612	0.759826
1952.90293	0.06601391	0.97290539	0.759956
2006.9428	-1.29982669	1.02699088	0.760315
1704.85434	-1.00012393	0.97295332	0.760358
3144.41452	-0.27521866	0.9730139	0.760866
1623.88225	5.04004966	0.97301772	0.760898
1915.81694	-2.94034878	1.02690289	0.761093
3038.48307	0.38541564	0.97304924	0.761163
1992.99089	1.81487298	0.9730647	0.761292
2717.27652	5.67042405	1.02676356	0.762325
2750.31241	7.64414324	0.97319715	0.762404
2455.17046	-1.12438018	1.0266802	0.763063
1358.6917	-0.19158918	1.0266654	0.763194
2739.20047	1.02522816	1.02664267	0.763396
3550.4758	0.33091207	1.02664053	0.763414
1608.61174	3.49719857	1.02663769	0.76344
1614.85002	2.21189871	1.02658899	0.763871

Information Classification: General

3092.37595	0.43215305	1.02658402	0.763915
1769.90952	0.98915544	0.97339665	0.764079
1807.91692	-1.37759874	0.97341587	0.76424
2778.28873	2.91803415	1.02653554	0.764344
2016.86394	0.0599447	0.97343987	0.764442
2366.23239	-1.53548224	0.97347018	0.764696
3090.53135	2.08601266	0.97348568	0.764826
1844.90696	1.59912621	1.02647272	0.764901
2214.12065	1.34715671	0.97350163	0.76496
2006.98171	0.6680973	0.97354731	0.765344
1229.63872	0.48890275	0.9735475	0.765346
1708.75347	-2.75623874	1.02642128	0.765357
1501.7631	-1.57323364	0.97358181	0.765634
1108.4793	0.02737591	0.97359111	0.765712
3022.26798	-4.89328273	1.02632708	0.766192
1537.7728	0.09231899	0.97364854	0.766194
3003.28397	2.24134624	0.97367706	0.766434
1953.98918	-0.16784078	0.97368368	0.76649
1836.83623	-1.20863286	1.02628131	0.766598
1535.75737	-0.9073585	0.97370103	0.766635
3189.63388	2.92539844	1.02626795	0.766716
1879.87346	-0.4040779	1.02626503	0.766742
1509.70696	0.15332673	0.97379139	0.767395
925.462694	0.21479323	1.02618534	0.767449
3444.63117	-1.26007386	0.97380498	0.767509
1713.81914	0.81927876	0.97380554	0.767514
2187.05693	3.38540084	1.02616979	0.767587
1295.62285	0.2045604	0.97383108	0.767728
1145.56926	-0.09325849	1.02613629	0.767885
2675.32851	-2.99155154	1.02613281	0.767915
2550.98801	0.33239143	1.02612391	0.767994
1916.94438	1.11804822	0.97387151	0.768068
2347.94267	0.95030083	0.97388469	0.768179
3423.64524	2.52873428	1.02609816	0.768223
1297.66631	1.51738884	1.02608361	0.768352
1508.79665	0.21160261	1.02607282	0.768448
3134.46457	7.33185079	1.0260342	0.768791
1854.84661	-3.89255049	1.02603031	0.768825
2247.1428	-0.87351689	1.02602461	0.768876
2673.22995	2.07063591	0.97397213	0.768914
2060.02365	-5.38660911	1.02600276	0.76907
2820.21604	-2.67629486	1.02598915	0.769191
1318.53533	-0.11708517	1.02595024	0.769536
2816.29795	0.48235308	1.02591809	0.769822
1348.59099	0.52994686	1.02590499	0.769938
2713.15068	0.55657225	1.02589944	0.769988
1767.92447	-0.5479143	0.97411353	0.770103
2069.91411	-0.9542431	1.02588461	0.770119
1997.95017	0.54212047	1.02587337	0.770219
1756.83489	1.48401307	0.97413124	0.770252
2528.37101	-0.73267252	0.97414656	0.770381
2785.30259	7.2585011	1.02585147	0.770414
2583.20578	-1.12752765	0.97419441	0.770784
3858.77451	-2.21055933	1.02578868	0.770972
2342.08481	2.00235463	0.97426966	0.771417

Information Classification: General

1372.69841	1.48871489	0.97427644	0.771474
1888.84824	1.92202776	1.02572903	0.771502
2881.24656	-1.52369868	1.02572377	0.771549
4939.20327	3.49276499	1.02572003	0.771582
2828.26242	1.68159398	1.02571403	0.771636
1733.78288	-0.27903562	1.02570803	0.771689
1544.83269	-0.08356872	1.02568841	0.771863
3854.87075	5.25826839	0.97434411	0.772043
2148.90923	0.03349897	0.97435346	0.772122
1940.77519	-0.26392386	0.97435423	0.772128
2444.18882	-3.76378834	0.97436289	0.772201
2767.27091	0.55031684	1.02564559	0.772244
2696.22782	0.94764079	0.97439081	0.772436
2065.90093	1.16714455	0.97444471	0.77289
1214.51421	-2.40223209	0.97447414	0.773137
1676.66411	-1.75061972	0.97448291	0.773211
1331.64592	0.94869227	0.97450312	0.773381
2859.28653	1.59371078	0.97452354	0.773553
2343.13262	0.00454866	1.02549712	0.773565
1213.62627	0.68060646	0.97453907	0.773684
2613.30136	-2.77181245	0.97457904	0.77402
2378.14048	1.59525769	0.97461215	0.774299
2432.13626	1.7253643	0.97461314	0.774307
2032.01969	1.4134571	1.02540536	0.774382
1498.66863	2.02940147	0.97462358	0.774395
2316.05014	0.78828847	1.02535932	0.774792
1943.89629	-1.43035762	1.02534037	0.774961
1322.70636	-0.63869199	1.02533511	0.775007
1277.56597	-1.42437459	1.02531242	0.775209
2318.01322	0.3026066	0.97473875	0.775365
2327.20145	-0.1053334	0.97475026	0.775462
2836.44614	3.11368838	1.02527818	0.775514
2199.0344	-0.22344111	1.02526298	0.77565
2594.18827	-16.4431882	1.0252261	0.775978
1857.877	3.1086147	1.02518786	0.776319
1312.58318	-0.45849928	0.97486651	0.776441
1372.75266	-2.55154191	0.97488755	0.776619
2596.2753	-1.27089487	1.02512707	0.776861
2302.11528	-1.02267956	1.02507892	0.77729
1810.88494	1.76708857	1.02505205	0.77753
2172.94463	1.42772937	1.025043	0.77761
1384.66838	3.90220486	0.97501965	0.777732
2450.00713	2.20300456	0.97502995	0.777818
1392.74768	-1.00090001	1.02501588	0.777852
1905.66216	-0.36644809	0.97503473	0.777859
1494.70989	1.26992265	1.02498265	0.778149
1504.71391	0.29007923	0.97508001	0.77824
1297.72344	0.19108126	1.02497029	0.778259
1550.72222	0.10979884	0.97509011	0.778325
2120.00737	1.61957809	0.9751028	0.778432
1077.47319	-0.2349664	1.02494424	0.778491
1744.8303	0.79668654	0.97513785	0.778728
2891.37741	2.14715084	1.02491595	0.778744
3254.41953	-0.51883869	0.97515103	0.778839
3968.67148	-1.5237829	1.02490204	0.778868

Information Classification: General

2755.20572	2.08417428	1.02488812	0.778992
2456.00615	2.7409599	0.97518048	0.779087
2254.00469	1.69075541	0.97518363	0.779114
2730.28232	0.33273116	1.02485109	0.779322
1713.81401	-2.72833427	1.02482381	0.779566
1711.89507	2.89260136	0.97524111	0.779598
1334.78081	0.61509537	0.97529294	0.780035
1850.8405	0.13312887	0.97534174	0.780447
1851.73491	0.62945295	1.02471744	0.780515
1741.76189	-0.26805095	0.97535223	0.780535
1383.71526	-0.12845151	0.97535529	0.780561
2717.48549	3.15440092	1.02470626	0.780615
3042.54544	0.85323577	0.9753656	0.780648
2816.38309	-5.61873386	1.02466293	0.781002
2955.41689	-5.06205298	0.9754362	0.781243
1845.86186	0.54809356	1.02462801	0.781314
1533.65129	-0.78021912	0.97544744	0.781338
2149.99028	2.69893022	0.97548016	0.781614
1772.8593	0.22575307	0.97549933	0.781776
1869.85625	-8.02015095	1.02452977	0.782192
2622.18637	4.99056227	0.97555828	0.782273
1526.71184	2.42631965	1.02444747	0.782927
2899.27494	0.62608622	0.97568096	0.783308
1929.81621	-0.10653443	1.02440205	0.783334
3070.49639	-0.55294337	1.02439942	0.783357
2217.14531	2.4878706	1.0243991	0.78336
1736.69402	0.86660361	0.97569098	0.783393
3169.68125	1.72011973	1.02439013	0.78344
1588.80045	-0.26001289	0.97571331	0.783581
3322.45896	2.24177101	1.02437277	0.783595
1925.00871	-0.47984669	1.0243669	0.783648
1419.6751	0.03601308	1.02436301	0.783683
1526.73467	0.9179325	1.02435032	0.783796
2124.98662	-0.44350191	1.02433731	0.783912
1423.68015	-0.7279736	0.9757619	0.783991
1583.71807	-0.17687689	1.02431934	0.784073
1440.74748	1.68372474	1.02428395	0.78439
3205.55581	9.46872885	0.97581644	0.784451
1822.83489	-0.09426749	0.975821	0.78449
2299.13603	0.88234313	0.97584206	0.784668
2385.09184	3.8045385	1.0242057	0.78509
1242.52727	-0.02094245	0.97590776	0.785222
3752.81923	-0.57149752	1.02418962	0.785234
2805.25981	3.6556057	1.02418183	0.785303
2789.2128	-1.62145856	1.02417411	0.785373
2684.30582	5.61857931	0.97594537	0.78554
3260.56215	-5.6403786	1.02414722	0.785613
1889.92107	-3.63887866	1.02414054	0.785673
2489.05533	-0.77029067	0.97596604	0.785714
1255.57939	-0.52724513	1.02413259	0.785744
3782.73301	0.58609575	0.97599529	0.785961
2587.181	-0.56544531	1.02410711	0.785972
1600.76799	0.28276096	0.97600077	0.786007
1149.57854	-0.86074181	0.97600422	0.786037
2383.09062	3.33829966	0.97601244	0.786106

. Information Classification: General

2894.26186	1.53449358	1.024092	0.786108
3704.71286	4.9843113	0.97601807	0.786154
2137.1668	0.29821988	0.97602195	0.786186
2264.13872	-1.58374848	1.02407551	0.786255
4000.79587	-0.34611746	1.02407368	0.786271
1361.63385	0.51293703	1.02404666	0.786513
1811.90642	1.8330798	0.97606572	0.786556
2359.96074	1.91725991	0.97607383	0.786624
1826.85808	-2.65235419	0.97608045	0.78668
1750.91324	2.32099459	0.9761018	0.786861
1961.75371	-0.81334974	0.97611346	0.786959
2634.38921	-0.79621682	1.02396381	0.787255
2210.18039	0.85727802	0.97617191	0.787453
1912.85088	-0.54244852	1.02393901	0.787478
3022.50986	-5.64249193	0.97621767	0.787839
3304.58969	-1.18456555	0.97622712	0.787919
3151.33518	1.41968247	0.97623249	0.787964
1126.52654	0.27656389	1.02386064	0.78818
1810.78508	2.2882221	0.97626321	0.788224
1631.85367	0.09164281	1.02385358	0.788243
1886.87491	0.70157382	1.02384797	0.788293
1443.62871	-0.35230103	0.97627538	0.788327
1862.78142	1.64343977	1.02384296	0.788338
2813.11979	1.11116097	1.0238419	0.788348
1573.71013	0.20480682	0.97627835	0.788352
2180.94902	0.67581044	1.02382824	0.78847
1863.87956	0.13890399	0.97631275	0.788642
2033.90531	5.83227243	0.97631445	0.788657
1143.52873	0.96964961	0.97635066	0.788963
2318.14231	-2.08882708	1.02376754	0.789014
1671.9511	-1.06276826	0.97638223	0.789229
2430.11553	3.70262824	0.97646591	0.789937
2191.0081	-1.37884503	0.97647008	0.789972
1921.86797	10.1787537	1.02362968	0.79025
1595.7968	-1.09335447	1.02362318	0.790309
2801.23198	1.70492574	1.02360039	0.790513
2135.98638	-5.4024578	1.02359391	0.790571
1753.94805	1.06294233	0.97655471	0.790687
1673.96389	3.70772242	1.02355319	0.790937
2288.97807	-1.51786404	0.9766007	0.791076
3595.62302	-1.45991607	0.97661324	0.791182
2212.06035	-2.37107766	0.97661418	0.79119
1098.58537	5.01230702	0.97663257	0.791345
3341.50486	0.71693138	1.02343191	0.792025
1481.7222	-1.19872598	1.02343004	0.792042
1695.86386	-1.84188207	1.02342964	0.792045
4346.83811	9.18152041	1.02340243	0.79229
2059.82109	0.00350546	0.97678034	0.792595
1957.91435	1.86726215	0.97679081	0.792683
1613.70928	0.34567216	1.02335617	0.792705
1908.91948	0.50598913	1.02331915	0.793038
2631.30532	1.3074338	0.97683428	0.793051
2669.21303	0.82949589	1.02331272	0.793095
1181.60698	1.83300584	1.02330121	0.793199
1486.65752	0.11569704	0.97687617	0.793405

Information Classification: General

2440.12662	-1.25167013	1.02325076	0.793652
1662.69988	0.7448129	0.9769257	0.793824
1841.75224	4.14581516	0.97692711	0.793836
2669.23294	-1.99008161	1.02320274	0.794083
1360.64336	3.31449501	1.023167	0.794404
1188.5352	-0.12742681	1.02316253	0.794445
1860.90415	2.80918385	1.02313561	0.794686
3185.39756	-0.90826183	0.97703829	0.794776
1902.87956	1.9349098	0.97705471	0.794915
1123.52593	-0.55966221	0.97706649	0.795015
2883.52365	-2.93479201	1.02309801	0.795024
1281.64139	-1.50878849	0.97708549	0.795176
3995.73374	5.03348495	1.02306704	0.795303
1962.96697	0.26910492	0.9771124	0.795403
1805.9216	2.22140128	1.02305224	0.795436
1860.8366	0.4031253	1.02304031	0.795543
1718.65251	-1.52554453	1.02301488	0.795772
1269.60307	-0.01524689	0.97716784	0.795872
1529.76963	-0.41958115	1.02297112	0.796165
2370.03765	0.94764529	1.02296292	0.796239
1504.77898	1.74774027	0.97723164	0.796412
1772.77922	-1.62056083	1.02291978	0.796627
2591.1575	0.63892506	1.02291514	0.796669
1978.92388	-0.05537163	1.02290488	0.796761
1083.52117	0.70645857	0.97728274	0.796845
1732.80791	0.84465616	0.97728754	0.796885
2337.09648	-1.02655342	0.97730111	0.797
3069.49088	0.33216548	0.97732327	0.797188
1868.84331	1.50575358	1.02285208	0.797236
1670.88774	1.25359185	1.02284612	0.79729
2233.88921	0.67078304	1.02280787	0.797634
3033.34037	-0.50212512	0.97739201	0.79777
2222.93852	0.42010215	0.97740263	0.79786
4293.94497	6.97999048	1.02277264	0.797951
2476.10813	0.40107863	0.97741357	0.797952
3644.71799	4.44818923	0.97744492	0.798218
1422.70557	2.44023858	1.02271685	0.798453
1506.74651	1.40651375	0.97750266	0.798707
2367.12041	3.0077164	1.02266859	0.798888
3228.37754	0.79925403	1.02266861	0.798888
1934.87554	0.15328118	0.97752989	0.798937
2361.11386	-0.84306872	1.02265714	0.798991
1497.79474	2.04574379	1.02265057	0.79905
2466.16806	1.01843728	1.02262755	0.799257
2610.03861	-0.51452454	0.97761658	0.799671
4270.96813	1.63877635	0.97764867	0.799943
1812.90433	-3.42007714	0.9776507	0.79996
1168.67044	0.92926847	1.02252546	0.800177
1411.70781	0.38461969	0.97769441	0.80033
1895.96207	0.05379072	1.02248052	0.800582
1864.83281	-2.87373181	0.9777287	0.800621
1810.89543	0.83110317	1.02245866	0.800779
1653.7968	0.11200193	0.9777501	0.800802
1243.62029	2.33074082	0.9777573	0.800863
1787.91922	0.50075275	1.02243498	0.800992

2843.48208	-2.29926893	1.02240687	0.801246
2378.97141	-0.15159322	1.0223942	0.80136
3423.49839	-1.21808057	0.97786033	0.801736
2317.94365	0.42961112	1.02230541	0.80216
1899.83164	-0.79383021	1.02230384	0.802174
2198.11528	-2.37945948	0.97792622	0.802294
2486.26406	1.10104631	1.02227436	0.80244
2213.11264	5.00910591	0.97797168	0.802679
2260.03067	3.12009074	0.97797185	0.802681
1685.74248	0.39919465	0.97797496	0.802707
950.458605	-0.08792844	0.97797658	0.802721
2393.08599	1.9367456	1.02221363	0.802988
1590.73844	-0.61470027	0.97801557	0.803051
3665.79311	-7.91309626	1.02218301	0.803264
2804.35544	-4.07453087	0.97812234	0.803956
2143.00957	-11.5100825	0.97813087	0.804029
1840.86345	1.92063081	0.97813143	0.804033
2384.08976	-1.04420061	1.02209657	0.804044
2710.27256	0.94375195	1.02209446	0.804063
1483.78032	0.82609011	0.97814051	0.80411
2310.98613	0.31730313	0.97814312	0.804132
1631.8665	1.59166015	1.02206408	0.804337
1986.94315	-0.76444728	0.9782036	0.804645
1138.56267	0.04775237	1.02200995	0.804826
1455.70683	-1.03976422	0.97823926	0.804947
2521.19805	1.58271804	0.97826325	0.805151
998.552904	0.31620164	1.02196674	0.805216
4303.85171	0.71632957	1.02196036	0.805274
2403.15946	2.989265	1.02193339	0.805517
1194.57451	-0.33887432	0.97833728	0.805778
1611.67998	1.34079929	1.02189628	0.805852
2352.23032	0.02614407	1.02187825	0.806015
2328.2875	3.14608565	1.02185518	0.806224
2561.18882	-0.05443151	0.97840627	0.806363
2153.92241	0.47939517	1.02182237	0.80652
1957.93096	1.70920321	0.97843126	0.806575
2512.14458	2.2966627	1.02180837	0.806646
2020.98039	-1.35776753	1.02179313	0.806784
4043.93105	5.18274894	1.02176351	0.807052
1248.56206	0.06649049	1.02174882	0.807184
2256.1904	2.42953994	1.02174223	0.807244
1941.93572	6.62289897	0.97851453	0.807282
2074.99179	5.69742313	1.0217057	0.807574
3008.27378	-0.04270618	0.97855834	0.807653
1723.70683	-0.52537465	0.97857406	0.807787
2463.04026	-0.20867693	0.9785985	0.807994
1155.5374	-1.2757576	0.97860771	0.808072
2060.01761	3.89655763	0.97865411	0.808466
2982.48703	0.78503747	0.97870281	0.808879
2513.1196	-1.27302997	0.97875903	0.809356
1874.91411	0.05962873	1.02148398	0.809579
3710.70737	-1.7858379	0.97882211	0.809891
2451.02689	-0.97905042	1.02144045	0.809973
2902.51274	1.13508933	0.97883827	0.810028
1639.72051	-1.78610737	1.02140658	0.810279

Information Classification: General

2861.16245	-6.10227388	0.97887245	0.810318
2089.99491	0.73153251	1.02139597	0.810375
2992.28354	1.52129738	0.97891096	0.810645
3280.71677	2.72034827	1.02133578	0.81092
1094.54753	-0.08437248	0.97897655	0.811202
1450.69242	-0.41451596	1.02130454	0.811203
1116.55657	-0.50077764	1.0212996	0.811247
3953.75718	0.21421792	0.97900598	0.811452
2728.33316	0.36496098	1.0212689	0.811525
2086.97832	-0.06670739	0.9790243	0.811607
3626.78464	1.49244757	0.9790281	0.81164
1776.89324	2.30164381	0.97902832	0.811641
1672.83684	4.97435897	0.97904676	0.811798
2880.34751	-0.73185722	1.02120528	0.812101
4092.0187	-2.91074825	1.02118988	0.812241
4057.03237	-3.14736489	0.9791382	0.812574
998.515673	-0.52648272	0.97914385	0.812622
2967.5535	2.02377117	0.97914482	0.81263
1096.60984	-1.83465577	0.9791615	0.812772
3200.30381	-16.7815865	0.97917081	0.812851
2307.14189	2.57504833	0.97919034	0.813017
2603.06841	4.23026676	0.97919183	0.81303
2254.0065	-0.70781566	0.97920366	0.81313
2434.23564	1.05782769	0.97928187	0.813794
3615.51113	-1.43636555	1.02101373	0.813836
1653.82413	0.04990375	1.02098209	0.814123
2528.19577	1.6365815	0.97932915	0.814196
2491.28325	5.59367296	1.02090479	0.814824
2774.30271	1.61705216	1.02085472	0.815278
1983.00505	0.59212233	0.97949334	0.81559
1528.78716	0.60091671	0.97949343	0.815591
2307.09038	0.85446588	1.02081038	0.81568
1306.59134	0.30418546	0.97950777	0.815713
2586.13456	-3.9528737	1.02079674	0.815804
2999.30216	0.87716263	0.97953706	0.815962
2169.02671	-2.23684861	1.02076461	0.816095
2085.93047	-0.722833	1.02076375	0.816103
3586.92075	2.01236238	0.97955795	0.816139
2123.9251	-0.00888597	1.02074377	0.816284
2778.25193	0.12487787	1.02069596	0.816718
2117.02226	3.23666678	0.97962702	0.816726
1973.9041	1.65713883	0.97963118	0.816761
2863.26303	-5.17754017	1.02066989	0.816954
1745.8665	-0.49008508	1.02065837	0.817059
1618.76328	-0.32711315	0.97968028	0.817179
2388.20469	-0.18142027	1.02064216	0.817206
2293.1625	0.43738369	0.97969978	0.817344
2020.8156	-3.34466116	0.97971454	0.81747
2180.04497	3.53578658	0.97971642	0.817486
2829.50699	2.37280211	1.02059459	0.817637
1647.82073	-1.59725525	0.97980051	0.8182
3138.33762	-0.24270761	0.97983561	0.818499
2973.44082	-1.26846164	0.97983982	0.818534
2191.00102	-1.66323005	0.97987204	0.818808
1742.91741	-2.11100239	1.02045914	0.818867

Information Classification: General

1445.67949	2.16691054	1.02045633	0.818893
2258.17087	1.60450865	0.97992036	0.819219
2080.89433	-0.71199765	0.97993917	0.819379
2851.41977	-0.08009278	1.02038827	0.819511
2235.15158	3.052207	0.97996361	0.819587
2446.0402	0.69295066	0.97996791	0.819623
1341.61064	-4.6153466	0.9799749	0.819683
1568.74468	-4.18729389	0.97999652	0.819866
2120.84104	0.2835056	1.0203485	0.819872
2185.05205	1.41930343	1.02032636	0.820073
1386.64043	0.57921153	1.02031607	0.820166
1626.76426	1.6886551	0.98008391	0.820609
1938.9345	-3.00652708	1.02026199	0.820658
4140.79262	4.27670415	1.02024505	0.820812
2420.15805	2.49234626	0.98011145	0.820844
2301.05815	-2.50981597	1.02022651	0.82098
1817.80387	6.0792368	0.98014578	0.821135
1248.61186	0.9920657	0.98015531	0.821216
3507.76462	-1.52368984	0.98017149	0.821354
2149.01103	1.2278331	1.02018435	0.821363
2607.34769	0.8879147	1.02017531	0.821446
1872.90129	1.62871153	0.98019773	0.821577
2783.33257	-0.84940263	1.02015118	0.821665
1969.98601	8.74128686	0.98021324	0.821709
3555.76133	0.05156596	1.02009196	0.822203
1429.73052	0.34625319	0.98030705	0.822507
1496.63152	-4.80276849	1.02003607	0.822712
1959.86112	-1.50376129	1.02003408	0.82273
2442.17656	3.19181289	0.98034005	0.822788
3479.58323	-1.21285494	1.02001868	0.82287
2784.19316	2.44039574	1.02000319	0.823011
1737.82779	0.75245741	0.9803695	0.823038
2279.98833	-0.68265755	0.98037355	0.823072
1786.8112	-1.00205029	1.01997463	0.82327
2540.08481	-2.33587304	0.98039775	0.823278
3513.5665	-6.93764085	1.01995237	0.823473
1460.77165	-1.84235225	0.98048199	0.823995
2583.28342	4.40682688	0.98049235	0.824083
2476.21964	0.0134659	0.98049704	0.824123
1744.82756	-0.02180892	1.01987376	0.824188
2040.00383	4.12081502	0.98052066	0.824324
2464.15757	0.0249946	0.98052106	0.824327
3643.6124	-2.87885314	1.01983289	0.82456
2325.06255	2.78672019	1.01983123	0.824575
1829.87969	-0.35468418	0.980566	0.82471
1381.5811	0.74768412	0.98058058	0.824834
3143.51308	0.76059151	0.98059802	0.824982
1388.69023	-0.7526188	0.98061015	0.825085
2016.043	0.73490612	1.01974324	0.825376
1296.71929	1.98777024	1.01973124	0.825485
2449.10088	0.77513217	1.01972467	0.825545
3122.34592	-2.32162527	0.98071575	0.825984
2286.95359	2.20822078	1.01965968	0.826137
1423.627	-0.01726312	0.98077743	0.826509
1171.58623	1.8384813	1.01960884	0.8266

Information Classification: General

1669.81729	5.74709255	1.01960459	0.826639
2390.18497	-1.43822518	1.01959975	0.826683
2553.12284	-0.17680829	0.98082242	0.826892
2044.94792	1.04563803	0.98082546	0.826918
3064.5419	-0.78772963	0.98083691	0.827015
2598.21585	0.67129328	0.98088185	0.827398
1694.84587	1.85886759	0.98088659	0.827438
1356.6281	-0.4414942	0.9809059	0.827602
1269.63359	1.18713039	1.01949439	0.827643
1729.83989	1.65336039	1.01947894	0.827783
2441.98368	-0.61776397	1.01947215	0.827845
2792.43747	0.28212527	0.98095129	0.827989
1652.78557	-1.12854344	0.98096529	0.828108
3426.64048	1.90383173	1.01938299	0.828658
2338.35221	1.74169823	1.01937014	0.828775
1427.72539	-2.28958902	1.01936736	0.8288
1760.76335	3.09238763	1.0193672	0.828802
1521.59099	3.05908854	1.01936153	0.828853
4729.19008	-1.87307284	1.01934955	0.828963
2231.99175	-2.12925631	0.98107586	0.829049
1525.74895	-0.20077619	0.98108109	0.829094
1726.94895	-0.3767184	0.98114063	0.829601
1495.68376	-0.51206074	1.01923458	0.830011
2690.11136	-1.19438277	1.01921594	0.830181
1310.69098	0.46337289	1.01920805	0.830253
2084.03325	2.82597398	1.01919694	0.830354
4121.93422	-3.47737051	1.0191914	0.830405
2834.41385	-2.42476527	1.01917936	0.830515
2747.22379	3.40479549	0.98127328	0.83073
2887.47109	-0.56390456	1.01914022	0.830872
1750.88896	-2.40259989	0.98130834	0.831029
2486.26645	0.99398412	0.98131369	0.831075
2029.99297	-0.48166639	0.9813707	0.83156
2380.14695	0.44428914	0.98138953	0.831721
3121.53447	-3.01578381	1.01901081	0.832052
1257.63457	1.22514374	1.019008	0.832078
2636.32907	-2.4619917	0.98143925	0.832144
2461.42957	0.5612142	1.01898909	0.832251
1768.84172	1.11604301	0.98146167	0.832335
3150.23142	-0.44151369	0.98147171	0.832421
1630.76383	0.82280318	0.98150044	0.832665
2510.9661	-0.7159374	1.01894214	0.832679
3774.63608	-0.82412829	1.01893452	0.832749
1886.75408	3.55617043	1.01892453	0.83284
3049.43186	1.38044823	0.98152143	0.832844
3437.76157	-0.1819507	0.98155508	0.833131
1628.76811	1.3480771	0.98157448	0.833296
1116.55693	-0.18175126	0.98160233	0.833533
1915.78044	1.30119637	0.98161242	0.833619
2567.03592	-7.38301931	1.01883192	0.833686
1690.75884	-0.55929842	1.01882738	0.833727
1978.98062	2.52133477	1.01882487	0.83375
2146.05887	2.77753003	1.01882047	0.83379
2190.20376	1.32624989	1.01879007	0.834068
1524.75537	0.01274205	1.01878326	0.83413

Information Classification: General

1204.64275	-0.09173337	1.01877338	0.83422
2781.17436	1.04837188	1.01871219	0.834779
1507.83844	1.73909624	0.98175135	0.834803
1739.96628	-0.36183981	1.01869405	0.834945
2581.30596	2.24983089	1.01865914	0.835264
1339.72364	0.82714616	1.01864748	0.835371
3428.7114	-0.38860126	1.0186404	0.835435
4116.9645	3.3099496	1.01863151	0.835517
1198.6303	-1.67831384	1.01862361	0.835589
2694.38779	0.82475136	0.98185226	0.835663
2882.37778	6.90937687	1.0185881	0.835913
1858.92278	0.18009943	1.01856412	0.836133
3240.72523	0.03353758	1.01854008	0.836352
1248.64678	-0.94677346	0.98195349	0.836526
2126.08811	6.93742783	0.98195713	0.836557
2274.21292	0.23413698	1.01847975	0.836904
1856.92278	-3.11871292	1.01846833	0.837008
2295.08567	10.178782	1.01845907	0.837093
1599.82366	0.16732918	0.98202835	0.837164
2305.92314	-0.58674901	0.98202885	0.837168
2529.18454	-1.64197995	0.98203737	0.837241
2920.35385	10.3369847	0.98207628	0.837573
2432.20171	-0.25513715	0.98209701	0.837749
2141.9063	-5.44267565	0.98210118	0.837785
2217.96897	2.09739536	0.98212806	0.838014
1883.83977	0.81375203	1.01834852	0.838104
1401.63481	-2.24885699	0.98214691	0.838175
2619.26749	-1.94141797	1.01832441	0.838325
1297.67521	-0.28762915	0.98219986	0.838626
1587.78557	-2.818542	1.01828378	0.838696
2483.33054	0.10519076	0.98224242	0.838989
2996.4003	2.45541526	1.01823297	0.839161
2798.37906	1.20182884	1.01820541	0.839414
1729.79558	0.3899451	1.01818957	0.839559
1884.84953	-0.08191453	0.98232516	0.839695
2003.0584	1.96844392	1.01814264	0.839988
2351.15361	-3.63236501	0.98239787	0.840315
1997.93096	0.57084149	0.98241574	0.840467
929.436083	-0.21792246	0.98242099	0.840512
1948.79216	0.92701346	0.98243073	0.840595
3700.61057	0.18983903	1.01805401	0.8408
3578.67881	1.5834964	0.98246012	0.840846
1505.62871	0.6006847	0.98246339	0.840874
2637.21709	4.24171162	0.98246946	0.840925
1864.87236	1.34687603	1.01803535	0.840971
3459.728	-3.21552419	1.01801736	0.841135
1365.59905	-0.02392929	0.98249779	0.841167
2687.12065	1.78807286	0.98250269	0.841209
2107.14189	0.29993323	1.01800337	0.841263
1939.96343	-0.63547332	0.9825099	0.84127
2376.07598	5.20902246	1.01799494	0.841341
4467.09153	-0.626011	1.01795521	0.841704
1861.83147	-1.25510974	1.01790861	0.842131
1532.76262	-0.50983698	0.98261696	0.842184
1192.62358	2.32886138	1.0179023	0.842189

Information Classification: General

2167.13449	-6.44939134	1.017899	0.842219
2495.31241	0.57058268	0.98262338	0.842238
2036.8991	2.94081839	1.01785345	0.842637
1424.65434	-0.3747058	0.98269522	0.842851
2742.11094	0.18114922	0.98271085	0.842985
2317.93218	0.99148932	1.01781323	0.843005
1743.77239	0.91821957	1.01780487	0.843082
1090.53838	-2.83128333	0.98272981	0.843146
1467.70366	4.48495168	1.01779353	0.843186
4614.23508	-1.00314805	1.01779216	0.843198
2340.03238	-0.86559266	1.01778482	0.843266
1769.9279	-0.57304929	1.01773608	0.843712
3045.37058	-1.08351807	1.01773394	0.843732
1933.898	2.76204718	1.01771948	0.843865
1392.67449	-1.30125504	0.98281612	0.843883
2968.27365	0.71066824	1.01769688	0.844072
3396.54673	0.85991659	1.01768843	0.844149
1471.62908	1.39071665	1.01768128	0.844215
2640.16367	1.6366812	0.98287544	0.844389
4179.85544	-1.51211756	0.98288425	0.844464
3004.37088	-2.61437039	0.98289906	0.84459
2055.04776	-0.32488562	0.98290742	0.844662
3384.45962	0.12620405	1.01761982	0.844778
3759.89682	-0.51719421	0.9829375	0.844918
4146.89077	-0.01700642	0.98297673	0.845253
2976.37479	-4.43972591	1.0175397	0.845513
2257.13432	0.78579395	1.01753009	0.845601
3330.26694	1.00610272	1.01752676	0.845632
1671.74626	2.15410624	0.9830315	0.845721
2347.12918	-3.15737075	0.98311478	0.846432
1819.86911	-0.95147382	0.98316999	0.846903
2809.23905	2.08544881	1.01738109	0.846968
1622.85564	3.10571005	1.01737777	0.846999
3734.71836	1.02139061	1.01737413	0.847032
1551.80168	-0.35762241	0.98322186	0.847346
2199.08896	0.35518117	0.98322478	0.84737
2263.09599	-0.72486347	1.01733108	0.847427
2490.05307	-0.35003144	0.98327877	0.847831
2668.28006	7.17134212	1.01728554	0.847845
1724.71616	0.86062893	1.01727915	0.847904
1553.72063	0.91609354	1.01726996	0.847988
2692.36795	-1.37471023	0.98329894	0.848004
1527.68437	0.31909119	0.9833147	0.848138
1428.62102	-1.89414934	1.01723616	0.848299
2183.05107	0.36540115	1.01723207	0.848336
2053.05576	1.27173847	1.01721853	0.848461
2225.96099	-1.28987043	1.01718791	0.848742
2909.40566	0.9366361	0.98339819	0.848851
2318.20017	-5.33209793	1.0171702	0.848904
3343.48386	1.13969024	0.98342739	0.8491
1821.9384	0.14643247	0.98343966	0.849205
3340.50088	2.74207326	0.98349185	0.849651
2650.19219	-3.37938864	1.0170878	0.849661
1546.81072	-0.26425724	0.98350568	0.849769
2720.18131	-1.68287812	0.98351714	0.849867

Information Classification: General

1848.93381	3.54026076	1.01706531	0.849868
2601.23233	5.22469941	0.98353699	0.850036
2378.18662	-0.3434024	0.98354551	0.850109
1686.93169	-0.29644132	1.01703589	0.850138
2036.94548	0.40888137	0.98355961	0.850229
1858.84807	-0.34927024	0.98359458	0.850528
1554.74089	0.94358187	0.9836025	0.850595
4174.05922	-0.86977503	1.01697027	0.850741
3572.71255	-0.94778966	1.01695165	0.850912
3712.77187	1.06604769	1.01695035	0.850924
1933.95865	-1.91109752	0.98365036	0.851004
3288.62583	-0.67830478	1.01693877	0.85103
2801.23825	0.52693382	0.98366627	0.85114
3376.61936	-2.8106269	0.98368658	0.851314
2953.42307	1.6206687	1.01687361	0.851629
2969.37778	1.33342224	1.01687006	0.851662
2686.15061	0.41456455	0.98373792	0.851752
2181.09043	-0.85144624	1.0168536	0.851813
3391.52451	-1.94309373	1.01683568	0.851978
1388.65483	0.6390753	0.98377498	0.852069
1670.71269	-0.78482487	1.01681145	0.852201
3298.71311	9.69153175	0.98379124	0.852207
1855.81914	-0.0462892	0.98380048	0.852286
1925.82158	-0.31077548	0.98382978	0.852537
2885.31621	-2.03395103	1.01675773	0.852695
2068.93633	2.74710998	1.0167396	0.852861
1507.81583	2.8947506	0.98388345	0.852995
2776.33902	1.86364493	1.01668403	0.853373
2131.90386	-0.56245853	0.98393602	0.853444
3750.598	3.77013963	0.98393673	0.85345
2637.25119	-3.71541388	0.98394133	0.85349
1911.83219	-2.21140086	1.01666014	0.853592
1520.81011	0.98295803	0.98396434	0.853686
1908.82866	-3.76902346	1.01663214	0.85385
1543.87639	1.58177961	1.01661208	0.854034
1487.77056	-2.33227411	1.01660917	0.854061
1013.47948	0.93090301	0.98401517	0.85412
3419.53818	2.41430993	1.01659786	0.854165
1437.74089	4.69070981	1.01658131	0.854317
2167.9915	1.33781746	1.01655903	0.854522
2010.85442	0.58652478	1.01654501	0.854651
1938.93454	-7.28692485	1.01653143	0.854776
1135.57148	-3.20763364	0.98409785	0.854827
2335.08506	3.52998919	0.98411007	0.854931
1747.76987	-1.85649262	0.98414771	0.855253
1595.7376	0.81537646	1.01646982	0.855343
1977.93791	-1.5240571	0.98416857	0.855431
1549.71062	-4.23525019	0.98417576	0.855492
2404.20627	-11.5074796	0.98419154	0.855627
2830.47988	3.36720443	1.01641278	0.855868
3045.43357	0.37484554	0.98423149	0.855969
1452.66643	1.58614162	1.01638702	0.856106
1156.57402	0.73225588	0.98424972	0.856124
1302.59929	-0.0910005	0.9843039	0.856587
1775.76909	-1.55692118	1.01633209	0.856611

3939.89687	5.12678188	1.01632647	0.856663
3145.39321	0.20057349	0.98431851	0.856712
1059.57182	2.69603479	1.01631782	0.856743
1325.65996	0.52910109	1.01626172	0.857259
2473.32035	0.36321714	1.01624449	0.857418
1934.78081	0.00982928	0.98441247	0.857515
3429.44492	-2.3726562	0.98442348	0.857609
1957.87639	1.05832636	1.01621974	0.857646
1010.55132	-1.25789752	1.01620375	0.857793
2284.28958	0.26425441	1.01619957	0.857832
3205.4509	-0.37886977	0.98448537	0.858138
3300.59804	13.0136619	1.01614987	0.85829
2982.47513	-0.21709868	0.98451761	0.858414
1493.71623	2.95827419	0.98453161	0.858534
1614.71855	2.88914629	1.0161195	0.858569
1194.59953	-0.44399529	0.98456004	0.858777
2386.24985	0.80869439	0.98466861	0.859705
2279.07859	-1.15524826	0.98467094	0.859725
1443.74265	-1.44028547	1.01599017	0.859761
1609.83708	-4.36352753	0.98471534	0.860104
2431.32199	0.39620485	1.01593947	0.860229
1639.6928	-0.1419033	0.98475788	0.860468
2441.08371	-1.27113561	1.01590847	0.860515
2372.04228	1.73716417	1.01589203	0.860666
2145.96074	4.73990876	0.98479668	0.8608
3550.79213	3.04148014	0.98480837	0.8609
2300.06211	7.02224788	0.98481083	0.860921
3551.63027	0.64517678	0.98483752	0.861149
1905.9296	-0.76011096	0.98484956	0.861252
2462.17607	0.20457082	0.98486952	0.861422
911.445421	-1.56253579	0.98493484	0.861981
2412.06133	-2.98019138	1.01572056	0.862247
3622.57395	-8.52752045	0.98496691	0.862255
1433.71489	1.49475238	1.01569116	0.862519
2515.02641	-2.90315589	1.01565432	0.862859
984.536364	-0.58381112	0.98504605	0.862932
1943.80229	-0.28892213	1.0156062	0.863303
1646.73052	3.08857381	0.98511906	0.863556
2132.98369	-3.14354168	1.01555801	0.863747
1704.68401	-0.02389904	0.9851735	0.864022
1818.7985	-0.69399309	0.98519704	0.864223
1321.60259	0.56095626	0.98525487	0.864718
4195.827	-3.54967343	1.01543171	0.864913
2074.03472	-0.06399724	0.98530508	0.865147
2176.98198	2.36377358	0.98530548	0.865151
1605.84453	-2.31982568	1.01538864	0.865311
1447.70622	-3.05168505	0.98532699	0.865335
1385.58928	0.26032042	1.01538581	0.865337
2169.07761	1.4819829	1.0153803	0.865388
2059.95664	0.15041882	1.01535395	0.865631
3749.68723	-0.80388663	0.98536721	0.865679
2842.26743	-0.06056014	0.98537806	0.865771
2154.12993	0.86304509	0.98538497	0.865831
1576.69292	-1.17436152	1.01532217	0.865925
3132.58957	3.7159118	0.98539617	0.865926

3460.62026	-2.9380098	0.98540871	0.866034
1655.72161	-1.64525489	0.98542835	0.866202
1441.70952	-1.25409798	1.01526629	0.866441
2103.95464	1.50627891	0.98550971	0.866898
2686.19419	-2.25907556	1.01521063	0.866955
1572.65386	-0.57807222	1.01519515	0.867098
2430.06279	0.59111204	0.98554114	0.867167
4415.01577	-5.00349827	1.01517736	0.867263
1333.65166	1.65232567	0.98555295	0.867268
2492.08379	1.68474798	1.01514354	0.867575
2432.16728	-2.13569731	0.98564074	0.868019
3464.61289	-4.77488951	0.98564576	0.868062
3684.7313	0.92491395	1.01508096	0.868153
2382.00949	0.37627959	1.01506484	0.868302
1662.72515	0.60139525	1.01505899	0.868356
2684.30747	7.17314786	1.01505166	0.868424
3249.56174	-0.92123336	1.01503927	0.868539
2459.16567	-0.14036468	0.98570597	0.868577
2953.40932	3.98731177	0.98572835	0.868768
1290.61846	0.26925221	0.98573581	0.868832
2123.90825	-1.63263938	0.98575084	0.868961
3092.40337	-4.31909656	1.01498135	0.869074
2683.39492	0.42217672	0.98576604	0.869091
3687.65915	3.63306481	1.01496791	0.869198
2427.27536	0.76029371	0.98579858	0.869369
1581.73899	-0.61862984	0.98580497	0.869424
2477.01543	3.35046687	0.98582137	0.869564
1760.89421	2.56706949	0.98582854	0.869626
1835.98467	-1.32439049	0.98583297	0.869663
3007.49697	0.7157607	1.01491764	0.869663
3432.52695	2.93030656	1.01488689	0.869948
2766.14609	-1.2992874	1.01488552	0.86996
1506.80225	-0.67598219	1.01487504	0.870057
2300.18753	-0.65827139	0.98589984	0.870236
3076.37986	0.05123869	1.01483929	0.870388
2443.95024	3.37470506	1.01478853	0.870857
2318.17875	1.43810733	0.98597777	0.870903
1752.82402	-2.64321564	1.01477945	0.870941
3034.43462	4.00193538	0.98599025	0.871009
3179.53408	-2.03079342	1.01474084	0.871299
2189.20205	-2.05427675	0.98605614	0.871573
1755.85215	0.23787239	0.98606582	0.871656
2286.21659	1.21099057	1.01469	0.871769
1932.94353	3.29742901	1.01468334	0.871831
1542.76462	1.64072643	1.01467659	0.871893
2957.34464	4.64854327	0.98610651	0.872004
2951.28513	2.46776129	1.01464497	0.872186
1423.56902	0.59148893	0.98613887	0.872281
3825.01723	-3.4979398	1.01462756	0.872347
3096.44126	3.95260102	1.01462025	0.872414
1535.7509	-5.73413895	0.98615693	0.872436
1648.75493	-0.48999929	0.98616254	0.872484
2696.28092	14.3800452	1.01459574	0.872641
1753.94573	-0.25940957	1.01457607	0.872823
2463.04936	0.14807052	0.98622621	0.873029

2389.06949	1.60349465	0.98623451	0.8731
1840.75652	1.27557626	0.98626103	0.873327
2291.1834	2.58334105	1.01449504	0.873573
2589.10991	3.86062405	0.98629094	0.873583
2768.1716	-0.89637631	1.01443655	0.874115
3299.5997	-0.25661825	1.0143959	0.874491
2542.06546	0.48367749	1.01439007	0.874545
2575.16509	2.76035167	0.98641825	0.874673
2181.90923	3.53084636	0.9864276	0.874753
3186.47092	0.91451731	1.01434854	0.87493
1711.72173	-1.17192749	0.9865108	0.875465
1300.60059	-0.71945818	0.9865179	0.875526
1922.88005	1.26590401	1.01428086	0.875557
2383.02495	2.78244779	0.98653461	0.875669
1994.92168	1.37005786	1.01425944	0.875756
1629.65715	0.6957236	1.01425904	0.875759
2498.17344	5.02196168	1.01425013	0.875842
1434.747	-0.99143547	0.98656468	0.875927
3017.25528	-1.16268724	0.98657824	0.876043
1715.7363	0.48139227	0.98659714	0.876204
2426.10953	0.72252412	1.01420787	0.876233
1207.56279	3.21013483	1.01417121	0.876573
1775.91483	-0.24338204	1.01411338	0.877109
2608.28006	2.07613795	1.01410881	0.877151
1902.77214	-0.09090667	0.98671066	0.877176
1685.88645	0.44630916	1.01410552	0.877182
1402.63689	-0.25443573	0.98671792	0.877238
2177.99613	-0.11221216	1.01408475	0.877374
1771.83501	5.35051865	0.98675206	0.877531
2157.09697	3.3897998	0.98675384	0.877546
4063.89328	-0.42610271	0.98675454	0.877552
3040.28208	-0.08146962	0.98679279	0.87788
2554.15207	1.70484233	1.01402942	0.877887
3061.34891	-6.49458625	0.98679458	0.877895
3143.48764	0.78088922	0.98685892	0.878446
2370.15781	2.4322141	0.98686164	0.878469
1925.72893	-0.2626877	1.01396354	0.878498
1205.58769	0.52155735	0.98686769	0.878521
3175.33115	2.43898184	1.01395352	0.878591
3497.73813	3.52999464	0.98688244	0.878647
2187.86577	-1.20896128	1.0139278	0.878829
3288.65659	-0.92286069	1.01392477	0.878857
1225.53923	1.73545571	1.01390713	0.879021
2221.9605	-0.1797883	0.98694635	0.879195
1775.90769	0.36919765	0.98698535	0.879529
2552.18296	-1.55231206	1.01383194	0.879718
2939.5854	10.1126993	1.01382196	0.879811
2295.13628	0.11318173	0.98703433	0.879948
3033.56449	-0.16845617	1.01380563	0.879962
2079.97556	1.40422548	0.98706642	0.880223
1155.59001	0.74258089	1.01375282	0.880452
2070.02251	2.10637142	0.9871213	0.880693
2124.17429	1.5964902	1.01372629	0.880698
3537.78883	3.68233367	1.01371456	0.880807
2678.34463	2.09715287	0.98713676	0.880825

Information Classification: General

2585.18753	-0.5535953	0.98714905	0.880931
1538.7918	-0.8205492	1.01369926	0.880949
3381.64084	0.95337085	1.01367091	0.881212
2471.15586	0.66713973	1.01364442	0.881458
2452.04129	1.22698503	0.98725624	0.881849
1493.76445	2.73376142	1.01358618	0.881999
2634.13994	2.43034805	1.01358188	0.882039
2598.23362	2.99526388	0.98728174	0.882067
2663.23844	3.98581385	1.01354524	0.882379
1660.79729	2.30040699	0.98732715	0.882456
4833.51479	-0.96143003	1.01353414	0.882482
1986.05547	0.62043616	1.01350927	0.882713
2013.01604	0.79508044	1.01350735	0.882731
2872.35257	3.9751091	0.98737435	0.88286
1486.70513	2.1190643	1.01347655	0.883016
2937.3693	-3.59895242	0.98741921	0.883245
2239.15519	-1.41829405	1.01344799	0.883282
2163.06809	2.35454981	1.01344113	0.883345
1548.6646	0.71615248	0.98748261	0.883788
2298.9567	-0.55421165	0.98749476	0.883892
2068.85552	1.16975242	0.98749877	0.883926
2220.02048	0.04307604	1.01334466	0.884241
2287.11655	1.20893079	0.98757582	0.884586
4619.33607	1.65727926	1.01326666	0.884966
1234.53215	-1.03942944	1.01325505	0.885074
3237.57529	4.86728449	0.98764098	0.885145
1746.90122	-0.49062054	0.9876441	0.885171
3113.42319	3.38617335	1.01322556	0.885348
1223.5916	1.12376803	0.98767889	0.885469
1441.64238	-1.20259844	0.98769813	0.885634
2104.91983	-3.23306515	1.01318251	0.885748
1736.69902	0.54523444	0.98774539	0.886039
1559.81797	0.21219223	0.98774721	0.886055
2067.00144	1.21560677	0.98775733	0.886141
1862.9102	-2.02610477	0.98776424	0.886201
3791.79116	2.59457888	1.01313037	0.886232
1462.74224	-0.09584823	1.0131134	0.88639
2680.31925	3.05923242	1.01303794	0.887091
2480.11521	-6.41414067	1.01301743	0.887282
2020.94034	0.52028727	0.98789844	0.88735
1503.80545	-0.54144356	0.98794519	0.887751
1288.6287	0.52843905	0.98794908	0.887784
3035.16922	2.36965551	1.01295144	0.887895
1342.68889	2.30289549	0.98796492	0.88792
1176.59075	1.06285124	0.9879812	0.88806
1766.8272	0.37170372	0.98798681	0.888108
1571.80412	-0.08687648	1.01291321	0.888251
2443.23117	-2.00718253	1.01287744	0.888583
2012.92681	1.437266	0.98805522	0.888694
1938.88982	0.48883983	1.01286168	0.88873
1974.79558	2.3524082	1.01285681	0.888775
2081.89653	1.4778226	0.98807984	0.888905
2733.53409	1.21581419	0.98808239	0.888927
1368.65311	-0.8219583	1.01282255	0.889094
2257.0977	-1.7613111	1.01281332	0.88918

Information Classification: General

2019.96878	-0.67533106	0.98811239	0.889184
2736.06113	-1.07817629	0.98812663	0.889306
2373.13652	2.01290911	0.98813064	0.88934
2607.19707	4.42267417	0.98816095	0.8896
1258.56133	-0.5151949	1.01276319	0.889646
2540.10898	1.19152614	1.01273701	0.88989
1303.52007	-0.88082265	0.98819696	0.889908
1572.78777	-2.27047461	0.9882087	0.890009
1733.68938	1.26513634	1.01272129	0.890036
3068.46878	-2.89530325	1.01271708	0.890075
2290.17143	4.911867	1.01269679	0.890264
1656.70207	1.25409751	0.98826473	0.890489
3394.52341	3.2087913	0.98826557	0.890496
2419.21433	2.99282534	0.98827809	0.890604
3870.96164	1.47114324	1.01261256	0.891048
3120.52861	2.62575765	1.0126034	0.891133
2046.78679	-1.12348643	0.98834256	0.891156
1297.63689	7.61624392	0.98835673	0.891278
1586.74564	-2.02217597	0.9883865	0.891533
2477.19138	0.59788162	0.98840212	0.891667
2764.28446	2.38386482	1.01253287	0.891789
2109.89678	0.76203461	0.98842599	0.891871
2510.12217	3.23338844	1.01250196	0.892077
2441.30229	-0.23250193	1.01248243	0.892259
1929.85967	1.40862023	1.01247503	0.892328
2394.14263	-2.81085019	0.9884795	0.89233
1909.76518	0.40581822	0.98849028	0.892422
1489.73796	-1.57454683	1.01245031	0.892558
1757.75994	1.51328632	1.01244218	0.892633
3289.61863	-1.42656022	1.01243574	0.892693
2016.98418	-1.54034023	0.98854404	0.892883
1698.71013	-0.81749877	1.01241103	0.892923
4684.25681	0.68799701	1.01240469	0.892983
2064.02617	0.86498207	1.01240457	0.892984
2714.46645	-1.72038597	0.98856156	0.893033
2870.42685	-1.03758087	0.98858363	0.893222
2224.9777	-5.40095969	1.01236627	0.89334
1738.81658	2.05922759	0.98861848	0.893521
1552.82256	0.54871973	0.98862686	0.893593
2017.94016	1.36047843	0.98862934	0.893614
2994.49436	-0.28733305	0.98866981	0.893961
2348.04961	2.12425053	1.01228918	0.894058
1280.64751	-1.19452366	0.98868856	0.894122
3609.72353	2.85344516	1.01226912	0.894245
2116.08208	0.17322048	1.01226184	0.894313
1439.56365	0.38618061	1.01225157	0.894409
1644.80522	-4.41080798	0.98874078	0.894569
1899.8969	-0.09322364	0.988744	0.894597
3091.40989	2.20123724	0.98874542	0.894609
3467.80996	-0.75294513	1.01222673	0.89464
1602.71843	0.4942152	0.98874991	0.894648
1964.86028	1.36233144	1.01222492	0.894657
1496.78496	1.63778244	0.98877076	0.894826
2624.35434	2.11401878	1.01217991	0.895076
1763.87187	0.69816331	0.98886417	0.895627

1671.81388	1.65546286	0.98886515	0.895636
1395.75127	1.81264523	0.98888359	0.895794
2526.28452	0.03195033	0.98888405	0.895798
1671.95377	0.53542384	0.98889738	0.895912
2646.27427	-1.22481281	1.01206023	0.896191
2411.18755	0.79475309	1.01202971	0.896476
1721.71135	-1.34981413	0.98899968	0.896789
2978.33548	-1.35811729	1.01199575	0.896792
1800.73943	-0.66518144	1.01196221	0.897105
1682.8427	4.93430904	0.98904029	0.897137
2184.02542	0.03959539	1.01195295	0.897191
2793.19406	0.73808479	1.01194198	0.897293
2089.99637	-2.01735136	1.01192926	0.897412
1989.92466	-2.01694351	1.0119081	0.897609
1424.67917	-0.89350928	1.01190754	0.897614
1638.84775	1.36872842	1.01190373	0.89765
3056.27909	0.6041944	0.98912227	0.89784
1416.6259	-2.58092772	1.01187681	0.897901
3360.56028	0.04063357	0.98914351	0.898022
1246.61357	-0.78236805	0.98914409	0.898027
2446.05132	-2.02496569	1.01184432	0.898204
2249.03472	-4.84195839	0.98918262	0.898357
1378.63298	-0.85311388	0.989183	0.898361
1782.80559	1.01036868	0.98919714	0.898482
1047.51665	1.74399257	0.98923099	0.898772
1419.74915	0.94653522	1.01174893	0.899093
2371.08286	-0.35698484	1.01172577	0.899309
1191.60161	1.01946719	1.0116978	0.89957
1313.63335	0.1317567	1.0116855	0.899685
2739.2045	-0.20567074	0.98935453	0.899831
1935.91788	0.73997562	1.0116527	0.899991
4077.92868	-0.13910133	0.98937671	0.900021
2033.91117	0.75650543	0.98938253	0.900071
2344.09165	-4.13054589	0.98939446	0.900173
1854.90837	-0.28869671	0.98940256	0.900243
1468.79961	-1.30048671	0.98940874	0.900296
1512.69458	1.38720196	1.01159364	0.900542
1749.8145	0.46029093	0.98943834	0.90055
1879.88127	1.21544095	1.01157685	0.900699
3240.38303	-3.24206802	0.9894633	0.900764
1270.61833	1.74594644	0.98948211	0.900925
3974.85569	0.50887579	0.98950821	0.901149
1337.66924	-0.94286995	0.98952686	0.901309
1454.73613	-1.44817801	1.01149661	0.901447
2449.17876	-1.72537988	1.01148775	0.90153
1270.6115	0.99361139	0.98955536	0.901553
2609.30117	-1.96609467	1.01147416	0.901657
2886.34165	2.33812303	1.01147206	0.901677
2487.3151	2.08028686	0.98959079	0.901857
3602.64255	-2.46404068	0.98959474	0.901891
1423.7566	1.19903335	1.01143156	0.902054
2730.22629	-0.39221051	1.01137918	0.902543
2398.07956	0.08242468	1.01133097	0.902993
1865.82622	3.9736679	0.98975345	0.903252
2090.09453	2.37727077	0.98977448	0.903432

Information Classification: General

1168.58293	-1.25961027	1.01128256	0.903445
3026.29282	-2.05614707	0.98979148	0.903578
2968.25699	0.19636445	1.01125985	0.903657
2789.39939	2.51268417	1.01125452	0.903707
1697.88323	-0.20907817	1.01124083	0.903835
2912.4706	2.06129484	1.01123361	0.903903
4203.15542	4.76497102	0.9898427	0.904017
3137.54863	3.13342812	0.98987163	0.904265
1712.89249	3.60904556	1.0111914	0.904297
3043.29746	-1.85779778	1.01118825	0.904326
2319.04106	0.5622685	0.98988199	0.904354
2499.20268	-0.23686854	0.98989692	0.904482
2024.96172	0.69592458	0.98991275	0.904618
2365.18961	-1.5004938	0.98991851	0.904667
3770.63706	1.10638549	0.98992027	0.904682
2740.36008	-2.17918841	0.98993167	0.90478
1481.72295	-0.06567397	0.98994807	0.904921
1770.9489	-0.22482986	0.98996779	0.90509
3125.27378	3.74992047	0.98996816	0.905093
1928.9769	0.2076376	1.01105018	0.905616
3058.30588	-1.26932355	0.99002982	0.905622
1505.80204	-6.83037629	0.99003119	0.905633
1613.81485	0.61047169	1.01102752	0.905828
2630.28017	-0.91976015	1.01094693	0.90658
1908.00803	-0.80111937	0.99015589	0.906703
2161.83501	0.80128051	1.01092741	0.906763
2376.03789	2.02063092	1.01091011	0.906925
1892.8947	-0.45506015	0.9901868	0.906968
3060.41764	-0.70565813	1.01088028	0.907203
1922.87456	-1.07962451	0.99021633	0.907221
1509.69658	0.78923268	0.99024152	0.907437
1831.81413	0.44175487	1.01084628	0.907521
3048.4332	2.94097186	0.99025782	0.907577
1325.62224	0.23738404	0.99025873	0.907585
2377.08891	1.04307678	1.01082787	0.907693
2112.09629	-0.42076976	1.01082511	0.907719
1735.88506	-1.14692826	0.99029016	0.907854
1685.82291	-0.75640869	0.99029906	0.907931
1681.79111	0.67339665	1.01079872	0.907966
2973.28434	-0.10741988	1.01077844	0.908155
2648.27689	-3.4885623	0.99033078	0.908203
1599.73589	-1.58017178	0.99037537	0.908585
1471.69084	-0.79826739	0.99040832	0.908868
1442.7177	1.06997089	1.01069799	0.908907
2597.20513	0.23233581	1.01069003	0.908982
3555.84739	-0.51269732	1.01067218	0.909149
2589.07305	-0.17058398	0.99045415	0.909261
1190.52593	1.18116285	1.01062608	0.90958
3037.439	0.58065235	0.99055382	0.910116
2187.09477	0.46447976	1.01053837	0.9104
1924.92449	-0.0983024	0.99060006	0.910512
1372.67339	-0.66137911	0.99066538	0.911072
1783.88518	-1.04203193	0.99071891	0.911532
3311.58945	7.45332615	1.01041595	0.911545
3657.7031	-0.36229917	1.0104121	0.911581

Information Classification: General

3613.5062	2.42268939	0.99073652	0.911683
1349.70659	-0.21318152	1.0103853	0.911832
2493.30613	2.34394039	0.99075606	0.91185
2046.93143	-0.50547042	1.0103779	0.911901
1042.45183	0.84466763	0.99079666	0.912199
2330.98003	-0.70838552	1.01034592	0.912201
1434.64458	-0.72871795	1.01027804	0.912836
2112.8721	-0.72405478	0.99092514	0.913301
2975.44608	-1.99305436	1.01019473	0.913616
4699.42541	-1.71115554	0.99098965	0.913854
2028.054	1.0721494	1.01014369	0.914094
1947.93583	-0.32400042	0.99103554	0.914248
2189.97173	1.24156226	1.01011708	0.914343
2948.48745	-0.19801212	1.01011497	0.914362
1014.46434	1.77111434	1.01011059	0.914403
3472.60544	0.33190272	0.99105639	0.914426
1486.60893	0.45675597	1.01010734	0.914434
1609.75835	-1.08085788	0.99108439	0.914667
2189.89945	-0.64938377	0.99109224	0.914734
1229.61628	0.19543286	0.99110595	0.914852
3683.6799	-5.4981819	0.99112077	0.914979
1384.72222	0.21684313	1.01004859	0.914984
2797.04001	-1.23563492	0.99113106	0.915067
2264.09159	1.15613111	1.01003177	0.915141
3088.49009	-2.13789844	0.99114021	0.915145
2628.31743	0.85800567	1.01002422	0.915212
1971.91605	0.47744291	0.99118686	0.915546
2908.33719	2.42339695	1.00996611	0.915756
2346.29897	-1.27877938	1.00994999	0.915907
1398.67424	0.79620733	0.99125039	0.91609
2282.02178	0.64145504	1.00992177	0.916172
2977.37107	1.13038218	0.99127322	0.916286
2905.38432	1.53186016	1.00989306	0.916441
2683.31797	-0.69243331	1.00989068	0.916463
1868.94536	4.61158412	1.00988583	0.916508
3057.33927	1.7123552	1.00988041	0.916559
4228.94126	0.62876184	0.99133925	0.916853
2497.06455	-0.19141976	0.99134261	0.916882
1436.66557	0.26425252	1.00984282	0.916911
2767.52433	-0.59083689	1.00984221	0.916917
995.578295	1.16451822	0.99135193	0.916961
1583.70622	0.4110968	1.00980935	0.917225
1962.93546	2.55925737	1.00978114	0.917489
1609.73369	-0.19576103	0.99142137	0.917557
2364.12847	-0.02230526	0.99142622	0.917599
2749.26055	-2.18809905	0.99142818	0.917616
1287.61223	1.51048265	1.009759	0.917697
1272.64458	0.51117866	0.99144132	0.917728
1906.06198	1.1560673	1.00975214	0.917761
1844.72978	-0.36394117	0.99148029	0.918063
1811.88388	-5.12397626	0.99151186	0.918333
2763.34934	-2.81431968	0.99151583	0.918367
3617.48159	-0.6082163	0.99151765	0.918383
3189.31784	-1.62890289	0.99152016	0.918405
1301.71208	0.91067109	0.99154362	0.918606

Information Classification: General

1844.84209	1.34066454	0.991548	0.918643
1914.92571	-1.76430947	1.00964571	0.918758
2252.97856	1.55967918	1.00960592	0.919131
2041.00957	0.10327395	1.00960075	0.91918
2048.99759	-5.03931969	0.99161592	0.919226
1718.83159	-1.64340271	1.00958865	0.919293
4773.25849	-11.9505687	1.00957609	0.919411
3499.62229	-3.54659122	0.99163879	0.919422
1504.72455	-0.11053544	1.0095627	0.919536
2739.28252	-1.0148636	0.99165366	0.91955
2862.38156	-12.2357502	1.00956047	0.919557
2088.01334	1.36709682	0.99166119	0.919614
1615.8062	6.18554533	0.99167369	0.919722
2631.09722	1.34820845	1.00953674	0.91978
2172.01169	-0.02183819	0.9916807	0.919782
1406.68071	0.66565301	1.00952325	0.919906
2779.36928	-0.2093636	1.00952048	0.919932
2625.15598	-2.32760395	1.00948981	0.92022
2901.27555	-1.71939971	1.00946141	0.920486
1639.73699	-1.22350395	1.00946048	0.920495
1591.7	-3.56884424	1.00944018	0.920685
2397.07505	0.9374172	0.99178643	0.920689
1931.86321	-1.31920394	0.99180233	0.920825
1874.93113	3.15060156	0.99180245	0.920826
2582.21372	-5.01122952	0.99188433	0.921528
3903.74192	-0.36780696	1.00934346	0.921592
3682.63547	4.80956212	1.00933466	0.921674
2054.14743	1.53141621	1.00930698	0.921934
2119.98198	1.95090927	0.99197281	0.922287
1166.51946	3.33854563	0.99197816	0.922333
2767.29325	-0.88579591	0.99198441	0.922387
3555.7958	-1.15789182	0.99205582	0.923
3555.69321	1.25576691	1.00918682	0.923061
1537.65129	0.23049175	0.99207072	0.923127
3693.56943	-0.84015613	0.99207956	0.923203
1084.52849	1.46117099	0.99210128	0.92339
1415.66619	-1.89310585	1.00913288	0.923567
1477.68035	-0.10817609	1.00912687	0.923624
2327.05387	0.33040067	0.99212998	0.923636
1741.81706	-0.71425393	1.00910907	0.923791
4677.26528	2.7894922	1.00910804	0.9238
2809.15189	1.31011823	0.99218473	0.924105
2494.05571	3.75787967	1.00906186	0.924234
2198.023	1.72178249	0.99222211	0.924426
2896.25974	-1.09816042	0.99222959	0.92449
3127.35422	0.01210738	1.00903282	0.924506
3064.42875	3.1310207	1.00897283	0.925069
2801.25418	1.8985221	1.00896825	0.925112
1536.69817	2.62993477	0.99231915	0.925259
3134.48618	-0.84738869	0.99233481	0.925393
1731.86431	4.94675664	0.99238615	0.925833
2650.31852	1.29542832	1.00887943	0.925946
2891.24137	-1.08781642	1.00885314	0.926193
2554.33047	-4.71209904	1.00879569	0.926732
1748.61724	1.80130831	0.99249639	0.926779

Information Classification: General

2580.0886	-0.53763113	0.99250166	0.926824
1414.65891	-0.56312934	1.0087737	0.926939
2702.16996	-1.86970943	0.992529	0.927059
4669.21352	-0.80326536	0.99255149	0.927252
1536.84148	1.01959951	0.99265674	0.928155
2076.02359	-0.84516253	1.00861469	0.928432
3876.93066	0.69477013	0.99271881	0.928687
1480.73638	-2.43296262	0.99272243	0.928718
2954.26206	2.63950116	1.00858081	0.92875
3346.6664	-0.30769668	0.99274552	0.928916
1584.80088	-0.47252782	0.99278789	0.92928
3090.55368	1.31716032	0.99278887	0.929288
2456.15557	7.92982218	0.99278936	0.929292
1729.85307	1.77855712	1.008522	0.929303
3129.42405	5.26681031	0.99281694	0.929529
1653.76006	-0.10759458	0.9928351	0.929685
1922.06595	5.85610555	0.99285654	0.929869
1418.64914	-1.10405095	1.00843436	0.930126
1699.89971	0.28501792	1.00842751	0.930191
3246.29258	0.29151909	1.0084112	0.930344
2544.19048	0.76212307	1.00839323	0.930513
2025.94621	-2.28718267	0.99294591	0.930635
2500.94267	-6.70177221	0.99294852	0.930658
1283.6071	-3.31256907	1.00837011	0.93073
2042.09587	1.58390902	0.99295836	0.930742
3222.63051	1.51508837	1.00836351	0.930792
3799.79001	-0.78244332	0.99298484	0.930969
3043.43218	0.18012723	0.99298558	0.930976
1281.6353	2.5016516	1.0083413	0.931001
3016.40641	1.8969336	0.99299976	0.931097
2328.07646	-3.55000609	0.99302393	0.931305
1522.68619	0.88401597	1.00829376	0.931448
4327.18946	0.24220907	0.99304144	0.931455
1360.64079	-0.83830423	0.99305658	0.931585
2474.2454	5.95623235	1.00826055	0.93176
1456.72576	-1.40019041	0.99308629	0.931839
1737.91948	3.18938395	0.9930975	0.931936
2530.24363	0.79326786	0.99313536	0.93226
2004.94316	-0.60473878	1.0082061	0.932272
2209.01396	-0.56798447	1.00819021	0.932421
1997.95915	2.09150625	1.00818747	0.932447
1291.52861	2.09577384	0.99316129	0.932483
2593.30167	-1.10492562	1.0081252	0.933032
1550.7341	0.58983456	0.99324195	0.933175
1941.8917	-1.47616368	1.00810864	0.933188
2128.06743	0.41921193	0.99326518	0.933374
1082.50005	0.03907506	0.99327731	0.933478
1876.90019	2.06126204	1.0080547	0.933695
1826.96903	4.06136768	1.00805364	0.933705
991.510424	0.98665157	0.9933045	0.933711
2916.35282	4.69732448	1.00803371	0.933892
2189.86186	0.16654247	1.00803314	0.933898
3234.46506	2.07505961	1.00801653	0.934054
1380.70012	-0.28569496	0.99335887	0.934178
1212.58647	0.82310006	0.99335961	0.934184

Information Classification: General

2031.05783	-2.21896916	0.99337691	0.934332
1761.91477	1.01181913	0.99340188	0.934547
1757.80095	-0.05596819	1.00794008	0.934773
3302.57773	-4.30887474	1.00790213	0.93513
1350.69011	-0.57542784	0.99349452	0.935341
2546.215	-3.37022889	0.99349584	0.935353
2331.94707	0.66072817	0.9935109	0.935482
2422.25882	-0.13544595	0.99352234	0.93558
2792.30124	-3.76379575	0.99353965	0.935728
2471.33548	3.74901391	1.00780162	0.936075
2704.25809	0.85993751	1.00779559	0.936132
2113.07004	1.32800925	0.99359893	0.936237
2777.14067	0.18519002	1.00778029	0.936276
2584.22037	-0.66953794	0.99360891	0.936323
1824.94841	-2.18496228	1.00776304	0.936438
3404.78501	1.82860206	1.0077603	0.936464
2479.21489	0.93257295	0.9936538	0.936708
1548.78996	-0.29100722	0.99366442	0.936799
3113.31809	-3.89293612	1.00772477	0.936799
3141.45683	-0.82146342	0.99369521	0.937063
1572.70603	-0.17262941	0.99369943	0.937099
2502.19187	3.42473675	0.99370181	0.937119
2557.23667	-1.13554092	1.0076742	0.937274
2245.02408	-0.65179528	1.00765917	0.937416
2364.03952	-2.42694824	1.00762409	0.937746
1403.6696	0.52573826	0.99377946	0.937785
3073.34844	3.69010246	1.00761905	0.937793
2961.47293	-1.6737472	1.00760175	0.937956
1765.93791	0.47482399	0.99380318	0.937989
1099.4904	0.20804553	1.00758645	0.9381
3380.53708	2.32927254	1.00753017	0.93863
2136.1016	3.07820206	1.00749946	0.938919
2714.28351	1.58485743	0.99391592	0.938956
2910.39969	-0.565539	0.99391659	0.938962
2844.23801	-1.12792018	0.99393749	0.939141
1948.86016	-1.29833809	0.9939439	0.939196
1602.72661	-0.97658655	0.99397211	0.939438
2316.89922	-2.27535981	0.99397621	0.939473
1827.92497	1.03125489	1.00743776	0.9395
2645.27433	-3.58834446	0.99400175	0.939692
1149.57817	-1.17060439	0.9940712	0.940288
3824.94692	0.47629942	0.99407545	0.940324
2548.15335	-0.50657671	1.00734554	0.940368
2524.17998	-1.00074505	0.99408292	0.940388
2720.36991	0.28521828	0.99408538	0.940409
2332.06523	6.65840964	1.00733794	0.94044
1371.66252	-0.92013545	1.00733481	0.940469
1640.76665	1.02626492	0.99409531	0.940495
3125.34446	-9.74700913	0.99411247	0.940642
1736.97844	-1.02312528	0.99411625	0.940674
2234.01798	-3.4690015	0.99411953	0.940702
3049.34049	0.11017264	0.99414671	0.940935
2918.34739	1.02212868	1.00724882	0.941279
1478.71074	-1.580662	0.99422615	0.941617
3099.40281	-0.76277648	0.99424256	0.941757

Information Classification: General

3238.37326	-1.43009061	0.99425961	0.941904
2059.99175	0.37646631	1.00716519	0.942067
2410.19822	-0.08712782	0.99428001	0.942079
2385.02056	2.77402693	0.994292	0.942182
2494.22843	1.28913896	1.00712616	0.942434
1355.60967	-1.238233	0.99432432	0.942459
2565.06113	-2.44787782	1.00711077	0.942579
3119.54844	4.96954337	1.00710717	0.942613
1129.50115	0.36381686	1.00710027	0.942678
1232.6165	0.63067213	1.00708513	0.942821
1213.64238	-0.68118656	0.99437362	0.942882
2506.0667	4.848909	1.00706518	0.943009
3065.59531	3.99098055	1.00704857	0.943165
1530.76543	-0.05869082	0.9944307	0.943371
2451.98613	1.09147564	0.99443216	0.943384
1157.70105	-0.76691694	1.00699605	0.94366
1804.77019	-0.09591232	0.9944849	0.943836
2472.15781	1.08234646	0.99450524	0.94401
2571.1187	0.99864798	0.99453949	0.944304
2475.16294	0.81072819	0.99454611	0.944361
3263.51528	0.47837833	0.99456156	0.944493
2414.18471	1.40908387	0.99456479	0.944521
1494.66142	0.70668304	1.00689113	0.944649
2576.37172	-0.44383832	0.99458545	0.944698
1508.75871	-0.24570467	1.00688318	0.944724
1229.63933	0.98526884	0.99458985	0.944736
3430.32492	-0.1594633	1.00685818	0.94496
1436.74455	-1.22768192	0.99462512	0.945038
1376.6668	-2.43534193	0.99462671	0.945052
2144.00224	4.27684458	1.00681728	0.945345
1000.4962	1.67625215	0.99469249	0.945616
2267.17472	1.3710763	0.99471553	0.945814
1602.76421	1.68424962	0.99472351	0.945882
1401.68791	0.98845804	0.99475174	0.946124
3209.55027	-0.92979197	0.99476217	0.946214
1926.94097	-1.76431586	1.00672389	0.946225
1236.60845	-1.76229085	1.00671403	0.946318
2354.18406	-3.54821516	1.00667182	0.946716
1728.86662	0.37969799	0.99482945	0.946791
2234.04819	0.40535859	1.00665174	0.946906
2466.26438	0.4294406	0.99485869	0.947041
4075.05507	4.35162631	0.99489658	0.947366
3362.69822	3.52395723	1.0065453	0.947909
3203.5497	0.53659949	0.99497891	0.948072
2420.13407	-2.23373763	0.99500608	0.948305
2321.99956	0.52886207	0.99503109	0.94852
2986.48696	0.2467389	0.9950716	0.948867
2508.14678	3.42396803	1.00643458	0.948953
1664.79325	1.93281837	1.00643435	0.948956
2845.34305	-1.23695789	0.99511568	0.949245
1654.75994	0.27193918	0.99513743	0.949432
2861.14336	1.94986581	0.99514001	0.949454
1752.79619	0.76499303	0.99515023	0.949541
1841.981	2.43017164	1.00636646	0.949596
2861.46054	-0.19357029	0.99518242	0.949817

Information Classification: General

3440.62387	2.79867394	0.99519106	0.949892
1705.84075	7.01328126	0.99525097	0.950405
1802.93718	0.81316633	1.00627888	0.950422
2591.22954	-2.79886966	1.00626897	0.950516
2813.43253	2.87690195	0.99527621	0.950622
1244.62534	-2.03994873	1.00625135	0.950682
2743.31192	-0.78900952	0.99531323	0.950939
3808.70249	-2.14785334	0.99533792	0.951151
3161.69968	5.57575046	0.9953417	0.951183
1400.69121	-0.05610935	1.00619075	0.951254
3356.42544	1.71505225	1.00618451	0.951313
3067.4789	2.19764898	0.99535783	0.951321
2462.12217	-2.89335765	0.99538172	0.951526
2024.94376	0.59331933	1.0061569	0.951573
2280.95395	10.2344501	1.00615473	0.951594
2936.31772	-2.48752351	0.99544689	0.952085
1278.62102	-0.13766516	0.99546102	0.952206
1617.77751	0.68310942	1.00607812	0.952317
2983.30991	1.77565884	0.99551911	0.952704
1487.71404	1.04291317	0.99553566	0.952846
2142.77226	-1.30247363	1.0059932	0.953118
1733.72209	0.58440577	1.00596022	0.953429
2827.27896	-0.41541129	0.9956467	0.953798
2006.76323	-1.1556458	1.00588261	0.954162
4490.26186	-0.69189144	1.00582544	0.954702
2053.98955	-1.40981639	0.99576089	0.954777
2348.08683	-2.70524727	1.00579923	0.954949
2131.97051	1.42322941	0.99581193	0.955215
2993.34867	-0.58173482	0.99582934	0.955364
3603.75806	1.09402465	1.00570993	0.955793
1937.97099	-0.0451171	0.99588073	0.955804
3327.44895	2.18258091	0.99589067	0.95589
3630.74648	-0.32655354	1.00569895	0.955896
3996.91572	-17.8276442	0.99589646	0.955939
1442.7601	-0.41818933	0.99589986	0.955968
1315.57646	0.3179704	0.99590314	0.955997
2470.21506	-0.47942878	0.99591692	0.956115
1388.66704	0.64917379	0.99593373	0.956259
2729.3342	-1.29103544	1.00565043	0.956354
1962.74883	1.52928158	1.00561456	0.956693
2457.05143	-0.54559139	0.99600607	0.956879
1635.7675	-5.03411796	0.99601492	0.956955
1606.59783	1.84652252	0.99603272	0.957107
3140.28989	1.90868028	0.99604414	0.957205
1325.64128	0.5534305	0.99605717	0.957317
2812.10611	0.60060106	1.00550806	0.957699
1843.01225	3.88675115	1.0054628	0.958127
2529.31729	0.09472696	0.99620634	0.958596
2224.96195	0.19277401	0.99622431	0.958749
3161.39682	2.95135462	0.99622845	0.958785
1367.5689	1.3088591	1.00538834	0.95883
2502.12394	-0.91793234	0.99624354	0.958914
1274.60515	3.15771344	1.0053747	0.958959
1566.76604	0.92133072	0.99625437	0.959007
1775.90605	-0.55117886	1.00534018	0.959285

Information Classification: General

1619.8068	0.26641922	0.99629165	0.959327
3074.56125	2.13172337	0.99629205	0.95933
1445.56145	-0.68231901	1.00533479	0.959336
1487.73516	-1.66715115	0.99629378	0.959345
1727.81833	-1.20140397	1.00532383	0.95944
1682.8001	-1.70066137	1.00530053	0.95966
1792.89507	-1.17975114	0.99634114	0.959751
1476.85801	2.56425718	1.00524878	0.960149
2191.01348	0.28937893	1.00520703	0.960544
3736.70505	4.52723508	1.00518236	0.960777
3728.77768	-1.00092368	1.00517867	0.960812
1213.61125	3.65467395	1.00516118	0.960977
1726.82756	-0.40134454	1.00514476	0.961132
3917.91578	4.51246145	0.99650413	0.961148
1127.59355	-1.44985032	1.00512736	0.961297
3439.43496	-0.18929581	1.00511772	0.961388
1421.71648	0.45224405	0.99655824	0.961611
2681.18534	-5.66033727	0.99657404	0.961747
1708.6906	-2.07406972	0.99658045	0.961802
2873.43583	-1.9906942	0.99660348	0.961999
2506.00957	3.31753846	1.00505143	0.962015
2948.27927	2.41282848	1.00504573	0.962068
2135.09453	1.81009345	0.99661403	0.96209
1885.75969	1.41367612	1.00502762	0.96224
2296.09458	0.95175059	1.00501904	0.962321
2636.24663	1.6445085	0.99664604	0.962364
2693.27927	17.154438	1.00499409	0.962557
2011.97087	5.04355913	0.99671894	0.962989
2172.06131	8.41787895	1.00494224	0.963047
1903.83268	1.1768124	1.0049076	0.963374
1223.58304	-1.06517916	1.00489677	0.963477
3035.29355	-1.34882547	0.99678251	0.963533
1618.7498	2.85106328	0.99681123	0.963779
1565.67375	-3.2201131	1.00486383	0.963788
3262.49778	-1.64795467	0.99681633	0.963823
1894.90227	-0.81219047	1.00485074	0.963912
3473.44187	-5.17862462	1.00484599	0.963957
2170.08158	-0.11805036	1.00483635	0.964048
2578.23868	-0.10343756	1.00483297	0.96408
2802.26712	1.40090003	1.00478942	0.964492
2339.26352	0.85694572	0.99692206	0.964729
2078.04277	4.32806689	1.004763	0.964742
2523.14975	-0.11807043	0.99693319	0.964824
3460.49851	0.8579612	0.99693416	0.964833
2919.32975	-1.18185813	1.00470755	0.965266
2493.06792	-4.34669566	0.99700207	0.965414
2520.15177	4.72521177	1.00469191	0.965414
2438.25748	-2.73919332	0.99702253	0.96559
1280.69776	0.87393064	1.00467209	0.965602
1615.78081	-0.68015568	0.99702465	0.965608
1640.7022	-1.76160168	1.00465405	0.965772
1294.63103	-0.22883426	0.99706122	0.965921
2315.09062	-2.86879887	1.00463022	0.965998
1560.64995	1.10251662	0.99707163	0.96601
2487.37204	4.20730373	0.99708919	0.966161

Information Classification: General

2215.01896	2.75562011	1.00461189	0.966171
3777.9426	0.0049684	1.00459428	0.966338
1848.94571	-0.13274415	1.00456258	0.966638
1885.95574	0.31696148	0.9971548	0.966723
2330.08696	0.54991993	1.00449563	0.967271
2601.28067	1.18845337	0.99722823	0.967352
1579.71109	-0.04594343	0.99723127	0.967378
1391.60759	0.17694935	1.00446512	0.96756
1370.68413	2.48877909	1.0044253	0.967937
2977.45131	0.15192566	1.00441654	0.968019
2029.9863	1.84530417	1.00441068	0.968075
2644.21341	3.73788233	0.99732256	0.96816
1767.79978	1.24759869	0.99732404	0.968173
1930.81072	0.04673895	1.00440003	0.968176
2513.20932	-2.96895161	1.00439342	0.968238
1258.59673	1.39996093	0.99733899	0.968301
2470.14603	-1.22990862	1.00437296	0.968432
1528.67168	-3.55200005	1.00434891	0.968659
2106.06113	0.8494738	0.99738123	0.968663
2423.08261	-2.39966282	1.00434615	0.968686
2465.11557	0.38594666	1.00434516	0.968695
1521.72368	3.75568267	0.99742346	0.969025
1784.79497	0.92681444	1.00430707	0.969055
2034.95403	1.39992889	1.00429812	0.96914
3193.38828	2.91210477	0.99748008	0.969509
3654.73606	0.72010715	1.00425216	0.969575
3481.55381	17.4056839	0.99749156	0.969608
2934.33139	2.65774876	0.99752109	0.969861
1789.94748	1.26516718	0.9975406	0.970028
2736.22922	2.22052451	0.99754238	0.970043
2423.22768	-1.7264004	0.99754436	0.97006
2877.46158	1.93528349	0.99755654	0.970164
2482.00815	-1.73386694	0.99759824	0.970522
1956.00748	1.18565058	1.00411913	0.970834
2610.12979	-1.11274369	0.9976582	0.971035
2152.95683	9.8694074	1.00409464	0.971066
2624.04892	0.61241713	0.99767843	0.971208
1426.74175	-1.10845744	1.00406229	0.971373
3993.76609	1.85040843	0.99771045	0.971483
2111.96025	-0.60731405	0.99772189	0.971581
1986.82732	-1.21862299	0.997727	0.971624
2109.14078	2.81769842	1.00402495	0.971726
4165.81892	-0.59948575	0.99776262	0.971929
1699.66448	-0.24239508	0.99778442	0.972116
3709.95864	-0.15887778	0.99779057	0.972169
1396.70488	-1.94509013	0.99781107	0.972344
2140.00493	1.79817868	0.99782075	0.972427
2290.06801	-0.46043611	1.0039184	0.972735
1988.88616	-0.30635379	1.00391406	0.972776
1314.60917	-1.12146509	1.00390937	0.97282
3136.36448	-0.10337755	1.00389989	0.97291
2834.33219	-2.98900298	0.9978801	0.972935
2000.91666	-6.88592952	0.99789064	0.973026
1911.99541	1.62323971	0.99790042	0.973109
1968.89444	-1.66081426	1.00387117	0.973182

Information Classification: General

2893.15775	0.96493048	0.99794002	0.973449
2440.17681	1.34298107	1.00380561	0.973803
2297.1727	4.94839128	0.99798675	0.973849
1739.70317	0.29734886	1.00379864	0.973869
2366.02495	-2.42404777	1.00379629	0.973891
2356.07425	1.21965695	1.00379403	0.973913
3484.59299	-3.31906903	1.00377135	0.974128
1786.88713	-1.65077036	0.99804532	0.97435
2161.11162	0.09650791	1.00371953	0.974618
1772.83733	2.02645338	1.00371763	0.974636
1578.76311	-1.52599069	0.99808198	0.974664
1090.57915	0.31255068	1.0037094	0.974714
2878.16544	2.75631334	1.00370406	0.974765
1839.03765	1.26367995	0.99811127	0.974915
1099.52641	-0.13688962	0.99812912	0.975068
2861.41404	-2.8851759	1.00366295	0.975154
1482.63872	0.27260434	0.99816759	0.975397
2506.94547	-0.9444072	0.99817246	0.975439
2382.96391	0.8055037	1.00360763	0.975678
3064.44962	0.1830062	1.00359395	0.975808
2146.96831	2.38057426	0.99822011	0.975847
1695.82976	3.7746789	0.99825198	0.97612
3655.6893	1.70689338	1.0035495	0.976229
2292.09121	-0.9777406	1.00353568	0.97636
2347.1563	0.48241302	0.99835073	0.976965
1547.79814	1.93694629	1.00347108	0.976972
1617.70232	-0.23316616	0.99837448	0.977168
1980.97856	1.26600247	0.99837607	0.977182
2674.37455	1.98834195	1.00344546	0.977215
1816.87149	-0.34905425	0.99847046	0.97799
1697.66924	0.12414189	0.99849711	0.978218
2746.28684	2.2157378	0.99849976	0.978241
1717.72612	-0.02531399	0.99851198	0.978345
1256.5656	1.22083285	1.00332076	0.978397
1706.72734	1.46140544	1.00330261	0.978569
2290.04697	1.33704135	1.00329898	0.978603
3215.3071	1.54937718	1.00329348	0.978655
2986.53725	0.85411667	1.00328417	0.978743
3368.49753	-1.4580949	1.0031959	0.97958
2261.05844	-0.07540854	1.00319498	0.979589
3060.31784	0.0911379	1.00318655	0.979669
2788.35415	-0.18348369	0.99867858	0.979771
2054.09452	0.4553219	0.99867875	0.979773
3065.40543	-0.514007	1.00316403	0.979882
1188.57793	2.31382231	0.99870086	0.979962
2601.17119	4.1534663	0.9987232	0.980153
1765.90079	1.79664016	1.00312854	0.980219
3260.41953	0.40377664	1.00312516	0.980251
3442.556	1.61452439	1.00311614	0.980336
2529.18491	1.01864463	0.9987735	0.980584
1549.79241	3.76030826	1.00308974	0.980586
1810.97405	1.32267063	0.99878358	0.98067
4179.11538	3.77779741	1.00307432	0.980733
2679.0313	-7.83928925	0.99880956	0.980892
1537.70976	2.47192007	0.99881588	0.980946

Information Classification: General

2198.11772	-0.87571288	0.9988345	0.981106
4630.88784	1.57351873	0.99884724	0.981215
1785.84697	-1.74923897	0.99886041	0.981327
2473.27622	2.87470292	0.99892241	0.981858
3610.5021	2.60756011	1.00295436	0.98187
1996.87444	0.22983277	1.00295323	0.981881
3121.59047	-0.2449048	1.0029467	0.981943
2560.29936	-0.76096375	0.99896664	0.982236
2117.90557	2.08832582	1.00288081	0.982567
1073.51543	0.47801653	0.99900593	0.982573
3051.49765	0.89185101	1.0028311	0.983039
2200.07792	-0.53783926	0.99906188	0.983051
1275.62883	2.59203802	1.00282899	0.983059
2544.19242	-1.82428622	0.99909079	0.983299
1576.84783	0.6155439	0.99911832	0.983534
2577.25797	0.82157963	0.99913536	0.98368
3107.42466	-0.52762844	0.99913785	0.983701
2609.2963	0.14697479	1.00274442	0.983861
903.420824	0.21990409	0.99916611	0.983943
3493.51023	0.24809529	1.00270415	0.984243
2397.89507	-0.47632193	1.00268315	0.984442
2054.99346	0.3929462	0.9992523	0.984681
1849.73943	-0.42212348	0.99925487	0.984703
1504.67461	-1.66159139	1.00264981	0.984758
2745.17143	-0.18031023	1.00258734	0.985351
1227.62737	1.47572035	1.00258193	0.985402
3367.57248	1.63161531	1.00258114	0.985409
1387.64653	-1.64546676	1.00257785	0.985441
2696.28774	2.56357376	1.00257763	0.985443
2564.09892	1.50394037	0.99936121	0.985612
1264.64202	-0.68234947	0.99937026	0.98569
1680.71562	1.99228863	1.00253723	0.985826
2214.8582	1.03780713	0.99940337	0.985973
2268.16733	-1.28596525	0.99943806	0.98627
1529.77934	-6.25176846	1.00248907	0.986283
1298.55412	0.49243014	1.00246948	0.986469
2194.94365	2.98678026	0.99949215	0.986732
2170.05766	0.81679224	1.00244041	0.986745
2388.16704	0.57721517	0.99950371	0.986831
1401.67627	-0.76560758	0.99950729	0.986862
3725.95009	-1.08679713	1.00240919	0.987041
1243.60755	-0.5216436	1.00239708	0.987156
3039.48012	1.43279272	0.99954547	0.987188
2295.0853	11.1041475	0.9995561	0.987279
1367.64653	-0.13477623	0.99957943	0.987479
4059.80893	1.27685273	0.99959228	0.987589
4743.22036	0.55770548	0.99963865	0.987985
2356.0728	0.5501549	0.99966671	0.988225
1671.71868	-0.34023163	0.99967642	0.988308
1444.6762	1.11634683	0.99967904	0.988331
1659.7564	1.45982568	0.9996833	0.988367
3154.49077	-0.39564072	1.00223278	0.988715
1480.648	0.14285468	1.0022327	0.988716
1500.66887	0.91661856	0.99973666	0.988823
2327.16777	2.19017648	0.99975133	0.988949

Information Classification: General

3079.45913	-0.27639553	1.00220219	0.989005
1826.89861	-1.76152684	0.99976128	0.989034
2279.94048	-1.19784432	1.00217927	0.989223
3496.78144	-2.65499436	1.00213586	0.989635
2760.31583	2.25762238	1.0021264	0.989724
3097.32419	2.24437829	0.99984759	0.989772
2583.27024	0.28587372	0.99985615	0.989845
1805.91387	-2.06341063	1.00211207	0.98986
2479.08843	-0.85184335	0.99986424	0.989914
1771.8001	-3.02114958	0.99986513	0.989922
1914.86125	3.39520862	1.00209968	0.989978
3317.60923	0.67149164	0.99992352	0.990421
3188.52866	1.45877335	0.99992879	0.990466
1771.8118	0.38572408	1.0020434	0.990512
1257.66985	0.34118925	1.00201681	0.990765
3181.58779	5.65471136	1.00200594	0.990868
2533.1248	-0.20205948	1.00199199	0.991
2199.11112	-1.71440326	1.001991	0.99101
1146.54314	0.5260326	1.00198416	0.991074
2798.19951	2.97876879	1.00000466	0.991115
2378.04654	-0.16541249	1.00003424	0.991368
1493.71208	0.18640243	1.00003676	0.991389
3117.3779	0.06239368	1.00193764	0.991516
1572.63066	0.67246121	1.00006177	0.991603
2305.80132	2.1887532	1.00009472	0.991885
3367.4747	-3.564908	1.00189556	0.991916
3144.57754	1.95035877	1.00186485	0.992207
1749.85637	0.78780234	1.00014849	0.992345
2206.92698	1.93290053	1.00184327	0.992412
2000.95183	1.38310442	1.00016866	0.992517
2244.13547	-1.77089841	1.00018577	0.992663
1971.91399	2.89197626	1.00023192	0.993058
2427.23435	4.50546409	1.00023909	0.993119
1989.04557	2.32678375	1.00176481	0.993157
2164.02134	-1.02442526	1.00175542	0.993246
840.409716	-0.01158066	1.00027951	0.993465
4105.95261	-3.49065501	1.00029082	0.993561
1362.71389	0.39596972	1.00167841	0.993977
3046.32712	-0.41812493	1.00167597	0.994001
2641.23484	1.54022595	1.00034283	0.994006
1684.78032	-1.14392584	1.00036857	0.994226
1864.9451	2.76534237	1.00163966	0.994345
1434.6469	-1.84513368	1.00041479	0.994621
2547.10562	0.16790535	1.00155982	0.995104
1764.91081	-1.57180729	1.0015415	0.995278
1373.66496	1.69349223	1.00153727	0.995318
1671.78374	0.45502596	1.00150131	0.995659
1687.81487	0.36060512	1.00144351	0.996208
1690.73467	0.19011817	1.0006253	0.99642
1374.66924	0.31407692	1.00070167	0.997072
1687.74321	-2.80708506	1.00070539	0.997104
2095.14365	-0.32858129	1.00133161	0.997271
2891.36382	1.95126949	1.00074618	0.997452
2763.35721	-1.05104518	1.0013087	0.997489
2172.96587	0.43240958	1.00128683	0.997697

Information Classification: General

3372.50864	-2.80331102	1.00127576	0.997802
1893.99625	1.92802528	1.00125275	0.99802
2386.21318	-0.19819504	1.00082531	0.998129
3292.51096	12.1582421	1.00122051	0.998327
3133.51828	0.71464847	1.00089847	0.998754
3048.3964	0.12733155	1.00116186	0.998884
2174.93266	0.29402577	1.00097545	0.999411
1708.94719	-1.01650102	1.00098164	0.999464
1772.96172	-1.38569229	1.00108437	0.99962
1685.82549	1.57296938	1.00105843	0.999867
2044.04807	-0.19396789	1.001058	0.999871
1427.59429	1.53445557	1.00104615	0.999983
2072.9937	-2.64755146	1.00104439	1

greater than 50 indicates that the phosphorylation is mo
