# Supplementary material for: Salt-induced phosphoproteomic changes in the subfornical organ in rats with chronic kidney disease
Source: Ren Fail. 2023 Jan 30;45(1):2171886. doi: 10.1080/0886022X.2023.2171886 (PMC9888458; doi:10.1080/0886022X.2023.2171886)
Supplement: Supplemental Material [file IRNF_A_2171886_SM9865.zip › 2171886/Copy of Supplementary_Table_S2.pdf]

Supplementary T

P15205
P34926
Q63330
P68370
Q5XIF6
F1M9N9
A0A0G2K6R9
P06687
P13233
D3ZQL7
F1M1Y0
Q62745
F1LRZ7
P07936
P12839
P06686
A0A0U1RRX4
F1LQN3
P06685
M0R5J4
P63319
P63102
P60711
P63259
F1MAQ5
P11275
F1LS42
A0A0G2JSM7
D3ZZ99
P61265
P47942
P23565
A0A0G2JSU4
P07323
G3V8Q2
P34058
G3V874
F8WFS9
P69897
D4A1Q2
P61983
Q5QD51
G3V7C6
B4F7C2
Q5HZA7
F1LMY3
P85108
D3ZQG6
F1M3W5
P62260
G3V6S0
A0A0G2JUX5

A0A0G2K1Q9
D3Z9C7
P32851
P19527
A0A0G2K5Z4
G3V984
Q9JKS6
P42123
F1LMV8
Q05175
P83868
A0A0G2K611
A0A0G2JZK7
P35213
F1LMW7
P02688
R9PXR4
G3V9G3
A0A0G2K0F3
A0A096MK73
A0A0G2JW88
G3V9Y1
G3V6D3
P63039
P07335
Q4QRB4
A0A0G2JZ56
F1LNI8
P05065
A0A0G2K2M9
Q6RJR6
Q5U2P5
A0A0G2K2V4
F1M2P8
A0A0G2K207
G3V9B3
A0A0G2K7M1
A0A0G2JVS2
P60203
P68035
P35565
Q4G061
F1M779
Q5RKG9
A0A0G2JWM2
A0A0G2JX56
P16086
O08629
A0A096MJN4
P31596
P15999
B5DF41
F1LMC7
P82995
A0A0G2K0M8

Information Classification: General

A0A0G2JYF7
B0K014
Q75Q41
A0A0G2K9L2
M0R660
D4A5J1
O35095
Q02563
Q9QXY2
D3ZFD0
Q8K5B5
A0A0H2UHB7
D4AD03
A0A0G2K7X3
D3ZHV2
D3ZT07
Q7M0E3
B5DFK6
Q05962
Q3ZB99
P08050
D3ZEI4
A0A0G2QC53
P26431
Q5RJN5
G3V6P7
A0A0G2K1Q7
A0A0G2K162
A0A0G2K1L8
D3ZF26
P02401
P0C1X8
F1LRT9
A0JPM9
Q52KJ9
D3ZKQ4
Q6IN33
Q63433
D4A9D8
P68511
A0A0G2K613
B2RYX0
B1WBW4
Q91XU8
D3ZVQ0
A0A0G2JUG7
Q5U2N3
Q64548
P61765
A0A0G2K0X9
G3V9X2
M0RAP5
Q9JJ54
Q8VHK7
Q99MC0

D4A559
P35465
B2RYB3
D4A404
O35264
Q62847
P11980
M0R423
P63329
Q63475
P70566
P09456
F1M062
A0A0G2K1R9
Q3B8P7
D4ACZ5
Q5FVJ0
P0C5Y8
A0A0G2K4N6
D3ZRF9
A0A0G2K9S4
P12369
P68255
A0A0G2JWK2
P62161
D4ACG7
G3V7G0
A0A0G2JW28
A0A0G2JZB6
P09951
F1LRY7
Q62952
A0A173DW30
G3V733
P11730
A0A0G2K1U5
P21575
P19132
A0A0H2UHZ1
P04897
A0A0G2JXK1
A0A0G2K315
B1WC16
F1M2D4
A0A0G2K4F6
O35430
P19945
Q09073
D4ACM9
Q6IRG7
Q920Q0
D4A1J3
Q19LA7
Q64542
A0A0G2K1J5

F1M6C2
D3ZJG4
Q66HF1
P48500
Q68FR9
P47819
P15791
A0A0G2K4Q1
A0A0G2K6A9
P70580
P04631
A0A0G2KA27
Q3ZB98
F1LT10
F1M842
Q923V4
P09117
P18422
Q07266
Q4QR73
O35867
B2GV22
G3V8G4
D4A6H8
A0A0G2K527
Q62785
P10824
O08875
D3ZE17
P10980
B0BNA5
D4A4D8
Q5BJT9
Q62747
G3V7X2
M0RCB1
Q9ERH3
A0A0G2JSH4
F1M9C3
Q78PB6
F1LUY5
D3ZU84
F1MA36
A0A0G2KAV8
F1LQ63
D4ABT8
Q6JE36
E9PT53
D3ZTF0
A0A0G2JTT6
A0A0H2UHL9
Q5HZV9
G3V8P8
Q14TE9
Q566D6

A0A0G2JY03
A0A0G2K428
Q8K585
A0A0H2UHI2
F1LZX5
Q6MG08
A0A0G2JVK3
Q5U318
A0A0G2JZ50
Q5U300
F1M3F8
G3V8I4
F1MAA1
B5DFC8
F1M324
E9PST5
A0A0G2JTR4
O55173
B0BNF1
Q4V8F6
Q9Z1T4
Q56R18
A0A0A0MXV8
Q7TSU1
F1M4I4
P04906
Q9Z0W5
M0R5U4
Q08877
P31016
A0A0G2K939
A0A0H2UHQ3
Q9Z2L0
A0A0G2JZ27
D3ZMX6
D3Z955
P60881
Q62950
Q5BJN1
A0A096MJ99
F1M386
G3V849
Q9JJS4
Q8K3W5
A0A0G2K8P5
G3V8Q9
A0A0H2UHV4
P58405
M9MMM8
A0A0H2UHA0
Q9JKM5
Q91Y81
B2RYG6
Q68FQ0
F1M787

Q5XI34
A0A0G2K9J0
Q00A22
D4AE00
D3ZJK8
A0A0G2K774
B5DEN5
D3ZSU3
F1MA89
G3V8Q6
P11507
F1LSL6
F1M1T9
A0A0G2K272
A0A140TAA3
D4A1G8
F1M403
P61227
F1LMT8
F1LS36
P68182
A0A0G2JT93
D3ZUC9
M0RD03
D3ZR35
A0A0G2K677
G3V8R0
Q8CGU9
Q5U2U7
A0A0G2JVW5
F1LVK0
D4A0I5
P31000
P62944
Q80XF7
P63142
Q02294
P47875
F1LQZ9
F1LSE6
Q68FY0
D3ZWC6
F1LM09
A0A0G2KB46
Q9WU70
Q5HZE4
G3V7X4
P50398
F1M4A0
Q6AZ40
P62804
D4AAX6
B3DMA1
Q6P685
D3ZC55

Information Classification: General

F1M863
A0A0G2K0B6
A0A0G2JZM8
P61980
Q6J4I0
C0IXW5
A0A0G2JW92
F1M2K6
O88989
P04785
Q5RJK5
F1LUV9
B2GV94
D3ZJ01
D4ABI6
P38652
P06302
G3V7E6
G3V9U0
A0A096MIY2
P11506
A0A0G2KB60
G3V8D6
A0A0G2JTB5
D3ZC56
A0A0H2UHQ8
Q9Z0G8
Q6P6V0
Q5PQP4
Q5BJV5
P51111
A0A140TAB1
F1M8A4
F1M6X3
M0RC12
P53987
D3ZDU2
F1LRK1
Q810W7
Q5JC29
G3V8E2
F1LSH6
D3ZBC7
Q9Z1W6
G3V9G5
D3ZBU7
F1M8L1
G3V746
D3ZWQ0
B2DD29
G3V6L8
Q5M7V8
B0LT89
Q80X08
P28572

Information Classification: General

O35791
B2RYN6
Q5FWU3
Q05140
Q9JI66
Q08163
G3V8K2
Q2KP10
D3ZKX8
F1M7V6
M0R5P8
D4ABI7
P86252
P31044
Q6GMN2
P10111
A0A0G2K808
D4A4L4
F1LMT5
Q5XI32
P11505
A0A0G2K7W7
Q9Z2X5
A1L1M0
A0A0G2KAL9
A0A0G2JW97
Q66H15
P04642
P39069
A0A0G2K7H9
A0A0G2JVV5
Q4KLM7
Q63638
E9PTU4
D4A3D9
M3ZCQ2
B5DFI3
A0A0H2UHV6
A0A0G2KB92
P16617
P28818
G3V6N7
F1LU97
A2VD12
Q05695
D3ZSZ6
M0RDJ4
F1LSM0
F1LYQ8
A0A0G2K876
Q923I5
D3ZK97
P59215
D3ZBN0
D3ZT47

Information Classification: General

O88953
D3ZCG2
P13084
Q6MG76
Q5BKB9
F1LR33
Q8R511
F1M084
D3ZV52
F1M820
P12785
D4A5B3
D3ZZQ0
A0A0G2K4Q4
A0A0G2K007
M0RDJ7
P45592
M0RBL8
D4A133
A0A0G2JZF2
F1LM19
D3ZCL8
F1LPG3
P97680
Q07009
F1M9X4
A0A0G2JXR9
D4A8B3
P70587
F1LT49
Q4KLL7
D4AC23
B2RYD7
Q5RK09
A0A0G2K508
R9PXS9
Q66HA5
O88637
D3ZC84
F1M031
P15865
A0A140UHX6
Q99PA1
D3ZLW4
G3V741
P62815
P22909
Q80Z30
O88339
D3ZXD2
O70593
M0R686
Q63768
Q5PQL9
P11497

Information Classification: General

D3ZAL7
D3ZL30
A0A0G2K2V2
D3ZWS0
O35303
A0A096MIT7
M0R9R0
A0A0G2JYC7
A0JPL9
Q63014
P63012
Q5EB70
Q641X2
Q5XIU9
B1H266
P31662
F1M4A4
Q5U1X7
D3ZQJ3
A0A0G2K4U2
Q0QF43
D4A4M0
O08813
A0A0G2JTW5
O35314
P47860
Q6IMX7
D4A648
Q3MHS9
Q03348
A1A5P0
D3ZBT9
D3ZAZ1
Q99M64
F1LMD9
Q9JJ19
A0A0G2K0J4
Q6P6U2
Q9QZ86
Q3T1K5
F1LX20
P11348
Q63345
D4A831
B1H264
D3ZIM7
A0A0G2K6U1
B0VXR4
Q63327
A0A0G2K654
Q499R8
O08729
Q9JHW0
F1MAA7
Q5BJT1

Information Classification: General

A0A0G2K5E7
P47196
Q99P55
D4A510
D3ZC31
Q63569
F1MAQ4
B4F786
D3ZPI4
Q00981
D3ZU56
A0A0G2KAD8
A0A0G2JZX5
A0A0G2JU89
F1LXQ7
B0BMZ5
A0A0G2K1P1
D3ZAU7
Q5U2Z3
D3ZBW5
P62747
A0A0A0MY13
D4AAB3
A0A0G2K6Q5
E9PTN6
M0RD40
D3ZN60
Q9ESH1
B2GV74
G3V6X7
D3ZNS1
F1M836
F1M7Y3
Q66H79
D4A8L4
A0A0G2K700
D4ABB2
D3ZNK1
P97834
A0A0G2K7R1
F1LP57
O35346
A0A0G2JXZ3
O35986
O08679
A0A0G2JTD8
Q641Y8
P50554
A0A0G2K2U5
P10860
D4AB17
D3ZF86
D4A4D5
A0A0G2JSH6
P70600

E9PSJ4
F1LQD2
Q9EPS1
D4A1D3
A0A0G2K9C8
P0CG51
F1LYA6
P10499
Q63624
D4A644
F1M8V2
Q63881
D3ZA31
D3ZBP2
B4F772
Q3T1J1
Q4QR85
D3ZKH2
P97846
D4A3C2
A0A0G2JSR0
D3ZCI2
M0RBB2
B1WBZ7
A0A0G2K1Z2
Q7TNK0
D4A0U0
Q4V8I7
A0A0G2K4J8
F1M5N7
F1M9V7
Q63622
Q66H20
Q6AXU6
A0A0G2K7H2
A0A0G2JZX3
Q62733
B5DFL0
F1LQ45
A0A0G2K904
A0A0G2K382
P05708
G3V927
A0A0G2JWR2
D3ZH78
F1M6V8
D3ZA84
D3ZX42
G3V8L3
P37805
F1LVS1
D3ZYS7
D3ZTB5
O54924
O35147

Information Classification: General

D3ZDR2
D3ZYB4
Q5I0K2
P50137
P85971
A0A1B0GWY5
Q56B11
Q9WTW8
G3V7I8
D4AAR7
B4F763
G3V6I1
P29066
A0A0G2K911
O88831
Q5FVI4
Q2V6G6
P37377
B5DF74
F1LW91
P97632
D4A0E2
B1VKB4
P47198
G3V7Q4
D3Z9V8
P61751
D4A554
Q3SWU3
Q9WVP7
A0A0G2K451
G3V6L7
Q5FVJ4
A0A0G2JYD4
D4A9D6
M0RCT5
A0A0G2K3C1
G3V8M8
A0A0G2K712
Q4QRB9
A0A0G2K9A9
P63170
O88420
A0A096P6M3
P63036
A0A0G2JTL7
Q9EST6
F1LN57
A0A0G2JV49
A0A0F7R5I4
P97577
D4A269
G3V6K8
Q3B7U1
A0A140TAJ5

Information Classification: General

Q5U206
F1LRF3
Q6P9X1
A0A0G2JY69
Q0D2L6
G3V6P8
Q66H43
P21818
Q6PCT3
P49621
D4A3S8
G3V9R8
G3V9G7
F1M4I1
Q3S4A5
A0A0G2K988
D4ACV3
A0A0G2K3G9
A0A0G2JU96
O88767
A0A0G2K9F7
D3ZJ32
Q4V7C7
Q66HC5
P25113
Q6PEC4
Q9JJP0
B5DFN0
Q99ND8
D2XV59
P17955
D4A0C3
Q6AYE6
P52481
F1LWK7
A0A0G2JWP1
A0A0G2K1B4
D3ZG78
P80386
D3ZU74
D4A031
G3V7L1
D4A1H8
A0A0G2K5T1
A0A0G2K4I4
A0A096MKC0
D3ZRN3
F1M241
G3V9T7
D4A0X3
D4A4K4
Q63092
A0A0G2K8L7
F1LNP8
A0A0G2K104

A0A0G2K0B4
Q6AXS5
G3V6Z3
A0A0G2JTD1
G3V9J8
Q793F9
Q99NA5
A0A0G2K931
F1LR18
Q9QWR2
A0A0G2K7K9
D3ZEX7
D4A6C5
A0A0G2JW01
A0A0G2K7C1
G3V917
F1LWN1
Q5GFD9
Q641Y2
A0A0G2K266
P21707
Q9QZM5
A0A0G2K4C6
M0RDG0
D3ZH75
D3ZWA1
D3ZPN3
Q62780
M0R4V3
Q5M7A4
A0A140TA95
P62762
Q5EB62
F1LRZ1
P09606
D4A927
A6Y7S3
P14668
D4A4Z0
G3V7T8
Q7TQ88
Q6TUE6
O09032
Q5PQL7
D4A054
A0A0G2JYB3
Q6AY24
M0R4A0
I6L9H8
F1LXC7
F1LMH0
Q9JKB7
Q63785
Q4KM36
D4ABK1

B5DEI5
Q4KM74
F1M1U0
Q9WVR6
D3ZVR9
M0R7B4
M0RBZ7
Q6P791
Q6P503
Q7TP42
Q2VC85
F1M0Z1
M0RAQ6
Q6P4Z9
D4A781
D4ACN3
P97618
D3ZYT8
Q99MZ4
X4YHC6
Q0ZCA7
Q8R497
A0A0G2K7P7
M0RDR2
A0A0G2JWK6
P17220
D3ZVU6
Q5BJR4
Q7TP36
Q4QQW4
D4A7V1
F1M0J7
E2RUH2
D4AAU6
A0A1B0GWN8
M0R4J7
P54311
Q63635
D3ZFB6
P40241
Q6PST4
A0A0G2JX77
D3ZEY4
D4A0A1
D4A2D3
Q9Z147
Q5PPM8
D4A3G2
F1LPS3
O54861
D4A4W6
A0A0G2JYI0
O35244
Q5XIM9
A0A096MKG6

F1M9G6
D3ZSZ4
O35077
Q8R491
A0A0G2JSQ4
D4AAZ8
B1H257
B1WC06
M0R3N4
D3ZMR2
Q9EQS0
F1LZS5
O88794
A0A0G2K0E5
F1M994
Q4V8E1
D3ZHG4
Q62882
Q794F9
Q62634
D4A7H9
Q4FZT2
B5DFF7
Q3KRC3
Q568Z1
P25093
F1LSC3
D3ZWL6
G3V6Y9
M0R9T2
Q68FX3
Q6P686
D3ZSY8
Q91ZY8
P34064
B2GUV0
D3ZN39
D3ZLQ8
P19234
O88377
P35435
F7FKI5
Q4KLL0
D3ZGU6
A0A0H2UI14
F1LNF0
D3ZY45
Q3B7K9
D4ADS9
P67779
F2Z3T8
B2RYA6
Q6AY84
D3ZHK4
Q64303

Information Classification: General

Q9WVR8
D3ZGN7
P70627
Q5M7T6
H1UBM8
F1LTJ8
D3ZW49
D4A9F1
P13221
D3ZNF5
D3ZWE0
F1M4U0
Q4V7E8
P54313
P0CD96
P10683
A0A0G2JXT9
D4ADF5
P63095
Q56A26
A0A0G2K7H0
Q5BJS4
D4AAT7
P14942
G3V6M0
E9PU01
G3V6M3
D3ZKP1
F1LQY6
D3ZRI1
D3ZHG8
P80254
P30349
A0A0G2JYN0
D4A997
D4A626
B1WC33
Q6AXY7
D3ZJR6
D3ZYR0
D3ZKG7
P97690
Q9ER34
P11030
A0A0G2K4P6
F1M4R7
P85972
P50408
A0A0G2K5K8
Q9R1N0
B1WBV4
F1LVX3
Q07205
A0A0G2K1W1
A0A0G2K7R8

G3V7J2
D4AEK9
Q6AY30
Q64428
F1LPH1
Q5RKH1
Q5U4E4
D3ZH14
F1M0U5
D3Z9D0
A0A0G2JWL3
P27139
A0A0G2K6Z8
P54921
D4ABN3
D3ZAY8
D3ZL45
Q5U2Y6
Q4QQV6
F1LQJ7
A0A096MJZ0
P85845
Q5M831
Q8K1Q0
Q76KC6
D4ACD9
M0RCJ9
D4A8V2
D3ZHB7
F1LWT1
D3ZJ08
A0A0G2JSR7
Q0PMD2
A0A0G2K5U6
B2GUZ9
A0A0G2JZF5
Q5EB89
Q4QQV8
A0A0G2K219
P02650
D3ZYS1
Q1WIM3
D3ZC89
Q8CFD0
M0R5H1
Q5RK33
P00507
F8WFH8
A0A096MKD9
G3V6C3
A0A0G2K0S0
F1LSM8
A0A0G2K4S1
A0A0G2JSV3
M0RBY8

B5DEY3
D4A3P4
F1LSD1
Q6AYG5
D3Z899
A0A0G2JTD7
Q6LDS4
Q6AYB6
B1WC49
F1LSG0
Q9WV48
Q63555
F2Z3T9
P20417
G3V7U4
D4A914
G3V7X5
Q3KRE3
D3Z941
F1LPM3
Q3ZAU5
D4A1D2
P48037
Q9JKC9
Q68HB8
Q923K6
D3ZZZ9
Q52KS1
D3ZHX3
A0A0G2K943
P60841
D3ZD09
A0A0G2KAI1
P22734
A0A0G2K3N1
A0A140TAA1
A2RRU1
F1LLX6
F1LPA3
D3Z8X7
Q5XIQ6
A0A140TAA4
Q62829
A0A0G2K2G8
P81155
Q5U3Y8
D3ZWH2
A1L1I3
Q5FVC7
A0A0G2JWC6
Q6MG48
Q5I0H3
Q66HG9
A0A0G2JVT2
B1WBS4

A0A0G2JSI1
A0A0G2JSH5
D3ZF21
D3ZWX2
F1LTW9
Q63198
F1LR76
G3V793
Q5XI97
A0A0G2K689
Q9EPC6
Q62658
G3V8Y7
Q923W4
F1LUG5
F1LQS9
D4A4F9
D3ZFC3
B4F774
A8IHN8
Q8R5H2
P63047
A0A0G2K875
D4ACB8
O88954
F1LW22
F1M9D6
D3ZWJ9
P61808
P0C6T3
A0A096MK93
D4A4T9
D3ZH40
B2RYN0
Q5XIW8
B2RYF8
A0A0G2JZ88
D4A5F4
A0A0G2K6Y9
G3V790
M0R3V7
Q642A2
Q8VIJ5
Q5XHX2
P24049
D3ZMS5
G3V8J5
M0R5P7
Q7TP95
A1L1K8
Q60587
A0A0G2K847
A0A1B0GWS4
P46844
Q32PZ5

Information Classification: General

D3ZY20
B2RZD5
F1LU68
Q3SWT4
Q2IBD4
F1LRS2
P41542
D3ZBB7
Q5XI21
F1M392
F1M8G8
Q8VHJ9
F1M5M9
D3ZXD8
O88397
D3ZYR4
G3V9N7
A0A0U1RS13
G3V700
A0A0G2K9Q6
B5DF62
Q63424
Q9WTT7
F1M110
F1LM10
Q04931
P43278
D4A3C4
Q63396
O08623
D3ZYD7
O35355
Q63941
D4A7I6
Q63610
D4A280
A0A0G2KB58
Q5FVL9
O35567
B0BNB2
Q4V8H5
Q9ESB5
Q4G027
A0A0G2JSZ0
Q9JHZ4
D3ZWV8
F1LQ41
D3Z8C7
D4ADP2
Q5FVT1
D4A3N4
D4A507
F1LSG8
Q6P136
Q78P75

Information Classification: General

D3ZXB1
M0R766
G3V9C5
Q9JKL8
Q5PQP2
A0JPI6
F1LS65
F7EPH4
B2GUZ5
A0A0G2JXS2
A0A0H4SRI7
B2RYB2
M0R961
D4A318
B2GV23
M0R851
Q6P7P5
Q6AY02
Q62760
Q6TQE1
F1LPA2
D3Z9L5
B1H262
A0A0G2JTA7
A0A0H2UHW6
D4ACJ7
F1MAA5
G3V8A5
M0R781
D4A0Y2
A0A0G2JTX2
Q6IE24
D3ZQR3
Q5XI73
D3ZIV8
P97924
A0A0H2UHW4
Q63002
Q66HK1
D4A4L2
Q6AYN4
P19836
Q7TQ19
G3V9E4
F1LX07
P06214
D3ZE26
A0A0G2K9B2
F1LNE4
P01830
A9CMA6
F1M4Q5
F1M8G9
A0A0G2JXZ7
Q3SWT7

P36972
Q66HR5
D3Z8L2
Q5M970
F1LNC4
P0C6P5
Q3KRD5
P30835
Q64350
P55280
Q921A2
P84087
A0A096MK75
A0A0G2JWF4
F1LSB5
A0A0G2K4S6
P59649
F1LS02
D3ZVF4
F1MAQ8
Q6AYH3
A0A0G2K7S6
D3ZUM5
F1M4V3
D3ZCV0
A0A0G2JYC6
D3ZGL5
Q8CJ04
E9PSN4
A0A0G2K779
Q5XI38
Q810G8
K4DIC3
B2GUV4
Q10758
P11345
D3Z8X6
A0A0G2JSW5
Q68FW9
F1M2N4
A0A0G2K6F5
D3ZLC1
D3ZG21
A0A0G2K472
D3ZC82
Q6PCU9
A0A0G2JTI7
Q63564
G3V7V7
Q5PPH0
Q4KM62
A0A097PE04
P62957
M0RDK4
A0A0G2K5Z1

Information Classification: General

D3ZL11
Q5BJT7
A0A0G2KA57
P0C627
Q9JHL4
A0A0G2KA12
B2RZ24
B0BMW2
F1MAS1
F1LRV4
D3ZG37
A0A0U1RRV1
A0A0G2JSQ1
Q8BFS3
D3ZXX7
B5DF40
G3V647
F1MAF8
Q63083
D4AB66
D3ZHI9
Q4V8H8
D3ZGD0
D4A758
Q5BJP9
Q6MFY6
D4A739
Q6AYK3
Q6MG51
D4ACS0
F1M3D2
Q4FZU8
Q6P6R2
A0A0G2JV78
Q5M9I5
P97536
G3V864
D4A853
D4ADT3
G3V7L8
D3ZQM0
A0A0G2JT63
P63159
F1LVR4
A0A0G2K1T0
D4A3X6
Q9ET45
D3ZZ32
P62483
Q70AM4
O54800
G3V715
B8XCX0
M0R7E6
D4A7Y9

D3ZWV2
P12346
A0A0G2K8B5
Q06647
B1WC25
D3ZHW0
D3ZWT6
A0A0G2JTG1
Q4KLH4
Q9QZX1
M0RD53
A0A0G2JSR2
Q63714
Q924N5
Q2HWF0
Q63081
Q8CGQ3
A0A0G2JU91
Q6AYH5
A1A5N6
F1M7K7
A0A0G2K5B0
Q9JK00
D3ZW56
Q99JD5
M0RB65
F1LNZ5
G3V6E4
O35179
A0A0G2K401
P97612
D3ZFC5
B5DEK0
B5DEJ8
D3ZJ86
P25122
F1M8Y4
D3ZCQ9
Q99PD6
A0A0G2K1S0
Q68FS9
D4AAL4
D4A205
O54857
A0A1B0GWQ5
D4ADU2
A0A0G2QC41
D3ZSX5
Q9QYN5
P20650
Q9WU49
F1LXV3
D3ZWU1
D4A3V4
P15650

Q4KM37
M0R7T1
D4ADX8
D4AEP0
B0BMX3
F1LMM2
D3ZKC9
Q5BJT2
F1LWX5
Q3T1K9
P40112
A3FM27
Q642A5
G3V803
F1M9F9
F1LYG2
P63043
O35052
D4AAI7
B2GV14
O70187
O55164
A0A0G2JUT1
Q63340
Q499R6
G3V8R7
A0A0A0MXY4
Q499N6
M0R5N4
O70436
F1M8I7
F1M8D5
P50399
D3ZFQ5
P32551
F7EMB2
Q62901
Q63358
F1M7S0
D4A1U1
F1M3L7
F1M4M5
E9PTK9
F1LVZ8
Q3T1I9
D4A3K7
D3ZD80
O55170
D4A3H5
Q920J4
F1M471
E9PTE1
Q75T81
F1LPM7
Q6AY17

Information Classification: General

Q5RK25
Q8CGU4
A0A0G2JYA4
A0A140TAB3
Q4G008
Q99MD2
P0C5X8
E9PTG1
D4AAS1
D3ZB48
A0A0G2K5M6
A0A0G2KB70
D3ZL75
A0A0G2K7T5
Q6LDZ3
Q66H98
D3ZH92
D3ZHE6
A0A0G2K2D5
D4AD05
Q9Z158
Q5U2U2
Q5FVF3
P06761
Q8R5M1
P62997
A1L1L5
P70501
D3ZYJ5
A0A0G2JXN8
F1LQC5
Q8K4Y6
A0A0G2K2E8
P13383
D3ZWX4
F1LM55
F1M9C9
B5DFF4
D4A3T0
G3V6S8
A0A0G2K101
D3ZXY2
Q9Z2X9
A0A0G2JWJ3
Q8R4T5
B2RYM6
A0A0G2JV35
D3ZUY8
P49620
D3ZP47
Q5XII5
Q6AYB2
A0A0G2K2T6
Q6P7C9
D3ZGN2

Information Classification: General

A0A140TAB4
F7IXA3
G3V8V0
D4ACN6
A0A0G2JXT6
O35458
M0R3U3
M0RBD9
M0RC54
D4A7B6
M0R920
D4A352
P07153
Q924W2
F7FLB2
Q6DG50
D4A929
Q4R1A4
D4ABZ4
D3ZHL1
D3Z9G0
A0A0G2K1I6
G3V8J0
Q5FVK6
A0A0G2JWD6
A0A0G2JVD6
A0A0H2UHE3
A0A0G2K652
F1MAL5
A6JUQ6
M0RAP6
D3ZBX9
P53534
G3V8P6
F1LMW3
D3ZJH2
D4A060
Q9JID1
Q5RJN0
F1LP59
B5DFB1
D4AA13
Q4FZU3
F1LM73
Q63472
D4A8C8
B4F7C1
P62963
Q541U0
F5HTD6
D3Z9C0
G3V8L9
F1LPJ6
A0A0G2K6J2
Q5XIT1

D3ZS72
M0R5E3
Q6QRP0
A0A0G2K3Q5
Q9QYL8
A0A0G2K8Q4
P07171
A0A0G2K590
D4A517
F1M0N1
A0A0G2JW51
A0A0G2K2L0
P62494
Q5PPP1
B1WC00
P81799
B0K020
Q01066
G3V9M3
D3ZDV9
B2RYF6
Q76MT4
Q8R5H8
P82808
O54698
D3ZV33
D3ZBT4
D4AC95
D3ZZX1
D3ZXL9
D4A9R4
A0A0G2JZG4
D3ZU55
A0A0H2UHH3
F1LQT3
Q9JM15
G3V8V4
G3V6N0
Q9R1X8
Q569C0
B2RYU7
Q3KR59
A4L691
Q1LZ53
B5DFE0
P19468
Q5EB71
D4A1V8
F1LS67
C0JPT7
A0A0G2K5K1
F1M8H6
D4A629
D4A1H7
D3ZT71

F1M801
D4A702
Q3MHS2
Q7TNM3
A0A0H2UHR5
Q7TQ77
Q62991
F1LN42
D3ZZK3
G3V8T1
Q1RP74
A0A0G2K4R1
B0BNL5
A0A0G2JZB4
D5MTG9
D3ZAF7
A0A0G2K0D3
A0A0G2K1U1
A0A0G2K4W2
F7EYF1
F1LPP8
F1LMV9
A0A0G2JT26
D3ZX21
Q6AZ61
D3ZVN5
D3ZTA8
D4A9G6
Q498N3
D3ZPJ0
D4AD99
F2Z3T7
P02625
P07340
A0A0G2JSV2
B8K2Q4
F1LWM1
Q4V7A6
A0A096MJH0
Q5RJR2
Q9JK73
F1LNU2
F1M949
D3Z994
B1H240
G3V6N1
B2GVB4
A0A0A0MY48
P23978
Q5XIR9
A0A0G2K3Z9
D3ZC15
B2RYB4
P0C5E3
D3ZQM2

A0A0G2JT23
Q4G017
E9PTG5
E2E1S0
D3ZY86
Q68FQ9
A0A0G2JWT3
Q9EPF2
A0A0G2K326
M0R6L4
O35796
Q7M0H6
B5DF63
E9PU32
Q5XXR3
A0A0G2JVM6
P41499
D3ZD52
F1M3G7
Q5XIB5
P05197
D3ZNQ6
P81377
D3ZMR7
B4F7A3
Q63713
A0A0U1RS39
Q496Z5
M0R6K4
A0A0G2K5A4
D3ZUQ2
Q63128
F1MAA3
Q63468
D3ZN37
Q6YDN7
D3ZKR9
M0R8A4
P50475
Q4KMA2
F7IXA1
P47728
Q5U317
Q811A7
G3V983
D4AAU4
Q5BJW6
D3ZY47
D3ZH36
D4A8G7
Q4V7D1
D3ZF03
Q52KK2
Q3KRC5
A0A0G2K8K1

Q4G036
Q62861
D4A1Z8
Q8CGS4
A9UK05
M0R963
F1M4W7
D3ZBL6
A0A0G2K9A2
P09217
M0R8V0
Q5XIC4
D3ZDI9
F1LT30
Q99JE4
P16970
D4A7M0
Q9JJZ1
A0A0G2K132
A0A0G2JW85
Q7TP93
D4A830
A0A0G2K4M7
P62516
A0A0G2K2R0
P22462
D4A0B4
B1WBQ5
D4A224
A0A0G2K9M4
Q80ZB2
Q66HP6
D3ZH41
Q3MID9
D4A3V5
D4A2C4
F1LVA9
D3Z9R2
P85515
E9PSV8
A0A096MJ75
Q00969
G3V656
Q5RJK8
O35141
Q6AY57
A0A0G2JZ82
F1M853
F8QYX1
B2RZ73
D4A105
D4A3J9
D3ZA89
B1WBT4
G3V6H5

Information Classification: General

P31647
Q6AXS3
O70150
Q05683
D3Z9E6
D4AAV2
Q4G045
Q6AYT4
M0RA26
D4A193
F1LNC7
Q5U1W8
A2VCX1
D4ABK7
Q5XII9
Q62806
P14925
F1LVX2
Q5BJN2
P62329
D4AD44
A0A0G2K8F9
O70521
D3ZFK6
Q5PQR0
D3ZMW5
D4A206
D3ZN16
Q9Z2J4
A0A0G2K3Z3
A0A0G2K2G7
G3V8F1
Q5XI44
D4A5L7
Q331S7
Q6PDU1
Q156J1
Q8VHX0
D4A720
F1LPB9
D3ZS76
A0A096MK35
A0A0G2JUK2
A7M775
Q09QM4
Q9JJ76
F1M2J2
Q3B7U0
Q6P6G5
Q9EQZ1
P97689
A0A0G2JUL7
D3ZMS1
A0A0G2JT21
A2RRU4

P0CE43
A0A0G2QC38
Q8K3P4
Q5BJX5
A0A0G2K9N0
D3ZFH5
A0A140TAD1
B4F7C7
Q6REY9
P70567
Q63190
D3ZK36
Q07936
Q5FVI6
Q66HR2
P20070
D3ZYW8
G3V7U9
A0A096MJM8
Q5PQT2
F7FFV2
F1LP21
Q792I0
D4A017
P18418
D4A3K5
D3ZAR2
D3ZGI9
B5DEJ5
D4A6H3
D3ZWB4
F1MAJ0
O35050
F1LQS1
P48303
F1LSD3
B5D5N9
Q27W01
D3ZQT0
F1M037
D4AA54
P67874
Q712J2
Q71RJ2
D3ZES2
D3ZIL9
P46462
F1LS72
A0A0G2KAJ5
D4A148
A0A096MJL8
B2RZ79
A0A0G2K5S2
A0A0G2K9M8
D4ADD3

Information Classification: General

D4A769
F1LNB2
Q4G016
P50393
P37996
Q4V7D4
A0A0H2UHF5
Q2THW7
D4A732
M0RB22
P0CC10
Q1EG89
M0R617
Q63259
A0A0G2JXM0
Q6DGF9
D4A3V6
B5DFG2
Q8R2E7
Z4YNN0
M0R8J3
Q9Z1X1
Q5U2S7
Q6P730
Q5XID7
Q4FZT9
A0A0G2K2E3
M0R3R6
Q99PF3
Q3ZAV8
Q499Q2
D3Z9J7
F1M9V3
Q6AY81
O08589
F1M3A4
A0A0G2K070
A0A1B0GWY6
O35832
D4A8L3
P31421
B1WC26
D3ZJ92
D3ZNI3
P97544
D4A6A8
D3Z9Z9
D4A3E1
Q63135
P97887
F1LTZ6
A0A0G2K0A8
D3ZKL3
Q505J5
A0A0G2JVK8

G3V913
Q5M860
B0BN02
P19814
D3ZB78
Q3MUI2
D4A6X3
P47197
B2GUX8
A9LRT4
A0A0G2K928
A0A0G2JW68
F1LNC3
Q3KR56
Q5XIK5
D3ZBB3
B2RYS2
Q561R9
D3ZEV0
F1M1D5
A1L1L6
Q5M7A3
D3ZGL1
D4AE69
D3ZSP7
P14604
D3ZUJ7
D4A1R8
B2RYN2
P19804
D3ZWM3
O70188
M0R4R4
A0A0A0MXW9
A0A0G2K8K9
Q6AYG3
Q68FR6
D3ZLS5
D3ZN76
Q4V8H7
Q3KRF2
P63086
F1MAH6
Q505J8
D3ZZM9
F1LQ62
F1MA98
D4ACC4
B0BNM9
A0A0G2K3F4
Q4KM87
A0A0G2JZE7
D3ZLC3
M0R809
D3ZG83

B2RYK0
D3ZYR1
A0A0G2K964
D4A6A2
Q68FQ2
D3Z9K4
Q9JM59
B5DFG5
D3ZDC2
F1M269
D3ZW27
A0A0U1RRV7
D3ZVM5
A0A0G2K0J7
F1M7L6
Q6NYB7
D3ZZH5
D4A2A3
Q6MG88
A0A0G2JY17
Q4V8K2
D4ACS3
D4A4Z9
E9PTI1
Q2KN99
A0A0G2K7X0
Q4V888
Q5U316
Q4KM57
A0A0G2K366
Q99MS0
F1M0A6
D3ZZ81
M0R3J7
A0A0G2JT30
D3ZW40
Q6AY65
Q6P502
D4ACV0
A0A0G2K6U6
D3ZU88
A0A0G2JT88
B1WC02
F7FLS6
A0JPQ3
F1MAD2
Q5BJP4
D4ABM3
Q9JKA7
F1M9G3
F1M953
F1M362
D3ZDP7
Q9QY02
D3ZWG2

D3ZN95
A0A0G2K6T6
A0A0G2JU75
P0C548
D3ZGK0
B5DF49
Q5BJN8
D3ZT16
P70531
D3ZAB6
P54645
A0A0G2KA88
D4A9T0
Q8VHW9
Q5U2M8
Q5I0L4
B5DFM1
Q4G086
F1M8S4
B0BMV7
G3V7P8
A0A0G2K3B8
A0A0G2JU07
Q62833
D3ZIT1
I6L9G6
Q32PX7
Q99N97
D4A8M2
D3ZPT0
G3V7Y3
Q4V896
A0A0G2K9R0
D3ZLG1
B4F7F3
A0A0G2K865
B3DMA0
A0A096MIY6
D3ZT56
Q9R1Q2
P47861
A0A0H2UHR7
P0C644
D3ZK16
F1LS01
F1LNT3
A0A0G2K9T8
P62775
P70673
A0A0G2JUQ3
A0JPP6
D3ZYQ8
D3ZSX2
Q9Z1Z1
A0A0G2JZ38

Q5PQS2
G3V8H8
A0A0G2KB48
F1LP76
D3ZDS0
Q5M9F1
Q63211
F1LSQ0
Q76EQ0
A0A0G2K2U3
Q5BJQ2
A0A0G2JZY0
Q6AXW2
D4A168
D3ZGZ9
D3ZUT9
Q5EB90
D3ZT79
A0A0H2UHH8
Q3B7D0
A0A0H2UHC0
D3ZJK3
G3V9D1
M0R8P6
Q99PJ6
Q66HA8
F1LPT0
A0A0G2K0P0
G3V857
B1H238
A0A0G2JW20
A0A0G2K5C0
D3ZAI6
Q4V893
F1M287
A0A0G2KB96
G3V936
Q9JJW1
B2RYP3
Q9ES26
F1M816
D4A8Y5
A0A0G2K0E2
G3V764
D4A198
D3ZVB7
Q6P6S0
D4A4J1
Q6TXF1
D3ZYQ9
A0A0G2JU83
F1MAK1
F1M6U2
D4A9L2
D3Z9R8

Information Classification: General

A0A0G2K8B7
B0BN72
Q569C9
P68101
Q06486
B2RZD6
B2RYW7
Q08013
D4A1D8
Q5XIA2
A0A0H2UHP9
D3Z8B9
P97531
Q9Z136
Q99JE6
Q3B8N7
Q68FY1
O35814
Q9EPY0
O08557
A1L108
Q5RKL5
Q9WUD9
P52909
Q5BJK8
Q9JM01
P07895
Q5XI06
P22288
Q4V7D3
Q5XIP1
Q561R7
O35824
P82458
P20272
Q62768
Q64535
Q4V8C2
Q6AYP2
P97710
O54701
O54715
D4A631
P20720
D3ZQL6
Q5FVR2
Q4V7D2
Q498D5
P54287
P10888
B1H267
O88764
Q5FVM4
Q03351
P19643

Q5I0D1
O54922
P23928
Q8VIP2
P54258
Q63410
P33568
Q66H86
Q9Z1H9
Q6AYC4
Q63089
Q63553
Q5PQJ7
Q5U2Y1
P0C6C0
Q63068
D3ZW55
P0C0R5
Q642G4
Q5BK20
Q5XI74
Q5XIG4
Q5BK32
P52296
P84817
Q62866
P55053
D3ZE55
Q566R3
P57769
D4A8G3
P80349
Q2VUH7
Q5M821
O88917
P50573
Q6AXP1
Q9WVI4
Q9JIH7
O35431
P30680
Q9P290
Q64595
Q63430
Q6MGB4
P19103
P0C5E9
B0BN86
Q62962
P63182
P0C6R4
Q5I0E3
Q5FVJ3
B0BNA9
Q5XIB9

Q9EQL9
Q9R0Z7
P47987
B1WBR8
D3ZCC3
Q5U3Z5
D4AEI6
B5DEJ1
F1M9W9
G3V7J3
Q6AZ64
A0JPJ0
D3Z9Z0
G3V887
D3ZIM5
B1H2A2
D3ZSC1
D3ZWQ5
D3ZT64
D4A919
D3ZN06
D3ZFJ6
B1H241
D4A0G0
A0A0G2K2E7
A0A096MJJ6
B5DEX3
D3ZUL4
B1H2A6
G3V6J5
D4ADE5
F1M8H5
G3V9N1
M0R6L8
G3V7H1
G3V9L1
B1WBV1
Q6AY21
G3V8F3
D3ZXL5
A0JN30
D3ZVF2
G3V799
D3ZTF6
D3ZMG0
F1M6I7
Q5D006
G3C8Z1
Q3MHS0
D3Z980
B1WBX7
B2RZ23
D3ZGJ3
G3V8Q8
B0BND5

Q5BJZ3
B2RZ74
Q5U205
Q80Z26
G3V7K0
G3V6R7
Q5PPJ6
Q6AXV0
D4A2G4
G3V7V0
D4A7C3
A0A0G2K6L1
D4A7D3
Q6MFX8
Q5XFX7
D3ZV88
Q99PJ8
G3V8W8
G3V727
G3V9W0
D3ZUB7
A0A096MJA0
M0RDF7
Q2TGJ5
B5DEG7
B0BN77
Q5J3J1
F1LV52
B1H246
F1LST1
Q8CH83
Q6IRK1
F1LR04
D3ZQ55
D3ZJU5
A0A0G2K7K2
D4A1H2
D4A3F5
Q5RJK6
G3V7H4
D4AEJ5
B2RZD1
D4A346
D3ZEI6
D4A1C0
F1M0G0
D4ACW0
B5DF65
M0R3Z8
D3ZNS8
B5DFL9
D4A4V3
F7FD99
D3ZVT3
Q5I0Q2

A0A0G2K2Y5
D3Z8C4
G3V7P7
B2RYU0
B0BN10
M0R5I3
D3ZCZ3
D4ACF1
F1LR10
E9PTB2
D3ZSF3
D3Z8L5
D4A4P8
F1M9N4
A0A097PIG6
A0A0G2K3H4
M0R970
F1M9Y7
Q66HA0
D4A1Q9
A0A0G2K0J0
A0A0G2JXD9
D4ABH1
D4ABS5
D3Z9I3
Q5D022
F1LW90
C5NTX8
F1MAS4
D4A9W8
M0R7G4
D3ZR47
A0A096MJ77
A7BJV7
F1M7H7
F1LPZ6
F1LQV8
D3ZHZ3
Q9WUY1
M0R965
M0R6D9
F1M5X7
F1MAM6
F1LZS9
D4A748
A0A0G2K400
M0RAT6
A0A0G2K0M5
D3ZDX5
F1LQ24
D4A3E3
D3ZF45
F1LXF1
F1M6A8
D3ZFG7

Information Classification: General

A0A0G2K809
D4A959
Q3B8P5
D3ZZZ0
A0A0G2K2J8
F1LQC8
A0A0G2JTA4
F1LMV6
E9PT44
D3Z8W1
F1LQR8
M0RBT5
A0A0H2UHT1
Q68FQ3
A0A0G2JW59
D3ZSV8
A0A0G2K3U1
A0A0G2KAH9
B0BMV0
G3V676
D3ZMM2
D3ZLR4
Q5UDQ9
A0A0G2K4U8
F1M4N6
F1M7S2
D4ABP3
M0RB44
F1MAB7
F1M7U7
A0JN16
M0R7A6
D4AC16
D4A1S2
F1LX86
A0A0G2K5C7
D4A306
F1LQJ2
D3ZTF1
D3ZE49
F1LPE9
F1M575
F1M9N5
D4AC36
Q4V8F0
A0A0G2JYP3
M0RD44
M0R991
Q8CIV0
Q9ET34
F1LPP6
A0A0G2JZ77
Q9WUX0
A0A0G2K5X0
D3Z881

Information Classification: General

D3ZH28
F1M9C8
Q6IE71
Q6IE65
A0A0G2K8K0
R9PXV8
D4A317
F1LPQ9
D3ZJF8
A0A0G2JTS9
F1LPG5
A0A0G2K526
F1M208
D3KR63
D4A5X1
D3ZDC0
D3ZLQ1
F1M0L3
D4A110
M0RDW5
Q562A6
Q9QYV3
D3ZEL0
E9PT56
F1MA70
A0A0G2KBA5
D3ZYK9
A0A0G2JU16
D3ZGH9
Q5BK33
A0A0G2JWS3
A0A0G2JSP3
D4AAF8
F1LPG6
D4AEG3
F1LUR6
F1LQN9
A0A0G2KB63
O70474
Q8CIY7
A0A0G2JYD6
A0A096MJC6
F1MA82
E9PTR3
A7L638
A0A0G2K4K3
Q6AYM6
Q7M0D5
A0A0G2JVA7
A0A0G2JYS8
A0A096MJT2
M0R3Z1
M0RCV5
A0A0G2K8X3
A0A0G2JYG9

F1LZD2
A0A0G2JX20
A0A0G2K2S2
D3ZH55
A0A0G2K036
Q4V7D0
F1M5Q4
D4AE85
A0A0G2JW08
A0A0G2JWM9
A0A0G2JX30
A0A0G2K6N2
B2GUW9
A0A0G2JU85
Q9JHE0
Q66LH8
Q5PSQ6
A0A140TAI1
A0A140TAI8
A0A140TAI4
A0A140TAG5
A0A140TAC8
A0A140TA99
Accession, Protein nu

Information Classification: General

Table S2. Complete list of proteins corresponding to the identified phosphopept

Microtubule-associated protein 1B OS=Rattus norvegicus GN=Map1b PE=1 SV=2 - [MAP1B_RAT]
Microtubule-associated protein 1A OS=Rattus norvegicus GN=Map1a PE=1 SV=1 - [MAP1A_RAT]
Microtubule associated protein 1A (Fragment) OS=Rattus norvegicus GN=Map1a PE=2 SV=1 - [Q63330_RAT]
Tubulin alpha-1A chain OS=Rattus norvegicus GN=Tuba1a PE=1 SV=1 - [TBA1A_RAT]
Tubulin alpha-4A chain OS=Rattus norvegicus GN=Tuba4a PE=1 SV=1 - [TBA4A_RAT]
Protein Ank2 OS=Rattus norvegicus GN=Ank2 PE=1 SV=3 - [F1M9N9_RAT]
Protein Ank2 OS=Rattus norvegicus GN=Ank2 PE=1 SV=1 - [A0A0G2K6R9_RAT]
Sodium/potassium-transporting ATPase subunit alpha-3 OS=Rattus norvegicus GN=Atp1a3 PE=1 SV=2 - [AT1A3_
2',3'-cyclic-nucleotide 3'-phosphodiesterase OS=Rattus norvegicus GN=Cnp PE=1 SV=2 - [CN37_RAT]
Protein Tppp OS=Rattus norvegicus GN=Tppp PE=1 SV=1 - [D3ZQL7_RAT]
SH3-containing GRB2-like protein 3-interacting protein 1 OS=Rattus norvegicus GN=Sgip1 PE=1 SV=3 - [F1M1Y0_
CD81 antigen OS=Rattus norvegicus GN=Cd81 PE=1 SV=1 - [CD81_RAT]
Neurofilament heavy polypeptide OS=Rattus norvegicus GN=Nefh PE=1 SV=1 - [F1LRZ7_RAT]
Neuromodulin OS=Rattus norvegicus GN=Gap43 PE=1 SV=1 - [NEUM_RAT]
Neurofilament medium polypeptide OS=Rattus norvegicus GN=Nefm PE=1 SV=4 - [NFM_RAT]
Sodium/potassium-transporting ATPase subunit alpha-2 OS=Rattus norvegicus GN=Atp1a2 PE=1 SV=1 - [AT1A2_
Microtubule-associated protein OS=Rattus norvegicus GN=Map2 PE=1 SV=1 - [A0A0U1RRX4_RAT]
Reticulon OS=Rattus norvegicus GN=Rtn4 PE=1 SV=1 - [F1LQN3_RAT]
Sodium/potassium-transporting ATPase subunit alpha-1 OS=Rattus norvegicus GN=Atp1a1 PE=1 SV=1 - [AT1A1_
Uncharacterized protein OS=Rattus norvegicus PE=3 SV=1 - [M0R5J4_RAT]
Protein kinase C gamma type OS=Rattus norvegicus GN=Prkcg PE=1 SV=1 - [KPCG_RAT]
14-3-3 protein zeta/delta OS=Rattus norvegicus GN=Ywhaz PE=1 SV=1 - [1433Z_RAT]
Actin, cytoplasmic 1 OS=Rattus norvegicus GN=Actb PE=1 SV=1 - [ACTB_RAT]
Actin, cytoplasmic 2 OS=Rattus norvegicus GN=Actg1 PE=1 SV=1 - [ACTG_RAT]
Microtubule-associated protein OS=Rattus norvegicus GN=Map2 PE=1 SV=3 - [F1MAQ5_RAT]
Calcium/calmodulin-dependent protein kinase type II subunit alpha OS=Rattus norvegicus GN=Camk2a PE=1 SV=1
Protein kinase C OS=Rattus norvegicus GN=Prkcb PE=1 SV=2 - [F1LS42_RAT]
Adducin 1 (Alpha), isoform CRA_b OS=Rattus norvegicus GN=Add1 PE=1 SV=1 - [A0A0G2JSM7_RAT]
Alpha-adducin OS=Rattus norvegicus GN=Add1 PE=1 SV=3 - [D3ZZ99_RAT]
Syntaxin-1B OS=Rattus norvegicus GN=Stx1b PE=1 SV=1 - [STX1B_RAT]
Dihydropyrimidinase-related protein 2 OS=Rattus norvegicus GN=Dpysl2 PE=1 SV=1 - [DPYL2_RAT]
Alpha-internexin OS=Rattus norvegicus GN=Ina PE=1 SV=2 - [AINX_RAT]
N-myc downstream regulated gene 2, isoform CRA_b OS=Rattus norvegicus GN=Ndrg2 PE=1 SV=1 - [A0A0G2JSU
Gamma-enolase OS=Rattus norvegicus GN=Eno2 PE=1 SV=2 - [ENOG_RAT]
Alpha-internexin OS=Rattus norvegicus GN=Ina PE=1 SV=1 - [G3V8Q2_RAT]
Heat shock protein HSP 90-beta OS=Rattus norvegicus GN=Hsp90ab1 PE=1 SV=4 - [HS90B_RAT]
Erythrocyte protein band 4.1-like 3, isoform CRA_b OS=Rattus norvegicus GN=Epb41l3 PE=1 SV=1 - [G3V874_RA
Beta-adducin OS=Rattus norvegicus GN=Add2 PE=1 SV=1 - [F8WFS9_RAT]
Tubulin beta-5 chain OS=Rattus norvegicus GN=Tubb5 PE=1 SV=1 - [TBB5_RAT]
Microtubule-associated protein OS=Rattus norvegicus GN=Mapt PE=1 SV=1 - [D4A1Q2_RAT]
14-3-3 protein gamma OS=Rattus norvegicus GN=Ywhag PE=1 SV=2 - [1433G_RAT]
A-kinase anchor protein 12 OS=Rattus norvegicus GN=Akap12 PE=1 SV=1 - [AKA12_RAT]
Tubulin beta chain OS=Rattus norvegicus GN=Tubb4b PE=1 SV=2 - [G3V7C6_RAT]
Tubulin beta chain OS=Rattus norvegicus GN=Tubb4a PE=1 SV=1 - [B4F7C2_RAT]
Bin1 protein OS=Rattus norvegicus GN=Bin1 PE=1 SV=1 - [Q5HZA7_RAT]
Receptor-type tyrosine-protein phosphatase zeta OS=Rattus norvegicus GN=Ptprz1 PE=1 SV=3 - [F1LMY3_RAT]
Tubulin beta-2A chain OS=Rattus norvegicus GN=Tubb2a PE=1 SV=1 - [TBB2A_RAT]
Tripartite motif-containing protein 2 OS=Rattus norvegicus GN=Trim2 PE=1 SV=2 - [TRIM2_RAT]
Protein Dmxl2 OS=Rattus norvegicus GN=Dmxl2 PE=1 SV=3 - [F1M3W5_RAT]
14-3-3 protein epsilon OS=Rattus norvegicus GN=Ywhae PE=1 SV=1 - [1433E_RAT]
Protein Sptbn1 OS=Rattus norvegicus GN=Sptbn1 PE=1 SV=3 - [G3V6S0_RAT]
Transcriptional activator protein Pur-beta OS=Rattus norvegicus GN=Purb PE=1 SV=1 - [A0A0G2JUX5_RAT]

Erythrocyte protein band 4.1-like 3, isoform CRA_e OS=Rattus norvegicus GN=Epb41l3 PE=1 SV=1 - [A0A0G2K1Q
Protein piccolo OS=Rattus norvegicus GN=Pclo PE=1 SV=3 - [D3Z9C7_RAT]
Syntaxin-1A OS=Rattus norvegicus GN=Stx1a PE=1 SV=1 - [STX1A_RAT]
Neurofilament light polypeptide OS=Rattus norvegicus GN=Nefl PE=1 SV=3 - [NFL_RAT]
Protein LOC100910792 OS=Rattus norvegicus GN=LOC100910792 PE=1 SV=1 - [A0A0G2K5Z4_RAT]
Protein bassoon OS=Rattus norvegicus GN=Bsn PE=4 SV=1 - [G3V984_RAT]
Protein piccolo OS=Rattus norvegicus GN=Pclo PE=1 SV=1 - [PCLO_RAT]
L-lactate dehydrogenase B chain OS=Rattus norvegicus GN=Ldhb PE=1 SV=2 - [LDHB_RAT]
Protein kinase C OS=Rattus norvegicus GN=Prkce PE=1 SV=3 - [F1LMV8_RAT]
Brain acid soluble protein 1 OS=Rattus norvegicus GN=Basp1 PE=1 SV=2 - [BASP1_RAT]
Prostaglandin E synthase 3 OS=Rattus norvegicus GN=Ptges3 PE=1 SV=2 - [TEBP_RAT]
Amino acid transporter OS=Rattus norvegicus GN=Slc1a3 PE=1 SV=1 - [A0A0G2K611_RAT]
Sodium/calcium exchanger 2 OS=Rattus norvegicus GN=Slc8a2 PE=3 SV=1 - [A0A0G2JZK7_RAT]
14-3-3 protein beta/alpha OS=Rattus norvegicus GN=Ywhab PE=1 SV=3 - [1433B_RAT]
Myristoylated alanine-rich C-kinase substrate OS=Rattus norvegicus GN=Marcks PE=1 SV=3 - [F1LMW7_RAT]
Myelin basic protein OS=Rattus norvegicus GN=Mbp PE=1 SV=3 - [MBP_RAT]
Mitochondrial import receptor subunit TOM70 OS=Rattus norvegicus GN=Tomm70 PE=1 SV=1 - [R9PXR4_RAT]
Calcium/calmodulin-dependent protein kinase II, beta, isoform CRA_a OS=Rattus norvegicus GN=Camk2b PE=1 S
Band 4.1-like protein 1 OS=Rattus norvegicus GN=Epb41l1 PE=1 SV=1 - [A0A0G2K0F3_RAT]
Stathmin (Fragment) OS=Rattus norvegicus GN=Stmn1 PE=1 SV=1 - [A0A096MK73_RAT]
Microtubule-associated protein OS=Rattus norvegicus GN=Map4 PE=1 SV=1 - [A0A0G2JW88_RAT]
Myosin, heavy polypeptide 10, non-muscle, isoform CRA_b OS=Rattus norvegicus GN=Myh10 PE=1 SV=1 - [G3V9
ATP synthase subunit beta OS=Rattus norvegicus GN=Atp5b PE=1 SV=1 - [G3V6D3_RAT]
60 kDa heat shock protein, mitochondrial OS=Rattus norvegicus GN=Hspd1 PE=1 SV=1 - [CH60_RAT]
Creatine kinase B-type OS=Rattus norvegicus GN=Ckb PE=1 SV=2 - [KCRB_RAT]
Tubulin beta-3 chain OS=Rattus norvegicus GN=Tubb3 PE=1 SV=1 - [TBB3_RAT]
Protein Ank2 OS=Rattus norvegicus GN=Ank2 PE=1 SV=1 - [A0A0G2JZ56_RAT]
Calcium/calmodulin-dependent protein kinase type II subunit beta OS=Rattus norvegicus GN=Camk2b PE=1 SV=1
Fructose-bisphosphate aldolase A OS=Rattus norvegicus GN=Aldoa PE=1 SV=2 - [ALDOA_RAT]
Protein Srrm2 OS=Rattus norvegicus GN=Srrm2 PE=1 SV=1 - [A0A0G2K2M9_RAT]
Reticulon-3 OS=Rattus norvegicus GN=Rtn3 PE=1 SV=1 - [RTN3_RAT]
Protein C2cd2l OS=Rattus norvegicus GN=C2cd2l PE=1 SV=1 - [Q5U2P5_RAT]
Protein C2cd2l OS=Rattus norvegicus GN=C2cd2l PE=1 SV=1 - [A0A0G2K2V4_RAT]
Protein kinase C OS=Rattus norvegicus GN=Prkca PE=1 SV=3 - [F1M2P8_RAT]
Sodium channel protein OS=Rattus norvegicus GN=Scn2a PE=1 SV=1 - [A0A0G2K207_RAT]
Myelin-associated glycoprotein OS=Rattus norvegicus GN=Mag PE=1 SV=1 - [G3V9B3_RAT]
Glyceraldehyde-3-phosphate dehydrogenase OS=Rattus norvegicus PE=1 SV=1 - [A0A0G2K7M1_RAT]
Proliferation-associated protein 2G4 OS=Rattus norvegicus GN=Pa2g4 PE=1 SV=1 - [A0A0G2JVS2_RAT]
Myelin proteolipid protein OS=Rattus norvegicus GN=Plp1 PE=1 SV=2 - [MYPR_RAT]
Actin, alpha cardiac muscle 1 OS=Rattus norvegicus GN=Actc1 PE=2 SV=1 - [ACTC_RAT]
Calnexin OS=Rattus norvegicus GN=Canx PE=1 SV=1 - [CALX_RAT]
Eukaryotic translation initiation factor 3 subunit B OS=Rattus norvegicus GN=Eif3b PE=1 SV=1 - [EIF3B_RAT]
Clathrin heavy chain OS=Rattus norvegicus GN=Cltc PE=1 SV=1 - [F1M779_RAT]
Eukaryotic translation initiation factor 4B OS=Rattus norvegicus GN=Eif4b PE=1 SV=1 - [Q5RKG9_RAT]
NAD-dependent protein deacetylase sirtuin-2 OS=Rattus norvegicus GN=Sirt2 PE=1 SV=1 - [A0A0G2JWM2_RAT]
DnaJ (Hsp40) homolog, subfamily C, member 5, isoform CRA_a OS=Rattus norvegicus GN=Dnajc5 PE=1 SV=1 - [
Spectrin alpha chain, non-erythrocytic 1 OS=Rattus norvegicus GN=Sptan1 PE=1 SV=2 - [SPTN1_RAT]
Transcription intermediary factor 1-beta OS=Rattus norvegicus GN=Trim28 PE=1 SV=2 - [TIF1B_RAT]
Protein Sept4 OS=Rattus norvegicus GN=Sept4 PE=1 SV=1 - [A0A096MJN4_RAT]
Excitatory amino acid transporter 2 OS=Rattus norvegicus GN=Slc1a2 PE=1 SV=2 - [EAA2_RAT]
ATP synthase subunit alpha, mitochondrial OS=Rattus norvegicus GN=Atp5a1 PE=1 SV=2 - [ATPA_RAT]
Syntaphilin OS=Rattus norvegicus GN=Snph PE=1 SV=1 - [SNPH_RAT]
Septin-7 OS=Rattus norvegicus GN=Sept7 PE=1 SV=3 - [F1LMC7_RAT]
Heat shock protein HSP 90-alpha OS=Rattus norvegicus GN=Hsp90aa1 PE=1 SV=3 - [HS90A_RAT]
Neural cell adhesion molecule 1 OS=Rattus norvegicus GN=Ncam1 PE=1 SV=1 - [A0A0G2K0M8_RAT]

Information Classification: General

Protein Ctnna1 OS=Rattus norvegicus GN=Ctnna1 PE=1 SV=1 - [A0A0G2JYF7_RAT]
D-tyrosyl-tRNA(Tyr) deacylase OS=Rattus norvegicus GN=Dtd1 PE=1 SV=1 - [B0K014_RAT]
Mitochondrial import receptor subunit TOM22 homolog OS=Rattus norvegicus GN=Tomm22 PE=1 SV=1 - [TOM22_
Protein Tom1l2 OS=Rattus norvegicus GN=Tom1l2 PE=1 SV=1 - [A0A0G2K9L2_RAT]
Glyceraldehyde-3-phosphate dehydrogenase OS=Rattus norvegicus PE=1 SV=1 - [M0R660_RAT]
Protein Kbtbd11 OS=Rattus norvegicus GN=Kbtbd11 PE=1 SV=1 - [D4A5J1_RAT]
Neurochondrin OS=Rattus norvegicus GN=Ncdn PE=1 SV=2 - [NCDN_RAT]
Synaptic vesicle glycoprotein 2A OS=Rattus norvegicus GN=Sv2a PE=1 SV=2 - [SV2A_RAT]
SRC kinase signaling inhibitor 1 OS=Rattus norvegicus GN=Srcin1 PE=1 SV=1 - [SRCN1_RAT]
Protein Myo18a OS=Rattus norvegicus GN=Myo18a PE=1 SV=2 - [D3ZFD0_RAT]
Amino acid transporter OS=Rattus norvegicus GN=Slc1a2 PE=2 SV=1 - [Q8K5B5_RAT]
Anion exchange protein OS=Rattus norvegicus GN=Slc4a4 PE=1 SV=1 - [A0A0H2UHB7_RAT]
Anion exchange protein OS=Rattus norvegicus GN=Slc4a10 PE=1 SV=2 - [D4AD03_RAT]
Nuclear ubiquitous casein and cyclin-dependent kinase substrate 1 OS=Rattus norvegicus GN=Nucks1 PE=1 SV=1
Microtubule-actin cross-linking factor 1 OS=Rattus norvegicus GN=Macf1 PE=1 SV=1 - [MACF1_RAT]
Platelet glycoprotein Ib beta chain OS=Rattus norvegicus GN=Sept5 PE=1 SV=2 - [D3ZT07_RAT]
Destrin OS=Rattus norvegicus GN=Dstn PE=1 SV=3 - [DEST_RAT]
Adaptor-related protein complex 3, delta 1 subunit, isoform CRA_b OS=Rattus norvegicus GN=Ap3d1 PE=1 SV=1 -
ADP/ATP translocase 1 OS=Rattus norvegicus GN=Slc25a4 PE=1 SV=3 - [ADT1_RAT]
Protein Tjp2 OS=Rattus norvegicus GN=Tjp2 PE=1 SV=1 - [Q3ZB99_RAT]
Gap junction alpha-1 protein OS=Rattus norvegicus GN=Gja1 PE=1 SV=2 - [CXA1_RAT]
Protein Hepacam OS=Rattus norvegicus GN=Hepacam PE=1 SV=2 - [D3ZEI4_RAT]
Epidermal growth factor receptor pathway substrate 15-like 1 OS=Rattus norvegicus GN=Eps15l1 PE=2 SV=1 - [A0
Sodium/hydrogen exchanger 1 OS=Rattus norvegicus GN=Slc9a1 PE=1 SV=2 - [SL9A1_RAT]
Nol5a protein (Fragment) OS=Rattus norvegicus GN=Nop56 PE=2 SV=1 - [Q5RJN5_RAT]
Myosin, heavy polypeptide 9, non-muscle OS=Rattus norvegicus GN=Myh9 PE=1 SV=1 - [G3V6P7_RAT]
Ankyrin-3 OS=Rattus norvegicus GN=Ank3 PE=1 SV=1 - [A0A0G2K1Q7_RAT]
Protein Epb41l2 OS=Rattus norvegicus GN=Epb41l2 PE=1 SV=1 - [A0A0G2K162_RAT]
Brain acid soluble protein 1 OS=Rattus norvegicus GN=Basp1 PE=1 SV=1 - [A0A0G2K1L8_RAT]
Protein Tnks1bp1 OS=Rattus norvegicus GN=Tnks1bp1 PE=1 SV=1 - [D3ZF26_RAT]
60S acidic ribosomal protein P2 OS=Rattus norvegicus GN=Rplp2 PE=1 SV=2 - [RLA2_RAT]
AP2-associated protein kinase 1 OS=Rattus norvegicus GN=Aak1 PE=1 SV=1 - [AAK1_RAT]
Cytoplasmic dynein 1 heavy chain 1 OS=Rattus norvegicus GN=Dync1h1 PE=1 SV=3 - [F1LRT9_RAT]
Eukaryotic translation initiation factor 3 subunit J OS=Rattus norvegicus GN=Eif3j PE=1 SV=1 - [EIF3J_RAT]
Protein Tmx1 OS=Rattus norvegicus GN=Tmx1 PE=1 SV=1 - [Q52KJ9_RAT]
Protein Rabl6 OS=Rattus norvegicus GN=Rabl6 PE=1 SV=1 - [D3ZKQ4_RAT]
Calcipressin-1 OS=Rattus norvegicus GN=Rcan1 PE=2 SV=1 - [RCAN1_RAT]
Serine/threonine-protein kinase N1 OS=Rattus norvegicus GN=Pkn1 PE=1 SV=2 - [PKN1_RAT]
Oxysterol-binding protein OS=Rattus norvegicus GN=Osbp PE=1 SV=2 - [D4A9D8_RAT]
14-3-3 protein eta OS=Rattus norvegicus GN=Ywhah PE=1 SV=2 - [1433F_RAT]
Uncharacterized protein OS=Rattus norvegicus PE=4 SV=1 - [A0A0G2K613_RAT]
Naca protein OS=Rattus norvegicus GN=Naca PE=1 SV=1 - [B2RYX0_RAT]
Armadillo repeat-containing protein 10 OS=Rattus norvegicus GN=Armc10 PE=1 SV=1 - [ARM10_RAT]
Phosphatidate cytidylyltransferase 2 OS=Rattus norvegicus GN=Cds2 PE=1 SV=1 - [CDS2_RAT]
Protein Usp5 OS=Rattus norvegicus GN=Usp5 PE=1 SV=1 - [D3ZVQ0_RAT]
Protein Iqsec1 OS=Rattus norvegicus GN=Iqsec1 PE=1 SV=1 - [A0A0G2JUG7_RAT]
Membrane-associated phosphatidylinositol transfer protein 1 OS=Rattus norvegicus GN=Pitpnm1 PE=1 SV=1 - [PIT
Reticulon-1 OS=Rattus norvegicus GN=Rtn1 PE=1 SV=1 - [RTN1_RAT]
Syntaxin-binding protein 1 OS=Rattus norvegicus GN=Stxbp1 PE=1 SV=1 - [STXB1_RAT]
Protein transport protein Sec31A OS=Rattus norvegicus GN=Sec31a PE=1 SV=1 - [A0A0G2K0X9_RAT]
G-protein signalling modulator 1 (AGS3-like, C. elegans), isoform CRA_c OS=Rattus norvegicus GN=Gpsm1 PE=1
Protein Sbf1 OS=Rattus norvegicus GN=Sbf1 PE=1 SV=1 - [M0RAP5_RAT]
Heterogeneous nuclear ribonucleoprotein D0 OS=Rattus norvegicus GN=Hnrnpd PE=1 SV=2 - [HNRPD_RAT]
Hepatoma-derived growth factor OS=Rattus norvegicus GN=Hdgf PE=1 SV=2 - [HDGF_RAT]
Protein phosphatase 1 regulatory subunit 14A OS=Rattus norvegicus GN=Ppp1r14a PE=1 SV=1 - [PP14A_RAT]

Erythrocyte protein band 4.9 (Predicted), isoform CRA_a OS=Rattus norvegicus GN=Dmtn PE=1 SV=1 - [D4A559_
Serine/threonine-protein kinase PAK 1 OS=Rattus norvegicus GN=Pak1 PE=1 SV=3 - [PAK1_RAT]
Protein Srrm1 OS=Rattus norvegicus GN=Srrm1 PE=1 SV=1 - [B2RYB3_RAT]
Protein Psd3 OS=Rattus norvegicus GN=Psd3 PE=1 SV=1 - [D4A404_RAT]
Platelet-activating factor acetylhydrolase IB subunit beta OS=Rattus norvegicus GN=Pafah1b2 PE=1 SV=1 - [PA1B
Gamma-adducin OS=Rattus norvegicus GN=Add3 PE=1 SV=2 - [ADDG_RAT]
Pyruvate kinase PKM OS=Rattus norvegicus GN=Pkm PE=1 SV=3 - [KPYM_RAT]
Gephyrin OS=Rattus norvegicus GN=Gphn PE=1 SV=2 - [M0R423_RAT]
Serine/threonine-protein phosphatase 2B catalytic subunit alpha isoform OS=Rattus norvegicus GN=Ppp3ca PE=1
Receptor-type tyrosine-protein phosphatase N2 OS=Rattus norvegicus GN=Ptprn2 PE=1 SV=1 - [PTPR2_RAT]
Tropomodulin-2 OS=Rattus norvegicus GN=Tmod2 PE=1 SV=1 - [TMOD2_RAT]
cAMP-dependent protein kinase type I-alpha regulatory subunit OS=Rattus norvegicus GN=Prkar1a PE=1 SV=2 - [
Protein Larp1 OS=Rattus norvegicus GN=Larp1 PE=1 SV=3 - [F1M062_RAT]
Ankyrin-3 OS=Rattus norvegicus GN=Ank3 PE=1 SV=1 - [A0A0G2K1R9_RAT]
RCG58555, isoform CRA_a OS=Rattus norvegicus GN=LOC683897 PE=2 SV=1 - [Q3B8P7_RAT]
Protein NDRG3 OS=Rattus norvegicus GN=Ndrg3 PE=1 SV=3 - [D4ACZ5_RAT]
Protein RUFY3 OS=Rattus norvegicus GN=Rufy3 PE=1 SV=1 - [RUFY3_RAT]
Alsin OS=Rattus norvegicus GN=Als2 PE=1 SV=1 - [ALS2_RAT]
Phosphoinositide phospholipase C OS=Rattus norvegicus GN=Plcl1 PE=1 SV=1 - [A0A0G2K4N6_RAT]
Mucolipin 1 (Predicted), isoform CRA_a OS=Rattus norvegicus GN=Mcoln1 PE=1 SV=1 - [D3ZRF9_RAT]
Unconventional myosin-Va OS=Rattus norvegicus GN=Myo5a PE=1 SV=1 - [A0A0G2K9S4_RAT]
cAMP-dependent protein kinase type II-beta regulatory subunit OS=Rattus norvegicus GN=Prkar2b PE=1 SV=3 - [K
14-3-3 protein theta OS=Rattus norvegicus GN=Ywhaq PE=1 SV=1 - [1433T_RAT]
Methyl-CpG-binding protein 2 OS=Rattus norvegicus GN=Mecp2 PE=1 SV=1 - [A0A0G2JWK2_RAT]
Calmodulin OS=Rattus norvegicus GN=Calm1 PE=1 SV=2 - [CALM_RAT]
Protein Cpne6 OS=Rattus norvegicus GN=Cpne6 PE=1 SV=2 - [D4ACG7_RAT]
Cytoplasmic dynein 1 light intermediate chain 1 OS=Rattus norvegicus GN=Dync1li1 PE=1 SV=1 - [G3V7G0_RAT]
Gamma-adducin OS=Rattus norvegicus GN=Add3 PE=1 SV=1 - [A0A0G2JW28_RAT]
Uncharacterized protein OS=Rattus norvegicus PE=1 SV=1 - [A0A0G2JZB6_RAT]
Synapsin-1 OS=Rattus norvegicus GN=Syn1 PE=1 SV=3 - [SYN1_RAT]
Potassium/sodium hyperpolarization-activated cyclic nucleotide-gated channel 2 OS=Rattus norvegicus GN=Hcn2 P
Dihydropyrimidinase-related protein 3 OS=Rattus norvegicus GN=Dpysl3 PE=1 SV=2 - [DPYL3_RAT]
Dynein cytoplasmic 1 intermediate chain 1C OS=Rattus norvegicus PE=2 SV=1 - [A0A173DW30_RAT]
Synapsin II, isoform CRA_a OS=Rattus norvegicus GN=Syn2 PE=1 SV=3 - [G3V733_RAT]
Calcium/calmodulin-dependent protein kinase type II subunit gamma OS=Rattus norvegicus GN=Camk2g PE=1 SV
Protein FAM134A OS=Rattus norvegicus GN=Fam134a PE=1 SV=1 - [A0A0G2K1U5_RAT]
Dynamin-1 OS=Rattus norvegicus GN=Dnm1 PE=1 SV=2 - [DYN1_RAT]
Ferritin heavy chain OS=Rattus norvegicus GN=Fth1 PE=1 SV=3 - [FRIH_RAT]
Aquaporin-4 OS=Rattus norvegicus GN=Aqp4 PE=1 SV=1 - [A0A0H2UHZ1_RAT]
Guanine nucleotide-binding protein G(i) subunit alpha-2 OS=Rattus norvegicus GN=Gnai2 PE=1 SV=3 - [GNAI2_R
Voltage-dependent P/Q-type calcium channel subunit alpha OS=Rattus norvegicus GN=Cacna1a PE=1 SV=1 - [A0
Uncharacterized protein OS=Rattus norvegicus PE=1 SV=1 - [A0A0G2K315_RAT]
BCL2-associated transcription factor 1, isoform CRA_a OS=Rattus norvegicus GN=Bclaf1 PE=1 SV=1 - [B1WC16_
Protein Arhgap23 OS=Rattus norvegicus GN=Arhgap23 PE=1 SV=3 - [F1M2D4_RAT]
Protein Srrm1 OS=Rattus norvegicus GN=Srrm1 PE=1 SV=1 - [A0A0G2K4F6_RAT]
Amyloid beta A4 precursor protein-binding family A member 1 OS=Rattus norvegicus GN=Apba1 PE=1 SV=1 - [APB
60S acidic ribosomal protein P0 OS=Rattus norvegicus GN=Rplp0 PE=1 SV=2 - [RLA0_RAT]
ADP/ATP translocase 2 OS=Rattus norvegicus GN=Slc25a5 PE=1 SV=3 - [ADT2_RAT]
Protein Mfap1a OS=Rattus norvegicus GN=Mfap1a PE=1 SV=1 - [D4ACM9_RAT]
Claudin OS=Rattus norvegicus GN=Cldn11 PE=1 SV=1 - [Q6IRG7_RAT]
Paralemmin-1 OS=Rattus norvegicus GN=Palm PE=1 SV=1 - [PALM_RAT]
Protein Palm3 OS=Rattus norvegicus GN=Palm3 PE=1 SV=2 - [D4A1J3_RAT]
Vesicle-associated membrane protein 2 (Fragment) OS=Rattus norvegicus PE=2 SV=1 - [Q19LA7_RAT]
Plasma membrane calcium-transporting ATPase 4 OS=Rattus norvegicus GN=Atp2b4 PE=1 SV=1 - [AT2B4_RAT]
Plectin OS=Rattus norvegicus GN=Plec PE=1 SV=1 - [A0A0G2K1J5_RAT]

Information Classification: General

Protein LOC103691939 OS=Rattus norvegicus GN=LOC103691939 PE=1 SV=1 - [F1M6C2_RAT]
Protein Pacs2 OS=Rattus norvegicus GN=Pacs2 PE=1 SV=2 - [D3ZJG4_RAT]
NADH-ubiquinone oxidoreductase 75 kDa subunit, mitochondrial OS=Rattus norvegicus GN=Ndufs1 PE=1 SV=1 - [
Triosephosphate isomerase OS=Rattus norvegicus GN=Tpi1 PE=1 SV=2 - [TPIS_RAT]
Elongation factor 1-delta OS=Rattus norvegicus GN=Eef1d PE=1 SV=2 - [EF1D_RAT]
Glial fibrillary acidic protein OS=Rattus norvegicus GN=Gfap PE=1 SV=2 - [GFAP_RAT]
Calcium/calmodulin-dependent protein kinase type II subunit delta OS=Rattus norvegicus GN=Camk2d PE=1 SV=1
Uncharacterized protein OS=Rattus norvegicus PE=4 SV=1 - [A0A0G2K4Q1_RAT]
Protein RUFY3 OS=Rattus norvegicus GN=Rufy3 PE=1 SV=1 - [A0A0G2K6A9_RAT]
Membrane-associated progesterone receptor component 1 OS=Rattus norvegicus GN=Pgrmc1 PE=1 SV=3 - [PGR
Protein S100-B OS=Rattus norvegicus GN=S100b PE=1 SV=2 - [S100B_RAT]
MAP kinase-activating death domain protein OS=Rattus norvegicus GN=Madd PE=1 SV=1 - [A0A0G2KA27_RAT]
Breast carcinoma-amplified sequence 1 homolog (Fragment) OS=Rattus norvegicus GN=Bcas1 PE=1 SV=2 - [BCA
Uncharacterized protein OS=Rattus norvegicus GN=Afdn PE=1 SV=3 - [F1LT10_RAT]
Protein Tp53bp1 OS=Rattus norvegicus GN=Tp53bp1 PE=1 SV=3 - [F1M842_RAT]
F-box only protein 6 OS=Rattus norvegicus GN=Fbxo6 PE=1 SV=1 - [FBX6_RAT]
Fructose-bisphosphate aldolase C OS=Rattus norvegicus GN=Aldoc PE=1 SV=3 - [ALDOC_RAT]
Proteasome subunit alpha type-3 OS=Rattus norvegicus GN=Psma3 PE=1 SV=3 - [PSA3_RAT]
Drebrin OS=Rattus norvegicus GN=Dbn1 PE=1 SV=3 - [DREB_RAT]
DnaJ (Hsp40) homolog, subfamily A, member 4 OS=Rattus norvegicus GN=Dnaja4 PE=1 SV=1 - [Q4QR73_RAT]
Neurabin-1 OS=Rattus norvegicus GN=Ppp1r9a PE=1 SV=1 - [NEB1_RAT]
Phosphatidylserine synthase 2 OS=Rattus norvegicus GN=Ptdss2 PE=1 SV=1 - [PTSS2_RAT]
Brevican core protein OS=Rattus norvegicus GN=Bcan PE=1 SV=1 - [G3V8G4_RAT]
Protein Ctnna2 OS=Rattus norvegicus GN=Ctnna2 PE=1 SV=3 - [D4A6H8_RAT]
ARF GTPase-activating protein GIT1 OS=Rattus norvegicus GN=Git1 PE=1 SV=1 - [A0A0G2K527_RAT]
28 kDa heat- and acid-stable phosphoprotein OS=Rattus norvegicus GN=Pdap1 PE=1 SV=1 - [HAP28_RAT]
Guanine nucleotide-binding protein G(i) subunit alpha-1 OS=Rattus norvegicus GN=Gnai1 PE=1 SV=3 - [GNAI1_RA
Serine/threonine-protein kinase DCLK1 OS=Rattus norvegicus GN=Dclk1 PE=1 SV=1 - [DCLK1_RAT]
Caskin-1 OS=Rattus norvegicus GN=Caskin1 PE=1 SV=2 - [D3ZE17_RAT]
Muscarinic acetylcholine receptor M2 OS=Rattus norvegicus GN=Chrm2 PE=1 SV=2 - [ACM2_RAT]
Coactosin-like protein OS=Rattus norvegicus GN=Cotl1 PE=1 SV=1 - [COTL1_RAT]
Protein Palm2 OS=Rattus norvegicus GN=Palm2 PE=1 SV=3 - [D4A4D8_RAT]
Creatine kinase, mitochondrial 1, ubiquitous OS=Rattus norvegicus GN=Ckmt1b PE=1 SV=1 - [Q5BJT9_RAT]
Synaptotagmin-7 OS=Rattus norvegicus GN=Syt7 PE=1 SV=1 - [SYT7_RAT]
Scg2 protein OS=Rattus norvegicus GN=Scg2 PE=1 SV=2 - [G3V7X2_RAT]
Uncharacterized protein OS=Rattus norvegicus PE=3 SV=1 - [M0RCB1_RAT]
WD repeat-containing protein 7 OS=Rattus norvegicus GN=Wdr7 PE=1 SV=1 - [WDR7_RAT]
Glycogen synthase kinase 3 beta, isoform CRA_b OS=Rattus norvegicus GN=Gsk3b PE=1 SV=1 - [A0A0G2JSH4_
Protein Braf OS=Rattus norvegicus GN=Braf PE=1 SV=3 - [F1M9C3_RAT]
Nuclear distribution protein nudE-like 1 OS=Rattus norvegicus GN=Ndel1 PE=1 SV=1 - [NDEL1_RAT]
Protein Ttc7b OS=Rattus norvegicus GN=Ttc7b PE=1 SV=3 - [F1LUY5_RAT]
Protein Stxbp5l OS=Rattus norvegicus GN=Stxbp5l PE=1 SV=3 - [D3ZU84_RAT]
Spectrin beta 3 OS=Rattus norvegicus GN=Sptbn2 PE=1 SV=2 - [F1MA36_RAT]
Regulating synaptic membrane exocytosis protein 1 OS=Rattus norvegicus GN=Rims1 PE=1 SV=1 - [A0A0G2KAV
Tenascin-R OS=Rattus norvegicus GN=Tnr PE=1 SV=3 - [F1LQ63_RAT]
Protein Hnrnpul2 OS=Rattus norvegicus GN=Hnrnpul2 PE=1 SV=1 - [D4ABT8_RAT]
Protein NDRG1 OS=Rattus norvegicus GN=Ndrg1 PE=1 SV=1 - [NDRG1_RAT]
Protein Wfs1 OS=Rattus norvegicus GN=Wfs1 PE=1 SV=2 - [E9PT53_RAT]
Protein RGD1560470 OS=Rattus norvegicus GN=RGD1560470 PE=1 SV=3 - [D3ZTF0_RAT]
Protein Herc1 OS=Rattus norvegicus GN=Herc1 PE=1 SV=1 - [A0A0G2JTT6_RAT]
Drebrin OS=Rattus norvegicus GN=Dbn1 PE=1 SV=1 - [A0A0H2UHL9_RAT]
Protein phosphatase 1 regulatory subunit 7 OS=Rattus norvegicus GN=Ppp1r7 PE=1 SV=1 - [PP1R7_RAT]
Anion exchange protein OS=Rattus norvegicus GN=Slc4a3 PE=1 SV=1 - [G3V8P8_RAT]
DNA topoisomerase 2 OS=Rattus norvegicus GN=Top2b PE=2 SV=1 - [Q14TE9_RAT]
PC4 and SFRS1-interacting protein OS=Rattus norvegicus GN=Psip1 PE=1 SV=1 - [Q566D6_RAT]

Information Classification: General

Uncharacterized protein OS=Rattus norvegicus PE=1 SV=1 - [A0A0G2JY03_RAT]
Dynactin subunit 1 OS=Rattus norvegicus GN=Dctn1 PE=1 SV=1 - [A0A0G2K428_RAT]
High mobility group protein HMG-I/HMG-Y OS=Rattus norvegicus GN=Hmga1 PE=1 SV=3 - [HMGA1_RAT]
Dual-specificity mitogen-activated protein kinase kinase 1 OS=Rattus norvegicus GN=Map2k1 PE=1 SV=1 - [A0A0H
Protein Hectd4 OS=Rattus norvegicus GN=Hectd4 PE=1 SV=3 - [F1LZX5_RAT]
ATP-binding cassette sub-family F member 1 OS=Rattus norvegicus GN=Abcf1 PE=1 SV=1 - [ABCF1_RAT]
5'-AMP-activated protein kinase subunit beta-2 OS=Rattus norvegicus GN=Prkab2 PE=1 SV=1 - [A0A0G2JVK3_RA
Astrocytic phosphoprotein PEA-15 OS=Rattus norvegicus GN=Pea15 PE=1 SV=1 - [PEA15_RAT]
Oxidation resistance protein 1 OS=Rattus norvegicus GN=Oxr1 PE=1 SV=1 - [A0A0G2JZ50_RAT]
Ubiquitin-like modifier-activating enzyme 1 OS=Rattus norvegicus GN=Uba1 PE=1 SV=1 - [UBA1_RAT]
Calcium/calmodulin-dependent protein kinase type II subunit gamma OS=Rattus norvegicus GN=Camk2g PE=1 SV
Syntaxin 4A (Placental), isoform CRA_a OS=Rattus norvegicus GN=Stx4 PE=1 SV=1 - [G3V8I4_RAT]
Protein Usp47 OS=Rattus norvegicus GN=Usp47 PE=1 SV=1 - [F1MAA1_RAT]
Eukaryotic translation initiation factor 3 subunit C OS=Rattus norvegicus GN=Eif3c PE=1 SV=1 - [EIF3C_RAT]
Phosphoinositide phospholipase C OS=Rattus norvegicus GN=Plcl2 PE=1 SV=2 - [F1M324_RAT]
Protein Acin1 OS=Rattus norvegicus GN=Acin1 PE=1 SV=1 - [E9PST5_RAT]
Protein Abr OS=Rattus norvegicus GN=Abr PE=1 SV=1 - [A0A0G2JTR4_RAT]
3-phosphoinositide-dependent protein kinase 1 OS=Rattus norvegicus GN=Pdpk1 PE=1 SV=2 - [PDPK1_RAT]
Septin-8 OS=Rattus norvegicus GN=Sept8 PE=1 SV=1 - [SEPT8_RAT]
Pcbp2 protein OS=Rattus norvegicus GN=Pcbp2 PE=1 SV=1 - [Q4V8F6_RAT]
Connector enhancer of kinase suppressor of ras 2 OS=Rattus norvegicus GN=Cnksr2 PE=1 SV=1 - [CNKR2_RAT]
Importin subunit alpha OS=Rattus norvegicus GN=Kpna3 PE=1 SV=1 - [Q56R18_RAT]
Gamma-aminobutyric acid type B receptor subunit 2 OS=Rattus norvegicus GN=Gabbr2 PE=1 SV=1 - [A0A0A0MX
Brefeldin A-inhibited guanine nucleotide-exchange protein 2 OS=Rattus norvegicus GN=Arfgef2 PE=1 SV=1 - [BIG2
Protein Vps51 OS=Rattus norvegicus GN=Vps51 PE=1 SV=2 - [F1M4I4_RAT]
Glutathione S-transferase P OS=Rattus norvegicus GN=Gstp1 PE=1 SV=2 - [GSTP1_RAT]
Protein kinase C and casein kinase substrate in neurons protein 1 OS=Rattus norvegicus GN=Pacsin1 PE=1 SV=1
Adenylate cyclase 9 (Predicted), isoform CRA_b OS=Rattus norvegicus GN=Adcy9 PE=1 SV=2 - [M0R5U4_RAT]
Dynamin-3 OS=Rattus norvegicus GN=Dnm3 PE=1 SV=2 - [DYN3_RAT]
Disks large homolog 4 OS=Rattus norvegicus GN=Dlg4 PE=1 SV=1 - [DLG4_RAT]
Protein Dock10 OS=Rattus norvegicus GN=Dock10 PE=1 SV=1 - [A0A0G2K939_RAT]
Microtubule-associated protein 1S OS=Rattus norvegicus GN=Map1s PE=1 SV=1 - [A0A0H2UHQ3_RAT]
Voltage-dependent anion-selective channel protein 1 OS=Rattus norvegicus GN=Vdac1 PE=1 SV=4 - [VDAC1_RAT
Protein Fmn2 OS=Rattus norvegicus GN=Fmn2 PE=1 SV=1 - [A0A0G2JZ27_RAT]
Protein Sntb2 OS=Rattus norvegicus GN=Sntb2 PE=1 SV=3 - [D3ZMX6_RAT]
Protein Pgm2l1 OS=Rattus norvegicus GN=Pgm2l1 PE=1 SV=1 - [D3Z955_RAT]
Synaptosomal-associated protein 25 OS=Rattus norvegicus GN=Snap25 PE=1 SV=1 - [SNP25_RAT]
Dihydropyrimidinase-related protein 1 OS=Rattus norvegicus GN=Crmp1 PE=1 SV=1 - [DPYL1_RAT]
Protein Stard10 OS=Rattus norvegicus GN=Stard10 PE=1 SV=1 - [Q5BJN1_RAT]
Protein Vamp4 OS=Rattus norvegicus GN=Vamp4 PE=1 SV=1 - [A0A096MJ99_RAT]
Rap guanine nucleotide exchange factor 2 OS=Rattus norvegicus GN=Rapgef2 PE=1 SV=2 - [RPGF2_RAT]
Discs, large (Drosophila) homolog-associated protein 1, isoform CRA_a OS=Rattus norvegicus GN=Dlgap1 PE=1 S
Putative anion exchanger isoform 2 (Fragment) OS=Rattus norvegicus GN=Slc4a2 PE=2 SV=1 - [Q9JJS4_RAT]
Myotubularin-related protein (Fragment) OS=Rattus norvegicus GN=Mtmr9 PE=2 SV=1 - [Q8K3W5_RAT]
Stathmin OS=Rattus norvegicus GN=Stmn3 PE=1 SV=1 - [A0A0G2K8P5_RAT]
Adenomatosis polyposis coli, isoform CRA_a OS=Rattus norvegicus GN=Apc PE=1 SV=1 - [G3V8Q9_RAT]
Eukaryotic translation initiation factor 5B OS=Rattus norvegicus GN=Eif5b PE=1 SV=1 - [A0A0H2UHV4_RAT]
Striatin-3 OS=Rattus norvegicus GN=Strn3 PE=1 SV=2 - [STRN3_RAT]
Serine/threonine-protein kinase BRSK2 OS=Rattus norvegicus GN=Brsk2 PE=1 SV=2 - [M9MMM8_RAT]
Protein phosphatase inhibitor 2 OS=Rattus norvegicus GN=Ppp1r2 PE=1 SV=1 - [A0A0H2UHA0_RAT]
Sphingosine 1-phosphate receptor 5 OS=Rattus norvegicus GN=S1pr5 PE=1 SV=1 - [S1PR5_RAT]
Septin-2 OS=Rattus norvegicus GN=Sept2 PE=1 SV=1 - [SEPT2_RAT]
Ubiquitin thioesterase OTUB1 OS=Rattus norvegicus GN=Otub1 PE=1 SV=1 - [OTUB1_RAT]
T-complex protein 1 subunit epsilon OS=Rattus norvegicus GN=Cct5 PE=1 SV=1 - [TCPE_RAT]
Catenin delta-2 OS=Rattus norvegicus GN=Ctnnd2 PE=1 SV=3 - [F1M787_RAT]

Information Classification: General

Protein Ppp2r1a OS=Rattus norvegicus GN=Ppp2r1a PE=1 SV=1 - [Q5XI34_RAT]
Protein Tanc2 OS=Rattus norvegicus GN=Tanc2 PE=1 SV=1 - [A0A0G2K9J0_RAT]
Protocadherin 1 (Fragment) OS=Rattus norvegicus GN=Pcdh1 PE=2 SV=1 - [Q00A22_RAT]
AP-3 complex subunit beta OS=Rattus norvegicus GN=Ap3b2 PE=1 SV=1 - [D4AE00_RAT]
Protein Atp8a1 OS=Rattus norvegicus GN=Atp8a1 PE=1 SV=3 - [D3ZJK8_RAT]
Ermin OS=Rattus norvegicus GN=Ermn PE=1 SV=1 - [A0A0G2K774_RAT]
Eukaryotic translation elongation factor 1 beta 2 OS=Rattus norvegicus GN=Eef1b2 PE=1 SV=1 - [B5DEN5_RAT]
Protein Slc7a14 OS=Rattus norvegicus GN=Slc7a14 PE=1 SV=2 - [D3ZSU3_RAT]
Protein Ccny OS=Rattus norvegicus GN=Ccny PE=1 SV=2 - [F1MA89_RAT]
Protein kinase, cAMP-dependent, regulatory, type 2, alpha, isoform CRA_a OS=Rattus norvegicus GN=Prkar2a PE=
Sarcoplasmic/endoplasmic reticulum calcium ATPase 2 OS=Rattus norvegicus GN=Atp2a2 PE=1 SV=1 - [AT2A2_F
Disks large-associated protein 2 OS=Rattus norvegicus GN=Dlgap2 PE=1 SV=3 - [F1LSL6_RAT]
Phosphodiesterase OS=Rattus norvegicus GN=Pde4b PE=1 SV=2 - [F1M1T9_RAT]
Tumor protein p63-regulated gene 1-like protein OS=Rattus norvegicus GN=Tprg1l PE=1 SV=1 - [A0A0G2K272_RA
Ral GTPase-activating protein subunit alpha-1 OS=Rattus norvegicus GN=Ralgapa1 PE=1 SV=1 - [A0A140TAA3_R
Protein Cep170b OS=Rattus norvegicus GN=Cep170b PE=1 SV=1 - [D4A1G8_RAT]
Protein Ube2o OS=Rattus norvegicus GN=Ube2o PE=1 SV=2 - [F1M403_RAT]
Ras-related protein Rap-2b OS=Rattus norvegicus GN=Rap2b PE=2 SV=1 - [RAP2B_RAT]
Rab3 GTPase-activating protein non-catalytic subunit OS=Rattus norvegicus GN=Rab3gap2 PE=1 SV=3 - [F1LMT8
Protein kinase C OS=Rattus norvegicus GN=Prkcb PE=1 SV=2 - [F1LS36_RAT]
cAMP-dependent protein kinase catalytic subunit beta OS=Rattus norvegicus GN=Prkacb PE=1 SV=2 - [KAPCB_RA
Catenin (Cadherin associated protein), beta 1, isoform CRA_a OS=Rattus norvegicus GN=Ctnnb1 PE=1 SV=1 - [A0
Oxidative-stress responsive 1 (Predicted) OS=Rattus norvegicus GN=Oxsr1 PE=1 SV=1 - [D3ZUC9_RAT]
Protein LOC100912571 OS=Rattus norvegicus GN=LOC100912571 PE=4 SV=1 - [M0RD03_RAT]
Small integral membrane protein 13 OS=Rattus norvegicus GN=Smim13 PE=3 SV=1 - [SIM13_RAT]
Protein Sptbn4 OS=Rattus norvegicus GN=Sptbn4 PE=1 SV=1 - [A0A0G2K677_RAT]
Protein RGD1311703 OS=Rattus norvegicus GN=RGD1311703 PE=1 SV=1 - [G3V8R0_RAT]
Tryptophan 5-hydroxylase 2 OS=Rattus norvegicus GN=Tph2 PE=1 SV=1 - [TPH2_RAT]
mRNA cap guanine-N7 methyltransferase OS=Rattus norvegicus GN=Rnmt PE=1 SV=1 - [MCES_RAT]
E3 ubiquitin-protein ligase HUWE1 OS=Rattus norvegicus GN=Huwe1 PE=1 SV=1 - [A0A0G2JVW5_RAT]
Protein RGD1307100 OS=Rattus norvegicus GN=RGD1307100 PE=1 SV=3 - [F1LVK0_RAT]
DnaJ (Hsp40) homolog, subfamily C, member 6 (Predicted) OS=Rattus norvegicus GN=Dnajc6 PE=1 SV=1 - [D4A0
Vimentin OS=Rattus norvegicus GN=Vim PE=1 SV=2 - [VIME_RAT]
AP-2 complex subunit beta OS=Rattus norvegicus GN=Ap2b1 PE=1 SV=1 - [AP2B1_RAT]
Gap junction gamma-2 protein OS=Rattus norvegicus GN=Gjc2 PE=1 SV=2 - [CXG2_RAT]
Potassium voltage-gated channel subfamily A member 2 OS=Rattus norvegicus GN=Kcna2 PE=1 SV=1 - [KCNA2_
Voltage-dependent N-type calcium channel subunit alpha-1B OS=Rattus norvegicus GN=Cacna1b PE=1 SV=1 - [CA
Cysteine and glycine-rich protein 1 OS=Rattus norvegicus GN=Csrp1 PE=1 SV=2 - [CSRP1_RAT]
Microtubule-associated protein 6 OS=Rattus norvegicus GN=Map6 PE=1 SV=3 - [F1LQZ9_RAT]
Liprin-alpha-3 OS=Rattus norvegicus GN=Ppfia3 PE=1 SV=2 - [F1LSE6_RAT]
Cytochrome b-c1 complex subunit 1, mitochondrial OS=Rattus norvegicus GN=Uqcrc1 PE=1 SV=1 - [QCR1_RAT]
Protein Sntb1 OS=Rattus norvegicus GN=Sntb1 PE=1 SV=1 - [D3ZWC6_RAT]
Ubiquitin carboxyl-terminal hydrolase 7 OS=Rattus norvegicus GN=Usp7 PE=1 SV=2 - [F1LM09_RAT]
Rho GTPase-activating protein 35 OS=Rattus norvegicus GN=Arhgap35 PE=1 SV=1 - [A0A0G2KB46_RAT]
Syntaxin-binding protein 5 OS=Rattus norvegicus GN=Stxbp5 PE=1 SV=1 - [STXB5_RAT]
Methylthioribose-1-phosphate isomerase OS=Rattus norvegicus GN=Mri1 PE=1 SV=1 - [MTNA_RAT]
ATP-binding cassette sub-family A member 2 OS=Rattus norvegicus GN=Abca2 PE=1 SV=2 - [G3V7X4_RAT]
Rab GDP dissociation inhibitor alpha OS=Rattus norvegicus GN=Gdi1 PE=1 SV=1 - [GDIA_RAT]
Protein Tjp1 OS=Rattus norvegicus GN=Tjp1 PE=1 SV=2 - [F1M4A0_RAT]
Phosphoribosyl pyrophosphate synthase-associated protein 2 OS=Rattus norvegicus GN=Prpsap2 PE=1 SV=1 - [Q
Histone H4 OS=Rattus norvegicus GN=Hist1h4b PE=1 SV=2 - [H4_RAT]
Phosphoinositide phospholipase C OS=Rattus norvegicus GN=Plch2 PE=1 SV=3 - [D4AAX6_RAT]
Atxn2l protein OS=Rattus norvegicus GN=Atxn2l PE=1 SV=1 - [B3DMA1_RAT]
Eukaryotic translation initiation factor 2, subunit 2 (Beta) OS=Rattus norvegicus GN=Eif2s2 PE=1 SV=1 - [Q6P685_
Heat shock 70kDa protein 12A (Predicted), isoform CRA_a OS=Rattus norvegicus GN=Hspa12a PE=1 SV=1 - [D3Z

Liprin-alpha-4 OS=Rattus norvegicus GN=Ppfia4 PE=1 SV=3 - [F1M863_RAT]
Clathrin coat assembly protein AP180 OS=Rattus norvegicus GN=Snap91 PE=1 SV=1 - [A0A0G2K0B6_RAT]
CLIP-associating protein 2 OS=Rattus norvegicus GN=Clasp2 PE=1 SV=1 - [A0A0G2JZM8_RAT]
Heterogeneous nuclear ribonucleoprotein K OS=Rattus norvegicus GN=Hnrnpk PE=1 SV=1 - [HNRPK_RAT]
Protein phosphatase 1 regulatory subunit 1B OS=Rattus norvegicus GN=Ppp1r1b PE=1 SV=1 - [PPR1B_RAT]
Furry-like protein OS=Rattus norvegicus GN=Fry PE=2 SV=1 - [C0IXW5_RAT]
Protein Mfsd6 OS=Rattus norvegicus GN=Mfsd6 PE=1 SV=1 - [A0A0G2JW92_RAT]
Protein Pkp4 OS=Rattus norvegicus GN=Pkp4 PE=1 SV=2 - [F1M2K6_RAT]
Malate dehydrogenase, cytoplasmic OS=Rattus norvegicus GN=Mdh1 PE=1 SV=3 - [MDHC_RAT]
Protein disulfide-isomerase OS=Rattus norvegicus GN=P4hb PE=1 SV=2 - [PDIA1_RAT]
Chromobox homolog 3 (HP1 gamma homolog, Drosophila) OS=Rattus norvegicus GN=Cbx3 PE=1 SV=1 - [Q5RJK[illegible]
Neural cell adhesion molecule 1 OS=Rattus norvegicus GN=Ncam1 PE=1 SV=3 - [F1LUV9_RAT]
Fam134c protein OS=Rattus norvegicus GN=Fam134c PE=1 SV=1 - [B2GV94_RAT]
Protein RGD1307235 OS=Rattus norvegicus GN=RGD1307235 PE=1 SV=1 - [D3ZJ01_RAT]
Ubiquitin carboxyl-terminal hydrolase OS=Rattus norvegicus GN=Uchl3 PE=1 SV=2 - [D4ABI6_RAT]
Phosphoglucomutase-1 OS=Rattus norvegicus GN=Pgm1 PE=1 SV=2 - [PGM1_RAT]
Prothymosin alpha OS=Rattus norvegicus GN=Ptma PE=1 SV=2 - [PTMA_RAT]
Pericentriolar material 1, isoform CRA_b OS=Rattus norvegicus GN=Pcm1 PE=1 SV=1 - [G3V7E6_RAT]
Acyl-CoA synthetase short-chain family member 2 (Predicted) OS=Rattus norvegicus GN=Acss2 PE=1 SV=1 - [G3V[illegible]
Cytoplasmic dynein 1 light intermediate chain 2 OS=Rattus norvegicus GN=Dync1li2 PE=1 SV=1 - [A0A096MIY2_R[illegible]
Plasma membrane calcium-transporting ATPase 2 OS=Rattus norvegicus GN=Atp2b2 PE=1 SV=2 - [AT2B2_RAT]
Protein Rps6kc1 OS=Rattus norvegicus GN=Rps6kc1 PE=1 SV=1 - [A0A0G2KB60_RAT]
Tripartite motif protein 3, isoform CRA_a OS=Rattus norvegicus GN=Trim3 PE=1 SV=1 - [G3V8D6_RAT]
Actin-binding LIM protein 2 OS=Rattus norvegicus GN=Ablim2 PE=1 SV=1 - [A0A0G2JTB5_RAT]
Protein Dst OS=Rattus norvegicus GN=Dst PE=1 SV=3 - [D3ZC56_RAT]
40S ribosomal protein S17 OS=Rattus norvegicus GN=Rps17 PE=3 SV=1 - [A0A0H2UHQ8_RAT]
WAS/WASL-interacting protein family member 3 OS=Rattus norvegicus GN=Wipf3 PE=1 SV=1 - [WIPF3_RAT]
Glucose-6-phosphate isomerase OS=Rattus norvegicus GN=Gpi PE=1 SV=1 - [G6PI_RAT]
Cdc42 effector protein 2 OS=Rattus norvegicus GN=Cdc42ep2 PE=1 SV=1 - [BORG1_RAT]
Nacad protein (Fragment) OS=Rattus norvegicus GN=Nacad PE=2 SV=1 - [Q5BJV5_RAT]
Huntingtin OS=Rattus norvegicus GN=Htt PE=1 SV=1 - [HD_RAT]
Phosphodiesterase OS=Rattus norvegicus GN=Pde4d PE=1 SV=1 - [A0A140TAB1_RAT]
Protein Ppfia2 OS=Rattus norvegicus GN=Ppfia2 PE=1 SV=2 - [F1M8A4_RAT]
Potassium voltage-gated channel subfamily KQT member 2 OS=Rattus norvegicus GN=Kcnq2 PE=1 SV=2 - [F1M6[illegible]
La-related protein 7 OS=Rattus norvegicus GN=Larp7 PE=1 SV=2 - [M0RC12_RAT]
Monocarboxylate transporter 1 OS=Rattus norvegicus GN=Slc16a1 PE=1 SV=1 - [MOT1_RAT]
Protein Rptor OS=Rattus norvegicus GN=Rptor PE=1 SV=2 - [D3ZDU2_RAT]
Sodium/potassium-transporting ATPase subunit alpha OS=Rattus norvegicus GN=Atp4a PE=1 SV=1 - [F1LRK1_R[illegible]
Microtubule-associated serine/threonine-protein kinase 1 OS=Rattus norvegicus GN=Mast1 PE=1 SV=1 - [MAST1_[illegible]
Epidermal growth factor receptor pathway substrate 15 isoform B OS=Rattus norvegicus GN=Eps15 PE=2 SV=1 - [[illegible]
Protein Strip1 OS=Rattus norvegicus GN=Strip1 PE=1 SV=2 - [G3V8E2_RAT]
Potassium/sodium hyperpolarization-activated cyclic nucleotide-gated channel 1 OS=Rattus norvegicus GN=Hcn1 P[illegible]
Protein Dhrs13 OS=Rattus norvegicus GN=Dhrs13 PE=1 SV=3 - [D3ZBC7_RAT]
Protein LYRIC OS=Rattus norvegicus GN=Mtdh PE=1 SV=2 - [LYRIC_RAT]
Protein Synm OS=Rattus norvegicus GN=Synm PE=1 SV=2 - [G3V9G5_RAT]
Protein RGD1310819 OS=Rattus norvegicus GN=RGD1310819 PE=1 SV=3 - [D3ZBU7_RAT]
Kinesin-like protein OS=Rattus norvegicus GN=Kif2a PE=1 SV=2 - [F1M8L1_RAT]
Glutamate receptor ionotropic, NMDA 2B OS=Rattus norvegicus GN=Grin2b PE=1 SV=1 - [G3V746_RAT]
Protein Prrt3 OS=Rattus norvegicus GN=Prrt3 PE=1 SV=2 - [D3ZWQ0_RAT]
Serine/threonine-protein kinase BRSK1 OS=Rattus norvegicus GN=Brsk1 PE=1 SV=1 - [BRSK1_RAT]
RCG61894, isoform CRA_a OS=Rattus norvegicus GN=Strn PE=1 SV=1 - [G3V6L8_RAT]
Thyroid hormone receptor-associated protein 3 OS=Rattus norvegicus GN=Thrap3 PE=1 SV=1 - [TR150_RAT]
Serine/threonine-protein kinase 24 OS=Rattus norvegicus GN=Stk24 PE=2 SV=1 - [STK24_RAT]
WASH complex subunit FAM21 OS=Rattus norvegicus GN=Fam21 PE=1 SV=1 - [FAM21_RAT]
Sodium- and chloride-dependent glycine transporter 1 OS=Rattus norvegicus GN=Slc6a9 PE=1 SV=2 - [SC6A9_RA[illegible]

Information Classification: General

RhoA (Fragment) OS=Rattus norvegicus GN=Rhoa PE=2 SV=1 - [O35791_RAT]
Adaptor-related protein complex 1, gamma 1 subunit, isoform CRA_b OS=Rattus norvegicus GN=Ap1g1 PE=2 SV=
Autophagy-related protein 9A OS=Rattus norvegicus GN=Atg9a PE=1 SV=1 - [ATG9A_RAT]
Clathrin coat assembly protein AP180 OS=Rattus norvegicus GN=Snap91 PE=1 SV=1 - [AP180_RAT]
Electrogenic sodium bicarbonate cotransporter 1 OS=Rattus norvegicus GN=Slc4a4 PE=1 SV=1 - [S4A4_RAT]
Adenylyl cyclase-associated protein 1 OS=Rattus norvegicus GN=Cap1 PE=1 SV=3 - [CAP1_RAT]
Guanine nucleotide-binding protein subunit gamma OS=Rattus norvegicus GN=Gng3 PE=1 SV=1 - [G3V8K2_RAT]
SMN splice variant OS=Rattus norvegicus PE=2 SV=1 - [Q2KP10_RAT]
Protein Fam169a OS=Rattus norvegicus GN=Fam169a PE=1 SV=2 - [D3ZKX8_RAT]
Cell adhesion molecule 4 OS=Rattus norvegicus GN=Cadm4 PE=1 SV=2 - [F1M7V6_RAT]
Protein Adam22 OS=Rattus norvegicus GN=Adam22 PE=1 SV=2 - [M0R5P8_RAT]
Protein Hacd3 OS=Rattus norvegicus GN=Hacd3 PE=1 SV=2 - [D4ABI7_RAT]
Transcriptional activator protein Pur-alpha (Fragments) OS=Rattus norvegicus GN=Pura PE=1 SV=1 - [PURA_RAT
Phosphatidylethanolamine-binding protein 1 OS=Rattus norvegicus GN=Pebp1 PE=1 SV=3 - [PEBP1_RAT]
Brain-specific angiogenesis inhibitor 1-associated protein 2 OS=Rattus norvegicus GN=Baiap2 PE=1 SV=1 - [BAIP2
Peptidyl-prolyl cis-trans isomerase A OS=Rattus norvegicus GN=Ppia PE=1 SV=2 - [PPIA_RAT]
Protein Asap2 OS=Rattus norvegicus GN=Asap2 PE=1 SV=1 - [A0A0G2K808_RAT]
Protein Map7d2 OS=Rattus norvegicus GN=Map7d2 PE=1 SV=3 - [D4A4L4_RAT]
PEX5-related protein OS=Rattus norvegicus GN=Pex5l PE=1 SV=2 - [F1LMT5_RAT]
F-actin-capping protein subunit beta OS=Rattus norvegicus GN=Capzb PE=1 SV=1 - [CAPZB_RAT]
Plasma membrane calcium-transporting ATPase 1 OS=Rattus norvegicus GN=Atp2b1 PE=1 SV=2 - [AT2B1_RAT]
Glycogen synthase kinase-3 alpha OS=Rattus norvegicus GN=Gsk3a PE=1 SV=1 - [A0A0G2K7W7_RAT]
Homer protein homolog 3 OS=Rattus norvegicus GN=Homer3 PE=1 SV=2 - [HOME3_RAT]
Protein kinase, cAMP-dependent, catalytic, alpha OS=Rattus norvegicus GN=Prkaca PE=1 SV=1 - [A1L1M0_RAT]
Protein LOC102556337 OS=Rattus norvegicus GN=Mff PE=1 SV=1 - [A0A0G2KAL9_RAT]
Scaffold attachment factor B1 OS=Rattus norvegicus GN=Safb PE=1 SV=1 - [A0A0G2JW97_RAT]
Regulator of microtubule dynamics protein 3 OS=Rattus norvegicus GN=Rmdn3 PE=1 SV=1 - [RMD3_RAT]
L-lactate dehydrogenase A chain OS=Rattus norvegicus GN=Ldha PE=1 SV=1 - [LDHA_RAT]
Adenylate kinase isoenzyme 1 OS=Rattus norvegicus GN=Ak1 PE=1 SV=3 - [KAD1_RAT]
Serine/threonine-protein kinase MARK1 OS=Rattus norvegicus GN=Mark1 PE=1 SV=1 - [A0A0G2K7H9_RAT]
Protein Usp14 OS=Rattus norvegicus GN=Usp14 PE=1 SV=1 - [A0A0G2JVV5_RAT]
Protein Specc1 OS=Rattus norvegicus GN=Specc1 PE=1 SV=1 - [Q4KLM7_RAT]
Striated muscle-specific serine/threonine-protein kinase OS=Rattus norvegicus GN=Speg PE=1 SV=2 - [SPEG_RA
Myosin-11 OS=Rattus norvegicus GN=Myh11 PE=1 SV=2 - [E9PTU4_RAT]
Protein Stk32c OS=Rattus norvegicus GN=Stk32c PE=1 SV=1 - [D4A3D9_RAT]
U5 small nuclear ribonucleoprotein 200 kDa helicase OS=Rattus norvegicus GN=Snrnp200 PE=1 SV=1 - [M3ZCQ2
Adaptor protein complex AP-1, sigma 1 (Predicted), isoform CRA_b OS=Rattus norvegicus GN=Ap1s1 PE=1 SV=1
Calcineurin subunit B type 1 OS=Rattus norvegicus GN=Ppp3r1 PE=4 SV=1 - [A0A0H2UHV6_RAT]
Serine/threonine-protein kinase DCLK1 OS=Rattus norvegicus GN=Dclk1 PE=1 SV=1 - [A0A0G2KB92_RAT]
Phosphoglycerate kinase 1 OS=Rattus norvegicus GN=Pgk1 PE=1 SV=2 - [PGK1_RAT]
Ras-specific guanine nucleotide-releasing factor 1 OS=Rattus norvegicus GN=Rasgrf1 PE=1 SV=1 - [RGRF1_RAT]
Protein Slc12a6 OS=Rattus norvegicus GN=Slc12a6 PE=1 SV=1 - [G3V6N7_RAT]
Protein Sash1 OS=Rattus norvegicus GN=Sash1 PE=1 SV=3 - [F1LU97_RAT]
Pre-B-cell leukemia transcription factor-interacting protein 1 OS=Rattus norvegicus GN=Pbxip1 PE=1 SV=1 - [PBIP
Neural cell adhesion molecule L1 OS=Rattus norvegicus GN=L1cam PE=1 SV=3 - [L1CAM_RAT]
Diacylglycerol kinase OS=Rattus norvegicus GN=Dgkh PE=1 SV=3 - [D3ZSZ6_RAT]
Glia maturation factor beta OS=Rattus norvegicus GN=Gmfb PE=1 SV=2 - [M0RDJ4_RAT]
Protein Usp24 OS=Rattus norvegicus GN=Usp24 PE=1 SV=2 - [F1LSM0_RAT]
FERM, RhoGEF and pleckstrin domain-containing protein 1 OS=Rattus norvegicus GN=Farp1 PE=1 SV=2 - [FARP
Phosphodiesterase OS=Rattus norvegicus GN=Pde2a PE=1 SV=1 - [A0A0G2K876_RAT]
PAK-interacting exchange factor beta2-PIX OS=Rattus norvegicus GN=Arhgef7 PE=2 SV=1 - [Q923I5_RAT]
Histone H3 OS=Rattus norvegicus GN=H3f3c PE=3 SV=1 - [D3ZK97_RAT]
Guanine nucleotide-binding protein G(o) subunit alpha OS=Rattus norvegicus GN=Gnao1 PE=1 SV=2 - [GNAO_RA
Histone H1.5 OS=Rattus norvegicus GN=Hist1h1b PE=1 SV=1 - [H15_RAT]
Protein Fam171a2 OS=Rattus norvegicus GN=Fam171a2 PE=1 SV=2 - [D3ZT47_RAT]

Information Classification: General

Dlg 2 (Fragment) OS=Rattus norvegicus GN=Mpp2 PE=2 SV=1 - [O88953_RAT]
Protein Kif21a OS=Rattus norvegicus GN=Kif21a PE=1 SV=2 - [D3ZCG2_RAT]
Nucleophosmin OS=Rattus norvegicus GN=Npm1 PE=1 SV=1 - [NPM_RAT]
Superkiller viralicidic activity 2-like (S. cerevisiae) OS=Rattus norvegicus GN=Skiv2l PE=4 SV=1 - [Q6MG76_RAT]
Rgs14 protein OS=Rattus norvegicus GN=Rgs14 PE=2 SV=1 - [Q5BKB9_RAT]
Phospholipid phosphatase-related protein type 2 OS=Rattus norvegicus GN=Plppr2 PE=1 SV=2 - [F1LR33_RAT]
Formin-binding protein 1 OS=Rattus norvegicus GN=Fnbp1 PE=1 SV=2 - [FNBP1_RAT]
Phosphoinositide phospholipase C OS=Rattus norvegicus GN=Plcb1 PE=1 SV=3 - [F1M084_RAT]
Intersectin 1 OS=Rattus norvegicus GN=Itsn1 PE=1 SV=3 - [D3ZV52_RAT]
Sorbin and SH3 domain-containing protein 1 OS=Rattus norvegicus GN=Sorbs1 PE=1 SV=1 - [F1M820_RAT]
Fatty acid synthase OS=Rattus norvegicus GN=Fasn PE=1 SV=3 - [FAS_RAT]
Protein C2cd5 OS=Rattus norvegicus GN=C2cd5 PE=1 SV=1 - [D4A5B3_RAT]
Protein Tnik OS=Rattus norvegicus GN=Tnik PE=1 SV=1 - [D3ZZQ0_RAT]
ADP-ribosylation factor 3 OS=Rattus norvegicus GN=Arf3 PE=1 SV=1 - [A0A0G2K4Q4_RAT]
STE20/SPS1-related proline-alanine-rich protein kinase OS=Rattus norvegicus GN=Stk39 PE=1 SV=1 - [A0A0G2K[illegible]
Protein NEWGENE_1594149 OS=Rattus norvegicus GN=Tceal5 PE=4 SV=1 - [M0RDJ7_RAT]
Cofilin-1 OS=Rattus norvegicus GN=Cfl1 PE=1 SV=3 - [COF1_RAT]
Protein LOC102548415 OS=Rattus norvegicus GN=Tceal6 PE=1 SV=1 - [M0RBL8_RAT]
Protein Atp6v1a OS=Rattus norvegicus GN=Atp6v1a PE=1 SV=1 - [D4A133_RAT]
CAP-Gly domain-containing linker protein 2 OS=Rattus norvegicus GN=Clip2 PE=1 SV=1 - [A0A0G2JZF2_RAT]
Alpha-2-HS-glycoprotein OS=Rattus norvegicus GN=Ahsg PE=1 SV=3 - [F1LM19_RAT]
Ras/Rap GTPase-activating protein SynGAP OS=Rattus norvegicus GN=Syngap1 PE=1 SV=3 - [D3ZCL8_RAT]
Phosphofurin acidic cluster sorting protein 1 OS=Rattus norvegicus GN=Pacs1 PE=1 SV=1 - [F1LPG3_RAT]
Ras and Rab interactor 1 OS=Rattus norvegicus GN=Rin1 PE=2 SV=2 - [RIN1_RAT]
Calpain-2 catalytic subunit OS=Rattus norvegicus GN=Capn2 PE=1 SV=3 - [CAN2_RAT]
Chloride intracellular channel protein OS=Rattus norvegicus GN=Clic6 PE=1 SV=1 - [F1M9X4_RAT]
Membrane-associated guanylate kinase, WW and PDZ domain-containing protein 2 OS=Rattus norvegicus GN=Ma
Calcium-transporting ATPase OS=Rattus norvegicus GN=Atp2b2 PE=1 SV=3 - [D4A8B3_RAT]
Leucine-rich repeat-containing protein 7 OS=Rattus norvegicus GN=Lrrc7 PE=1 SV=2 - [LRRC7_RAT]
Protein Lrrc47 OS=Rattus norvegicus GN=Lrrc47 PE=1 SV=2 - [F1LT49_RAT]
Protein Vps4b OS=Rattus norvegicus GN=Vps4b PE=1 SV=1 - [Q4KLL7_RAT]
Protein Cct7 OS=Rattus norvegicus GN=Cct7 PE=1 SV=1 - [D4AC23_RAT]
Protein Stt3b OS=Rattus norvegicus GN=Stt3b PE=1 SV=1 - [B2RYD7_RAT]
Eukaryotic translation initiation factor 3 subunit G OS=Rattus norvegicus GN=Eif3g PE=1 SV=1 - [EIF3G_RAT]
Protein Rras2 OS=Rattus norvegicus GN=Rras2 PE=1 SV=1 - [A0A0G2K508_RAT]
Transcriptional coactivator YAP1 OS=Rattus norvegicus GN=Yap1 PE=1 SV=1 - [R9PXS9_RAT]
Coiled-coil and C2 domain-containing protein 1A OS=Rattus norvegicus GN=Cc2d1a PE=1 SV=2 - [C2D1A_RAT]
Ethanolamine-phosphate cytidylyltransferase OS=Rattus norvegicus GN=Pcyt2 PE=1 SV=1 - [PCY2_RAT]
Protein Usp9x OS=Rattus norvegicus GN=Usp9x PE=1 SV=1 - [D3ZC84_RAT]
Protein Nav1 OS=Rattus norvegicus GN=Nav1 PE=1 SV=2 - [F1M031_RAT]
Histone H1.4 OS=Rattus norvegicus GN=Hist1h1e PE=1 SV=3 - [H14_RAT]
Protein Sptb OS=Rattus norvegicus GN=Sptb PE=1 SV=1 - [A0A140UHX6_RAT]
Histone deacetylase 2 (Fragment) OS=Rattus norvegicus GN=Hdac2 PE=2 SV=1 - [Q99PA1_RAT]
Tuberin OS=Rattus norvegicus GN=Tsc2 PE=1 SV=3 - [D3ZLW4_RAT]
Phosphate carrier protein, mitochondrial OS=Rattus norvegicus GN=Slc25a3 PE=1 SV=1 - [G3V741_RAT]
V-type proton ATPase subunit B, brain isoform OS=Rattus norvegicus GN=Atp6v1b2 PE=1 SV=1 - [VATB2_RAT]
Alpha-2A adrenergic receptor OS=Rattus norvegicus GN=Adra2a PE=1 SV=2 - [ADA2A_RAT]
Protein phosphatase 1E OS=Rattus norvegicus GN=Ppm1e PE=1 SV=1 - [PPM1E_RAT]
Epsin-1 OS=Rattus norvegicus GN=Epn1 PE=1 SV=1 - [EPN1_RAT]
Protein Abca8 OS=Rattus norvegicus GN=Abca8a PE=1 SV=3 - [D3ZXD2_RAT]
Small glutamine-rich tetratricopeptide repeat-containing protein alpha OS=Rattus norvegicus GN=Sgta PE=1 SV=1 -
Protein Irgq OS=Rattus norvegicus GN=Irgq PE=1 SV=2 - [M0R686_RAT]
Adapter molecule crk OS=Rattus norvegicus GN=Crk PE=1 SV=1 - [CRK_RAT]
Protein Ptges3l1 OS=Rattus norvegicus GN=Ptges3l1 PE=2 SV=1 - [Q5PQL9_RAT]
Acetyl-CoA carboxylase 1 OS=Rattus norvegicus GN=Acaca PE=1 SV=1 - [ACACA_RAT]

Information Classification: General

Protein Gab1 OS=Rattus norvegicus GN=Gab1 PE=1 SV=2 - [D3ZAL7_RAT]
Protein Mast3 OS=Rattus norvegicus GN=Mast3 PE=1 SV=1 - [D3ZL30_RAT]
AP complex subunit beta OS=Rattus norvegicus GN=Ap1b1 PE=1 SV=1 - [A0A0G2K2V2_RAT]
Protein Scrib OS=Rattus norvegicus GN=Scrib PE=1 SV=1 - [D3ZWS0_RAT]
Dynamin-1-like protein OS=Rattus norvegicus GN=Dnm1l PE=1 SV=1 - [DNM1L_RAT]
Synapsin-3 OS=Rattus norvegicus GN=Syn3 PE=1 SV=2 - [A0A096MIT7_RAT]
G protein-coupled receptor associated sorting protein 1 OS=Rattus norvegicus GN=Gprasp1 PE=1 SV=1 - [M0R9R[illegible]
Hepatoma-derived growth factor-related protein 2 OS=Rattus norvegicus GN=Hdgfrp2 PE=1 SV=1 - [A0A0G2JYC7_[illegible]
Rem2 protein OS=Rattus norvegicus GN=Rem2 PE=2 SV=1 - [A0JPL9_RAT]
A-kinase anchor protein 8 OS=Rattus norvegicus GN=Akap8 PE=1 SV=1 - [AKAP8_RAT]
Ras-related protein Rab-3A OS=Rattus norvegicus GN=Rab3a PE=1 SV=1 - [RAB3A_RAT]
Rap1gap protein (Fragment) OS=Rattus norvegicus GN=Rap1gap PE=2 SV=1 - [Q5EB70_RAT]
RNA polymerase-associated protein LEO1 OS=Rattus norvegicus GN=Leo1 PE=1 SV=1 - [LEO1_RAT]
Membrane-associated progesterone receptor component 2 OS=Rattus norvegicus GN=Pgrmc2 PE=1 SV=1 - [PGR[illegible]
Nibp protein (Fragment) OS=Rattus norvegicus GN=Trappc9 PE=2 SV=1 - [B1H266_RAT]
Sodium-dependent neutral amino acid transporter SLC6A17 OS=Rattus norvegicus GN=Slc6a17 PE=1 SV=1 - [S6[illegible]
Protein Kif1a OS=Rattus norvegicus GN=Kif1a PE=1 SV=3 - [F1M4A4_RAT]
Zinc transporter ZIP3 OS=Rattus norvegicus GN=Slc39a3 PE=1 SV=1 - [S39A3_RAT]
PERQ amino acid rich, with GYF domain 1 (Predicted) OS=Rattus norvegicus GN=Gigyf1 PE=1 SV=1 - [D3ZQJ3_R[illegible]
Protein Gstm7 OS=Rattus norvegicus GN=Gstm7 PE=1 SV=1 - [A0A0G2K4U2_RAT]
Malate dehydrogenase (Fragment) OS=Rattus norvegicus GN=Mdh2 PE=2 SV=1 - [Q0QF43_RAT]
Protein Shisa6 OS=Rattus norvegicus GN=Shisa6 PE=1 SV=3 - [D4A4M0_RAT]
Rap 1A (Fragment) OS=Rattus norvegicus GN=Rap1a PE=2 SV=1 - [O08813_RAT]
26S proteasome non-ATPase regulatory subunit 1 OS=Rattus norvegicus GN=Psmd1 PE=1 SV=1 - [A0A0G2JTW5[illegible]
Secretogranin-1 OS=Rattus norvegicus GN=Chgb PE=1 SV=2 - [SCG1_RAT]
ATP-dependent 6-phosphofructokinase, platelet type OS=Rattus norvegicus GN=Pfkp PE=1 SV=2 - [PFKAP_RAT]
Hsp70-binding protein 1 OS=Rattus norvegicus GN=Hspbp1 PE=1 SV=1 - [HPBP1_RAT]
Protein Stk4 OS=Rattus norvegicus GN=Stk4 PE=1 SV=1 - [D4A648_RAT]
Chaperonin containing Tcp1, subunit 6A (Zeta 1) OS=Rattus norvegicus GN=Cct6a PE=1 SV=1 - [Q3MHS9_RAT]
Receptor-type tyrosine-protein phosphatase alpha OS=Rattus norvegicus GN=Ptpra PE=1 SV=1 - [PTPRA_RAT]
Cdc42 effector protein 1 OS=Rattus norvegicus GN=Cdc42ep1 PE=1 SV=1 - [BORG5_RAT]
Protein Ppp6r3 OS=Rattus norvegicus GN=Ppp6r3 PE=1 SV=2 - [D3ZBT9_RAT]
Formin-like 1 (Predicted), isoform CRA_a OS=Rattus norvegicus GN=Fmnl1 PE=1 SV=1 - [D3ZAZ1_RAT]
Phosphatidylinositol 4-kinase type 2-alpha OS=Rattus norvegicus GN=Pi4k2a PE=1 SV=1 - [P4K2A_RAT]
Cyclin-G-associated kinase OS=Rattus norvegicus GN=Gak PE=1 SV=1 - [F1LMD9_RAT]
Na(+)/H(+) exchange regulatory cofactor NHE-RF1 OS=Rattus norvegicus GN=Slc9a3r1 PE=1 SV=3 - [NHRF1_RA[illegible]
Protein Nipbl OS=Rattus norvegicus GN=Nipbl PE=1 SV=1 - [A0A0G2K0J4_RAT]
26S protease regulatory subunit 6A OS=Rattus norvegicus GN=Psmc3 PE=1 SV=1 - [Q6P6U2_RAT]
Nucleolar protein 58 OS=Rattus norvegicus GN=Nop58 PE=1 SV=1 - [NOP58_RAT]
F-actin-capping protein subunit alpha-2 OS=Rattus norvegicus GN=Capza2 PE=1 SV=1 - [CAZA2_RAT]
Protein Tnfaip8l3 OS=Rattus norvegicus GN=Tnfaip8l3 PE=1 SV=2 - [F1LX20_RAT]
Dihydropteridine reductase OS=Rattus norvegicus GN=Qdpr PE=1 SV=1 - [DHPR_RAT]
Myelin-oligodendrocyte glycoprotein OS=Rattus norvegicus GN=Mog PE=1 SV=1 - [MOG_RAT]
Brain-specific angiogenesis inhibitor 3 (Predicted) OS=Rattus norvegicus GN=Adgrb3 PE=1 SV=1 - [D4A831_RAT]
Protein Tbc1d17 OS=Rattus norvegicus GN=Tbc1d17 PE=1 SV=1 - [B1H264_RAT]
Protein Fam177a1 OS=Rattus norvegicus GN=Fam177a1 PE=1 SV=1 - [D3ZIM7_RAT]
Vesicle-fusing ATPase OS=Rattus norvegicus GN=Nsf PE=1 SV=1 - [A0A0G2K6U1_RAT]
JIP3 protein OS=Rattus norvegicus GN=Mapk8ip3 PE=2 SV=1 - [B0VXR4_RAT]
Myelin-associated oligodendrocyte basic protein OS=Rattus norvegicus GN=Mobp PE=1 SV=1 - [MOBP_RAT]
Protein Hist1h1c OS=Rattus norvegicus GN=Hist1h1c PE=1 SV=1 - [A0A0G2K654_RAT]
Heterogeneous nuclear ribonucleoprotein H OS=Rattus norvegicus GN=Hnrnph1 PE=1 SV=1 - [Q499R8_RAT]
Poly A binding protein (Fragment) OS=Rattus norvegicus GN=Pabpn1 PE=2 SV=1 - [O08729_RAT]
Proteasome subunit beta type-7 OS=Rattus norvegicus GN=Psmb7 PE=1 SV=1 - [PSB7_RAT]
Protein Lamc1 OS=Rattus norvegicus GN=Lamc1 PE=1 SV=1 - [F1MAA7_RAT]
Ankyrin repeat domain-containing protein 34A OS=Rattus norvegicus GN=Ankrd34a PE=1 SV=1 - [AN34A_RAT]

ATP-citrate synthase OS=Rattus norvegicus GN=Acly PE=1 SV=1 - [A0A0G2K5E7_RAT]
RAC-alpha serine/threonine-protein kinase OS=Rattus norvegicus GN=Akt1 PE=1 SV=1 - [AKT1_RAT]
Sphingosine-1-phosphate phosphatase 1 OS=Rattus norvegicus GN=Sgpp1 PE=1 SV=2 - [SGPP1_RAT]
Protein Smarcc2 OS=Rattus norvegicus GN=Smarcc2 PE=1 SV=3 - [D4A510_RAT]
Protein RGD1560608 OS=Rattus norvegicus GN=RGD1560608 PE=1 SV=1 - [D3ZC31_RAT]
26S protease regulatory subunit 6A OS=Rattus norvegicus GN=Psmc3 PE=2 SV=1 - [PRS6A_RAT]
Protein Trappc10 OS=Rattus norvegicus GN=Trappc10 PE=1 SV=1 - [F1MAQ4_RAT]
CD2 antigen (Cytoplasmic tail) binding protein 2 (Predicted), isoform CRA_a OS=Rattus norvegicus GN=Cd2bp2 PE
GTPase activating RANGAP domain-like 4 (Predicted), isoform CRA_a OS=Rattus norvegicus GN=Rap1gap2 PE=
Ubiquitin carboxyl-terminal hydrolase isozyme L1 OS=Rattus norvegicus GN=Uchl1 PE=1 SV=2 - [UCHL1_RAT]
Sister chromatid cohesion protein PDS5 homolog B OS=Rattus norvegicus GN=Pds5b PE=1 SV=1 - [D3ZU56_RAT
Synaptophysin OS=Rattus norvegicus GN=Syp PE=1 SV=1 - [A0A0G2KAD8_RAT]
Protein Iqsec2 OS=Rattus norvegicus GN=Iqsec2 PE=1 SV=1 - [A0A0G2JZX5_RAT]
E3 ubiquitin-protein ligase UBR4 OS=Rattus norvegicus GN=Ubr4 PE=1 SV=1 - [A0A0G2JU89_RAT]
Protein Arhgap21 OS=Rattus norvegicus GN=Arhgap21 PE=1 SV=3 - [F1LXQ7_RAT]
LOC687696 protein OS=Rattus norvegicus GN=Stambpl1 PE=1 SV=1 - [B0BMZ5_RAT]
Protein Mtmr7 OS=Rattus norvegicus GN=Mtmr7 PE=1 SV=1 - [A0A0G2K1P1_RAT]
Protein Ttbk1 OS=Rattus norvegicus GN=Ttbk1 PE=1 SV=3 - [D3ZAU7_RAT]
Nucleosome assembly protein 1-like 4 OS=Rattus norvegicus GN=Nap1l4 PE=1 SV=1 - [NP1L4_RAT]
Protein LOC685203 OS=Rattus norvegicus GN=LOC685203 PE=1 SV=1 - [D3ZBW5_RAT]
Rho-related GTP-binding protein RhoB OS=Rattus norvegicus GN=Rhob PE=1 SV=1 - [RHOB_RAT]
Probable G-protein-coupled receptor 158 OS=Rattus norvegicus GN=Gpr158 PE=1 SV=1 - [A0A0A0MY13_RAT]
Protein Tmem88b OS=Rattus norvegicus GN=Tmem88b PE=1 SV=1 - [D4AAB3_RAT]
Guanine nucleotide-binding protein-like 1 OS=Rattus norvegicus GN=Gnl1 PE=1 SV=1 - [A0A0G2K6Q5_RAT]
Glyceraldehyde-3-phosphate dehydrogenase OS=Rattus norvegicus PE=3 SV=1 - [E9PTN6_RAT]
Protein Sik3 OS=Rattus norvegicus GN=Sik3 PE=1 SV=2 - [M0RD40_RAT]
Protein Ttbk2 OS=Rattus norvegicus GN=Ttbk2 PE=1 SV=1 - [D3ZN60_RAT]
26S proteasome subunit S5a OS=Rattus norvegicus GN=Psmd4 PE=2 SV=1 - [Q9ESH1_RAT]
Kinesin light chain 2 (Predicted), isoform CRA_b OS=Rattus norvegicus GN=Klc2 PE=1 SV=1 - [B2GV74_RAT]
Proprotein convertase subtilisin/kexin type 1 inhibitor OS=Rattus norvegicus GN=LOC103690091 PE=4 SV=1 - [G3
Pleckstrin homology-like domain family B member 1 OS=Rattus norvegicus GN=Phldb1 PE=1 SV=2 - [D3ZNS1_RA
MAP/microtubule affinity-regulating kinase 3 OS=Rattus norvegicus GN=Mark3 PE=1 SV=1 - [F1M836_RAT]
Protein Rap1gds1 OS=Rattus norvegicus GN=Rap1gds1 PE=1 SV=2 - [F1M7Y3_RAT]
Protein Trim32 OS=Rattus norvegicus GN=Trim32 PE=1 SV=1 - [Q66H79_RAT]
Protein Dcaf6 OS=Rattus norvegicus GN=Dcaf6 PE=1 SV=2 - [D4A8L4_RAT]
H/ACA ribonucleoprotein complex subunit 4 OS=Rattus norvegicus GN=Dkc1 PE=1 SV=1 - [A0A0G2K700_RAT]
Megalencephalic leukoencephalopathy with subcortical cysts 1 homolog (Human) (Predicted) OS=Rattus norvegicus
Protein Mtx3 OS=Rattus norvegicus GN=Mtx3 PE=1 SV=2 - [D3ZNK1_RAT]
COP9 signalosome complex subunit 1 OS=Rattus norvegicus GN=Gps1 PE=1 SV=1 - [CSN1_RAT]
Protein Hist1h2bh OS=Rattus norvegicus GN=Hist1h2bh PE=3 SV=1 - [A0A0G2K7R1_RAT]
Protein Map2k4 OS=Rattus norvegicus GN=Map2k4 PE=1 SV=3 - [F1LP57_RAT]
Focal adhesion kinase 1 OS=Rattus norvegicus GN=Ptk2 PE=1 SV=1 - [FAK1_RAT]
Transcriptional regulator ATRX OS=Rattus norvegicus GN=Atrx PE=1 SV=1 - [A0A0G2JXZ3_RAT]
Zinc finger Ran-binding domain-containing protein 2 OS=Rattus norvegicus GN=Zranb2 PE=1 SV=2 - [ZRAB2_RAT
Serine/threonine-protein kinase MARK2 OS=Rattus norvegicus GN=Mark2 PE=1 SV=1 - [MARK2_RAT]
Protein Mark4 OS=Rattus norvegicus GN=Mark4 PE=1 SV=1 - [A0A0G2JTD8_RAT]
ATP-dependent RNA helicase DDX1 OS=Rattus norvegicus GN=Ddx1 PE=1 SV=1 - [DDX1_RAT]
4-aminobutyrate aminotransferase, mitochondrial OS=Rattus norvegicus GN=Abat PE=1 SV=3 - [GABT_RAT]
RILP-like protein 1 OS=Rattus norvegicus GN=Rilpl1 PE=1 SV=1 - [A0A0G2K2U5_RAT]
Glutamate dehydrogenase 1, mitochondrial OS=Rattus norvegicus GN=Glud1 PE=1 SV=2 - [DHE3_RAT]
Protein LOC100912917 OS=Rattus norvegicus GN=LOC100912917 PE=1 SV=3 - [D4AB17_RAT]
Protein Arfgef3 OS=Rattus norvegicus GN=Arfgef3 PE=1 SV=3 - [D3ZF86_RAT]
Protein LOC100362751 OS=Rattus norvegicus GN=LOC498555 PE=3 SV=1 - [D4A4D5_RAT]
Transient receptor potential cation channel subfamily V member 2 OS=Rattus norvegicus GN=Trpv2 PE=1 SV=1 - [
Protein-tyrosine kinase 2-beta OS=Rattus norvegicus GN=Ptk2b PE=1 SV=1 - [FAK2_RAT]

Protein Spag9 OS=Rattus norvegicus GN=Spag9 PE=1 SV=2 - [E9PSJ4_RAT]
Calcium/calmodulin-dependent protein kinase kinase 1 OS=Rattus norvegicus GN=Camkk1 PE=1 SV=2 - [F1LQD2_
Phosphodiesterase 1A (Fragment) OS=Rattus norvegicus GN=Pde1a PE=2 SV=1 - [Q9EPS1_RAT]
Protein Sacs OS=Rattus norvegicus GN=Sacs PE=1 SV=2 - [D4A1D3_RAT]
Protein Phkb OS=Rattus norvegicus GN=Phkb PE=1 SV=1 - [A0A0G2K9C8_RAT]
Polyubiquitin-B OS=Rattus norvegicus GN=Ubb PE=1 SV=1 - [UBB_RAT]
Protein Abi2 OS=Rattus norvegicus GN=Abi2 PE=4 SV=3 - [F1LYA6_RAT]
Potassium voltage-gated channel subfamily A member 1 OS=Rattus norvegicus GN=Kcna1 PE=1 SV=1 - [KCNA1_
Splicing factor, arginine/serine-rich 19 OS=Rattus norvegicus GN=Scaf1 PE=1 SV=2 - [SFR19_RAT]
Protein Map7d1 OS=Rattus norvegicus GN=Map7d1 PE=1 SV=1 - [D4A644_RAT]
Protein Ube4b OS=Rattus norvegicus GN=Ube4b PE=1 SV=3 - [F1M8V2_RAT]
Potassium voltage-gated channel subfamily D member 2 OS=Rattus norvegicus GN=Kcnd2 PE=1 SV=1 - [KCND2_
Myotubularin related protein 2 (Predicted), isoform CRA_b OS=Rattus norvegicus GN=Mtmr2 PE=1 SV=1 - [D3ZA3
Protein Sin3a OS=Rattus norvegicus GN=Sin3a PE=1 SV=1 - [D3ZBP2_RAT]
Heat shock 70 kDa protein 4L OS=Rattus norvegicus GN=Hspa4l PE=1 SV=1 - [B4F772_RAT]
Eukaryotic translation initiation factor 5A-1 OS=Rattus norvegicus GN=Eif5a PE=1 SV=3 - [IF5A1_RAT]
Methylosome protein 50 OS=Rattus norvegicus GN=Wdr77 PE=1 SV=1 - [MEP50_RAT]
Protein Mon2 OS=Rattus norvegicus GN=Mon2 PE=1 SV=3 - [D3ZKH2_RAT]
Contactin-associated protein 1 OS=Rattus norvegicus GN=Cntnap1 PE=1 SV=1 - [CNTP1_RAT]
Protein RGD1304884 OS=Rattus norvegicus GN=RGD1304884 PE=1 SV=1 - [D4A3C2_RAT]
Voltage-dependent anion-selective channel protein 3 OS=Rattus norvegicus GN=Vdac3 PE=1 SV=1 - [A0A0G2JSR
Protein Slc9a7 OS=Rattus norvegicus GN=Slc9a7 PE=1 SV=2 - [D3ZCI2_RAT]
Fibroblast growth factor 12 OS=Rattus norvegicus GN=Fgf12 PE=3 SV=2 - [M0RBB2_RAT]
LOC688393 protein OS=Rattus norvegicus GN=Gpn1 PE=1 SV=1 - [B1WBZ7_RAT]
Protein Arhgap12 OS=Rattus norvegicus GN=Arhgap12 PE=1 SV=1 - [A0A0G2K1Z2_RAT]
Serine incorporator 1 OS=Rattus norvegicus GN=Serinc1 PE=1 SV=1 - [SERC1_RAT]
Protein kinase C OS=Rattus norvegicus GN=Prkcd PE=1 SV=3 - [D4A0U0_RAT]
Volume-regulated anion channel subunit LRRC8A OS=Rattus norvegicus GN=Lrrc8a PE=1 SV=1 - [LRC8A_RAT]
RIMS-binding protein 2 OS=Rattus norvegicus GN=Rimbp2 PE=1 SV=1 - [A0A0G2K4J8_RAT]
Kinesin family member 21B (Predicted) OS=Rattus norvegicus GN=Kif21b PE=1 SV=2 - [F1M5N7_RAT]
Protein Npepps OS=Rattus norvegicus GN=Npepps PE=1 SV=1 - [F1M9V7_RAT]
Disks large homolog 2 OS=Rattus norvegicus GN=Dlg2 PE=1 SV=1 - [DLG2_RAT]
Polypyrimidine tract-binding protein 2 OS=Rattus norvegicus GN=Ptbp2 PE=1 SV=1 - [PTBP2_RAT]
Hematological and neurological expressed 1 protein OS=Rattus norvegicus GN=Hn1 PE=1 SV=3 - [HN1_RAT]
Protein LOC103690160 OS=Rattus norvegicus GN=Shank2 PE=1 SV=1 - [A0A0G2K7H2_RAT]
Synaptic vesicle 2-related protein OS=Rattus norvegicus GN=Svop PE=1 SV=1 - [A0A0G2JZX3_RAT]
Lamina-associated polypeptide 2, isoform beta OS=Rattus norvegicus GN=Tmpo PE=1 SV=3 - [LAP2_RAT]
Protein Snta1 OS=Rattus norvegicus GN=Snta1 PE=1 SV=1 - [B5DFL0_RAT]
Epsin-2 OS=Rattus norvegicus GN=Epn2 PE=1 SV=2 - [F1LQ45_RAT]
Protein Sltm OS=Rattus norvegicus GN=Sltm PE=1 SV=1 - [A0A0G2K904_RAT]
Misshapen-like kinase 1 OS=Rattus norvegicus GN=Mink1 PE=1 SV=1 - [A0A0G2K382_RAT]
Hexokinase-1 OS=Rattus norvegicus GN=Hk1 PE=1 SV=4 - [HXK1_RAT]
Discs, large homolog-associated protein 4 (Drosophila) OS=Rattus norvegicus GN=Dlgap4 PE=1 SV=1 - [G3V927_
Protein kinase C and casein kinase substrate in neurons protein 1 OS=Rattus norvegicus GN=Pacsin1 PE=1 SV=1
Protein Gprin2 OS=Rattus norvegicus GN=Gprin2 PE=1 SV=1 - [D3ZH78_RAT]
Protein Strn4 OS=Rattus norvegicus GN=Strn4 PE=1 SV=1 - [F1M6V8_RAT]
Protein Tln2 OS=Rattus norvegicus GN=Tln2 PE=1 SV=3 - [D3ZA84_RAT]
G protein-coupled receptor 21 (Predicted), isoform CRA_a OS=Rattus norvegicus GN=Rabgap1 PE=1 SV=1 - [D3Z
Lamin A, isoform CRA_b OS=Rattus norvegicus GN=Lmna PE=1 SV=1 - [G3V8L3_RAT]
Transgelin-3 OS=Rattus norvegicus GN=Tagln3 PE=1 SV=2 - [TAGL3_RAT]
Protein Baiap3 OS=Rattus norvegicus GN=Baiap3 PE=1 SV=2 - [F1LVS1_RAT]
Protein G3bp1 OS=Rattus norvegicus GN=G3bp1 PE=1 SV=1 - [D3ZYS7_RAT]
Protein S100a13 OS=Rattus norvegicus GN=S100a13 PE=1 SV=2 - [D3ZTB5_RAT]
Exocyst complex component 8 OS=Rattus norvegicus GN=Exoc8 PE=1 SV=1 - [EXOC8_RAT]
Bcl2-associated agonist of cell death OS=Rattus norvegicus GN=Bad PE=1 SV=2 - [BAD_RAT]

Information Classification: General

Chromatin modifying protein 6 (Predicted) OS=Rattus norvegicus GN=Chmp6 PE=1 SV=1 - [D3ZDR2_RAT]
Lipin 2 (Predicted) OS=Rattus norvegicus GN=Lpin2 PE=1 SV=1 - [D3ZYB4_RAT]
RGD1308350 protein (Fragment) OS=Rattus norvegicus GN=Inf2 PE=2 SV=1 - [Q5I0K2_RAT]
Transketolase OS=Rattus norvegicus GN=Tkt PE=1 SV=1 - [TKT_RAT]
6-phosphogluconolactonase OS=Rattus norvegicus GN=Pgls PE=1 SV=1 - [6PGL_RAT]
Rho guanine nucleotide exchange factor 2 OS=Rattus norvegicus GN=Arhgef2 PE=4 SV=1 - [A0A1B0GWY5_RAT]
Proline-, glutamic acid- and leucine-rich protein 1 OS=Rattus norvegicus GN=Pelp1 PE=1 SV=2 - [PELP1_RAT]
Solute carrier family 23 member 2 OS=Rattus norvegicus GN=Slc23a2 PE=1 SV=2 - [S23A2_RAT]
RCG57812, isoform CRA_b OS=Rattus norvegicus GN=Slk PE=1 SV=1 - [G3V7I8_RAT]
Protein Ccdc136 OS=Rattus norvegicus GN=Ccdc136 PE=1 SV=3 - [D4AAR7_RAT]
Protein Vps53 OS=Rattus norvegicus GN=Vps53 PE=1 SV=1 - [B4F763_RAT]
Lethal(2) giant larvae protein homolog 1 OS=Rattus norvegicus GN=Llgl1 PE=1 SV=2 - [G3V6I1_RAT]
Beta-arrestin-1 OS=Rattus norvegicus GN=Arrb1 PE=1 SV=1 - [ARRB1_RAT]
NSFL1 cofactor p47 OS=Rattus norvegicus GN=Nsfl1c PE=1 SV=1 - [A0A0G2K911_RAT]
Calcium/calmodulin-dependent protein kinase kinase 2 OS=Rattus norvegicus GN=Camkk2 PE=1 SV=1 - [KKCC2_
Cell cycle exit and neuronal differentiation protein 1 OS=Rattus norvegicus GN=Cend1 PE=1 SV=1 - [CEND_RAT]
Bromodomain adjacent to zinc finger domain protein 1B (Fragment) OS=Rattus norvegicus GN=Baz1b PE=4 SV=1
Alpha-synuclein OS=Rattus norvegicus GN=Snca PE=1 SV=1 - [SYUA_RAT]
Oxysterol-binding protein OS=Rattus norvegicus GN=Osbpl11 PE=1 SV=1 - [B5DF74_RAT]
Protein Numa1 OS=Rattus norvegicus GN=Numa1 PE=1 SV=2 - [F1LW91_RAT]
Monomeric GTP-binding protein (Fragment) OS=Rattus norvegicus GN=Rab31 PE=2 SV=1 - [P97632_RAT]
Protein Napg OS=Rattus norvegicus GN=Napg PE=1 SV=1 - [D4A0E2_RAT]
Synaptopodin OS=Rattus norvegicus GN=Synpo PE=2 SV=1 - [B1VKB4_RAT]
60S ribosomal protein L22 OS=Rattus norvegicus GN=Rpl22 PE=2 SV=2 - [RL22_RAT]
Protein Ptpn12 OS=Rattus norvegicus GN=Ptpn12 PE=1 SV=1 - [G3V7Q4_RAT]
Protein Tmem25 OS=Rattus norvegicus GN=Tmem25 PE=1 SV=1 - [D3Z9V8_RAT]
ADP-ribosylation factor 4 OS=Rattus norvegicus GN=Arf4 PE=2 SV=2 - [ARF4_RAT]
Eukaryotic translation initiation factor 4 gamma, 3 (Predicted), isoform CRA_a OS=Rattus norvegicus GN=Eif4g3 PE
Heterogeneous nuclear ribonucleoprotein D-like OS=Rattus norvegicus GN=Hnrnpdl PE=1 SV=1 - [HNRDL_RAT]
Activity and neurotransmitter-induced early gene protein 4 OS=Rattus norvegicus GN=Dclk1 PE=1 SV=1 - [Q9WVP
Arf-GAP with SH3 domain, ANK repeat and PH domain-containing protein 1 OS=Rattus norvegicus GN=Asap1 PE=
Potassium voltage-gated channel subfamily A member 4 OS=Rattus norvegicus GN=Kcna4 PE=1 SV=1 - [G3V6L7_
RELT-like protein 2 OS=Rattus norvegicus GN=Rell2 PE=2 SV=1 - [RELL2_RAT]
Protein Vps13d OS=Rattus norvegicus GN=Vps13d PE=1 SV=1 - [A0A0G2JYD4_RAT]
DEAH (Asp-Glu-Ala-His) box polypeptide 9 (Predicted) OS=Rattus norvegicus GN=Dhx9 PE=1 SV=1 - [D4A9D6_RA
Echinoderm microtubule-associated protein-like 1 OS=Rattus norvegicus GN=Eml1 PE=1 SV=2 - [M0RCT5_RAT]
Hsp90 co-chaperone Cdc37 OS=Rattus norvegicus GN=Cdc37 PE=1 SV=1 - [A0A0G2K3C1_RAT]
Poly (ADP-ribose) glycohydrolase, isoform CRA_b OS=Rattus norvegicus GN=Parg PE=1 SV=1 - [G3V8M8_RAT]
Protein Tbc1d5 OS=Rattus norvegicus GN=Tbc1d5 PE=1 SV=1 - [A0A0G2K712_RAT]
Prkag2 protein (Fragment) OS=Rattus norvegicus GN=Prkag2 PE=2 SV=1 - [Q4QRB9_RAT]
Protein Fbxo41 OS=Rattus norvegicus GN=Fbxo41 PE=1 SV=1 - [A0A0G2K9A9_RAT]
Dynein light chain 1, cytoplasmic OS=Rattus norvegicus GN=Dynll1 PE=1 SV=1 - [DYL1_RAT]
Sodium channel protein type 8 subunit alpha OS=Rattus norvegicus GN=Scn8a PE=1 SV=1 - [SCN8A_RAT]
Regulating synaptic membrane exocytosis protein 2 OS=Rattus norvegicus GN=Rims2 PE=1 SV=2 - [A0A096P6M3
DnaJ homolog subfamily A member 1 OS=Rattus norvegicus GN=Dnaja1 PE=1 SV=1 - [DNJA1_RAT]
Protein Ankib1 OS=Rattus norvegicus GN=Ankib1 PE=1 SV=1 - [A0A0G2JTL7_RAT]
Acidic leucine-rich nuclear phosphoprotein 32 family member B OS=Rattus norvegicus GN=Anp32b PE=1 SV=1 - [A
Sterile alpha motif domain-containing protein 14 OS=Rattus norvegicus GN=Samd14 PE=1 SV=3 - [F1LN57_RAT]
Protein Ppp6r2 OS=Rattus norvegicus GN=Ppp6r2 PE=1 SV=1 - [A0A0G2JV49_RAT]
Amino acid transporter OS=Rattus norvegicus GN=ratASCT1 PE=2 SV=1 - [A0A0F7R5I4_RAT]
Fasciculation and elongation protein zeta-1 OS=Rattus norvegicus GN=Fez1 PE=1 SV=1 - [FEZ1_RAT]
Uncharacterized protein OS=Rattus norvegicus PE=4 SV=1 - [D4A269_RAT]
Calcium channel, voltage-dependent, beta 1 subunit, isoform CRA_a OS=Rattus norvegicus GN=Cacnb1 PE=1 SV=
Melanoma antigen, family D, 2 OS=Rattus norvegicus GN=Maged2 PE=1 SV=1 - [Q3B7U1_RAT]
Phosphatidylinositol 4-kinase alpha OS=Rattus norvegicus GN=Pi4ka PE=1 SV=1 - [A0A140TAJ5_RAT]

Information Classification: General

Calmodulin-like protein 3 OS=Rattus norvegicus GN=Calml3 PE=2 SV=1 - [CALL3_RAT]
Clavesin-1 OS=Rattus norvegicus GN=Clvs1 PE=4 SV=3 - [F1LRF3_RAT]
Chloride channel, nucleotide-sensitive, 1A OS=Rattus norvegicus GN=Clns1a PE=1 SV=1 - [Q6P9X1_RAT]
Protein LOC100911356 OS=Rattus norvegicus GN=Pex19 PE=1 SV=1 - [A0A0G2JY69_RAT]
Protein Rragc OS=Rattus norvegicus GN=Rragc PE=1 SV=1 - [Q0D2L6_RAT]
Guanine nucleotide-binding protein subunit gamma OS=Rattus norvegicus GN=Gng12 PE=1 SV=1 - [G3V6P8_RAT
BLOC-1-related complex subunit 6 OS=Rattus norvegicus GN=Borcs6 PE=2 SV=2 - [BORC6_RAT]
Stathmin-2 OS=Rattus norvegicus GN=Stmn2 PE=1 SV=2 - [STMN2_RAT]
Tumor protein D54 OS=Rattus norvegicus GN=Tpd52l2 PE=1 SV=1 - [TPD54_RAT]
Diacylglycerol kinase beta OS=Rattus norvegicus GN=Dgkb PE=1 SV=1 - [DGKB_RAT]
NOL1/NOP2/Sun domain family, member 2 (Predicted) OS=Rattus norvegicus GN=Nsun2 PE=1 SV=1 - [D4A3S8_
Heterogeneous nuclear ribonucleoprotein C OS=Rattus norvegicus GN=Hnrnpc PE=1 SV=2 - [HNRPC_RAT]
Protein R3hdm2 OS=Rattus norvegicus GN=R3hdm2 PE=1 SV=1 - [G3V9G7_RAT]
Protein Ctnna3 OS=Rattus norvegicus GN=Ctnna3 PE=1 SV=3 - [F1M4I1_RAT]
ADP-ribosylation factor GTPase activating protein 1 brain isoform OS=Rattus norvegicus GN=Arfgap1 PE=1 SV=1 -
Protein Daam2 OS=Rattus norvegicus GN=Daam2 PE=1 SV=1 - [A0A0G2K988_RAT]
Histone H2A OS=Rattus norvegicus GN=Hist2h2ac PE=3 SV=2 - [D4ACV3_RAT]
Serine/threonine-protein phosphatase 1 regulatory subunit 10 OS=Rattus norvegicus GN=Ppp1r10 PE=1 SV=1 - [A
Protein Ahnak OS=Rattus norvegicus GN=Ahnak PE=1 SV=1 - [A0A0G2JU96_RAT]
Protein deglycase DJ-1 OS=Rattus norvegicus GN=Park7 PE=1 SV=1 - [PARK7_RAT]
Protein Zmynd8 OS=Rattus norvegicus GN=Zmynd8 PE=1 SV=1 - [A0A0G2K9F7_RAT]
Protein Esyt2 OS=Rattus norvegicus GN=Esyt2 PE=1 SV=3 - [D3ZJ32_RAT]
Actin-related protein 3 OS=Rattus norvegicus GN=Actr3 PE=1 SV=1 - [ARP3_RAT]
Nuclear pore complex protein Nup93 OS=Rattus norvegicus GN=Nup93 PE=1 SV=1 - [NUP93_RAT]
Phosphoglycerate mutase 1 OS=Rattus norvegicus GN=Pgam1 PE=1 SV=4 - [PGAM1_RAT]
S-phase kinase-associated protein 1 OS=Rattus norvegicus GN=Skp1 PE=1 SV=3 - [SKP1_RAT]
Sodium-dependent phosphate transporter 1 OS=Rattus norvegicus GN=Slc20a1 PE=1 SV=1 - [S20A1_RAT]
Myeloma overexpressed 2 OS=Rattus norvegicus GN=Cops9 PE=1 SV=1 - [B5DFN0_RAT]
Ppm1b protein OS=Rattus norvegicus GN=Ppm1b PE=1 SV=1 - [Q99ND8_RAT]
GTP-binding protein 1 OS=Rattus norvegicus GN=Gtpbp1 PE=1 SV=1 - [GTPB1_RAT]
Nuclear pore glycoprotein p62 OS=Rattus norvegicus GN=Nup62 PE=1 SV=1 - [NUP62_RAT]
Protein Hid1 OS=Rattus norvegicus GN=Hid1 PE=1 SV=2 - [D4A0C3_RAT]
Putative uncharacterized protein RGD1303117 OS=Rattus norvegicus GN=Fam98c PE=2 SV=1 - [Q6AYE6_RAT]
Adenylyl cyclase-associated protein 2 OS=Rattus norvegicus GN=Cap2 PE=1 SV=1 - [CAP2_RAT]
Protein Ablim1 (Fragment) OS=Rattus norvegicus GN=Ablim1 PE=1 SV=3 - [F1LWK7_RAT]
Protein Cstf2 OS=Rattus norvegicus GN=Cstf2 PE=1 SV=1 - [A0A0G2JWP1_RAT]
Rab-3A-interacting protein OS=Rattus norvegicus GN=Rab3ip PE=1 SV=1 - [A0A0G2K1B4_RAT]
Protein Zzef1 OS=Rattus norvegicus GN=Zzef1 PE=1 SV=2 - [D3ZG78_RAT]
5'-AMP-activated protein kinase subunit beta-1 OS=Rattus norvegicus GN=Prkab1 PE=1 SV=4 - [AAKB1_RAT]
Cytoplasmic dynein 1 intermediate chain 2 OS=Rattus norvegicus GN=Dync1i2 PE=1 SV=1 - [D3ZU74_RAT]
DEAD (Asp-Glu-Ala-Asp) box polypeptide 42 (Predicted) OS=Rattus norvegicus GN=Ddx42 PE=1 SV=1 - [D4A031_
Protein Utrn OS=Rattus norvegicus GN=Utrn PE=1 SV=3 - [G3V7L1_RAT]
Protein Pwp1 OS=Rattus norvegicus GN=Pwp1 PE=1 SV=1 - [D4A1H8_RAT]
Calmodulin-regulated spectrin-associated protein 1 OS=Rattus norvegicus GN=Camsap1 PE=1 SV=1 - [A0A0G2K5
Endoplasmin OS=Rattus norvegicus GN=Hsp90b1 PE=1 SV=1 - [A0A0G2K4I4_RAT]
Protein Gigyf2 (Fragment) OS=Rattus norvegicus GN=Gigyf2 PE=1 SV=1 - [A0A096MKC0_RAT]
Protein Actbl2 OS=Rattus norvegicus GN=Actbl2 PE=1 SV=1 - [D3ZRN3_RAT]
Protein Dennd1a OS=Rattus norvegicus GN=Dennd1a PE=1 SV=2 - [F1M241_RAT]
ATPase Asna1 OS=Rattus norvegicus GN=Asna1 PE=1 SV=1 - [G3V9T7_RAT]
Protein Btbd8 OS=Rattus norvegicus GN=Btbd8 PE=1 SV=2 - [D4A0X3_RAT]
Protein Vps13c OS=Rattus norvegicus GN=Vps13c PE=1 SV=2 - [D4A4K4_RAT]
CaM kinase-like vesicle-associated protein OS=Rattus norvegicus GN=Camkv PE=1 SV=1 - [CAMKV_RAT]
Voltage-dependent calcium channel subunit alpha-2/delta-1 OS=Rattus norvegicus GN=Cacna2d1 PE=1 SV=1 - [A
Poliovirus receptor-related 1 OS=Rattus norvegicus GN=Nectin1 PE=1 SV=2 - [F1LNP8_RAT]
Calcium-activated potassium channel subunit alpha-1 OS=Rattus norvegicus GN=Kcnma1 PE=1 SV=1 - [A0A0G2K

E3 ubiquitin-protein ligase NEDD4 OS=Rattus norvegicus GN=Nedd4 PE=1 SV=1 - [A0A0G2K0B4_RAT]
Plasminogen activator inhibitor 1 RNA-binding protein OS=Rattus norvegicus GN=Serbp1 PE=1 SV=2 - [PAIRB_RA
Actin filament-associated protein 1 OS=Rattus norvegicus GN=Afap1 PE=1 SV=1 - [G3V6Z3_RAT]
Protein Rprd2 OS=Rattus norvegicus GN=Rprd2 PE=1 SV=1 - [A0A0G2JTD1_RAT]
Glycerol-3-phosphate acyltransferase 1, mitochondrial OS=Rattus norvegicus GN=Gpam PE=1 SV=2 - [G3V9J8_R
Vacuolar protein sorting-associated protein 4A OS=Rattus norvegicus GN=Vps4a PE=1 SV=1 - [VPS4A_RAT]
Isocitrate dehydrogenase [NAD] subunit alpha, mitochondrial OS=Rattus norvegicus GN=Idh3a PE=1 SV=1 - [IDH3
Phosphoserine aminotransferase OS=Rattus norvegicus GN=Psat1 PE=1 SV=1 - [A0A0G2K931_RAT]
Rho GTPase-activating protein 39 OS=Rattus norvegicus GN=Arhgap39 PE=1 SV=1 - [F1LR18_RAT]
Calcium-binding protein P23K beta (Fragment) OS=Rattus norvegicus GN=Hpcal4 PE=2 SV=1 - [Q9QWR2_RAT]
Protein Camsap2 OS=Rattus norvegicus GN=Camsap2 PE=1 SV=1 - [A0A0G2K7K9_RAT]
Protein Spire1 OS=Rattus norvegicus GN=Spire1 PE=1 SV=1 - [D3ZEX7_RAT]
Protein Arhgap1 OS=Rattus norvegicus GN=Arhgap1 PE=1 SV=1 - [D4A6C5_RAT]
Protein Ablim1 OS=Rattus norvegicus GN=Ablim1 PE=1 SV=1 - [A0A0G2JW01_RAT]
Large proline-rich protein BAG6 OS=Rattus norvegicus GN=Bag6 PE=1 SV=1 - [A0A0G2K7C1_RAT]
Protein TANC1 OS=Rattus norvegicus GN=Tanc1 PE=1 SV=3 - [G3V917_RAT]
Protein Ncoa7 OS=Rattus norvegicus GN=Ncoa7 PE=1 SV=3 - [F1LWN1_RAT]
Protein IMPACT OS=Rattus norvegicus GN=Impact PE=1 SV=1 - [IMPCT_RAT]
NADH dehydrogenase [ubiquinone] iron-sulfur protein 2, mitochondrial OS=Rattus norvegicus GN=Ndufs2 PE=1 SV
E3 UFM1-protein ligase 1 OS=Rattus norvegicus GN=Ufl1 PE=1 SV=1 - [A0A0G2K266_RAT]
Synaptotagmin-1 OS=Rattus norvegicus GN=Syt1 PE=1 SV=3 - [SYT1_RAT]
Abl interactor 1 OS=Rattus norvegicus GN=Abi1 PE=1 SV=3 - [ABI1_RAT]
Malic enzyme OS=Rattus norvegicus GN=Me3 PE=3 SV=1 - [A0A0G2K4C6_RAT]
Protein Anln OS=Rattus norvegicus GN=Anln PE=1 SV=2 - [M0RDG0_RAT]
AKT1 substrate 1 (Proline-rich) (Predicted), isoform CRA_d OS=Rattus norvegicus GN=Akt1s1 PE=1 SV=1 - [D3ZH
Protein Fam63b OS=Rattus norvegicus GN=Fam63b PE=1 SV=2 - [D3ZWA1_RAT]
Myeloid leukemia factor 2 (Predicted), isoform CRA_a OS=Rattus norvegicus GN=Mlf2 PE=1 SV=1 - [D3ZPN3_RA
Probable ATP-dependent RNA helicase DDX46 OS=Rattus norvegicus GN=Ddx46 PE=1 SV=1 - [DDX46_RAT]
Synaptosomal-associated protein OS=Rattus norvegicus GN=Snap23 PE=1 SV=2 - [M0R4V3_RAT]
Ubiquitin-like modifier-activating enzyme 5 OS=Rattus norvegicus GN=Uba5 PE=1 SV=1 - [UBA5_RAT]
Myosin phosphatase Rho-interacting protein OS=Rattus norvegicus GN=Mprip PE=1 SV=1 - [A0A140TA95_RAT]
Visinin-like protein 1 OS=Rattus norvegicus GN=Vsnl1 PE=1 SV=2 - [VISL1_RAT]
Solute carrier family 25 member 46 OS=Rattus norvegicus GN=Slc25a46 PE=1 SV=2 - [S2546_RAT]
Ryanodine receptor 2 OS=Rattus norvegicus GN=Ryr2 PE=1 SV=3 - [F1LRZ1_RAT]
Glutamine synthetase OS=Rattus norvegicus GN=Glul PE=1 SV=3 - [GLNA_RAT]
Protein Tacc1 OS=Rattus norvegicus GN=Tacc1 PE=1 SV=1 - [D4A927_RAT]
Potassium channel tetramerisation domain containing 8 (Predicted) OS=Rattus norvegicus GN=Kctd8 PE=2 SV=1 -
Annexin A5 OS=Rattus norvegicus GN=Anxa5 PE=1 SV=3 - [ANXA5_RAT]
Coiled-coil domain containing 12 (Predicted), isoform CRA_a OS=Rattus norvegicus GN=Ccdc12 PE=1 SV=1 - [D4
Disks large-associated protein 3 OS=Rattus norvegicus GN=Dlgap3 PE=1 SV=2 - [G3V7T8_RAT]
Ac1075 (Fragment) OS=Rattus norvegicus GN=Rab10 PE=2 SV=1 - [Q7TQ88_RAT]
LRRGT00098 OS=Rattus norvegicus GN=Arhgap5 PE=1 SV=1 - [Q6TUE6_RAT]
ELAV-like protein 4 OS=Rattus norvegicus GN=Elavl4 PE=1 SV=1 - [ELAV4_RAT]
Integral membrane protein 2C OS=Rattus norvegicus GN=Itm2c PE=1 SV=1 - [ITM2C_RAT]
Protein Ranbp2 OS=Rattus norvegicus GN=Ranbp2 PE=1 SV=2 - [D4A054_RAT]
Protein Tmf1 OS=Rattus norvegicus GN=Tmf1 PE=1 SV=1 - [A0A0G2JYB3_RAT]
Bone marrow stromal cell-derived ubiquitin-like protein OS=Rattus norvegicus GN=Ubl7 PE=1 SV=1 - [Q6AY24_RA
Protein Ppp1r3g OS=Rattus norvegicus GN=Ppp1r3g PE=1 SV=1 - [M0R4A0_RAT]
Stub1 protein OS=Rattus norvegicus GN=Stub1 PE=2 SV=1 - [I6L9H8_RAT]
Protein LOC100362814 OS=Rattus norvegicus GN=LOC100362814 PE=1 SV=3 - [F1LXC7_RAT]
Neuronal-specific septin-3 OS=Rattus norvegicus GN=Sept3 PE=3 SV=1 - [F1LMH0_RAT]
Guanine deaminase OS=Rattus norvegicus GN=Gda PE=1 SV=1 - [Q9JKB7_RAT]
NFI-A4 (Fragment) OS=Rattus norvegicus GN=NFI-A PE=2 SV=1 - [Q63785_RAT]
ATG4 autophagy related 4 homolog B (S. cerevisiae) OS=Rattus norvegicus GN=Atg4b PE=2 SV=1 - [Q4KM36_RA
Protein Syngr3 OS=Rattus norvegicus GN=Syngr3 PE=1 SV=1 - [D4ABK1_RAT]

Rics protein (Fragment) OS=Rattus norvegicus GN=Rics PE=2 SV=1 - [B5DEI5_RAT]
Vesicle-trafficking protein SEC22b OS=Rattus norvegicus GN=Sec22b PE=1 SV=3 - [SC22B_RAT]
Protein Mxra7 OS=Rattus norvegicus GN=Mxra7 PE=4 SV=2 - [F1M1U0_RAT]
Large neutral amino acids transporter small subunit 2 OS=Rattus norvegicus GN=Slc7a8 PE=1 SV=1 - [LAT2_RAT]
Protein Pgm5 OS=Rattus norvegicus GN=Pgm5 PE=1 SV=3 - [D3ZVR9_RAT]
Protein LOC684828 OS=Rattus norvegicus GN=LOC684828 PE=1 SV=2 - [M0R7B4_RAT]
SH3 domain-containing kinase-binding protein 1 OS=Rattus norvegicus GN=Sh3kbp1 PE=1 SV=2 - [M0RBZ7_RAT
Ragulator complex protein LAMTOR1 OS=Rattus norvegicus GN=Lamtor1 PE=1 SV=1 - [LTOR1_RAT]
ATPase, H+ transporting, V1 subunit D, isoform CRA_c OS=Rattus norvegicus GN=Atp6v1d PE=1 SV=1 - [Q6P503
Ab2-292 OS=Rattus norvegicus GN=Sec62 PE=1 SV=1 - [Q7TP42_RAT]
Glutamate cysteine ligase modifier subunit (Fragment) OS=Rattus norvegicus GN=Gcln PE=2 SV=1 - [Q2VC85_RA
Protein Trio OS=Rattus norvegicus GN=Trio PE=1 SV=3 - [F1M0Z1_RAT]
Hexokinase-1 OS=Rattus norvegicus GN=Hk1 PE=1 SV=2 - [M0RAQ6_RAT]
COP9 signalosome complex subunit 8 OS=Rattus norvegicus GN=Cops8 PE=2 SV=1 - [CSN8_RAT]
Protein Ipo5 OS=Rattus norvegicus GN=Ipo5 PE=1 SV=1 - [D4A781_RAT]
Hect (Homologous to the E6-AP (UBE3A) carboxyl terminus) domain and RCC1 (CHC1)-like domain (RLD) 2 (Predi
Inositol monophosphate (Fragment) OS=Rattus norvegicus GN=Impa1 PE=2 SV=1 - [P97618_RAT]
Protein Pikfyve OS=Rattus norvegicus GN=Pikfyve PE=1 SV=2 - [D3ZYT8_RAT]
Gamma-glutamyltransferase 7 OS=Rattus norvegicus GN=Ggt7 PE=1 SV=2 - [GGT7_RAT]
Protein Tacc2 OS=Rattus norvegicus GN=Tacc2 PE=1 SV=1 - [X4YHC6_RAT]
C-type lectin domain family 2 member L OS=Rattus norvegicus GN=Clec2l PE=1 SV=2 - [CLC2L_RAT]
Putative regulation protein GS3 OS=Rattus norvegicus GN=Dnajc21 PE=2 SV=1 - [Q8R497_RAT]
Protein Mtch2 OS=Rattus norvegicus GN=Mtch2 PE=1 SV=1 - [A0A0G2K7P7_RAT]
Protein LOC100909521 OS=Rattus norvegicus GN=LOC100909521 PE=4 SV=1 - [M0RDR2_RAT]
Protein Amer2 OS=Rattus norvegicus GN=Amer2 PE=1 SV=1 - [A0A0G2JWK6_RAT]
Proteasome subunit alpha type-2 OS=Rattus norvegicus GN=Psma2 PE=1 SV=3 - [PSA2_RAT]
Protein Avl9 OS=Rattus norvegicus GN=Avl9 PE=1 SV=1 - [D3ZVU6_RAT]
Protein prune homolog 2 OS=Rattus norvegicus GN=Prune2 PE=1 SV=1 - [PRUN2_RAT]
Protein Shroom2 OS=Rattus norvegicus GN=Shroom2 PE=1 SV=1 - [SHRM2_RAT]
Histone deacetylase 1 OS=Rattus norvegicus GN=Hdac1 PE=1 SV=1 - [HDAC1_RAT]
Endophilin-B2 OS=Rattus norvegicus GN=Sh3glb2 PE=1 SV=3 - [D4A7V1_RAT]
Prickle-like 2 (Drosophila) (Predicted) OS=Rattus norvegicus GN=Prickle2 PE=1 SV=2 - [F1M0J7_RAT]
Ribonuclease inhibitor OS=Rattus norvegicus GN=Rnh1 PE=1 SV=1 - [E2RUH2_RAT]
Coiled-coil domain containing 25 (Predicted) OS=Rattus norvegicus GN=Ccdc25 PE=1 SV=1 - [D4AAU6_RAT]
Neuronal membrane glycoprotein M6-a OS=Rattus norvegicus GN=Gpm6a PE=4 SV=1 - [A0A1B0GWN8_RAT]
Protein Dact3 OS=Rattus norvegicus GN=Dact3 PE=1 SV=1 - [M0R4J7_RAT]
Guanine nucleotide-binding protein G(I)/G(S)/G(T) subunit beta-1 OS=Rattus norvegicus GN=Gnb1 PE=1 SV=4 - [G
Syntaxin-6 OS=Rattus norvegicus GN=Stx6 PE=1 SV=1 - [STX6_RAT]
Proline-rich transmembrane protein 2 OS=Rattus norvegicus GN=Prrt2 PE=1 SV=1 - [PRRT2_RAT]
CD9 antigen OS=Rattus norvegicus GN=Cd9 PE=1 SV=2 - [CD9_RAT]
Atlastin-1 OS=Rattus norvegicus GN=Atl1 PE=1 SV=1 - [ATLA1_RAT]
WD repeat-containing protein 44 OS=Rattus norvegicus GN=Wdr44 PE=1 SV=1 - [A0A0G2JX77_RAT]
Diacylglycerol kinase OS=Rattus norvegicus GN=Dgkq PE=1 SV=1 - [D3ZEY4_RAT]
Protein Soga3 OS=Rattus norvegicus GN=Soga3 PE=1 SV=1 - [D4A0A1_RAT]
Protein Mycbp2 OS=Rattus norvegicus GN=Mycbp2 PE=1 SV=3 - [D4A2D3_RAT]
Dystrophin (Fragment) OS=Rattus norvegicus GN=Dmd PE=2 SV=2 - [Q9Z147_RAT]
Type 1 phosphatidylinositol 4,5-bisphosphate 4-phosphatase OS=Rattus norvegicus GN=Tmem55b PE=1 SV=1 - [T
Cyclin-dependent kinase 11B OS=Rattus norvegicus GN=Cdk11b PE=1 SV=3 - [D4A3G2_RAT]
Nucleolar and coiled-body phosphoprotein 1 OS=Rattus norvegicus GN=Nolc1 PE=1 SV=1 - [F1LPS3_RAT]
Sortilin OS=Rattus norvegicus GN=Sort1 PE=1 SV=3 - [SORT_RAT]
Protein Slirp OS=Rattus norvegicus GN=Slirp PE=1 SV=1 - [D4A4W6_RAT]
Protein Lrba OS=Rattus norvegicus GN=Lrba PE=1 SV=1 - [A0A0G2JYI0_RAT]
Peroxiredoxin-6 OS=Rattus norvegicus GN=Prdx6 PE=1 SV=3 - [PRDX6_RAT]
T-complex protein 1 subunit beta OS=Rattus norvegicus GN=Cct2 PE=1 SV=3 - [TCPB_RAT]
Heterochromatin protein 1-binding protein 3 (Fragment) OS=Rattus norvegicus GN=Hp1bp3 PE=1 SV=1 - [A0A096

Serine/threonine-protein kinase TAO1 OS=Rattus norvegicus GN=Taok1 PE=1 SV=3 - [F1M9G6_RAT]
PiggyBac transposable element derived 5 (Predicted) OS=Rattus norvegicus GN=Pgbd5 PE=1 SV=1 - [D3ZSZ4_RA
Glycerol-3-phosphate dehydrogenase [NAD(+)], cytoplasmic OS=Rattus norvegicus GN=Gpd1 PE=1 SV=4 - [GPDA
EH domain-containing protein 3 OS=Rattus norvegicus GN=Ehd3 PE=1 SV=2 - [EHD3_RAT]
Tropomyosin 1, alpha, isoform CRA_p OS=Rattus norvegicus GN=Tpm1 PE=1 SV=1 - [A0A0G2JSQ4_RAT]
Interferon regulatory factor 2 binding protein 1 (Predicted) OS=Rattus norvegicus GN=Irf2bp1 PE=1 SV=1 - [D4AAZ
Loss of heterozygosity, 12, chromosomal region 1 homolog (Human) OS=Rattus norvegicus GN=Borcs5 PE=1 SV=
Mon1a protein OS=Rattus norvegicus GN=Mon1a PE=1 SV=1 - [B1WC06_RAT]
Protein Vat1l OS=Rattus norvegicus GN=Vat1l PE=1 SV=2 - [M0R3N4_RAT]
Protein Uhrf1bp1 OS=Rattus norvegicus GN=Uhrf1bp1 PE=1 SV=3 - [D3ZMR2_RAT]
Transaldolase OS=Rattus norvegicus GN=Taldo1 PE=1 SV=2 - [TALDO_RAT]
Metabotropic glutamate receptor 7 OS=Rattus norvegicus GN=Grm7 PE=3 SV=2 - [F1LZS5_RAT]
Pyridoxine-5'-phosphate oxidase OS=Rattus norvegicus GN=Pnpo PE=1 SV=1 - [PNPO_RAT]
Brain-enriched guanylate kinase-associated protein OS=Rattus norvegicus GN=Begain PE=1 SV=1 - [A0A0G2K0E5
Splicing factor, arginine/serine-rich 15 OS=Rattus norvegicus GN=Scaf4 PE=1 SV=3 - [F1M994_RAT]
GATA zinc finger domain containing 2B OS=Rattus norvegicus GN=Gatad2b PE=1 SV=1 - [Q4V8E1_RAT]
Oxysterol-binding protein OS=Rattus norvegicus GN=Osbp2 PE=1 SV=2 - [D3ZHG4_RAT]
Putative uncharacterized protein OS=Rattus norvegicus GN=Nol3 PE=2 SV=1 - [Q62882_RAT]
4F2 cell-surface antigen heavy chain OS=Rattus norvegicus GN=Slc3a2 PE=1 SV=1 - [4F2_RAT]
Vesicular glutamate transporter 1 OS=Rattus norvegicus GN=Slc17a7 PE=1 SV=1 - [VGLU1_RAT]
CHMP family, member 7 (Predicted), isoform CRA_a OS=Rattus norvegicus GN=Chmp7 PE=1 SV=1 - [D4A7H9_R
Protein phosphatase methylesterase 1 OS=Rattus norvegicus GN=Ppme1 PE=1 SV=2 - [PPME1_RAT]
Dusp3 protein OS=Rattus norvegicus GN=Dusp3 PE=2 SV=1 - [B5DFF7_RAT]
Protein Srpra OS=Rattus norvegicus GN=Srpra PE=1 SV=1 - [Q3KRC3_RAT]
Protein FAM131B OS=Rattus norvegicus GN=Fam131b PE=1 SV=1 - [F131B_RAT]
Fumarylacetoacetase OS=Rattus norvegicus GN=Fah PE=1 SV=1 - [FAAA_RAT]
Protein Sf1 OS=Rattus norvegicus GN=Sf1 PE=1 SV=2 - [F1LSC3_RAT]
Adenosylhomocysteinase OS=Rattus norvegicus GN=Ahcyl2 PE=1 SV=3 - [D3ZWL6_RAT]
Peptidyl-prolyl cis-trans isomerase G OS=Rattus norvegicus GN=Ppig PE=1 SV=1 - [G3V6Y9_RAT]
Protein Erbin OS=Rattus norvegicus GN=Erbin PE=1 SV=2 - [M0R9T2_RAT]
DEAD (Asp-Glu-Ala-Asp) box polypeptide 19a OS=Rattus norvegicus GN=Ddx19a PE=1 SV=1 - [Q68FX3_RAT]
Osteoclast-stimulating factor 1 OS=Rattus norvegicus GN=Ostf1 PE=1 SV=1 - [OSTF1_RAT]
Protein Tbc1d10b OS=Rattus norvegicus GN=Tbc1d10b PE=1 SV=1 - [D3ZSY8_RAT]
E3 ubiquitin-protein ligase TRIM9 OS=Rattus norvegicus GN=Trim9 PE=1 SV=1 - [TRIM9_RAT]
Proteasome subunit alpha type-5 OS=Rattus norvegicus GN=Psma5 PE=1 SV=1 - [PSA5_RAT]
Cyth1 protein OS=Rattus norvegicus GN=Cyth1 PE=1 SV=1 - [B2GUV0_RAT]
Protein Usp8 OS=Rattus norvegicus GN=Usp8 PE=1 SV=2 - [D3ZN39_RAT]
Protein Usp20 OS=Rattus norvegicus GN=Usp20 PE=1 SV=1 - [D3ZLQ8_RAT]
NADH dehydrogenase [ubiquinone] flavoprotein 2, mitochondrial OS=Rattus norvegicus GN=Ndufv2 PE=1 SV=2 - [
Phosphatidylinositol 5-phosphate 4-kinase type-2 beta OS=Rattus norvegicus GN=Pip4k2b PE=1 SV=1 - [PI42B_R
ATP synthase subunit gamma, mitochondrial OS=Rattus norvegicus GN=Atp5c1 PE=1 SV=2 - [ATPG_RAT]
Pyruvate dehydrogenase E1 component subunit alpha OS=Rattus norvegicus GN=Pdha1 PE=1 SV=2 - [F7FKI5_R
Transcription elongation factor A protein 1 OS=Rattus norvegicus GN=Tcea1 PE=1 SV=1 - [TCEA1_RAT]
Mitogen activated protein kinase kinase kinase 4 (Predicted) OS=Rattus norvegicus GN=Map3k4 PE=1 SV=1 - [D3
F-box/LRR-repeat protein 20 OS=Rattus norvegicus GN=Fbxl20 PE=1 SV=1 - [A0A0H2UI14_RAT]
Protein Myh14 OS=Rattus norvegicus GN=Myh14 PE=1 SV=1 - [F1LNF0_RAT]
Protein Prrc2b OS=Rattus norvegicus GN=Prrc2b PE=1 SV=2 - [D3ZY45_RAT]
RUN domain-containing protein 3B OS=Rattus norvegicus GN=Rundc3b PE=1 SV=1 - [RUN3B_RAT]
Protein Efr3a OS=Rattus norvegicus GN=Efr3a PE=1 SV=1 - [D4ADS9_RAT]
Prohibitin OS=Rattus norvegicus GN=Phb PE=1 SV=1 - [PHB_RAT]
Guanine nucleotide-binding protein subunit gamma OS=Rattus norvegicus PE=3 SV=1 - [F2Z3T8_RAT]
Papillary renal cell carcinoma (Translocation-associated) (Predicted) OS=Rattus norvegicus GN=Prcc PE=1 SV=1 -
Secernin-1 OS=Rattus norvegicus GN=Scrn1 PE=1 SV=1 - [SCRN1_RAT]
Protein Rb1cc1 OS=Rattus norvegicus GN=Rb1cc1 PE=1 SV=1 - [D3ZHK4_RAT]
Serine/threonine-protein kinase PAK 2 OS=Rattus norvegicus GN=Pak2 PE=1 SV=1 - [PAK2_RAT]

Menin OS=Rattus norvegicus GN=Men1 PE=1 SV=1 - [MEN1_RAT]
Protein Mical3 OS=Rattus norvegicus GN=Mical3 PE=1 SV=2 - [D3ZGN7_RAT]
Glutamate carboxypeptidase 2 OS=Rattus norvegicus GN=Folh1 PE=1 SV=1 - [FOLH1_RAT]
ATPase, H+ transporting, lysosomal 38kDa, V0 subunit d1 OS=Rattus norvegicus GN=Atp6v0d1 PE=1 SV=1 - [Q5M
Copine 4 protein OS=Rattus norvegicus GN=Cpne4 PE=2 SV=1 - [H1UBM8_RAT]
Protein Prpf40b OS=Rattus norvegicus GN=Prpf40b PE=1 SV=2 - [F1LTJ8_RAT]
Protein Tmem9b OS=Rattus norvegicus GN=Tmem9b PE=1 SV=1 - [D3ZW49_RAT]
Protein Tango6 OS=Rattus norvegicus GN=Tango6 PE=1 SV=3 - [D4A9F1_RAT]
Aspartate aminotransferase, cytoplasmic OS=Rattus norvegicus GN=Got1 PE=1 SV=3 - [AATC_RAT]
Protein Tmem63c OS=Rattus norvegicus GN=Tmem63c PE=1 SV=1 - [D3ZNF5_RAT]
Histone H2A OS=Rattus norvegicus GN=Hist2h2ab PE=3 SV=1 - [D3ZWE0_RAT]
Protein Ccnyl1 OS=Rattus norvegicus GN=Ccnyl1 PE=1 SV=3 - [F1M4U0_RAT]
Leucine-rich repeat flightless-interacting protein 2 OS=Rattus norvegicus GN=Lrrfip2 PE=1 SV=1 - [LRRF2_RAT]
Guanine nucleotide-binding protein G(I)/G(S)/G(T) subunit beta-2 OS=Rattus norvegicus GN=Gnb2 PE=1 SV=4 - [G
Overexpressed in colon carcinoma 1 protein homolog OS=Rattus norvegicus PE=3 SV=1 - [OCC1_RAT]
Galanin peptides OS=Rattus norvegicus GN=Gal PE=1 SV=1 - [GALA_RAT]
Protein Arhgef17 OS=Rattus norvegicus GN=Arhgef17 PE=1 SV=1 - [A0A0G2JXT9_RAT]
Protein LOC100912106 OS=Rattus norvegicus GN=Pdcd5 PE=1 SV=1 - [D4ADF5_RAT]
Guanine nucleotide-binding protein G(s) subunit alpha isoforms short OS=Rattus norvegicus GN=Gnas PE=1 SV=1
Oligodendrocytic myelin paranodal and inner loop protein OS=Rattus norvegicus GN=Opalin PE=2 SV=1 - [Q56A26_
Protein Ten1 OS=Rattus norvegicus GN=Ten1 PE=4 SV=1 - [A0A0G2K7H0_RAT]
FUN14 domain-containing protein 1 OS=Rattus norvegicus GN=Fundc1 PE=1 SV=1 - [FUND1_RAT]
ATP-dependent (S)-NAD(P)H-hydrate dehydratase OS=Rattus norvegicus GN=Naxd PE=3 SV=1 - [NNRD_RAT]
Glutathione S-transferase alpha-4 OS=Rattus norvegicus GN=Gsta4 PE=1 SV=2 - [GSTA4_RAT]
A kinase (PRKA) anchor protein 6 OS=Rattus norvegicus GN=Akap6 PE=1 SV=1 - [G3V6M0_RAT]
Protein Chd4 OS=Rattus norvegicus GN=Chd4 PE=1 SV=3 - [E9PU01_RAT]
Synaptotagmin II OS=Rattus norvegicus GN=Syt2 PE=1 SV=1 - [G3V6M3_RAT]
Protein Ankrd34b OS=Rattus norvegicus GN=Ankrd34b PE=1 SV=1 - [D3ZKP1_RAT]
EF hand calcium binding protein 2 OS=Rattus norvegicus GN=Necab2 PE=4 SV=2 - [F1LQY6_RAT]
Ectonucleotide pyrophosphatase/phosphodiesterase 6 (Predicted), isoform CRA_a OS=Rattus norvegicus GN=Enp
Golgi associated, gamma adaptin ear containing, ARF binding protein 3 (Predicted) OS=Rattus norvegicus GN=Gga
D-dopachrome decarboxylase OS=Rattus norvegicus GN=Ddt PE=1 SV=3 - [DOPD_RAT]
Leukotriene A-4 hydrolase OS=Rattus norvegicus GN=Lta4h PE=2 SV=2 - [LKHA4_RAT]
DDB1- and CUL4-associated factor 8 OS=Rattus norvegicus GN=Dcaf8 PE=1 SV=1 - [A0A0G2JYN0_RAT]
HIV TAT specific factor 1 (Predicted) OS=Rattus norvegicus GN=Htatsf1 PE=1 SV=2 - [D4A997_RAT]
Protein Clmn OS=Rattus norvegicus GN=Clmn PE=1 SV=1 - [D4A626_RAT]
CDC42 effector protein (Rho GTPase binding) 4 (Predicted), isoform CRA_a OS=Rattus norvegicus GN=Cdc42ep4
Pre-mRNA-splicing factor 38B OS=Rattus norvegicus GN=Prpf38b PE=1 SV=1 - [PR38B_RAT]
Protein Smcr8 OS=Rattus norvegicus GN=Smcr8 PE=1 SV=1 - [D3ZJR6_RAT]
Protein Arhgef12 OS=Rattus norvegicus GN=Arhgef12 PE=1 SV=3 - [D3ZYR0_RAT]
Inositol polyphosphate-5-phosphatase F (Predicted), isoform CRA_c OS=Rattus norvegicus GN=Inpp5f PE=1 SV=1
Structural maintenance of chromosomes protein 3 OS=Rattus norvegicus GN=Smc3 PE=1 SV=1 - [SMC3_RAT]
Aconitate hydratase, mitochondrial OS=Rattus norvegicus GN=Aco2 PE=1 SV=2 - [ACON_RAT]
Acyl-CoA-binding protein OS=Rattus norvegicus GN=Dbi PE=1 SV=3 - [ACBP_RAT]
Kynurenine/alpha-aminoadipate aminotransferase, mitochondrial OS=Rattus norvegicus GN=Aadat PE=1 SV=1 - [A
Chondroitin sulfate proteoglycan 5 OS=Rattus norvegicus GN=Cspg5 PE=4 SV=3 - [F1M4R7_RAT]
Vinculin OS=Rattus norvegicus GN=Vcl PE=1 SV=1 - [VINC_RAT]
V-type proton ATPase subunit F OS=Rattus norvegicus GN=Atp6v1f PE=1 SV=1 - [VATF_RAT]
H/ACA ribonucleoprotein complex non-core subunit NAF1 OS=Rattus norvegicus GN=Naf1 PE=1 SV=1 - [A0A0G2K
Bumetanide-sensitive Na-K-2Cl cotransporter (Fragment) OS=Rattus norvegicus GN=NKCC1 PE=2 SV=1 - [Q9R1N
Pleckstrin homology domain containing, family F (With FYVE domain) member 2 OS=Rattus norvegicus GN=Plekhf
Protein Nebl OS=Rattus norvegicus GN=Nebl PE=4 SV=3 - [F1LVX3_RAT]
Eukaryotic translation initiation factor 5 OS=Rattus norvegicus GN=Eif5 PE=1 SV=1 - [IF5_RAT]
Protein Rab11fip5 OS=Rattus norvegicus GN=Rab11fip5 PE=1 SV=1 - [A0A0G2K1W1_RAT]
CDW92 antigen, isoform CRA_a OS=Rattus norvegicus GN=Slc44a1 PE=1 SV=1 - [A0A0G2K7R8_RAT]

Interferon-inducible double-stranded RNA-dependent protein kinase activator A OS=Rattus norvegicus GN=Prkra PI
Protein Ccdc6 OS=Rattus norvegicus GN=Ccdc6 PE=1 SV=3 - [D4AEK9_RAT]
Saccharopine dehydrogenase-like oxidoreductase OS=Rattus norvegicus GN=Sccpdh PE=1 SV=1 - [SCPDL_RAT]
Trifunctional enzyme subunit alpha, mitochondrial OS=Rattus norvegicus GN=Hadha PE=1 SV=2 - [ECHA_RAT]
Calpastatin OS=Rattus norvegicus GN=Cast PE=1 SV=1 - [F1LPH1_RAT]
Serine/threonine-protein kinase PRP4 homolog OS=Rattus norvegicus GN=Prpf4b PE=1 SV=1 - [PRP4B_RAT]
TRM1 tRNA methyltransferase 1 homolog (S. cerevisiae) OS=Rattus norvegicus GN=Trmt1 PE=1 SV=1 - [Q5U4E4_
Protein Tmcc1 OS=Rattus norvegicus GN=Tmcc1 PE=1 SV=1 - [D3ZH14_RAT]
Protein Nbas OS=Rattus norvegicus GN=Nbas PE=1 SV=2 - [F1M0U5_RAT]
Protein RGD1306271 OS=Rattus norvegicus GN=RGD1306271 PE=1 SV=3 - [D3Z9D0_RAT]
Neurofibromin OS=Rattus norvegicus GN=Nf1 PE=1 SV=1 - [A0A0G2JWL3_RAT]
Carbonic anhydrase 2 OS=Rattus norvegicus GN=Ca2 PE=1 SV=2 - [CAH2_RAT]
Protein Plekha7 OS=Rattus norvegicus GN=Plekha7 PE=1 SV=1 - [A0A0G2K6Z8_RAT]
Alpha-soluble NSF attachment protein OS=Rattus norvegicus GN=Napa PE=1 SV=2 - [SNAA_RAT]
Synaptojanin-1 OS=Rattus norvegicus GN=Synj1 PE=1 SV=3 - [D4ABN3_RAT]
Protein Pnn OS=Rattus norvegicus GN=Pnn PE=1 SV=1 - [D3ZAY8_RAT]
Protein RGD1560784 OS=Rattus norvegicus GN=RGD1560784 PE=4 SV=1 - [D3ZL45_RAT]
Tuftelin-interacting protein 11 OS=Rattus norvegicus GN=Tfip11 PE=1 SV=1 - [TFP11_RAT]
Lymphocyte specific 1, isoform CRA_a OS=Rattus norvegicus GN=Lsp1 PE=1 SV=1 - [Q4QQV6_RAT]
Protein Pck2 OS=Rattus norvegicus GN=Pck2 PE=1 SV=2 - [F1LQJ7_RAT]
Protein Dnal1 OS=Rattus norvegicus GN=Dnal1 PE=1 SV=1 - [A0A096MJZ0_RAT]
Fascin OS=Rattus norvegicus GN=Fscn1 PE=1 SV=2 - [FSCN1_RAT]
Dysbindin domain-containing protein 1 OS=Rattus norvegicus GN=Dbndd1 PE=1 SV=1 - [DBND1_RAT]
Glycylpeptide N-tetradecanoyltransferase 1 OS=Rattus norvegicus GN=Nmt1 PE=1 SV=1 - [NMT1_RAT]
Phosphodiesterase OS=Rattus norvegicus GN=Pde8a PE=1 SV=1 - [Q76KC6_RAT]
Protein Ptpdc1 OS=Rattus norvegicus GN=Ptpdc1 PE=1 SV=1 - [D4ACD9_RAT]
Protein LOC100911027 OS=Rattus norvegicus GN=LOC100911027 PE=4 SV=2 - [M0RCJ9_RAT]
Protein Ccdc177 OS=Rattus norvegicus GN=Ccdc177 PE=1 SV=1 - [D4A8V2_RAT]
Protein Ube3c OS=Rattus norvegicus GN=Ube3c PE=1 SV=3 - [D3ZHB7_RAT]
Protein Cadps2 OS=Rattus norvegicus GN=Cadps2 PE=4 SV=3 - [F1LWT1_RAT]
Histone H3 OS=Rattus norvegicus GN=Hist2h3c2 PE=1 SV=1 - [D3ZJ08_RAT]
Matrin-3 OS=Rattus norvegicus GN=Matr3 PE=1 SV=1 - [A0A0G2JSR7_RAT]
Anthrax toxin receptor 1 OS=Rattus norvegicus GN=Antxr1 PE=1 SV=2 - [ANTR1_RAT]
Cell adhesion molecule 2 OS=Rattus norvegicus GN=Cadm2 PE=1 SV=1 - [A0A0G2K5U6_RAT]
Fam49b protein OS=Rattus norvegicus GN=Fam49b PE=1 SV=1 - [B2GUZ9_RAT]
Protein Ccm2 OS=Rattus norvegicus GN=Ccm2 PE=1 SV=1 - [A0A0G2JZF5_RAT]
Bckdha protein (Fragment) OS=Rattus norvegicus GN=Bckdha PE=2 SV=1 - [Q5EB89_RAT]
Charged multivesicular body protein 5 OS=Rattus norvegicus GN=Chmp5 PE=2 SV=1 - [CHMP5_RAT]
Protein Fchsd2 OS=Rattus norvegicus GN=Fchsd2 PE=1 SV=1 - [A0A0G2K219_RAT]
Apolipoprotein E OS=Rattus norvegicus GN=Apoe PE=1 SV=2 - [APOE_RAT]
Protein Purg OS=Rattus norvegicus GN=Purg PE=1 SV=1 - [D3ZYS1_RAT]
Cell adhesion molecule 3 OS=Rattus norvegicus GN=Cadm3 PE=1 SV=1 - [CADM3_RAT]
Protein Fam114a2 OS=Rattus norvegicus GN=Fam114a2 PE=1 SV=3 - [D3ZC89_RAT]
Sideroflexin-5 OS=Rattus norvegicus GN=Sfxn5 PE=2 SV=1 - [SFXN5_RAT]
Protein Etl4 OS=Rattus norvegicus GN=Etl4 PE=1 SV=2 - [M0R5H1_RAT]
Protein Zrsr1 OS=Rattus norvegicus GN=Zrsr1 PE=2 SV=1 - [Q5RK33_RAT]
Aspartate aminotransferase, mitochondrial OS=Rattus norvegicus GN=Got2 PE=1 SV=2 - [AATM_RAT]
Tryptophan--tRNA ligase, cytoplasmic OS=Rattus norvegicus GN=Wars PE=1 SV=1 - [F8WFH8_RAT]
Protein Trim46 (Fragment) OS=Rattus norvegicus GN=Trim46 PE=1 SV=1 - [A0A096MKD9_RAT]
Protein Pbdc1 OS=Rattus norvegicus GN=Pbdc1 PE=1 SV=1 - [G3V6C3_RAT]
Phosphatase and actin regulator OS=Rattus norvegicus GN=Phactr3 PE=1 SV=1 - [A0A0G2K0S0_RAT]
Dedicator of cytokinesis protein 9 OS=Rattus norvegicus GN=Dock9 PE=1 SV=2 - [F1LSM8_RAT]
Protein Reps2 OS=Rattus norvegicus GN=Reps2 PE=1 SV=1 - [A0A0G2K4S1_RAT]
Protein LOC314140 OS=Rattus norvegicus GN=LOC314140 PE=1 SV=1 - [A0A0G2JSV3_RAT]
Protein Brap OS=Rattus norvegicus GN=Brap PE=1 SV=2 - [M0RBY8_RAT]

Exoc6b protein OS=Rattus norvegicus GN=Exoc6b PE=1 SV=1 - [B5DEY3_RAT]
Nucleolar protein 4 (Predicted), isoform CRA_b OS=Rattus norvegicus GN=Nol4 PE=1 SV=1 - [D4A3P4_RAT]
Adenylate cyclase 6, isoform CRA_b OS=Rattus norvegicus GN=Adcy6 PE=1 SV=2 - [F1LSD1_RAT]
Ethylmalonyl-CoA decarboxylase OS=Rattus norvegicus GN=Echdc1 PE=1 SV=1 - [ECHD1_RAT]
Similar to CG12125-PA (Predicted), isoform CRA_b OS=Rattus norvegicus GN=Miga2 PE=1 SV=1 - [D3Z899_RAT]
Protein Clasp1 OS=Rattus norvegicus GN=Clasp1 PE=1 SV=1 - [A0A0G2JTD7_RAT]
Superoxide dismutase [Cu-Zn] OS=Rattus norvegicus GN=Sod1 PE=2 SV=1 - [Q6LDS4_RAT]
Synapse associated protein 1 OS=Rattus norvegicus GN=Syap1 PE=2 SV=1 - [Q6AYB6_RAT]
Api5 protein OS=Rattus norvegicus GN=Api5 PE=1 SV=1 - [B1WC49_RAT]
Neural Wiskott-Aldrich syndrome protein OS=Rattus norvegicus GN=Wasl PE=1 SV=2 - [F1LSG0_RAT]
SH3 and multiple ankyrin repeat domains protein 1 OS=Rattus norvegicus GN=Shank1 PE=1 SV=1 - [SHAN1_RAT]
SP120 OS=Rattus norvegicus PE=2 SV=1 - [Q63555_RAT]
Protein U2af2 OS=Rattus norvegicus GN=U2af2 PE=1 SV=2 - [F2Z3T9_RAT]
Tyrosine-protein phosphatase non-receptor type 1 OS=Rattus norvegicus GN=Ptpn1 PE=1 SV=1 - [PTN1_RAT]
Lamin-B1 OS=Rattus norvegicus GN=Lmnb1 PE=1 SV=1 - [G3V7U4_RAT]
5'-3' exoribonuclease 2 (Predicted), isoform CRA_a OS=Rattus norvegicus GN=Xrn2 PE=1 SV=3 - [D4A914_RAT]
SPARC-like 1 (Mast9, hevin), isoform CRA_a OS=Rattus norvegicus GN=Sparcl1 PE=1 SV=1 - [G3V7X5_RAT]
Guanine nucleotide-binding protein subunit gamma OS=Rattus norvegicus GN=Gng10 PE=1 SV=1 - [Q3KRE3_RAT
Protein Mars OS=Rattus norvegicus GN=Mars PE=1 SV=1 - [D3Z941_RAT]
Sorbin and SH3 domain-containing protein 2 OS=Rattus norvegicus GN=Sorbs2 PE=1 SV=2 - [F1LPM3_RAT]
DDHD domain containing 1 OS=Rattus norvegicus GN=Ddhd1 PE=1 SV=1 - [Q3ZAU5_RAT]
Protein Arhgef26 OS=Rattus norvegicus GN=Arhgef26 PE=1 SV=3 - [D4A1D2_RAT]
Annexin A6 OS=Rattus norvegicus GN=Anxa6 PE=1 SV=2 - [ANXA6_RAT]
Synergin gamma OS=Rattus norvegicus GN=Synrg PE=1 SV=3 - [SYNRG_RAT]
Protein Pcdh7 OS=Rattus norvegicus GN=Pcdh7 PE=1 SV=1 - [Q68HB8_RAT]
Voltage-dependent R-type calcium channel subunit alpha OS=Rattus norvegicus GN=Cacna1e PE=2 SV=1 - [Q923
Catenin (Cadherin associated protein), delta 1 (Predicted), isoform CRA_a OS=Rattus norvegicus GN=Ctnnd1 PE=
ATP-dependent 6-phosphofructokinase OS=Rattus norvegicus GN=Pfkm PE=1 SV=1 - [Q52KS1_RAT]
Protein Diras2 OS=Rattus norvegicus GN=Diras2 PE=4 SV=1 - [D3ZHX3_RAT]
AP-2 complex subunit alpha-2 OS=Rattus norvegicus GN=Ap2a2 PE=1 SV=1 - [A0A0G2K943_RAT]
Alpha-endosulfine OS=Rattus norvegicus GN=Ensa PE=1 SV=1 - [ENSA_RAT]
Cytochrome c oxidase subunit 6B1 OS=Rattus norvegicus GN=Cox6b1 PE=1 SV=1 - [D3ZD09_RAT]
Phosphodiesterase OS=Rattus norvegicus GN=Pde1c PE=1 SV=1 - [A0A0G2KAI1_RAT]
Catechol O-methyltransferase OS=Rattus norvegicus GN=Comt PE=1 SV=2 - [COMT_RAT]
Protein Gbf1 OS=Rattus norvegicus GN=Gbf1 PE=1 SV=1 - [A0A0G2K3N1_RAT]
Immunoglobulin superfamily, member 8, isoform CRA_b OS=Rattus norvegicus GN=Igsf8 PE=1 SV=1 - [A0A140TA
Glycogen [starch] synthase, muscle OS=Rattus norvegicus GN=Gys1 PE=1 SV=1 - [GYS1_RAT]
Calcium-dependent secretion activator 1 OS=Rattus norvegicus GN=Cadps PE=1 SV=3 - [F1LLX6_RAT]
IQ motif and SEC7 domain-containing protein 3 OS=Rattus norvegicus GN=Iqsec3 PE=1 SV=1 - [F1LPA3_RAT]
Centrosomal protein of 104 kDa OS=Rattus norvegicus GN=Cep104 PE=2 SV=3 - [CE104_RAT]
Protein Trmt2a OS=Rattus norvegicus GN=Trmt2a PE=1 SV=1 - [Q5XIQ6_RAT]
Programmed cell death 6-interacting protein OS=Rattus norvegicus GN=Pdcd6ip PE=1 SV=1 - [A0A140TAA4_RAT]
Serine/threonine-protein kinase PAK 3 OS=Rattus norvegicus GN=Pak3 PE=1 SV=1 - [PAK3_RAT]
Tropomyosin alpha-4 chain OS=Rattus norvegicus GN=Tpm4 PE=1 SV=1 - [A0A0G2K2G8_RAT]
Voltage-dependent anion-selective channel protein 2 OS=Rattus norvegicus GN=Vdac2 PE=1 SV=2 - [VDAC2_RAT
Transcription factor BTF3 OS=Rattus norvegicus GN=Btf3 PE=1 SV=1 - [Q5U3Y8_RAT]
Junctophilin 3 (Predicted) OS=Rattus norvegicus GN=Jph3 PE=1 SV=1 - [D3ZWH2_RAT]
Numb-like protein OS=Rattus norvegicus GN=Numbl PE=1 SV=1 - [NUMBL_RAT]
Arf-GAP with coiled-coil, ANK repeat and PH domain-containing protein 2 OS=Rattus norvegicus GN=Acap2 PE=1
Protein RGD621098 OS=Rattus norvegicus GN=RGD621098 PE=1 SV=1 - [A0A0G2JWC6_RAT]
Protein PRRC2A OS=Rattus norvegicus GN=Prrc2a PE=1 SV=1 - [PRC2A_RAT]
Small ubiquitin-related modifier 1 OS=Rattus norvegicus GN=Sumo1 PE=1 SV=1 - [SUMO1_RAT]
Mitochondrial antiviral-signaling protein OS=Rattus norvegicus GN=Mavs PE=1 SV=1 - [MAVS_RAT]
Glutathione S-transferase alpha-1 OS=Rattus norvegicus GN=Gsta1 PE=1 SV=1 - [A0A0G2JVT2_RAT]
Protein Vps26b OS=Rattus norvegicus GN=Vps26b PE=1 SV=1 - [B1WBS4_RAT]

Information Classification: General

4-trimethylaminobutyraldehyde dehydrogenase OS=Rattus norvegicus GN=Aldh9a1 PE=1 SV=1 - [A0A0G2JSI1_RA
Serum albumin OS=Rattus norvegicus GN=Alb PE=1 SV=1 - [A0A0G2JSH5_RAT]
Protein Gprin3 OS=Rattus norvegicus GN=Gprin3 PE=1 SV=2 - [D3ZF21_RAT]
Protein Tmem185a OS=Rattus norvegicus GN=Tmem185a PE=4 SV=1 - [D3ZWX2_RAT]
Protein Efr3b OS=Rattus norvegicus GN=Efr3b PE=1 SV=2 - [F1LTW9_RAT]
Contactin-1 OS=Rattus norvegicus GN=Cntn1 PE=1 SV=2 - [CNTN1_RAT]
Protein Hexdc OS=Rattus norvegicus GN=Hexdc PE=1 SV=1 - [F1LR76_RAT]
Protein CASC3 OS=Rattus norvegicus GN=Casc3 PE=1 SV=2 - [G3V793_RAT]
Alanyl-tRNA editing protein Aarsd1 OS=Rattus norvegicus GN=Aarsd1 PE=1 SV=1 - [AASD1_RAT]
Uncharacterized protein OS=Rattus norvegicus PE=4 SV=1 - [A0A0G2K689_RAT]
Profilin-2 OS=Rattus norvegicus GN=Pfn2 PE=1 SV=3 - [PROF2_RAT]
Peptidyl-prolyl cis-trans isomerase FKBP1A OS=Rattus norvegicus GN=Fkbp1a PE=1 SV=3 - [FKB1A_RAT]
AP-4 complex accessory subunit tepsin OS=Rattus norvegicus GN=Enthd2 PE=1 SV=1 - [AP4AT_RAT]
Hepatoma-derived growth factor-related protein 3 OS=Rattus norvegicus GN=Hdgfrp3 PE=1 SV=1 - [HDGR3_RAT]
Protein Tmem94 OS=Rattus norvegicus GN=Tmem94 PE=1 SV=2 - [F1LUG5_RAT]
Rho guanine nucleotide exchange factor 11 OS=Rattus norvegicus GN=Arhgef11 PE=1 SV=3 - [F1LQS9_RAT]
RCG20461 OS=Rattus norvegicus GN=Stum PE=1 SV=3 - [D4A4F9_RAT]
Versican core protein OS=Rattus norvegicus GN=Vcan PE=4 SV=2 - [D3ZFC3_RAT]
Ganglioside-induced differentiation-associated protein 1-like 1 (Predicted) OS=Rattus norvegicus GN=Gdap1l1 PE=
Uncharacterized protein C11orf96 homolog OS=Rattus norvegicus GN=Ag2 PE=1 SV=1 - [CK096_RAT]
Cadherin-10 (Fragment) OS=Rattus norvegicus PE=2 SV=1 - [Q8R5H2_RAT]
Sulfotransferase 4A1 OS=Rattus norvegicus GN=Sult4a1 PE=1 SV=1 - [ST4A1_RAT]
Protein Ablim3 OS=Rattus norvegicus GN=Ablim3 PE=1 SV=1 - [A0A0G2K875_RAT]
Chaperonin subunit 8 (Theta) (Predicted), isoform CRA_a OS=Rattus norvegicus GN=Cct8 PE=1 SV=1 - [D4ACB8_
MAGUK p55 subfamily member 3 OS=Rattus norvegicus GN=Mpp3 PE=1 SV=3 - [MPP3_RAT]
Protein Pard3b OS=Rattus norvegicus GN=Pard3b PE=1 SV=2 - [F1LW22_RAT]
Signal transducer and activator of transcription OS=Rattus norvegicus GN=Stat1 PE=1 SV=2 - [F1M9D6_RAT]
Protein Fam234b OS=Rattus norvegicus GN=Fam234b PE=1 SV=1 - [D3ZWJ9_RAT]
Stannin OS=Rattus norvegicus GN=Snn PE=1 SV=1 - [SNN_RAT]
Proline-rich protein 7 OS=Rattus norvegicus GN=Prr7 PE=1 SV=1 - [PRR7_RAT]
Solute carrier family 12 member 9 (Fragment) OS=Rattus norvegicus GN=Slc12a9 PE=1 SV=6 - [A0A096MK93_RA
Cysteine and histidine-rich domain-containing protein 1 OS=Rattus norvegicus GN=Chordc1 PE=3 SV=1 - [CHRD1_
Protein Otud7b OS=Rattus norvegicus GN=Otud7b PE=1 SV=1 - [D3ZH40_RAT]
Pgm3 protein OS=Rattus norvegicus GN=Pgm3 PE=1 SV=1 - [B2RYN0_RAT]
U4/U6.U5 tri-snRNP-associated protein 1 OS=Rattus norvegicus GN=Sart1 PE=1 SV=1 - [SNUT1_RAT]
Cnpy3 protein OS=Rattus norvegicus GN=Cnpy3 PE=2 SV=1 - [B2RYF8_RAT]
Uncharacterized protein OS=Rattus norvegicus PE=1 SV=1 - [A0A0G2JZ88_RAT]
Protein RGD1311575 OS=Rattus norvegicus GN=RGD1311575 PE=1 SV=3 - [D4A5F4_RAT]
Syntaxin-7 OS=Rattus norvegicus GN=Stx7 PE=3 SV=1 - [A0A0G2K6Y9_RAT]
Transcription activator BRG1 OS=Rattus norvegicus GN=Smarca4 PE=1 SV=2 - [G3V790_RAT]
Sodium/calcium exchanger 1 OS=Rattus norvegicus GN=Slc8a1 PE=3 SV=2 - [M0R3V7_RAT]
Type-1 angiotensin II receptor-associated protein OS=Rattus norvegicus GN=Agtrap PE=1 SV=1 - [ATRAP_RAT]
Protein O-GlcNAcase OS=Rattus norvegicus GN=Mgea5 PE=1 SV=1 - [OGA_RAT]
Taperin OS=Rattus norvegicus GN=Tprn PE=1 SV=2 - [TPRN_RAT]
60S ribosomal protein L17 OS=Rattus norvegicus GN=Rpl17 PE=2 SV=3 - [RL17_RAT]
Protein Tiam2 OS=Rattus norvegicus GN=Tiam2 PE=1 SV=2 - [D3ZMS5_RAT]
PH and SEC7 domain-containing protein 1 OS=Rattus norvegicus GN=Psd PE=1 SV=1 - [G3V8J5_RAT]
Glutamate receptor 1 OS=Rattus norvegicus GN=Gria1 PE=3 SV=2 - [M0R5P7_RAT]
Ab1-351 OS=Rattus norvegicus GN=LOC364556 PE=2 SV=1 - [Q7TP95_RAT]
Hyaluronan binding protein 4 OS=Rattus norvegicus GN=Habp4 PE=1 SV=1 - [A1L1K8_RAT]
Trifunctional enzyme subunit beta, mitochondrial OS=Rattus norvegicus GN=Hadhb PE=1 SV=1 - [ECHB_RAT]
Protein Agap1 OS=Rattus norvegicus GN=Agap1 PE=4 SV=1 - [A0A0G2K847_RAT]
Src substrate cortactin (Fragment) OS=Rattus norvegicus GN=Cttn PE=4 SV=1 - [A0A1B0GWS4_RAT]
Biliverdin reductase A OS=Rattus norvegicus GN=Blvra PE=1 SV=1 - [BIEA_RAT]
Ctr9 protein (Fragment) OS=Rattus norvegicus GN=Ctr9 PE=2 SV=1 - [Q32PZ5_RAT]

Protein Pcmtd2 OS=Rattus norvegicus GN=Pcmtd2 PE=4 SV=2 - [D3ZY20_RAT]
Protein Rpl22l1 OS=Rattus norvegicus GN=Rpl22l1 PE=2 SV=1 - [B2RZD5_RAT]
Protein Grid2ip OS=Rattus norvegicus GN=Grid2ip PE=1 SV=2 - [F1LU68_RAT]
Protein IWS1 homolog OS=Rattus norvegicus GN=Iws1 PE=1 SV=1 - [IWS1_RAT]
Cortactin-binding protein 2 OS=Rattus norvegicus GN=Cttnbp2 PE=1 SV=1 - [CTTB2_RAT]
Protein Dock7 OS=Rattus norvegicus GN=Dock7 PE=1 SV=2 - [F1LRS2_RAT]
General vesicular transport factor p115 OS=Rattus norvegicus GN=Uso1 PE=1 SV=1 - [USO1_RAT]
Protein Usp32 OS=Rattus norvegicus GN=Usp32 PE=1 SV=1 - [D3ZBB7_RAT]
Protein Tom1 OS=Rattus norvegicus GN=Tom1 PE=1 SV=1 - [Q5XI21_RAT]
Protein Limch1 OS=Rattus norvegicus GN=Limch1 PE=1 SV=2 - [F1M392_RAT]
Signal-induced proliferation-associated 1-like protein 2 OS=Rattus norvegicus GN=Sipa1l2 PE=1 SV=3 - [F1M8G8_
Nesprin-1 OS=Rattus norvegicus GN=Syne1 PE=2 SV=1 - [Q8VHJ9_RAT]
Protein Srgap3 OS=Rattus norvegicus GN=Srgap3 PE=1 SV=2 - [F1M5M9_RAT]
Transmembrane protein 245 OS=Rattus norvegicus GN=Tmem245 PE=1 SV=1 - [TM245_RAT]
Solute carrier organic anion transporter family member 1A5 OS=Rattus norvegicus GN=Slco1a5 PE=2 SV=2 - [SO1
Protein Sema4d OS=Rattus norvegicus GN=Sema4d PE=1 SV=3 - [D3ZYR4_RAT]
Protein Pacsin3 OS=Rattus norvegicus GN=Pacsin3 PE=1 SV=3 - [G3V9N7_RAT]
SH3 and multiple ankyrin repeat domains protein 3 (Fragment) OS=Rattus norvegicus GN=Shank3 PE=1 SV=2 - [A
Nardilysin, N-arginine dibasic convertase 1 OS=Rattus norvegicus GN=Nrd1 PE=1 SV=1 - [G3V700_RAT]
Calcium-transporting ATPase OS=Rattus norvegicus GN=Atp2b3 PE=1 SV=1 - [A0A0G2K9Q6_RAT]
Protein Pak4 OS=Rattus norvegicus GN=Pak4 PE=1 SV=1 - [B5DF62_RAT]
Solute carrier family 15 member 2 OS=Rattus norvegicus GN=Slc15a2 PE=1 SV=1 - [S15A2_RAT]
Basic leucine zipper and W2 domain-containing protein 2 OS=Rattus norvegicus GN=Bzw2 PE=1 SV=1 - [BZW2_R
Glycylpeptide N-tetradecanoyltransferase OS=Rattus norvegicus GN=Nmt2 PE=1 SV=3 - [F1M110_RAT]
Protein kinase C theta type OS=Rattus norvegicus GN=Prkcq PE=1 SV=1 - [F1LM10_RAT]
FACT complex subunit SSRP1 OS=Rattus norvegicus GN=Ssrp1 PE=1 SV=2 - [SSRP1_RAT]
Histone H1.0 OS=Rattus norvegicus GN=H1f0 PE=2 SV=2 - [H10_RAT]
Protein Mlip OS=Rattus norvegicus GN=Mlip PE=1 SV=3 - [D4A3C4_RAT]
Activated RNA polymerase II transcriptional coactivator p15 OS=Rattus norvegicus GN=Sub1 PE=1 SV=3 - [TCP4_
Sequestosome-1 OS=Rattus norvegicus GN=Sqstm1 PE=1 SV=1 - [SQSTM_RAT]
Protein Ccdc88a OS=Rattus norvegicus GN=Ccdc88a PE=1 SV=3 - [D3ZYD7_RAT]
Guanine nucleotide-binding protein subunit gamma (Fragment) OS=Rattus norvegicus PE=2 SV=1 - [O35355_RAT]
Ras-related protein Rab-3B OS=Rattus norvegicus GN=Rab3b PE=1 SV=2 - [RAB3B_RAT]
Protein RGD1309995 OS=Rattus norvegicus GN=RGD1309995 PE=1 SV=1 - [D4A7I6_RAT]
Tropomyosin alpha-3 chain OS=Rattus norvegicus GN=Tpm3 PE=1 SV=2 - [TPM3_RAT]
Serine/threonine-protein kinase PAK 7 OS=Rattus norvegicus GN=Pak7 PE=1 SV=1 - [PAK7_RAT]
Serine/threonine-protein kinase MRCK beta OS=Rattus norvegicus GN=Cdc42bpb PE=1 SV=1 - [A0A0G2KB58_RA
Fam103a1 protein OS=Rattus norvegicus GN=Fam103a1 PE=2 SV=1 - [Q5FVL9_RAT]
Bifunctional purine biosynthesis protein PURH OS=Rattus norvegicus GN=Atic PE=1 SV=2 - [PUR9_RAT]
Density-regulated protein OS=Rattus norvegicus GN=Denr PE=2 SV=1 - [B0BNB2_RAT]
Aspartyl aminopeptidase OS=Rattus norvegicus GN=Dnpep PE=1 SV=1 - [Q4V8H5_RAT]
N-terminal EF-hand calcium-binding protein 1 OS=Rattus norvegicus GN=Necab1 PE=1 SV=1 - [NECA1_RAT]
Ttc7 protein (Fragment) OS=Rattus norvegicus GN=Ttc7a PE=2 SV=1 - [Q4G027_RAT]
Calcium channel, voltage-dependent, beta 2 subunit, isoform CRA_d OS=Rattus norvegicus GN=Cacnb2 PE=1 SV=
GRIP1-associated protein 1 OS=Rattus norvegicus GN=Gripap1 PE=1 SV=1 - [GRAP1_RAT]
Protein Tiam1 OS=Rattus norvegicus GN=Tiam1 PE=1 SV=3 - [D3ZWV8_RAT]
Neuroligin-2 OS=Rattus norvegicus GN=Nlgn2 PE=1 SV=3 - [F1LQ41_RAT]
Protein Wdr48 OS=Rattus norvegicus GN=Wdr48 PE=1 SV=1 - [D3Z8C7_RAT]
Protein Ube2q2l OS=Rattus norvegicus GN=Ube2q2l PE=1 SV=1 - [D4ADP2_RAT]
RCG55460, isoform CRA_a OS=Rattus norvegicus GN=Ralbp1 PE=1 SV=1 - [Q5FVT1_RAT]
Adenylate cyclase 1 (Predicted) OS=Rattus norvegicus GN=Adcy1 PE=1 SV=2 - [D4A3N4_RAT]
Protein Clip3 OS=Rattus norvegicus GN=Clip3 PE=1 SV=1 - [D4A507_RAT]
Syndetin OS=Rattus norvegicus GN=Vps50 PE=1 SV=1 - [VPS50_RAT]
Hyou1 protein OS=Rattus norvegicus GN=Hyou1 PE=1 SV=1 - [Q6P136_RAT]
Dynein light chain 2, cytoplasmic OS=Rattus norvegicus GN=Dynll2 PE=1 SV=1 - [DYL2_RAT]

Mitogen-activated protein kinase kinase kinase kinase OS=Rattus norvegicus GN=Map4k2 PE=1 SV=1 - [D3ZXB1_
Golgi apparatus membrane protein TVP23 homolog OS=Rattus norvegicus GN=Tvp23b PE=1 SV=1 - [M0R766_RA
Glutamate receptor ionotropic, NMDA 2A OS=Rattus norvegicus GN=Grin2a PE=1 SV=2 - [G3V9C5_RAT]
Activity-dependent neuroprotector homeobox protein OS=Rattus norvegicus GN=Adnp PE=1 SV=2 - [ADNP_RAT]
Receptor-binding cancer antigen expressed on SiSo cells OS=Rattus norvegicus GN=Ebag9 PE=1 SV=1 - [RCAS1_
Palmitoyltransferase OS=Rattus norvegicus GN=Zdhhc20 PE=2 SV=1 - [A0JPI6_RAT]
Signal-induced proliferation-associated 1-like protein 1 (Fragment) OS=Rattus norvegicus GN=Sipa1l1 PE=1 SV=3
Protein Ppa1 OS=Rattus norvegicus GN=Ppa1 PE=1 SV=1 - [F7EPH4_RAT]
F-actin-capping protein subunit alpha-1 OS=Rattus norvegicus GN=Capza1 PE=1 SV=1 - [CAZA1_RAT]
Protein Kri1 OS=Rattus norvegicus GN=Kri1 PE=4 SV=1 - [A0A0G2JXS2_RAT]
Kinase D-interacting substrate 220 isoform 7 OS=Rattus norvegicus GN=Kidins220 PE=2 SV=1 - [A0A0H4SRI7_RA
Putative uncharacterized protein RGD1306215_predicted OS=Rattus norvegicus GN=RGD1306215 PE=2 SV=1 - [
Far upstream element-binding protein 2 OS=Rattus norvegicus GN=Khsrp PE=1 SV=1 - [M0R961_RAT]
Phospholipase D1 OS=Rattus norvegicus GN=Pld1 PE=1 SV=2 - [D4A318_RAT]
Rnf14 protein (Fragment) OS=Rattus norvegicus GN=Rnf14 PE=2 SV=1 - [B2GV23_RAT]
Similar to Ubiquitin carboxyl-terminal hydrolase 4 (Ubiquitin thiolesterase 4) (Ubiquitin-specific processing protease
Basic leucine zipper and W2 domain-containing protein 1 OS=Rattus norvegicus GN=Bzw1 PE=1 SV=1 - [BZW1_R
Protein Rbm17 OS=Rattus norvegicus GN=Rbm17 PE=1 SV=1 - [Q6AY02_RAT]
Mitochondrial import receptor subunit TOM20 homolog OS=Rattus norvegicus GN=Tomm20 PE=1 SV=2 - [TOM20_
Zinc finger CCCH domain-containing protein 18 OS=Rattus norvegicus GN=Zc3h18 PE=1 SV=1 - [ZCH18_RAT]
Potassium voltage-gated channel subfamily KQT member 3 OS=Rattus norvegicus GN=Kcnq3 PE=3 SV=3 - [F1LP
Protein Wdr11 OS=Rattus norvegicus GN=Wdr11 PE=1 SV=2 - [D3Z9L5_RAT]
Protein phosphatase 1, regulatory subunit 9B OS=Rattus norvegicus GN=Ppp1r9b PE=2 SV=1 - [B1H262_RAT]
Protein Rasal2 OS=Rattus norvegicus GN=Rasal2 PE=1 SV=1 - [A0A0G2JTA7_RAT]
Metabotropic glutamate receptor 5 OS=Rattus norvegicus GN=Grm5 PE=1 SV=1 - [A0A0H2UHW6_RAT]
Uncharacterized protein OS=Rattus norvegicus PE=4 SV=2 - [D4ACJ7_RAT]
Protein Rangap1 OS=Rattus norvegicus GN=Rangap1 PE=1 SV=2 - [F1MAA5_RAT]
Protein Vps35 OS=Rattus norvegicus GN=Vps35 PE=1 SV=1 - [G3V8A5_RAT]
Protein Dpp9 OS=Rattus norvegicus GN=Dpp9 PE=1 SV=1 - [M0R781_RAT]
Protein Dopey1 OS=Rattus norvegicus GN=Dopey1 PE=1 SV=2 - [D4A0Y2_RAT]
Protein Praf2 OS=Rattus norvegicus GN=Praf2 PE=1 SV=1 - [A0A0G2JTX2_RAT]
Inactive ubiquitin carboxyl-terminal hydrolase 54 OS=Rattus norvegicus GN=Usp54 PE=1 SV=2 - [UBP54_RAT]
Protein Arpp21 OS=Rattus norvegicus GN=Arpp21 PE=1 SV=1 - [D3ZQR3_RAT]
Rho GDP-dissociation inhibitor 1 OS=Rattus norvegicus GN=Arhgdia PE=1 SV=1 - [GDIR1_RAT]
Protein Ythdc2 OS=Rattus norvegicus GN=Ythdc2 PE=1 SV=3 - [D3ZIV8_RAT]
Kalirin OS=Rattus norvegicus GN=Kalrn PE=1 SV=3 - [KALRN_RAT]
PEST proteolytic signal-containing nuclear protein OS=Rattus norvegicus GN=Pcnp PE=1 SV=1 - [A0A0H2UHW4_
Mannose 6-phosphate/insulin-like growth factor II receptor OS=Rattus norvegicus GN=Igf2r PE=2 SV=1 - [Q63002_
Choline kinase alpha OS=Rattus norvegicus GN=Chka PE=2 SV=1 - [Q66HK1_RAT]
Protein Ccdc97 OS=Rattus norvegicus GN=Ccdc97 PE=1 SV=1 - [D4A4L2_RAT]
Phytanoyl-CoA hydroxylase-interacting protein-like OS=Rattus norvegicus GN=Phyhipl PE=1 SV=2 - [PHIPL_RAT]
Choline-phosphate cytidylyltransferase A OS=Rattus norvegicus GN=Pcyt1a PE=1 SV=2 - [PCY1A_RAT]
Zuotin related factor 4 OS=Rattus norvegicus GN=Dnajc2 PE=2 SV=1 - [Q7TQ19_RAT]
Acylamino-acid-releasing enzyme OS=Rattus norvegicus GN=Apeh PE=1 SV=1 - [G3V9E4_RAT]
Protein Slc25a12 OS=Rattus norvegicus GN=Slc25a12 PE=1 SV=3 - [F1LX07_RAT]
Delta-aminolevulinic acid dehydratase OS=Rattus norvegicus GN=Alad PE=1 SV=1 - [HEM2_RAT]
Protein Tmcc2 OS=Rattus norvegicus GN=Tmcc2 PE=1 SV=1 - [D3ZE26_RAT]
Glycerophosphocholine phosphodiesterase GPCPD1 OS=Rattus norvegicus GN=Gpcpd1 PE=1 SV=1 - [A0A0G2K
Glutamate receptor 2 OS=Rattus norvegicus GN=Gria2 PE=3 SV=2 - [F1LNE4_RAT]
Thy-1 membrane glycoprotein OS=Rattus norvegicus GN=Thy1 PE=1 SV=1 - [THY1_RAT]
Transmembrane protein 163 OS=Rattus norvegicus GN=Tmem163 PE=1 SV=1 - [TM163_RAT]
Protein Ssh2 OS=Rattus norvegicus GN=Ssh2 PE=1 SV=2 - [F1M4Q5_RAT]
Protein Ncam2 OS=Rattus norvegicus GN=Ncam2 PE=1 SV=2 - [F1M8G9_RAT]
Protein Xpr1 OS=Rattus norvegicus GN=Xpr1 PE=4 SV=1 - [A0A0G2JXZ7_RAT]
Nuclear receptor binding protein OS=Rattus norvegicus GN=Nrbp1 PE=1 SV=1 - [Q3SWT7_RAT]

Information Classification: General

Adenine phosphoribosyltransferase OS=Rattus norvegicus GN=Aprt PE=1 SV=1 - [APT_RAT]
Calcium-binding and coiled-coil domain-containing protein 1 OS=Rattus norvegicus GN=Calcoco1 PE=2 SV=1 - [CA
Protein Spata33 OS=Rattus norvegicus GN=Spata33 PE=1 SV=1 - [D3Z8L2_RAT]
Rab8a protein (Fragment) OS=Rattus norvegicus GN=Rab8a PE=2 SV=1 - [Q5M970_RAT]
Vesicle transport through interaction with t-SNAREs homolog 1B OS=Rattus norvegicus GN=Vti1b PE=1 SV=2 - [F1
Rho guanine nucleotide exchange factor 28 OS=Rattus norvegicus GN=Arhgef28 PE=1 SV=1 - [ARG28_RAT]
Mitochondrial import receptor subunit TOM34 OS=Rattus norvegicus GN=Tomm34 PE=1 SV=1 - [TOM34_RAT]
ATP-dependent 6-phosphofructokinase, liver type OS=Rattus norvegicus GN=Pfkl PE=1 SV=3 - [PFKAL_RAT]
Translation initiation factor eIF-2B subunit epsilon OS=Rattus norvegicus GN=Eif2b5 PE=1 SV=2 - [EI2BE_RAT]
Cadherin-6 OS=Rattus norvegicus GN=Cdh6 PE=1 SV=1 - [CADH6_RAT]
Proton myo-inositol cotransporter OS=Rattus norvegicus GN=Slc2a13 PE=1 SV=2 - [MYCT_RAT]
Complexin-2 OS=Rattus norvegicus GN=Cplx2 PE=1 SV=1 - [CPLX2_RAT]
Protein Rhog (Fragment) OS=Rattus norvegicus GN=Rhog PE=1 SV=1 - [A0A096MK75_RAT]
Protein Aspscr1 OS=Rattus norvegicus GN=Aspscr1 PE=1 SV=1 - [A0A0G2JWF4_RAT]
Protein Lmtk3 OS=Rattus norvegicus GN=Lmtk3 PE=4 SV=3 - [F1LSB5_RAT]
Protein Elmo1 OS=Rattus norvegicus GN=Elmo1 PE=1 SV=1 - [A0A0G2K4S6_RAT]
FXYD domain-containing ion transport regulator 7 OS=Rattus norvegicus GN=Fxyd7 PE=1 SV=1 - [FXYD7_RAT]
Nuclear pore complex protein Nup155 OS=Rattus norvegicus GN=Nup155 PE=1 SV=1 - [F1LS02_RAT]
Trafficking protein particle complex subunit 2 OS=Rattus norvegicus GN=Trappc2 PE=3 SV=1 - [TPPC2_RAT]
Protein Pnisr OS=Rattus norvegicus GN=Pnisr PE=1 SV=2 - [F1MAQ8_RAT]
E3 ubiquitin-protein ligase RNF34 OS=Rattus norvegicus GN=Rnf34 PE=1 SV=1 - [RNF34_RAT]
WD repeat-containing protein 44 OS=Rattus norvegicus GN=Wdr44 PE=1 SV=1 - [A0A0G2K7S6_RAT]
Protein Trim33 OS=Rattus norvegicus GN=Trim33 PE=1 SV=3 - [D3ZUM5_RAT]
Protein Rcsd1 OS=Rattus norvegicus GN=Rcsd1 PE=1 SV=2 - [F1M4V3_RAT]
Protein Actn2 OS=Rattus norvegicus GN=Actn2 PE=1 SV=1 - [D3ZCV0_RAT]
Protein Ubap2l OS=Rattus norvegicus GN=Ubap2l PE=1 SV=1 - [A0A0G2JYC6_RAT]
PRP38 pre-mRNA processing factor 38 (Yeast) domain containing A (Predicted), isoform CRA_b OS=Rattus norveg
Somatic angiotensin-1 converting enzyme (Fragment) OS=Rattus norvegicus GN=Ace PE=2 SV=1 - [Q8CJ04_RAT
Protein Zc3h13 OS=Rattus norvegicus GN=Zc3h13 PE=1 SV=2 - [E9PSN4_RAT]
Protein Cep76 OS=Rattus norvegicus GN=Cep76 PE=1 SV=1 - [A0A0G2K779_RAT]
Lymphocyte cytosolic protein 1 OS=Rattus norvegicus GN=Lcp1 PE=1 SV=1 - [Q5XI38_RAT]
ATP-binding cassette protein C1 variant B OS=Rattus norvegicus GN=Abcc1 PE=2 SV=1 - [Q810G8_RAT]
Neuronal acetylcholine receptor subunit alpha-4 (Fragment) OS=Rattus norvegicus GN=Chrna4 PE=1 SV=3 - [K4DI
Protein LOC303140 OS=Rattus norvegicus GN=Slc22a5 PE=1 SV=1 - [B2GUV4_RAT]
Keratin, type II cytoskeletal 8 OS=Rattus norvegicus GN=Krt8 PE=1 SV=3 - [K2C8_RAT]
RAF proto-oncogene serine/threonine-protein kinase OS=Rattus norvegicus GN=Raf1 PE=1 SV=1 - [RAF1_RAT]
Protein Dtx3l OS=Rattus norvegicus GN=Dtx3l PE=1 SV=1 - [D3Z8X6_RAT]
Potassium voltage-gated channel subfamily D member 3 OS=Rattus norvegicus GN=Kcnd3 PE=1 SV=1 - [A0A0G2
COP9 signalosome complex subunit 3 OS=Rattus norvegicus GN=Cops3 PE=1 SV=1 - [CSN3_RAT]
Uncharacterized protein OS=Rattus norvegicus PE=4 SV=2 - [F1M2N4_RAT]
Protein Tmbim1 (Fragment) OS=Rattus norvegicus GN=Tmbim1 PE=1 SV=1 - [A0A0G2K6F5_RAT]
Protein Lmnb2 OS=Rattus norvegicus GN=Lmnb2 PE=1 SV=2 - [D3ZLC1_RAT]
Protein Tcf20 OS=Rattus norvegicus GN=Tcf20 PE=1 SV=1 - [D3ZG21_RAT]
Protein Cyfip1 OS=Rattus norvegicus GN=Cyfip1 PE=1 SV=1 - [A0A0G2K472_RAT]
Protein Nufip2 OS=Rattus norvegicus GN=Nufip2 PE=1 SV=2 - [D3ZC82_RAT]
Arpc1a protein OS=Rattus norvegicus GN=Arpc1a PE=2 SV=1 - [Q6PCU9_RAT]
Protein Prpf3 OS=Rattus norvegicus GN=Prpf3 PE=1 SV=1 - [A0A0G2JTI7_RAT]
Synaptic vesicle glycoprotein 2B OS=Rattus norvegicus GN=Sv2b PE=1 SV=1 - [SV2B_RAT]
Insulin receptor substrate 1 OS=Rattus norvegicus GN=Irs1 PE=1 SV=1 - [G3V7V7_RAT]
Enolase-phosphatase E1 OS=Rattus norvegicus GN=Enoph1 PE=2 SV=1 - [ENOPH_RAT]
Palmdelphin OS=Rattus norvegicus GN=Palmd PE=1 SV=1 - [PALMD_RAT]
Cytochrome c oxidase subunit 2 OS=Rattus norvegicus GN=COX2 PE=3 SV=1 - [A0A097PE04_RAT]
Voltage-dependent calcium channel gamma-7 subunit OS=Rattus norvegicus GN=Cacng7 PE=1 SV=1 - [CCG7_RA
Protein Pitpnm3 OS=Rattus norvegicus GN=Pitpnm3 PE=1 SV=2 - [M0RDK4_RAT]
Serine/threonine-protein kinase MRCK alpha OS=Rattus norvegicus GN=Cdc42bpa PE=1 SV=1 - [A0A0G2K5Z1_R

Protein Rbsn OS=Rattus norvegicus GN=Rbsn PE=1 SV=1 - [D3ZL11_RAT]
Coiled-coil domain-containing protein 93 OS=Rattus norvegicus GN=Ccdc93 PE=1 SV=1 - [CCD93_RAT]
Ral GTPase-activating protein subunit beta OS=Rattus norvegicus GN=Ralgapb PE=1 SV=1 - [A0A0G2KA57_RAT]
UBX domain-containing protein 2B OS=Rattus norvegicus GN=Ubxn2b PE=1 SV=1 - [UBX2B_RAT]
Drebrin-like protein OS=Rattus norvegicus GN=Dbnl PE=1 SV=1 - [DBNL_RAT]
Kinesin-like protein KIF1B OS=Rattus norvegicus GN=Kif1b PE=1 SV=1 - [A0A0G2KA12_RAT]
Succinate-CoA ligase subunit beta (Fragment) OS=Rattus norvegicus GN=Sucla2 PE=2 SV=1 - [B2RZ24_RAT]
3-hydroxyacyl-CoA dehydrogenase type-2 OS=Rattus norvegicus GN=Hsd17b10 PE=1 SV=1 - [B0BMW2_RAT]
Downstream of Stk11 (Predicted), isoform CRA_a OS=Rattus norvegicus GN=Cbarp PE=1 SV=2 - [F1MAS1_RAT]
Heat shock 70 kDa protein 4 OS=Rattus norvegicus GN=Hspa4 PE=1 SV=1 - [F1LRV4_RAT]
Protein Ppp6r1 OS=Rattus norvegicus GN=Ppp6r1 PE=1 SV=1 - [D3ZG37_RAT]
Protein Stk38 (Fragment) OS=Rattus norvegicus GN=Stk38 PE=1 SV=2 - [A0A0U1RRV1_RAT]
Beta-synuclein OS=Rattus norvegicus GN=Sncb PE=1 SV=1 - [A0A0G2JSQ1_RAT]
Relaxin-3 OS=Rattus norvegicus GN=Rln3 PE=2 SV=1 - [REL3_RAT]
Bromodomain containing 7 (Predicted) OS=Rattus norvegicus GN=Brd7 PE=1 SV=1 - [D3ZXX7_RAT]
Putative uncharacterized protein RGD1310680_predicted OS=Rattus norvegicus GN=Tmem151a PE=2 SV=1 - [B5I
Pyridoxal kinase OS=Rattus norvegicus GN=Pdxk PE=1 SV=1 - [G3V647_RAT]
Protein Atg2b OS=Rattus norvegicus GN=Atg2b PE=1 SV=2 - [F1MAF8_RAT]
Nucleobindin-1 OS=Rattus norvegicus GN=Nucb1 PE=1 SV=1 - [NUCB1_RAT]
Stonin-2 OS=Rattus norvegicus GN=Ston2 PE=1 SV=1 - [STON2_RAT]
Protein Ppp2r5e OS=Rattus norvegicus GN=Ppp2r5e PE=1 SV=1 - [D3ZHI9_RAT]
EH domain-containing protein 2 OS=Rattus norvegicus GN=Ehd2 PE=1 SV=1 - [EHD2_RAT]
Protein Gemin5 OS=Rattus norvegicus GN=Gemin5 PE=1 SV=2 - [D3ZGD0_RAT]
Protein Lrrc8b OS=Rattus norvegicus GN=Lrrc8b PE=1 SV=1 - [D4A758_RAT]
Phytanoyl-CoA dioxygenase domain-containing protein 1 OS=Rattus norvegicus GN=Phyhd1 PE=2 SV=1 - [PHYD1_
Protein phosphatase 1 regulatory subunit 11 OS=Rattus norvegicus GN=Ppp1r11 PE=2 SV=2 - [PP1RB_RAT]
Catenin (Cadherin associated protein), alpha-like 1 (Predicted) OS=Rattus norvegicus GN=Ctnnal1 PE=1 SV=1 - [D
Inositol-3-phosphate synthase 1 OS=Rattus norvegicus GN=Isyna1 PE=3 SV=2 - [INO1_RAT]
Uncharacterized protein C6orf47 homolog OS=Rattus norvegicus GN=G4 PE=1 SV=1 - [CF047_RAT]
Protein phosphatase 1 regulatory subunit 12A OS=Rattus norvegicus GN=Ppp1r12a PE=1 SV=2 - [D4ACS0_RAT]
Protein Dmwd OS=Rattus norvegicus GN=Dmwd PE=1 SV=3 - [F1M3D2_RAT]
Protein FAM65A OS=Rattus norvegicus GN=Fam65a PE=1 SV=2 - [FA65A_RAT]
Dihydrolipoyl dehydrogenase, mitochondrial OS=Rattus norvegicus GN=Dld PE=1 SV=1 - [DLDH_RAT]
Oxysterol-binding protein OS=Rattus norvegicus GN=Osbpl5 PE=1 SV=1 - [A0A0G2JV78_RAT]
Cytochrome b-c1 complex subunit 6, mitochondrial OS=Rattus norvegicus GN=Uqcrh PE=3 SV=1 - [QCR6_RAT]
Cullin-associated NEDD8-dissociated protein 1 OS=Rattus norvegicus GN=Cand1 PE=1 SV=1 - [CAND1_RAT]
Phospholipid phosphatase-related protein type 4 OS=Rattus norvegicus GN=Plppr4 PE=1 SV=1 - [G3V864_RAT]
Protein Cic OS=Rattus norvegicus GN=Cic PE=1 SV=2 - [D4A853_RAT]
Protein Wapl OS=Rattus norvegicus GN=Wapl PE=1 SV=2 - [D4ADT3_RAT]
ATPase, H+ transporting, V1 subunit E isoform 1, isoform CRA_a OS=Rattus norvegicus GN=Atp6v1e1 PE=1 SV=1
Protein Sf3a1 OS=Rattus norvegicus GN=Sf3a1 PE=1 SV=1 - [D3ZQM0_RAT]
Protein Cyfip2 OS=Rattus norvegicus GN=Cyfip2 PE=1 SV=1 - [A0A0G2JT63_RAT]
High mobility group protein B1 OS=Rattus norvegicus GN=Hmgb1 PE=1 SV=2 - [HMGB1_RAT]
Protein Camk1d OS=Rattus norvegicus GN=Camk1d PE=1 SV=2 - [F1LVR4_RAT]
Glutaminase kidney isoform, mitochondrial OS=Rattus norvegicus GN=Gls PE=1 SV=1 - [A0A0G2K1T0_RAT]
Phospholipid-transporting ATPase OS=Rattus norvegicus GN=Atp8a2 PE=1 SV=3 - [D4A3X6_RAT]
BCL2/adenovirus E1B 19 kDa-interacting protein 3 OS=Rattus norvegicus GN=Bnip3 PE=1 SV=1 - [Q9ET45_RAT]
Prolyl endopeptidase-like OS=Rattus norvegicus GN=Prepl PE=1 SV=3 - [D3ZZ32_RAT]
Voltage-gated potassium channel subunit beta-2 OS=Rattus norvegicus GN=Kcnab2 PE=1 SV=1 - [KCAB2_RAT]
Kinesin 13B OS=Rattus norvegicus GN=Kif13b PE=1 SV=1 - [Q70AM4_RAT]
Cadherin-8 OS=Rattus norvegicus GN=Cdh8 PE=1 SV=1 - [CADH8_RAT]
5'-AMP-activated protein kinase catalytic subunit alpha-2 OS=Rattus norvegicus GN=Prkaa2 PE=1 SV=1 - [G3V715
T-type Cav3.1 calcium channel (Fragment) OS=Rattus norvegicus GN=Cacna1g PE=2 SV=1 - [B8XCX0_RAT]
Uncharacterized protein OS=Rattus norvegicus PE=1 SV=2 - [M0R7E6_RAT]
Protein Smdt1 OS=Rattus norvegicus GN=Smdt1 PE=4 SV=1 - [D4A7Y9_RAT]

Glyceraldehyde-3-phosphate dehydrogenase OS=Rattus norvegicus PE=3 SV=3 - [D3ZWV2_RAT]
Serotransferrin OS=Rattus norvegicus GN=Tf PE=1 SV=3 - [TRFE_RAT]
Double C2-like domain-containing protein OS=Rattus norvegicus GN=Doc2b PE=1 SV=1 - [A0A0G2K8B5_RAT]
ATP synthase subunit O, mitochondrial OS=Rattus norvegicus GN=Atp5o PE=1 SV=1 - [ATPO_RAT]
Protein Tra2a OS=Rattus norvegicus GN=Tra2a PE=1 SV=1 - [B1WC25_RAT]
Protein Dhx29 OS=Rattus norvegicus GN=Dhx29 PE=1 SV=2 - [D3ZHW0_RAT]
Protein Sh2d5 OS=Rattus norvegicus GN=Sh2d5 PE=4 SV=2 - [D3ZWT6_RAT]
10 kDa heat shock protein, mitochondrial OS=Rattus norvegicus GN=Hspe1 PE=1 SV=1 - [A0A0G2JTG1_RAT]
Paraspeckle component 1 OS=Rattus norvegicus GN=Pspc1 PE=1 SV=1 - [PSPC1_RAT]
Nucleolin-related protein NRP OS=Rattus norvegicus GN=NRP PE=2 SV=1 - [Q9QZX1_RAT]
Neurotensin receptor type 2 OS=Rattus norvegicus GN=Ntsr2 PE=1 SV=1 - [M0RD53_RAT]
Synaptotagmin III, isoform CRA_a OS=Rattus norvegicus GN=Syt3 PE=4 SV=1 - [A0A0G2JSR2_RAT]
Rat GCP360 OS=Rattus norvegicus GN=Golgb1 PE=2 SV=1 - [Q63714_RAT]
Long-chain-fatty-acid--CoA ligase ACSBG1 OS=Rattus norvegicus GN=Acsbg1 PE=1 SV=1 - [ACBG1_RAT]
Formin-binding protein 1-like OS=Rattus norvegicus GN=Fnbp1l PE=1 SV=2 - [FBP1L_RAT]
Protein disulfide-isomerase A6 OS=Rattus norvegicus GN=Pdia6 PE=1 SV=2 - [PDIA6_RAT]
Harvey ras1 protein (Fragment) OS=Rattus norvegicus GN=Hras PE=4 SV=1 - [Q8CGQ3_RAT]
Protein Ncor2 OS=Rattus norvegicus GN=Ncor2 PE=1 SV=1 - [A0A0G2JU91_RAT]
Dynactin subunit 2 OS=Rattus norvegicus GN=Dctn2 PE=1 SV=1 - [DCTN2_RAT]
Hyaluronan and proteoglycan link protein 1 OS=Rattus norvegicus GN=Hapln1 PE=2 SV=1 - [A1A5N6_RAT]
Voltage-dependent calcium channel gamma-8 subunit OS=Rattus norvegicus GN=Cacng8 PE=1 SV=1 - [F1M7K7_
Protein Tbc1d24 OS=Rattus norvegicus GN=Tbc1d24 PE=1 SV=1 - [A0A0G2K5B0_RAT]
Sodium channel subunit beta-3 OS=Rattus norvegicus GN=Scn3b PE=1 SV=1 - [SCN3B_RAT]
Neurofascin OS=Rattus norvegicus GN=Nfasc PE=1 SV=3 - [D3ZW56_RAT]
Branched-chain-amino-acid aminotransferase OS=Rattus norvegicus GN=Bcat1 PE=2 SV=1 - [Q99JD5_RAT]
Protein LOC100364509 OS=Rattus norvegicus GN=LOC100364509 PE=1 SV=1 - [M0RB65_RAT]
Gamma-aminobutyric acid receptor subunit alpha-3 OS=Rattus norvegicus GN=Gabra3 PE=1 SV=2 - [F1LNZ5_RA
Golgi resident protein GCP60 OS=Rattus norvegicus GN=Acbd3 PE=1 SV=2 - [G3V6E4_RAT]
Endophilin-A1 OS=Rattus norvegicus GN=Sh3gl2 PE=1 SV=2 - [SH3G2_RAT]
Propionyl-CoA carboxylase alpha chain, mitochondrial OS=Rattus norvegicus GN=Pcca PE=1 SV=1 - [A0A0G2K40
Fatty-acid amide hydrolase 1 OS=Rattus norvegicus GN=Faah PE=1 SV=1 - [FAAH1_RAT]
Phospholipid-transporting ATPase OS=Rattus norvegicus GN=Atp11c PE=1 SV=3 - [D3ZFC5_RAT]
Protein Rprd1b OS=Rattus norvegicus GN=Rprd1b PE=1 SV=1 - [B5DEK0_RAT]
Rabgef1 protein (Fragment) OS=Rattus norvegicus GN=Rabgef1 PE=2 SV=1 - [B5DEJ8_RAT]
Sodium/hydrogen exchanger OS=Rattus norvegicus GN=Slc9a6 PE=1 SV=2 - [D3ZJ86_RAT]
Potassium voltage-gated channel subfamily C member 1 OS=Rattus norvegicus GN=Kcnc1 PE=1 SV=1 - [KCNC1_
Protein Deptor OS=Rattus norvegicus GN=Deptor PE=1 SV=2 - [F1M8Y4_RAT]
Myeloid leukemia factor 1 (Predicted), isoform CRA_a OS=Rattus norvegicus GN=Mlf1 PE=1 SV=1 - [D3ZCQ9_RA
Transforming growth factor beta-1-induced transcript 1 protein OS=Rattus norvegicus GN=Tgfb1i1 PE=1 SV=2 - [T
Pre-mRNA-splicing factor SLU7 OS=Rattus norvegicus GN=Slu7 PE=1 SV=1 - [A0A0G2K1S0_RAT]
COMM domain containing 10 OS=Rattus norvegicus GN=Commd10 PE=1 SV=1 - [Q68FS9_RAT]
Adhesion G protein-coupled receptor L3 OS=Rattus norvegicus GN=Adgrl3 PE=1 SV=3 - [D4AAL4_RAT]
Protein Apc2 OS=Rattus norvegicus GN=Apc2 PE=4 SV=1 - [D4A205_RAT]
Phosphatase and tensin homolog, isoform CRA_a OS=Rattus norvegicus GN=Pten PE=1 SV=1 - [O54857_RAT]
ELAV-like protein 2 OS=Rattus norvegicus GN=Elavl2 PE=4 SV=1 - [A0A1B0GWQ5_RAT]
Protein Slc7a11 OS=Rattus norvegicus GN=Slc7a11 PE=1 SV=1 - [D4ADU2_RAT]
Protein Hdac6 OS=Rattus norvegicus GN=Hdac6 PE=1 SV=1 - [A0A0G2QC41_RAT]
Protein RGD1309651 OS=Rattus norvegicus GN=RGD1309651 PE=4 SV=3 - [D3ZSX5_RAT]
B-cell lymphoma/leukemia 10 OS=Rattus norvegicus GN=Bcl10 PE=2 SV=1 - [BCL10_RAT]
Protein phosphatase 1A OS=Rattus norvegicus GN=Ppm1a PE=1 SV=1 - [PPM1A_RAT]
Calcium-regulated heat stable protein 1 OS=Rattus norvegicus GN=Carhsp1 PE=1 SV=1 - [CHSP1_RAT]
Protein Stk26 OS=Rattus norvegicus GN=Stk26 PE=1 SV=2 - [F1LXV3_RAT]
Bromodomain containing 3 (Predicted), isoform CRA_b OS=Rattus norvegicus GN=Brd3 PE=1 SV=2 - [D3ZWU1_R
Protein Rnf214 OS=Rattus norvegicus GN=Rnf214 PE=1 SV=3 - [D4A3V4_RAT]
Long-chain specific acyl-CoA dehydrogenase, mitochondrial OS=Rattus norvegicus GN=Acadl PE=1 SV=1 - [ACAD

Information Classification: General

Centrosomal protein of 41 kDa OS=Rattus norvegicus GN=Cep41 PE=1 SV=1 - [CEP41_RAT]
Phosphatase and actin regulator OS=Rattus norvegicus GN=Phactr4 PE=1 SV=2 - [M0R7T1_RAT]
Protein Raph1 OS=Rattus norvegicus GN=Raph1 PE=1 SV=2 - [D4ADX8_RAT]
Adenylosuccinate synthetase isozyme 2 OS=Rattus norvegicus GN=Adss PE=1 SV=1 - [D4AEP0_RAT]
Protein S100a16 OS=Rattus norvegicus GN=S100a16 PE=1 SV=1 - [B0BMX3_RAT]
Sorting nexin-17 OS=Rattus norvegicus GN=Snx17 PE=1 SV=3 - [F1LMM2_RAT]
Protein RGD1559904 OS=Rattus norvegicus GN=RGD1559904 PE=1 SV=3 - [D3ZKC9_RAT]
Ubiquitin-like protein 3 OS=Rattus norvegicus GN=Ubl3 PE=3 SV=1 - [UBL3_RAT]
PHD finger protein 2 (Predicted) OS=Rattus norvegicus GN=Phf2 PE=1 SV=2 - [F1LWX5_RAT]
Nicolin 1 OS=Rattus norvegicus GN=Nicn1 PE=1 SV=1 - [Q3T1K9_RAT]
Proteasome subunit beta type-3 OS=Rattus norvegicus GN=Psmb3 PE=1 SV=1 - [PSB3_RAT]
Chloride intracellular channel protein OS=Rattus norvegicus GN=Clic4 PE=2 SV=1 - [A3FM27_RAT]
DNA polymerase epsilon subunit 3 OS=Rattus norvegicus GN=Pole3 PE=1 SV=1 - [DPOE3_RAT]
Cadherin-2 OS=Rattus norvegicus GN=Cdh2 PE=1 SV=1 - [G3V803_RAT]
Jouberin OS=Rattus norvegicus GN=Ahi1 PE=1 SV=2 - [F1M9F9_RAT]
Protein Sipa1l3 OS=Rattus norvegicus GN=Sipa1l3 PE=1 SV=3 - [F1LYG2_RAT]
Stathmin-4 OS=Rattus norvegicus GN=Stmn4 PE=1 SV=1 - [STMN4_RAT]
Phosphatidate cytidylyltransferase 1 OS=Rattus norvegicus GN=Cds1 PE=1 SV=2 - [CDS1_RAT]
Protein Fam219a OS=Rattus norvegicus GN=Fam219a PE=1 SV=2 - [D4AAI7_RAT]
Protein Txlna OS=Rattus norvegicus GN=Txlna PE=1 SV=1 - [B2GV14_RAT]
Nuclear factor 1 OS=Rattus norvegicus GN=Nfib PE=1 SV=2 - [O70187_RAT]
Multiple PDZ domain protein OS=Rattus norvegicus GN=Mpdz PE=1 SV=1 - [MPDZ_RAT]
Protein Cadm1 OS=Rattus norvegicus GN=Cadm1 PE=1 SV=1 - [A0A0G2JUT1_RAT]
Dual specificity protein phosphatase 7 (Fragment) OS=Rattus norvegicus GN=Dusp7 PE=2 SV=1 - [DUS7_RAT]
Protein Zfyve19 OS=Rattus norvegicus GN=Zfyve19 PE=1 SV=1 - [Q499R6_RAT]
Protein Gatad2a OS=Rattus norvegicus GN=Gatad2a PE=1 SV=2 - [G3V8R7_RAT]
E3 ubiquitin-protein ligase TRIP12 OS=Rattus norvegicus GN=Trip12 PE=1 SV=2 - [A0A0A0MXY4_RAT]
UBX domain-containing protein 1 OS=Rattus norvegicus GN=Ubxn1 PE=3 SV=2 - [UBXN1_RAT]
Prefoldin subunit 4 OS=Rattus norvegicus GN=Pfdn4 PE=1 SV=2 - [M0R5N4_RAT]
Mothers against decapentaplegic homolog 2 OS=Rattus norvegicus GN=Smad2 PE=1 SV=1 - [SMAD2_RAT]
Ena/VASP-like protein OS=Rattus norvegicus GN=Evl PE=1 SV=1 - [F1M8I7_RAT]
Amyotrophic lateral sclerosis 2 (Juvenile) chromosome region, candidate 13 (Predicted) OS=Rattus norvegicus GN=
Rab GDP dissociation inhibitor beta OS=Rattus norvegicus GN=Gdi2 PE=1 SV=2 - [GDIB_RAT]
Protein Cdh9 OS=Rattus norvegicus GN=Cdh9 PE=1 SV=1 - [D3ZFQ5_RAT]
Cytochrome b-c1 complex subunit 2, mitochondrial OS=Rattus norvegicus GN=Uqcrc2 PE=1 SV=2 - [QCR2_RAT]
Protein RGD1304704 OS=Rattus norvegicus GN=RGD1304704 PE=1 SV=1 - [F7EMB2_RAT]
Arginine-glutamic acid dipeptide repeats protein OS=Rattus norvegicus GN=Rere PE=1 SV=2 - [RERE_RAT]
Unconventional myosin-IXb OS=Rattus norvegicus GN=Myo9b PE=1 SV=1 - [MYO9B_RAT]
Membrane-associated guanylate kinase, WW and PDZ domain-containing protein 3 OS=Rattus norvegicus GN=Ma
Protein Zbtb20 OS=Rattus norvegicus GN=Zbtb20 PE=1 SV=1 - [D4A1U1_RAT]
Epidermal growth factor receptor kinase substrate 8 OS=Rattus norvegicus GN=Eps8 PE=1 SV=2 - [EPS8_RAT]
Protein Gpatch8 OS=Rattus norvegicus GN=Gpatch8 PE=1 SV=3 - [F1M4M5_RAT]
Protein Ankhd1 OS=Rattus norvegicus GN=Ankhd1 PE=1 SV=2 - [E9PTK9_RAT]
Protein Tmem74b OS=Rattus norvegicus GN=Tmem74b PE=1 SV=2 - [F1LVZ8_RAT]
RNA polymerase II-associated protein 1 OS=Rattus norvegicus GN=Rpap1 PE=1 SV=1 - [RPAP1_RAT]
Protein Frmpd4 OS=Rattus norvegicus GN=Frmpd4 PE=4 SV=1 - [D4A3K7_RAT]
Protein Gtf3c4 OS=Rattus norvegicus GN=Gtf3c4 PE=1 SV=1 - [D3ZD80_RAT]
Transcription factor SOX-10 OS=Rattus norvegicus GN=Sox10 PE=1 SV=1 - [SOX10_RAT]
Chloride channel protein OS=Rattus norvegicus GN=Clcn6 PE=1 SV=1 - [D4A3H5_RAT]
Thioredoxin-like protein 1 OS=Rattus norvegicus GN=Txnl1 PE=1 SV=3 - [TXNL1_RAT]
Protein Epm2aip1 OS=Rattus norvegicus GN=Epm2aip1 PE=1 SV=3 - [F1M471_RAT]
Protein Son OS=Rattus norvegicus GN=Son PE=1 SV=2 - [E9PTE1_RAT]
Asc-type amino acid transporter 1 OS=Rattus norvegicus GN=Slc7a10 PE=1 SV=1 - [Q75T81_RAT]
Partitioning defective 6 homolog alpha OS=Rattus norvegicus GN=Pard6a PE=1 SV=2 - [F1LPM7_RAT]
Protein ABHD17B OS=Rattus norvegicus GN=Abhd17b PE=1 SV=1 - [AB17B_RAT]

Phosphomannomutase OS=Rattus norvegicus GN=Pmm1 PE=1 SV=1 - [Q5RK25_RAT]
Arf-GAP with GTPase, ANK repeat and PH domain-containing protein 2 OS=Rattus norvegicus GN=Agap2 PE=1 S\
Serine/threonine-protein phosphatase OS=Rattus norvegicus GN=LOC100362453 PE=3 SV=1 - [A0A0G2JYA4_RA
Kinesin light chain 1 OS=Rattus norvegicus GN=Klc1 PE=1 SV=1 - [A0A140TAB3_RAT]
Uncharacterized protein KIAA0930 homolog OS=Rattus norvegicus PE=1 SV=2 - [K0930_RAT]
Serologically defined breast cancer antigen NY-BR-16-like protein (Fragment) OS=Rattus norvegicus GN=Ankrd17 I
Protein tweety homolog 1 OS=Rattus norvegicus GN=Ttyh1 PE=1 SV=1 - [TTYH1_RAT]
Protein Smarca2 OS=Rattus norvegicus GN=Smarca2 PE=1 SV=1 - [E9PTG1_RAT]
G protein-coupled receptor 162 (Predicted) OS=Rattus norvegicus GN=Gpr162 PE=1 SV=1 - [D4AAS1_RAT]
Hook homolog 1 (Drosophila) (Predicted) OS=Rattus norvegicus GN=Hook1 PE=1 SV=1 - [D3ZB48_RAT]
Protein Prr12 OS=Rattus norvegicus GN=Prr12 PE=1 SV=1 - [A0A0G2K5M6_RAT]
Protein Reps1 OS=Rattus norvegicus GN=Reps1 PE=1 SV=1 - [A0A0G2KB70_RAT]
Protein Cep164 OS=Rattus norvegicus GN=Cep164 PE=4 SV=1 - [D3ZL75_RAT]
Serine/threonine-protein phosphatase OS=Rattus norvegicus GN=Ppp3cb PE=1 SV=1 - [A0A0G2K7T5_RAT]
Leukocyte common antigen OS=Rattus norvegicus GN=Ptprc PE=2 SV=1 - [Q6LDZ3_RAT]
Serum deprivation-response protein OS=Rattus norvegicus GN=Sdpr PE=1 SV=3 - [SDPR_RAT]
Kelch-like 26 (Drosophila) (Predicted), isoform CRA_d OS=Rattus norvegicus GN=Klhl26 PE=1 SV=1 - [D3ZH92_R
Protein LOC680579 OS=Rattus norvegicus GN=LOC680579 PE=4 SV=1 - [D3ZHE6_RAT]
Protein Mtss1 OS=Rattus norvegicus GN=Mtss1 PE=1 SV=1 - [A0A0G2K2D5_RAT]
Protein Crocc OS=Rattus norvegicus GN=Crocc PE=1 SV=2 - [D4AD05_RAT]
Syntaxin-17 OS=Rattus norvegicus GN=Stx17 PE=1 SV=1 - [STX17_RAT]
Crk-like protein OS=Rattus norvegicus GN=Crkl PE=1 SV=1 - [CRKL_RAT]
Golgi associated, gamma adaptin ear containing, ARF binding protein 1 OS=Rattus norvegicus GN=Gga1 PE=1 SV
78 kDa glucose-regulated protein OS=Rattus norvegicus GN=Hspa5 PE=1 SV=1 - [GRP78_RAT]
G protein coupled inward rectifier potassium channel Kir3.2B OS=Rattus norvegicus GN=Kcnj6 PE=2 SV=1 - [Q8R5
Transformer-2 protein homolog beta OS=Rattus norvegicus GN=Tra2b PE=1 SV=1 - [TRA2B_RAT]
Ccnk protein OS=Rattus norvegicus GN=Ccnk PE=1 SV=1 - [A1L1L5_RAT]
RNA-binding protein 10 OS=Rattus norvegicus GN=Rbm10 PE=1 SV=1 - [RBM10_RAT]
Protein Gramd1b OS=Rattus norvegicus GN=Gramd1b PE=1 SV=1 - [D3ZYJ5_RAT]
Oxysterol-binding protein OS=Rattus norvegicus GN=Osbpl8 PE=1 SV=1 - [A0A0G2JXN8_RAT]
Receptor protein serine/threonine kinase OS=Rattus norvegicus GN=Bmpr2 PE=1 SV=2 - [F1LQC5_RAT]
Solute carrier family 30 protein (Fragment) OS=Rattus norvegicus GN=Slc30a3 PE=2 SV=1 - [Q8K4Y6_RAT]
Protein Dhx16 (Fragment) OS=Rattus norvegicus GN=Dhx16 PE=1 SV=1 - [A0A0G2K2E8_RAT]
Nucleolin OS=Rattus norvegicus GN=Ncl PE=1 SV=3 - [NUCL_RAT]
Protein Nckipsd OS=Rattus norvegicus GN=Nckipsd PE=1 SV=3 - [D3ZWX4_RAT]
Protein Ccar2 OS=Rattus norvegicus GN=Ccar2 PE=1 SV=1 - [F1LM55_RAT]
Protein Hars2 OS=Rattus norvegicus GN=Hars2 PE=1 SV=1 - [F1M9C9_RAT]
Protein LOC100910929 OS=Rattus norvegicus GN=Vps37c PE=1 SV=1 - [B5DFF4_RAT]
Protein Sos1 OS=Rattus norvegicus GN=Sos1 PE=1 SV=2 - [D4A3T0_RAT]
Serine/arginine-rich splicing factor 6 OS=Rattus norvegicus GN=Srsf6 PE=1 SV=1 - [SRSF6_RAT]
Leucine zipper protein 1 OS=Rattus norvegicus GN=Luzp1 PE=1 SV=1 - [A0A0G2K101_RAT]
PDZ domain containing 8 (Predicted) OS=Rattus norvegicus GN=Pdzd8 PE=1 SV=1 - [D3ZXY2_RAT]
BEIGE OS=Rattus norvegicus GN=Lyst PE=2 SV=1 - [Q9Z2X9_RAT]
Midline 2 (Predicted) OS=Rattus norvegicus GN=Mid2 PE=4 SV=1 - [A0A0G2JWJ3_RAT]
General receptor for phosphoinositides 1-associated scaffold protein OS=Rattus norvegicus GN=Grasp PE=1 SV=1
Zc3hc1 protein OS=Rattus norvegicus GN=Zc3hc1 PE=1 SV=1 - [B2RYM6_RAT]
Hyaluronoglucosaminidase 3, isoform CRA_d OS=Rattus norvegicus GN=Nat6 PE=1 SV=1 - [A0A0G2JV35_RAT]
Adaptor protein complex AP-2, alpha 1 subunit (Predicted) OS=Rattus norvegicus GN=Ap2a1 PE=1 SV=1 - [D3ZUY
Diacylglycerol kinase gamma OS=Rattus norvegicus GN=Dgkg PE=2 SV=1 - [DGKG_RAT]
Phosphohistidine phosphatase 1 (Predicted), isoform CRA_a OS=Rattus norvegicus GN=Phpt1 PE=1 SV=1 - [D3ZF
Telomerase Cajal body protein 1 OS=Rattus norvegicus GN=Wrap53 PE=1 SV=1 - [WAP53_RAT]
Protein Sphk2 OS=Rattus norvegicus GN=Sphk2 PE=1 SV=1 - [Q6AYB2_RAT]
Interleukin enhancer-binding factor 3 OS=Rattus norvegicus GN=Ilf3 PE=1 SV=1 - [A0A0G2K2T6_RAT]
RanBP-type and C3HC4-type zinc finger containing 1 OS=Rattus norvegicus GN=Rbck1 PE=2 SV=1 - [Q6P7C9_R
Copine 5 protein OS=Rattus norvegicus GN=Cpne5 PE=1 SV=1 - [D3ZGN2_RAT]

Information Classification: General

Core histone macro-H2A OS=Rattus norvegicus GN=H2afy PE=1 SV=1 - [A0A140TAB4_RAT]
Zinc finger and BTB domain containing 16 protein (Fragment) OS=Rattus norvegicus GN=Zbtb16 PE=2 SV=1 - [F7I
ADP-ribosylation factor-like protein 2 OS=Rattus norvegicus GN=Arl2 PE=1 SV=2 - [G3V8V0_RAT]
Procollagen, type IV, alpha 3 (Goodpasture antigen) binding protein (Predicted), isoform CRA_a OS=Rattus norvegi
Protein Mtmr6 OS=Rattus norvegicus GN=Mtmr6 PE=1 SV=1 - [A0A0G2JXT6_RAT]
Vesicular inhibitory amino acid transporter OS=Rattus norvegicus GN=Slc32a1 PE=1 SV=1 - [VIAAT_RAT]
Protein Ildr2 OS=Rattus norvegicus GN=Ildr2 PE=1 SV=2 - [M0R3U3_RAT]
Protein Znrf2 OS=Rattus norvegicus GN=Znrf2 PE=1 SV=1 - [M0RBD9_RAT]
Protein Bod1l1 OS=Rattus norvegicus GN=Bod1l1 PE=1 SV=2 - [M0RC54_RAT]
Protein Tmem87b OS=Rattus norvegicus GN=Tmem87b PE=1 SV=2 - [D4A7B6_RAT]
Protein Ranbp3 OS=Rattus norvegicus GN=Ranbp3 PE=1 SV=2 - [M0R920_RAT]
Protein Myrf OS=Rattus norvegicus GN=Myrf PE=1 SV=2 - [D4A352_RAT]
Dolichyl-diphosphooligosaccharide--protein glycosyltransferase subunit 1 OS=Rattus norvegicus GN=Rpn1 PE=2 S
Integrin alpha 6 subchain (Fragment) OS=Rattus norvegicus GN=Itga6 PE=2 SV=1 - [Q924W2_RAT]
Pgm2 protein OS=Rattus norvegicus GN=Pgm2 PE=1 SV=2 - [F7FLB2_RAT]
TANK-binding kinase 1-binding protein 1 OS=Rattus norvegicus GN=Tbkbp1 PE=2 SV=1 - [TBKB1_RAT]
WD repeat-containing protein 81 OS=Rattus norvegicus GN=Wdr81 PE=3 SV=1 - [WDR81_RAT]
Protein Tfg OS=Rattus norvegicus GN=Tfg PE=1 SV=1 - [Q4R1A4_RAT]
Protein Otud7a OS=Rattus norvegicus GN=Otud7a PE=1 SV=2 - [D4ABZ4_RAT]
Kinase suppressor of ras 1 (Predicted) OS=Rattus norvegicus GN=Ksr1 PE=1 SV=2 - [D3ZHL1_RAT]
Protein Traf3 OS=Rattus norvegicus GN=Traf3 PE=4 SV=1 - [D3Z9G0_RAT]
Secretory carrier membrane protein 1 OS=Rattus norvegicus GN=Scamp1 PE=1 SV=1 - [A0A0G2K1I6_RAT]
SH3 domain-binding protein 4 OS=Rattus norvegicus GN=Sh3bp4 PE=1 SV=1 - [G3V8J0_RAT]
Coiled-coil and C2 domain-containing protein 1B OS=Rattus norvegicus GN=Cc2d1b PE=1 SV=2 - [C2D1B_RAT]
AP-3 complex subunit beta OS=Rattus norvegicus GN=Ap3b1 PE=1 SV=1 - [A0A0G2JWD6_RAT]
Protein Kmt2d OS=Rattus norvegicus GN=Kmt2d PE=1 SV=1 - [A0A0G2JVD6_RAT]
WD repeat, SAM and U-box domain-containing protein 1 OS=Rattus norvegicus GN=Wdsub1 PE=1 SV=1 - [A0A0H
Protein Cul9 OS=Rattus norvegicus GN=Cul9 PE=1 SV=1 - [A0A0G2K652_RAT]
Protein Irs2 OS=Rattus norvegicus GN=Irs2 PE=1 SV=2 - [F1MAL5_RAT]
Clavesin-2 OS=Rattus norvegicus GN=Clvs2 PE=1 SV=1 - [CLVS2_RAT]
Protein Fam208a OS=Rattus norvegicus GN=Fam208a PE=1 SV=2 - [M0RAP6_RAT]
Protein Ccdc92 OS=Rattus norvegicus GN=Ccdc92 PE=1 SV=1 - [D3ZBX9_RAT]
Glycogen phosphorylase, brain form (Fragment) OS=Rattus norvegicus GN=Pygb PE=1 SV=3 - [PYGB_RAT]
Zinc finger and BTB domain containing 7a OS=Rattus norvegicus GN=Zbtb7a PE=1 SV=1 - [G3V8P6_RAT]
Adhesion G-protein-coupled receptor G1 OS=Rattus norvegicus GN=Adgrg1 PE=1 SV=1 - [F1LMW3_RAT]
Protein Sugp2 OS=Rattus norvegicus GN=Sugp2 PE=1 SV=1 - [D3ZJH2_RAT]
Protein Snx30 OS=Rattus norvegicus GN=Snx30 PE=1 SV=2 - [D4A060_RAT]
Programmed cell death protein 4 OS=Rattus norvegicus GN=Pdcd4 PE=1 SV=2 - [PDCD4_RAT]
NADH dehydrogenase (Ubiquinone) Fe-S protein 7 OS=Rattus norvegicus GN=Ndufs7 PE=1 SV=1 - [Q5RJN0_RA
Rab3 GTPase-activating protein catalytic subunit OS=Rattus norvegicus GN=Rab3gap1 PE=1 SV=3 - [F1LP59_RA
Protein Sytl2 OS=Rattus norvegicus GN=Sytl2 PE=1 SV=1 - [B5DFB1_RAT]
Protein Dmxl1 OS=Rattus norvegicus GN=Dmxl1 PE=1 SV=1 - [D4AA13_RAT]
Nuclear speckle splicing regulatory protein 1 OS=Rattus norvegicus GN=Nsrp1 PE=1 SV=1 - [NSRP1_RAT]
Netrin receptor UNC5C OS=Rattus norvegicus GN=Unc5c PE=1 SV=3 - [F1LM73_RAT]
Potassium voltage-gated channel subfamily H member 1 OS=Rattus norvegicus GN=Kcnh1 PE=1 SV=1 - [KCNH1_
Protein Paxbp1 OS=Rattus norvegicus GN=Paxbp1 PE=1 SV=1 - [D4A8C8_RAT]
G protein-coupled receptor 37-like 1, isoform CRA_a OS=Rattus norvegicus GN=Gpr37l1 PE=1 SV=1 - [B4F7C1_R
Profilin-1 OS=Rattus norvegicus GN=Pfn1 PE=1 SV=2 - [PROF1_RAT]
Histamine H3 receptor H3S isoform OS=Rattus norvegicus GN=Hrh3 PE=2 SV=1 - [Q541U0_RAT]
Glutamate receptor interacting protein 1 isoform b (Fragment) OS=Rattus norvegicus GN=Grip1 PE=2 SV=1 - [F5H
Protein Mios OS=Rattus norvegicus GN=Mios PE=1 SV=1 - [D3Z9C0_RAT]
Polymerase I and transcript release factor OS=Rattus norvegicus GN=Ptrf PE=1 SV=1 - [G3V8L9_RAT]
Ubiquitin carboxyl-terminal hydrolase CYLD OS=Rattus norvegicus GN=Cyld PE=1 SV=3 - [F1LPJ6_RAT]
Serine/threonine-protein kinase N2 OS=Rattus norvegicus GN=Pkn2 PE=1 SV=1 - [A0A0G2K6J2_RAT]
Microtubule-associated protein RP/EB family member 3 OS=Rattus norvegicus GN=Mapre3 PE=1 SV=1 - [MARE3_

Protein Prex1 OS=Rattus norvegicus GN=Prex1 PE=1 SV=3 - [D3ZS72_RAT]
Protein Tmem200c OS=Rattus norvegicus GN=Tmem200c PE=1 SV=1 - [M0R5E3_RAT]
Neuronal cell adhesion molecule OS=Rattus norvegicus GN=Nrcam PE=1 SV=1 - [Q6QRP0_RAT]
Neuronal cell adhesion molecule OS=Rattus norvegicus GN=Nrcam PE=1 SV=1 - [A0A0G2K3Q5_RAT]
Acyl-protein thioesterase 2 OS=Rattus norvegicus GN=Lypla2 PE=1 SV=1 - [LYPA2_RAT]
Phosphatase and actin regulator OS=Rattus norvegicus GN=Phactr2 PE=1 SV=1 - [A0A0G2K8Q4_RAT]
Calbindin OS=Rattus norvegicus GN=Calb1 PE=1 SV=2 - [CALB1_RAT]
Spastin OS=Rattus norvegicus GN=Spast PE=1 SV=1 - [A0A0G2K590_RAT]
Protein Slc39a10 OS=Rattus norvegicus GN=Slc39a10 PE=1 SV=1 - [D4A517_RAT]
Tyrosine-protein kinase OS=Rattus norvegicus GN=Abl2 PE=1 SV=2 - [F1M0N1_RAT]
Putative ATP-dependent RNA helicase DHX30 OS=Rattus norvegicus GN=Dhx30 PE=1 SV=1 - [A0A0G2JW51_RA
DNA mismatch repair protein Mlh1 OS=Rattus norvegicus GN=Mlh1 PE=1 SV=1 - [A0A0G2K2L0_RAT]
Ras-related protein Rab-11A OS=Rattus norvegicus GN=Rab11a PE=1 SV=3 - [RB11A_RAT]
Clathrin, light polypeptide (Lca), isoform CRA_d OS=Rattus norvegicus GN=Clta PE=2 SV=1 - [Q5PPP1_RAT]
Zfr protein OS=Rattus norvegicus GN=Zfr PE=1 SV=1 - [B1WC00_RAT]
N-acetyl-D-glucosamine kinase OS=Rattus norvegicus GN=Nagk PE=1 SV=4 - [NAGK_RAT]
CDGSH iron-sulfur domain-containing protein 1 OS=Rattus norvegicus GN=Cisd1 PE=3 SV=1 - [CISD1_RAT]
Calcium/calmodulin-dependent 3',5'-cyclic nucleotide phosphodiesterase 1B OS=Rattus norvegicus GN=Pde1b PE=
Protein Wdr47 OS=Rattus norvegicus GN=Wdr47 PE=1 SV=1 - [G3V9M3_RAT]
NIMA (Never in mitosis gene a)-related expressed kinase 4 OS=Rattus norvegicus GN=Nek4 PE=1 SV=2 - [D3ZDV
Cleft lip and palate associated transmembrane protein 1 OS=Rattus norvegicus GN=Clptm1 PE=1 SV=1 - [B2RYF6_
ESF1 homolog OS=Rattus norvegicus GN=Esf1 PE=1 SV=1 - [ESF1_RAT]
Fas apoptotic inhibitory molecule 1 OS=Rattus norvegicus GN=Faim PE=2 SV=1 - [FAIM1_RAT]
Glutamine--fructose-6-phosphate aminotransferase [isomerizing] 1 OS=Rattus norvegicus GN=Gfpt1 PE=1 SV=3 - [
Equilibrative nucleoside transporter 1 OS=Rattus norvegicus GN=Slc29a1 PE=1 SV=3 - [S29A1_RAT]
Pogo transposable element with ZNF domain (Predicted), isoform CRA_a OS=Rattus norvegicus GN=Pogz PE=1 S
Metal response element binding transcription factor 1 (Predicted) OS=Rattus norvegicus GN=Mtf1 PE=1 SV=1 - [D3
Leucine-rich repeats and IQ motif containing 2 (Predicted), isoform CRA_b OS=Rattus norvegicus GN=Cep97 PE=1
Inositol polyphosphate-5-phosphatase A (Predicted), isoform CRA_a OS=Rattus norvegicus GN=Inpp5a PE=1 SV=
Protein Kctd4 OS=Rattus norvegicus GN=Kctd4 PE=1 SV=1 - [D3ZXL9_RAT]
Proline-rich transmembrane protein 4 OS=Rattus norvegicus GN=Prrt4 PE=3 SV=1 - [PRRT4_RAT]
Nuclear receptor coactivator 1 (Predicted), isoform CRA_b OS=Rattus norvegicus GN=Ncoa1 PE=1 SV=1 - [A0A0G
Protein Foxk1 OS=Rattus norvegicus GN=Foxk1 PE=1 SV=2 - [D3ZU55_RAT]
ATP-binding cassette sub-family B member 8, mitochondrial OS=Rattus norvegicus GN=Abcb8 PE=1 SV=1 - [A0A0
Rho-associated protein kinase OS=Rattus norvegicus GN=Rock2 PE=1 SV=3 - [F1LQT3_RAT]
Sodium-coupled neutral amino acid transporter 1 OS=Rattus norvegicus GN=Slc38a1 PE=1 SV=1 - [S38A1_RAT]
Longevity assurance homolog 2 (S. cerevisiae) OS=Rattus norvegicus GN=Cers2 PE=1 SV=1 - [G3V8V4_RAT]
Adenylate cyclase 8, isoform CRA_a OS=Rattus norvegicus GN=Adcy8 PE=1 SV=1 - [G3V6N0_RAT]
PKC lambda protein (Fragment) OS=Rattus norvegicus GN=Prkci PE=2 SV=1 - [Q9R1X8_RAT]
Transmembrane protein 100 OS=Rattus norvegicus GN=Tmem100 PE=1 SV=1 - [TM100_RAT]
Cbx5 protein OS=Rattus norvegicus GN=Cbx5 PE=1 SV=1 - [B2RYU7_RAT]
Ubiquitin carboxyl-terminal hydrolase 10 OS=Rattus norvegicus GN=Usp10 PE=1 SV=1 - [UBP10_RAT]
PIN2/TERF1-interacting telomerase inhibitor 1 OS=Rattus norvegicus GN=Pinx1 PE=1 SV=1 - [PINX1_RAT]
DNA (cytosine-5)-methyltransferase 3A OS=Rattus norvegicus GN=Dnmt3a PE=1 SV=1 - [DNM3A_RAT]
Mpp6 protein OS=Rattus norvegicus GN=Mpp6 PE=1 SV=1 - [B5DFE0_RAT]
Glutamate--cysteine ligase catalytic subunit OS=Rattus norvegicus GN=Gclc PE=1 SV=2 - [GSH1_RAT]
Rbm5 protein (Fragment) OS=Rattus norvegicus GN=Rbm5 PE=2 SV=1 - [Q5EB71_RAT]
Protein Dzip3 OS=Rattus norvegicus GN=Dzip3 PE=1 SV=3 - [D4A1V8_RAT]
Regulator of G-protein-signaling 6 OS=Rattus norvegicus GN=Rgs6 PE=1 SV=3 - [F1LS67_RAT]
Filamin alpha OS=Rattus norvegicus GN=Flna PE=1 SV=1 - [C0JPT7_RAT]
Anoctamin OS=Rattus norvegicus GN=Ano8 PE=1 SV=1 - [A0A0G2K5K1_RAT]
Phosphatidylinositol 4-phosphate 5-kinase type-1 gamma OS=Rattus norvegicus GN=Pip5k1c PE=1 SV=1 - [F1M8H
Protein Pcmtd1 OS=Rattus norvegicus GN=Pcmtd1 PE=1 SV=1 - [D4A629_RAT]
Protein Mul1 OS=Rattus norvegicus GN=Mul1 PE=1 SV=1 - [D4A1H7_RAT]
Protein Bcl2l13 OS=Rattus norvegicus GN=Bcl2l13 PE=1 SV=3 - [D3ZT71_RAT]

Information Classification: General

Anaphase promoting complex subunit 1 (Predicted) OS=Rattus norvegicus GN=Anapc1 PE=1 SV=2 - [F1M801_RAT
Protein Synpo2 OS=Rattus norvegicus GN=Synpo2 PE=1 SV=2 - [D4A702_RAT]
Zinc finger protein 830 OS=Rattus norvegicus GN=Znf830 PE=1 SV=1 - [ZN830_RAT]
Oligodendrocyte-myelin glycoprotein OS=Rattus norvegicus PE=2 SV=1 - [Q7TNM3_RAT]
[3-methyl-2-oxobutanoate dehydrogenase [lipoamide]] kinase, mitochondrial OS=Rattus norvegicus GN=Bckdk PE=
Ac1288 OS=Rattus norvegicus GN=Hook3 PE=1 SV=1 - [Q7TQ77_RAT]
Sec1 family domain-containing protein 1 OS=Rattus norvegicus GN=Scfd1 PE=1 SV=1 - [SCFD1_RAT]
Protein Tns1 OS=Rattus norvegicus GN=Tns1 PE=1 SV=2 - [F1LN42_RAT]
Protein Epha4 OS=Rattus norvegicus GN=Epha4 PE=3 SV=1 - [D3ZZK3_RAT]
M-phase phosphoprotein 8 OS=Rattus norvegicus GN=Mphosph8 PE=2 SV=1 - [MPP8_RAT]
Protein LOC103690005 OS=Rattus norvegicus GN=LOC103690005 PE=1 SV=1 - [Q1RP74_RAT]
Protein Ppp1r12c OS=Rattus norvegicus GN=Ppp1r12c PE=1 SV=1 - [A0A0G2K4R1_RAT]
Receptor expression-enhancing protein OS=Rattus norvegicus GN=Reep3 PE=2 SV=1 - [B0BNL5_RAT]
Arginine/serine-rich coiled-coil protein 2 OS=Rattus norvegicus GN=Rsrc2 PE=1 SV=1 - [A0A0G2JZB4_RAT]
UBR1 protein OS=Rattus norvegicus GN=Ubr1 PE=2 SV=1 - [D5MTG9_RAT]
Protein Tbc1d2b OS=Rattus norvegicus GN=Tbc1d2b PE=1 SV=2 - [D3ZAF7_RAT]
Leiomodin-1 OS=Rattus norvegicus GN=Lmod1 PE=1 SV=1 - [LMOD1_RAT]
Ubiquitin carboxyl-terminal hydrolase 15 OS=Rattus norvegicus GN=Usp15 PE=1 SV=1 - [A0A0G2K1U1_RAT]
Transcription factor BTF3 OS=Rattus norvegicus PE=3 SV=1 - [A0A0G2K4W2_RAT]
E3 ubiquitin-protein ligase OS=Rattus norvegicus GN=Wwp2 PE=1 SV=1 - [F7EYF1_RAT]
Protein Chd3 OS=Rattus norvegicus GN=Chd3 PE=1 SV=3 - [F1LPP8_RAT]
Coronin OS=Rattus norvegicus GN=Coro2b PE=3 SV=3 - [F1LMV9_RAT]
Protein Strip2 OS=Rattus norvegicus GN=Strip2 PE=1 SV=1 - [A0A0G2JT26_RAT]
Protein Ap4e1 OS=Rattus norvegicus GN=Ap4e1 PE=1 SV=2 - [D3ZX21_RAT]
Probable lysosomal cobalamin transporter OS=Rattus norvegicus GN=Lmbrd1 PE=1 SV=1 - [LMBD1_RAT]
Protein RGD1565616 OS=Rattus norvegicus GN=RGD1565616 PE=1 SV=1 - [D3ZVN5_RAT]
Protein Rbm33 OS=Rattus norvegicus GN=Rbm33 PE=1 SV=1 - [D3ZTA8_RAT]
Protein Arhgap33 OS=Rattus norvegicus GN=Arhgap33 PE=1 SV=3 - [D4A9G6_RAT]
Dctn4 protein OS=Rattus norvegicus GN=Dctn4 PE=2 SV=1 - [Q498N3_RAT]
Protein Shisa7 OS=Rattus norvegicus GN=Shisa7 PE=1 SV=1 - [D3ZPJ0_RAT]
Protein Cpeb3 OS=Rattus norvegicus GN=Cpeb3 PE=1 SV=3 - [D4AD99_RAT]
Isochorismatase domain-containing protein 1 OS=Rattus norvegicus GN=LOC103694869 PE=1 SV=1 - [F2Z3T7_R
Parvalbumin alpha OS=Rattus norvegicus GN=Pvalb PE=1 SV=2 - [PRVA_RAT]
Sodium/potassium-transporting ATPase subunit beta-1 OS=Rattus norvegicus GN=Atp1b1 PE=1 SV=1 - [AT1B1_R
Carbonyl reductase [NADPH] 1 OS=Rattus norvegicus GN=Cbr1 PE=1 SV=1 - [A0A0G2JSV2_RAT]
Mu opioid receptor splice variant MOR-1G1 OS=Rattus norvegicus GN=Oprm1 PE=2 SV=1 - [B8K2Q4_RAT]
Protein Ssh1 OS=Rattus norvegicus GN=Ssh1 PE=1 SV=3 - [F1LWM1_RAT]
PHD finger protein 10 OS=Rattus norvegicus GN=Phf10 PE=1 SV=2 - [PHF10_RAT]
Protein Ube2j1 (Fragment) OS=Rattus norvegicus GN=Ube2j1 PE=1 SV=1 - [A0A096MJH0_RAT]
Twinfilin-1 OS=Rattus norvegicus GN=Twf1 PE=1 SV=1 - [TWF1_RAT]
Brain-enriched SH3-domain protein OS=Rattus norvegicus GN=Ngef PE=2 SV=1 - [Q9JK73_RAT]
Cubilin OS=Rattus norvegicus GN=Cubn PE=1 SV=3 - [F1LNU2_RAT]
Protein Ckap5 OS=Rattus norvegicus GN=Ckap5 PE=1 SV=3 - [F1M949_RAT]
Protein Tbc1d22a OS=Rattus norvegicus GN=Tbc1d22a PE=1 SV=3 - [D3Z994_RAT]
Sfrs4 protein (Fragment) OS=Rattus norvegicus GN=Srsf4 PE=2 SV=1 - [B1H240_RAT]
Receptor protein-tyrosine kinase OS=Rattus norvegicus GN=Erbb3 PE=1 SV=1 - [G3V6N1_RAT]
Sept9 protein (Fragment) OS=Rattus norvegicus GN=Sept9 PE=2 SV=1 - [B2GVB4_RAT]
Dynamin-2 OS=Rattus norvegicus GN=Dnm2 PE=1 SV=2 - [A0A0A0MY48_RAT]
Sodium- and chloride-dependent GABA transporter 1 OS=Rattus norvegicus GN=Slc6a1 PE=1 SV=1 - [SC6A1_RAT
Ubiquitin-associated domain-containing protein 1 OS=Rattus norvegicus GN=Ubac1 PE=1 SV=1 - [UBAC1_RAT]
Uncharacterized protein OS=Rattus norvegicus PE=4 SV=1 - [A0A0G2K3Z9_RAT]
Protein Carmil2 OS=Rattus norvegicus GN=Carmil2 PE=1 SV=2 - [D3ZC15_RAT]
Fam84a protein OS=Rattus norvegicus GN=Fam84a PE=2 SV=1 - [B2RYB4_RAT]
Palladin (Fragment) OS=Rattus norvegicus GN=Palld PE=1 SV=1 - [PALLD_RAT]
Protein Zfp521 OS=Rattus norvegicus GN=Zfp521 PE=1 SV=2 - [D3ZQM2_RAT]

Information Classification: General

Protein Nrbp2 OS=Rattus norvegicus GN=Nrbp2 PE=1 SV=1 - [A0A0G2JT23_RAT]
Nischarin OS=Rattus norvegicus GN=Nisch PE=2 SV=2 - [NISCH_RAT]
Protein Plekha5 OS=Rattus norvegicus GN=Plekha5 PE=1 SV=2 - [E9PTG5_RAT]
Cyclin-dependent kinase-like 5 OS=Rattus norvegicus GN=Cdkl5 PE=1 SV=1 - [E2E1S0_RAT]
Protein Tango2 OS=Rattus norvegicus GN=Tango2 PE=1 SV=1 - [D3ZY86_RAT]
LanC lantibiotic synthetase component C-like 2 (Bacterial) OS=Rattus norvegicus GN=Lancl2 PE=1 SV=1 - [Q68FQ
Eyes absent homolog OS=Rattus norvegicus GN=Eya1 PE=3 SV=1 - [A0A0G2JWT3_RAT]
Cell surface glycoprotein MUC18 OS=Rattus norvegicus GN=Mcam PE=1 SV=2 - [MUC18_RAT]
Regulator of G-protein signaling 12, isoform CRA_b OS=Rattus norvegicus GN=Rgs12 PE=1 SV=1 - [A0A0G2K326
40S ribosomal protein S3a OS=Rattus norvegicus GN=LOC100365839 PE=3 SV=1 - [M0R6L4_RAT]
Complement component 1 Q subcomponent-binding protein, mitochondrial OS=Rattus norvegicus GN=C1qbp PE=1
GTP-binding regulatory protein G42 alpha chain (Fragments) OS=Rattus norvegicus PE=4 SV=1 - [Q7M0H6_RAT]
Sarcolemmal membrane-associated protein OS=Rattus norvegicus GN=Slmap PE=1 SV=1 - [B5DF63_RAT]
Chloride channel protein OS=Rattus norvegicus GN=Clcn2 PE=3 SV=2 - [E9PU32_RAT]
Rho guanine nucleotide exchange factor 6 OS=Rattus norvegicus GN=Arhgef6 PE=1 SV=1 - [ARHG6_RAT]
Dystrobrevin OS=Rattus norvegicus GN=Dtnb PE=1 SV=1 - [A0A0G2JVM6_RAT]
Tyrosine-protein phosphatase non-receptor type 11 OS=Rattus norvegicus GN=Ptpn11 PE=1 SV=4 - [PTN11_RAT]
Protein Fam13c OS=Rattus norvegicus GN=Fam13c PE=1 SV=3 - [D3ZD52_RAT]
A-kinase anchor protein 13 OS=Rattus norvegicus GN=Akap13 PE=1 SV=2 - [AKP13_RAT]
Coiled-coil domain-containing protein 86 OS=Rattus norvegicus GN=Ccdc86 PE=1 SV=1 - [CCD86_RAT]
Elongation factor 2 OS=Rattus norvegicus GN=Eef2 PE=1 SV=4 - [EF2_RAT]
Protein Ube2m OS=Rattus norvegicus GN=Ube2m PE=1 SV=1 - [D3ZNQ6_RAT]
cAMP-dependent protein kinase type I-beta regulatory subunit OS=Rattus norvegicus GN=Prkar1b PE=2 SV=2 - [K
Gamma-tubulin complex component OS=Rattus norvegicus GN=Tubgcp3 PE=1 SV=1 - [D3ZMR7_RAT]
Galectin OS=Rattus norvegicus GN=Lgalsl PE=1 SV=1 - [B4F7A3_RAT]
Ras GTPase-activating protein 2 OS=Rattus norvegicus GN=Rasa2 PE=2 SV=1 - [RASA2_RAT]
Mitochondrial dynamics protein MID51 OS=Rattus norvegicus GN=Mief1 PE=1 SV=1 - [A0A0U1RS39_RAT]
Peripherin OS=Rattus norvegicus GN=Prph PE=2 SV=1 - [Q496Z5_RAT]
Protein Dock4 OS=Rattus norvegicus GN=Dock4 PE=1 SV=2 - [M0R6K4_RAT]
Uncharacterized protein OS=Rattus norvegicus PE=4 SV=1 - [A0A0G2K5A4_RAT]
Protein Naa10 OS=Rattus norvegicus GN=Naa10 PE=1 SV=1 - [D3ZUQ2_RAT]
CPG2 protein OS=Rattus norvegicus GN=cpg2 PE=2 SV=1 - [Q63128_RAT]
Protein LOC100909464 OS=Rattus norvegicus GN=LOC100909464 PE=1 SV=2 - [F1MAA3_RAT]
Phosphoribosyl pyrophosphate synthase-associated protein 1 OS=Rattus norvegicus GN=Prpsap1 PE=1 SV=1 - [K
Rho-associated protein kinase OS=Rattus norvegicus GN=Rock1 PE=1 SV=2 - [D3ZN37_RAT]
Anaphase-promoting complex subunit CDC26 OS=Rattus norvegicus GN=Cdc26 PE=1 SV=1 - [CDC26_RAT]
Protein Zcchc6 OS=Rattus norvegicus GN=Zcchc6 PE=1 SV=3 - [D3ZKR9_RAT]
Microtubule-associated protein RP/EB family member 2 OS=Rattus norvegicus GN=Mapre2 PE=1 SV=2 - [M0R8A4
Alanine--tRNA ligase, cytoplasmic OS=Rattus norvegicus GN=Aars PE=1 SV=3 - [SYAC_RAT]
UV excision repair protein RAD23 homolog B OS=Rattus norvegicus GN=Rad23b PE=1 SV=1 - [RD23B_RAT]
Phosphatidylinositol 3-kinase-associated protein (Fragment) OS=Rattus norvegicus PE=2 SV=1 - [F7IXA1_RAT]
Calretinin OS=Rattus norvegicus GN=Calb2 PE=1 SV=1 - [CALB2_RAT]
Pre-mRNA 3'-end-processing factor FIP1 OS=Rattus norvegicus GN=Fip1l1 PE=1 SV=1 - [FIP1_RAT]
Lysosomal ATPase OS=Rattus norvegicus GN=Atp6G PE=4 SV=1 - [Q811A7_RAT]
Glutathione S-transferase OS=Rattus norvegicus GN=Gstm1 PE=1 SV=2 - [G3V983_RAT]
Protein Rprd1a OS=Rattus norvegicus GN=Rprd1a PE=1 SV=1 - [D4AAU4_RAT]
Wdr20a protein (Fragment) OS=Rattus norvegicus GN=Wdr20 PE=2 SV=1 - [Q5BJW6_RAT]
Protein RGD1559896 OS=Rattus norvegicus GN=RGD1559896 PE=1 SV=1 - [D3ZY47_RAT]
Protein Elfn2 OS=Rattus norvegicus GN=Elfn2 PE=1 SV=3 - [D3ZH36_RAT]
Protein Snw1 OS=Rattus norvegicus GN=Snw1 PE=1 SV=1 - [D4A8G7_RAT]
Signal sequence receptor, alpha OS=Rattus norvegicus GN=Ssr1 PE=1 SV=1 - [Q4V7D1_RAT]
Protein Aifm3 OS=Rattus norvegicus GN=Aifm3 PE=1 SV=1 - [D3ZF03_RAT]
Txndc13 protein (Fragment) OS=Rattus norvegicus GN=Tmx4 PE=2 SV=1 - [Q52KK2_RAT]
tRNA-dihydrouridine(47) synthase [NAD(P)(+)]-like OS=Rattus norvegicus GN=Dus3l PE=2 SV=1 - [DUS3L_RAT]
Protein Ccdc186 OS=Rattus norvegicus GN=Ccdc186 PE=1 SV=1 - [A0A0G2K8K1_RAT]

V-type proton ATPase subunit a (Fragment) OS=Rattus norvegicus GN=Atp6v0a2 PE=2 SV=1 - [Q4G036_RAT]
Sodium channel beta 2 subunit OS=Rattus norvegicus GN=Scn2b PE=4 SV=1 - [Q62861_RAT]
Protein Radil OS=Rattus norvegicus GN=Radil PE=1 SV=2 - [D4A1Z8_RAT]
Charged multivesicular body protein 3 OS=Rattus norvegicus GN=Chmp3 PE=1 SV=3 - [CHMP3_RAT]
Protein inhibitor of activated STAT-1 OS=Rattus norvegicus GN=Pias1 PE=2 SV=1 - [A9UK05_RAT]
Protein Knop1 OS=Rattus norvegicus GN=Knop1 PE=1 SV=1 - [M0R963_RAT]
Protein Cstf3 OS=Rattus norvegicus GN=Cstf3 PE=1 SV=3 - [F1M4W7_RAT]
Protein Nup160 OS=Rattus norvegicus GN=Nup160 PE=1 SV=3 - [D3ZBL6_RAT]
Actin related protein 2/3 complex, subunit 2 (Predicted), isoform CRA_a OS=Rattus norvegicus GN=Arpc2 PE=1 SV
Protein kinase C zeta type OS=Rattus norvegicus GN=Prkcz PE=1 SV=2 - [KPCZ_RAT]
Protein LOC100910056 OS=Rattus norvegicus GN=LOC100910056 PE=1 SV=2 - [M0R8V0_RAT]
Maspardin OS=Rattus norvegicus GN=Spg21 PE=2 SV=1 - [SPG21_RAT]
Protein Usp13 OS=Rattus norvegicus GN=Usp13 PE=2 SV=2 - [D3ZDI9_RAT]
Protein Rbm25 OS=Rattus norvegicus GN=Rbm25 PE=4 SV=2 - [F1LT30_RAT]
Ras-specific guanine nucleotide-releasing factor 2 OS=Rattus norvegicus GN=Rasgrf2 PE=1 SV=1 - [RGRF2_RAT]
ATP-binding cassette sub-family D member 3 OS=Rattus norvegicus GN=Abcd3 PE=1 SV=3 - [ABCD3_RAT]
Protein Plxnc1 OS=Rattus norvegicus GN=Plxnc1 PE=1 SV=3 - [D4A7M0_RAT]
Solute carrier family 2, facilitated glucose transporter member 8 OS=Rattus norvegicus GN=Slc2a8 PE=1 SV=1 - [G
Protein Fmnl2 OS=Rattus norvegicus GN=Fmnl2 PE=1 SV=1 - [A0A0G2K132_RAT]
Ras GTPase-activating protein 3 OS=Rattus norvegicus GN=Rasa3 PE=1 SV=1 - [A0A0G2JW85_RAT]
Ab1-188 OS=Rattus norvegicus GN=Slc20a2 PE=1 SV=1 - [Q7TP93_RAT]
Protein Ppa2 OS=Rattus norvegicus GN=Ppa2 PE=1 SV=2 - [D4A830_RAT]
Protein Bcl11a OS=Rattus norvegicus GN=Bcl11a PE=1 SV=1 - [A0A0G2K4M7_RAT]
POU domain, class 3, transcription factor 4 OS=Rattus norvegicus GN=Pou3f4 PE=2 SV=1 - [PO3F4_RAT]
Protein Frmd4a OS=Rattus norvegicus GN=Frmd4a PE=1 SV=1 - [A0A0G2K2R0_RAT]
Potassium voltage-gated channel subfamily C member 2 OS=Rattus norvegicus GN=Kcnc2 PE=1 SV=1 - [KCNC2_
Protein Ankrd17 OS=Rattus norvegicus GN=Ankrd17 PE=1 SV=1 - [D4A0B4_RAT]
Serine/threonine kinase 3 (STE20 homolog, yeast) OS=Rattus norvegicus GN=Stk3 PE=1 SV=1 - [B1WBQ5_RAT]
Protein Ric1 OS=Rattus norvegicus GN=Ric1 PE=1 SV=1 - [D4A224_RAT]
Protein Wdfy3 OS=Rattus norvegicus GN=Wdfy3 PE=1 SV=1 - [A0A0G2K9M4_RAT]
ATP-binding cassette 1 OS=Rattus norvegicus GN=Abca1 PE=2 SV=1 - [Q80ZB2_RAT]
Protein Spata2 OS=Rattus norvegicus GN=Spata2 PE=1 SV=1 - [Q66HP6_RAT]
Cytoskeleton-associated protein 4 (Predicted) OS=Rattus norvegicus GN=Ckap4 PE=1 SV=2 - [D3ZH41_RAT]
Ogfr protein OS=Rattus norvegicus GN=Ogfr PE=1 SV=1 - [Q3MID9_RAT]
Protein Cgnl1 OS=Rattus norvegicus GN=Cgnl1 PE=1 SV=1 - [D4A3V5_RAT]
Protein Morc2 OS=Rattus norvegicus GN=Morc2 PE=1 SV=2 - [D4A2C4_RAT]
Protein Dock5 OS=Rattus norvegicus GN=Dock5 PE=1 SV=2 - [F1LVA9_RAT]
Protein Mettl22 OS=Rattus norvegicus GN=Mettl22 PE=1 SV=1 - [D3Z9R2_RAT]
Alpha-centractin OS=Rattus norvegicus GN=Actr1a PE=1 SV=1 - [ACTZ_RAT]
Neuronal membrane glycoprotein M6-b OS=Rattus norvegicus GN=Gpm6b PE=4 SV=2 - [E9PSV8_RAT]
GMP synthase [glutamine-hydrolyzing] (Fragment) OS=Rattus norvegicus GN=Gmps PE=1 SV=1 - [A0A096MJ75_F
Cyclic AMP-dependent transcription factor ATF-2 OS=Rattus norvegicus GN=Atf2 PE=1 SV=2 - [ATF2_RAT]
Protein LOC100910540 OS=Rattus norvegicus GN=LOC100910540 PE=1 SV=1 - [G3V656_RAT]
Acyl-CoA-binding domain-containing protein 6 OS=Rattus norvegicus GN=Acbd6 PE=1 SV=1 - [ACBD6_RAT]
Ras association domain-containing protein 5 OS=Rattus norvegicus GN=Rassf5 PE=1 SV=1 - [RASF5_RAT]
WD repeat domain phosphoinositide-interacting protein 2 OS=Rattus norvegicus GN=Wipi2 PE=1 SV=1 - [WIPI2_R
Protein-serine/threonine kinase OS=Rattus norvegicus GN=Grk3 PE=3 SV=1 - [A0A0G2JZ82_RAT]
Protein Rrbp1 OS=Rattus norvegicus GN=Rrbp1 PE=1 SV=3 - [F1M853_RAT]
Chromogranin A OS=Rattus norvegicus GN=Chga PE=4 SV=1 - [F8QYX1_RAT]
Ppp4r2 protein OS=Rattus norvegicus GN=Ppp4r2 PE=1 SV=1 - [B2RZ73_RAT]
Protein Tmem63b OS=Rattus norvegicus GN=Tmem63b PE=1 SV=2 - [D4A105_RAT]
Protein Ccp110 OS=Rattus norvegicus GN=Ccp110 PE=1 SV=3 - [D4A3J9_RAT]
Protein Tbck OS=Rattus norvegicus GN=Tbck PE=1 SV=1 - [D3ZA89_RAT]
Protein Srpk2 OS=Rattus norvegicus GN=Srpk2 PE=1 SV=1 - [B1WBT4_RAT]
Mitochondrial 2-oxoglutarate/malate carrier protein OS=Rattus norvegicus GN=Slc25a11 PE=1 SV=1 - [G3V6H5_R/

Information Classification: General

Sodium- and chloride-dependent GABA transporter 3 OS=Rattus norvegicus GN=Slc6a11 PE=1 SV=1 - [S6A11_RA
Protein DEK OS=Rattus norvegicus GN=Dek PE=1 SV=1 - [DEK_RAT]
Calcium/calmodulin-dependent protein kinase type 1B OS=Rattus norvegicus GN=Pnck PE=1 SV=1 - [KCC1B_RAT
Glutamate decarboxylase 2 OS=Rattus norvegicus GN=Gad2 PE=1 SV=1 - [DCE2_RAT]
Cleavage and polyadenylation specific factor 2 (Predicted) OS=Rattus norvegicus GN=Cpsf2 PE=1 SV=1 - [D3Z9E6
Rho GTPase-activating protein 17 OS=Rattus norvegicus GN=Arhgap17 PE=1 SV=3 - [D4AAV2_RAT]
Bmp2k protein (Fragment) OS=Rattus norvegicus GN=Bmp2k PE=2 SV=1 - [Q4G045_RAT]
Protein FAM122A OS=Rattus norvegicus GN=Fam122a PE=1 SV=1 - [F122A_RAT]
Protein LOC100362987 OS=Rattus norvegicus GN=LOC100362987 PE=3 SV=1 - [M0RA26_RAT]
Receptor expression-enhancing protein OS=Rattus norvegicus GN=Reep1 PE=1 SV=1 - [D4A193_RAT]
Calcium-activated potassium channel subunit alpha-1 OS=Rattus norvegicus GN=Kcnma1 PE=1 SV=3 - [F1LNC7_I
High-mobility group nucleosome binding domain 1 OS=Rattus norvegicus GN=LOC100911295 PE=1 SV=1 - [Q5U1
TIP41-like protein OS=Rattus norvegicus GN=Tiprl PE=1 SV=1 - [TIPRL_RAT]
Heterogeneous nuclear ribonucleoprotein H3 (2H9) (Predicted), isoform CRA_c OS=Rattus norvegicus GN=Hnrnph
Mitochondrial fission regulator 1-like OS=Rattus norvegicus GN=Mtfr1l PE=1 SV=1 - [MFR1L_RAT]
Zinc finger protein 148 OS=Rattus norvegicus GN=Znf148 PE=1 SV=1 - [ZN148_RAT]
Peptidyl-glycine alpha-amidating monooxygenase OS=Rattus norvegicus GN=Pam PE=1 SV=1 - [AMD_RAT]
Protein Ehbp1 OS=Rattus norvegicus GN=Ehbp1 PE=1 SV=3 - [F1LVX2_RAT]
Death-associated kinase 2 OS=Rattus norvegicus GN=Dapk2 PE=1 SV=1 - [Q5BJN2_RAT]
Thymosin beta-4 OS=Rattus norvegicus GN=Tmsb4x PE=1 SV=2 - [TYB4_RAT]
Protein Josd2 OS=Rattus norvegicus GN=Josd2 PE=4 SV=1 - [D4AD44_RAT]
Protein Pum2 OS=Rattus norvegicus GN=Pum2 PE=1 SV=1 - [A0A0G2K8F9_RAT]
Regulator of G-protein signaling 19 OS=Rattus norvegicus GN=Rgs19 PE=1 SV=1 - [RGS19_RAT]
Protein Atg16l1 OS=Rattus norvegicus GN=Atg16l1 PE=1 SV=1 - [D3ZFK6_RAT]
Protein Raly OS=Rattus norvegicus GN=Raly PE=1 SV=1 - [Q5PQR0_RAT]
Protein Sh3pxd2a OS=Rattus norvegicus GN=Sh3pxd2a PE=1 SV=1 - [D3ZMW5_RAT]
Protein Tcof1 OS=Rattus norvegicus GN=Tcof1 PE=1 SV=2 - [D4A206_RAT]
Protein Sergef OS=Rattus norvegicus GN=Sergef PE=1 SV=2 - [D3ZN16_RAT]
Nexilin OS=Rattus norvegicus GN=Nexn PE=1 SV=1 - [NEXN_RAT]
Protein Rab22a OS=Rattus norvegicus GN=Rab22a PE=1 SV=1 - [A0A0G2K3Z3_RAT]
Protein Anks1a OS=Rattus norvegicus GN=Anks1a PE=1 SV=1 - [A0A0G2K2G7_RAT]
Transporter OS=Rattus norvegicus GN=Slc6a7 PE=1 SV=1 - [G3V8F1_RAT]
Protein Xrcc4 OS=Rattus norvegicus GN=Xrcc4 PE=1 SV=1 - [Q5XI44_RAT]
Protein Slc35d3 OS=Rattus norvegicus GN=Slc35d3 PE=1 SV=1 - [D4A5L7_RAT]
Protein Dbndd2 OS=Rattus norvegicus GN=Dbndd2 PE=1 SV=1 - [Q331S7_RAT]
Serine/arginine-rich splicing factor 2 OS=Rattus norvegicus GN=Srsf2 PE=1 SV=3 - [SRSF2_RAT]
Bcl-2-interacting death suppressor OS=Rattus norvegicus PE=2 SV=1 - [Q156J1_RAT]
Voltage-dependent calcium channel gamma-3 subunit OS=Rattus norvegicus GN=Cacng3 PE=1 SV=1 - [CCG3_RA
Protein Srsf7 OS=Rattus norvegicus GN=Srsf7 PE=1 SV=1 - [D4A720_RAT]
Rabphilin-3A OS=Rattus norvegicus GN=Rph3a PE=1 SV=1 - [F1LPB9_RAT]
Protein Ttc9 OS=Rattus norvegicus GN=Ttc9 PE=1 SV=1 - [D3ZS76_RAT]
Protein Zmym3 (Fragment) OS=Rattus norvegicus GN=Zmym3 PE=1 SV=1 - [A0A096MK35_RAT]
Protein Lsm14a OS=Rattus norvegicus GN=Lsm14a PE=1 SV=1 - [A0A0G2JUK2_RAT]
Protein Krt81 OS=Rattus norvegicus GN=Krt81 PE=2 SV=1 - [A7M775_RAT]
Uracil nucleotide/cysteinyl leukotriene receptor OS=Rattus norvegicus GN=Gpr17 PE=2 SV=1 - [GPR17_RAT]
Casein kinase 1 epsilon OS=Rattus norvegicus GN=Csnk1e PE=1 SV=1 - [Q9JJ76_RAT]
Ankyrin repeat and sterile alpha motif domain-containing protein 1B OS=Rattus norvegicus GN=Anks1b PE=1 SV=3
Ripk2 protein (Fragment) OS=Rattus norvegicus GN=Ripk2 PE=2 SV=1 - [Q3B7U0_RAT]
Protein YIPF1 OS=Rattus norvegicus GN=Yipf1 PE=2 SV=1 - [YIPF1_RAT]
TSC22 domain family protein 3 OS=Rattus norvegicus GN=Tsc22d3 PE=1 SV=1 - [T22D3_RAT]
Urea transporter 1 OS=Rattus norvegicus GN=Slc14a1 PE=2 SV=2 - [UT1_RAT]
Septin 6 (Predicted), isoform CRA_b OS=Rattus norvegicus GN=Sept11 PE=1 SV=1 - [A0A0G2JUL7_RAT]
Protein Sf3b2 OS=Rattus norvegicus GN=Sf3b2 PE=1 SV=3 - [D3ZMS1_RAT]
Protein Jakmip2 OS=Rattus norvegicus GN=Jakmip2 PE=1 SV=1 - [A0A0G2JT21_RAT]
Sterol regulatory element-binding protein cleavage-activating protein OS=Rattus norvegicus GN=Scap PE=2 SV=1 -

Information Classification: General

Growth factor receptor-bound protein 10 OS=Rattus norvegicus GN=Grb10 PE=3 SV=1 - [GRB10_RAT]
Protein Srsf11 OS=Rattus norvegicus GN=Srsf11 PE=1 SV=1 - [A0A0G2QC38_RAT]
RNA-binding protein Musashi homolog 1 OS=Rattus norvegicus GN=Msi1 PE=2 SV=1 - [MSI1H_RAT]
Protein FAM110B OS=Rattus norvegicus GN=Fam110b PE=2 SV=1 - [F110B_RAT]
Protein Smap2 OS=Rattus norvegicus GN=Smap2 PE=1 SV=1 - [A0A0G2K9N0_RAT]
Protein Itih2 OS=Rattus norvegicus GN=Itih2 PE=1 SV=3 - [D3ZFH5_RAT]
ADP-sugar pyrophosphatase OS=Rattus norvegicus GN=Nudt5 PE=1 SV=1 - [A0A140TAD1_RAT]
Hebp1 protein OS=Rattus norvegicus GN=Hebp1 PE=1 SV=1 - [B4F7C7_RAT]
Rho GTPase-activating protein 20 OS=Rattus norvegicus GN=Arhgap20 PE=1 SV=2 - [RHG20_RAT]
Tropomodulin-1 OS=Rattus norvegicus GN=Tmod1 PE=2 SV=1 - [TMOD1_RAT]
Emerin OS=Rattus norvegicus GN=Emd PE=1 SV=1 - [EMD_RAT]
Protein Uhrf2 OS=Rattus norvegicus GN=Uhrf2 PE=1 SV=1 - [D3ZK36_RAT]
Annexin A2 OS=Rattus norvegicus GN=Anxa2 PE=1 SV=2 - [ANXA2_RAT]
V-type proton ATPase subunit C 1 OS=Rattus norvegicus GN=Atp6v1c1 PE=2 SV=1 - [VATC1_RAT]
Microtubule-associated protein RP/EB family member 1 OS=Rattus norvegicus GN=Mapre1 PE=1 SV=3 - [MARE1_
NADH-cytochrome b5 reductase 3 OS=Rattus norvegicus GN=Cyb5r3 PE=1 SV=2 - [NB5R3_RAT]
Protein Tmem266 OS=Rattus norvegicus GN=Tmem266 PE=1 SV=1 - [D3ZYW8_RAT]
Glucocorticoid receptor OS=Rattus norvegicus GN=Nr3c1 PE=1 SV=3 - [G3V7U9_RAT]
Protein Las1l (Fragment) OS=Rattus norvegicus GN=Las1l PE=1 SV=1 - [A0A096MJM8_RAT]
Myotubularin-related protein 3 OS=Rattus norvegicus GN=Mtmr3 PE=1 SV=1 - [MTMR3_RAT]
Keratin, type II cytoskeletal 5 (Fragment) OS=Rattus norvegicus GN=Krt5 PE=1 SV=2 - [F7FFV2_RAT]
Protein Timm8a1 OS=Rattus norvegicus GN=Timm8a1 PE=1 SV=3 - [F1LP21_RAT]
Protein lin-7 homolog C OS=Rattus norvegicus GN=Lin7c PE=1 SV=1 - [LIN7C_RAT]
Protein Tmem87a OS=Rattus norvegicus GN=Tmem87a PE=1 SV=3 - [D4A017_RAT]
Calreticulin OS=Rattus norvegicus GN=Calr PE=1 SV=1 - [CALR_RAT]
Histone H1.1 OS=Rattus norvegicus GN=Hist1h1a PE=1 SV=1 - [H11_RAT]
Microtubule associated serine/threonine kinase 2 (Predicted), isoform CRA_a OS=Rattus norvegicus GN=Mast2 PE
Solute carrier family 12 member 5 OS=Rattus norvegicus GN=Slc12a5 PE=1 SV=3 - [D3ZGI9_RAT]
Eefsec protein OS=Rattus norvegicus GN=Eefsec PE=1 SV=1 - [B5DEJ5_RAT]
Protein Steap2 OS=Rattus norvegicus GN=Steap2 PE=1 SV=1 - [D4A6H3_RAT]
Protein Spata13 OS=Rattus norvegicus GN=Spata13 PE=1 SV=3 - [D3ZWB4_RAT]
Protein Ephb2 OS=Rattus norvegicus GN=Ephb2 PE=1 SV=3 - [F1MAJ0_RAT]
Proteasome p45/SUG (Fragment) OS=Rattus norvegicus GN=Psmc5 PE=4 SV=1 - [O35050_RAT]
Metastasis-associated protein MTA1 OS=Rattus norvegicus GN=Mta1 PE=1 SV=2 - [F1LQS1_RAT]
Sphingosine 1-phosphate receptor 1 OS=Rattus norvegicus GN=S1pr1 PE=1 SV=1 - [S1PR1_RAT]
Integrin beta OS=Rattus norvegicus GN=Itgb4 PE=1 SV=1 - [F1LSD3_RAT]
Cationic amino acid transporter 2 OS=Rattus norvegicus GN=Slc7a2 PE=1 SV=1 - [CTR2_RAT]
RNA-binding protein 8A OS=Rattus norvegicus GN=Rbm8a PE=1 SV=1 - [RBM8A_RAT]
Uncharacterized protein OS=Rattus norvegicus PE=3 SV=1 - [D3ZQT0_RAT]
Protein RGD1308428 OS=Rattus norvegicus GN=RGD1308428 PE=1 SV=3 - [F1M037_RAT]
Protein Plekhh1 OS=Rattus norvegicus GN=Plekhh1 PE=4 SV=3 - [D4AA54_RAT]
Casein kinase II subunit beta OS=Rattus norvegicus GN=Csnk2b PE=1 SV=1 - [CSK2B_RAT]
Bicaudal D protein (Fragment) OS=Rattus norvegicus GN=Bicd2 PE=2 SV=1 - [Q712J2_RAT]
Voltage-dependent calcium channel gamma-2 subunit OS=Rattus norvegicus GN=Cacng2 PE=1 SV=1 - [CCG2_RA
Protein Trappc6b OS=Rattus norvegicus GN=Trappc6b PE=1 SV=1 - [D3ZES2_RAT]
DIS3-like exonuclease 2 OS=Rattus norvegicus GN=Dis3l2 PE=1 SV=2 - [D3ZIL9_RAT]
Transitional endoplasmic reticulum ATPase OS=Rattus norvegicus GN=Vcp PE=1 SV=3 - [TERA_RAT]
Protein Uba2 OS=Rattus norvegicus GN=Uba2 PE=1 SV=3 - [F1LS72_RAT]
Protein Ccdc85a OS=Rattus norvegicus GN=Ccdc85a PE=1 SV=1 - [A0A0G2KAJ5_RAT]
Protein Zfr2 OS=Rattus norvegicus GN=Zfr2 PE=1 SV=3 - [D4A148_RAT]
Protein Kdm5a (Fragment) OS=Rattus norvegicus GN=Kdm5a PE=1 SV=1 - [A0A096MJL8_RAT]
Iron-sulfur cluster scaffold homolog (E. coli) OS=Rattus norvegicus GN=Iscu PE=1 SV=1 - [B2RZ79_RAT]
ATP-dependent DNA helicase Q1 OS=Rattus norvegicus GN=Recql PE=1 SV=1 - [A0A0G2K5S2_RAT]
Partitioning defective 3 homolog OS=Rattus norvegicus GN=Pard3 PE=1 SV=1 - [A0A0G2K9M8_RAT]
Protein Hecw2 OS=Rattus norvegicus GN=Hecw2 PE=1 SV=1 - [D4ADD3_RAT]

Protein Samd4b OS=Rattus norvegicus GN=Samd4b PE=1 SV=1 - [D4A769_RAT]
Phosphatase and actin regulator OS=Rattus norvegicus GN=Phactr1 PE=3 SV=3 - [F1LNB2_RAT]
Apbb1ip protein (Fragment) OS=Rattus norvegicus GN=Apbb1ip PE=2 SV=1 - [Q4G016_RAT]
Cytosolic phospholipase A2 OS=Rattus norvegicus GN=Pla2g4a PE=1 SV=1 - [PA24A_RAT]
ADP-ribosylation factor-like protein 3 OS=Rattus norvegicus GN=Arl3 PE=1 SV=2 - [ARL3_RAT]
Family with sequence similarity 126, member B OS=Rattus norvegicus GN=Fam126b PE=1 SV=1 - [Q4V7D4_RAT]
ATP-sensitive inward rectifier potassium channel 10 OS=Rattus norvegicus GN=Kcnj10 PE=1 SV=1 - [A0A0H2UHF
Palmitoyltransferase ZDHHC5 OS=Rattus norvegicus GN=Zdhhc5 PE=1 SV=1 - [ZDHC5_RAT]
Protein Mvb12b OS=Rattus norvegicus GN=Mvb12b PE=4 SV=2 - [D4A732_RAT]
Protein Ptprd OS=Rattus norvegicus GN=Ptprd PE=1 SV=2 - [M0RB22_RAT]
Leucine-rich repeat-containing protein 4B OS=Rattus norvegicus GN=Lrrc4b PE=1 SV=1 - [LRC4B_RAT]
Myocardial ischemic preconditioning associated protein 7 OS=Rattus norvegicus GN=Pxn PE=1 SV=1 - [Q1EG89_F
SH2-B PH domain containing signaling mediator 1, isoform CRA_a OS=Rattus norvegicus GN=Sh2b1 PE=1 SV=1 -
Receptor-type tyrosine-protein phosphatase-like N OS=Rattus norvegicus GN=Ptprn PE=1 SV=2 - [PTPRN_RAT]
Protein Ctage5 OS=Rattus norvegicus GN=Ctage5 PE=1 SV=1 - [A0A0G2JXM0_RAT]
Acyl-CoA-binding domain-containing protein 4 OS=Rattus norvegicus GN=Acbd4 PE=1 SV=1 - [ACBD4_RAT]
Biogenesis of lysosome-related organelles complex 1 subunit 3 OS=Rattus norvegicus GN=Bloc1s3 PE=1 SV=1 - [D
Hnrnpl protein (Fragment) OS=Rattus norvegicus GN=Hnrnpl PE=2 SV=1 - [B5DFG2_RAT]
FAS-associated death domain protein OS=Rattus norvegicus GN=Fadd PE=1 SV=1 - [Q8R2E7_RAT]
ERC protein 2 OS=Rattus norvegicus GN=Erc2 PE=1 SV=2 - [Z4YNN0_RAT]
Protein Jmy OS=Rattus norvegicus GN=Jmy PE=1 SV=2 - [M0R8J3_RAT]
Extended synaptotagmin-1 OS=Rattus norvegicus GN=Esyt1 PE=1 SV=1 - [ESYT1_RAT]
Proteasome (Prosome, macropain) 26S subunit, non-ATPase, 3 OS=Rattus norvegicus GN=Psmd3 PE=1 SV=1 - [C
Disabled homolog 2-interacting protein OS=Rattus norvegicus GN=Dab2ip PE=1 SV=1 - [DAB2P_RAT]
Armadillo repeat-containing X-linked protein 3 OS=Rattus norvegicus GN=Armcx3 PE=1 SV=1 - [ARMX3_RAT]
26S proteasome non-ATPase regulatory subunit 2 OS=Rattus norvegicus GN=Psmd2 PE=1 SV=1 - [PSMD2_RAT]
Tyrosine-protein kinase OS=Rattus norvegicus GN=Fyn PE=1 SV=1 - [A0A0G2K2E3_RAT]
Protein Rbm14 OS=Rattus norvegicus GN=Rbm14 PE=1 SV=2 - [M0R3R6_RAT]
DNA-(apurinic or apyrimidinic site) lyase (Fragment) OS=Rattus norvegicus GN=Apex1 PE=2 SV=1 - [Q99PF3_RAT
Enhancer of mRNA-decapping protein 4 OS=Rattus norvegicus GN=Edc4 PE=1 SV=1 - [EDC4_RAT]
Protein Tpd52l1 OS=Rattus norvegicus GN=Tpd52l1 PE=2 SV=1 - [Q499Q2_RAT]
Protein Ulk2 OS=Rattus norvegicus GN=Ulk2 PE=4 SV=1 - [D3Z9J7_RAT]
Protein RGD1559534 OS=Rattus norvegicus GN=RGD1559534 PE=4 SV=1 - [F1M9V3_RAT]
Neural proliferation, differentiation and control, 1 OS=Rattus norvegicus GN=Npdc1 PE=2 SV=1 - [Q6AY81_RAT]
Phospholemman OS=Rattus norvegicus GN=Fxyd1 PE=1 SV=2 - [PLM_RAT]
Protein Nyap2 OS=Rattus norvegicus GN=Nyap2 PE=1 SV=2 - [F1M3A4_RAT]
Kinesin-like protein OS=Rattus norvegicus GN=Kif5c PE=1 SV=1 - [A0A0G2K070_RAT]
Protein Fbxo10 OS=Rattus norvegicus GN=Fbxo10 PE=4 SV=1 - [A0A1B0GWY6_RAT]
Cyclin-dependent kinase 18 OS=Rattus norvegicus GN=Cdk18 PE=1 SV=1 - [CKD18_RAT]
Sphingomyelin phosphodiesterase 3 OS=Rattus norvegicus GN=Smpd3 PE=1 SV=2 - [D4A8L3_RAT]
Metabotropic glutamate receptor 2 OS=Rattus norvegicus GN=Grm2 PE=1 SV=1 - [GRM2_RAT]
N-acetylneuraminic acid synthase OS=Rattus norvegicus GN=Nans PE=1 SV=1 - [B1WC26_RAT]
Pre-mRNA processing factor 40 homolog A (Yeast) (Predicted) OS=Rattus norvegicus GN=Prpf40a PE=1 SV=1 - [D
Protein Pdcd11 OS=Rattus norvegicus GN=Pdcd11 PE=1 SV=1 - [D3ZNI3_RAT]
Phospholipid phosphatase 3 OS=Rattus norvegicus GN=Plpp3 PE=1 SV=1 - [PLPP3_RAT]
Protein Ppp1r13b OS=Rattus norvegicus GN=Ppp1r13b PE=1 SV=1 - [D4A6A8_RAT]
SWI/SNF-related matrix-associated actin-dependent regulator of chromatin subfamily A containing DEAD/H box 1 C
Protein Hnrnpll OS=Rattus norvegicus GN=Hnrnpll PE=1 SV=1 - [D4A3E1_RAT]
Complement component receptor 1-like protein OS=Rattus norvegicus GN=Cr1l PE=1 SV=1 - [CR1L_RAT]
Presenilin-1 OS=Rattus norvegicus GN=Psen1 PE=1 SV=1 - [PSN1_RAT]
Protein RGD1559921 OS=Rattus norvegicus GN=RGD1559921 PE=4 SV=1 - [F1LTZ6_RAT]
Protein Mia3 OS=Rattus norvegicus GN=Mia3 PE=1 SV=1 - [A0A0G2K0A8_RAT]
Cytoplasmic polyadenylation element binding protein 4 (Predicted) OS=Rattus norvegicus GN=Cpeb4 PE=1 SV=1 -
Synaptotagmin XI OS=Rattus norvegicus GN=Syt11 PE=2 SV=1 - [Q505J5_RAT]
Protein Rfc1 OS=Rattus norvegicus GN=Rfc1 PE=1 SV=1 - [A0A0G2JVK8_RAT]

Heat shock 27kDa protein 1 OS=Rattus norvegicus GN=Hspb1 PE=1 SV=1 - [G3V913_RAT]
Protein Arhgdib OS=Rattus norvegicus GN=Arhgdib PE=1 SV=1 - [Q5M860_RAT]
Mtx1 protein OS=Rattus norvegicus GN=Mtx1 PE=1 SV=1 - [B0BN02_RAT]
Trans-Golgi network integral membrane protein TGN38 OS=Rattus norvegicus GN=Ttgn1 PE=1 SV=1 - [TGON3_R
Protein Phf24 OS=Rattus norvegicus GN=Phf24 PE=1 SV=1 - [D3ZB78_RAT]
Numb-like OS=Rattus norvegicus GN=Numbl PE=2 SV=1 - [Q3MUI2_RAT]
Protein Kank4 OS=Rattus norvegicus GN=Kank4 PE=1 SV=1 - [D4A6X3_RAT]
RAC-beta serine/threonine-protein kinase OS=Rattus norvegicus GN=Akt2 PE=1 SV=1 - [AKT2_RAT]
Protein Sh3bp5l OS=Rattus norvegicus GN=Sh3bp5l PE=1 SV=1 - [B2GUX8_RAT]
C-jun N-terminal kinase-1 (Fragment) OS=Rattus norvegicus GN=Jnk1 PE=2 SV=1 - [A9LRT4_RAT]
Serine/threonine-protein kinase OS=Rattus norvegicus GN=Prkd1 PE=1 SV=1 - [A0A0G2K928_RAT]
Synaptotagmin-6 OS=Rattus norvegicus GN=Syt6 PE=1 SV=1 - [A0A0G2JW68_RAT]
PH domain leucine-rich repeat protein phosphatase 1 OS=Rattus norvegicus GN=Phlpp1 PE=1 SV=2 - [F1LNC3_R
GRAM domain-containing protein 1A OS=Rattus norvegicus GN=Gramd1a PE=2 SV=2 - [GRM1A_RAT]
E3 ubiquitin-protein ligase RNF146 OS=Rattus norvegicus GN=Rnf146 PE=1 SV=1 - [RN146_RAT]
Protein Tmem47 OS=Rattus norvegicus GN=Tmem47 PE=1 SV=1 - [D3ZBB3_RAT]
Cytochrome b-c1 complex subunit 7 OS=Rattus norvegicus GN=Uqcrb PE=1 SV=1 - [B2RYS2_RAT]
Endoribonuclease LACTB2 OS=Rattus norvegicus GN=Lactb2 PE=2 SV=1 - [LACB2_RAT]
Protein LOC100912427 OS=Rattus norvegicus GN=LOC100912427 PE=4 SV=1 - [D3ZEV0_RAT]
Protein Tbcd OS=Rattus norvegicus GN=Tbcd PE=1 SV=3 - [F1M1D5_RAT]
Mitochondrial Rho GTPase OS=Rattus norvegicus GN=Rhot1 PE=1 SV=1 - [A1L1L6_RAT]
Solute carrier family 35 member G2 OS=Rattus norvegicus GN=Slc35g2 PE=1 SV=1 - [S35G2_RAT]
Protein Arhgap25 OS=Rattus norvegicus GN=Arhgap25 PE=1 SV=1 - [D3ZGL1_RAT]
Protein Tmem51 OS=Rattus norvegicus GN=Tmem51 PE=1 SV=1 - [D4AE69_RAT]
Protein Ttc3 OS=Rattus norvegicus GN=Ttc3 PE=4 SV=3 - [D3ZSP7_RAT]
Enoyl-CoA hydratase, mitochondrial OS=Rattus norvegicus GN=Echs1 PE=1 SV=1 - [ECHM_RAT]
Ankyrin repeat domain 13 family, member D (Predicted) OS=Rattus norvegicus GN=Ankrd13d PE=1 SV=1 - [D3ZU
Copine-1 OS=Rattus norvegicus GN=Cpne1 PE=1 SV=1 - [CPNE1_RAT]
F-box only protein 31 OS=Rattus norvegicus GN=Fbxo31 PE=1 SV=1 - [FBX31_RAT]
Nucleoside diphosphate kinase B OS=Rattus norvegicus GN=Nme2 PE=1 SV=1 - [NDKB_RAT]
G protein-coupled receptor 155 (Predicted), isoform CRA_a OS=Rattus norvegicus GN=Gpr155 PE=1 SV=1 - [D3Z
Nuclear factor 1 OS=Rattus norvegicus GN=Nfic PE=1 SV=2 - [O70188_RAT]
Growth arrest-specific protein 7 OS=Rattus norvegicus GN=Gas7 PE=4 SV=2 - [M0R4R4_RAT]
RNA-binding motif protein, X-linked-like-1 OS=Rattus norvegicus GN=Rbmxl1 PE=1 SV=1 - [A0A0A0MXW9_RAT]
Protein Rbmxl1b OS=Rattus norvegicus GN=Rbmxl1b PE=4 SV=1 - [A0A0G2K8K9_RAT]
Protein prune homolog OS=Rattus norvegicus GN=Prune PE=1 SV=1 - [PRUNE_RAT]
Elongation factor 1-gamma OS=Rattus norvegicus GN=Eef1g PE=2 SV=3 - [EF1G_RAT]
Protein Hectd1 OS=Rattus norvegicus GN=Hectd1 PE=1 SV=3 - [D3ZLS5_RAT]
Protein Sec16a OS=Rattus norvegicus GN=Sec16a PE=1 SV=1 - [D3ZN76_RAT]
E3 ubiquitin-protein ligase OS=Rattus norvegicus GN=Wwp1 PE=1 SV=1 - [Q4V8H7_RAT]
High density lipoprotein binding protein (Vigilin) OS=Rattus norvegicus GN=Hdlbp PE=1 SV=1 - [Q3KRF2_RAT]
Mitogen-activated protein kinase 1 OS=Rattus norvegicus GN=Mapk1 PE=1 SV=3 - [MK01_RAT]
Protein Cdh11 OS=Rattus norvegicus GN=Cdh11 PE=1 SV=1 - [F1MAH6_RAT]
Phenylalanine--tRNA ligase alpha subunit OS=Rattus norvegicus GN=Farsa PE=1 SV=1 - [SYFA_RAT]
Protein Ccsap OS=Rattus norvegicus GN=Ccsap PE=1 SV=1 - [D3ZZM9_RAT]
Ras-related protein Ral-B OS=Rattus norvegicus GN=Ralb PE=1 SV=3 - [F1LQ62_RAT]
Nucleoprotein TPR OS=Rattus norvegicus GN=Tpr PE=1 SV=1 - [TPR_RAT]
Dual-specificity tyrosine-(Y)-phosphorylation regulated kinase 1b (Predicted), isoform CRA_a OS=Rattus norvegicus
Glycolipid transfer protein OS=Rattus norvegicus GN=GLTP PE=2 SV=1 - [GLTP_RAT]
Protein Spred3 (Fragment) OS=Rattus norvegicus GN=Spred3 PE=4 SV=1 - [A0A0G2K3F4_RAT]
Actin-like 6A OS=Rattus norvegicus GN=Actl6a PE=1 SV=1 - [Q4KM87_RAT]
Protein Arhgef40 OS=Rattus norvegicus GN=Arhgef40 PE=1 SV=1 - [A0A0G2JZE7_RAT]
Protein Ssfa2 OS=Rattus norvegicus GN=Ssfa2 PE=1 SV=1 - [D3ZLC3_RAT]
Guanine nucleotide-binding protein subunit gamma OS=Rattus norvegicus GN=Gng4 PE=3 SV=1 - [M0R809_RAT]
Protein Map3k10 OS=Rattus norvegicus GN=Map3k10 PE=1 SV=3 - [D3ZG83_RAT]

Information Classification: General

Protein Glcci1 OS=Rattus norvegicus GN=Glcci1 PE=1 SV=1 - [B2RYK0_RAT]
F-BAR domain only protein 2 OS=Rattus norvegicus GN=Fcho2 PE=1 SV=1 - [FCHO2_RAT]
Protein Ube2e3 OS=Rattus norvegicus GN=Ube2e3 PE=1 SV=1 - [A0A0G2K964_RAT]
Heterogeneous nuclear ribonucleoprotein A3 OS=Rattus norvegicus GN=Hnrnpa3 PE=1 SV=3 - [D4A6A2_RAT]
Junctional adhesion molecule C OS=Rattus norvegicus GN=Jam3 PE=1 SV=1 - [JAM3_RAT]
Protein Plekho2 OS=Rattus norvegicus GN=Plekho2 PE=1 SV=2 - [D3Z9K4_RAT]
Kv channel-interacting protein 2 OS=Rattus norvegicus GN=Kcnip2 PE=1 SV=2 - [KCIP2_RAT]
Protein Sept6 OS=Rattus norvegicus GN=Sept6 PE=1 SV=1 - [B5DFG5_RAT]
Protein Slc43a2 OS=Rattus norvegicus GN=Slc43a2 PE=1 SV=1 - [D3ZDC2_RAT]
Uncharacterized protein OS=Rattus norvegicus PE=4 SV=3 - [F1M269_RAT]
Protein Map3k5 OS=Rattus norvegicus GN=Map3k5 PE=1 SV=3 - [D3ZW27_RAT]
Protein Srsf3 OS=Rattus norvegicus GN=Srsf3 PE=1 SV=1 - [A0A0U1RRV7_RAT]
Protein Hspa12b OS=Rattus norvegicus GN=Hspa12b PE=1 SV=3 - [D3ZVM5_RAT]
Protein Ctdp1 OS=Rattus norvegicus GN=Ctdp1 PE=1 SV=1 - [A0A0G2K0J7_RAT]
Secretogranin III, isoform CRA_a OS=Rattus norvegicus GN=Scg3 PE=1 SV=2 - [F1M7L6_RAT]
Ras-related protein Rab-1A OS=Rattus norvegicus GN=Rab1A PE=1 SV=3 - [RAB1A_RAT]
Protein Fam196b OS=Rattus norvegicus GN=Fam196b PE=4 SV=2 - [D3ZZH5_RAT]
Protein Tmem132e OS=Rattus norvegicus GN=Tmem132e PE=4 SV=1 - [D4A2A3_RAT]
G-protein-signaling modulator 3 OS=Rattus norvegicus GN=Gpsm3 PE=1 SV=1 - [GPSM3_RAT]
Serine/threonine-protein kinase DCLK2 (Fragment) OS=Rattus norvegicus GN=Dclk2 PE=4 SV=1 - [A0A0G2JY17_
Beta-catenin-like protein 1 OS=Rattus norvegicus GN=Ctnnbl1 PE=1 SV=1 - [CTBL1_RAT]
Protein Mb21d2 OS=Rattus norvegicus GN=Mb21d2 PE=1 SV=1 - [D4ACS3_RAT]
Protein Ktn1 OS=Rattus norvegicus GN=Ktn1 PE=1 SV=2 - [D4A4Z9_RAT]
Protein Atp6v1h OS=Rattus norvegicus GN=Atp6v1h PE=1 SV=1 - [E9PTI1_RAT]
Cytospin-A OS=Rattus norvegicus GN=Specc1l PE=1 SV=1 - [CYTSA_RAT]
Protein LOC100909983 OS=Rattus norvegicus GN=LOC100909983 PE=4 SV=1 - [A0A0G2K7X0_RAT]
Type 2 phosphatidylinositol 4,5-bisphosphate 4-phosphatase OS=Rattus norvegicus GN=Tmem55a PE=1 SV=1 - [T
Ras-related protein Rab-35 OS=Rattus norvegicus GN=Rab35 PE=1 SV=1 - [RAB35_RAT]
LEM domain containing 2 OS=Rattus norvegicus GN=Lemd2 PE=1 SV=1 - [Q4KM57_RAT]
Ribosomal protein S6 kinase OS=Rattus norvegicus GN=Rps6ka5 PE=1 SV=1 - [A0A0G2K366_RAT]
SEC14-like protein 2 OS=Rattus norvegicus GN=Sec14l2 PE=1 SV=1 - [S14L2_RAT]
Tyrosine-protein kinase OS=Rattus norvegicus GN=LOC100909750 PE=1 SV=1 - [F1M0A6_RAT]
Protein Ppfia1 OS=Rattus norvegicus GN=Ppfia1 PE=1 SV=1 - [D3ZZ81_RAT]
Protein Samd12 OS=Rattus norvegicus GN=Samd12 PE=4 SV=2 - [M0R3J7_RAT]
Methionine adenosyltransferase 2 subunit beta OS=Rattus norvegicus GN=Mat2b PE=1 SV=1 - [A0A0G2JT30_RAT
Protein Mtmr4 OS=Rattus norvegicus GN=Mtmr4 PE=3 SV=2 - [D3ZW40_RAT]
Arfaptin-2 OS=Rattus norvegicus GN=Arfip2 PE=2 SV=1 - [ARFP2_RAT]
T-complex protein 1 subunit gamma OS=Rattus norvegicus GN=Cct3 PE=1 SV=1 - [TCPG_RAT]
Protein Nelfb OS=Rattus norvegicus GN=Nelfb PE=1 SV=1 - [D4ACV0_RAT]
Protein Hecw1 OS=Rattus norvegicus GN=Hecw1 PE=1 SV=1 - [A0A0G2K6U6_RAT]
Protein RGD1309104 OS=Rattus norvegicus GN=RGD1309104 PE=1 SV=1 - [D3ZU88_RAT]
Protein Cdc16 OS=Rattus norvegicus GN=Cdc16 PE=1 SV=1 - [A0A0G2JT88_RAT]
CTP synthase OS=Rattus norvegicus GN=Ctps1 PE=1 SV=1 - [B1WC02_RAT]
Leucine zipper putative tumor suppressor 2 OS=Rattus norvegicus GN=Lzts2 PE=1 SV=1 - [F7FLS6_RAT]
Protein Mtus2 OS=Rattus norvegicus GN=Mtus2 PE=1 SV=1 - [A0JPQ3_RAT]
Protein Patj OS=Rattus norvegicus GN=Patj PE=1 SV=2 - [F1MAD2_RAT]
Protein LOC100910882 OS=Rattus norvegicus GN=Rbm39 PE=1 SV=1 - [Q5BJP4_RAT]
Dishevelled associated activator of morphogenesis 1 (Predicted) OS=Rattus norvegicus GN=Daam1 PE=1 SV=1 - [
Potassium/sodium hyperpolarization-activated cyclic nucleotide-gated channel 4 OS=Rattus norvegicus GN=Hcn4 P
Opioid growth factor receptor-like protein 1 OS=Rattus norvegicus GN=Ogfrl1 PE=4 SV=1 - [F1M9G3_RAT]
Stress-70 protein, mitochondrial OS=Rattus norvegicus GN=Hspa9 PE=1 SV=1 - [F1M953_RAT]
Protein Golga7b OS=Rattus norvegicus GN=Golga7b PE=4 SV=3 - [F1M362_RAT]
Protein Nyap1 OS=Rattus norvegicus GN=Nyap1 PE=4 SV=2 - [D3ZDP7_RAT]
YTH domain-containing protein 1 OS=Rattus norvegicus GN=Ythdc1 PE=1 SV=2 - [YTDC1_RAT]
Regulator of G-protein-signaling 7 OS=Rattus norvegicus GN=Rgs7 PE=1 SV=2 - [D3ZWG2_RAT]

Information Classification: General

Protein Hcfc1 OS=Rattus norvegicus GN=Hcfc1 PE=1 SV=1 - [D3ZN95_RAT]
Protein Setd1a OS=Rattus norvegicus GN=Setd1a PE=1 SV=1 - [A0A0G2K6T6_RAT]
Guanine nucleotide exchange factor DBS OS=Rattus norvegicus GN=Mcf2l PE=1 SV=1 - [A0A0G2JU75_RAT]
Patatin-like phospholipase domain-containing protein 2 OS=Rattus norvegicus GN=Pnpla2 PE=1 SV=1 - [PLPL2_R
Protein Znf768 OS=Rattus norvegicus GN=Znf768 PE=1 SV=2 - [D3ZGK0_RAT]
Dennd5a protein (Fragment) OS=Rattus norvegicus GN=Dennd5a PE=2 SV=1 - [B5DF49_RAT]
Protein SDE2 homolog OS=Rattus norvegicus GN=Sde2 PE=1 SV=2 - [SDE2_RAT]
Protein Map6d1 OS=Rattus norvegicus GN=Map6d1 PE=1 SV=1 - [D3ZT16_RAT]
Eukaryotic elongation factor 2 kinase OS=Rattus norvegicus GN=Eef2k PE=1 SV=1 - [EF2K_RAT]
Protein Rnf219 OS=Rattus norvegicus GN=Rnf219 PE=4 SV=1 - [D3ZAB6_RAT]
5'-AMP-activated protein kinase catalytic subunit alpha-1 OS=Rattus norvegicus GN=Prkaa1 PE=1 SV=2 - [AAPK1_
Protein Soga1 OS=Rattus norvegicus GN=Soga1 PE=1 SV=1 - [A0A0G2KA88_RAT]
Cask-interacting protein 2 (Predicted) OS=Rattus norvegicus GN=Caskin2 PE=1 SV=1 - [D4A9T0_RAT]
Voltage-dependent calcium channel gamma-4 subunit OS=Rattus norvegicus GN=Cacng4 PE=1 SV=1 - [CCG4_R
Mediator of DNA damage checkpoint protein 1 OS=Rattus norvegicus GN=Mdc1 PE=1 SV=2 - [MDC1_RAT]
Cytosolic Fe-S cluster assembly factor NUBP1 OS=Rattus norvegicus GN=Nubp1 PE=1 SV=1 - [NUBP1_RAT]
Uncharacterized protein (Fragment) OS=Rattus norvegicus GN=Tom1l1 PE=2 SV=1 - [B5DFM1_RAT]
BCL2/adenovirus E1B interacting protein 3-like OS=Rattus norvegicus GN=Bnip3l PE=1 SV=1 - [Q4G086_RAT]
Protein Usp35 OS=Rattus norvegicus GN=Usp35 PE=1 SV=2 - [F1M8S4_RAT]
Spata6 protein (Fragment) OS=Rattus norvegicus GN=Spata6 PE=2 SV=1 - [B0BMV7_RAT]
N-myc downstream regulated gene 4, isoform CRA_c OS=Rattus norvegicus GN=Ndrg4 PE=1 SV=1 - [G3V7P8_R
Potassium voltage-gated channel subfamily H member 7 OS=Rattus norvegicus GN=Kcnh7 PE=1 SV=1 - [A0A0G2
Ubiquitin-conjugating enzyme E2 variant 2 OS=Rattus norvegicus GN=Ube2v2 PE=1 SV=1 - [A0A0G2JU07_RAT]
G protein-coupled receptor kinase 5 OS=Rattus norvegicus GN=Grk5 PE=2 SV=1 - [GRK5_RAT]
Protein Olr170 OS=Rattus norvegicus GN=Olr170 PE=3 SV=1 - [D3ZIT1_RAT]
Protein Tardbp OS=Rattus norvegicus GN=Tardbp PE=1 SV=1 - [I6L9G6_RAT]
Far upstream element-binding protein 1 OS=Rattus norvegicus GN=Fubp1 PE=1 SV=1 - [FUBP1_RAT]
H-caldesmon (Fragment) OS=Rattus norvegicus GN=Cald1 PE=2 SV=1 - [Q99N97_RAT]
Peripheral plasma membrane protein CASK OS=Rattus norvegicus GN=Cask PE=1 SV=2 - [D4A8M2_RAT]
Protein Prr36 OS=Rattus norvegicus GN=Prr36 PE=4 SV=2 - [D3ZPT0_RAT]
ATP synthase subunit delta, mitochondrial OS=Rattus norvegicus GN=Atp5d PE=1 SV=1 - [G3V7Y3_RAT]
Sorting nexin-15 OS=Rattus norvegicus GN=Snx15 PE=2 SV=1 - [SNX15_RAT]
Protein Zswim8 OS=Rattus norvegicus GN=Zswim8 PE=1 SV=1 - [A0A0G2K9R0_RAT]
Protein Camta2 OS=Rattus norvegicus GN=Camta2 PE=1 SV=2 - [D3ZLG1_RAT]
Arvcf protein OS=Rattus norvegicus GN=Arvcf PE=1 SV=1 - [B4F7F3_RAT]
Protein Tpd52 OS=Rattus norvegicus GN=Tpd52 PE=1 SV=1 - [A0A0G2K865_RAT]
Tumor protein p53-inducible protein 11 OS=Rattus norvegicus GN=Tp53i11 PE=1 SV=1 - [P5I11_RAT]
Uncharacterized protein OS=Rattus norvegicus PE=3 SV=1 - [A0A096MIY6_RAT]
Protein Zfp687 OS=Rattus norvegicus GN=Zfp687 PE=1 SV=1 - [D3ZT56_RAT]
Cyclin-L1 OS=Rattus norvegicus GN=Ccnl1 PE=1 SV=1 - [CCNL1_RAT]
Synaptotagmin-5 OS=Rattus norvegicus GN=Syt5 PE=1 SV=1 - [SYT5_RAT]
Filamin-C OS=Rattus norvegicus GN=Flnc PE=1 SV=1 - [A0A0H2UHR7_RAT]
Inositol hexakisphosphate and diphosphoinositol-pentakisphosphate kinase 1 OS=Rattus norvegicus GN=Ppip5k1 P
Protein Ice1 OS=Rattus norvegicus GN=Ice1 PE=1 SV=3 - [D3ZK16_RAT]
Protein Pcdh9 OS=Rattus norvegicus GN=Pcdh9 PE=4 SV=3 - [F1LS01_RAT]
Protein R3hdm1 OS=Rattus norvegicus GN=R3hdm1 PE=1 SV=1 - [F1LNT3_RAT]
TCF3 (E2A) fusion partner, isoform CRA_b OS=Rattus norvegicus GN=Tfpt PE=1 SV=1 - [A0A0G2K9T8_RAT]
Myotrophin OS=Rattus norvegicus GN=Mtpn PE=1 SV=2 - [MTPN_RAT]
ATP-sensitive inward rectifier potassium channel 11 OS=Rattus norvegicus GN=Kcnj11 PE=1 SV=1 - [KCJ11_RAT]
Reticulophagy receptor FAM134B OS=Rattus norvegicus GN=Fam134b PE=1 SV=1 - [A0A0G2JUQ3_RAT]
Protein Prr15 OS=Rattus norvegicus GN=Prr15 PE=1 SV=1 - [A0JPP6_RAT]
Uridine kinase OS=Rattus norvegicus GN=Uckl1 PE=3 SV=1 - [D3ZYQ8_RAT]
AF4/FMR2 family, member 4 (Predicted) OS=Rattus norvegicus GN=Aff4 PE=1 SV=2 - [D3ZSX2_RAT]
Eukaryotic translation initiation factor 2-alpha kinase 3 OS=Rattus norvegicus GN=Eif2ak3 PE=1 SV=1 - [E2AK3_R
Protein Fhod1 OS=Rattus norvegicus GN=Fhod1 PE=1 SV=1 - [A0A0G2JZ38_RAT]

Information Classification: General

Protein ZNF365 OS=Rattus norvegicus GN=Znf365 PE=1 SV=1 - [ZN365_RAT]
P2Y purinoceptor 2 OS=Rattus norvegicus GN=P2ry2 PE=3 SV=1 - [G3V8H8_RAT]
Protein RGD1308106 OS=Rattus norvegicus GN=RGD1308106 PE=1 SV=1 - [A0A0G2KB48_RAT]
Elongator complex protein 1 OS=Rattus norvegicus GN=Ikbkap PE=1 SV=2 - [F1LP76_RAT]
Protein Prr18 OS=Rattus norvegicus GN=Prr18 PE=4 SV=1 - [D3ZDS0_RAT]
RNA-binding protein 34 OS=Rattus norvegicus GN=Rbm34 PE=1 SV=1 - [RBM34_RAT]
Glutamate decarboxylase OS=Rattus norvegicus GN=Gad1 PE=2 SV=1 - [Q63211_RAT]
FAS-associated factor 1 OS=Rattus norvegicus GN=Faf1 PE=1 SV=3 - [F1LSQ0_RAT]
Serine racemase OS=Rattus norvegicus GN=Srr PE=1 SV=1 - [SRR_RAT]
Protein RGD1566007 OS=Rattus norvegicus GN=RGD1566007 PE=4 SV=1 - [A0A0G2K2U3_RAT]
Ubiquitin carboxyl-terminal hydrolase MINDY-1 OS=Rattus norvegicus GN=Fam63a PE=2 SV=1 - [FA63A_RAT]
Protein Lsm14b OS=Rattus norvegicus GN=Lsm14b PE=1 SV=1 - [A0A0G2JZY0_RAT]
Protein Tmod3 OS=Rattus norvegicus GN=Tmod3 PE=1 SV=1 - [Q6AXW2_RAT]
Protein Zfpm1 OS=Rattus norvegicus GN=Zfpm1 PE=1 SV=3 - [D4A168_RAT]
Protein Wdr59 OS=Rattus norvegicus GN=Wdr59 PE=1 SV=3 - [D3ZGZ9_RAT]
Protein Ints3 OS=Rattus norvegicus GN=Ints3 PE=1 SV=1 - [D3ZUT9_RAT]
Polymerase (RNA) II (DNA directed) polypeptide C, 33kDa OS=Rattus norvegicus GN=Polr2c PE=1 SV=1 - [Q5EB9
Protein N4bp1 OS=Rattus norvegicus GN=N4bp1 PE=1 SV=2 - [D3ZT79_RAT]
Glutamate receptor-interacting protein 2 OS=Rattus norvegicus GN=Grip2 PE=4 SV=1 - [A0A0H2UHH8_RAT]
Oxygen-dependent coproporphyrinogen-III oxidase, mitochondrial OS=Rattus norvegicus GN=Cpox PE=1 SV=1 - [
Rho GTPase-activating protein 44 OS=Rattus norvegicus GN=Arhgap44 PE=1 SV=1 - [A0A0H2UHC0_RAT]
Protein Sap30l OS=Rattus norvegicus GN=Sap30l PE=1 SV=1 - [D3ZJK3_RAT]
Phosphoinositide phospholipase C OS=Rattus norvegicus GN=Plcd1 PE=1 SV=1 - [G3V9D1_RAT]
Protein Wwc3 OS=Rattus norvegicus GN=Wwc3 PE=1 SV=2 - [M0R8P6_RAT]
Protein Zfp709 OS=Rattus norvegicus GN=Zfp709 PE=2 SV=1 - [Q99PJ6_RAT]
Heat shock protein 105 kDa OS=Rattus norvegicus GN=Hsph1 PE=1 SV=1 - [HS105_RAT]
Gap junction protein OS=Rattus norvegicus GN=Gjc3 PE=1 SV=1 - [F1LPT0_RAT]
Protein Akap8l OS=Rattus norvegicus GN=Akap8l PE=1 SV=1 - [A0A0G2K0P0_RAT]
Protein Gas2 OS=Rattus norvegicus GN=Gas2 PE=1 SV=1 - [G3V857_RAT]
RGD1310269 protein OS=Rattus norvegicus GN=Tmem179 PE=2 SV=1 - [B1H238_RAT]
Protein Epb41l4a OS=Rattus norvegicus GN=Epb41l4a PE=4 SV=1 - [A0A0G2JW20_RAT]
Protein Camsap3 OS=Rattus norvegicus GN=Camsap3 PE=1 SV=1 - [A0A0G2K5C0_RAT]
Protein Nt5dc3 OS=Rattus norvegicus GN=Nt5dc3 PE=1 SV=2 - [D3ZAI6_RAT]
GPALPP motifs-containing protein 1 OS=Rattus norvegicus GN=Gpalpp1 PE=1 SV=1 - [GPAM1_RAT]
Protein Srgap1 OS=Rattus norvegicus GN=Srgap1 PE=1 SV=2 - [F1M287_RAT]
Protein Stil OS=Rattus norvegicus GN=Stil PE=4 SV=1 - [A0A0G2KB96_RAT]
Citrate synthase OS=Rattus norvegicus GN=Cs PE=1 SV=1 - [G3V936_RAT]
Tetraspanin-2 OS=Rattus norvegicus GN=Tspan2 PE=1 SV=1 - [TSN2_RAT]
Fam91a1 protein OS=Rattus norvegicus GN=Fam91a1 PE=1 SV=1 - [B2RYP3_RAT]
Transcription factor FKHR (Fragment) OS=Rattus norvegicus GN=Foxo1 PE=2 SV=1 - [Q9ES26_RAT]
Protein Spen OS=Rattus norvegicus GN=Spen PE=1 SV=3 - [F1M816_RAT]
Staphylococcal nuclease domain-containing protein 1 OS=Rattus norvegicus GN=Snd1 PE=1 SV=2 - [D4A8Y5_RA
Protein RGD1311745 OS=Rattus norvegicus GN=RGD1311745 PE=1 SV=1 - [A0A0G2K0E2_RAT]
Thyroid hormone receptor alpha OS=Rattus norvegicus GN=Thra PE=3 SV=1 - [G3V764_RAT]
G protein pathway suppressor 2 (Predicted) OS=Rattus norvegicus GN=Neurl4 PE=1 SV=2 - [D4A198_RAT]
Osteoglycin (Predicted) OS=Rattus norvegicus GN=Ogn PE=1 SV=1 - [D3ZVB7_RAT]
Synaptosomal-associated protein 47 OS=Rattus norvegicus GN=Snap47 PE=2 SV=1 - [SNP47_RAT]
Protein Fam196a OS=Rattus norvegicus GN=Fam196a PE=4 SV=1 - [D4A4J1_RAT]
Arrestin domain-containing protein 3 OS=Rattus norvegicus GN=Arrdc3 PE=2 SV=1 - [ARRD3_RAT]
Protein Rnf20 OS=Rattus norvegicus GN=Rnf20 PE=1 SV=1 - [D3ZYQ9_RAT]
Lamin-B receptor OS=Rattus norvegicus GN=Lbr PE=1 SV=1 - [A0A0G2JU83_RAT]
Protein Fnbp4 OS=Rattus norvegicus GN=Fnbp4 PE=1 SV=3 - [F1MAK1_RAT]
Protein LOC688526 OS=Rattus norvegicus GN=LOC688526 PE=4 SV=1 - [F1M6U2_RAT]
Protein Srsf1 OS=Rattus norvegicus GN=Srsf1 PE=1 SV=1 - [D4A9L2_RAT]
6.8 kDa mitochondrial proteolipid OS=Rattus norvegicus GN=Mp68 PE=1 SV=1 - [68MP_RAT]

Eukaryotic initiation factor 4A-II OS=Rattus norvegicus GN=Eif4a2 PE=1 SV=1 - [A0A0G2K8B7_RAT]
Protein FAM195B OS=Rattus norvegicus GN=Fam195b PE=1 SV=1 - [F195B_RAT]
Golgi phosphoprotein 3 OS=Rattus norvegicus GN=Golph3 PE=1 SV=1 - [Q569C9_RAT]
Eukaryotic translation initiation factor 2 subunit 1 OS=Rattus norvegicus GN=Eif2s1 PE=1 SV=2 - [IF2A_RAT]
Casein kinase I isoform delta OS=Rattus norvegicus GN=Csnk1d PE=1 SV=2 - [KC1D_RAT]
Ndufa4 protein OS=Rattus norvegicus GN=Ndufa4 PE=1 SV=1 - [B2RZD6_RAT]
Protein Srp14 OS=Rattus norvegicus GN=Srp14 PE=1 SV=1 - [B2RYW7_RAT]
Translocon-associated protein subunit gamma OS=Rattus norvegicus GN=Ssr3 PE=1 SV=2 - [SSRG_RAT]
Surfeit 4, isoform CRA_a OS=Rattus norvegicus GN=Surf4 PE=1 SV=1 - [D4A1D8_RAT]
SUZ domain-containing protein 1 OS=Rattus norvegicus GN=Szrd1 PE=1 SV=2 - [SZRD1_RAT]
RCG39700, isoform CRA_d OS=Rattus norvegicus GN=Rab6a PE=1 SV=1 - [A0A0H2UHP9_RAT]
Protein Fam76b OS=Rattus norvegicus GN=Fam76b PE=1 SV=1 - [D3Z8B9_RAT]
Cdc42-interacting protein 4 OS=Rattus norvegicus GN=Trip10 PE=1 SV=3 - [CIP4_RAT]
Hamartin OS=Rattus norvegicus GN=Tsc1 PE=1 SV=1 - [TSC1_RAT]
1-phosphatidylinositol 4,5-bisphosphate phosphodiesterase beta-3 OS=Rattus norvegicus GN=Plcb3 PE=1 SV=2 - [
TSC22 domain family protein 4 OS=Rattus norvegicus GN=Tsc22d4 PE=1 SV=1 - [T22D4_RAT]
Nucleoporin NUP53 OS=Rattus norvegicus GN=Nup35 PE=1 SV=1 - [NUP53_RAT]
Stress-induced-phosphoprotein 1 OS=Rattus norvegicus GN=Stip1 PE=1 SV=1 - [STIP1_RAT]
Caspase recruitment domain-containing protein 9 OS=Rattus norvegicus GN=Card9 PE=1 SV=1 - [CARD9_RAT]
N(G),N(G)-dimethylarginine dimethylaminohydrolase 1 OS=Rattus norvegicus GN=Ddah1 PE=1 SV=3 - [DDAH1_R
Actin-related protein 2/3 complex subunit 5-like protein OS=Rattus norvegicus GN=Arpc5l PE=1 SV=2 - [ARP5L_R
Metalloreductase STEAP3 OS=Rattus norvegicus GN=Steap3 PE=1 SV=1 - [STEA3_RAT]
Proto-oncogene tyrosine-protein kinase Src OS=Rattus norvegicus GN=Src PE=1 SV=4 - [SRC_RAT]
Transcription factor jun-D OS=Rattus norvegicus GN=Jund PE=1 SV=1 - [JUND_RAT]
Golgi integral membrane protein 4 OS=Rattus norvegicus GN=Golim4 PE=1 SV=2 - [GOLI4_RAT]
Cyclin-dependent kinase-like 3 OS=Rattus norvegicus GN=Cdkl3 PE=1 SV=2 - [CDKL3_RAT]
Superoxide dismutase [Mn], mitochondrial OS=Rattus norvegicus GN=Sod2 PE=1 SV=2 - [SODM_RAT]
Histone acetyltransferase KAT8 OS=Rattus norvegicus GN=Kat8 PE=1 SV=1 - [KAT8_RAT]
GTP cyclohydrolase 1 OS=Rattus norvegicus GN=Gch1 PE=1 SV=1 - [GCH1_RAT]
Macoilin OS=Rattus norvegicus GN=Tmem57 PE=1 SV=1 - [MACOI_RAT]
Protein pelota homolog OS=Rattus norvegicus GN=Pelo PE=1 SV=1 - [PELO_RAT]
General transcription factor IIH subunit 3 OS=Rattus norvegicus GN=Gtf2h3 PE=2 SV=1 - [TF2H3_RAT]
DnaJ homolog subfamily A member 2 OS=Rattus norvegicus GN=Dnaja2 PE=1 SV=1 - [DNJA2_RAT]
E3 ubiquitin-protein ligase Midline-1 OS=Rattus norvegicus GN=Mid1 PE=2 SV=1 - [TRI18_RAT]
Cannabinoid receptor 1 OS=Rattus norvegicus GN=Cnr1 PE=1 SV=1 - [CNR1_RAT]
Protein unc-13 homolog A OS=Rattus norvegicus GN=Unc13a PE=1 SV=1 - [UN13A_RAT]
Copper-transporting ATPase 2 OS=Rattus norvegicus GN=Atp7b PE=1 SV=1 - [ATP7B_RAT]
Centromere/kinetochore protein zw10 homolog OS=Rattus norvegicus GN=Zw10 PE=2 SV=3 - [ZW10_RAT]
Microfibrillar-associated protein 3-like OS=Rattus norvegicus GN=Mfap3l PE=1 SV=1 - [MFA3L_RAT]
Tyrosine-protein phosphatase non-receptor type substrate 1 OS=Rattus norvegicus GN=Sirpa PE=1 SV=1 - [SHPS
Sodium/potassium/calcium exchanger 2 OS=Rattus norvegicus GN=Slc24a2 PE=1 SV=1 - [NCKX2_RAT]
V-type proton ATPase subunit S1 OS=Rattus norvegicus GN=Atp6ap1 PE=2 SV=1 - [VAS1_RAT]
Brefeldin A-inhibited guanine nucleotide-exchange protein 1 OS=Rattus norvegicus GN=Arfgef1 PE=1 SV=1 - [BIG1
UDP-glucuronosyltransferase 1-2 OS=Rattus norvegicus GN=Ugt1a2 PE=2 SV=2 - [UD12_RAT]
MICAL-like protein 1 OS=Rattus norvegicus GN=Micall1 PE=1 SV=1 - [MILK1_RAT]
Thymidine phosphorylase OS=Rattus norvegicus GN=Tymp PE=1 SV=1 - [TYPH_RAT]
Protein rogdi homolog OS=Rattus norvegicus GN=Rogdi PE=2 SV=1 - [ROGDI_RAT]
Regulator of microtubule dynamics protein 2 OS=Rattus norvegicus GN=Rmdn2 PE=1 SV=1 - [RMD2_RAT]
Voltage-dependent L-type calcium channel subunit beta-3 OS=Rattus norvegicus GN=Cacnb3 PE=1 SV=1 - [CACB
Cytochrome c oxidase subunit 4 isoform 1, mitochondrial OS=Rattus norvegicus GN=Cox4i1 PE=1 SV=1 - [COX41_
Sorting nexin-5 OS=Rattus norvegicus GN=Snx5 PE=1 SV=1 - [SNX5_RAT]
Death-associated protein kinase 3 OS=Rattus norvegicus GN=Dapk3 PE=1 SV=1 - [DAPK3_RAT]
Non-POU domain-containing octamer-binding protein OS=Rattus norvegicus GN=Nono PE=1 SV=3 - [NONO_RAT]
NT-3 growth factor receptor OS=Rattus norvegicus GN=Ntrk3 PE=1 SV=2 - [NTRK3_RAT]
Amine oxidase [flavin-containing] B OS=Rattus norvegicus GN=Maob PE=1 SV=3 - [AOFB_RAT]

Information Classification: General

Glyoxalase domain-containing protein 4 OS=Rattus norvegicus GN=Glod4 PE=1 SV=1 - [GLOD4_RAT]
Exocyst complex component 7 OS=Rattus norvegicus GN=Exoc7 PE=1 SV=1 - [EXOC7_RAT]
Alpha-crystallin B chain OS=Rattus norvegicus GN=Cryab PE=1 SV=1 - [CRYAB_RAT]
Carbohydrate-responsive element-binding protein OS=Rattus norvegicus GN=Mlxipl PE=1 SV=1 - [MLXPL_RAT]
Atrophin-1 OS=Rattus norvegicus GN=Atn1 PE=1 SV=1 - [ATN1_RAT]
Homeobox protein OTX1 OS=Rattus norvegicus GN=Otx1 PE=2 SV=1 - [OTX1_RAT]
Retinoblastoma-associated protein OS=Rattus norvegicus GN=Rb1 PE=1 SV=3 - [RB_RAT]
Olfactomedin-like protein 1 OS=Rattus norvegicus GN=Olfml1 PE=2 SV=1 - [OLFL1_RAT]
Protein kinase C delta-binding protein OS=Rattus norvegicus GN=Prkcdbp PE=1 SV=1 - [PRDBP_RAT]
Macrophage-capping protein OS=Rattus norvegicus GN=Capg PE=1 SV=1 - [CAPG_RAT]
Solute carrier family 22 member 1 OS=Rattus norvegicus GN=Slc22a1 PE=1 SV=1 - [S22A1_RAT]
SNF-related serine/threonine-protein kinase OS=Rattus norvegicus GN=Snrk PE=1 SV=1 - [SNRK_RAT]
Tubulin-specific chaperone cofactor E-like protein OS=Rattus norvegicus GN=Tbcel PE=1 SV=1 - [TBCEL_RAT]
General transcription factor II-I OS=Rattus norvegicus GN=Gtf2i PE=1 SV=2 - [GTF2I_RAT]
A-kinase anchor protein SPHKAP OS=Rattus norvegicus GN=Sphkap PE=1 SV=1 - [SPKAP_RAT]
Phosphorylated adapter RNA export protein OS=Rattus norvegicus GN=Phax PE=1 SV=2 - [PHAX_RAT]
Inosine triphosphate pyrophosphatase OS=Rattus norvegicus GN=Itpa PE=3 SV=1 - [ITPA_RAT]
Phosphoinositide 3-kinase regulatory subunit 4 OS=Rattus norvegicus GN=Pik3r4 PE=3 SV=2 - [PI3R4_RAT]
Peroxisomal membrane protein PEX14 OS=Rattus norvegicus GN=Pex14 PE=1 SV=1 - [PEX14_RAT]
Hematological and neurological expressed 1-like protein OS=Rattus norvegicus GN=Hn1l PE=2 SV=1 - [HN1L_RAT
Endonuclease/exonuclease/phosphatase family domain-containing protein 1 OS=Rattus norvegicus GN=Eepd1 PE=
OCIA domain-containing protein 1 OS=Rattus norvegicus GN=Ociad1 PE=1 SV=1 - [OCAD1_RAT]
FAS-associated factor 2 OS=Rattus norvegicus GN=Faf2 PE=2 SV=1 - [FAF2_RAT]
Importin subunit beta-1 OS=Rattus norvegicus GN=Kpnb1 PE=1 SV=1 - [IMB1_RAT]
Mitochondrial fission 1 protein OS=Rattus norvegicus GN=Fis1 PE=1 SV=1 - [FIS1_RAT]
Folate transporter 1 OS=Rattus norvegicus GN=Slc19a1 PE=2 SV=1 - [S19A1_RAT]
Fatty acid-binding protein, epidermal OS=Rattus norvegicus GN=Fabp5 PE=1 SV=3 - [FABP5_RAT]
Protocadherin-8 OS=Rattus norvegicus GN=Pcdh8 PE=1 SV=2 - [PCDH8_RAT]
G patch domain-containing protein 4 OS=Rattus norvegicus GN=Gpatch4 PE=2 SV=1 - [GPTC4_RAT]
Sorting nexin-16 OS=Rattus norvegicus GN=Snx16 PE=1 SV=2 - [SNX16_RAT]
Leucine rich adaptor protein 1 OS=Rattus norvegicus GN=Lurap1 PE=1 SV=1 - [LURA1_RAT]
Fuctinin-3 (Fragment) OS=Rattus norvegicus PE=1 SV=1 - [FUC3_RAT]
Dixin OS=Rattus norvegicus GN=Dixdc1 PE=2 SV=1 - [DIXC1_RAT]
Protein phosphatase 1H OS=Rattus norvegicus GN=Ppm1h PE=1 SV=2 - [PPM1H_RAT]
Adhesion G protein-coupled receptor L1 OS=Rattus norvegicus GN=Adgrl1 PE=1 SV=1 - [AGRL1_RAT]
Gamma-aminobutyric acid receptor subunit rho-3 OS=Rattus norvegicus GN=Gabrr3 PE=1 SV=1 - [GBRR3_RAT]
Uncharacterized protein C19orf44 homolog OS=Rattus norvegicus PE=1 SV=1 - [CS044_RAT]
Guanylate cyclase soluble subunit alpha-2 OS=Rattus norvegicus GN=Gucy1a2 PE=1 SV=2 - [GCYA2_RAT]
Serine/threonine-protein kinase WNK1 OS=Rattus norvegicus GN=Wnk1 PE=1 SV=2 - [WNK1_RAT]
Amyloid beta A4 precursor protein-binding family A member 2 OS=Rattus norvegicus GN=Apba2 PE=1 SV=1 - [APE
Somatostatin receptor type 2 OS=Rattus norvegicus GN=Sstr2 PE=1 SV=1 - [SSR2_RAT]
Solute carrier family 22 member 17 OS=Rattus norvegicus GN=Slc22a17 PE=2 SV=2 - [S22AH_RAT]
cGMP-dependent protein kinase 2 OS=Rattus norvegicus GN=Prkg2 PE=1 SV=1 - [KGP2_RAT]
Cold shock domain-containing protein C2 OS=Rattus norvegicus GN=Csdc2 PE=1 SV=3 - [CSDC2_RAT]
H2-K region expressed gene 4, rat orthologue OS=Rattus norvegicus GN=Slc39a7 PE=1 SV=1 - [Q6MGB4_RAT]
Protein phosphatase 1 regulatory subunit 1A OS=Rattus norvegicus GN=Ppp1r1a PE=1 SV=1 - [PPR1A_RAT]
Beta-crystallin S OS=Rattus norvegicus GN=Crygs PE=3 SV=1 - [CRBS_RAT]
Transmembrane protein 11, mitochondrial OS=Rattus norvegicus GN=Tmem11 PE=2 SV=1 - [TMM11_RAT]
Acid-sensing ion channel 2 OS=Rattus norvegicus GN=Asic2 PE=1 SV=1 - [ASIC2_RAT]
Cerebellin-1 OS=Rattus norvegicus GN=Cbln1 PE=1 SV=2 - [CBLN1_RAT]
Intermediate filament family orphan 2 OS=Rattus norvegicus GN=Iffo2 PE=4 SV=1 - [IFFO2_RAT]
Uncharacterized protein C7orf50 homolog OS=Rattus norvegicus PE=1 SV=1 - [CG050_RAT]
Inactive phospholipid phosphatase 7 OS=Rattus norvegicus GN=Plpp7 PE=2 SV=1 - [PLPP7_RAT]
CCR4-NOT transcription complex subunit 11 OS=Rattus norvegicus GN=Cnot11 PE=2 SV=1 - [CNO11_RAT]
TRAF-interacting protein with FHA domain-containing protein A OS=Rattus norvegicus GN=Tifa PE=2 SV=1 - [TIFA

Sharpin OS=Rattus norvegicus GN=Sharpin PE=1 SV=1 - [SHRPN_RAT]
Alpha- and gamma-adaptin-binding protein p34 OS=Rattus norvegicus GN=Aagab PE=1 SV=1 - [AAGAB_RAT]
Plasmolipin OS=Rattus norvegicus GN=Pllp PE=1 SV=2 - [PLLP_RAT]
F-box and leucine-rich repeat protein 4 (Predicted), isoform CRA_c OS=Rattus norvegicus GN=Fbxl4 PE=2 SV=1 -
Protein Slc24a4 OS=Rattus norvegicus GN=Slc24a4 PE=2 SV=2 - [D3ZCC3_RAT]
Bri3 binding protein OS=Rattus norvegicus GN=Bri3bp PE=1 SV=1 - [Q5U3Z5_RAT]
Protein Usp29 OS=Rattus norvegicus GN=Usp29 PE=3 SV=1 - [D4AEI6_RAT]
SLIT-ROBO Rho GTPase-activating protein 2 OS=Rattus norvegicus GN=Srgap2 PE=1 SV=1 - [B5DEJ1_RAT]
Protein LOC103689930 OS=Rattus norvegicus GN=Trappc8 PE=1 SV=2 - [F1M9W9_RAT]
Leucine rich repeat and fibronectin type III domain containing 2 OS=Rattus norvegicus GN=Lrfn2 PE=4 SV=1 - [G3V
Protein Xrcc6 OS=Rattus norvegicus GN=Xrcc6 PE=1 SV=1 - [Q6AZ64_RAT]
Nicotinamide-nucleotide adenylyltransferase OS=Rattus norvegicus GN=Nmnat1 PE=1 SV=1 - [A0JPJ0_RAT]
Ankyrin 1, erythroid OS=Rattus norvegicus GN=Ank1 PE=1 SV=1 - [D3Z9Z0_RAT]
V-type proton ATPase subunit a OS=Rattus norvegicus GN=Tcirg1 PE=1 SV=1 - [G3V887_RAT]
FCH domain only 1 (Predicted) OS=Rattus norvegicus GN=Fcho1 PE=1 SV=2 - [D3ZIM5_RAT]
Fsd1 protein OS=Rattus norvegicus GN=Fsd1 PE=1 SV=1 - [B1H2A2_RAT]
Protein Susd5 OS=Rattus norvegicus GN=Susd5 PE=4 SV=1 - [D3ZSC1_RAT]
Protein Sncaip OS=Rattus norvegicus GN=Sncaip PE=4 SV=1 - [D3ZWQ5_RAT]
Protein Atg2a OS=Rattus norvegicus GN=Atg2a PE=1 SV=1 - [D3ZT64_RAT]
Protein Scrt1 OS=Rattus norvegicus GN=Scrt1 PE=4 SV=1 - [D4A919_RAT]
CD248 antigen, endosialin (Predicted) OS=Rattus norvegicus GN=Cd248 PE=4 SV=1 - [D3ZN06_RAT]
Lactamase, beta (Predicted) OS=Rattus norvegicus GN=Lactb PE=1 SV=1 - [D3ZFJ6_RAT]
Resistance to inhibitors of cholinesterase 8 homolog A (C. elegans) OS=Rattus norvegicus GN=Ric8a PE=1 SV=1 -
Oxysterol-binding protein OS=Rattus norvegicus GN=Osbpl7 PE=1 SV=1 - [D4A0G0_RAT]
Olfactory receptor 1329 (Predicted) OS=Rattus norvegicus GN=Olr1329 PE=3 SV=1 - [A0A0G2K2E7_RAT]
Acyl-CoA-binding domain-containing protein 5 OS=Rattus norvegicus GN=Acbd5 PE=1 SV=1 - [A0A096MJJ6_RAT]
Copine 8 protein OS=Rattus norvegicus GN=Cpne8 PE=1 SV=1 - [B5DEX3_RAT]
B-cell CLL/lymphoma 7C (Predicted), isoform CRA_a OS=Rattus norvegicus GN=Bcl7c PE=1 SV=1 - [D3ZUL4_RA
Fxr2 protein OS=Rattus norvegicus GN=Fxr2 PE=1 SV=1 - [B1H2A6_RAT]
Cyclin-dependent kinase 17 OS=Rattus norvegicus GN=Cdk17 PE=1 SV=1 - [G3V6J5_RAT]
Histone-lysine N-methyltransferase SETD7 OS=Rattus norvegicus GN=Setd7 PE=1 SV=1 - [D4ADE5_RAT]
Protein Abcf2 OS=Rattus norvegicus GN=Abcf2 PE=1 SV=2 - [F1M8H5_RAT]
RCG21137 OS=Rattus norvegicus GN=Pgam5 PE=1 SV=1 - [G3V9N1_RAT]
Protein Dnajc19 OS=Rattus norvegicus GN=Dnajc19 PE=1 SV=1 - [M0R6L8_RAT]
RCG43241 OS=Rattus norvegicus GN=Dlc1 PE=1 SV=3 - [G3V7H1_RAT]
Protein Tex2 OS=Rattus norvegicus GN=Tex2 PE=1 SV=1 - [G3V9L1_RAT]
LOC682999 protein OS=Rattus norvegicus GN=Aida PE=1 SV=1 - [B1WBV1_RAT]
GTPase activating protein (SH3 domain) binding protein 2 OS=Rattus norvegicus GN=G3bp2 PE=1 SV=1 - [Q6AY2
BUD13 homolog OS=Rattus norvegicus GN=Bud13 PE=1 SV=1 - [G3V8F3_RAT]
Protein Ncbp3 OS=Rattus norvegicus GN=Ncbp3 PE=1 SV=1 - [D3ZXL5_RAT]
Canopy 2 homolog (Zebrafish) OS=Rattus norvegicus GN=Cnpy2 PE=1 SV=1 - [A0JN30_RAT]
Protein Zfp295 OS=Rattus norvegicus GN=Zfp295 PE=1 SV=3 - [D3ZVF2_RAT]
G protein-coupled receptor 180 OS=Rattus norvegicus GN=Gpr180 PE=2 SV=2 - [G3V799_RAT]
Phosphatidylinositol 3-kinase, C2 domain containing, alpha polypeptide (Predicted), isoform CRA_a OS=Rattus norv
Protein Ulk1 OS=Rattus norvegicus GN=Ulk1 PE=1 SV=2 - [D3ZMG0_RAT]
Proteasome subunit alpha type OS=Rattus norvegicus GN=Psma8 PE=3 SV=2 - [F1M6I7_RAT]
Ubiquitin carboxyl-terminal hydrolase 11 OS=Rattus norvegicus GN=Usp11 PE=1 SV=1 - [UBP11_RAT]
E3 ubiquitin-protein ligase MGRN1 isoform I OS=Rattus norvegicus GN=Mgrn1 PE=2 SV=1 - [G3C8Z1_RAT]
Protease, serine, 16 (Thymus) OS=Rattus norvegicus GN=Prss16 PE=2 SV=1 - [Q3MHS0_RAT]
Protein Cmtr2 OS=Rattus norvegicus GN=Cmtr2 PE=4 SV=1 - [D3Z980_RAT]
Coatomer protein complex, subunit epsilon OS=Rattus norvegicus GN=Cope PE=2 SV=1 - [B1WBX7_RAT]
Centromere protein A OS=Rattus norvegicus GN=Cenpa PE=1 SV=1 - [B2RZ23_RAT]
Protein Zbtb37 OS=Rattus norvegicus GN=Zbtb37 PE=4 SV=1 - [D3ZGJ3_RAT]
Protein Sec23ip OS=Rattus norvegicus GN=Sec23ip PE=1 SV=1 - [G3V8Q8_RAT]
Protein Rrp9 OS=Rattus norvegicus GN=Rrp9 PE=1 SV=1 - [B0BND5_RAT]

Nicotinamide nucleotide transhydrogenase OS=Rattus norvegicus GN=Nnt PE=1 SV=1 - [Q5BJZ3_RAT]
Protein Snrnp70 OS=Rattus norvegicus GN=Snrnp70 PE=1 SV=1 - [B2RZ74_RAT]
Protein Stard3nl OS=Rattus norvegicus GN=Stard3nl PE=1 SV=1 - [Q5U205_RAT]
Ectonucleoside triphosphate diphosphohydrolase 3, isoform CRA_a OS=Rattus norvegicus GN=Entpd3 PE=1 SV=1
Ceh-10 homeo domain containing homolog (C. elegans), isoform CRA_a OS=Rattus norvegicus GN=Vsx2 PE=3 S\
3-oxoacyl-[acyl-carrier-protein] synthase, mitochondrial OS=Rattus norvegicus GN=Oxsm PE=1 SV=1 - [G3V6R7_R
Leucyl-tRNA synthetase OS=Rattus norvegicus GN=Lars PE=1 SV=1 - [Q5PPJ6_RAT]
Polynucleotide kinase 3'-phosphatase OS=Rattus norvegicus GN=Pnkp PE=1 SV=1 - [Q6AXV0_RAT]
DiGeorge syndrome critical region gene 8 (Predicted), isoform CRA_a OS=Rattus norvegicus GN=Dgcr8 PE=1 SV=
SHC-transforming protein 3 OS=Rattus norvegicus GN=Shc3 PE=4 SV=1 - [G3V7V0_RAT]
Protein Rimkla OS=Rattus norvegicus GN=Rimkla PE=1 SV=1 - [D4A7C3_RAT]
Protein Marveld3 OS=Rattus norvegicus GN=Marveld3 PE=4 SV=1 - [A0A0G2K6L1_RAT]
Protein Tbk1 OS=Rattus norvegicus GN=Tbk1 PE=1 SV=1 - [D4A7D3_RAT]
Gamma-aminobutyric acid (GABA) B receptor 1 OS=Rattus norvegicus GN=Gabbr1 PE=1 SV=1 - [Q6MFX8_RAT]
Protein Rad23a OS=Rattus norvegicus GN=Rad23a PE=1 SV=1 - [Q5XFX7_RAT]
Protein Zfp444 OS=Rattus norvegicus GN=Zfp444 PE=1 SV=1 - [D3ZV88_RAT]
Protein Zfp483 OS=Rattus norvegicus GN=Zfp483 PE=2 SV=1 - [Q99PJ8_RAT]
Protein Cnksr1 OS=Rattus norvegicus GN=Cnksr1 PE=1 SV=1 - [G3V8W8_RAT]
DEAD (Asp-Glu-Ala-Asp) box polypeptide 47, isoform CRA_a OS=Rattus norvegicus GN=Ddx47 PE=1 SV=1 - [G3\
Centrin 2, isoform CRA_a OS=Rattus norvegicus GN=Cetn2 PE=1 SV=1 - [G3V9W0_RAT]
Anaphase promoting complex subunit 4, isoform CRA_a OS=Rattus norvegicus GN=Anapc4 PE=1 SV=1 - [D3ZUB
RCG44919, isoform CRA_b OS=Rattus norvegicus GN=Tcp1 PE=1 SV=1 - [A0A096MJA0_RAT]
Bystin OS=Rattus norvegicus GN=Bysl PE=1 SV=1 - [M0RDF7_RAT]
Palmitoyltransferase OS=Rattus norvegicus GN=Zdhhc14 PE=2 SV=1 - [Q2TGJ5_RAT]
Protein Champ1 OS=Rattus norvegicus GN=Champ1 PE=1 SV=1 - [B5DEG7_RAT]
Itfg2 protein OS=Rattus norvegicus GN=Itfg2 PE=1 SV=1 - [B0BN77_RAT]
Protein Vom1r43 OS=Rattus norvegicus GN=Vom1r43 PE=2 SV=1 - [Q5J3J1_RAT]
Protein Gpatch11 OS=Rattus norvegicus GN=Gpatch11 PE=1 SV=2 - [F1LV52_RAT]
Pms2 protein OS=Rattus norvegicus GN=Pms2 PE=1 SV=1 - [B1H246_RAT]
Fibronectin OS=Rattus norvegicus GN=Fn1 PE=1 SV=3 - [F1LST1_RAT]
KOR-3A splice variant OS=Rattus norvegicus GN=Oprl1 PE=2 SV=1 - [Q8CH83_RAT]
Methionine aminopeptidase 2 OS=Rattus norvegicus GN=Metap2 PE=1 SV=1 - [Q6IRK1_RAT]
Zinc finger protein 423 OS=Rattus norvegicus GN=Zfp423 PE=4 SV=3 - [F1LR04_RAT]
Junctophilin 1 (Predicted) OS=Rattus norvegicus GN=Jph1 PE=1 SV=1 - [D3ZQ55_RAT]
Protein Smarcc1 OS=Rattus norvegicus GN=Smarcc1 PE=1 SV=1 - [D3ZJU5_RAT]
Apoptosis-inducing factor 1, mitochondrial OS=Rattus norvegicus GN=Aifm1 PE=1 SV=1 - [A0A0G2K7K2_RAT]
Protein Plcxd3 OS=Rattus norvegicus GN=Plcxd3 PE=4 SV=1 - [D4A1H2_RAT]
G protein-coupled receptor, family C, group 5, member B (Predicted), isoform CRA_a OS=Rattus norvegicus GN=G
Inositol polyphosphate-1-phosphatase OS=Rattus norvegicus GN=Inpp1 PE=1 SV=1 - [Q5RJK6_RAT]
Regulating synaptic membrane exocytosis 3, isoform CRA_a OS=Rattus norvegicus GN=Rims3 PE=1 SV=1 - [G3V
Protein Wwc2 OS=Rattus norvegicus GN=Wwc2 PE=1 SV=1 - [D4AEJ5_RAT]
Protein Sec61b OS=Rattus norvegicus GN=Sec61b PE=1 SV=1 - [B2RZD1_RAT]
Centaurin, beta 5 (Predicted), isoform CRA_a OS=Rattus norvegicus GN=Acap3 PE=1 SV=1 - [D4A346_RAT]
Nuclear receptor coactivator 5 (Predicted) OS=Rattus norvegicus GN=Ncoa5 PE=1 SV=1 - [D3ZEI6_RAT]
Cyclin M1 (Predicted) OS=Rattus norvegicus GN=Cnnm1 PE=1 SV=2 - [D4A1C0_RAT]
Protein Rgs17 OS=Rattus norvegicus GN=Rgs17 PE=4 SV=2 - [F1M0G0_RAT]
Protein Rbm6 OS=Rattus norvegicus GN=Rbm6 PE=1 SV=1 - [D4ACW0_RAT]
Biliverdin reductase B (Flavin reductase (NADPH)) OS=Rattus norvegicus GN=Blvrb PE=1 SV=1 - [B5DF65_RAT]
Protein Rbm15 OS=Rattus norvegicus GN=Rbm15 PE=1 SV=2 - [M0R3Z8_RAT]
Achalasia, adrenocortical insufficiency, alacrimia (Allgrove, triple-A) (Predicted), isoform CRA_a OS=Rattus norvegi
Protein Sestd1 OS=Rattus norvegicus GN=Sestd1 PE=2 SV=1 - [B5DFL9_RAT]
Kruppel-like factor 3 (Basic) (Mapped), isoform CRA_b OS=Rattus norvegicus GN=Klf3 PE=1 SV=1 - [D4A4V3_RA
Protein LOC499276 OS=Rattus norvegicus GN=LOC499276 PE=4 SV=1 - [F7FD99_RAT]
Caspase recruitment domain family, member 6 (Predicted) OS=Rattus norvegicus GN=Card6 PE=1 SV=1 - [D3ZVT
Protein Sh2d2a OS=Rattus norvegicus GN=Sh2d2a PE=2 SV=1 - [Q5I0Q2_RAT]

Protein Fam217b OS=Rattus norvegicus GN=Fam217b PE=4 SV=1 - [A0A0G2K2Y5_RAT]
Minichromosome maintenance deficient 10 (S. cerevisiae) (Predicted) OS=Rattus norvegicus GN=Mcm10 PE=4 SV
Retinoblastoma-like 2, isoform CRA_a OS=Rattus norvegicus GN=Rbl2 PE=1 SV=1 - [G3V7P7_RAT]
NADH dehydrogenase (Ubiquinone) 1 beta subcomplex, 2 (Predicted), isoform CRA_b OS=Rattus norvegicus GN=I
Protein Sh2d3c OS=Rattus norvegicus GN=Sh2d3c PE=1 SV=1 - [B0BN10_RAT]
High mobility group nucleosomal binding domain 3, isoform CRA_a OS=Rattus norvegicus GN=Hmgn3 PE=1 SV=1
Protein Cys1 OS=Rattus norvegicus GN=Cys1 PE=4 SV=1 - [D3ZCZ3_RAT]
Protein Eif4enif1 OS=Rattus norvegicus GN=Eif4enif1 PE=1 SV=1 - [D4ACF1_RAT]
Epithelial protein lost in neoplasm OS=Rattus norvegicus GN=Lima1 PE=1 SV=2 - [F1LR10_RAT]
Transcription elongation factor SPT5 OS=Rattus norvegicus GN=Supt5h PE=1 SV=2 - [E9PTB2_RAT]
Peptidyl-prolyl cis-trans isomerase OS=Rattus norvegicus PE=3 SV=1 - [D3ZSF3_RAT]
Protein Pum1 OS=Rattus norvegicus GN=Pum1 PE=1 SV=2 - [D3Z8L5_RAT]
Protein tweety homolog OS=Rattus norvegicus GN=Ttyh2 PE=1 SV=2 - [D4A4P8_RAT]
Protein Mbnl1 OS=Rattus norvegicus GN=Mbnl1 PE=1 SV=2 - [F1M9N4_RAT]
Mitogen-activated protein kinase kinase kinase kinase 4 (Fragment) OS=Rattus norvegicus GN=Map4k4 PE=2 SV=
Band 4.1-like protein 4B OS=Rattus norvegicus GN=Epb41l4b PE=1 SV=1 - [A0A0G2K3H4_RAT]
Uncharacterized protein OS=Rattus norvegicus PE=1 SV=1 - [M0R970_RAT]
Protein Tcp11l1 OS=Rattus norvegicus GN=Tcp11l1 PE=1 SV=3 - [F1M9Y7_RAT]
Uncharacterized protein OS=Rattus norvegicus GN=Akap10 PE=2 SV=1 - [Q66HA0_RAT]
Protein Ttll12 OS=Rattus norvegicus GN=Ttll12 PE=1 SV=1 - [D4A1Q9_RAT]
Protein Supt6h OS=Rattus norvegicus GN=Supt6h PE=1 SV=1 - [A0A0G2K0J0_RAT]
Protein Dopey2 OS=Rattus norvegicus GN=Dopey2 PE=1 SV=1 - [A0A0G2JXD9_RAT]
Protein LOC688906 OS=Rattus norvegicus GN=Scaf11 PE=1 SV=3 - [D4ABH1_RAT]
Protein Pithd1 OS=Rattus norvegicus GN=Pithd1 PE=1 SV=2 - [D4ABS5_RAT]
Protein Fgd2 OS=Rattus norvegicus GN=Fgd2 PE=1 SV=1 - [D3Z9I3_RAT]
Sdccag1 protein (Fragment) OS=Rattus norvegicus GN=Nemf PE=2 SV=1 - [Q5D022_RAT]
Protein Fam184a OS=Rattus norvegicus GN=Fam184a PE=4 SV=3 - [F1LW90_RAT]
Tensin3 OS=Rattus norvegicus GN=TSN3 PE=2 SV=1 - [C5NTX8_RAT]
Protein Celsr1 OS=Rattus norvegicus GN=Celsr1 PE=3 SV=3 - [F1MAS4_RAT]
Methyl-CpG-binding domain protein 4 OS=Rattus norvegicus GN=Mbd4 PE=4 SV=3 - [D4A9W8_RAT]
MICOS complex subunit OS=Rattus norvegicus GN=Apoo PE=1 SV=2 - [M0R7G4_RAT]
Protein Frmd3 OS=Rattus norvegicus GN=Frmd3 PE=4 SV=3 - [D3ZR47_RAT]
Protein Mdn1 (Fragment) OS=Rattus norvegicus GN=Mdn1 PE=1 SV=1 - [A0A096MJ77_RAT]
Histone acetyltransferase (Fragment) OS=Rattus norvegicus GN=HBO1 PE=2 SV=1 - [A7BJV7_RAT]
Membrane-associated guanylate kinase, WW and PDZ domain-containing protein 1 OS=Rattus norvegicus GN=Ma
Glutamate receptor ionotropic, NMDA 3B OS=Rattus norvegicus GN=Grin3b PE=3 SV=2 - [F1LPZ6_RAT]
Potassium voltage-gated channel subfamily G member 3 OS=Rattus norvegicus GN=Kcng3 PE=3 SV=1 - [F1LQV8_
Oxysterol-binding protein OS=Rattus norvegicus GN=Osbpl3 PE=1 SV=2 - [D3ZHZ3_RAT]
Metabotropic glutamate receptor type 1 (Fragment) OS=Rattus norvegicus GN=Grm1 PE=2 SV=1 - [Q9WUY1_RAT
Protein LOC685025 OS=Rattus norvegicus GN=LOC685025 PE=4 SV=2 - [M0R965_RAT]
Protein Sh3rf3 OS=Rattus norvegicus GN=Sh3rf3 PE=4 SV=2 - [M0R6D9_RAT]
Protein Dchs2 OS=Rattus norvegicus GN=Dchs2 PE=4 SV=2 - [F1M5X7_RAT]
Protein Dnah8 OS=Rattus norvegicus GN=Dnah8 PE=1 SV=3 - [F1MAM6_RAT]
Protein Catsper3 OS=Rattus norvegicus GN=Catsper3 PE=1 SV=3 - [F1LZS9_RAT]
Protein Wdfy4 OS=Rattus norvegicus GN=Wdfy4 PE=1 SV=3 - [D4A748_RAT]
Protein Jakmip3 OS=Rattus norvegicus GN=Jakmip3 PE=1 SV=1 - [A0A0G2K400_RAT]
Protein Tmed8 OS=Rattus norvegicus GN=Tmed8 PE=1 SV=2 - [M0RAT6_RAT]
Phosphoprotein-associated with glycosphingolipid-enriched microdomains 1 OS=Rattus norvegicus GN=Pag1 PE=1
Protein Plxnb1 OS=Rattus norvegicus GN=Plxnb1 PE=1 SV=1 - [D3ZDX5_RAT]
Rho GTPase-activating protein 27 OS=Rattus norvegicus GN=Arhgap27 PE=1 SV=1 - [F1LQ24_RAT]
Protein Arid1a OS=Rattus norvegicus GN=Arid1a PE=1 SV=3 - [D4A3E3_RAT]
Protein Larp4b OS=Rattus norvegicus GN=Larp4b PE=1 SV=2 - [D3ZF45_RAT]
Protein Bcr OS=Rattus norvegicus GN=Bcr PE=1 SV=3 - [F1LXF1_RAT]
RAC-gamma serine/threonine-protein kinase OS=Rattus norvegicus GN=Akt3 PE=3 SV=3 - [F1M6A8_RAT]
Protein Zfp462 OS=Rattus norvegicus GN=Zfp462 PE=4 SV=2 - [D3ZFG7_RAT]

Protein Mllt6 OS=Rattus norvegicus GN=Mllt6 PE=1 SV=1 - [A0A0G2K809_RAT]
Protein Plekhm3 OS=Rattus norvegicus GN=Plekhm3 PE=1 SV=1 - [D4A959_RAT]
Psmd8 protein (Fragment) OS=Rattus norvegicus GN=Psmd8 PE=2 SV=1 - [Q3B8P5_RAT]
Protein Ahctf1 OS=Rattus norvegicus GN=Ahctf1 PE=1 SV=1 - [D3ZZZ0_RAT]
Protein Swt1 OS=Rattus norvegicus GN=Swt1 PE=4 SV=1 - [A0A0G2K2J8_RAT]
Cyclin-dependent kinase 7 OS=Rattus norvegicus GN=Cdk7 PE=1 SV=3 - [F1LQC8_RAT]
Protein LOC499229 OS=Rattus norvegicus GN=LOC499229 PE=1 SV=1 - [A0A0G2JTA4_RAT]
Protein Dsp OS=Rattus norvegicus GN=Dsp PE=1 SV=1 - [F1LMV6_RAT]
Glucocorticoid receptor OS=Rattus norvegicus GN=Nr3c1 PE=1 SV=3 - [E9PT44_RAT]
Protein LOC102554602 OS=Rattus norvegicus GN=LOC102554602 PE=4 SV=1 - [D3Z8W1_RAT]
Uncharacterized protein OS=Rattus norvegicus PE=1 SV=3 - [F1LQR8_RAT]
Protein Suds3 OS=Rattus norvegicus GN=Suds3 PE=1 SV=2 - [M0RBT5_RAT]
Pleckstrin homology domain-containing family O member 1 OS=Rattus norvegicus GN=Plekho1 PE=1 SV=1 - [A0A(
Wdr22 protein (Fragment) OS=Rattus norvegicus GN=Dcaf5 PE=2 SV=1 - [Q68FQ3_RAT]
Protein Flt3 OS=Rattus norvegicus GN=Flt3 PE=3 SV=1 - [A0A0G2JW59_RAT]
Protein Unc79 OS=Rattus norvegicus GN=Unc79 PE=4 SV=3 - [D3ZSV8_RAT]
AMP deaminase 2 OS=Rattus norvegicus GN=Ampd2 PE=1 SV=1 - [A0A0G2K3U1_RAT]
Inositol 1,4,5-trisphosphate receptor type 1 OS=Rattus norvegicus GN=Itpr1 PE=1 SV=1 - [A0A0G2KAH9_RAT]
Rap2ip protein (Fragment) OS=Rattus norvegicus GN=Rap2ip PE=2 SV=1 - [B0BMV0_RAT]
Multidrug resistance-associated protein 5 OS=Rattus norvegicus GN=Abcc5 PE=4 SV=3 - [G3V676_RAT]
Protein Wdr90 OS=Rattus norvegicus GN=Wdr90 PE=1 SV=2 - [D3ZMM2_RAT]
Protein Ralgps1 OS=Rattus norvegicus GN=Ralgps1 PE=4 SV=1 - [D3ZLR4_RAT]
Liprin beta 2 (Fragment) OS=Rattus norvegicus GN=Ppfibp2 PE=2 SV=1 - [Q5UDQ9_RAT]
Syntaxin-12 OS=Rattus norvegicus GN=Stx12 PE=1 SV=1 - [A0A0G2K4U8_RAT]
Protein Dock3 OS=Rattus norvegicus GN=Dock3 PE=1 SV=3 - [F1M4N6_RAT]
Proteasome inhibitor PI31 subunit OS=Rattus norvegicus GN=Psmf1 PE=1 SV=2 - [F1M7S2_RAT]
Protein Cdr2l OS=Rattus norvegicus GN=Cdr2l PE=4 SV=2 - [D4ABP3_RAT]
Protein Shroom1 OS=Rattus norvegicus GN=Shroom1 PE=1 SV=2 - [M0RB44_RAT]
Diacylglycerol kinase OS=Rattus norvegicus GN=Dgki PE=1 SV=1 - [F1MAB7_RAT]
Tyrosine-protein kinase receptor TYRO3 OS=Rattus norvegicus GN=Tyro3 PE=1 SV=1 - [F1M7U7_RAT]
Gorasp1 protein (Fragment) OS=Rattus norvegicus GN=Gorasp1 PE=2 SV=1 - [A0JN16_RAT]
Protein Itsn2 OS=Rattus norvegicus GN=Itsn2 PE=1 SV=2 - [M0R7A6_RAT]
Protein Tbc1d1 OS=Rattus norvegicus GN=Tbc1d1 PE=1 SV=1 - [D4AC16_RAT]
Protein Prdm1 OS=Rattus norvegicus GN=Prdm1 PE=4 SV=1 - [D4A1S2_RAT]
Eyes absent homolog OS=Rattus norvegicus GN=Eya4 PE=3 SV=3 - [F1LX86_RAT]
Protein Nek1 OS=Rattus norvegicus GN=Nek1 PE=1 SV=1 - [A0A0G2K5C7_RAT]
Protein Rnf207 OS=Rattus norvegicus GN=Rnf207 PE=4 SV=1 - [D4A306_RAT]
Protein Fhod3 OS=Rattus norvegicus GN=Fhod3 PE=4 SV=3 - [F1LQJ2_RAT]
Protein Slc4a1ap OS=Rattus norvegicus GN=Slc4a1ap PE=1 SV=3 - [D3ZTF1_RAT]
Protein Trappc12 OS=Rattus norvegicus GN=Trappc12 PE=1 SV=1 - [D3ZE49_RAT]
ELKS/Rab6-interacting/CAST family member 1 OS=Rattus norvegicus GN=Erc1 PE=1 SV=1 - [F1LPE9_RAT]
Protein Nuggc OS=Rattus norvegicus GN=Nuggc PE=4 SV=3 - [F1M575_RAT]
Ubiquitin conjugation factor E4 A OS=Rattus norvegicus GN=Ube4a PE=1 SV=1 - [F1M9N5_RAT]
Eukaryotic translation initiation factor 3 subunit F OS=Rattus norvegicus GN=Eif3f PE=1 SV=3 - [D4AC36_RAT]
Tle3 protein OS=Rattus norvegicus GN=Tle3 PE=2 SV=1 - [Q4V8F0_RAT]
Protein LOC679087 OS=Rattus norvegicus GN=LOC679087 PE=4 SV=1 - [A0A0G2JYP3_RAT]
Protein Zfp318 OS=Rattus norvegicus GN=Zfp318 PE=1 SV=2 - [M0RD44_RAT]
Protein Nktr OS=Rattus norvegicus GN=Nktr PE=1 SV=2 - [M0R991_RAT]
Metalloproteinase (Fragment) OS=Rattus norvegicus GN=Slc7a6os PE=2 SV=1 - [Q8CIV0_RAT]
Spliceosomal protein SAP155 (Fragment) OS=Rattus norvegicus GN=Sf3b1 PE=2 SV=1 - [Q9ET34_RAT]
A-kinase anchor protein 5 OS=Rattus norvegicus GN=Akap5 PE=1 SV=1 - [F1LPP6_RAT]
Fibroblast growth factor OS=Rattus norvegicus GN=Fgf13 PE=1 SV=1 - [A0A0G2JZ77_RAT]
Beta II spectrin (Fragment) OS=Rattus norvegicus GN=Sptbn1 PE=2 SV=1 - [Q9WUX0_RAT]
Serine/threonine-protein phosphatase 2A 55 kDa regulatory subunit B OS=Rattus norvegicus GN=Ppp2r2c PE=3 S\
Protein Tbc1d4 OS=Rattus norvegicus GN=Tbc1d4 PE=1 SV=3 - [D3Z881_RAT]

Protein Alpk3 OS=Rattus norvegicus GN=Alpk3 PE=1 SV=2 - [D3ZH28_RAT]
Kinesin-like protein KIF1C OS=Rattus norvegicus GN=Kif1c PE=1 SV=1 - [F1M9C8_RAT]
Ubiquitin specific protease 11 OS=Rattus norvegicus GN=Usp11 PE=2 SV=1 - [Q6IE71_RAT]
Endothelin-converting enzyme 2 OS=Rattus norvegicus GN=Ece2 PE=2 SV=1 - [Q6IE65_RAT]
Protein Sfpq OS=Rattus norvegicus GN=Sfpq PE=1 SV=1 - [A0A0G2K8K0_RAT]
ATPase WRNIP1 OS=Rattus norvegicus GN=Wrnip1 PE=1 SV=1 - [R9PXV8_RAT]
Protein Ccdc18 OS=Rattus norvegicus GN=Ccdc18 PE=1 SV=3 - [D4A317_RAT]
A-kinase anchor protein 2 OS=Rattus norvegicus GN=Akap2 PE=1 SV=2 - [F1LPQ9_RAT]
Protein Fcgbp OS=Rattus norvegicus GN=Fcgbp PE=1 SV=3 - [D3ZJF8_RAT]
Protein Fbxo42 OS=Rattus norvegicus GN=Fbxo42 PE=4 SV=1 - [A0A0G2JTS9_RAT]
Protein Ndufb4 OS=Rattus norvegicus GN=Ndufb4 PE=1 SV=1 - [F1LPG5_RAT]
Guanine nucleotide-binding protein G(olf) subunit alpha OS=Rattus norvegicus GN=Gnal PE=1 SV=1 - [A0A0G2K52
Piezo-type mechanosensitive ion channel component OS=Rattus norvegicus GN=Piezo2 PE=3 SV=3 - [F1M208_RA
CAMPS OS=Rattus norvegicus GN=Rapgef4 PE=2 SV=1 - [D3KR63_RAT]
Protein Stim2 OS=Rattus norvegicus GN=Stim2 PE=1 SV=2 - [D4A5X1_RAT]
Neuroligin-3 OS=Rattus norvegicus GN=Nlgn3 PE=1 SV=1 - [D3ZDC0_RAT]
Protein Dennd2a OS=Rattus norvegicus GN=Dennd2a PE=1 SV=1 - [D3ZLQ1_RAT]
Protein Kmt2a OS=Rattus norvegicus GN=Kmt2a PE=1 SV=3 - [F1M0L3_RAT]
Protein Cmtm4 OS=Rattus norvegicus GN=Cmtm4 PE=4 SV=1 - [D4A110_RAT]
CXXC-type zinc finger protein 4 OS=Rattus norvegicus GN=Cxxc4 PE=4 SV=1 - [M0RDW5_RAT]
Lingo1 protein (Fragment) OS=Rattus norvegicus GN=Lingo1 PE=2 SV=1 - [Q562A6_RAT]
C3G protein (Fragment) OS=Rattus norvegicus GN=Rapgef1 PE=2 SV=1 - [Q9QYV3_RAT]
Protein LOC500684 OS=Rattus norvegicus GN=LOC500684 PE=4 SV=2 - [D3ZEL0_RAT]
Voltage-dependent L-type calcium channel subunit alpha-1C OS=Rattus norvegicus GN=Cacna1c PE=1 SV=3 - [E9
Cation-transporting ATPase OS=Rattus norvegicus GN=Atp13a5 PE=3 SV=2 - [F1MA70_RAT]
Protein Pcp4l1 OS=Rattus norvegicus GN=Pcp4l1 PE=1 SV=1 - [A0A0G2KBA5_RAT]
Protein Npm3 OS=Rattus norvegicus GN=Npm3 PE=1 SV=1 - [D3ZYK9_RAT]
Band 4.1-like protein 4B OS=Rattus norvegicus GN=Epb41l4b PE=1 SV=1 - [A0A0G2JU16_RAT]
Protein Pom121l2 OS=Rattus norvegicus GN=Pom121l2 PE=4 SV=2 - [D3ZGH9_RAT]
p55 protein OS=Rattus norvegicus GN=LOC652956 PE=2 SV=1 - [Q5BK33_RAT]
Piezo-type mechanosensitive ion channel component OS=Rattus norvegicus GN=Piezo1 PE=1 SV=1 - [A0A0G2JW
Cullin-3 OS=Rattus norvegicus GN=Cul3 PE=1 SV=1 - [A0A0G2JSP3_RAT]
RNA-binding protein Nova-1 OS=Rattus norvegicus GN=Nova1 PE=4 SV=2 - [D4AAF8_RAT]
Guanine nucleotide exchange factor for Rab-3A OS=Rattus norvegicus GN=Rab3il1 PE=1 SV=1 - [F1LPG6_RAT]
Protein Ppil4 OS=Rattus norvegicus GN=Ppil4 PE=1 SV=3 - [D4AEG3_RAT]
Glutamate receptor ionotropic, delta-1 OS=Rattus norvegicus GN=Grid1 PE=3 SV=2 - [F1LUR6_RAT]
Protein Exoc1 OS=Rattus norvegicus GN=Exoc1 PE=1 SV=3 - [F1LQN9_RAT]
Prohibitin-2 OS=Rattus norvegicus GN=Phb2 PE=1 SV=1 - [A0A0G2KB63_RAT]
Neurocan (Fragment) OS=Rattus norvegicus GN=Ncan PE=2 SV=1 - [O70474_RAT]
11beta-hydroxysteroid dehydrogenase type 1 (Fragment) OS=Rattus norvegicus GN=Hsd11b1 PE=2 SV=1 - [Q8CI
Protein Mfsd8 OS=Rattus norvegicus GN=Mfsd8 PE=4 SV=1 - [A0A0G2JYD6_RAT]
E3 ubiquitin-protein ligase rififylin OS=Rattus norvegicus GN=Rffl PE=1 SV=1 - [A0A096MJC6_RAT]
Protein Map7 OS=Rattus norvegicus GN=Map7 PE=1 SV=3 - [F1MA82_RAT]
Protein Drosha OS=Rattus norvegicus GN=Drosha PE=3 SV=3 - [E9PTR3_RAT]
Transient receptor potential cation channel subfamily M member 3 (Fragment) OS=Rattus norvegicus GN=Trpm3 P
Canalicular multispecific organic anion transporter 2 OS=Rattus norvegicus GN=Abcc3 PE=1 SV=1 - [A0A0G2K4K3
Uncharacterized protein OS=Rattus norvegicus GN=LOC367515 PE=2 SV=1 - [Q6AYM6_RAT]
Acid-soluble phosphoprotein 22 K OS=Rattus norvegicus PE=1 SV=1 - [Q7M0D5_RAT]
Eukaryotic translation initiation factor 1A OS=Rattus norvegicus GN=Eif1a PE=1 SV=1 - [A0A0G2JVA7_RAT]
Serine/threonine-protein phosphatase OS=Rattus norvegicus GN=Ppp1ca PE=1 SV=1 - [A0A0G2JYS8_RAT]
Tyrosine-protein phosphatase non-receptor type OS=Rattus norvegicus GN=Ptpn3 PE=1 SV=1 - [A0A096MJT2_RA
Protein LOC100910255 OS=Rattus norvegicus GN=LOC100910255 PE=1 SV=2 - [M0R3Z1_RAT]
Protein Rusc2 OS=Rattus norvegicus GN=Rusc2 PE=1 SV=2 - [M0RCV5_RAT]
Protein Pid1 OS=Rattus norvegicus GN=Pid1 PE=1 SV=1 - [A0A0G2K8X3_RAT]
60S ribosomal protein L13 OS=Rattus norvegicus PE=3 SV=1 - [A0A0G2JYG9_RAT]

Information Classification: General

Protein LOC100362109 OS=Rattus norvegicus GN=LOC100362109 PE=4 SV=2 - [F1LZD2_RAT]
Katanin p80 WD40 repeat-containing subunit B1 OS=Rattus norvegicus GN=Katnb1 PE=3 SV=1 - [A0A0G2JX20_R
Solute carrier family 2, facilitated glucose transporter member 1 OS=Rattus norvegicus GN=Slc2a1 PE=1 SV=1 - [A
Protein Fam53c OS=Rattus norvegicus GN=Fam53c PE=1 SV=1 - [D3ZH55_RAT]
Protein LOC685321 OS=Rattus norvegicus GN=LOC685321 PE=4 SV=1 - [A0A0G2K036_RAT]
Polymerase (DNA-directed), delta 3, accessory subunit OS=Rattus norvegicus GN=Pold3 PE=2 SV=1 - [Q4V7D0_R
Protein Fbn2 OS=Rattus norvegicus GN=Fbn2 PE=4 SV=3 - [F1M5Q4_RAT]
Protein Vash1 OS=Rattus norvegicus GN=Vash1 PE=4 SV=1 - [D4AE85_RAT]
High affinity cAMP-specific 3',5'-cyclic phosphodiesterase 7A OS=Rattus norvegicus GN=Pde7a PE=3 SV=1 - [A0A
Protein Tbc1d22b OS=Rattus norvegicus GN=Tbc1d22b PE=1 SV=1 - [A0A0G2JWM9_RAT]
Protein Atad2b OS=Rattus norvegicus GN=LOC103692579 PE=1 SV=1 - [A0A0G2JX30_RAT]
Protein Adgrb2 OS=Rattus norvegicus GN=Adgrb2 PE=1 SV=1 - [A0A0G2K6N2_RAT]
RGD1562161 protein OS=Rattus norvegicus GN=RGD1562161 PE=2 SV=1 - [B2GUW9_RAT]
Dendrin OS=Rattus norvegicus GN=Ddn PE=4 SV=1 - [A0A0G2JU85_RAT]
Yotiao protein (Fragment) OS=Rattus norvegicus GN=Akap9 PE=2 SV=1 - [Q9JHE0_RAT]
CENP-C (Fragment) OS=Rattus norvegicus PE=2 SV=1 - [Q66LH8_RAT]
Glycine receptor alpha 1 variant OS=Rattus norvegicus PE=2 SV=1 - [Q5PSQ6_RAT]
Ubiquilin 1, isoform CRA_a OS=Rattus norvegicus GN=Ubqln1 PE=1 SV=1 - [A0A140TAI1_RAT]
Adenosylhomocysteinase 2 OS=Rattus norvegicus GN=Ahcyl1 PE=1 SV=1 - [A0A140TAI8_RAT]
NMDA receptor synaptonuclear-signaling and neuronal migration factor OS=Rattus norvegicus GN=Nsmf PE=1 SV=
MICOS complex subunit MIC60 OS=Rattus norvegicus GN=Immt PE=1 SV=1 - [A0A140TAG5_RAT]
E3 ubiquitin-protein ligase MIB2 OS=Rattus norvegicus GN=Mib2 PE=1 SV=1 - [A0A140TAC8_RAT]
Transcription elongation factor B polypeptide 2 OS=Rattus norvegicus GN=Tceb2 PE=1 SV=1 - [A0A140TA99_RAT

mbering in the Protein Sequence Database (FASTA database); Description, Protein functional description

ides from the SFO in rats with chronic renal failure.

37.94	1	2	269.334233	4.80517578
33.85	1	32	299.348366	4.94482422
37.69	1	4	155.691586	5.66845703
71.62	3	9	50.1036119	5.05908203
56.03	2	4	49.8923716	5.05908203
17.09	7	1	427.001686	5.14794922
16.71	7	2	429.639143	5.17333984
37.81	6	18	111.619932	5.41455078
71.9	18	31	47.2385508	8.89501953
34.86	2	7	23.532143	9.40771484
34.7	2	21	88.4841607	8.17724609
8.47	2	1	25.8711257	5.51611328
48.21	3	100	114.342814	5.80810547
70.8	2	18	23.5892843	4.70361328
42.43	2	37	95.7335817	4.79248047
32.84	6	14	112.145477	5.55419922
26.9	6	1	198.783838	4.83056641
23.65	5	22	126.324127	4.48779297
27.57	2	11	112.981868	5.45263672
50.46	4	12	47.0451555	6.36669922
24.25	1	10	78.3070755	7.45947266
65.31	2	9	27.7537453	4.79248047
64	8	1	41.7097321	5.47802734
64	8	1	41.7657947	5.47802734
26.74	6	1	198.341596	4.84326172
34.52	6	13	54.080549	7.07861328
19.05	3	2	76.6650878	6.93212891
20.41	2	3	80.2892924	6.15087891
23.16	1	2	74.7385593	6.45556641
28.47	1	7	33.2238319	5.37646484
48.6	1	15	62.2385939	6.37939453
59.8	1	4	56.0816431	5.22412109
33.33	3	7	39.2303404	5.49072266
46.31	2	9	47.1109186	5.16064453
56.13	1	1	56.223681	5.19873047
29.83	2	13	83.2291212	5.03369141
19	10	1	107.022442	5.23681641
23.03	2	22	80.526209	5.97314453
72.75	2	4	49.6389736	4.89404297
25.66	6	20	71.7218631	5.73193359
46.56	4	6	28.2849139	4.89404297
22.47	2	21	181.001187	4.39892578
67.52	3	1	61.1475263	4.85595703
73.65	1	5	49.5539031	4.88134766
27.07	4	6	45.8049327	5.41455078
3.63	2	4	255.212169	4.86865234
65.17	2	5	49.8749588	4.89404297
8.6	3	5	81.3761224	7.04931641
9.75	1	20	341.078026	6.37939453
45.88	1	9	29.1554174	4.74169922
13.67	6	26	273.146009	5.73193359
23.66	2	4	33.5116308	5.42724609

Information Classification: General

19.93	11	1	102.629006	5.19873047
9.25	2	1	395.101002	5.55419922
31.6	2	7	33.0467093	5.23681641
56.83	1	28	61.2980407	4.65283203
23.13	4	7	66.8858474	4.52587891
6.2	3	12	417.769602	7.59130859
6.45	1	2	552.375981	6.53662109
27.54	1	5	36.5890676	6.04931641
23.91	3	16	61.825061	6.85888672
66.36	1	1	21.7774428	4.51318359
59.38	1	5	18.7094194	4.55126953
12.53	5	4	47.1203487	8.86572266
4.68	3	5	95.4271327	4.95751953
56.1	4	6	28.0368369	4.88134766
36.89	2	8	29.9419647	4.36083984
61.54	3	17	21.4889681	11.2387695
4.11	2	1	61.8991382	7.48876953
23.62	6	4	60.4226733	7.28369141
15.41	14	25	170.964019	5.33837891
39.68	2	5	14.6276917	7.48876953
8.81	2	18	233.249899	5.61767578
6.48	5	8	228.856926	5.54150391
52.36	3	14	56.3094842	5.27490234
25.65	1	10	60.9173932	6.17626953
61.68	3	15	42.6982801	5.66845703
59.78	1	8	50.3862416	4.93212891
11.47	4	2	202.524174	6.25244141
18.17	5	1	64.9941575	7.37158203
35.71	2	9	39.3272815	8.08935547
19.1	2	48	295.932224	12.0151367
15.32	3	7	101.454088	5.10986328
18.56	1	1	76.0140834	7.45947266
19.16	1	1	70.9601196	5.45263672
19.81	3	5	72.7232305	7.45947266
4.62	3	10	210.870941	5.50341797
18.04	3	8	64.2511242	4.97021484
18.92	2	2	35.6690376	7.69384766
6.99	2	1	30.2983633	6.18896484
31.05	7	9	30.0570987	8.35302734
36.07	7	3	41.9918826	5.38916016
8.29	1	4	67.2126129	4.62744141
13.17	1	4	90.8540051	5.08447266
14.81	2	15	191.437516	5.69384766
9.33	1	3	69.0232778	5.80810547
52.58	2	15	43.1354136	5.52880859
25.15	2	2	18.7407794	5.59228516
15.25	7	26	284.462285	5.32568359
12.93	1	3	88.8995214	5.77001953
29.85	10	7	53.049645	6.17626953
21.47	7	4	62.0657214	6.74169922
26.22	3	11	59.7166172	9.18798828
11.11	1	6	54.4600769	6.15087891
21.1	7	9	50.5479151	8.57275391
25.1	1	12	84.7617799	5.00830078
17.91	1	3	108.482035	4.86865234

Information Classification: General

4.57	2	1	89.261858	5.46533203
7.66	1	2	23.3798764	7.86962891
32.39	1	2	15.480816	4.33544922
13.64	2	3	57.9109736	4.81787109
40.84	18	5	35.7600878	8.25048828
10.27	1	3	67.8064397	5.33837891
15.64	1	7	78.8726817	5.61767578
14.15	1	6	82.6079467	5.56689453
26.82	1	20	129.664648	9.36376953
5.68	3	7	232.523444	6.35400391
15.84	3	1	60.877173	6.60986328
13.1	1	4	123.468904	6.78564453
6.81	11	5	123.845403	6.63916016
63.79	2	16	27.0350294	5.04638672
3.59	5	10	619.216175	5.40185547
14.91	3	5	42.7966942	6.66845703
15.76	2	2	18.5215405	8.03076172
10.63	1	8	135.581183	7.15185547
46.31	3	7	32.9682379	9.78857422
13.75	2	11	131.276848	6.72705078
31.94	2	11	43.0039147	8.76318359
24.22	1	10	46.5004725	9.37841797
9.57	2	5	96.3871198	5.07177734
11.1	1	6	91.5886678	7.18115234
14.03	2	5	55.4693045	9.31982422
4.95	2	7	226.27257	5.65576172
4.48	5	1	214.075194	6.49365234
9.89	13	7	87.7533963	5.71923828
66.21	1	1	21.706388	4.51318359
12.38	1	10	179.903563	4.79248047
14.78	1	1	11.6848589	4.53857422
10.08	2	5	103.696556	6.50732422
2.82	3	7	531.472481	6.37939453
10.42	1	2	29.1694867	4.80517578
9.35	1	4	31.4147274	5.30029297
11.08	1	5	80.2040402	5.47802734
7.54	3	1	22.9334773	6.01123047
4.65	1	2	104.402812	6.17626953
13.34	1	6	77.7576382	6.82958984
32.93	5	3	28.1940254	4.89404297
61.81	2	8	19.8163438	4.69091797
16.28	2	1	23.3697129	4.56396484
11.11	1	2	33.4006796	7.97216797
10.38	2	5	51.2888279	7.04931641
7.46	1	3	95.7182874	5.00830078
13.83	1	10	108.0133	7.67919922
6.52	1	4	134.899213	5.96044922
23.17	2	12	82.9505521	4.61474609
32.66	1	15	67.5258645	6.96142578
3.04	3	2	131.886641	6.71240234
8.62	2	3	74.394256	6.30322266
3.8	1	4	211.273998	7.26904297
9.07	1	3	38.1942184	7.81103516
30.38	3	8	26.4716276	4.80517578
34.69	1	3	16.6866677	8.01611328

30.94	2	9	35.668096	6.55126953
10.48	1	2	60.5397184	5.85888672
30.07	1	2	102.371965	11.8540039
5.6	4	3	58.5289827	8.85107422
37.12	1	4	25.5651602	5.92236328
11.06	1	1	78.7547242	5.66845703
21.28	4	7	57.7808796	7.06396484
12.46	5	9	72.1361716	6.58056641
18.23	5	4	58.6062801	5.85888672
6.57	1	3	111.793593	5.73193359
25.64	1	4	39.4676391	5.42724609
11.02	2	3	43.0680195	5.35107422
9.67	1	5	106.873551	8.20654297
5.2	10	4	277.291216	7.38623047
8.33	2	2	27.5045221	9.23193359
15.61	2	3	38.3011122	5.75732422
11.73	1	1	52.8731539	5.49072266
4.36	2	6	182.333075	6.34130859
5.22	3	4	112.870754	5.61767578
9.14	1	2	65.4374145	7.76708984
2.41	4	3	202.940089	8.76318359
21.88	3	4	46.0939371	4.98291016
45.71	4	5	27.7607836	4.77978516
15.65	2	4	53.0159396	9.96435547
77.85	3	5	16.8268352	4.22119141
23.34	2	6	61.7096582	5.59228516
7.27	2	3	56.6350928	6.29052734
13.43	3	2	74.916994	6.34130859
26.21	2	3	15.7868521	5.71923828
28.98	1	8	73.9425972	9.80322266
12.28	2	8	93.2004622	8.73388672
12.11	1	2	61.9280389	6.49365234
16.17	5	6	68.1344393	5.22412109
20.99	2	8	63.3727181	8.42626953
19.17	5	3	59.0010604	7.70849609
18.41	2	3	51.8040594	4.46240234
11	1	3	97.2338844	6.87353516
4.95	2	1	21.1133128	6.30322266
21.14	4	7	26.4336224	6.52197266
14.65	5	1	40.4730595	5.45263672
7.43	5	10	267.682735	8.90966797
9.83	3	8	125.658902	6.88818359
14.88	4	15	106.000929	9.99365234
10.3	1	10	159.131607	8.92431641
27.96	1	1	102.846245	11.8979492
12.04	2	5	92.5962648	4.88134766
6.31	1	1	34.1937939	6.25244141
32.55	2	2	32.8801477	9.72998047
19.59	1	5	51.9366795	4.98291016
6.76	2	1	22.0993145	7.91357422
31.07	1	8	41.9009924	4.90673828
17.46	1	9	78.5383901	4.44970703
68.57	5	2	4.03711248	9.31982422
4.66	1	3	133.010108	6.56591797
3.44	13	9	498.926524	5.65576172

Information Classification: General

33.66	4	5	44.2747529	7.97216797
8.05	1	3	95.9124873	6.48095703
4.68	1	2	79.3615756	5.89697266
37.75	4	6	26.8317513	7.23974609
15.3	1	3	31.3108729	5.05908203
42.79	21	18	49.9265935	5.43994141
17.07	4	3	60.0425522	7.26904297
18.28	3	1	9.9089351	4.18310547
9.26	1	1	71.9965193	5.35107422
22.05	2	3	21.5846944	4.60205078
40.22	1	4	10.7370448	4.55126953
4.36	2	7	181.023877	6.21435547
16.4	3	6	58.5884317	5.73193359
4.23	2	6	201.010862	6.43017578
8.77	1	9	212.728161	4.64013672
4.23	1	1	32.7655111	6.85888672
27.55	4	4	39.2591938	7.12255859
5.49	2	1	28.4011035	5.43994141
14.43	2	1	77.4242136	4.50048828
7.93	1	2	61.837278	8.45556641
12.24	1	7	122.660665	5.13525391
3.4	1	2	54.6866778	6.58056641
12.91	2	6	95.94113	4.94482422
14.29	1	2	52.7558609	5.14794922
6.78	2	4	83.3154097	6.94677734
9.94	1	2	20.5925159	7.38623047
19.49	3	2	40.3192366	5.97314453
17.32	2	2	47.6510992	5.75732422
12.24	2	9	150.173768	9.14404297
3.86	1	2	51.5055431	8.92431641
44.37	1	5	15.9219786	5.40185547
12.03	4	3	31.9898324	4.91943359
30.62	5	7	46.9322116	8.32373047
11.66	1	3	45.4536799	9.27587891
16.75	3	6	66.622137	4.74169922
23.71	11	7	70.3859685	5.59228516
1.28	1	1	163.085973	7.02001953
7.86	3	1	46.6809356	8.77783203
12.92	1	5	79.6023636	8.01611328
8.7	1	2	38.3424069	5.23681641
11	3	6	64.5172004	6.40478516
9.54	1	6	121.938762	6.59521484
3.81	2	5	270.848974	5.83349609
12.43	6	11	161.225072	9.64208984
3.98	3	3	149.425662	4.91943359
9.8	1	5	84.8068849	4.90673828
8.63	1	3	42.9269122	6.18896484
5.17	2	4	100.497981	7.88427734
7.49	1	5	107.025978	9.02685547
2.33	1	6	531.206643	6.03662109
14.31	1	1	77.2961551	4.50048828
19.17	2	8	41.2714471	4.91943359
3.42	2	4	135.295928	6.50732422
8.74	1	11	181.851906	7.94287109
22.36	3	9	37.343047	9.17333984

8.95	1	1	28.2352804	6.26513672
1.98	3	1	139.753959	5.69384766
17.76	1	2	11.6991897	10.315918
11.68	2	3	40.9340926	6.62451172
2.67	1	6	452.218347	6.17626953
11.92	2	6	95.1930751	6.65380859
6.64	2	1	30.2202386	6.45556641
37.69	1	4	15.030788	5.02099609
15.05	5	5	92.6075273	5.12255859
10.21	5	8	117.712981	5.56689453
12.76	6	1	59.1080987	7.75244141
10.74	2	2	34.1737934	6.13818359
5.98	2	5	154.594045	5.08447266
6.26	1	3	105.368643	5.78271484
4	1	3	113.596872	6.17626953
9.63	2	8	150.948152	5.85888672
5.82	1	2	95.579211	6.48095703
5.55	1	2	63.5532054	7.16650391
20.14	4	6	51.2199125	6.08740234
4.78	2	1	35.3241807	8.00146484
5.91	1	6	117.316853	6.78564453
8.25	1	2	57.7368262	4.94482422
5.64	2	5	105.640573	8.67529297
2.46	1	2	201.845044	6.50732422
8.71	1	3	85.9881567	6.23974609
17.14	1	2	23.4240694	7.35693359
28.57	1	2	50.4175619	5.23681641
5.54	1	5	150.906198	7.09326172
4.26	1	2	97.8529435	8.35302734
13.81	2	8	80.4157626	5.88427734
3.42	6	6	236.460998	7.25439453
5.68	2	3	102.370109	7.10791016
30.39	1	6	30.7365739	8.54345703
8.49	1	7	126.760564	5.19873047
10.54	1	3	56.4902464	8.68994141
2.09	1	1	70.1892832	6.49365234
39.32	4	5	23.3002077	4.76708984
18.36	1	7	62.1565993	7.12255859
5.17	2	1	32.8834585	7.12255859
12.06	2	2	16.3584993	7.34228516
6.89	2	8	166.284219	6.60986328
11.79	2	10	110.178906	7.16650391
23.15	2	3	35.1533831	5.68115234
3.01	2	1	57.6162189	6.97607422
21.11	3	4	20.9247904	7.48876953
5.56	3	7	310.3163	7.37158203
5.48	4	4	136.328515	5.51611328
9.82	3	4	87.057306	5.32568359
5.78	2	2	78.6404447	8.85107422
32.73	2	3	18.794711	4.65283203
8	1	2	42.3415581	9.71533203
5.54	1	2	41.5663377	6.59521484
15.13	1	2	31.250403	4.94482422
8.87	2	2	59.4987783	5.71923828
9.89	1	7	133.44247	7.47412109

Information Classification: General

16.64	3	6	65.2809321	5.10986328
2.41	2	4	228.869961	8.19189453
25.95	2	3	19.867337	9.55419922
2.4	1	2	119.067943	5.63037109
16.04	1	1	12.1094225	6.02392578
20.51	2	6	31.0327931	4.66552734
17.33	1	4	24.6602838	4.71630859
11.89	2	3	45.9450568	5.13525391
9.68	1	4	39.3429438	7.19580078
8.23	3	2	45.4516904	4.93212891
4.7	3	3	114.693652	5.33837891
5.18	3	4	113.015736	7.18115234
10.11	4	3	73.0468792	5.09716797
32.28	2	2	17.5930795	5.59228516
1.97	3	3	204.155136	5.98583984
13.14	1	11	171.59092	6.72705078
8	1	5	125.197122	4.98291016
22.95	4	3	20.4912087	4.80517578
3.96	3	5	151.514483	5.93505859
15.07	2	1	76.5220282	6.90283203
12.54	1	2	40.6820336	8.55810547
4.87	2	4	85.4162008	5.85888672
3.98	1	1	58.1680844	6.56591797
5.66	3	4	59.4546678	5.40185547
35.23	1	1	9.98903287	6.12548828
3.28	2	6	288.639414	5.99853516
12.71	1	1	19.9490837	4.84326172
8.04	1	5	55.5857553	6.23974609
7.81	1	3	52.7832948	6.39208984
2.88	1	6	447.377807	5.40185547
2	2	8	502.037363	6.62451172
7.79	2	3	99.2633247	7.43017578
27.47	21	9	53.7000331	5.12255859
14.3	2	6	104.486047	5.37646484
13.86	1	3	46.9606933	7.12255859
11.42	1	5	56.664473	4.85595703
2.87	6	4	262.088601	8.55810547
7.77	1	1	20.5997514	8.57275391
6.83	2	5	100.377282	9.37841797
6.22	2	5	129.670167	5.79541016
9.17	1	2	52.815405	5.88427734
7.61	1	3	58.2561419	8.30908203
3.99	2	2	128.349368	5.55419922
4.6	3	4	170.372422	6.58056641
8.68	1	6	127.578727	7.12255859
5.15	1	1	39.5624785	6.04931641
1.64	2	2	270.774071	6.50732422
37.81	2	8	50.5041572	5.13525391
9.06	2	9	193.912083	6.58056641
9.89	2	1	31.2724294	6.93212891
32.04	1	4	11.3603823	11.355957
2.81	1	3	125.71042	7.03466797
7.28	2	5	112.784451	8.58740234
3.9	1	1	38.2192975	5.79541016
12.44	1	5	74.7834817	6.60986328

Information Classification: General

5.65	3	2	133.821475	6.26513672
9.48	2	2	91.372465	4.95751953
2.19	2	2	164.593033	8.03076172
9.72	3	3	50.9444052	5.54150391
21.46	2	2	22.8988512	4.60205078
5.41	6	9	338.15889	6.07470703
6.5	2	4	82.2182876	5.78271484
11.18	1	9	131.382897	8.92431641
22.16	1	6	36.4600499	6.58056641
12.38	1	2	56.9156877	4.93212891
6.56	1	2	20.7983504	5.32568359
19.93	4	3	91.5786541	4.90673828
12.02	1	2	51.4958276	4.97021484
11.66	1	6	134.566288	5.30029297
6.09	2	2	26.0930172	5.10986328
4.09	3	1	61.3645738	6.75634766
25.89	3	4	12.3751483	3.84033203
3.06	3	4	228.593015	5.03369141
3.57	2	2	78.8373055	6.63916016
11.49	3	3	51.5132921	6.34130859
8.85	1	2	136.724366	5.97314453
6.12	2	7	114.819604	4.89404297
4.03	2	3	80.7099257	7.81103516
10.51	5	3	68.4827941	7.82568359
0.47	1	3	846.682783	5.33837891
16.42	5	1	15.3692824	9.77392578
20.41	1	4	49.3501528	10.2719727
6.81	1	3	62.787126	7.76708984
11.21	1	3	23.0338512	5.56689453
5.1	2	2	61.6475946	5.87158203
0.68	2	1	343.543724	6.32861328
6	2	2	83.112899	4.97021484
9.04	2	5	131.316012	6.53662109
7.51	2	6	93.9081172	9.27587891
7.97	3	2	43.3563383	9.74462891
17.81	1	6	53.203409	8.32373047
3.37	1	2	149.397358	6.87353516
3.39	2	1	113.962973	5.88427734
4.46	3	3	170.922723	8.41162109
10.23	3	4	86.6759937	4.64013672
4.26	1	2	101.520819	6.59521484
3.66	2	3	101.363965	8.44091797
5.41	1	2	40.2789567	8.25048828
10.67	1	5	63.930746	9.29052734
5.2	3	5	172.77506	5.09716797
16.51	1	11	123.137555	7.67919922
10.09	2	2	79.6395557	6.68310547
3.31	2	3	165.923069	6.87353516
12.88	1	7	101.364935	7.73779297
12.85	1	6	85.1297834	9.31982422
7.56	2	3	86.0877567	5.21142578
12.62	1	13	108.187799	10.1547852
3.71	1	1	47.9595877	5.42724609
13.86	4	8	145.061508	4.76708984
3.45	1	1	71.0140659	7.50341797

Information Classification: General

23.56	6	1	19.6737627	5.02099609
8.52	1	3	91.2921376	6.80029297
7.51	1	3	94.4280803	6.63916016
7.43	1	2	93.4603196	4.93212891
10.94	2	2	121.265569	6.85888672
15.82	1	4	51.5564794	7.51806641
22.67	1	2	8.29916111	7.78173828
18.69	3	3	21.7696691	7.48876953
18.7	1	6	73.0247791	4.67822266
5.24	2	2	42.0313962	5.84619141
8	2	5	85.6395413	7.84033203
3.31	1	1	43.0763538	9.04150391
16.67	2	1	15.3126081	4.80517578
44.39	1	4	20.7883917	5.79541016
1.87	1	1	59.1458807	9.04150391
42.68	3	7	17.8628069	8.16259766
2.73	1	2	94.2639418	7.00537109
4.62	2	2	80.2792101	8.00146484
7.14	2	7	66.8315783	5.35107422
22.06	2	4	30.6093981	5.99853516
6.52	1	2	138.631898	5.99853516
20.79	3	3	50.7649964	8.92431641
3.35	1	1	39.8672108	5.36376953
12.54	2	1	40.5799945	8.79248047
4.47	1	1	32.7809898	7.44482422
8.83	5	4	94.5855069	5.37646484
3.4	1	1	52.2799908	5.02099609
29.82	6	6	36.4273467	8.26513672
18.56	3	2	21.5701731	7.89892578
5.15	2	1	82.3147943	9.31982422
6.77	2	2	52.2602468	5.55419922
3.2	2	2	86.7767846	5.85888672
2.64	1	6	353.948247	8.27978516
2.38	4	4	227.156059	5.49072266
3.28	1	2	55.169464	6.27783203
0.75	2	1	244.379615	6.06201172
15.82	1	2	18.7206046	5.73193359
43.13	4	4	18.1961171	4.91943359
7.54	2	1	80.9534957	8.73388672
21.34	4	6	44.5099887	7.89892578
4.18	4	4	142.576976	7.02001953
3.04	5	2	127.511232	7.13720703
8.29	1	6	117.802078	6.91748047
2.63	1	1	80.2268587	5.41455078
3.26	3	4	140.846402	5.85888672
1.98	1	2	134.228025	6.59521484
27.86	2	2	16.4423922	5.43994141
1.11	2	2	293.847165	6.13818359
6.67	1	4	118.767667	7.95751953
9.61	3	4	103.209813	5.45263672
4.96	5	3	70.2731243	7.12255859
43.38	7	1	15.3485141	11.2680664
33.05	1	5	40.0428729	5.52880859
9.01	1	2	22.6354897	10.9604492
8.88	1	4	87.3662822	8.04541016

11.11	2	1	25.3912816	5.42724609
4.4	6	3	185.175618	6.25244141
25.34	3	2	32.5398147	4.76708984
2.01	2	2	136.869163	6.40478516
16.8	2	4	41.1389773	7.72314453
5.83	2	2	36.8861521	9.49560547
5.52	3	2	71.2485058	5.79541016
8.81	3	7	129.263548	5.97314453
4.73	3	6	194.076239	7.84033203
10	5	10	143.27868	8.22119141
1.8	1	3	272.477891	6.39208984
5.4	2	3	113.606546	6.26513672
5.17	2	5	154.175422	7.09326172
31.11	7	2	20.459636	6.80029297
8.51	2	4	55.9907899	7.19580078
21	1	2	22.0077073	5.89697266
37.95	3	5	18.5206692	8.08935547
20.6	1	2	22.3228577	5.51611328
18.96	1	5	68.2215611	5.57958984
6.6	3	5	115.461671	6.45556641
5.11	3	2	38.3225986	7.45947266
8.49	3	8	141.881662	8.85107422
13.11	3	5	104.68627	7.54736328
1.81	2	1	84.6741033	8.36767578
6.14	2	2	79.8688162	4.95751953
16.78	3	7	64.2455211	4.36083984
3.97	3	3	119.259361	5.97314453
6.17	1	1	131.441831	6.31591797
3.76	18	3	166.773902	6.60986328
2.59	1	1	63.4916392	8.23583984
5.86	1	4	49.4205135	7.10791016
4.04	1	1	59.620169	8.00146484
1.82	1	1	93.3755563	9.01220703
7.81	1	1	35.6289169	5.89697266
9.25	2	4	20.3761267	5.49072266
15.82	3	3	37.978903	6.74169922
7.86	1	4	103.523066	8.16259766
4.95	1	1	45.1915247	6.66845703
2.94	2	4	289.255239	5.78271484
7.88	3	8	195.850535	8.14794922
13.24	1	1	21.9740383	11.1069336
2.45	2	3	267.858905	5.45263672
29.89	3	3	19.7693796	4.77978516
5.32	3	6	198.478471	7.13720703
14.89	3	2	39.5054113	9.26123047
10.57	2	3	56.5148504	5.80810547
6.89	2	2	48.9085148	9.62744141
7.87	1	3	83.3864923	4.89404297
4	2	1	60.1206579	4.80517578
1.18	2	1	183.840919	7.25439453
8.92	1	2	34.1358591	5.12255859
4.11	2	1	49.6057906	4.66552734
13.82	1	3	33.8240366	5.55419922
33.56	1	1	17.3408321	4.58935547
0.85	1	1	265.024835	6.39208984

Information Classification: General

3.9	3	1	76.6133986	6.02392578
6.36	4	5	142.289212	8.32373047
5.52	3	1	103.757795	5.17333984
5.65	1	5	179.743331	5.17333984
3.44	1	2	83.8561359	7.07861328
8.29	2	3	63.3048755	9.43701172
4.83	2	3	151.434268	4.99560547
6.76	2	4	71.8504655	8.98291016
15.92	2	4	36.3235747	8.44091797
7.71	2	2	76.1143491	5.13525391
10.91	1	2	24.9540761	5.03369141
5.82	2	1	28.8660119	6.69775391
8.11	1	5	76.9116248	4.43701172
23.5	1	4	23.3886638	5.26220703
2.28	2	1	62.7929222	5.18603516
9.49	1	4	81.0023533	6.41748047
3.93	1	5	192.813116	6.20166016
10.41	1	1	33.9422203	6.63916016
6.14	1	3	115.943818	5.43994141
19.72	5	3	25.6449641	7.32763672
20.27	2	4	31.6726197	7.63525391
5.57	1	3	58.1462667	9.43701172
15.96	4	1	10.7852694	4.41162109
6.2	7	4	105.558628	5.38916016
17.19	1	6	77.489024	5.03369141
8.5	2	4	85.6654483	7.28369141
4.48	1	1	39.165537	5.28759766
4.52	1	1	55.3356534	5.22412109
7.16	2	2	57.9806672	7.07861328
3.27	3	2	90.2031968	6.63916016
18.81	1	4	41.0035175	7.35693359
7.1	2	3	97.4526031	4.60205078
5.51	2	3	115.461517	5.52880859
9	1	3	54.2706845	8.07470703
2.15	2	1	143.624931	5.73193359
10.96	1	4	38.8065739	5.99853516
1.04	1	2	315.146034	7.69384766
11.09	1	1	49.5175583	5.18603516
4.49	1	4	60.0326184	8.51416016
10.49	1	2	32.9466167	5.84619141
12.25	1	1	23.2610589	7.63525391
33.61	1	4	25.535882	7.81103516
16.33	2	4	27.8637781	8.35302734
2.83	1	3	171.124365	7.06396484
6.19	1	1	72.749597	5.22412109
12.98	1	3	23.6231915	4.55126953
9.41	5	4	82.5909704	6.94677734
6.58	2	4	145.528065	5.45263672
32.35	1	8	19.091073	11.0336914
13.68	1	1	21.3037423	11.0043945
8.7	5	1	20.567338	5.28759766
24.71	2	2	9.2743398	4.20849609
6.86	1	1	29.9082532	7.97216797
2.49	2	2	177.272928	5.22412109
16.77	1	5	52.4241902	9.45166016

3.73	4	3	120.465669	7.34228516
10.42	1	2	55.6998037	5.89697266
3.02	1	2	47.6181642	8.42626953
4.76	1	5	124.661474	5.43994141
32.77	1	2	11.790947	4.97021484
10.48	1	2	49.1294167	5.23681641
3.1	1	2	141.571397	6.06201172
7.65	1	2	37.5170447	4.61474609
3.9	2	1	45.087471	7.40087891
46.64	1	5	24.8224448	5.23681641
2.55	5	2	160.490421	8.70458984
4.59	2	1	30.6026925	4.98291016
12.96	1	7	105.123534	7.23974609
1.25	3	5	573.511393	6.02392578
10.28	1	11	216.243458	7.92822266
3.44	1	1	49.6423962	7.35693359
6.97	1	3	75.7874198	6.41748047
4.59	2	5	141.518925	5.50341797
19.95	2	5	43.8894527	4.67822266
14.02	1	1	12.125745	4.20849609
36.22	5	2	22.1091272	5.23681641
4.31	2	4	134.749964	8.01611328
13.94	1	1	18.0417466	8.38232422
5.45	4	3	68.5594691	5.87158203
24.92	15	1	35.9922783	8.03076172
6.56	1	4	145.900642	6.93212891
2.64	2	3	132.530327	7.38623047
6.63	1	1	40.7052264	4.79248047
12.7	1	8	68.5691336	7.03466797
5.77	2	1	27.4558388	5.59228516
4.71	3	6	151.339583	8.98291016
7.28	4	2	88.6661316	9.40771484
3.97	2	1	52.2155647	5.79541016
2.14	1	1	72.1074486	6.90283203
3.61	1	2	105.095023	5.38916016
3.54	2	2	56.5427356	9.34912109
13.55	1	5	44.2770749	8.03076172
15.76	1	3	34.7071582	7.76708984
3.18	3	1	53.3941133	6.84423828
12.71	13	1	13.1260351	10.3891602
10.75	3	4	42.3705767	8.01611328
1.52	2	1	119.640503	6.68310547
3.8	4	6	279.187088	6.90283203
19.09	2	5	37.3279844	9.89111328
3.88	2	1	80.8214864	9.61279297
4.94	2	1	75.3535699	9.78857422
4.32	2	2	82.4448009	7.22509766
6	1	3	56.4194277	7.92822266
4.38	2	1	44.8263214	5.07177734
15.23	1	6	61.3772755	8.00146484
1.89	2	1	136.807742	5.69384766
2.75	1	3	212.90293	5.93505859
14.78	1	1	11.6988745	4.53857422
6.57	2	4	86.67474	6.77099609
1.49	1	2	115.710091	6.32861328

5.3	3	4	145.916103	5.19873047
8.71	2	3	55.8626158	5.96044922
14.81	2	1	12.4292477	4.65283203
0.63	1	2	521.150069	7.09326172
2.49	2	3	123.986861	7.00537109
32.79	10	2	34.3475001	7.53271484
7.49	2	3	59.2630774	9.27587891
5.05	1	1	56.3434355	5.13525391
3.02	1	3	133.775242	9.31982422
14.69	1	7	90.3639556	9.97900391
4.6	1	3	133.215265	6.01123047
3.65	1	2	70.5034333	8.07470703
5.75	1	2	73.2260057	7.25439453
2.13	2	1	127.722761	6.60986328
5.61	1	4	94.1613856	5.69384766
15.58	1	1	16.8214054	5.23681641
3.8	2	1	37.0520935	5.27490234
2	2	2	165.176181	5.96044922
1.01	1	1	155.768789	6.99072266
3.69	1	2	46.2461866	5.02099609
3.89	2	1	30.764332	8.58740234
12.81	1	2	40.3108996	5.77001953
14.66	2	2	13.3106735	9.64208984
5.36	1	1	41.4444981	4.77978516
6.09	2	2	95.2857543	7.59130859
2.87	2	1	50.5193701	6.27783203
7.74	3	3	77.2216364	7.72314453
2.22	1	2	94.1016589	7.94287109
5.17	4	3	136.451081	5.52880859
5.32	2	5	182.449807	7.28369141
5.98	2	4	103.278814	5.77001953
11.97	2	7	94.8752823	6.23974609
5.65	1	1	57.4528187	8.66064453
20.13	1	2	15.565589	5.12255859
3.1	5	3	124.595738	6.50732422
4.39	2	2	60.4873554	7.47412109
10.62	1	3	50.2470831	9.39306641
6.01	1	2	53.3295404	6.80029297
7.03	4	2	62.2772807	7.44482422
3	1	3	117.097654	8.01611328
8.41	4	5	149.831858	8.04541016
8.82	2	3	102.342113	6.71240234
10.48	2	6	107.95039	7.10791016
26.53	1	1	50.3435217	5.22412109
12.75	1	3	47.0689769	5.98583984
12.78	3	4	81.3864138	5.36376953
1.14	4	2	271.514302	5.55419922
6.29	1	3	120.889918	5.27490234
7.97	7	6	74.2667383	6.97607422
14.57	2	3	22.4861689	7.32763672
1.22	1	1	128.699298	6.22705078
3.66	1	1	51.7548442	5.59228516
25.51	1	1	11.1909216	6.12548828
3.07	1	1	80.9922282	5.40185547
11.71	1	3	22.2148269	8.52880859

Information Classification: General

6	1	1	23.2741167	5.31298828
6.49	2	3	99.6464726	5.46533203
13.39	2	3	47.6429268	5.12255859
11.88	2	4	67.6005697	7.48876953
16.34	2	2	27.217264	5.84619141
6.7	5	5	110.070542	6.93212891
5.22	2	2	119.064326	4.32275391
2.63	1	1	70.0242664	7.69384766
5.82	2	3	137.556155	5.02099609
1.1	1	1	137.397232	4.83056641
3.49	2	1	91.0703174	6.60986328
2.34	1	2	115.77276	6.53662109
5.02	1	2	46.9902484	6.27783203
19.12	2	6	37.2268697	5.51611328
4.77	2	2	64.405402	5.89697266
38.93	1	3	15.0338689	8.96826172
14.41	2	1	13.7336095	6.90283203
49.29	1	3	14.5061966	4.76708984
1.99	1	1	83.9117967	7.18115234
3.27	3	4	226.771489	5.64306641
53.85	3	1	4.40118626	7.38623047
13.48	2	3	25.58573	4.90673828
8.84	3	4	96.6588657	9.65673828
32.03	2	2	14.7797477	9.02685547
1.96	1	1	85.930008	5.83349609
5.21	1	1	39.178543	8.19189453
21.67	2	1	20.3836147	7.13720703
6	2	5	175.326191	5.78271484
4.97	2	1	35.2722549	9.07080078
60.71	2	1	5.9099708	5.18603516
9.26	4	6	103.965271	6.97607422
7.03	2	2	73.4092409	5.28759766
13.58	1	3	32.1139504	6.45556641
1.56	2	4	487.545489	6.52197266
3.41	1	2	131.647113	6.35400391
1.67	3	1	85.6977937	7.88427734
7.97	3	1	42.9692023	5.55419922
4.12	2	2	108.987834	7.02001953
6.53	2	3	93.0043617	6.53662109
2.9	2	1	50.3643128	8.60205078
1.96	2	1	93.9709988	8.26513672
24.72	1	1	10.3591084	7.40087891
4.55	4	9	225.012916	6.30322266
2.4	4	2	169.553648	9.46630859
6.05	1	1	44.8394547	7.07861328
1.21	2	1	111.20568	5.24951172
16.18	2	3	31.042046	3.99267578
15.87	2	4	45.1396971	9.27587891
4.02	2	2	96.9027794	4.83056641
2.65	2	1	55.8186575	6.11279297
7.12	1	2	45.2158655	4.33544922
15.87	2	1	13.7671517	7.59130859
15.08	3	4	65.4559542	6.62451172
3.56	1	1	65.7134827	9.14404297
0.78	2	1	231.553136	6.99072266

Information Classification: General

21.48	1	1	16.7918205	4.31005859
7.53	3	2	38.3197507	6.81494141
25.73	2	3	26.5750845	4.11962891
17.41	3	2	31.8413789	4.43701172
3.52	1	1	44.078754	5.09716797
41.67	1	2	7.98007957	8.96826172
13.33	1	2	37.8047097	5.19873047
27.93	1	3	20.7428989	8.63134766
15.45	1	2	23.9770772	6.15087891
5.62	2	3	90.2292351	8.00146484
2.81	1	1	88.0344833	7.03466797
27.52	4	5	32.8370537	4.99560547
2.72	3	2	108.747156	8.55810547
3.3	1	1	61.0806209	7.19580078
18.36	4	4	44.3349578	5.55419922
1.16	1	1	128.02353	6.78564453
27.78	17	1	13.63662	10.8579102
4.04	2	1	92.4289855	8.92431641
0.93	2	3	571.489966	6.22705078
17.46	2	2	19.961428	6.77099609
4.58	2	2	130.005144	6.75634766
2.37	1	1	93.8922238	8.23583984
11	4	2	47.3269679	5.88427734
3.91	1	1	93.243416	5.71923828
40.55	3	6	28.8138678	7.18115234
33.74	2	3	18.6602614	4.53857422
2.06	1	2	74.1391775	7.21044922
35.09	1	1	6.19273946	3.82763672
2.8	1	1	50.9776288	5.05908203
8.82	1	4	72.4430677	8.33837891
2.67	1	1	53.3644881	5.31298828
6.13	1	3	86.1421047	6.07470703
5.22	2	1	27.4586058	9.83251953
7.97	1	4	52.8791188	7.16650391
9.33	3	1	67.0294991	8.51416016
9.14	3	2	55.5137135	7.03466797
4.5	2	1	49.0256754	6.40478516
1.55	1	2	332.162825	5.94775391
13.7	1	2	30.3753081	6.22705078
12.25	4	3	68.3206934	5.28759766
6.24	1	2	102.004506	7.03466797
1.08	4	2	391.068719	5.28759766
4.8	1	2	55.7753337	4.85595703
2.92	2	2	175.722808	7.00537109
3.11	3	1	74.2906786	5.10986328
3.92	2	2	83.3818205	5.04638672
15.16	2	2	41.9359016	5.49072266
1.76	2	1	111.877196	6.46826172
9.77	1	1	38.7974294	4.90673828
3.58	1	3	147.739527	7.21044922
0.96	1	2	418.359488	6.84423828
19.84	2	4	54.0720597	5.54150391
1.97	6	1	109.113308	5.33837891
9.32	2	2	57.120469	6.30322266
3.64	2	1	117.630612	7.21044922

Information Classification: General

4.97	3	2	102.370577	5.26220703
10.32	1	3	44.7273333	8.54345703
5.47	3	2	80.696674	8.67529297
1.1	1	1	154.222073	7.28369141
3.11	3	2	87.4654704	8.22119141
4.81	3	4	48.8760582	7.79638672
8.47	2	2	39.5880441	6.91748047
17.57	5	4	40.4704034	7.69384766
9.29	1	6	125.450635	7.88427734
14.58	2	1	16.8472095	4.79248047
7.96	2	6	156.95181	6.62451172
10.28	2	6	85.1942542	8.85107422
6.38	1	3	50.5912352	6.53662109
12.41	6	1	48.6092775	8.07470703
3.24	2	2	112.811732	5.87158203
1.3	2	1	200.229044	8.29443359
11.26	1	6	108.90556	5.50341797
16.4	1	3	35.9721758	5.16064453
7.56	1	2	52.5275888	6.99072266
4.33	2	3	83.5813993	7.43017578
12.11	1	4	47.3687412	8.41162109
3.36	2	1	51.6729013	7.06396484
4.03	2	1	63.3957027	7.82568359
2.04	2	2	118.356158	8.06005859
27.24	1	4	27.5695558	4.81787109
6.84	1	3	65.6558841	4.64013672
4.86	1	1	28.0371585	6.97607422
2.71	1	1	117.312551	9.26123047
12.86	2	1	22.9141123	5.79541016
5.96	1	1	44.8661107	4.95751953
8.96	4	5	113.042981	6.16357422
17.8	2	3	22.1279658	5.14794922
14.59	1	2	46.1808869	7.81103516
1.16	3	3	560.081276	6.12548828
12.6	1	5	42.2402998	7.07861328
2.98	2	1	84.2306573	5.02099609
13.03	1	3	52.7894636	8.26513672
7.21	3	2	35.7222988	5.04638672
14.46	1	2	18.9790946	6.34130859
10.77	2	9	104.642989	8.80712891
16.79	3	2	15.3896639	6.78564453
3.94	2	5	142.668381	6.77099609
8.58	2	3	40.9377285	9.39306641
8.18	1	1	30.4607436	8.58740234
1.27	2	2	341.188021	5.98583984
5.75	4	3	90.1548418	4.83056641
6.04	1	1	40.5373648	5.00830078
4.03	1	1	37.9418909	4.90673828
8.48	2	1	25.3817241	7.15185547
7.63	1	4	144.72704	9.40771484
10.61	2	2	40.6281222	7.19580078
11.01	2	4	50.86867	5.71923828
5.6	8	1	37.7684977	6.40478516
6.25	2	1	24.9871064	4.62744141
5.03	1	1	21.3559611	9.04150391

Information Classification: General

4.02	2	5	173.298248	7.16650391
6.51	1	1	24.7247513	8.51416016
28.08	2	2	15.8837052	4.19580078
4.69	1	2	58.1525555	5.73193359
4.06	1	1	62.1647596	7.82568359
13.18	1	1	22.2201599	11.1069336
7.48	4	3	71.8082602	8.70458984
7.45	1	2	17.709784	5.02099609
9.31	1	1	28.291321	9.45166016
9.26	2	4	67.8880297	7.57666016
6.58	2	1	25.5550653	4.93212891
1.31	1	3	343.660031	6.39208984
6.38	2	1	99.493136	7.13720703
19.14	1	2	23.2209616	5.19873047
6.75	2	3	123.625911	4.95751953
1.13	2	2	520.790765	6.25244141
15.48	3	1	9.55868117	4.93212891
1.33	1	2	238.026423	6.59521484
6.8	1	2	70.3386121	5.02099609
7.93	4	5	111.390794	4.95751953
6.16	1	2	23.6077619	7.19580078
6.23	3	1	30.9420273	5.27490234
9.8	3	1	26.9810795	8.00146484
5.22	3	2	60.8241723	4.60205078
2.16	1	1	53.6811677	8.48486328
8.97	2	1	25.9102773	7.43017578
7.7	1	2	71.962391	6.18896484
6.83	1	2	36.9562611	4.99560547
3.72	2	4	157.891475	6.78564453
10.79	1	3	55.0576794	5.47802734
3.64	3	2	36.2133317	6.32861328
4.13	1	3	96.0326885	7.12255859
2.85	2	1	49.88711	4.79248047
8.17	1	1	24.4501877	6.94677734
7.44	2	2	24.4132265	6.17626953
5.57	1	3	63.3201591	10.418457
16.18	4	3	37.3530148	5.99853516
3.92	1	1	29.0387653	4.88134766
20.64	1	3	35.7986286	4.62744141
11.06	1	1	25.1978729	7.26904297
2.51	1	2	63.3352978	6.10009766
12.77	1	1	86.8123124	5.65576172
1.6	1	1	102.480103	7.29833984
2.54	1	2	102.962926	6.11279297
0.86	1	3	498.598541	7.09326172
41.67	10	1	4.28105193	6.77099609
24.65	1	2	29.9730328	8.82177734
2.68	3	2	91.3904594	5.38916016
5.82	3	4	73.5206002	9.53955078
4.36	1	2	91.1113088	5.94775391
12.61	1	1	12.5683797	7.64990234
2.37	3	3	309.261171	5.74462891
7.59	1	1	24.8029819	5.93505859
9.16	1	3	57.4221337	6.45556641
15.65	3	2	32.5661793	9.20263672

Information Classification: General

1.21	1	1	114.843639	7.37158203
2.93	1	1	46.4496537	9.24658203
12.89	1	2	37.4282347	6.59521484
5.79	2	2	60.7527396	6.45556641
8.87	12	1	28.5394932	4.76708984
2.23	1	1	61.7559924	8.17724609
6.67	1	1	22.1622302	6.55126953
5.95	1	2	62.1156675	6.13818359
8.47	1	2	41.5761521	5.32568359
1.86	1	2	158.511413	6.07470703
8.61	1	3	37.4364512	7.03466797
2.02	2	1	83.8253529	7.98681641
9.2	1	1	30.1649973	8.41162109
17.35	2	6	66.7405648	5.87158203
3.92	3	1	98.8053713	6.93212891
3.54	1	2	65.2199985	9.70068359
2.34	1	2	99.7663286	6.68310547
23.02	3	2	15.4498785	3.99267578
1.71	1	1	58.0361086	5.32568359
2.32	1	1	61.625259	7.34228516
5.33	1	1	50.5121116	5.18603516
8.81	1	2	42.2891985	5.97314453
9.73	3	1	20.4552183	6.53662109
4.87	1	1	69.6132104	8.95361328
13.25	2	5	35.905485	4.44970703
5.49	2	2	45.9460487	7.16650391
4.07	1	1	68.3029213	8.98291016
2.94	1	1	66.7745414	7.35693359
4.12	2	5	88.2658448	10.2573242
3.97	1	3	140.041339	5.98583984
3.97	7	1	53.8697935	6.74169922
7.01	1	1	23.6538566	5.68115234
5.91	1	3	86.5955516	9.07080078
2.11	1	1	79.155076	6.97607422
5.81	2	2	26.3741726	4.85595703
5.54	2	2	46.1873058	5.63037109
1.48	1	1	122.474299	8.51416016
3.93	1	2	102.065104	6.08740234
8.47	1	2	27.3608832	6.68310547
8.17	1	1	47.2337915	7.51806641
4.76	2	1	30.1717183	8.83642578
11.05	5	4	43.0855934	8.19189453
4.98	1	3	33.8719603	8.38232422
1.3	2	2	157.199802	6.65380859
4.13	1	1	48.3645248	7.48876953
2.65	1	2	228.774734	5.55419922
1.88	3	2	144.116462	6.97607422
3.97	2	1	44.7644089	5.17333984
3.05	1	1	92.4647172	6.82958984
19.85	2	3	29.8018989	5.75732422
14.93	2	1	7.18276486	9.84716797
9	1	2	52.0882829	5.04638672
10.87	1	2	46.3665943	4.84326172
1.7	1	2	182.112618	5.43994141
7.25	1	3	57.9237989	5.77001953

4.92	1	1	67.2933922	6.39208984
3.86	1	4	223.788887	5.49072266
4.65	2	2	84.4868016	7.48876953
6.27	1	1	40.2752124	4.99560547
2.33	2	1	62.367971	6.32861328
1.96	1	1	98.9649861	7.06396484
23.39	2	1	14.4346438	8.63134766
1.52	2	1	101.147459	6.04931641
9.44	1	2	46.3995078	7.21044922
4.11	1	2	92.9012186	7.63525391
30	5	1	13.9907852	10.887207
9.07	1	2	41.1096499	5.94775391
4.58	1	1	49.742192	5.94775391
8.82	6	1	37.307061	5.99853516
22.22	1	3	6.37111495	8.16259766
18.55	1	1	13.3200785	6.15087891
1.36	1	1	221.883822	6.27783203
8.8	2	2	14.1953088	5.68115234
9.14	5	2	45.6349188	5.96044922
30.28	1	2	15.6629767	4.97021484
14.41	3	1	12.3233436	6.03662109
15.48	1	1	17.1477764	8.63134766
6.71	2	1	36.4949006	7.51806641
8.11	1	1	25.4934476	7.32763672
3.2	2	5	254.242372	5.07177734
3.96	1	4	218.046081	5.80810547
9.95	2	1	47.207235	7.98681641
3.94	1	1	55.5433746	8.01611328
10.51	2	2	39.4236411	4.83056641
11.6	2	2	42.0410552	6.60986328
7.14	2	3	71.6701162	5.64306641
29.66	1	2	13.1248404	6.53662109
2.95	2	1	69.1321241	6.03662109
2.62	2	1	64.2673028	6.32861328
13.92	1	10	88.5704965	4.38623047
7.15	1	3	113.791281	4.94482422
7.71	1	2	37.9036474	5.61767578
2.03	1	1	63.8938889	10.5649414
1.6	1	1	104.932186	5.46533203
2.05	3	2	158.191075	5.84619141
3.01	1	2	127.180543	6.97607422
1.76	3	1	138.362331	7.85498047
6.03	1	3	85.3799769	7.82568359
26.44	2	2	10.0211541	8.82177734
3.15	2	1	46.3548632	7.69384766
7.01	2	2	61.0204085	4.51318359
2.81	3	3	116.542281	6.08740234
14.29	1	1	13.3619884	5.82080078
4.66	3	1	44.6104223	4.85595703
7.02	4	5	92.3629663	6.91748047
8.43	1	1	27.7228333	8.20654297
12.68	3	1	8.18476202	8.85107422
7.46	1	2	48.9237081	5.51611328
4.86	1	2	123.73538	4.95751953
2.6	3	1	73.0662489	8.74853516

Information Classification: General

5.75	2	2	34.3635531	8.55810547
6.17	1	2	52.9408396	7.34228516
13.05	1	3	47.0581095	8.83642578
1.97	1	1	82.6125426	9.05615234
3.79	5	3	77.2499164	5.19873047
6.45	2	7	116.935452	10.2280273
5.91	2	1	47.6128582	6.03662109
2	1	1	71.6364736	6.55126953
0.85	1	1	265.633691	5.85888672
1.61	1	3	201.82205	5.80810547
1.69	3	3	312.585176	7.32763672
28.46	1	4	29.0955943	7.40087891
7.12	1	5	135.567981	9.20263672
6.44	1	1	33.1712497	5.45263672
6.64	2	5	144.688763	7.43017578
9.47	1	7	82.5604757	7.40087891
14.76	1	3	34.7510891	6.82958984
2.75	1	1	96.0913705	5.94775391
11.48	1	2	36.5375387	4.66552734
1.87	2	1	71.1611388	8.33837891
5.26	1	1	21.4893884	5.75732422
8.92	3	4	54.4569341	6.74169922
8.75	1	1	17.3184331	4.41162109
2.42	1	1	56.8239528	7.98681641
1.82	1	1	93.2096587	6.13818359
1.46	2	2	84.4703739	6.46826172
13.38	2	1	16.8907519	4.80517578
3.99	1	2	79.3912579	10.7260742
2.46	2	1	106.538442	5.71923828
4.76	1	1	137.039723	6.35400391
43.38	6	1	15.378507	11.2680664
1.89	2	4	94.4437646	6.25244141
3.56	2	1	62.2797536	7.59130859
6.68	3	1	40.7236759	5.47802734
5.86	1	1	36.7526557	6.06201172
6.26	3	2	49.1585494	5.38916016
7.32	4	2	37.3111875	6.85888672
12.33	1	1	24.5603134	4.79248047
2.1	1	1	86.4820282	5.46533203
4.81	4	1	35.7313329	5.27490234
14.25	1	3	39.8102104	9.61279297
8.84	2	3	42.8794784	5.79541016
5.81	1	2	54.4212863	4.90673828
6.43	1	1	37.2808389	9.43701172
1.94	1	3	214.525079	8.00146484
5.14	1	1	50.7690961	8.89501953
8.14	1	3	47.2841037	8.99755859
5.68	2	1	53.392677	6.60986328
4.02	1	1	40.4205918	6.91748047
7.07	1	1	22.4098186	4.52587891
3.7	3	1	51.8182558	6.97607422
2.62	2	3	234.655102	7.51806641
9.77	3	5	57.714035	8.23583984
4.72	4	1	34.7839233	6.97607422
2.56	1	1	65.5418411	5.79541016

Information Classification: General

3.9	2	1	48.1784587	6.93212891
2.96	1	1	63.0892927	5.16064453
1.03	3	1	130.436764	8.04541016
12.04	1	1	32.6099809	7.03466797
7.94	2	2	61.3538335	5.28759766
2.13	7	2	160.900931	8.96826172
15.79	3	2	15.6997225	6.35400391
16.26	2	2	36.7064841	4.79248047
2.58	1	2	56.74978	5.92236328
1.6	2	1	54.2623203	7.92822266
1.29	1	2	226.196133	8.33837891
5.14	3	3	87.6936441	6.23974609
4.03	1	2	53.0869877	9.08544922
6.25	1	1	49.6425198	6.16357422
7.33	2	2	66.6457198	5.16064453
1.68	1	1	108.577287	7.45947266
16.09	3	5	70.5100209	4.69091797
16.18	1	1	7.22476759	7.85498047
3.1	1	1	101.51723	6.45556641
3.51	2	3	134.055048	8.41162109
2.92	1	1	95.1138664	5.84619141
1.5	1	1	97.2479631	8.95361328
8.77	2	3	75.7064877	5.56689453
2.56	3	2	141.269952	5.08447266
1.78	4	1	116.085491	5.21142578
5.1	6	8	259.063651	7.70849609
3	1	3	104.007591	6.94677734
12.95	4	5	85.2885655	7.88427734
20.1	1	2	22.4835942	8.76318359
5.46	3	3	101.520943	7.07861328
23.14	2	2	13.3267297	7.23974609
16.28	1	1	10.064879	8.71923828
2.69	1	1	71.8875029	6.72705078
4.55	1	2	29.5782714	5.65576172
2.32	2	2	205.203641	5.78271484
3.11	1	1	65.0264285	7.98681641
4.07	1	2	84.0186047	6.17626953
6.61	2	3	129.699092	5.92236328
3.77	2	2	128.907326	6.21435547
2.71	1	1	103.724407	8.29443359
2.6	1	1	67.8334968	7.10791016
4.61	2	2	95.8221768	6.52197266
10.29	1	2	60.6727173	5.51611328
15.38	12	3	23.740086	4.72900391
11.19	1	2	31.7256287	7.48876953
31.48	4	2	17.6882071	7.50341797
5.74	1	2	81.8048514	9.26123047
8.14	2	3	65.4718987	8.82177734
3.64	2	3	87.1747873	6.68310547
4.91	3	1	45.9601568	7.72314453
3.7	2	4	228.907691	9.45166016
15.84	1	2	11.549706	5.51611328
6.51	1	1	53.771165	6.45556641
30.34	3	2	16.6626286	8.29443359
3.57	1	1	39.1001741	7.37158203

Information Classification: General

3.22	1	1	54.0151949	6.66845703
2.14	2	1	68.7141115	6.48095703
1.84	1	1	81.2845794	6.59521484
2.57	2	1	40.5924601	7.22509766
3.43	1	2	92.3808217	6.66845703
3.33	1	2	113.423061	6.11279297
5.97	1	1	54.4649127	6.08740234
5.81	2	3	68.9707324	5.84619141
12.86	1	3	45.0652329	6.48095703
9.85	3	1	14.3927199	6.15087891
17.86	2	1	14.9922579	6.99072266
12.04	1	1	11.9151075	8.16259766
2.99	1	1	60.2431435	5.98583984
26.73	1	2	22.4329421	8.39697266
3.24	1	2	151.434919	6.45556641
4.58	3	3	168.49485	5.79541016
9.42	1	1	14.7797083	7.81103516
2.56	4	1	58.1060659	6.68310547
5.74	1	1	41.6703559	6.59521484
14.05	1	2	26.0804403	10.1547852
5.19	2	1	29.8510541	4.75439453
5.99	1	1	33.0322621	5.52880859
4.58	2	2	66.4977224	8.23583984
4.01	1	1	59.5504702	5.54150391
3.42	1	2	66.5471569	6.50732422
1.25	1	1	132.82652	8.20654297
2.67	4	1	87.1951849	5.82080078
4.65	1	1	66.9984334	5.07177734
31.82	1	2	9.49489316	5.31298828
10.41	1	1	30.3575559	8.60205078
2.46	4	1	46.066241	7.28369141
4.23	1	1	37.3376759	7.89892578
5.47	1	2	91.8979062	6.85888672
3.69	2	1	59.6472915	6.23974609
4.09	1	1	90.9554603	5.77001953
6.52	2	1	30.5977275	6.12548828
3.05	1	4	192.29392	9.01220703
4.96	1	3	143.722711	5.36376953
5.16	3	1	29.0440402	5.31298828
3.16	2	3	181.285438	8.00146484
7.46	10	4	107.379618	4.97021484
20	1	1	17.3867907	5.73193359
2.51	2	1	102.851833	4.91943359
3.85	1	1	80.2227231	6.20166016
9.78	3	1	21.38337	10.184082
1.81	1	2	191.728065	7.25439453
2.05	1	1	109.460953	6.96142578
1.99	4	1	83.6482988	7.76708984
10.5	2	1	78.2482082	5.37646484
6.08	1	1	45.7984188	6.84423828
3.37	2	1	51.3815373	9.46630859
1.24	1	1	97.4239595	8.60205078
14.08	4	2	23.6724	4.77978516
3.39	2	1	33.5445175	6.12548828
7.71	2	2	55.5004649	6.31591797

Information Classification: General

5.29	1	1	40.7007742	6.69775391
12.3	2	1	14.4585733	9.45166016
3.42	1	1	112.359159	6.81494141
12.04	2	7	85.3964383	4.71630859
0.79	1	1	178.655612	8.10400391
0.99	2	1	239.908215	7.13720703
3.44	1	2	107.095736	4.95751953
0.98	2	1	161.818368	6.37939453
10.77	1	2	54.0552372	4.95751953
10.05	1	8	121.425234	5.63037109
4.36	2	5	189.432177	6.74169922
1.8	1	2	324.896023	5.28759766
5.55	1	4	124.369427	6.66845703
2.97	2	2	97.219754	8.71923828
2.84	1	2	74.5268471	7.72314453
1.28	1	1	95.5234612	8.52880859
13.44	2	3	48.5688136	6.10009766
4.43	5	5	174.826204	8.54345703
0.95	3	1	132.919946	4.89404297
4.45	3	4	138.485388	5.93505859
2.7	1	1	64.7097059	9.80322266
3.02	2	2	81.2672665	8.03076172
4.53	1	1	48.0188911	6.68310547
5.67	2	2	60.432931	7.79638672
2.83	2	1	81.723668	7.70849609
5.36	1	2	80.8643608	6.87353516
6.7	1	1	20.8722282	10.9018555
7.32	4	1	26.9583181	5.88427734
14.17	1	1	14.432435	9.59814453
20.05	2	3	47.6509537	5.17333984
1.46	1	1	173.791246	6.63916016
44.44	3	1	3.93801846	7.18115234
10.96	2	1	24.7691338	5.02099609
1.71	1	2	136.048821	7.38623047
8.87	10	1	28.9886933	4.77978516
4.18	2	2	80.8980749	8.20654297
1.59	2	1	194.023946	6.84423828
22.06	2	1	8.08183806	4.88134766
7.09	1	2	64.1681844	7.13720703
13.4	1	1	21.7349651	5.28759766
8.84	1	1	52.5219484	7.25439453
5.97	1	1	40.8781763	4.93212891
3.85	3	1	28.8737872	5.78271484
9.6	3	4	68.1402225	8.58740234
5.14	1	2	96.0150449	5.21142578
1.89	2	2	176.841727	6.48095703
2.88	3	1	90.6177052	6.10009766
3.7	2	1	76.0313922	7.06396484
7.53	1	1	42.163124	4.84326172
4.17	9	2	74.9896223	5.92236328
1.55	1	1	108.191162	8.44091797
6.22	1	2	59.5492735	7.94287109
5.39	2	4	111.1179	6.07470703
2.1	2	1	111.151125	5.18603516
24.72	1	1	10.3430659	7.37158203

Information Classification: General

3.08	1	1	86.795692	6.71240234
13.17	1	1	23.2198881	8.35302734
1.61	2	3	148.417727	7.45947266
2.9	1	2	123.548197	7.32763672
9.39	1	1	24.1722312	6.29052734
5.79	3	1	38.270117	8.77783203
1.78	5	2	130.338542	8.20654297
5.54	3	1	32.7501123	5.46533203
6.29	1	1	32.8893341	5.69384766
3.2	1	1	79.968564	5.92236328
2.1	8	2	185.301459	6.50732422
11.11	2	1	11.4408178	7.29833984
3.19	3	2	74.1665216	6.96142578
1.35	4	1	119.253198	8.80712891
8.38	2	2	43.3817342	5.35107422
2.41	2	1	103.643944	5.47802734
3.34	1	1	48.0127485	5.92236328
3.95	1	2	45.3200622	5.82080078
8.97	1	1	16.2736389	8.60205078
10.56	2	7	105.474141	8.00146484
3.63	3	2	83.7496308	8.51416016
4.58	1	3	135.6985	7.12255859
4.41	2	2	89.6077611	4.91943359
3.36	2	3	143.586474	7.54736328
4.47	3	2	105.048341	7.73779297
5.1	4	2	26.7116114	9.90576172
5.46	1	1	63.1033462	4.71630859
6.03	2	3	91.6688353	5.43994141
2.44	1	1	98.0188551	6.56591797
0.73	1	1	275.135439	6.37939453
10.11	1	1	19.5063942	9.23193359
1.83	2	2	176.04486	7.63525391
4.7	3	4	88.821112	7.23974609
35.29	1	3	23.392816	5.19873047
1.25	1	1	142.244148	8.79248047
2.5	2	4	336.375372	6.01123047
12.77	2	1	20.0688043	6.35400391
1.61	2	2	273.22226	5.68115234
7.36	2	1	49.7121392	6.85888672
6.47	1	1	38.6684541	4.55126953
9.6	2	3	42.3849471	6.58056641
8.72	4	3	41.654761	7.03466797
12.19	3	2	32.65719	6.66845703
2.05	2	1	81.3154239	5.73193359
5.34	2	2	71.8712974	8.11865234
3.64	1	1	36.00839	6.78564453
8.36	3	4	77.0961827	6.75634766
3.43	2	1	76.1223236	5.70654297
1.7	3	1	98.7242415	7.57666016
13.04	1	2	18.1606711	9.27587891
9.72	1	2	31.3244975	8.29443359
1.54	1	2	159.163366	5.71923828
4.88	3	3	91.1084641	6.10009766
1.78	2	1	78.9277768	8.36767578
5.23	1	2	59.7550642	5.08447266

13.89	1	2	19.5333985	6.56591797
1.59	2	1	77.2274638	4.83056641
13.24	1	1	15.4237972	9.34912109
9.87	2	2	17.4749691	9.31982422
5.97	2	1	23.0819558	8.36767578
1.12	1	2	190.637806	5.70654297
9.39	1	2	34.4398505	9.12939453
4.87	2	5	85.2843712	7.40087891
1.4	1	1	80.1564732	5.02099609
1.9	2	2	88.2860837	5.13525391
4.4	1	2	69.1050427	6.56591797
17.16	1	1	15.3846777	5.08447266
9.55	3	1	19.8600831	7.32763672
7.03	3	1	48.1476519	8.99755859
13.72	2	6	73.4822389	10.0229492
3.09	3	2	70.7532459	7.07861328
31.25	1	3	8.48124328	7.84033203
0.72	3	1	154.759136	6.23974609
10	2	1	16.4421286	6.52197266
3.93	1	2	93.3823875	10.0668945
4.72	1	1	42.6529604	4.85595703
10.93	3	1	101.542736	5.65576172
2.57	2	1	122.074654	6.68310547
10.73	2	2	40.5915674	4.89404297
4.03	7	2	103.768329	5.45263672
3.46	3	2	112.440344	6.59521484
8.33	1	1	37.3953709	9.93505859
9.85	2	2	15.0907207	9.59814453
3.24	2	5	203.405061	9.37841797
2.74	2	1	73.8422405	6.49365234
3.51	2	2	70.0777175	5.28759766
2.44	3	1	79.5803158	8.99755859
1.92	3	1	69.7529223	7.06396484
2.13	3	1	52.5884713	6.87353516
7.04	18	2	53.9852743	5.99853516
4.32	2	2	72.8817668	9.21728516
1.73	1	1	83.0506962	8.03076172
3.14	3	1	71.3799518	8.25048828
4.02	1	1	47.8280003	6.65380859
19.29	8	1	30.3912297	7.84033203
9.46	2	1	16.4090731	5.65576172
3.08	2	1	68.8088561	5.26220703
1.17	1	2	214.187283	9.04150391
1.92	2	2	144.839699	6.94677734
3.91	1	2	75.6146561	8.70458984
7.83	3	1	24.4533348	9.05615234
3.37	1	3	77.3929038	9.49560547
6.88	1	1	77.4511788	5.64306641
1.05	2	1	131.128522	8.77783203
10.34	1	1	28.8565686	4.97021484
2.18	2	1	62.3804244	5.59228516
11.89	5	2	25.8770724	4.72900391
6.55	1	1	30.9825182	7.12255859
1.84	1	1	101.137984	7.28369141
2.86	3	3	186.667008	6.52197266

Information Classification: General

4.6	1	1	88.4221997	5.21142578
1.91	1	1	72.590637	7.60595703
0.78	4	1	157.93685	6.75634766
13.6	1	3	37.0528686	5.26220703
7.57	1	2	48.5825195	4.91943359
2.95	4	4	202.034332	5.57958984
5.48	2	2	47.3576498	6.45556641
7.28	2	1	27.2325169	8.58740234
22.97	2	5	45.7015572	6.81494141
6.31	2	3	93.9671497	5.21142578
4.31	2	1	94.8796223	4.62744141
14.62	3	1	19.8318004	8.07470703
25.37	2	1	14.2129856	4.41162109
29.29	1	1	14.9124146	6.50732422
4.19	2	1	45.1029672	4.91943359
9.05	2	3	22.6663861	8.63134766
4.49	2	1	34.8326191	6.80029297
2.18	2	3	220.207591	5.96044922
6.54	1	3	53.4739013	5.12255859
4.36	1	1	99.4287544	5.23681641
3.5	4	1	50.3142959	8.16259766
4.24	1	1	61.1988161	6.55126953
0.8	1	1	165.95199	6.99072266
3.24	1	2	92.1523975	6.74169922
3.09	1	1	32.5322963	5.83349609
40.94	1	2	13.927633	6.66845703
1.64	1	1	81.5822887	6.55126953
1.44	1	1	60.8451089	6.03662109
9.22	1	1	31.8056546	7.16650391
2.88	5	2	109.044234	5.50341797
10.08	2	3	69.8698282	7.23974609
1.23	2	1	131.649143	5.78271484
6.48	1	1	54.0040682	7.86962891
3.89	3	2	98.8288578	7.64990234
30.34	1	3	10.4169769	4.97021484
2.52	1	2	136.27465	5.78271484
7.18	2	4	83.280031	8.83642578
3.12	1	3	163.681865	8.55810547
2	1	1	133.635819	5.46533203
18.14	2	3	26.1268185	8.42626953
6.19	1	2	88.5323622	5.22412109
1.65	1	1	111.955208	7.43017578
14.88	1	1	24.8781625	5.74462891
6.27	1	1	42.6896063	7.16650391
8.17	2	3	73.8180873	7.86962891
2.4	1	2	130.850147	7.22509766
6.42	1	1	20.9654545	7.13720703
2.89	3	1	83.5689958	6.35400391
3.27	2	1	40.9950462	8.99755859
1.75	2	2	196.503144	5.65576172
1.75	1	1	88.2786331	4.74169922
1.81	2	1	62.1735863	7.73779297
3.68	6	1	30.7188728	7.59130859
5.11	1	1	54.9503235	6.55126953
19.63	1	1	11.5791241	8.61669922

Information Classification: General

14.77	8	1	34.9897678	7.88427734
1.72	4	1	76.3456435	7.28369141
3.66	2	1	45.7314731	8.30908203
10.33	1	1	23.3827785	10.0229492
3.9	1	1	32.559017	11.2680664
1.24	1	1	154.022194	8.11865234
2.8	1	1	47.3071276	8.60205078
15.73	3	1	9.38220561	9.70068359
3.07	1	1	58.7222468	6.66845703
7.13	1	1	77.3926955	4.66552734
2.16	2	1	46.2043071	9.08544922
3.4	4	3	63.3026229	6.62451172
0.44	3	2	364.075054	5.05908203
2.5	2	2	80.4675342	5.92236328
1.82	1	1	69.9304751	6.72705078
5.68	2	1	48.1433096	5.13525391
26.8	5	1	10.9973392	4.62744141
3.57	2	4	268.959498	7.57666016
5.97	2	1	44.1207417	5.26220703
7.91	2	1	40.1829829	7.75244141
4.28	2	1	43.2240725	9.20263672
6.41	4	2	63.3691799	7.53271484
9.3	1	1	24.7833872	4.74169922
9.08	3	3	130.178343	6.75634766
9.52	3	2	44.7414115	5.63037109
7.89	3	1	21.9681344	10.3012695
6.09	2	2	55.3945774	8.93896484
5.49	4	1	53.1945459	5.24951172
4.55	1	1	39.8740461	5.38916016
2.35	3	1	79.7610204	7.21044922
8.46	1	2	63.3156222	8.14794922
1.33	1	1	129.072047	6.87353516
10.43	1	1	36.8611923	5.97314453
6.52	2	2	42.590526	5.49072266
5.55	1	2	79.7862958	6.11279297
12.65	1	4	65.814975	6.17626953
2.69	1	1	46.1972561	8.07470703
13.83	1	3	32.1997567	9.15869141
2.6	1	1	50.089869	6.91748047
1.58	2	1	65.9476833	6.43017578
7.43	1	1	22.8120813	6.43017578
1.23	4	2	172.342837	6.68310547
1.8	2	2	243.779283	8.67529297
13.15	2	2	47.0881495	6.36669922
4.61	4	2	38.0642842	9.04150391
2.79	1	1	55.4059042	9.23193359
1.22	2	1	125.414719	5.83349609
18.92	1	1	11.9536524	5.41455078
4.72	1	1	25.9832715	6.25244141
4.71	2	1	42.3897079	5.36376953
12.24	1	2	15.8960451	8.20654297
6.01	1	1	46.5446233	5.23681641
1.52	2	1	79.610119	9.36376953
7.54	1	3	77.674534	6.80029297
4.42	1	1	47.8424096	7.73779297

Information Classification: General

5.31	2	1	36.969436	5.63037109
3.82	1	2	74.2545752	5.79541016
6.8	1	3	136.546023	9.02685547
4.61	1	1	50.0536635	6.37939453
22.76	1	1	14.2148315	5.51611328
3.3	3	1	40.6770014	8.76318359
0.61	1	1	201.403027	4.98291016
12.82	1	1	13.1716717	6.91748047
3.17	2	2	107.635135	9.30517578
3.76	2	1	24.3933166	7.07861328
7.32	1	1	22.9494472	6.55126953
8.7	2	1	28.6186305	5.59228516
17.24	1	1	16.6641722	4.74169922
6.73	2	2	99.6063737	4.77978516
2.1	3	2	119.626383	7.44482422
2.53	1	2	194.808465	8.35302734
5.82	2	3	22.0732387	5.98583984
8.46	1	2	52.9351537	7.70849609
7.74	1	1	18.6738015	4.70361328
2.87	1	1	62.5286867	6.58056641
13.1	3	3	47.4689926	8.86572266
1.95	2	2	218.457064	5.04638672
6.75	3	2	42.80519	4.98291016
6.43	4	1	30.6489424	5.08447266
2.07	1	1	43.0021111	6.21435547
3.18	1	1	67.3992368	9.87646484
1.22	3	3	217.970774	8.48486328
5.39	1	1	33.5611021	5.26220703
8.63	1	1	15.7728438	4.72900391
2.57	1	1	52.2057273	6.58056641
13.01	3	3	42.0100621	8.35302734
9.28	1	1	37.6578448	7.84033203
8.54	1	1	50.5046922	6.25244141
1.65	1	1	88.7370492	4.95751953
3.1	1	1	48.3661847	9.14404297
6.56	2	1	28.1527085	6.88818359
3.21	1	3	171.708453	7.67919922
4.29	4	4	224.897218	8.33837891
1.63	3	1	160.457523	8.13330078
8.53	1	2	73.3747208	6.49365234
7.54	1	1	91.871159	7.62060547
2.05	1	2	166.043436	7.75244141
1.35	2	1	267.241143	5.94775391
3.88	1	1	24.9927505	6.91748047
1.43	1	1	154.661642	6.65380859
0.88	1	1	186.975152	5.33837891
4.89	1	2	91.6554023	6.78564453
6.87	1	2	50.0083261	6.59521484
2.76	1	1	96.9262153	7.03466797
12.8	2	2	32.2287505	4.95751953
3.47	1	1	70.0858534	5.79541016
1.25	2	3	252.487444	5.60498047
6.98	1	2	57.6341675	8.54345703
16.47	3	1	17.6437386	4.84326172
10.42	1	2	32.1798145	6.29052734

Information Classification: General

5.73	1	1	29.670037	5.59228516
3.63	2	3	124.361477	9.86181641
6.8	3	1	35.5003498	5.42724609
3.99	3	2	62.813772	5.93505859
14.36	1	3	45.9287808	8.19189453
38.37	1	1	8.62429988	9.42236328
7.78	1	3	49.0011261	4.98291016
3.82	3	3	182.239807	7.07861328
3.4	1	2	64.006883	8.79248047
2.61	1	1	84.3188821	5.22412109
0.95	1	1	207.727458	7.63525391
5.38	2	2	66.3337187	5.61767578
1.43	2	1	148.757449	5.59228516
4.83	2	1	56.3610726	5.79541016
1.35	2	2	109.864891	6.60986328
10.55	1	3	46.358251	5.28759766
3.63	1	1	66.9315093	7.00537109
4.33	4	1	22.6925952	10.9750977
2.92	2	1	78.4061017	7.32763672
3.09	2	3	208.792535	5.46533203
4.65	1	1	33.1615617	7.21044922
5.94	1	1	33.8439516	6.74169922
2.14	1	1	61.7238084	5.73193359
7.34	2	2	72.3024344	5.16064453
8.56	4	1	37.4573371	7.48876953
3.82	1	1	33.6455601	11.253418
3.57	1	2	65.3252285	8.41162109
3.4	2	2	94.3302232	6.72705078
6.49	1	4	101.140317	6.27783203
4.95	2	2	101.09624	6.96142578
3.08	1	2	114.80866	6.23974609
13.7	2	1	7.95488815	6.90283203
1.68	2	1	94.7187794	5.88427734
11.64	3	2	77.1009991	4.74169922
3.69	1	2	77.454183	6.04931641
4.12	1	2	102.729359	5.16064453
1.97	1	1	57.2907998	7.85498047
4.25	1	1	38.4165184	5.21142578
3.34	1	3	150.998381	6.87353516
5.9	1	2	39.0021014	11.4584961
1.71	3	1	117.078867	8.44091797
5.12	1	3	128.171064	6.07470703
1.56	2	5	426.334865	6.52197266
1.84	1	1	79.7155189	7.04931641
4.82	2	2	42.2948612	9.01220703
4.99	2	1	55.2889857	5.68115234
9.94	2	1	34.1999018	8.32373047
6.14	1	3	107.606916	7.06396484
2.54	1	1	88.4638475	6.94677734
15.32	1	1	14.0017259	5.52880859
4.51	1	1	58.1585814	4.43701172
3.57	1	1	65.4819114	6.74169922
3.79	4	2	95.799287	8.76318359
2.01	3	1	56.4273802	6.29052734
5.06	1	2	65.5646389	5.77001953

Information Classification: General

5.98	2	1	39.046095	9.81787109
5.6	3	1	37.9755412	4.60205078
22.86	3	2	19.8531664	5.42724609
1.92	1	1	71.0248878	5.43994141
2.14	1	1	75.5291564	7.92822266
5.33	1	1	57.369528	6.63916016
7.13	5	2	58.4006862	8.47021484
21.63	1	3	24.3917124	7.03466797
1.18	1	2	328.377342	5.32568359
5.05	1	1	62.880249	5.99853516
8.01	3	1	46.6675628	4.91943359
2.1	1	1	123.427348	7.18115234
1.98	2	1	68.261585	6.50732422
1.23	2	1	108.730354	6.93212891
4.35	2	2	69.0146215	6.34130859
4.57	1	1	67.112544	5.85888672
1.35	1	1	212.126669	5.80810547
8.51	2	1	31.0062837	4.95751953
6.71	1	2	80.5173177	9.18798828
5	1	2	58.3613478	7.73779297
1.41	2	1	64.1450767	8.04541016
12.17	4	2	33.9329039	7.38623047
2.29	2	1	107.41506	7.51806641
2.47	1	1	93.4760883	5.32568359
1	1	2	121.425315	5.99853516
0.19	1	1	628.434693	5.87158203
2.74	2	1	52.1397206	6.44287109
0.44	2	1	280.387548	5.51611328
2.19	2	2	137.153221	8.66064453
13.15	1	2	37.8923789	6.06201172
2.19	1	2	169.522211	6.16357422
4.78	1	1	35.1274726	9.37841797
5.61	6	3	96.1130979	6.68310547
2.28	2	1	60.5033419	5.12255859
1.75	2	1	77.2357324	8.58740234
4.78	1	2	117.981855	8.01611328
6.64	2	1	49.4452357	5.35107422
4.05	1	2	51.7039245	5.16064453
6.42	1	1	23.9294045	9.99365234
1.3	3	1	85.8268961	6.30322266
3.46	1	1	42.6458898	9.04150391
0.56	1	1	336.760374	6.44287109
4.57	1	1	64.1418349	8.70458984
2.11	3	1	89.270052	6.03662109
3.85	3	3	108.222348	7.69384766
5.23	1	2	104.611774	5.66845703
5.2	2	2	52.8108209	7.18115234
17.86	1	1	14.9475114	8.27978516
2.91	2	1	45.8586714	9.49560547
5.23	5	1	18.7908188	7.43017578
1.49	1	1	98.3468959	6.71240234
14.29	2	3	43.8819959	5.51611328
3.05	2	1	106.432224	5.78271484
4.6	3	2	98.8831799	6.35400391
6.05	1	2	31.94597	5.54150391

Information Classification: General

3.53	1	2	184.275014	6.31591797
2.05	1	1	65.6973816	9.80322266
32.58	11	3	19.2913271	4.74169922
2.83	1	1	144.176968	5.96044922
10.39	1	1	24.790709	7.22509766
3.45	3	1	60.3137117	5.68115234
13.41	1	2	29.9750523	4.83056641
2.27	2	1	57.8472577	9.37841797
3.24	1	1	94.2479149	6.75634766
0.83	1	1	131.388559	8.73388672
1.2	2	1	130.972925	8.66064453
3.01	1	1	85.1142399	5.79541016
6.02	3	1	24.3784139	6.56591797
9.63	1	1	23.5513748	4.50048828
11.72	3	1	14.4042424	6.56591797
4.66	1	1	37.1727086	5.89697266
12.04	1	1	12.0891401	8.83642578
7.85	2	3	61.2207155	5.71923828
1.95	1	1	102.314257	5.93505859
1.13	1	1	89.1412504	7.70849609
3.31	1	1	75.2287637	6.01123047
1.54	1	1	97.4743252	5.05908203
10.45	1	1	22.6901631	4.97021484
1.91	2	1	76.7783191	6.84423828
2.41	1	1	49.9840265	7.98681641
1.4	2	1	149.756421	7.50341797
1.93	1	1	72.7325049	5.50341797
1.89	1	1	94.1360889	5.05908203
1.94	1	1	47.5916516	6.91748047
4.25	1	1	29.9434605	7.40087891
1.22	1	1	93.7712175	6.23974609
1.36	2	1	151.948291	6.13818359
2.27	1	1	65.1833078	9.45166016
2.82	2	1	77.457965	9.52490234
0.87	3	1	159.343086	5.88427734
2.06	1	2	53.8111605	7.34228516
7.11	1	1	44.9221873	8.74853516
1.84	2	1	139.719887	6.94677734
18.18	2	2	40.3470594	5.04638672
9.7	1	2	14.3427381	9.48095703
6.28	1	1	22.1719902	5.85888672
4.53	1	2	87.2568428	5.17333984
4.23	1	1	36.6876765	9.64208984
1.65	1	1	101.604016	6.65380859
5	2	1	60.8957325	6.17626953
2.51	1	1	72.5730197	5.63037109
5.49	2	1	39.6867783	7.78173828
4.17	1	2	133.86049	5.24951172
5.56	2	1	48.2045139	7.75244141
1.36	1	2	280.319578	6.03662109
5.97	1	3	102.453241	5.28759766
5.52	3	3	75.5660704	6.22705078
5.32	1	1	40.6275123	5.65576172
6.82	1	1	39.7555012	8.08935547
4.61	1	2	46.7053959	4.52587891

Information Classification: General

0.77	1	1	215.940365	6.32861328
2.77	1	3	135.941126	8.51416016
6.22	1	1	41.5910221	5.30029297
5.45	3	1	49.2339396	7.73779297
3.42	2	1	46.184983	8.49951172
2.26	1	1	80.4442659	6.88818359
2.98	1	1	72.2170807	6.50732422
1.68	1	2	203.783831	7.76708984
3.45	1	1	109.767578	6.41748047
2.23	3	1	96.7235867	6.11279297
14.75	2	2	27.2302847	4.99560547
6.51	1	2	84.7149671	5.94775391
9.06	2	1	29.1812097	9.58349609
8.47	2	4	50.8574543	11.355957
1.94	1	1	200.034456	6.04931641
2.03	1	1	96.4994251	6.08740234
1.68	1	1	66.1756325	9.34912109
4.01	3	1	90.970957	5.12255859
18.99	2	2	17.1699759	6.35400391
1.72	2	1	86.5625672	6.50732422
0.98	5	1	207.0765	6.60986328
6.46	1	1	54.929192	8.26513672
2.62	1	1	95.9617004	5.61767578
1.59	1	1	125.436399	5.69384766
4.1	1	1	60.9788459	7.72314453
1.45	1	1	132.288289	6.58056641
1.47	1	1	136.3614	6.53662109
1.3	1	1	140.368352	8.83642578
5.43	2	1	52.2405849	7.69384766
2.87	1	1	58.3185068	9.87646484
3.84	1	1	82.9909903	7.54736328
4.71	2	1	32.0120499	7.38623047
11.82	1	1	11.9180305	5.18603516
14.47	2	3	35.178959	8.64599609
8.3	6	2	30.6348802	7.76708984
5.67	5	1	34.3707205	8.67529297
1.07	1	1	114.451496	7.09326172
2.41	1	1	55.84798	6.68310547
16.3	1	1	10.1482676	10.3745117
5.43	1	1	40.0654886	6.66845703
5.34	4	1	15.5218438	8.96826172
0.47	2	1	398.754005	5.68115234
0.65	1	1	187.800591	8.11865234
5.91	1	1	41.8222248	6.53662109
3.72	2	1	39.5786165	11.8979492
2.32	3	2	147.407346	6.59521484
5.09	3	3	61.9930246	8.16259766
5.81	3	1	97.1935466	7.62060547
3.51	1	1	66.9571032	7.95751953
8.56	1	1	45.5180759	4.94482422
4.02	2	1	22.1503745	8.39697266
6.8	1	4	148.267078	6.93212891
4.79	2	1	32.6692455	5.60498047
3.81	2	2	66.6630987	6.26513672
1.37	2	1	122.703828	6.99072266

Information Classification: General

9.22	2	3	57.3420525	6.35400391
1.2	1	1	166.398571	5.37646484
3.67	4	3	145.496976	7.31298828
2.51	2	2	98.5639834	9.37841797
13.41	2	1	30.8344114	5.03369141
3.11	1	1	50.9356336	7.45947266
2.51	2	1	61.121623	5.77001953
1.54	1	1	71.282202	6.08740234
2.03	4	1	80.1576567	7.78173828
4.55	2	1	29.8887091	9.72998047
14.34	1	2	30.9775233	4.86865234
25	4	1	8.74238397	4.61474609
4.94	3	2	85.9677378	5.28759766
3.95	4	2	96.924371	8.52880859
2.72	2	2	86.9578786	5.79541016
3.09	1	1	69.6048706	8.33837891
2.51	1	1	68.4153534	7.28369141
5.58	2	1	45.7135494	6.94677734
1.45	1	2	301.215808	5.46533203
9.38	1	1	38.5615869	10.0522461
4.31	1	2	95.2228817	6.82958984
5.46	1	1	20.8866496	7.69384766
9.71	3	2	43.2550575	5.82080078
3.63	2	1	67.8560238	8.88037109
12.79	1	1	18.9436422	5.35107422
1.53	1	1	96.3129719	7.69384766
7.3	2	1	14.5151379	8.82177734
2.57	3	1	53.3892123	5.40185547
1.09	2	2	220.886631	7.76708984
0.69	2	1	162.266706	5.31298828
5.11	1	1	26.5948809	5.64306641
1.91	2	1	108.968588	5.90966797
4.2	2	1	69.1399991	8.17724609
7.87	2	1	39.4106268	7.18115234
1.99	1	1	157.928736	5.89697266
28.24	1	1	9.79598831	6.81494141
1.15	1	1	168.233718	6.66845703
29.15	4	3	28.1199335	5.31298828
0.83	1	1	106.723232	5.61767578
4.1	1	1	43.4697753	4.83056641
3.18	3	1	91.1109955	5.47802734
5.9	1	1	31.3844875	5.02099609
3.17	2	1	60.1515875	5.56689453
18.02	2	1	12.0279287	7.19580078
13.43	4	2	25.6499759	7.85498047
7.69	1	1	35.708586	7.57666016
11.69	3	2	24.7655764	9.02685547
12.35	1	2	35.6466113	5.07177734
1.71	1	1	89.903274	7.32763672
2.8	1	1	61.4254796	9.52490234
10.45	1	1	32.187234	4.43701172
5.96	1	2	66.6025842	9.04150391
6.25	2	1	21.4030271	4.22119141
2.81	1	1	71.4939362	7.62060547
2.72	1	1	105.044211	5.69384766

Information Classification: General

4.41	3	1	54.1598729	7.02001953
15.05	2	3	20.8544865	5.65576172
1.77	1	1	117.257501	6.50732422
15.25	1	1	25.0457315	5.12255859
2.15	2	1	71.5162132	7.28369141
5.26	2	1	42.5330284	10.3305664
1.92	1	1	72.1211382	8.04541016
2.12	1	1	137.774621	5.59228516
9.33	5	2	34.3255024	7.35693359
6.59	1	1	67.6898211	5.75732422
0.61	1	1	162.771758	6.50732422
3.83	2	1	29.5569693	5.59228516
2.91	1	1	96.6866979	5.52880859
5.71	2	2	36.1363389	4.90673828
4.96	2	4	135.740553	7.85498047
3.79	1	1	75.2671776	9.24658203
0.89	1	1	176.40407	7.82568359
2.93	1	1	51.425112	8.23583984
1.83	1	1	117.897509	8.06005859
1.32	2	1	95.9314295	7.34228516
13.41	2	3	29.687892	5.12255859
4.28	1	1	35.0243918	6.52197266
5.76	3	1	26.8342365	6.58056641
4.71	4	1	39.3926408	7.51806641
2.17	2	2	86.3470116	9.40771484
2.35	1	1	70.1458547	7.69384766
0.55	2	1	249.872987	6.44287109
4.28	2	1	56.099726	5.14794922
2.04	1	1	158.552998	6.41748047
0.74	1	2	391.321601	6.84423828
1.14	2	1	246.397117	6.53662109
2.35	2	1	57.4446608	8.68994141
3.99	1	1	36.2936617	4.81787109
4.03	2	1	63.7304093	4.71630859
1.01	1	1	147.849128	5.55419922
3.67	2	2	111.639527	8.52880859
2.46	1	3	214.539712	7.69384766
3.05	1	1	43.71069	4.80517578
6.38	3	1	42.5869075	6.63916016
7.17	3	1	28.9571181	5.75732422
2.51	3	1	48.3851938	6.60986328
3.08	1	1	52.2545844	7.22509766
2.27	1	1	71.5300266	9.24658203
13.83	1	1	30.7911259	5.04638672
4.84	1	1	46.6503152	9.21728516
2.25	1	1	48.4881214	5.85888672
3.32	6	1	70.1221082	7.67919922
0.69	1	2	157.409152	9.07080078
6.13	4	1	50.8395027	4.74169922
4	2	1	41.5443165	4.53857422
1.56	1	1	94.6830224	7.48876953
1.69	1	1	111.345271	8.98291016
1.63	2	1	96.7510645	6.50732422
12.92	3	4	76.8998479	4.85595703
3.18	2	1	34.1149148	9.96435547

Information Classification: General

3.51	1	1	69.9003161	6.97607422
7.67	1	1	42.8658915	7.69384766
7.58	1	2	38.4146318	6.46826172
1.37	1	1	65.359599	6.90283203
2.94	1	1	88.2947017	5.10986328
3.46	4	1	82.4287407	8.10400391
6.56	2	2	68.934641	5.65576172
8.74	1	1	30.4530242	6.78564453
13.1	2	1	9.50993252	9.70068359
5.47	1	1	22.2263807	9.49560547
3.06	1	1	137.977199	7.21044922
34.38	1	2	10.1183401	9.75927734
5.17	1	1	31.2045636	5.51611328
4.91	1	1	36.8460619	6.87353516
2.77	1	1	31.710238	6.10009766
2.14	1	1	88.6925363	6.48095703
1.64	1	1	108.606021	6.17626953
5.23	1	2	136.105329	5.36376953
5.58	2	1	24.9600504	8.01611328
25	1	1	5.04951649	5.05908203
10.64	1	1	20.7850166	7.43017578
2.23	2	2	106.080806	6.85888672
12.5	1	1	24.7216645	5.60498047
4.78	2	1	67.9206549	6.29052734
3.39	2	1	31.0310501	9.46630859
2.52	2	2	118.138015	8.68994141
2.15	1	2	144.576168	9.10009766
2	1	1	47.5604105	6.45556641
2.44	2	1	78.3453313	5.36376953
7.49	2	1	20.8285818	7.73779297
1.01	2	1	118.990839	5.92236328
7.69	4	2	71.0295905	6.60986328
3.1	1	1	36.455211	4.89404297
3.1	1	1	44.5992768	6.30322266
27.85	1	2	17.1761378	4.29736328
8.14	1	3	25.461185	11.8540039
4.01	2	1	61.4870502	7.00537109
2.22	1	1	35.4929834	9.48095703
7.56	1	3	27.3610062	11.824707
1.9	2	1	75.755711	8.17724609
19.46	2	2	24.4175733	8.98291016
17.15	4	1	26.5151256	8.16259766
6.91	2	2	50.5753292	9.52490234
3.12	23	1	52.8657443	5.87158203
3.54	1	2	37.8446904	8.61669922
2.4	2	1	47.2621975	9.65673828
2.14	2	2	134.751693	6.32861328
2.03	2	1	44.1373452	7.19580078
7.19	1	1	34.2606735	5.08447266
16.42	1	1	14.8264443	4.56396484
3.13	2	1	42.0055824	7.70849609
3.03	2	1	49.3101747	6.81494141
2.96	1	2	98.0858703	5.61767578
3	2	1	77.1293051	6.20166016
1.1	1	2	139.410155	6.90283203

Information Classification: General

6.68	2	1	67.8393867	8.95361328
9.39	2	3	56.626925	10.6967773
8.56	2	2	39.1082617	7.86962891
3.01	1	1	40.3386135	9.31982422
10.83	2	2	43.1660062	9.15869141
2.66	1	1	92.311274	6.69775391
4.11	2	1	24.1152894	5.37646484
9.47	1	1	21.1104133	5.42724609
1.1	1	1	131.055176	6.87353516
4.18	2	1	40.4548392	5.04638672
5.38	2	1	29.6574449	5.19873047
1.62	1	1	90.1416539	8.03076172
2.95	1	1	38.6538065	7.69384766
3.93	1	1	43.8729299	7.45947266
10.07	1	1	29.9851711	5.22412109
3.99	1	1	34.1527233	8.38232422
4.73	1	1	57.9379168	4.93212891
4.6	3	1	44.3616295	4.93212891
2.13	2	1	75.5359534	4.32275391
2.51	2	2	133.415158	6.08740234
4.81	28	1	43.9879476	5.33837891
11.58	2	1	10.8812484	5.16064453
5.08	5	1	21.8204654	8.42626953
3.88	1	1	61.6270163	8.29443359
12.74	1	2	47.965839	4.48779297
9.81	1	1	21.991045	10.9897461
1.73	4	2	191.076923	7.75244141
1.16	3	1	123.427309	6.62451172
1.89	1	1	63.6191568	8.29443359
3.89	1	1	55.7565459	9.27587891
1.29	1	1	137.673541	7.66455078
1.87	3	1	107.401679	5.70654297
43.75	2	1	3.61174892	4.32275391
1.58	3	1	78.4400899	9.11474609
2.87	1	1	42.7177269	9.48095703
0.78	3	1	200.289579	5.89697266
1.67	3	1	71.647002	6.82958984
5.17	1	1	19.8767317	5.71923828
10.84	1	1	35.8434619	8.80712891
0.47	1	1	115.570201	7.34228516
1.78	1	2	149.790136	7.81103516
17.21	5	2	24.9260033	5.55419922
1.07	3	1	73.5165238	5.69384766
4.95	2	2	35.8719904	8.98291016
8.86	1	1	17.9652996	8.67529297
2.28	2	1	98.337054	5.93505859
5.21	1	2	89.2927348	5.26220703
3.36	2	1	66.2974637	5.16064453
7.42	2	3	46.8491912	7.59130859
4.15	1	2	89.7615204	9.52490234
2.37	3	1	95.1935617	6.49365234
10.78	1	2	17.9555569	9.29052734
2.35	2	1	57.1147196	8.29443359
2.62	2	2	149.500322	7.43017578
0.76	1	1	176.407653	5.38916016

3.06	1	1	74.8649064	6.93212891
3.61	2	1	47.0084882	5.57958984
3.79	2	1	34.4254138	8.90966797
2.39	2	1	85.6517499	5.35107422
3.85	1	1	20.4437552	7.23974609
1.97	1	1	50.7929564	7.73779297
3.44	2	2	42.3622167	8.27978516
9.23	1	4	77.380967	8.90966797
2.21	1	1	35.3727674	7.92822266
0.41	1	1	217.053737	6.53662109
1.69	1	1	76.0706069	7.13720703
6.46	1	1	60.8761742	6.30322266
1.79	2	1	70.8193546	5.79541016
2.85	1	2	106.16116	7.03466797
1.32	5	1	85.6013711	5.22412109
7.06	1	1	37.2927681	5.49072266
8.72	1	1	20.2885041	5.07177734
1.93	3	1	61.9450452	7.12255859
4.81	1	1	23.1100554	5.55419922
1.63	4	1	76.9457205	7.12255859
2.06	1	1	109.930919	7.02001953
1.75	2	1	121.084376	5.69384766
3.02	1	1	60.6503381	8.44091797
5.12	3	3	109.936066	7.10791016
3.96	2	1	42.5259299	8.36767578
1.32	1	1	100.123677	5.19873047
14.11	8	4	54.4190821	6.22705078
2.66	2	2	61.3580254	9.59814453
5.54	2	1	32.3325264	8.57275391
1.99	2	1	152.501217	5.78271484
6.75	2	1	18.3362184	6.40478516
2.41	1	1	112.646644	8.58740234
6.16	1	1	39.6461271	7.62060547
11.78	1	3	35.4938794	6.93212891
17.39	2	1	10.3583896	8.54345703
3.77	1	1	78.2918544	8.71923828
3.23	2	1	88.587826	6.23974609
1.66	3	1	67.0978574	7.76708984
6.87	2	2	51.8490071	8.92431641
2.2	2	1	68.967072	6.77099609
1.95	1	2	95.7123149	7.79638672
1.95	1	1	40.0254354	6.84423828
1.26	1	1	108.40537	7.56201172
0.75	1	1	209.346505	8.89501953
3.85	2	1	35.2952026	9.14404297
2.59	2	1	114.263632	6.45556641
2.25	1	1	116.713725	5.56689453
3.05	1	1	64.322459	5.94775391
1.25	2	1	61.6397322	5.45263672
4.91	1	1	52.7561497	5.30029297
8.04	4	1	12.3281134	6.77099609
1.79	2	1	87.1284699	8.95361328
2.64	2	1	79.0956212	6.90283203
3.26	2	1	48.1812714	9.05615234
1.52	4	1	72.9390664	9.55419922

Information Classification: General

8.74	2	2	22.7935474	6.21435547
9.5	1	1	22.8695394	5.10986328
9.49	3	3	48.4127844	9.46630859
5.32	2	1	38.2821235	5.14794922
8.82	1	2	41.1891869	5.43994141
4.86	1	1	65.8830853	8.82177734
3.66	1	2	109.498853	5.12255859
3.74	2	1	55.5079764	6.36669922
4.59	1	1	43.4139059	5.88427734
13.27	8	1	12.9034651	7.12255859
1.6	2	1	98.6176205	6.60986328
2.29	2	1	49.3109355	7.70849609
3.24	2	2	183.411394	6.34130859
2.63	2	1	80.6339639	6.85888672
9.66	2	2	38.2005625	5.40185547
6.08	1	1	19.955369	6.72705078
9.01	1	1	13.5499229	9.30517578
3.13	1	1	32.4637089	6.32861328
13.35	4	2	35.6955764	9.87646484
2.67	1	1	133.457664	6.26513672
3.17	2	1	72.1105677	6.40478516
4.85	1	2	46.5649951	8.07470703
4.48	1	1	73.5049745	6.32861328
5.22	1	1	27.2920159	6.37939453
1.45	1	2	225.639488	6.58056641
5.86	1	1	31.4961903	8.13330078
3.64	1	1	68.0655606	5.59228516
1.68	1	1	58.8063522	5.64306641
4.14	1	1	57.3627968	6.88818359
11.18	2	1	17.2718946	7.44482422
5.99	1	3	96.0945817	7.32763672
2.96	1	1	48.7117427	8.32373047
3.59	3	1	47.7579626	7.56201172
6.25	3	1	40.0460967	9.83251953
5.93	3	1	42.2732588	9.89111328
8.37	1	1	49.9665214	5.07177734
9.84	1	2	50.0291602	6.74169922
1.46	1	2	288.852834	5.36376953
1.28	1	2	253.136087	5.80810547
0.76	1	1	104.534912	6.25244141
0.63	2	1	141.608255	6.94677734
10.89	1	1	41.2491821	6.97607422
1.88	1	1	87.9819012	4.89404297
2.76	1	1	57.6837391	8.27978516
3.17	1	1	28.3561064	9.23193359
9.76	3	1	23.2457959	6.04931641
0.42	1	1	267.143829	4.99560547
1.36	3	1	64.9213189	9.02685547
7.18	1	1	23.6884169	7.38623047
2.44	3	1	42.3851291	8.61669922
1.86	1	1	47.3903383	5.60498047
0.79	1	1	165.235276	5.46533203
1.04	1	1	137.106015	5.22412109
20	1	1	8.39918099	7.07861328
1.81	1	1	102.995743	6.78564453

Information Classification: General

3.26	1	1	47.5282696	8.14794922
3.58	1	1	88.657135	6.88818359
10.7	1	1	20.6753442	7.76708984
2.81	3	1	36.8901066	8.57275391
2.58	1	1	34.7605617	7.25439453
1.82	1	1	53.9742976	5.19873047
9.63	1	2	30.9130671	5.04638672
4.68	2	1	48.7478237	6.66845703
1.58	1	1	62.4603321	7.64990234
15.89	4	1	27.3316675	6.41748047
1.68	2	1	153.350951	5.80810547
5.65	2	1	14.1941909	10.081543
5.84	1	2	76.1470844	8.41162109
2.43	1	2	113.260288	6.06201172
4.03	2	1	53.2198699	5.07177734
29.76	6	5	22.6633803	6.21435547
4.47	1	1	48.9986162	8.30908203
1.32	2	1	107.169079	6.07470703
7.59	1	1	17.480896	5.84619141
6.4	3	1	21.8266686	8.35302734
2.66	1	1	64.9070826	5.04638672
4.68	1	2	55.725294	7.32763672
3.17	1	2	151.701906	6.01123047
7.71	3	2	50.7926375	6.68310547
2.5	1	2	124.262376	5.79541016
5.24	2	1	26.3001405	4.00537109
19.46	1	2	28.0052011	8.67529297
8.96	1	1	23.0107677	8.29443359
3.83	1	1	36.2196935	6.97607422
4.51	2	2	89.2955259	7.26904297
2.48	1	1	46.1365732	7.37158203
2.41	5	1	122.163744	8.82177734
3.11	5	2	134.548519	6.26513672
18.18	1	1	9.13379378	9.29052734
10.22	2	1	36.2133	6.46826172
1.26	1	1	132.857464	6.23974609
4.99	1	1	37.7492762	5.87158203
1.83	1	1	60.6083545	6.63916016
4.48	1	1	65.5923877	6.02392578
0.82	2	1	148.874173	5.40185547
19.83	1	2	13.879654	5.22412109
1.66	2	1	69.0592082	5.70654297
2.37	1	1	66.598085	6.58056641
1.49	2	1	72.5348358	6.97607422
4.76	2	1	36.8857342	5.43994141
0.54	1	1	199.344992	4.98291016
4.96	1	2	58.6473439	10.1254883
2.17	1	1	112.566058	6.87353516
1.17	1	1	128.679631	8.98291016
8.17	2	2	52.9412598	6.37939453
1.62	2	1	73.69876	6.16357422
4.19	1	1	18.3090124	6.01123047
1.32	1	1	87.6794983	9.55419922
1.22	1	1	85.8499132	6.06201172
4.88	2	1	52.8168194	8.22119141

Information Classification: General

1.33	1	1	209.022187	7.00537109
0.93	1	1	186.381866	5.08447266
4.01	4	2	98.7787295	6.23974609
4.81	1	1	52.5329576	6.60986328
1.76	1	1	62.5557219	7.94287109
2.72	2	1	50.4412144	8.13330078
2.46	2	2	48.0535867	5.55419922
4.71	1	1	20.4002346	9.87646484
1.93	2	1	81.4381472	5.26220703
1.93	1	1	79.9800555	6.03662109
5.9	1	2	63.9325759	8.11865234
2	1	2	163.225573	6.23974609
2.33	1	2	126.906412	7.19580078
4.89	1	2	36.4994648	9.10009766
1.25	1	1	136.864526	4.90673828
3.13	1	1	34.0230617	6.07470703
5.71	3	1	52.2585437	5.42724609
3.21	3	1	23.6314073	5.87158203
1.49	1	1	111.94687	5.93505859
1.86	3	1	49.3954923	8.27978516
3.98	3	1	38.4351043	6.31591797
2.41	3	2	121.622929	7.85498047
6.9	3	1	16.4462755	8.07470703
1.19	2	1	67.7392527	8.19189453
6.85	1	1	35.9987688	8.57275391
4.56	1	2	32.1270851	6.99072266
3.76	2	1	67.1553358	7.60595703
4.85	2	1	26.6212054	4.98291016
4.21	4	1	40.8197532	7.12255859
2.76	2	1	105.149164	10.1254883
8.33	2	1	17.5521997	5.23681641
5.62	1	1	37.882303	5.45263672
1.67	2	2	193.322472	6.99072266
2	1	1	131.665818	7.37158203
2.36	1	2	105.421905	6.56591797
4.86	1	1	26.8663509	4.95751953
5.29	1	1	20.925352	9.40771484
26.79	2	1	12.5810104	4.64013672
2.11	1	1	130.13054	8.00146484
4.93	1	1	59.7543044	10.6967773
5.7	1	1	43.0996862	9.37841797
0.33	2	1	287.121668	5.90966797
0.7	2	1	159.51813	5.21142578
0.49	1	1	240.939274	5.63037109
4.55	2	3	113.666163	5.41455078
2.82	4	2	124.039937	8.70458984
5.22	2	1	27.9500352	4.97021484
10.17	1	1	12.8526217	5.51611328
2.31	1	1	43.5799148	8.19189453
4.61	3	1	38.0528824	4.69091797
19.84	1	1	14.154944	8.13330078
3.65	1	1	60.7883129	7.15185547
1.57	1	1	125.606998	9.37841797
1.08	1	1	124.691734	5.46533203
0.86	1	1	126.387924	6.10009766

Information Classification: General

2.45	1	1	46.9383948	9.36376953
4.01	2	1	42.0569459	9.34912109
7.99	1	1	34.0500173	4.86865234
1.73	2	1	149.103877	6.34130859
5.52	1	1	32.3080509	11.2680664
5.84	1	1	47.6315856	10.1987305
3.14	2	1	25.0534335	6.29052734
1.31	2	1	69.6785217	4.91943359
4.5	1	1	35.6707387	6.02392578
2.86	1	1	54.8173278	8.16259766
7.68	1	2	52.6958992	4.95751953
4.48	1	1	46.1812158	9.40771484
4.26	1	1	39.4471481	4.89404297
1.1	1	1	106.08038	6.96142578
1.07	1	1	105.620858	8.01611328
1.63	1	2	117.819779	5.79541016
7.27	1	1	31.4237222	4.91943359
2.23	1	1	90.1277086	5.79541016
2.32	2	1	101.999095	6.21435547
3.61	1	1	49.2465688	8.52880859
6.02	2	2	87.0272029	6.17626953
16.48	1	1	20.7725983	9.48095703
4.5	1	1	85.8558777	6.34130859
0.89	1	1	128.065787	6.29052734
4.35	1	1	84.8550123	8.89501953
3.03	1	4	96.3573814	5.55419922
5.81	1	1	29.0129778	8.52880859
1.87	2	1	71.1055304	5.08447266
7.64	1	1	34.9078789	8.57275391
3	2	1	26.3553268	6.18896484
1.79	2	1	57.3981189	10.0229492
2.05	2	2	131.950692	8.35302734
3.3	1	1	63.0860488	8.61669922
6.03	1	2	39.150275	5.57958984
0.92	1	1	123.96013	6.84423828
2.51	1	1	113.258786	6.17626953
3.22	2	1	51.7974991	8.41162109
3.17	1	1	24.1732769	7.95751953
2.63	1	2	93.5152697	6.31591797
6.01	2	1	35.9713409	8.99755859
0.39	2	1	390.029738	8.32373047
2.84	2	2	98.9909972	6.84423828
18.18	2	1	15.9431893	5.73193359
2.64	2	1	54.9925163	7.23974609
0.58	1	1	167.248386	5.94775391
6.04	1	2	34.0477324	6.18896484
3.1	1	2	47.0125253	6.96142578
2.34	1	1	51.8751711	7.70849609
4.11	1	1	46.2712648	6.39208984
2.36	1	1	113.390275	5.96044922
1.77	2	1	70.5750519	9.46630859
1.62	2	1	113.017759	4.75439453
2.99	2	1	34.1086391	11.590332
7.66	1	2	27.7278162	10.3598633
18.33	1	1	6.90965313	9.92041016

Information Classification: General

3.59	3	1	41.2641585	5.64306641
18.56	1	1	11.0946291	9.14404297
5.37	1	1	33.731488	6.44287109
8.89	1	1	36.0854363	5.08447266
4.1	1	1	47.2862081	9.74462891
14.63	1	1	9.32086855	9.52490234
11.82	1	1	12.5017702	10.1694336
4.32	1	1	21.0512028	9.61279297
5.58	2	1	30.3607895	7.78173828
13.82	1	1	16.9879045	8.99755859
11.06	2	1	23.5308929	5.54150391
6.01	1	1	26.6319532	9.21728516
3.11	1	1	62.7601908	5.41455078
1.98	1	1	128.941562	6.31591797
1.3	2	1	139.363002	5.83349609
8.01	2	1	40.0165863	7.44482422
4.62	1	1	34.7795764	9.24658203
1.66	1	2	62.5304021	6.80029297
3.36	1	1	62.5928613	5.64306641
3.51	1	1	31.4059348	6.12548828
21.57	1	2	16.9998157	6.80029297
2.66	2	1	54.6388402	9.21728516
2.61	1	1	59.9350743	7.41552734
9.68	1	1	34.8532004	7.44482422
1.68	3	1	76.5506099	4.85595703
4.55	3	1	67.5102605	9.30517578
6.31	1	1	24.6585776	8.80712891
12.88	1	2	52.5988745	8.38232422
10.79	1	1	27.0399141	8.27978516
1.66	1	1	76.034548	9.08544922
4.68	1	1	43.380006	5.98583984
6.8	1	1	34.2555279	7.00537109
8.01	2	1	45.7365615	6.48095703
1.95	1	1	75.1625629	6.72705078
5.29	2	2	52.8102832	8.26513672
0.52	1	1	196.232217	5.21142578
1.24	3	1	155.890945	6.85888672
2.57	1	1	87.9103389	5.99853516
5.62	1	1	45.3478564	4.97021484
2.55	2	1	55.6560275	7.44482422
1.94	1	1	74.6087527	6.34130859
1.51	2	1	51.0902443	5.51611328
1.3	1	2	207.758012	5.77001953
2.25	2	1	60.0058682	8.39697266
1.29	1	1	92.586519	6.90283203
4.62	1	1	49.870067	6.07470703
8.01	1	1	32.1847109	8.17724609
2.67	1	1	47.1874063	7.10791016
1.86	1	1	54.530305	6.26513672
4.14	1	1	19.5020733	9.43701172
3.47	1	1	46.7640651	6.62451172
2.01	1	1	51.41785	8.70458984
6.93	1	1	54.8906031	8.95361328
1.74	1	1	97.0017169	6.60986328
5.38	1	1	58.4213431	8.20654297

Information Classification: General

5.37	1	1	33.2464806	5.27490234
2.6	1	1	74.9988237	6.71240234
7.43	1	1	20.0763454	7.32763672
2.43	2	1	94.8615192	8.08935547
1.61	2	1	124.702282	8.90966797
5.92	1	1	37.579081	9.43701172
3.37	1	1	104.958194	7.82568359
3.98	1	1	45.5711214	7.54736328
12.93	1	1	27.8936632	6.10009766
5.73	1	1	38.7746408	6.55126953
2.88	1	1	61.5006437	6.11279297
3.75	1	1	81.5764593	7.48876953
4.25	1	2	48.0154588	5.52880859
1.02	2	1	110.145894	6.74169922
1.72	3	2	184.335384	5.05908203
6.49	1	1	43.1395659	5.18603516
7.07	1	1	21.9131059	5.65576172
1.91	1	1	152.350384	7.18115234
3.46	1	1	40.9116499	5.00830078
8.42	1	1	20.0579927	8.76318359
2.28	1	1	62.8292124	8.22119141
8.91	1	1	27.6417934	7.41552734
5.2	2	1	41.0538412	5.96044922
1.03	2	1	97.0620026	4.76708984
9.87	1	1	16.9839539	8.52880859
3.91	2	1	58.0566039	9.05615234
6.67	1	1	15.0495346	7.18115234
2.06	1	1	113.122037	5.38916016
7.62	1	1	46.0445756	9.10009766
8.72	3	1	38.8242876	5.27490234
6.28	1	1	25.8746646	4.75439453
46.15	4	1	4.47703912	4.22119141
1.48	2	1	76.0949395	6.11279297
1.95	1	1	56.344696	6.72705078
0.73	1	1	166.509429	6.63916016
7.54	1	1	53.9959821	6.59521484
2.72	1	1	68.2022373	5.36376953
3.84	2	1	81.7346015	7.69384766
1.13	7	2	224.973923	6.49365234
2.27	1	1	82.7981371	4.88134766
4.34	1	1	41.1725335	9.04150391
1.15	1	1	55.7652717	7.54736328
2.49	1	1	87.1271021	8.38232422
11.04	1	1	16.8664456	7.54736328
3.21	1	1	49.4689553	6.99072266
4.68	1	1	18.7263053	5.41455078
8.99	1	1	20.9279534	7.32763672
4.74	1	1	21.3278595	7.35693359
5.08	1	1	57.7016947	5.18603516
10.88	1	1	21.0697366	7.28369141
8.36	3	1	32.9785016	4.43701172
9.84	1	1	22.1128939	9.78857422
7.01	1	1	29.7485934	9.99365234
5.16	1	1	54.8012033	6.55126953
5.95	1	1	21.5343613	4.99560547

Information Classification: General

2.89	1	1	40.2609975	5.98583984
6.98	1	1	34.3416253	4.60205078
12.64	1	1	19.8203794	9.40771484
2.09	1	1	70.1131503	6.52197266
2.83	2	1	62.8233075	5.40185547
4.35	1	1	28.2980641	8.92431641
1.49	1	1	98.8134575	5.79541016
2.56	3	2	98.3063662	6.06201172
2.02	1	1	160.248996	6.91748047
3.05	2	1	84.8642206	6.74169922
1.48	3	1	69.4428579	6.72705078
4.91	2	1	32.5138697	8.88037109
0.53	1	1	187.008136	6.69775391
2.4	2	1	93.1508709	6.43017578
4.46	1	1	59.8408232	7.89892578
2.62	1	1	55.5919834	7.28369141
2.25	1	1	67.5129369	4.83056641
1.14	1	1	105.728878	6.48095703
1.57	1	1	211.231522	5.99853516
5.46	1	1	35.9226058	9.53955078
1.31	1	1	81.7747154	5.78271484
1.09	1	1	60.3820445	8.82177734
4.53	1	1	59.7886752	5.68115234
1.22	2	1	92.6285492	8.07470703
8.71	1	1	34.87971	8.10400391
2.77	5	1	52.3572632	5.52880859
2.6	1	1	64.5833743	5.97314453
17.51	1	1	23.4708572	5.19873047
2.96	1	1	74.3291631	6.10009766
2.68	2	1	59.4455763	9.02685547
9.56	1	1	40.48209	4.65283203
1.61	3	1	50.2518093	8.08935547
4.17	1	1	31.9805181	8.67529297
7.87	1	1	19.3508036	8.89501953
2.02	3	1	123.314672	7.54736328
1.25	1	1	124.329572	5.75732422
4.26	1	1	34.8382678	6.74169922
4.45	1	1	50.7390549	5.42724609
8.96	2	3	71.8361385	9.94970703
4.72	1	2	70.0431005	5.87158203
8.24	1	1	20.6962519	5.13525391
2.03	1	1	97.8546832	6.87353516
5.6	1	1	38.189603	6.93212891
0.65	1	1	190.792993	8.01611328
2.19	1	1	112.366257	8.60205078
7.6	1	1	27.8756629	8.68994141
2.39	1	1	105.172404	4.97021484
2.07	3	1	58.4949591	4.86865234
5.86	1	1	55.1532148	7.31298828
4.04	1	2	86.9946189	6.96142578
13.25	2	1	18.652413	4.99560547
11.32	1	2	17.8027468	11.7368164
2.18	1	1	56.0176362	6.71240234
1.2	1	1	110.873501	5.66845703
2.09	1	1	52.4698681	7.69384766

Information Classification: General

1.29	1	1	113.796087	7.88427734
2.88	1	1	52.1019708	9.93505859
15.74	1	2	26.7017746	4.89404297
2.27	2	1	59.0760303	6.20166016
12.47	1	1	39.394987	7.54736328
6.8	1	1	47.9742339	7.34228516
1.7	1	1	134.192165	7.28369141
5.94	2	1	57.1505373	8.57275391
2.59	1	1	86.3050844	5.97314453
1.18	2	1	64.0442924	8.20654297
2.89	1	1	41.5299509	6.78564453
2.13	2	1	42.066832	8.58740234
1.1	1	1	83.3345854	6.78564453
4.06	4	3	108.13544	8.20654297
7.69	2	1	38.0439754	4.72900391
9.61	1	1	36.1111891	8.67529297
1.77	1	1	84.1371984	8.99755859
1.14	2	1	78.3184073	5.40185547
5.05	1	1	50.6647317	8.93896484
6.98	1	1	19.8288851	4.99560547
3.35	1	1	91.7183309	5.40185547
12.22	2	2	43.8901141	7.72314453
5.05	2	1	49.9953158	8.26513672
6.75	1	1	53.6516897	8.04541016
2.5	1	1	87.3357191	7.56201172
3.89	3	1	45.3455616	4.98291016
7.87	1	1	34.6369423	9.31982422
7.31	1	1	30.399062	5.31298828
2.23	1	1	94.4313076	6.74169922
1.09	3	1	262.590961	5.80810547
2.04	3	1	37.3972808	8.42626953
3.17	4	1	48.8763647	7.03466797
0.86	2	1	131.523116	7.41552734
6.82	1	3	71.9816312	9.31982422
2.32	2	2	120.427899	5.87158203
1.15	3	1	66.0925285	8.89501953
3.74	1	1	36.2644223	6.45556641
3.3	3	1	44.0492119	8.39697266
5.05	1	1	43.3320623	5.04638672
5.21	2	1	32.5965739	9.29052734
0.84	1	1	132.969505	5.78271484
19.79	1	1	9.98211362	11.5610352
2.84	2	1	61.5857507	6.02392578
1.73	1	1	65.2777315	9.81787109
3.08	1	1	66.0883833	5.59228516
6.19	1	1	24.3045452	5.70654297
0.8	1	1	128.215784	6.20166016
10.19	1	1	22.080337	6.80029297
2.39	1	2	105.695168	10.081543
1.28	1	1	59.3230506	6.96142578
4.89	1	1	79.3713168	5.09716797
3.49	1	1	38.6015684	9.37841797
4.97	2	1	39.4527222	8.86572266
2	2	1	121.98507	6.29052734
5.05	2	1	41.3116751	8.39697266

Information Classification: General

4.11	2	1	42.055592	9.45166016
2.92	1	1	98.9206355	8.63134766
2.38	2	2	127.703076	7.72314453
13.33	1	1	11.8348564	6.36669922
2.96	2	1	40.348069	8.55810547
22.92	3	2	10.3674627	9.90576172
13.79	1	1	15.5636033	5.09716797
1.73	1	2	108.242419	7.89892578
1.46	1	1	83.7458614	6.25244141
1.48	1	1	120.963504	5.16064453
17.76	1	1	16.4647333	5.09716797
1.43	1	1	126.211519	6.85888672
2.12	1	1	62.4942215	5.92236328
2.07	2	1	41.7189555	8.89501953
2.59	3	1	77.8654551	7.51806641
1.21	1	1	83.3226464	9.05615234
22.12	1	1	11.197858	8.73388672
1.57	2	1	56.0311974	5.51611328
3.39	2	1	39.0696701	8.33837891
1.25	1	1	73.8507624	5.64306641
1.16	2	1	198.821621	4.93212891
0.87	1	1	257.591922	6.01123047
0.63	1	1	159.928941	7.28369141
18.64	1	1	13.739598	5.69384766
2.6	1	1	74.4754966	6.44287109
6.21	2	1	47.5086379	5.47802734
0.84	1	1	125.634661	5.80810547
0.62	2	1	155.710333	6.66845703
1.09	1	1	300.739892	5.54150391
4.95	1	1	63.3514615	8.89501953
5.61	2	1	22.3555373	9.12939453
2.8	1	1	57.4631664	6.20166016
0.56	2	1	298.872563	7.23974609
1.98	3	1	69.8706637	8.66064453
1.29	4	1	109.950892	5.55419922
2.1	2	1	109.059097	8.85107422
5.54	2	1	49.2451782	8.35302734
1.24	1	1	100.511063	7.07861328
13.89	5	1	7.92525636	11.3120117
1.92	2	1	85.9285848	9.33447266
3.17	1	1	74.144833	9.17333984
1.67	1	1	70.9265606	4.64013672
0.15	1	1	540.903534	6.32861328
3.8	1	1	45.786881	7.84033203
0.78	1	1	354.92648	6.50732422
2.33	2	1	90.6949134	5.74462891
3.07	1	1	35.5583139	4.83056641
6.37	2	1	44.3631715	4.97021484
0.71	1	1	231.66403	5.60498047
2.07	2	1	97.1497527	5.77001953
0.84	1	1	217.442788	6.77099609
1.22	1	1	81.0349861	7.69384766
1.32	1	1	120.037543	6.85888672
5.53	3	1	52.5214838	6.41748047
0.76	1	1	283.733962	7.35693359

Information Classification: General

1.95	1	1	99.1088969	8.98291016
3.42	1	1	86.6443157	7.43017578
2.81	2	1	32.3166877	6.37939453
0.45	2	1	247.729164	6.58056641
2.62	1	1	99.7579005	7.23974609
2.02	1	1	39.2457826	8.93896484
0.99	1	1	278.894597	6.25244141
0.8	1	1	332.186742	6.82958984
6.54	1	1	43.1808208	8.54345703
8.06	10	1	14.2727515	10.418457
0.58	3	1	234.003775	6.53662109
6.63	1	1	38.8318268	5.79541016
5.5	3	1	45.2373053	9.15869141
1.67	2	1	71.7077701	5.23681641
1.5	1	1	113.748307	6.60986328
0.44	1	1	282.634318	6.43017578
2.01	4	2	92.0263971	6.22705078
0.35	6	1	293.444168	5.92236328
4.32	3	1	36.3201831	5.17333984
1.32	3	1	160.197708	8.29443359
1.75	1	1	204.797364	6.77099609
1.54	1	1	65.416619	9.40771484
1.83	3	2	61.7604862	7.92822266
3.68	3	1	31.0869097	5.59228516
0.77	1	1	237.473546	6.97607422
5.17	2	2	29.8638858	5.26220703
4.09	1	1	53.1900262	5.69384766
2.91	1	1	92.7170436	7.73779297
2.29	2	1	115.820302	7.85498047
3.18	2	1	95.8416264	5.78271484
25.42	4	1	11.8995519	3.82763672
1.86	1	1	195.453854	8.17724609
0.64	1	1	142.241254	7.21044922
1.21	1	1	91.8789141	8.49951172
2.21	1	1	63.8629544	5.49072266
1.57	2	1	115.239019	5.52880859
2.83	1	1	70.5302839	6.72705078
0.95	2	1	163.346797	5.66845703
2.53	1	1	78.9750394	5.08447266
3.26	1	1	87.4918392	4.77978516
2.11	2	1	108.768749	6.60986328
3.14	1	1	90.9586372	8.73388672
2.35	3	1	122.39999	5.24951172
1.94	1	1	37.9732977	5.45263672
2.79	2	1	57.4104086	7.79638672
2.01	2	1	86.9261382	7.51806641
2.1	2	1	128.260486	9.31982422
0.68	1	1	164.857544	10.0668945
6.57	2	1	22.9561572	4.14501953
7.46	2	3	54.4627987	5.99853516
1.54	2	2	75.9261642	4.74169922
5.31	2	1	25.6148807	8.86572266
20.73	1	1	9.52059707	8.52880859
3.44	4	1	36.7813095	6.31591797
2.81	2	1	140.27549	7.35693359

Information Classification: General

0.48	1	1	180.12101	5.97314453
1.28	2	1	122.215823	7.50341797
3.14	1	1	80.0578206	5.17333984
1.7	2	1	86.1319391	5.14794922
3.3	2	1	75.0390364	9.39306641
2.12	2	1	71.7327904	6.10009766
0.79	1	1	161.560971	5.85888672
1.26	2	1	95.8832558	5.17333984
0.7	1	1	274.980014	5.08447266
1.12	1	1	77.7486012	7.69384766
19.38	1	1	15.0549871	9.81787109
3.13	1	1	51.3060607	6.85888672
0.55	1	1	313.729352	5.59228516
1.98	2	1	115.454931	6.87353516
3.1	1	1	83.4440559	6.74169922
1.45	3	2	91.5287702	5.79541016
1.7	1	1	112.766245	8.82177734
0.47	1	1	418.564964	9.12939453
6.73	1	1	22.8017	5.59228516
10.96	1	1	36.4206684	9.07080078
2.51	2	1	58.4654956	8.52880859
4.24	2	2	113.107017	5.60498047
1.03	3	1	101.342342	7.50341797
0.68	9	1	214.20489	6.53662109
1.64	1	1	136.37445	8.04541016
20.59	2	1	7.45882206	6.74169922
25.43	1	2	18.9054077	5.24951172
2.5	2	1	58.9636696	9.24658203
2.31	1	1	56.3941621	10.6381836
2.78	2	1	50.8868466	6.34130859
0.36	2	1	290.144585	7.72314453
1.34	2	1	86.4131389	7.98681641
2.36	1	1	47.4798039	8.54345703
2.93	2	1	41.8141332	6.62451172
7.11	1	2	57.2245651	6.02392578
2.08	2	1	85.3752711	7.13720703
1.49	4	1	75.9210277	5.78271484
3.37	2	1	33.1478591	9.83251953
3.43	3	1	85.5249776	4.71630859
14.68	2	1	27.5593035	7.92822266
4.01	1	1	57.9842656	6.71240234
5.67	2	1	37.3980139	6.30322266
1.7	2	1	73.0398929	9.46630859
0.95	1	1	158.939187	7.86962891
10.24	2	1	14.4920752	5.35107422
0.91	5	1	158.403959	7.04931641
2.04	2	1	57.721342	10.0668945
46.34	1	1	4.45408562	4.08154297
20.83	4	1	16.4892655	5.23681641
3.15	6	1	36.0020197	6.53662109
0.66	2	1	103.594099	7.37158203
1.57	4	1	196.812901	4.34814453
0.76	1	1	140.923161	6.78564453
7.37	3	1	24.7823114	6.99072266
4.95	4	1	23.0625632	11.0043945

Information Classification: General

1.77	1	1	169.962969	8.68994141
2.09	3	1	63.1719386	6.60986328
6.11	2	2	53.8454265	8.98291016
2.56	1	1	42.8751835	7.76708984
15.15	1	1	29.8032164	8.06005859
3.25	2	1	50.8130982	9.42236328
0.46	2	1	279.348761	4.71630859
2.89	1	1	42.1256964	9.17333984
1.44	5	1	48.1934477	7.81103516
3.11	2	1	56.5389685	7.09326172
0.83	1	1	164.014454	6.84423828
1.7	2	2	166.214435	7.35693359
1.48	2	1	88.9631885	9.45166016
1.68	2	1	69.9137816	10.315918
4.05	3	1	45.1151812	4.64013672
3.78	3	1	42.0534511	9.53955078
3.82	3	1	48.5517883	9.15869141
4.3	2	1	61.9752769	4.94482422
3.37	2	1	52.8616178	7.97216797
2.67	5	1	37.6640533	10.6235352
2.8	3	1	67.0071211	5.79541016
1.15	2	1	103.797968	7.97216797
24.79	3	1	13.0306084	5.00830078

in database based on protein sequence; Unique Peptides, The num

Information Classification: General
