# Supplementary material for: Salt-induced phosphoproteomic changes in the subfornical organ in rats with chronic kidney disease
Source: Ren Fail. 2023 Jan 30;45(1):2171886. doi: 10.1080/0886022X.2023.2171886 (PMC9888458; doi:10.1080/0886022X.2023.2171886)
Supplement: Supplemental Material [file IRNF_A_2171886_SM9865.zip › 2171886/Copy of Supplementary_Table_S3.pdf]

**the biological processes of differential phosphoproteins in NC/NS comparison**

| | number | percentage |
|---|---|---|
| cellular process | 269 | 0.121280433 |
| single-organism process | 246 | 0.11091073 |
| biological regulation | 223 | 0.100541028 |
| regulation of biological process | 213 | 0.096032462 |
| cellular component organization or biogenesis | 161 | 0.072587917 |
| response to stimulus | 150 | 0.067628494 |
| localization | 144 | 0.064923354 |
| metabolic process | 143 | 0.064472498 |
| multicellular organismal process | 134 | 0.060414788 |
| signaling | 126 | 0.056807935 |
| developmental process | 124 | 0.055906222 |
| positive regulation of biological process | 112 | 0.050495942 |
| negative regulation of biological process | 82 | 0.036970243 |
| locomotion | 43 | 0.019386835 |
| biological adhesion | 24 | 0.010820559 |
| multi-organism process | 24 | 0.010820559 |
| | 2218 | |

**the molecular function of differential phosphoproteins in NC/NS comparison**

| | number | percentage |
|---|---|---|
| binding | 262 | 0.56223176 |
| catalytic activity | 83 | 0.178111588 |
| molecular function regulator | 35 | 0.075107296 |
| transporter activity | 31 | 0.066523605 |
| structural molecule activity | 18 | 0.038626609 |
| signal transducer activity | 13 | 0.027896996 |
| molecular transducer activity | 11 | 0.02360515 |
| transcription factor activity, protein binding | 7 | 0.015021459 |
| nucleic acid binding transcription factor activity | 5 | 0.010729614 |
| translation regulator activity | 1 | 0.002145923 |
| | 466 | |

Information Classification: General

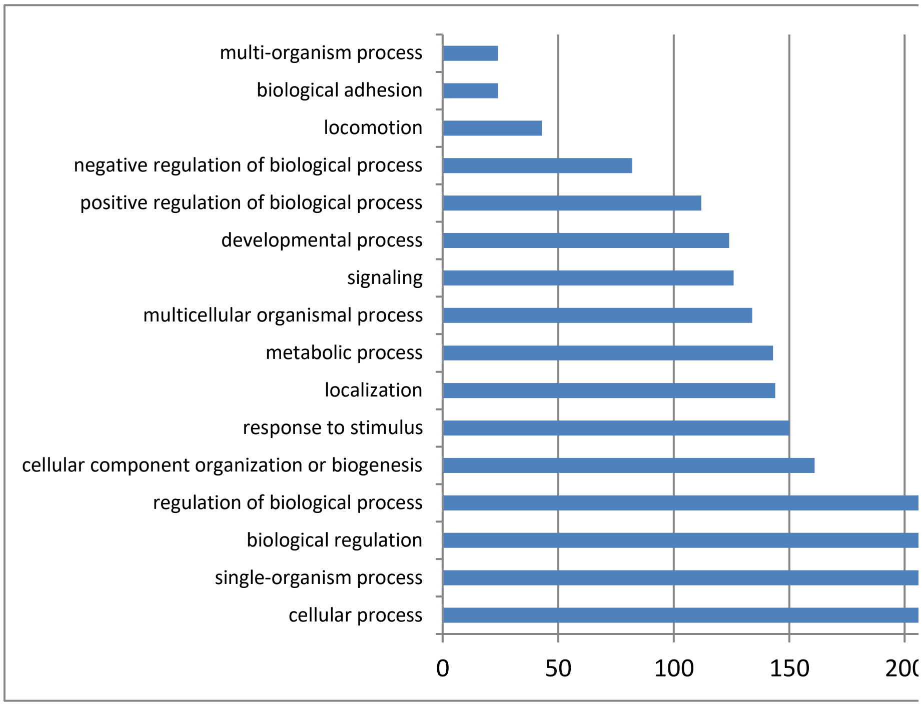
multi-organism process
biological adhesion
locomotion
negative regulation of biological process
positive regulation of biological process
developmental process
signaling
multicellular organismal process
metabolic process
localization
response to stimulus
cellular component organization or biogenesis
regulation of biological process
biological regulation
single-organism process
cellular process
0
50
100
150

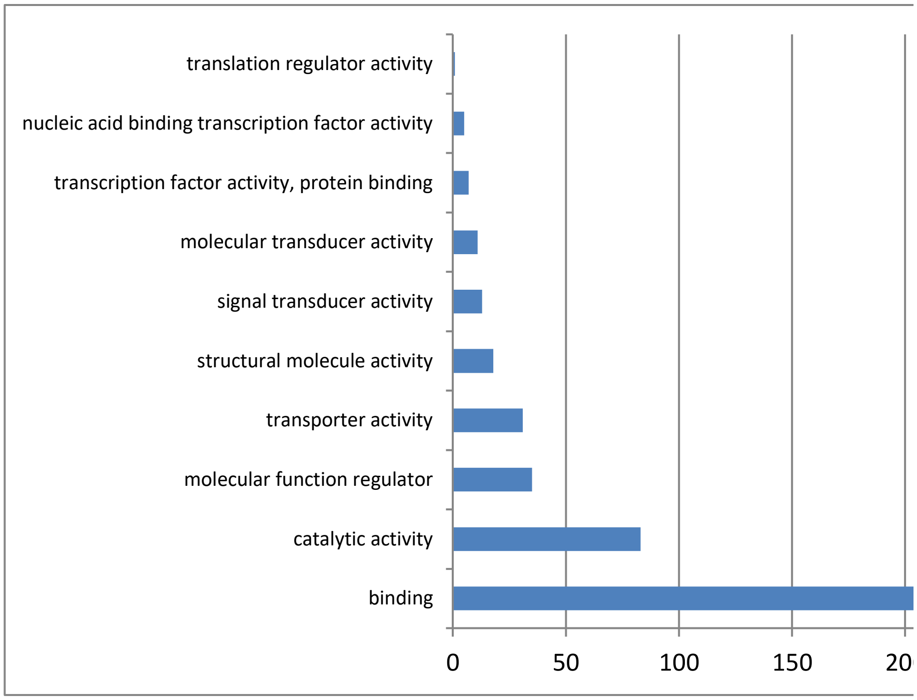
translation regulator activity
nucleic acid binding transcription factor activity
transcription factor activity, protein binding
molecular transducer activity
signal transducer activity
structural molecule activity
transporter activity
molecular function regulator
catalytic activity
binding
0
50
100
150

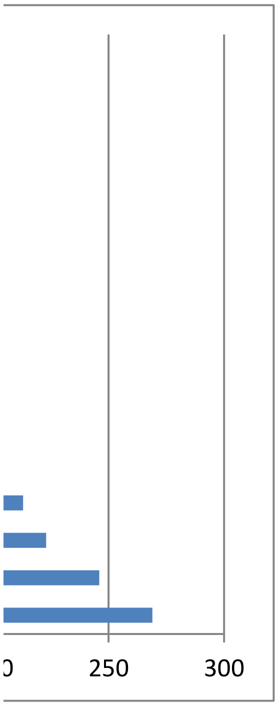


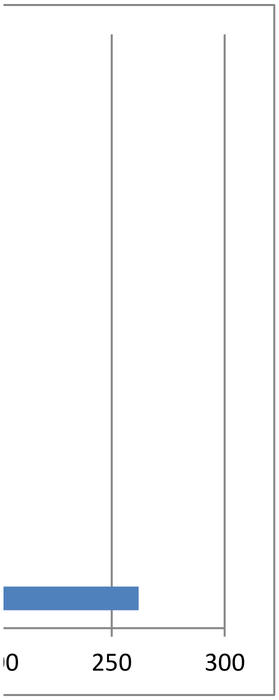


Information Classification: General
