# Supplementary material for: Salt-induced phosphoproteomic changes in the subfornical organ in rats with chronic kidney disease
Source: Ren Fail. 2023 Jan 30;45(1):2171886. doi: 10.1080/0886022X.2023.2171886 (PMC9888458; doi:10.1080/0886022X.2023.2171886)
Supplement: Supplemental Material [file IRNF_A_2171886_SM9865.zip › 2171886/Copy of Supplementary_Table_S4.pdf]

Level	GO_ID	Term
1	GO:0005575	cellular_component
2	GO:0005623	cell
2	GO:0044456	synapse part
2	GO:0044425	membrane part
2	GO:0009295	nucleoid
2	GO:0005576	extracellular region
2	GO:0031974	membrane-enclosed lumen
2	GO:0030054	cell junction
2	GO:0044421	extracellular region part
2	GO:0099080	supramolecular complex
2	GO:0016020	membrane
2	GO:0045202	synapse
2	GO:0032991	macromolecular complex
2	GO:0044422	organelle part
2	GO:0043226	organelle
2	GO:0044464	cell part
3	GO:0098590	plasma membrane region
3	GO:0044459	plasma membrane part
3	GO:0048475	coated membrane
3	GO:0031975	envelope
3	GO:0097223	sperm part
3	GO:0043229	intracellular organelle
3	GO:0043230	extracellular organelle
3	GO:0044463	cell projection part
3	GO:0098794	postsynapse
3	GO:0044441	ciliary part
3	GO:0043233	organelle lumen
3	GO:0044455	mitochondrial membrane part
3	GO:0044449	contractile fiber part
3	GO:0060076	excitatory synapse
3	GO:0031090	organelle membrane
3	GO:0032155	cell division site part
3	GO:0045177	apical part of cell
3	GO:0098796	membrane protein complex
3	GO:0012505	endomembrane system
3	GO:0044420	extracellular matrix component
3	GO:0031224	intrinsic component of membrane
3	GO:0097458	neuron part
3	GO:0099572	postsynaptic specialization
3	GO:0005789	endoplasmic reticulum membrane
3	GO:0044297	cell body
3	GO:0000785	chromatin
3	GO:0044424	intracellular part
3	GO:0005886	plasma membrane
3	GO:0031252	cell leading edge
3	GO:0048786	presynaptic active zone

Information Classification: General

3	GO:0005615	extracellular space
3	GO:0098793	presynapse
3	GO:0009986	cell surface
3	GO:0035748	myelin sheath abaxonal region
3	GO:0043209	myelin sheath
3	GO:1903561	extracellular vesicle
3	GO:0030427	site of polarized growth
3	GO:0043220	Schmidt-Lanterman incisure
3	GO:0030055	cell-substrate junction
3	GO:0042175	nuclear outer membrane-endoplasmic reticulum membrane network
3	GO:1905354	exoribonuclease complex
3	GO:0005905	clathrin-coated pit
3	GO:0071944	cell periphery
3	GO:0098552	side of membrane
3	GO:0032426	stereocilium tip
3	GO:0042645	mitochondrial nucleoid
3	GO:0099081	supramolecular polymer
3	GO:0070938	contractile ring
3	GO:0032420	stereocilium
3	GO:0030672	synaptic vesicle membrane
3	GO:0005911	cell-cell junction
3	GO:0042995	cell projection
3	GO:0032153	cell division site
3	GO:0008287	protein serine/threonine phosphatase complex
3	GO:0030904	retromer complex
3	GO:0043227	membrane-bounded organelle
3	GO:0090637	inner dense plaque of desmosome
3	GO:0043218	compact myelin
3	GO:0008021	synaptic vesicle
3	GO:0070161	anchoring junction
3	GO:0031300	intrinsic component of organelle membrane
3	GO:0044232	organelle membrane contact site
3	GO:0019867	outer membrane
3	GO:0043219	lateral loop
3	GO:0030496	midbody
3	GO:0043234	protein complex
3	GO:0031594	neuromuscular junction
3	GO:0097060	synaptic membrane
3	GO:0045178	basal part of cell
3	GO:0098862	cluster of actin-based cell projections
3	GO:1990904	ribonucleoprotein complex
3	GO:0005622	intracellular
3	GO:0031012	extracellular matrix
3	GO:0090636	outer dense plaque of desmosome
3	GO:0098805	whole membrane
3	GO:0072562	blood microparticle
3	GO:0098589	membrane region

Information Classification: General

3	GO:0005929	cilium
3	GO:0097610	cell surface furrow
3	GO:0060077	inhibitory synapse
3	GO:0070062	extracellular exosome
3	GO:0019898	extrinsic component of membrane
3	GO:0044446	intracellular organelle part
3	GO:0043228	non-membrane-bounded organelle
4	GO:0005835	fatty acid synthase complex
4	GO:0031430	M band
4	GO:0031301	integral component of organelle membrane
4	GO:0030141	secretory granule
4	GO:0031967	organelle envelope
4	GO:0044433	cytoplasmic vesicle part
4	GO:0031982	vesicle
4	GO:0016459	myosin complex
4	GO:0032433	filopodium tip
4	GO:0031984	organelle subcompartment
4	GO:0005913	cell-cell adherens junction
4	GO:0000792	heterochromatin
4	GO:0097418	neurofibrillary tangle
4	GO:0044438	microbody part
4	GO:0043231	intracellular membrane-bounded organelle
4	GO:0098588	bounding membrane of organelle
4	GO:0005635	nuclear envelope
4	GO:1990391	DNA repair complex
4	GO:0045211	postsynaptic membrane
4	GO:0030666	endocytic vesicle membrane
4	GO:0005826	actomyosin contractile ring
4	GO:0005903	brush border
4	GO:0044431	Golgi apparatus part
4	GO:0005838	proteasome regulatory particle
4	GO:0033017	sarcoplasmic reticulum membrane
4	GO:0032838	cell projection cytoplasm
4	GO:0035145	exon-exon junction complex
4	GO:0060170	ciliary membrane
4	GO:0098797	plasma membrane protein complex
4	GO:0009898	cytoplasmic side of plasma membrane
4	GO:0000790	nuclear chromatin
4	GO:0043005	neuron projection
4	GO:0031256	leading edge membrane
4	GO:0097708	intracellular vesicle
4	GO:0031082	BLOC complex
4	GO:0035327	transcriptionally active chromatin
4	GO:0016021	integral component of membrane
4	GO:0005783	endoplasmic reticulum
4	GO:0072372	primary cilium
4	GO:0043204	perikaryon

Information Classification: General

4	GO:0030018	Z disc
4	GO:0001726	ruffle
4	GO:0070160	occluding junction
4	GO:0030868	smooth endoplasmic reticulum membrane
4	GO:0032809	neuronal cell body membrane
4	GO:0044427	chromosomal part
4	GO:0031674	I band
4	GO:0030315	T-tubule
4	GO:0005794	Golgi apparatus
4	GO:0005768	endosome
4	GO:0016323	basolateral plasma membrane
4	GO:0016324	apical plasma membrane
4	GO:0042383	sarcolemma
4	GO:0042734	presynaptic membrane
4	GO:0034045	pre-autophagosomal structure membrane
4	GO:0031968	organelle outer membrane
4	GO:0005774	vacuolar membrane
4	GO:0005938	cell cortex
4	GO:0030119	AP-type membrane coat adaptor complex
4	GO:0044429	mitochondrial part
4	GO:0030057	desmosome
4	GO:0045259	proton-transporting ATP synthase complex
4	GO:0032154	cleavage furrow
4	GO:0032589	neuron projection membrane
4	GO:0022624	proteasome accessory complex
4	GO:0032839	dendrite cytoplasm
4	GO:1990730	VCP-NSFL1C complex
4	GO:0005667	transcription factor complex
4	GO:0005875	microtubule associated complex
4	GO:0030176	integral component of endoplasmic reticulum membrane
4	GO:0097228	sperm principal piece
4	GO:0032421	stereocilium bundle
4	GO:0043235	receptor complex
4	GO:0031312	extrinsic component of organelle membrane
4	GO:0030658	transport vesicle membrane
4	GO:0044444	cytoplasmic part
4	GO:0001520	outer dense fiber
4	GO:0034993	LINC complex
4	GO:0005952	cAMP-dependent protein kinase complex
4	GO:0044428	nuclear part
4	GO:0045298	tubulin complex
4	GO:0031931	TORC1 complex
4	GO:0072379	ER membrane insertion complex
4	GO:0000502	proteasome complex
4	GO:0044853	plasma membrane raft
4	GO:0030027	lamellipodium
4	GO:0030133	transport vesicle

Information Classification: General

4	GO:0044298	cell body membrane
4	GO:0036126	sperm flagellum
4	GO:0043296	apical junction complex
4	GO:0033010	paranodal junction
4	GO:0014069	postsynaptic density
4	GO:0034750	Scrib-APC-beta-catenin complex
4	GO:0036064	ciliary basal body
4	GO:0030132	clathrin coat of coated pit
4	GO:0031227	intrinsic component of endoplasmic reticulum membrane
4	GO:0020016	ciliary pocket
4	GO:0033267	axon part
4	GO:0099512	supramolecular fiber
4	GO:0098800	inner mitochondrial membrane protein complex
4	GO:0090665	glycoprotein complex
4	GO:1990351	transporter complex
4	GO:0098636	protein complex involved in cell adhesion
4	GO:0012506	vesicle membrane
4	GO:0036038	MKS complex
4	GO:0031514	motile cilium
4	GO:0019866	organelle inner membrane
4	GO:0044437	vacuolar part
4	GO:0097539	ciliary transition fiber
4	GO:0030426	growth cone
4	GO:0038201	TOR complex
4	GO:0016592	mediator complex
4	GO:0030529	intracellular ribonucleoprotein complex
4	GO:0098857	membrane microdomain
4	GO:0031965	nuclear membrane
4	GO:0035869	ciliary transition zone
4	GO:0043198	dendritic shaft
4	GO:0005578	proteinaceous extracellular matrix
4	GO:0031672	A band
4	GO:0097225	sperm midpiece
4	GO:0043197	dendritic spine
4	GO:0005887	integral component of plasma membrane
4	GO:0043232	intracellular non-membrane-bounded organelle
4	GO:0043025	neuronal cell body
4	GO:0005912	adherens junction
4	GO:0000178	exosome (RNase complex)
4	GO:0098802	plasma membrane receptor complex
4	GO:0016234	inclusion body
4	GO:0046930	pore complex
4	GO:0044430	cytoskeletal part
4	GO:0030660	Golgi-associated vesicle membrane
4	GO:0098562	cytoplasmic side of membrane
4	GO:0001750	photoreceptor outer segment
4	GO:0097386	glial cell projection

Information Classification: General

4	GO:0009897	external side of plasma membrane
4	GO:0098858	actin-based cell projection
4	GO:0044609	DBIRD complex
4	GO:0005921	gap junction
4	GO:0032437	cuticular plate
4	GO:0030662	coated vesicle membrane
4	GO:0019897	extrinsic component of plasma membrane
4	GO:0098798	mitochondrial protein complex
4	GO:0031966	mitochondrial membrane
4	GO:0043195	terminal bouton
4	GO:0044291	cell-cell contact zone
4	GO:0043034	costamere
4	GO:0002102	podosome
4	GO:0016469	proton-transporting two-sector ATPase complex
4	GO:0005924	cell-substrate adherens junction
4	GO:0016328	lateral plasma membrane
4	GO:0044432	endoplasmic reticulum part
4	GO:0031523	Myb complex
4	GO:0071203	WASH complex
4	GO:0097224	sperm connecting piece
4	GO:0009925	basal plasma membrane
4	GO:0044306	neuron projection terminus
4	GO:0001533	cornified envelope
4	GO:0032584	growth cone membrane
4	GO:0030117	membrane coat
4	GO:0030017	sarcomere
4	GO:0031903	microbody membrane
4	GO:1902494	catalytic complex
4	GO:0031095	platelet dense tubular network membrane
4	GO:0005604	basement membrane
4	GO:0070013	intracellular organelle lumen
4	GO:0031226	intrinsic component of plasma membrane
4	GO:0036477	somatodendritic compartment
4	GO:0031234	extrinsic component of cytoplasmic side of plasma membrane
4	GO:0031253	cell projection membrane
4	GO:0061574	ASAP complex
4	GO:0008541	proteasome regulatory particle, lid subcomplex
4	GO:0005737	cytoplasm
4	GO:1902495	transmembrane transporter complex
5	GO:0005914	spot adherens junction
5	GO:0005902	microvillus
5	GO:0032587	ruffle membrane
5	GO:1905368	peptidase complex
5	GO:0031094	platelet dense tubular network
5	GO:0055037	recycling endosome
5	GO:0016010	dystrophin-associated glycoprotein complex
5	GO:0016460	myosin II complex

5	GO:0005743	mitochondrial inner membrane
5	GO:0044224	juxtaparanode region of axon
5	GO:0005901	caveola
5	GO:0097038	perinuclear endoplasmic reticulum
5	GO:0000306	extrinsic component of vacuolar membrane
5	GO:0005741	mitochondrial outer membrane
5	GO:0005770	late endosome
5	GO:0043292	contractile fiber
5	GO:0008305	integrin complex
5	GO:0031527	filopodium membrane
5	GO:0036464	cytoplasmic ribonucleoprotein granule
5	GO:0005795	Golgi stack
5	GO:0030175	filopodium
5	GO:0097425	smooth endoplasmic reticulum part
5	GO:0005681	spliceosomal complex
5	GO:0030864	cortical actin cytoskeleton
5	GO:0008352	katanin complex
5	GO:0005790	smooth endoplasmic reticulum
5	GO:0030118	clathrin coat
5	GO:0005740	mitochondrial envelope
5	GO:1904115	axon cytoplasm
5	GO:0045495	pole plasm
5	GO:0016363	nuclear matrix
5	GO:0005925	focal adhesion
5	GO:0005813	centrosome
5	GO:0031235	intrinsic component of the cytoplasmic side of the plasma membrane
5	GO:0010008	endosome membrane
5	GO:0034399	nuclear periphery
5	GO:0031091	platelet alpha granule
5	GO:0048471	perinuclear region of cytoplasm
5	GO:0097449	astrocyte projection
5	GO:0044454	nuclear chromosome part
5	GO:0005883	neurofilament
5	GO:0005814	centriole
5	GO:0005720	nuclear heterochromatin
5	GO:0031045	dense core granule
5	GO:0031981	nuclear lumen
5	GO:0098878	neurotransmitter receptor complex
5	GO:0005697	telomerase holoenzyme complex
5	GO:0032541	cortical endoplasmic reticulum
5	GO:0043194	axon initial segment
5	GO:0030286	dynein complex
5	GO:0005856	cytoskeleton
5	GO:1903293	phosphatase complex
5	GO:0005922	connexin complex
5	GO:0098574	cytoplasmic side of lysosomal membrane
5	GO:0016529	sarcoplasmic reticulum

5	GO:0005634	nucleus
5	GO:0099501	exocytic vesicle membrane
5	GO:0044448	cell cortex part
5	GO:0000228	nuclear chromosome
5	GO:0030131	clathrin adaptor complex
5	GO:0005915	zonula adherens
5	GO:0005759	mitochondrial matrix
5	GO:0098533	ATPase dependent transmembrane transport complex
5	GO:0044309	neuron spine
5	GO:0001518	voltage-gated sodium channel complex
5	GO:0033269	internode region of axon
5	GO:0030424	axon
5	GO:0098687	chromosomal region
5	GO:0000118	histone deacetylase complex
5	GO:0000407	pre-autophagosomal structure
5	GO:0005916	fascia adherens
5	GO:0031513	nonmotile primary cilium
5	GO:0005815	microtubule organizing center
5	GO:0005793	endoplasmic reticulum-Golgi intermediate compartment
5	GO:0000922	spindle pole
5	GO:0044304	main axon
5	GO:0032432	actin filament bundle
5	GO:0044439	peroxisomal part
5	GO:0042641	actomyosin
5	GO:0031988	membrane-bounded vesicle
5	GO:0044452	nucleolar part
5	GO:0005844	polysome
5	GO:0000109	nucleotide-excision repair complex
5	GO:0005840	ribosome
5	GO:0005891	voltage-gated calcium channel complex
5	GO:0034702	ion channel complex
5	GO:0030122	AP-2 adaptor complex
5	GO:0000139	Golgi membrane
5	GO:0098827	endoplasmic reticulum subcompartment
5	GO:0044445	cytosolic part
5	GO:0016023	cytoplasmic, membrane-bounded vesicle
5	GO:0005732	small nucleolar ribonucleoprotein complex
5	GO:0000177	cytoplasmic exosome (RNase complex)
5	GO:0005829	cytosol
5	GO:0070382	exocytic vesicle
5	GO:0099513	polymeric cytoskeletal fiber
5	GO:0005694	chromosome
5	GO:0071782	endoplasmic reticulum tubular network
5	GO:0030673	axolemma
5	GO:1904949	ATPase complex
5	GO:0044295	axonal growth cone
5	GO:0005753	mitochondrial proton-transporting ATP synthase complex

Information Classification: General

5	GO:0090498	extrinsic component of Golgi membrane
5	GO:0099568	cytoplasmic region
5	GO:0005654	nucleoplasm
5	GO:0098791	Golgi subcompartment
5	GO:0005819	spindle
5	GO:0005871	kinesin complex
5	GO:0016342	catenin complex
5	GO:0030532	small nuclear ribonucleoprotein complex
5	GO:0005773	vacuole
5	GO:0035770	ribonucleoprotein granule
5	GO:0016528	sarcoplasm
5	GO:0032982	myosin filament
5	GO:0014704	intercalated disc
5	GO:0098852	lytic vacuole membrane
5	GO:0030669	clathrin-coated endocytic vesicle membrane
5	GO:0031528	microvillus membrane
5	GO:0033268	node of Ranvier
5	GO:0005798	Golgi-associated vesicle
5	GO:0031083	BLOC-1 complex
5	GO:0005890	sodium:potassium-exchanging ATPase complex
5	GO:0005881	cytoplasmic microtubule
5	GO:0044451	nucleoplasm part
5	GO:0030120	vesicle coat
5	GO:0034992	microtubule organizing center attachment site
5	GO:0008091	spectrin
5	GO:0000421	autophagosome membrane
5	GO:0005652	nuclear lamina
5	GO:0005778	peroxisomal membrane
5	GO:0005739	mitochondrion
5	GO:0005923	bicellular tight junction
5	GO:1990234	transferase complex
5	GO:0005769	early endosome
5	GO:0043679	axon terminus
5	GO:0042579	microbody
5	GO:0071818	BAT3 complex
5	GO:0030665	clathrin-coated vesicle membrane
5	GO:0031410	cytoplasmic vesicle
5	GO:0005802	trans-Golgi network
5	GO:0045121	membrane raft
5	GO:0031526	brush border membrane
5	GO:0020018	ciliary pocket membrane
5	GO:0016600	flotillin complex
5	GO:0033270	paranode region of axon
5	GO:0097648	G-protein coupled receptor complex
5	GO:0017071	intracellular cyclic nucleotide activated cation channel complex
5	GO:0005730	nucleolus
5	GO:0044450	microtubule organizing center part

Information Classification: General

5	GO:0030659	cytoplasmic vesicle membrane
5	GO:0030425	dendrite
5	GO:0008076	voltage-gated potassium channel complex
5	GO:0072588	box H/ACA RNP complex
5	GO:0044440	endosomal part
5	GO:0008328	ionotropic glutamate receptor complex
6	GO:0014731	spectrin-associated cytoskeleton
6	GO:0030135	coated vesicle
6	GO:0005776	autophagosome
6	GO:1990357	terminal web
6	GO:0097517	contractile actin filament bundle
6	GO:0005882	intermediate filament
6	GO:0034703	cation channel complex
6	GO:0005884	actin filament
6	GO:0097431	mitotic spindle pole
6	GO:0000932	cytoplasmic mRNA processing body
6	GO:0048770	pigment granule
6	GO:0031429	box H/ACA snoRNP complex
6	GO:0015629	actin cytoskeleton
6	GO:0090533	cation-transporting ATPase complex
6	GO:0097450	astrocyte end-foot
6	GO:0072687	meiotic spindle
6	GO:0043186	P granule
6	GO:0071010	prespliceosome
6	GO:1905369	endopeptidase complex
6	GO:0005765	lysosomal membrane
6	GO:0038037	G-protein coupled receptor dimeric complex
6	GO:0030016	myofibril
6	GO:0097513	myosin II filament
6	GO:0005874	microtubule
6	GO:0030139	endocytic vesicle
6	GO:0001725	stress fiber
6	GO:0031313	extrinsic component of endosome membrane
6	GO:0030863	cortical cytoskeleton
6	GO:0032391	photoreceptor connecting cilium
6	GO:0015630	microtubule cytoskeleton
6	GO:0097422	tubular endosome
6	GO:0071942	XPC complex
6	GO:0030137	COPI-coated vesicle
6	GO:0032281	AMPA glutamate receptor complex
6	GO:0031985	Golgi cisterna
6	GO:0072686	mitotic spindle
6	GO:0005777	peroxisome
6	GO:0005684	U2-type spliceosomal complex
6	GO:0090661	box H/ACA telomerase RNP complex
6	GO:0071013	catalytic step 2 spliceosome
6	GO:0030125	clathrin vesicle coat

Information Classification: General

6	GO:0044300	cerebellar mossy fiber
6	GO:0055038	recycling endosome membrane
6	GO:0000781	chromosome, telomeric region
6	GO:0099503	secretory vesicle
6	GO:0034707	chloride channel complex
6	GO:0016604	nuclear body
6	GO:0000323	lytic vacuole
6	GO:0005689	U12-type spliceosomal complex
6	GO:0061695	transferase complex, transferring phosphorus-containing groups
6	GO:0031901	early endosome membrane
6	GO:0097525	spliceosomal snRNP complex
6	GO:0005945	6-phosphofructokinase complex
6	GO:0045111	intermediate filament cytoskeleton
6	GO:0071011	precatalytic spliceosome
6	GO:0031902	late endosome membrane
6	GO:0060293	germ plasm
6	GO:0030128	clathrin coat of endocytic vesicle
7	GO:1902911	protein kinase complex
7	GO:0005764	lysosome
7	GO:0034705	potassium channel complex
7	GO:0030136	clathrin-coated vesicle
7	GO:0015030	Cajal body
7	GO:0034704	calcium channel complex
7	GO:0042470	melanosome
7	GO:0000138	Golgi trans cisterna
7	GO:0034706	sodium channel complex
7	GO:0016607	nuclear speck
7	GO:0005682	U5 snRNP
7	GO:0016605	PML body
7	GO:0038039	G-protein coupled receptor heterodimeric complex
7	GO:0005685	U1 snRNP
7	GO:0005797	Golgi medial cisterna
7	GO:0005686	U2 snRNP
7	GO:0071004	U2-type prespliceosome
7	GO:0045334	clathrin-coated endocytic vesicle
8	GO:1902554	serine/threonine protein kinase complex

Type	Seqs_Num	Sequences
Cellular Component	216	A0A096MJT2, F1M031, F1LRZ7, Q566D6, G3V8J0, D4A0C3, D4A702,
Cellular Component	206	A0A096MJT2, F1M031, F1LRZ7, Q566D6, G3V8J0, D4A0C3, D4A702,
Cellular Component	31	F1LYA6, G3V9N7, Q5BKB9, G3V7J3, D4A8M2, M0R423, G3V6L7, Q0
Cellular Component	91	P57769, Q9P290, Q9Z2L0, F1LMT5, A0A096MJT2, P70580, A0A0G2K
Cellular Component	1	Q9Z2L0
Cellular Component	52	Q9Z2L0, P09606, P70580, P82808, F1LST1, P0C6C0, Q9R1N0, P2509
Cellular Component	41	A0A0U1RRX4, Q9Z2L0, F1LM55, P70580, G3V9N7, Q5BKB9, F1LW91
Cellular Component	49	A0A0G2K6R9, Q5BKB9, D4A702, P15205, P31016, A0A0G2K6Z8, P3
Cellular Component	51	Q9Z2L0, P09606, P70580, P82808, F1LST1, P0C6C0, Q9R1N0, P2509
Cellular Component	27	P12839, A0A0U1RRX4, F1LMV6, D4A1Q2, P34926, Q5BKB9, F1LRZ7,
Cellular Component	144	Q9P290, Q9Z2L0, A0A096MJT2, A0A0G2K6R9, F1M6X3, Q5BKB9, F1
Cellular Component	39	F1LYA6, Q5BKB9, G3V9N7, D3ZCL8, G3V7J3, D4A8M2, M0R423, G3V
Cellular Component	83	Q9Z2L0, F1LMT5, D4A1Q2, P34926, Q5BKB9, F1M6X3, A0A0G2JX30
Cellular Component	121	Q9P290, Q9Z2L0, A0A0G2K6R9, Q5BKB9, F1LRZ7, Q566D6, A0A0G2
Cellular Component	177	Q9P290, Q9Z2L0, F1M031, A0A096MJT2, A0A0G2K6R9, Q5BKB9, F1
Cellular Component	206	A0A096MJT2, F1M031, F1LRZ7, Q566D6, G3V8J0, D4A0C3, D4A702,
Cellular Component	36	Q62866, F1LMV6, Q5BKB9, A0A0G2K6R9, F1M6X3, D4A8M2, G3V7J
Cellular Component	56	Q9P290, A0A096MJT2, Q5BKB9, A0A0G2K6R9, F1M6X3, Q9R1N0, P
Cellular Component	2	D3ZUY8, F1M1Y0
Cellular Component	6	Q9Z2L0, D3Z9R8, G3V9N1, D3ZF03, A0A0G2JXN8, Q8VHJ9
Cellular Component	4	P39069, Q52KS1, B0BMV7, Q9JJ19
Cellular Component	165	Q9Z2L0, Q9P290, F1M031, A0A096MJT2, A0A0G2K6R9, Q5BKB9, F1
Cellular Component	42	Q9Z2L0, P09606, P70580, F1LMV6, G3V9N7, P82808, F1LW91, P0C
Cellular Component	51	D4A1Q2, Q5BKB9, A0A0G2K6R9, P15205, P31016, Q9JJ19, P39069,
Cellular Component	17	Q63622, P07936, Q5BKB9, F1M287, G3V7J3, M0R423, D3ZWS0, Q6
Cellular Component	9	P39069, Q52KS1, F1M9F9, G3V9W0, Q4V8H8, D4A8M2, B0BMV7, D
Cellular Component	41	A0A0U1RRX4, Q9Z2L0, F1LM55, P70580, G3V9N7, Q5BKB9, F1LW91
Cellular Component	1	D3Z9R8
Cellular Component	9	P38652, P85972, A0A0G2K6R9, G3V6S0, A0A0G2K207, A0A0G2K1R
Cellular Component	9	Q63622, Q9JKS6, P07936, G3V984, Q5BKB9, F1LRL9, P15205, D4A7
Cellular Component	31	P57769, Q9Z2L0, Q9P290, F1LMT5, P70580, F1LW91, D4A8M2, A0A
Cellular Component	2	E9PTU4, G3V9Y1
Cellular Component	8	Q52KS1, P08050, Q62866, F1LW91, Q9R1N0, F1MA36, F1LPM3, Q9
Cellular Component	28	Q9Z2L0, F1LMT5, F1M6X3, G3V6L7, Q8VHW9, Q9JJ19, P31016, E9P
Cellular Component	54	P57769, P70580, F1M6X3, F1M110, A0A0G2JX77, G3V8J0, A0A0G2J
Cellular Component	2	F1LST1, D4A8M2
Cellular Component	54	Q9P290, Q9Z2L0, F1LMT5, P70580, A0A0G2K6N2, F1M6X3, Q9R1N
Cellular Component	62	D4A1Q2, Q5BKB9, A0A0G2K6R9, F1LRZ7, Q63330, P15205, D4A702,
Cellular Component	9	Q63622, Q9JKS6, P07936, G3V984, Q5BKB9, F1LRL9, P15205, D4A7
Cellular Component	6	P08050, P0C5X8, G3V9G3, P70580, Q6RJR6, D3ZJ32
Cellular Component	13	Q63622, F1M241, A0A0G2JWM2, A0A0G2K8P5, A0A1B0GWY5, G3V
Cellular Component	6	D4A853, P61980, F1LM55, A0A0G2K9F7, A0A0G2JWM2, Q566D6
Cellular Component	188	Q9Z2L0, Q9P290, F1M031, A0A096MJT2, A0A0G2K6R9, Q5BKB9, F1
Cellular Component	94	Q9P290, Q9Z2L0, A0A096MJT2, D4A1Q2, Q5BKB9, A0A0G2K6R9, F1
Cellular Component	17	A0A0G2K8P5, A0A1B0GWY5, G3V6L7, A0A0G2K3N1, P31000, D3ZW
Cellular Component	5	Q9JKS6, G3V733, G3V984, Q02563, A0A0G2KAV8

Information Classification: General

Cellular Component	9	F1M3W5, F1LST1, G3V7X2, F1MA36, F1LM19, D3ZL75, P13233, Q4\
Cellular Component	18	F1LYA6, G3V9N7, F1M3W5, D4A8M2, G3V6L7, Q02563, D3ZWS0, Q
Cellular Component	7	E9PTU4, G3V984, A0A0G2K6R9, P48303, G3V7J3, G3V9Y1, A0A0G2I
Cellular Component	1	D3ZWS0
Cellular Component	13	Q9Z2L0, P09606, Q63327, A0A0G2JWM2, F1LRZ7, D3ZWS0, Q6IRG7
Cellular Component	42	Q9Z2L0, P09606, P70580, F1LMV6, G3V9N7, P82808, F1LW91, P0C6
Cellular Component	11	Q63622, P07936, D4A1Q2, A0A0G2JWM2, A0A0G2K8P5, P28818, G
Cellular Component	1	A0A0G2JWM2
Cellular Component	12	A0A1B0GWY5, D4A8M2, P31000, D4A702, C0JPT7, G3V913, P0805(
Cellular Component	6	P08050, P0C5X8, P70580, G3V9G3, Q6RJR6, D3ZJ32
Cellular Component	1	Q9WU49
Cellular Component	2	G3V8J0, F1M1Y0
Cellular Component	97	Q9P290, Q9Z2L0, A0A096MJT2, Q5BKB9, A0A0G2K6R9, F1M6X3, A(
Cellular Component	10	P38652, A0A0G2K2E3, A0A096MJT2, D3ZCL8, P47819, P48303, M0I
Cellular Component	1	Q9JJ19
Cellular Component	1	Q9Z2L0
Cellular Component	27	P12839, A0A0U1RRX4, F1LMV6, D4A1Q2, P34926, Q5BKB9, F1LRZ7,
Cellular Component	2	E9PTU4, G3V9Y1
Cellular Component	1	Q9JJ19
Cellular Component	1	G3V733
Cellular Component	23	F1M9F9, A0A0G2JWM2, A0A0G2K6R9, A0A1B0GWY5, D4A8M2, A0.
Cellular Component	67	P09606, D4A1Q2, Q5BKB9, A0A0G2K6R9, F1LRZ7, P15205, Q9JJ19, I
Cellular Component	2	E9PTU4, G3V9Y1
Cellular Component	1	G3V9N1
Cellular Component	1	G3V8A5
Cellular Component	151	Q9P290, Q9Z2L0, Q5BKB9, F1LST1, F1M6X3, P82808, F1LRZ7, Q566I
Cellular Component	1	P85972
Cellular Component	1	A0A0G2JWM2
Cellular Component	3	G3V733, F1M3W5, P31016
Cellular Component	15	F1M9F9, A0A1B0GWY5, D4A8M2, P31000, D3ZWS0, D4A702, C0JPT
Cellular Component	1	Q9P290
Cellular Component	1	D3ZJ32
Cellular Component	2	Q9Z2L0, G3V9N1
Cellular Component	1	A0A0G2JWM2
Cellular Component	2	A0A0G2JWM2, G3V9Y1
Cellular Component	73	Q9Z2L0, F1LMT5, D4A1Q2, P34926, Q5BKB9, F1M6X3, A0A0G2JX30
Cellular Component	2	G3V9Y1, Q02563
Cellular Component	12	Q63622, Q5BKB9, G3V7J3, D4A8M2, M0R423, G3V6L7, D3ZWS0, Q(
Cellular Component	2	A0A0G2K1R9, Q6IRG7
Cellular Component	3	P85972, G3V9Y1, Q9JJ19
Cellular Component	14	D3ZMS1, F1M062, Q8K3P4, F1LM55, A0A0G2KB60, A0A0G2K700, D
Cellular Component	191	Q9Z2L0, Q9P290, F1M031, A0A096MJT2, A0A0G2K6R9, Q5BKB9, F1
Cellular Component	2	F1LST1, D4A8M2
Cellular Component	1	P85972
Cellular Component	24	P57769, Q9P290, Q9Z2L0, F1LMT5, F1M241, Q4V8H8, G3V6L7, G3V
Cellular Component	2	F1LM19, Q4V8H5
Cellular Component	10	P08050, Q9Z2L0, A0A0G2JYF7, Q9Z1H9, P85972, Q4V8H8, P48303,

Information Classification: General

Cellular Component	11	Q52KS1, P39069, F1M9F9, G3V9W0, F1LRL9, Q4V8H8, D4A8M2, B0
Cellular Component	2	E9PTU4, G3V9Y1
Cellular Component	1	M0R423
Cellular Component	42	Q9Z2L0, P09606, P70580, F1LMV6, G3V9N7, P82808, F1LW91, P0C6
Cellular Component	12	P57769, A0A0G2K2E3, A0A0G2JYF7, G3V874, P85972, F1M110, Q4V
Cellular Component	116	Q9Z2L0, Q9P290, A0A0G2K6R9, Q5BKB9, F1LRZ7, Q566D6, A0A0G2
Cellular Component	91	Q9Z2L0, D4A1Q2, F1M031, A0A096MJT2, P70580, A0A0G2K6R9, P3
Cellular Component	1	G3V6R7
Cellular Component	1	G3V6S0
Cellular Component	1	Q9P290
Cellular Component	3	Q63327, P61765, G3V7X2
Cellular Component	6	Q9Z2L0, D3Z9R8, G3V9N1, D3ZF03, A0A0G2JXN8, Q8VHJ9
Cellular Component	7	P08050, Q63092, G3V733, F1M241, Q9Z0W5, F1M4A4, F1M1Y0
Cellular Component	63	Q9Z2L0, P09606, P70580, P82808, P0C6C0, Q9R1N0, P25093, G3V8
Cellular Component	3	E9PTU4, G3V9Y1, Q99N97
Cellular Component	2	P0C5X8, D3ZUY8
Cellular Component	4	Q5FWU3, A0A0G2K3N1, D4A0C3, A0A0G2JXN8
Cellular Component	5	P38652, A0A0G2JYF7, P85972, D3ZWS0, A0A0G2K6Z8
Cellular Component	2	A0A0G2JWM2, Q566D6
Cellular Component	2	P12839, F1LRZ7
Cellular Component	1	F1LMT5
Cellular Component	133	Q9Z2L0, Q9P290, Q5BKB9, F1M6X3, F1LST1, F1LRZ7, Q566D6, A0A0
Cellular Component	24	P57769, Q9P290, Q9Z2L0, F1LMT5, F1LW91, D4A8M2, A0A0G2K3N
Cellular Component	2	A0A0G2JXN8, Q8VHJ9
Cellular Component	1	G3V9W0
Cellular Component	8	Q63622, Q5BKB9, G3V7J3, M0R423, D3ZWS0, Q6MFX8, P31016, A0
Cellular Component	1	F1M1Y0
Cellular Component	2	E9PTU4, G3V9Y1
Cellular Component	3	P85972, G3V9Y1, Q9JJ19
Cellular Component	9	P08050, F1LPG3, Q6RJR6, F1LW91, Q5FWU3, Q9Z0W5, A0A0G2K3N
Cellular Component	1	Q5U2S7
Cellular Component	1	G3V9G3
Cellular Component	5	Q63622, G3V9G3, D4A3V6, P11275, P31016
Cellular Component	4	Q5M7V8, D3ZAY8, D4A9L2, E9PST5
Cellular Component	2	Q4V8H8, D4A8M2
Cellular Component	20	Q63622, F1M6X3, G3V6L7, A0A0G2K207, P31000, Q6MFX8, Q8VHW
Cellular Component	7	P38652, A0A0G2K2E3, A0A096MJT2, D3ZCL8, M0R423, D3ZJ32, P31
Cellular Component	4	P61980, F1LM55, A0A0G2JWM2, Q566D6
Cellular Component	47	P12839, A0A0U1RRX4, F1LYA6, D4A1Q2, A0A0G2K8P5, G3V9N7, Q5
Cellular Component	6	G3V874, G3V6S0, A0A1B0GWY5, Q9Z0W5, D3ZHV2, O35867
Cellular Component	20	Q63327, F1M241, D4A3V6, F1M3W5, A0A1B0GWY5, G3V8J0, D3ZU
Cellular Component	1	D4A3V6
Cellular Component	1	Q566D6
Cellular Component	53	Q9P290, Q9Z2L0, F1LMT5, P70580, A0A0G2K6N2, F1M6X3, Q9R1N0
Cellular Component	14	P70580, G3V9G3, F1LMV8, G3V6L7, Q02563, D4A0G0, P31016, P08
Cellular Component	4	F1M9F9, G3V9W0, F1LRL9, P15205
Cellular Component	2	A0A0G2JWM2, G3V6L7

Cellular Component	5	P38652, P85972, A0A0G2K207, D4A702, G3V913
Cellular Component	7	E9PTU4, A0A1B0GWY5, Q9Z0W5, G3V9Y1, D3ZHV2, O35867, Q9JJ1
Cellular Component	5	A0A1B0GWY5, Q3ZB99, A0A0G2K1R9, F1M7S0, Q6IRG7
Cellular Component	1	P0C5X8
Cellular Component	1	G3V6L7
Cellular Component	9	D4A853, A0A0G2K9F7, P61980, F1LM55, A0A0G2JWM2, R9PXV8, Q
Cellular Component	5	P38652, P85972, A0A0G2K207, D4A702, G3V913
Cellular Component	3	A0A0G2K6R9, A0A0G2K207, A0A0G2K1R9
Cellular Component	19	A0A0G2K8P5, F1LW91, F1LMV8, A0A1B0GWY5, F1M110, A0A0G2K
Cellular Component	12	P57769, Q80X08, D3ZCI2, A0A0G2K2E3, G3V9N7, Q5FWU3, A0A0G
Cellular Component	9	Q63622, Q62866, F1LMV6, A0A0G2K6R9, F1M6X3, D4A8M2, A0A0
Cellular Component	7	Q52KS1, P08050, Q62866, Q9R1N0, F1MA36, F1LPM3, Q9JJ19
Cellular Component	7	P38652, A0A0G2K6R9, P85972, A0A0G2K207, A0A0G2K1R9, D3ZHV
Cellular Component	6	D4A8M2, G3V6L7, D3ZWS0, Q6MFX8, A0A0G2KAV8, P11275
Cellular Component	1	D3Z9J7
Cellular Component	2	Q9Z2L0, G3V9N1
Cellular Component	9	P57769, Q9P290, Q80X08, Q5FWU3, P47819, Q4V8H8, A0A0G2JX7
Cellular Component	13	A0A0G2K6R9, F1LW91, F1M3G7, A0A0G2K1R9, F1MA36, D4A559, F
Cellular Component	2	D3ZUY8, F1M1Y0
Cellular Component	5	Q9Z2L0, F1LM55, D3Z9R8, D3ZF03, G3V9N1
Cellular Component	1	P85972
Cellular Component	1	D3Z9R8
Cellular Component	2	E9PTU4, G3V9Y1
Cellular Component	2	G3V874, G3V6S0
Cellular Component	1	Q5U2S7
Cellular Component	4	Q63622, G3V9G3, P11275, P31016
Cellular Component	1	P0C627
Cellular Component	5	A0A0G2K9F7, A0A0G2JWM2, Q5M7V8, D3ZG21, C0JPT7
Cellular Component	5	A0A0U1RRX4, D4A1Q2, A0A0G2JX20, A0A0G2JW88, F1M4A4
Cellular Component	1	Q6RJR6
Cellular Component	1	Q52KS1
Cellular Component	1	Q9JJ19
Cellular Component	10	E9PTU4, Q63622, F1LMT5, P47819, G3V9Y1, Q6MFX8, P31000, P31
Cellular Component	2	P57769, D4A0C3
Cellular Component	1	G3V733
Cellular Component	117	Q9Z2L0, Q9P290, A0A0G2K6R9, F1M6X3, F1LRZ7, A0A0G2JX77, Q5
Cellular Component	1	P39069
Cellular Component	1	Q8VHJ9
Cellular Component	1	F1M3G7
Cellular Component	50	P70580, Q5BKB9, Q566D6, A0A0G2JX77, A0A0G2JZX5, P61980, D3Z
Cellular Component	19	A0A0U1RRX4, D4A1Q2, P34926, A0A0G2JWM2, A0A0G2K8P5, Q5BI
Cellular Component	1	F1M062
Cellular Component	1	A0A0G2K7C1
Cellular Component	2	A0A0G2JX30, Q5U2S7
Cellular Component	3	A0A0G2JYF7, Q9Z1H9, Q4V8H8
Cellular Component	6	A0A0G2JYF7, A0A0G2K8P5, G3V9Y1, G3V6L7, F1LPM3, O35867
Cellular Component	4	G3V733, F1M3W5, D4A3V6, P31016

Cellular Component	1	G3V6L7
Cellular Component	4	P39069, Q52KS1, B0BMV7, Q9JJ19
Cellular Component	9	A0A0G2JYF7, P85972, A0A1B0GWY5, A0A0G2KAJ5, Q3ZB99, A0A0G[illegible]
Cellular Component	1	A0A0G2JWM2
Cellular Component	9	Q63622, Q9JKS6, P07936, G3V984, Q5BKB9, F1LRL9, P15205, D4A7[illegible]
Cellular Component	1	D3ZWS0
Cellular Component	2	F1M9F9, G3V9W0
Cellular Component	1	F1M1Y0
Cellular Component	2	Q6RJR6, D3ZJ32
Cellular Component	1	Q4V8H8
Cellular Component	22	Q63622, F1LYA6, D4A1Q2, G3V9N7, A0A0G2JWM2, A0A0G2K6R9, [illegible]
Cellular Component	27	P12839, A0A0U1RRX4, F1LMV6, D4A1Q2, P34926, Q5BKB9, F1LRZ7[illegible]
Cellular Component	1	D3Z9R8
Cellular Component	1	P38652
Cellular Component	14	Q63622, F1LMT5, G3V9G3, F1M6X3, G3V6L7, A0A0G2K207, P1127[illegible]
Cellular Component	4	E9PTU4, P47819, G3V9Y1, P31000
Cellular Component	6	Q63092, G3V733, F1M241, Q9Z0W5, F1M4A4, F1M1Y0
Cellular Component	1	F1M9F9
Cellular Component	4	P39069, Q52KS1, B0BMV7, Q9JJ19
Cellular Component	3	Q9Z2L0, D3Z9R8, D3ZF03
Cellular Component	9	P57769, Q9P290, Q80X08, Q5FWU3, P47819, Q4V8H8, A0A0G2JX7[illegible]
Cellular Component	1	D3ZL75
Cellular Component	11	Q63622, P07936, D4A1Q2, A0A0G2JWM2, A0A0G2K8P5, P28818, G[illegible]
Cellular Component	1	F1M062
Cellular Component	1	Q5M7V8
Cellular Component	14	D3ZMS1, F1M062, Q8K3P4, F1LM55, A0A0G2KB60, A0A0G2K700, D[illegible]
Cellular Component	8	P08050, Q9Z2L0, A0A0G2JYF7, Q9Z1H9, P85972, Q4V8H8, P48303, [illegible]
Cellular Component	1	A0A0G2JXN8
Cellular Component	1	F1M9F9
Cellular Component	4	Q63622, D3ZCL8, A0A1B0GWY5, P31016
Cellular Component	2	F1LST1, D4A8M2
Cellular Component	1	G3V6S0
Cellular Component	1	Q9JJ19
Cellular Component	5	Q5BKB9, F1LRL9, G3V9Y1, P15205, P31016
Cellular Component	18	Q63622, Q9P290, Q62866, F1M6X3, G3V6L7, A0A0G2K207, D3ZCC3[illegible]
Cellular Component	91	Q9Z2L0, D4A1Q2, F1M031, A0A096MJT2, P70580, A0A0G2K6R9, P3[illegible]
Cellular Component	13	Q63622, F1M241, A0A0G2JWM2, A0A0G2K8P5, A0A1B0GWY5, G3V[illegible]
Cellular Component	15	F1M9F9, A0A1B0GWY5, D4A8M2, P31000, D3ZWS0, D4A702, C0JPT[illegible]
Cellular Component	1	Q9WU49
Cellular Component	9	E9PTU4, Q63622, P47819, G3V9Y1, Q6MFX8, P31000, P31016, Q8V[illegible]
Cellular Component	2	P12839, F1LRZ7
Cellular Component	1	Q9Z2L0
Cellular Component	54	D4A1Q2, Q5BKB9, P34926, A0A0G2K6R9, F1LRZ7, Q566D6, D4A0C3[illegible]
Cellular Component	1	P08050
Cellular Component	9	P38652, A0A0G2K2E3, A0A096MJT2, D3ZCL8, P47819, M0R423, P3[illegible]
Cellular Component	2	F1LRL9, P15205
Cellular Component	4	P09606, A0A0G2JWM2, P47819, P31000

Cellular Component	1	P48303
Cellular Component	7	Q63622, P07936, P0C5X8, D3ZUY8, Q9JJ19, P31016, O35867
Cellular Component	1	F1LM55
Cellular Component	3	P08050, P47819, P31000
Cellular Component	1	G3V6S0
Cellular Component	2	F1M241, F1M1Y0
Cellular Component	7	A0A0G2K2E3, A0A0G2JYF7, P85972, M0R423, D3ZJ32, Q9JJ19, P310
Cellular Component	1	D3Z9R8
Cellular Component	4	Q9Z2L0, D3Z9R8, D3ZF03, G3V9N1
Cellular Component	7	G3V733, F1LYA6, G3V9N7, P61765, Q9Z0W5, Q02563, F1M1Y0
Cellular Component	9	P38652, A0A0G2JYF7, P85972, A0A0G2K6R9, P47819, A0A0G2K207
Cellular Component	5	P38652, A0A0G2K6R9, P85972, A0A0G2K1R9, C0JPT7
Cellular Component	1	P61980
Cellular Component	1	D3Z9R8
Cellular Component	12	A0A1B0GWY5, D4A8M2, P31000, D4A702, C0JPT7, G3V913, P0805
Cellular Component	2	P08050, A0A0G2K1R9
Cellular Component	8	P08050, P0C5X8, P70580, G3V9G3, Q6RJR6, D3ZJ32, D4A0G0, A0A0
Cellular Component	1	C0JPT7
Cellular Component	1	Q80X08
Cellular Component	1	B0BMV7
Cellular Component	1	A0A0G2K1R9
Cellular Component	10	G3V733, F1LYA6, G3V984, G3V9N7, P61765, Q9Z0W5, G3V6L7, Q02
Cellular Component	1	F1LMV6
Cellular Component	1	P07936
Cellular Component	2	D3ZUY8, F1M1Y0
Cellular Component	6	P38652, P85972, G3V6S0, A0A0G2K207, D4A702, G3V913
Cellular Component	1	F1LMT5
Cellular Component	11	E9PTU4, Q52KS1, A0A0G2JWM2, A0A0G2JX20, F1M3G7, A0A0G2JX
Cellular Component	1	D4A559
Cellular Component	2	F1LST1, D4A8M2
Cellular Component	41	A0A0U1RRX4, Q9Z2L0, F1LM55, P70580, G3V9N7, Q5BKB9, F1LW91
Cellular Component	20	Q9P290, Q63622, Q62866, D3ZCL8, F1M6X3, G3V6L7, D3ZCC3, A0A
Cellular Component	26	A0A0U1RRX4, D4A1Q2, Q5BKB9, D3ZCL8, A0A0G2K8P5, G3V6L7, M
Cellular Component	4	A0A0G2K2E3, M0R423, D3ZJ32, P31016
Cellular Component	12	P07936, G3V874, P0C5X8, G3V6S0, A0A1B0GWY5, Q4V8H8, D4A8M
Cellular Component	1	E9PST5
Cellular Component	1	Q5U2S7
Cellular Component	155	Q9Z2L0, Q9P290, A0A096MJT2, A0A0G2K6R9, F1M6X3, Q5BKB9, F1
Cellular Component	14	Q63622, F1LMT5, G3V9G3, F1M6X3, G3V6L7, A0A0G2K207, P11275
Cellular Component	1	P38652
Cellular Component	3	Q63622, Q9JJ19, P31016
Cellular Component	4	A0A1B0GWY5, Q9Z0W5, D3ZHV2, O35867
Cellular Component	2	A0A0G2JX30, Q5U2S7
Cellular Component	1	D4A559
Cellular Component	2	Q5FWU3, Q4V8H8
Cellular Component	1	P38652
Cellular Component	2	E9PTU4, G3V9Y1

Cellular Component	3	Q9Z2L0, D3Z9R8, D3ZF03
Cellular Component	5	Q63622, G3V874, A0A0G2JWM2, G3V6L7, P31016
Cellular Component	3	A0A0G2JYF7, Q9Z1H9, Q4V8H8
Cellular Component	1	D4A0G0
Cellular Component	1	P57769
Cellular Component	2	Q9Z2L0, G3V9N1
Cellular Component	3	P57769, Q5FWU3, G3V8A5
Cellular Component	11	P08050, P38652, P85972, A0A0G2K6R9, G3V6S0, A0A0G2K207, A0A
Cellular Component	4	E9PTU4, P47819, G3V9Y1, P31000
Cellular Component	2	P07936, P0C5X8
Cellular Component	1	Q9WU49
Cellular Component	1	D4A0C3
Cellular Component	5	P07936, P0C5X8, D3ZUY8, O35867, Q9JJ19
Cellular Component	1	P0C5X8
Cellular Component	7	D3ZMS1, P61980, F1LM55, D3ZAY8, D4A9L2, D3ZJ92, B2RYB3
Cellular Component	10	E9PTU4, A0A0G2K6R9, P85972, G3V6S0, F1M3G7, G3V9Y1, A0A0G2
Cellular Component	1	A0A0G2JX20
Cellular Component	2	P0C5X8, Q6RJR6
Cellular Component	2	D3ZUY8, F1M1Y0
Cellular Component	4	Q9Z2L0, D3Z9R8, D3ZF03, G3V9N1
Cellular Component	1	D4A3V6
Cellular Component	1	Q9WU49
Cellular Component	5	G3V6S0, D4A8M2, D3ZJ92, F1MA36, P31000
Cellular Component	12	P08050, P38652, A0A0G2JYF7, P61980, P85972, A0A1B0GWY5, D4A
Cellular Component	10	F1M9F9, A0A0G2JWM2, G3V9W0, Q5BKB9, A0A0G2JX20, D3ZLQ8,
Cellular Component	1	D3ZCL8
Cellular Component	5	P57769, Q80X08, Q5FWU3, Q4V8H8, A0A0G2JX77
Cellular Component	6	G3V6S0, D4A8M2, D3ZJ92, Q566D6, F1MA36, P31000
Cellular Component	1	P61765
Cellular Component	13	Q63327, A0A0G2JWM2, G3V9N7, F1LMV8, Q3SWT7, R9PXV8, Q4V8
Cellular Component	2	P47819, P31000
Cellular Component	4	P61980, F1LM55, A0A0G2JWM2, Q566D6
Cellular Component	2	P12839, F1LRZ7
Cellular Component	4	F1M9F9, A0A0G2JWM2, G3V9W0, D3ZL75
Cellular Component	2	A0A0G2JWM2, Q566D6
Cellular Component	2	Q63327, G3V7X2
Cellular Component	40	A0A0U1RRX4, F1LM55, P70580, G3V9N7, Q5BKB9, F1LW91, D4A8M
Cellular Component	3	Q63622, P31016, Q8VHW9
Cellular Component	1	A0A0G2K700
Cellular Component	1	A0A0G2JXN8
Cellular Component	5	A0A0G2K6R9, G3V6S0, A0A0G2K207, A0A0G2K1R9, F1MA36
Cellular Component	1	A0A0G2JX20
Cellular Component	67	D4A1Q2, F1M031, A0A096MJT2, Q5BKB9, A0A0G2K6R9, P34926, F1
Cellular Component	1	G3V9N1
Cellular Component	1	P08050
Cellular Component	2	P47819, P31000
Cellular Component	1	G3V9G3

Information Classification: General

Cellular Component	76	Q9Z2L0, P09606, P70580, G3V8F3, Q5BKB9, Q566D6, A0A0G2JX77,
Cellular Component	1	G3V733
Cellular Component	12	A0A0G2K6R9, F1M3G7, A0A0G2K1R9, F1MA36, D4A559, P31016, E[illegible]
Cellular Component	4	P61980, F1LM55, A0A0G2JWM2, Q566D6
Cellular Component	2	D3ZUY8, F1M1Y0
Cellular Component	3	A0A0G2JYF7, P85972, A0A0G2K6Z8
Cellular Component	2	Q9Z2L0, F1LM55
Cellular Component	2	E9PTU4, G3V9Y1
Cellular Component	5	Q5BKB9, F1LRL9, G3V9Y1, P15205, P31016
Cellular Component	1	A0A0G2K207
Cellular Component	1	P02688
Cellular Component	28	P12839, F1LYA6, D4A1Q2, G3V9N7, A0A0G2K6R9, A0A0G2K8P5, F1
Cellular Component	2	R9PXV8, F1M842
Cellular Component	2	A0A0G2JWM2, M0RBT5
Cellular Component	2	Q5FWU3, D3Z9J7
Cellular Component	1	P85972
Cellular Component	4	F1M9F9, G3V9W0, F1LRL9, P15205
Cellular Component	11	F1M9F9, G3V9G3, G3V9W0, A0A0G2JWM2, Q5BKB9, A0A0G2JX20,
Cellular Component	1	A0A0G2K3N1
Cellular Component	6	Q498D5, Q5FVT1, Q5BKB9, G3V9W0, F1LW91, A0A0G2JX20
Cellular Component	12	Q63622, A0A0G2JWM2, A0A0G2K6R9, G3V6L7, A0A0G2K207, A0A[illegible]
Cellular Component	3	E9PTU4, P38652, G3V9Y1
Cellular Component	1	F1LMT5
Cellular Component	3	E9PTU4, P38652, G3V9Y1
Cellular Component	60	Q9Z2L0, P09606, P70580, P82808, P0C6C0, Q9R1N0, P25093, G3V8[illegible]
Cellular Component	1	A0A0G2K700
Cellular Component	2	Q8K3P4, Q5GFD9
Cellular Component	1	G3V9W0
Cellular Component	2	F1M062, A0A0G2KB60
Cellular Component	2	B8K2Q4, Q8VHW9
Cellular Component	12	Q63622, F1LMT5, G3V9G3, F1M6X3, G3V6L7, A0A0G2K207, P1127[illegible]
Cellular Component	1	F1M1Y0
Cellular Component	5	P08050, Q6RJR6, F1LW91, A0A0G2K3N1, D4A0C3
Cellular Component	1	A0A0G2JXN8
Cellular Component	4	Q52KS1, D4A3V6, A0A0G2K7C1, G3V6R7
Cellular Component	19	Q63327, F1M241, D4A3V6, F1M3W5, A0A1B0GWY5, G3V8J0, D3ZU
Cellular Component	1	A0A0G2K700
Cellular Component	1	Q9WU49
Cellular Component	42	P57769, F1LMT5, F1LMV8, F1M3G7, D4A8M2, G3V6L7, A0A0G2K3N
Cellular Component	3	G3V733, F1M3W5, P31016
Cellular Component	27	P12839, A0A0U1RRX4, F1LMV6, D4A1Q2, P34926, Q5BKB9, F1LRZ7,
Cellular Component	9	D4A853, A0A0G2K9F7, P61980, F1LM55, A0A0G2JWM2, R9PXV8, Q
Cellular Component	1	A0A0G2JXN8
Cellular Component	2	G3V874, G3V6S0
Cellular Component	2	E9PTU4, G3V9Y1
Cellular Component	1	D4A1Q2
Cellular Component	1	D3Z9R8

Cellular Component	1	D4A0C3
Cellular Component	17	Q63622, G3V9G3, A0A0G2K6R9, D4A3V6, F1LW91, F1M3G7, A0A0(
Cellular Component	32	F1LM55, Q5BKB9, G3V9N7, F1LW91, A0A0G2JX77, Q566D6, Q5U2S
Cellular Component	3	Q5FWU3, A0A0G2K3N1, D4A0C3
Cellular Component	11	Q498D5, Q5FVT1, Q5BKB9, G3V9W0, A0A0G2JWM2, F1LW91, A0A1
Cellular Component	1	F1M4A4
Cellular Component	3	A0A0G2JYF7, P85972, Q9JJ19
Cellular Component	3	D3ZMS1, D3ZJ92, B4F786
Cellular Component	19	P57769, Q9P290, D3ZCI2, A0A0G2K2E3, G3V9N7, F1M6X3, A0A0G2
Cellular Component	1	Q9WU49
Cellular Component	3	G3V9G3, D3ZHV2, C0JPT7
Cellular Component	2	E9PTU4, G3V9Y1
Cellular Component	8	P38652, A0A0G2JYF7, P85972, A0A0G2K6R9, P47819, A0A0G2K207
Cellular Component	3	P47819, P31000, G3V8A5
Cellular Component	1	F1M1Y0
Cellular Component	1	Q9JJ19
Cellular Component	4	G3V6S0, A0A0G2K207, A0A0G2K1R9, F1MA36
Cellular Component	3	P08050, F1LPG3, Q9Z0W5
Cellular Component	1	D4A3V6
Cellular Component	2	E9PTU4, G3V9Y1
Cellular Component	2	D4A0C3, P82458
Cellular Component	10	A0A0G2JWM2, Q5BKB9, Q5M7V8, Q6AYH3, A0A0G2K700, D4A9L2,
Cellular Component	1	F1M1Y0
Cellular Component	1	Q8VHJ9
Cellular Component	5	A0A0G2K6R9, G3V6S0, A0A0G2K1R9, F1MA36, D4A559
Cellular Component	1	Q5FWU3
Cellular Component	1	D4A8M2
Cellular Component	1	F1LMT5
Cellular Component	19	Q9Z2L0, P09606, Q63327, Q498D5, F1LM55, G3V9G3, F1LRZ7, F1LN
Cellular Component	5	A0A1B0GWY5, Q3ZB99, A0A0G2K1R9, F1M7S0, Q6IRG7
Cellular Component	3	Q52KS1, F1M3G7, A0A0G2JX77
Cellular Component	5	P57769, Q80X08, A0A0G2KB60, Q4V8H8, G3V8A5
Cellular Component	8	G3V733, F1LYA6, G3V9N7, P61765, Q9Z0W5, G3V6L7, Q02563, F1N
Cellular Component	3	F1LMT5, A0A0G2JY69, P31000
Cellular Component	1	A0A0G2K7C1
Cellular Component	2	F1M241, F1M1Y0
Cellular Component	20	Q63327, F1M241, D4A3V6, F1M3W5, A0A1B0GWY5, G3V8J0, D3ZU
Cellular Component	2	Q5FWU3, A0A0G2K3N1
Cellular Component	8	P08050, Q9Z2L0, A0A0G2JYF7, Q9Z1H9, P85972, Q4V8H8, P48303,
Cellular Component	1	Q9JJ19
Cellular Component	1	Q4V8H8
Cellular Component	1	A0A0G2JYF7
Cellular Component	7	Q63622, A0A0G2JWM2, G3V6L7, A0A0G2K207, A0A0G2K1R9, G3V9
Cellular Component	2	Q6MFX8, A0A0A0MXV8
Cellular Component	1	F1LMT5
Cellular Component	9	A0A0U1RRX4, P70580, G3V6S0, A0A0G2K700, D4A8M2, A0A0G2JZ5
Cellular Component	5	F1M9F9, A0A0G2JWM2, G3V9W0, A0A0G2JX20, D3ZL75

Cellular Component	6	Q63092, G3V733, F1M241, Q9Z0W5, F1M4A4, F1M1Y0
Cellular Component	18	A0A0U1RRX4, Q63622, F1M241, G3V9G3, D4A1Q2, Q5BKB9, D3ZCL
Cellular Component	5	Q63622, F1M6X3, G3V6L7, A0A0G2K3B8, P31016
Cellular Component	1	A0A0G2K700
Cellular Component	6	P57769, Q80X08, Q5FWU3, Q4V8H8, A0A0G2JX77, G3V8A5
Cellular Component	3	Q63622, P31016, Q8VHW9
Cellular Component	3	A0A0G2K6R9, A0A0G2K1R9, D4A559
Cellular Component	5	F1LPG3, F1M241, Q9Z0W5, G3V8J0, F1M1Y0
Cellular Component	2	Q5FWU3, D4A0G0
Cellular Component	1	P85972
Cellular Component	3	E9PTU4, P38652, G3V9Y1
Cellular Component	6	P12839, P08050, F1LMV6, F1LRZ7, P47819, P31000
Cellular Component	11	Q63622, F1LMT5, G3V9G3, F1M6X3, B8K2Q4, G3V6L7, A0A0G2K3B
Cellular Component	3	E9PTU4, G3V9Y1, D4A559
Cellular Component	1	F1LW91
Cellular Component	1	Q9WU49
Cellular Component	1	P13233
Cellular Component	1	A0A0G2K700
Cellular Component	16	A0A0G2K2E3, A0A0G2K6R9, F1M3G7, A0A0G2K1R9, F1MA36, D4A
Cellular Component	2	E9PTU4, G3V9Y1
Cellular Component	1	P47819
Cellular Component	1	A0A0G2JWM2
Cellular Component	1	Q9WU49
Cellular Component	1	D3ZJ92
Cellular Component	2	A0A0G2JX30, Q5U2S7
Cellular Component	3	P47819, P31000, G3V8A5
Cellular Component	2	Q6MFX8, A0A0A0MXV8
Cellular Component	9	P38652, P85972, A0A0G2K6R9, G3V6S0, A0A0G2K207, A0A0G2K1R
Cellular Component	1	G3V9Y1
Cellular Component	17	A0A0U1RRX4, Q498D5, D4A1Q2, P34926, A0A0G2JWM2, Q5BKB9,
Cellular Component	1	F1M1Y0
Cellular Component	3	E9PTU4, P38652, G3V9Y1
Cellular Component	1	P57769
Cellular Component	11	E9PTU4, A0A0G2K6R9, P85972, G3V6S0, F1M3G7, G3V9Y1, A0A0G
Cellular Component	1	G3V9W0
Cellular Component	33	A0A0U1RRX4, D4A644, Q810W7, D4A1Q2, F1M031, Q5BKB9, P349
Cellular Component	1	G3V8A5
Cellular Component	1	G3V9W0
Cellular Component	2	F1LPG3, Q9Z0W5
Cellular Component	2	P31016, Q8VHW9
Cellular Component	1	D4A0C3
Cellular Component	3	A0A0G2JWM2, F1LW91, A0A0G2JW88
Cellular Component	3	F1LMT5, A0A0G2JY69, P31000
Cellular Component	2	D3ZMS1, D3ZJ92
Cellular Component	1	A0A0G2K700
Cellular Component	5	D3ZMS1, P61980, D3ZAY8, D4A9L2, B2RYB3
Cellular Component	1	F1M1Y0

Information Classification: General

Cellular Component	1	P31016
Cellular Component	1	Q4V8H8
Cellular Component	2	R9PXV8, F1M842
Cellular Component	6	G3V733, Q63327, F1M3W5, P61765, G3V7X2, P31016
Cellular Component	1	P0C5X8
Cellular Component	7	Q5BKB9, Q5M7V8, Q6AYH3, A0A0G2K700, D4A9L2, B4F786, E9PST5
Cellular Component	5	P57769, P47819, P31000, A0A0G2K1R9, G3V8A5
Cellular Component	1	D3ZMS1
Cellular Component	2	Q52KS1, F1M3G7
Cellular Component	3	P57769, Q80X08, Q4V8H8
Cellular Component	3	D3ZMS1, D3ZJ92, B4F786
Cellular Component	1	Q52KS1
Cellular Component	6	P12839, P08050, F1LMV6, F1LRZ7, P47819, P31000
Cellular Component	1	D3ZMS1
Cellular Component	2	P57769, Q5FWU3
Cellular Component	1	Q9WU49
Cellular Component	1	F1M1Y0
Cellular Component	1	F1M3G7
Cellular Component	5	P57769, P47819, P31000, A0A0G2K1R9, G3V8A5
Cellular Component	5	Q63622, F1M6X3, G3V6L7, A0A0G2K3B8, P31016
Cellular Component	3	F1M241, G3V8J0, F1M1Y0
Cellular Component	1	A0A0G2K700
Cellular Component	4	G3V9G3, B8K2Q4, P11275, Q8VHW9
Cellular Component	1	P13233
Cellular Component	1	D4A0C3
Cellular Component	1	A0A0G2K207
Cellular Component	5	Q5M7V8, Q6AYH3, D4A9L2, B4F786, E9PST5
Cellular Component	1	B4F786
Cellular Component	1	Q5BKB9
Cellular Component	2	Q6MFX8, A0A0A0MXV8
Cellular Component	1	D3ZJ92
Cellular Component	1	D4A0C3
Cellular Component	1	D3ZMS1
Cellular Component	1	D3ZJ92
Cellular Component	1	F1M1Y0
Cellular Component	1	F1M3G7

Information Classification: General

, Q9JJ19, Q8VHW9, A0A0G2K6Z8, F1M836, G3V7X2, D3ZG21, D3ZF03, M0RD40, Q63622, F1M9F9, A0A0H2UHZ1,
, Q9JJ19, Q8VHW9, A0A0G2K6Z8, F1M836, G3V7X2, D3ZG21, D3ZF03, M0RD40, Q63622, F1M9F9, A0A0H2UHZ1,
2563, P15205, D4A702, P31016, G3V733, F1LRL9, B8K2Q4, P61765, Q9Z0W5, Q63622, P07936, F1M287, F1M3W
K6N2, Q5BKB9, A0A0G2K6R9, F1M6X3, F1LST1, F1M110, Q9R1N0, G3V8J0, D4A0C3, P31016, Q9JJ19, Q8VHW9, E

3, G3V8J0, D4A0C3, P31016, Q9JJ19, A0A0G2K6Z8, P39069, P61980, G3V7X2, G3V8A5, Q63622, F1M3W5, Q4V8
L, D4A8M2, A0A0G2JZ50, Q566D6, A0A0G2JX77, Q5U2S7, P61980, D3ZAY8, P61765, D3ZG21, A0A0G2K7C1, D3Z
8652, G3V733, P61980, Q63622, F1M9F9, A0A1B0GWY5, P31000, D3ZWS0, P08050, Q9JKS6, A0A0G2JYF7, G3V98
3, G3V8J0, D4A0C3, Q9JJ19, P31016, A0A0G2K6Z8, P39069, P61980, G3V7X2, G3V8A5, Q63622, F1M3W5, Q4V8
, D4A0C3, Q63330, P15205, F1M4A4, E9PTU4, P39069, P47819, F1LRL9, Q498D5, A0A0G2JWM2, A0A1B0GWY5,
LST1, A0A0G2K4N6, G3V8J0, A0A0G2JX77, A0A0G2K613, D4A0C3, D4A702, Q9JJ19, Q8VHW9, E9PTU4, P39069,
V6L7, Q02563, A0A0G2K1R9, P15205, D4A702, P31016, D4A0I5, E9PTU4, G3V733, F1LRL9, B8K2Q4, P61765, Q9Z
, Q566D6, A0A0G2JX77, Q63330, P15205, Q8VHW9, P31016, Q9JJ19, G3V6R7, E9PTU4, P38652, P61980, B8K2Q4
JX77, D4A0C3, Q63330, D4A702, Q9JJ19, A0A0G2K6Z8, E9PTU4, P39069, F1M836, P38652, D3ZG21, G3V8A5, D3
LST1, F1M6X3, P82808, F1LRZ7, Q566D6, A0A0G2JX77, G3V8J0, D4A0C3, Q63330, D4A702, Q9JJ19, A0A0G2K6Z8
, Q9JJ19, Q8VHW9, A0A0G2K6Z8, F1M836, G3V7X2, D3ZG21, D3ZF03, M0RD40, Q63622, F1M9F9, A0A0H2UHZ1,
3, Q9R1N0, M0R423, G3V6L7, A0A0G2K1R9, Q9JJ19, P31016, Q9Z1H9, G3V874, P0C5X8, Q9Z0W5, O35867, Q52
31016, Q9JJ19, Q8VHW9, E9PTU4, P38652, B8K2Q4, Q63622, A0A0H2UHZ1, A0A1B0GWY5, Q4V8H8, D3ZCC3, D3

LST1, F1M6X3, F1LRZ7, Q566D6, A0A0G2JX77, G3V8J0, D4A0C3, Q63330, D4A702, Q9JJ19, A0A0G2K6Z8, E9PTU4
C0, Q9R1N0, P25093, G3V8J0, D4A0C3, Q9JJ19, A0A0G2K6Z8, Q5U2S7, G3V913, P39069, P61980, P85972, P617
E9PTU4, G3V733, P02688, Q63622, F1M9F9, D4A3V6, A0A1B0GWY5, Q4V8H8, D4A559, G3V984, G3V6S0, G3V9Y

L, D4A8M2, A0A0G2JZ50, Q566D6, A0A0G2JX77, Q5U2S7, P61980, D3ZAY8, P61765, D3ZG21, A0A0G2K7C1, D3Z

0G2K3N1, A0A0G2JX77, D4A0C3, F1M4A4, G3V9N1, Q80X08, G3V733, P0C5X8, Q5FWU3, P47819, Q9Z0W5, D3Z

TU4, P38652, P0C5X8, P85972, B8K2Q4, P47819, G3V8A5, Q63622, G3V9G3, A0A0G2K207, D3ZUY8, P31000, Q6
ZX5, D4A0C3, D4A0G0, P31016, Q9JJ19, G3V733, G3V7X2, G3V8A5, D3ZF03, Q8VHJ9, Q63622, D4A3V6, F1M3W

, Q8VHW9, P31016, E9PTU4, B8K2Q4, Q8VHJ9, Q63622, A0A0H2UHZ1, Q8R5H2, D3ZCC3, P31000, P08050, A0A
, P31016, Q9JJ19, E9PTU4, G3V733, P02688, Q63622, F1M241, D4A3V6, F1M3W5, F1M287, A0A1B0GWY5, P310

LST1, F1M6X3, F1LRZ7, A0A0G2K4N6, Q566D6, A0A0G2JX77, G3V8J0, D4A0C3, Q63330, A0A0G2K613, D4A702,
M6X3, A0A0G2K4N6, F1M110, Q9R1N0, A0A0G2K613, P15205, D4A0G0, P31016, Q9JJ19, Q8VHW9, E9PTU4, P3
S0, Q9JJ19, E9PTU4, A0A0G2JYF7, G3V874, G3V6S0, D4ADX8, G3V9Y1, Q9Z0W5, D3ZHV2, F1LPM3, O35867

6MFX8, P11275, P31016, F1M1Y0, Q9JKS6, G3V733, G3V984, P61765, Q9Z0W5, A0A0G2KAV8, D3ZJ32

5C0, Q9R1N0, P25093, G3V8J0, D4A0C3, Q9JJ19, A0A0G2K6Z8, Q5U2S7, G3V913, P39069, P61980, P85972, P617[illegible]

0A0G2K4N6, A0A0G2K613, Q9JJ19, Q8VHW9, E9PTU4, P39069, F1M836, P38652, B8K2Q4, P02688, M0RD40, Q63

, D4A0C3, Q63330, P15205, F1M4A4, E9PTU4, P39069, P47819, F1LRL9, Q498D5, A0A0G2JWM2, A0A1B0GWY5, [illegible]

A0G2K207, A0A0G2K1R9, P31000, D3ZWS0, Q02563, Q6IRG7, A0A0G2K6Z8, P08050, P38652, A0A0G2JYF7, G3V8
P31016, P39069, E9PTU4, G3V733, P02688, Q63622, F1M9F9, F1M241, D4A3V6, F1M287, A0A1B0GWY5, Q4V8H[illegible]

D6, A0A0G2JX77, G3V8J0, D4A0C3, D4A702, Q9JJ19, A0A0G2K6Z8, E9PTU4, P39069, G3V7X2, D3ZG21, G3V8A5, [illegible]

, Q566D6, A0A0G2JX77, Q63330, P15205, Q8VHW9, Q9JJ19, P31016, G3V6R7, E9PTU4, P38652, P61980, B8K2Q4

LST1, F1M6X3, F1LRZ7, A0A0G2K4N6, Q566D6, A0A0G2JX77, G3V8J0, D4A0C3, Q63330, A0A0G2K613, D4A702, [illegible]

/8J0, A0A0G2JX77, P31000, D3ZUY8, G3V9N1, F1M1Y0, P08050, Q80X08, G3V733, A0A0G2JYF7, Q9Z1H9, P85972,

Information Classification: General

5C0, Q9R1N0, P25093, G3V8J0, D4A0C3, Q9JJ19, A0A0G2K6Z8, Q5U2S7, G3V913, P39069, P61980, P85972, P6176

JX77, D4A0C3, Q63330, Q9JJ19, A0A0G2K6Z8, E9PTU4, P39069, F1M836, P38652, D3ZG21, G3V8A5, D3ZF03, A0A
34926, Q5BKB9, F1LRZ7, Q566D6, D4A0C3, Q63330, P15205, D4A702, P31016, Q9JJ19, A0A0G2K6Z8, E9PTU4, P39

J0, D4A0C3, Q9JJ19, P31016, A0A0G2K6Z8, E9PTU4, P39069, G3V733, P61980, G3V7X2, G3V8A5, F1M241, D4A3V

0G2JX77, G3V8J0, D4A0C3, D4A702, Q9JJ19, A0A0G2K6Z8, E9PTU4, G3V7X2, D3ZG21, G3V8A5, D3ZF03, A0A0G2K
1, A0A0G2JX77, D4A0C3, G3V9N1, Q80X08, G3V733, P0C5X8, Q5FWU3, P47819, G3V8A5, F1M241, G3V9G3, Q4V

W9, Q9JJ19, P31016, F1M1Y0, E9PTU4, P08050, P38652, A0A0G2JYF7, P85972, B8K2Q4, P47819, G3V9Y1, A0A0G2

5BKB9, D3ZCL8, A0A0G2K6R9, F1LRZ7, G3V6L7, M0R423, A0A0G2K1R9, Q02563, F1M4A4, P15205, P31016, E9PTU

Y8, D4A559, F1M4A4, P13233, P31016, F1M1Y0, P08050, Q63092, F1LPG3, G3V733, Q5FWU3, P61765, Q9Z0W5,

0, Q8VHW9, P31016, E9PTU4, B8K2Q4, Q8VHJ9, Q63622, A0A0H2UHZ1, Q8R5H2, D3ZCC3, P31000, P08050, A0A0

3N1, A0A0G2JXZ7, A0A0G2JX77, D4A0C3, A0A0G2JZX5, A0A0G2JSU4, P08050, F1LPG3, A0A0G2JYF7, Q6RJR6, Q5[illegible]

56D6, G3V8J0, D4A0C3, D4A702, Q9JJ19, E9PTU4, P38652, B8K2Q4, G3V7X2, G3V8A5, D3ZF03, A0A0G2K7C1, Q6[illegible]

[illegible]G21, Q8VHJ9, A0A0G2K7C1, D3ZMS1, F1M9F9, A0A0G2KB60, A0A0G2K700, P31000, D3ZWS0, P13233, Q4V8H5,
[illegible]KB9, A0A1B0GWY5, A0A0G2JX20, Q566D6, Q63330, A0A0G2JW88, P15205, F1M4A4, F1M1Y0, P08050, G3V9W0,

Information Classification: General

)4A3V6, G3V6L7, A0A0G2K207, Q02563, A0A0G2K1R9, F1MA36, F1M4A4, P31016, F1M1Y0, G3V733, G3V874, G3
, D4A0C3, Q63330, P15205, F1M4A4, E9PTU4, P39069, P47819, F1LRL9, Q498D5, A0A0G2JWM2, A0A1B0GWY5, A

3, Q6MFX8, P31000, P31016, Q8VHW9, E9PTU4, P08050, P47819, G3V9Y1, A0A0G2K3B8, D3ZJ32, A0A0A0MXV8
34926, Q5BKB9, F1LRZ7, Q566D6, D4A0C3, Q63330, P15205, D4A702, P31016, Q9JJ19, A0A0G2K6Z8, E9PTU4, P39

, Q63330, P15205, Q9JJ19, A0A0G2K6Z8, E9PTU4, P39069, F1M836, P38652, P61980, M0RD40, F1M9F9, A0A1B0

l, D4A8M2, A0A0G2JZ50, Q566D6, A0A0G2JX77, Q5U2S7, P61980, D3ZAY8, P61765, D3ZG21, A0A0G2K7C1, D3ZN
0G2K207, Q6MFX8, P31000, P31016, Q8VHW9, E9PTU4, P08050, P47819, P48303, G3V9Y1, A0A0G2K3B8, D3ZJ3
0R423, P15205, P31016, E9PTU4, G3V733, F1LRL9, P47819, P02688, Q63622, F1M241, G3V9G3, A0A0G2JWM2, A

LST1, F1LRZ7, A0A0G2K4N6, A0A0G2JX77, Q566D6, G3V8J0, D4A0C3, Q63330, A0A0G2K613, D4A702, Q9JJ19, A0

12, A0A0G2JZ50, Q566D6, A0A0G2JX77, Q5U2S7, P61980, D3ZAY8, P61765, D3ZG21, A0A0G2K7C1, D3ZMS1, F1M

LLRZ7, Q566D6, D4A0C3, Q63330, P15205, D4A702, P31016, Q9JJ19, A0A0G2K6Z8, E9PTU4, P39069, F1M836, P3

Information Classification: General

G3V8J0, A0A0G2JZX5, D4A702, A0A0G2K7X3, A0A0G2K6Z8, E9PTU4, P61980, D3ZG21, Q8VHJ9, A0A0G2K7C1, Q5

LRZ7, G3V6L7, A0A0G2K1R9, Q02563, F1M4A4, P31016, G3V733, G3V874, P61765, Q9Z0W5, P02688, Q63622, P(

J0, D4A0C3, Q9JJ19, P31016, A0A0G2K6Z8, P39069, E9PTU4, G3V733, P61980, G3V7X2, G3V8A5, F1M241, D4A3V

Y8, F1M4A4, D4A559, P13233, P31016, F1M1Y0, P08050, Q63092, F1LPG3, G3V733, P61765, Q9Z0W5, G3V7X2

N1, Q566D6, A0A0G2JX77, O70593, P15205, D4A702, D4A0G0, P31016, G3V6R7, E9PTU4, P38652, Q80X08, F1LRL

, D4A0C3, Q63330, P15205, F1M4A4, E9PTU4, P39069, P47819, F1LRL9, Q498D5, A0A0G2JWM2, A0A1B0GWY5, A

G2K1R9, F1MA36, D4A559, P11275, P31016, E9PTU4, P85972, G3V6S0, G3V9Y1, A0A0G2JXN8, O35867
7, P61980, D3ZAY8, P61765, D3ZG21, A0A0G2K7C1, D3ZMS1, F1M062, A0A0G2JWM2, A0A0G2K700, Q3SWT4, D4

KB60, Q4V8H8, A0A0G2JX77, P31000, A0A0G2K1R9, D4A0G0, Q80X08, Q5FWU3, P47819, P48303, P61765, Q9Z0

MV8, P0C6C0, A0A0G2JZ50, F1M8H5, G3V9N1, F1M4A4, P11275, G3V6R7, D3Z9R8, P61765, G3V9Y1, D3ZF03

Y8, D4A559, F1M4A4, P13233, P31016, F1M1Y0, P08050, Q63092, F1LPG3, G3V733, Q5FWU3, P61765, Q9Z0W5,

Information Classification: General

.8, A0A1B0GWY5, M0R423, G3V6L7, A0A0G2JW88, P15205, P11275, P31016, G3V733, G3V984, F1LRL9, G3V9Y1

A0A1B0GWY5, A0A0G2JX20, D4A0C3, Q63330, A0A0G2JW88, P15205, F1M4A4, P13233, F1LRL9, D3ZHV2, P8245[illegible]

26, F1LW91, D4A0C3, Q63330, F1M4A4, P15205, Q9JJ19, G3V913, A0A0G2K6Z8, F1LRL9, Q498D5, F1M9F9, G3V9[illegible]

Information Classification: General

Information Classification: General

F1M287, D3ZWS0, D4A4L4, P08050, Q9JKS6, F1LPG3, B0BMV7, B0BN77, D3ZJ92, D3ZHV2, F1M7S0, F1M842, Q6
F1M287, D3ZWS0, D4A4L4, P08050, Q9JKS6, F1LPG3, B0BN77, B0BMV7, D3ZJ92, D3ZHV2, F1M7S0, F1M842, Q6
5, D3ZWS0, Q6MFX8, P11275, F1M1Y0, Q9JKS6, G3V984, G3V9Y1, P49621, D3ZJ32, A0A0G2KAV8, A0A0A0MXV8
9PTU4, P38652, B8K2Q4, G3V8A5, Q8VHJ9, Q63622, A0A0H2UHZ1, A0A1B0GWY5, Q8R5H2, Q4V8H8, D3ZCC3, D3

H8, P31000, D3ZWS0, P13233, Q4V8H5, P08050, Q9JKS6, Q6RJR6, G3V6S0, G3V9Y1, B0BMV7, F1LMV6, G3V9N7,
MS1, F1M062, A0A0G2JWM2, Q3SWT4, A0A0G2K700, D4A9L2, D3ZWS0, F1MA36, P31000, A0A0G2JSU4, C0JPT7,
4, F1LS01, F1M7S0, F1LMV6, D3ZCL8, D4A8M2, G3V7J3, M0R423, G3V6L7, A0A0G2K1R9, Q02563, G3V913, G3V
H8, P31000, D3ZWS0, P13233, Q4V8H5, P08050, Q9JKS6, Q6RJR6, G3V6S0, G3V9Y1, F1LMV6, G3V9N7, F1LW91, D

1M836, P38652, B8K2Q4, P02688, D3ZF03, G3V8A5, M0RD40, A0A0G2K7C1, Q63622, A0A0H2UHZ1, F1M287, A0
W5, Q63622, P07936, F1M241, G3V9G3, F1M287, F1M3W5, G3V927, D3ZWS0, Q6MFX8, P11275, F1M1Y0, Q9JK
, D3ZG21, G3V8A5, Q8VHJ9, A0A0G2K7C1, Q63622, D3ZMS1, F1M9F9, D4A3V6, A0A0G2KB60, A0A1B0GWY5, A0
F03, A0A0G2K7C1, M0RD40, F1M9F9, A0A1B0GWY5, D3ZWS0, D4A559, P13233, P08050, F1LPG3, B0BMV7, D3Z
, E9PTU4, F1M836, P39069, P38652, D3ZG21, G3V7X2, G3V8A5, D3ZF03, A0A0G2K7C1, M0RD40, Q63622, F1M9
F1M287, D3ZWS0, D4A4L4, P08050, Q9JKS6, F1LPG3, B0BN77, B0BMV7, D3ZJ92, D3ZHV2, F1M7S0, F1M842, Q6
KS1, Q63622, P07936, A0A0H2UHZ1, A0A1B0GWY5, Q4V8H8, D3ZWS0, Q6MFX8, F1MA36, D4A559, P11275, P08
ZWS0, P31000, D4A559, P08050, A0A0G2JYF7, G3V6S0, G3V9Y1, D3ZHV2, Q62866, F1LMV6, D3ZCL8, G3V7J3, D4

, F1M836, P39069, P38652, D3ZG21, G3V7X2, G3V8A5, D3ZF03, A0A0G2K7C1, M0RD40, Q63622, F1M9F9, F1M2
55, G3V8A5, F1LM19, Q52KS1, Q4V8H8, D4A9L2, A0A0G2JW88, P31000, D3ZWS0, A0A0G2JSU4, Q6IRG7, C0JPT7,
1, B0BMV7, F1LS01, D3ZHV2, F1LYA6, A0A0G2K8P5, G3V9N7, D3ZCL8, D4A8M2, G3V6L7, A0A0G2K1R9, Q02563,

MS1, F1M062, A0A0G2JWM2, Q3SWT4, A0A0G2K700, D4A9L2, D3ZWS0, F1MA36, P31000, A0A0G2JSU4, C0JPT7,

F03, G3V8A5, F1M241, G3V9G3, Q4V8H8, P31000, D4A559, F1M1Y0, P08050, Q63092, Q6RJR6, D3Z9R8, A0A0G2

, A0A1B0GWY5, A0A0G2KB60, Q4V8H8, P08050, F1LPG3, A0A0G2JYF7, Q6RJR6, D3ZHV2, A0A0G2K8P5, G3V9N7,

0G2K1U5, Q6RJR6, D3Z9D0, G3V9Y1, F1LS01, Q62866, D3ZCL8, D4A8M2, G3V7J3, G3V6L7, A0A0G2JXZ7, Q02563,
0, D3ZWS0, Q9JKS6, G3V984, G3V6S0, G3V9Y1, F1LS01, F1M7S0, A0A0U1RRX4, P12839, F1LYA6, A0A0G2K8P5,

Q9JJ19, A0A0G2K6Z8, E9PTU4, F1M836, P39069, P38652, B8K2Q4, D3ZG21, G3V7X2, G3V8A5, D3ZF03, A0A0G2K
9069, F1M836, P38652, B8K2Q4, P02688, M0RD40, Q63622, A0A0H2UHZ1, F1M287, A0A1B0GWY5, Q8R5H2, Q4

;5, G3V8A5, F1LM19, Q52KS1, Q4V8H8, D4A9L2, A0A0G2JW88, P31000, D3ZWS0, A0A0G2JSU4, Q6IRG7, C0JPT7,

3622, A0A0H2UHZ1, F1M287, A0A1B0GWY5, D3ZCC3, D3ZWS0, D4A559, P08050, D3ZHV2, F1M7S0, E9PST5, Q62

8, P31000, D4A559, A0A0G2JYF7, G3V984, G3V6S0, G3V9Y1, B0BMV7, F1LS01, D3ZHV2, F1M7S0, P12839, A0A0U

)3ZF03, A0A0G2K7C1, Q63622, F1M9F9, F1M287, A0A1B0GWY5, D3ZWS0, D4A559, P13233, P08050, F1LPG3, Q9

, D3ZG21, G3V8A5, Q8VHJ9, A0A0G2K7C1, Q63622, F1M9F9, D4A3V6, A0A1B0GWY5, P31000, D3ZWS0, P08050,

Q9JJ19, A0A0G2K6Z8, E9PTU4, F1M836, P39069, P38652, B8K2Q4, D3ZG21, G3V7X2, G3V8A5, D3ZF03, A0A0G2K7

Information Classification: General

55, G3V8A5, F1LM19, Q52KS1, Q4V8H8, D4A9L2, A0A0G2JW88, P31000, D3ZWS0, A0A0G2JSU4, Q6IRG7, C0JPT7,

A0G2K7C1, M0RD40, F1M9F9, A0A1B0GWY5, D3ZWS0, D4A559, P13233, P08050, F1LPG3, D3ZJ92, D3ZHV2, B4F7[illegible]
9069, F1M836, P38652, G3V733, P61980, M0RD40, Q63622, F1M9F9, A0A1B0GWY5, A0A0G2KB60, A0A0G2K700,

V6, F1M287, F1M3W5, A0A1B0GWY5, Q4V8H8, P31000, D3ZWS0, D4A559, P13233, P08050, Q9JKS6, F1LPG3, Q6[illegible]

7C1, Q63622, F1M9F9, F1M287, A0A1B0GWY5, D3ZWS0, D4A559, P13233, P08050, F1LPG3, D3ZJ92, D3ZHV2, F1

J4, G3V733, G3V874, P28818, F1LRL9, P61765, Q9Z0W5, P02688, O35867, Q63622, P07936, F1M241, G3V9G3, A

0G2K1U5, Q6RJR6, D3Z9D0, G3V9Y1, F1LS01, Q62866, D4A8M2, G3V7J3, G3V6L7, A0A0G2JXZ7, Q02563, G3V9N1,

Information Classification: General

[illegible]622, F1M9F9, F1M287, A0A1B0GWY5, D3ZWS0, D4A559, P13233, P08050, F1LPG3, D3ZHV2, F1M7S0, F1LM55, [illegible]

Q5BJN8, G3V6S0, D3ZJ92, M0RBT5, Q4G045, B4F786, E9PST5, A0A0U1RRX4, F1LM55, G3V9N7, F1LW91, D4A8M[illegible]

)069, F1M836, P38652, G3V733, P61980, M0RD40, Q63622, F1M9F9, A0A1B0GWY5, A0A0G2KB60, A0A0G2K700,

)GWY5, D3ZLQ8, P31000, D4A559, P13233, P08050, G3V6S0, G3V9Y1, D3ZHV2, A0A0U1RRX4, P12839, F1LMV6, A

/IS1, F1M062, A0A0G2JWM2, Q3SWT4, A0A0G2K700, D4A9L2, D3ZWS0, F1MA36, P31000, A0A0G2JSU4, C0JPT7,

)A0G2K6Z8, E9PTU4, P39069, F1M836, P38652, B8K2Q4, G3V7X2, G3V8A5, D3ZF03, A0A0G2K7C1, Q63622, F1M[illegible]

I062, A0A0G2JWM2, Q3SWT4, A0A0G2K700, D4A9L2, D3ZWS0, F1MA36, P31000, A0A0G2JSU4, C0JPT7, P13233,

8652, P61980, M0RD40, F1M9F9, A0A1B0GWY5, A0A0G2JW20, D3ZLQ8, P31000, D4A559, P13233, D4A4L4, P08[illegible]

5XI97, D3ZMS1, F1M9F9, F1M287, A0A0G2KB60, A0A0G2K700, P31000, D3ZWS0, P13233, D3ZYS1, Q4V8H5, M0R

/6, F1M3W5, F1M287, A0A1B0GWY5, Q4V8H8, P31000, D3ZWS0, D4A559, P13233, P08050, Q9JKS6, F1LPG3, Q6I

.9, P28818, B8K2Q4, Q9Z0W5, G3V8A5, A0A0G2K7C1, Q52KS1, Q63622, A0A0G2K2E3, D4A3V6, A0A0G2JWM2, Q

4A9L2, D3ZWS0, A0A0G2JSU4, P13233, B2RYB3, Q4V8H5, Q5M7V8, Q6AYH3, D3ZJ92, M0RBT5, Q4G045, B4F786,

Information Classification: General

G3, A0A0G2JWM2, A0A1B0GWY5, A0A0G2JX20, D3ZLQ8, A0A0G2JW88, A0A0G2JSU4, P13233, D4A4L4, Q5FVT1,

2866, G3V9N7, D3ZCL8, G3V7J3, F1M3G7, Q02563, O70593, G3V9N1, Q5FWU3, D3ZAY8, D4A853, F1M1U0, A0A0
2866, D3ZCL8, G3V9N7, G3V7J3, F1M3G7, Q02563, O70593, G3V9N1, Q5FWU3, D3ZAY8, D4A853, A0A0G2JWM2,

3ZWS0, P31000, D4A559, P08050, A0A0G2K1U5, A0A0G2JYF7, Q6RJR6, G3V6S0, D3Z9D0, G3V9Y1, F1LS01, D3ZHV

F1LW91, D4A8M2, Q5U2S7, G3V913, P85972, P61765, F1LM19, Q52KS1, D4A9L2, A0A0G2JW88, F1MA36, A0A0G
P13233, B2RYB3, Q4V8H5, Q5M7V8, G3V6S0, Q6AYH3, D3ZJ92, M0RBT5, Q4G045, B4F786, D3ZL75, D3ZBT9, E9P
374, P85972, P47819, F1LRL9, Q9Z0W5, O35867, P07936, G3V9G3, A0A0G2JWM2, A0A0G2K207, F1MA36, Q6MF
D4A8M2, Q5U2S7, G3V913, P85972, P61765, F1LM19, Q52KS1, D4A9L2, A0A0G2JW88, F1MA36, A0A0G2JSU4, Q6

0A1B0GWY5, D3ZCC3, D3ZWS0, D4A559, P13233, P08050, D3ZJ92, D3ZHV2, F1M7S0, E9PST5, Q62866, F1LMV6,

A0G2K700, P31000, D3ZWS0, P08050, A0A0G2JYF7, G3V9Y1, D3ZJ92, D3ZHV2, M0RBT5, B4F786, E9PST5, A0A0U
J92, D3ZHV2, B4F786, F1M842, E9PST5, F1LM55, F1LMV6, G3V9N7, F1LW91, D4A8M2, F1M3G7, G3V9N1, F1M4
F9, F1M287, A0A1B0GWY5, D3ZWS0, D4A559, P13233, D4A4L4, P08050, F1LPG3, Q9JKS6, B0BMV7, D3ZJ92, D3Z
2866, D3ZCL8, G3V9N7, G3V7J3, F1M3G7, Q02563, O70593, G3V9N1, Q5FWU3, D3ZAY8, D4A853, A0A0G2JWM2,

A8M2, M0R423, G3V6L7, A0A0G2K1R9, G3V874, Q9Z1H9, P0C5X8, P85972, P47819, P48303, Q9Z0W5, O35867,

87, A0A1B0GWY5, D3ZWS0, D4A559, P13233, D4A4L4, P08050, F1LPG3, Q9JKS6, D3ZJ92, D3ZHV2, F1M7S0, B4F7

F1M4A4, G3V874, P0C5X8, P28818, F1LRL9, P61765, Q9Z0W5, O35867, Q52KS1, P07936, G3V9G3, A0A0G2JWM

P13233, B2RYB3, Q4V8H5, Q5M7V8, G3V6S0, Q6AYH3, D3ZJ92, M0RBT5, Q4G045, B4F786, D3ZL75, D3ZBT9, E9P

F1LMV8, F1LW91, A0A0G2JXZ7, A0A0G2K3N1, G3V6L7, Q02563, F1M4A4, Q80X08, P0C5X8, Q5FWU3, P48303,

G3V9N1, P0C5X8, Q5FWU3, P47819, P48303, F1M5X7, F1LM19, D3ZCI2, F1M1U0, Q498D5, G3V9G3, A0A0G2K20
G3V9N7, D3ZCL8, D4A8M2, G3V7J3, G3V6L7, M0R423, A0A0G2K1R9, Q02563, F1M4A4, G3V874, P28818, F1LRL9,

7C1, M0RD40, Q63622, F1M9F9, A0A0H2UHZ1, F1M287, A0A1B0GWY5, D3ZWS0, D4A559, P13233, D4A4L4, P08
V8H8, D3ZCC3, D3ZWS0, P31000, D4A559, P08050, A0A0G2JYF7, G3V6S0, G3V9Y1, F1LS01, D3ZHV2, F1M7S0, E9P

Information Classification: General

[illegible]66, F1LMV6, G3V9N7, D3ZCL8, F1LMV8, F1LW91, G3V7J3, F1M3G7, D4A8M2, P48303, Q52KS1, G3V9G3, A0A0G

1RRX4, F1LYA6, A0A0G2K8P5, G3V9N7, D3ZCL8, D4A8M2, G3V6L7, M0R423, Q02563, A0A0G2K1R9, F1M4A4, G3

[illegible]JKS6, D3ZJ92, D3ZHV2, F1M7S0, B4F786, E9PST5, F1LMV6, F1LM55, G3V9N7, F1LMV8, F1LW91, D4A8M2, F1M3[illegible]

A0A0G2JYF7, G3V9Y1, D3ZHV2, M0RBT5, E9PST5, A0A0U1RRX4, F1LM55, A0A0G2K8P5, F1M3G7, G3V6L7, F1M4

[illegible]C1, M0RD40, Q63622, F1M9F9, A0A0H2UHZ1, F1M287, A0A1B0GWY5, D3ZWS0, D4A559, P13233, D4A4L4, P08[illegible]

86, F1M842, E9PST5, F1LM55, F1LMV6, G3V9N7, F1LW91, D4A8M2, F1M3G7, G3V9N1, F1M4A4, Q5FWU3, D3ZA
, A0A0G2JW20, D3ZLQ8, P31000, D4A559, P13233, D4A4L4, P08050, Q9JKS6, A0A0G2JYF7, G3V984, G3V6S0, G3V

RJR6, G3V6S0, G3V9Y1, F1LMV6, G3V9N7, F1LW91, D4A8M2, F1M4A4, Q5U2S7, G3V913, D4A0I5, P85972, Q5FW

M7S0, B4F786, E9PST5, F1LMV6, F1LM55, G3V9N7, F1LMV8, F1LW91, D4A8M2, F1M3G7, Q02563, F1M4A4, G3V

A0A0G2JWM2, D4A3V6, A0A1B0GWY5, A0A0G2K207, A0A0G2JW88, F1MA36, P31000, A0A0G2JSU4, P11275, F1M

, P0C5X8, P47819, Q5FWU3, P48303, F1M5X7, F1LM19, D3ZCI2, F1M1U0, Q498D5, G3V9G3, A0A0G2K207, Q6MF

G3V9N7, F1LMV8, F1LW91, F1M3G7, D4A8M2, O70593, Q02563, F1M4A4, G3V9N1, Q5FWU3, P28818, P48303, C

2, A0A0G2JZ50, Q5U2S7, D3ZAY8, P47819, P61765, D4A853, F1M062, A0A0G2JWM2, Q3SWT4, A0A0G2JX20, D4A

Information Classification: General

A0A0G2JW20, D3ZLQ8, P31000, D4A559, P13233, D4A4L4, P08050, Q9JKS6, A0A0G2JYF7, G3V984, G3V6S0, G3V

A0A0G2K8P5, F1LW91, F1M3G7, A0A0G2K1R9, F1M4A4, G3V913, P85972, P47819, F1LRL9, O35867, Q498D5, A0A

P13233, B2RYB3, Q4V8H5, Q5M7V8, G3V6S0, Q6AYH3, D3ZJ92, M0RBT5, Q4G045, B4F786, D3ZL75, D3ZBT9, E9P

9F9, F1M287, A0A1B0GWY5, D3ZWS0, D4A559, P13233, P08050, F1LPG3, D3ZJ92, D3ZHV2, F1M7S0, B4F786, F1M

B2RYB3, Q4V8H5, Q5M7V8, G3V6S0, Q6AYH3, D3ZJ92, M0RBT5, Q4G045, B4F786, D3ZL75, D3ZBT9, E9PST5

[illegible]50, A0A0G2JYF7, G3V6S0, G3V9Y1, D3ZHV2, A0A0U1RRX4, P12839, D4A644, Q810W7, F1LMV6, A0A0G2K8P5, F[illegible]

BL8, Q5BJN8, G3V6S0, G3V9Y1, D3ZJ92, M0RBT5, Q4G045, F1M7S0, B4F786, E9PST5, A0A0U1RRX4, Q810W7, Q[illegible]

RJR6, G3V6S0, G3V9Y1, F1LMV6, G3V9N7, F1LW91, F1M4A4, Q5U2S7, G3V913, P85972, P61765, Q9Z0W5, F1LM[illegible]

3SWT7, Q4V8H8, F1MA36, P31000, D4A559, Q9WU49, P08050, A0A0G2JYF7, Q5FVT1, G3V6S0, Q6AYH3, G3V9Y1

)G2JWM2, R9PXV8, A0A0G2K207, F1MA36, A0A0G2JSU4, Q99N97, Q6IRG7, Q9WU49, A7L638, Q5FVT1, Q3ZB98,
, R9PXV8, A0A0G2K207, F1MA36, A0A0G2JSU4, Q99N97, Q6IRG7, Q9WU49, A7L638, Q5FVT1, Q3ZB98, D3Z9J7, A

/2, Q62866, F1LMV6, D3ZCL8, G3V9N7, G3V7J3, D4A8M2, M0R423, G3V6L7, A0A0G2JXZ7, A0A0G2K1R9, Q02563,

;3V9N7, D3ZCL8, F1LMV8, F1LW91, D4A8M2, G3V7J3, F1M3G7, Q02563, O70593, G3V9N1, F1M4A4, Q5FWU3, D

1RRX4, Q8K3P4, F1LM55, A0A0G2K8P5, F1M3G7, G3V6L7, F1M4A4, G3V9N1, Q5U2S7, Q80X08, P0C5X8, Q9Z1H9
A4, Q5FWU3, D3ZAY8, Q52KS1, D4A853, Q498D5, G3V9G3, A0A0G2JWM2, R9PXV8, A0A0G2K207, F1MA36, A0A0
IV2, F1M7S0, B4F786, F1M842, E9PST5, F1LM55, F1LMV6, G3V9N7, F1LW91, F1LMV8, D4A8M2, F1M3G7, Q0256
, R9PXV8, A0A0G2K207, F1MA36, A0A0G2JSU4, Q99N97, Q6IRG7, Q9WU49, A7L638, Q5FVT1, Q3ZB98, D3Z9J7, A

Q52KS1, A0A0G2K2E3, P07936, G3V9G3, A0A0G2K207, Q6MFX8, F1MA36, P11275, F1M1Y0, A0A0G2K3B8, Q3ZB

'86, F1M842, E9PST5, F1LM55, F1LMV6, G3V9N7, F1LW91, F1LMV8, F1M3G7, D4A8M2, Q02563, G3V9N1, F1M4

'61765, Q9Z0W5, Q63327, D3ZCI2, A0A0G2K2E3, G3V9G3, Q3SWT7, D3ZUY8, A0A0G2JSU4, F1M1Y0, Q6AYH3, D3

)7, Q6MFX8, Q6IRG7, P11275, D3ZWM3, D4A1D8, A7L638, D3Z9R8, A0A0G2K3B8, D3ZJ32, A0A0G2JXN8, G3V9B3,
, P47819, P61765, Q9Z0W5, O35867, P07936, G3V9G3, A0A0G2JWM2, A0A0G2K207, A0A0G2JW88, F1MA36, Q6I

)50, F1LPG3, Q9JKS6, B0BN77, D3ZJ92, D3ZHV2, F1M7S0, B4F786, F1M842, E9PST5, F1LM55, F1LMV6, G3V9N7, D
'ST5, Q62866, Q810W7, F1LMV6, D3ZCL8, G3V9N7, F1LMV8, G3V7J3, D4A8M2, M0R423, G3V6L7, A0A0G2JXZ7, C

2JWM2, A0A0G2K207, D3ZUY8, F1MA36, Q6IRG7, P11275, A0A0G2K3B8, Q3ZB99, P49621, D3ZJ32, A0A0G2JXN8,

V874, P0C5X8, F1LRL9, P28818, P47819, P61765, Q9Z0W5, O35867, Q52KS1, D3ZCI2, P07936, G3V9G3, A0A0G2J

G7, Q02563, F1M4A4, G3V9N1, Q5FWU3, D3ZAY8, P48303, Q52KS1, D4A853, Q63327, Q498D5, G3V9G3, A0A0G2

A4, G3V9N1, Q5U2S7, Q80X08, P0C5X8, Q9Z1H9, P85972, D3ZAY8, F1LRL9, P47819, Q52KS1, F1M062, G3V9G3, A

050, F1LPG3, Q9JKS6, B0BN77, D3ZJ92, D3ZHV2, F1M7S0, B4F786, F1M842, E9PST5, F1LM55, F1LMV6, G3V9N7, D

Y8, D4A853, Q498D5, G3V9G3, A0A0G2JWM2, R9PXV8, F1MA36, A0A0G2JSU4, Q99N97, P11275, B2RYB3, A0A0G
'9Y1, D3ZHV2, F1M842, E9PST5, A0A0U1RRX4, P12839, D4A644, Q810W7, F1LYA6, F1LM55, F1LMV6, A0A0G2K8F

U3, P61765, Q9Z0W5, F1LM19, Q52KS1, Q63327, D4A9L2, D3ZUY8, A0A0G2JW88, A0A0G2JSU4, Q6IRG7, C0JPT7,

'9N1, Q5FWU3, D3ZAY8, P48303, D4A853, Q63327, Q498D5, G3V9G3, A0A0G2JWM2, R9PXV8, D3ZUY8, F1MA36,

X8, P11275, Q6IRG7, D3ZWM3, D4A1D8, A7L638, D3Z9R8, A0A0G2K3B8, D3ZJ32, A0A0G2JXN8, G3V9B3, A0A0A0

Information Classification: General

Q52KS1, Q63327, Q498D5, G3V9G3, A0A0G2JWM2, R9PXV8, A0A0G2K207, F1MA36, D3ZUY8, A0A0G2JSU4, Q6AX

A9L2, F1MA36, A0A0G2JSU4, C0JPT7, B2RYB3, G3V9W0, Q5M7V8, Q6AYH3, A0A0G2JXN8, D3ZL75, D3ZBT9

[illegible]9Y1, D3ZHV2, F1M842, E9PST5, A0A0U1RRX4, P12839, D4A644, Q810W7, F1LYA6, F1LM55, F1LMV6, A0A0G2K8[illegible]

[illegible]0G2K2E3, G3V9G3, A0A0G2JWM2, A0A0G2JX20, A0A0G2JW88, F1MA36, A0A0G2JSU4, Q99N97, F1M1Y0, Q5FV[illegible]

Information Classification: General

M842, E9PST5, F1LM55, G3V9N7, D3ZCL8, F1LMV8, F1LW91, F1M3G7, D4A8M2, O70593, Q02563, F1M4A4, G3V9

1LW91, F1LMV8, F1M3G7, M0R423, A0A0G2K1R9, F1M4A4, G3V913, G3V874, P85972, P47819, F1LRL9, F1LPP6, [illegible]

Information Classification: General

3K3P4, F1LM55, F1LMV6, G3V9N7, F1LW91, F1LMV8, D4A8M2, F1M3G7, A0A0G2JZ50, Q5U2S7, G3V913, D3ZAY8

19, Q52KS1, Q63327, D4A9L2, D3ZUY8, A0A0G2JW88, A0A0G2JSU4, Q6IRG7, C0JPT7, F1M1Y0, Q9WU49, D3ZWM

D3Z9J7, A0A0G2K3B8, Q3ZB99, A0A0G2JXN8, F1M820, P57769, P09606, F1LMT5, P34926, P0C6C0, P25093, D4A(
0A0G2K3B8, Q3ZB99, A0A0G2JXN8, F1M820, P57769, P09606, F1LMT5, P34926, P0C6C0, D4A0G0, A0A0G2K7X3,

, G3V9N1, G3V874, P0C5X8, Q9Z1H9, P85972, P47819, Q5FWU3, P48303, P61765, Q9Z0W5, F1M5X7, F1LM19, O

)3ZAY8, P48303, Q52KS1, F1M1U0, Q498D5, G3V9G3, A0A0G2JWM2, R9PXV8, A0A0G2K207, D3ZUY8, F1MA36, A

, P85972, P47819, D3ZAY8, F1LRL9, Q5GFD9, Q52KS1, D4A853, F1M062, G3V9G3, A0A0G2JWM2, A0A0G2JX20, D
)G2JSU4, Q99N97, P11275, B2RYB3, A0A0G2K9F7, Q5FVT1, G3V9W0, D3Z9R8, D3Z9J7, D3ZJ32, A0A0G2JXN8, D3Z
53, G3V9N1, F1M4A4, Q5FWU3, D3ZAY8, P48303, Q52KS1, D4A853, Q63327, Q498D5, G3V9G3, A0A0G2JWM2, R
0A0G2K3B8, Q3ZB99, A0A0G2JXN8, F1M820, P57769, P09606, F1LMT5, P34926, P0C6C0, D4A0G0, A0A0G2K7X3,

\4, Q5FWU3, D3ZAY8, P48303, D4A853, Q63327, Q498D5, G3V9G3, A0A0G2JWM2, R9PXV8, A0A0G2K207, D3ZU

MFX8, A0A0G2JSU4, P11275, F1M1Y0, Q3ZB99, P49621, D3ZJ32, A0A0G2KAV8, G3V9B3, A0A0A0MXV8

)3ZCL8, F1LW91, F1LMV8, F1M3G7, D4A8M2, O70593, Q02563, G3V9N1, F1M4A4, Q5FWU3, P28818, D3ZAY8, P
Q6MG88, A0A0G2K1R9, G3V913, Q80X08, G3V874, Q9Z1H9, P0C5X8, P85972, P47819, F1LRL9, P48303, P61765, (

Information Classification: General

, F1LPM3, A0A0G2KAV8, D3ZBT9, A0A0A0MXV8, D4A1Q2, F1M110, Q9R1N0, P15205, D4A0G0, P31016, Q8R5H2,

WM2, A0A0G2K207, F1MA36, D3ZUY8, A0A0G2JW88, A0A0G2JSU4, P11275, F1M1Y0, G3V9W0, Q3ZB99, F1LPM3

2JWM2, R9PXV8, D3ZUY8, F1MA36, A0A0G2JSU4, Q6AXS3, P11275, Q6IRG7, B2RYB3, Q9WU49, D3ZWM3, G3V9W

A0A0G2JWM2, A0A0G2JX20, D4A9L2, A0A0G2K207, A0A0G2JW88, D3ZUY8, Q6MFX8, Q99N97, P11275, C0JPT7, F

D3ZCL8, F1LW91, F1LMV8, F1M3G7, D4A8M2, O70593, Q02563, G3V9N1, F1M4A4, Q5FWU3, P28818, D3ZAY8, P4

G2K9F7, Q5FVT1, G3V9W0, D3Z9R8, D3Z9J7, D3ZJ32, A0A0G2JXN8, D3ZL75, D3ZBT9, P57769, F1LMT5, D4A1Q2, P
P5, F1LW91, F1LMV8, F1M3G7, D4A8M2, M0R423, A0A0G2JZ50, A0A0G2K1R9, F1M4A4, G3V913, G3V874, P8597

A0A0G2JSU4, Q6AXS3, P11275, B2RYB3, G3V9W0, D3Z9R8, D3Z9J7, Q3ZB99, D3ZJ32, A0A0G2JXN8, D3ZL75, D3Z

S3, P11275, Q9WU49, Q5FVT1, G3V9W0, D3Z9R8, D3Z9J7, D3ZJ32, A0A0G2JXN8, F1LPM3, D3ZL75, P57769, P096

?5, F1LW91, F1LMV8, F1M3G7, D4A8M2, M0R423, A0A0G2JZ50, A0A0G2K1R9, F1M4A4, G3V913, G3V874, P8597

N1, Q5FWU3, P28818, P48303, Q52KS1, Q63327, Q498D5, G3V9G3, A0A0G2JWM2, R9PXV8, A0A0G2K207, F1MA

O35867, Q498D5, A0A0G2K2E3, G3V9G3, A0A0G2JWM2, A0A0G2JX20, A0A0G2JW88, F1MA36, A0A0G2JSU4, Q9[illegible]

Information Classification: General

, P47819, P61765, D4A853, F1M062, G3V9G3, A0A0G2JWM2, R9PXV8, Q3SWT4, A0A0G2JX20, D4A9L2, F1MA36,

Information Classification: General

)G0, A0A0G2K7X3, Q8VHJ9, Q5XI97, D3ZMS1, Q4V8H8, D3ZLQ8, F1M8H5, Q6AY21, A0A0G2K4S1, Q4V8H5, Q5BJN
Q8VHJ9, Q5XI97, D3ZMS1, Q4V8H8, D3ZLQ8, F1M8H5, Q6AY21, A0A0G2K4S1, Q4V8H5, Q5BJN8, Q6RJR6, F1LS01

35867, Q52KS1, D3ZCI2, F1M1U0, Q498D5, A0A0G2K2E3, P07936, G3V9G3, A0A0G2K207, Q6MFX8, D3ZUY8, F1M

.0A0G2JSU4, Q6IRG7, P11275, D3ZWM3, A7L638, Q5FVT1, D3Z9R8, D3Z9J7, A0A0G2K3B8, Q3ZB99, P49621, D3ZJ

)4A9L2, A0A0G2K207, Q6MFX8, A0A0G2JW88, D3ZUY8, Q99N97, P11275, C0JPT7, Q9WU49, F1M1Y0, B2RYB3, A0
L75, D3ZBT9, P57769, F1LMT5, P70580, D4A1Q2, P34926, A0A0G2JX30, A0A0G2JZX5, D4A0G0, P15205, G3V733,
9PXV8, A0A0G2K207, D3ZUY8, F1MA36, Q6AXS3, A0A0G2JSU4, Q99N97, P11275, Q6IRG7, B2RYB3, Q9WU49, D3Z
Q8VHJ9, Q5XI97, D3ZMS1, Q4V8H8, D3ZLQ8, F1M8H5, Q6AY21, A0A0G2K4S1, Q4V8H5, Q5BJN8, Q6RJR6, F1LS01

Y8, F1MA36, Q6AXS3, A0A0G2JSU4, Q99N97, P11275, B2RYB3, Q9WU49, A0A0G2K9F7, Q5FVT1, G3V9W0, D3Z9R

48303, Q52KS1, D4A853, Q63327, Q498D5, G3V9G3, A0A0G2JWM2, R9PXV8, A0A0G2K207, D3ZUY8, F1MA36, Q6
Q9Z0W5, F1M5X7, F1LPP6, O35867, Q52KS1, A0A0G2K2E3, D3ZCI2, P07936, G3V9G3, A0A0G2JWM2, A0A0G2JX2

, Q4V8H8, P31000, A0A0G2JYF7, G3V6S0, G3V9Y1, F1LS01, Q810W7, M0R423, G3V6L7, A0A0G2JXZ7, Q6MG88, A

V0, D3Z9R8, Q3ZB98, D3Z9J7, D3ZC55, Q3ZB99, D3ZJ32, A0A0G2JXN8, D3ZL75, D3ZBT9, P57769, P09606, F1LMT5

F1M1Y0, A0A0G2K9F7, D3Z9R8, G3V9W0, Q5M7V8, A0A0G2K3B8, P82458, D3ZL75, A0A0A0MXV8, P0C627

48303, Q52KS1, D4A853, Q63327, Q498D5, G3V9G3, A0A0G2JWM2, R9PXV8, A0A0G2K207, D3ZUY8, F1MA36, Q6

Information Classification: General

'70580, P34926, A0A0G2JX30, A0A0G2JZX5, D4A0G0, P15205, G3V733, P61980, Q8VHJ9, D3ZMS1, F1M241, A0A0
'2, F1LRL9, P47819, F1LPP6, O35867, D4A853, F1M062, A0A0G2K2E3, Q498D5, P07936, G3V9G3, A0A0G2JWM2,

BT9, P57769, P09606, F1LMT5, G3V8F3, P70580, F1M110, P0C6C0, A0A0G2JX30, A0A0G2JZX5, D4A0G0, A0A0G2[illegible]

[illegible]06, F1LMT5, P70580, F1M110, P0C6C0, A0A0G2JX30, A0A0G2JZX5, D4A0G0, P15205, P31016, G3V6R7, G3V733,

'2, F1LRL9, P47819, F1LPP6, O35867, D4A853, F1M062, A0A0G2K2E3, Q498D5, P07936, G3V9G3, A0A0G2JWM2,

A36, D3ZUY8, A0A0G2JSU4, Q6AXS3, P11275, Q9WU49, Q5FVT1, G3V9W0, D3Z9R8, Q3ZB98, D3Z9J7, Q3ZB99, P4[illegible]

A0A0G2JSU4, Q6AXS3, C0JPT7, P11275, B2RYB3, G3V9W0, Q5M7V8, Q6AYH3, Q3ZB99, A0A0G2JXN8, D3ZL75, D[illegible]

N8, Q6RJR6, F1LS01, M0RBT5, Q4G045, D4A644, Q810W7, Q8K3P4, G3V6L7, A0A0G2JXZ7, A0A0G2K3N1, Q6MG8[illegible]
[illegible], M0RBT5, Q4G045, D4A644, Q810W7, Q8K3P4, G3V6L7, A0A0G2JXZ7, A0A0G2K3N1, Q6MG88, G3V913, Q5U2S[illegible]

MA36, P11275, Q6IRG7, F1M1Y0, D3ZWM3, D4A1D8, A7L638, D3Z9R8, A0A0G2K3B8, Q3ZB99, D3ZJ32, F1LPM3, A

32, F1LPM3, A0A0G2KAV8, A0A0G2JXN8, D3ZBT9, A0A0A0MXV8, P57769, F1LMT5, D4A1Q2, P70580, A0A0G2K6[illegible]

P61980, Q8VHJ9, D3ZMS1, F1M241, A0A0G2KB60, A0A0G2K700, Q4V8H8, D3ZLQ8, P31000, Q4V8H5, Q5BJN8, C
ZWM3, A0A0G2K9F7, Q5FVT1, G3V9W0, D3Z9R8, Q3ZB98, D3Z9J7, D3ZC55, Q3ZB99, D3ZJ32, A0A0G2JXN8, D3ZL[illegible]
[illegible], M0RBT5, Q4G045, D4A644, Q810W7, Q8K3P4, G3V6L7, A0A0G2JXZ7, A0A0G2K3N1, Q6MG88, G3V913, Q5U2S[illegible]

[illegible]8, D3Z9J7, Q3ZB99, D3ZJ32, A0A0G2JXN8, D3ZL75, D3ZBT9, P57769, P09606, F1LMT5, P70580, G3V8F3, D4A1Q2

[illegible]AXS3, A0A0G2JSU4, Q99N97, P11275, Q9WU49, B2RYB3, Q5FVT1, A0A0G2K9F7, G3V9W0, D3Z9R8, Q3ZB98, D3Z
0, A0A0G2K207, Q6MFX8, F1MA36, A0A0G2JW88, D3ZUY8, C0JPT7, P11275, Q6IRG7, F1M1Y0, Q63092, Q6AYH3,

0A0G2K1R9, G3V913, Q80X08, G3V874, Q9Z1H9, P0C5X8, P85972, F1LRL9, P47819, P61765, Q9Z0W5, F1M5X7, F

, P70580, G3V8F3, F1M110, P0C6C0, Q9R1N0, A0A0G2JX30, P25093, A0A0G2JZX5, D4A0G0, A0A0G2K7X3, P3101

5AXS3, A0A0G2JSU4, Q99N97, P11275, Q9WU49, B2RYB3, D3ZWM3, A7L638, Q5FVT1, A0A0G2K9F7, G3V9W0, D3

Information Classification: General

)G2KB60, A0A0G2K700, Q4V8H8, D3ZLQ8, P31000, Q4V8H5, Q5BJN8, Q6RJR6, G3V6S0, G3V9Y1, Q4G045, M0RBT
R9PXV8, A0A0G2JX20, A0A0G2K207, A0A0G2JW88, F1MA36, A0A0G2JSU4, Q6AXS3, Q99N97, C0JPT7, Q9WU49,

K7X3, P31016, G3V6R7, G3V733, P61980, Q8VHJ9, Q5XI97, D3ZMS1, F1M241, A0A0G2JY69, D4A3V6, F1M3W5, A

F1M241, D4A3V6, A0A0G2JY69, F1M3W5, A0A0G2KB60, Q4V8H8, F1M8H5, D3ZLQ8, P31000, A0A0G2JYF7, Q6RJ

R9PXV8, A0A0G2JX20, A0A0G2K207, A0A0G2JW88, F1MA36, A0A0G2JSU4, Q6AXS3, Q99N97, C0JPT7, Q9WU49,

9621, D3ZJ32, A0A0G2JXN8, F1LPM3, D3ZL75, D3ZBT9, A0A0A0MXV8, P57769, P09606, F1LMT5, P70580, D4A1Q

8, D4A0I5, G3V913, Q5U2S7, F1LRL9, A0A140TA95, Q5GFD9, F1LM19, F1M062, D4A9L2, G3V927, Q6MFX8, A0A0
7, D4A0I5, F1LRL9, A0A140TA95, Q5GFD9, F1M062, D4A9L2, G3V927, Q6MFX8, A0A0G2JW88, C0JPT7, F1M1Y0, (

N2, F1M110, Q9R1N0, A0A0G2JX30, A0A0G2JZX5, P15205, D4A0G0, P31016, G3V733, P61980, Q8VHJ9, F1M241,

Q6RJR6, G3V6S0, G3V9Y1, Q4G045, M0RBT5, P12839, A0A0U1RRX4, Q810W7, A0A0G2K8P5, A0A0G2K3N1, A0A0(
75, D3ZBT9, P57769, P09606, F1LMT5, P70580, G3V8F3, D4A1Q2, P34926, F1M110, P0C6C0, Q9R1N0, A0A0G2JX
7, D4A0I5, F1LRL9, A0A140TA95, Q5GFD9, F1M062, D4A9L2, G3V927, Q6MFX8, A0A0G2JW88, C0JPT7, F1M1Y0, (

, P34926, F1M110, P0C6C0, A0A0G2JX30, A0A0G2JZX5, D4A0G0, P15205, A0A0G2K7X3, P31016, G3V6R7, G3V73

Z9J7, Q3ZB99, P49621, D3ZJ32, A0A0G2JXN8, F1LPM3, D3ZL75, F1M820, D3ZBT9, A0A0A0MXV8, P57769, P09606

1LPP6, O35867, D3ZCI2, A0A0G2K2E3, P07936, A0A0G2JX20, Q6MFX8, A0A0G2JW88, C0JPT7, F1M1Y0, Q63092,

6, G3V6R7, G3V733, P61980, Q8VHJ9, Q5XI97, D3ZMS1, F1M241, A0A0G2JY69, D4A3V6, F1M3W5, A0A0G2KB60

Z9R8, Q3ZB98, D3Z9J7, Q3ZB99, P49621, D3ZJ32, A0A0G2JXN8, F1LPM3, A0A0G2KAV8, D3ZL75, F1M820, D3ZBT

'5, P12839, A0A0U1RRX4, A0A0G2K8P5, A0A0G2K3N1, A0A0G2JZ50, A0A0G2K1R9, Q5U2S7, G3V913, Q80X08, P0

.0A0G2KB60, A0A0G2K700, Q4V8H8, F1M8H5, D3ZLQ8, P31000, D3ZYS1, Q4V8H5, M0RBL8, A0A0G2JYF7, Q5BJN8

R6, G3V6S0, D4ADX8, G3V9Y1, P12839, A0A0G2K8P5, A0A0G2JXZ7, A0A0G2K3N1, A0A0G2JZ50, G3V6L7, A0A0G2

2, P34926, F1M110, P0C6C0, A0A0G2JX30, A0A0G2JZX5, D4A0G0, P15205, P31016, G3V6R7, G3V733, Q5XI97, F1

Information Classification: General

G2JW88, C0JPT7, F1M1Y0, Q63092, Q5M7V8, A0A0G2KAJ5, G3V9B3, P0C627, Q9P290, Q9Z2L0, A0A0G2K6R9, F1
Q63092, Q5M7V8, G3V9B3, P0C627, Q9Z2L0, Q9P290, A0A0G2K6R9, F1LST1, Q5BKB9, F1M6X3, A0A0G2K4N6, A0

Q8R5H2, Q4V8H8, F1M8H5, P31000, A0A0G2K1U5, A0A0G2JYF7, Q6RJR6, G3V6S0, D4ADX8, D3Z9D0, G3V9Y1, F1

G2JZ50, A0A0G2K1R9, Q5U2S7, G3V913, Q80X08, P0C5X8, P85972, P47819, F1LRL9, P61765, Q9Z0W5, O35867, F
30, P25093, A0A0G2JZX5, D4A0G0, P15205, A0A0G2K7X3, P31016, G3V6R7, G3V733, P61980, Q8VHJ9, Q5XI97, D
Q63092, Q5M7V8, G3V9B3, P0C627, Q9Z2L0, Q9P290, A0A0G2K6R9, F1LST1, Q5BKB9, F1M6X3, A0A0G2K4N6, A0

3, P61980, Q8VHJ9, Q5XI97, D3ZMS1, F1M241, A0A0G2JY69, F1M3W5, D4A3V6, A0A0G2KB60, A0A0G2K700, Q4

, F1LMT5, P70580, G3V8F3, D4A1Q2, P34926, F1M110, P0C6C0, A0A0G2JX30, A0A0G2JZX5, D4A0G0, P15205, A0

Information Classification: General

, A0A0G2K700, Q4V8H8, F1M8H5, D3ZLQ8, P31000, D3ZYS1, Q4V8H5, M0RBL8, A0A0G2JYF7, Q5BJN8, Q6RJR6, G

9, A0A0A0MXV8, P57769, P09606, F1LMT5, P70580, G3V8F3, D4A1Q2, P34926, F1M110, P0C6C0, A0A0G2JX30, A

Information Classification: General

C5X8, P85972, P47819, F1LRL9, P61765, Q9Z0W5, O35867, F1M062, A0A0G2K2E3, D3ZCI2, A0A0G2JX20, Q3SWT

3, Q6RJR6, G3V6S0, G3V9Y1, Q4G045, M0RBT5, A0A0U1RRX4, P12839, Q810W7, Q8K3P4, A0A0G2K8P5, A0A0G2.

[illegible]K1R9, G3V913, Q80X08, P0C5X8, P85972, P47819, F1LRL9, P61765, Q9Z0W5, O35867, D3ZCI2, A0A0G2K2E3, F1[illegible]

Information Classification: General

M241, D4A3V6, A0A0G2JY69, F1M3W5, A0A0G2KB60, Q4V8H8, F1M8H5, D3ZLQ8, P31000, Q6AY21, Q4V8H5, A0

1LS01, Q810W7, F1LYA6, G3V6L7, A0A0G2JXZ7, A0A0G2K3N1, M0R423, Q6MG88, A0A0G2K1R9, G3V9
