# Supplementary material for: Salt-induced phosphoproteomic changes in the subfornical organ in rats with chronic kidney disease
Source: Ren Fail. 2023 Jan 30;45(1):2171886. doi: 10.1080/0886022X.2023.2171886 (PMC9888458; doi:10.1080/0886022X.2023.2171886)
Supplement: Supplemental Material [file IRNF_A_2171886_SM9865.zip › 2171886/Copy of Supplementary_Table_S5.pdf]

## Supplementary Table S5-2. The anlysis of KEEG in HC/NC.

| Map_ID | Map_Name | Seqs | Seqs_Num | URL |
|---|---|---|---|---|
| map04530 | Tight junction | D4A8M2 E | 9 | http://wwv |
| map04144 | Endocytosis | D3ZUY8 A0 | 7 | http://wwv |
| map05205 | Proteoglycans in cancer | F1LST1 C0J | 6 | http://wwv |
| map04010 | MAPK signaling pathway | G3V913 C0 | 5 | http://wwv |
| map03040 | Spliceosome | D3ZMS1 D3 | 5 | http://wwv |
| map04727 | GABAergic synapse | A0A0G2K4 | 5 | http://wwv |
| map03015 | mRNA surveillance pathway | D3ZAY8 B2 | 4 | http://wwv |
| map04510 | Focal adhesion | F1LST1 C0J | 4 | http://wwv |
| map05412 | Arrhythmogenic right ventricular cardiomyopathy (ARVC) | P08050 Q8 | 4 | http://wwv |
| map04080 | Neuroactive ligand-receptor interaction | B8K2Q4 P4 | 4 | http://wwv |
| map04024 | cAMP signaling pathway | G3V9G3 P1 | 4 | http://wwv |
| map04390 | Hippo signaling pathway | Q63622 D3 | 4 | http://wwv |
| map04810 | Regulation of actin cytoskeleton | F1LST1 P85 | 3 | http://wwv |
| map04014 | Ras signaling pathway | D3ZCL8 P28 | 3 | http://wwv |
| map05100 | Bacterial invasion of epithelial cells | F1LST1 P85 | 3 | http://wwv |
| map04925 | Aldosterone synthesis and secretion | F1LMV8 G3 | 3 | http://wwv |
| map04020 | Calcium signaling pathway | G3V9G3 Q9 | 3 | http://wwv |
| map04725 | Cholinergic synapse | F1M6X3 G3 | 3 | http://wwv |
| map05206 | MicroRNAs in cancer | F1LMV8 P3 | 3 | http://wwv |
| map03013 | RNA transport | D3ZAY8 B2 | 3 | http://wwv |
| map04750 | Inflammatory mediator regulation of TRP channels | F1LMV8 G3 | 3 | http://wwv |
| map04921 | Oxytocin signaling pathway | Q8VHW9 G | 3 | http://wwv |
| map04261 | Adrenergic signaling in cardiomyocytes | Q8VHW9 G | 3 | http://wwv |
| map04911 | Insulin secretion | G3V9G3 P1 | 3 | http://wwv |
| map05016 | Huntington's disease | D3ZUY8 Q9 | 3 | http://wwv |
| map04520 | Adherens junction | F1M820 P8 | 3 | http://wwv |
| map05032 | Morphine addiction | B8K2Q4 Q6 | 3 | http://wwv |
| map05169 | Epstein-Barr virus infection | G3V913 P3 | 3 | http://wwv |
| map04721 | Synaptic vesicle cycle | P61765 D3 | 3 | http://wwv |
| map04742 | Taste transduction | A0A0G2K2 | 3 | http://wwv |
| map05200 | Pathways in cancer | F1LST1 A0A | 3 | http://wwv |
| map04360 | Axon guidance | G3V9G3 P1 | 3 | http://wwv |
| map04915 | Estrogen signaling pathway | B8K2Q4 Q6 | 3 | http://wwv |
| map05146 | Amoebiasis | G3V913 F1 | 3 | http://wwv |
| map04670 | Leukocyte transendothelial migration | P85972 A0 | 3 | http://wwv |
| map04624 | Toll and Imd signaling pathway | A0A0G2K1 | 2 | http://wwv |
| map04666 | Fc gamma R-mediated phagocytosis | F1LMV8 A0 | 2 | http://wwv |
| map04720 | Long-term potentiation | G3V9G3 P1 | 2 | http://wwv |
| map05152 | Tuberculosis | G3V9G3 P1 | 2 | http://wwv |
| map00590 | Arachidonic acid metabolism | Q5PQL9 P8 | 2 | http://wwv |
| map00030 | Pentose phosphate pathway | Q52KS1 P3 | 2 | http://wwv |
| map05014 | Amyotrophic lateral sclerosis (ALS) | F1LRZ7 P12 | 2 | http://wwv |
| map04922 | Glucagon signaling pathway | G3V9G3 P1 | 2 | http://wwv |
| map04931 | Insulin resistance | F1LMV8 P8 | 2 | http://wwv |
| map00230 | Purine metabolism | P39069 P3 | 2 | http://wwv |
| map04713 | Circadian entrainment | G3V9G3 P1 | 2 | http://wwv |

Information Classification: General

| | | | | |
|---|---|---|---|---|
| map04146 | Peroxisome | A0A0G2JY6 | 2 | http://www |
| map05203 | Viral carcinogenesis | P61980 D3 | 2 | http://www |
| map00520 | Amino sugar and nucleotide sugar metabolism | P82808 P3 | 2 | http://www |
| map04071 | Sphingolipid signaling pathway | F1LMV8 P4 | 2 | http://www |
| map05031 | Amphetamine addiction | G3V9G3 P1 | 2 | http://www |
| map04514 | Cell adhesion molecules (CAMs) | Q6IRG7 G3 | 2 | http://www |
| map05214 | Glioma | G3V9G3 P1 | 2 | http://www |
| map04022 | cGMP-PKG signaling pathway | F1LMV8 Q9 | 2 | http://www |
| map04015 | Rap1 signaling pathway | Q5BKB9 F1 | 2 | http://www |
| map04971 | Gastric acid secretion | G3V9G3 P1 | 2 | http://www |
| map04066 | HIF-1 signaling pathway | G3V9G3 P1 | 2 | http://www |
| map04745 | Phototransduction - fly | G3V9G3 P1 | 2 | http://www |
| map04916 | Melanogenesis | G3V9G3 P1 | 2 | http://www |
| map05168 | Herpes simplex infection | P61980 D4 | 2 | http://www |
| map04114 | Oocyte meiosis | G3V9G3 P1 | 2 | http://www |
| map00250 | Alanine, aspartate and glutamate metabolism | P82808 P0 | 2 | http://www |
| map04310 | Wnt signaling pathway | G3V9G3 P1 | 2 | http://www |
| map04722 | Neurotrophin signaling pathway | G3V9G3 P1 | 2 | http://www |
| map04391 | Hippo signaling pathway - fly | D3ZWS0 F1 | 2 | http://www |
| map04740 | Olfactory transduction | G3V9G3 P1 | 2 | http://www |
| map04724 | Glutamatergic synapse | P31016 P0 | 2 | http://www |
| map04728 | Dopaminergic synapse | G3V9G3 P1 | 2 | http://www |
| map04621 | NOD-like receptor signaling pathway | F1M842 Q9 | 2 | http://www |
| map01230 | Biosynthesis of amino acids | Q52KS1 P0 | 2 | http://www |
| map00010 | Glycolysis / Gluconeogenesis | Q52KS1 P3 | 2 | http://www |
| map05110 | Vibrio cholerae infection | Q3ZB99 Q9 | 2 | http://www |
| map05010 | Alzheimer's disease | Q6RJR6 D4 | 2 | http://www |
| map04512 | ECM-receptor interaction | F1LST1 Q0 | 2 | http://www |
| map00052 | Galactose metabolism | Q52KS1 P3 | 2 | http://www |
| map04912 | GnRH signaling pathway | G3V9G3 P1 | 2 | http://www |
| map04933 | AGE-RAGE signaling pathway in diabetic complications | F1LST1 F1L | 2 | http://www |
| map04012 | ErbB signaling pathway | G3V9G3 P1 | 2 | http://www |
| map05164 | Influenza A | Q9Z2L0 | 1 | http://www |
| map04392 | Hippo signaling pathway -multiple species | F1M5X7 | 1 | http://www |
| map03018 | RNA degradation | Q52KS1 | 1 | http://www |
| map04370 | VEGF signaling pathway | G3V913 | 1 | http://www |
| map04962 | Vasopressin-regulated water reabsorption | A0A0H2UH | 1 | http://www |
| map01212 | Fatty acid metabolism | G3V6R7 | 1 | http://www |
| map04140 | Autophagy | D3Z9J7 | 1 | http://www |
| map04723 | Retrograde endocannabinoid signaling | A0A0G2KA | 1 | http://www |
| map05131 | Shigellosis | P85972 | 1 | http://www |
| map04139 | Mitophagy - yeast | D3Z9J7 | 1 | http://www |
| map04630 | Jak-STAT signaling pathway | P47819 | 1 | http://www |
| map05410 | Hypertrophic cardiomyopathy (HCM) | Q8VHW9 | 1 | http://www |
| map04270 | Vascular smooth muscle contraction | F1LMV8 | 1 | http://www |
| map00220 | Arginine biosynthesis | P09606 | 1 | http://www |
| map05160 | Hepatitis C | Q6IRG7 | 1 | http://www |
| map05012 | Parkinson's disease | Q9Z2L0 | 1 | http://www |
| map04976 | Bile secretion | A0A0H2UH | 1 | http://www |
| map00780 | Biotin metabolism | G3V6R7 | 1 | http://www |

Information Classification: General

| | | | | |
|---|---|---|---|---|
| map04151 | PI3K-Akt signaling pathway | F1LST1 | 1 | http://wwv |
| map00630 | Glyoxylate and dicarboxylate metabolism | P09606 | 1 | http://wwv |
| map03008 | Ribosome biogenesis in eukaryotes | A0A0G2K7 | 1 | http://wwv |
| map00500 | Starch and sucrose metabolism | P38652 | 1 | http://wwv |
| map04540 | Gap junction | P08050 | 1 | http://wwv |
| map03050 | Proteasome | Q5U2S7 | 1 | http://wwv |
| map04212 | Longevity regulating pathway - worm | D3Z9J7 | 1 | http://wwv |
| map00051 | Fructose and mannose metabolism | Q52KS1 | 1 | http://wwv |
| map05414 | Dilated cardiomyopathy | Q8VHW9 | 1 | http://wwv |
| map04977 | Vitamin digestion and absorption | Q62866 | 1 | http://wwv |
| map03420 | Nucleotide excision repair | G3V9W0 | 1 | http://wwv |
| map04070 | Phosphatidylinositol signaling system | P49621 | 1 | http://wwv |
| map01523 | Antifolate resistance | Q62866 | 1 | http://wwv |
| map05202 | Transcriptional misregulation in cancer | Q4G045 | 1 | http://wwv |
| map04120 | Ubiquitin mediated proteolysis | P82458 | 1 | http://wwv |
| map04260 | Cardiac muscle contraction | Q8VHW9 | 1 | http://wwv |
| map04152 | AMPK signaling pathway | Q52KS1 | 1 | http://wwv |
| map05222 | Small cell lung cancer | F1LST1 | 1 | http://wwv |
| map05212 | Pancreatic cancer | Q5FVT1 | 1 | http://wwv |
| map02020 | Two-component system | P09606 | 1 | http://wwv |
| map00564 | Glycerophospholipid metabolism | P49621 | 1 | http://wwv |
| map01200 | Carbon metabolism | Q52KS1 | 1 | http://wwv |
| map04961 | Endocrine and other factor-regulated calcium reabsorption | D3ZUY8 | 1 | http://wwv |
| map00061 | Fatty acid biosynthesis | G3V6R7 | 1 | http://wwv |
| map05132 | Salmonella infection | C0JPT7 | 1 | http://wwv |
| map00680 | Methane metabolism | Q52KS1 | 1 | http://wwv |
| map05213 | Endometrial cancer | A0A0G2JYF | 1 | http://wwv |
| map00521 | Streptomycin biosynthesis | P38652 | 1 | http://wwv |
| map00790 | Folate biosynthesis | M0R423 | 1 | http://wwv |
| map05130 | Pathogenic Escherichia coli infection | A0A1B0GV | 1 | http://wwv |
| map04013 | MAPK signaling pathway - fly | D4A853 | 1 | http://wwv |
| map00561 | Glycerolipid metabolism | P49621 | 1 | http://wwv |
| map00350 | Tyrosine metabolism | P25093 | 1 | http://wwv |
| map05140 | Leishmaniasis | A0A0G2K6 | 1 | http://wwv |
| map05030 | Cocaine addiction | P31016 | 1 | http://wwv |
| map00643 | Styrene degradation | P25093 | 1 | http://wwv |
| map05230 | Central carbon metabolism in cancer | Q52KS1 | 1 | http://wwv |
| map04668 | TNF signaling pathway | G3V9N1 | 1 | http://wwv |
| map05231 | Choline metabolism in cancer | P49621 | 1 | http://wwv |
| map04072 | Phospholipase D signaling pathway | P49621 | 1 | http://wwv |
| map03320 | PPAR signaling pathway | F1M820 | 1 | http://wwv |
| map05166 | HTLV-I infection | Q9Z2L0 | 1 | http://wwv |
| map04972 | Pancreatic secretion | Q9R1N0 | 1 | http://wwv |
| map04910 | Insulin signaling pathway | F1M820 | 1 | http://wwv |
| map04068 | FoxO signaling pathway | P48303 | 1 | http://wwv |
| map04930 | Type II diabetes mellitus | F1LMV8 | 1 | http://wwv |
| map04150 | mTOR signaling pathway | D3Z9J7 | 1 | http://wwv |
| map04211 | Longevity regulating pathway | D3Z9J7 | 1 | http://wwv |
| map04970 | Salivary secretion | Q9R1N0 | 1 | http://wwv |
| map00910 | Nitrogen metabolism | P09606 | 1 | http://wwv |

Information Classification: General

v.kegg.jp/kegg-bin/show_pathway?map04530+K06103+K10352+K18050+K10352+K05691+K06107+K0
v.kegg.jp/kegg-bin/show_pathway?map04144+K11824+K18443+K12495+K12469+K18462+K09526+K1
v.kegg.jp/kegg-bin/show_pathway?map05205+K05717+K04437+K10380+K04515+K04515+K10380

Information Classification: General

Information Classification: General

Information Classification: General

Information Classification: General

6087+K06098+K06112
