# Supplementary figures and images for: Salt-induced phosphoproteomic changes in the subfornical organ in rats with chronic kidney disease
Source: Ren Fail. 2023 Jan 30;45(1):2171886. doi: 10.1080/0886022X.2023.2171886 (PMC9888458; doi:10.1080/0886022X.2023.2171886)

NC/NS

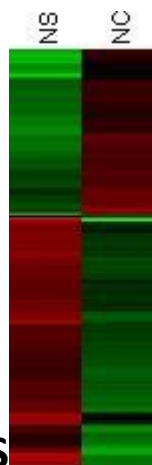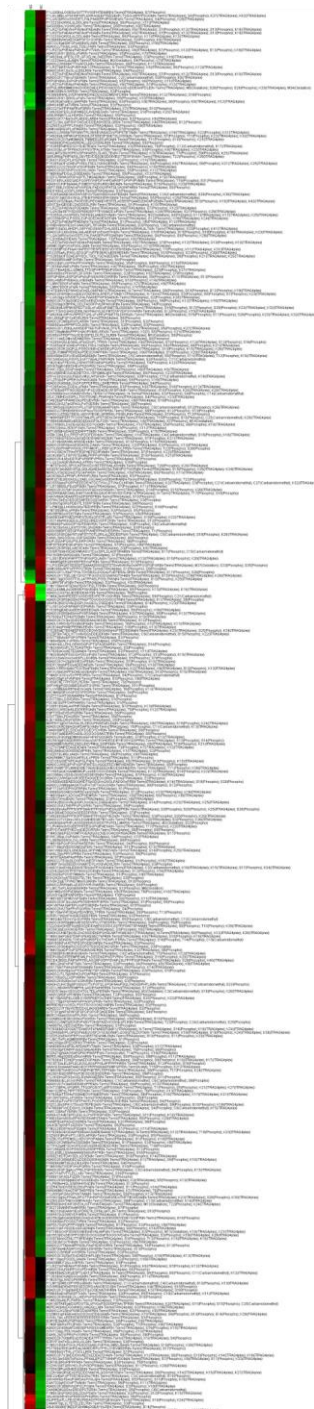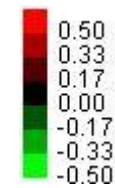

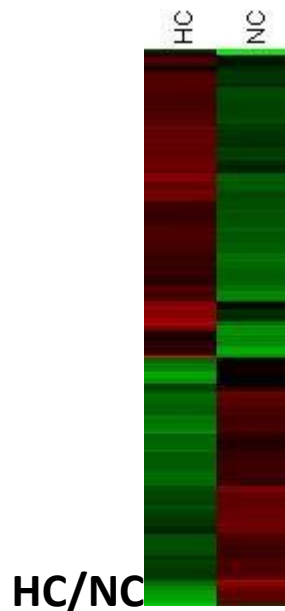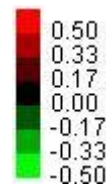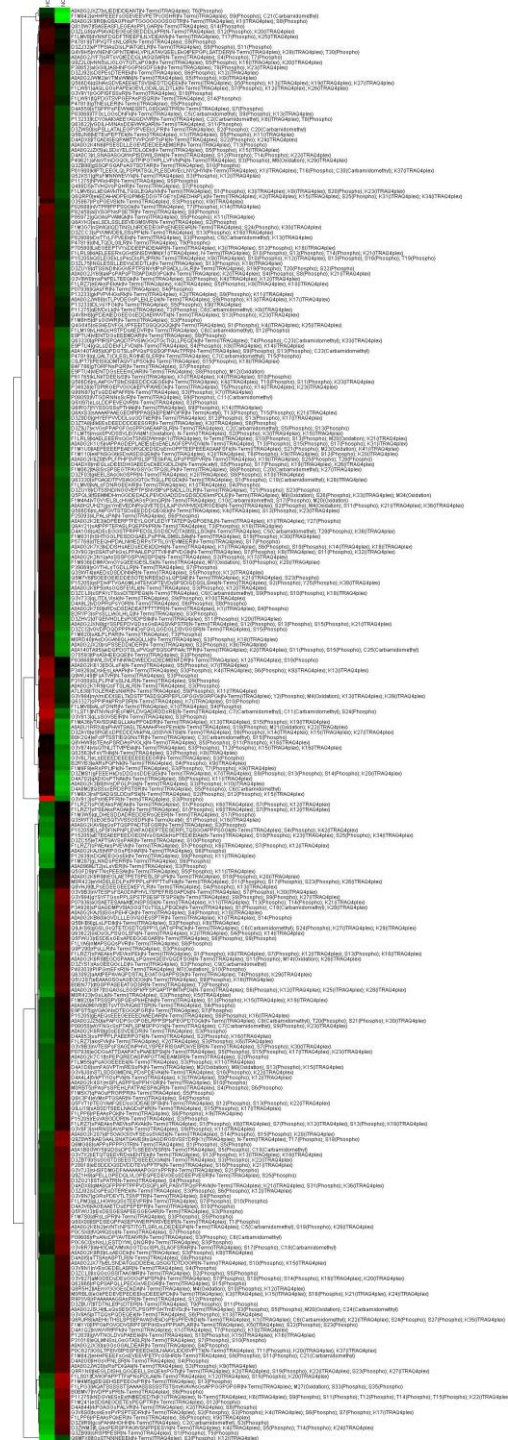

Supplement: Supplemental Material [file IRNF_A_2171886_SM9865.zip › 2171886/Supplementary_Figure_S1.pdf]
